# Using simulated fluorescence cell micrographs for the evaluation of cell image segmentation algorithms

**DOI:** 10.1186/s12859-017-1591-2

**Published:** 2017-03-18

**Authors:** Veit Wiesmann, Matthias Bergler, Ralf Palmisano, Martin Prinzen, Daniela Franz, Thomas Wittenberg

**Affiliations:** 1 Fraunhofer Institute for Integrated Circuits IIS, Am Wolfsmantel 33, Erlangen, 91058 Germany; 2 Optical Imaging Centre Erlangen (OICE), Hartmannstraße 14, Erlangen, 91052 Germany; 3 Friedrich-Alexander University Erlangen-Nuremberg, Cauerstraße 11, Erlangen Erlangen, 91058 Germany

**Keywords:** Fluorescence microscopy, Simulation, Evaluation, Image analysis, Cell segmentation

## Abstract

**Background:**

Manual assessment and evaluation of fluorescent micrograph cell experiments is time-consuming and tedious. Automated segmentation pipelines can ensure efficient and reproducible evaluation and analysis with constant high quality for all images of an experiment. Such cell segmentation approaches are usually validated and rated in comparison to manually annotated micrographs. Nevertheless, manual annotations are prone to errors and display inter- and intra-observer variability which influence the validation results of automated cell segmentation pipelines.

**Results:**

We present a new approach to simulate fluorescent cell micrographs that provides an objective ground truth for the validation of cell segmentation methods. The cell simulation was evaluated twofold: (1) An expert observer study shows that the proposed approach generates realistic fluorescent cell micrograph simulations. (2) An automated segmentation pipeline on the simulated fluorescent cell micrographs reproduces segmentation performances of that pipeline on real fluorescent cell micrographs.

**Conclusion:**

The proposed simulation approach produces realistic fluorescent cell micrographs with corresponding ground truth. The simulated data is suited to evaluate image segmentation pipelines more efficiently and reproducibly than it is possible on manually annotated real micrographs.

**Electronic supplementary material:**

The online version of this article (doi:10.1186/s12859-017-1591-2) contains supplementary material, which is available to authorized users.

## Background

Fluorescence microscopy is widely used for quantitative cell experiments in e.g. microbiology, virology and adjacent fields of life science. This imaging technique allows the acquisition of micrographs depicting cell compartments (as e.g. nuclei, plasma, sub-cellular structures by the means of markers and dyes for specific proteins, e.g. showing cell nuclei by marking the DNA with DAPI or the cell plasma by an Alexa marker or Boron. However, the quantitative manual assessment and analysis of the resulting image material revealing various functional and morphological properties of cells and cell compartments demand high precision, much time and repetitive work. Human observers perceive these analysis tasks as tedious and hence tend to errors. Novel large-scale imaging techniques, e.g. the whole slide scanning technology, enable the acquisition of huge amounts of image data in the terabyte scale and further increase the problems described. Possible solutions to conquer the rising challenge of fluorescent “big data” [1] are automated or semi- automated image processing approaches and methods which ensure a time-efficient, objective and reproducible analysis. In the past years, a plethora of different methods and tools have been proposed for the automated and interactive assessment and analysis of fluorescent micrographs of various applications [2–4]. An overview of free image analysis tools, specifically for fluorescent micrographs, is given in [5].

In order to find the best fitting image-processing method for a specific analysis task, and more specifically the segmentation approach for the most crucial step within an image-processing pipeline, the methods have to be evaluated with respect to some ground truth data. To date, expert based cell-delineation is generally considered as “gold standard” for the generation of ground truth data. To this end, various benchmark collections of cell micrographs including user annotations are offered on the Internet, e. g. by the Broad Institute [6, 7]. Nevertheless, manual assessment and cell-based annotations of image data is tedious, error prone and suffers from a high inter- and intra-observer variability. Especially so, if fluorescent micrographs with complex image data depicting touching, overlapping over even overlaying cells are considered. A widely accepted alternative method to validate image segmentation algorithms is based on simulated or synthetic image data which implicitly provide ground truth information.

Some cell simulation approaches use exclusively graphical methods. Nattkemper et al. [8] model the cell shape with a sum of radial Gaussian functions for the fluorescent contour, the cell contour and the corona contour. The cell texture is interpolated between these three lines. Lehmussola et al. describe a cell shape model based on deformed regular polygons with cell texture generate with Perlin noise [9]. The method is extended in the framework *SimuCell* [10]. In [11] cells and nuclei are modeled using basic geometric shapes such as circles and ellipses and the borders are then varied. Also some variations in illumination and noise are considered. E.g. Ghaye et al. [12] model the physical imaging process. In first step, cells shapes are simulated with the method presented by Lehmussola et al. [9]. The cell texture is then simulated with fluorescent clusters aiming at modeling a fluorescent dye received by surface receptors. Svoboda et al. [13] also model the imaging process to simulate three dimensional cells. The cell shapes are generated by deformation of geometrical objects by partial differential equations. The texture is initially simulated by Perlin noise and then distorted with the imaging system. In [14] a method is proposed for generation of 3D+t benchmark based on an object video database. This database is filled with synthetic cells proposed in [15].

The Murphy lab is the leading laboratory in building cell models. They focus on extracting biological meaningful parameters instead of simulating realistic fluorescent microscopy images. Nevertheless, their models can be used to simulate cell images. For example, Zhao et al. [16] describe generative statistical models for cell and nuclei separately. They propose a parametric medial axes model for the shape of the nuclei and use the ratios between distances from cell outline and nucleus outline to the cell center for the cell shape. Buck et al. [17] give an overview of the cell models developed at Murphy’s laboratory.

In order to simulate own synthetic fluorescent images for comparison and evaluation, the software framework *SimuCell* [10] is a freely available tool on the Internet: https://github.com/AltschulerWu-Lab/simucell. Also, Ruusuvuori et al. [18] describe the evaluation of image processing methods for micrographs using a synthetic benchmark data set which can be downloaded from: http://www.cs.tut.fi/sgn/csb/simcep/benchmark.

In summary, there has been quite some work in the field of fluorescent micrograph simulation, ranging from image rendering over geometric and biological modeling of cell compartments to the availability of cell synthesis tools. Although all of these approaches synthesize fluorescent micrographs, expert human observers can easily distinguish between simulated and real micrographs based on the visual appearance of the simulated cells.

Our method aims at simulating photo-realistic fluorescent cell micrographs. To cover the visual appearance of cell nuclei and plasma textures and structures depicted in real fluorescent micrographs, the methods to simulate and render individual cells are based on the textural input from real image data.

For a visual evaluation of our approach, we have conducted an expert observer study with four exemplary data sets. We can show that images simulated with our approach cannot be distinguished from real images in contrast to images simulated with *SimuCell*. Additionally, the graphical image features depicted by real images and in micrographs simulated with our method are visually comparable.

Finally we exemplarily show that the simulated fluorescent micrographs can be used to validate an image segmentation pipeline. To this end we apply such an image segmentation pipeline to simulated data sets containing cells with increasing degree of overlap and show that the computed segmentation performances yield valid results.

## Methods

First we present four image data sets which have been used as input material for the simulation methods in this study. In the following, we explain our cell simulation approach and then we describe the evaluation of the simulated fluorescent images with an expert observer study and objective measurements. Finally an image segmentation pipeline is applied to a set of the synthetically generated fluorescent micrographs to show that the described simulation approach can be used for the evaluation of segmentation algorithms.

### Basic data sets

Cell segmentation algorithms are often developed and optimized only for especially dedicated sets of fluorescent cell image data, mostly related to some clinical problem. The segmentation complexity of such data sets narrows the segmentation performance and depends on the *cell type* (e.g. macrophages, stem cells, protoplasts, etc.), the sample preparation, the fluorescent dyes and the imaging process (e.g. bright-field, confocal, phase contrast, etc.) process. The complexity from cell type results from the related cell shapes ranging from round over bipolar to irregular shapes. The complexity resulting from the *preparation process* results from the cell density and the distribution on the slide. The complexity from the *imaging process* results from noise and sharpness, since not all cells can be imaged sharp in one field of view. Furthermore, the complexity of touching and overlapping to overlaying cells is considered.

All those parameters contribute to a total complexity. The data sets used in this paper cover a broad range of segmentation complexity and are presented in order of increasing segmentation complexity: protoplasts, B cells and macrophages.

**Fig. 1:**
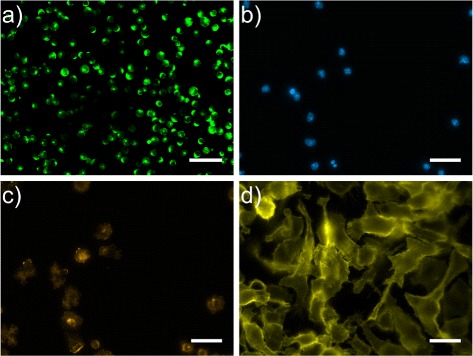
Example data sets ordered with respect to increasing segmentation complexity **a**) micrograph showing chlorophyll inside chloroplasts after Fluorescein diacetate (FDA) staining; **b**) Multi channel data set of naive murine B cells from C57Bl/6 mice B cells. Immunofluorescent staining was performed for DNA (DAPI); **c**) Immunofluorescent staining was performed for F-Actin (Phalloidin-Rhodamin); **d**) LPS activated macrophages with immunofluorescent staining CD11b/APC; Scale bars corresponds to 30 *μ*m

The data set with *protoplasts* (cf. Figure 1 a) has a low segmentation complexity. The protoplasts are round and show high intensity with partly intensity variability inside single cells. The background shows weak fluorescence. The images of the data set show a high signal to noise ratio (in short snr). The data set is described in detail in Held [19] and is available as Additional file 1.

The B cell data set consists of two channels. The first channel depicts DAPI stained *B cell nuclei* (cf. Figure 1 b) while the second channel shows the corresponding Phalloidin-Rhodamin stained *B cell cytoskeleton* (cf. Figure 1 c). The segmentation complexity is different for both channels. For the DAPI stained nuclei, the segmentation complexity lies between the protoplasts and the B cell cytoskeleton. DAPI staining often leads to images with high SNR so that the nuclei can be separated from the background using thresholding. A separation step is rarely necessary as nuclei often lie isolated due to their location inside the cytoplasm. For these reasons the segmentation of nuclei is usually used as seed points for the segmentation of the cytoplasm in literature. Due to the high variability of intensities inside the B cells as well as the high variability of shape, the micrographs with the B cell cytoskeleton show a higher segmentation complexity than the corresponding nuclei. The experimental data is described in more detail in [20] and is available as Additional file 2.

The data set with the CD11b/APC-stained (cf. Figure 1 d) cytomembrane of murine bone marrow *macrophages* shows the highest segmentation complexity. The intensity variability of the cell texture is high inside the cytomembrane and the texture shows high variability over the whole image data set. Also the shape variability is high. Additionally, overlaps in the cytoplasmic channel increase segmentation complexity. The data set is described in detail in Held et al. [21] and is available as Additional file 3.

### Cell image simulation

The proposed approach for the simulation of fluorescent cell images is an extension of the cell simulation framework presented in [11], which makes use of information obtained from real fluorescent images. The individual cell shapes are simulated with a statistical shape model (ASM) described in the next section. Individual cell textures are added by mapping the textures of real hand-labeled cells to simulated cell shapes.

#### Cell shape simulation

In [22] an statistical shape model (ASM) based on the works of Cotes et al. [23] is presented, which has specifically been adapted for the generation of cell shapes. To simulate cell shapes in this paper, we improve the method by preventing the generation of invalid, small cell shapes.

In order to build a cell model for a certain type of cell (stem cells, macrophages, protoplasts, etc.), a representative and hand-labeled set of real cells is needed.

First, the contours of the hand-labeled cells are sampled with *N* many equally spaced points. In the next steps, the mean point of the N contour points is calculated and the mean point is removed from the N contour points to transform the contour to an image independent coordinate system. The the contour is aligned to other contours with respect to the first and second principal axis. The contour points are stored into a descriptive vector ***v***_*i*_ for each shape. To ensure a one to one point correspondence between the descriptive vectors ***v***_*i*_ of all shapes, we aligned the contour representations by minimizing the Eucledian distances between corresponding points. A principle component analysis (PCA) on the contour points results in a mean cell shape ***x***_*m*_ and a transformation matrix ***P*** composed of the Eigenvectors from the PCA.

Using the mean cell shape ***x***_*m*_ and the first n Eigenvectors a new cell shape ***x*** can be generated with a random vector ***b*** and

$$\boldsymbol{x}=\boldsymbol{x}_{m}+\boldsymbol{P}\boldsymbol{b}\quad\text{with}\quad\left|\boldsymbol{b}-\boldsymbol P^{-1}\boldsymbol{x}_{m}\right|> r_{min}. $$

This constraint prevents the generation of invalid, small cell shapes. The parameter *r*_*min*_ depends on the simulated cell type.

#### Cell texture simulation

The texture mapping approach presented in [22] is able to map the cell texture of any delineated real cell (from a reference image) to the shape of a simulated cell shape based on both contours. During the synthesis of cells with irregular shapes this approach may in some cases lead to a distorted texture, especially salient in bright texture regions. To reduce this distortion effect in comparison to previous work [22], we developed a new texture mapping algorithm based on energy minimization. In addition to the shape information which has already been used in the previous work [22], the new algorithm also incorporates the intensity information inside the cells. During the mapping process, the texture of a hand-labeled reference cell acts like an elastic tissue spanned between fixation points. The ductility of the elastic tissue varies according to the local intensities of the cell patch. Adjacent pixels with high intensity have less ductility compared to adjacent pixels with low intensities.

The texture mapping process is conducted by minimizing an energy function consisting of three energy terms acting as elastic energies between different sets of points. The term *E*_fix_ is calculated between the fixation points ***v***_fix_, the energy term *E*_border_ between points ***v***_border_ on the boundary of the real texture patch and the energy term *E*_bulk_ between adjacent points ***v***_bulk_ of the real texture patch. The three energy terms are weighted with factors *w*_fix_,*w*_border_ and *w*_bulk_ leading to the equation

$$E=w_{\text{fix}} E_{\text{fix}}\left(\boldsymbol{v}_{\text{fix}}\right)+w_{\text{border}} E_{\text{border}}\left(\boldsymbol{v}_{\text{border}}\right)+w_{\text{bulk}} E_{\text{bulk}}\left(\boldsymbol{v}_{\mathrm{c}}\right). $$

To prepare the real texture for the texture mapping, reference points of the texture are initially aligned to the new cell shape. Then the points of the real texture patch are stored to the appropriate vectors ***v***_fix_, ***v***_bor_ and ***v***_bulk_ and are used as input to the texture mapping process.

**Fig. 2:**
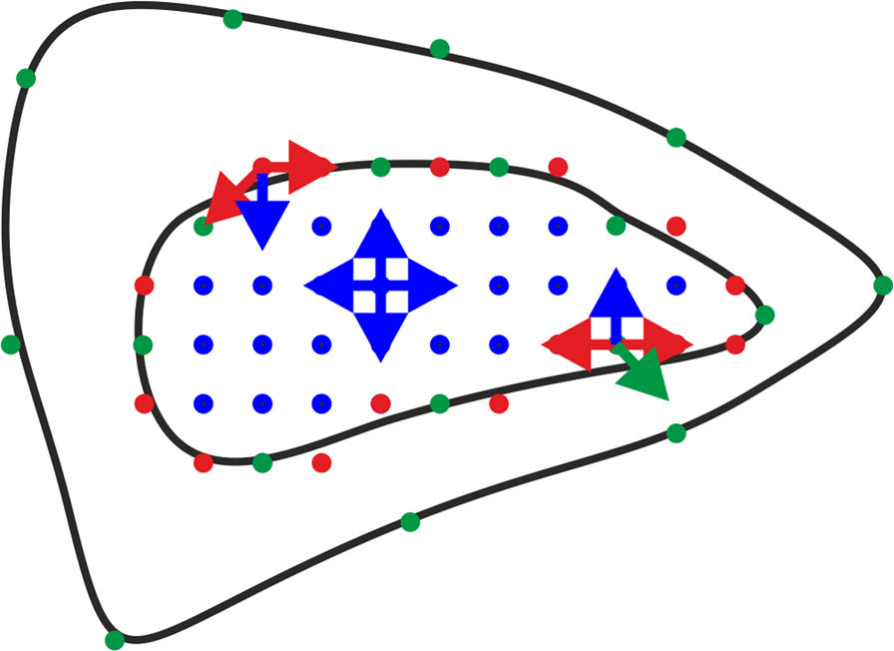
Force diagram of texture mapping approach during the adaption process. The outer contour represents new cell shape. The inner contour represents contour of the used texture. *Green* points are fixation points ***v***_fix_ between new cell shape and texture. *Red* points are border points ***v***_bor_ on texture which are not fixation points. *Blue* points are points in the inner of the texture. *Green arrows* indicate forces between corresponding points. *Red arrows* indicate forces between border points. *Blue arrows* indicate forces between bulk points ***v***_bulk_

**Fig. 3:**
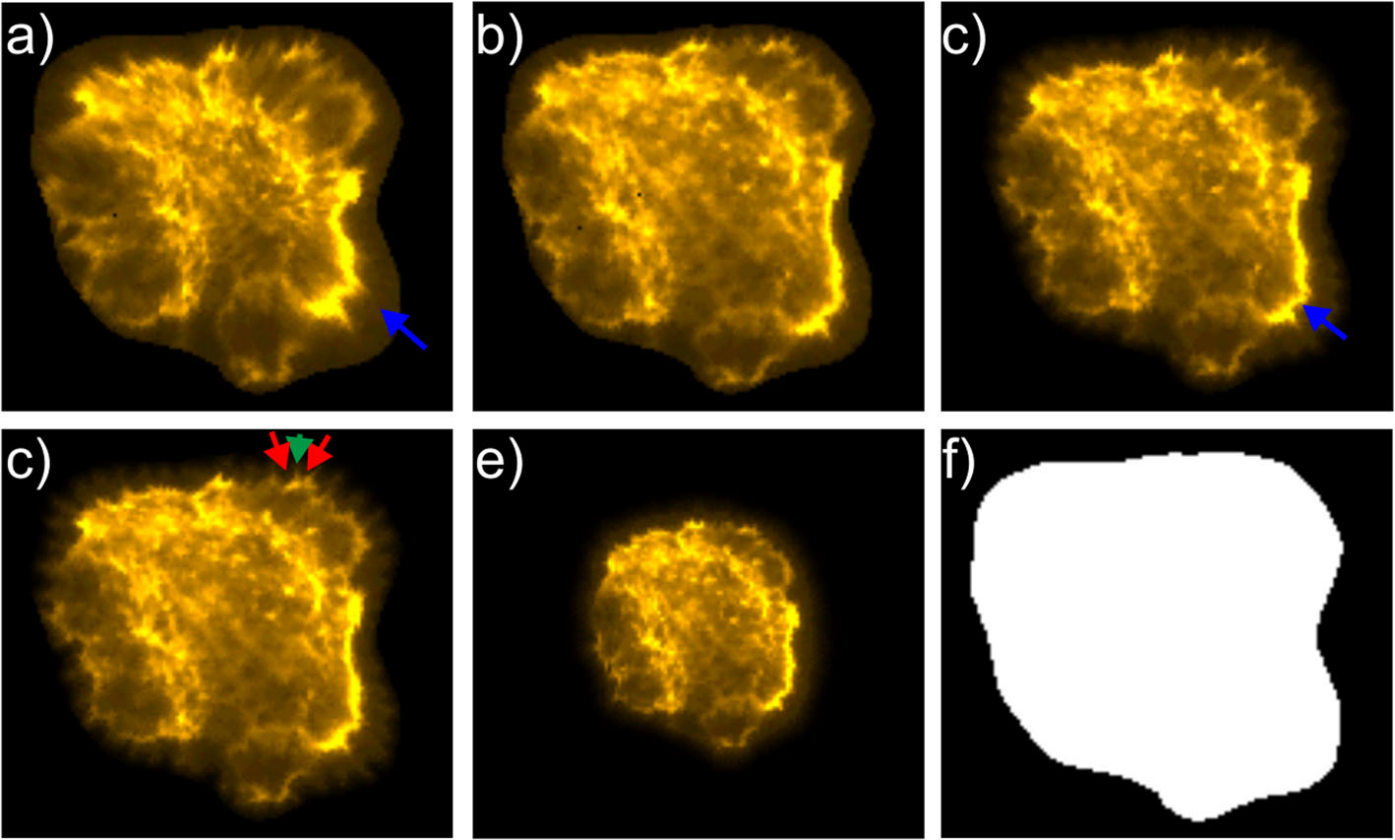
Visualization of the effect of forces employed in the energy mapping approach for a exemplary B cellcytoskeleton. The different textures in **a**) and **c**) - **e**) show textures with varying settings compared to texture **b**) simulated with settings used for evaluation: **a**) weaker force between bulk points resulting in stronger distortion of areas with high intensity indicated by *blue arrow*; **c**) stronger force between bulk points resulting in smaller areas with high intensity indicated by *blue arrow*; **c**) weaker force between border points resulting in serrated contour (corresponding point indicated by *green arrow* surrounded by serrated areas with border points indicated by *red arrows*); **e**) weaker force between corresponding points resulting in smaller texture size

The minimization process is conducted with a gradient descent algorithm [24]. The points of the real texture are expanded iteratively to the new cell shape by applying forces according to the energy terms. The weights *w*_fix_, *w*_border_ and *w*_bulk_ have to be adapted for each cell and texture type. The forces corresponding to the energy equation are illustrated in Fig. 2 and their effects on the texture are shown in Fig. 3. The forces corresponding to the energy terms *E*_border_ and *E*_fix_ are proportional to the distance between the point and its neighbors and act like springs.

The force ***f***_bulk_ corresponding to the energy term *E*_bulk_ uses spring constants depending on the intensity of adjacent pixels according to the following equation

$$\boldsymbol{f}_{\text{bulk}}(\boldsymbol{p})=\sum_{\boldsymbol{n}\in {N}} log\left(1+I\left(\boldsymbol{p}\right)\right) log(1+I(\boldsymbol{n}))*\left(\boldsymbol{p}-\boldsymbol{n}\right) $$

where ***p***=(*x*_*p*_,*y*_*p*_)^*T*^ is the currently processed pixel, ***n***=(*x*_*n*_,*y*_*n*_)^*T*^ the adjacent neighbor in the 8-connected neighborhood N and *I*(***p***) the intensity of corresponding pixels in the texture. During the energy minimization process, the points in the texture are processed in random order. The forces applying at the current point are summed up and the point is then moved a limited distance according to the force. After convergence of the energy minimization empty pixels in the new cell shape are filled by interpolation.

#### Cell location simulation

To determine the location of *N*_*c*_ cells on an image, we assign each cell to a defined number of cell clusters *N*_*cluster*_ and arrange each cell regarding a defined maximum cell overlap *J*_*max*_ in the respective cluster.

In a first step, we generate *N*_*clusters*_ randomized coordinates to determine the center of each cell cluster. Then all *N*_*c*_ cells on an image are subsequently processed. The current cell is assigned randomly to a certain cluster. If this cell is the first cell in a cluster, the cluster center is determined as the cell location. Each further cell in a cluster is arranged at a final location regarding the maximum overlap *J*_*max*_ with the cells which have already been arranged in this cluster. To determine the final location, the cell is initially placed at the cluster center and a direction of movement is randomly generated. Then the cell is incrementally moved along the random direction while the overlap to adjacent cells *J*_*cur*_ is calculated with the Jaccard index. The final location is reached when the overlap to adjacent cells *J*_*cur*_ drops below the maximum overlap *J*_*max*_. These steps are repeated until a location has been assigned to each cell.

#### Cell image generation

The simulation algorithms presented above are integrated into a common software framework [11]. The framework implements a pipeline for fluorescent cell image simulation within several steps. First steps simulate the cell shape and texture. Then the cells are positioned on the image. In the last steps, artifacts and noise are added to the simulated images. For this paper, we used the newly developed methods to simulate cell shapes and textures. The cells are positioned in a fixed amount of clusters located at randomized positions on the image. Background artifacts were simulated with Perlin noise [25]. In a last step Gaussian noise was added to the images.

To simulate fluorescent images for the evaluation of simulation methods, parameters of the simulation algorithms have been separately adapted to reproduce the visual appearance of real microscopic images from the four segmentation tasks described in the past section. These parameters are then used to simulate four data sets with 30 microscopy images for each segmentation task. These data sets are later referred to as data sets reproducing realistic overlap.

In order to examine the influence of overlapping cells with respect to graphical image properties, as well as the segmentation performance, we additionally simulate a sequence of data sets for each of the four tasks where all simulation parameters are kept constant except the parameters to steer the cell overlap. All cells on each image are located in a single cluster. Then the cell overlap is varied in four degrees, namely from isolated over touching to overlapping and finally overlying cells. We simulated 30 images per segmentation task and degree of cell overlap.

### Evaluation

Two types of evaluations were applied. First, we evaluate the visual appearance of the simulated fluorescent cell micrograph with individual ratings obtained from a group of life-science- as well as computer science experts. Secondly, we evaluate the synthetic fluorescent micrograph cell data with graphical image features. After validation we use the simulated cell images for evaluation of commonly used segmentation pipelines for cell segmentation.

#### Simulation: expert observer study

For this study we have prepared image sets of fluorescent micrographs for all four tasks described above. Each image set contained ten real images, ten images simulated with our approach and furthermore ten micrographs simulated with the *SimuCell* software tool [10] as reference. The images simulated with our own approach were randomly chosen from the available data sets with realistic overlap. For the images simulated with the *SimuCell* tool, the parameters were adapted in such a way that simulated images have the most likely appearance for the corresponding data set. Experts from biology and micro-biology visually assessed all four image data sets of 3 ×10 fluorescent micrographs and rated the realism with respect to the expected and known biological properties. Experts from image-processing domain rated the same images with respect to the graphical properties. This means the image processing experts search for image distortions like simulation artifacts. Both groups rated the images on a scale from 0 to 5 with “0” being “confidently simulated” and “5” being “confidently real”. The four image sets were shown in sequence, but for each image set the real and simulated micrographs were presented in random order. A LCD-TV (Phillips 52PFL96320/10) was used to display each single micrograph for 20 seconds, alternating with a dark gray screen depicted for two seconds. All observers noted the rating of the observed images on a questionnaire. After each one of the four image sets the observers took a break of five minutes.

#### Simulation: objective measurements

In literature (as e.g. [26]), various objective measures for the description of image properties have been proposed. Some of these measures have e.g. been applied in [16] for the comparison of real and simulated images cells. In this work we try to address and evaluate image properties with high influence on the segmentation quality as well as on human visual perception. Thus, we calculate the average intensity inside the cell shape and the edge strength. Specifically, the edge strength is computed with the summed mean difference (SMD) method

$$\text{SMD}=\frac{1}{N_{C}}\sum\limits_{\boldsymbol{c}}^{N_{C}} \frac{1}{N_{c}} \sum\limits_{\boldsymbol{p}_{c}}^{N_{c}} \text{abs}(I(\boldsymbol{p_{c}}) - A_{c}) $$

where *N*_*C*_ is the the number of cells, *N*_*c*_ the number of pixels in cell *c*, *I*(***p***_*c*_) the intensity of pixel ***p***_*c*_ and $A_{c}=\frac {1}{N_{c}} {\sum \nolimits }_{\boldsymbol {p}_{c}}^{N_{c}} I\left (\boldsymbol {p_{c}}\right)$ the average intensity of cell *c*.

#### Segmentation: evaluation of cell segmentation algorithms

In order to proof that the fluorescent micrographs simulated with our approach are suited for the evaluation of cell segmentation algorithms, we examine the influence of increasing overlaps between adjacent cells to the segmentation performance of a state of the art image processing pipeline for fluorescent image segmentation. For each of the four fluorescent image data sets described above (protoplasts in FDA staining, B cells in DAPI and Rhodamin stains, macrophages with CD11b/APC stains) we simulated five data sets with increasing cell overlap including one set with the same degree of overlap measured on corresponding original image data set. To measure the degree of overlap, human experts annotated the original data sets including the overlaps between adjacent cells. The Jaccard index describing the degree of overlap is calculated based on these expert annotations and then input to the simulation.

**Fig. 4:**
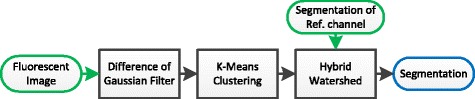
Typical segmentation pipeline for fluorescence cellular micrographs

Figure 4 shows a state of the art image-processing pipeline conventionally used for the segmentation of cellular fluorescent micrographs [19]. For preprocessing and conditioning a *Difference of Gaussian* (DoG) filter with parameters *σ*_high_ and *σ*_low_ is applied; a *k-means clustering* approach [19] with parameter *k* for the number of clusters is used for figure-ground separation. For the separation of adjacent cells a a *hybrid watershed* [27] approach is used for protoplasts and the B cell nuclei. For the B cell cytoskeleton and macrophage cyto-membrane *seeded watershed* approach [28] with nuclei position from DAPI channel is performed. The watershed algorithms have a parameter *w* for weighting edge-strength against the distance transform, a parameter *σ*_ws_ for edge calculation with a derivative of Gaussian filter and the parameter *a*_min_ describing the minimum cell size.

For all examined data sets these parameters are automatically optimized by a coordinate descent algorithm to the simulated ground truth data with respect to the combined Jaccard metric. Further details are layed out in [19]. All results are validated using a three-fold cross-validation.

To avoid a bias during evaluation of cell segmentation, a second segmentation pipeline presented in the Additional file 4 has been applied for the segmentation of the synthetic images.

## Results

In the following section, we present the results obtained from the observers study evaluating and graphical image features of the simulated images in comparison to real microscopic images. Furthermore, we present segmentation results of the simulated fluorescent micrographs obtained with a state-of-the-art image-processing pipeline.

### Simulated fluorescence images

**Fig. 5:**
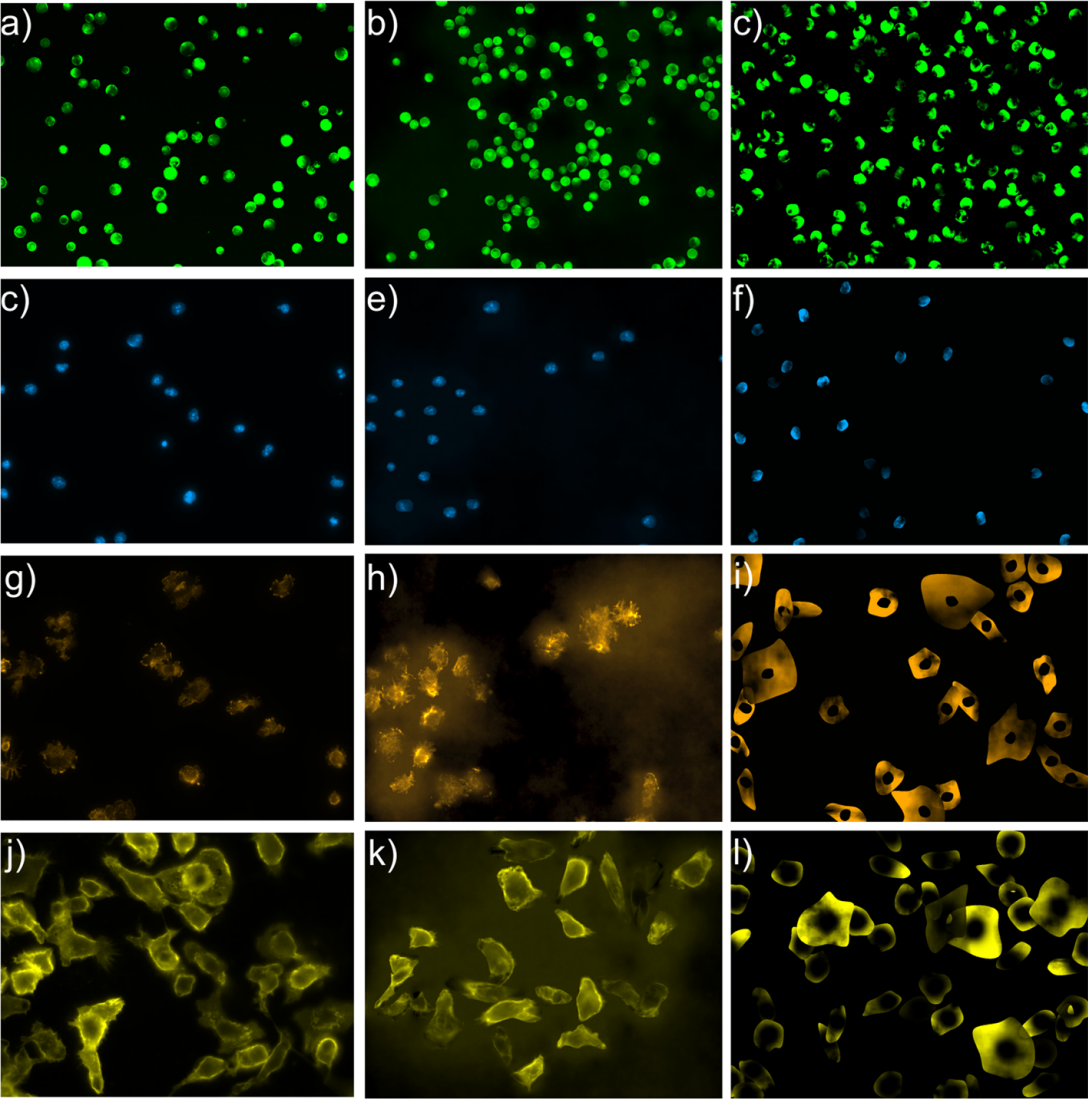
Comparison between real and simulated images: **a**), **b**), **c**) protoplast; **d**), **e**), **f**) DAPI stained nuclei; **f**), **g**), **h**) F-Actin channel of B cells; **i**), **j**), **k**) macrophages; **a**), **d**), **f**, and **i**) are real images, **b**), **e**), **g**), and **j**) are simulated with our approach while **c**), **f**), **h**), and **k**) have been simulated with *SimuCell*

Figure 5 shows fluorescent micrographs simulated with our approach described above, using the basic data sets as input data for shape and texture modeling. Furthermore, Fig. 5 depicts fluorescent micrographs rendered with the freely available *SimuCell* software tool which is based on computer graphic methods. The software allows parameters to be controlled on simulated images, such as the amount of cells per field-of-view, the cell distribution and the cell overlap. The data sets simulated by our approach are provided as additional files named Additional files 5, 6 and 7. The images simulated with *SimuCell* can be found in the Additional file 8.

### Simulation: expert observer study

**Fig. 6:**
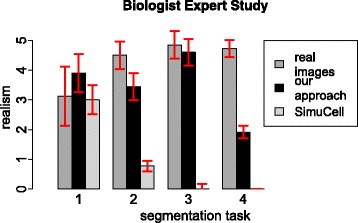
Review of graphical image properties by life scientists. The segmentation task corresponding to: (1) protoplasts, (2) B cell nuclei, (3) B cell cytoskeleton and (4) macrophages. Error bars represent the corresponding standard deviation

**Fig. 7:**
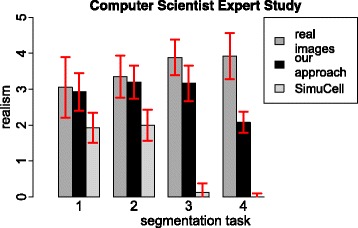
Review of graphical image properties by computational scientists. The segmentation task corresponding to: (1) protoplasts, (2) B cell nuclei, (3) B cell cytoskeleton and (4) macrophages. Error bars represent the corresponding standard deviation

Results of our expert observer study are presented separately for biologists in Fig. 6 and for image processing experts in Fig. 7. While the image processing experts only had a short introduction on how the fluorescent cell images look like, the graphs shows similar characteristics for both groups. The protoplasts received a medium rating but with higher standard deviation compared to other cell types. The B cell nuclei and the B cell cytoskeleton images received high ratings of realism for real images and images simulated with our approach. The ratings for the images simulated with *SimuCell* lie in the range of ratings for simulated images ranged equal and smaller to 2. For the macrophages, both user groups can distinguish between simulated and real images. As the experts can determine the images simulated with *SimuCell* as simulated, further evaluations are only done with images simulated with our approach.

### Simulation: objective measurements

To determine how an increasing overlap between adjacent cells influences the image properties, we measured the intensity and the SMD within the cells on the simulated micrographs. The intensity measurements are shown in Table 1. For all cell types (protoplasts, B cells, macrophages) the mean intensity inside the cell contour increases within cells with a rising overlap. Table 2 shows that this observation is not as clear for the SMD measurements. The average SMD values increase for protoplast with increasing overlap, but decrease for B cell nuclei, B cell cytoskeleton and the macrophages with increasing overlap. For all types of cells, the fluorescent micrographs simulated with parameters (amount of cells and overlap factor) reproducing properties of real images show a lower intensity and lower SMD inside cells. Nevertheless, the differences are less than 0.387 *σ* for the intensity measurement and hence acceptable. The difference for B cell nuclei is 1.58 *σ*, while for other cell types it is less than 0.46 *σ*.

**Table 1:** Average mean intensity measured on simulated and real fluorescent cellular micrographs

Intensity	Not touching	Touching	Overlapping	Overlaying	Realistic overlap	Real image
protoplasts	149,55	152,41	156,93	173,65	150,16	156,26
nuclei	105,93	105,90	108,06	115,86	105,16	107,13
B cells	27,22	27,13	27,76	30,39	28,04	30,77
macrophages	74,98	72,48	74,25	80,46	71,41	80,67

**Table 2:** Average summed mean differences (SMD) on simulated and real fluorescent cellular micrographs

smd	Not touching	Touching	Overlapping	Overlaying	Realistic overlap	Real image
protoplasts	19,02	18,96	18,96	17,75	19,14	22,66
nuclei	16,31	16,34	16,46	16,30	16,27	13,05
B cells	4,82	4,88	5,03	5,55	5,07	5,06
macrophages	8,14	7,79	8,22	8,93	7,66	7,48

### Segmentation: evaluation of segmentation pipeline

**Fig. 8:**
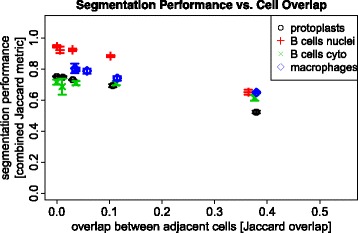
Visualization of segmentation results obtained on series of data sets showing cells with increasing overlap and the data sets with realistic overlap for all four segmentation tasks. Error bars represent the corresponding standard deviation

Figure 8 visualizes the segmentation performances obtained with the state-of-the-art cell segmentation pipeline (described above) for fluorescent cellular micrographs on the simulated images. For all data sets (protoplasts, B-cells, macrophages) the segmentation performance decreases with increasing overlap. Performance measurements using the same image segmentation pipelines shows a combined Jaccard index of 0.74 on images simulated with our approach compared to 0.62 on the corresponding real images. A segmentation performance of 0.92 was obtained on simulated images with the B cell nuclei and Wiesmann et al. [20] calculated a performance of 0.73 on real images. For B cell cytoskeleton, we obtained 0.68 on simulated images and again Wiesmann et al. [20] reached 0.64. We calculated a segmentation performance of 0.78 on simulated macrophages images in comparison to 0.688 on real images of Held [19]. Hence, the segmentation performance values obtained over all simulated data sets are higher than on real images.

## Discussion

### Simulated fluorescence images

While the simulation of fluorescent cellular micrographs (or in the large scale of any other micrograph) gives the possibility to control important parameters in the resulting images, such as the number of cells, cell distribution in the field-of-view, and the cell overlap, still some of the parameters depend on the quality of the input image data used for cell shape and texture modeling. Thus, image artifacts resulting from various sources during the imaging process cannot be easily removed from the images. Slide preparation leaves dye or cell fragments in the background. Illumination artifacts may result from microscopy and noise may be present in real images especially on images taken from cells marked with fluorescent makers with long wavelength.

In the following we discuss some solutions to these problems. The simulated fluorescent images contain an equal or higher background gray value level compared to the original micrographs, while otherwise simulated images would depict an unnatural edge between the cytoplasm and the image background. Nevertheless, the higher background levels increase segmentation complexity as the figure-ground separation is usually based on this contrast. Illumination artifacts in the input images used for cell modeling can be estimated with retrospective shading correction [29] but not fully removed form input images. If a retrospective illumination correction is performed before using the cell data for texture simulation, the resulting correction artifacts propagate to simulated images. The minimum noise level of the micrograph can be determined by the noise on the cells in the input images. The noise existing on the images, and on which the simulation is based on, can not be removed without producing artifacts that can be recognized by human observers. Higher noise levels can be simulated by adding additional noise. Thus, an input data set with high quality is mandatory to simulate high quality images.

### Simulation: expert observer study and objective measurements

The proposed texture simulation algorithm reduces distortion of regions with high intensity. In image data sets with high cell shapes variability, e.g. in macrophage cell spreading experiments, the proposed approach reaches its limit when relatively small cell patches are mapped to relatively large shapes. In these cases, the texture mapping algorithm has to stretch the patch strongly which leads to stronger distortions.

This also implicates that some of the simulated images depict reduced intensities inside the cells compared to the cells on real images (see Table 1). This also coincides with lower SMD values (see Table 2).

Table 1 shows an increasing mean intensity in the cells with an increasing overlap factor or cells with adjacent cells. This phenomenon was to be expected, because in the overlapping regions the fluorescent intensities of both cells are additive. The SMD values of the edges also rise with increasing overlap. Here, we also observe an additional contribution in regions where cells overlap, since we did not explicitly blur the texture of cells lying in the background. This observation can be used to extend the cell simulation model in a next step to generate even more realistic fluorescent micrographs.

During the observer study most of the biologists looked distinctively at cells and made their decision based on the appearance of the cells in order to decide if the image could be real or not. On the other hand, the image processing experts and computer scientist searched for simulation artifacts and for unnatural edges, repetitive structures and distortions in the cell textures. Together, both groups cover a wide set of image appearance properties, thus supporting the hypothesis that the proposed fluorescent micrograph simulation delivers more realistic images than the compared computer graphics approach.

For all assessed cell types (protoplasts, B cells, macrophages), the observed realism for the *SimuCell* images is lower compared to real fluorescent micrographs as well as fluorescent micrographs simulated based on our proposed approach using shape and texture modeling. The cell texture of images simulated with the *SimuCell* software is generated with Perlin noise [25] and therefore appears homogenous for all cells depicted in the simulated images set.

Protoplasts show average rating for realism with large standard deviation for all images. The experts, both biologists and computer scientists, were not able to distinguish between real images and simulated images and assigned ratings for realistic images to simulated images and ratings for simulated images to realistic images.

For both B cells compartments, namely the nuclei and the cytoskeleton, both expert groups assigned high realism ratings for real images and images simulated with our approach. The ratings for the images simulated with the *SimuCell* tool were significantly reduced. In contrast to the protoplast images the experts of both groups could clearly determine the images provided by the *SimuCell* tool as “simulated". All experts tend to rate real images and images simulated with our approach with higher ratings of realism.

The ratings for the macrophages show, that the experts were able to distinguish between simulated and real cells and also between simulated images from our approach and the *SimuCell* tool. This shows that the proposed approach currently comes to its limits when cells with highly variable shapes are simulated. This problem could be solved in a next development step if manually annotated cells are grouped according to their shape and then multiple cell shape models are calculated, each of them with less variability.

### Segmentation: evaluation of segmentation pipeline

The performance values (cf. Figure 8) obtained from the cell-segmentation experiments show that the images simulated for this study with our approach can be segmented in suitable quality by the state-of-the-art image processing pipeline presented above. Overall, the performance values on four experimental data sets and the related segmentation tasks, are higher on the simulated images than on the real images. This can be explained based on the fact that the simulation models depict less image disturbance than the real images. Also, the simulated fluorescent micrographs depict less artifacts such as cell fragments or dye blobs, which can have a high impact on the segmentation performance. Additionally, the evaluation of the original microscopy images based on human expert annotation can impair the segmentation accuracy. Human observers visually assess the cells on the microscopy images and we use this as the gold standard but the real ground truth remains unknown. The visual assessment is a subjective task and different observers create different annotations of the same micrographs. This fact weakens the validity of the evaluation and was one of the reasons reason to simulate fluorescence micrographs with a well-known ground truth.

A state-of-the-art segmentation pipeline (consisting of DoG filtering, k-means clustering and watershed-approaches) has been applied to the series of simulated image data sets with increasing overlap of adjacent cells. As this segmentation pipeline is only able to separate touching cells, the performance decreases with a rising overlap factor.

Usually, data sets which are used to evaluate segmentation algorithms, depict cells that overlap or overlay each other. Nevertheless, this fact is often omitted in the evaluations of segmentation algorithms and the resulting performance measure is calculated as mixture of cells with different overlap. Thus, the performance values obtained on the various simulated data sets give a coarse estimate of the segmentation quality to be expected on new input data sets with various degrees of overlap.

Among the examined examples, the B cell nuclei can be segmented and separated with the highest performance, even when they overlap. This results from their high intensity which enables exact figure-ground separation, and from their convex shape which is preferred by the watershed algorithms used for cell separation. The second-best performance was obtained on the data set with macrophages which depict sufficient intensity for figure-ground separation, but a much higher variability on the cell shapes. On the image data set with protoplasts, the observed segmentation performance decreases because they show high intensity variations and thus the figure-ground separation is not consistently feasible with the k-means clustering algorithm. Errors obtained in this step directly propagate to the successive cell separation step and hence lead to a lower performance. The segmentation pipeline yields the lowest performance on the micrographs with the B cell cytoskeleton. This data set shows high a intensity variability inside the cells inhibiting exact figure-ground separation. Additionally, the cell shape has a high variability so that the edges of the cells in the images would be needed for the separation step. As Table 2 shows, the edge strength is weak for the B cells, thus preventing an accurate segmentation of the images.

## Conclusion

We have presented and validated a new approach for realistic fluorescent cell image simulation in order to evaluate cell segmentation algorithms. Ratings from an expert observation study show that the simulated images cannot be easily distinguished from real images by either biologists or image processing experts. Additionally, graphical image features of cells lie in the same value range for cells on real images and cells in micrographs simulated with our proposed approach. Furthermore, we determined the limits of our approach on image sets with high shape and texture variability. A work around lies in clustering the cells before starting with simulation.

Fluorescent cellular image simulation enables the evaluation of cell segmentation algorithms that are independent from various image disturbances. If necessary, these disturbances can also be included in the simulation and their influence to the segmentation performance can be measured.

In particular, the presented method allows for the objective evaluation of image processing algorithms trying to resolve overlaps of overlapping and overlaying cells. To gain an adequate ground truth based on expert annotation is time consuming and error prone, especially for overlapping and overlaying cells. Fortunately, a simulation inherently provides its own ground truth, even for difficult scenarios due to overlapping or overlaying cells.

Further improvement of the simulation can be achieved by optimizing the arrangement of the cells on the images.

## Additional files

Additional file 1 The zip archive contains real images showing protoplasts. (ZIP 4 kb)

Additional file 2 The zip archive contains real images showing B cell nuclei and cytoskeleton. (ZIP 12390 kb)

Additional file 3 The zip archive contains real images showing macrophages. (ZIP 28979 kb)

Additional file 4 A second image segmentation pipeline was applied to the images. The results and conclusions for this second pipeline are described in the pdf-file. (PDF 1423 kb)

Additional file 5 The zip archive contains simulated images showing protoplasts with corresponding ground truth. (ZIP 72704 kb)

Additional file 6 The zip archive contains simulated images showing B cell nuclei and cytoskeleton with corresponding ground truth. (ZIP 119808 kb)

Additional file 7 The zip archive contains simulated images showing macrophages with corresponding ground truth. (ZIP 55091 kb)

Additional file 8 The zip archive contains all files simulated with *SimuCell*. These images have been used during the expert observer study. (ZIP 7618.56 kb)

## Abbreviations

ASM: Statistical shape model
DoG: Difference of Gaussian
FDA: Fluorescein diacetate
PCA: Principle component analysis
SMD: Summed mean difference
