# Supplementary figures and images for: Using simulated fluorescence cell micrographs for the evaluation of cell image segmentation algorithms (part 1 of 6)
Source: BMC Bioinformatics. 2017 Mar 18;18:176. doi: 10.1186/s12859-017-1591-2 (PMC5357336; doi:10.1186/s12859-017-1591-2)

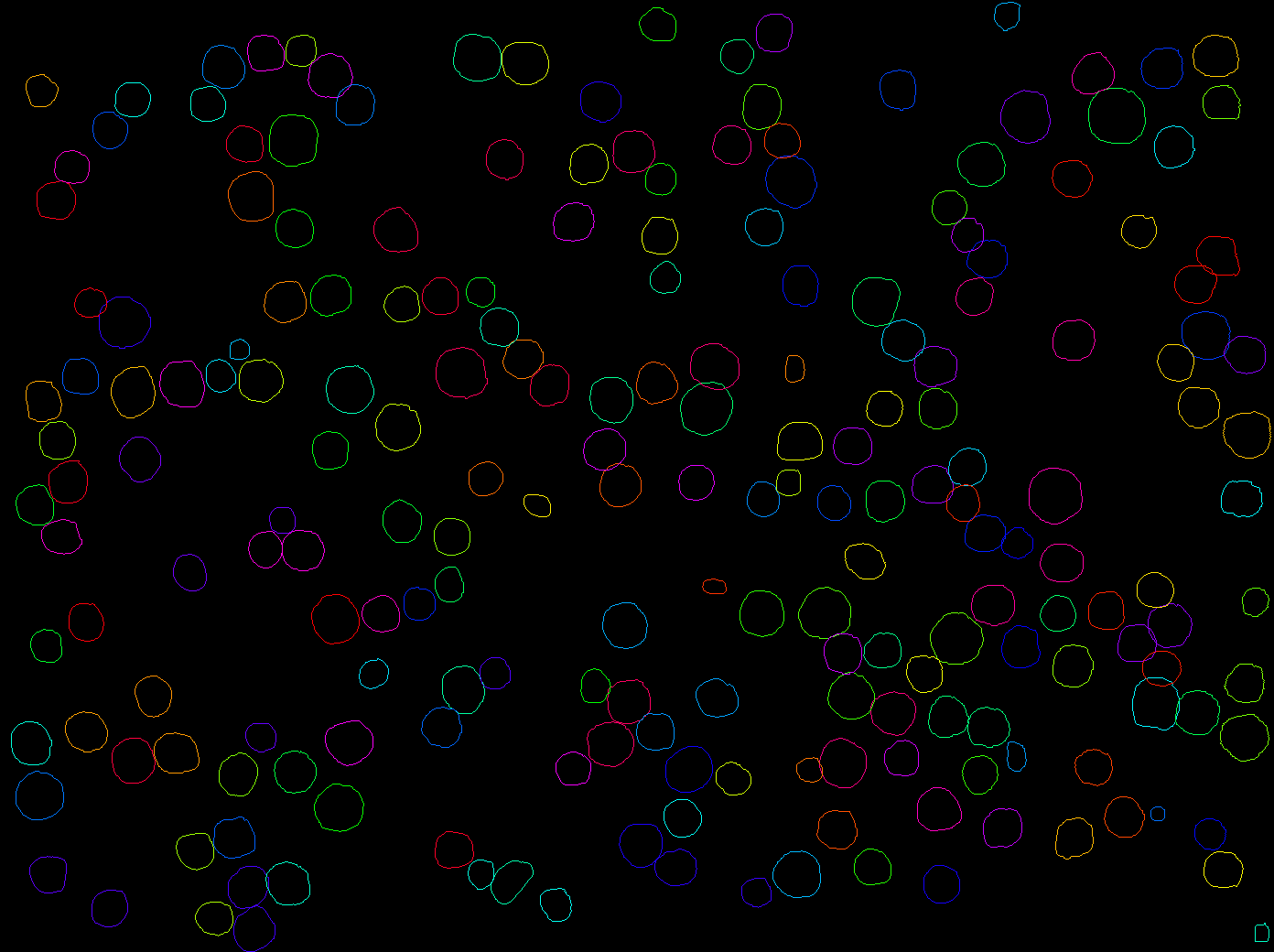

Supplement: Additional file 1 — The zip archive contains real images showing protoplasts. (ZIP 4 kb) [file 12859_2017_1591_MOESM1_ESM.zip › protoplasts/20111129-02A (2) gt.png]

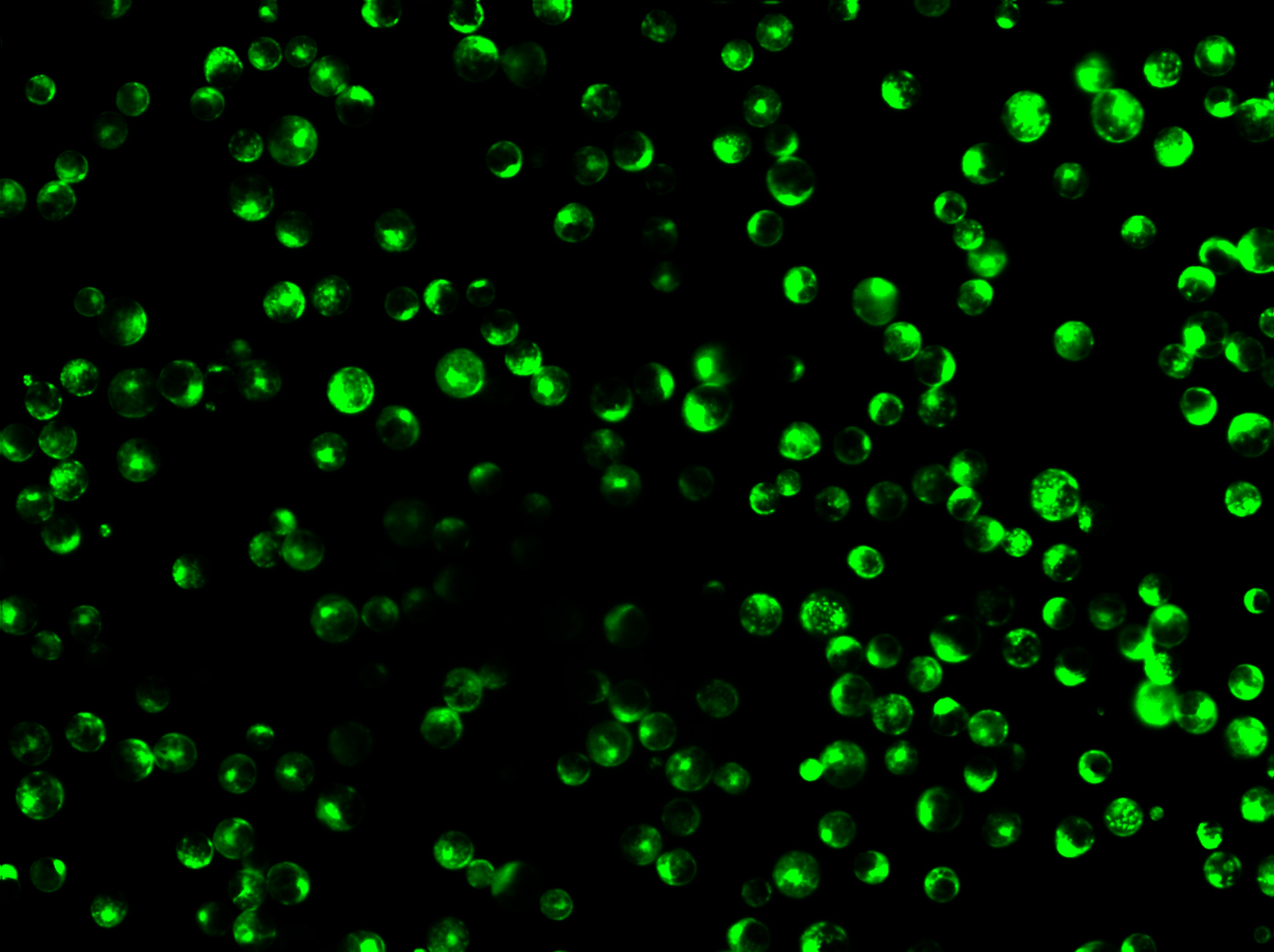

Supplement: Additional file 1 — The zip archive contains real images showing protoplasts. (ZIP 4 kb) [file 12859_2017_1591_MOESM1_ESM.zip › protoplasts/20111129-02A (2).png]

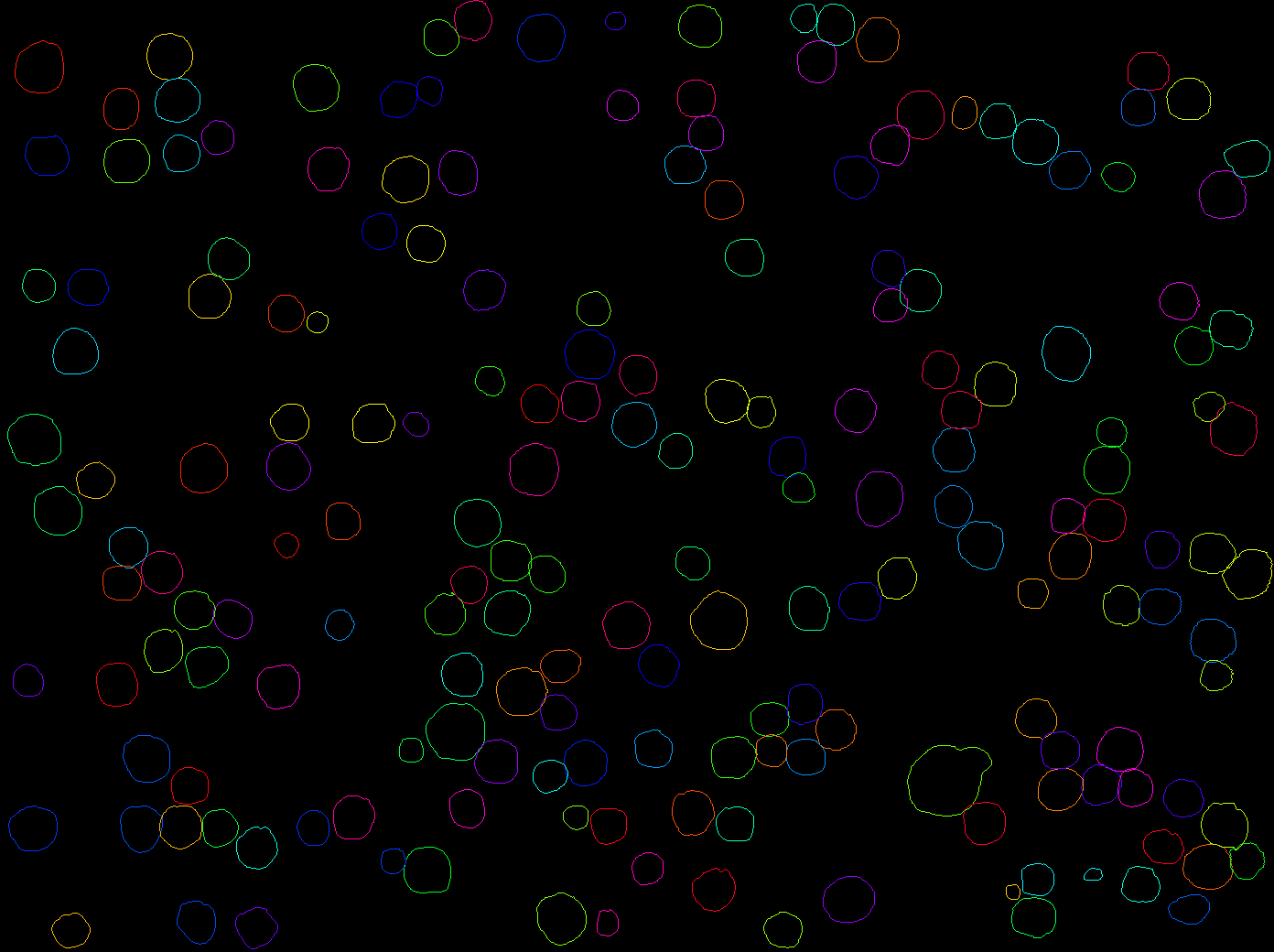

Supplement: Additional file 1 — The zip archive contains real images showing protoplasts. (ZIP 4 kb) [file 12859_2017_1591_MOESM1_ESM.zip › protoplasts/20111129-02B (2) gt.png]

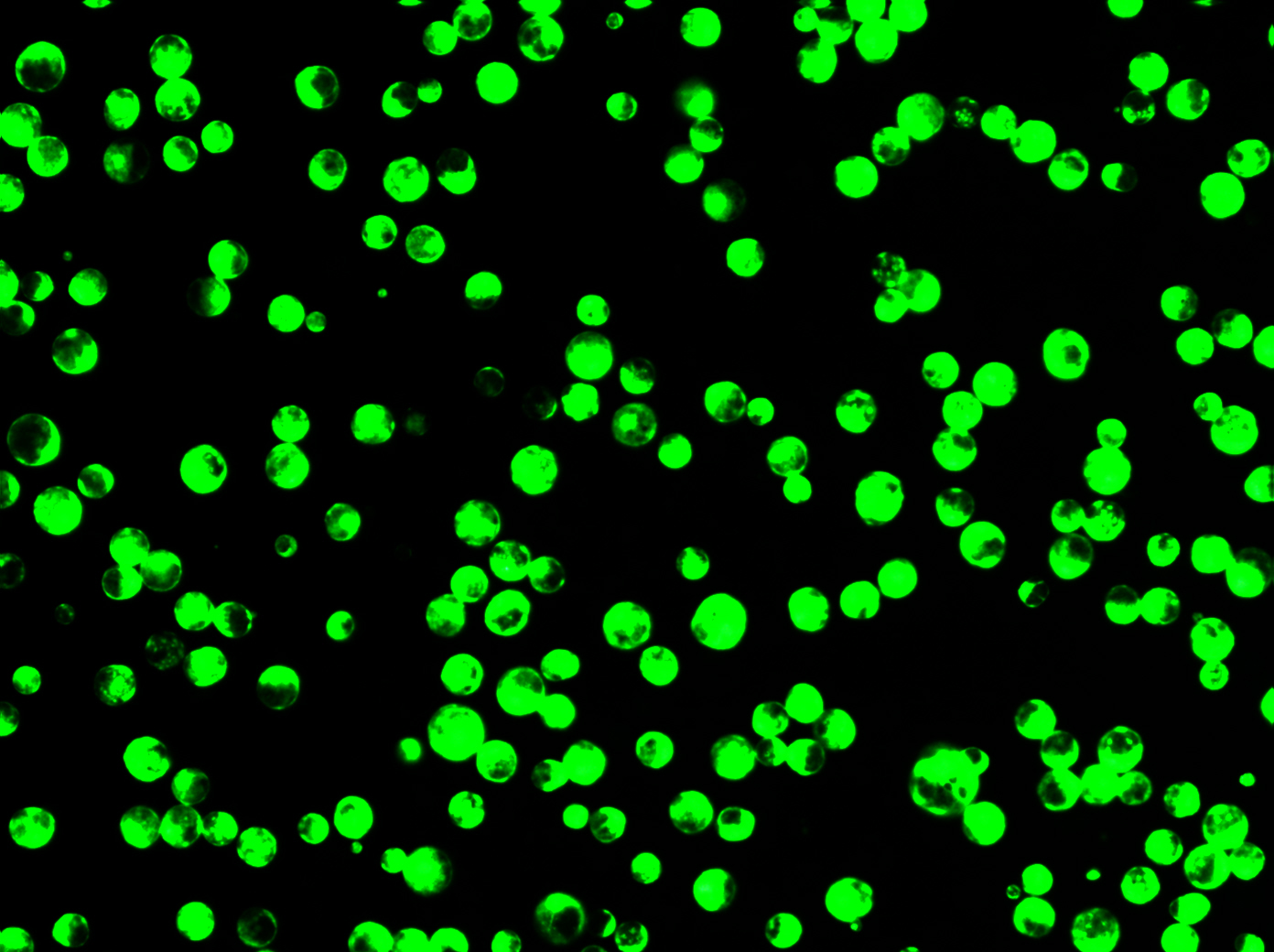

Supplement: Additional file 1 — The zip archive contains real images showing protoplasts. (ZIP 4 kb) [file 12859_2017_1591_MOESM1_ESM.zip › protoplasts/20111129-02B (2).png]

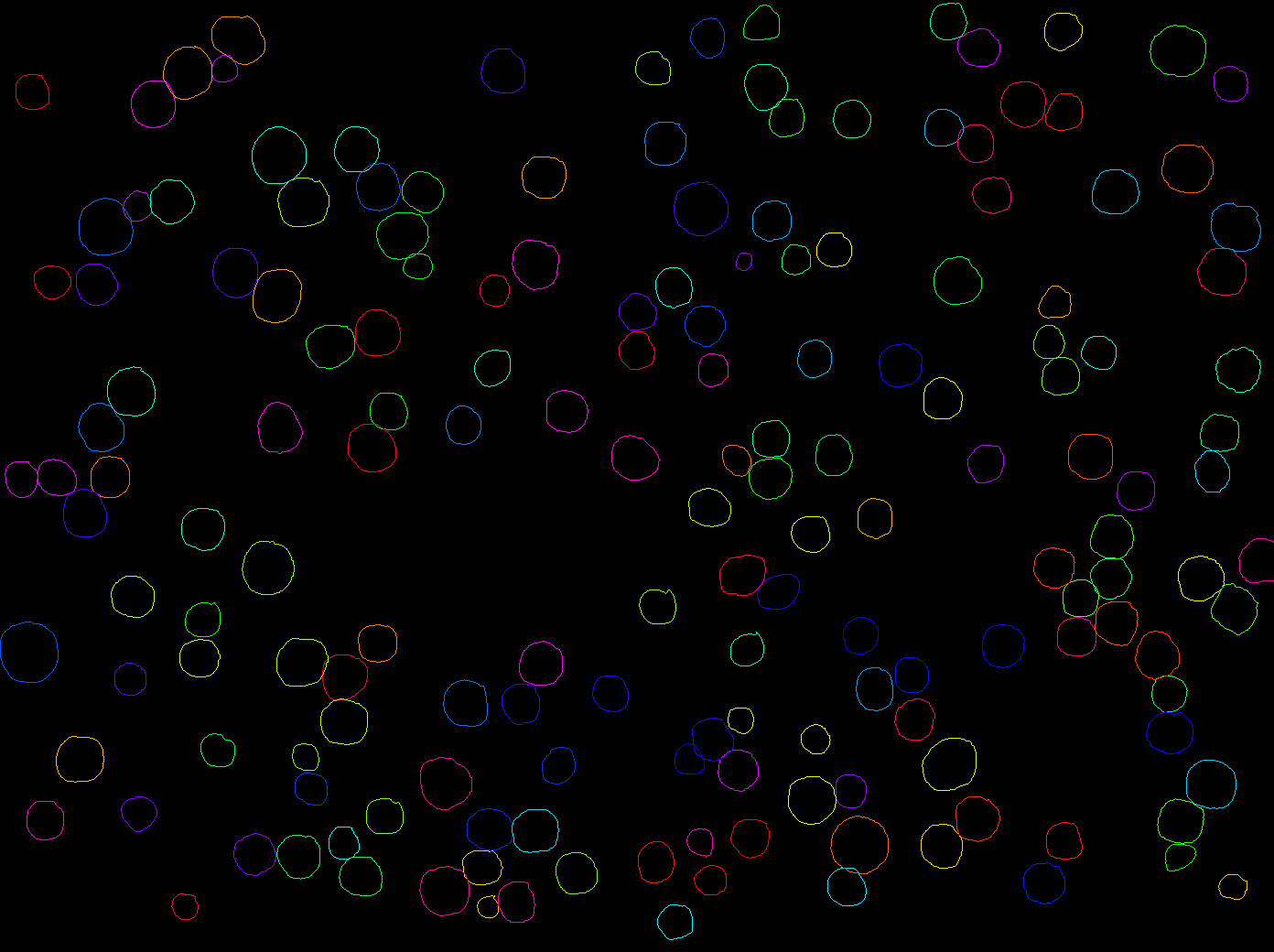

Supplement: Additional file 1 — The zip archive contains real images showing protoplasts. (ZIP 4 kb) [file 12859_2017_1591_MOESM1_ESM.zip › protoplasts/20111129-03A (2) gt.png]

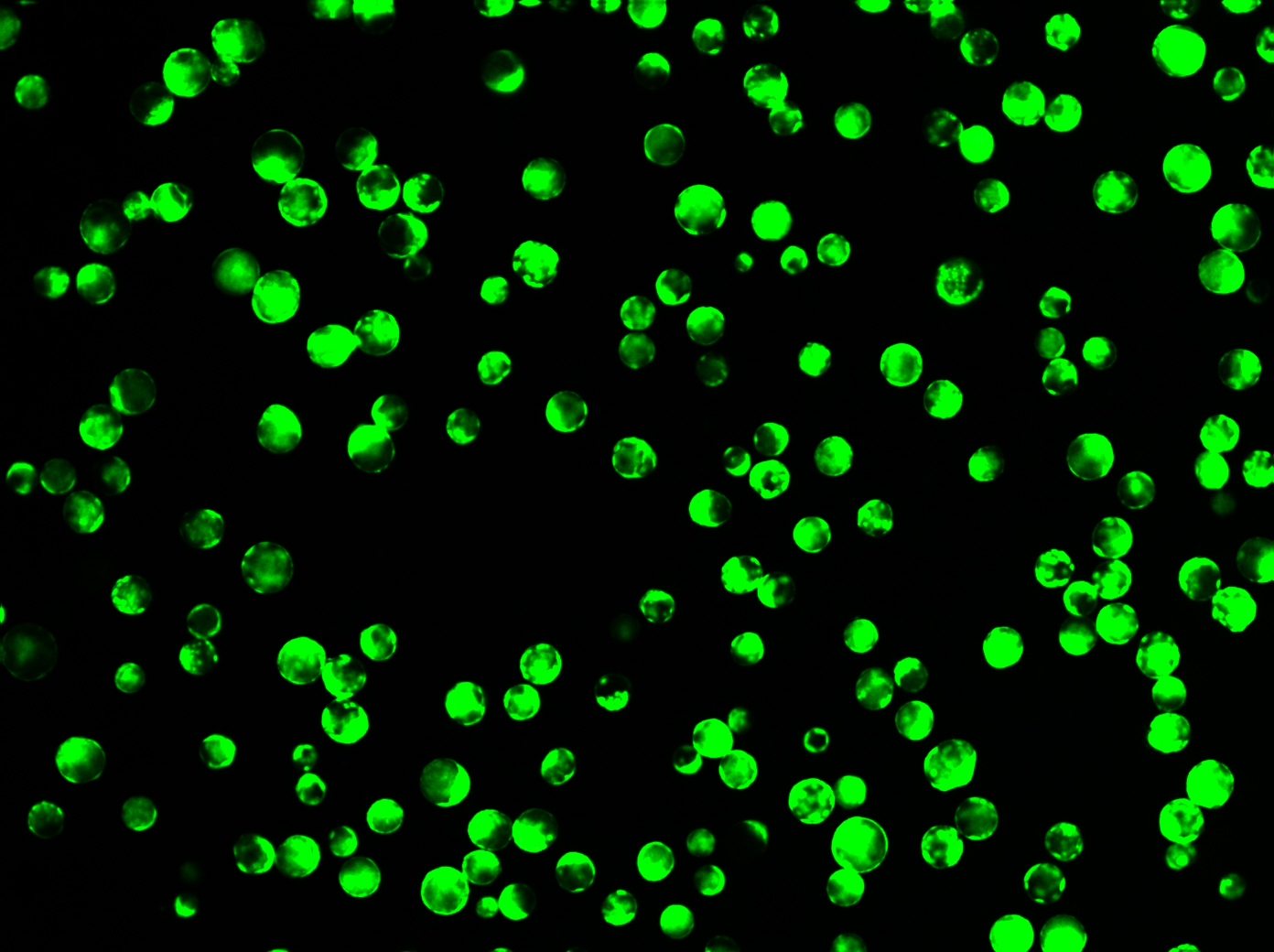

Supplement: Additional file 1 — The zip archive contains real images showing protoplasts. (ZIP 4 kb) [file 12859_2017_1591_MOESM1_ESM.zip › protoplasts/20111129-03A (2).png]

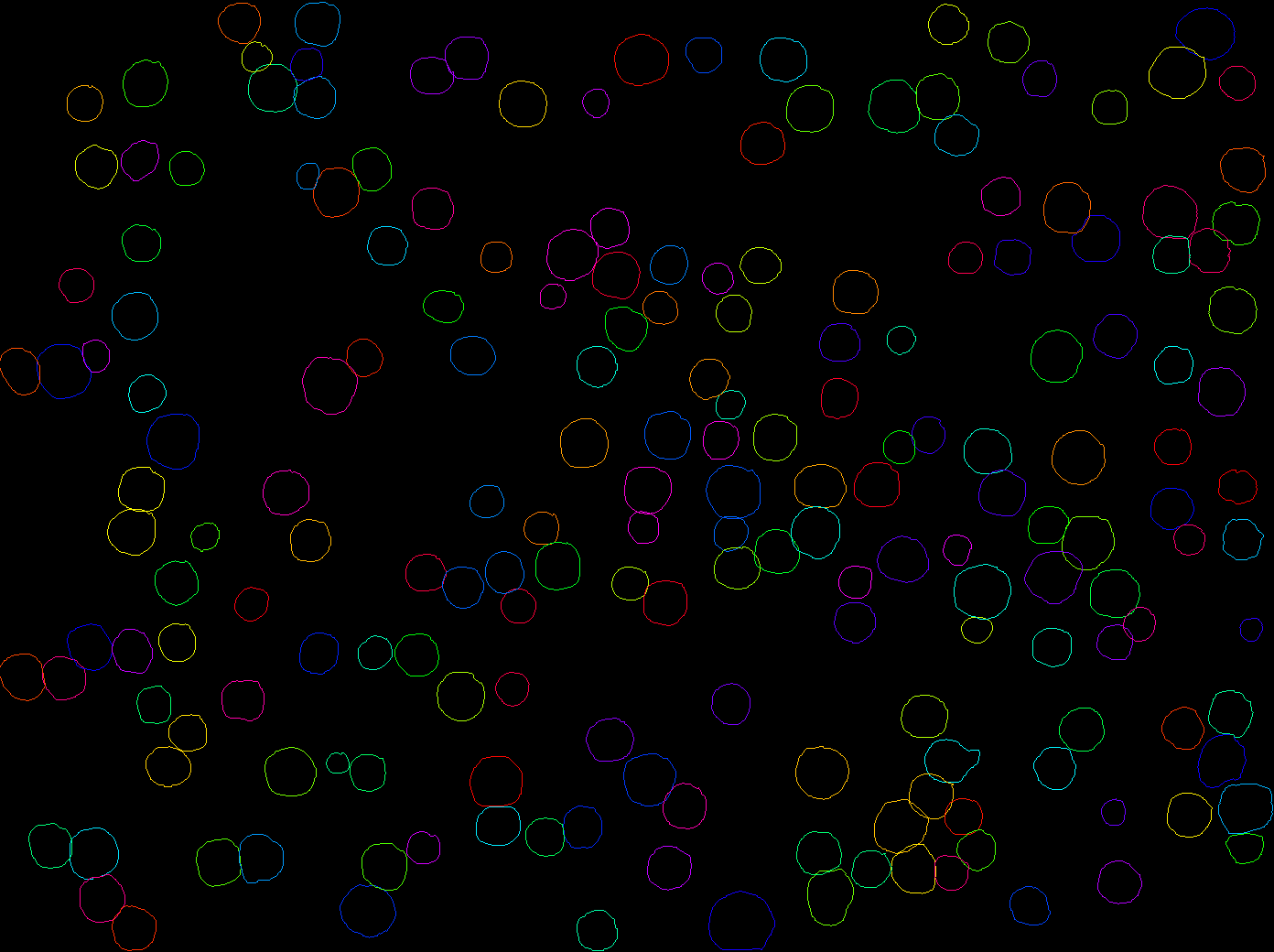

Supplement: Additional file 1 — The zip archive contains real images showing protoplasts. (ZIP 4 kb) [file 12859_2017_1591_MOESM1_ESM.zip › protoplasts/20111129-03B (2) gt.png]

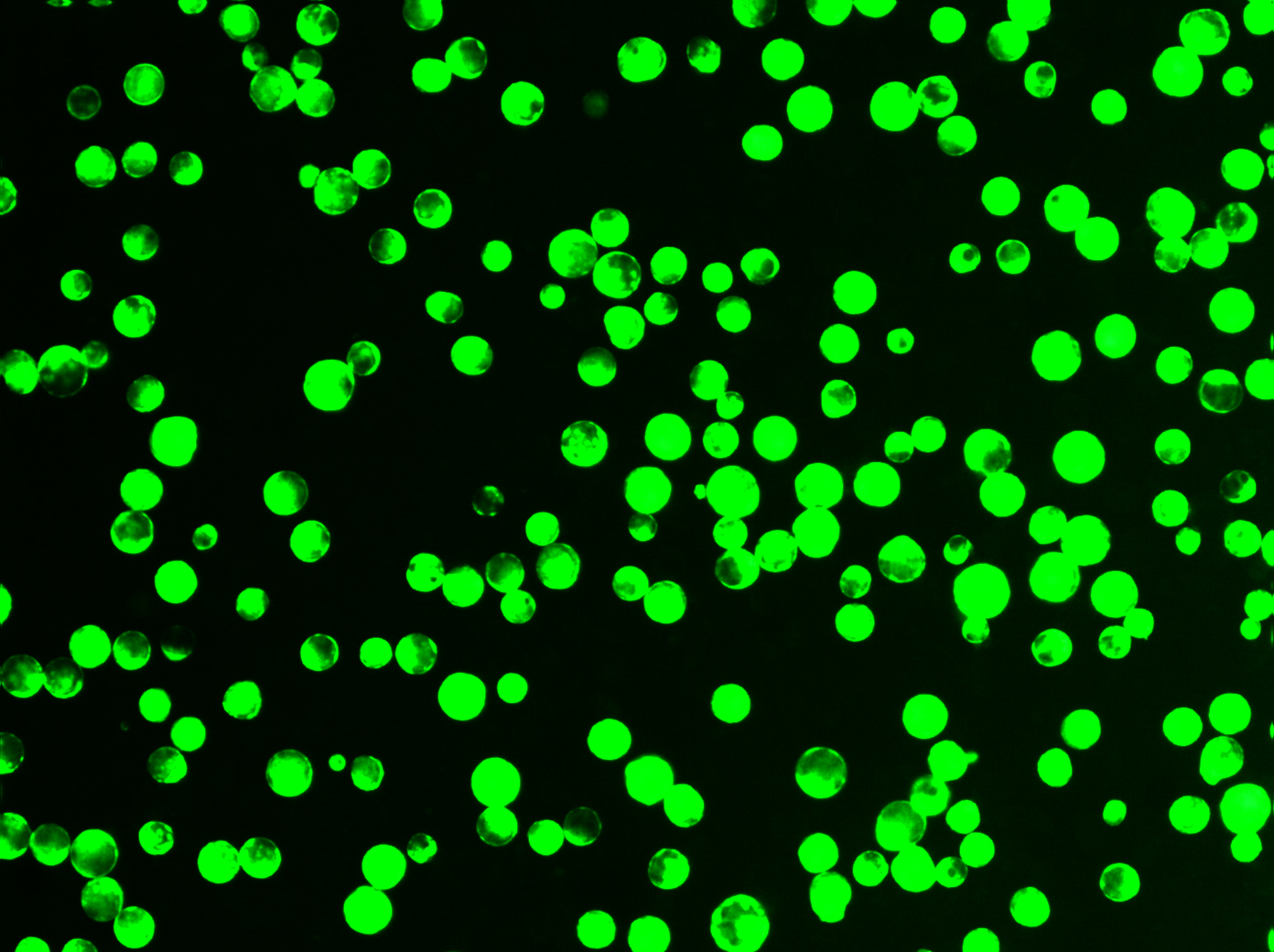

Supplement: Additional file 1 — The zip archive contains real images showing protoplasts. (ZIP 4 kb) [file 12859_2017_1591_MOESM1_ESM.zip › protoplasts/20111129-03B (2).png]

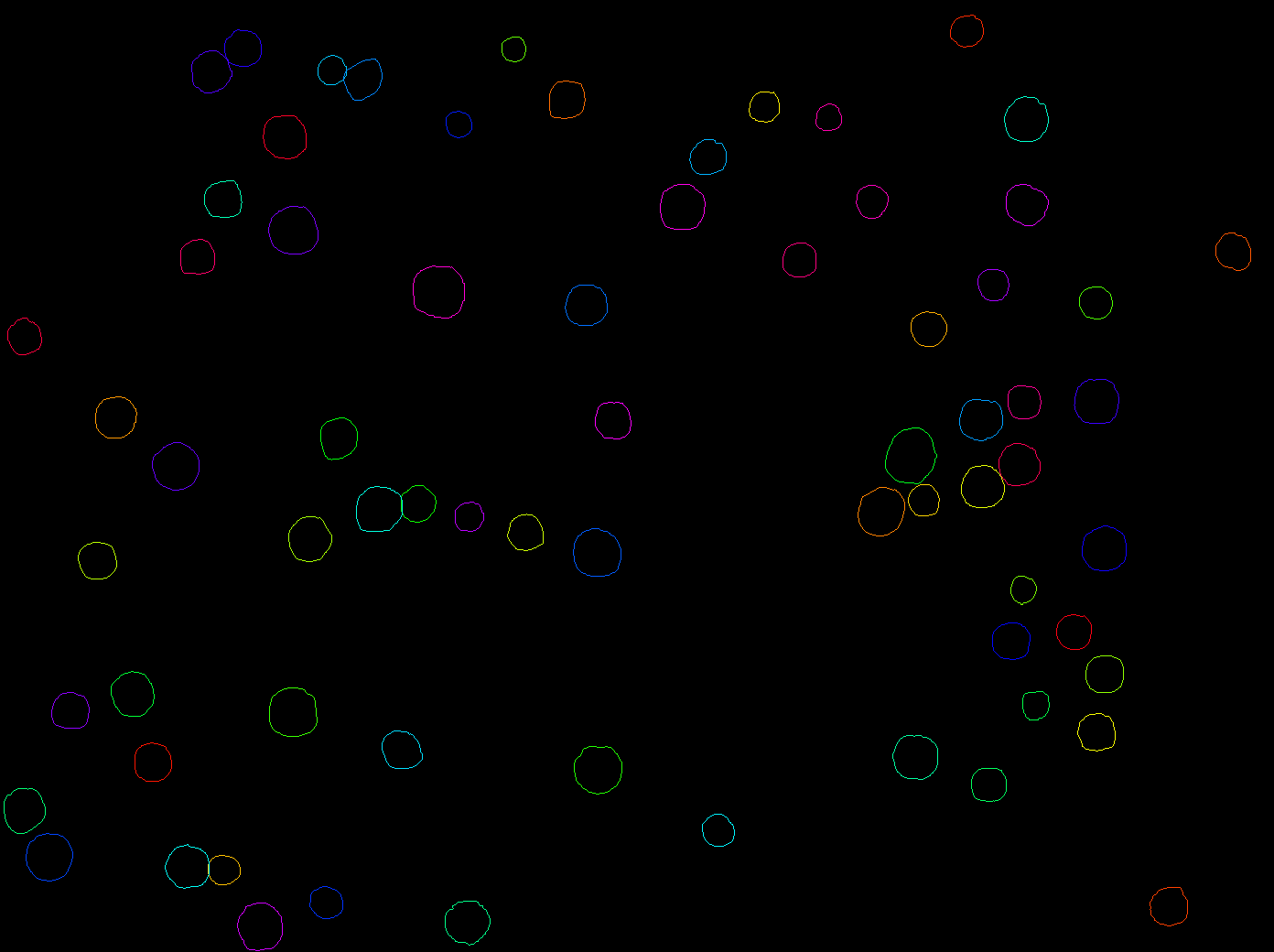

Supplement: Additional file 1 — The zip archive contains real images showing protoplasts. (ZIP 4 kb) [file 12859_2017_1591_MOESM1_ESM.zip › protoplasts/20111129-07A (2) gt.png]

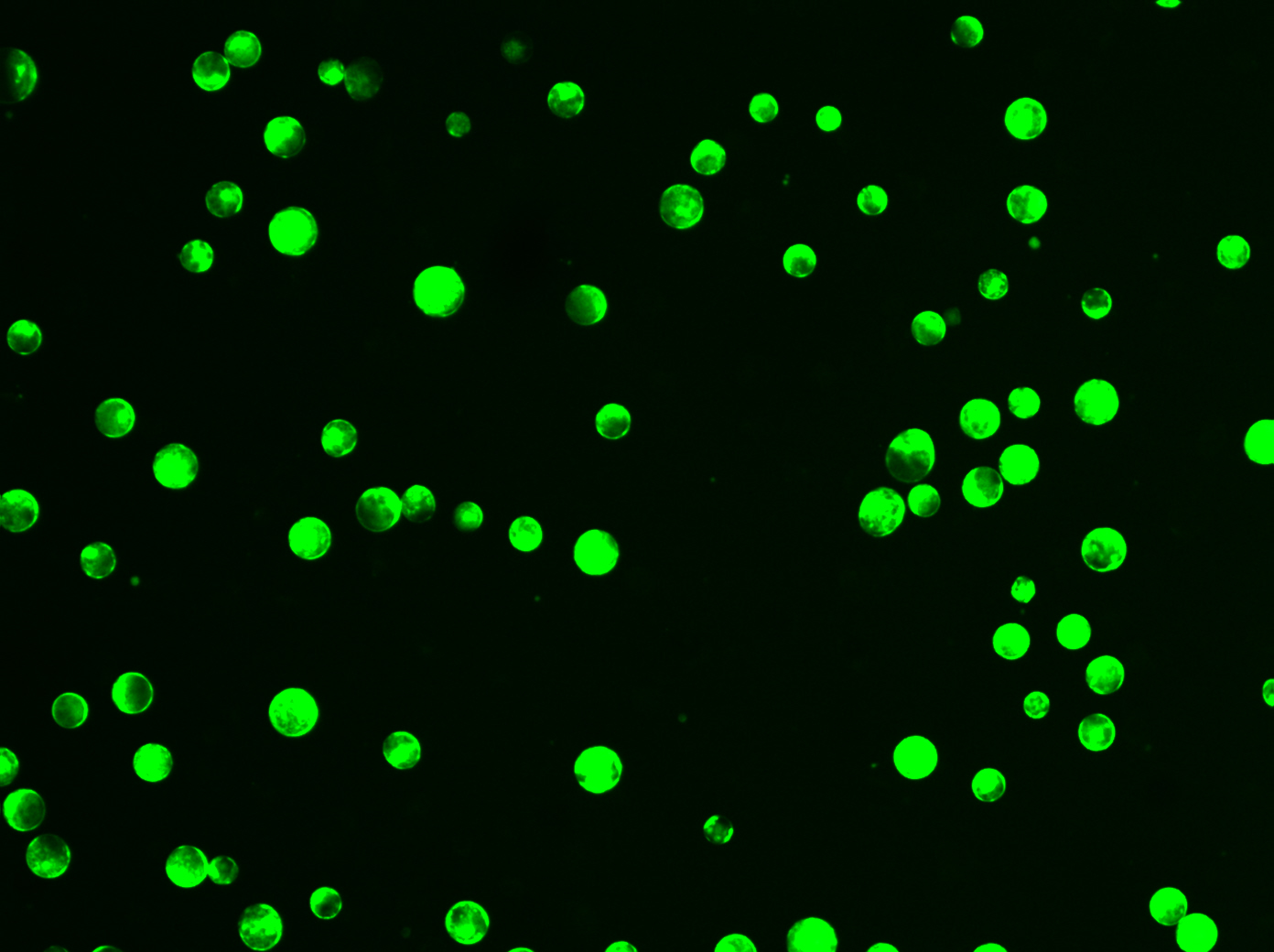

Supplement: Additional file 1 — The zip archive contains real images showing protoplasts. (ZIP 4 kb) [file 12859_2017_1591_MOESM1_ESM.zip › protoplasts/20111129-07A (2).png]

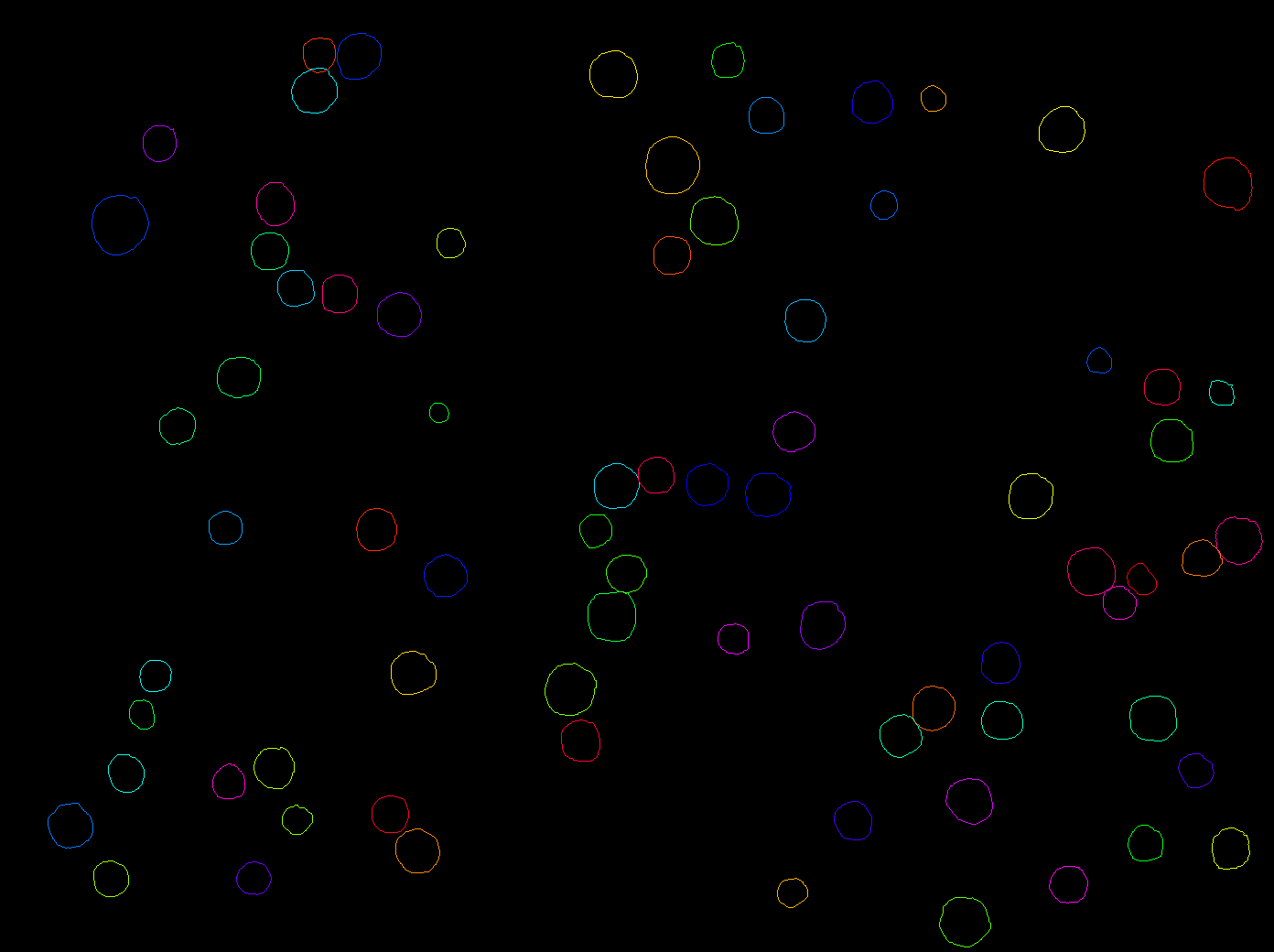

Supplement: Additional file 1 — The zip archive contains real images showing protoplasts. (ZIP 4 kb) [file 12859_2017_1591_MOESM1_ESM.zip › protoplasts/20111129-07B (2) gt.png]

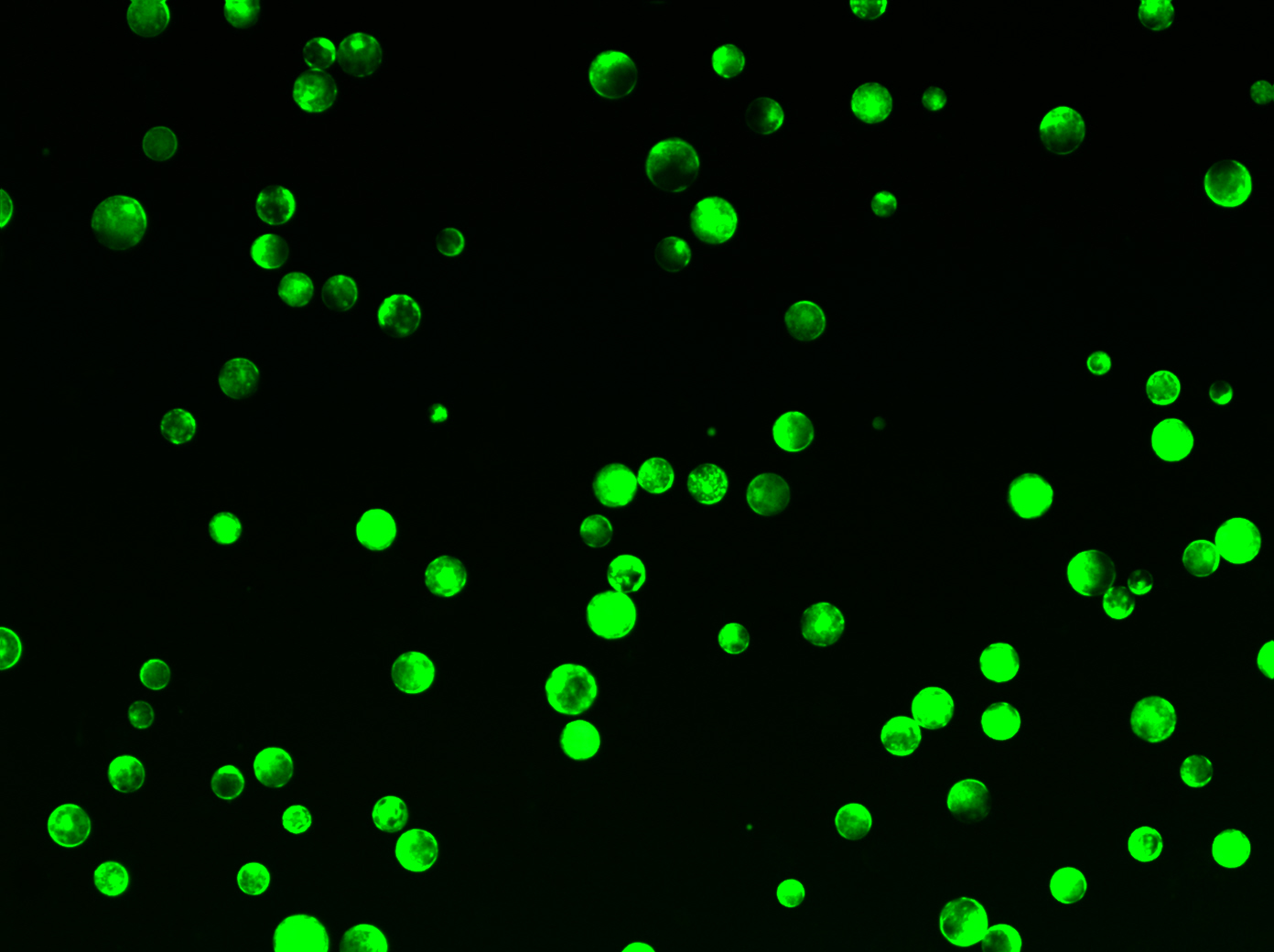

Supplement: Additional file 1 — The zip archive contains real images showing protoplasts. (ZIP 4 kb) [file 12859_2017_1591_MOESM1_ESM.zip › protoplasts/20111129-07B (2).png]

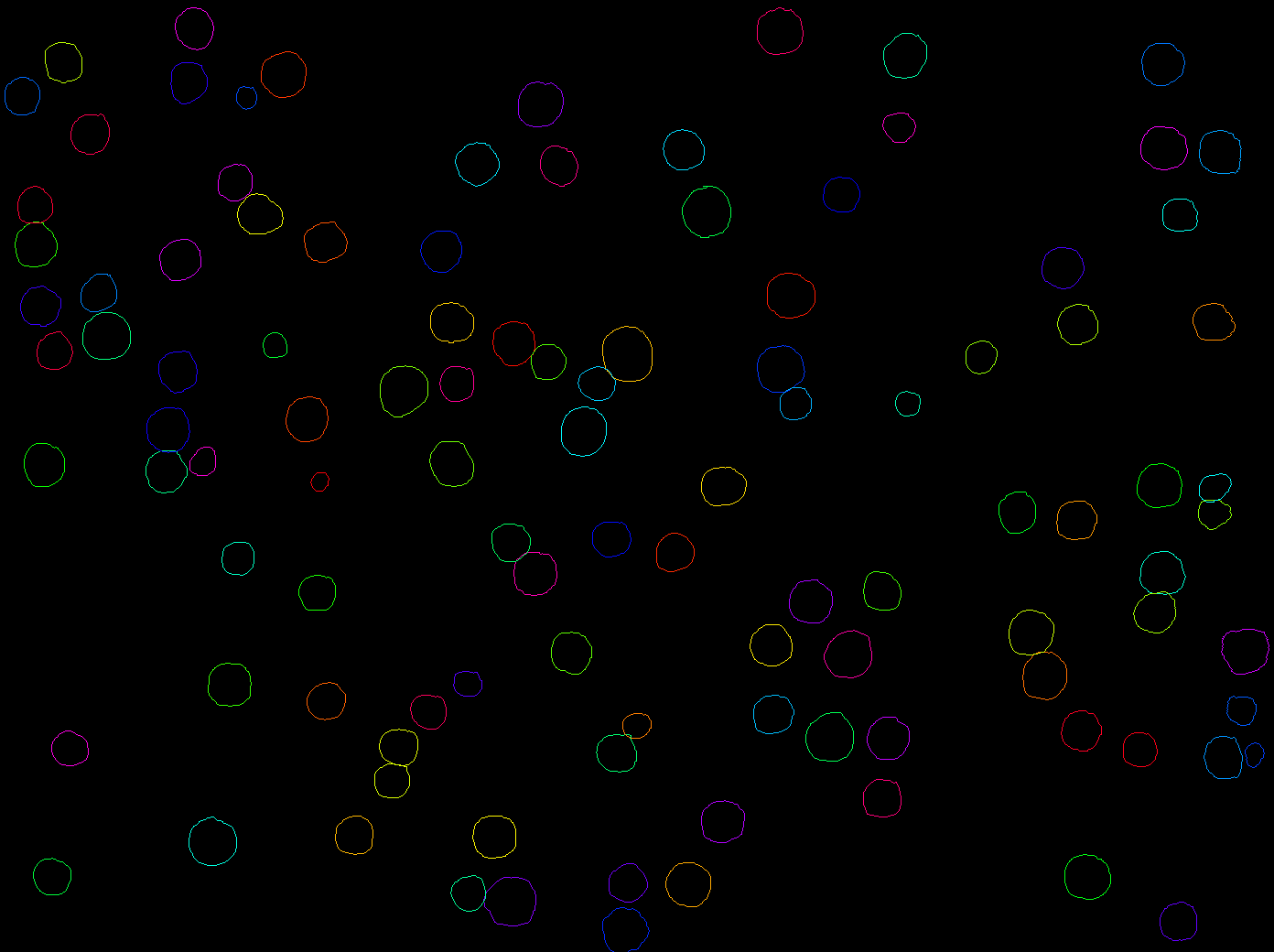

Supplement: Additional file 1 — The zip archive contains real images showing protoplasts. (ZIP 4 kb) [file 12859_2017_1591_MOESM1_ESM.zip › protoplasts/20111129-08A (2) gt.png]

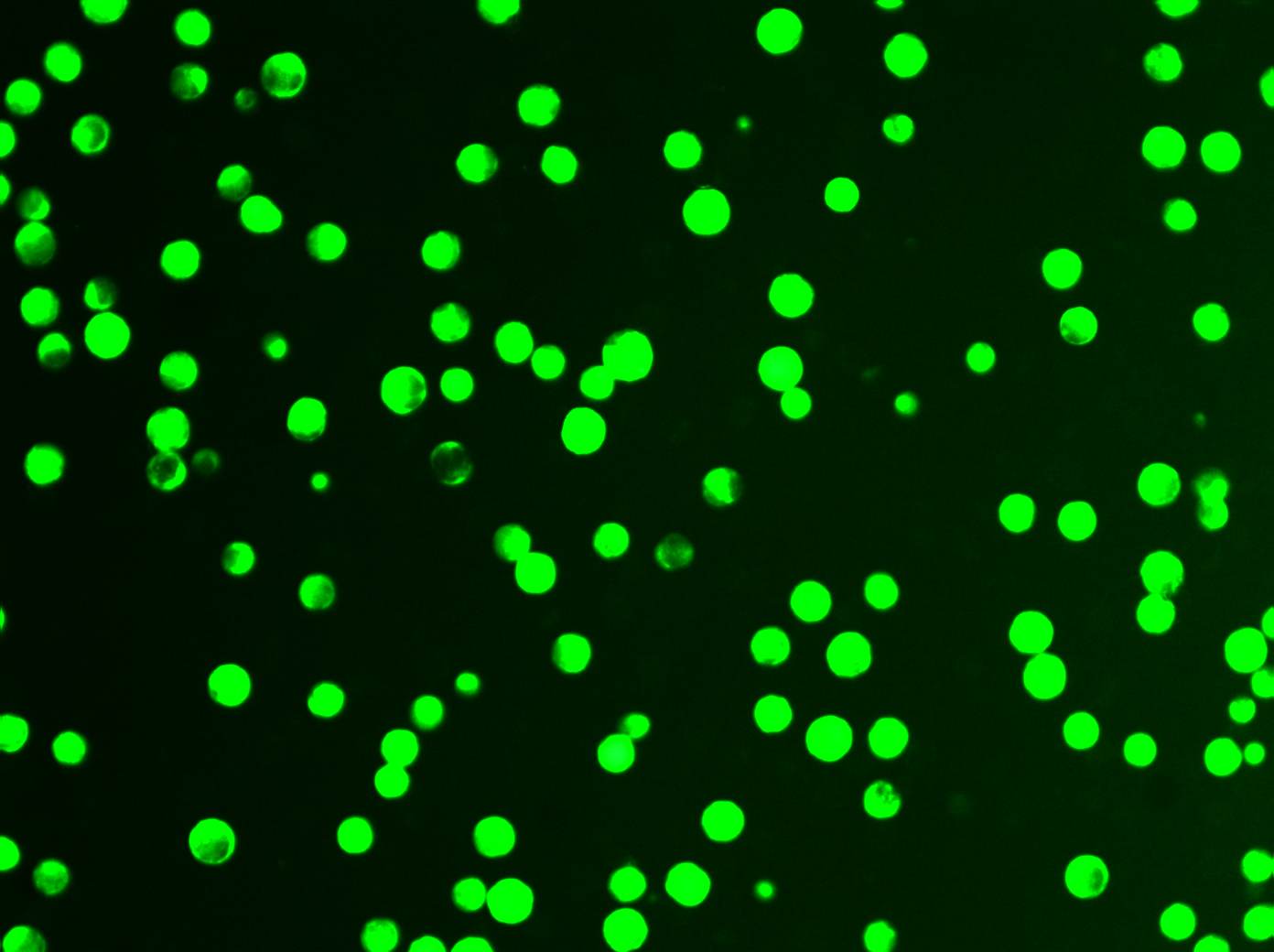

Supplement: Additional file 1 — The zip archive contains real images showing protoplasts. (ZIP 4 kb) [file 12859_2017_1591_MOESM1_ESM.zip › protoplasts/20111129-08A (2).png]

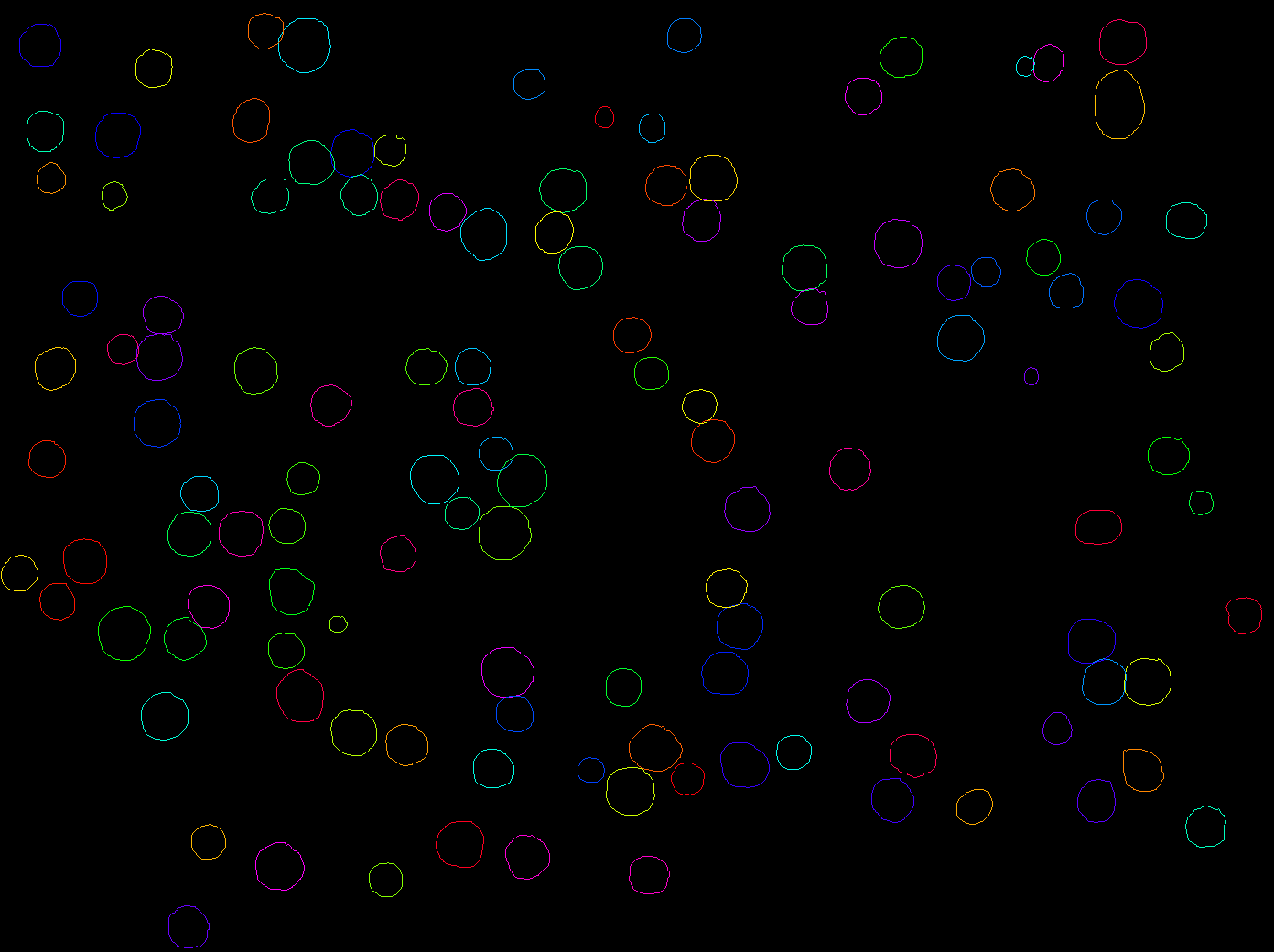

Supplement: Additional file 1 — The zip archive contains real images showing protoplasts. (ZIP 4 kb) [file 12859_2017_1591_MOESM1_ESM.zip › protoplasts/20111129-08B (2) gt.png]

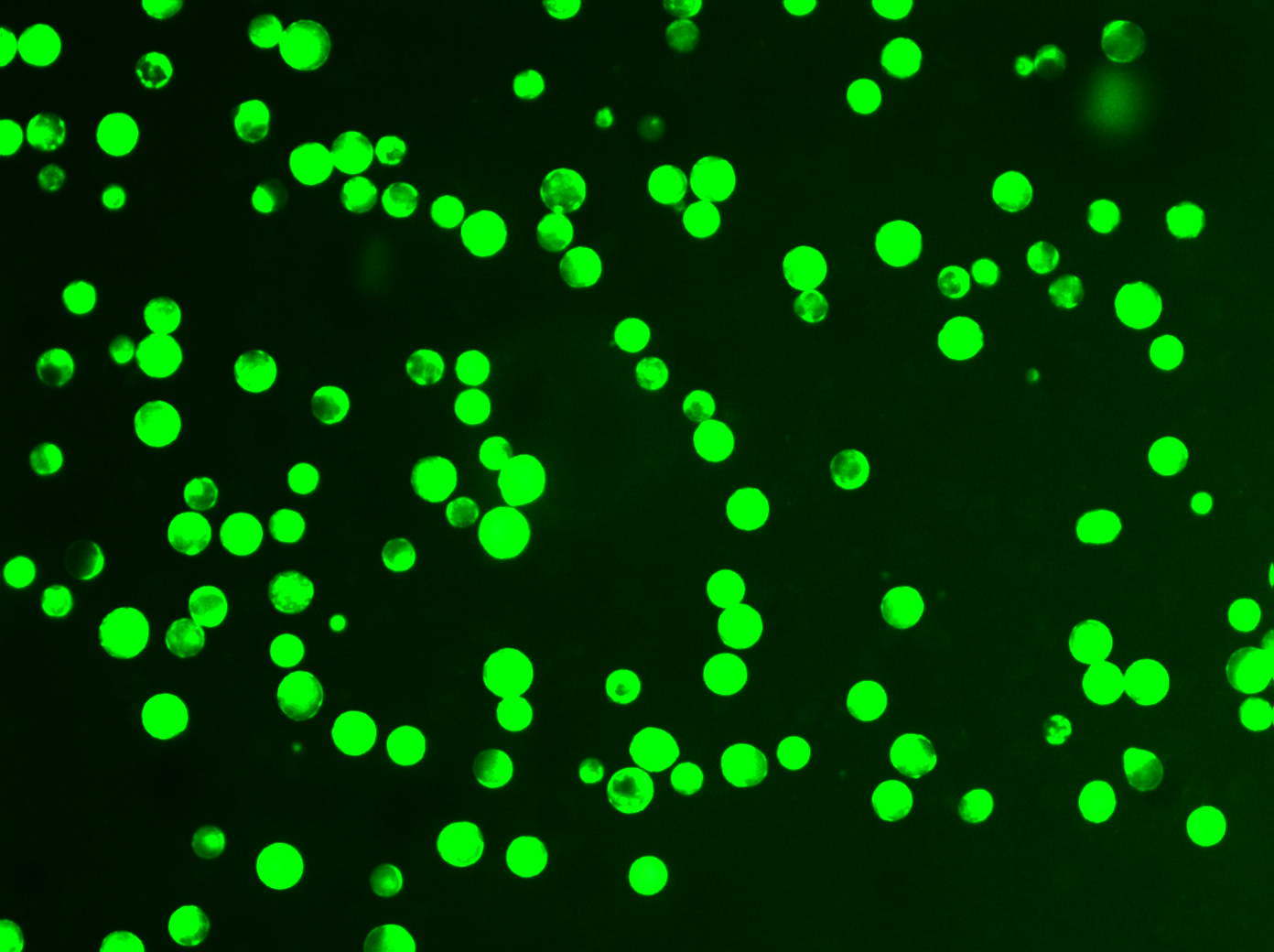

Supplement: Additional file 1 — The zip archive contains real images showing protoplasts. (ZIP 4 kb) [file 12859_2017_1591_MOESM1_ESM.zip › protoplasts/20111129-08B (2).png]

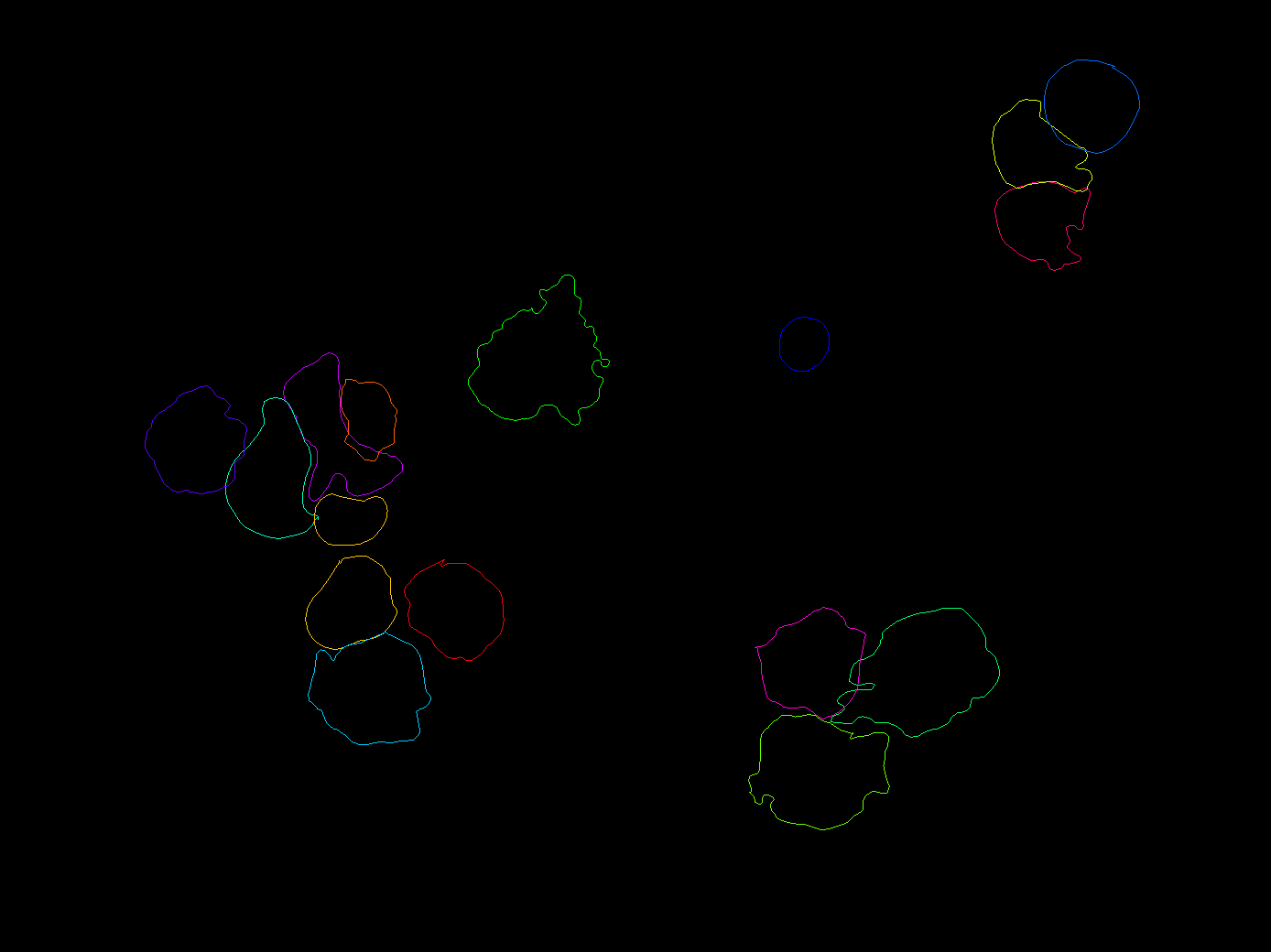

Supplement: Additional file 2 — The zip archive contains real images showing B cell nuclei and cytoskeleton. (ZIP 12390 kb) [file 12859_2017_1591_MOESM2_ESM.zip › B cells/cells0001 gt.png]

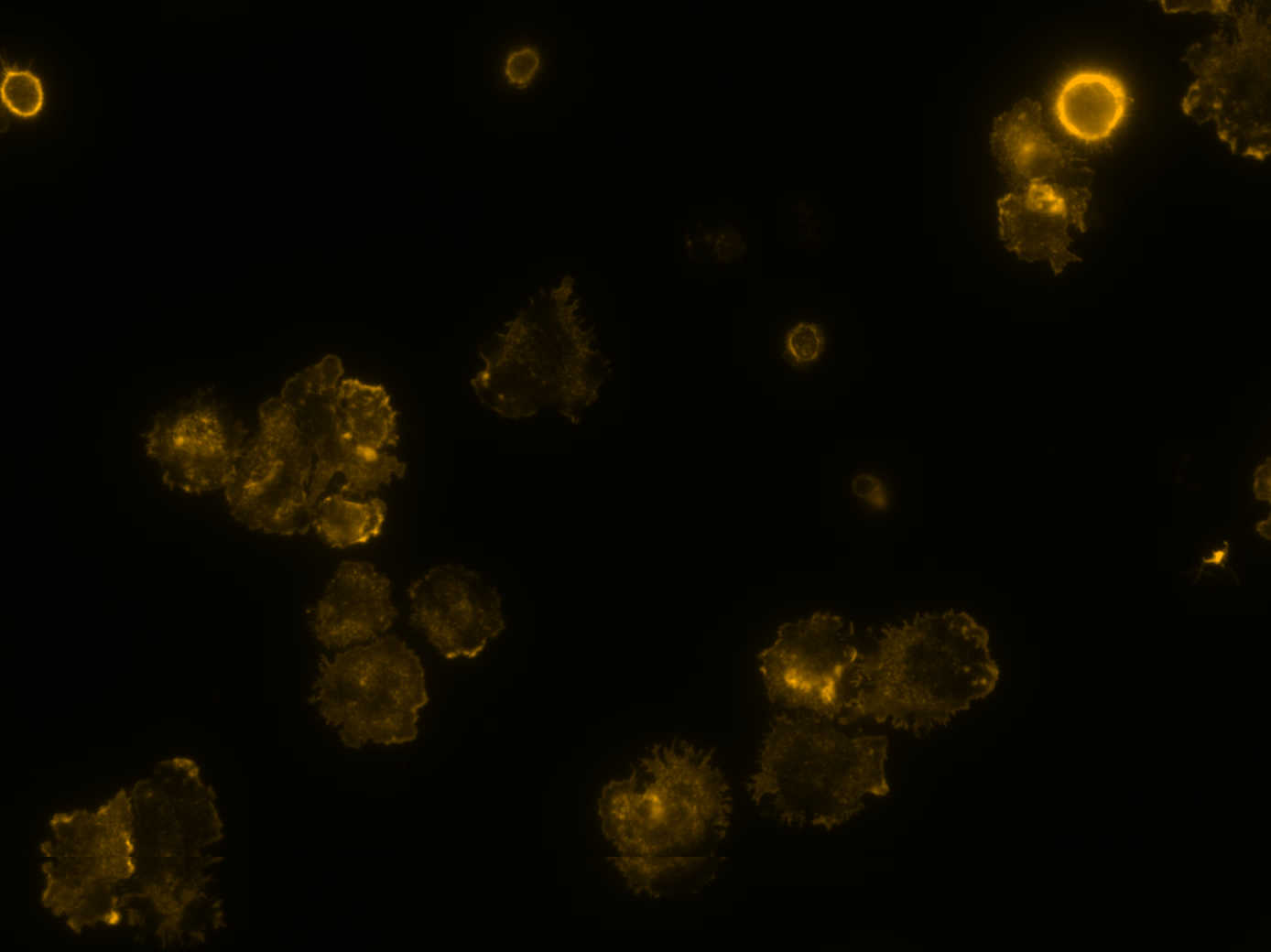

Supplement: Additional file 2 — The zip archive contains real images showing B cell nuclei and cytoskeleton. (ZIP 12390 kb) [file 12859_2017_1591_MOESM2_ESM.zip › B cells/cells0001.png]

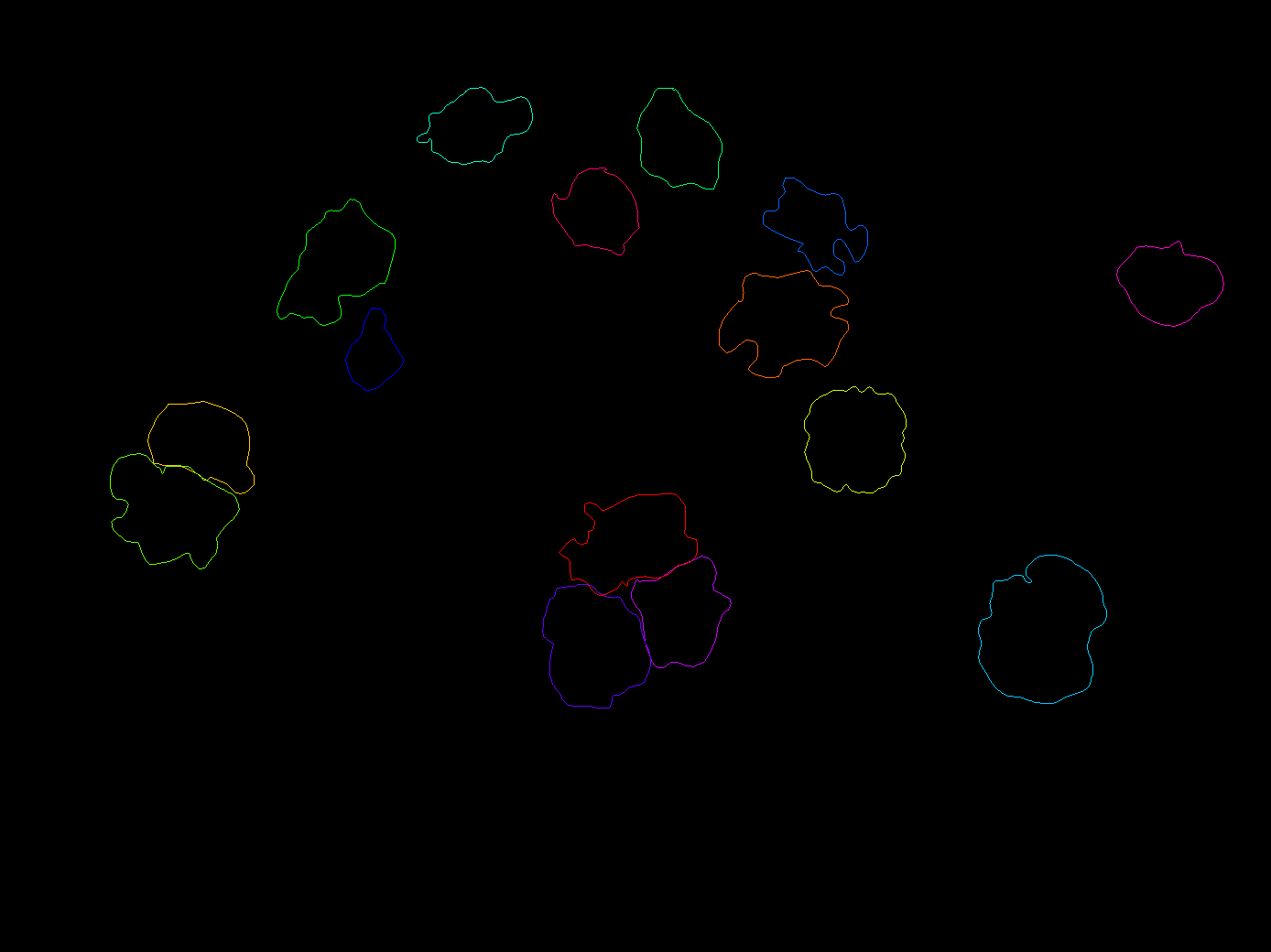

Supplement: Additional file 2 — The zip archive contains real images showing B cell nuclei and cytoskeleton. (ZIP 12390 kb) [file 12859_2017_1591_MOESM2_ESM.zip › B cells/cells0002 gt.png]

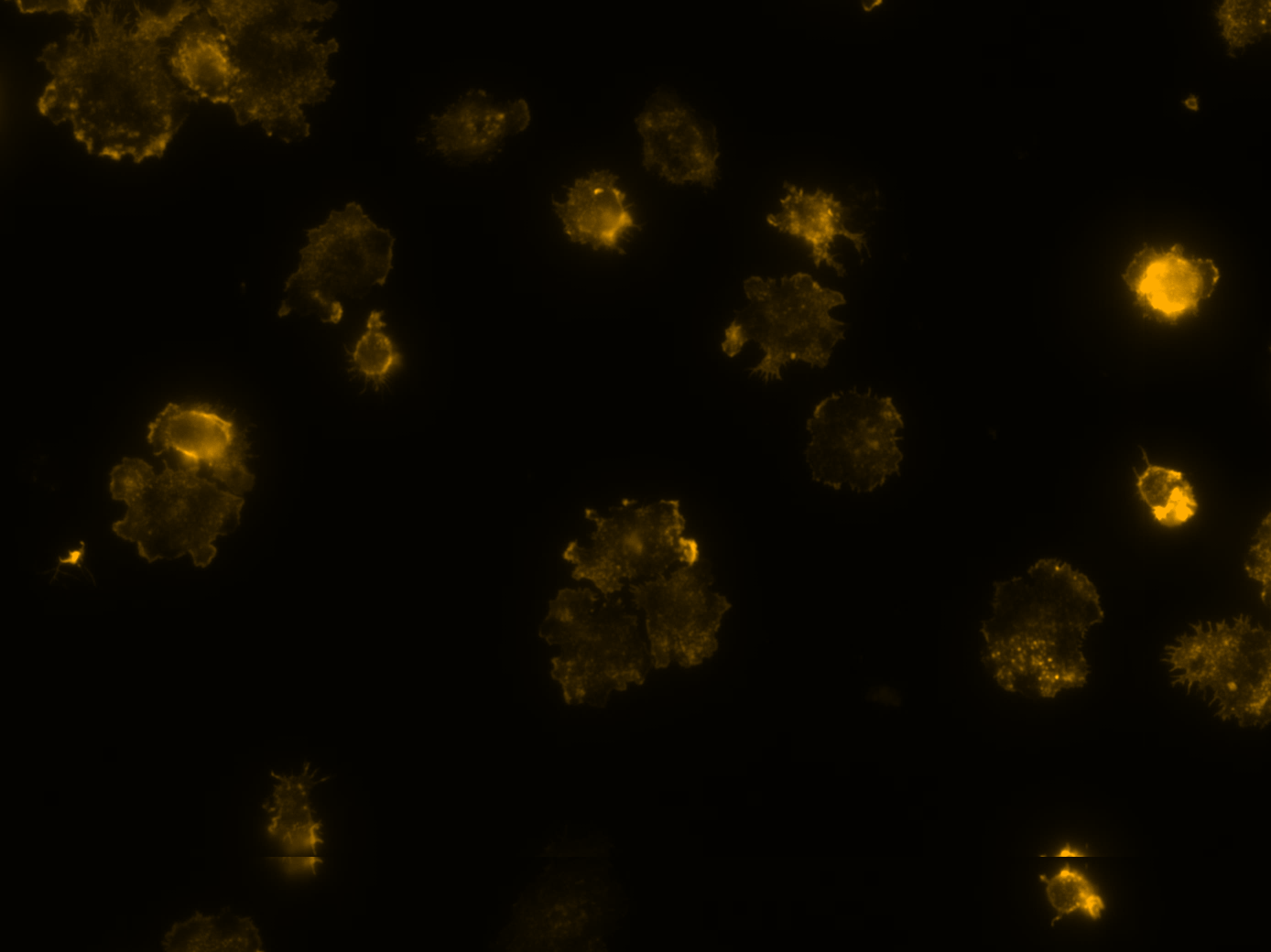

Supplement: Additional file 2 — The zip archive contains real images showing B cell nuclei and cytoskeleton. (ZIP 12390 kb) [file 12859_2017_1591_MOESM2_ESM.zip › B cells/cells0002.png]

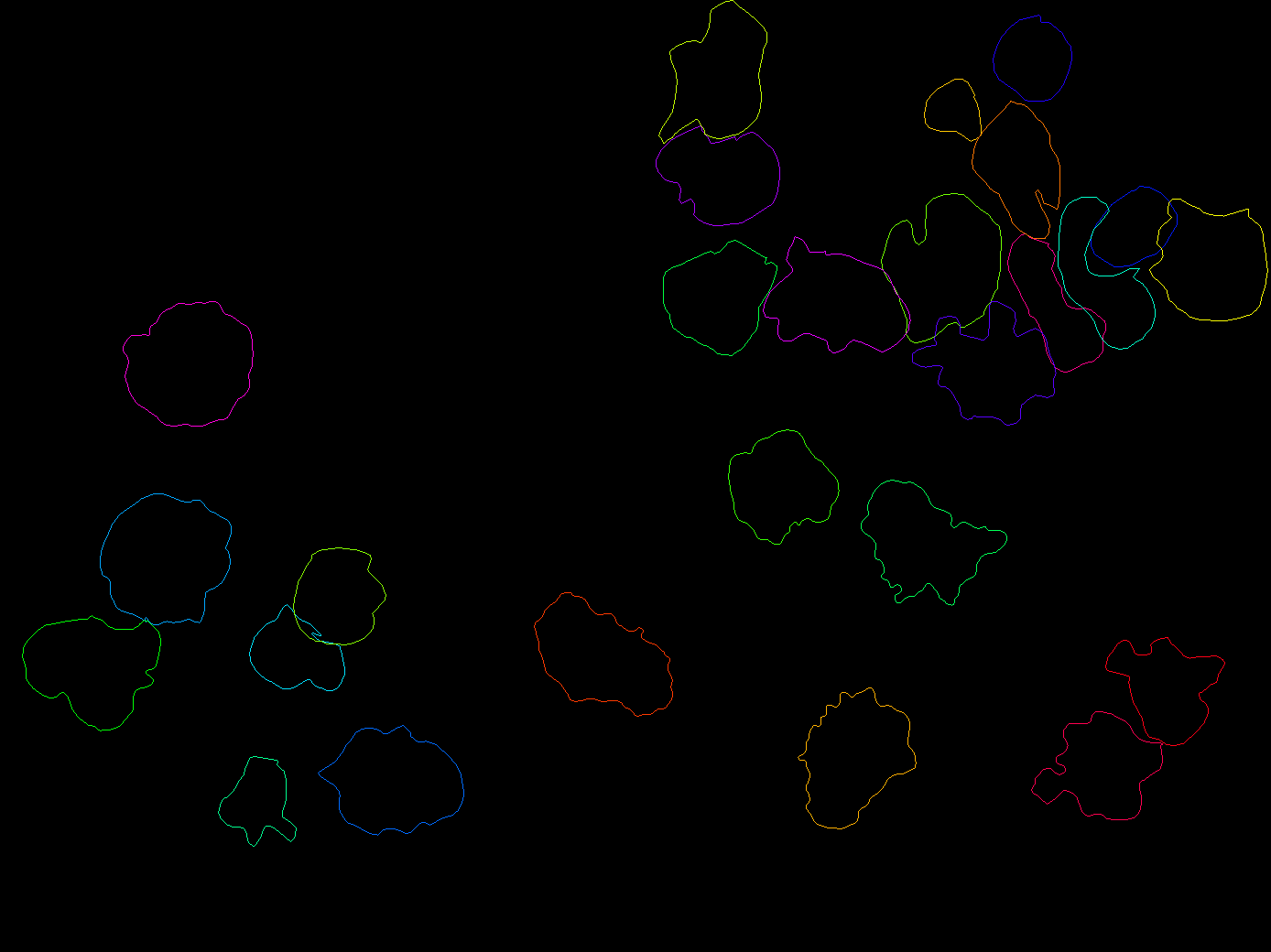

Supplement: Additional file 2 — The zip archive contains real images showing B cell nuclei and cytoskeleton. (ZIP 12390 kb) [file 12859_2017_1591_MOESM2_ESM.zip › B cells/cells0003 gt.png]

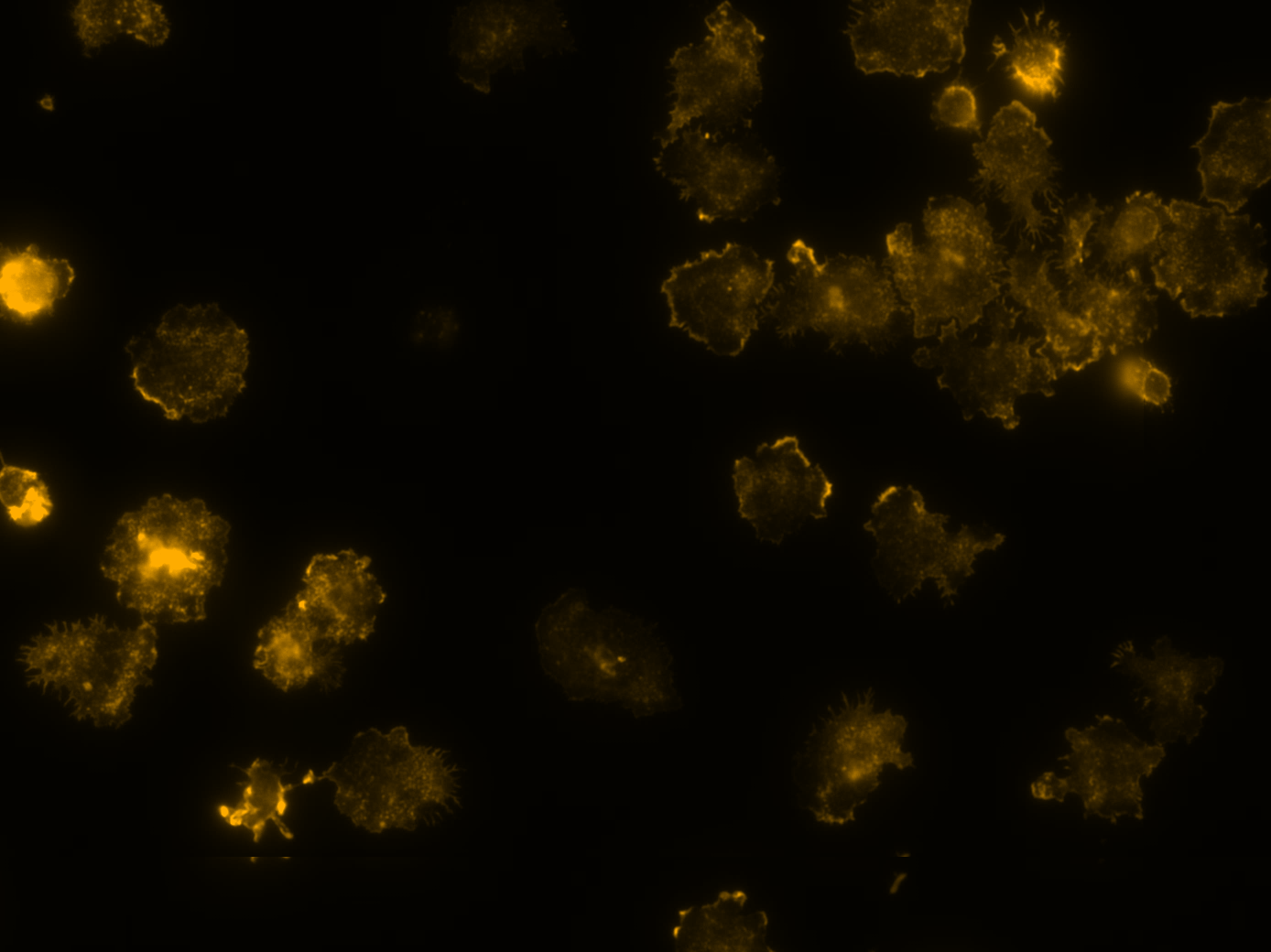

Supplement: Additional file 2 — The zip archive contains real images showing B cell nuclei and cytoskeleton. (ZIP 12390 kb) [file 12859_2017_1591_MOESM2_ESM.zip › B cells/cells0003.png]

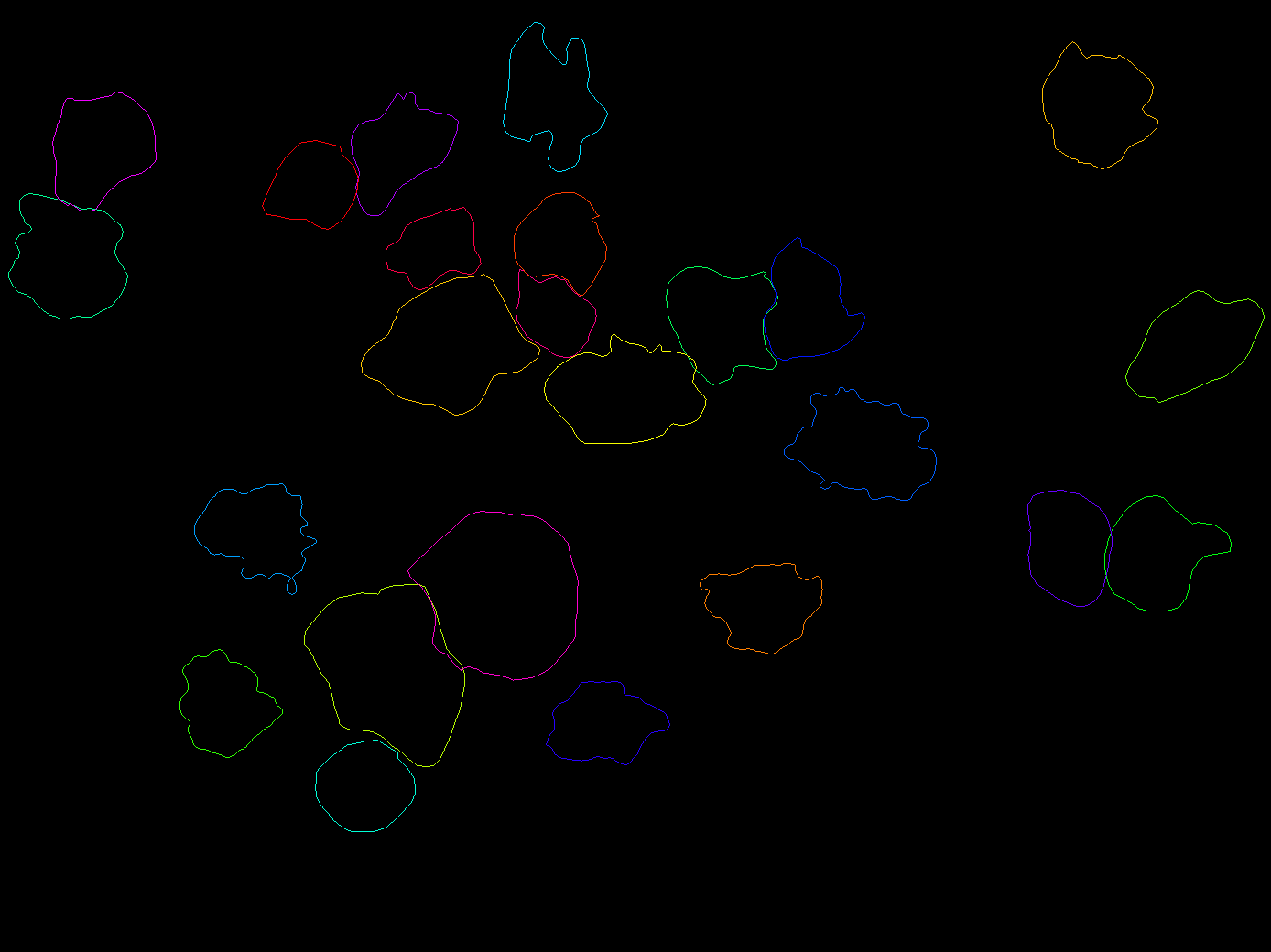

Supplement: Additional file 2 — The zip archive contains real images showing B cell nuclei and cytoskeleton. (ZIP 12390 kb) [file 12859_2017_1591_MOESM2_ESM.zip › B cells/cells0004 gt.png]

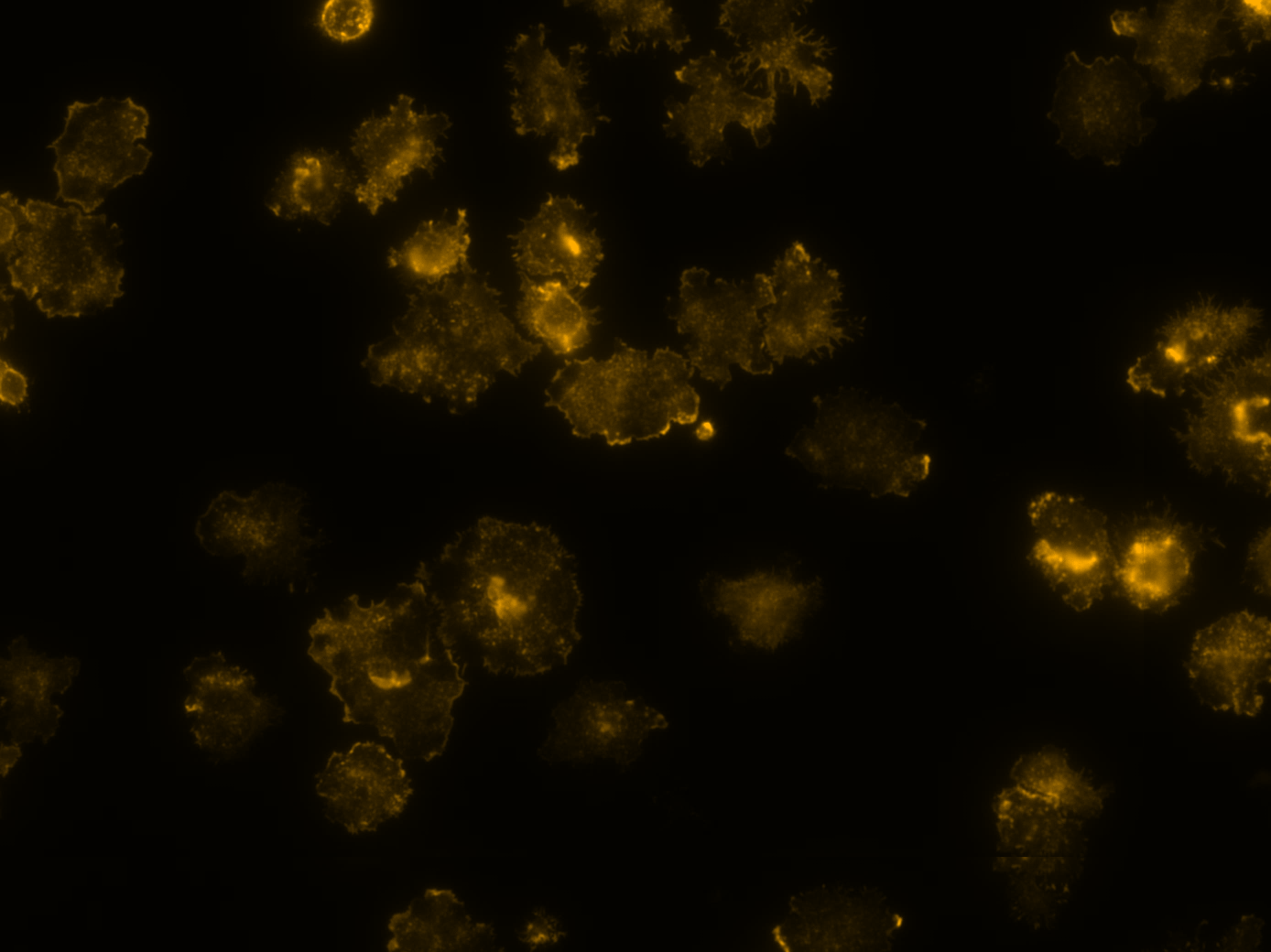

Supplement: Additional file 2 — The zip archive contains real images showing B cell nuclei and cytoskeleton. (ZIP 12390 kb) [file 12859_2017_1591_MOESM2_ESM.zip › B cells/cells0004.png]

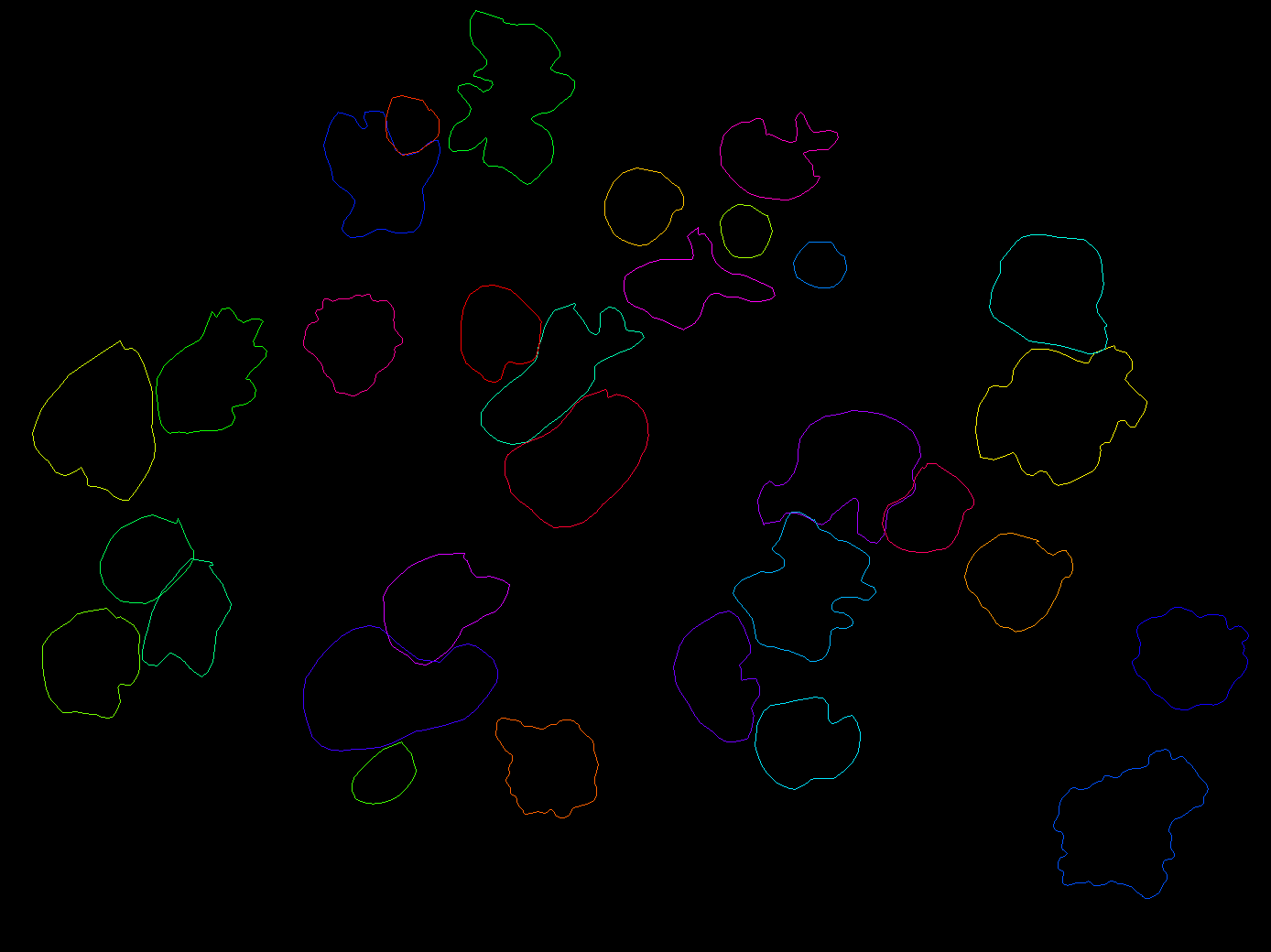

Supplement: Additional file 2 — The zip archive contains real images showing B cell nuclei and cytoskeleton. (ZIP 12390 kb) [file 12859_2017_1591_MOESM2_ESM.zip › B cells/cells0005 gt.png]

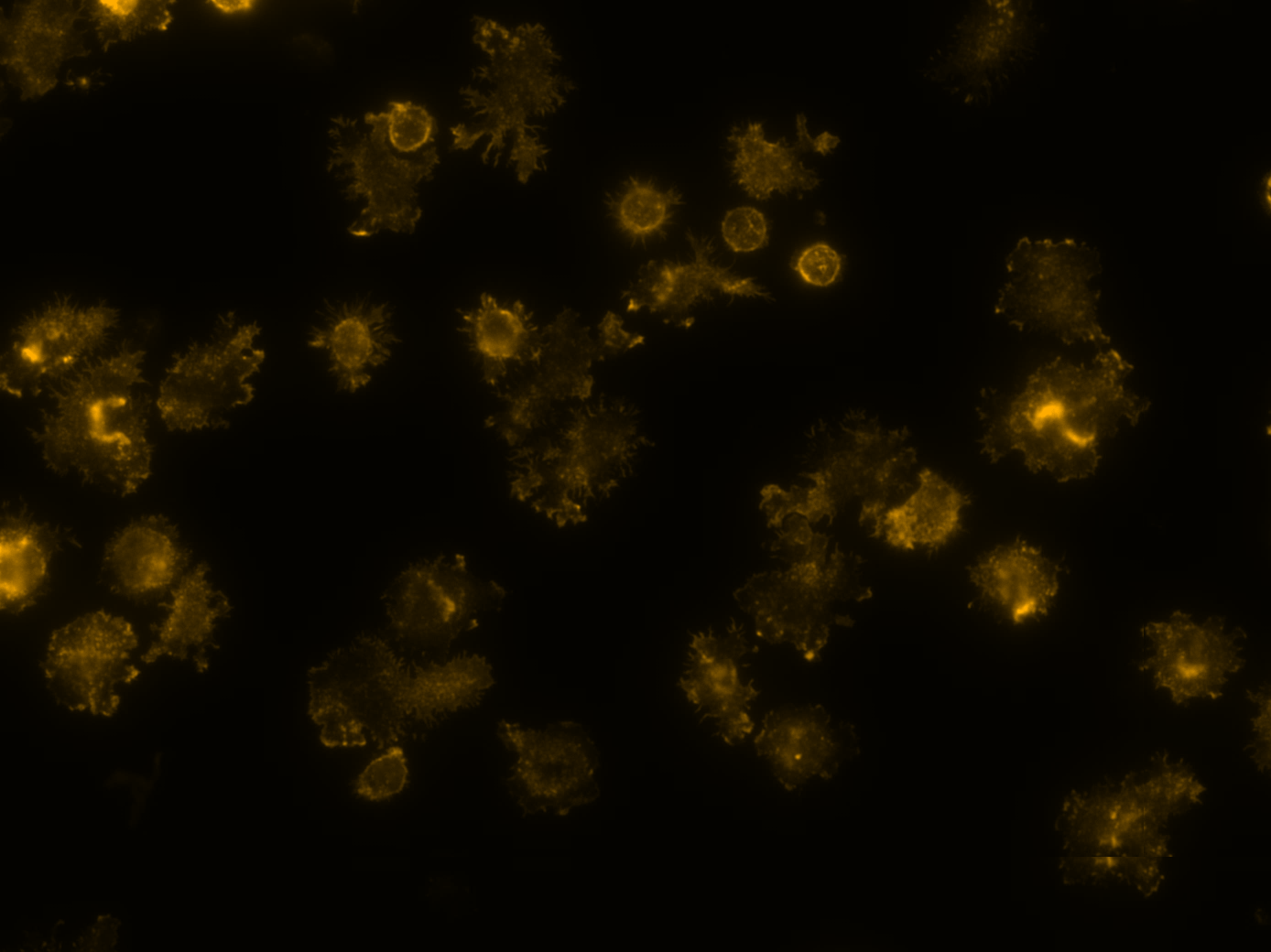

Supplement: Additional file 2 — The zip archive contains real images showing B cell nuclei and cytoskeleton. (ZIP 12390 kb) [file 12859_2017_1591_MOESM2_ESM.zip › B cells/cells0005.png]

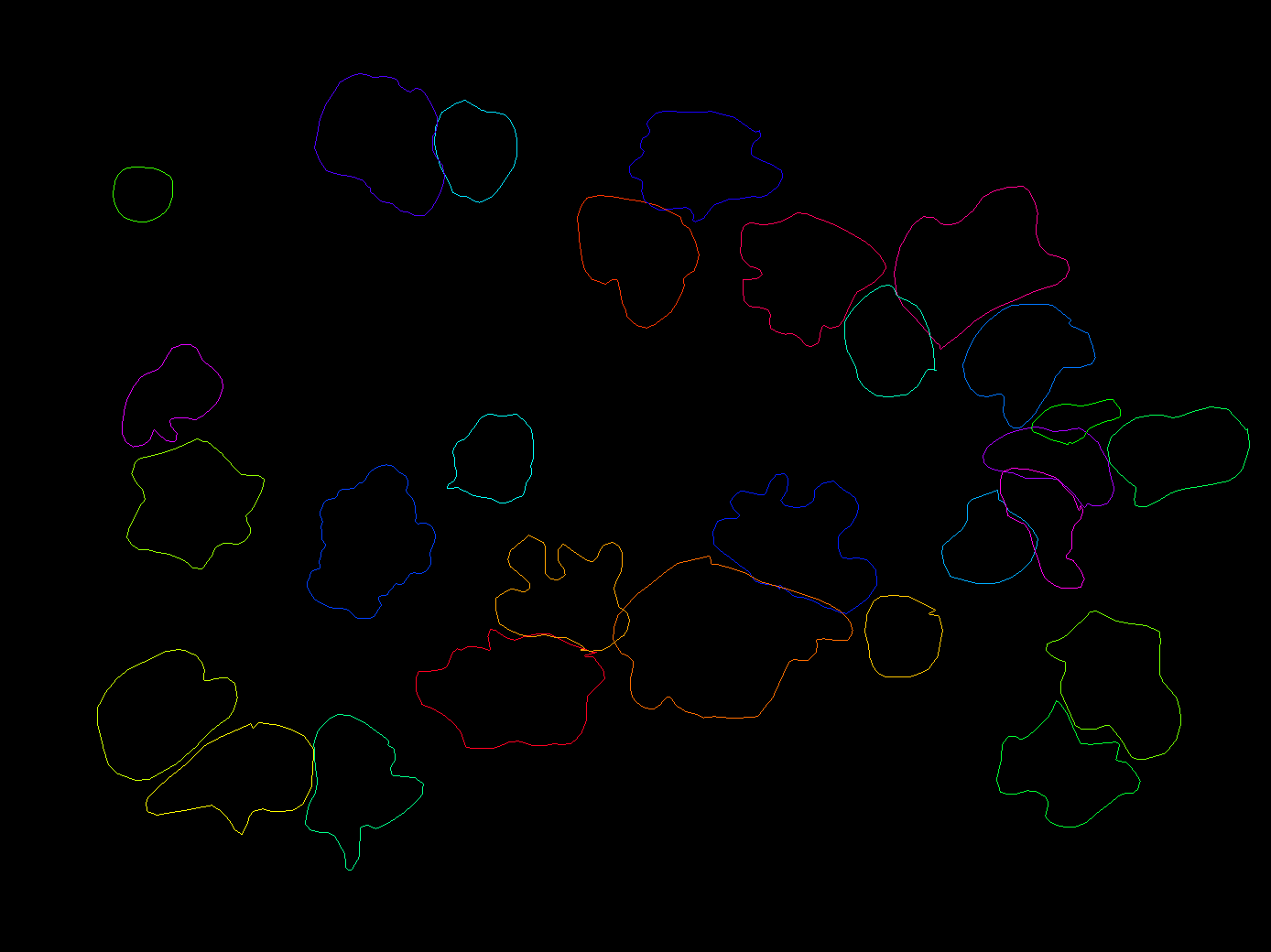

Supplement: Additional file 2 — The zip archive contains real images showing B cell nuclei and cytoskeleton. (ZIP 12390 kb) [file 12859_2017_1591_MOESM2_ESM.zip › B cells/cells0006 gt.png]

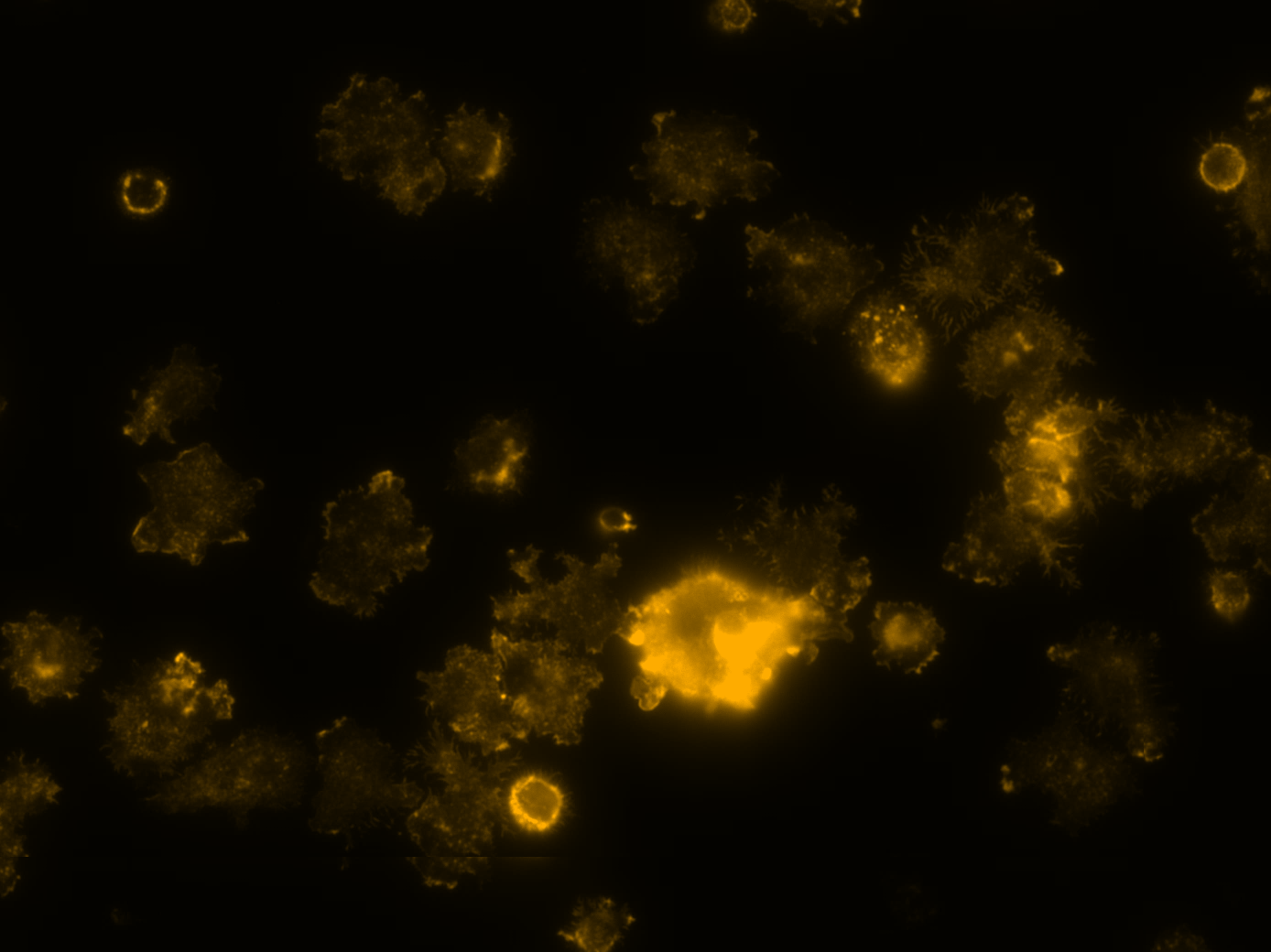

Supplement: Additional file 2 — The zip archive contains real images showing B cell nuclei and cytoskeleton. (ZIP 12390 kb) [file 12859_2017_1591_MOESM2_ESM.zip › B cells/cells0006.png]

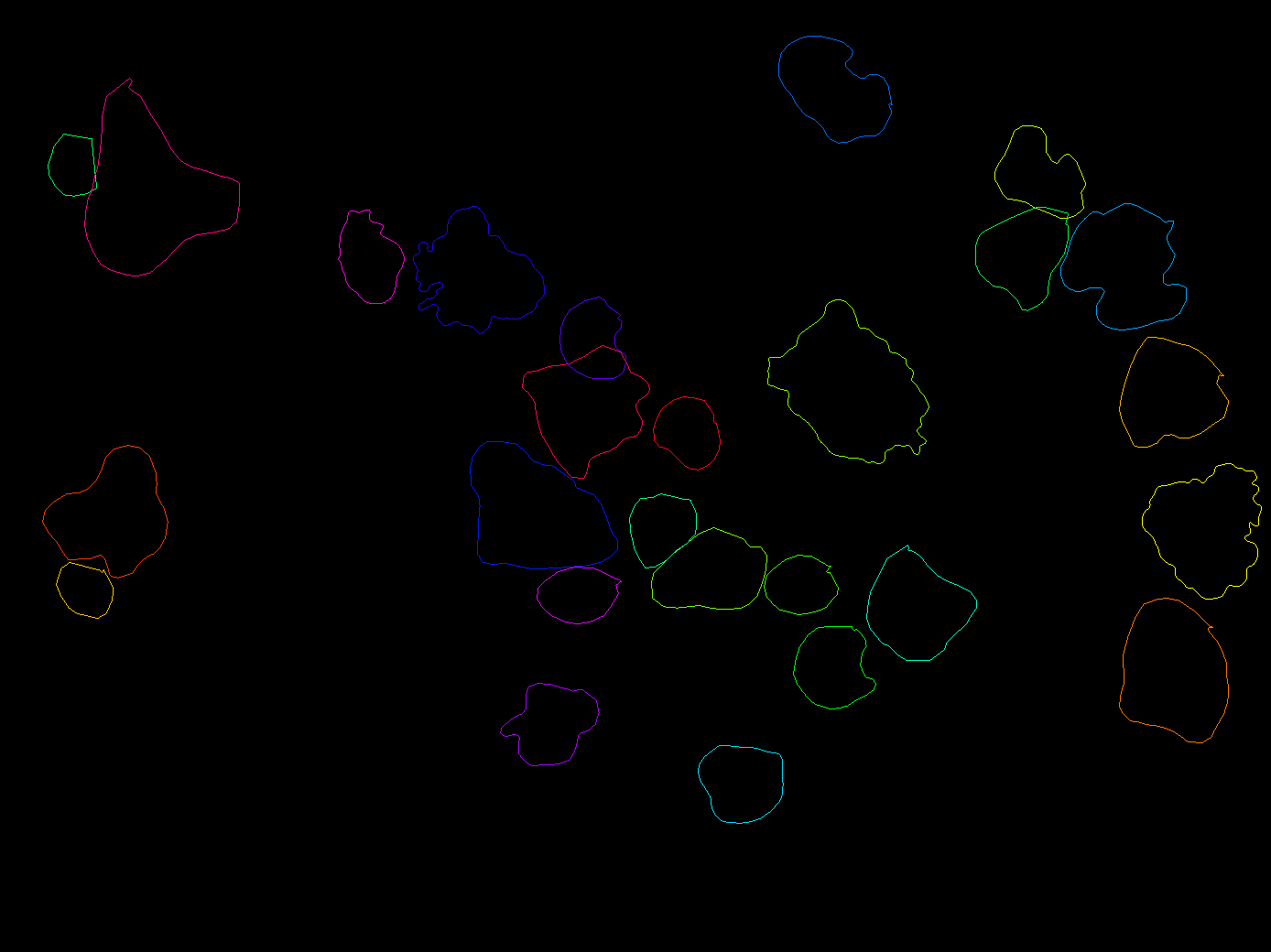

Supplement: Additional file 2 — The zip archive contains real images showing B cell nuclei and cytoskeleton. (ZIP 12390 kb) [file 12859_2017_1591_MOESM2_ESM.zip › B cells/cells0007 gt.png]

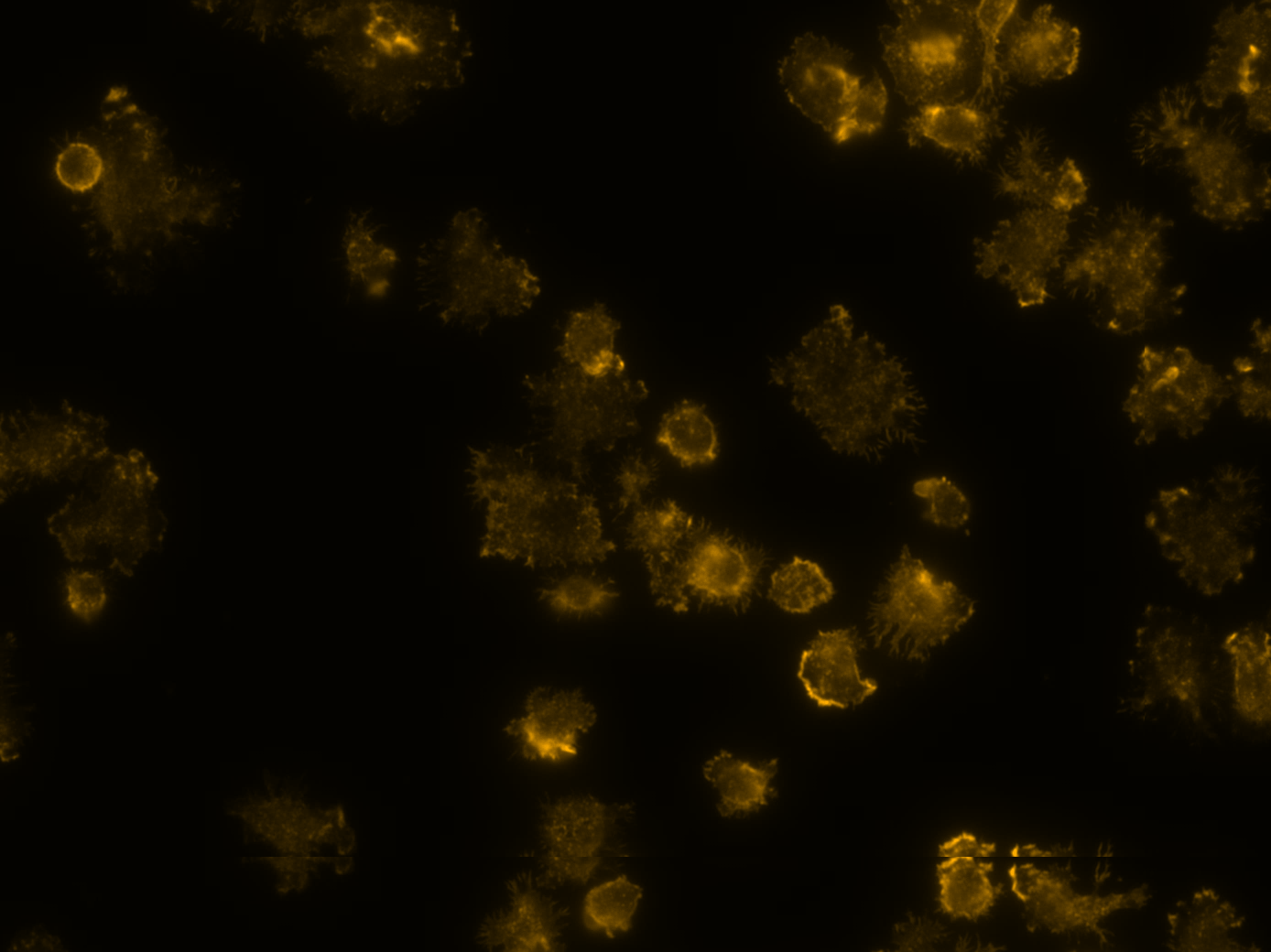

Supplement: Additional file 2 — The zip archive contains real images showing B cell nuclei and cytoskeleton. (ZIP 12390 kb) [file 12859_2017_1591_MOESM2_ESM.zip › B cells/cells0007.png]

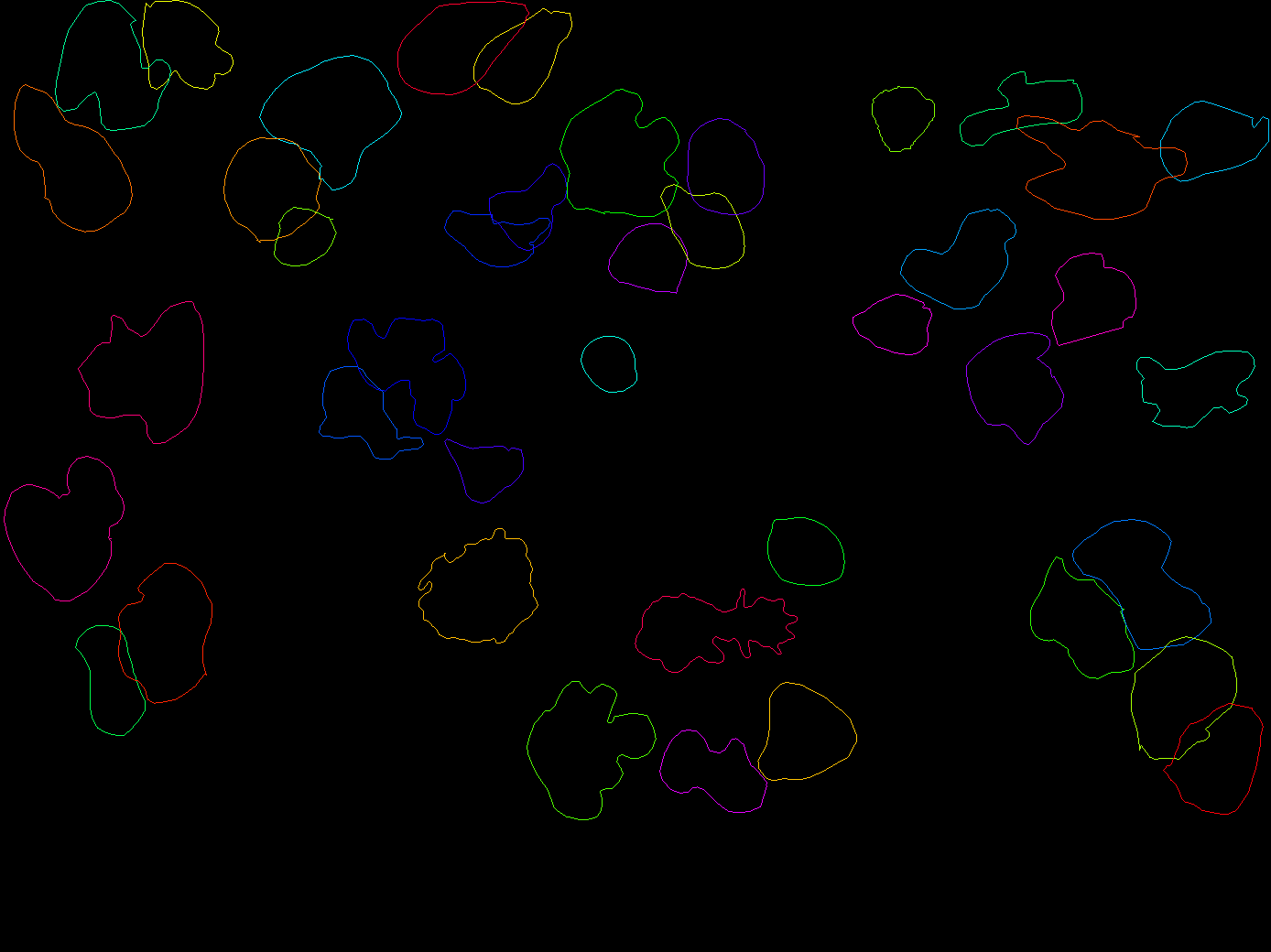

Supplement: Additional file 2 — The zip archive contains real images showing B cell nuclei and cytoskeleton. (ZIP 12390 kb) [file 12859_2017_1591_MOESM2_ESM.zip › B cells/cells0008 gt.png]

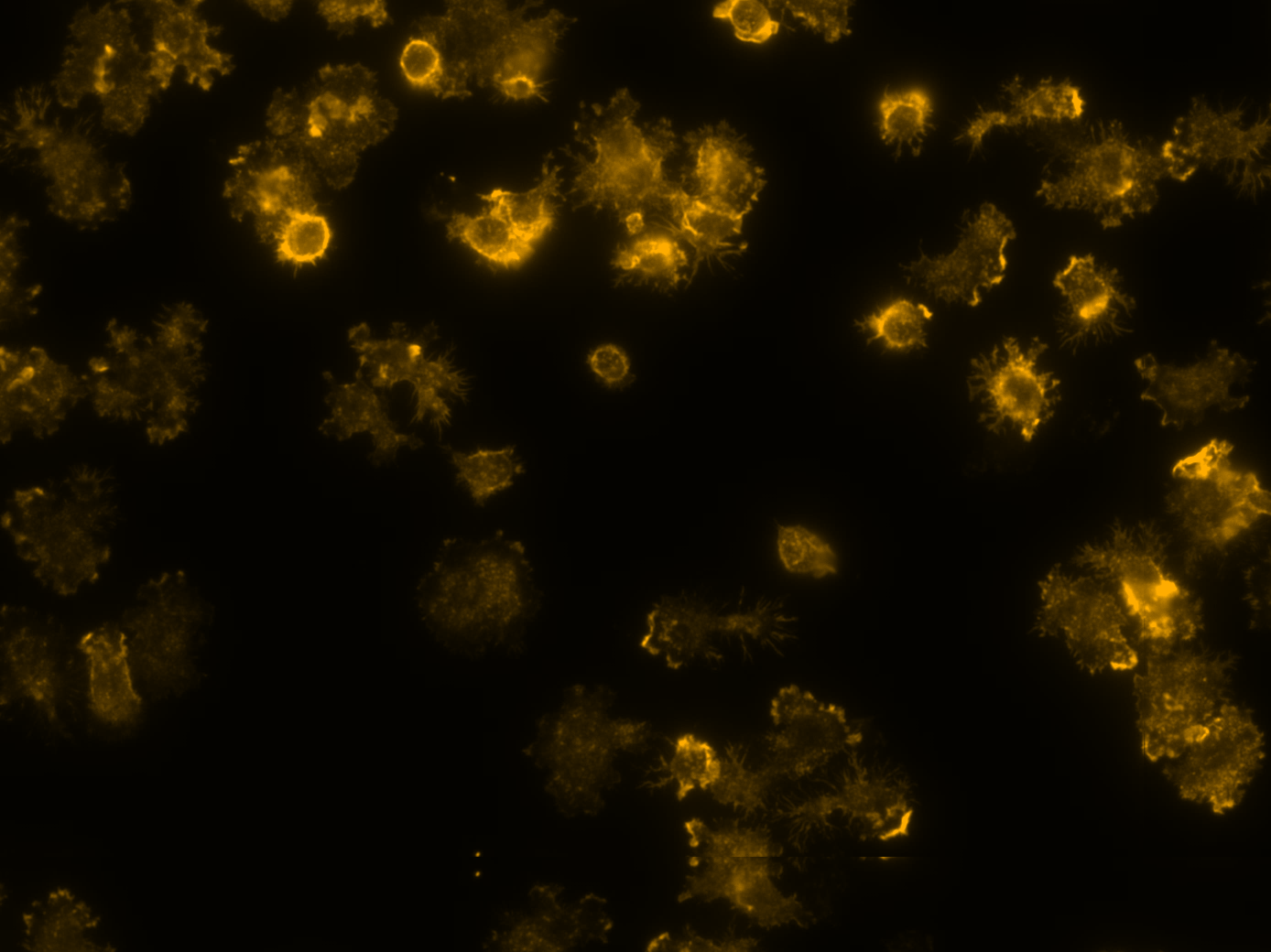

Supplement: Additional file 2 — The zip archive contains real images showing B cell nuclei and cytoskeleton. (ZIP 12390 kb) [file 12859_2017_1591_MOESM2_ESM.zip › B cells/cells0008.png]

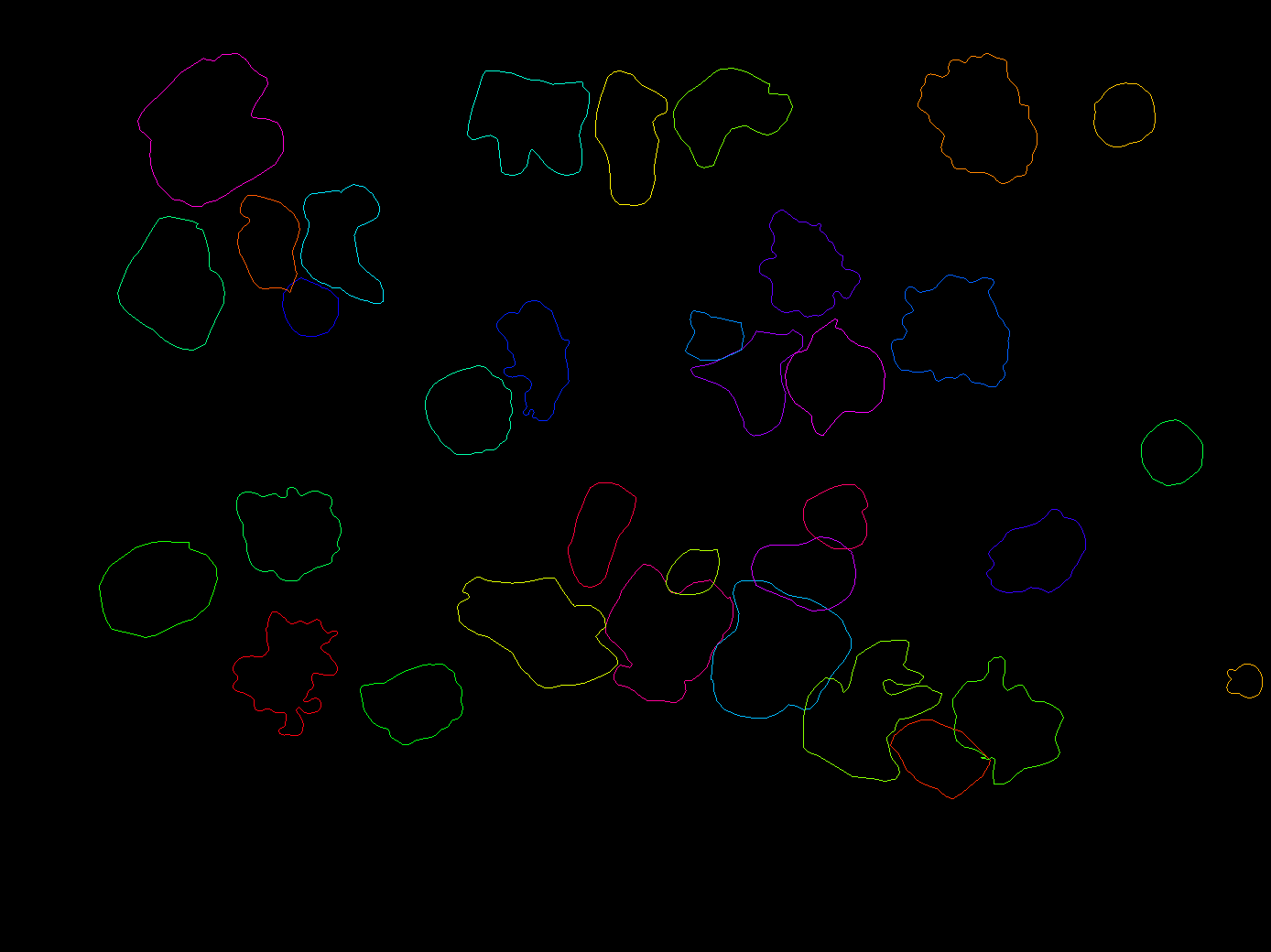

Supplement: Additional file 2 — The zip archive contains real images showing B cell nuclei and cytoskeleton. (ZIP 12390 kb) [file 12859_2017_1591_MOESM2_ESM.zip › B cells/cells0009 gt.png]

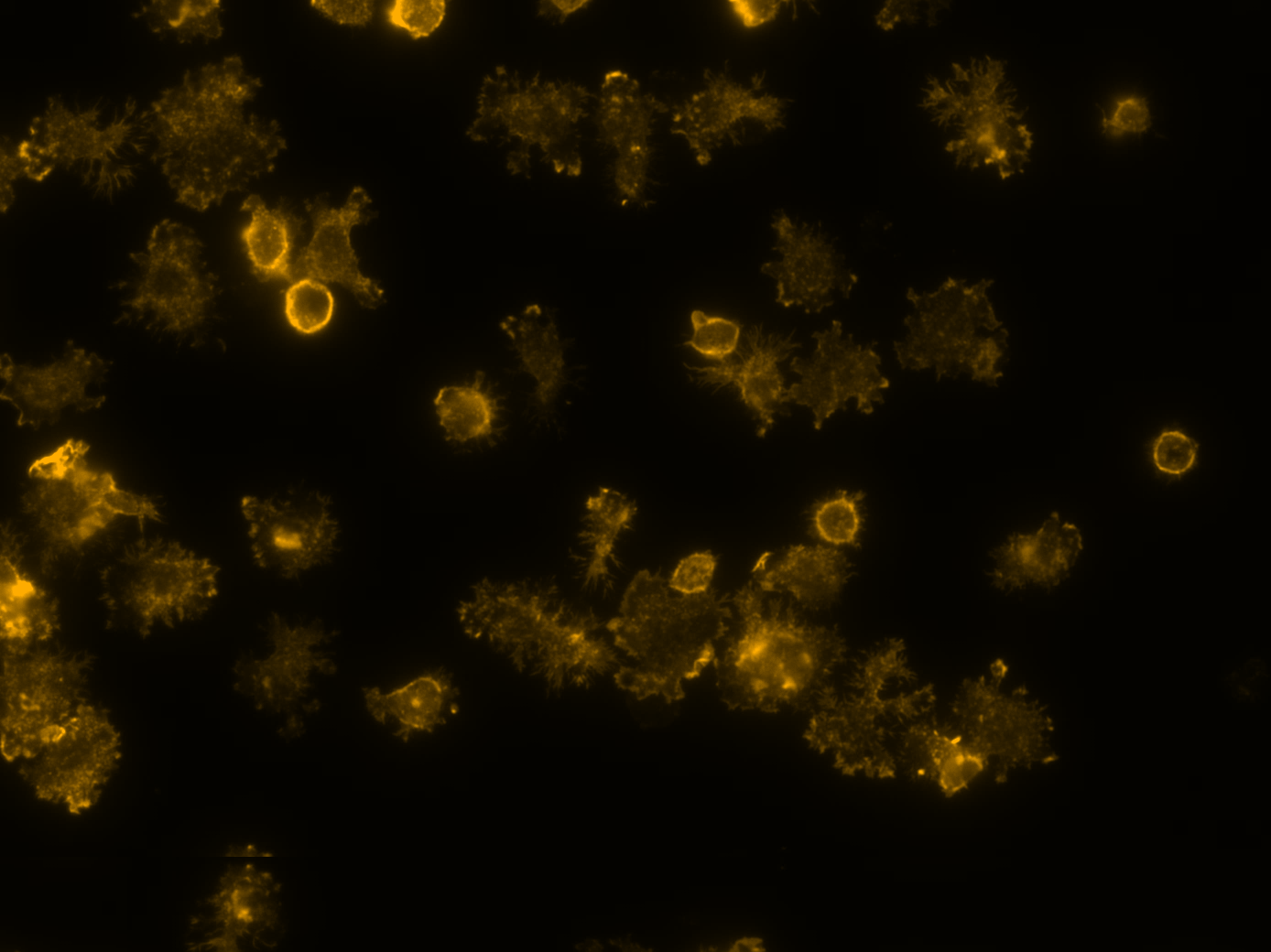

Supplement: Additional file 2 — The zip archive contains real images showing B cell nuclei and cytoskeleton. (ZIP 12390 kb) [file 12859_2017_1591_MOESM2_ESM.zip › B cells/cells0009.png]

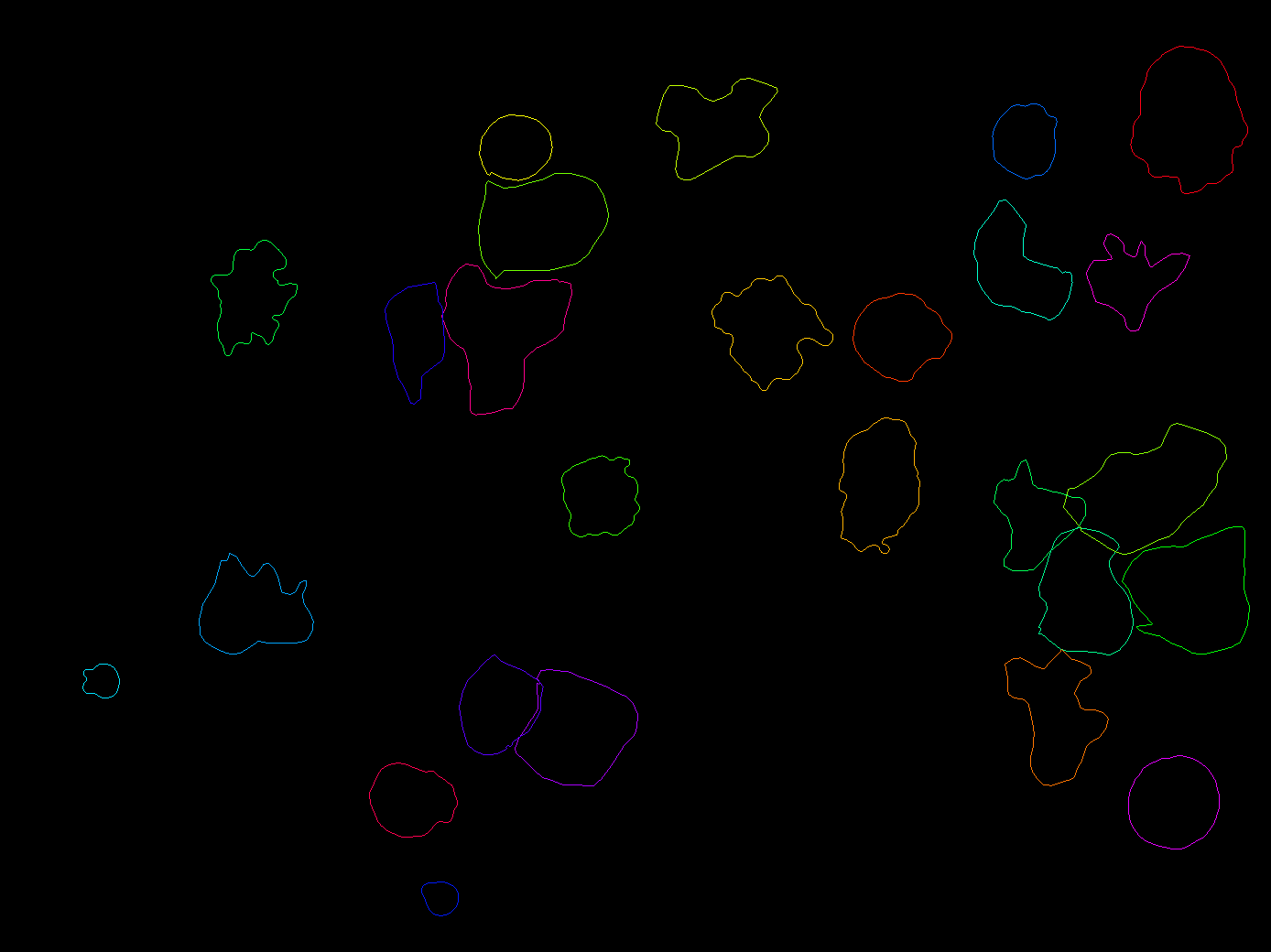

Supplement: Additional file 2 — The zip archive contains real images showing B cell nuclei and cytoskeleton. (ZIP 12390 kb) [file 12859_2017_1591_MOESM2_ESM.zip › B cells/cells0010 gt.png]

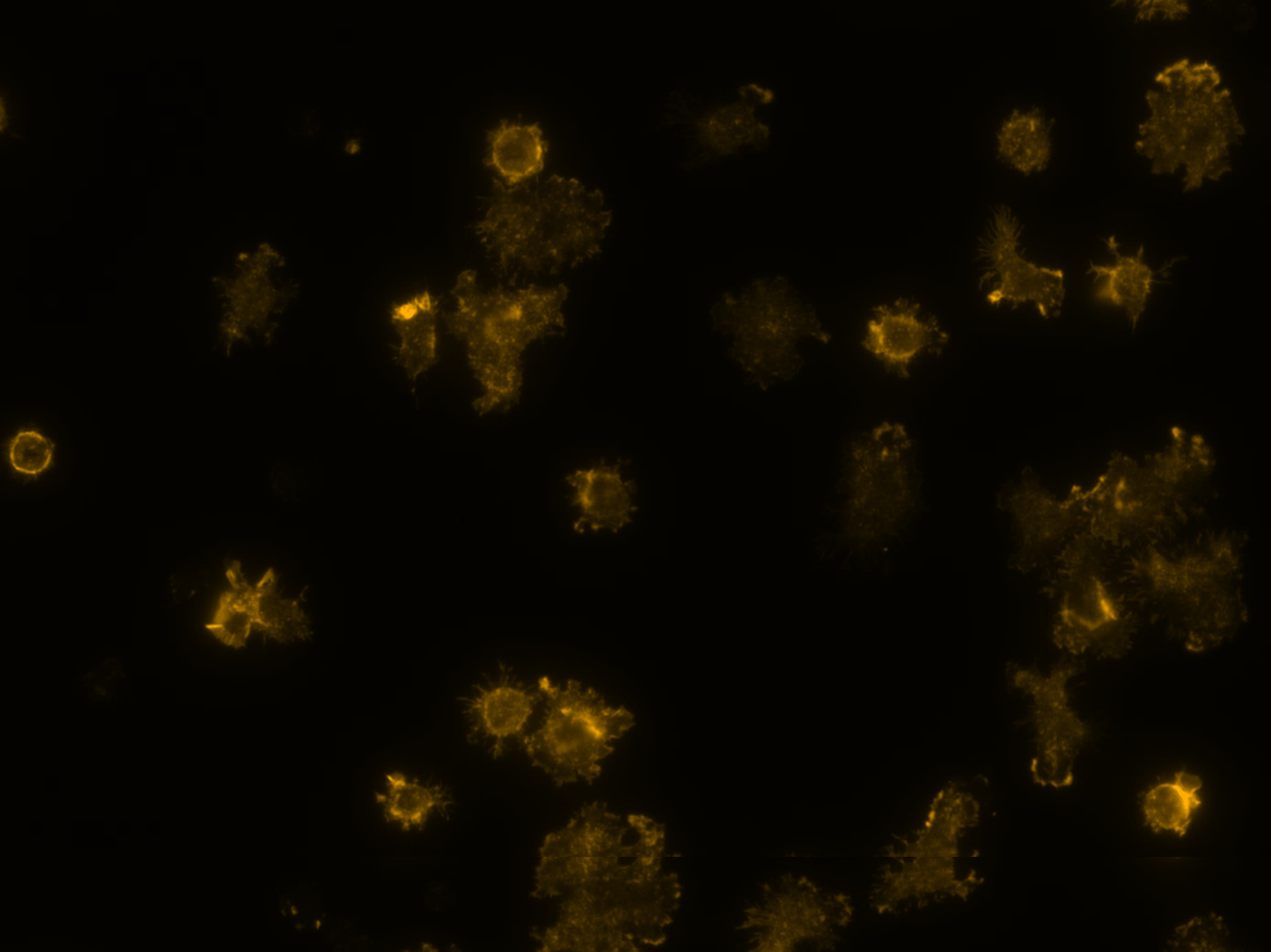

Supplement: Additional file 2 — The zip archive contains real images showing B cell nuclei and cytoskeleton. (ZIP 12390 kb) [file 12859_2017_1591_MOESM2_ESM.zip › B cells/cells0010.png]

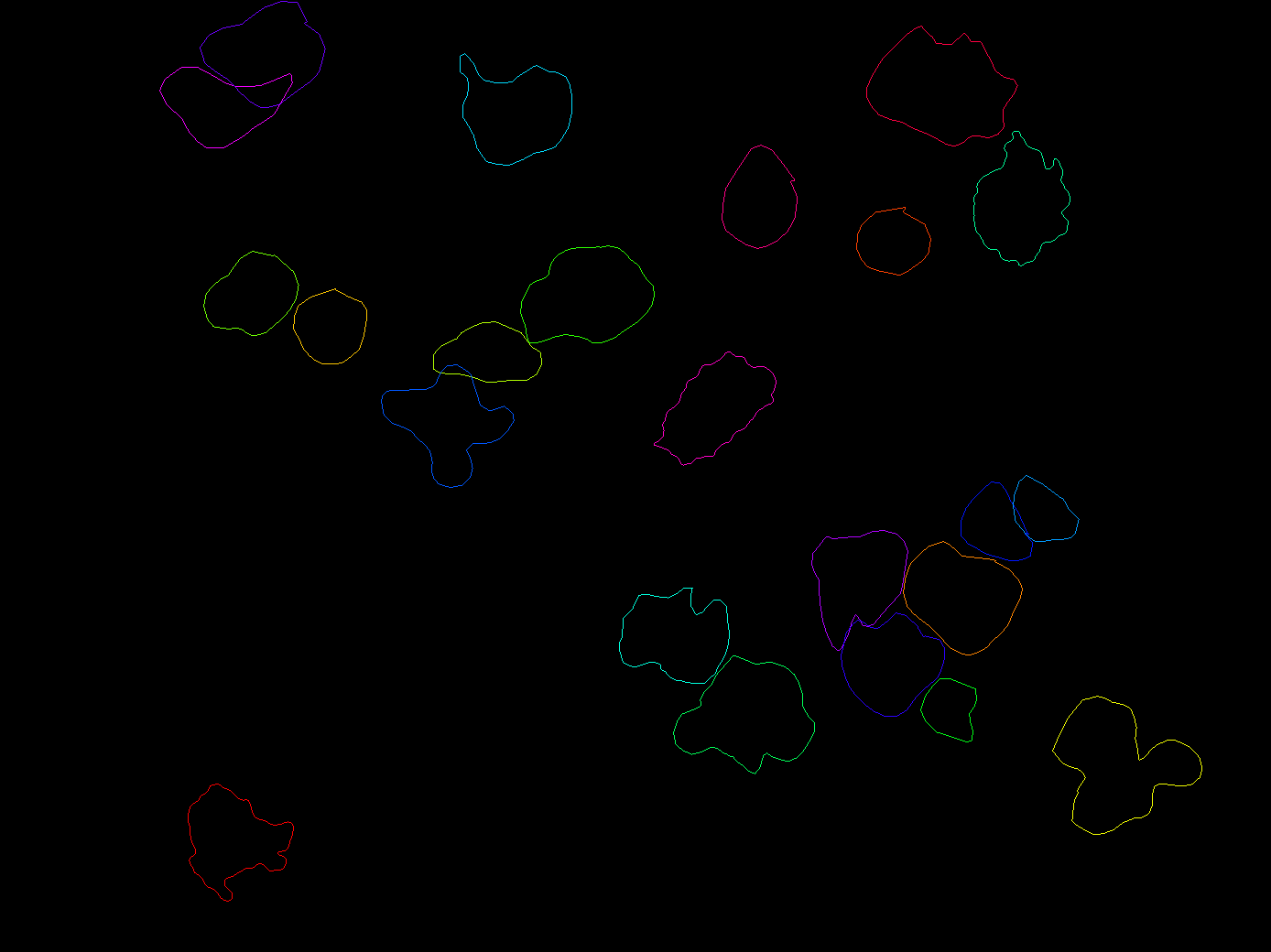

Supplement: Additional file 2 — The zip archive contains real images showing B cell nuclei and cytoskeleton. (ZIP 12390 kb) [file 12859_2017_1591_MOESM2_ESM.zip › B cells/cells0011 gt.png]

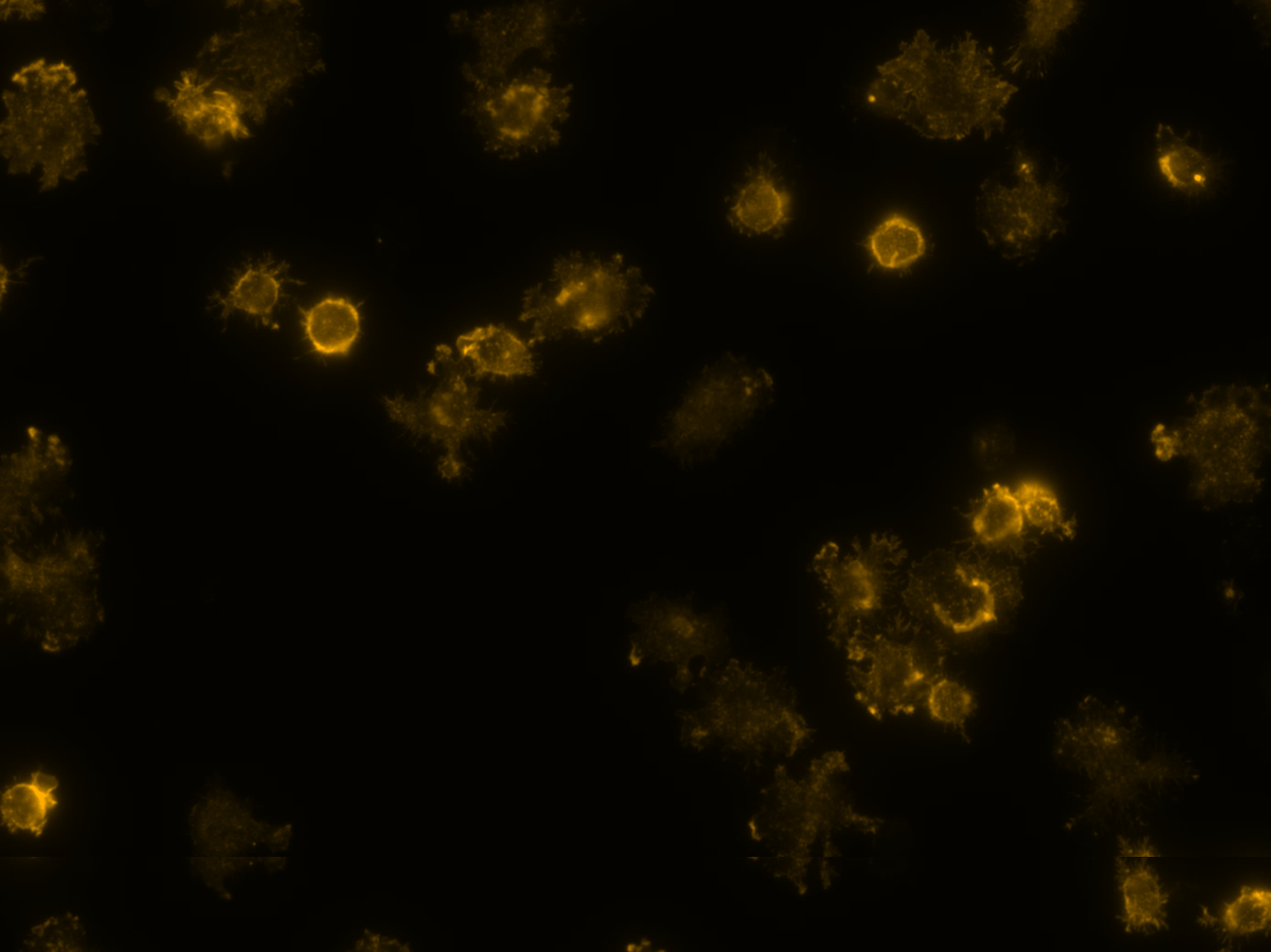

Supplement: Additional file 2 — The zip archive contains real images showing B cell nuclei and cytoskeleton. (ZIP 12390 kb) [file 12859_2017_1591_MOESM2_ESM.zip › B cells/cells0011.png]

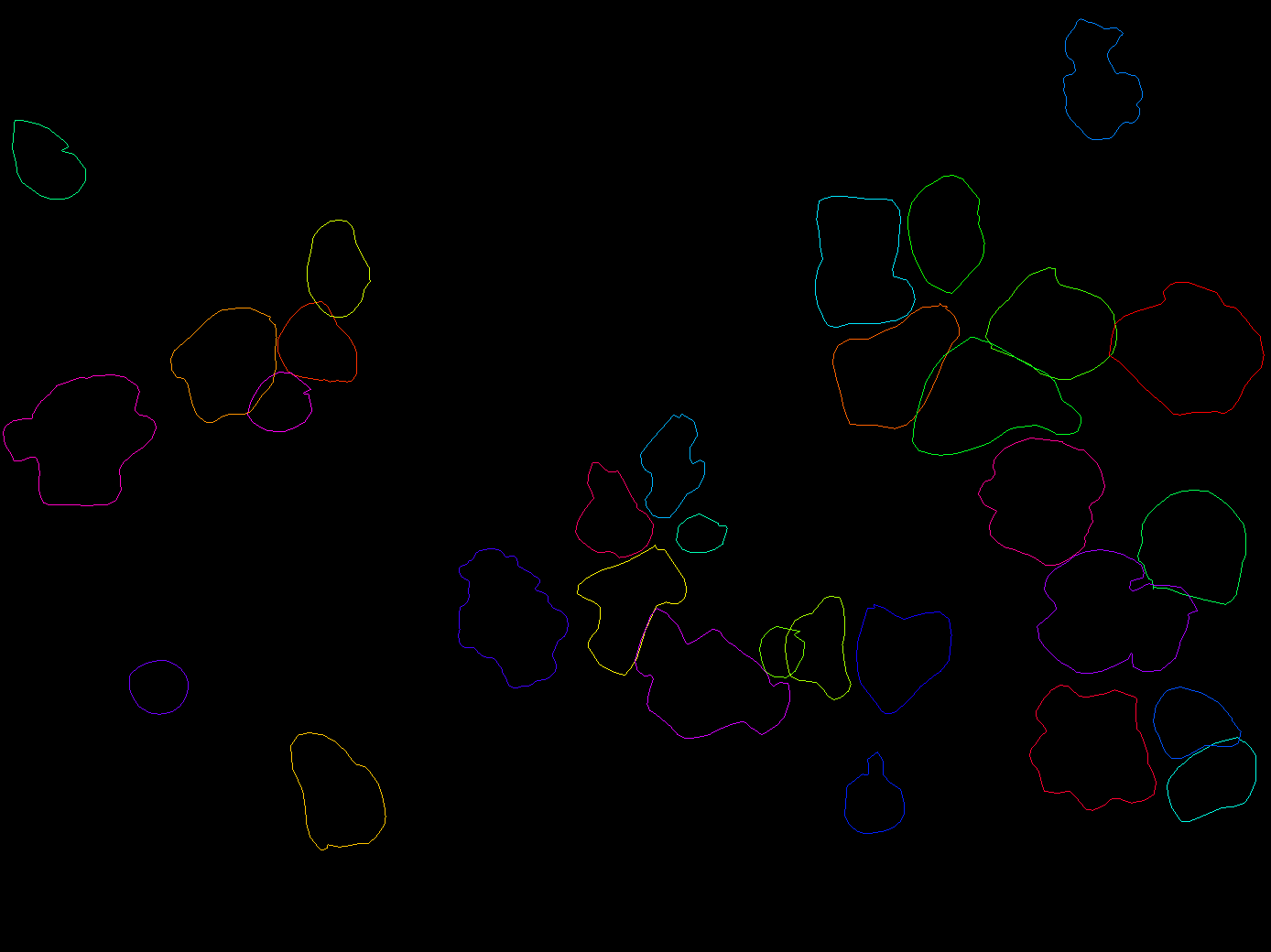

Supplement: Additional file 2 — The zip archive contains real images showing B cell nuclei and cytoskeleton. (ZIP 12390 kb) [file 12859_2017_1591_MOESM2_ESM.zip › B cells/cells0012 gt.png]

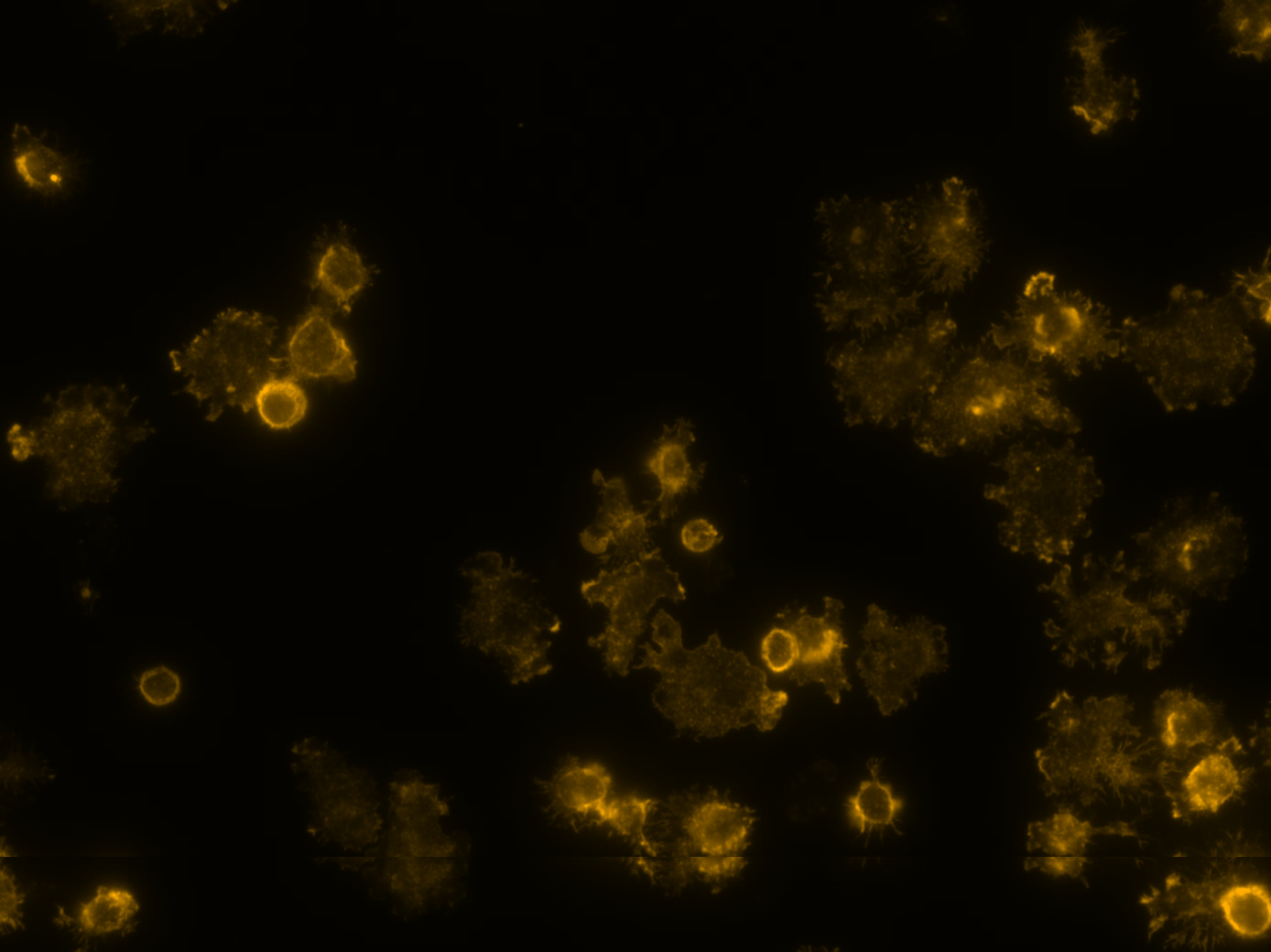

Supplement: Additional file 2 — The zip archive contains real images showing B cell nuclei and cytoskeleton. (ZIP 12390 kb) [file 12859_2017_1591_MOESM2_ESM.zip › B cells/cells0012.png]

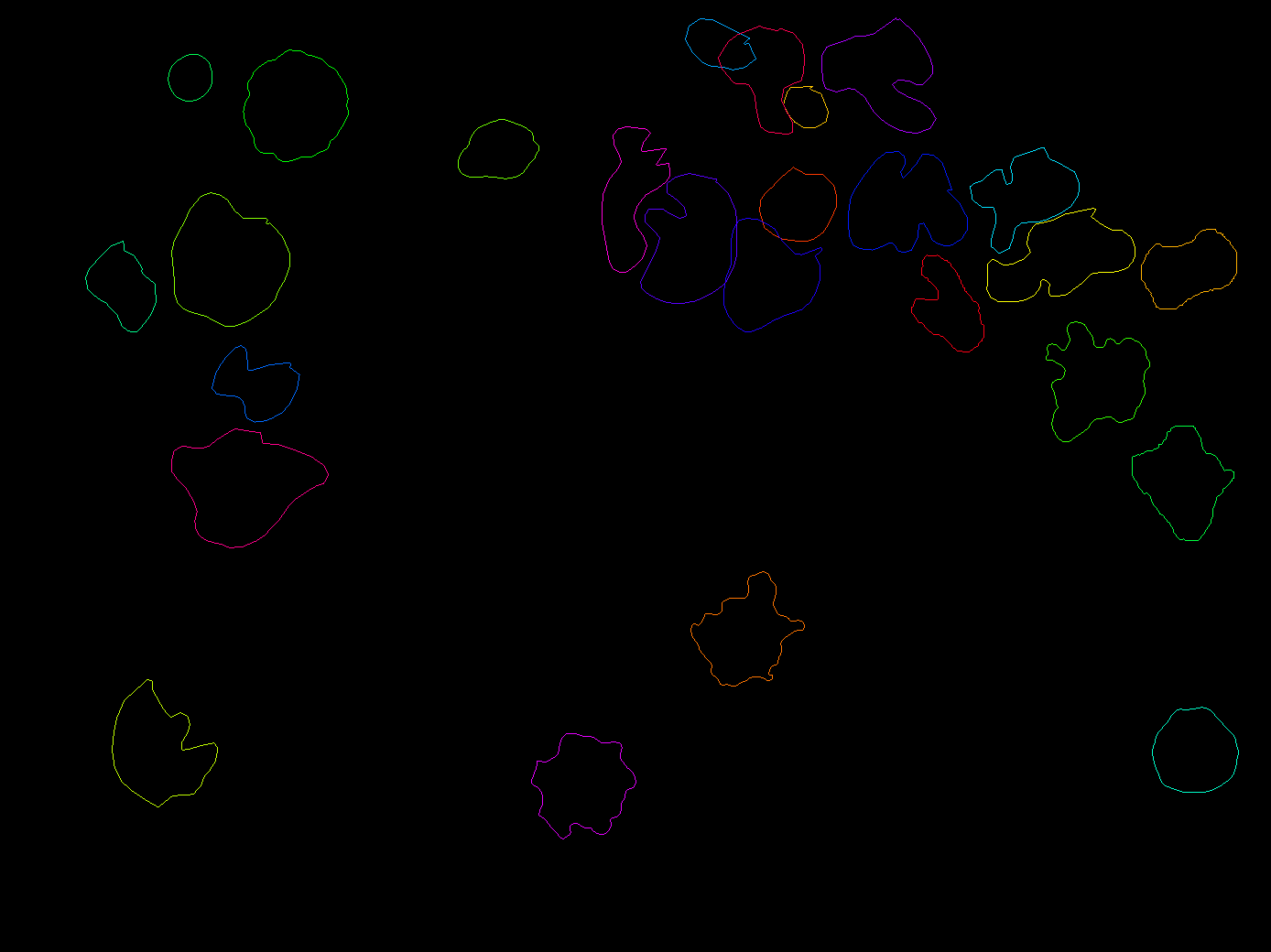

Supplement: Additional file 2 — The zip archive contains real images showing B cell nuclei and cytoskeleton. (ZIP 12390 kb) [file 12859_2017_1591_MOESM2_ESM.zip › B cells/cells0013 gt.png]

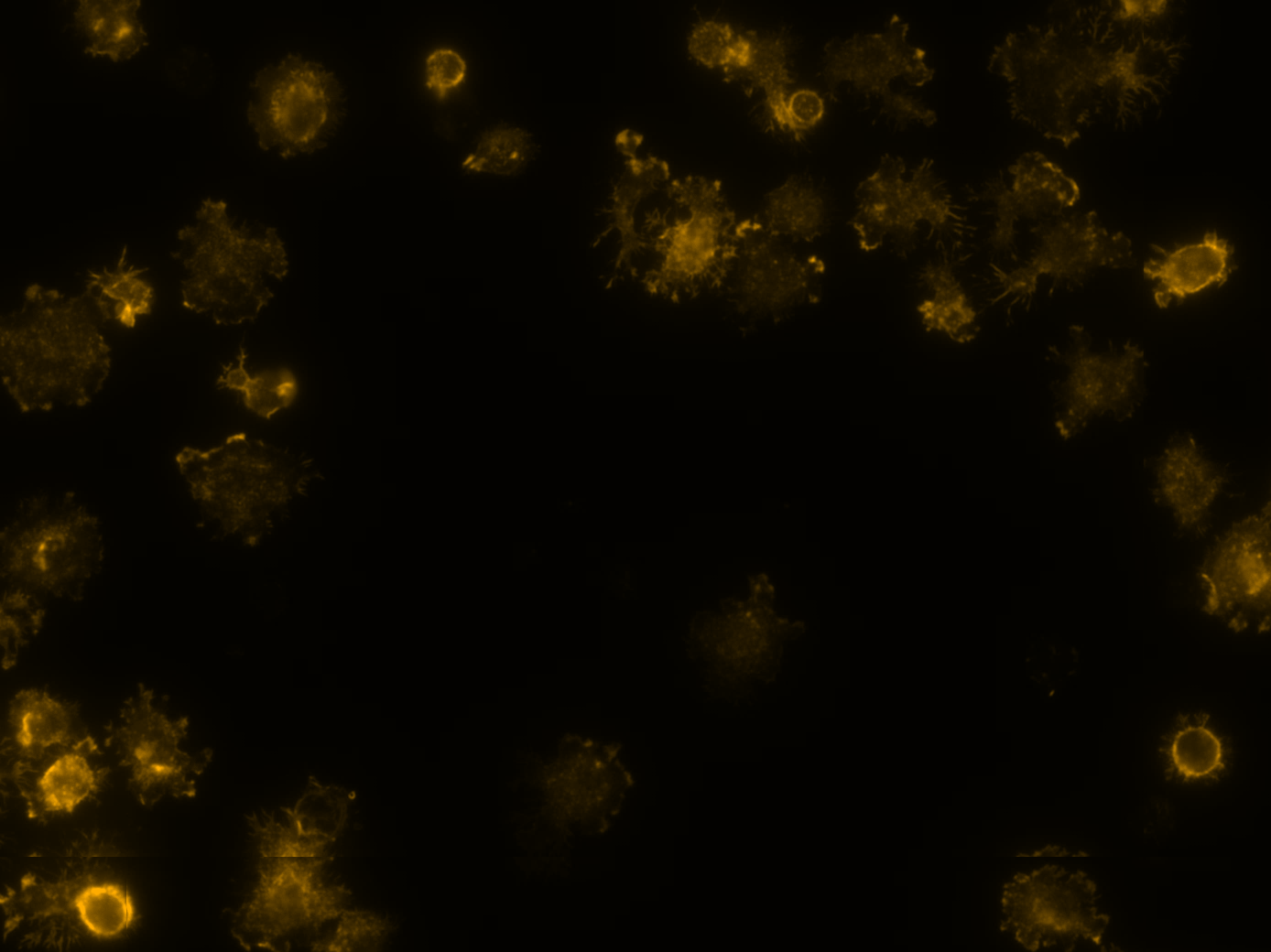

Supplement: Additional file 2 — The zip archive contains real images showing B cell nuclei and cytoskeleton. (ZIP 12390 kb) [file 12859_2017_1591_MOESM2_ESM.zip › B cells/cells0013.png]

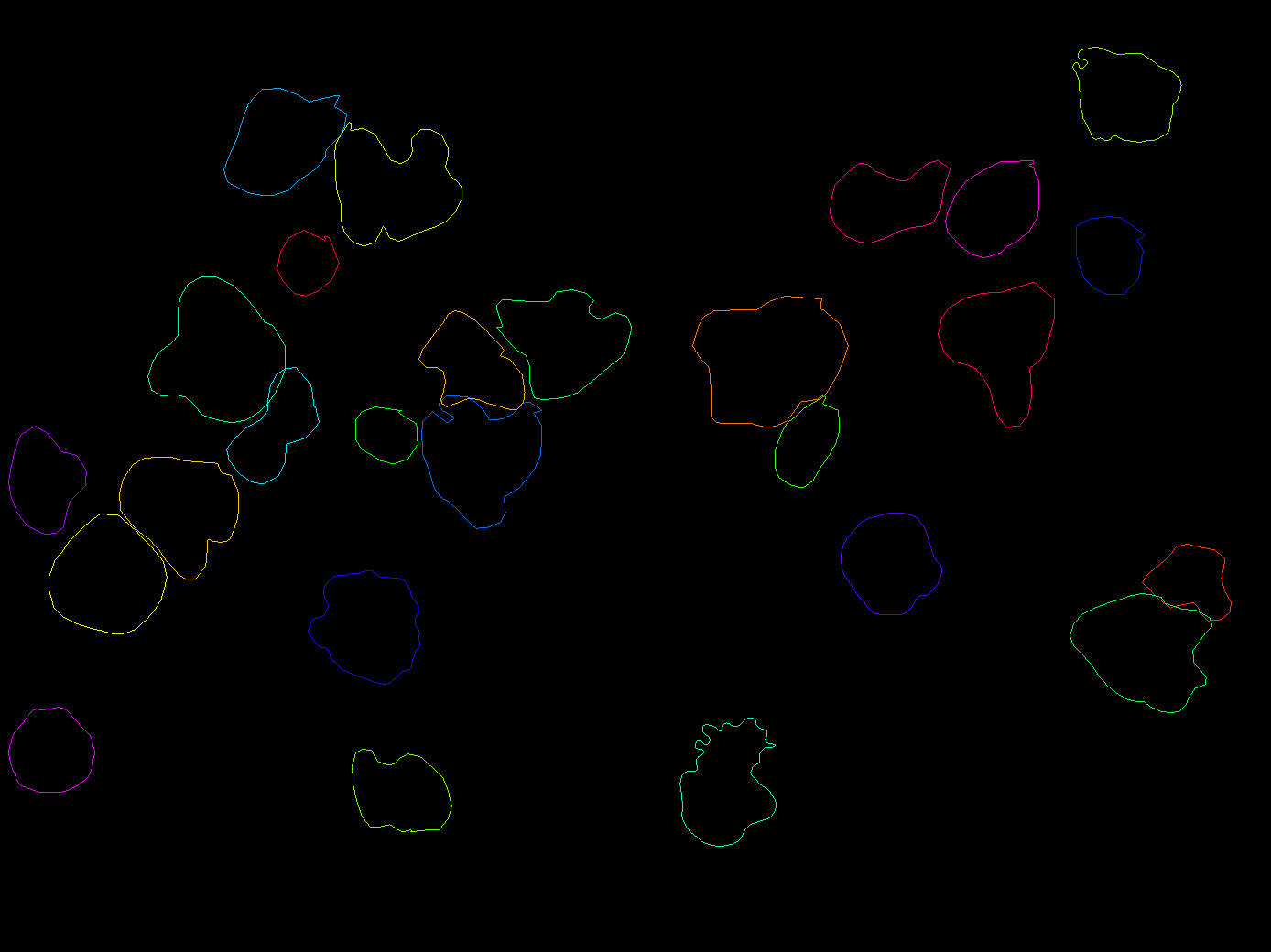

Supplement: Additional file 2 — The zip archive contains real images showing B cell nuclei and cytoskeleton. (ZIP 12390 kb) [file 12859_2017_1591_MOESM2_ESM.zip › B cells/cells0014 gt.png]

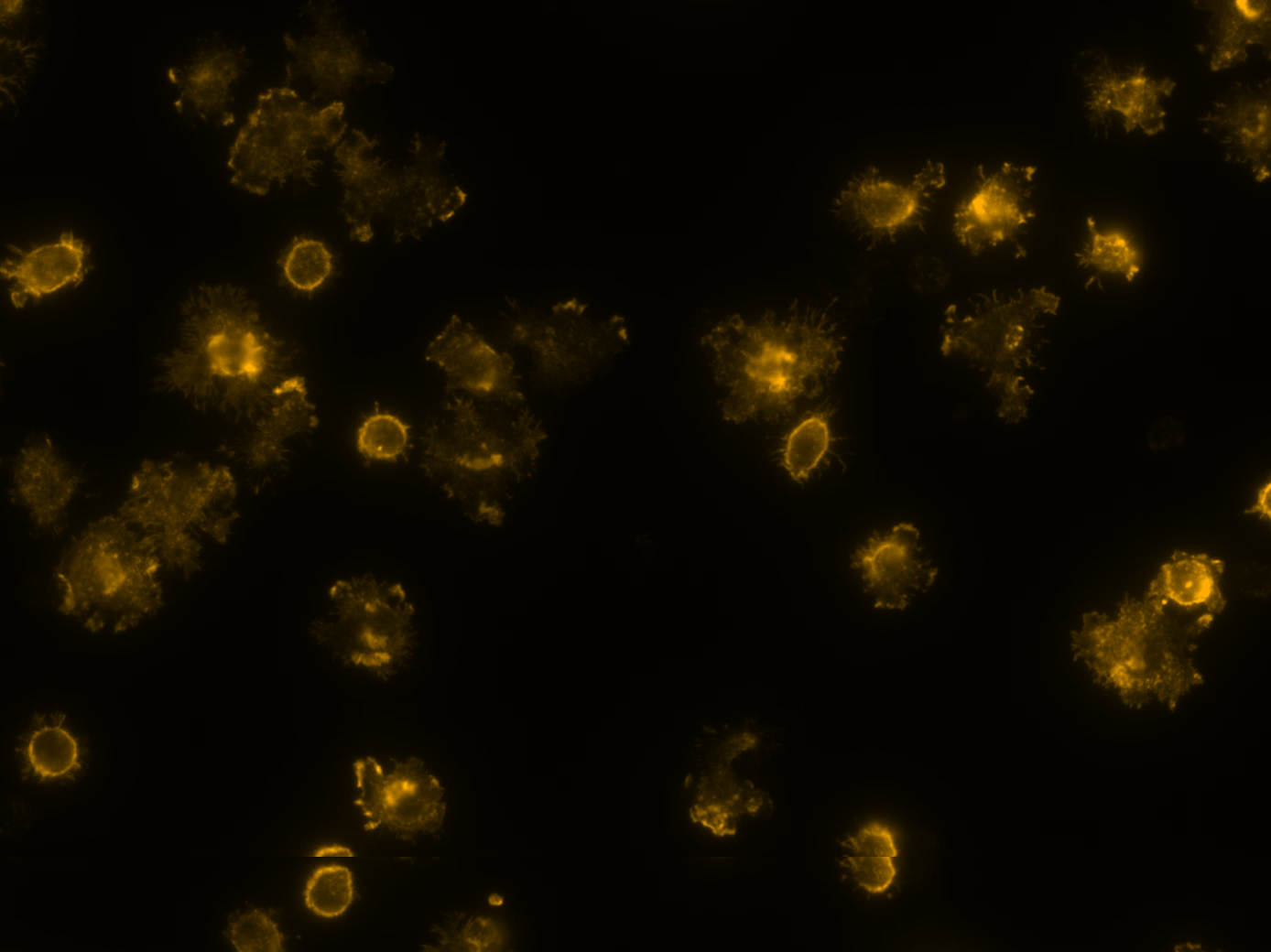

Supplement: Additional file 2 — The zip archive contains real images showing B cell nuclei and cytoskeleton. (ZIP 12390 kb) [file 12859_2017_1591_MOESM2_ESM.zip › B cells/cells0014.png]

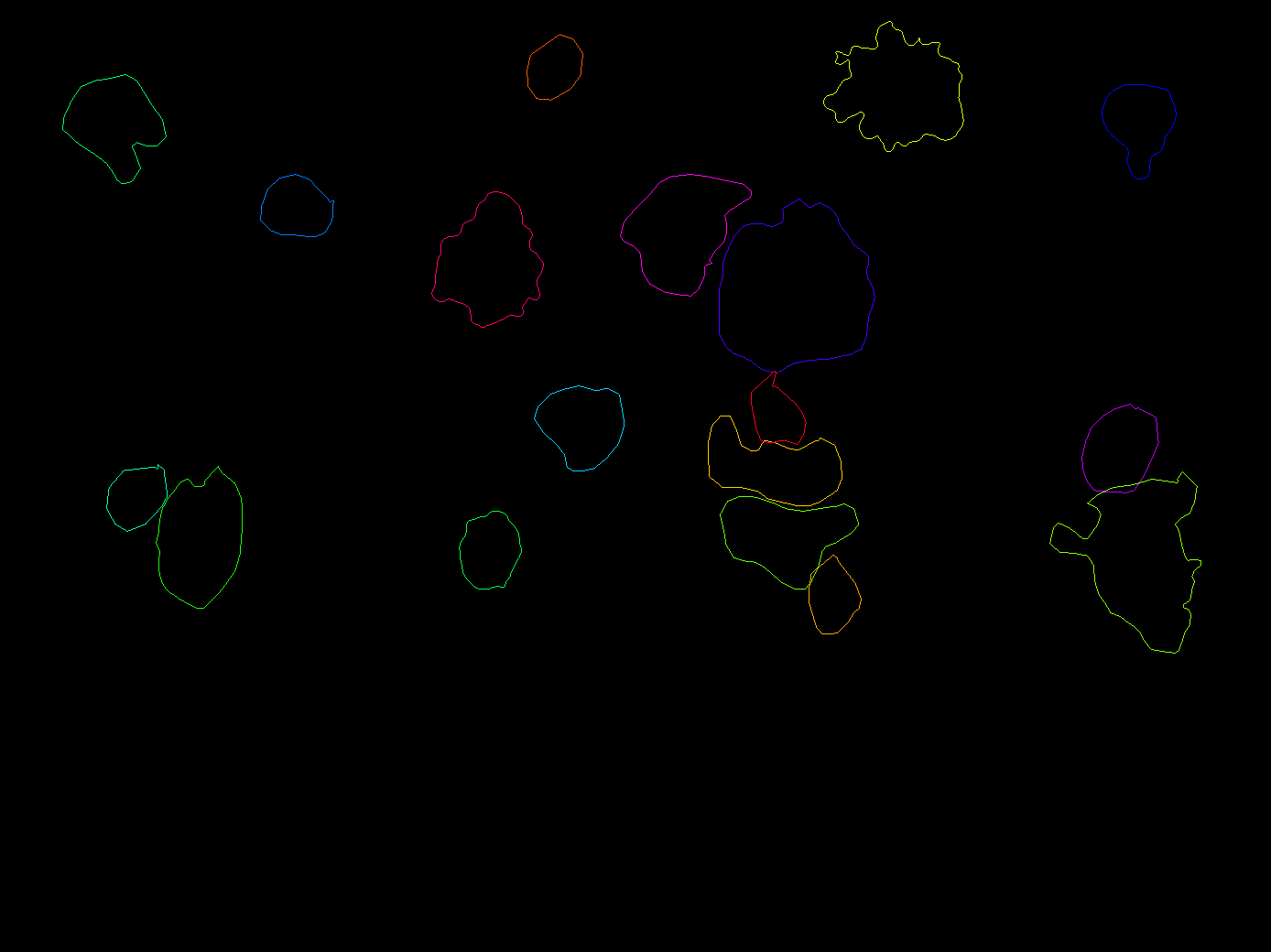

Supplement: Additional file 2 — The zip archive contains real images showing B cell nuclei and cytoskeleton. (ZIP 12390 kb) [file 12859_2017_1591_MOESM2_ESM.zip › B cells/cells0015 gt.png]

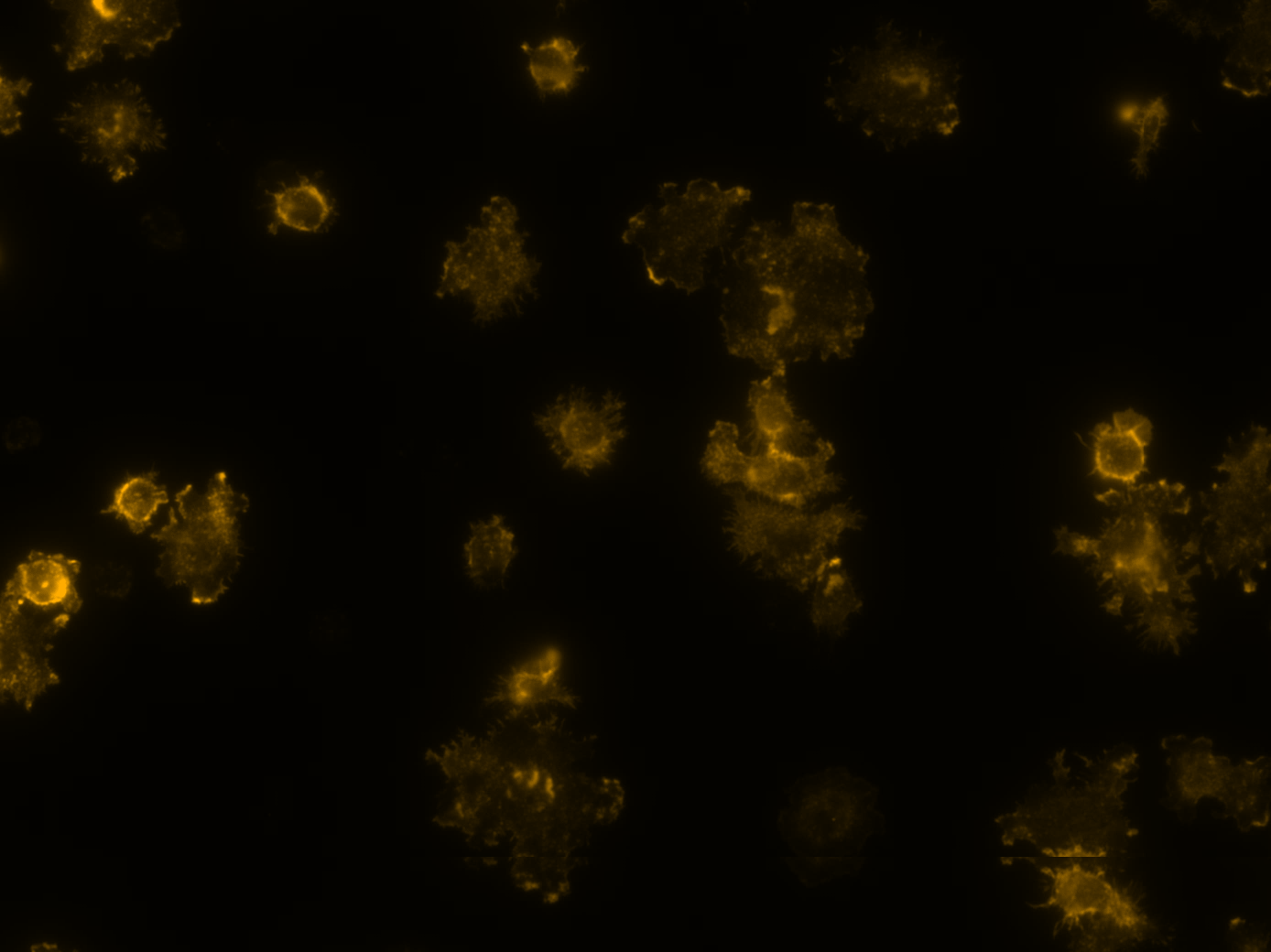

Supplement: Additional file 2 — The zip archive contains real images showing B cell nuclei and cytoskeleton. (ZIP 12390 kb) [file 12859_2017_1591_MOESM2_ESM.zip › B cells/cells0015.png]

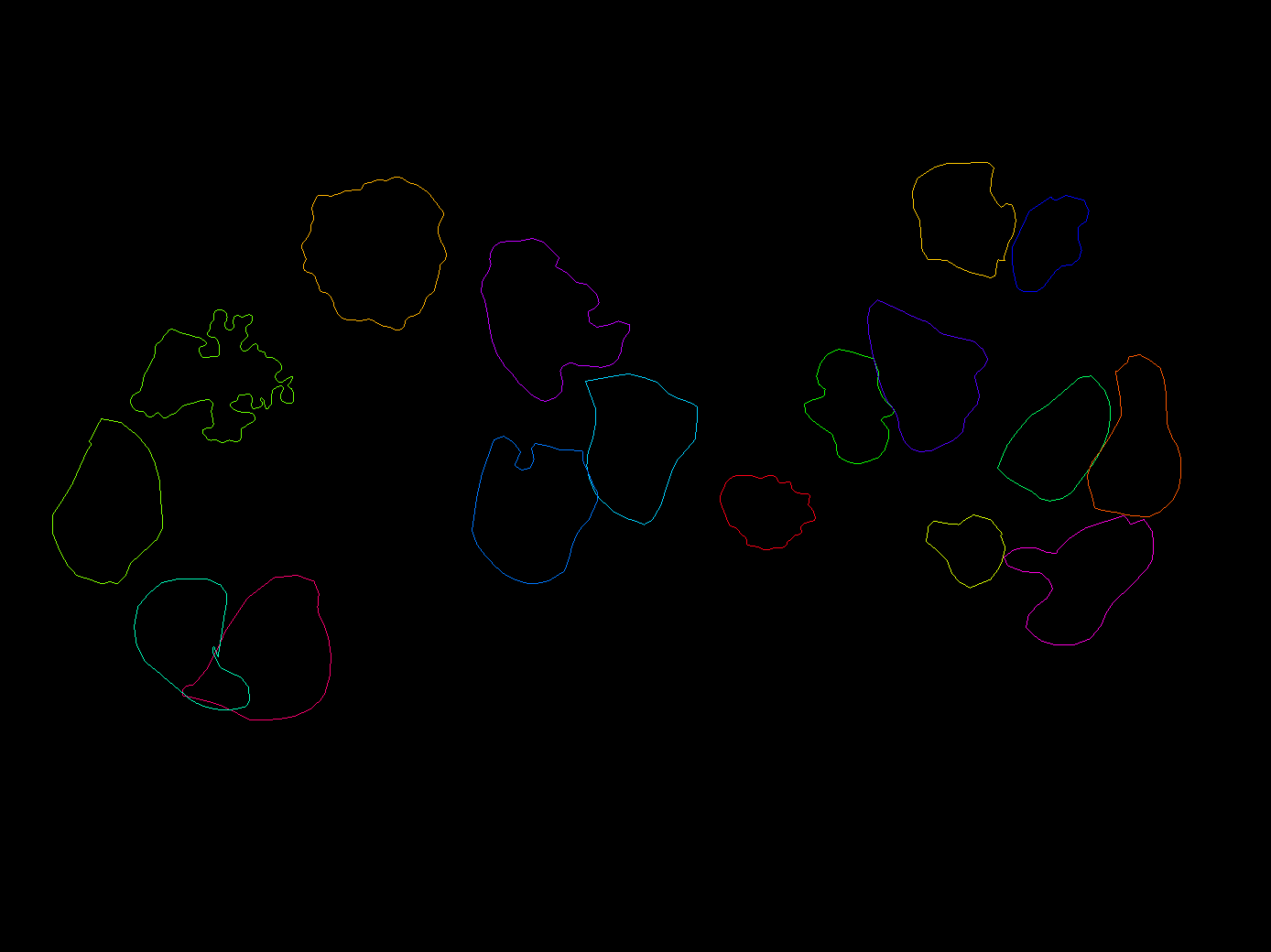

Supplement: Additional file 2 — The zip archive contains real images showing B cell nuclei and cytoskeleton. (ZIP 12390 kb) [file 12859_2017_1591_MOESM2_ESM.zip › B cells/cells0016 gt.png]

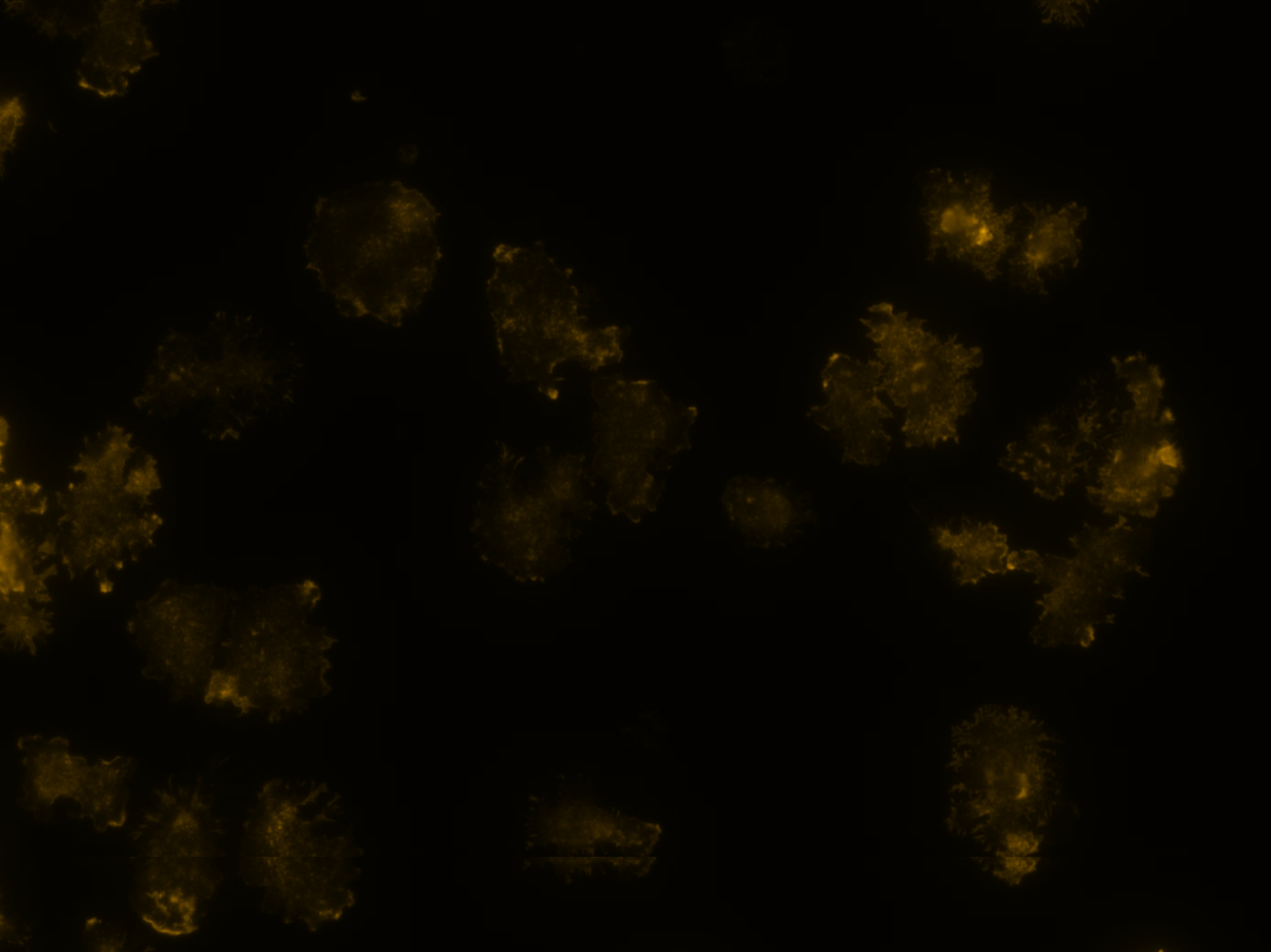

Supplement: Additional file 2 — The zip archive contains real images showing B cell nuclei and cytoskeleton. (ZIP 12390 kb) [file 12859_2017_1591_MOESM2_ESM.zip › B cells/cells0016.png]

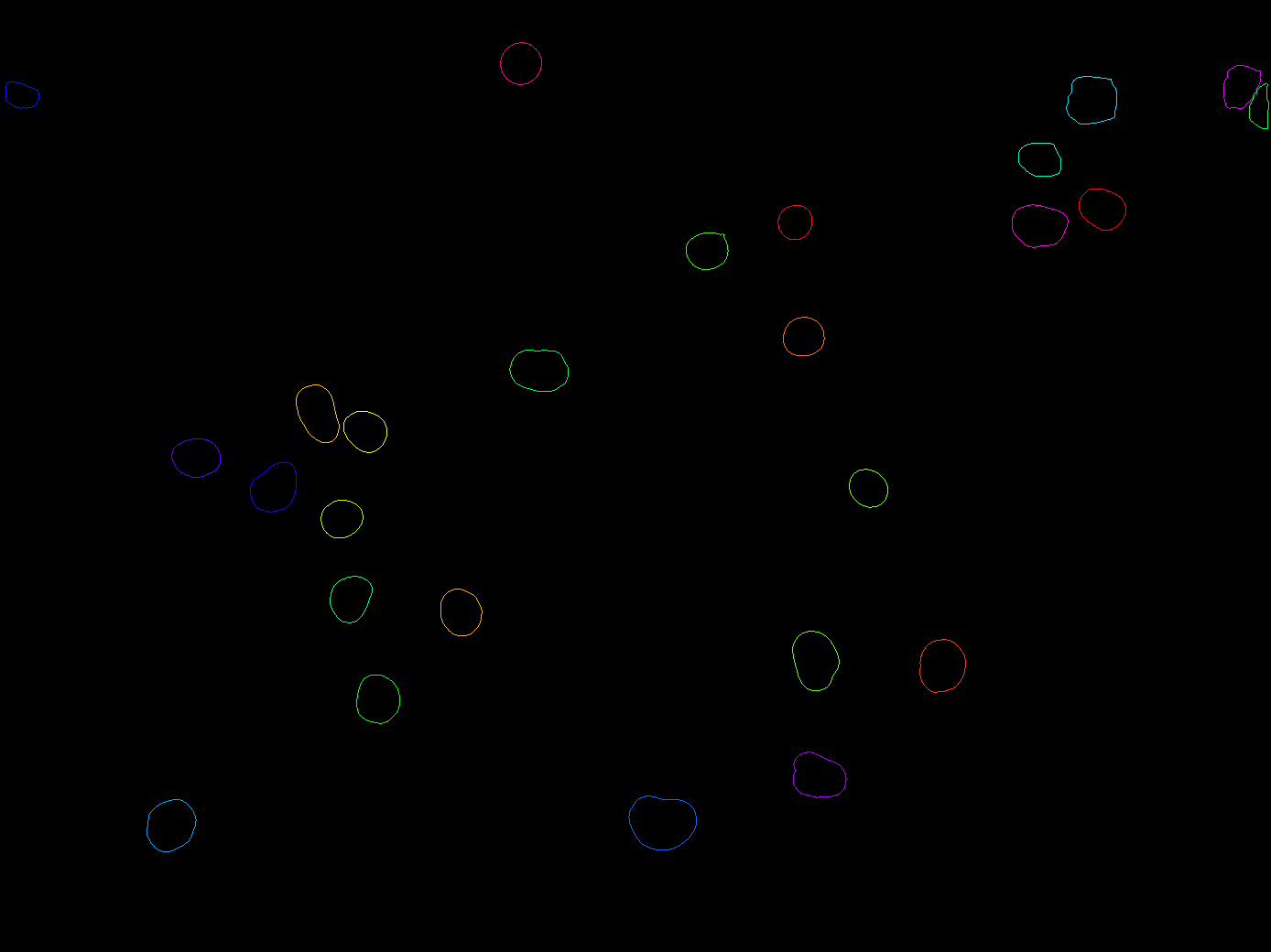

Supplement: Additional file 2 — The zip archive contains real images showing B cell nuclei and cytoskeleton. (ZIP 12390 kb) [file 12859_2017_1591_MOESM2_ESM.zip › B cells/nuclei0001 gt.png]

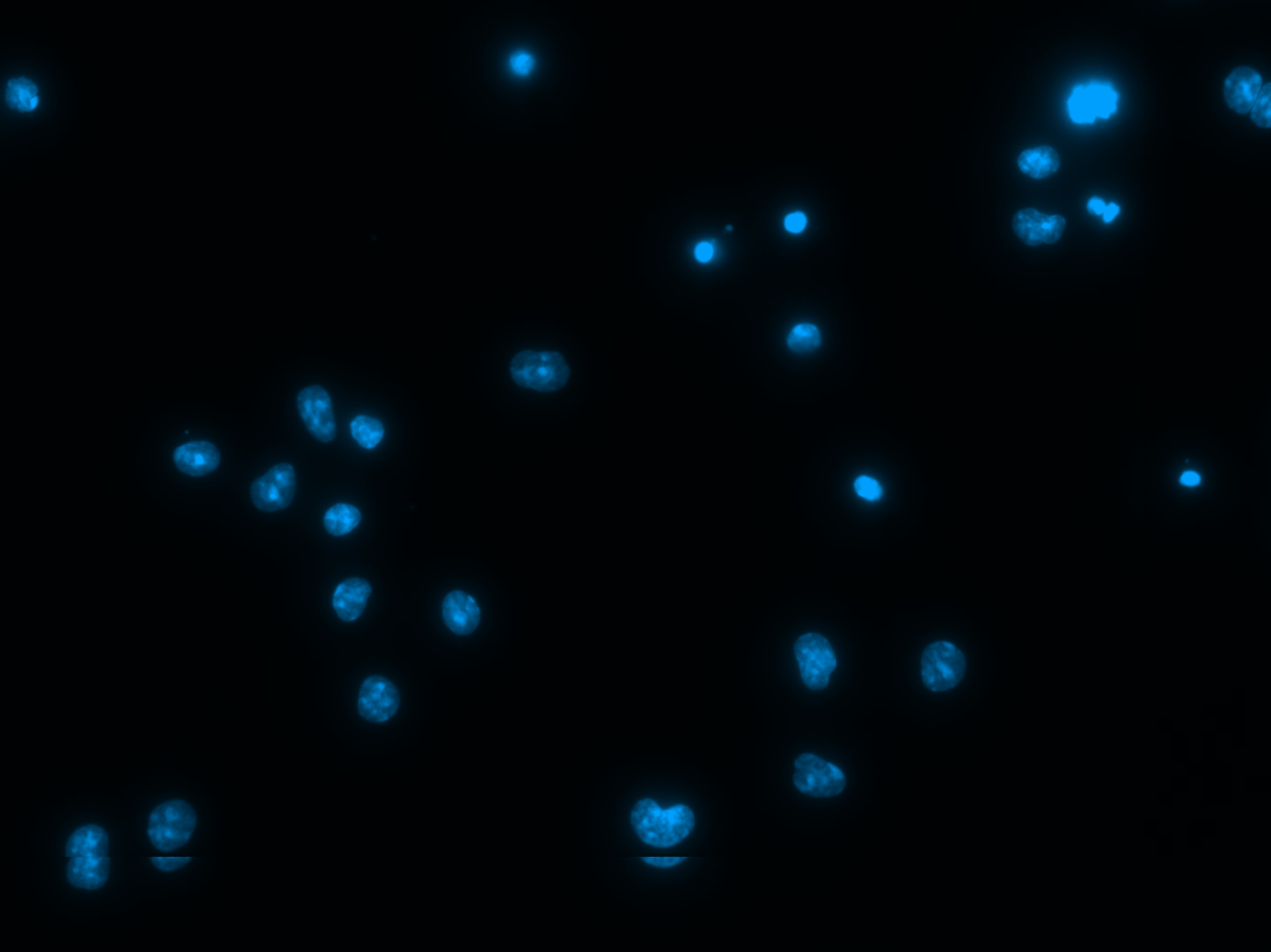

Supplement: Additional file 2 — The zip archive contains real images showing B cell nuclei and cytoskeleton. (ZIP 12390 kb) [file 12859_2017_1591_MOESM2_ESM.zip › B cells/nuclei0001.png]

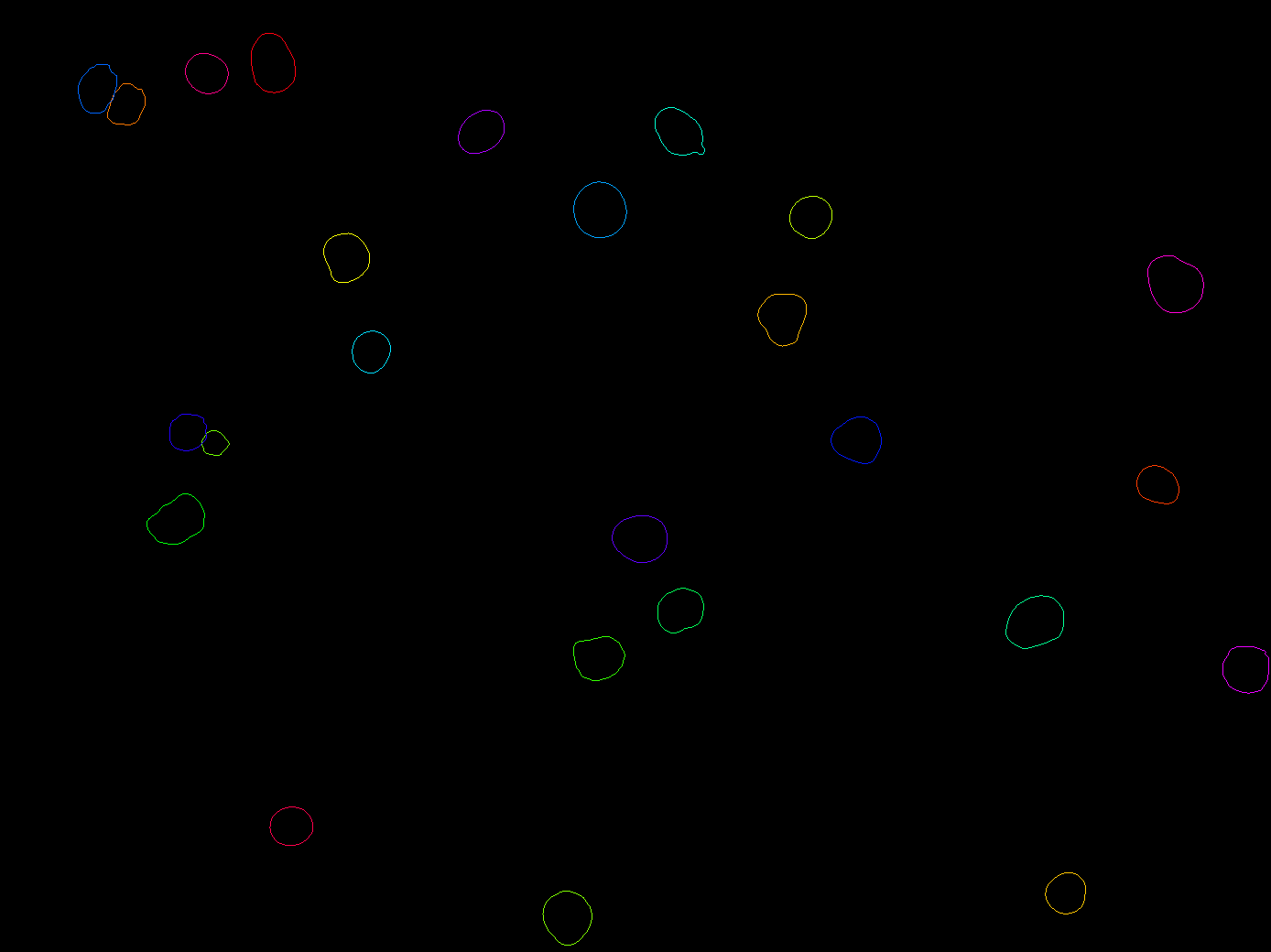

Supplement: Additional file 2 — The zip archive contains real images showing B cell nuclei and cytoskeleton. (ZIP 12390 kb) [file 12859_2017_1591_MOESM2_ESM.zip › B cells/nuclei0002 gt.png]

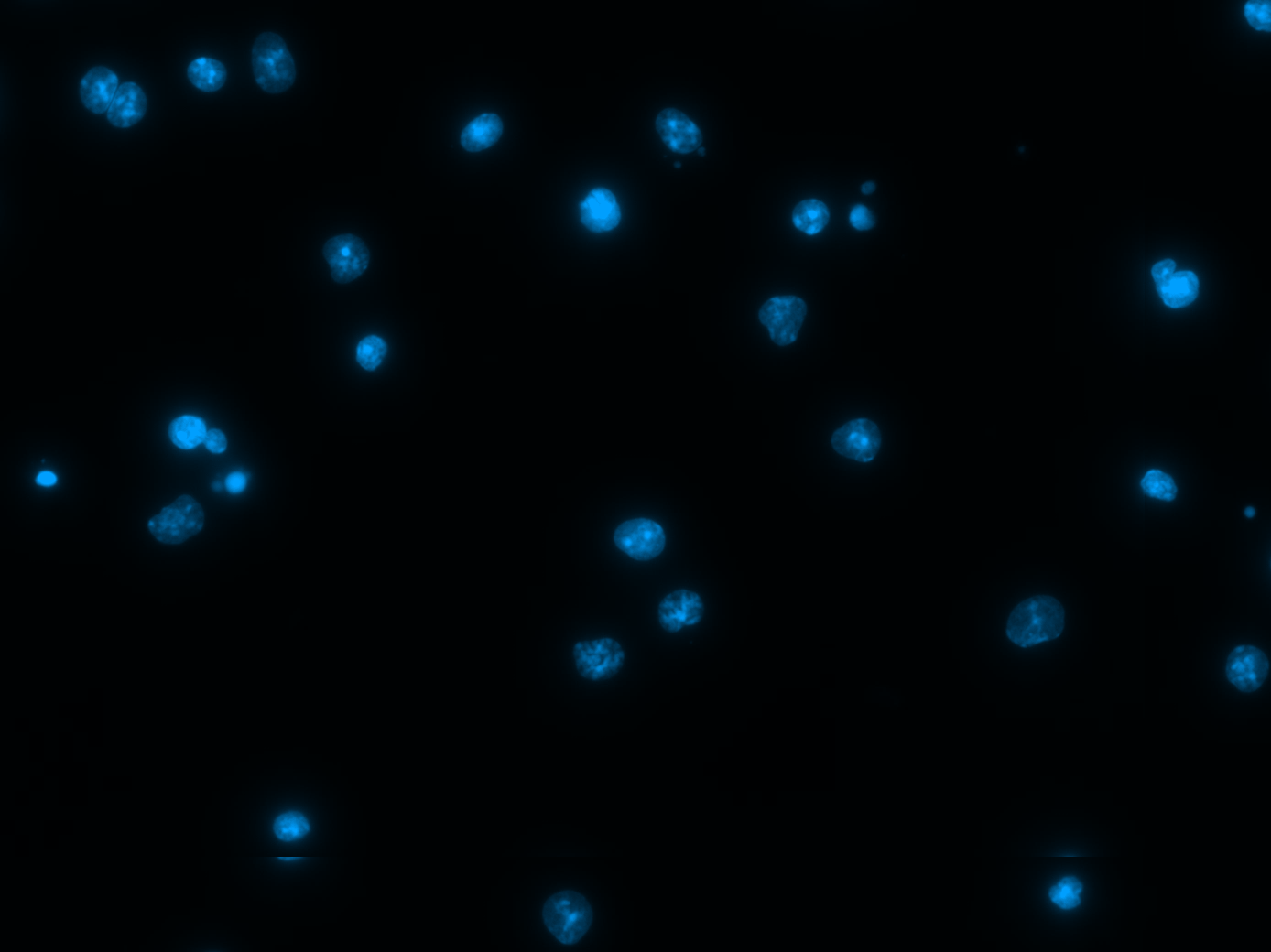

Supplement: Additional file 2 — The zip archive contains real images showing B cell nuclei and cytoskeleton. (ZIP 12390 kb) [file 12859_2017_1591_MOESM2_ESM.zip › B cells/nuclei0002.png]

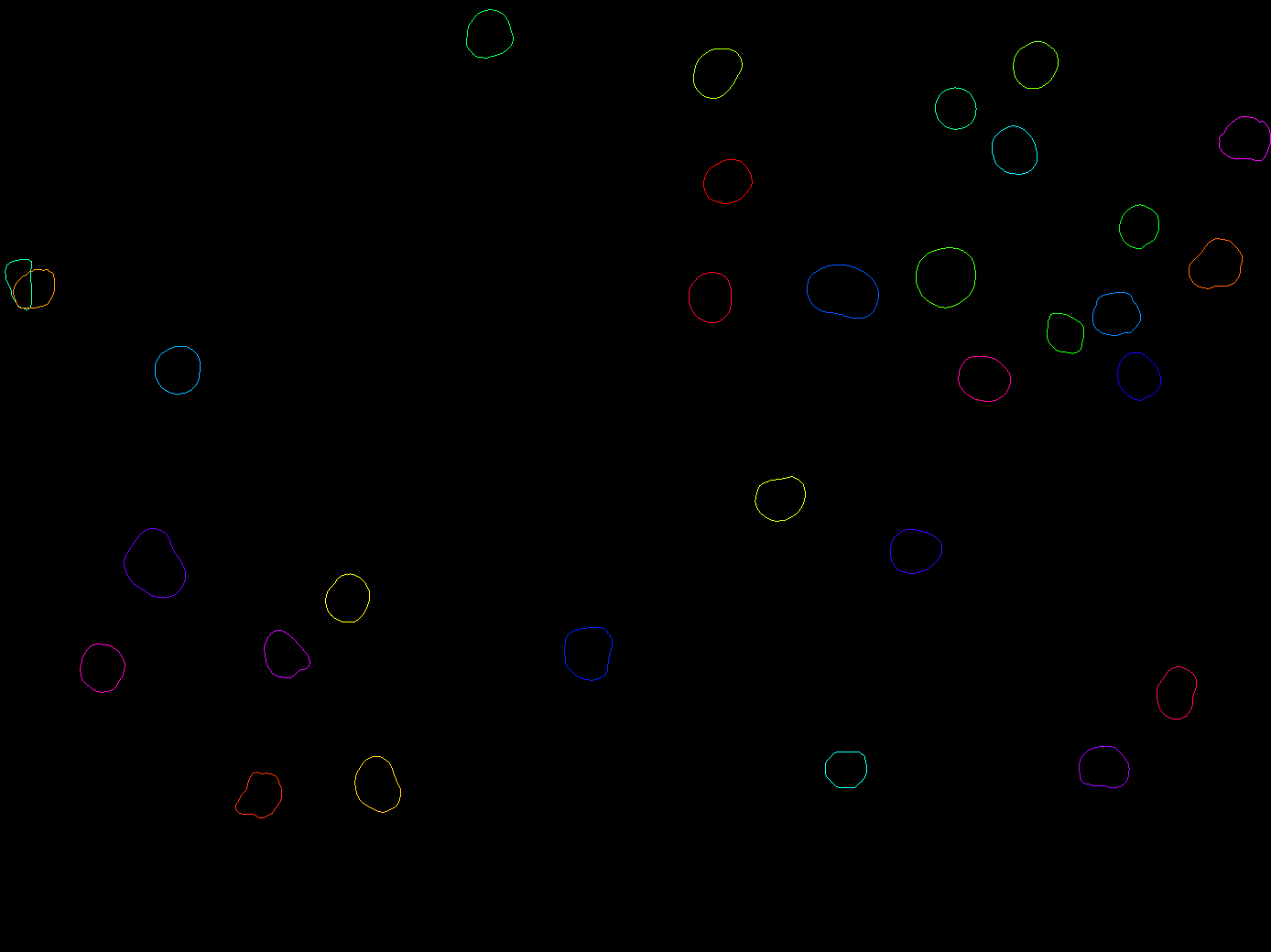

Supplement: Additional file 2 — The zip archive contains real images showing B cell nuclei and cytoskeleton. (ZIP 12390 kb) [file 12859_2017_1591_MOESM2_ESM.zip › B cells/nuclei0003 gt.png]

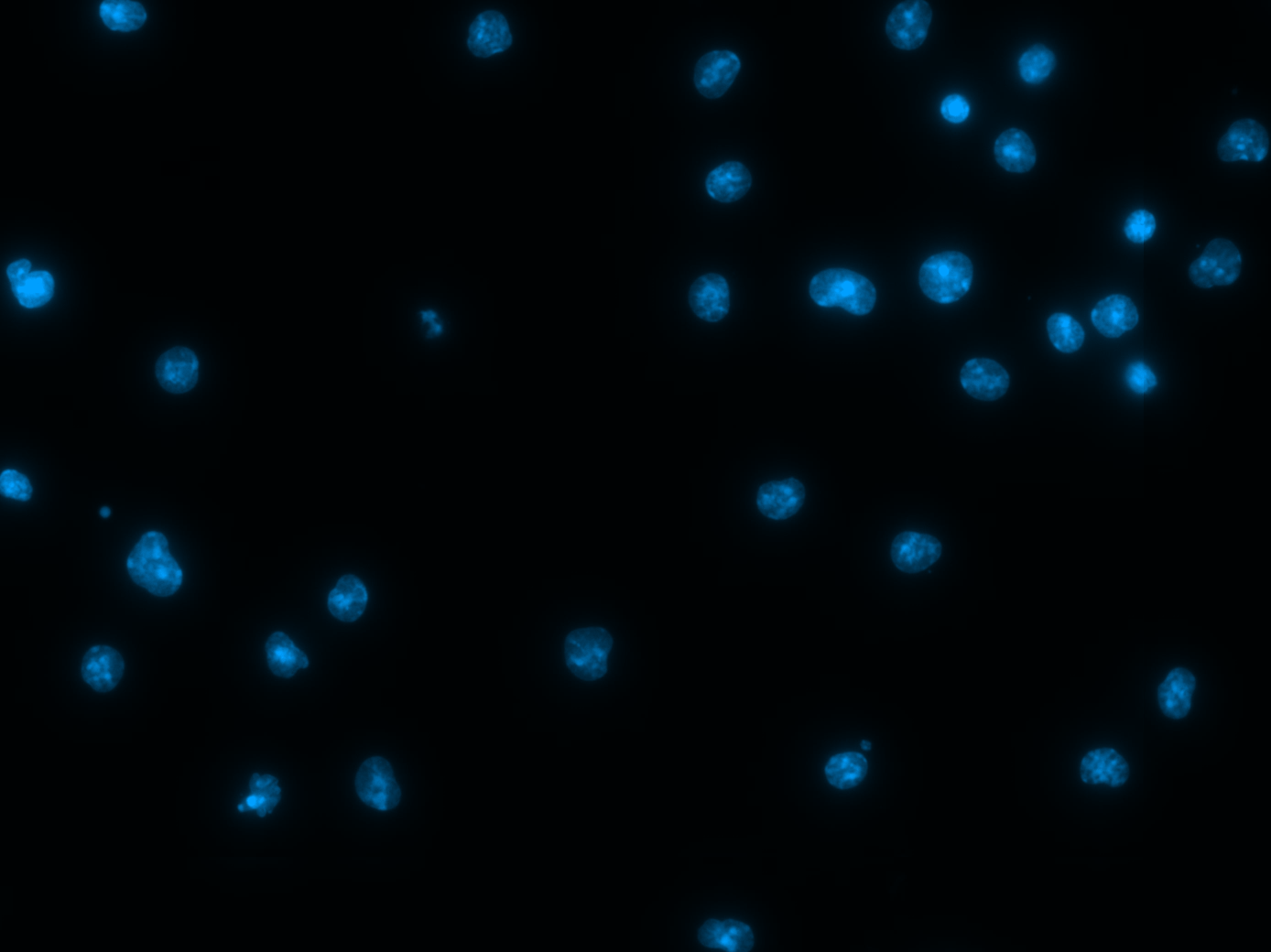

Supplement: Additional file 2 — The zip archive contains real images showing B cell nuclei and cytoskeleton. (ZIP 12390 kb) [file 12859_2017_1591_MOESM2_ESM.zip › B cells/nuclei0003.png]

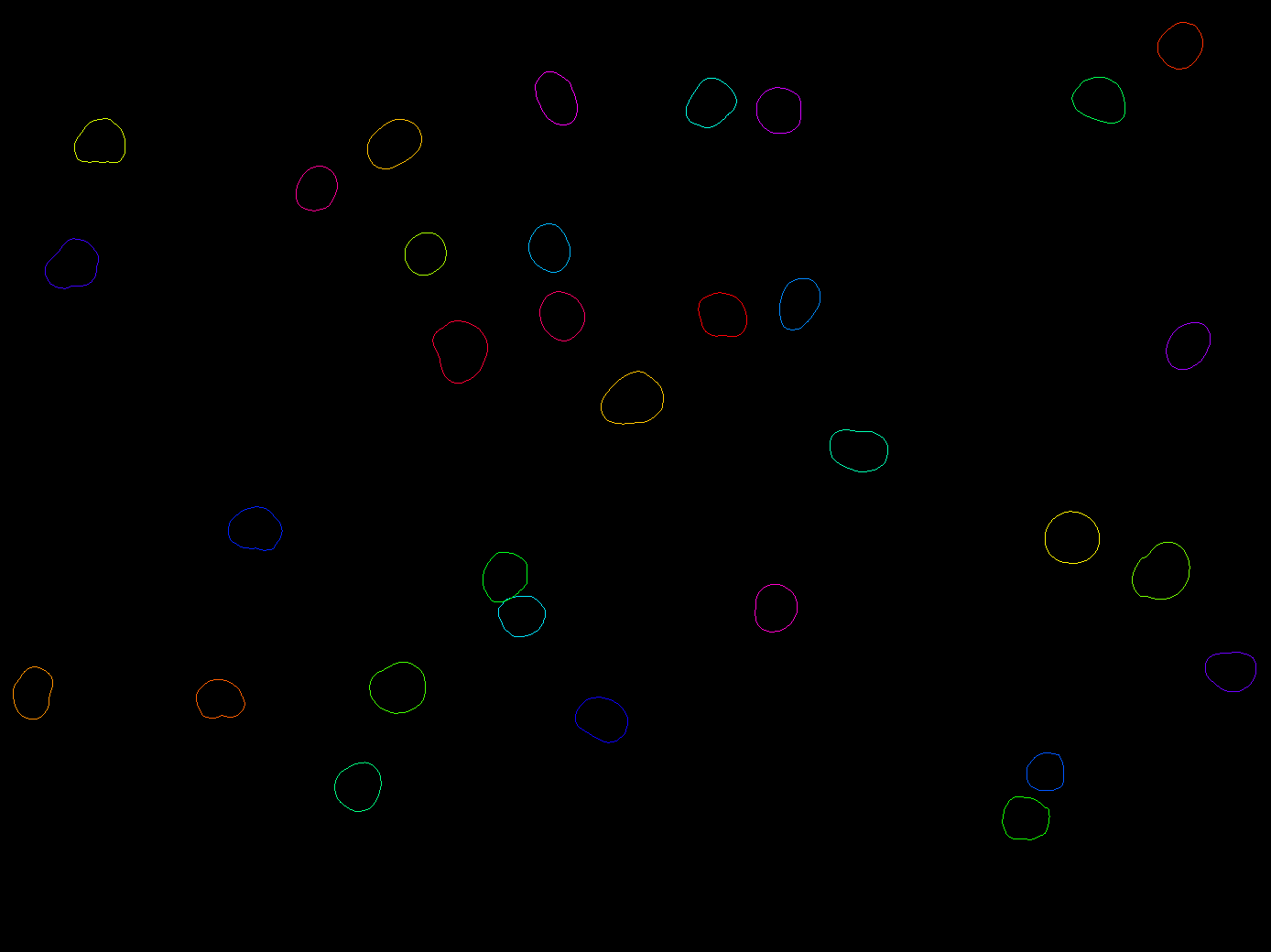

Supplement: Additional file 2 — The zip archive contains real images showing B cell nuclei and cytoskeleton. (ZIP 12390 kb) [file 12859_2017_1591_MOESM2_ESM.zip › B cells/nuclei0004 gt.png]

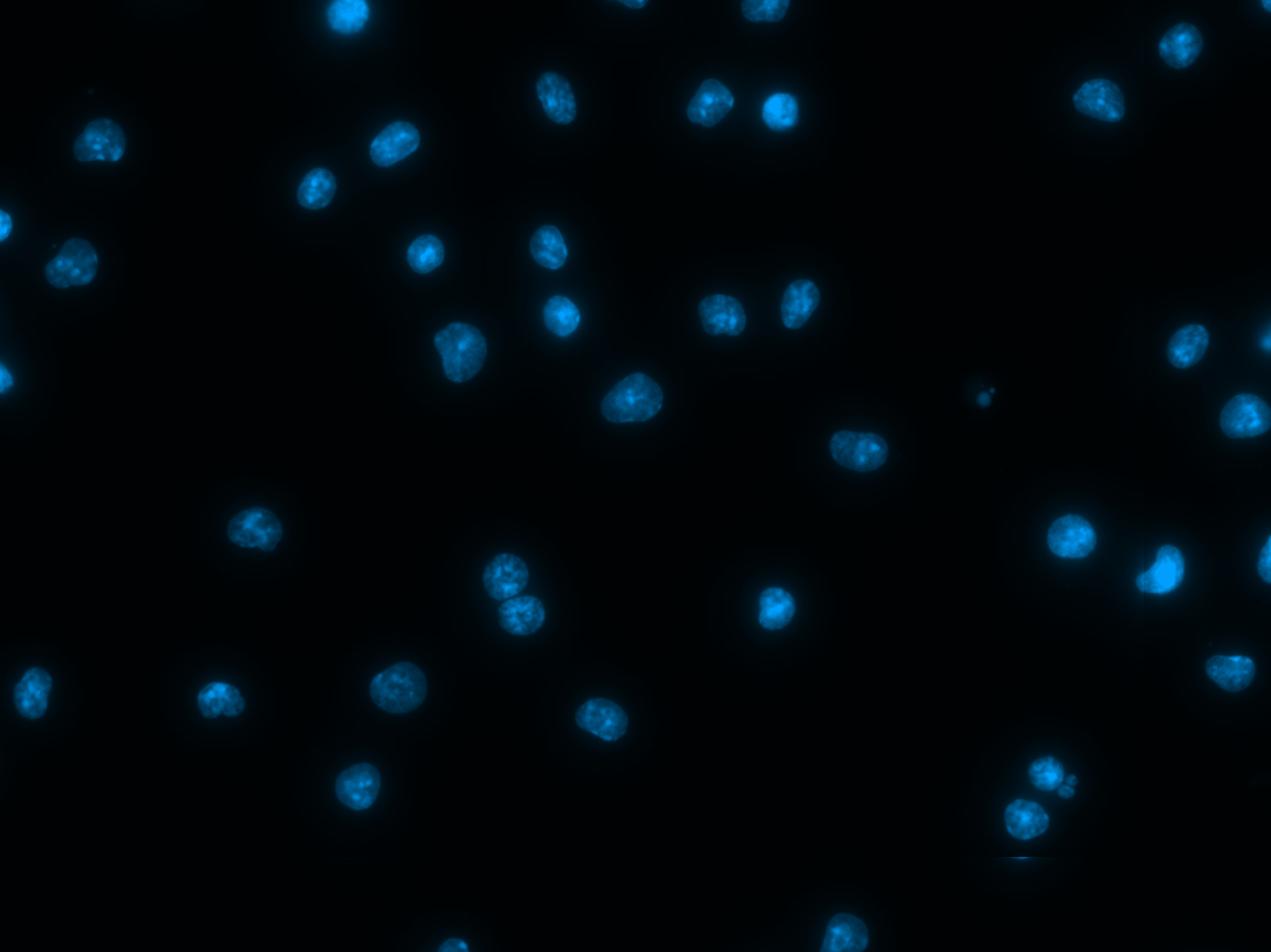

Supplement: Additional file 2 — The zip archive contains real images showing B cell nuclei and cytoskeleton. (ZIP 12390 kb) [file 12859_2017_1591_MOESM2_ESM.zip › B cells/nuclei0004.png]

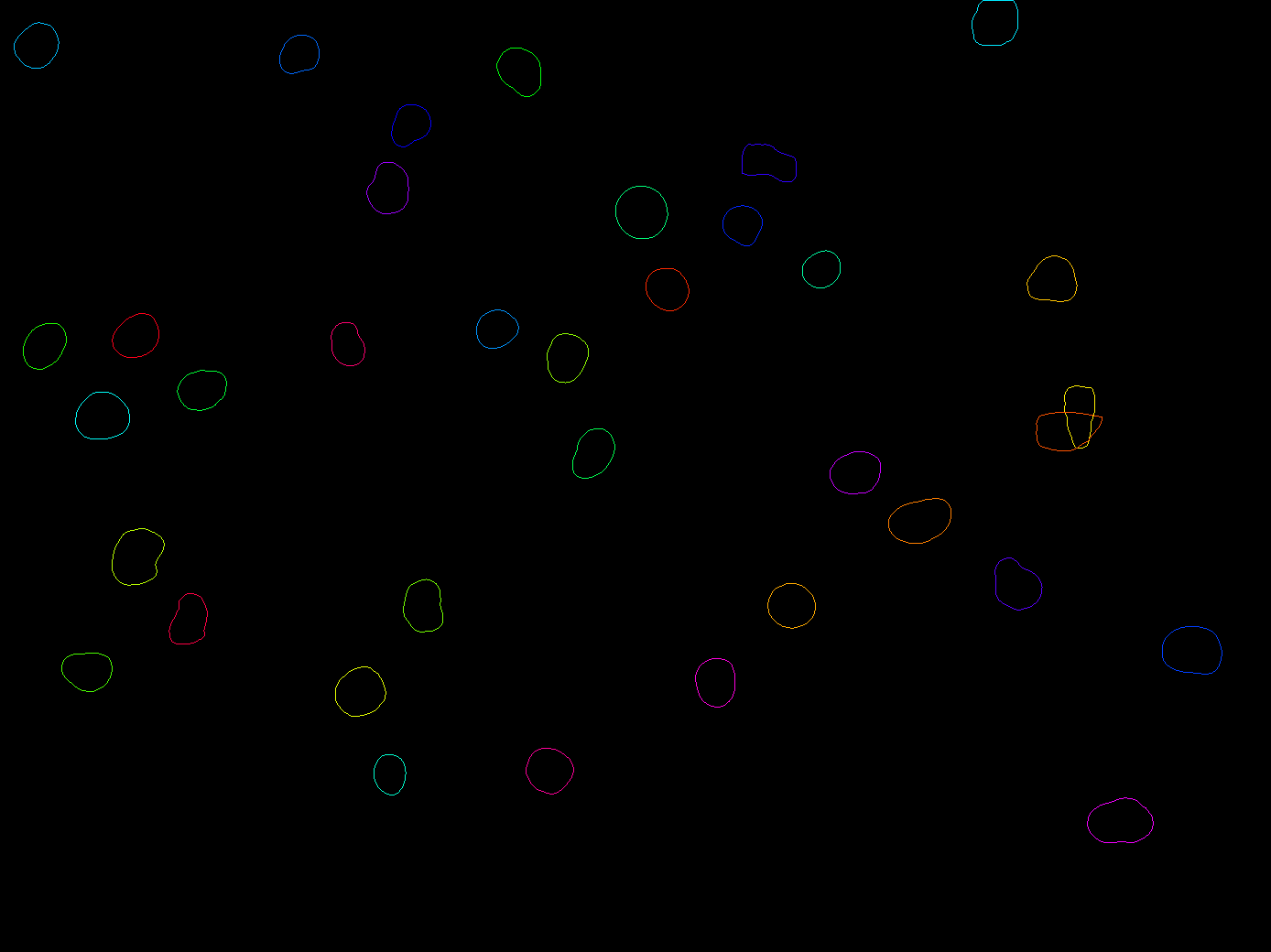

Supplement: Additional file 2 — The zip archive contains real images showing B cell nuclei and cytoskeleton. (ZIP 12390 kb) [file 12859_2017_1591_MOESM2_ESM.zip › B cells/nuclei0005 gt.png]

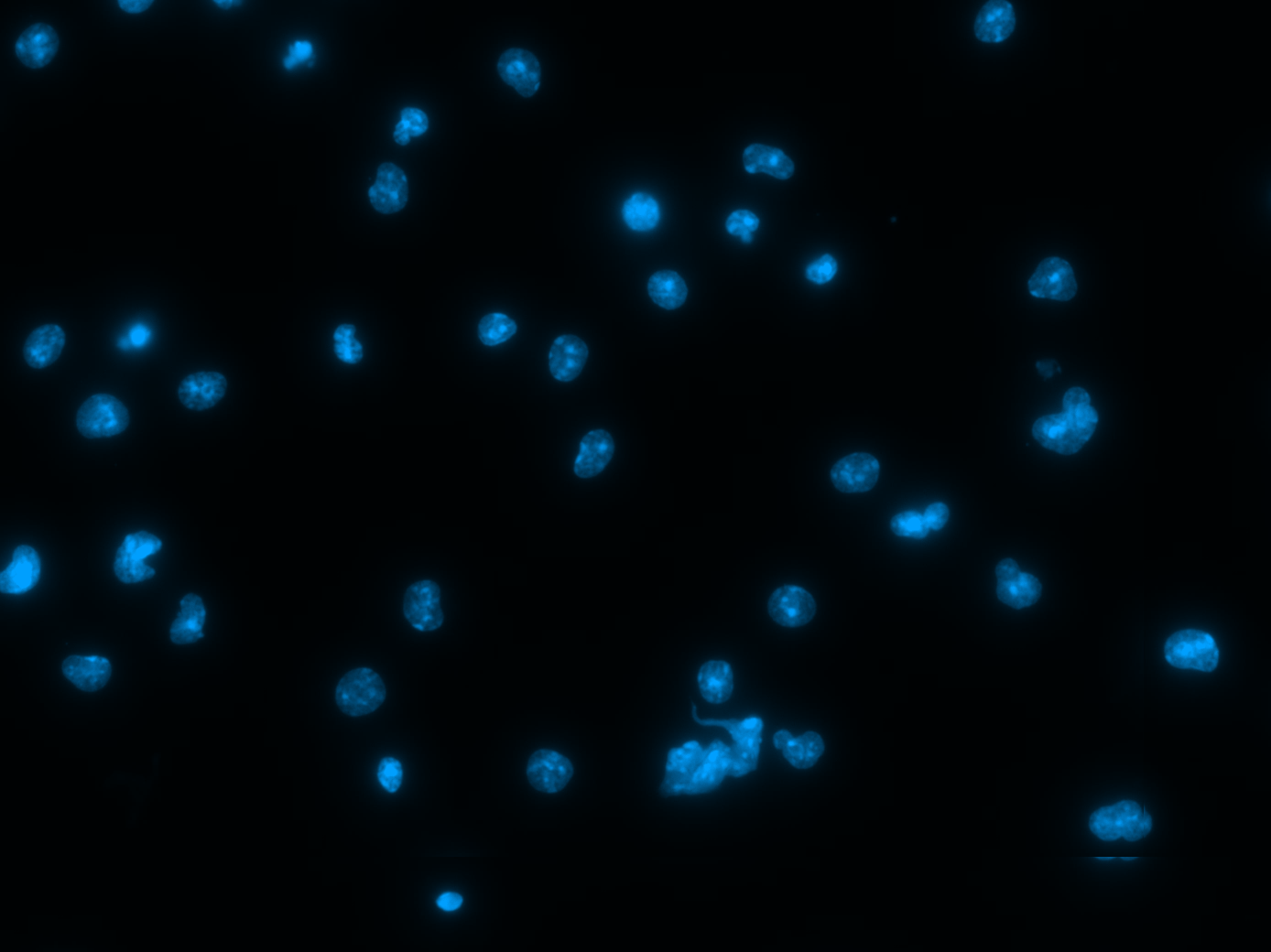

Supplement: Additional file 2 — The zip archive contains real images showing B cell nuclei and cytoskeleton. (ZIP 12390 kb) [file 12859_2017_1591_MOESM2_ESM.zip › B cells/nuclei0005.png]

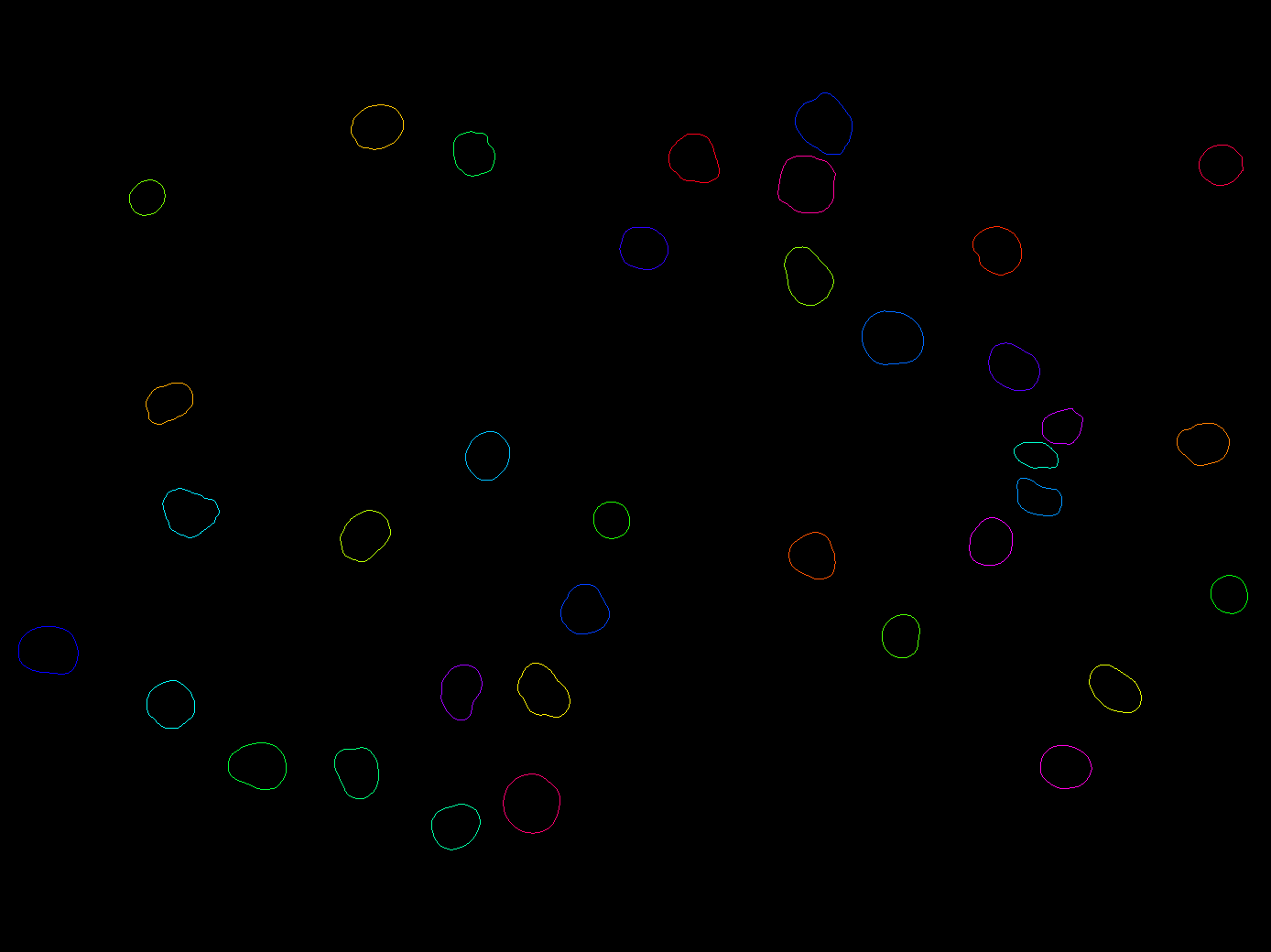

Supplement: Additional file 2 — The zip archive contains real images showing B cell nuclei and cytoskeleton. (ZIP 12390 kb) [file 12859_2017_1591_MOESM2_ESM.zip › B cells/nuclei0006 gt.png]

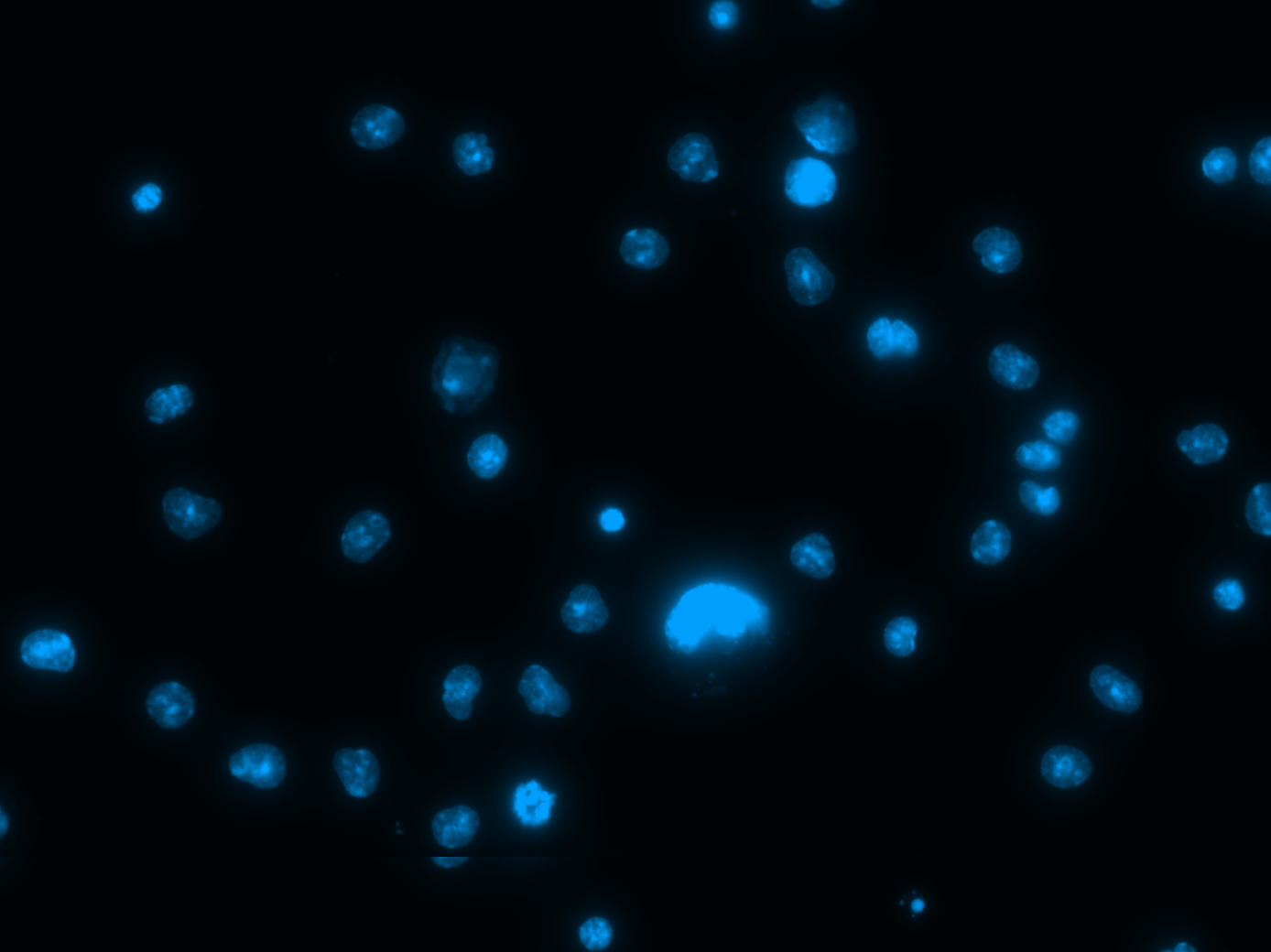

Supplement: Additional file 2 — The zip archive contains real images showing B cell nuclei and cytoskeleton. (ZIP 12390 kb) [file 12859_2017_1591_MOESM2_ESM.zip › B cells/nuclei0006.png]

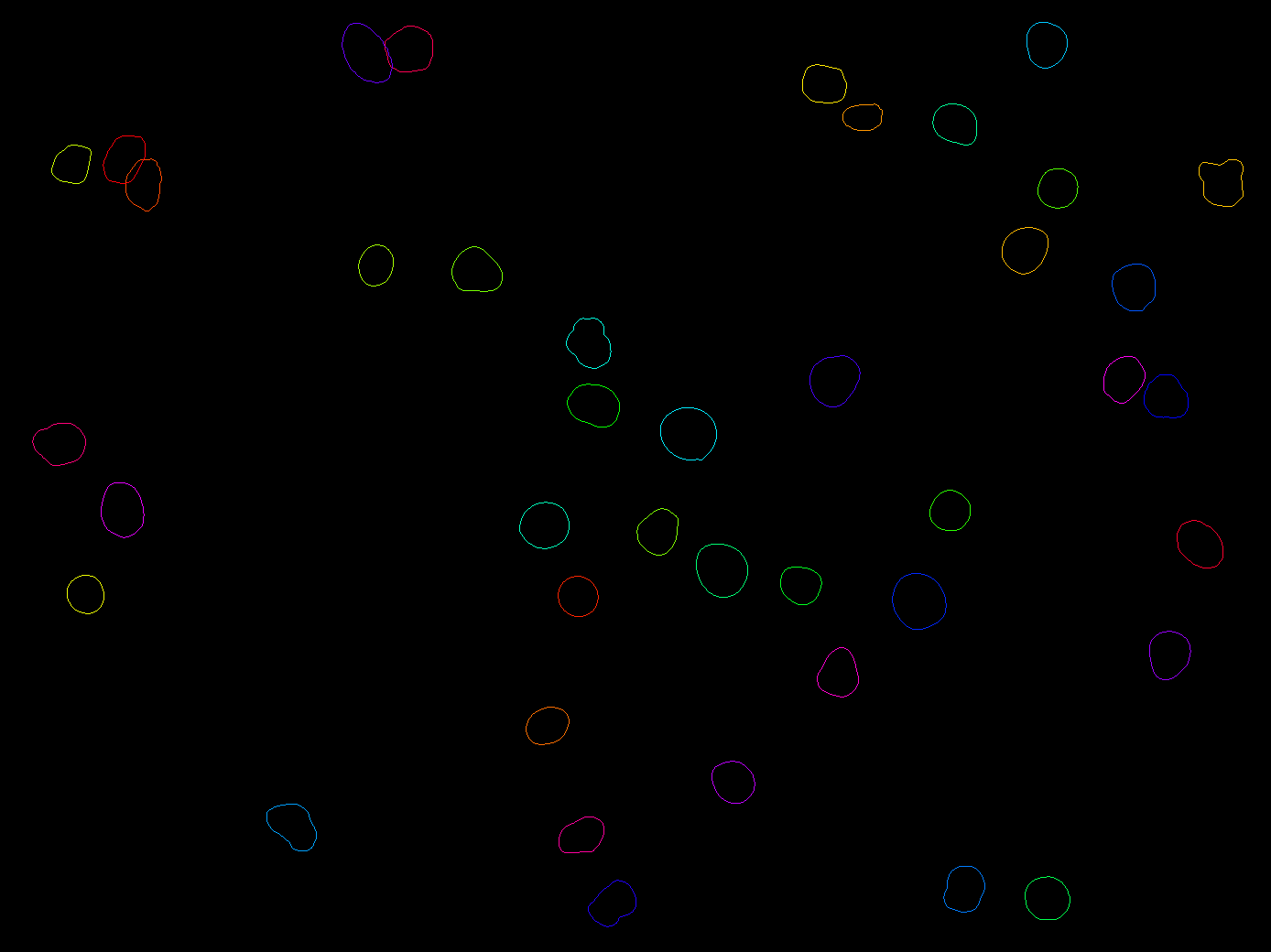

Supplement: Additional file 2 — The zip archive contains real images showing B cell nuclei and cytoskeleton. (ZIP 12390 kb) [file 12859_2017_1591_MOESM2_ESM.zip › B cells/nuclei0007 gt.png]

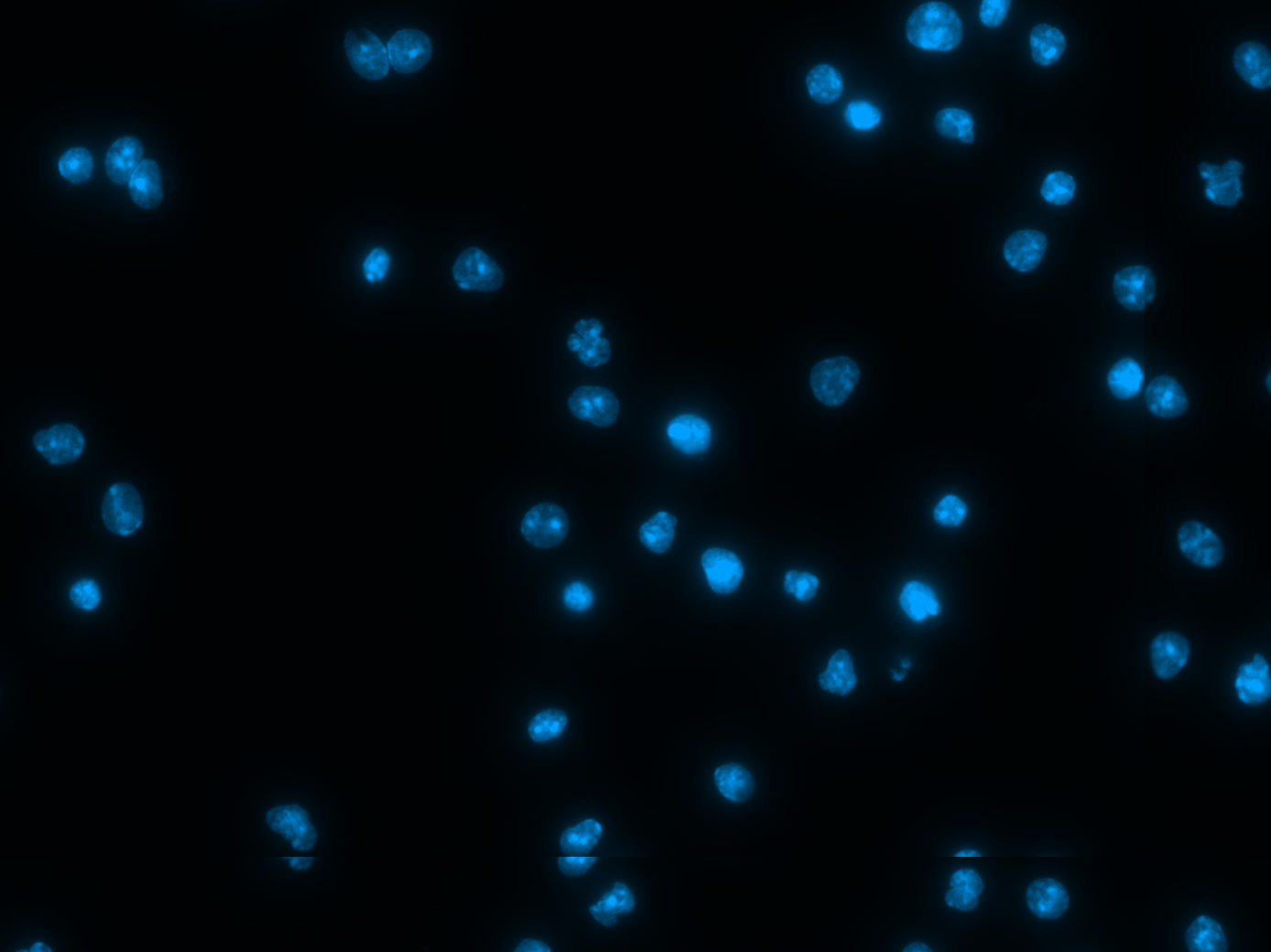

Supplement: Additional file 2 — The zip archive contains real images showing B cell nuclei and cytoskeleton. (ZIP 12390 kb) [file 12859_2017_1591_MOESM2_ESM.zip › B cells/nuclei0007.png]

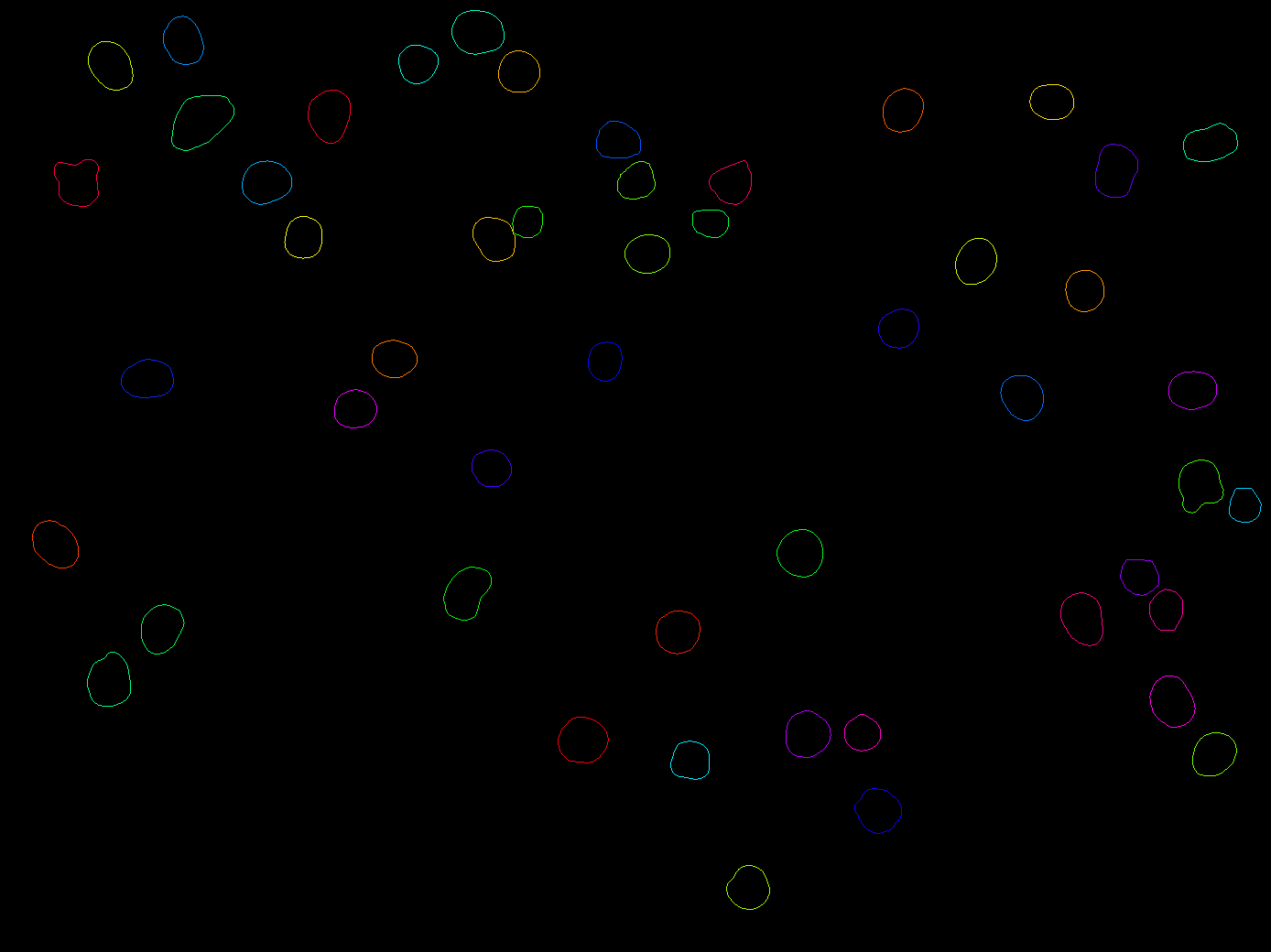

Supplement: Additional file 2 — The zip archive contains real images showing B cell nuclei and cytoskeleton. (ZIP 12390 kb) [file 12859_2017_1591_MOESM2_ESM.zip › B cells/nuclei0008 gt.png]

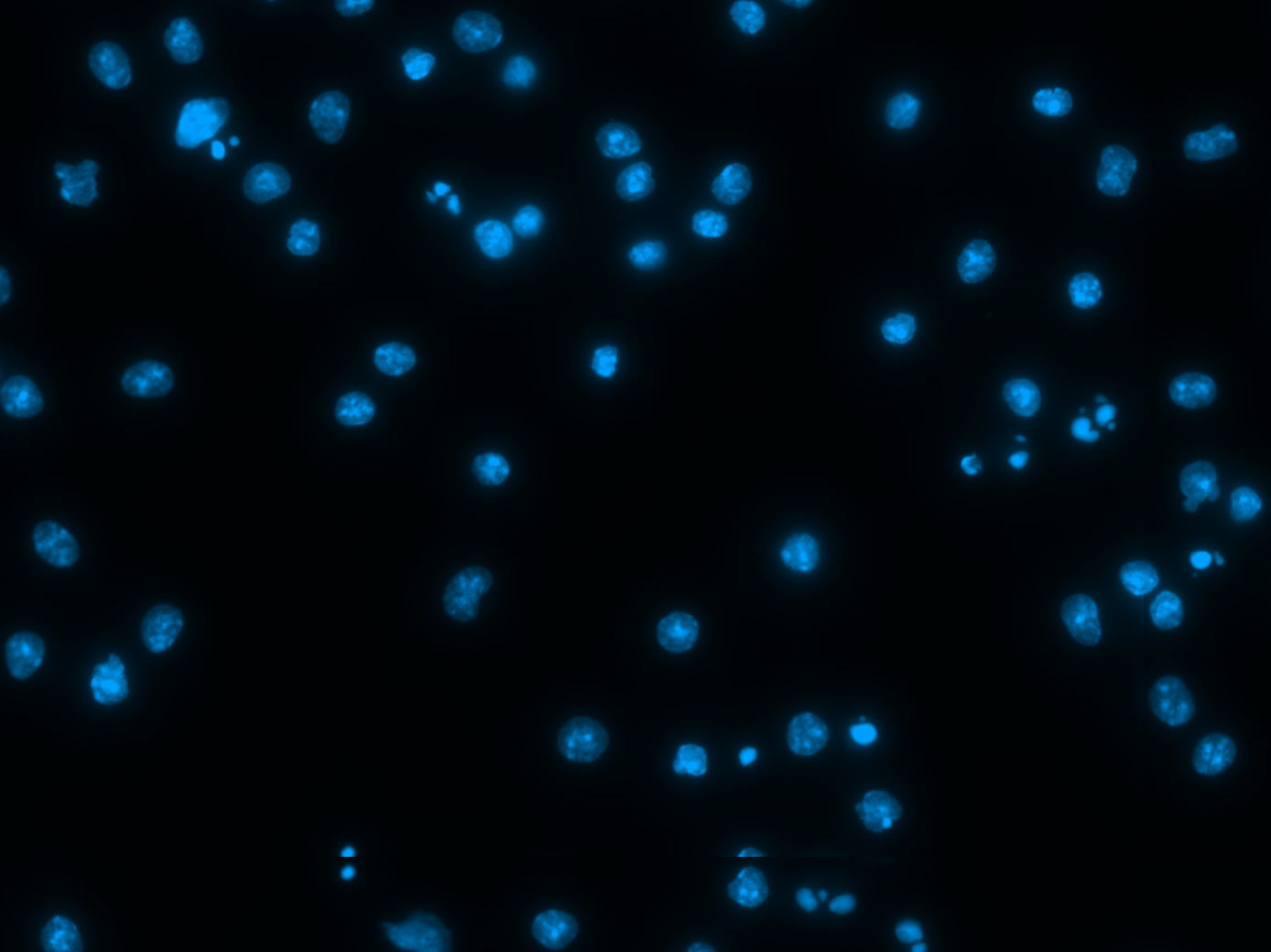

Supplement: Additional file 2 — The zip archive contains real images showing B cell nuclei and cytoskeleton. (ZIP 12390 kb) [file 12859_2017_1591_MOESM2_ESM.zip › B cells/nuclei0008.png]

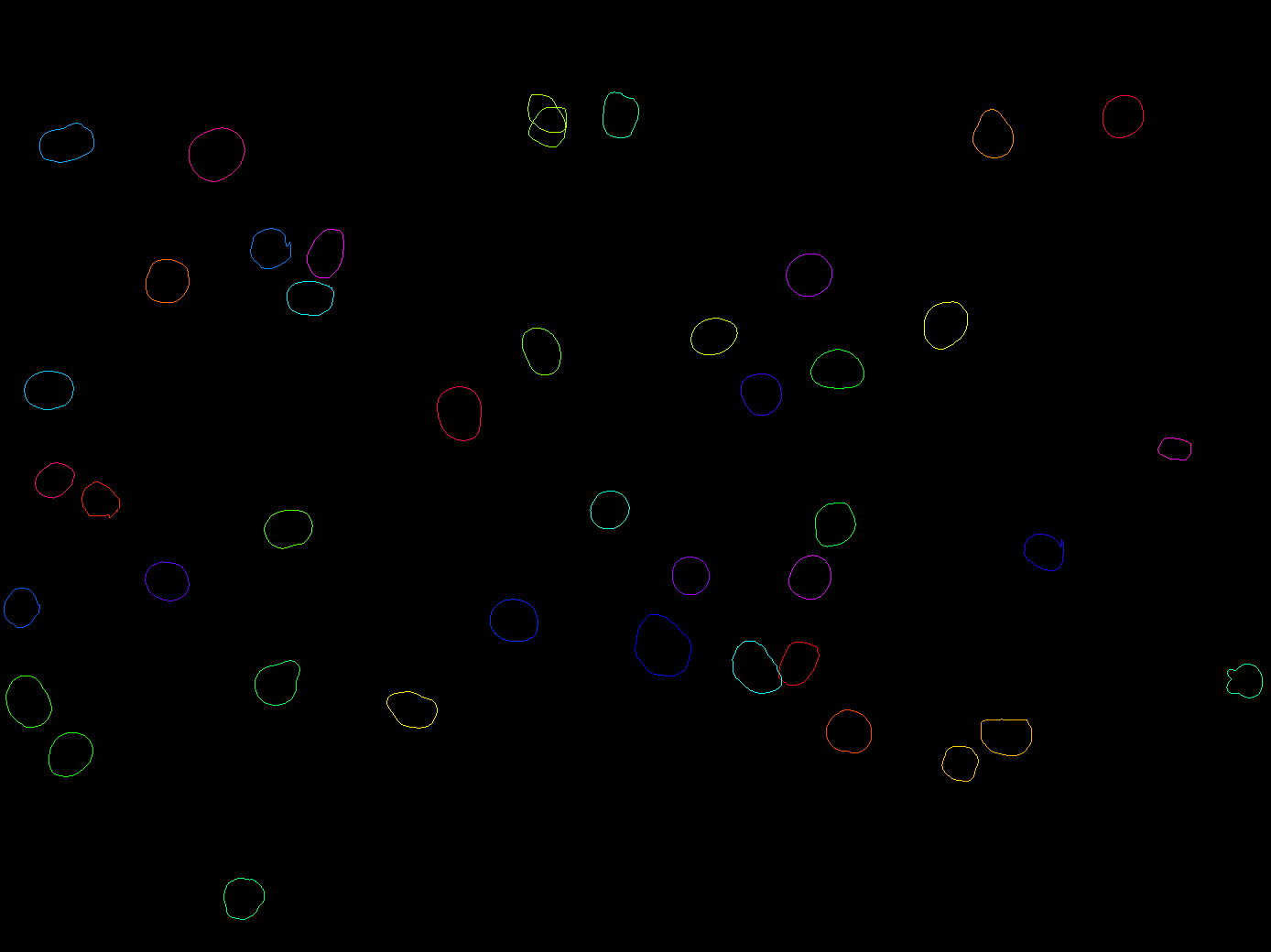

Supplement: Additional file 2 — The zip archive contains real images showing B cell nuclei and cytoskeleton. (ZIP 12390 kb) [file 12859_2017_1591_MOESM2_ESM.zip › B cells/nuclei0009 gt.png]

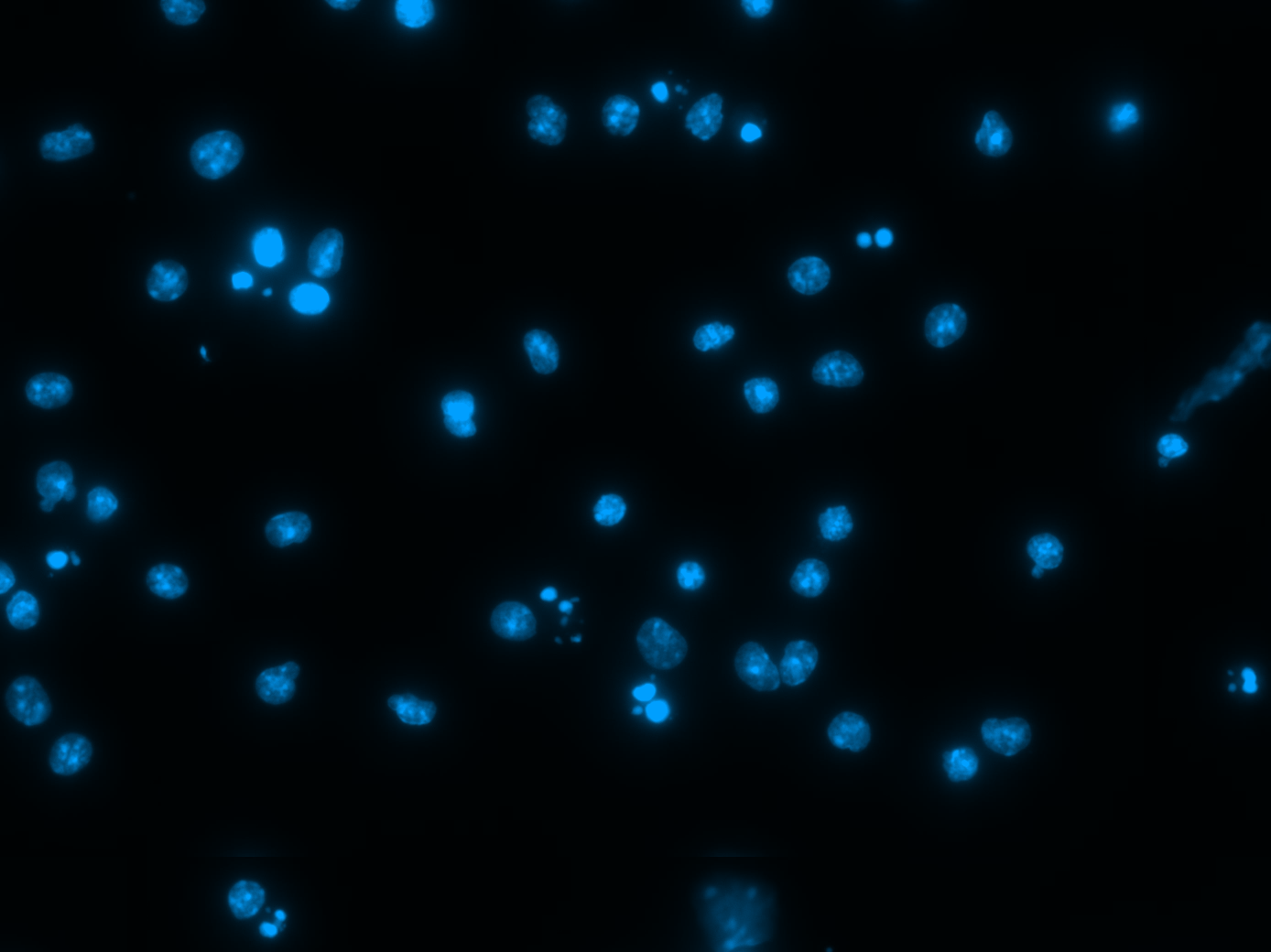

Supplement: Additional file 2 — The zip archive contains real images showing B cell nuclei and cytoskeleton. (ZIP 12390 kb) [file 12859_2017_1591_MOESM2_ESM.zip › B cells/nuclei0009.png]

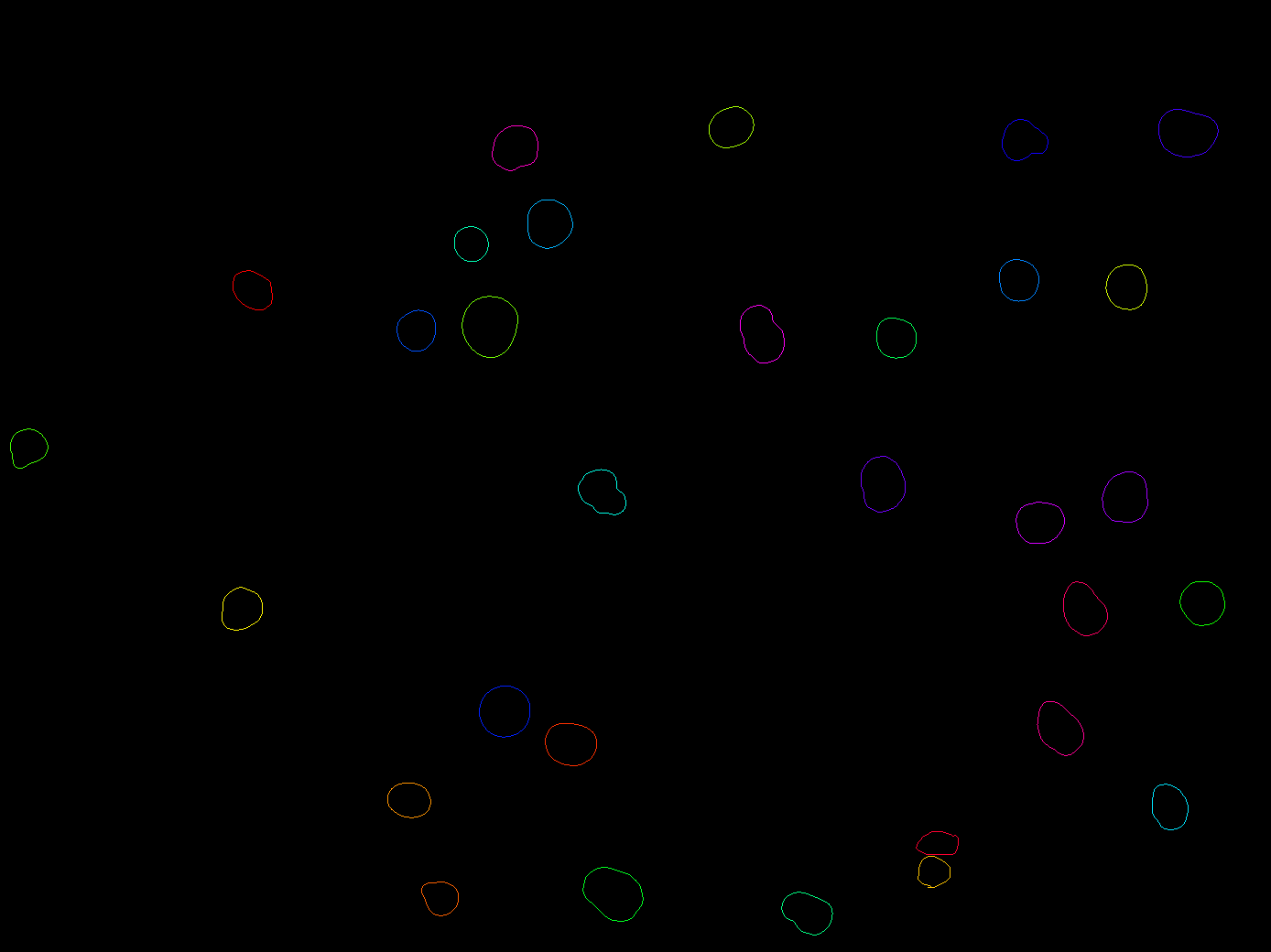

Supplement: Additional file 2 — The zip archive contains real images showing B cell nuclei and cytoskeleton. (ZIP 12390 kb) [file 12859_2017_1591_MOESM2_ESM.zip › B cells/nuclei0010 gt.png]

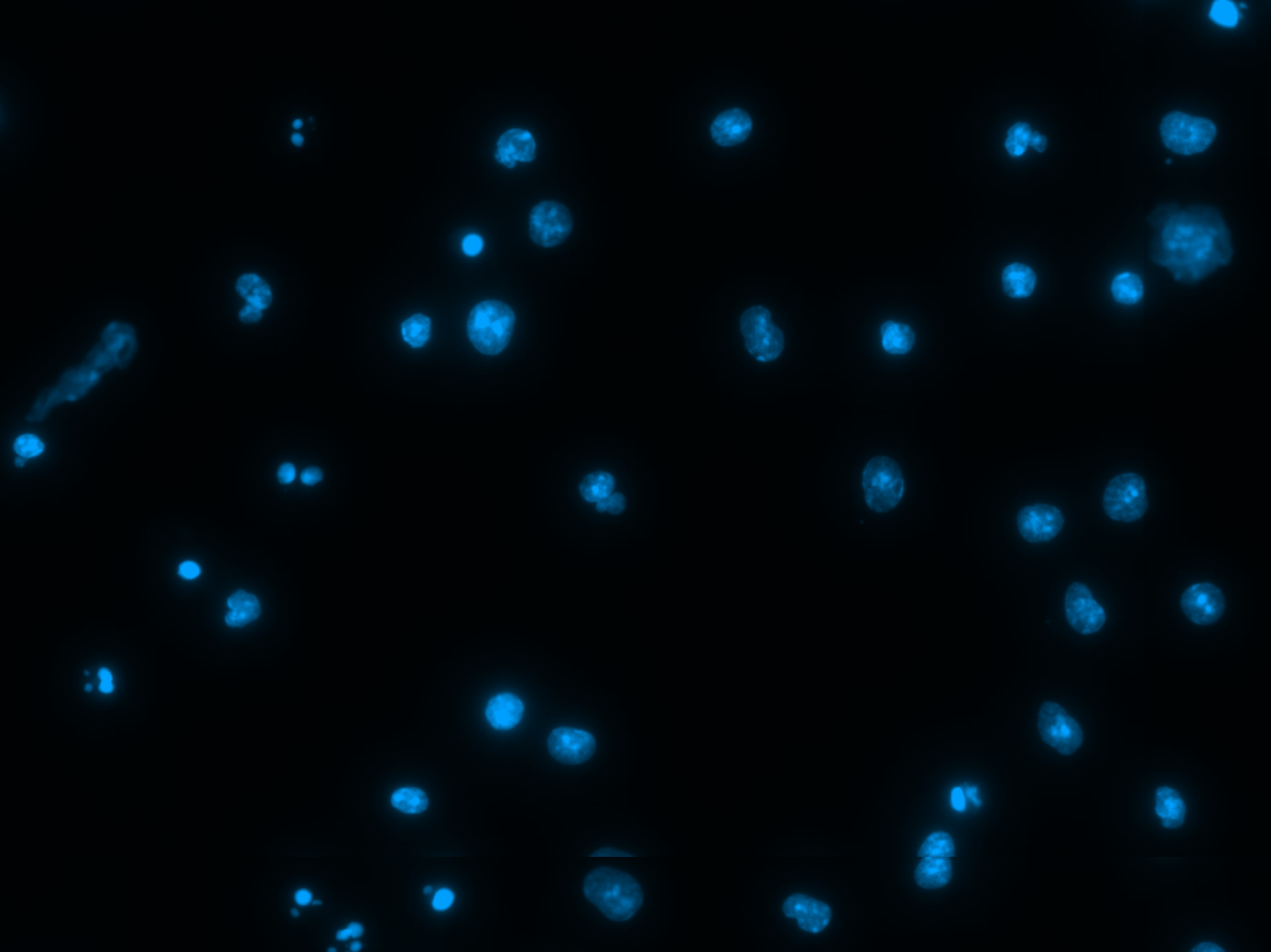

Supplement: Additional file 2 — The zip archive contains real images showing B cell nuclei and cytoskeleton. (ZIP 12390 kb) [file 12859_2017_1591_MOESM2_ESM.zip › B cells/nuclei0010.png]

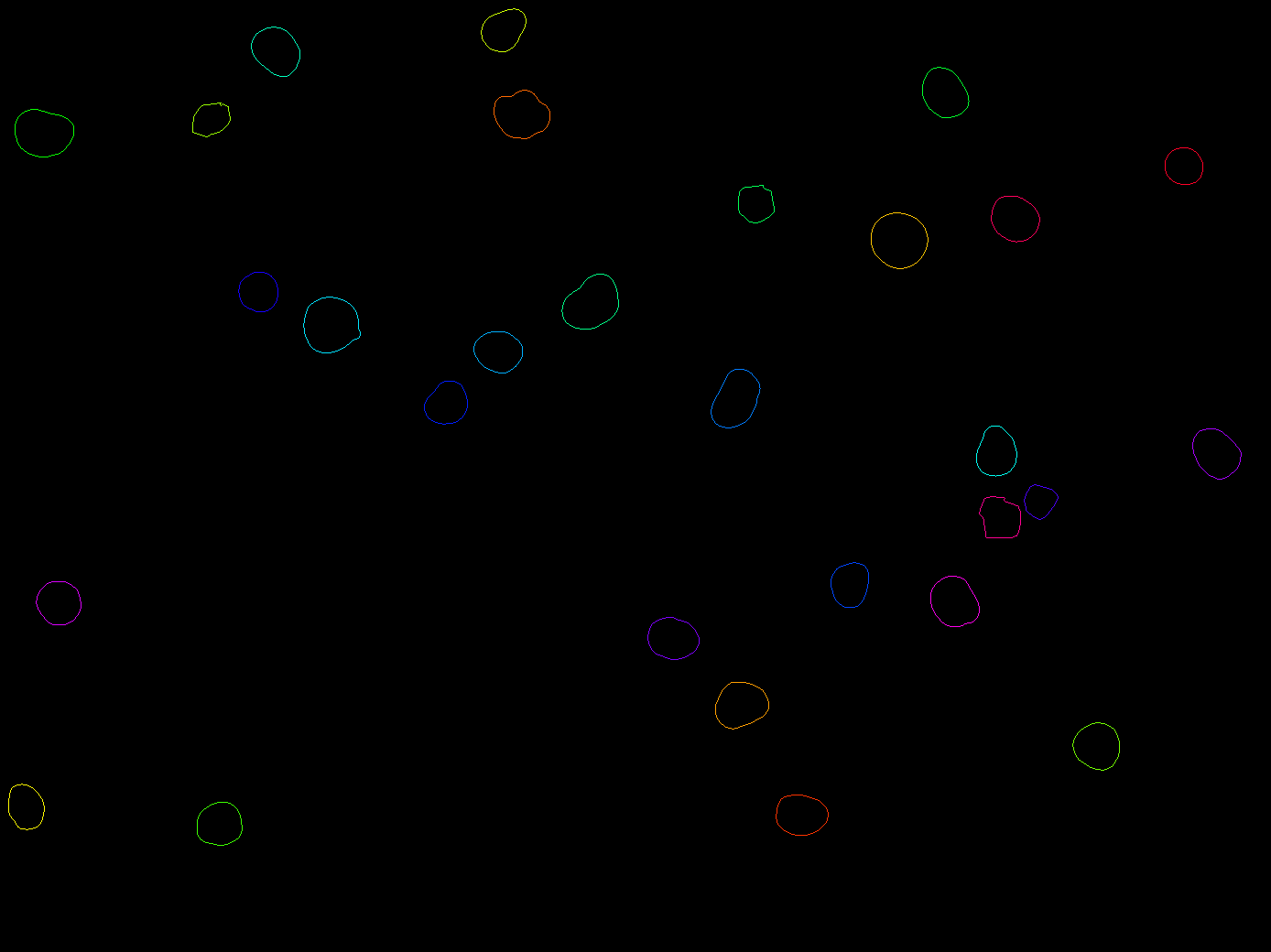

Supplement: Additional file 2 — The zip archive contains real images showing B cell nuclei and cytoskeleton. (ZIP 12390 kb) [file 12859_2017_1591_MOESM2_ESM.zip › B cells/nuclei0011 gt.png]

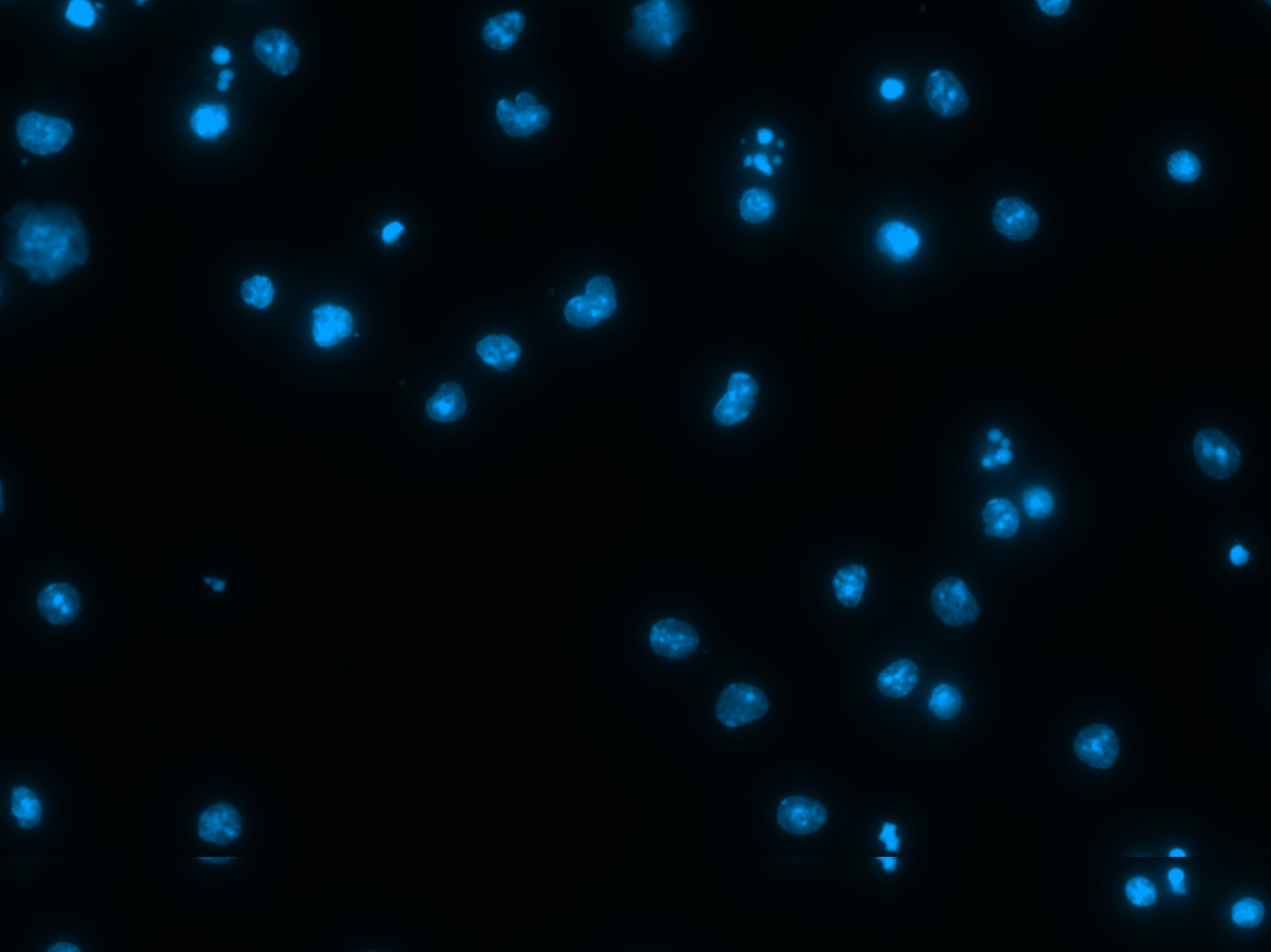

Supplement: Additional file 2 — The zip archive contains real images showing B cell nuclei and cytoskeleton. (ZIP 12390 kb) [file 12859_2017_1591_MOESM2_ESM.zip › B cells/nuclei0011.png]

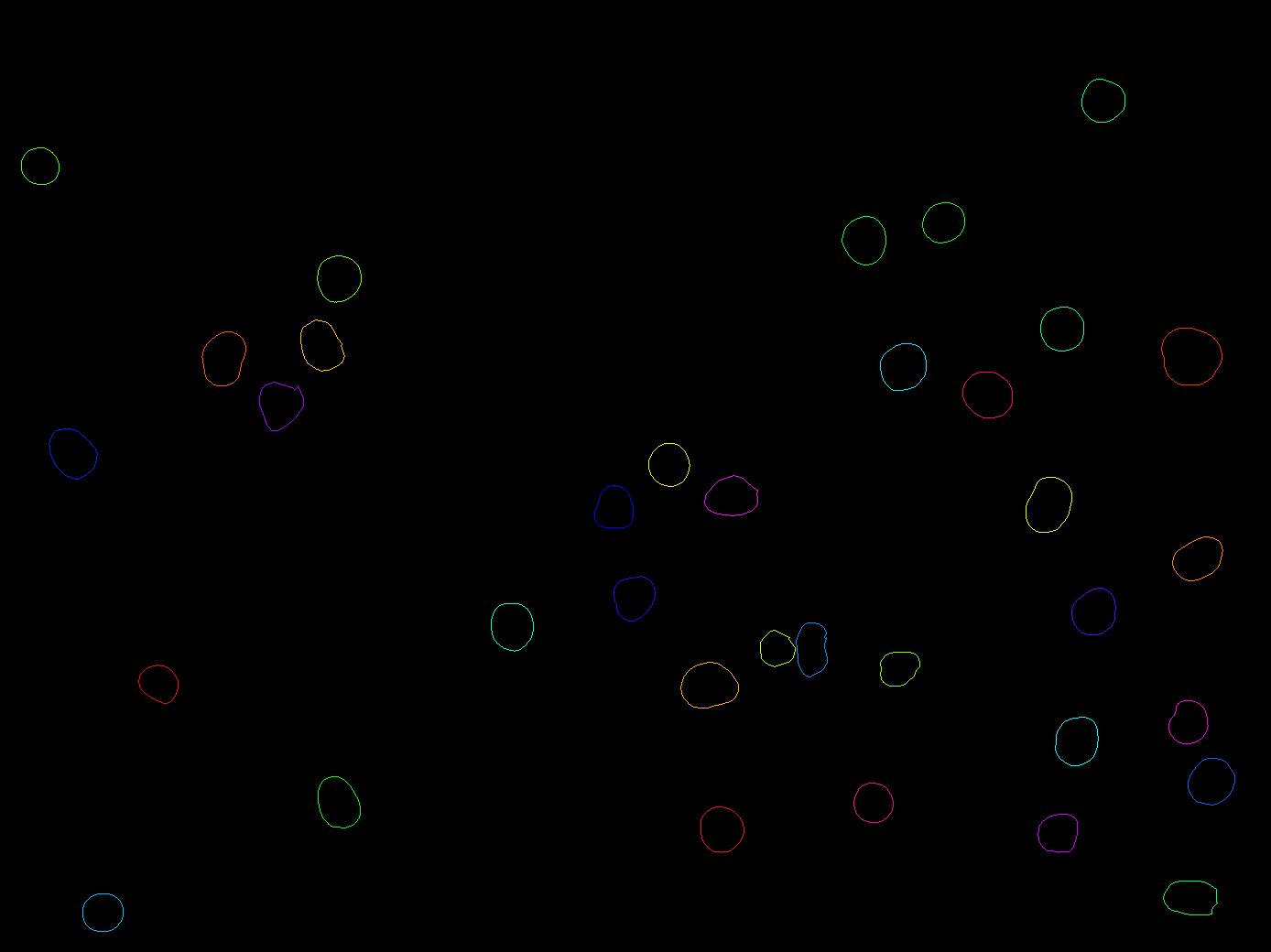

Supplement: Additional file 2 — The zip archive contains real images showing B cell nuclei and cytoskeleton. (ZIP 12390 kb) [file 12859_2017_1591_MOESM2_ESM.zip › B cells/nuclei0012 gt.png]

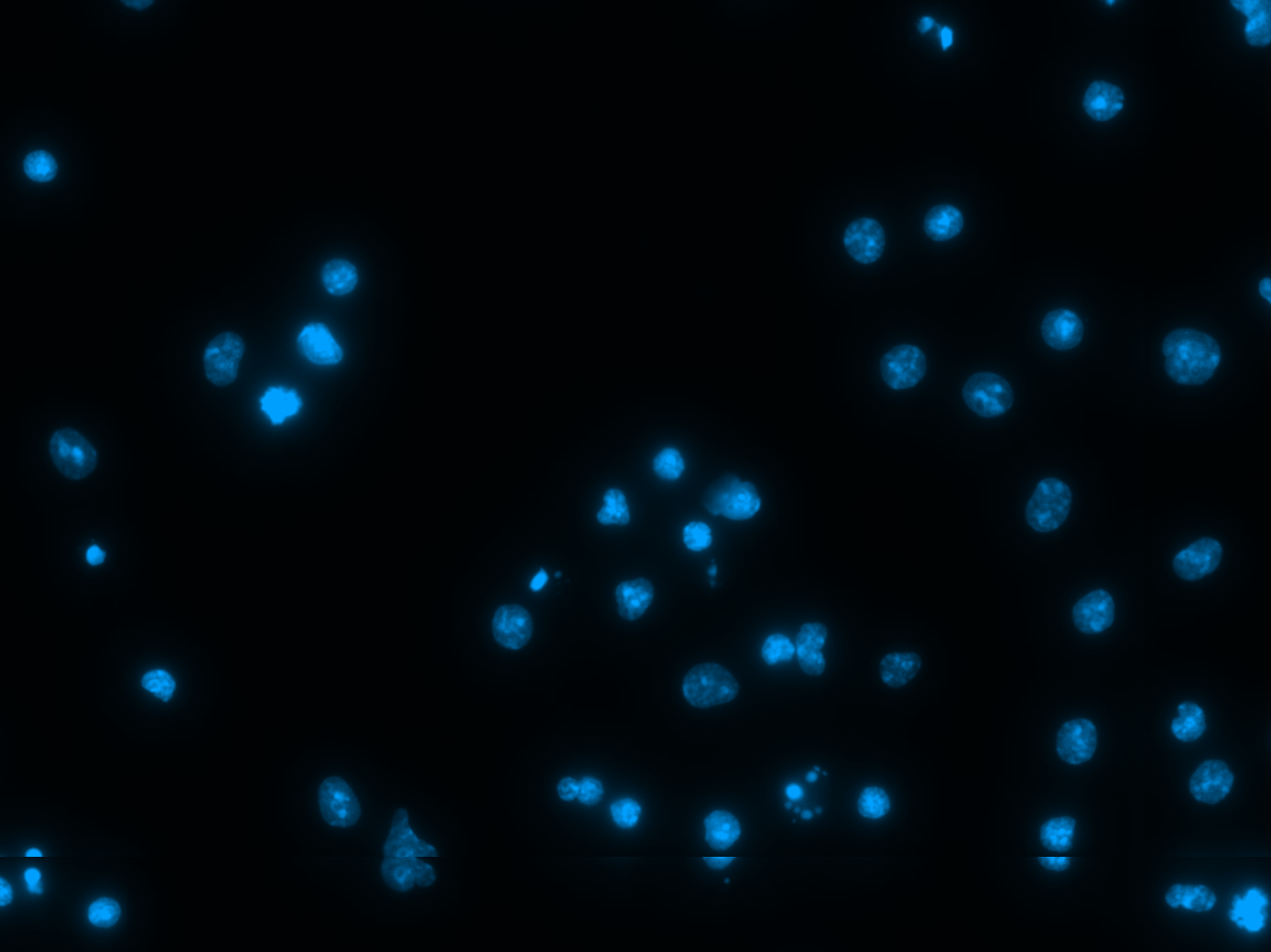

Supplement: Additional file 2 — The zip archive contains real images showing B cell nuclei and cytoskeleton. (ZIP 12390 kb) [file 12859_2017_1591_MOESM2_ESM.zip › B cells/nuclei0012.png]

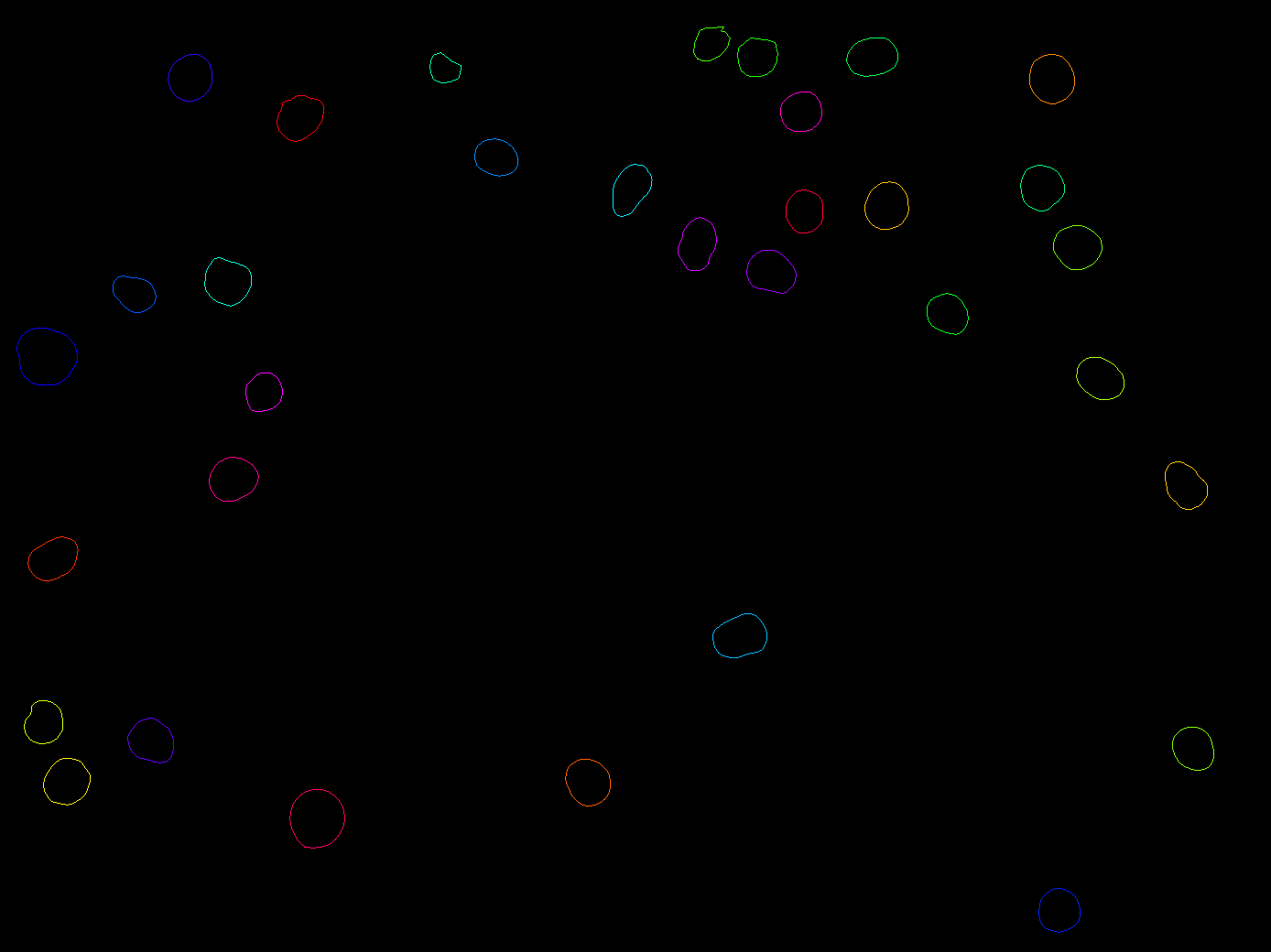

Supplement: Additional file 2 — The zip archive contains real images showing B cell nuclei and cytoskeleton. (ZIP 12390 kb) [file 12859_2017_1591_MOESM2_ESM.zip › B cells/nuclei0013 gt.png]

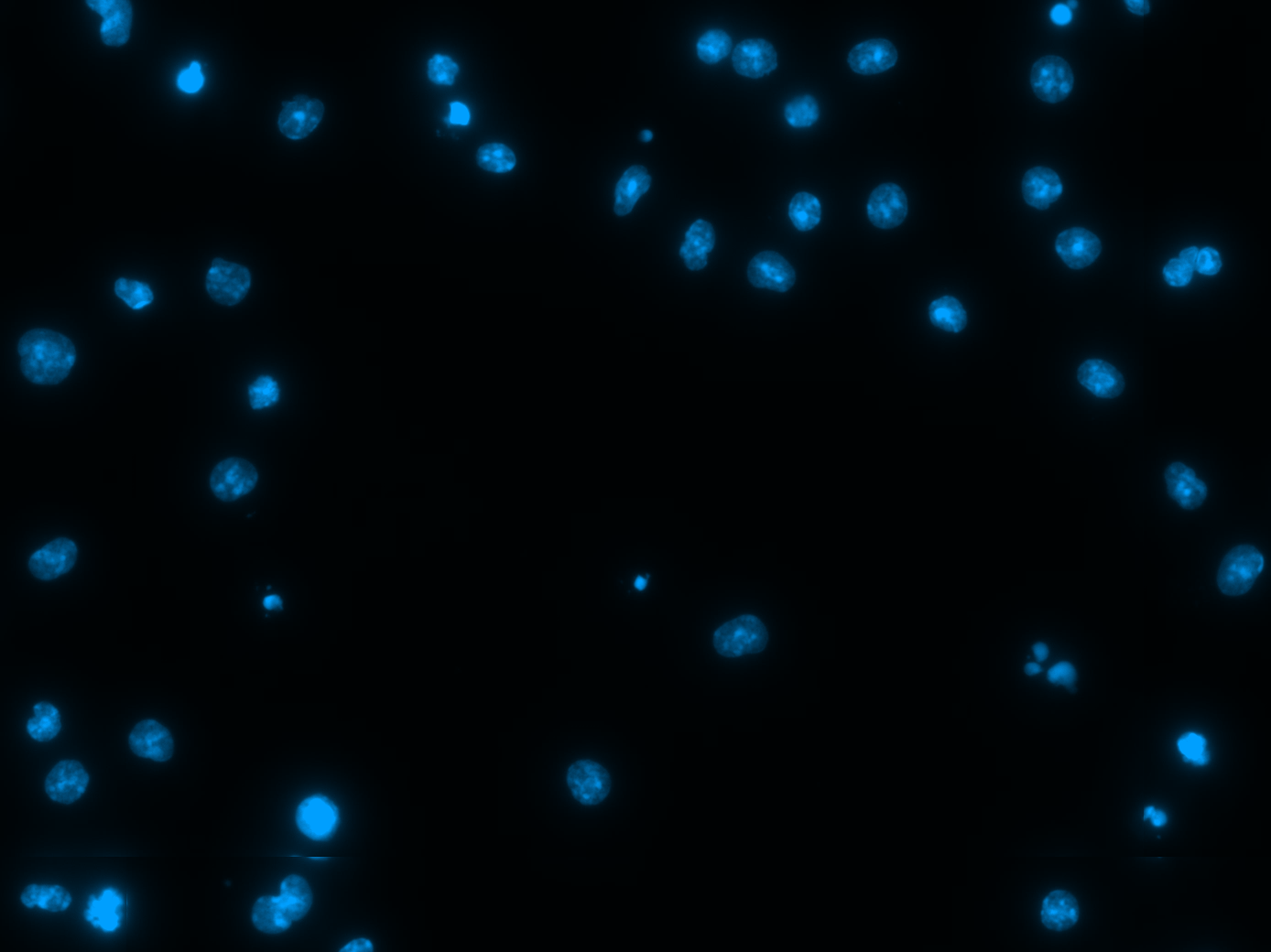

Supplement: Additional file 2 — The zip archive contains real images showing B cell nuclei and cytoskeleton. (ZIP 12390 kb) [file 12859_2017_1591_MOESM2_ESM.zip › B cells/nuclei0013.png]

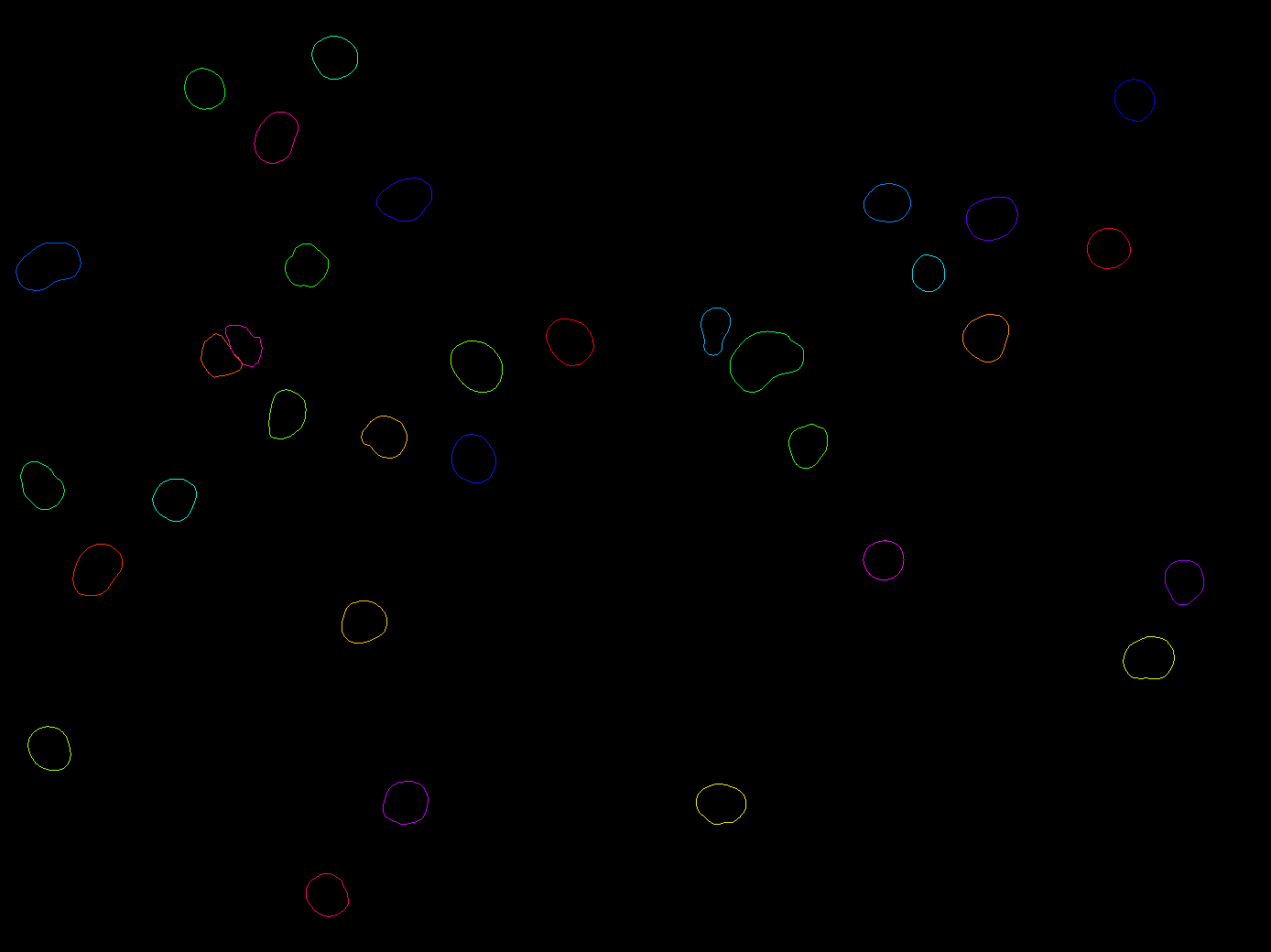

Supplement: Additional file 2 — The zip archive contains real images showing B cell nuclei and cytoskeleton. (ZIP 12390 kb) [file 12859_2017_1591_MOESM2_ESM.zip › B cells/nuclei0014 gt.png]

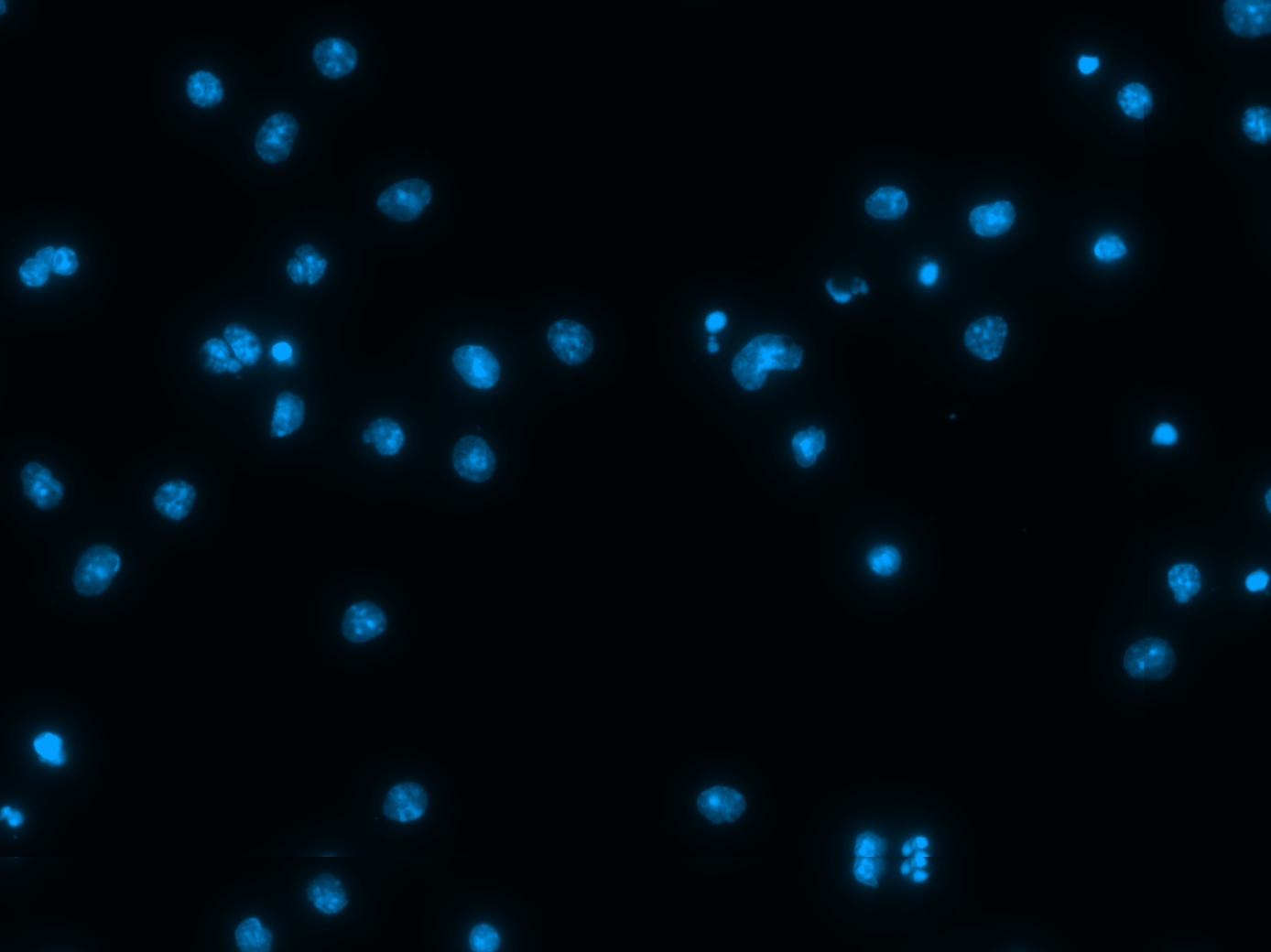

Supplement: Additional file 2 — The zip archive contains real images showing B cell nuclei and cytoskeleton. (ZIP 12390 kb) [file 12859_2017_1591_MOESM2_ESM.zip › B cells/nuclei0014.png]

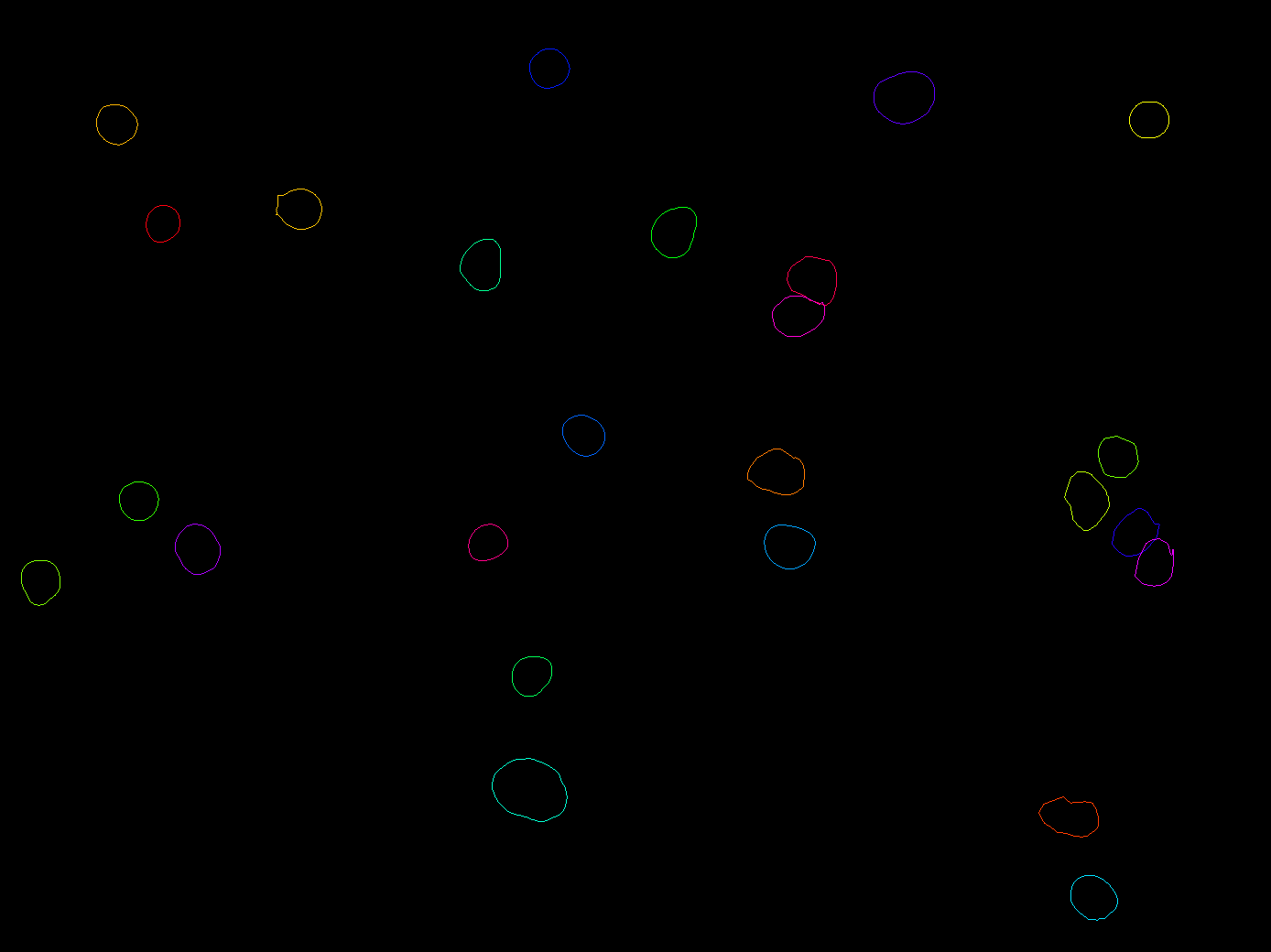

Supplement: Additional file 2 — The zip archive contains real images showing B cell nuclei and cytoskeleton. (ZIP 12390 kb) [file 12859_2017_1591_MOESM2_ESM.zip › B cells/nuclei0015 gt.png]

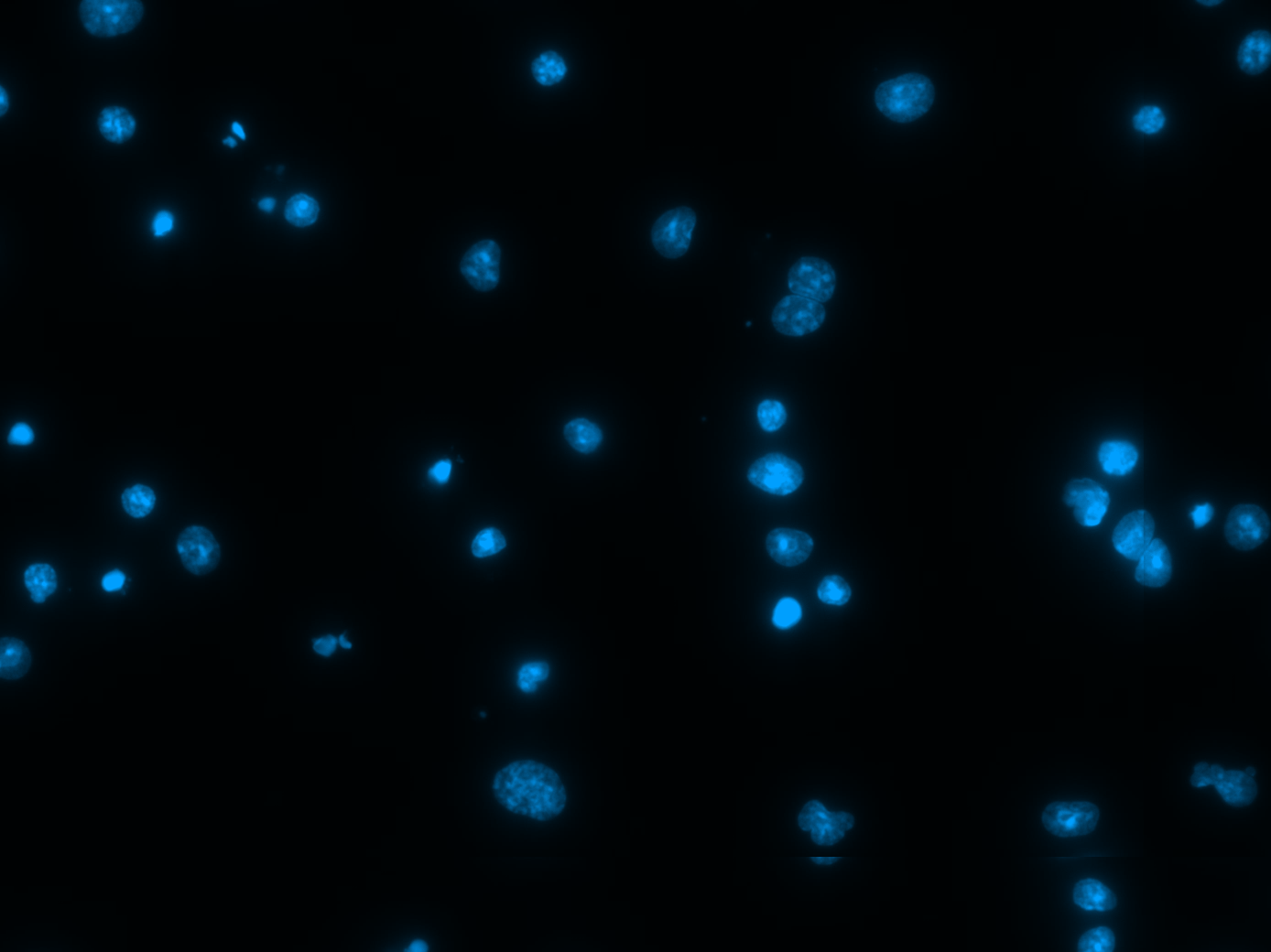

Supplement: Additional file 2 — The zip archive contains real images showing B cell nuclei and cytoskeleton. (ZIP 12390 kb) [file 12859_2017_1591_MOESM2_ESM.zip › B cells/nuclei0015.png]

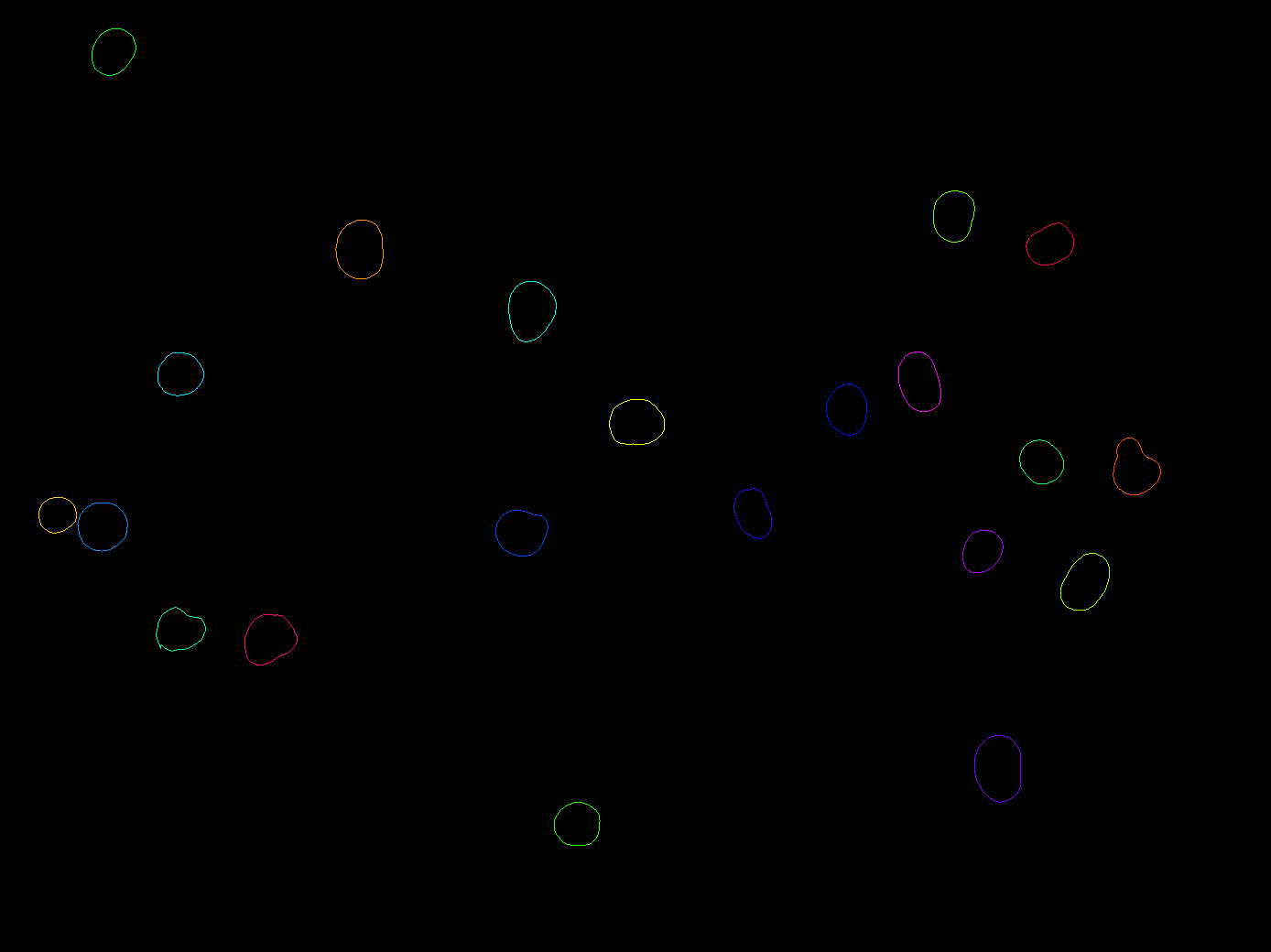

Supplement: Additional file 2 — The zip archive contains real images showing B cell nuclei and cytoskeleton. (ZIP 12390 kb) [file 12859_2017_1591_MOESM2_ESM.zip › B cells/nuclei0016 gt.png]

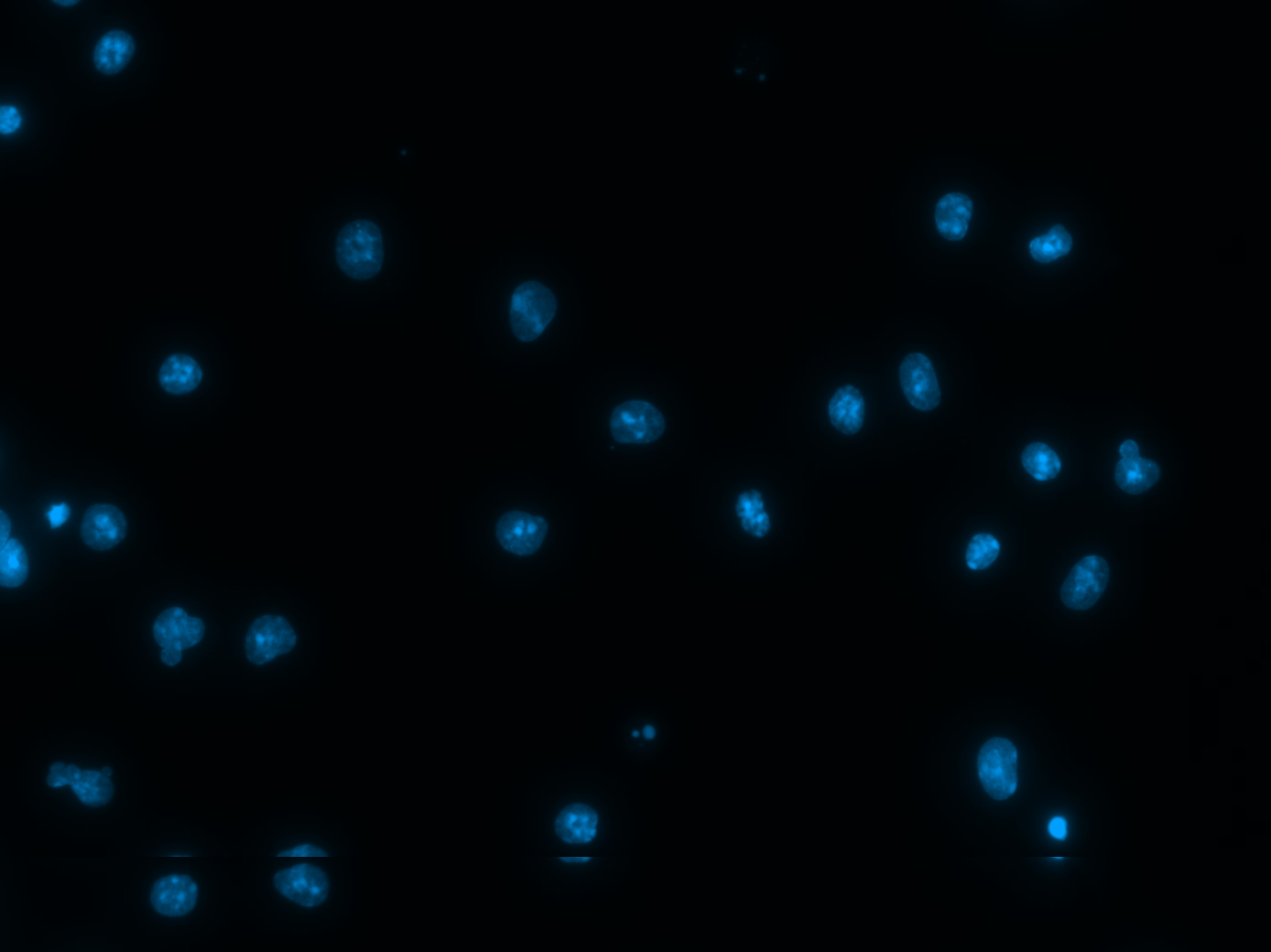

Supplement: Additional file 2 — The zip archive contains real images showing B cell nuclei and cytoskeleton. (ZIP 12390 kb) [file 12859_2017_1591_MOESM2_ESM.zip › B cells/nuclei0016.png]

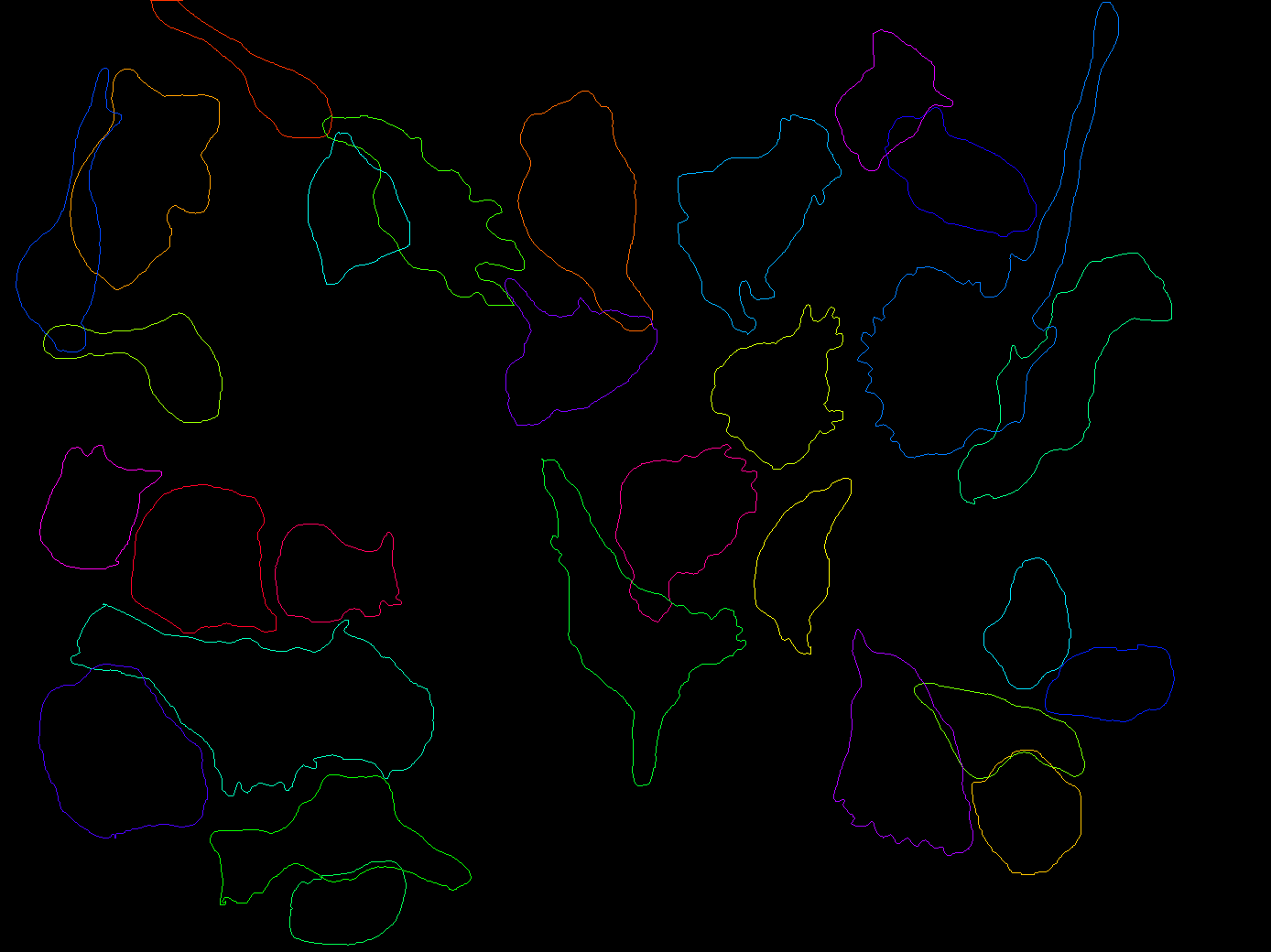

Supplement: Additional file 3 — The zip archive contains real images showing macrophages. (ZIP 28979 kb) [file 12859_2017_1591_MOESM3_ESM.zip › macrophages/jw-15min 1_c1 gt.png]

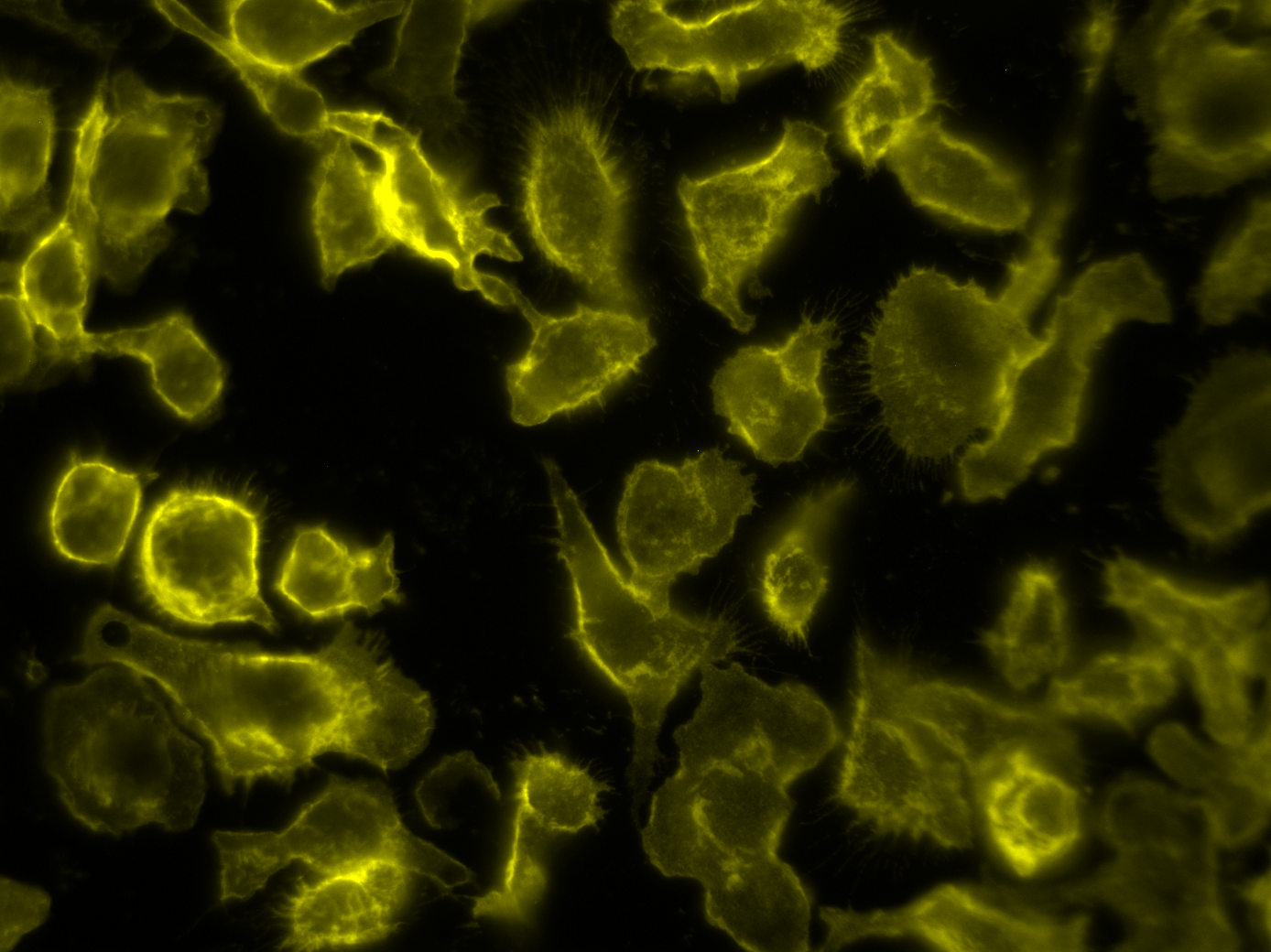

Supplement: Additional file 3 — The zip archive contains real images showing macrophages. (ZIP 28979 kb) [file 12859_2017_1591_MOESM3_ESM.zip › macrophages/jw-15min 1_c1.png]

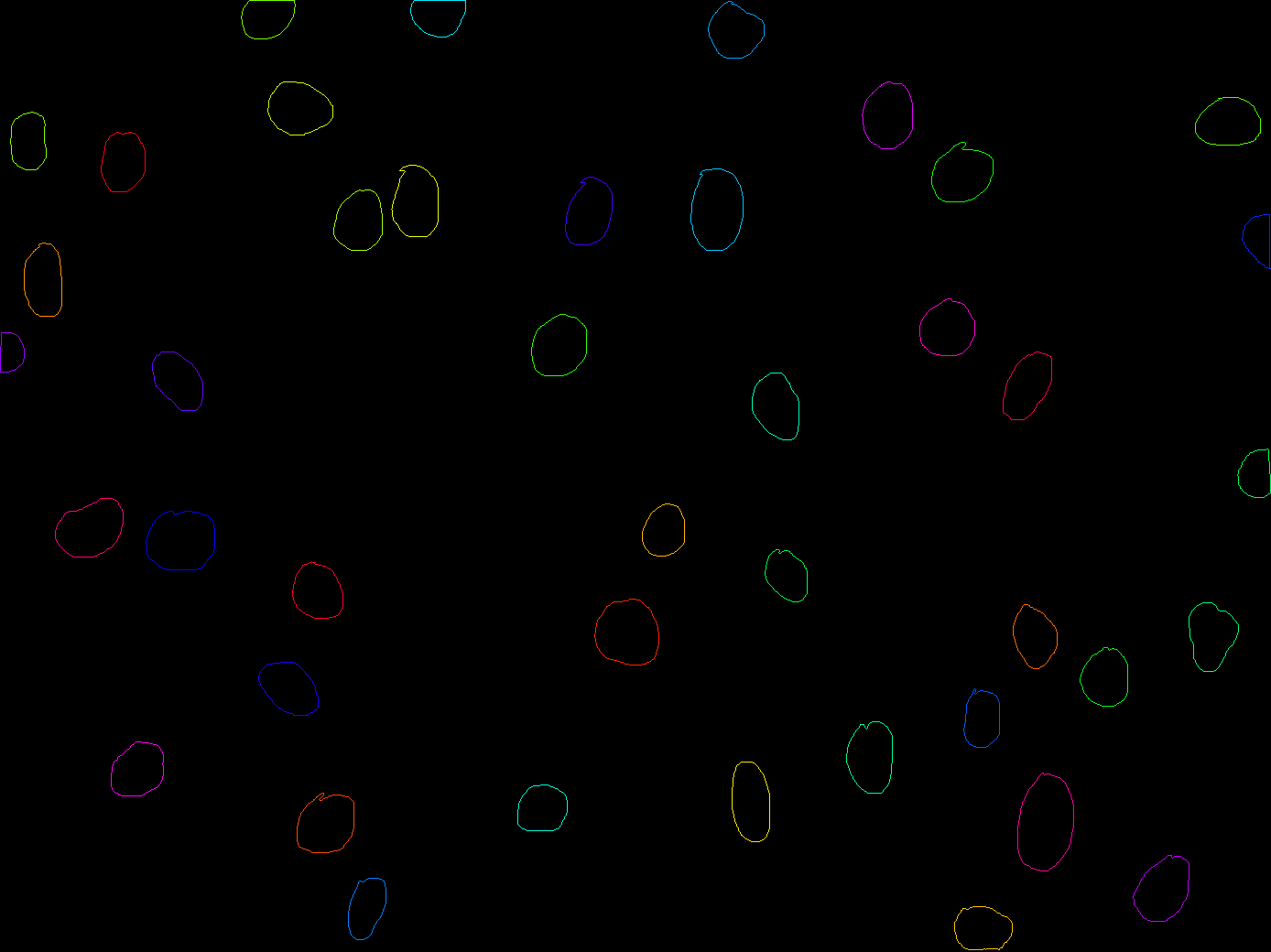

Supplement: Additional file 3 — The zip archive contains real images showing macrophages. (ZIP 28979 kb) [file 12859_2017_1591_MOESM3_ESM.zip › macrophages/jw-15min 1_c5 gt.png]

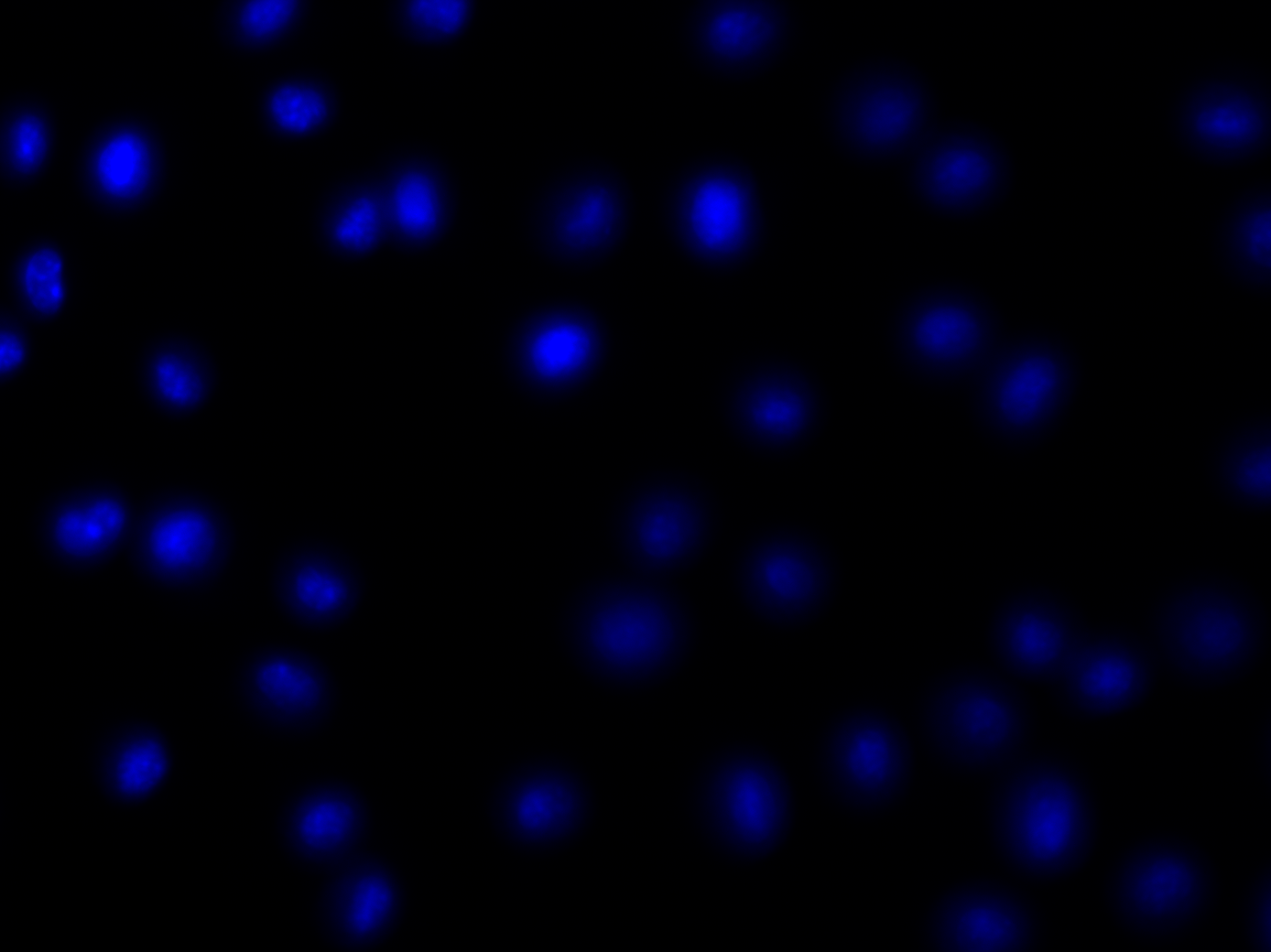

Supplement: Additional file 3 — The zip archive contains real images showing macrophages. (ZIP 28979 kb) [file 12859_2017_1591_MOESM3_ESM.zip › macrophages/jw-15min 1_c5.png]

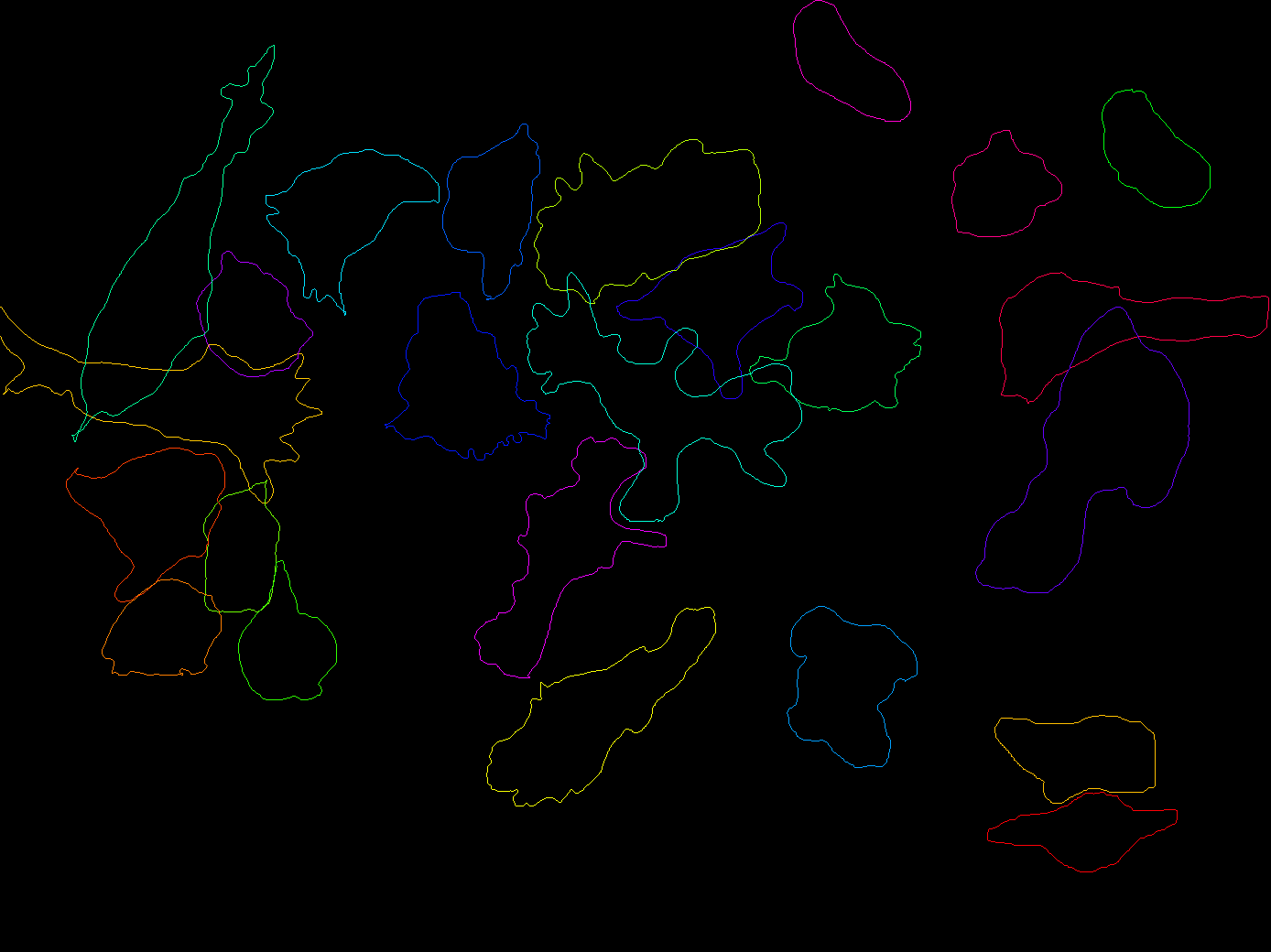

Supplement: Additional file 3 — The zip archive contains real images showing macrophages. (ZIP 28979 kb) [file 12859_2017_1591_MOESM3_ESM.zip › macrophages/jw-15min 2_c1 gt.png]

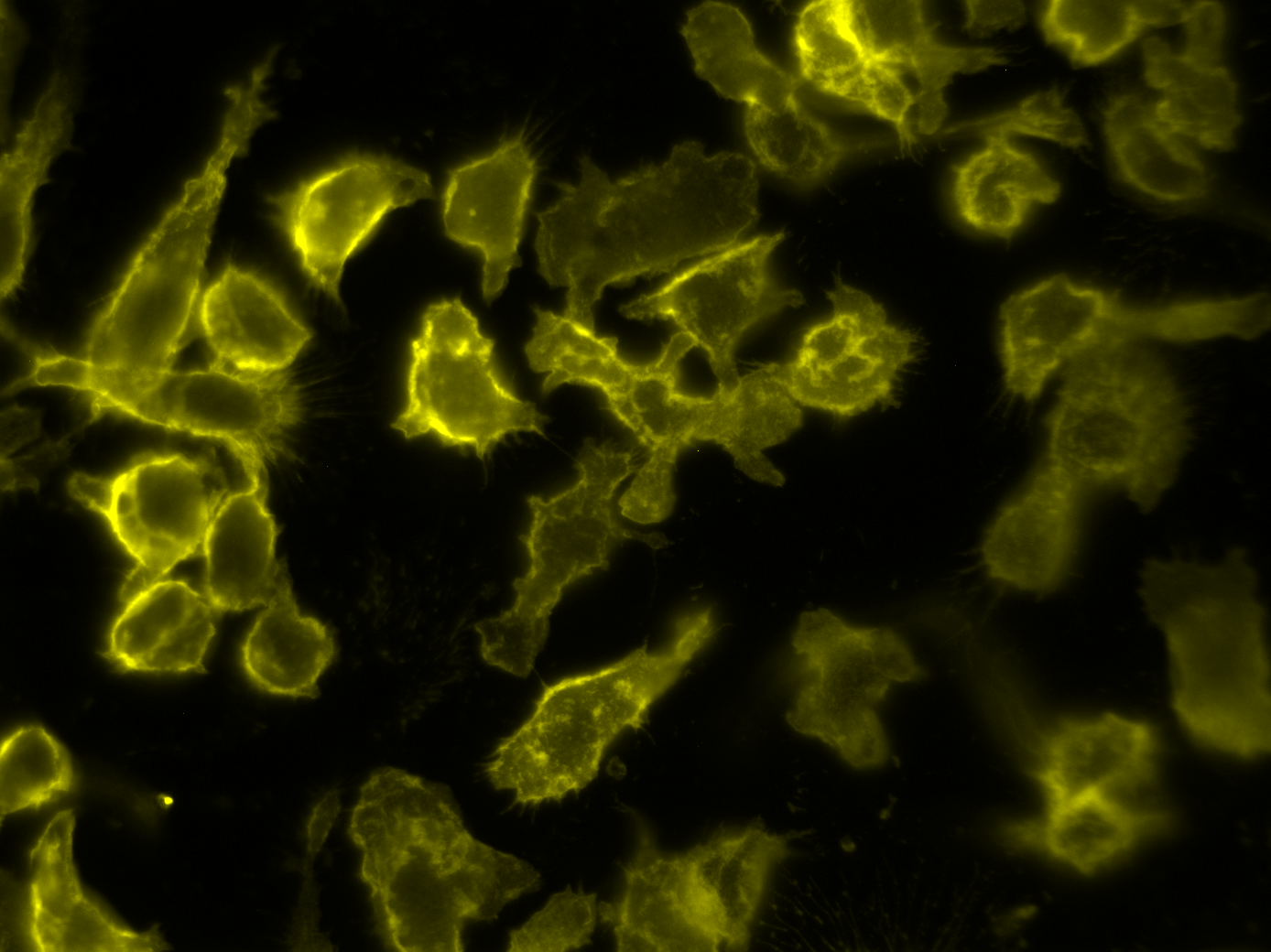

Supplement: Additional file 3 — The zip archive contains real images showing macrophages. (ZIP 28979 kb) [file 12859_2017_1591_MOESM3_ESM.zip › macrophages/jw-15min 2_c1.png]

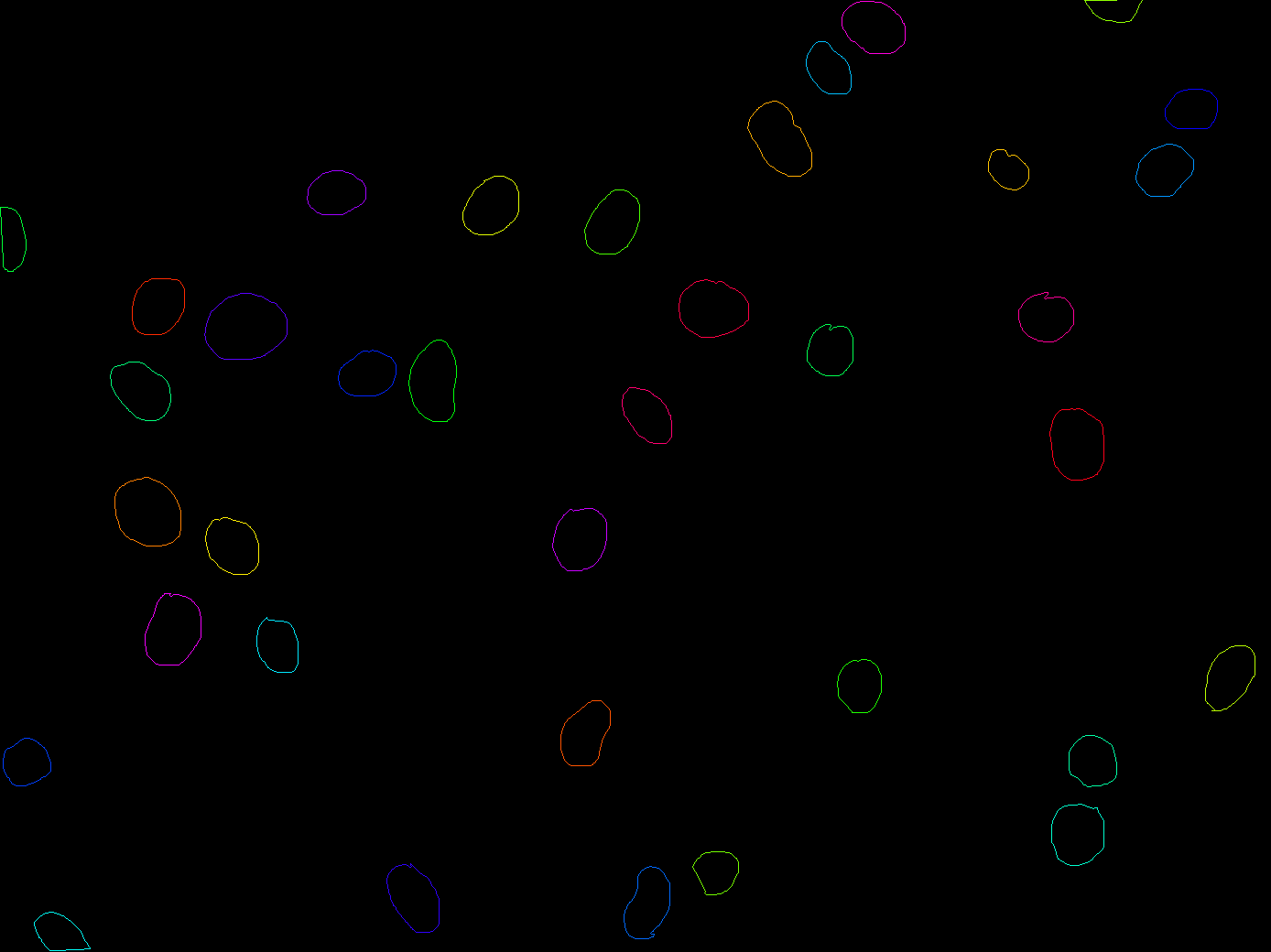

Supplement: Additional file 3 — The zip archive contains real images showing macrophages. (ZIP 28979 kb) [file 12859_2017_1591_MOESM3_ESM.zip › macrophages/jw-15min 2_c5 gt.png]

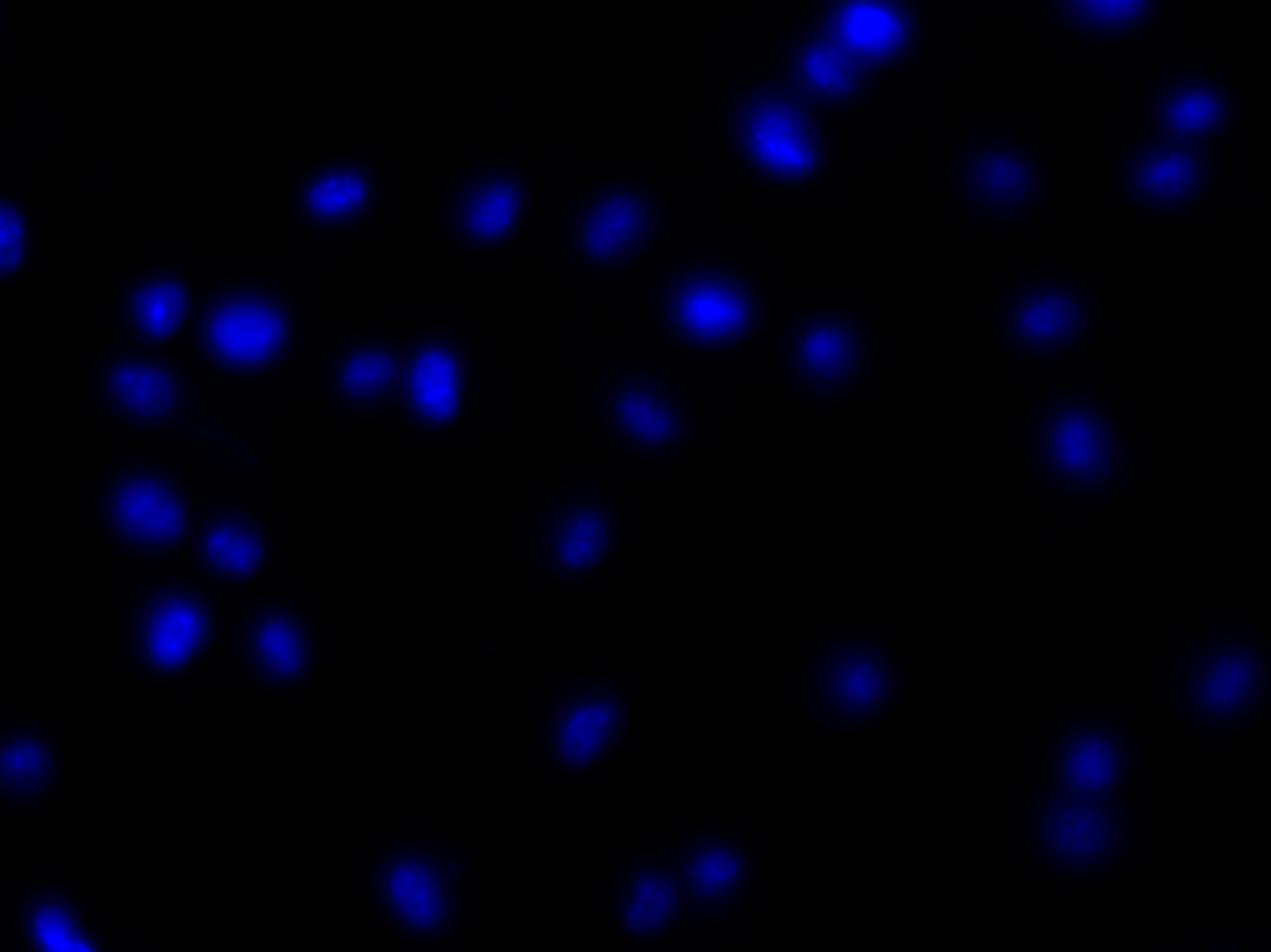

Supplement: Additional file 3 — The zip archive contains real images showing macrophages. (ZIP 28979 kb) [file 12859_2017_1591_MOESM3_ESM.zip › macrophages/jw-15min 2_c5.png]

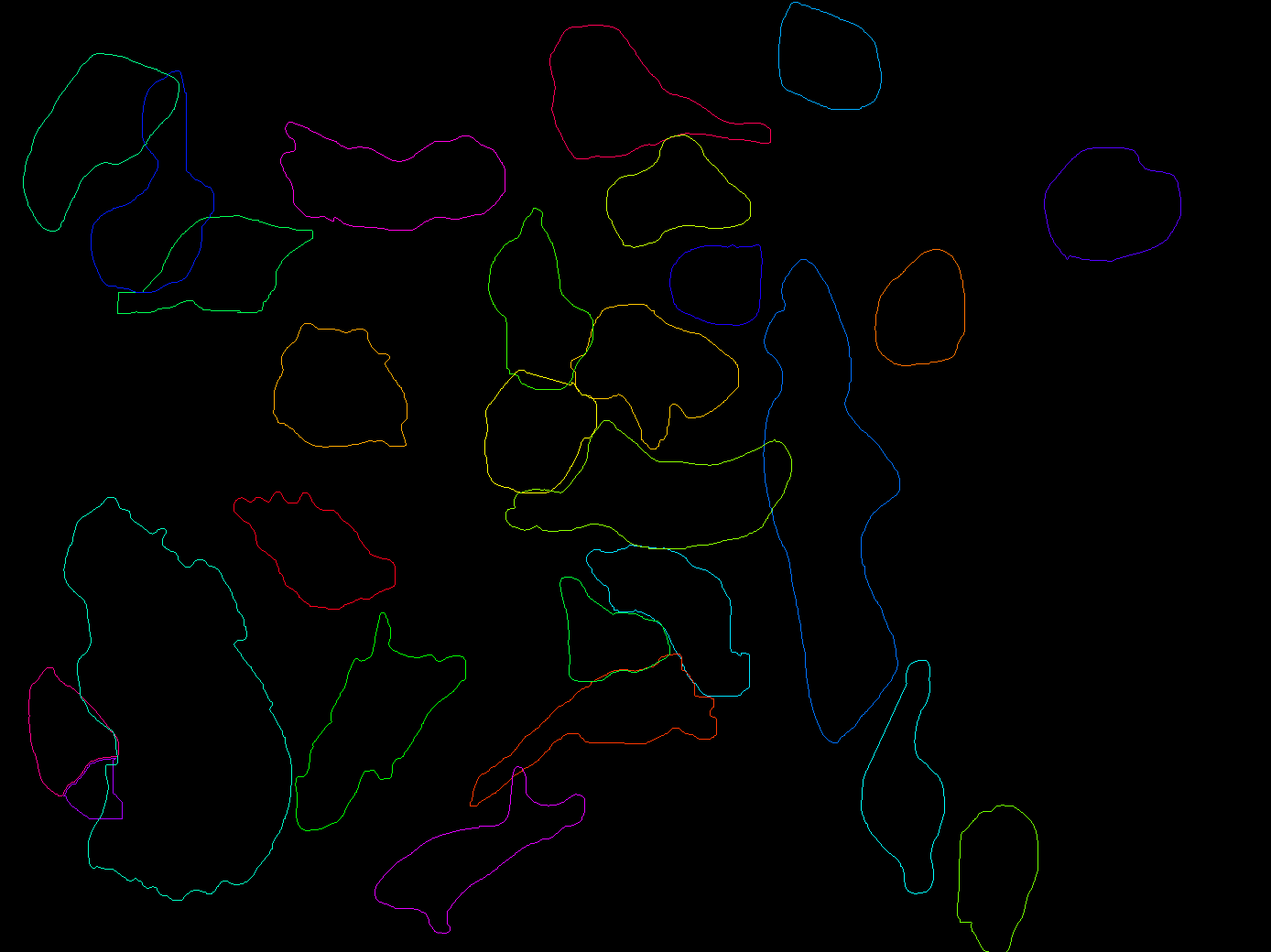

Supplement: Additional file 3 — The zip archive contains real images showing macrophages. (ZIP 28979 kb) [file 12859_2017_1591_MOESM3_ESM.zip › macrophages/jw-15min 3_c1 gt.png]

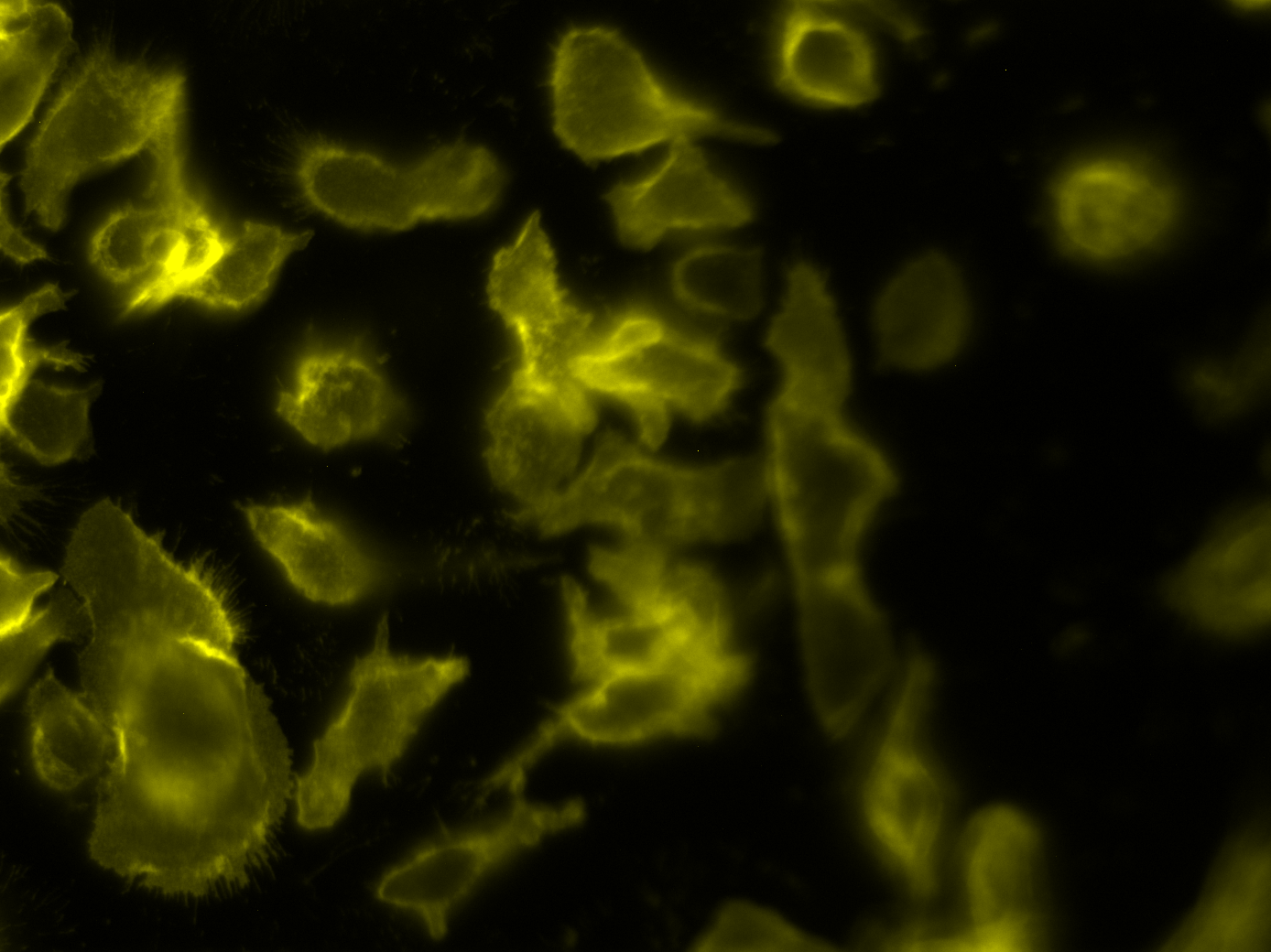

Supplement: Additional file 3 — The zip archive contains real images showing macrophages. (ZIP 28979 kb) [file 12859_2017_1591_MOESM3_ESM.zip › macrophages/jw-15min 3_c1.png]

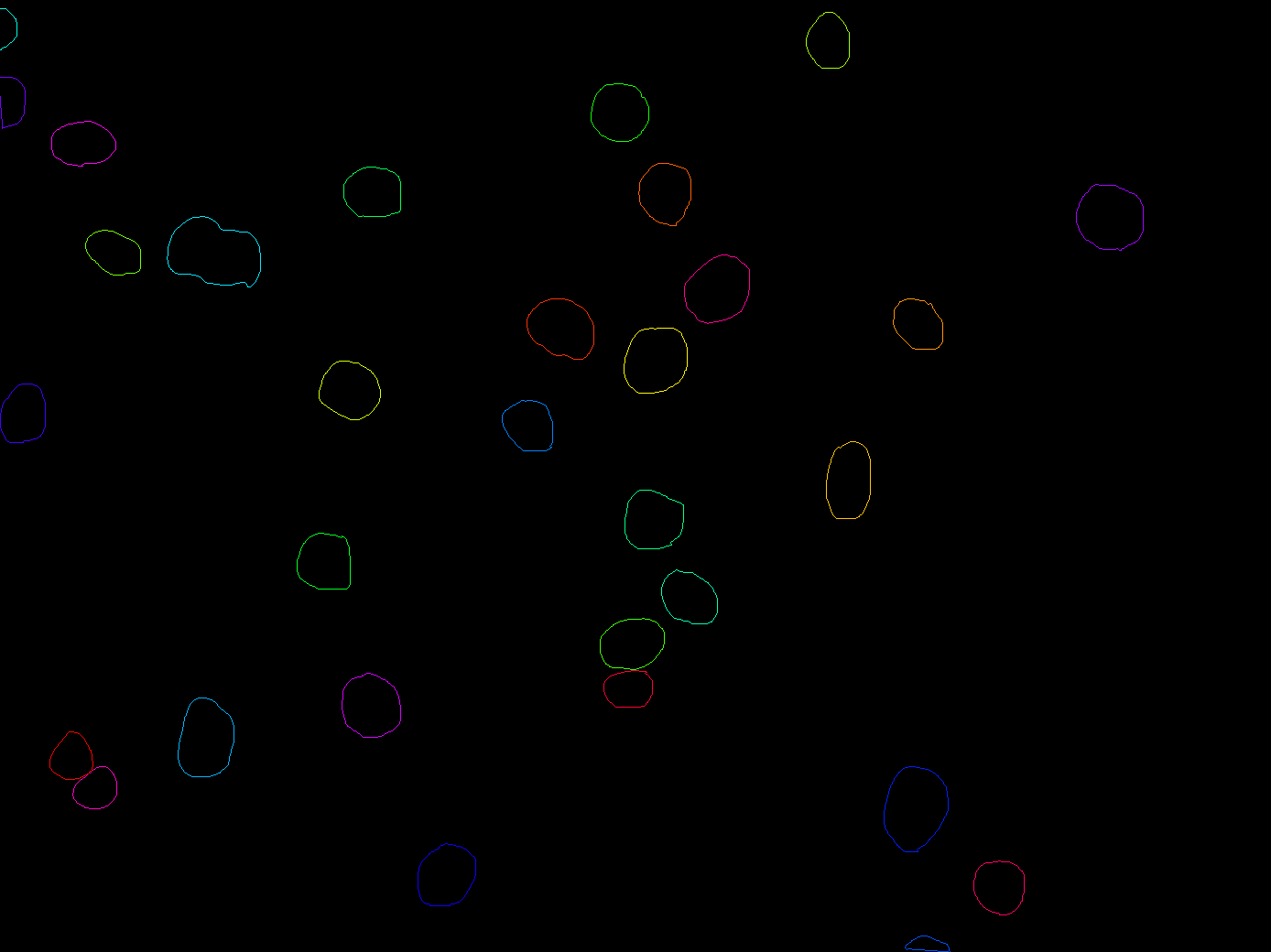

Supplement: Additional file 3 — The zip archive contains real images showing macrophages. (ZIP 28979 kb) [file 12859_2017_1591_MOESM3_ESM.zip › macrophages/jw-15min 3_c5 gt.png]

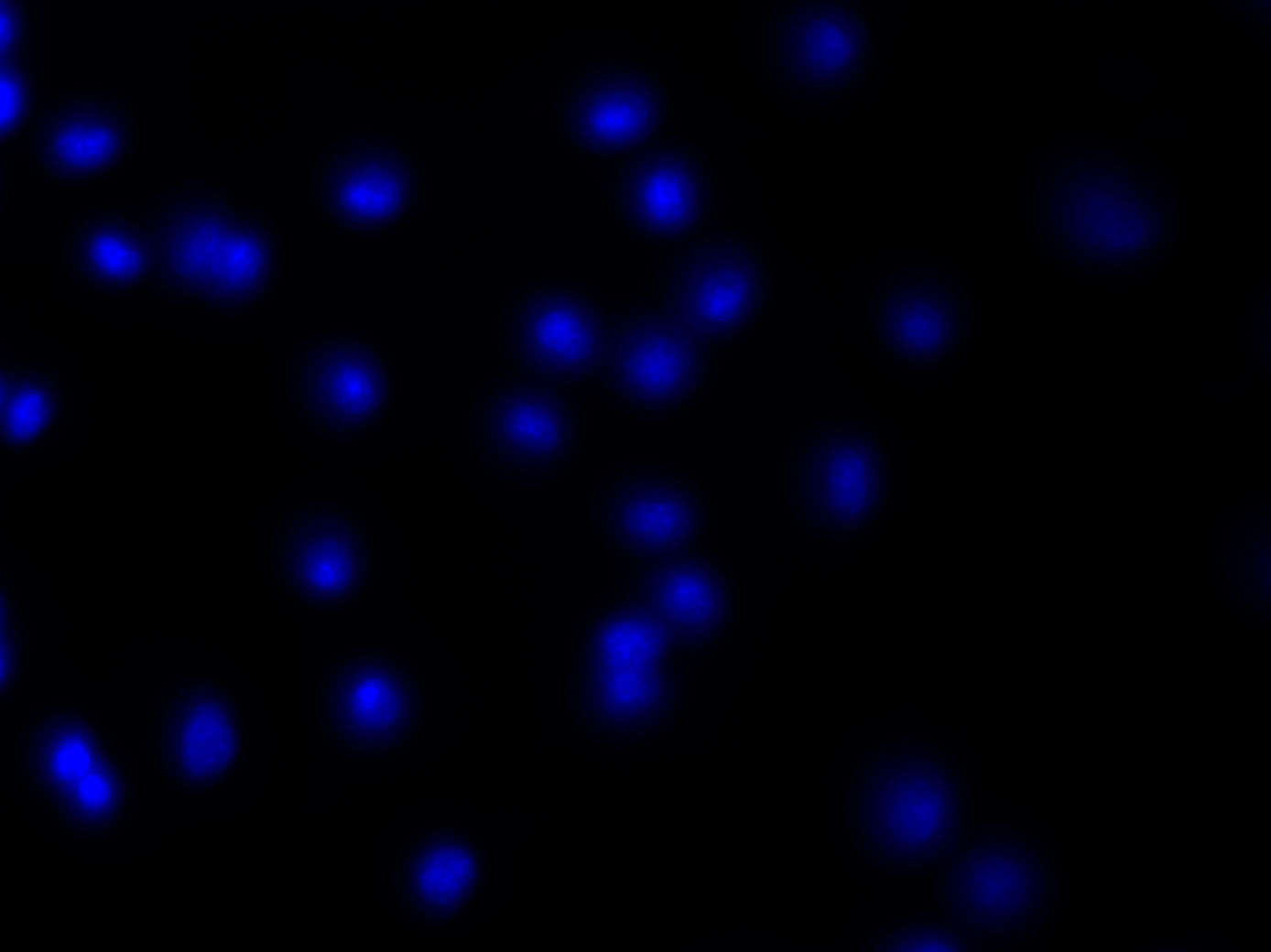

Supplement: Additional file 3 — The zip archive contains real images showing macrophages. (ZIP 28979 kb) [file 12859_2017_1591_MOESM3_ESM.zip › macrophages/jw-15min 3_c5.png]

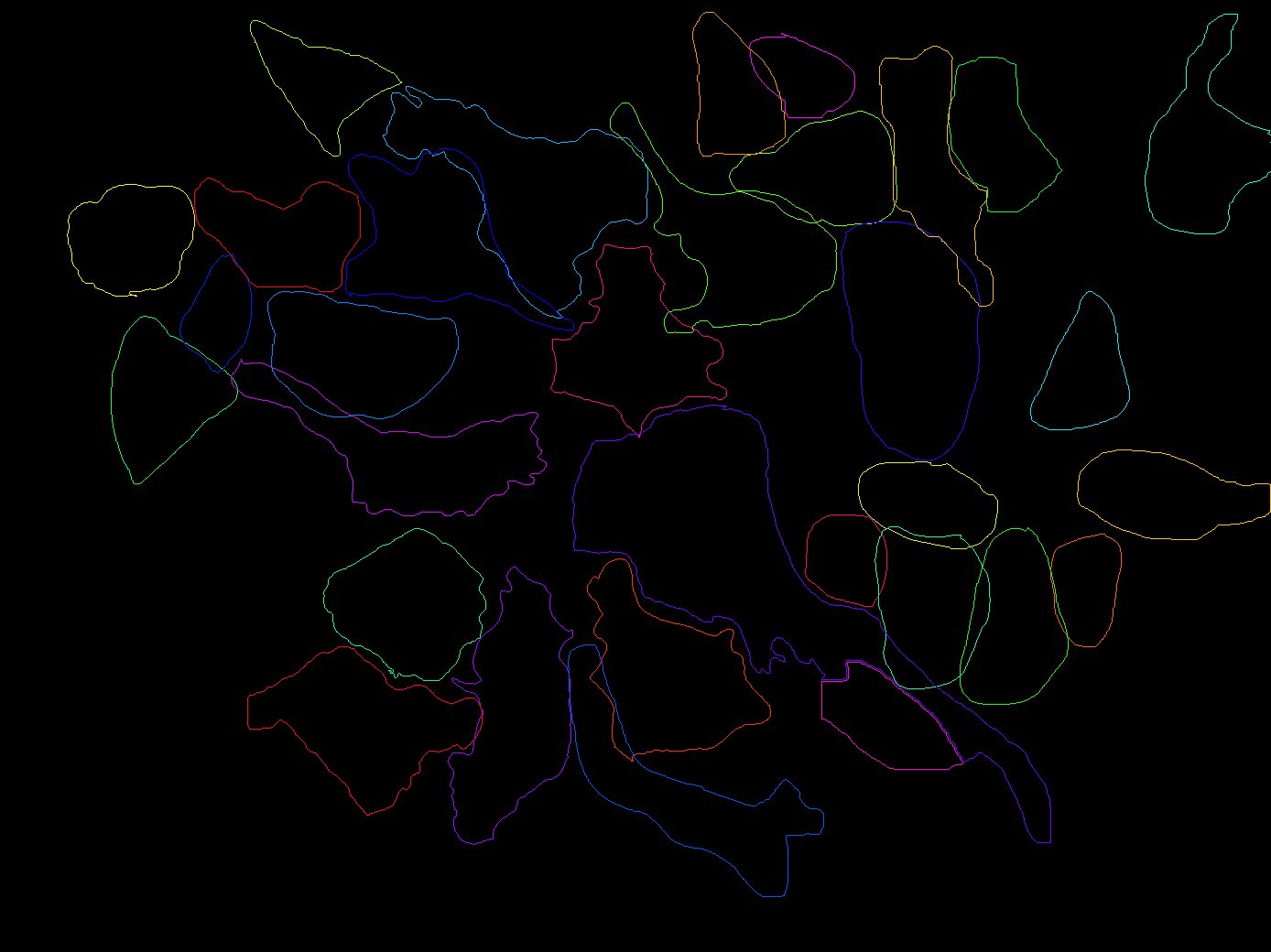

Supplement: Additional file 3 — The zip archive contains real images showing macrophages. (ZIP 28979 kb) [file 12859_2017_1591_MOESM3_ESM.zip › macrophages/jw-15min 4_c1 gt.png]

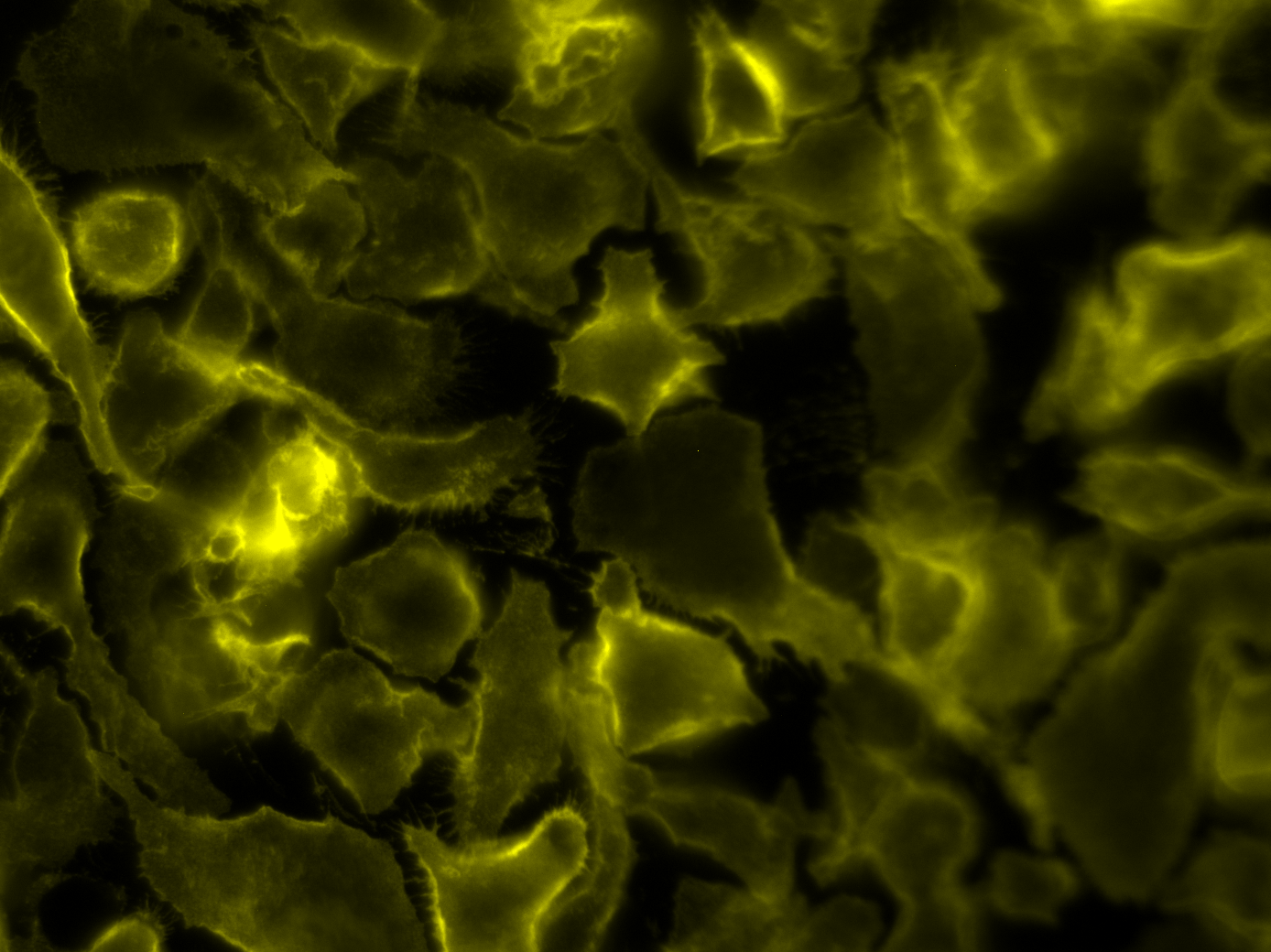

Supplement: Additional file 3 — The zip archive contains real images showing macrophages. (ZIP 28979 kb) [file 12859_2017_1591_MOESM3_ESM.zip › macrophages/jw-15min 4_c1.png]

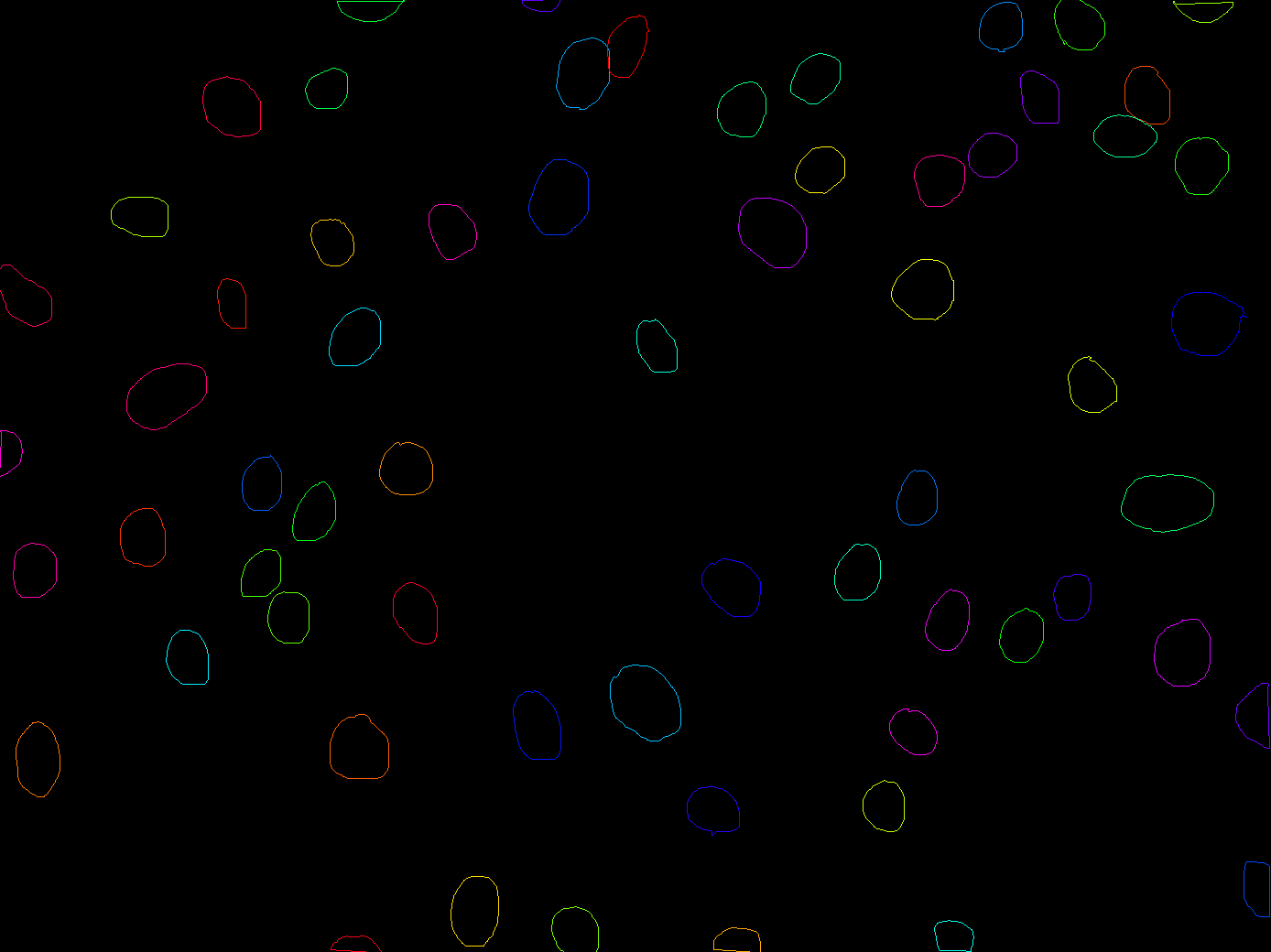

Supplement: Additional file 3 — The zip archive contains real images showing macrophages. (ZIP 28979 kb) [file 12859_2017_1591_MOESM3_ESM.zip › macrophages/jw-15min 4_c5 gt.png]

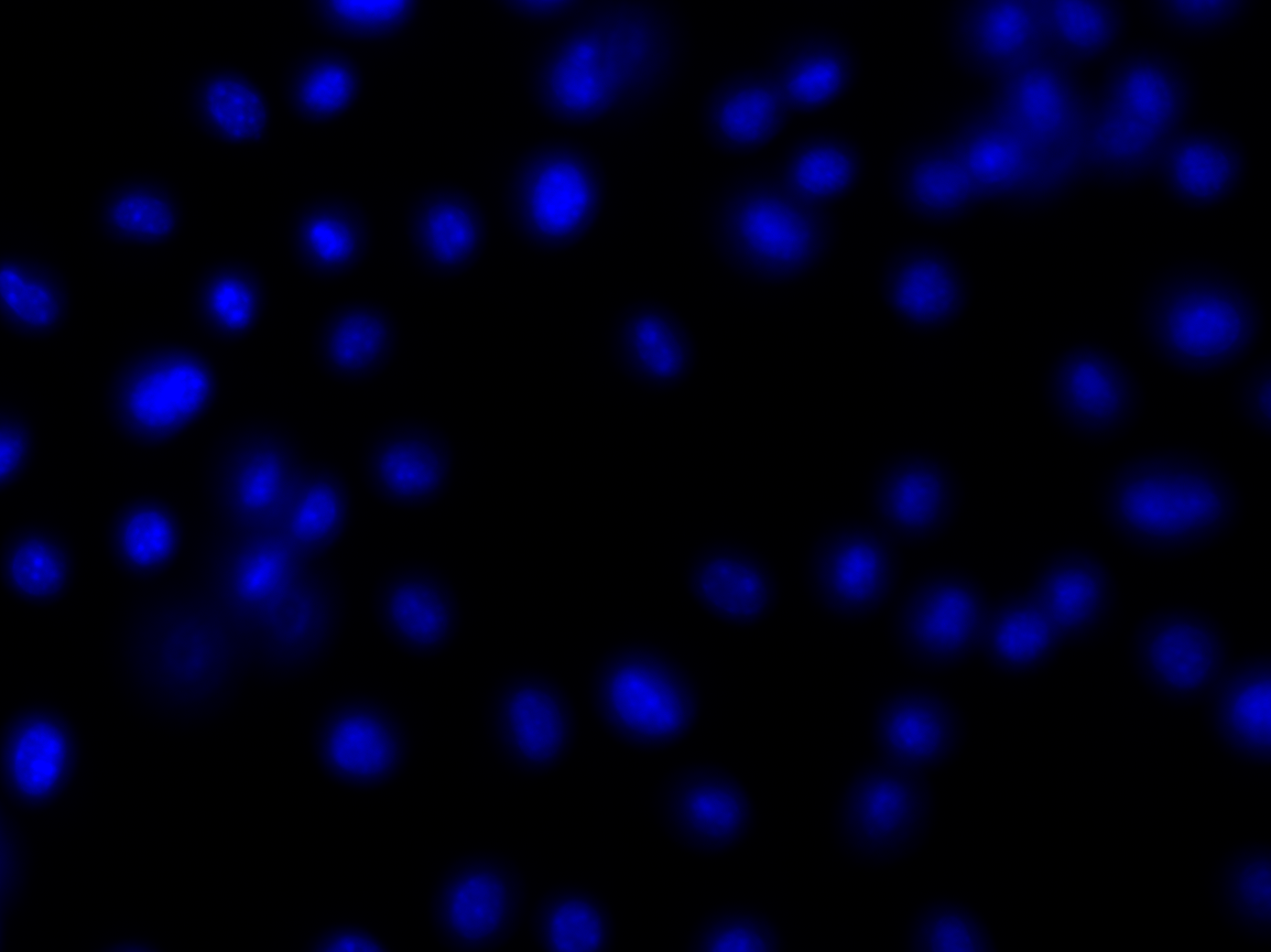

Supplement: Additional file 3 — The zip archive contains real images showing macrophages. (ZIP 28979 kb) [file 12859_2017_1591_MOESM3_ESM.zip › macrophages/jw-15min 4_c5.png]

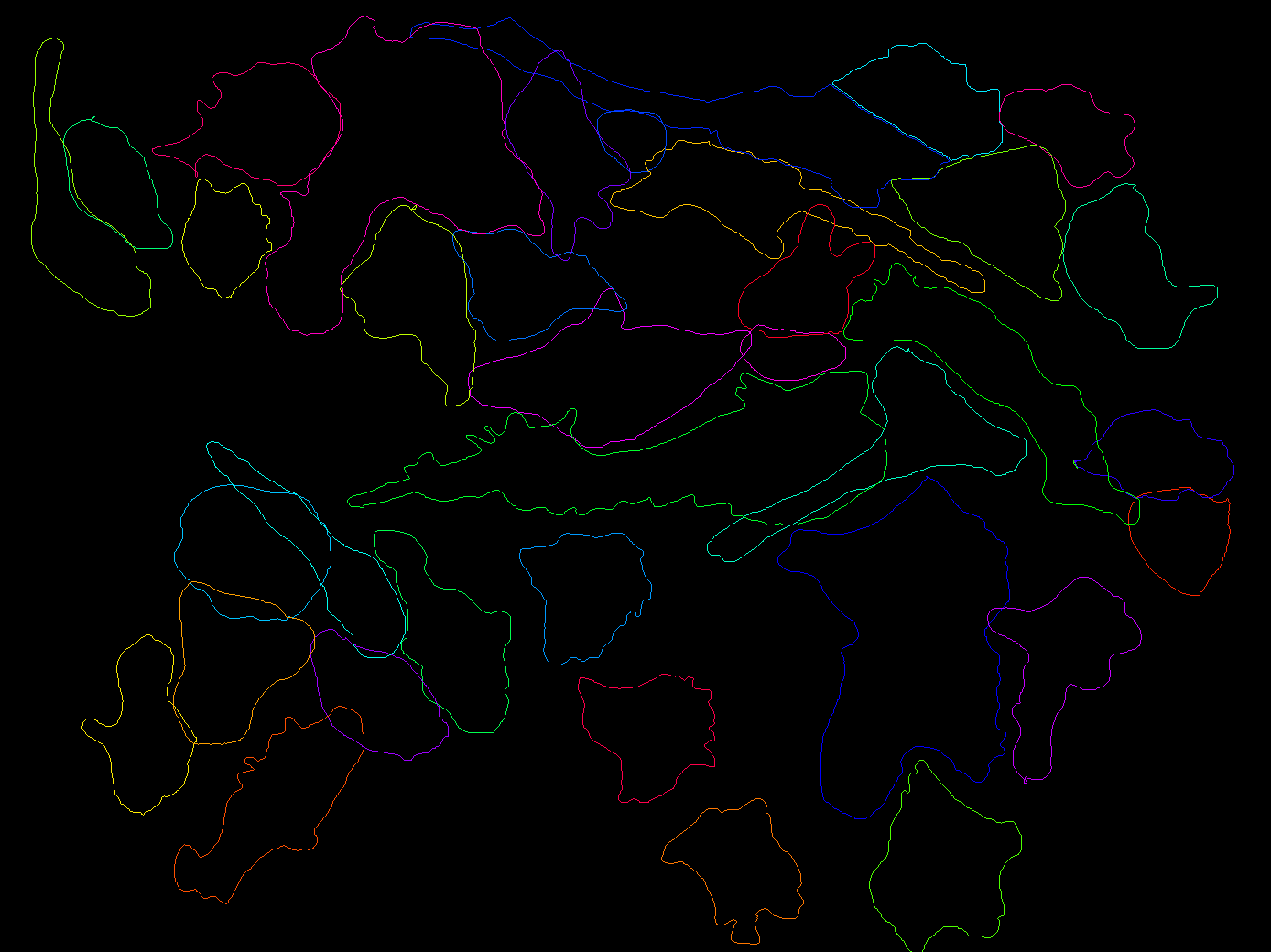

Supplement: Additional file 3 — The zip archive contains real images showing macrophages. (ZIP 28979 kb) [file 12859_2017_1591_MOESM3_ESM.zip › macrophages/jw-15min 5_c1 gt.png]

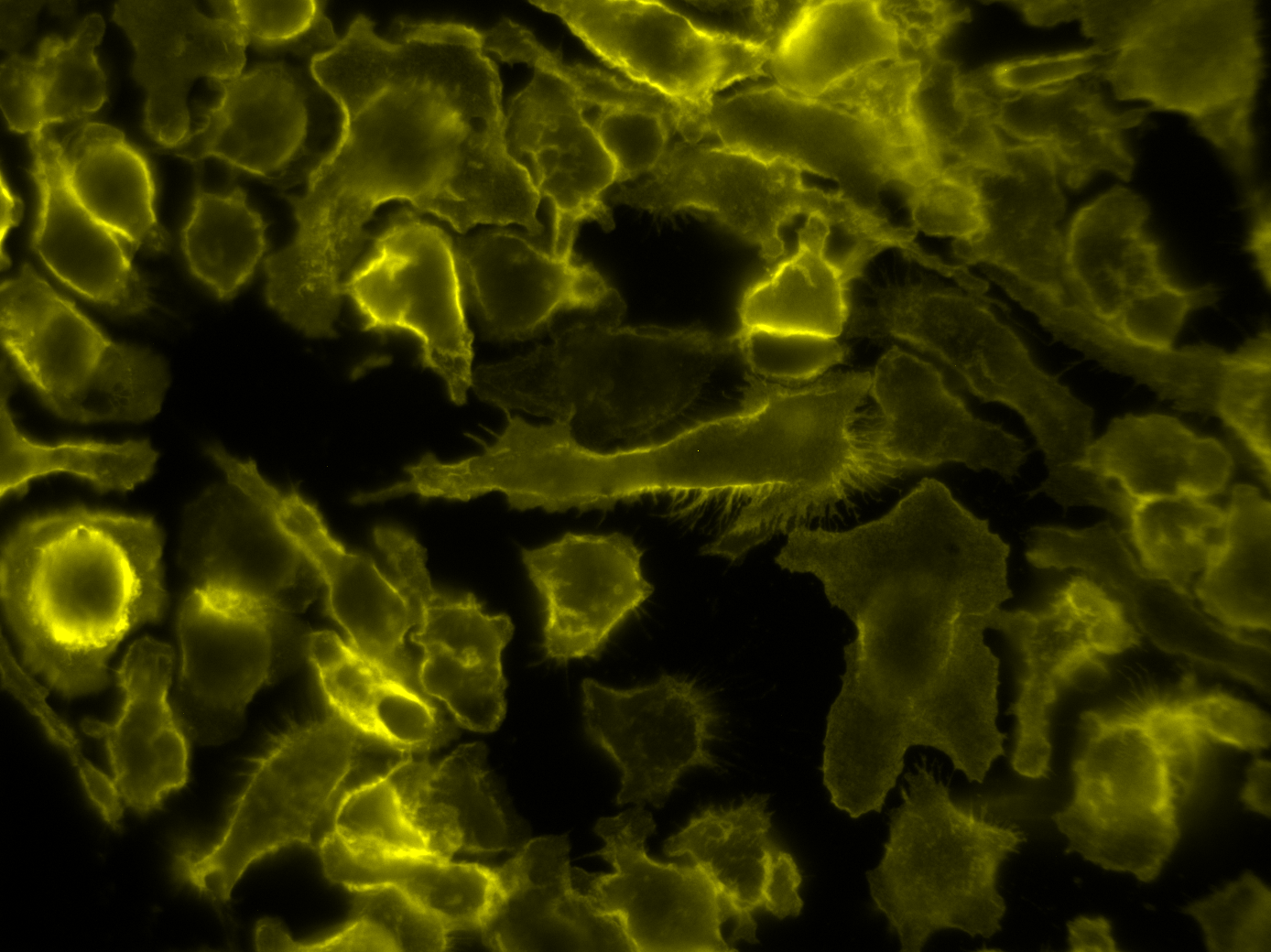

Supplement: Additional file 3 — The zip archive contains real images showing macrophages. (ZIP 28979 kb) [file 12859_2017_1591_MOESM3_ESM.zip › macrophages/jw-15min 5_c1.png]

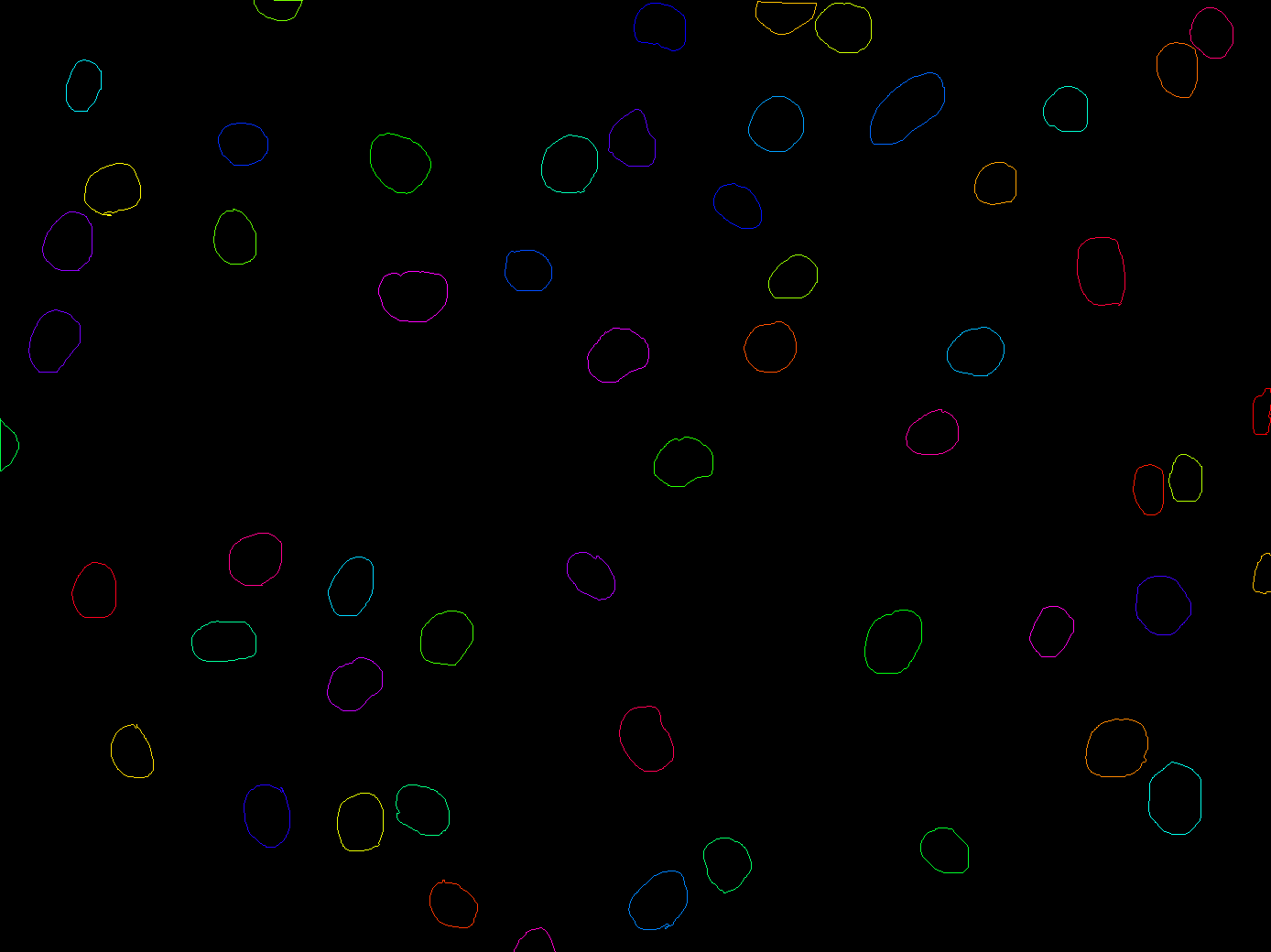

Supplement: Additional file 3 — The zip archive contains real images showing macrophages. (ZIP 28979 kb) [file 12859_2017_1591_MOESM3_ESM.zip › macrophages/jw-15min 5_c5 gt.png]

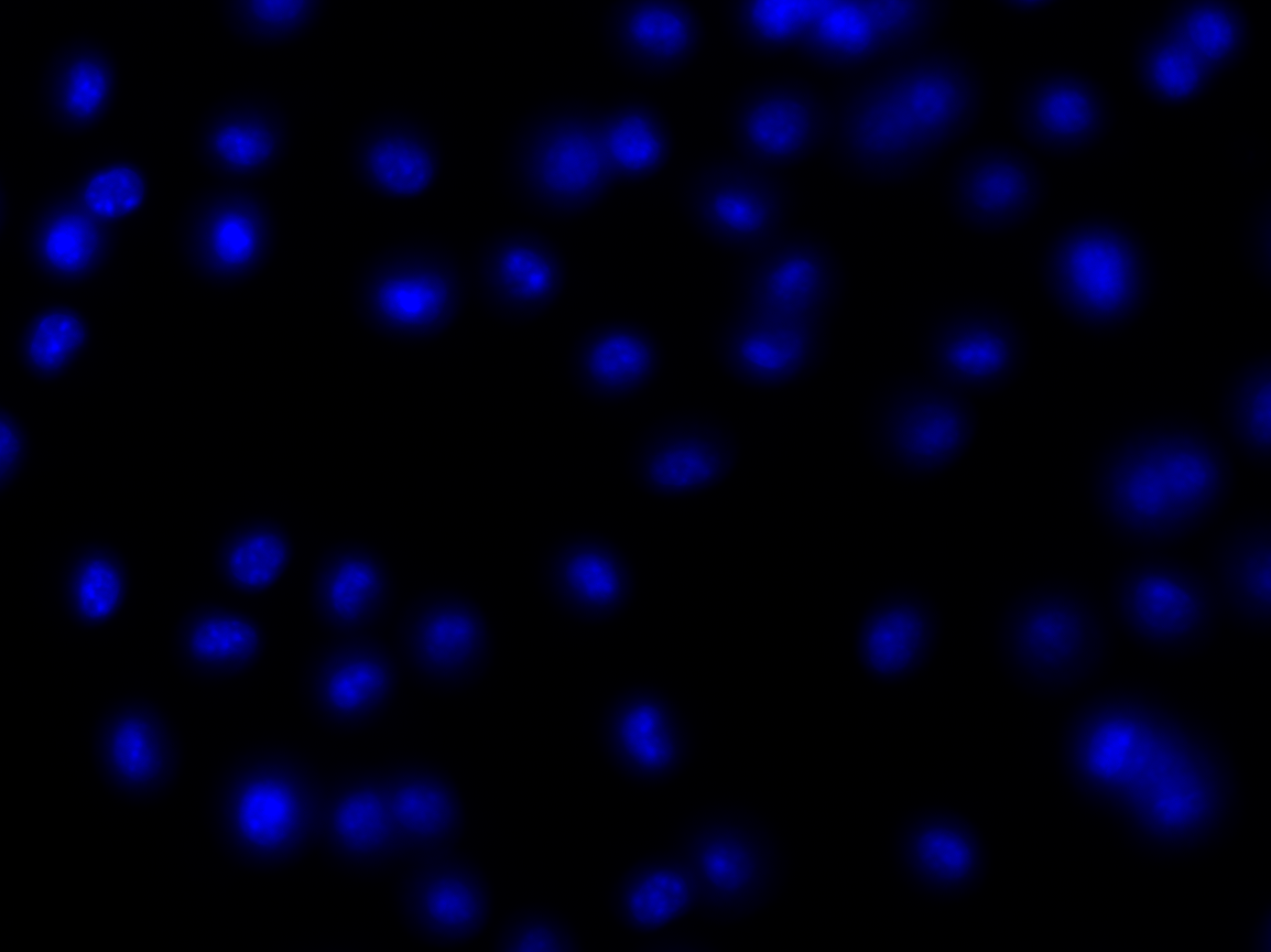

Supplement: Additional file 3 — The zip archive contains real images showing macrophages. (ZIP 28979 kb) [file 12859_2017_1591_MOESM3_ESM.zip › macrophages/jw-15min 5_c5.png]
