# Supplementary figures and images for: Using simulated fluorescence cell micrographs for the evaluation of cell image segmentation algorithms (part 2 of 6)
Source: BMC Bioinformatics. 2017 Mar 18;18:176. doi: 10.1186/s12859-017-1591-2 (PMC5357336; doi:10.1186/s12859-017-1591-2)

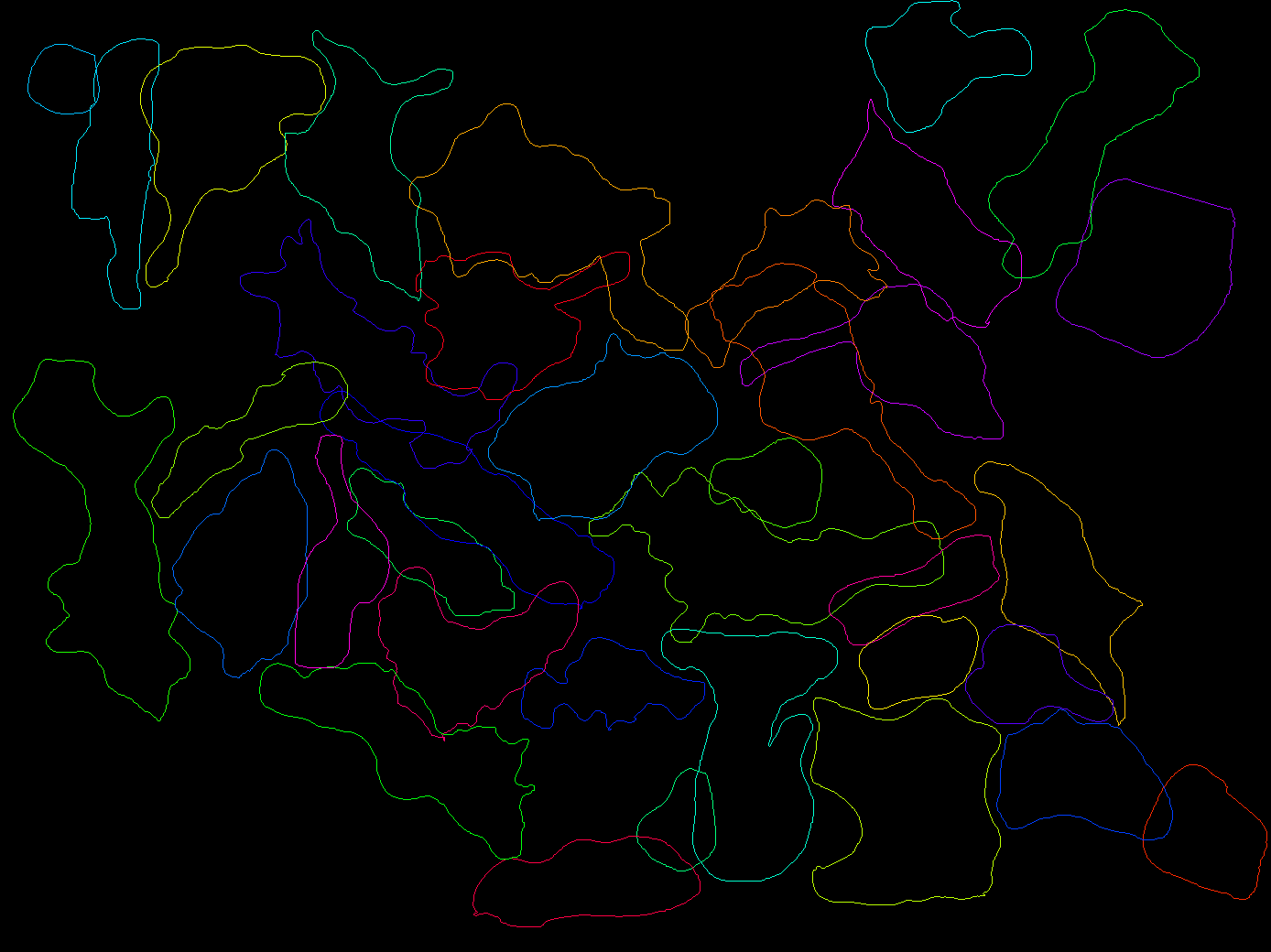

Supplement: Additional file 3 — The zip archive contains real images showing macrophages. (ZIP 28979 kb) [file 12859_2017_1591_MOESM3_ESM.zip › macrophages/jw-1h 1_c1 gt.png]

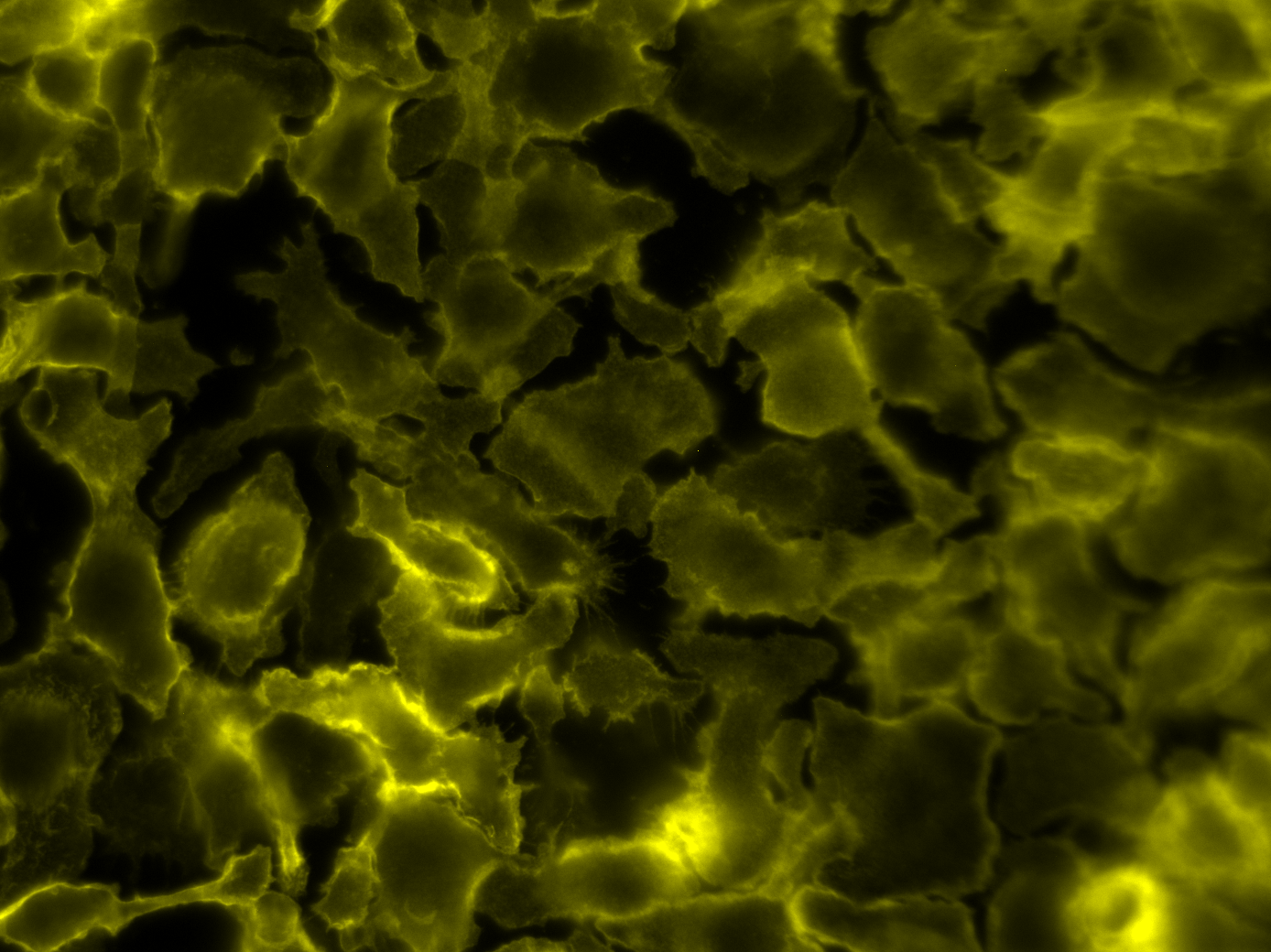

Supplement: Additional file 3 — The zip archive contains real images showing macrophages. (ZIP 28979 kb) [file 12859_2017_1591_MOESM3_ESM.zip › macrophages/jw-1h 1_c1.png]

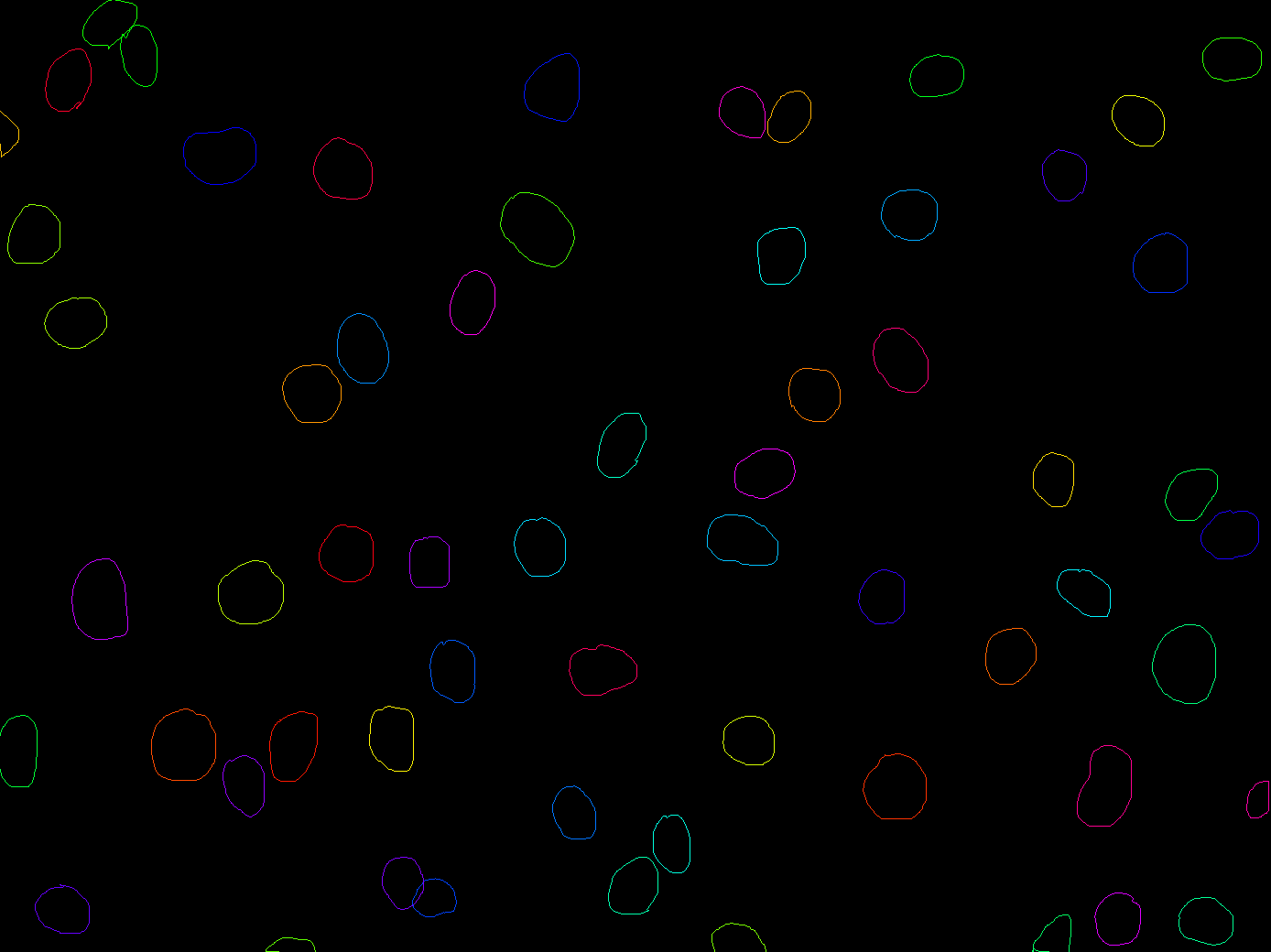

Supplement: Additional file 3 — The zip archive contains real images showing macrophages. (ZIP 28979 kb) [file 12859_2017_1591_MOESM3_ESM.zip › macrophages/jw-1h 1_c5 gt.png]

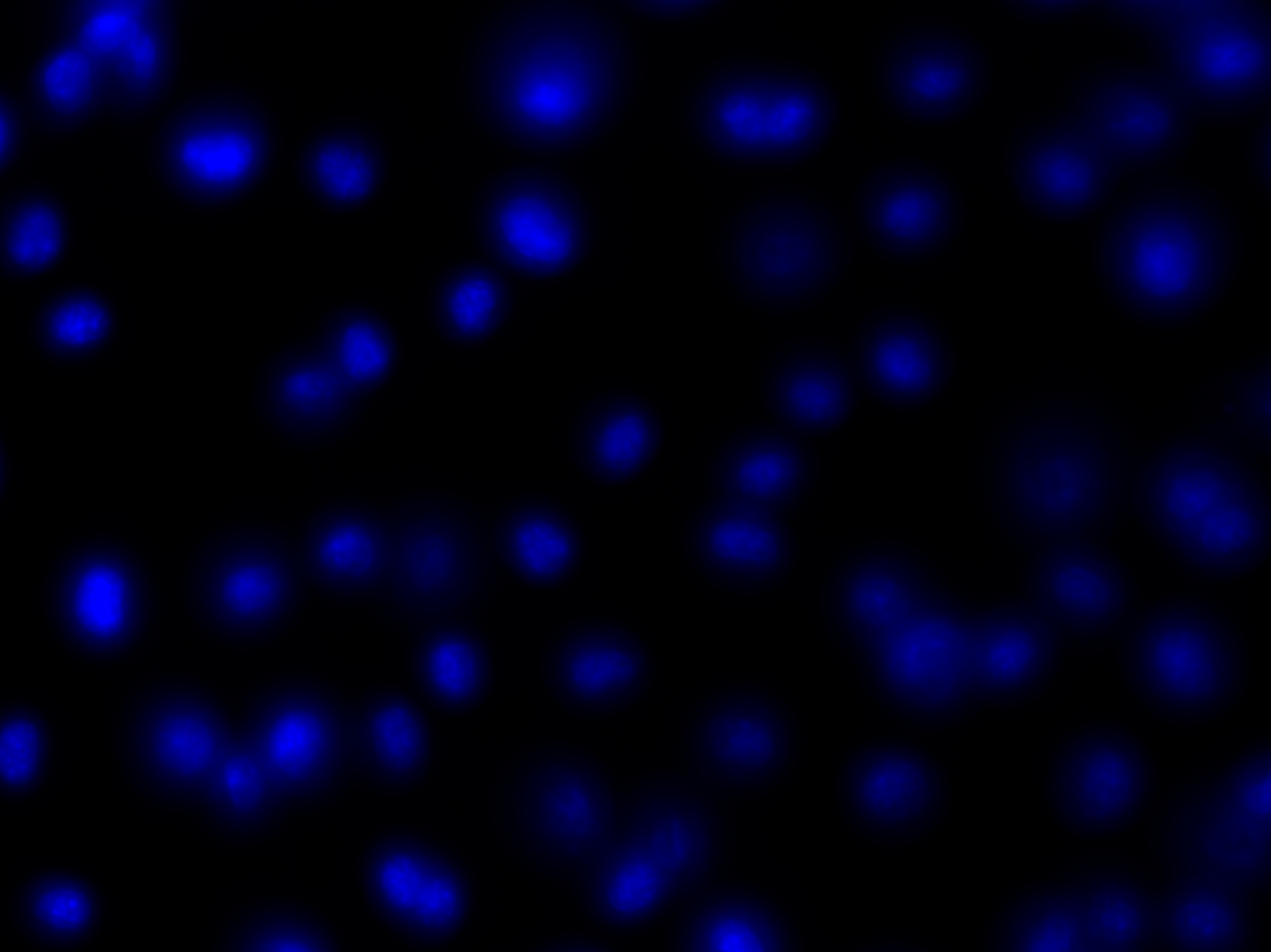

Supplement: Additional file 3 — The zip archive contains real images showing macrophages. (ZIP 28979 kb) [file 12859_2017_1591_MOESM3_ESM.zip › macrophages/jw-1h 1_c5.png]

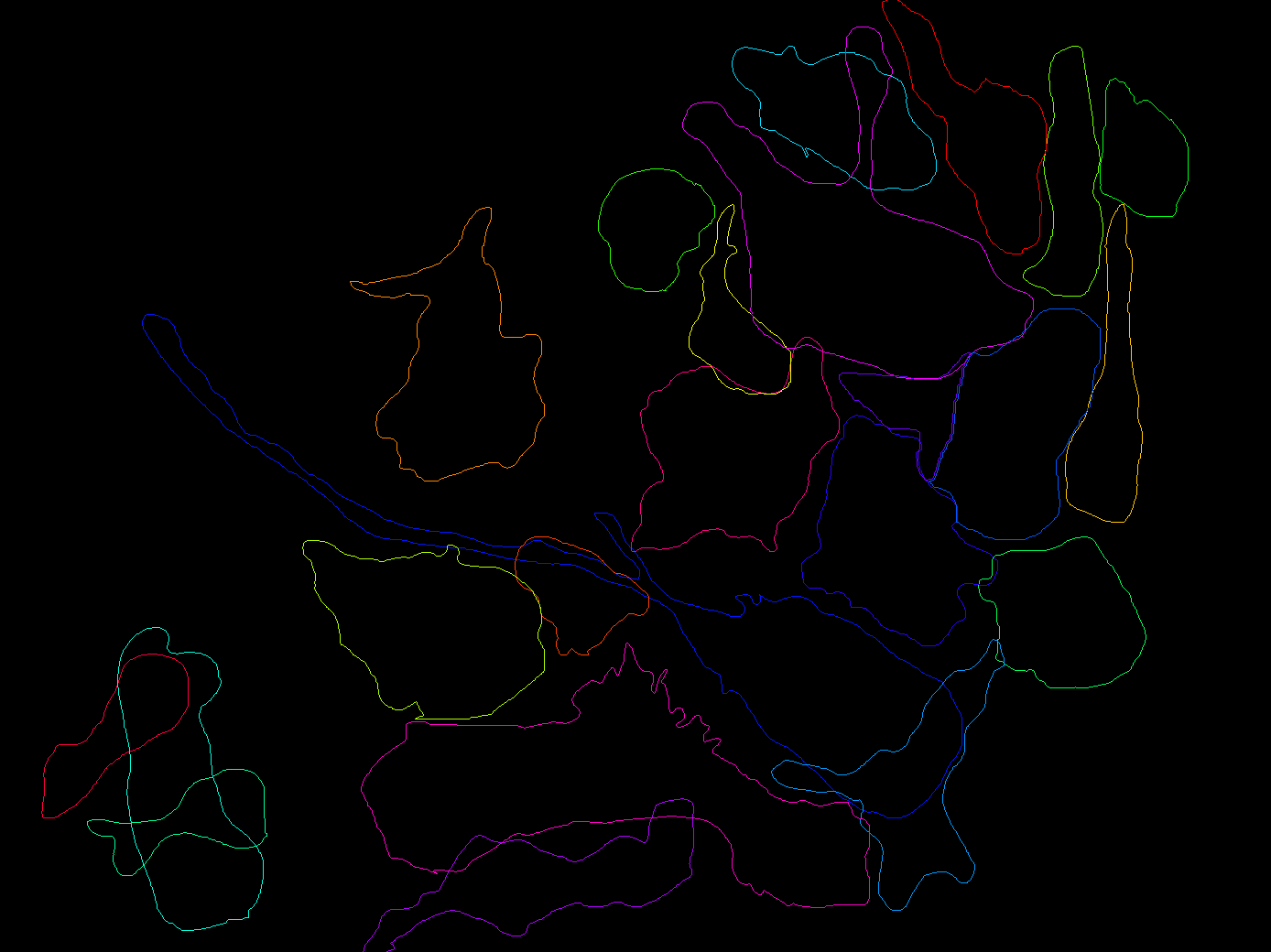

Supplement: Additional file 3 — The zip archive contains real images showing macrophages. (ZIP 28979 kb) [file 12859_2017_1591_MOESM3_ESM.zip › macrophages/jw-1h 2_c1 gt.png]

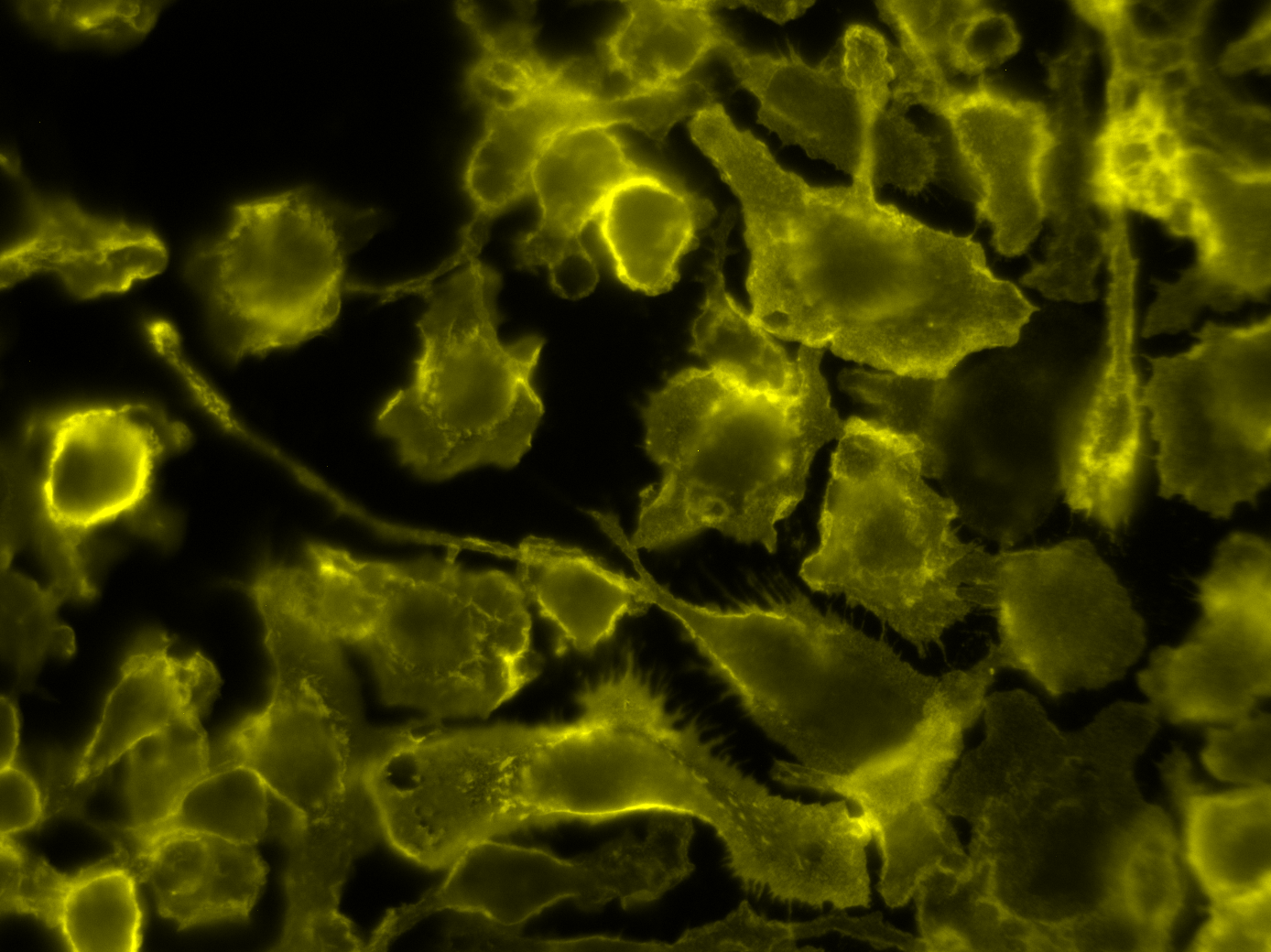

Supplement: Additional file 3 — The zip archive contains real images showing macrophages. (ZIP 28979 kb) [file 12859_2017_1591_MOESM3_ESM.zip › macrophages/jw-1h 2_c1.png]

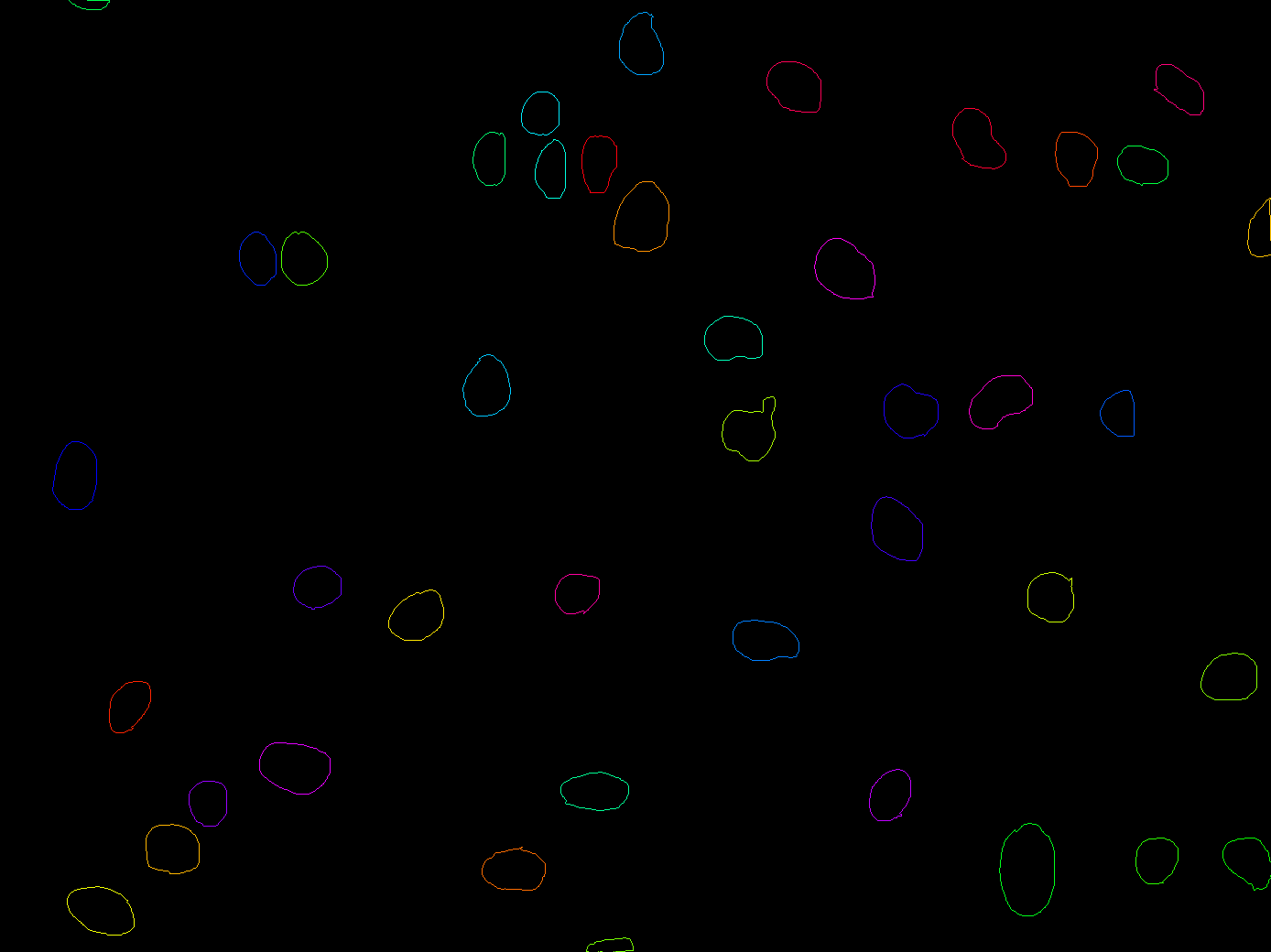

Supplement: Additional file 3 — The zip archive contains real images showing macrophages. (ZIP 28979 kb) [file 12859_2017_1591_MOESM3_ESM.zip › macrophages/jw-1h 2_c5 gt.png]

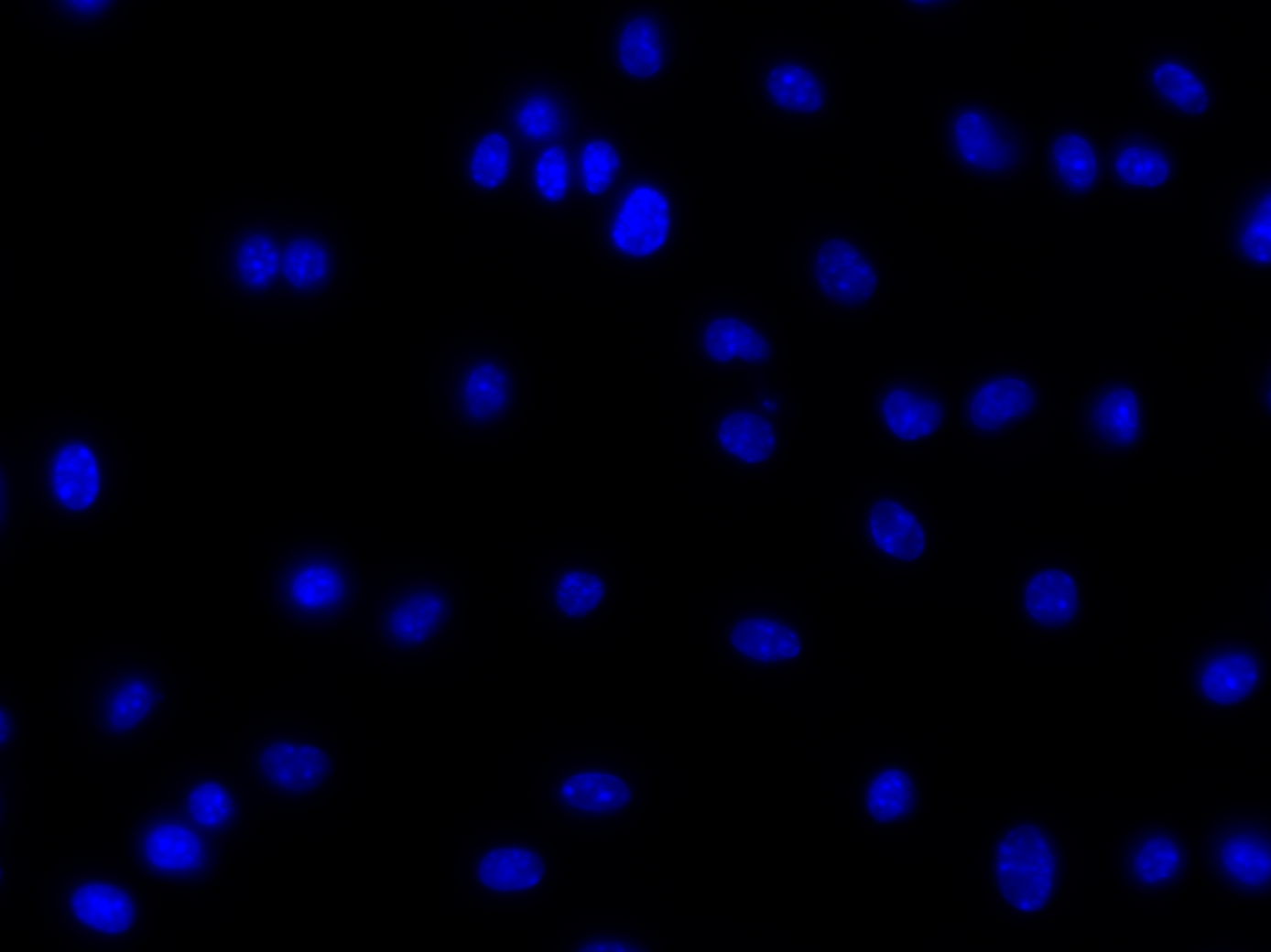

Supplement: Additional file 3 — The zip archive contains real images showing macrophages. (ZIP 28979 kb) [file 12859_2017_1591_MOESM3_ESM.zip › macrophages/jw-1h 2_c5.png]

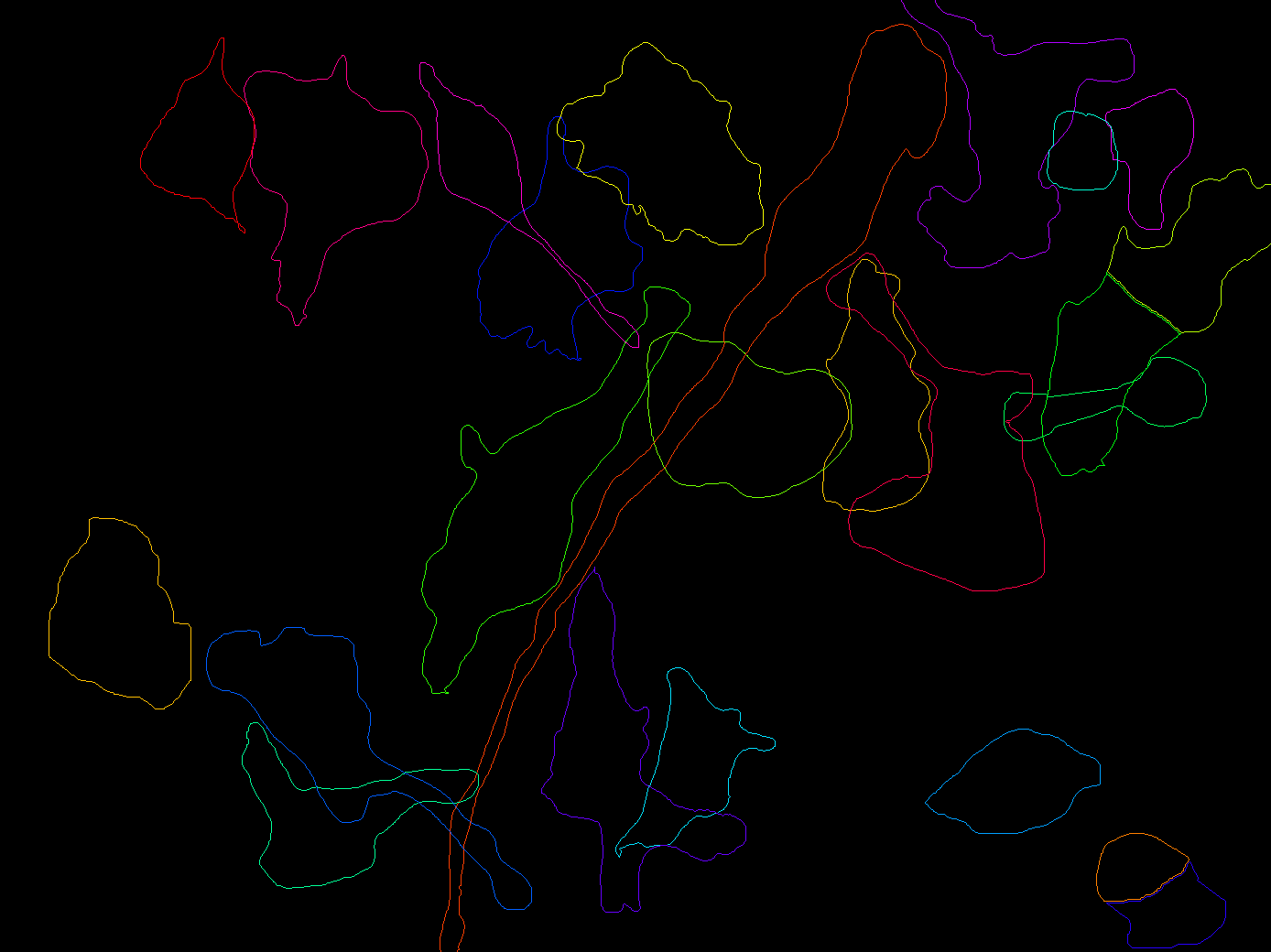

Supplement: Additional file 3 — The zip archive contains real images showing macrophages. (ZIP 28979 kb) [file 12859_2017_1591_MOESM3_ESM.zip › macrophages/jw-1h 3_c1 gt.png]

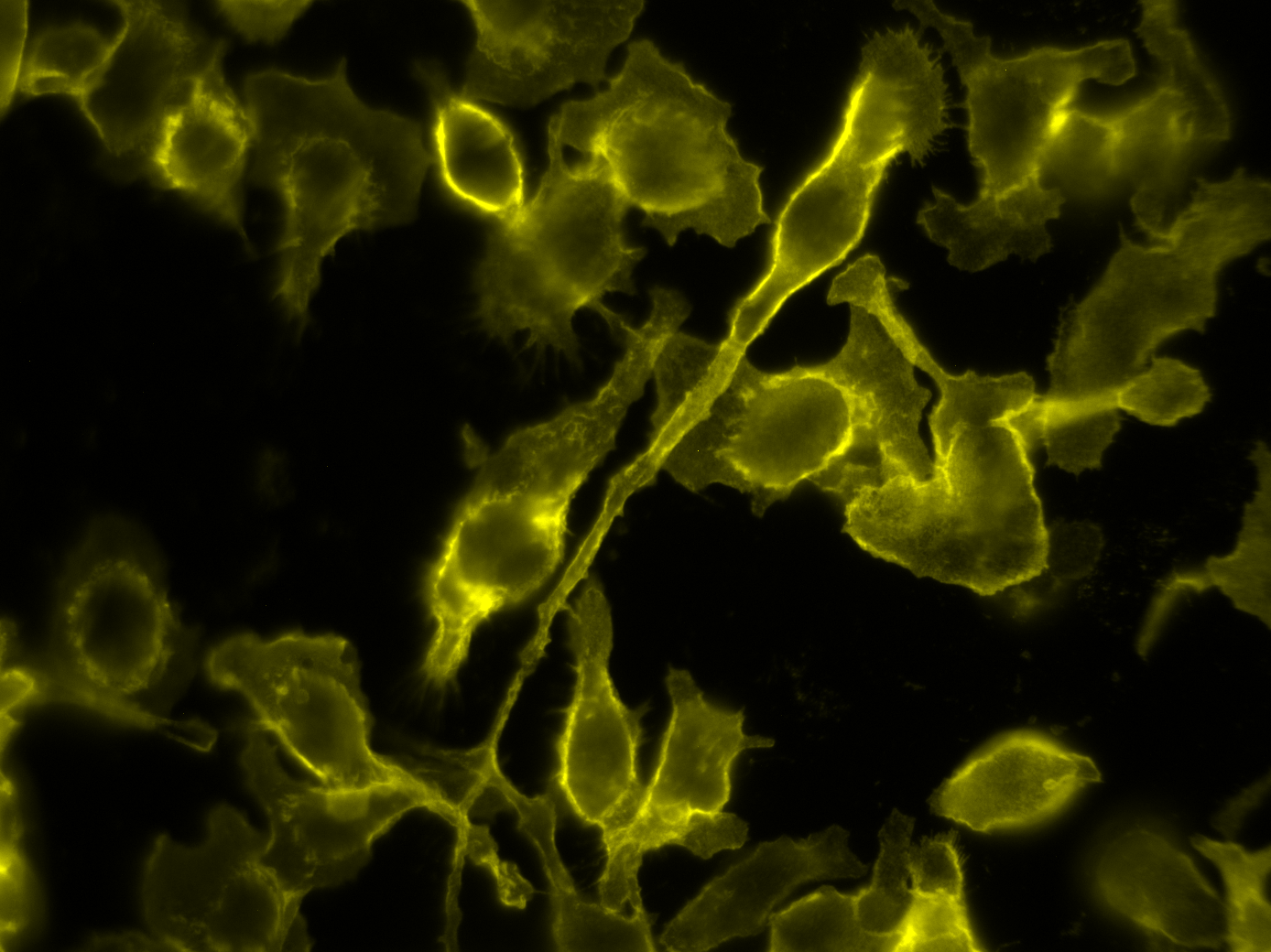

Supplement: Additional file 3 — The zip archive contains real images showing macrophages. (ZIP 28979 kb) [file 12859_2017_1591_MOESM3_ESM.zip › macrophages/jw-1h 3_c1.png]

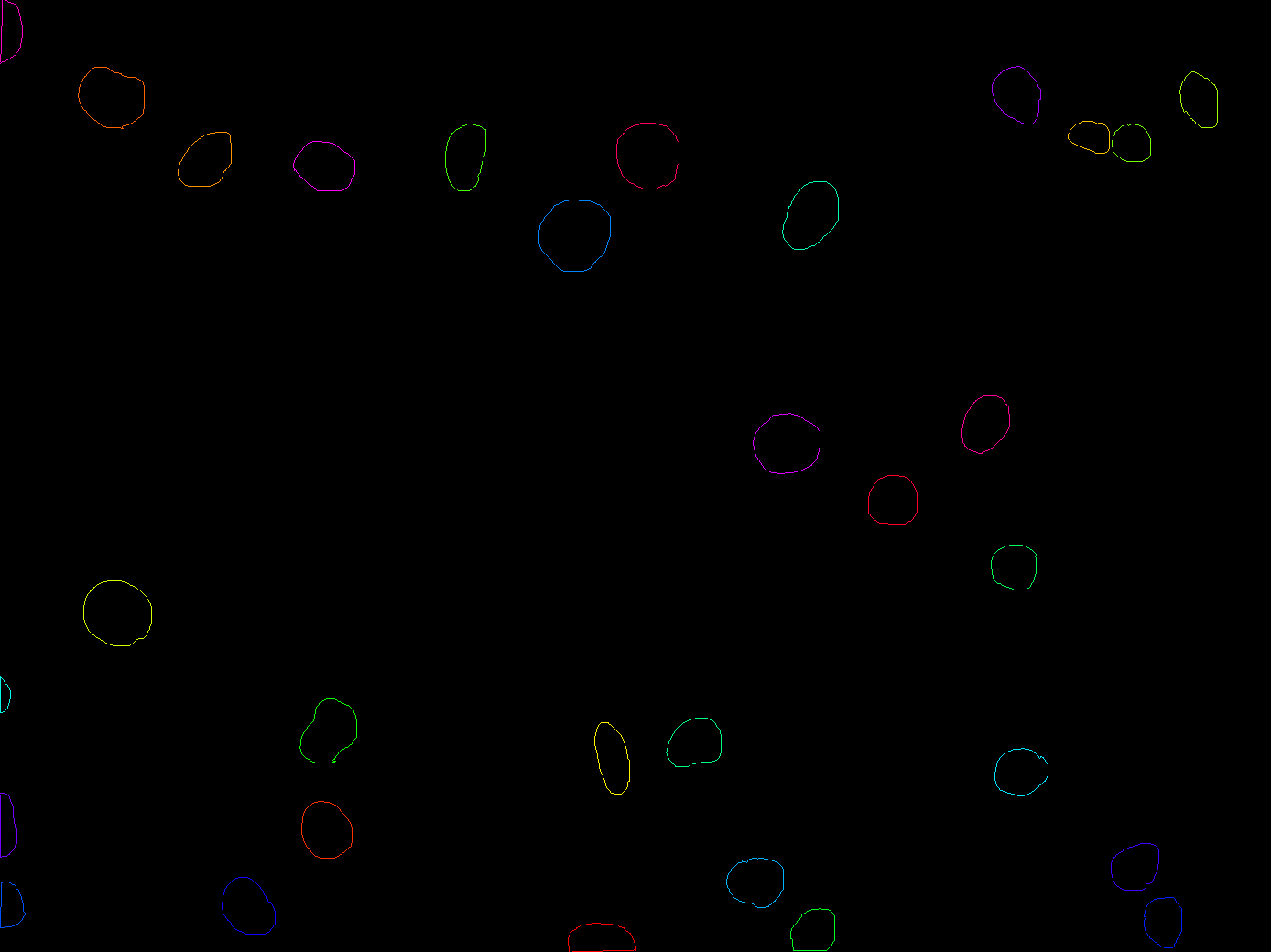

Supplement: Additional file 3 — The zip archive contains real images showing macrophages. (ZIP 28979 kb) [file 12859_2017_1591_MOESM3_ESM.zip › macrophages/jw-1h 3_c5 gt.png]

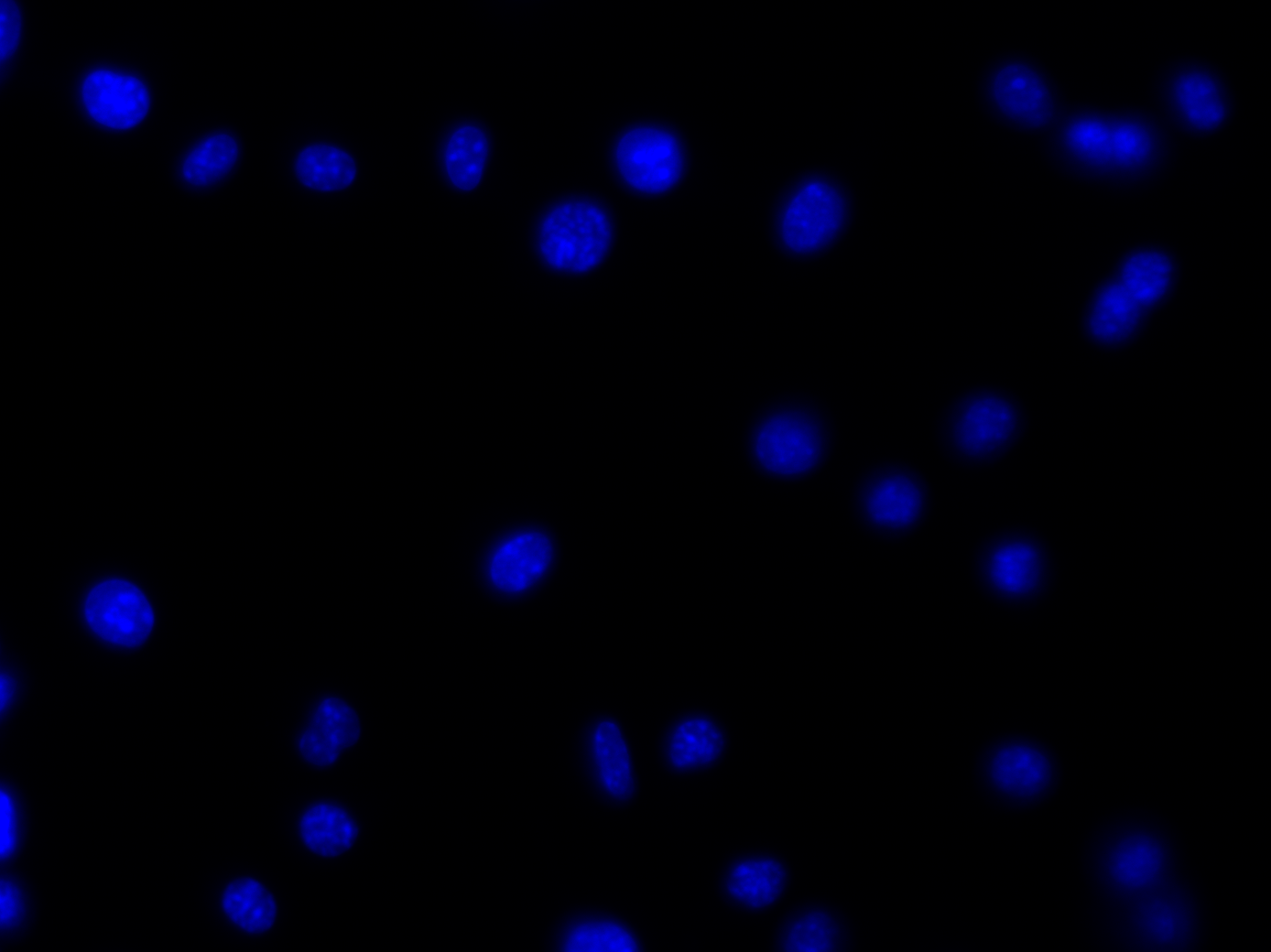

Supplement: Additional file 3 — The zip archive contains real images showing macrophages. (ZIP 28979 kb) [file 12859_2017_1591_MOESM3_ESM.zip › macrophages/jw-1h 3_c5.png]

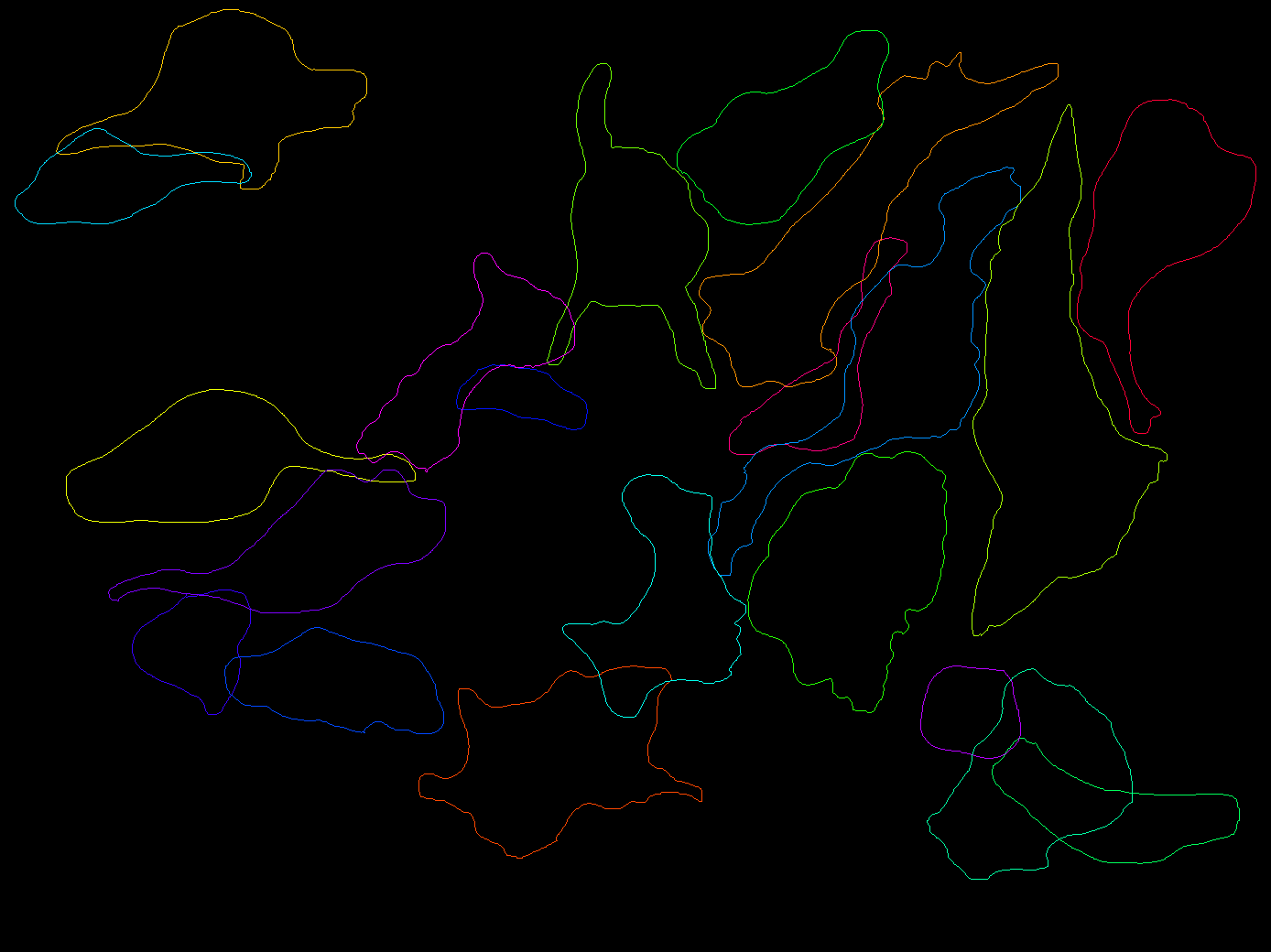

Supplement: Additional file 3 — The zip archive contains real images showing macrophages. (ZIP 28979 kb) [file 12859_2017_1591_MOESM3_ESM.zip › macrophages/jw-1h 4_c1 gt.png]

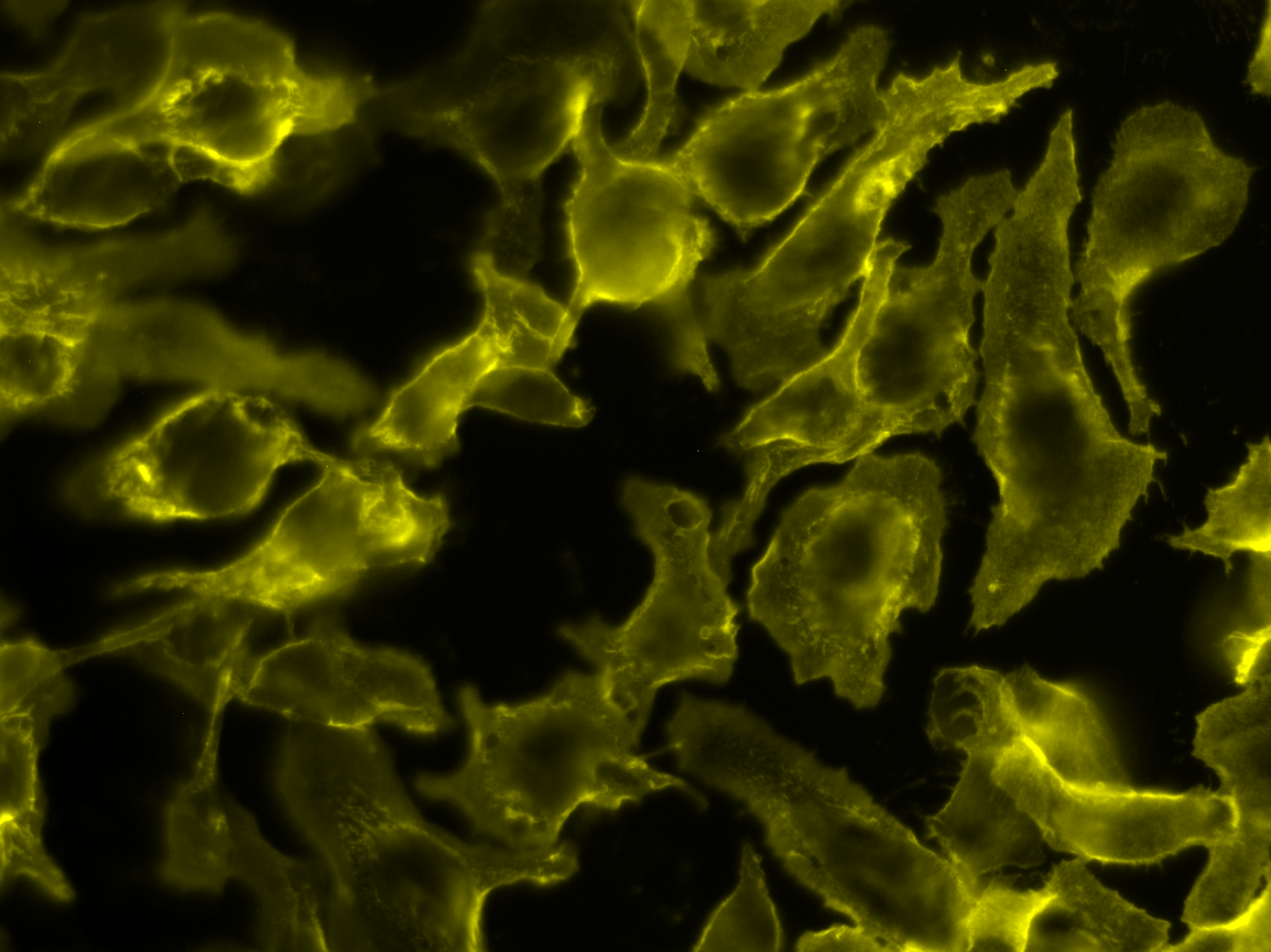

Supplement: Additional file 3 — The zip archive contains real images showing macrophages. (ZIP 28979 kb) [file 12859_2017_1591_MOESM3_ESM.zip › macrophages/jw-1h 4_c1.png]

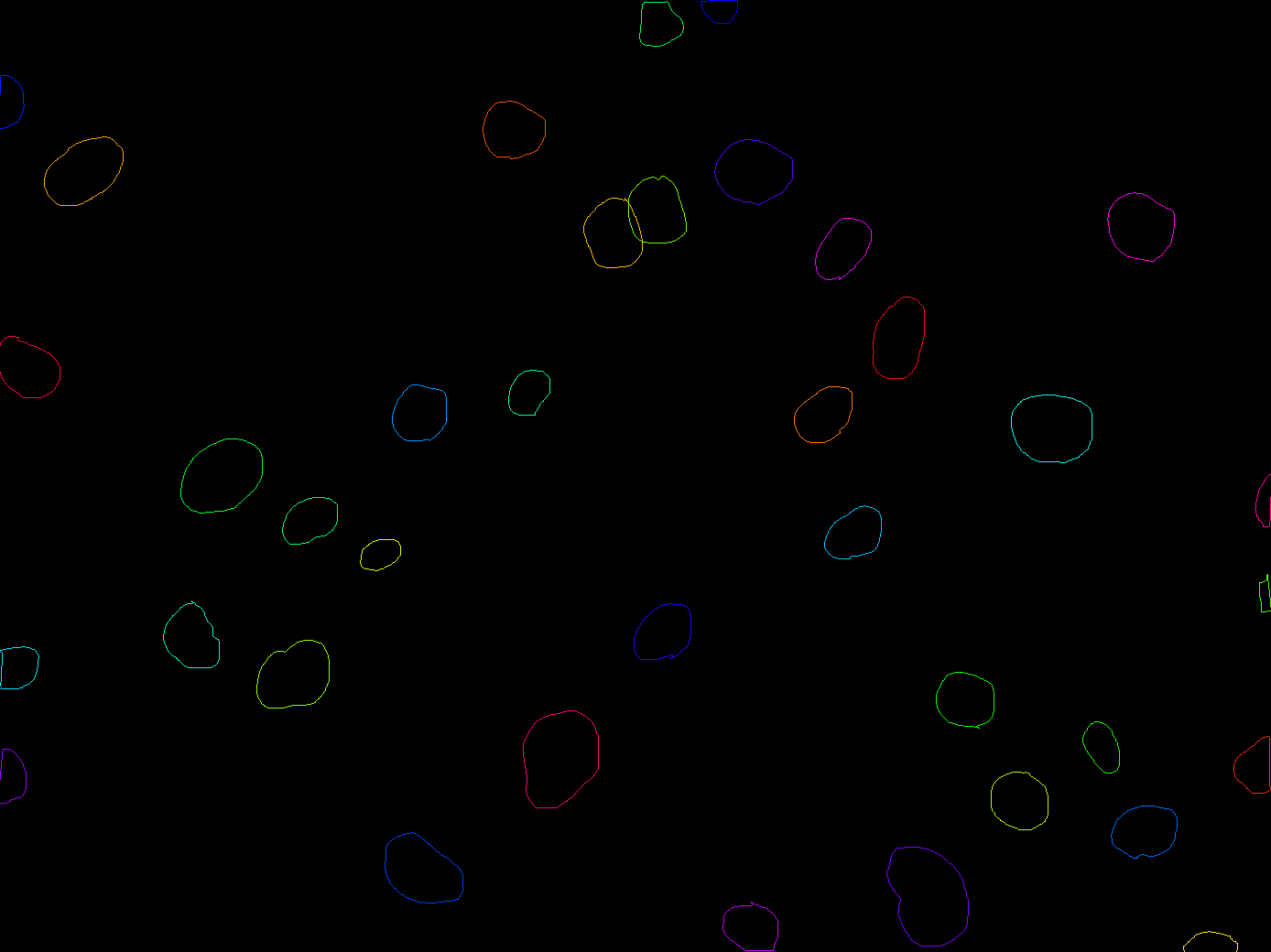

Supplement: Additional file 3 — The zip archive contains real images showing macrophages. (ZIP 28979 kb) [file 12859_2017_1591_MOESM3_ESM.zip › macrophages/jw-1h 4_c5 gt.png]

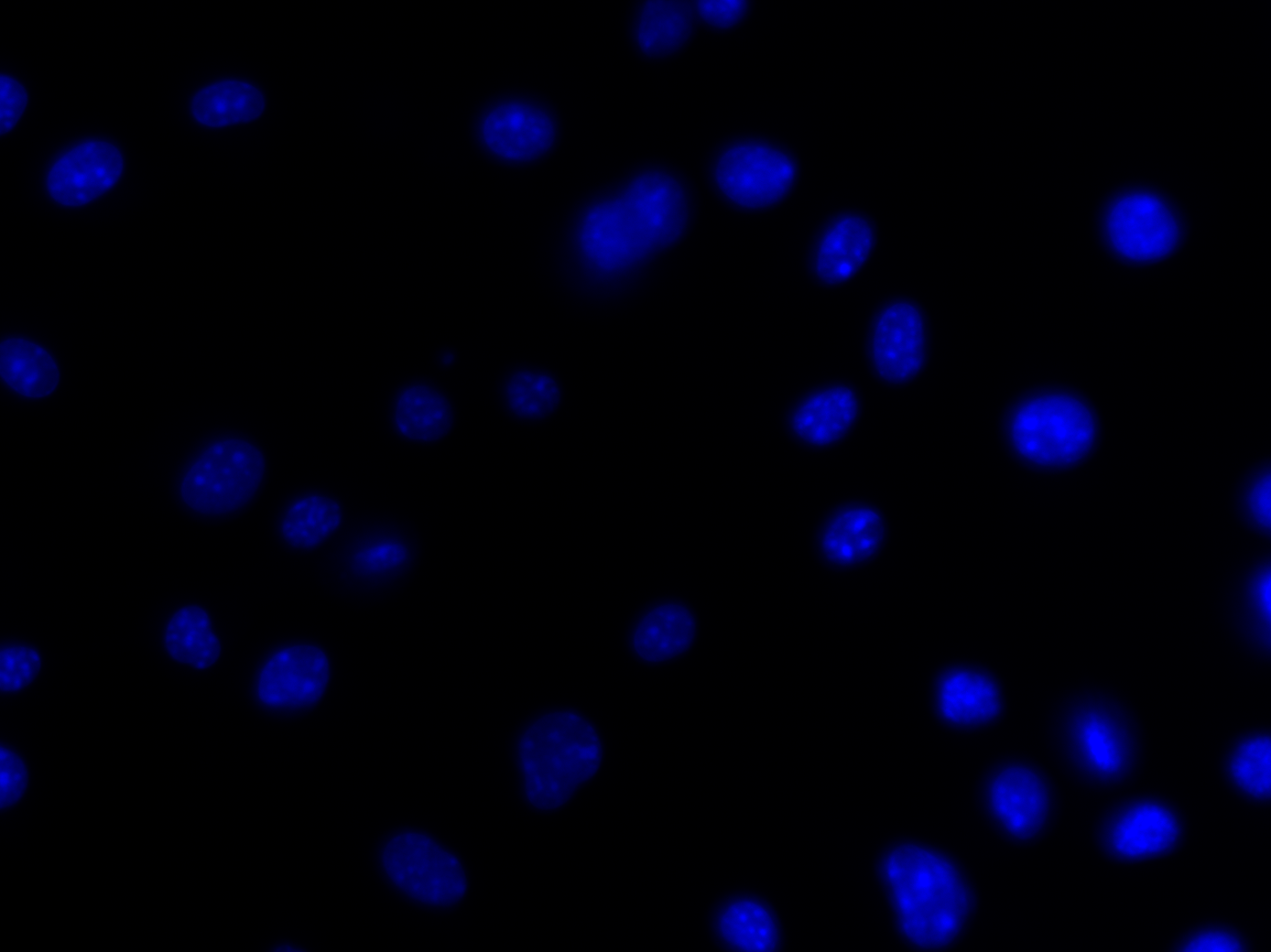

Supplement: Additional file 3 — The zip archive contains real images showing macrophages. (ZIP 28979 kb) [file 12859_2017_1591_MOESM3_ESM.zip › macrophages/jw-1h 4_c5.png]

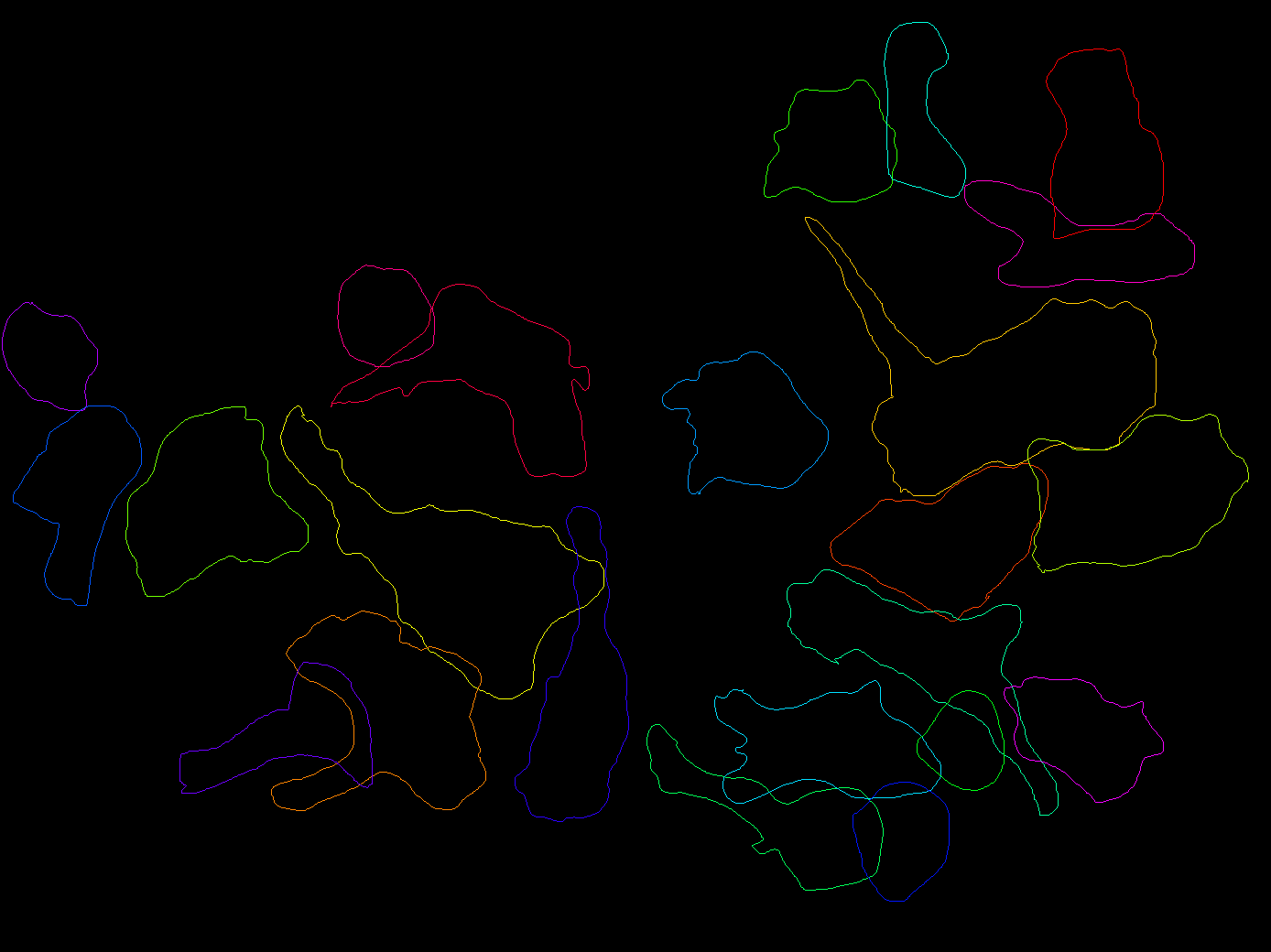

Supplement: Additional file 3 — The zip archive contains real images showing macrophages. (ZIP 28979 kb) [file 12859_2017_1591_MOESM3_ESM.zip › macrophages/jw-1h 5_c1 gt.png]

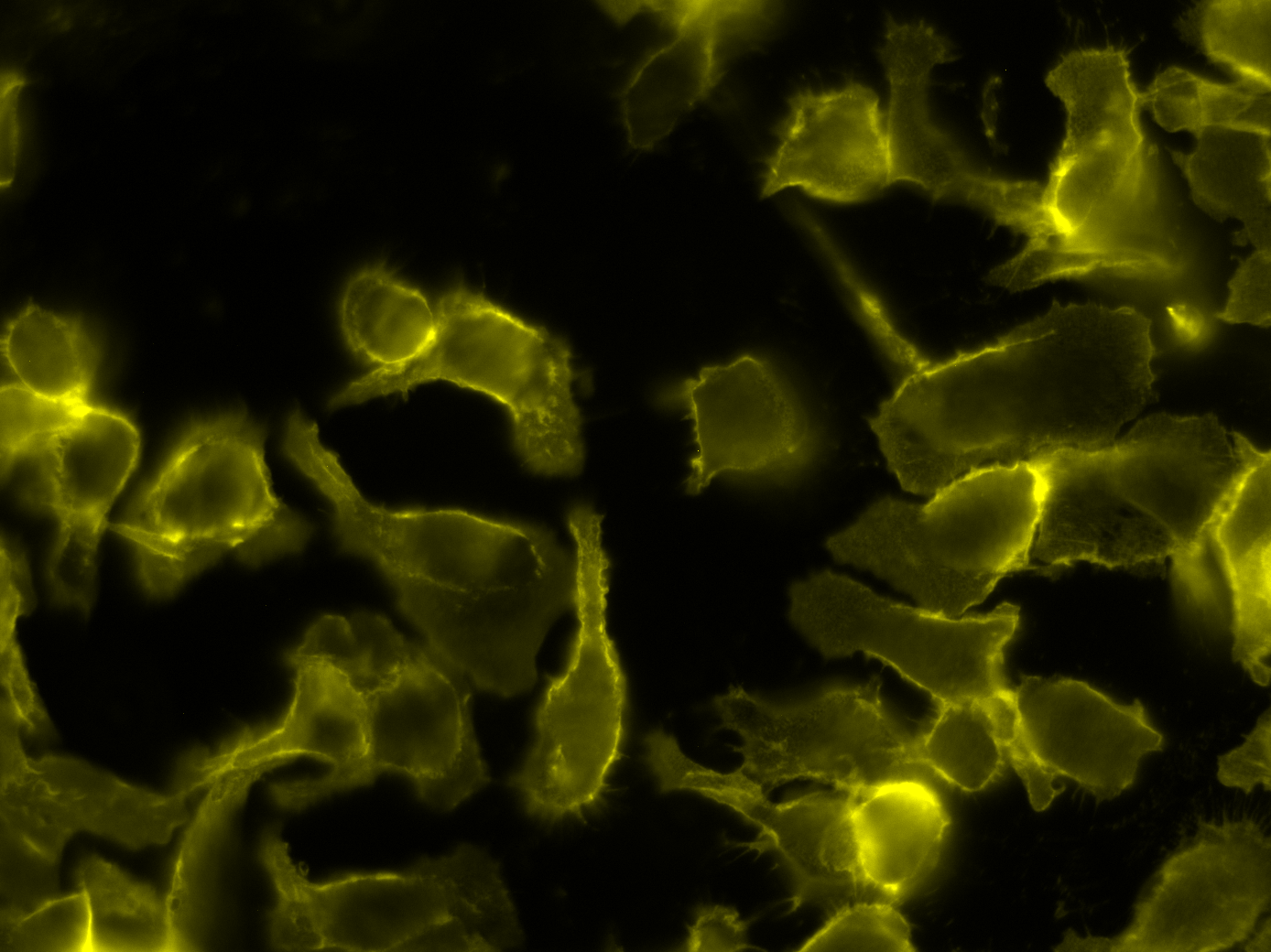

Supplement: Additional file 3 — The zip archive contains real images showing macrophages. (ZIP 28979 kb) [file 12859_2017_1591_MOESM3_ESM.zip › macrophages/jw-1h 5_c1.png]

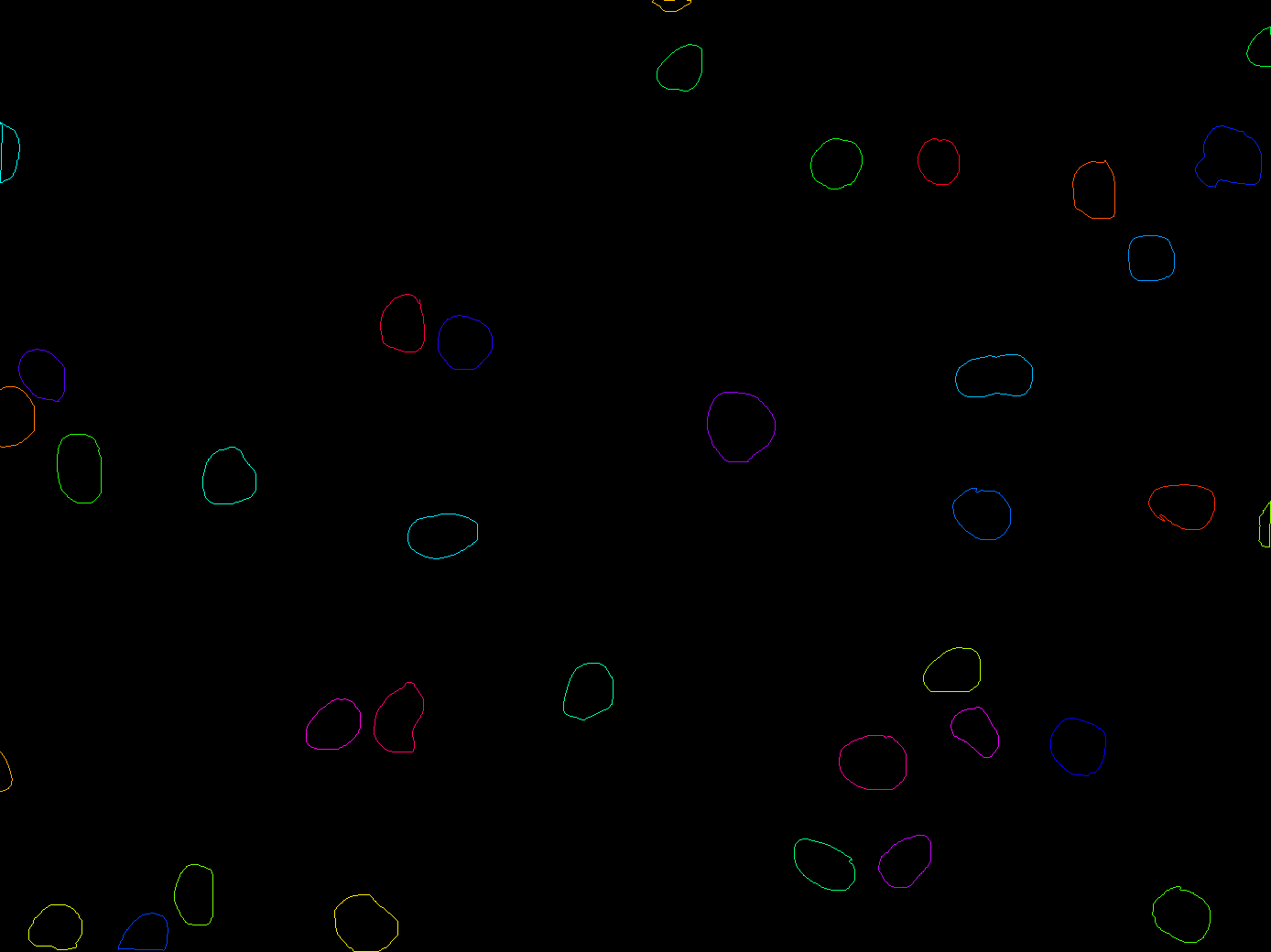

Supplement: Additional file 3 — The zip archive contains real images showing macrophages. (ZIP 28979 kb) [file 12859_2017_1591_MOESM3_ESM.zip › macrophages/jw-1h 5_c5 gt.png]

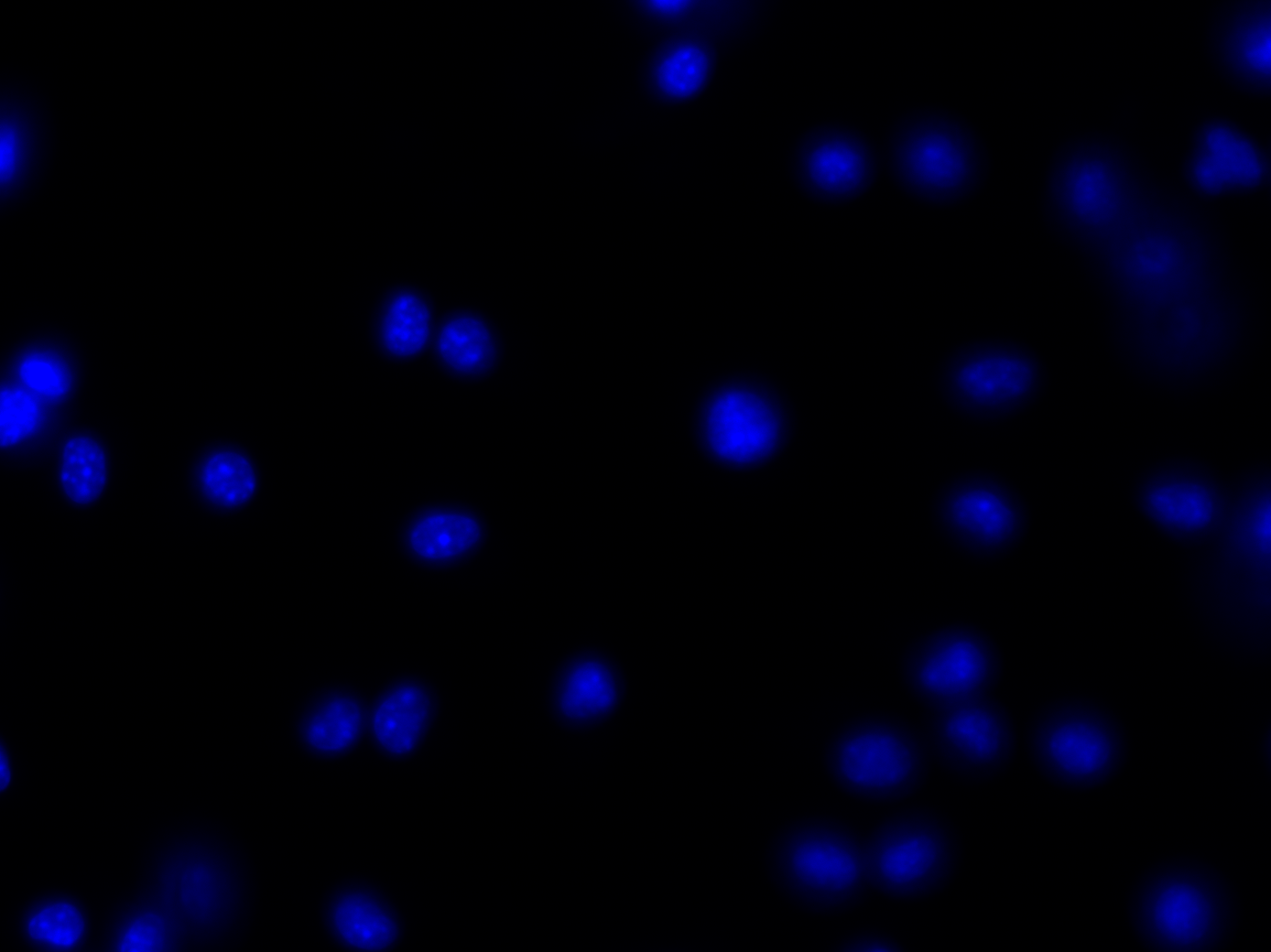

Supplement: Additional file 3 — The zip archive contains real images showing macrophages. (ZIP 28979 kb) [file 12859_2017_1591_MOESM3_ESM.zip › macrophages/jw-1h 5_c5.png]

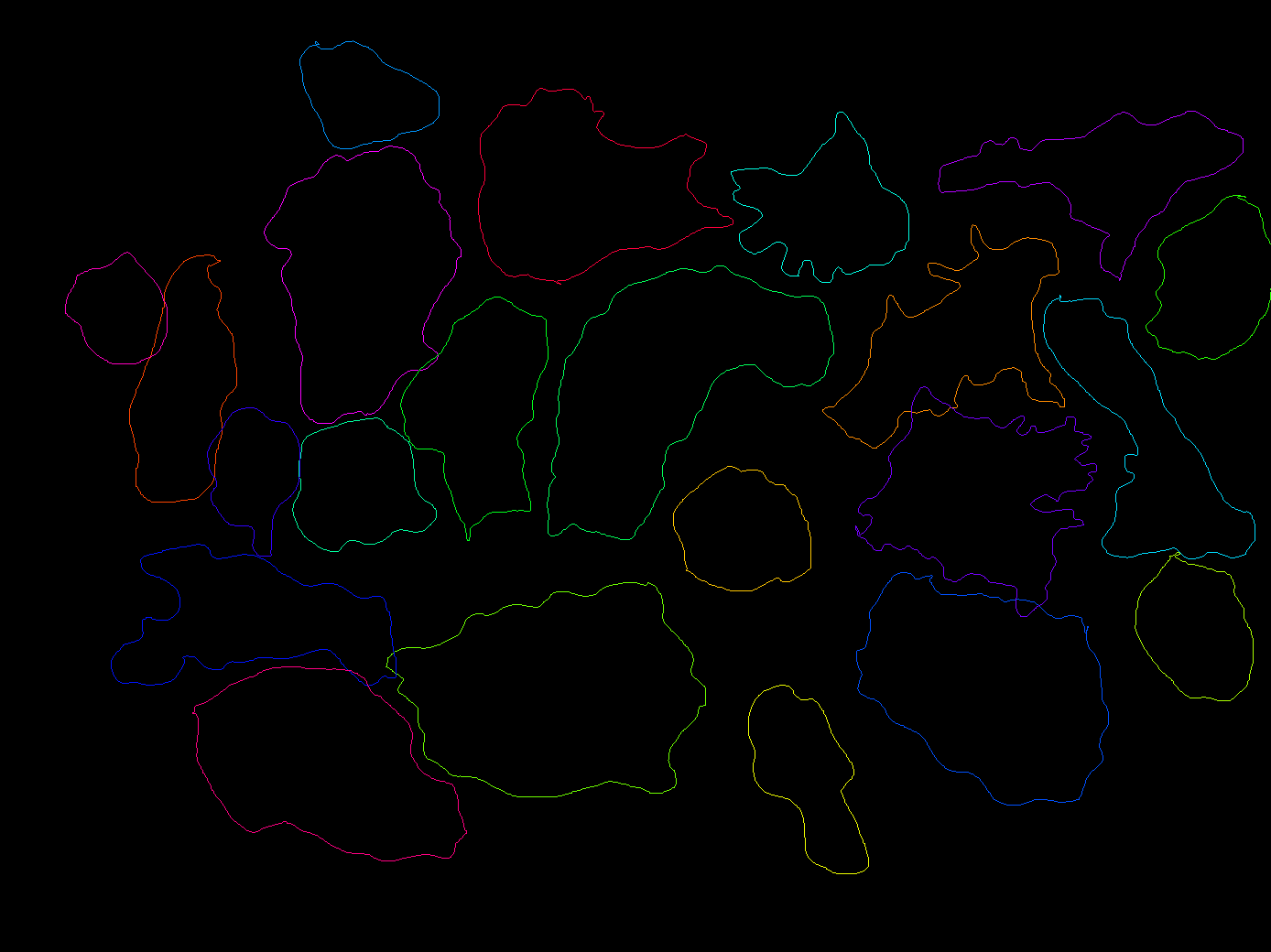

Supplement: Additional file 3 — The zip archive contains real images showing macrophages. (ZIP 28979 kb) [file 12859_2017_1591_MOESM3_ESM.zip › macrophages/jw-24h 1_c1 gt.png]

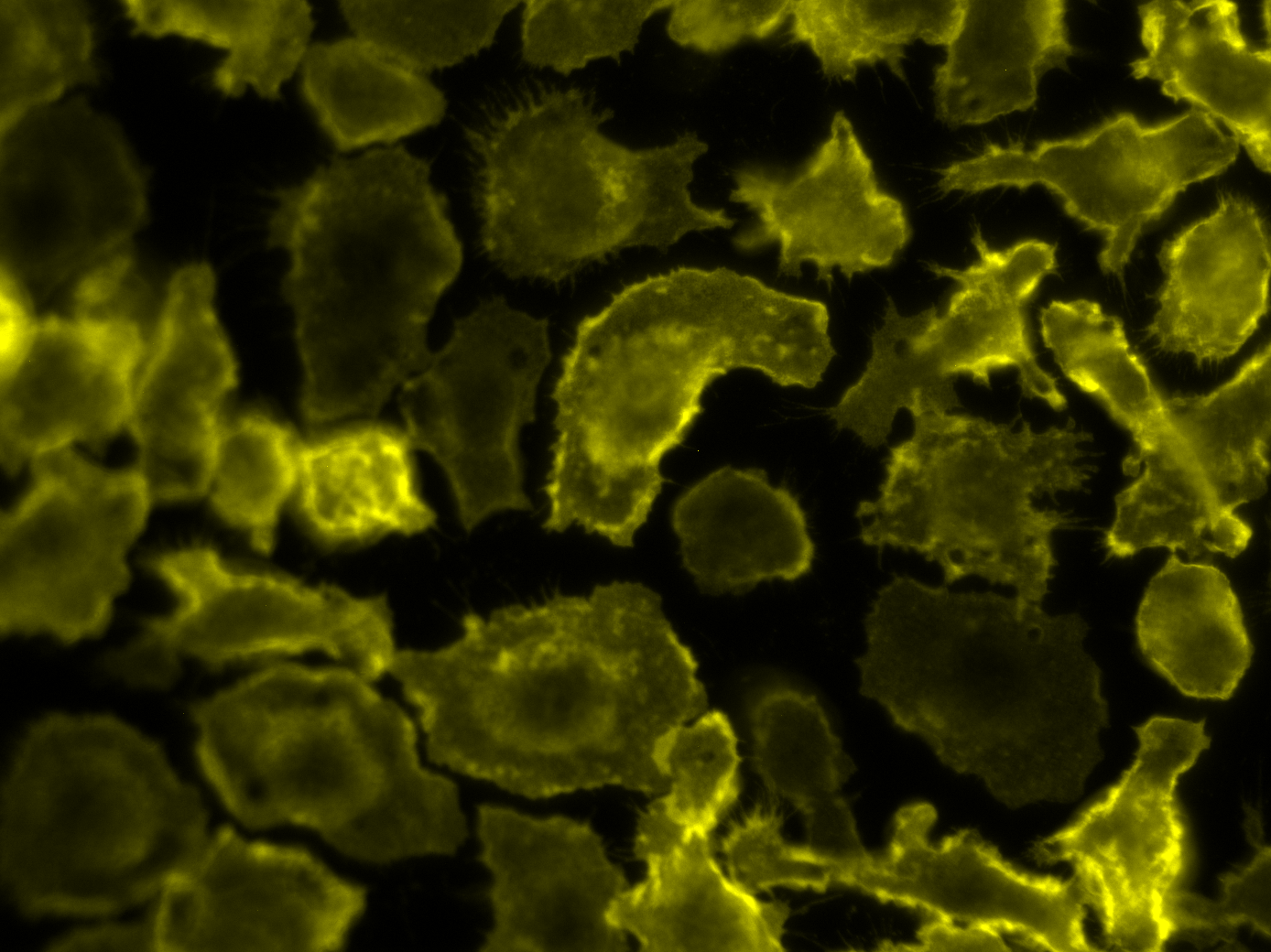

Supplement: Additional file 3 — The zip archive contains real images showing macrophages. (ZIP 28979 kb) [file 12859_2017_1591_MOESM3_ESM.zip › macrophages/jw-24h 1_c1.png]

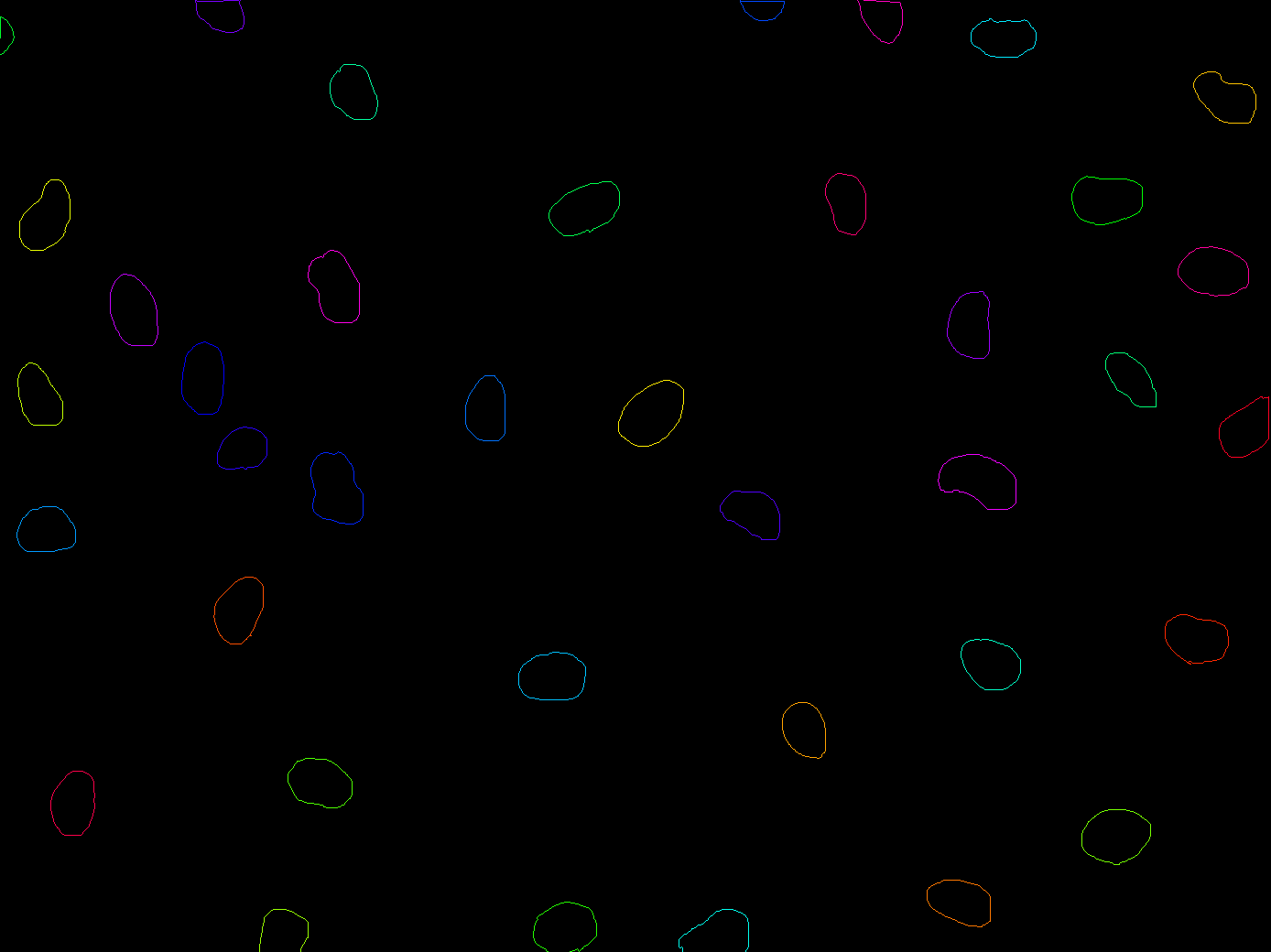

Supplement: Additional file 3 — The zip archive contains real images showing macrophages. (ZIP 28979 kb) [file 12859_2017_1591_MOESM3_ESM.zip › macrophages/jw-24h 1_c5 gt.png]

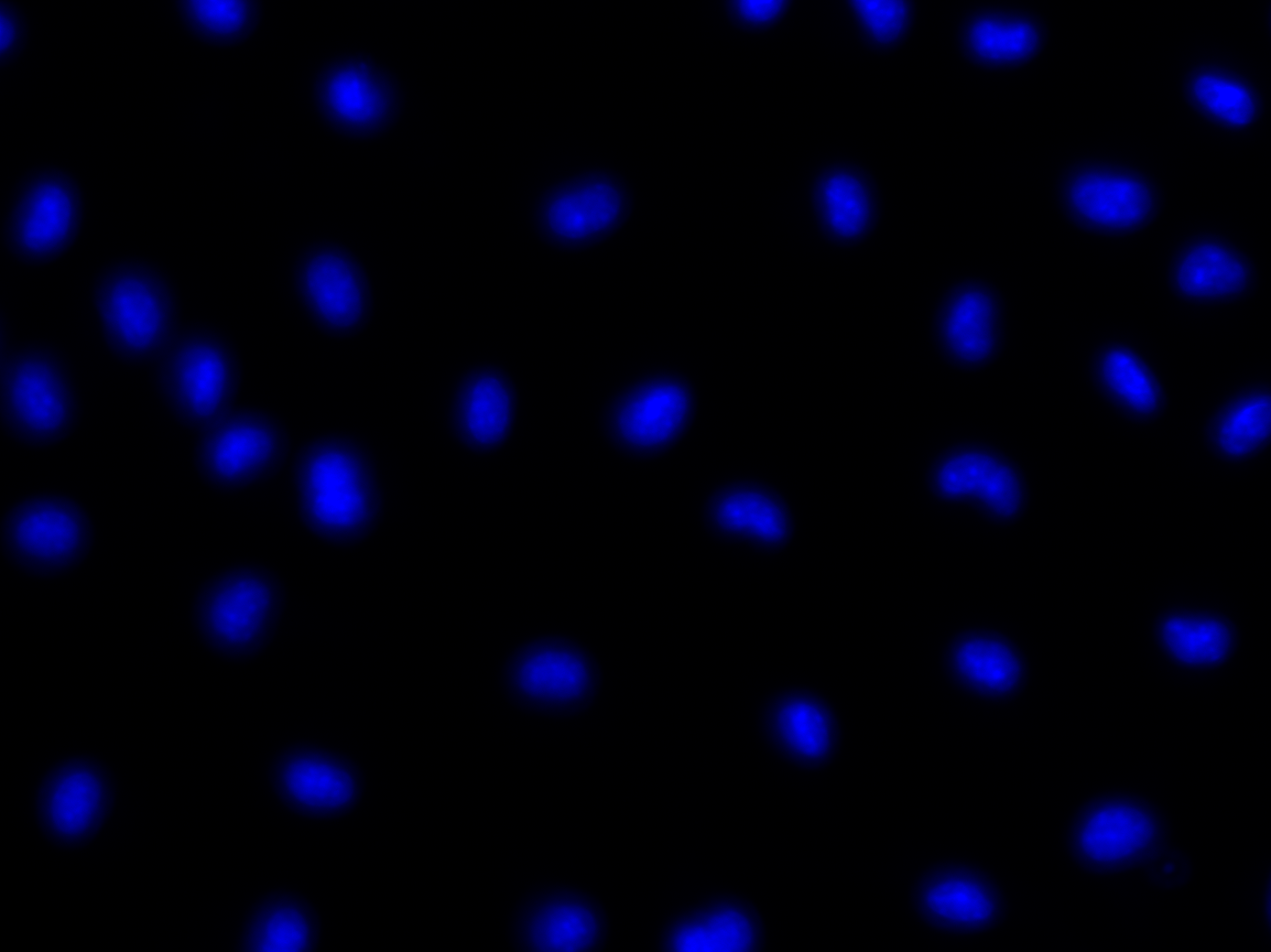

Supplement: Additional file 3 — The zip archive contains real images showing macrophages. (ZIP 28979 kb) [file 12859_2017_1591_MOESM3_ESM.zip › macrophages/jw-24h 1_c5.png]

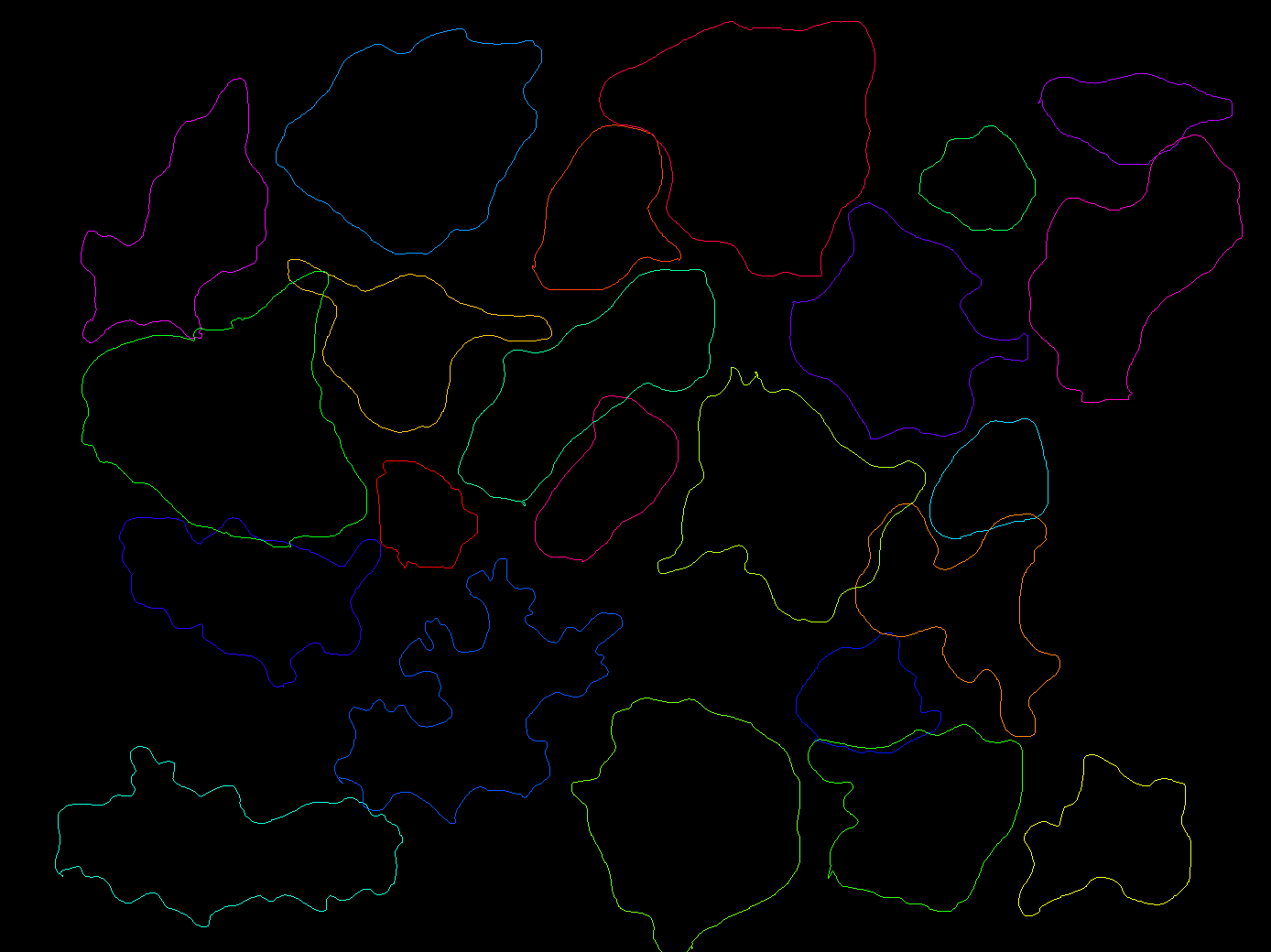

Supplement: Additional file 3 — The zip archive contains real images showing macrophages. (ZIP 28979 kb) [file 12859_2017_1591_MOESM3_ESM.zip › macrophages/jw-24h 2_c1 gt.png]

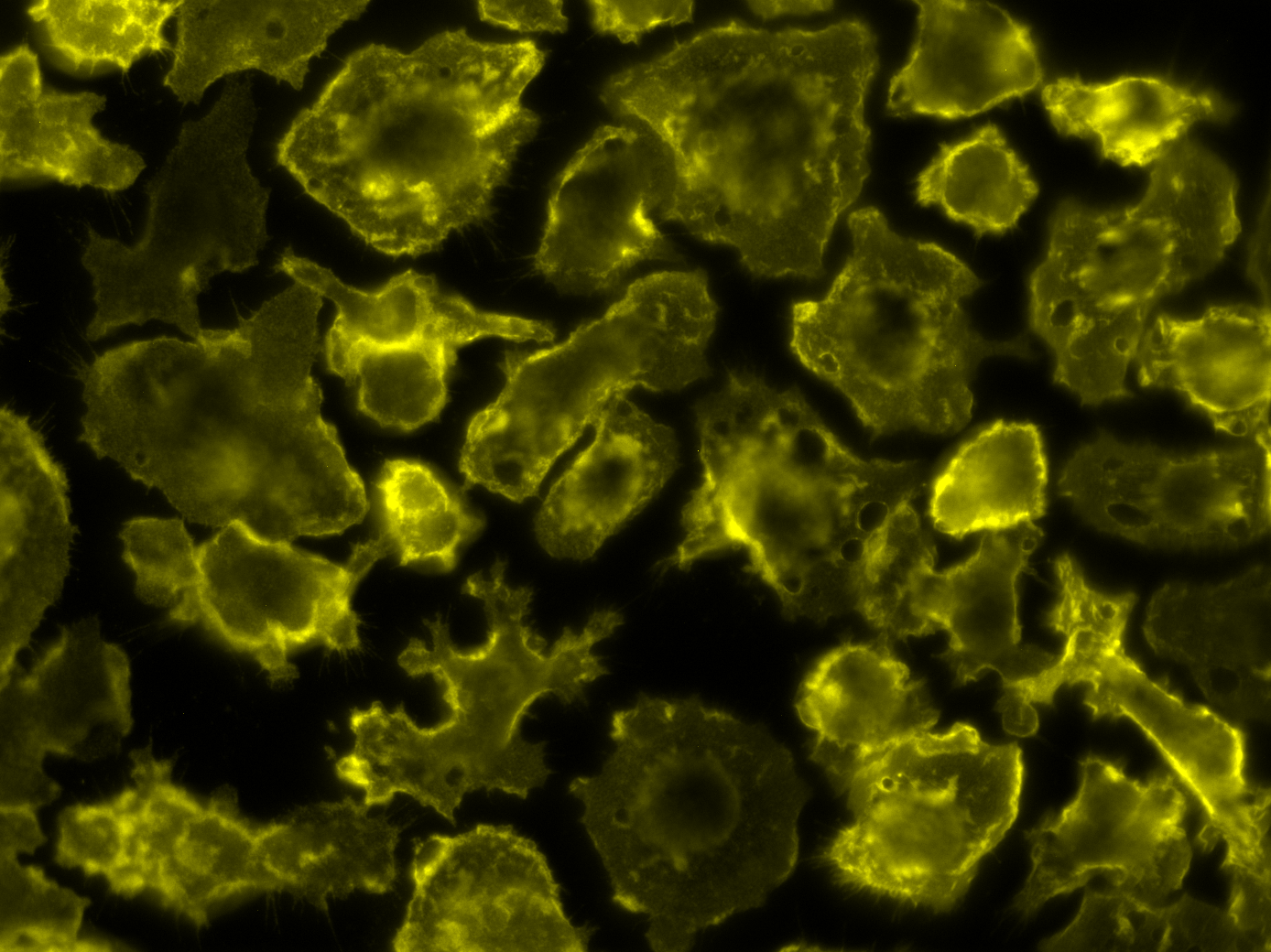

Supplement: Additional file 3 — The zip archive contains real images showing macrophages. (ZIP 28979 kb) [file 12859_2017_1591_MOESM3_ESM.zip › macrophages/jw-24h 2_c1.png]

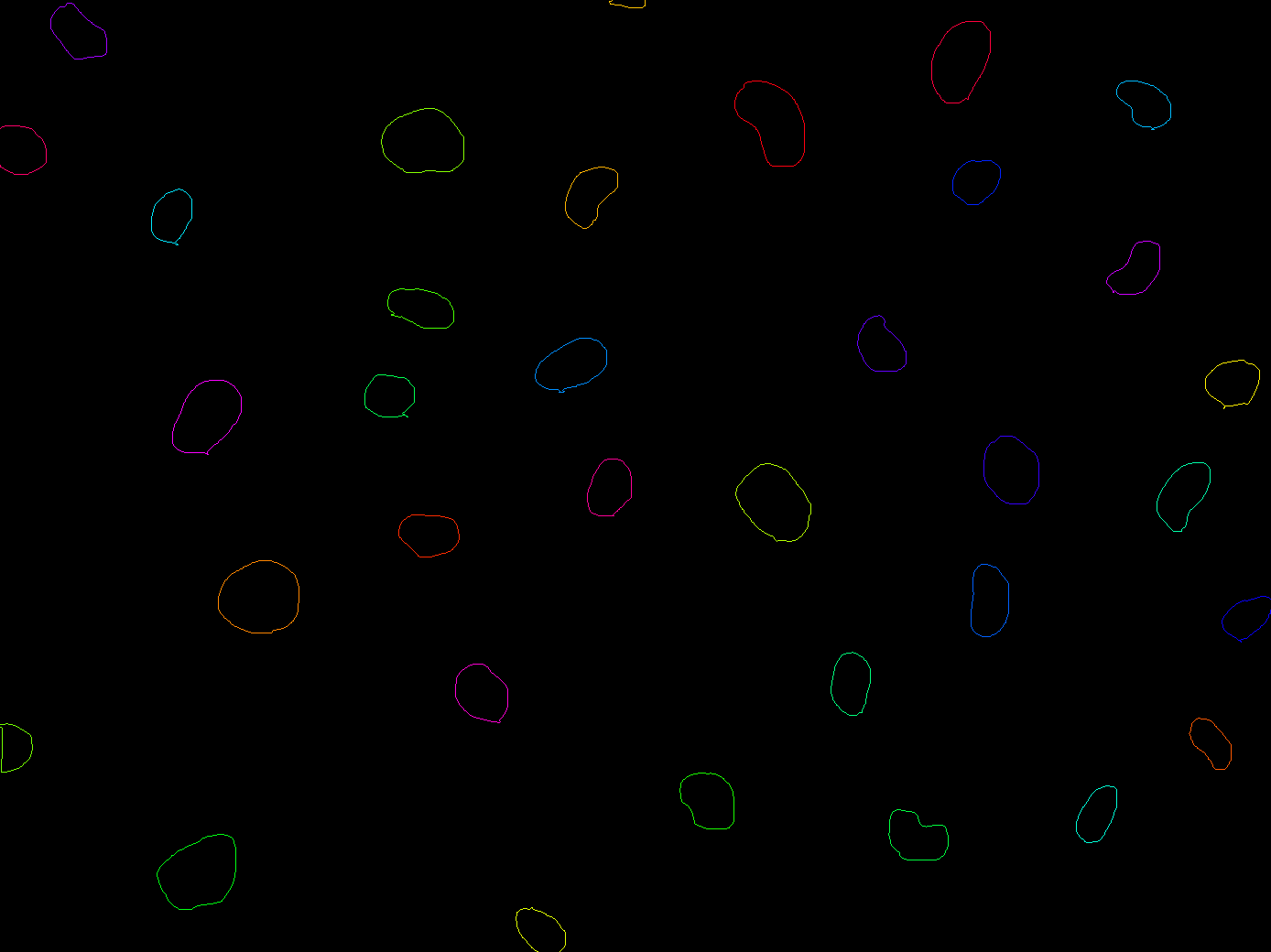

Supplement: Additional file 3 — The zip archive contains real images showing macrophages. (ZIP 28979 kb) [file 12859_2017_1591_MOESM3_ESM.zip › macrophages/jw-24h 2_c5 gt.png]

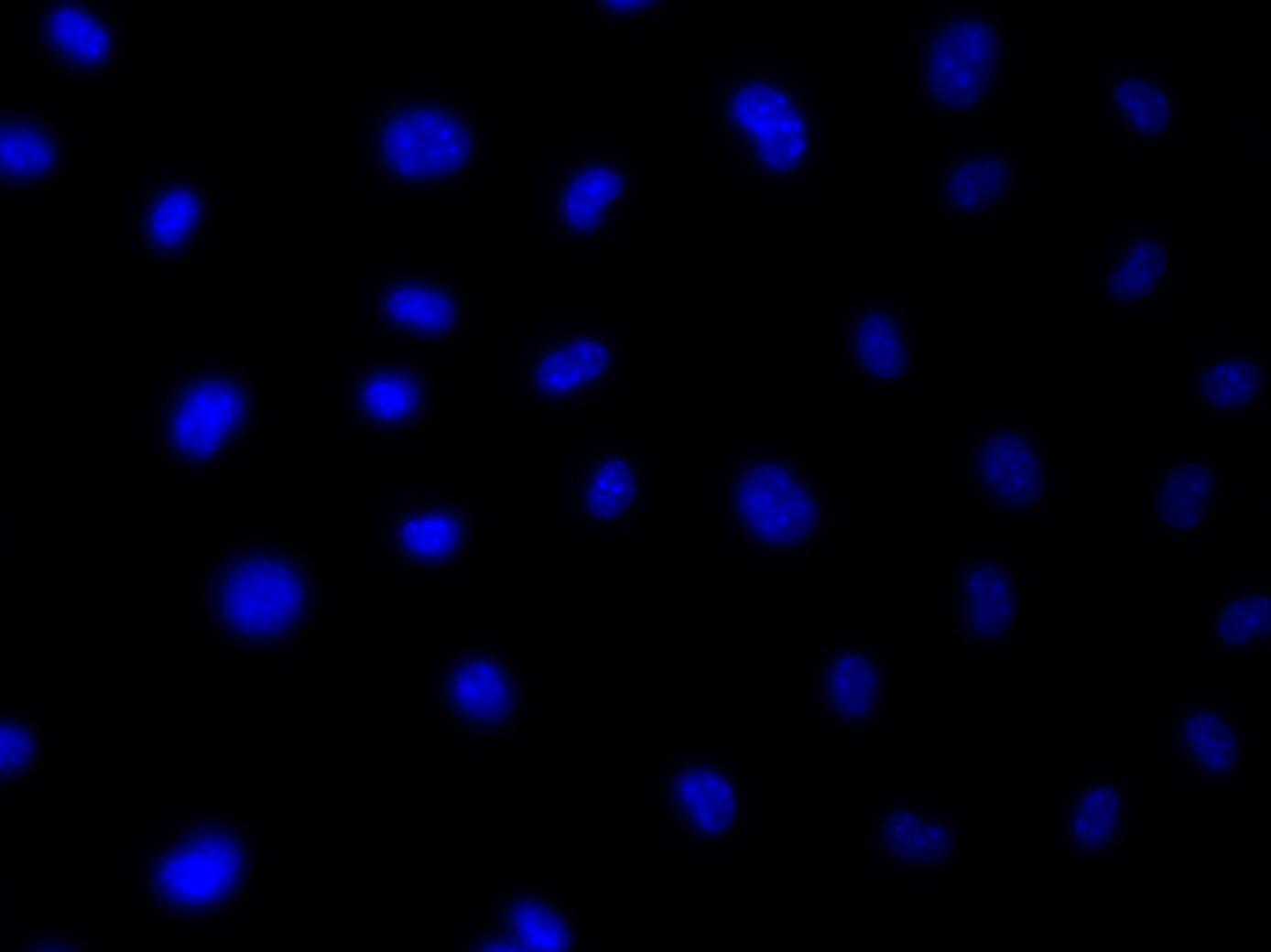

Supplement: Additional file 3 — The zip archive contains real images showing macrophages. (ZIP 28979 kb) [file 12859_2017_1591_MOESM3_ESM.zip › macrophages/jw-24h 2_c5.png]

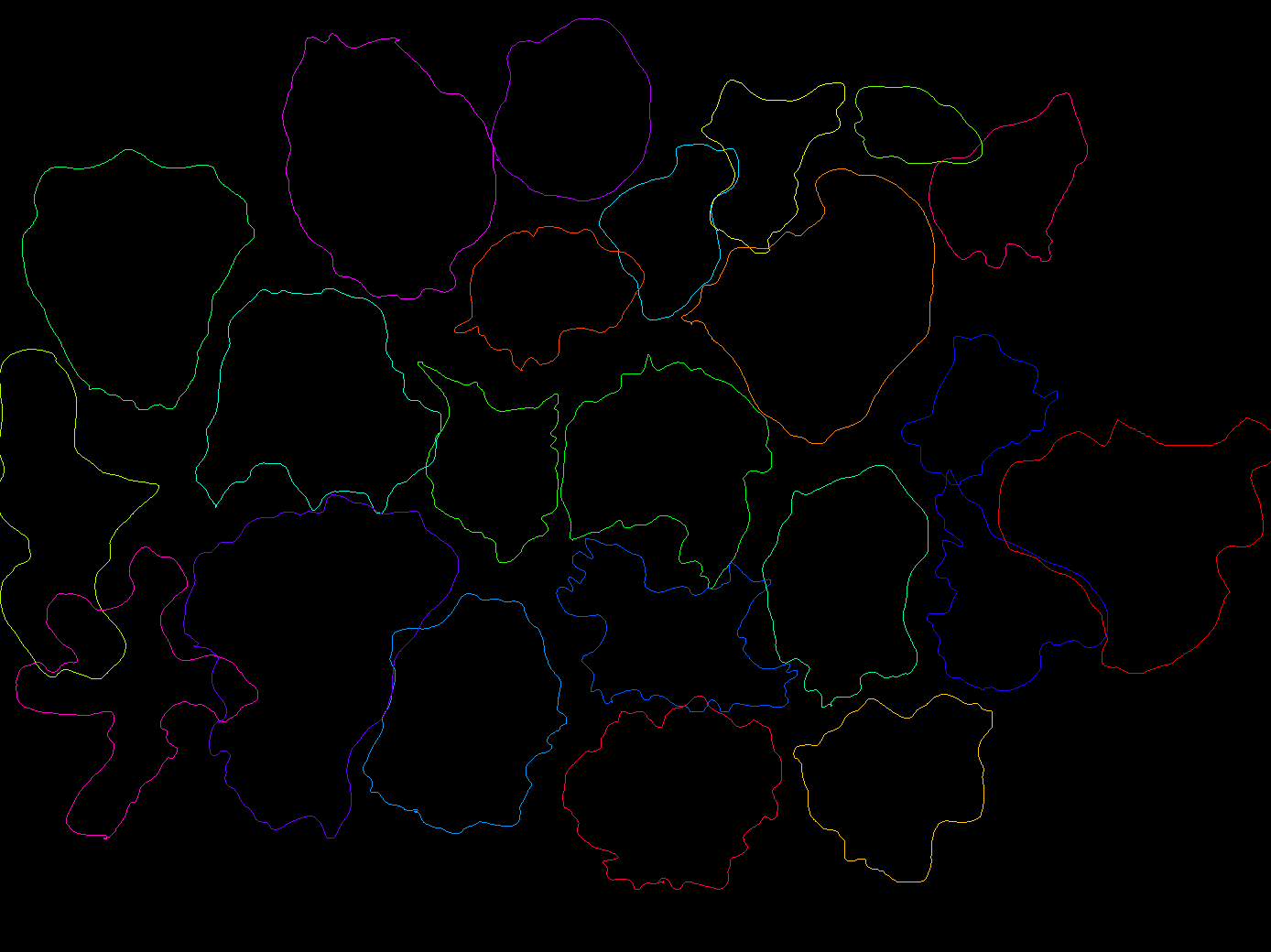

Supplement: Additional file 3 — The zip archive contains real images showing macrophages. (ZIP 28979 kb) [file 12859_2017_1591_MOESM3_ESM.zip › macrophages/jw-24h 3_c1 gt.png]

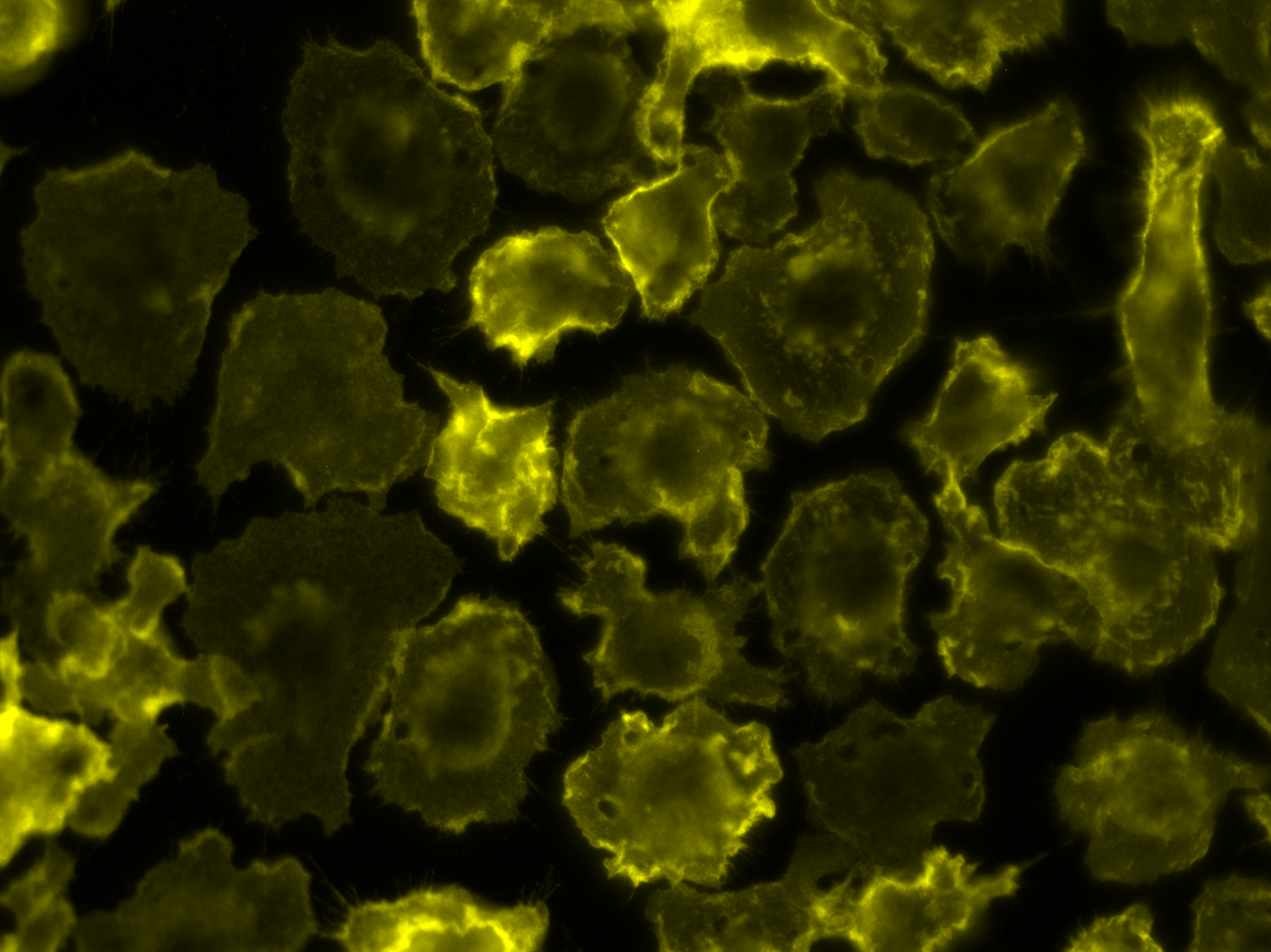

Supplement: Additional file 3 — The zip archive contains real images showing macrophages. (ZIP 28979 kb) [file 12859_2017_1591_MOESM3_ESM.zip › macrophages/jw-24h 3_c1.png]

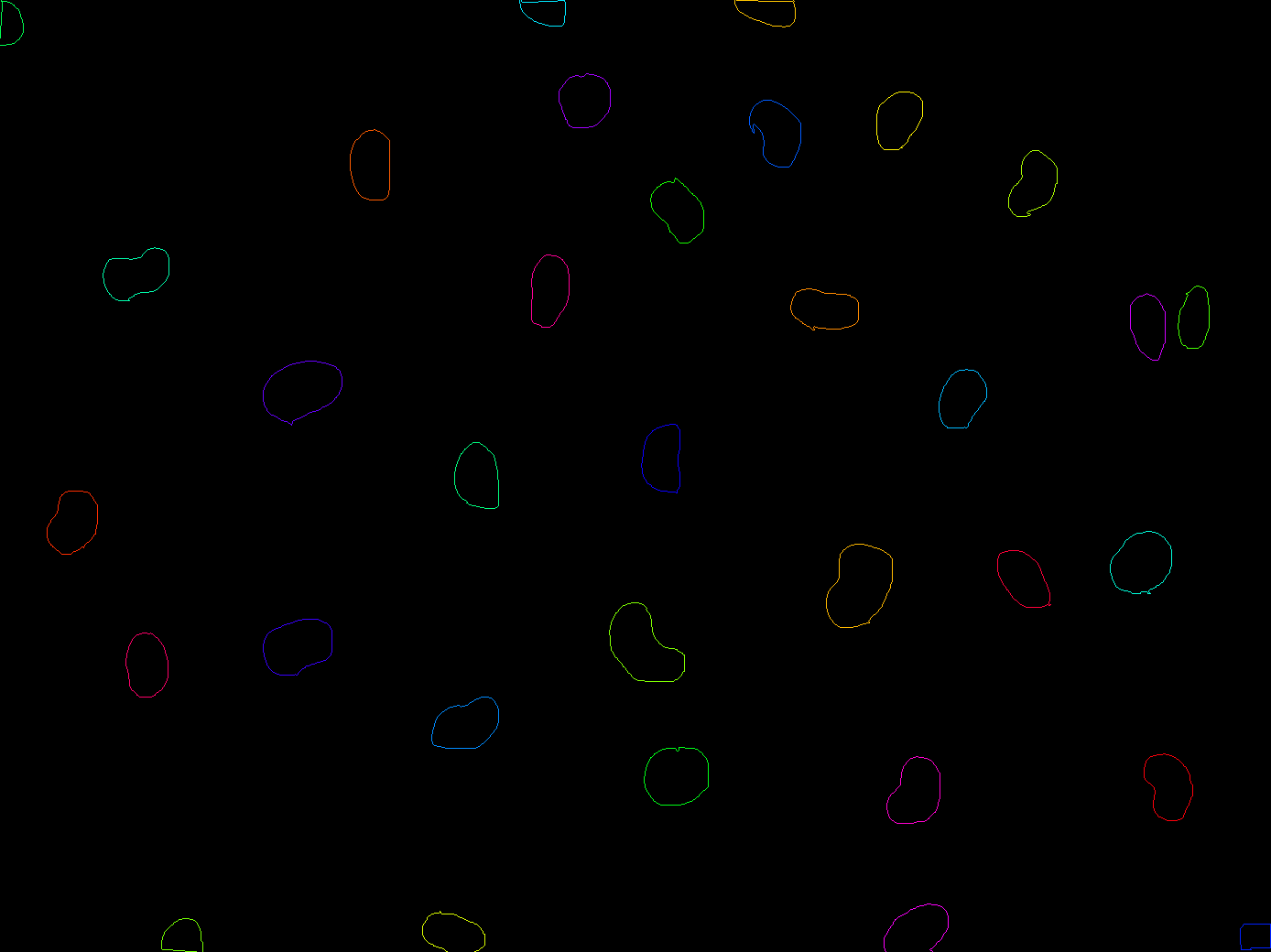

Supplement: Additional file 3 — The zip archive contains real images showing macrophages. (ZIP 28979 kb) [file 12859_2017_1591_MOESM3_ESM.zip › macrophages/jw-24h 3_c5 gt.png]

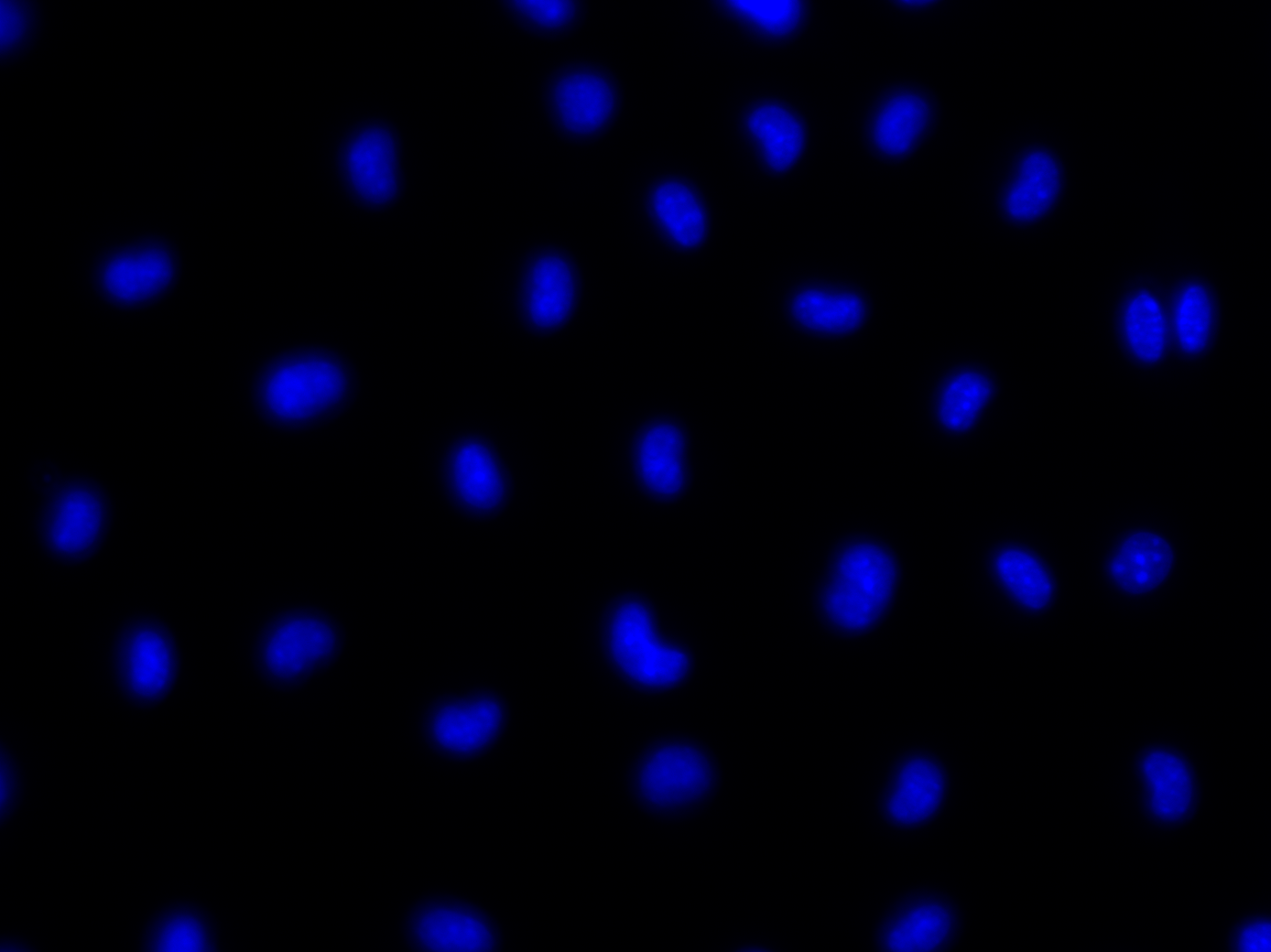

Supplement: Additional file 3 — The zip archive contains real images showing macrophages. (ZIP 28979 kb) [file 12859_2017_1591_MOESM3_ESM.zip › macrophages/jw-24h 3_c5.png]

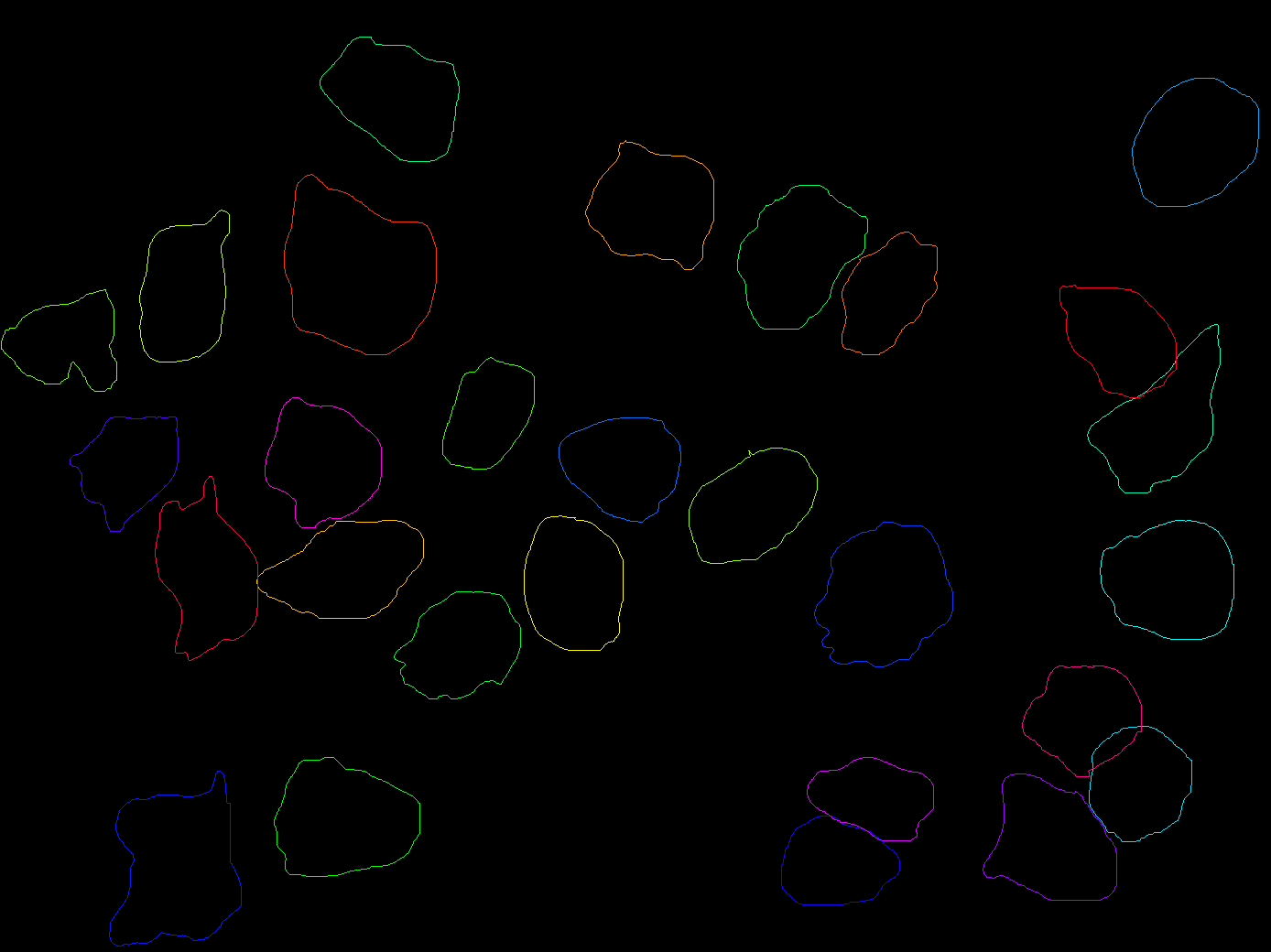

Supplement: Additional file 3 — The zip archive contains real images showing macrophages. (ZIP 28979 kb) [file 12859_2017_1591_MOESM3_ESM.zip › macrophages/jw-24h 4_c1 gt.png]

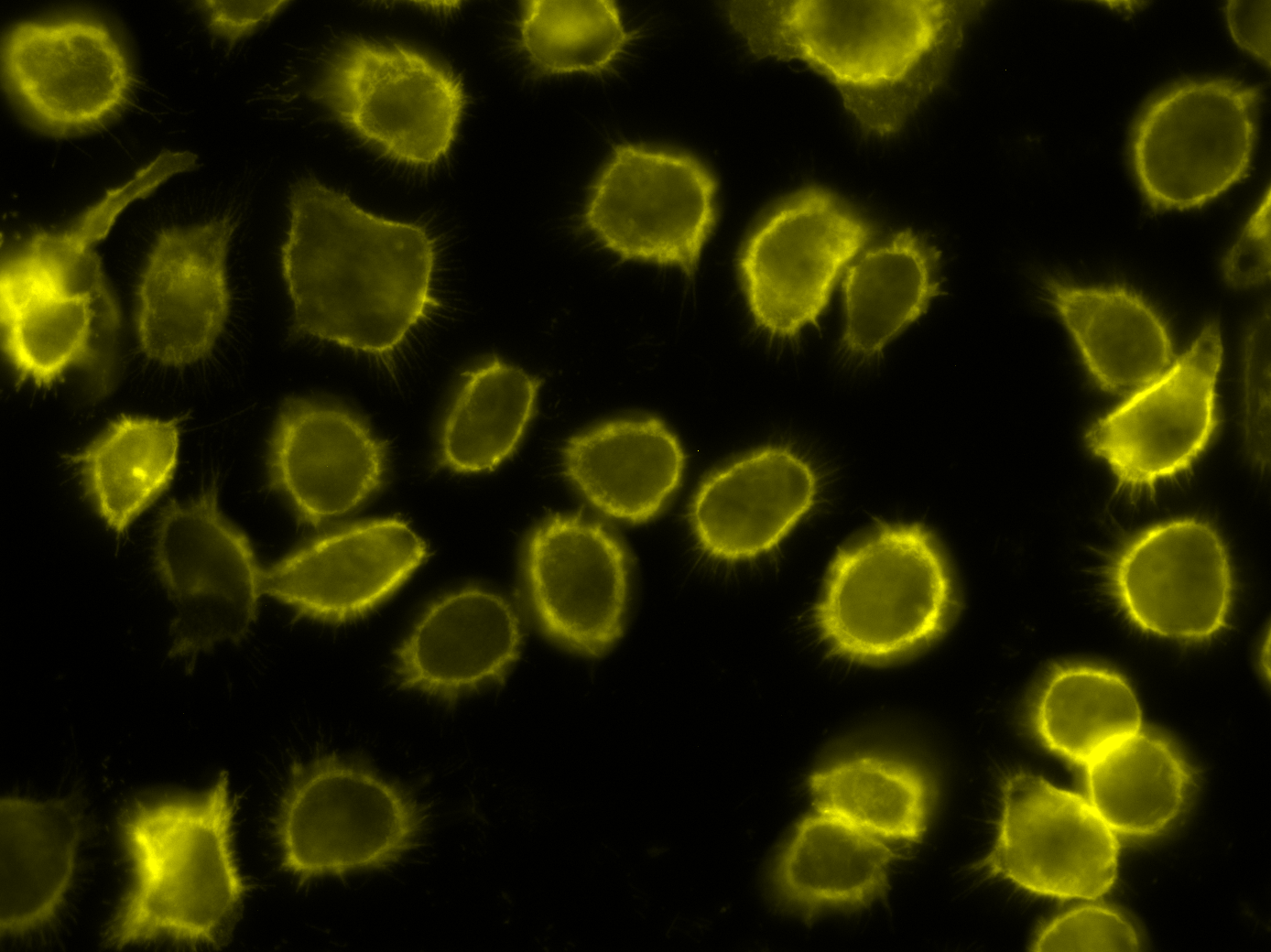

Supplement: Additional file 3 — The zip archive contains real images showing macrophages. (ZIP 28979 kb) [file 12859_2017_1591_MOESM3_ESM.zip › macrophages/jw-24h 4_c1.png]

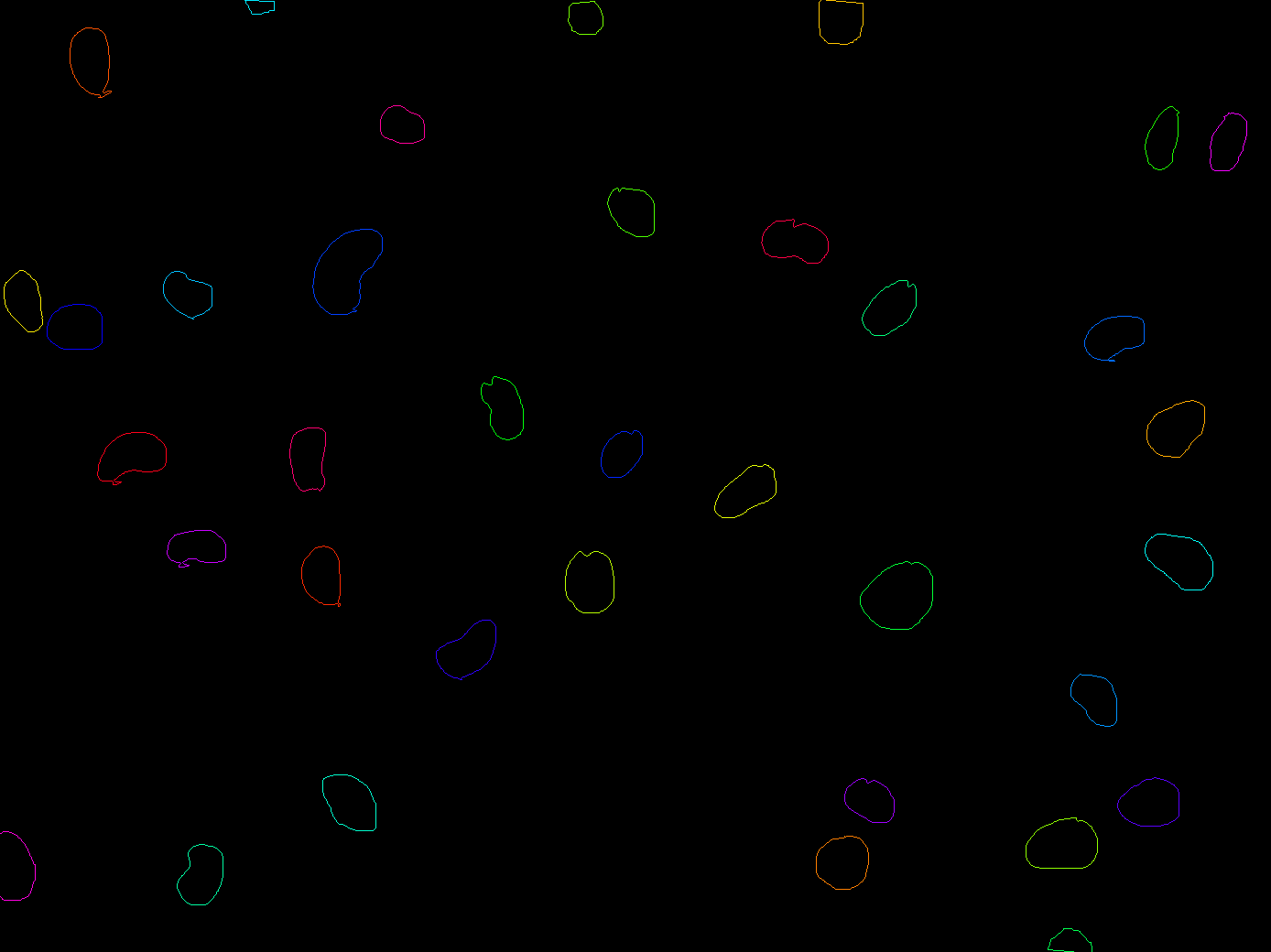

Supplement: Additional file 3 — The zip archive contains real images showing macrophages. (ZIP 28979 kb) [file 12859_2017_1591_MOESM3_ESM.zip › macrophages/jw-24h 4_c5 gt.png]

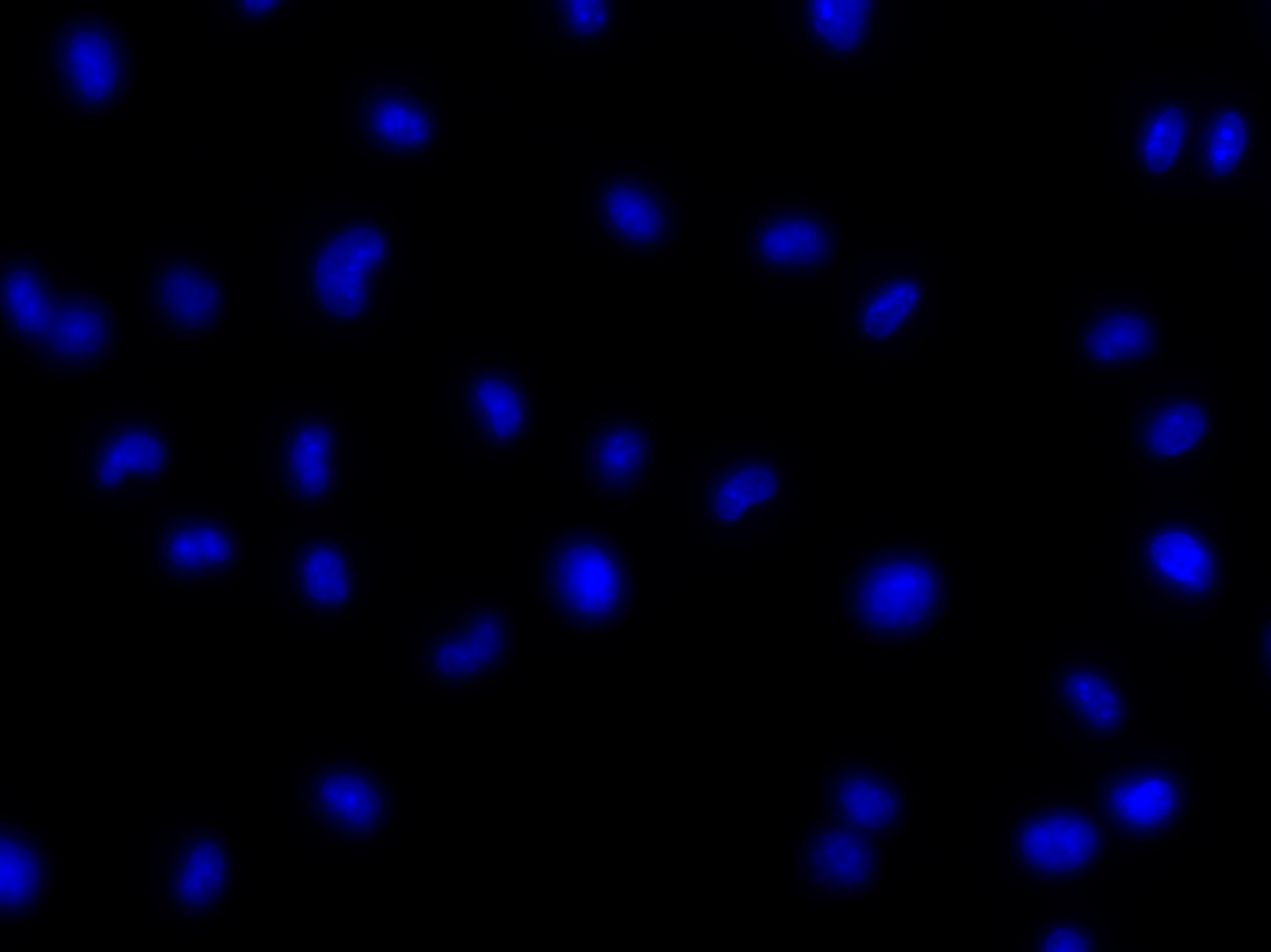

Supplement: Additional file 3 — The zip archive contains real images showing macrophages. (ZIP 28979 kb) [file 12859_2017_1591_MOESM3_ESM.zip › macrophages/jw-24h 4_c5.png]

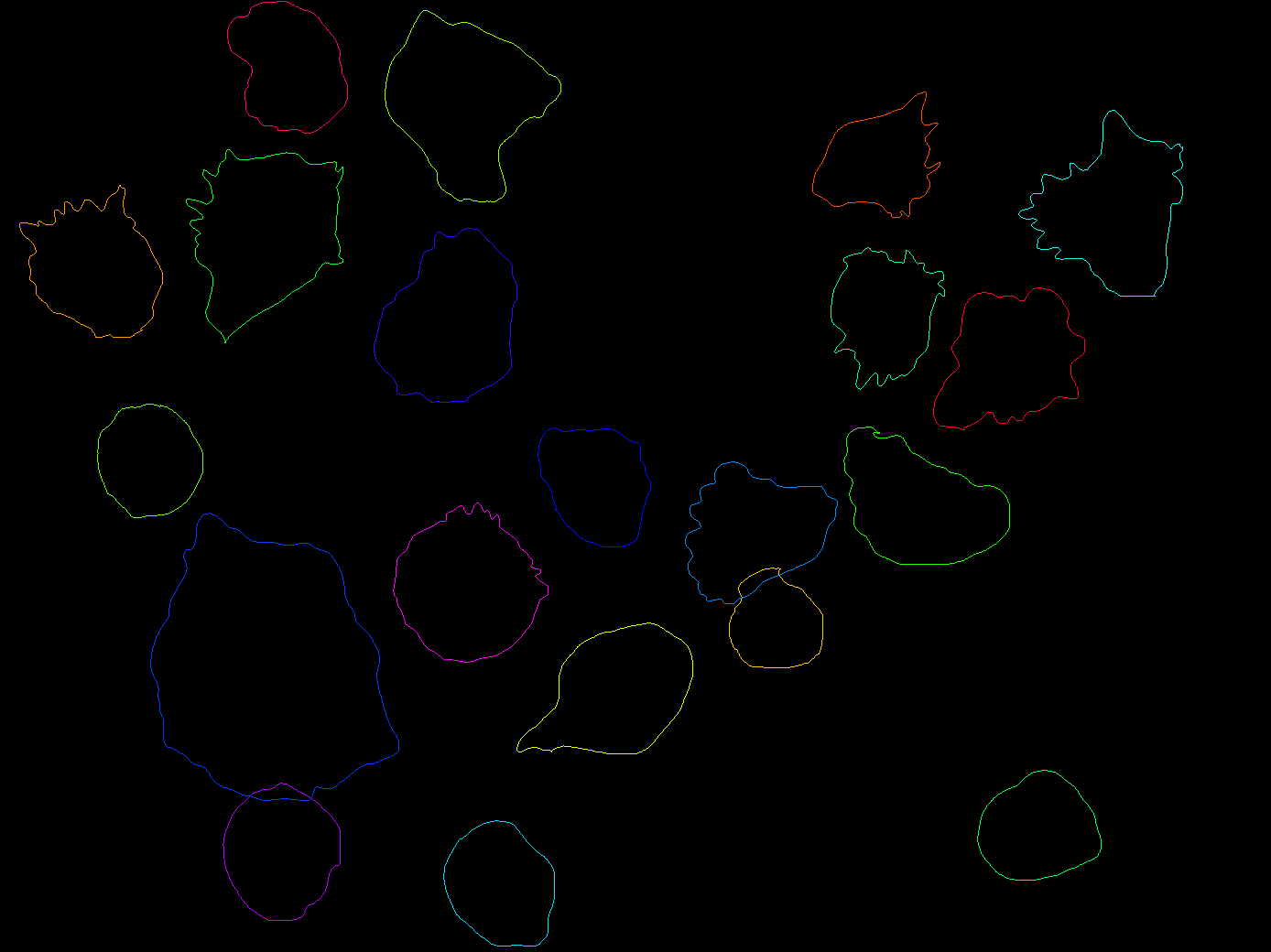

Supplement: Additional file 3 — The zip archive contains real images showing macrophages. (ZIP 28979 kb) [file 12859_2017_1591_MOESM3_ESM.zip › macrophages/jw-24h 5_c1 gt.png]

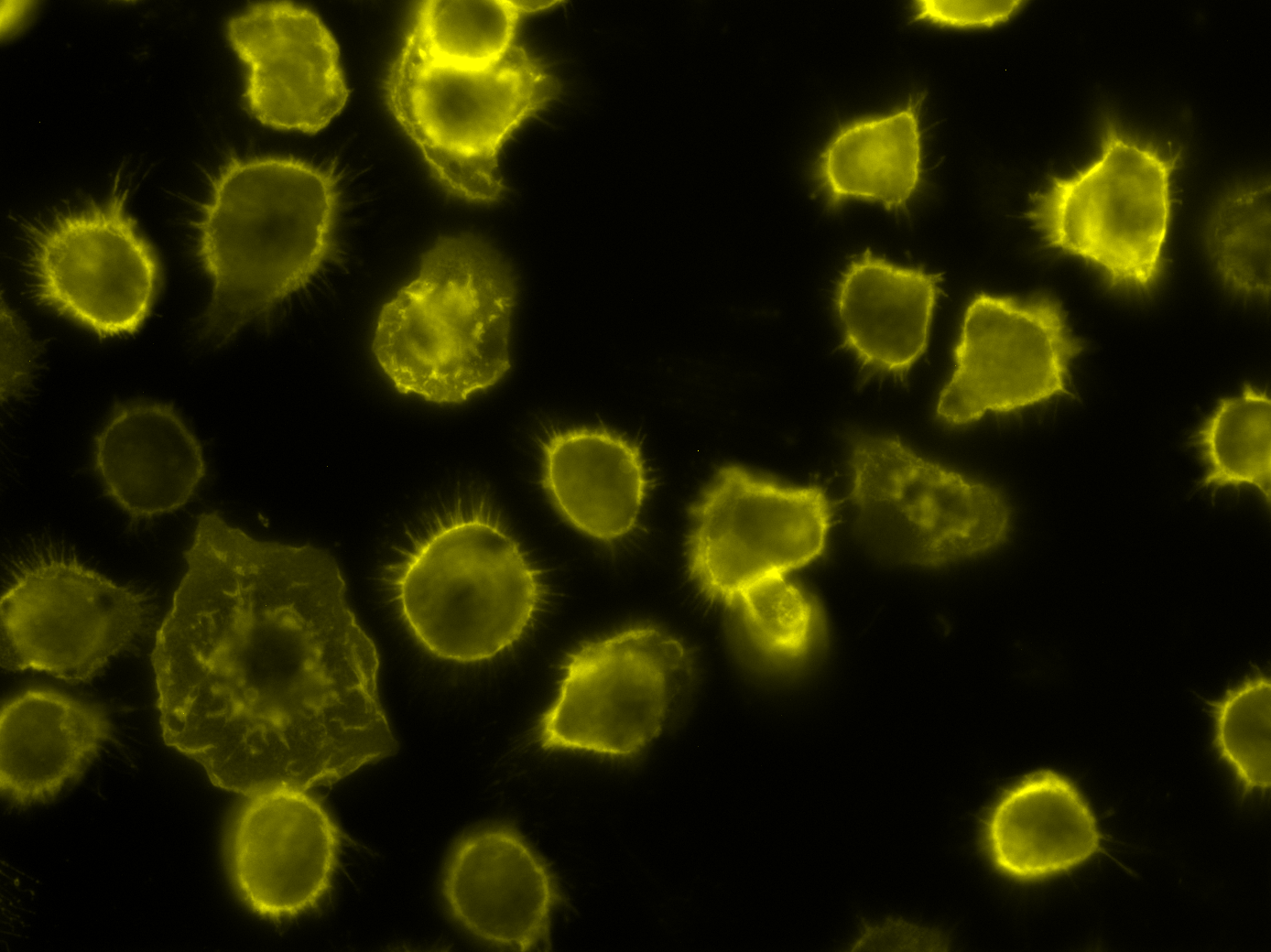

Supplement: Additional file 3 — The zip archive contains real images showing macrophages. (ZIP 28979 kb) [file 12859_2017_1591_MOESM3_ESM.zip › macrophages/jw-24h 5_c1.png]

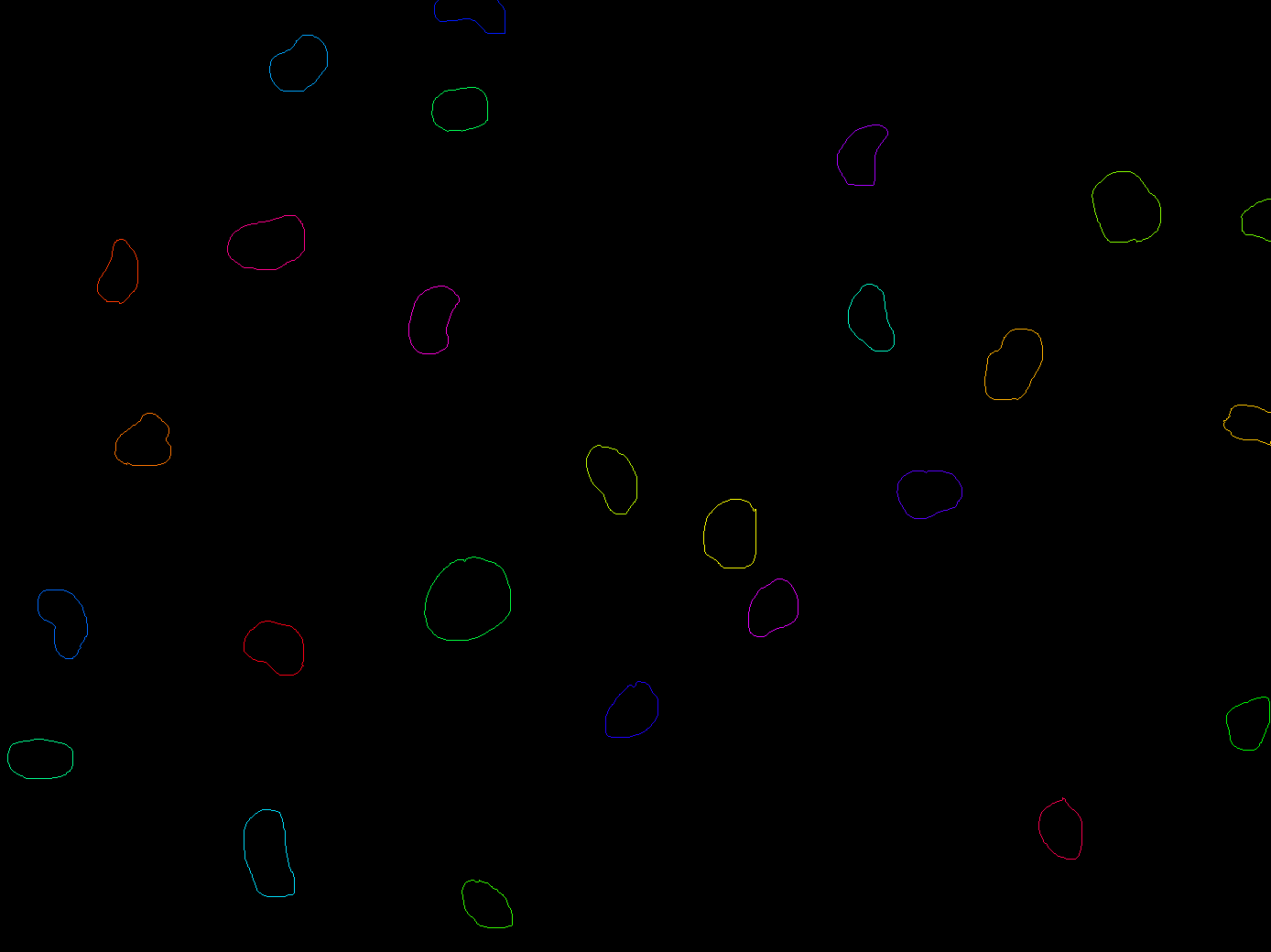

Supplement: Additional file 3 — The zip archive contains real images showing macrophages. (ZIP 28979 kb) [file 12859_2017_1591_MOESM3_ESM.zip › macrophages/jw-24h 5_c5 gt.png]

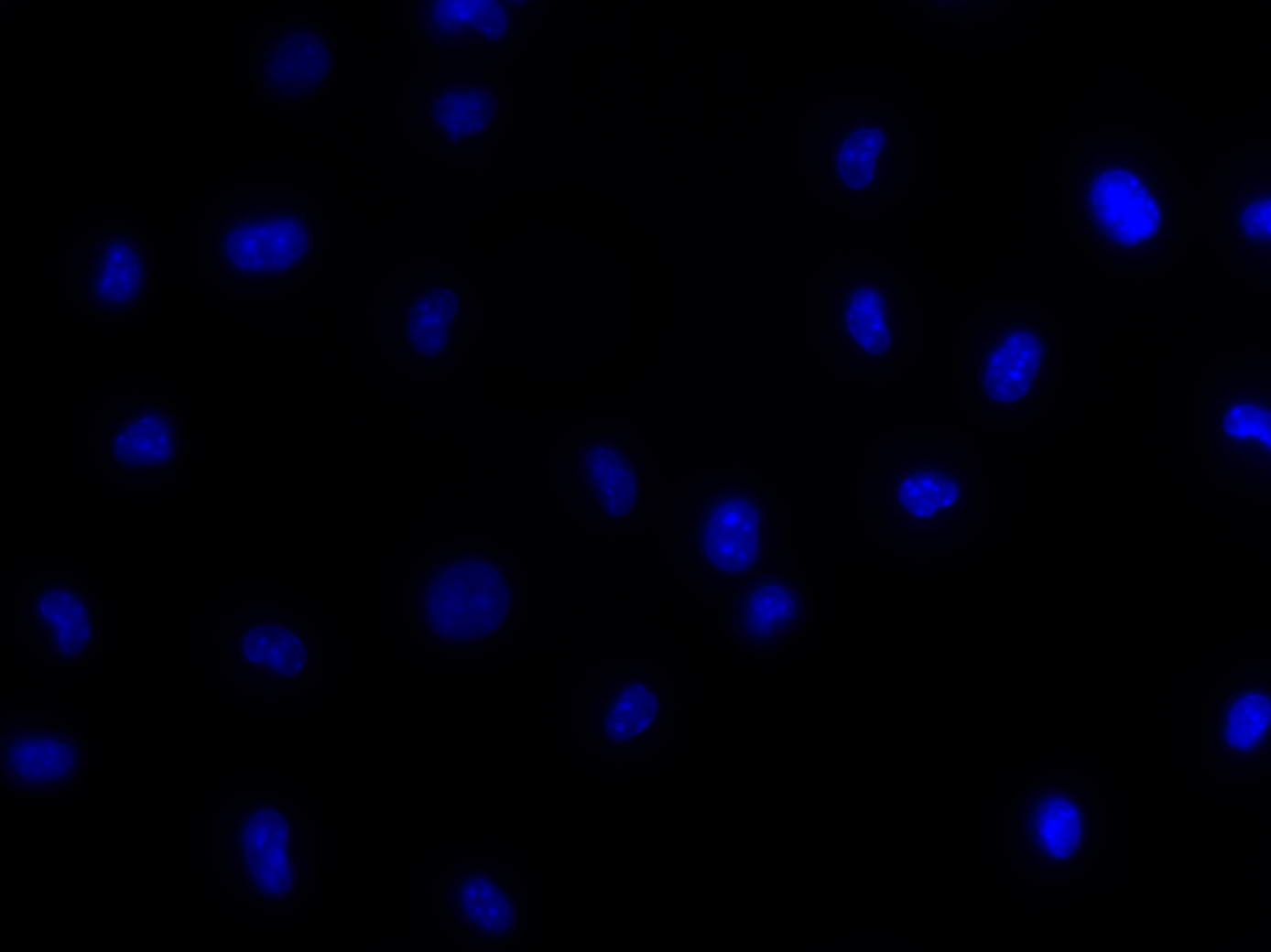

Supplement: Additional file 3 — The zip archive contains real images showing macrophages. (ZIP 28979 kb) [file 12859_2017_1591_MOESM3_ESM.zip › macrophages/jw-24h 5_c5.png]

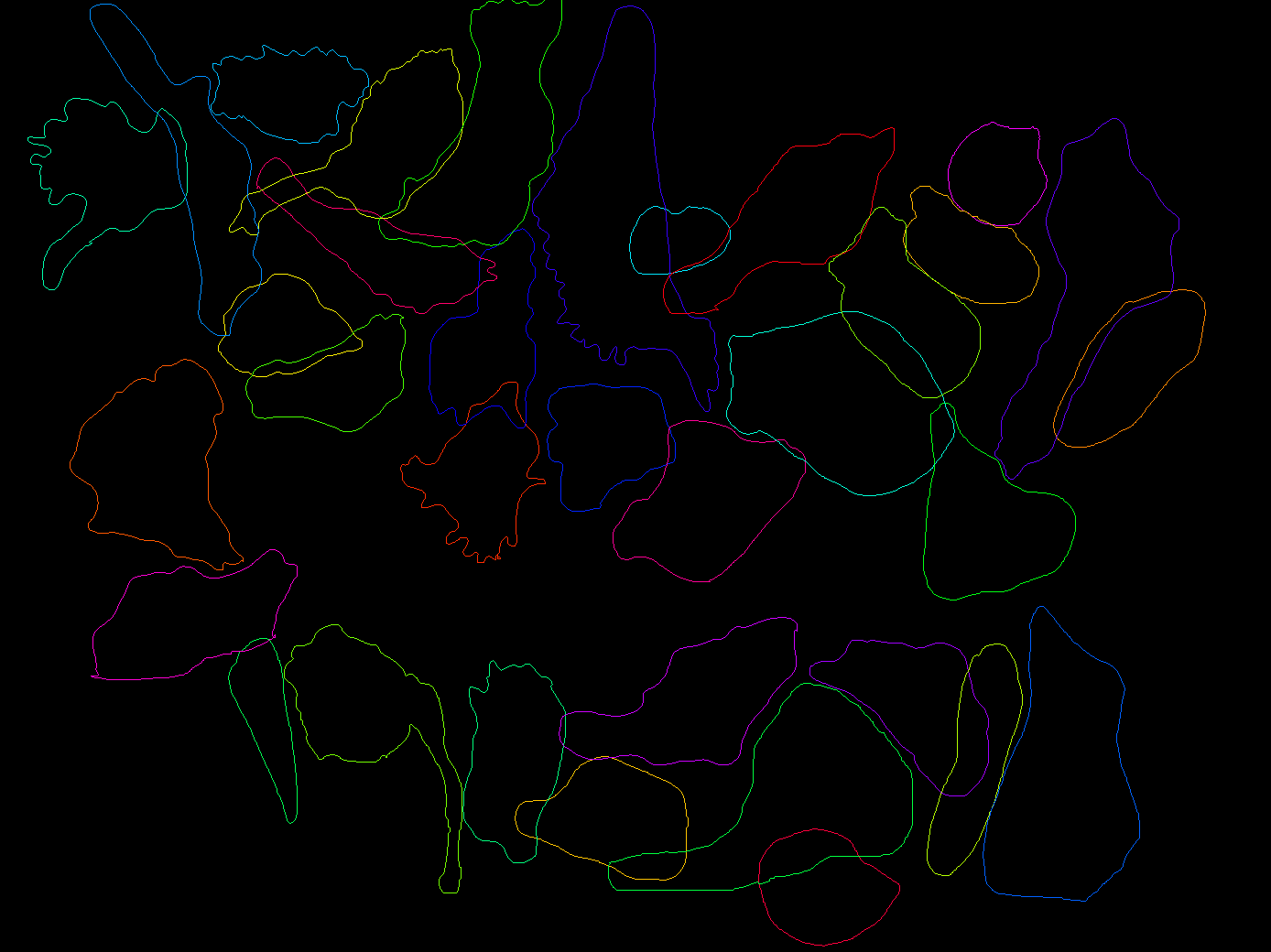

Supplement: Additional file 3 — The zip archive contains real images showing macrophages. (ZIP 28979 kb) [file 12859_2017_1591_MOESM3_ESM.zip › macrophages/jw-2h 1_c1 gt.png]

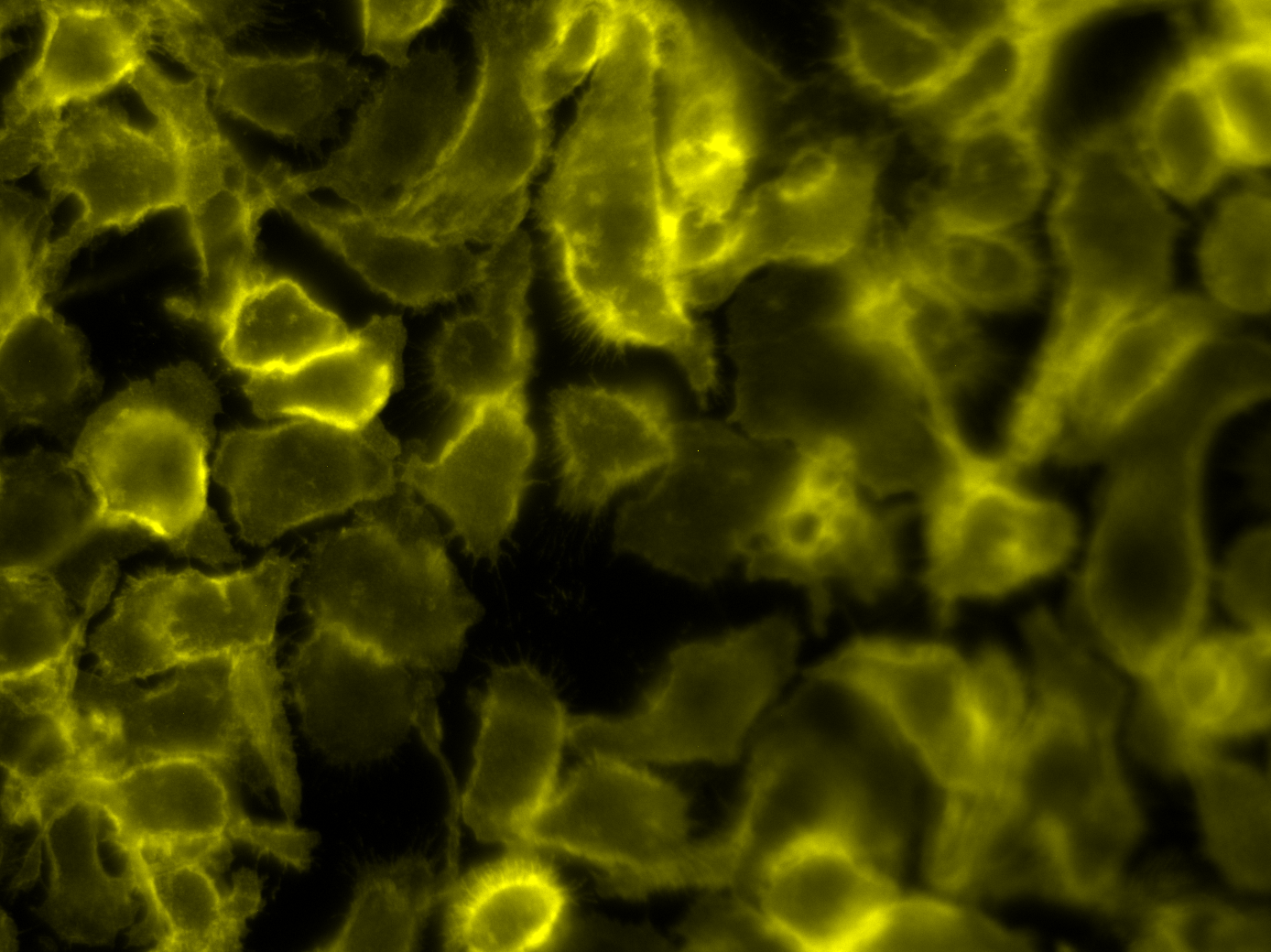

Supplement: Additional file 3 — The zip archive contains real images showing macrophages. (ZIP 28979 kb) [file 12859_2017_1591_MOESM3_ESM.zip › macrophages/jw-2h 1_c1.png]

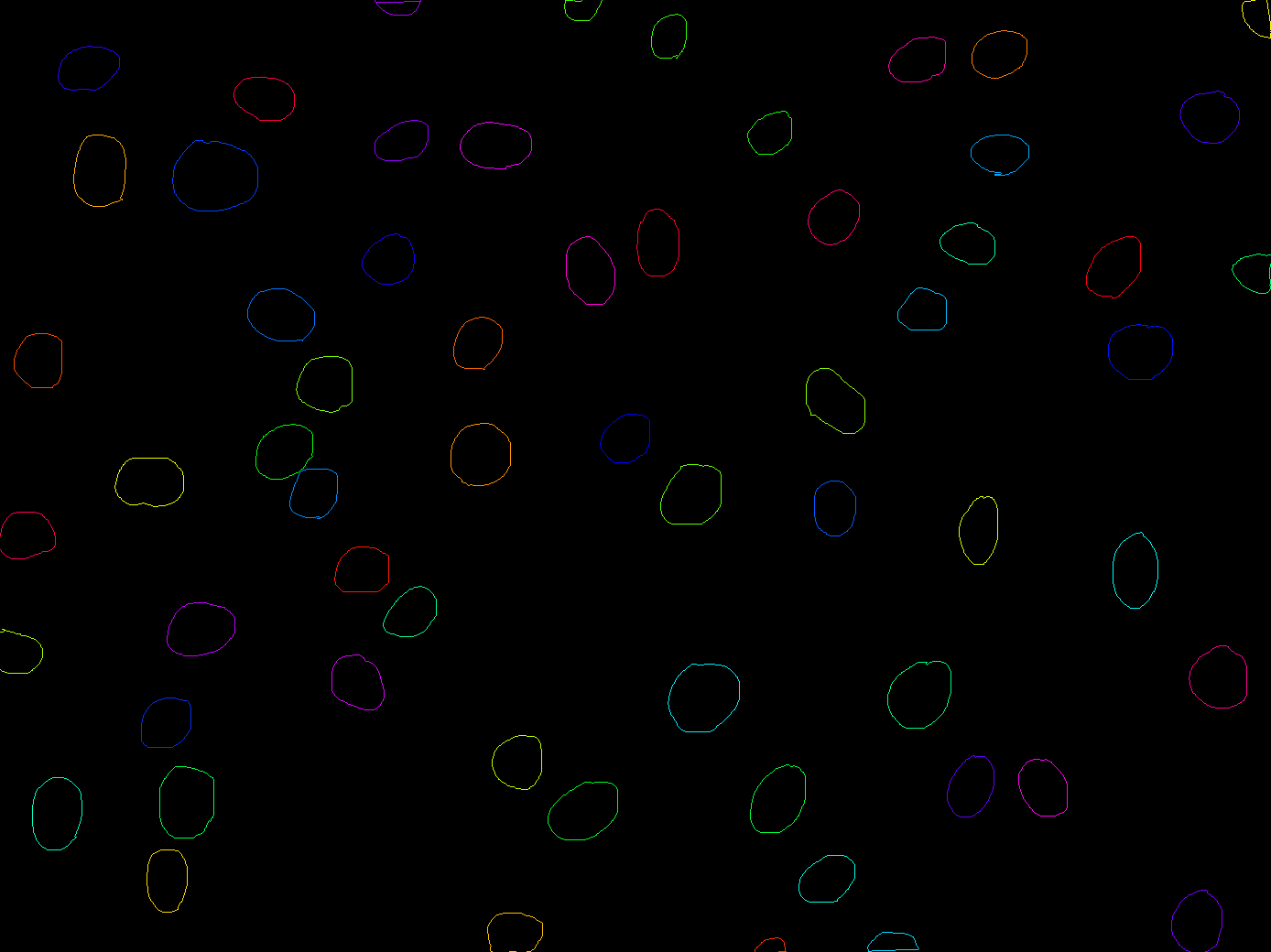

Supplement: Additional file 3 — The zip archive contains real images showing macrophages. (ZIP 28979 kb) [file 12859_2017_1591_MOESM3_ESM.zip › macrophages/jw-2h 1_c5 gt.png]

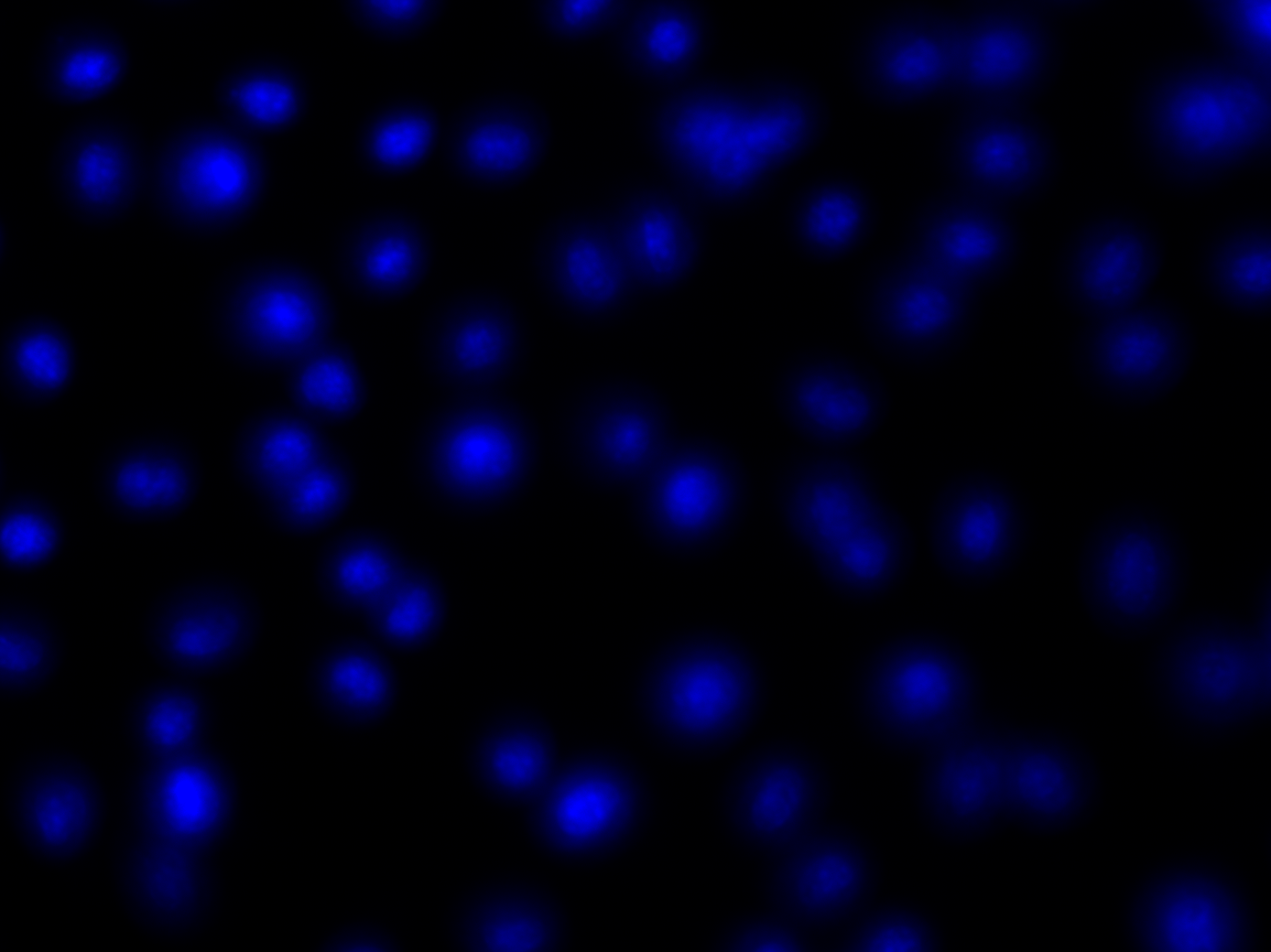

Supplement: Additional file 3 — The zip archive contains real images showing macrophages. (ZIP 28979 kb) [file 12859_2017_1591_MOESM3_ESM.zip › macrophages/jw-2h 1_c5.png]

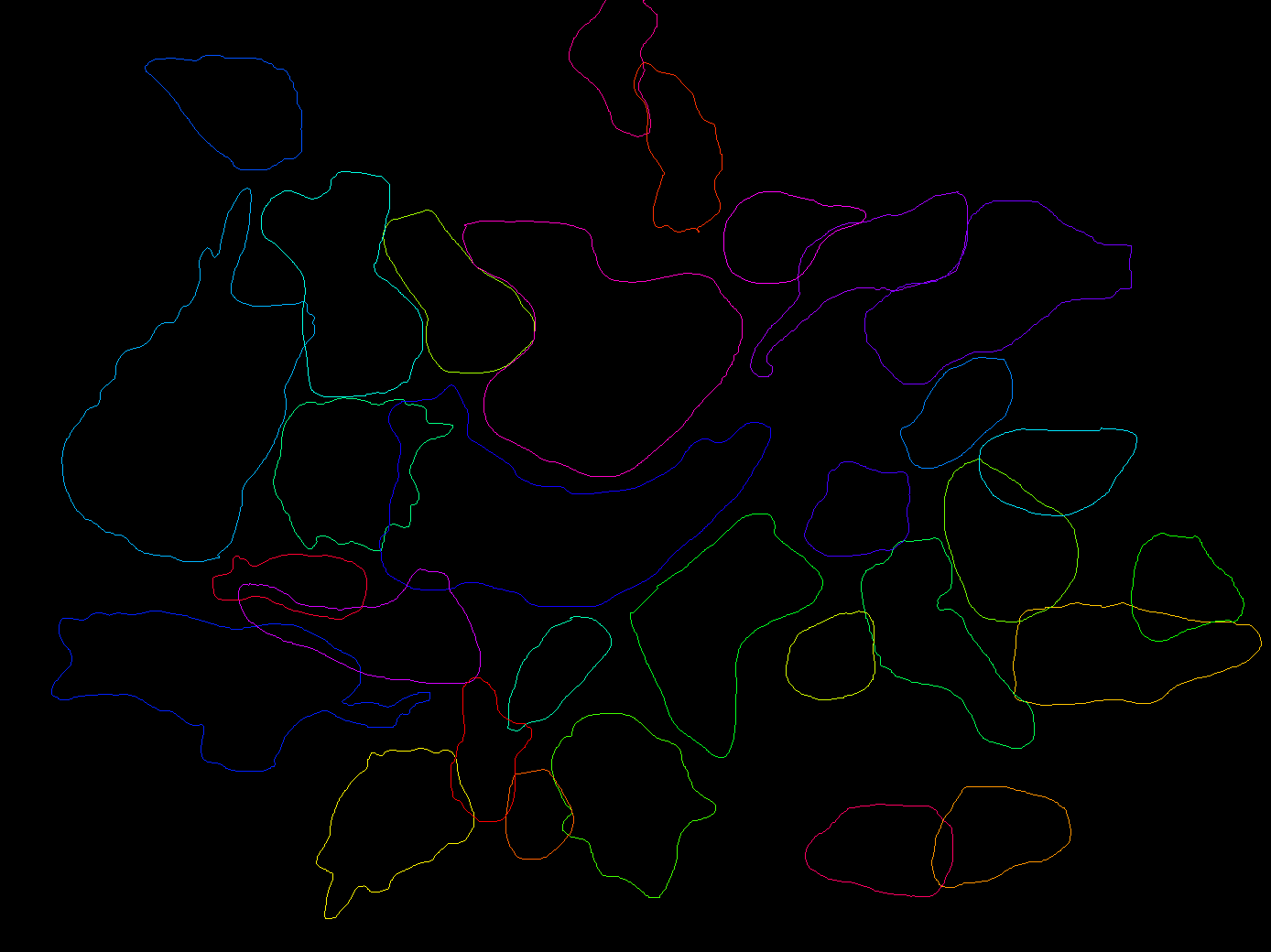

Supplement: Additional file 3 — The zip archive contains real images showing macrophages. (ZIP 28979 kb) [file 12859_2017_1591_MOESM3_ESM.zip › macrophages/jw-2h 2_c1 gt.png]

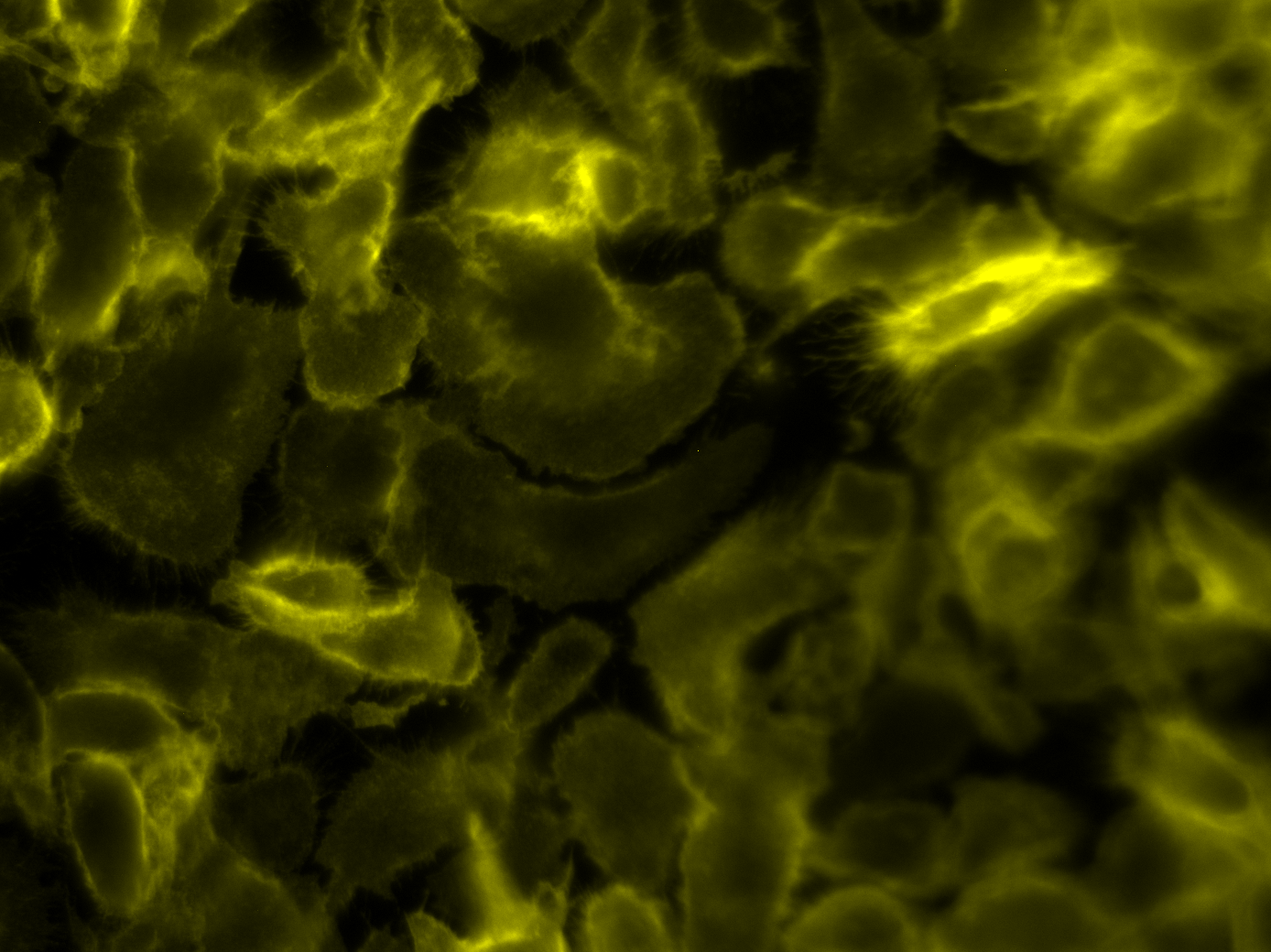

Supplement: Additional file 3 — The zip archive contains real images showing macrophages. (ZIP 28979 kb) [file 12859_2017_1591_MOESM3_ESM.zip › macrophages/jw-2h 2_c1.png]

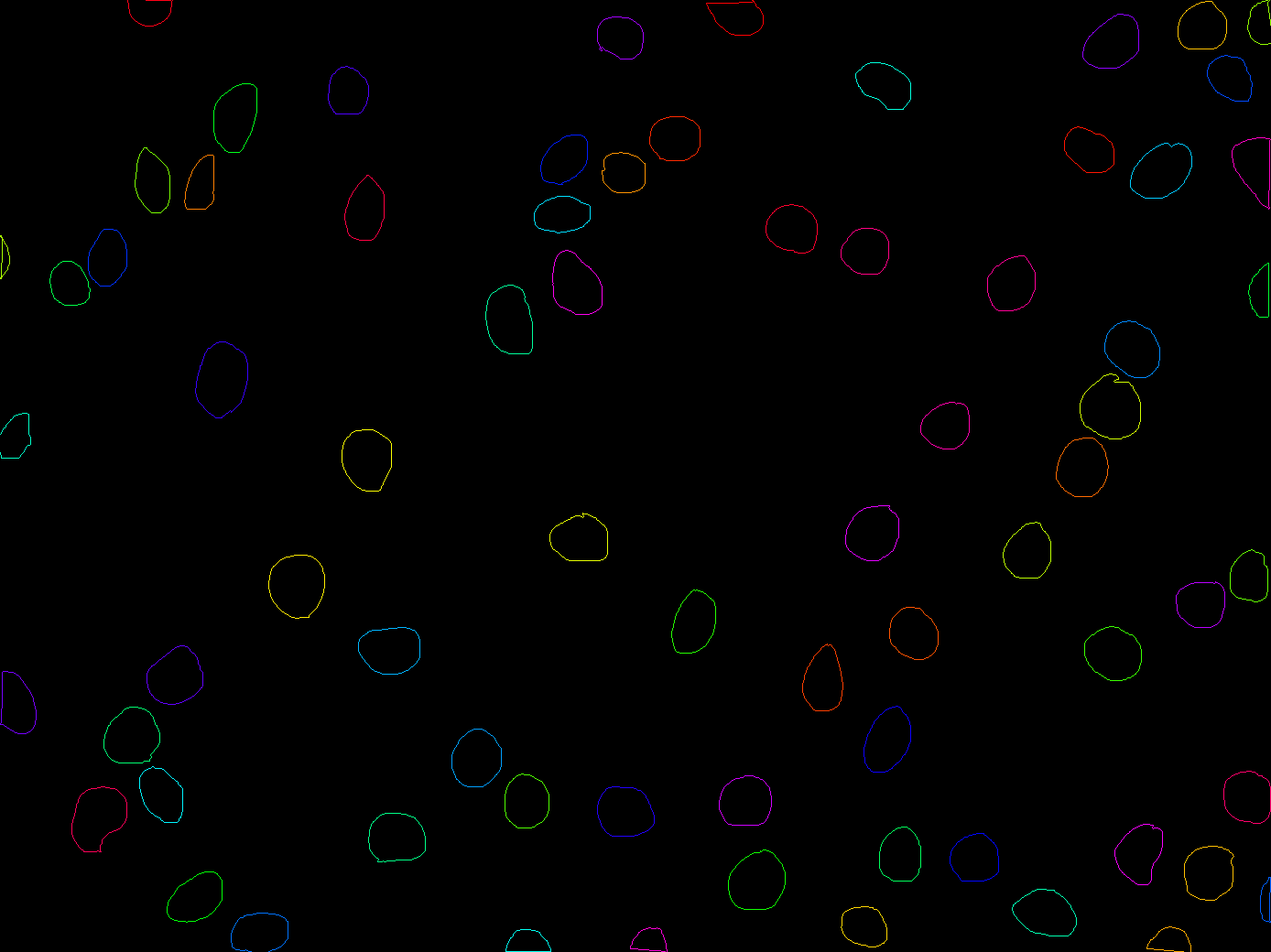

Supplement: Additional file 3 — The zip archive contains real images showing macrophages. (ZIP 28979 kb) [file 12859_2017_1591_MOESM3_ESM.zip › macrophages/jw-2h 2_c5 gt.png]

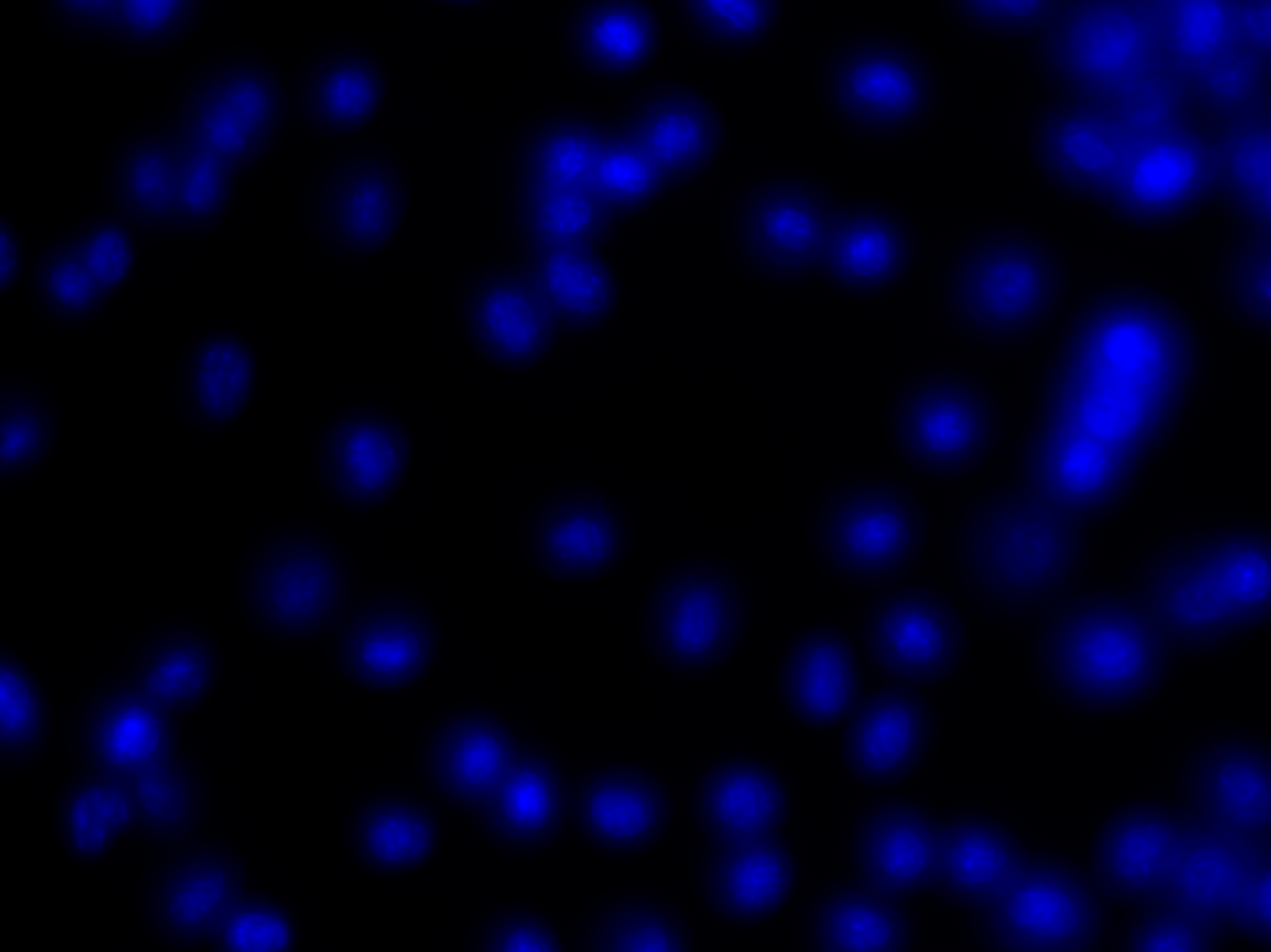

Supplement: Additional file 3 — The zip archive contains real images showing macrophages. (ZIP 28979 kb) [file 12859_2017_1591_MOESM3_ESM.zip › macrophages/jw-2h 2_c5.png]

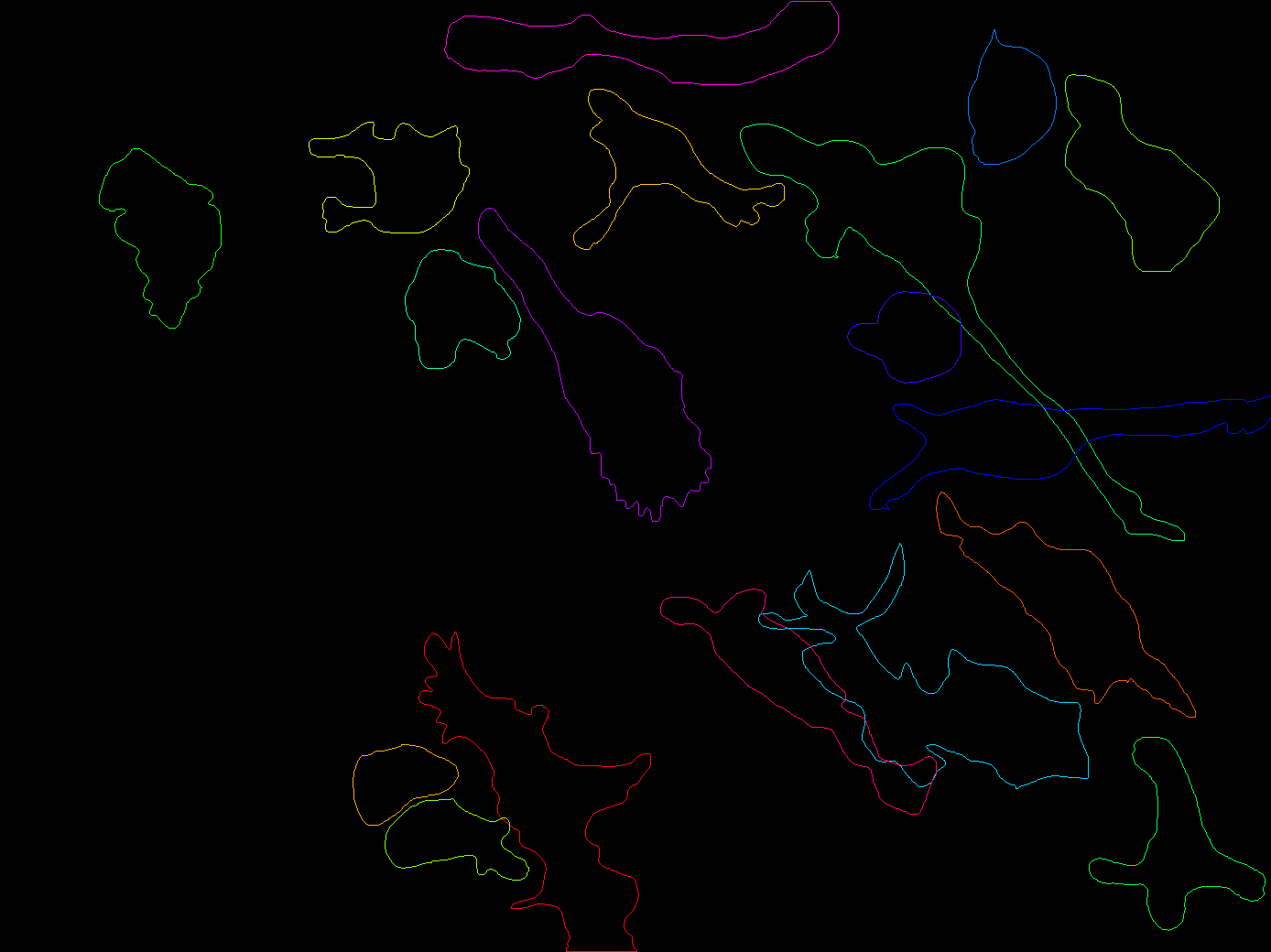

Supplement: Additional file 3 — The zip archive contains real images showing macrophages. (ZIP 28979 kb) [file 12859_2017_1591_MOESM3_ESM.zip › macrophages/jw-Kontrolle1_c1 gt.png]

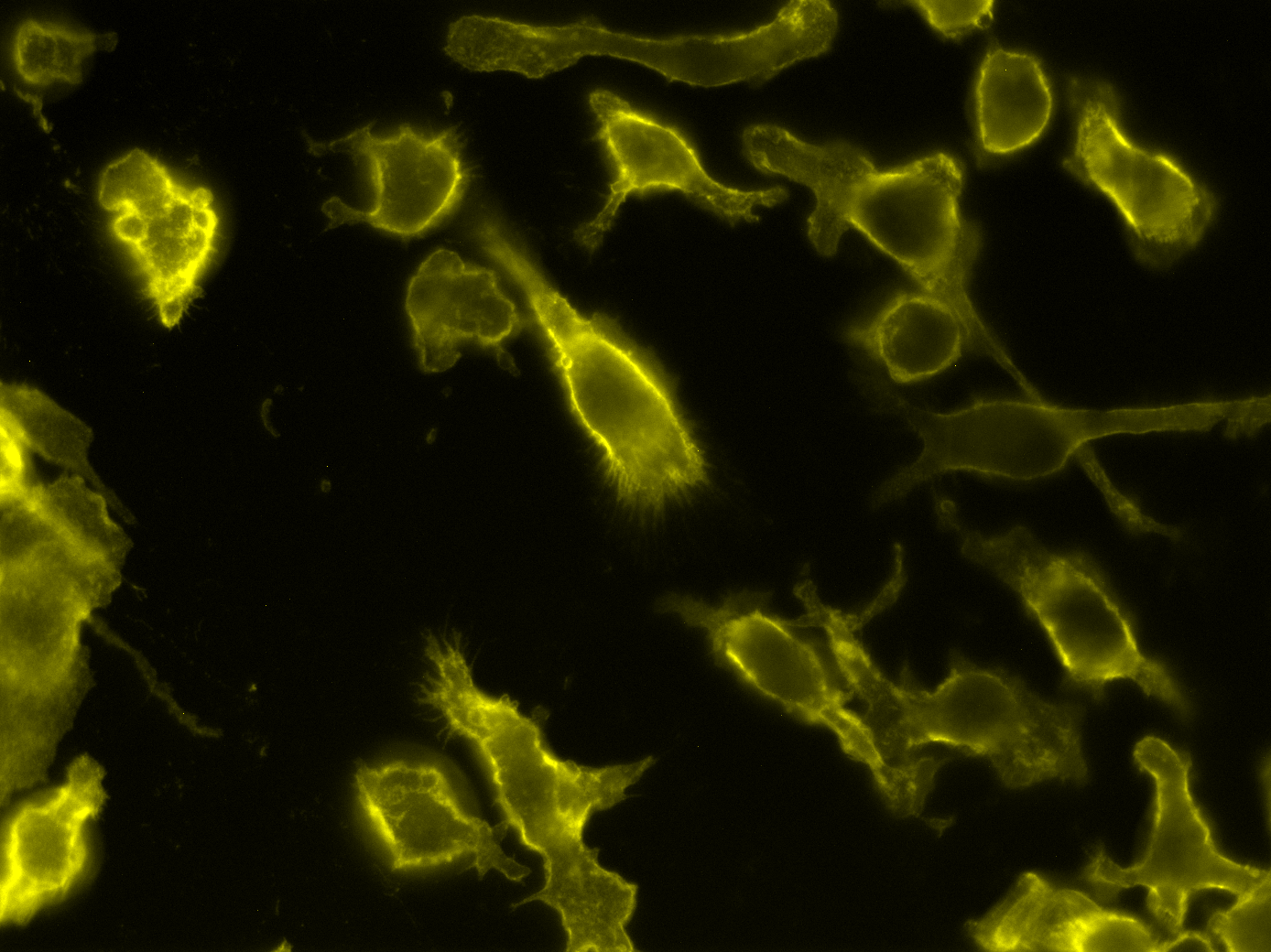

Supplement: Additional file 3 — The zip archive contains real images showing macrophages. (ZIP 28979 kb) [file 12859_2017_1591_MOESM3_ESM.zip › macrophages/jw-Kontrolle1_c1.png]

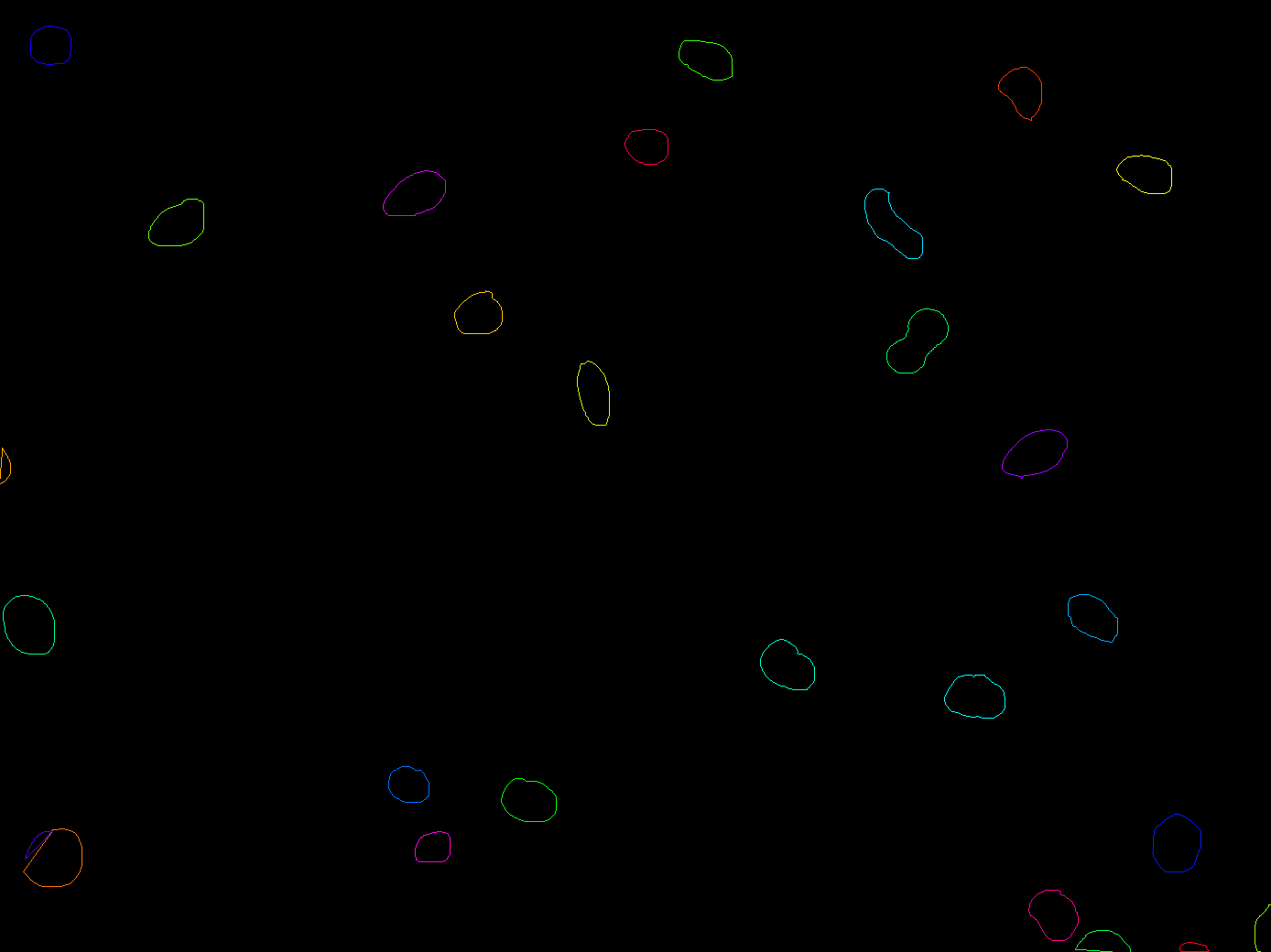

Supplement: Additional file 3 — The zip archive contains real images showing macrophages. (ZIP 28979 kb) [file 12859_2017_1591_MOESM3_ESM.zip › macrophages/jw-Kontrolle1_c5 gt.png]

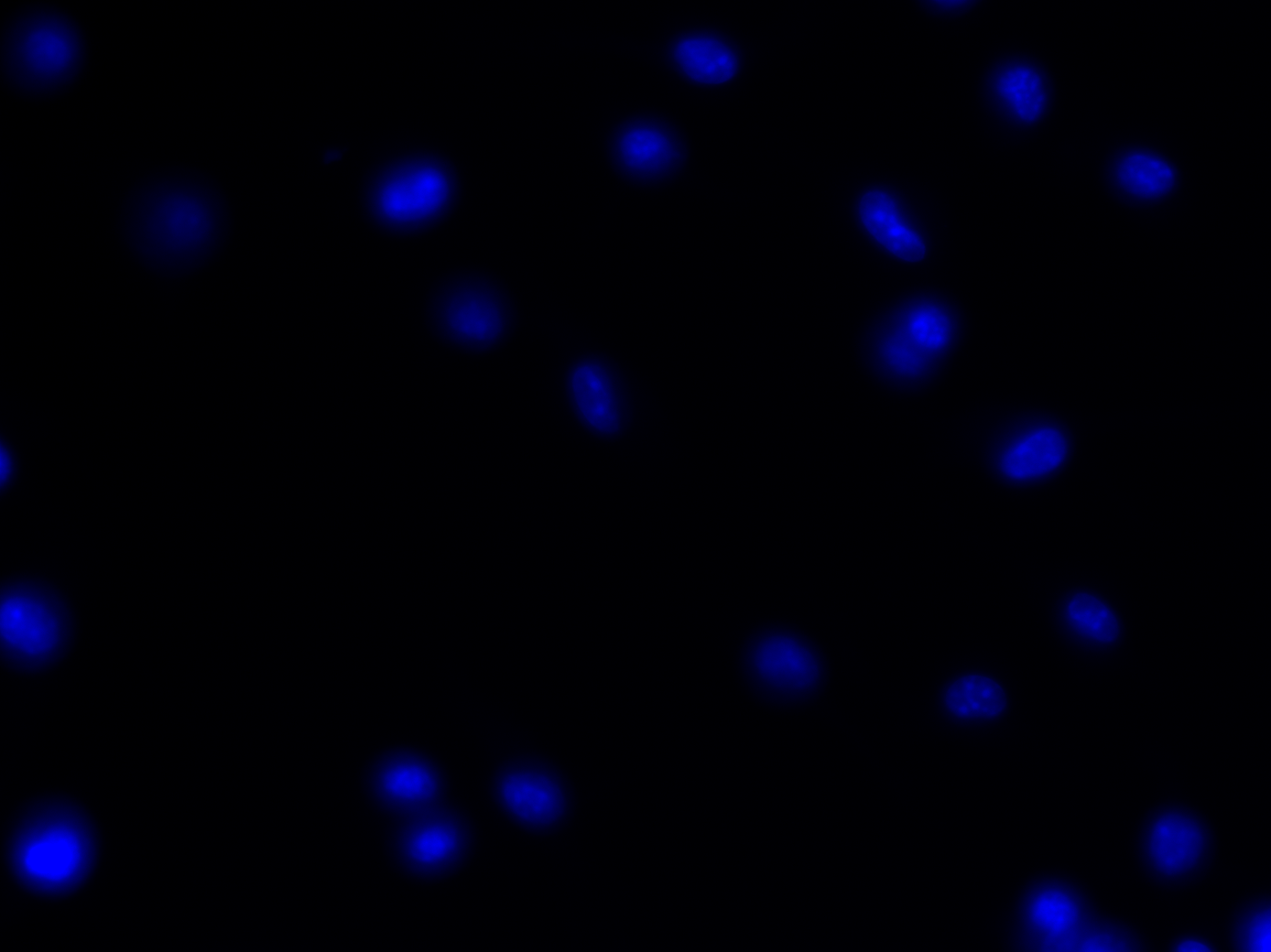

Supplement: Additional file 3 — The zip archive contains real images showing macrophages. (ZIP 28979 kb) [file 12859_2017_1591_MOESM3_ESM.zip › macrophages/jw-Kontrolle1_c5.png]

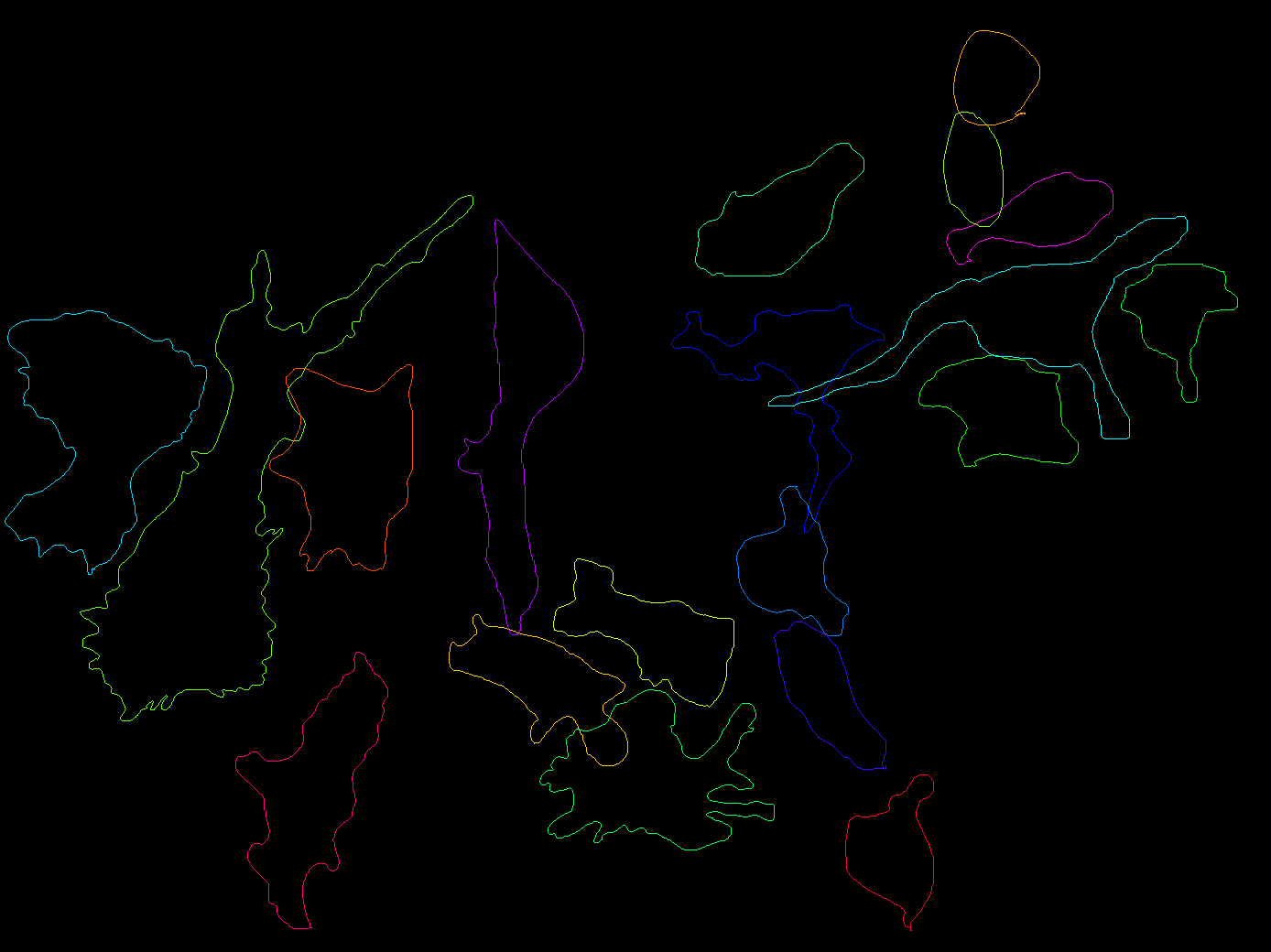

Supplement: Additional file 3 — The zip archive contains real images showing macrophages. (ZIP 28979 kb) [file 12859_2017_1591_MOESM3_ESM.zip › macrophages/jw-Kontrolle2_c1 gt.png]

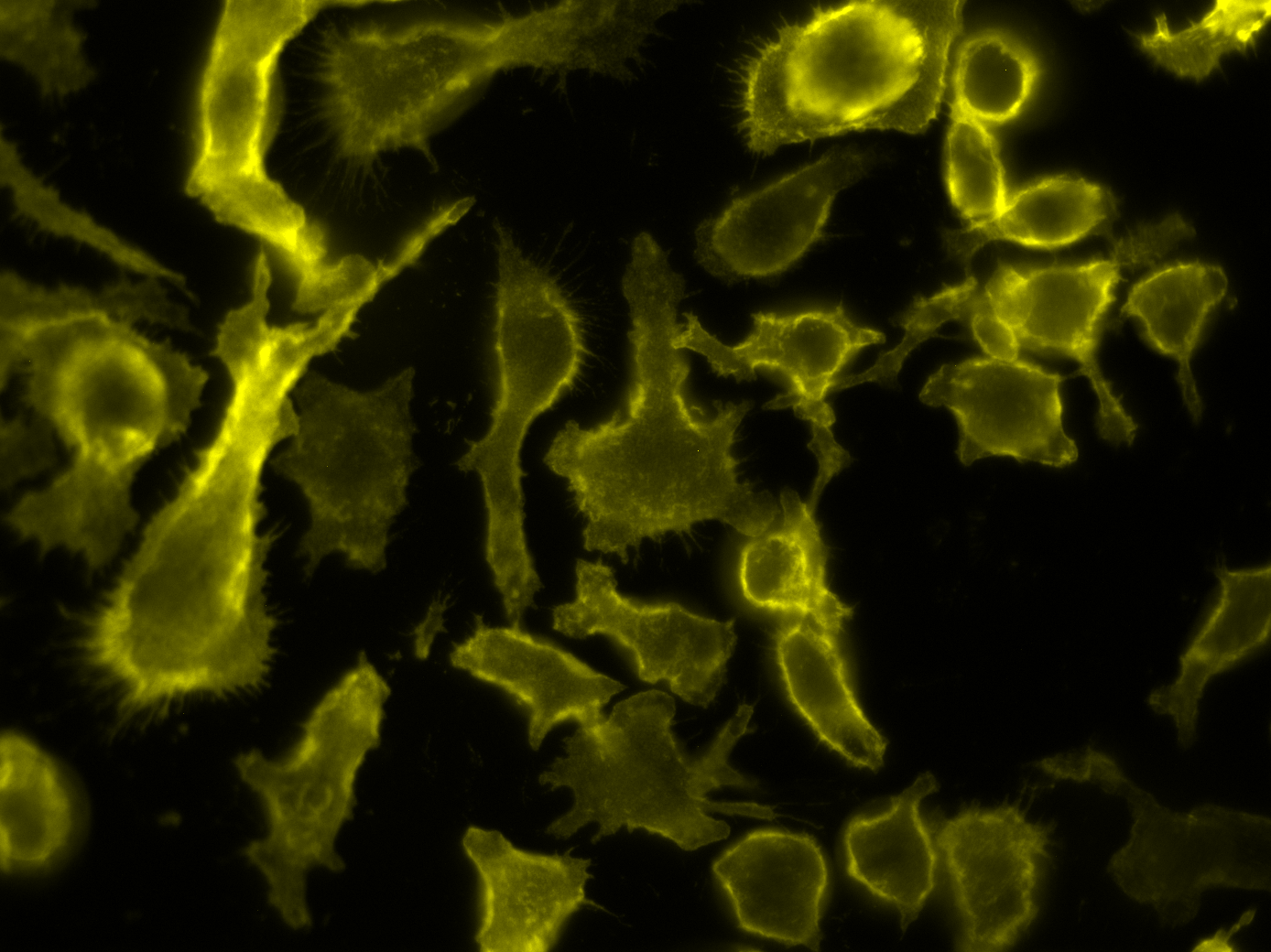

Supplement: Additional file 3 — The zip archive contains real images showing macrophages. (ZIP 28979 kb) [file 12859_2017_1591_MOESM3_ESM.zip › macrophages/jw-Kontrolle2_c1.png]

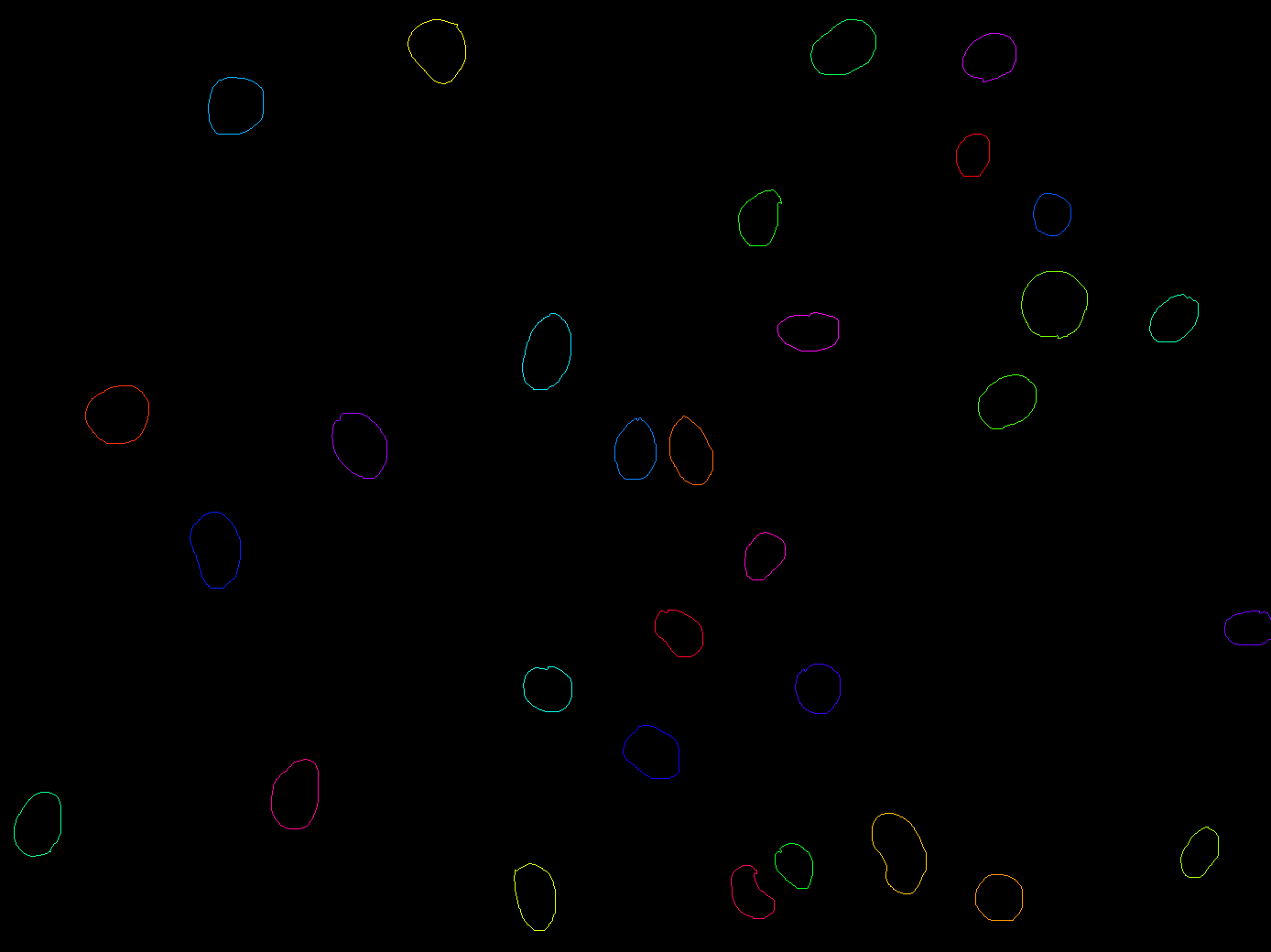

Supplement: Additional file 3 — The zip archive contains real images showing macrophages. (ZIP 28979 kb) [file 12859_2017_1591_MOESM3_ESM.zip › macrophages/jw-Kontrolle2_c5 gt.png]

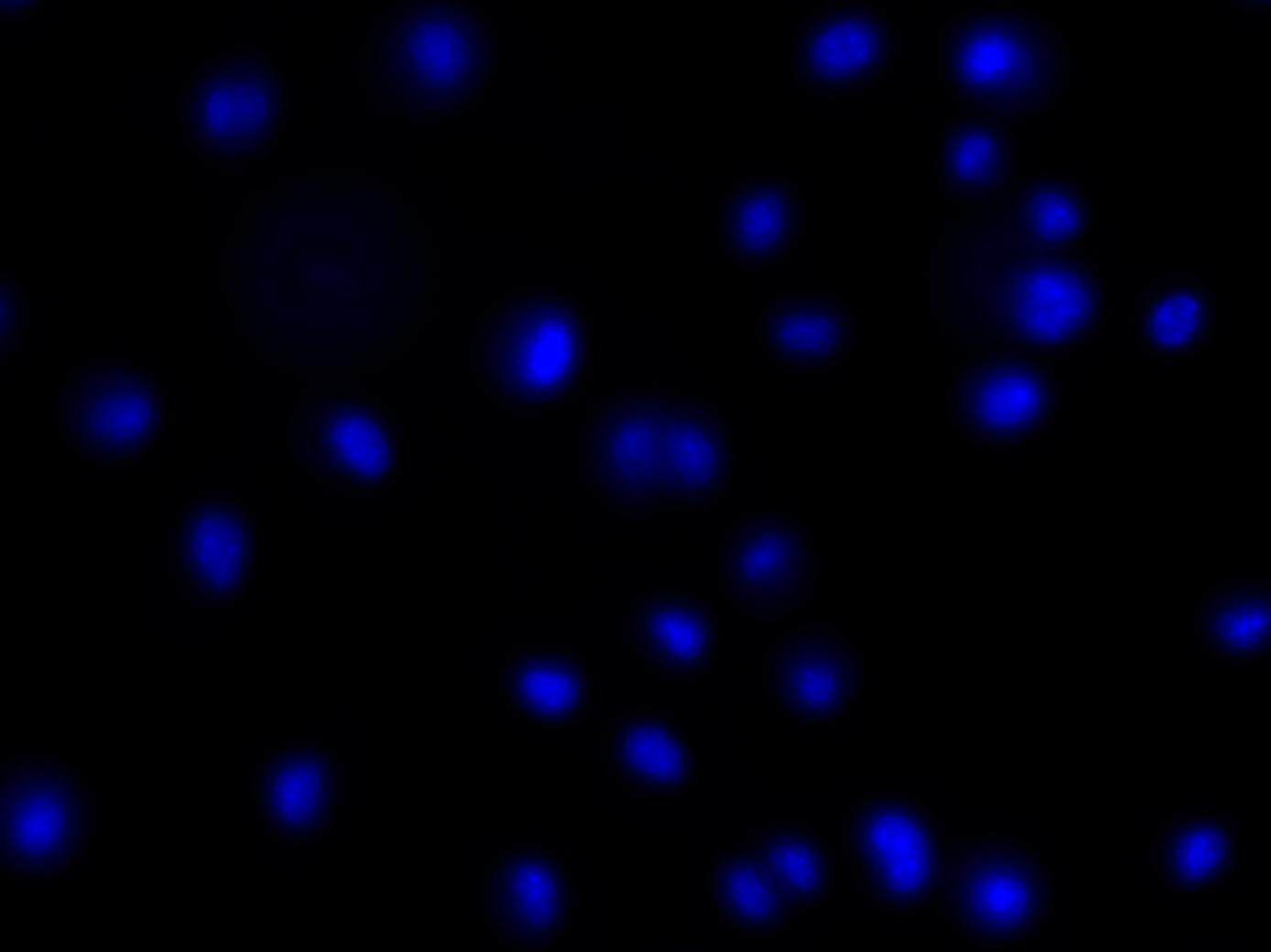

Supplement: Additional file 3 — The zip archive contains real images showing macrophages. (ZIP 28979 kb) [file 12859_2017_1591_MOESM3_ESM.zip › macrophages/jw-Kontrolle2_c5.png]

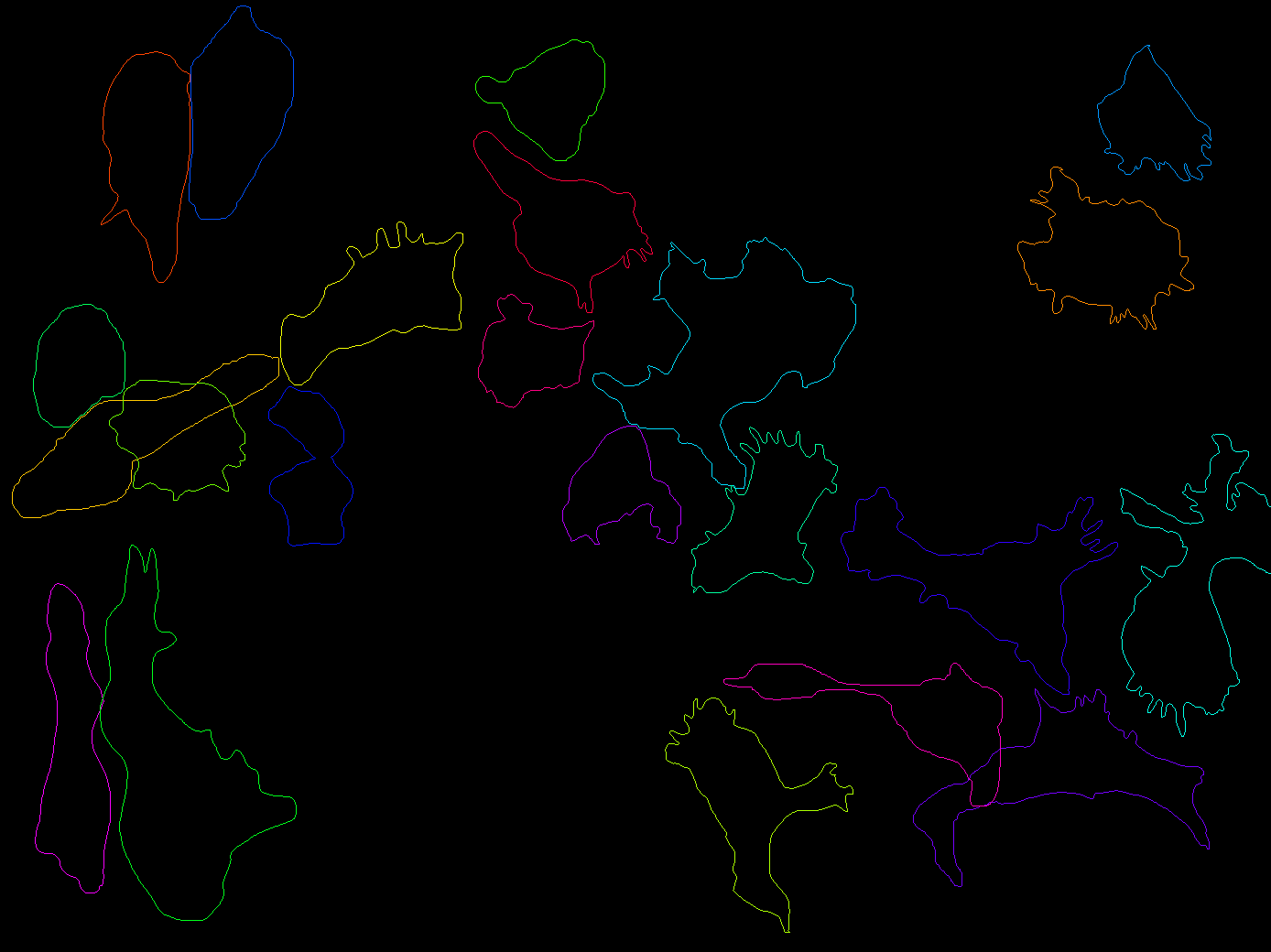

Supplement: Additional file 3 — The zip archive contains real images showing macrophages. (ZIP 28979 kb) [file 12859_2017_1591_MOESM3_ESM.zip › macrophages/jw-Kontrolle3_c1 gt.png]

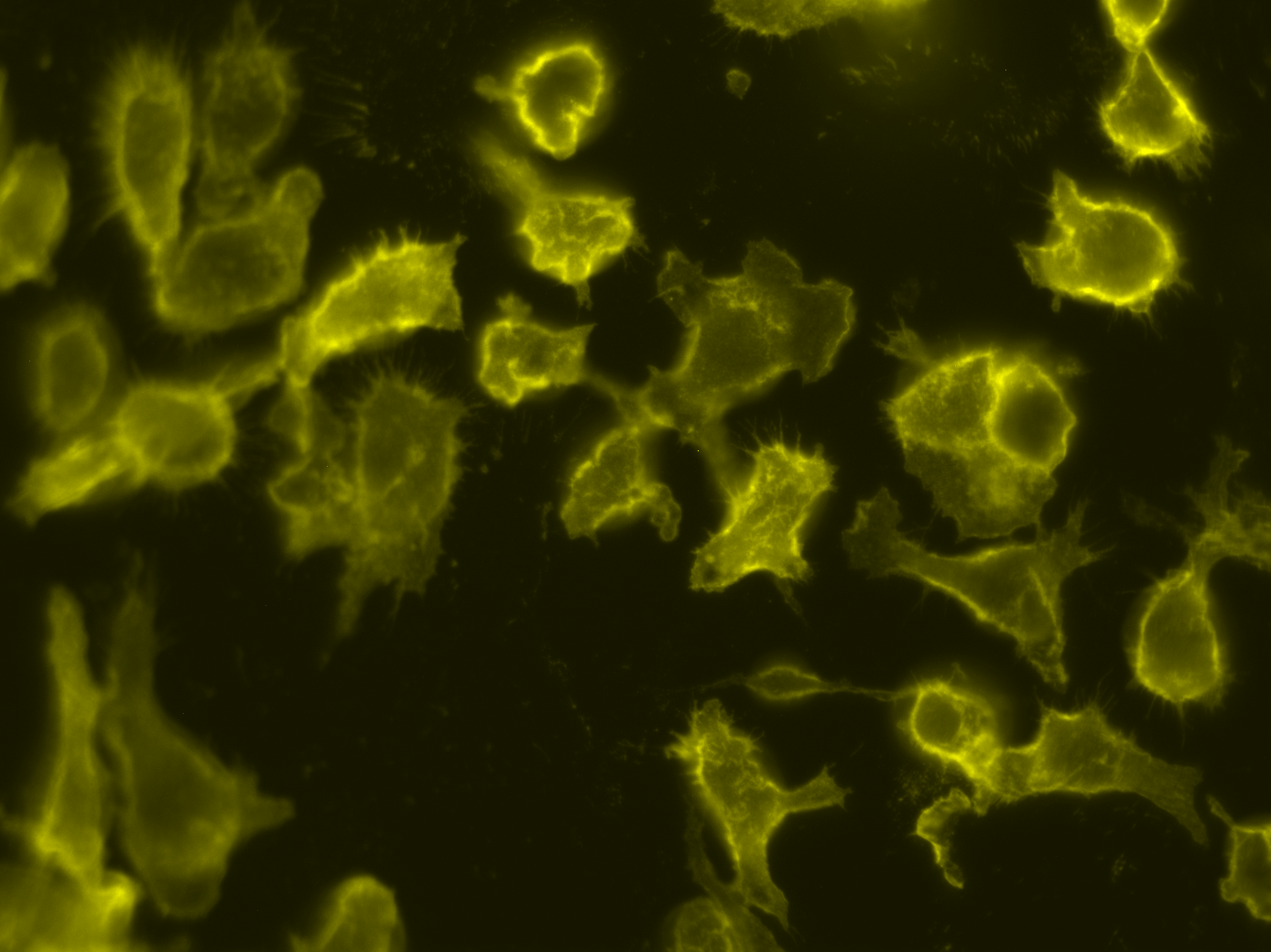

Supplement: Additional file 3 — The zip archive contains real images showing macrophages. (ZIP 28979 kb) [file 12859_2017_1591_MOESM3_ESM.zip › macrophages/jw-Kontrolle3_c1.png]

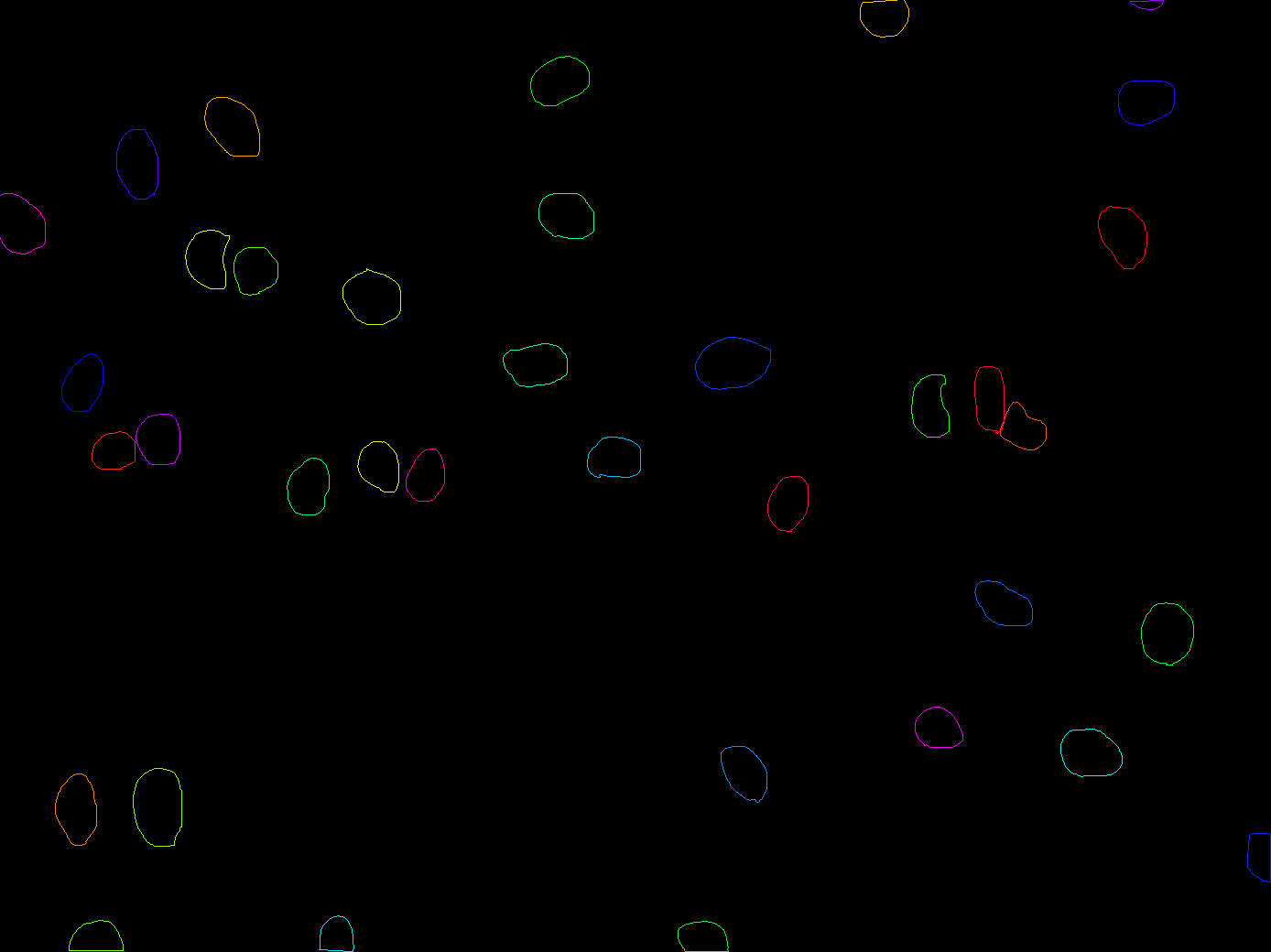

Supplement: Additional file 3 — The zip archive contains real images showing macrophages. (ZIP 28979 kb) [file 12859_2017_1591_MOESM3_ESM.zip › macrophages/jw-Kontrolle3_c5 gt.png]

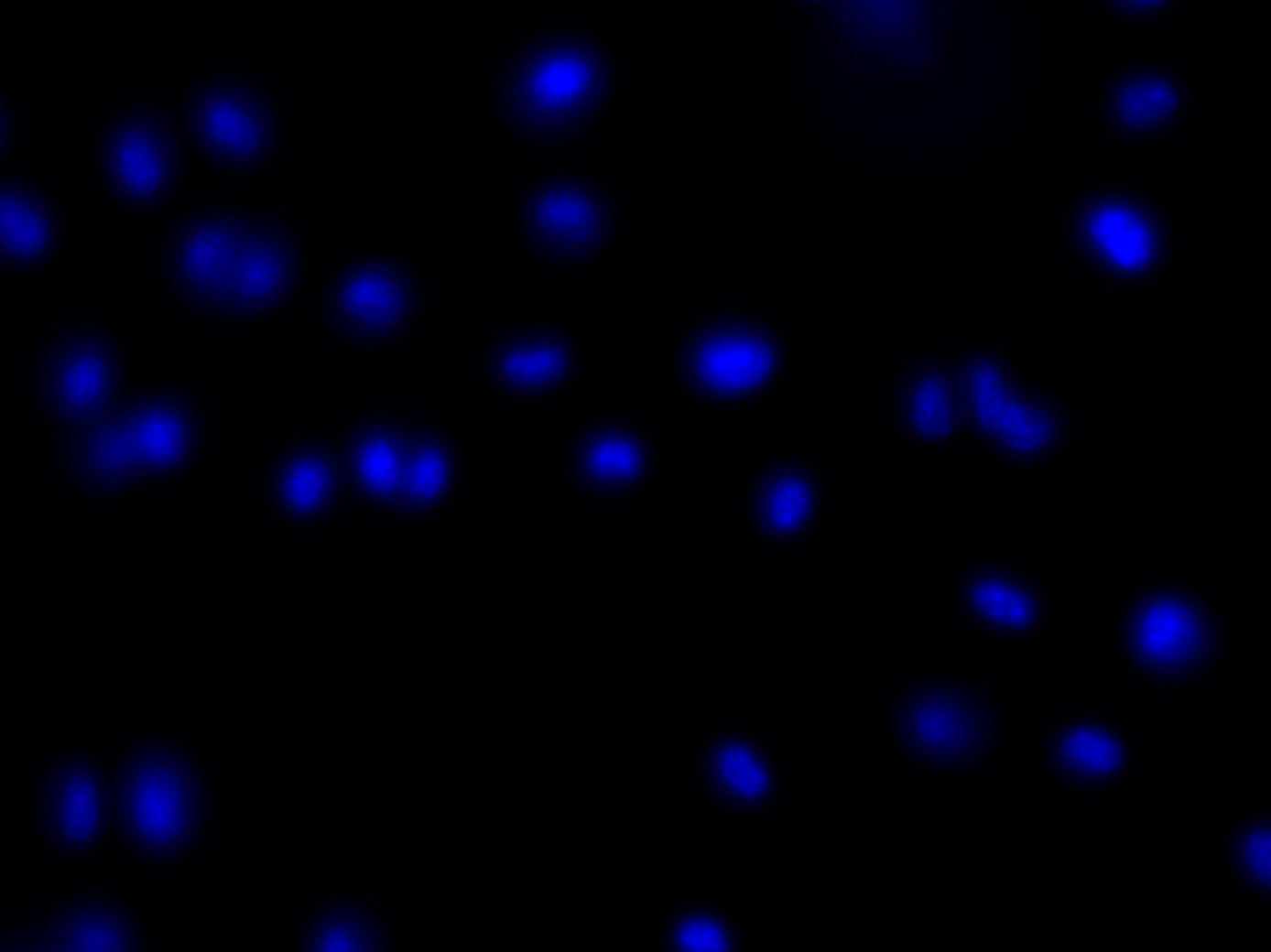

Supplement: Additional file 3 — The zip archive contains real images showing macrophages. (ZIP 28979 kb) [file 12859_2017_1591_MOESM3_ESM.zip › macrophages/jw-Kontrolle3_c5.png]

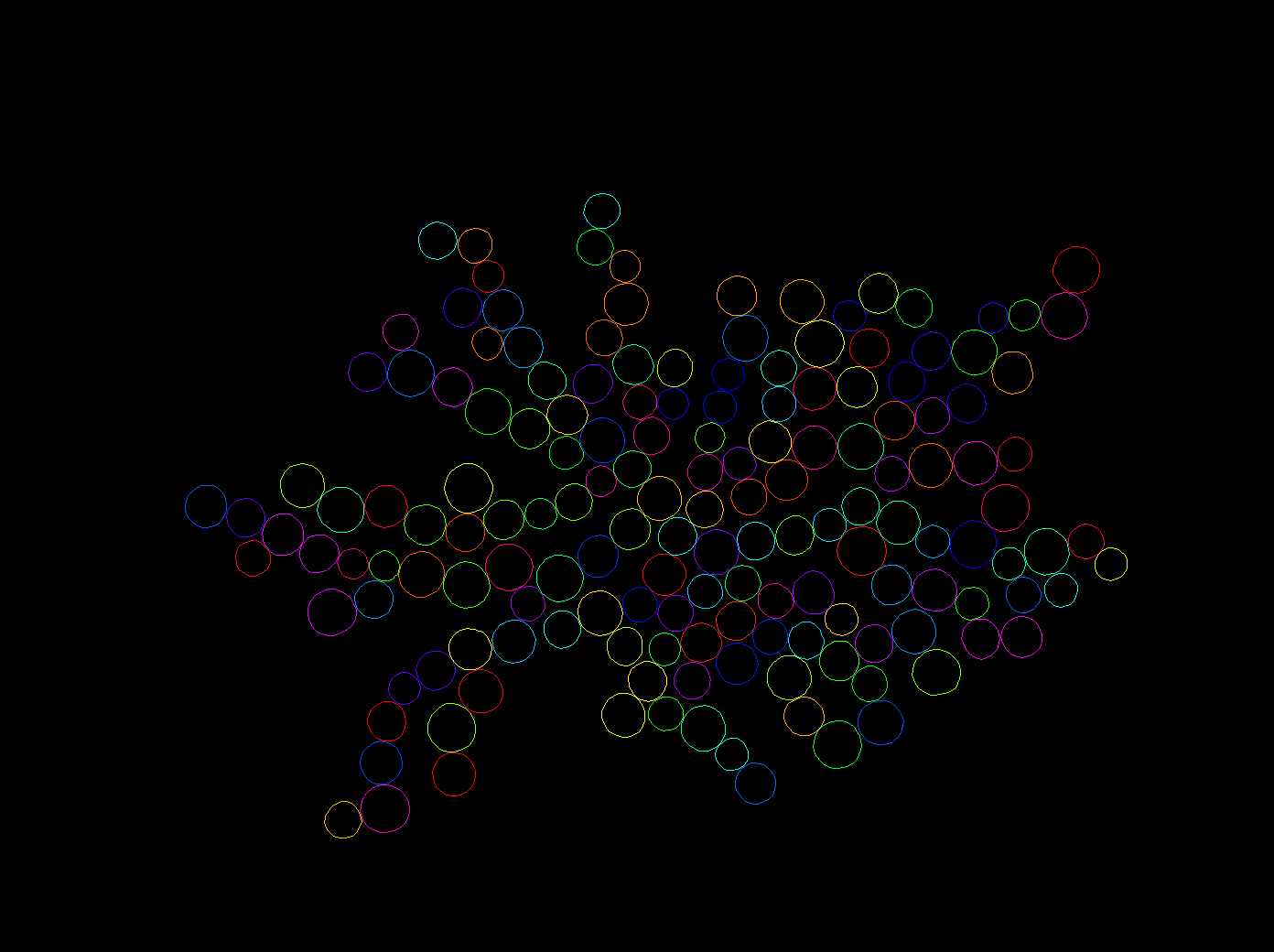

Supplement: Additional file 5 — The zip archive contains simulated images showing protoplasts with corresponding ground truth. (ZIP 72704 kb) [file 12859_2017_1591_MOESM5_ESM.zip › simulated protoplasts/nottouching/nottouching001 gt.png]

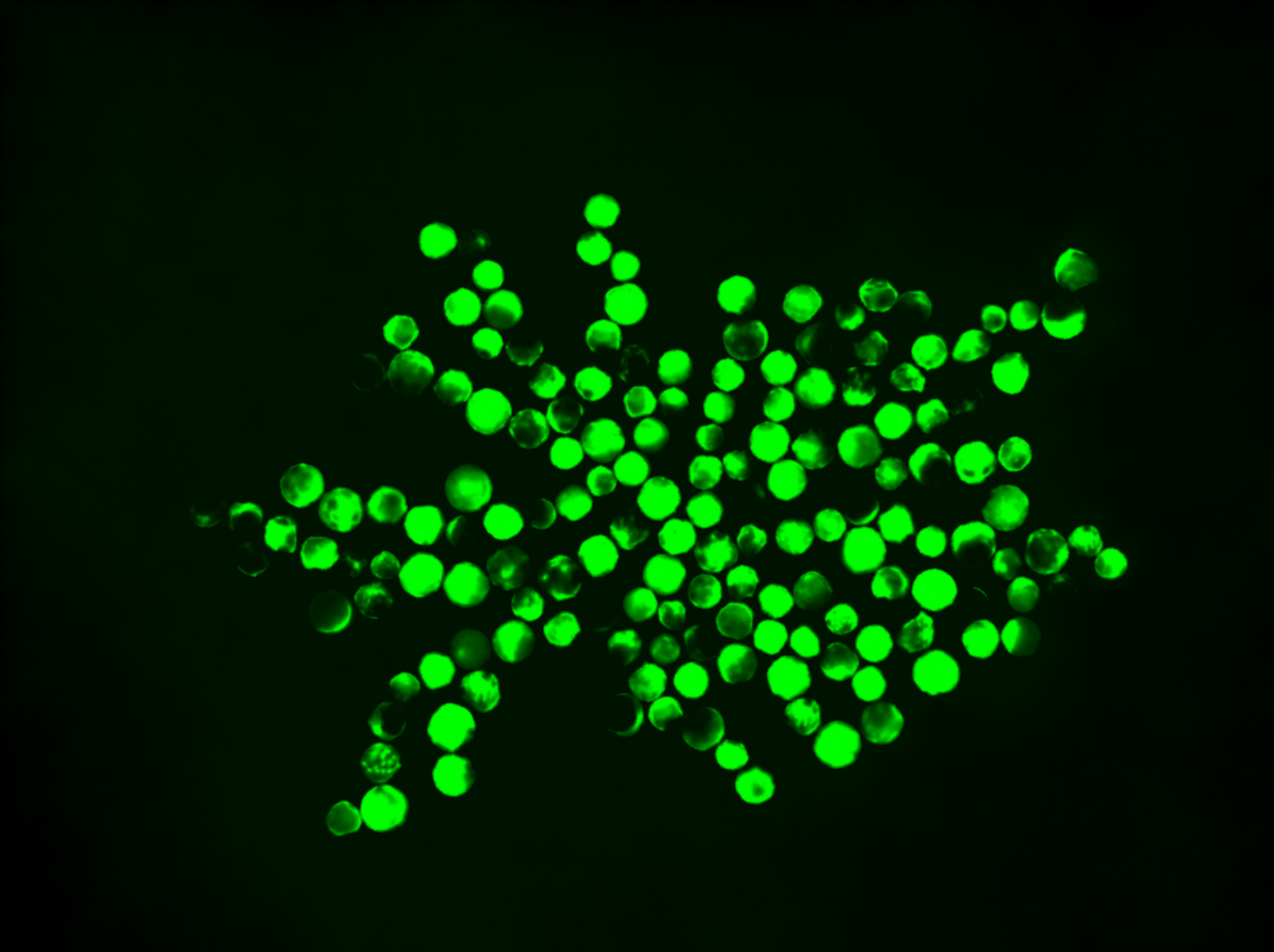

Supplement: Additional file 5 — The zip archive contains simulated images showing protoplasts with corresponding ground truth. (ZIP 72704 kb) [file 12859_2017_1591_MOESM5_ESM.zip › simulated protoplasts/nottouching/nottouching001.png]

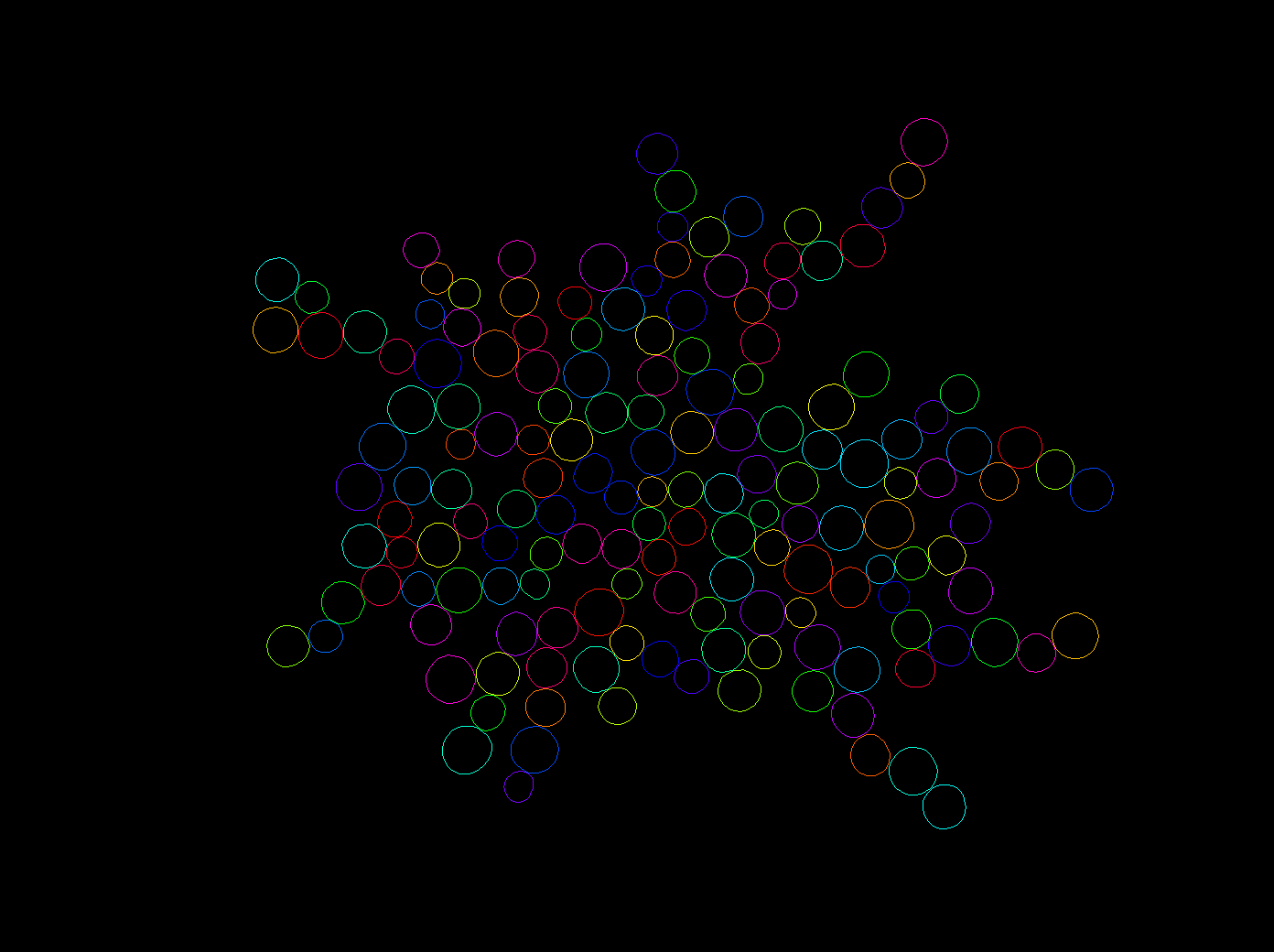

Supplement: Additional file 5 — The zip archive contains simulated images showing protoplasts with corresponding ground truth. (ZIP 72704 kb) [file 12859_2017_1591_MOESM5_ESM.zip › simulated protoplasts/nottouching/nottouching002 gt.png]

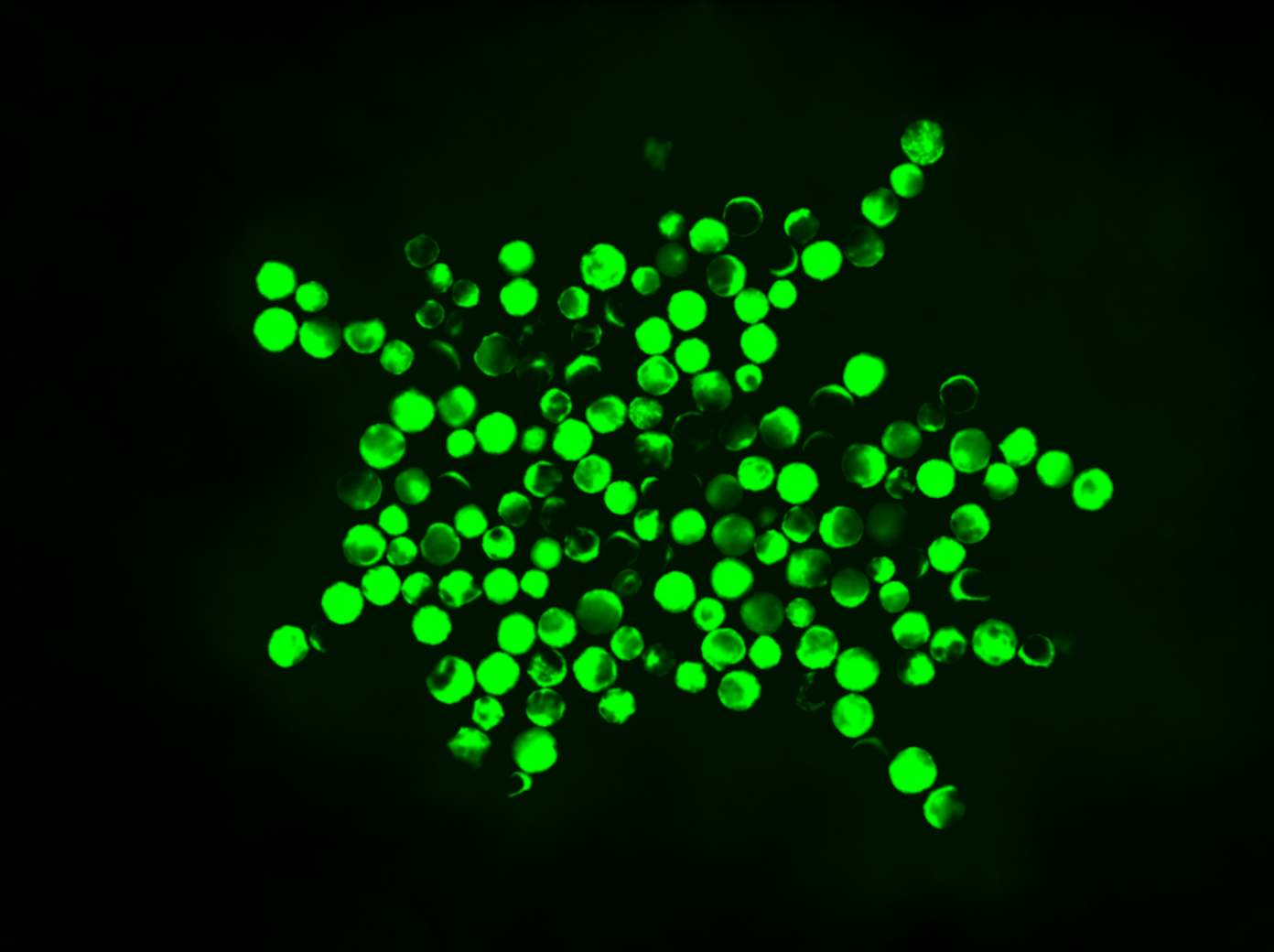

Supplement: Additional file 5 — The zip archive contains simulated images showing protoplasts with corresponding ground truth. (ZIP 72704 kb) [file 12859_2017_1591_MOESM5_ESM.zip › simulated protoplasts/nottouching/nottouching002.png]

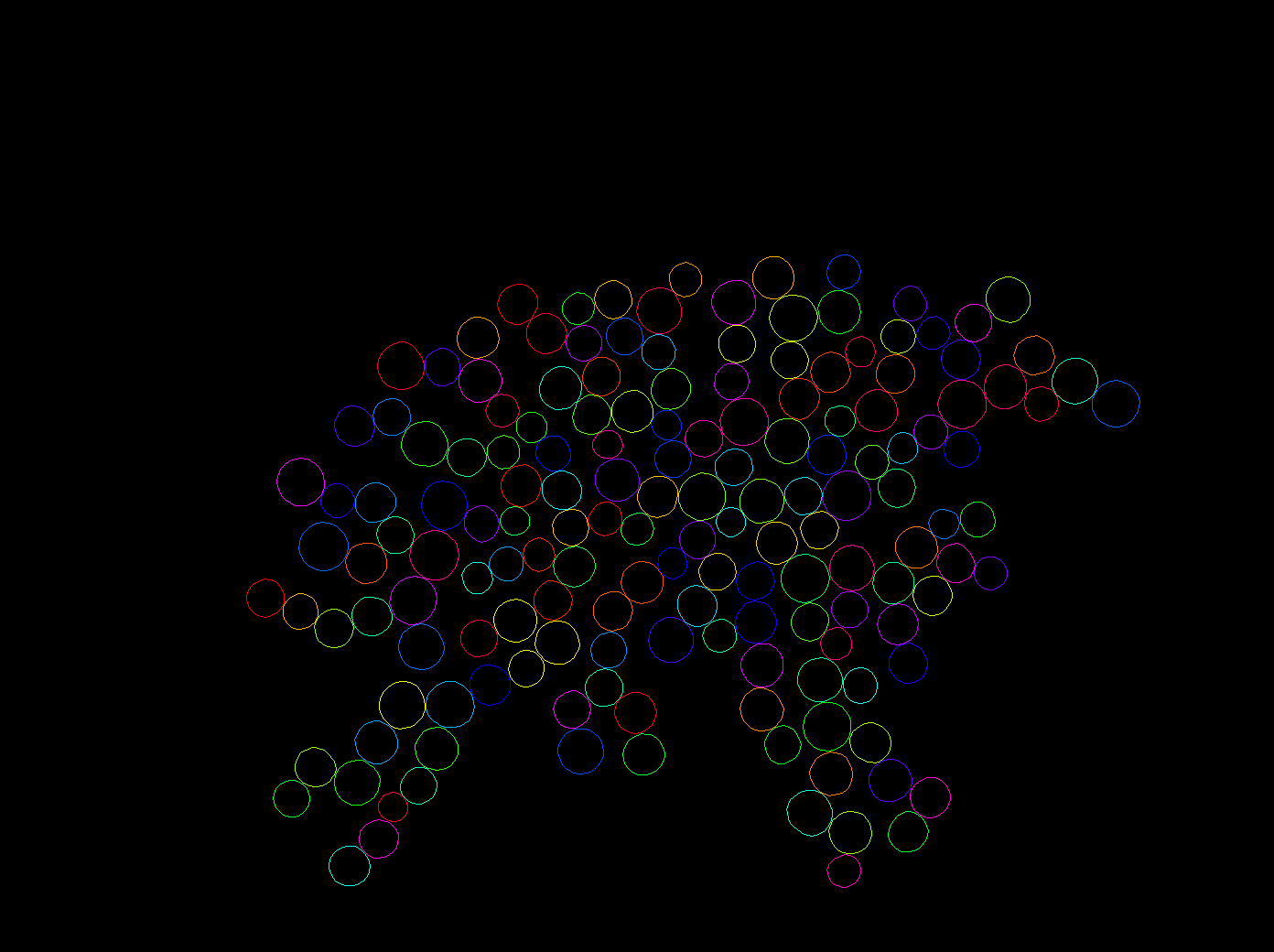

Supplement: Additional file 5 — The zip archive contains simulated images showing protoplasts with corresponding ground truth. (ZIP 72704 kb) [file 12859_2017_1591_MOESM5_ESM.zip › simulated protoplasts/nottouching/nottouching003 gt.png]

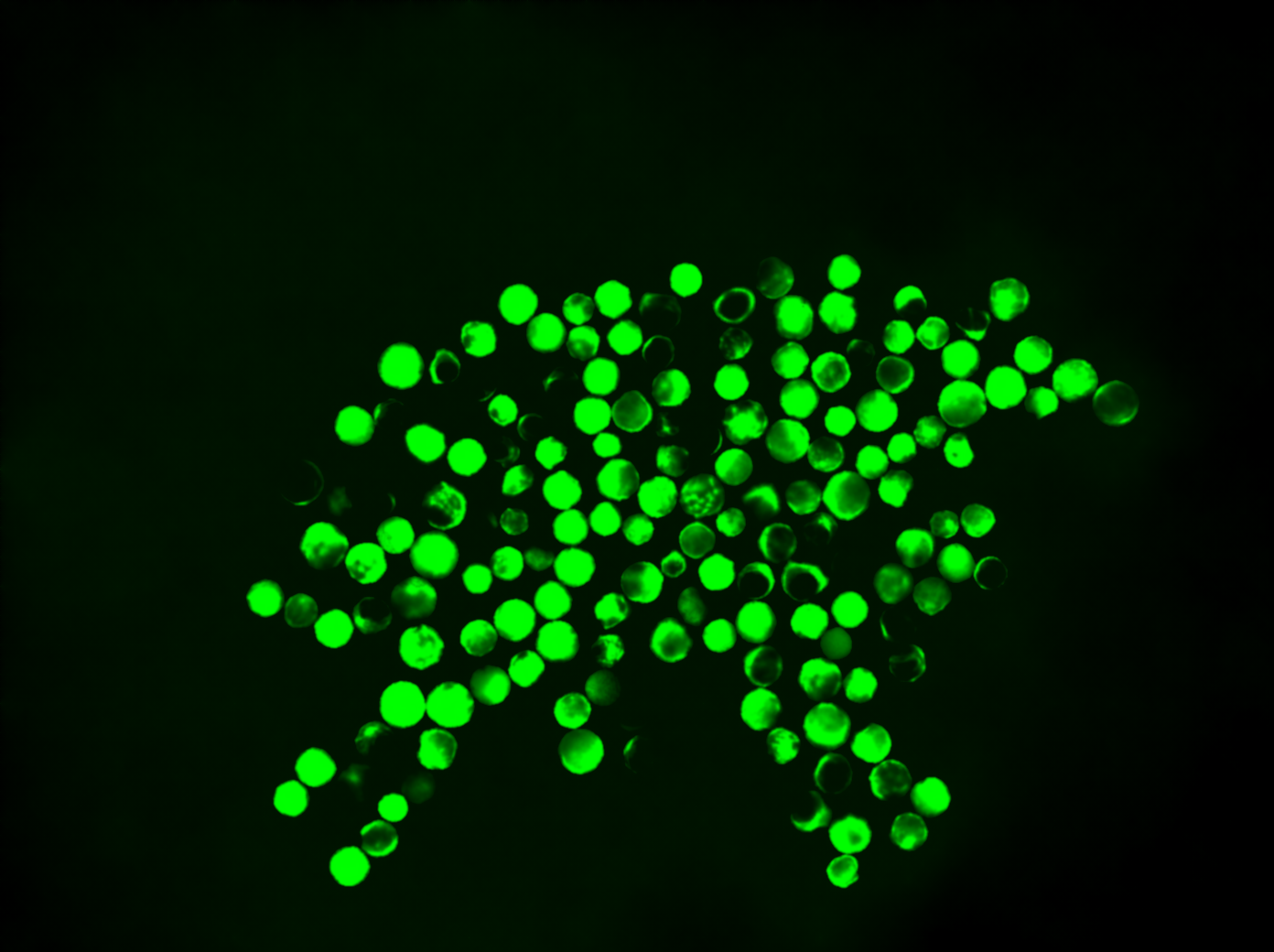

Supplement: Additional file 5 — The zip archive contains simulated images showing protoplasts with corresponding ground truth. (ZIP 72704 kb) [file 12859_2017_1591_MOESM5_ESM.zip › simulated protoplasts/nottouching/nottouching003.png]

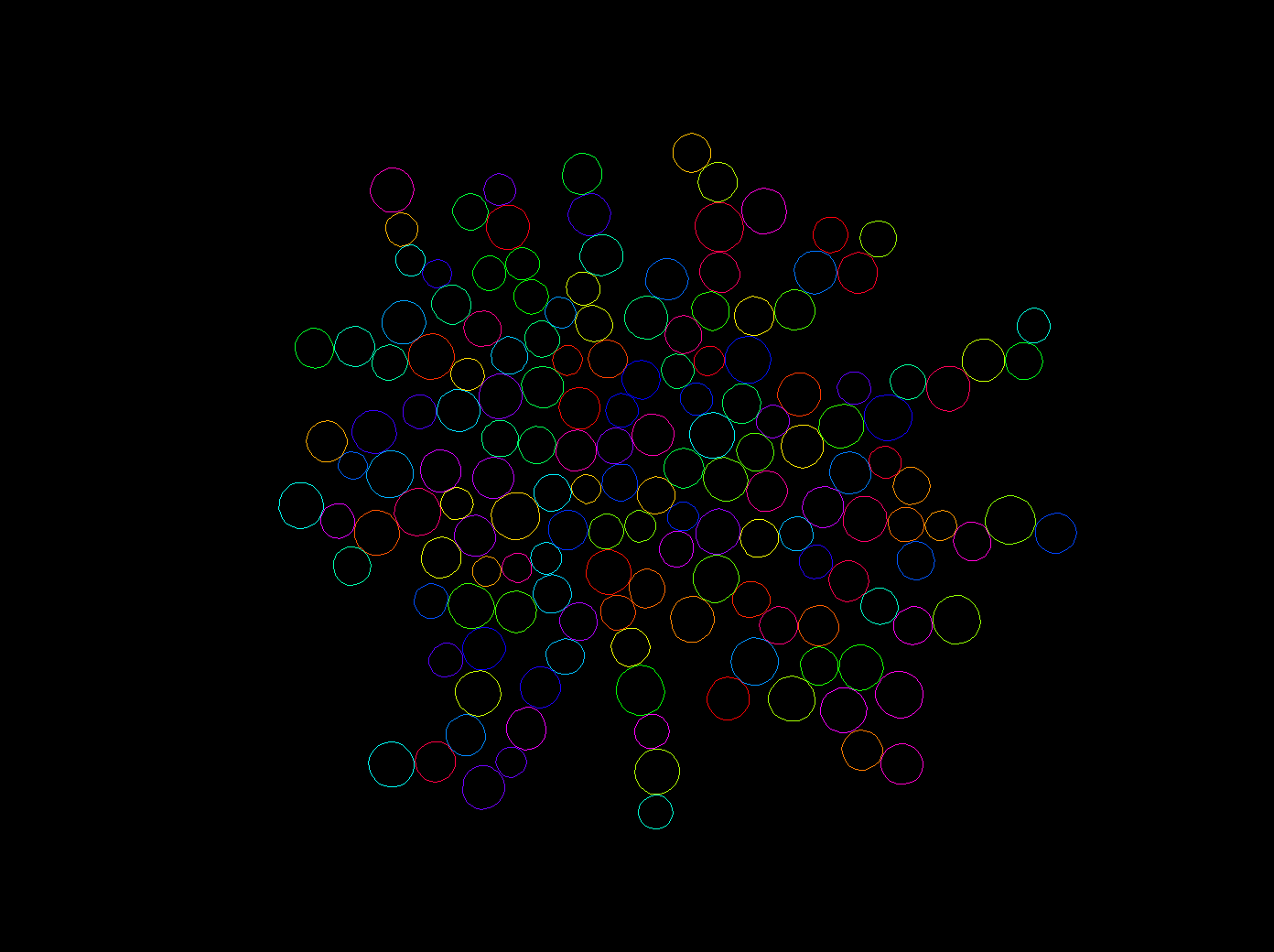

Supplement: Additional file 5 — The zip archive contains simulated images showing protoplasts with corresponding ground truth. (ZIP 72704 kb) [file 12859_2017_1591_MOESM5_ESM.zip › simulated protoplasts/nottouching/nottouching004 gt.png]

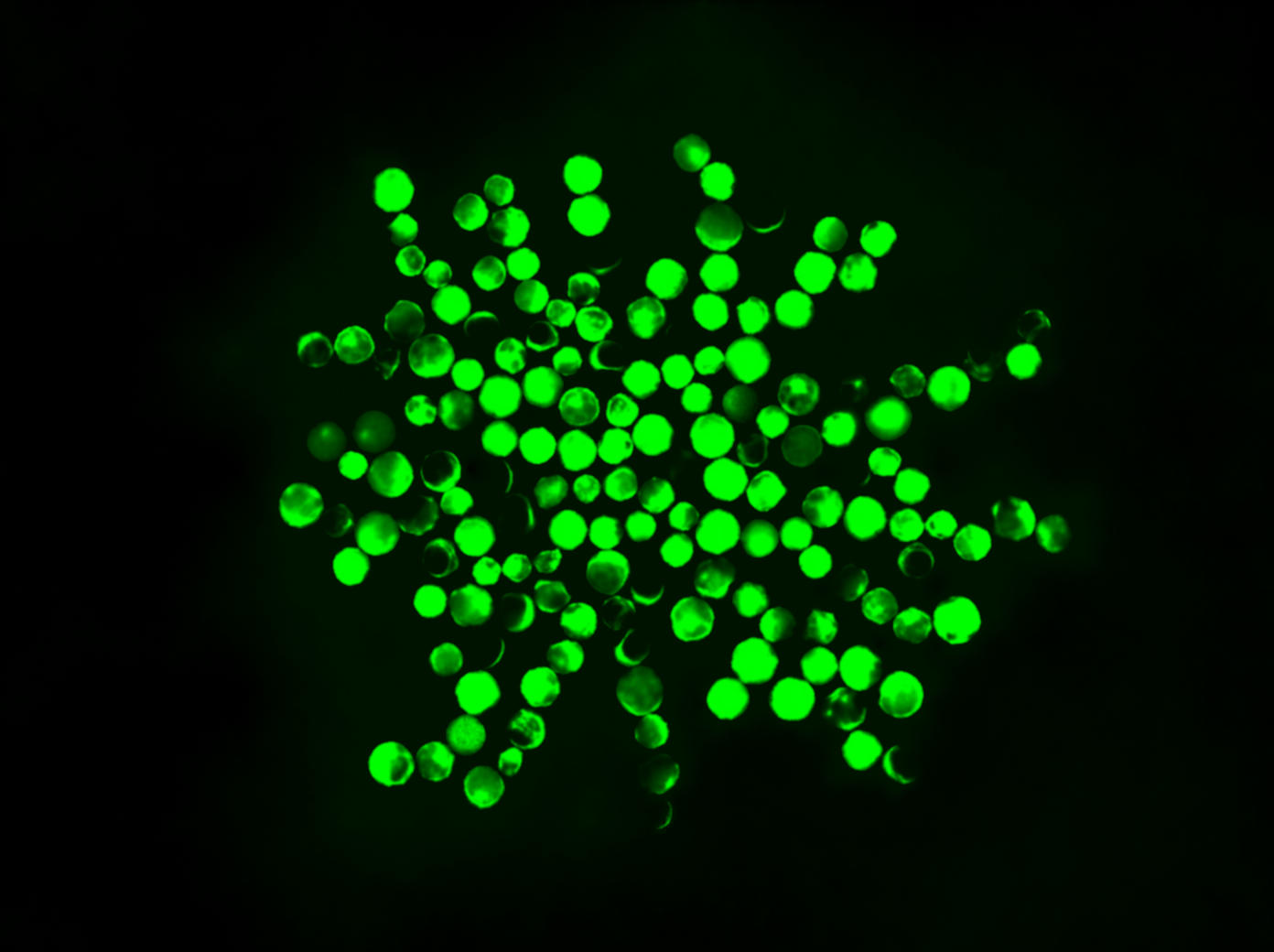

Supplement: Additional file 5 — The zip archive contains simulated images showing protoplasts with corresponding ground truth. (ZIP 72704 kb) [file 12859_2017_1591_MOESM5_ESM.zip › simulated protoplasts/nottouching/nottouching004.png]

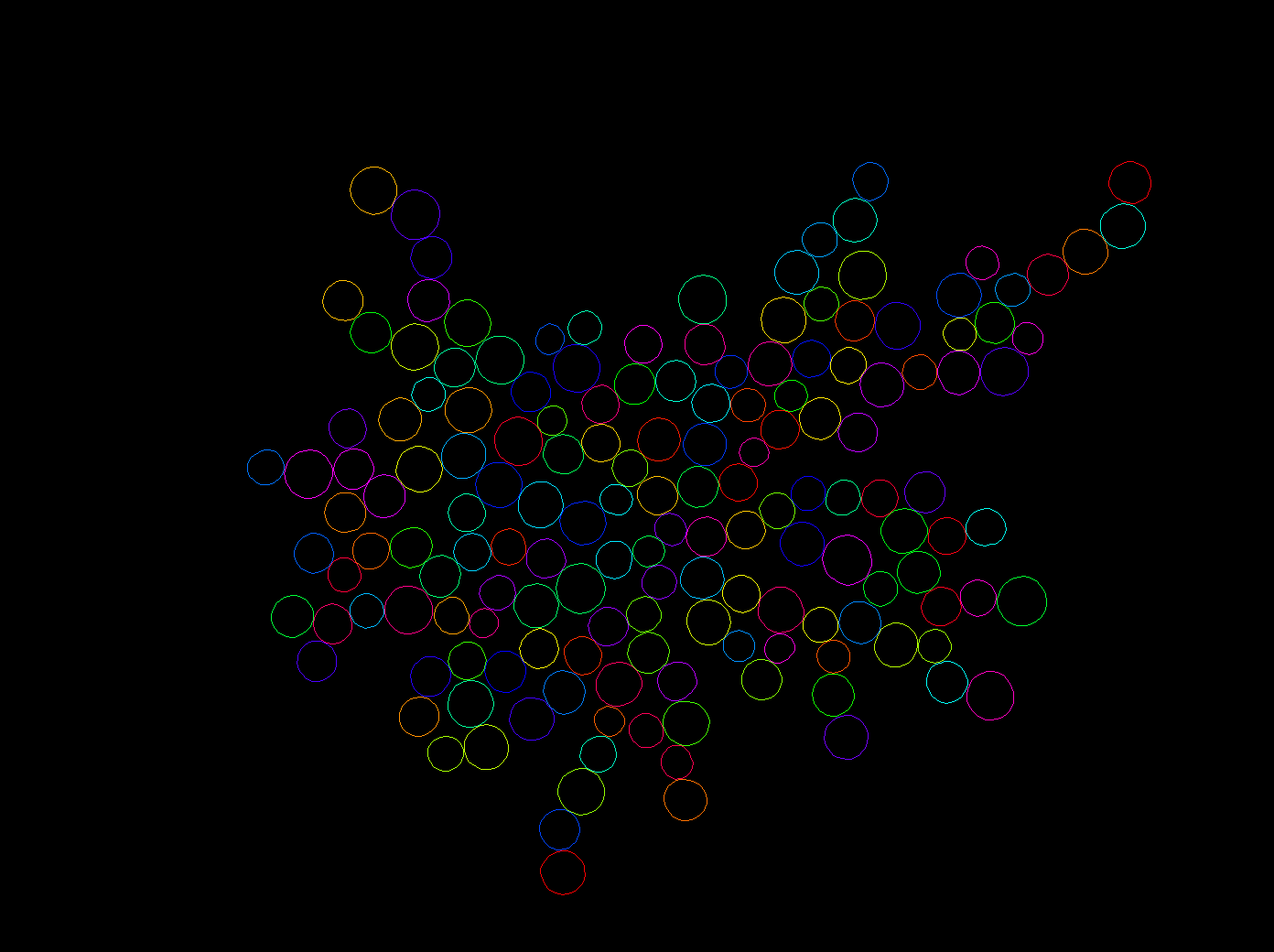

Supplement: Additional file 5 — The zip archive contains simulated images showing protoplasts with corresponding ground truth. (ZIP 72704 kb) [file 12859_2017_1591_MOESM5_ESM.zip › simulated protoplasts/nottouching/nottouching005 gt.png]

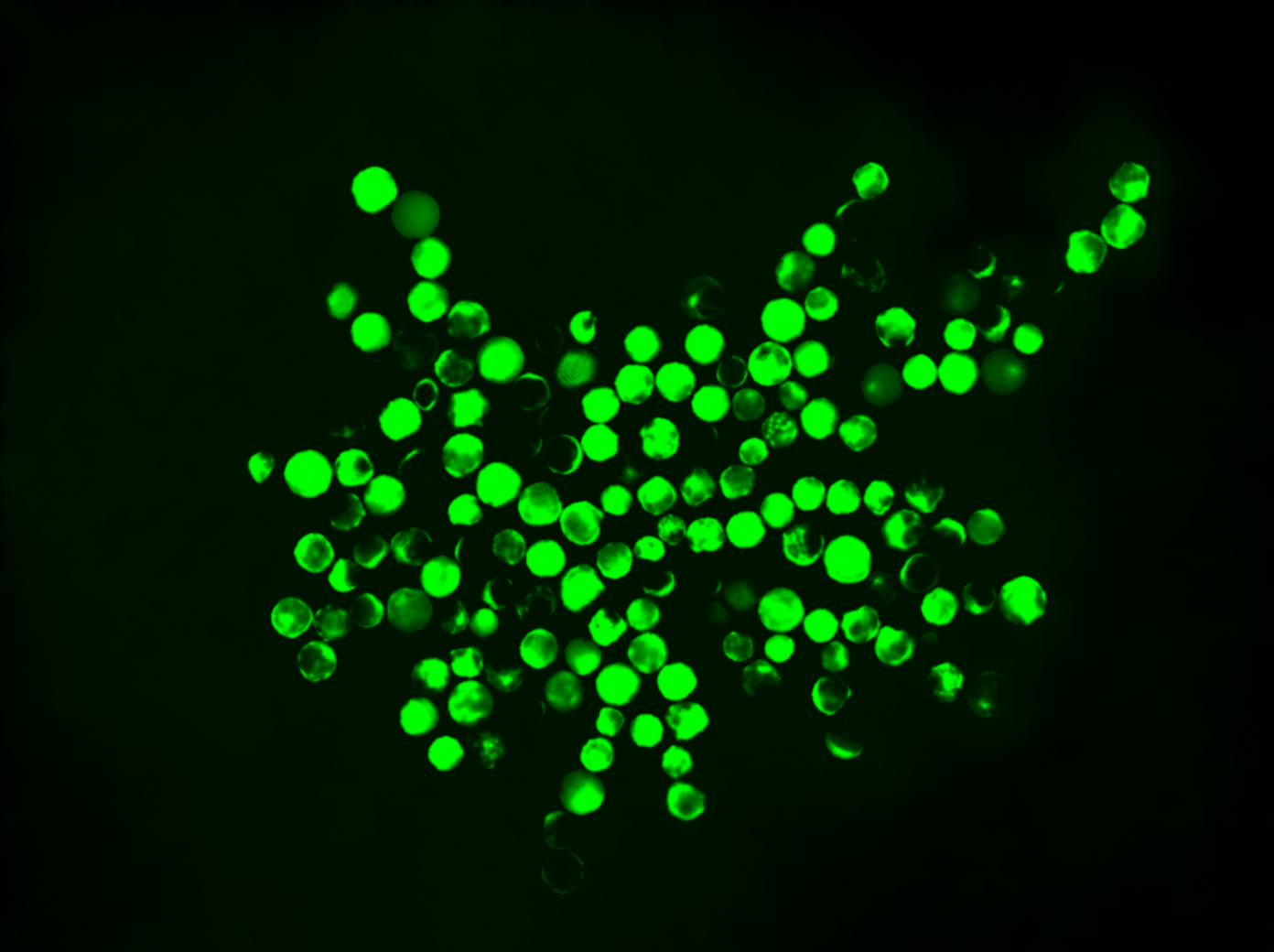

Supplement: Additional file 5 — The zip archive contains simulated images showing protoplasts with corresponding ground truth. (ZIP 72704 kb) [file 12859_2017_1591_MOESM5_ESM.zip › simulated protoplasts/nottouching/nottouching005.png]

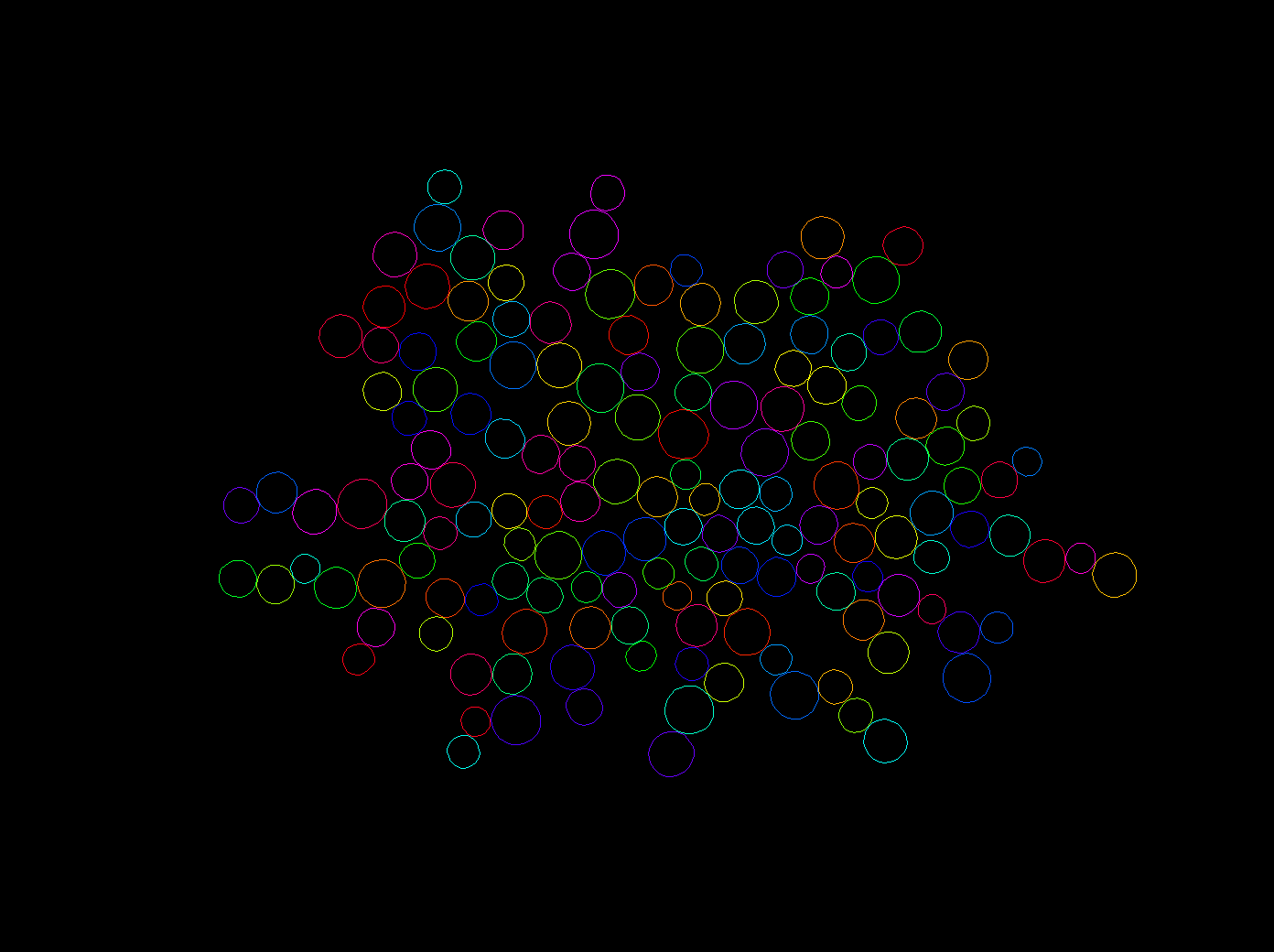

Supplement: Additional file 5 — The zip archive contains simulated images showing protoplasts with corresponding ground truth. (ZIP 72704 kb) [file 12859_2017_1591_MOESM5_ESM.zip › simulated protoplasts/nottouching/nottouching006 gt.png]

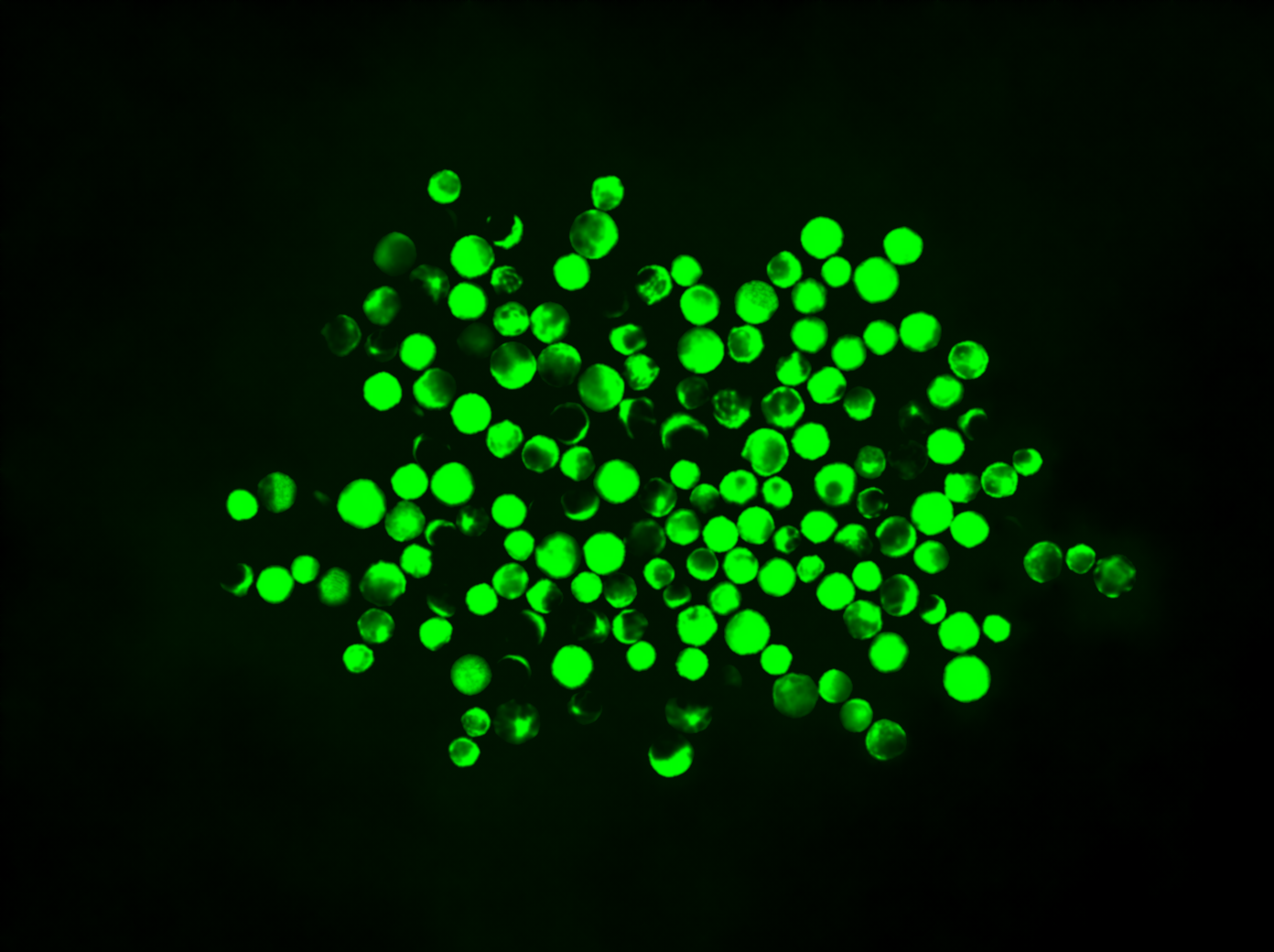

Supplement: Additional file 5 — The zip archive contains simulated images showing protoplasts with corresponding ground truth. (ZIP 72704 kb) [file 12859_2017_1591_MOESM5_ESM.zip › simulated protoplasts/nottouching/nottouching006.png]

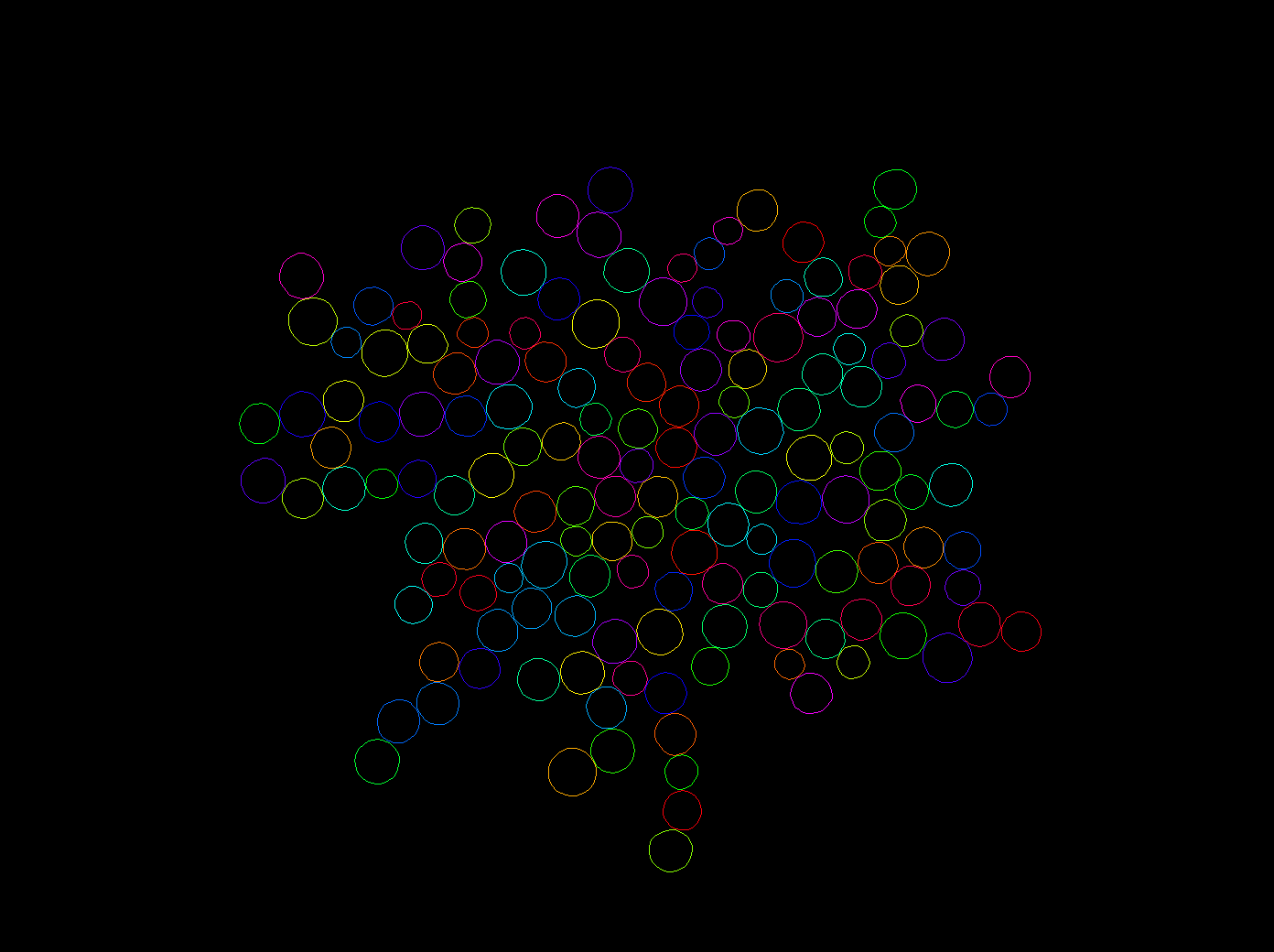

Supplement: Additional file 5 — The zip archive contains simulated images showing protoplasts with corresponding ground truth. (ZIP 72704 kb) [file 12859_2017_1591_MOESM5_ESM.zip › simulated protoplasts/nottouching/nottouching007 gt.png]

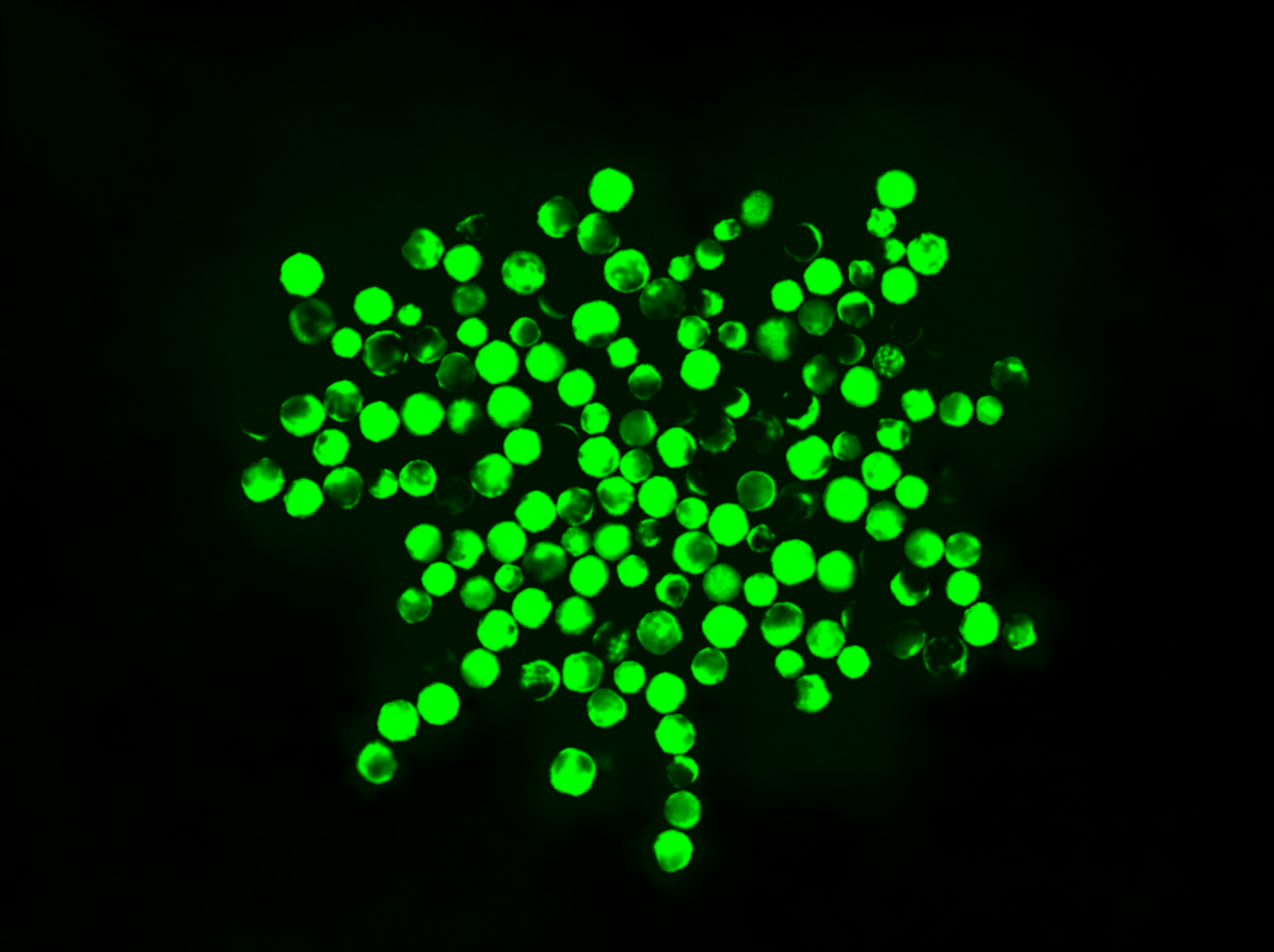

Supplement: Additional file 5 — The zip archive contains simulated images showing protoplasts with corresponding ground truth. (ZIP 72704 kb) [file 12859_2017_1591_MOESM5_ESM.zip › simulated protoplasts/nottouching/nottouching007.png]

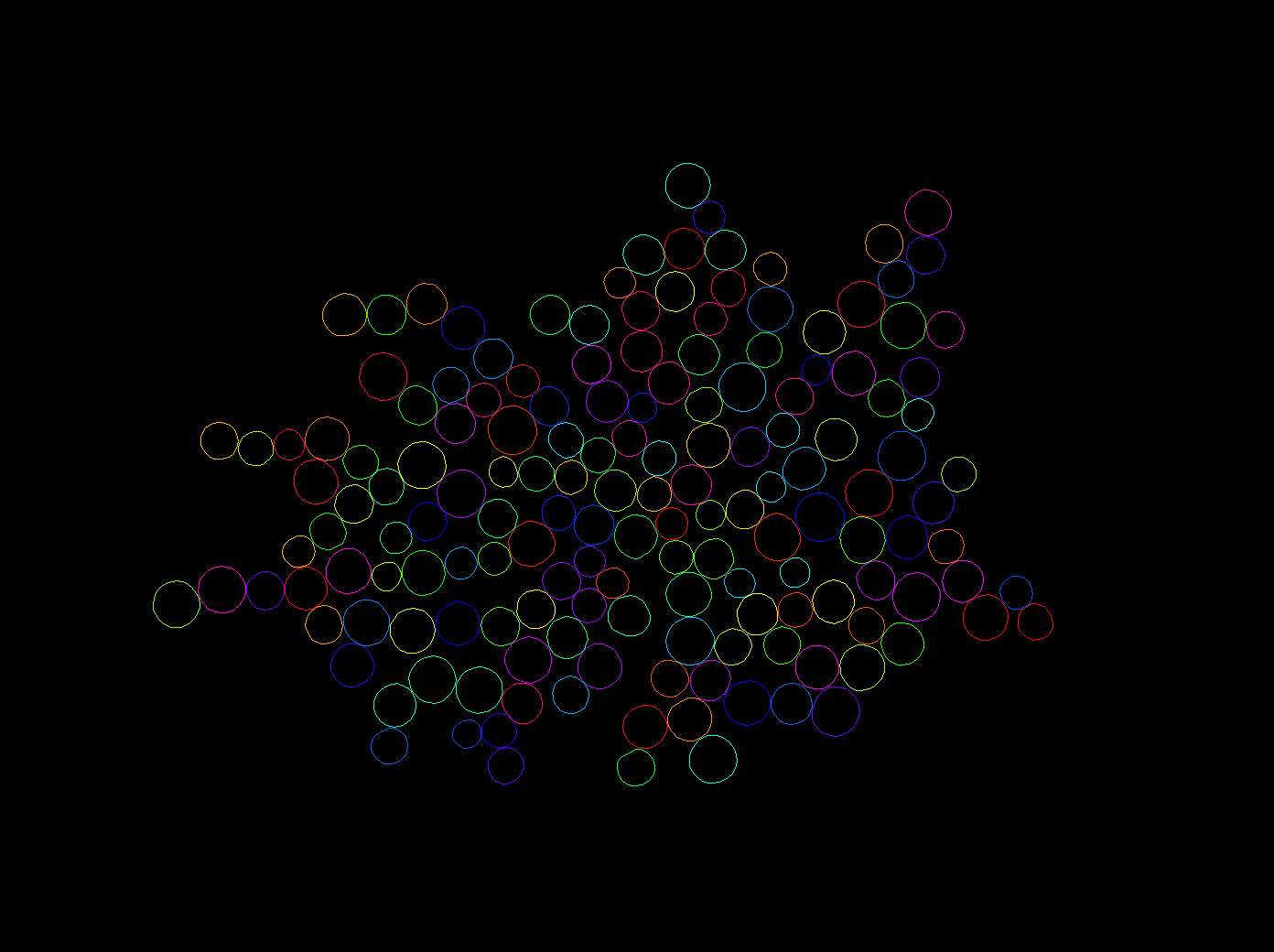

Supplement: Additional file 5 — The zip archive contains simulated images showing protoplasts with corresponding ground truth. (ZIP 72704 kb) [file 12859_2017_1591_MOESM5_ESM.zip › simulated protoplasts/nottouching/nottouching008 gt.png]

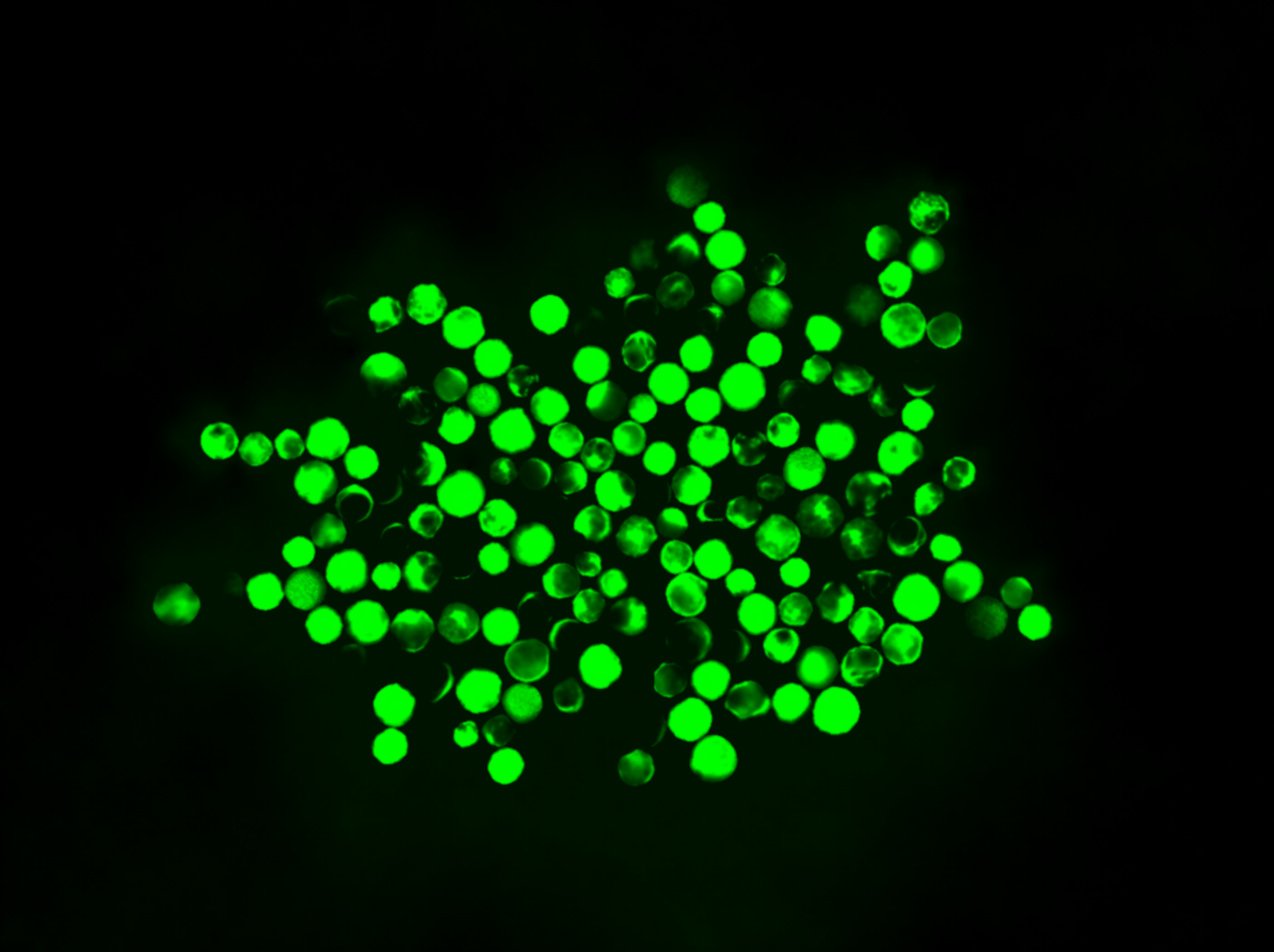

Supplement: Additional file 5 — The zip archive contains simulated images showing protoplasts with corresponding ground truth. (ZIP 72704 kb) [file 12859_2017_1591_MOESM5_ESM.zip › simulated protoplasts/nottouching/nottouching008.png]

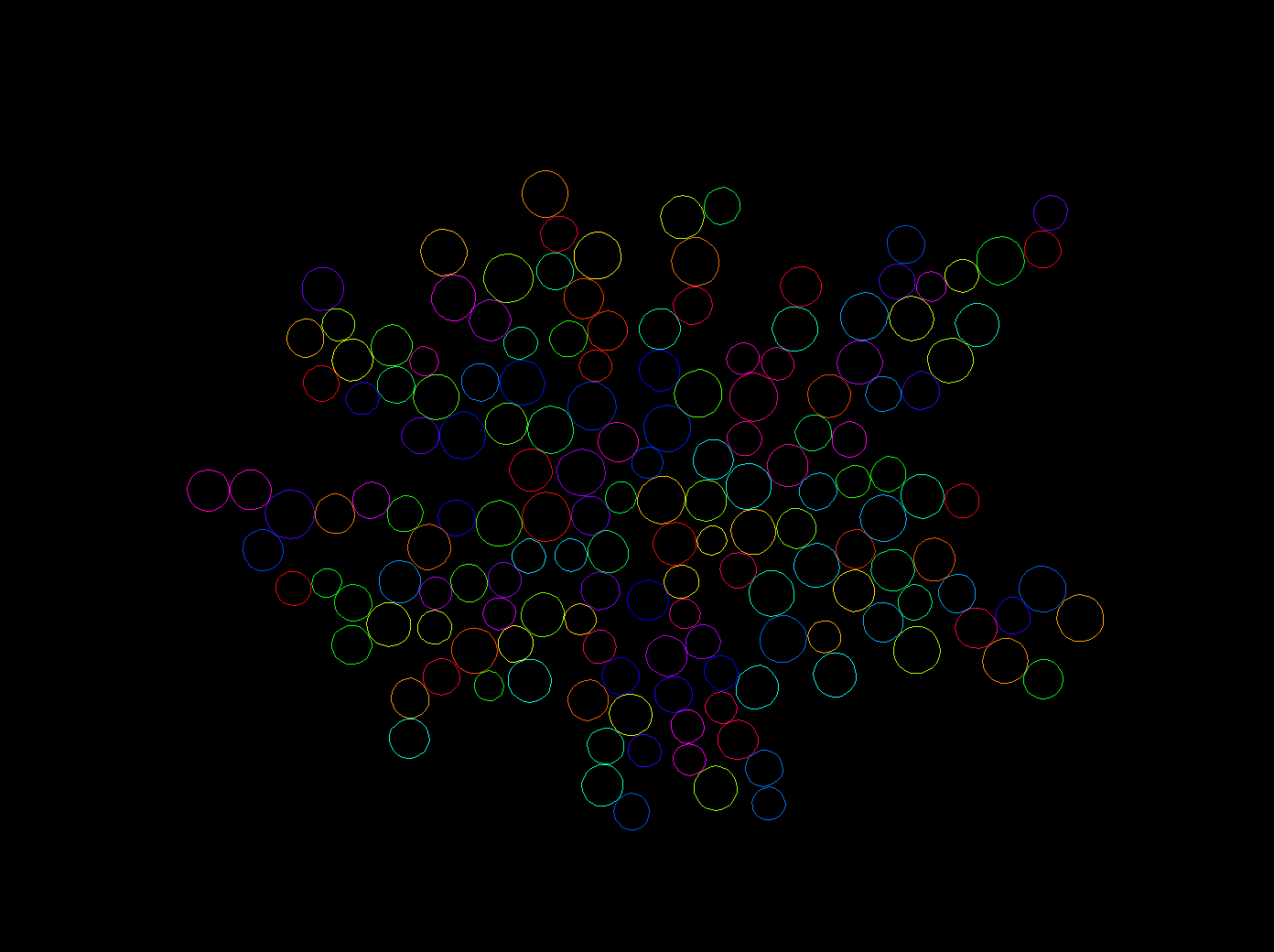

Supplement: Additional file 5 — The zip archive contains simulated images showing protoplasts with corresponding ground truth. (ZIP 72704 kb) [file 12859_2017_1591_MOESM5_ESM.zip › simulated protoplasts/nottouching/nottouching009 gt.png]

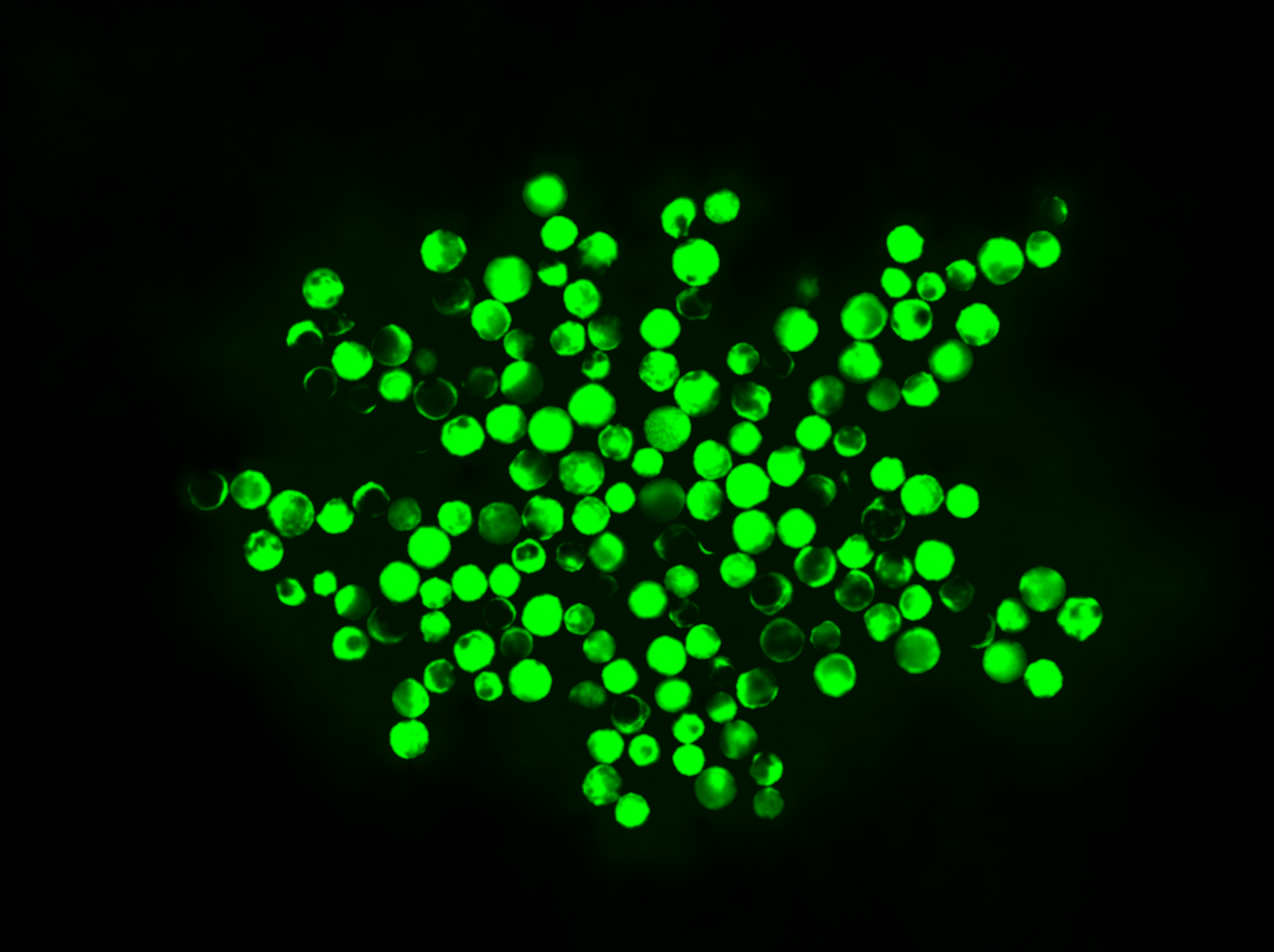

Supplement: Additional file 5 — The zip archive contains simulated images showing protoplasts with corresponding ground truth. (ZIP 72704 kb) [file 12859_2017_1591_MOESM5_ESM.zip › simulated protoplasts/nottouching/nottouching009.png]

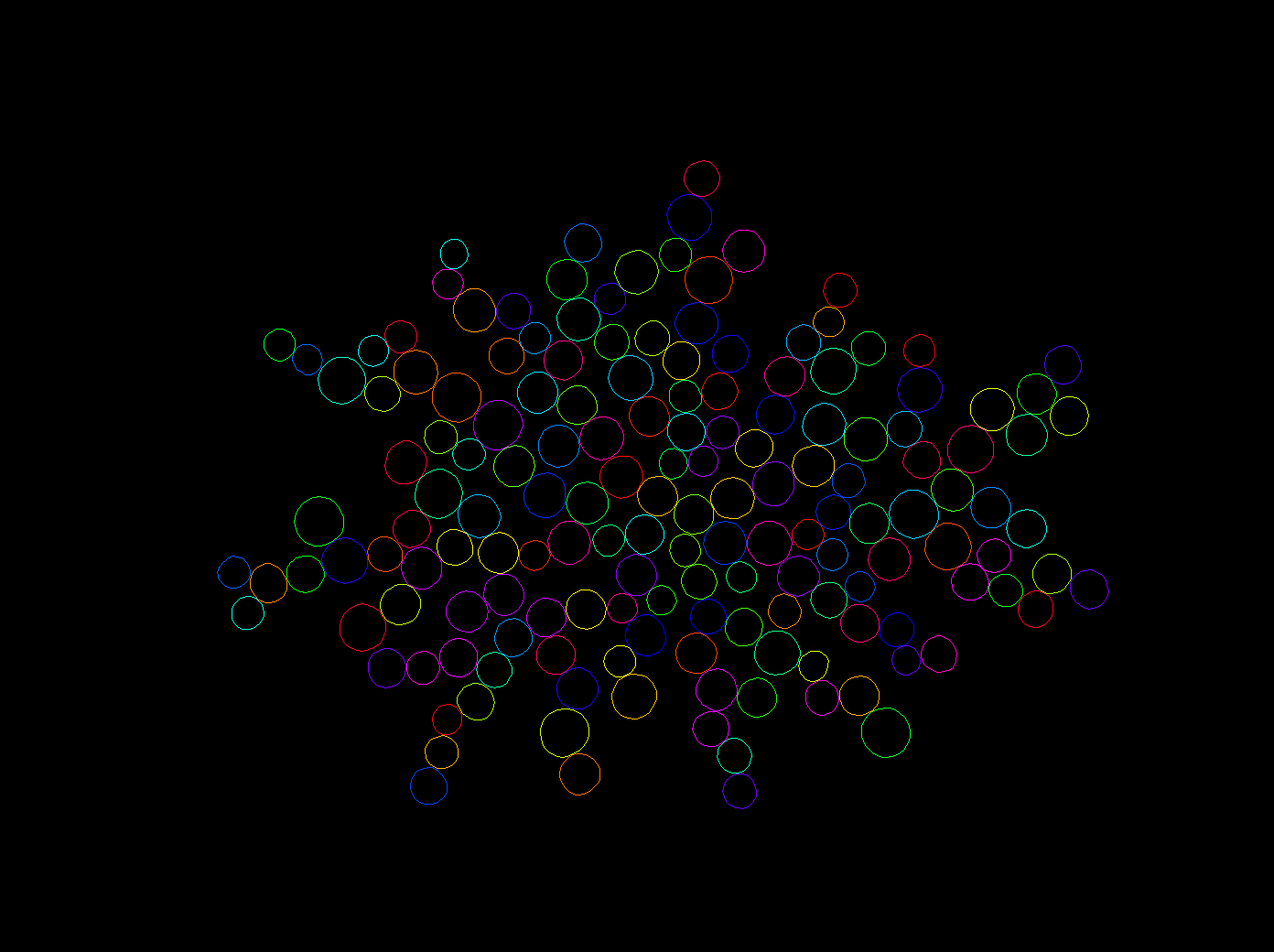

Supplement: Additional file 5 — The zip archive contains simulated images showing protoplasts with corresponding ground truth. (ZIP 72704 kb) [file 12859_2017_1591_MOESM5_ESM.zip › simulated protoplasts/nottouching/nottouching010 gt.png]

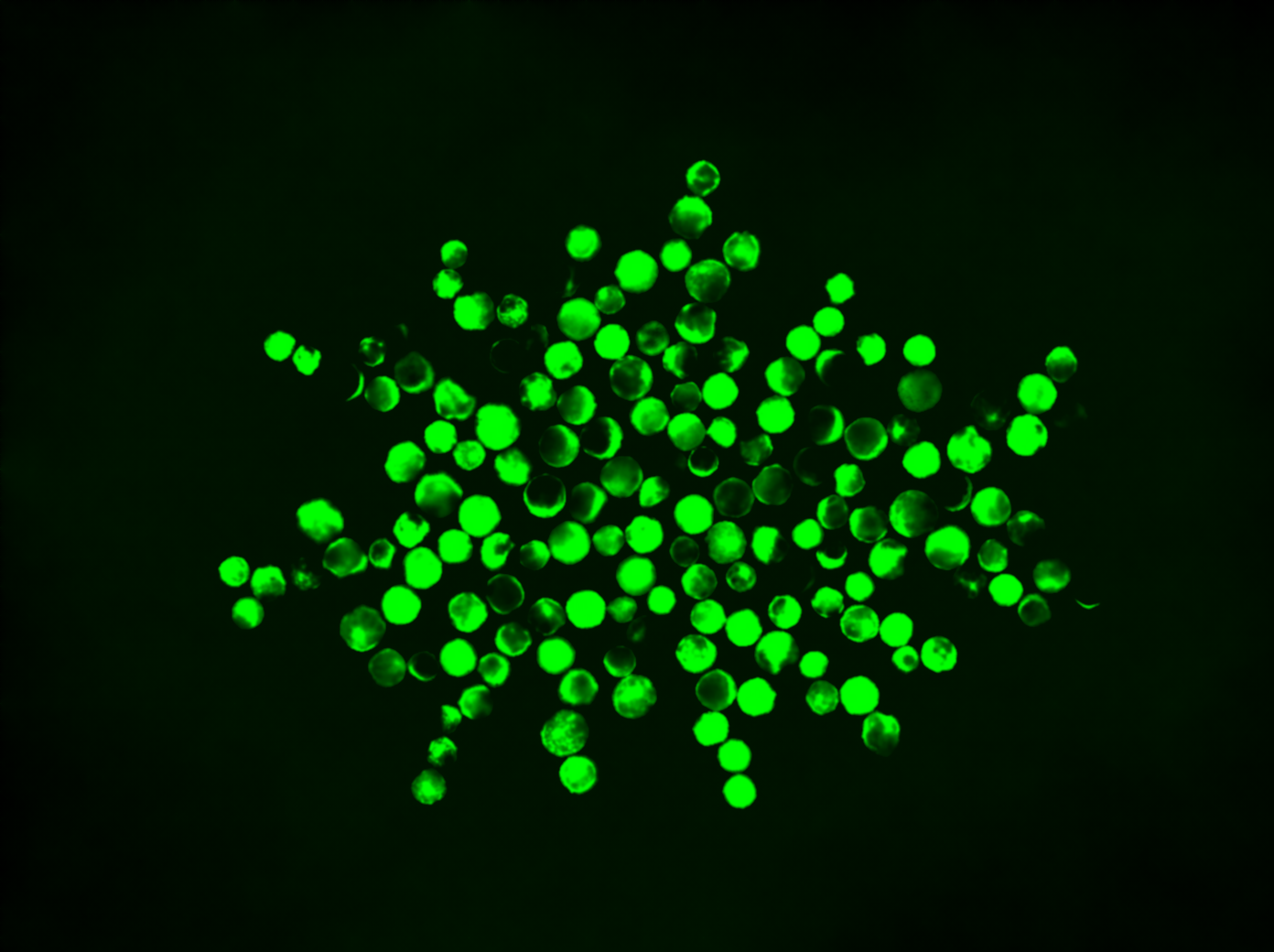

Supplement: Additional file 5 — The zip archive contains simulated images showing protoplasts with corresponding ground truth. (ZIP 72704 kb) [file 12859_2017_1591_MOESM5_ESM.zip › simulated protoplasts/nottouching/nottouching010.png]

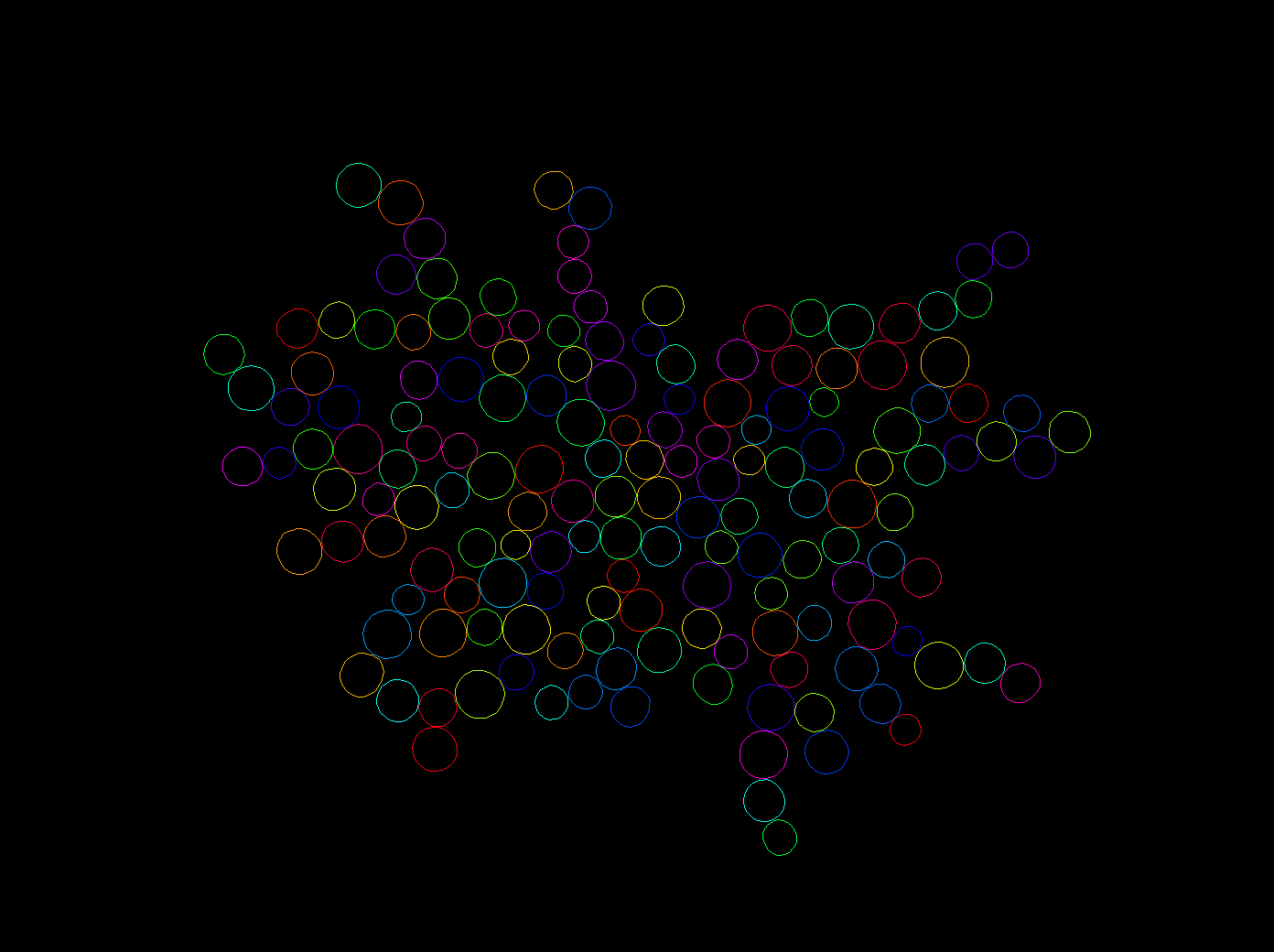

Supplement: Additional file 5 — The zip archive contains simulated images showing protoplasts with corresponding ground truth. (ZIP 72704 kb) [file 12859_2017_1591_MOESM5_ESM.zip › simulated protoplasts/nottouching/nottouching011 gt.png]

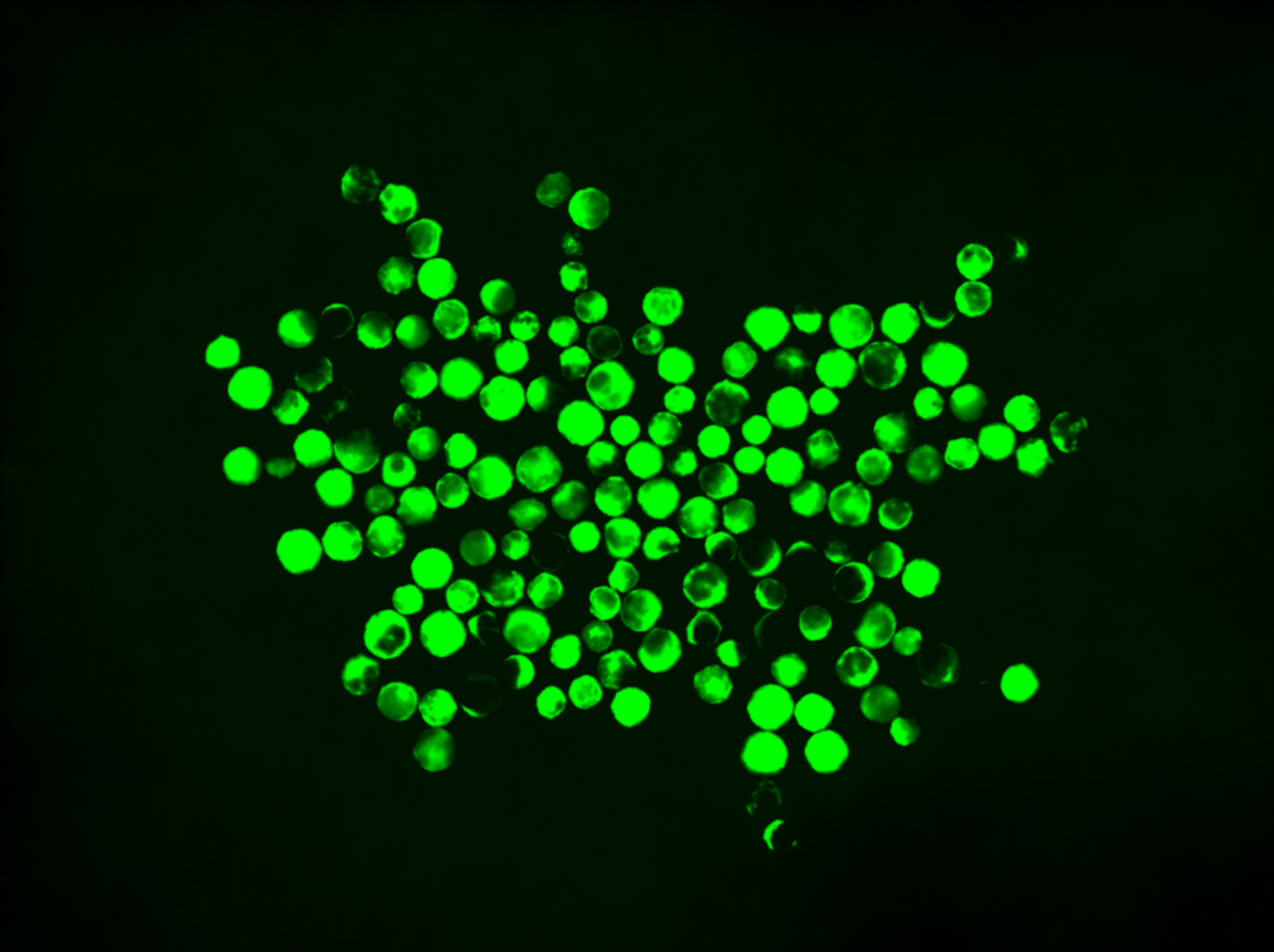

Supplement: Additional file 5 — The zip archive contains simulated images showing protoplasts with corresponding ground truth. (ZIP 72704 kb) [file 12859_2017_1591_MOESM5_ESM.zip › simulated protoplasts/nottouching/nottouching011.png]

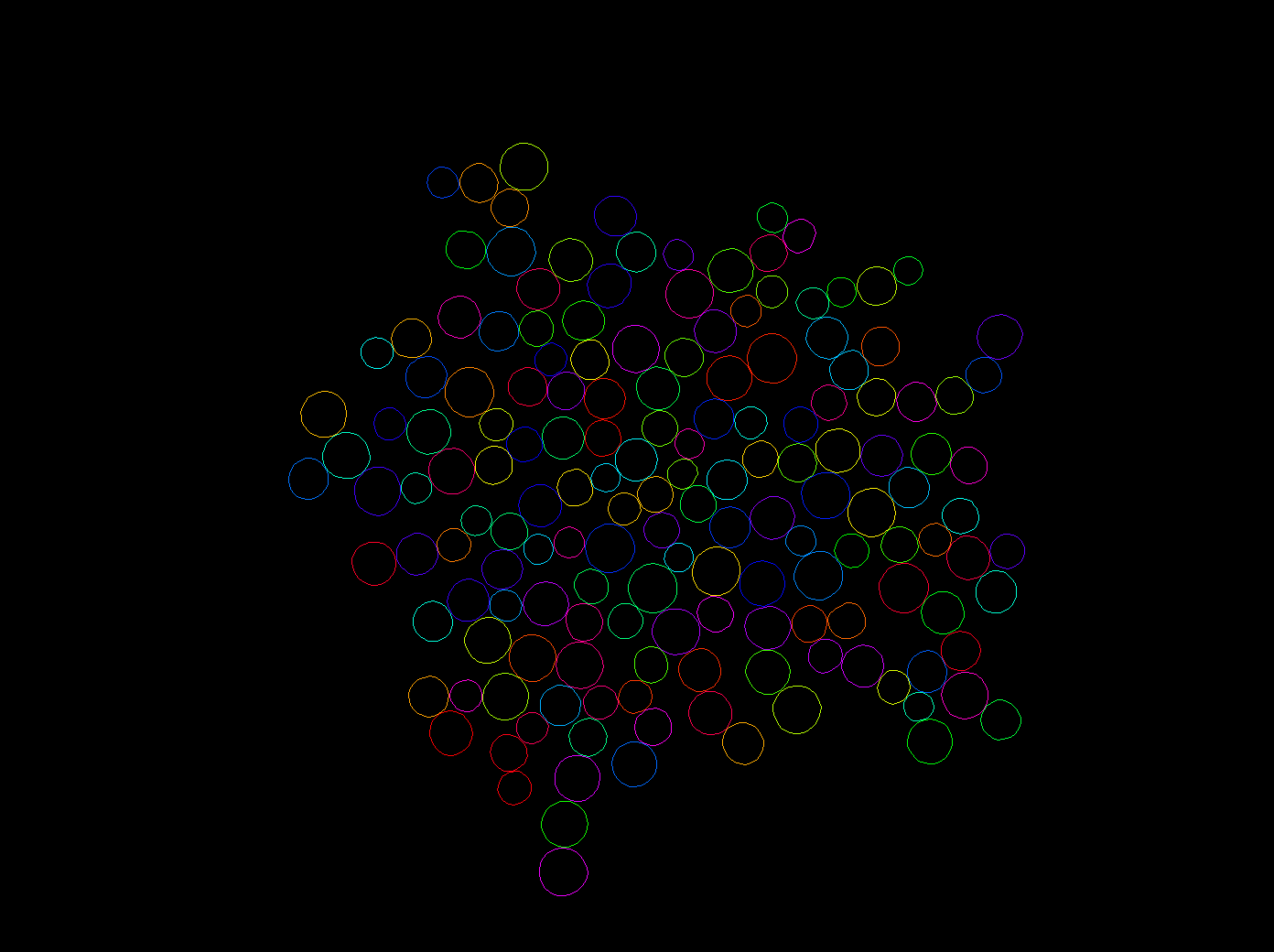

Supplement: Additional file 5 — The zip archive contains simulated images showing protoplasts with corresponding ground truth. (ZIP 72704 kb) [file 12859_2017_1591_MOESM5_ESM.zip › simulated protoplasts/nottouching/nottouching012 gt.png]

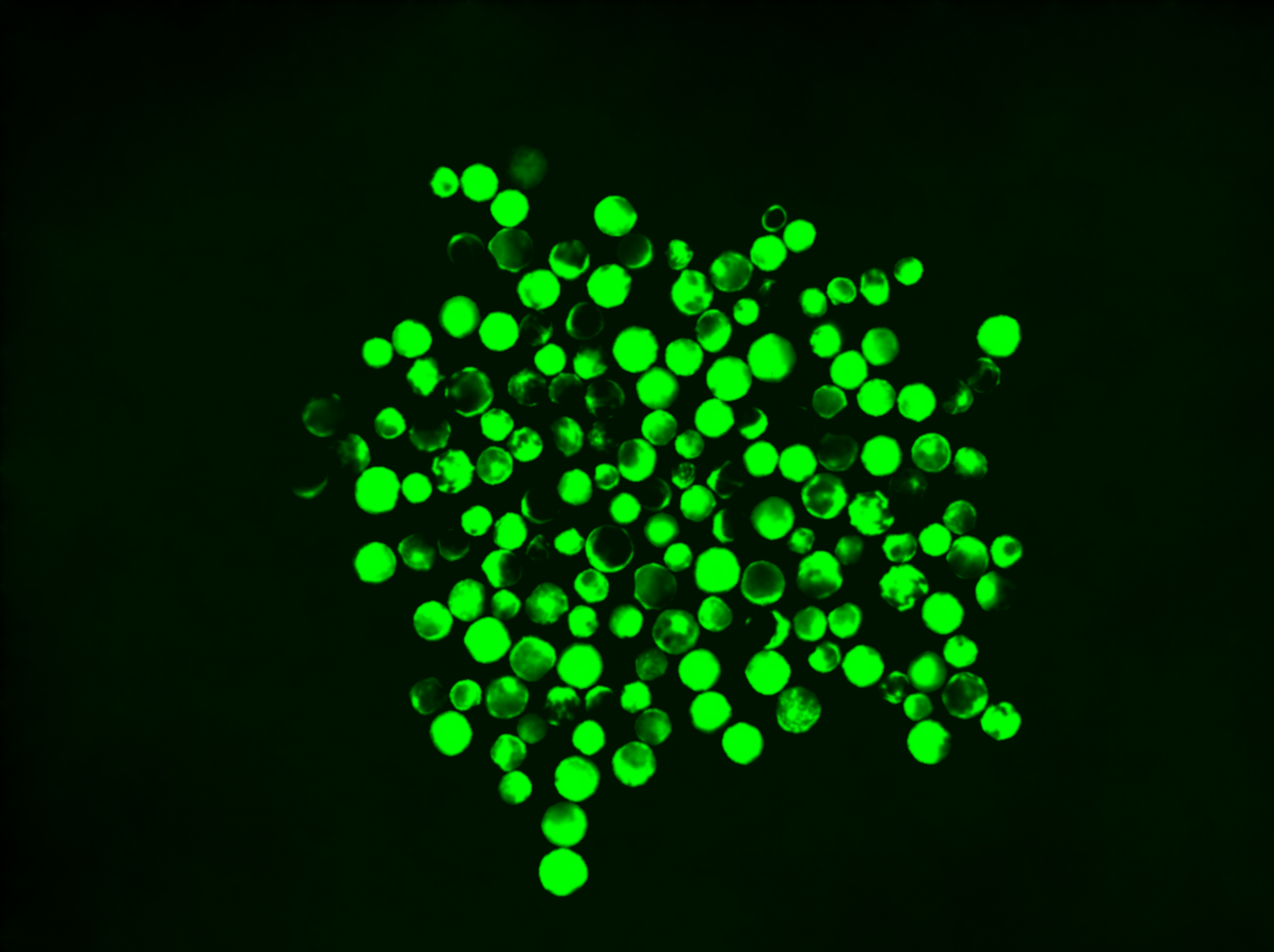

Supplement: Additional file 5 — The zip archive contains simulated images showing protoplasts with corresponding ground truth. (ZIP 72704 kb) [file 12859_2017_1591_MOESM5_ESM.zip › simulated protoplasts/nottouching/nottouching012.png]

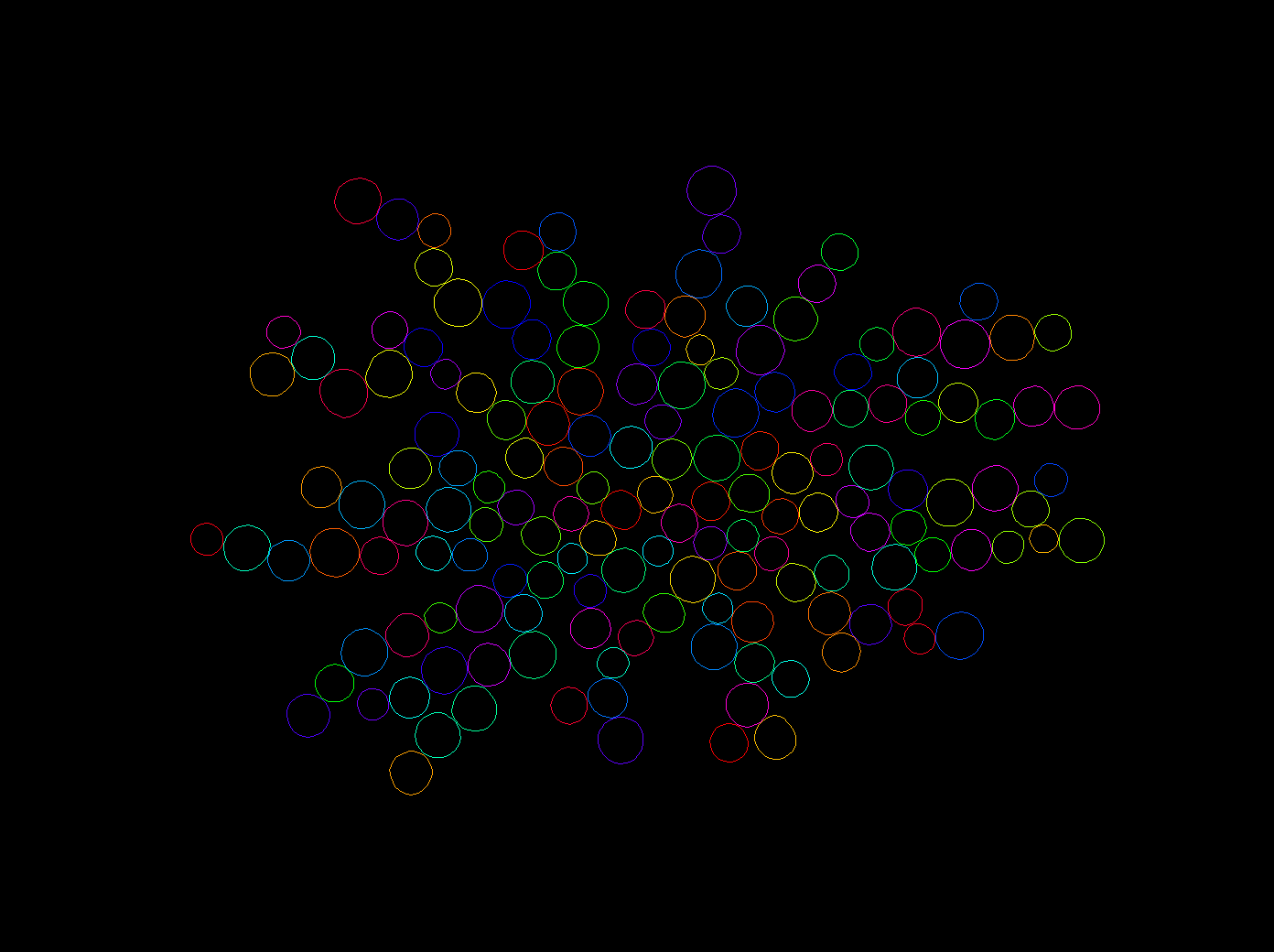

Supplement: Additional file 5 — The zip archive contains simulated images showing protoplasts with corresponding ground truth. (ZIP 72704 kb) [file 12859_2017_1591_MOESM5_ESM.zip › simulated protoplasts/nottouching/nottouching013 gt.png]

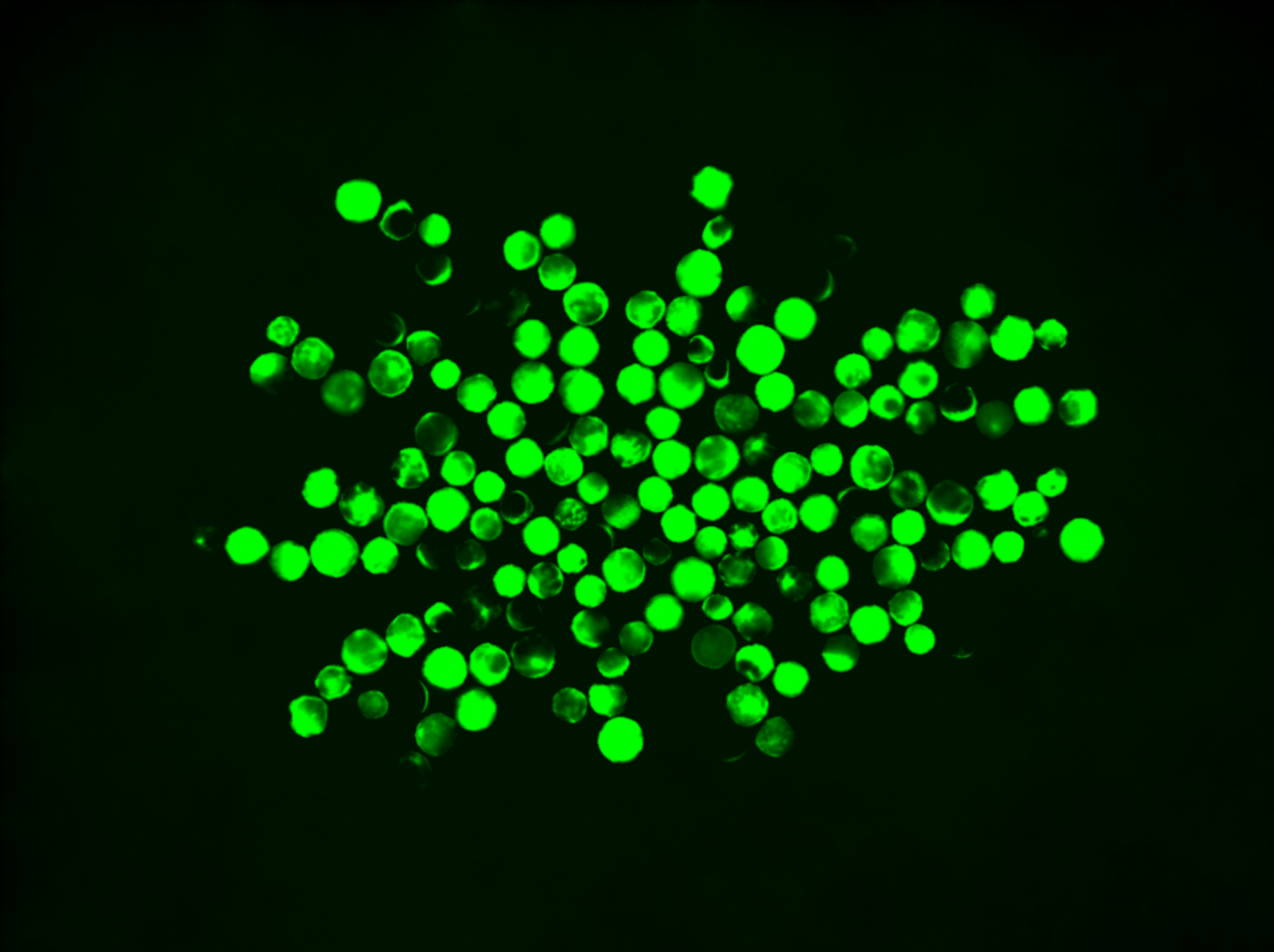

Supplement: Additional file 5 — The zip archive contains simulated images showing protoplasts with corresponding ground truth. (ZIP 72704 kb) [file 12859_2017_1591_MOESM5_ESM.zip › simulated protoplasts/nottouching/nottouching013.png]

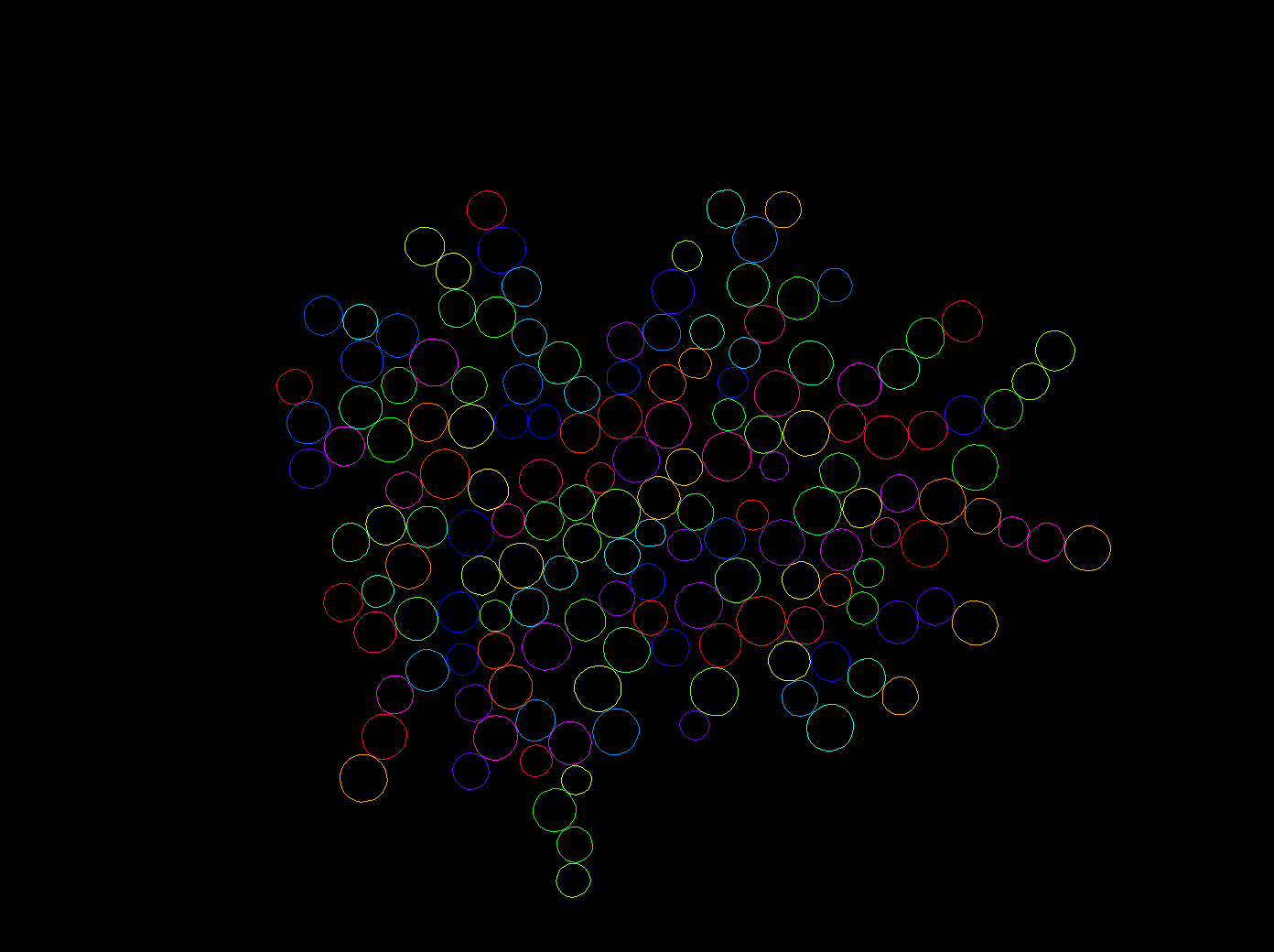

Supplement: Additional file 5 — The zip archive contains simulated images showing protoplasts with corresponding ground truth. (ZIP 72704 kb) [file 12859_2017_1591_MOESM5_ESM.zip › simulated protoplasts/nottouching/nottouching014 gt.png]

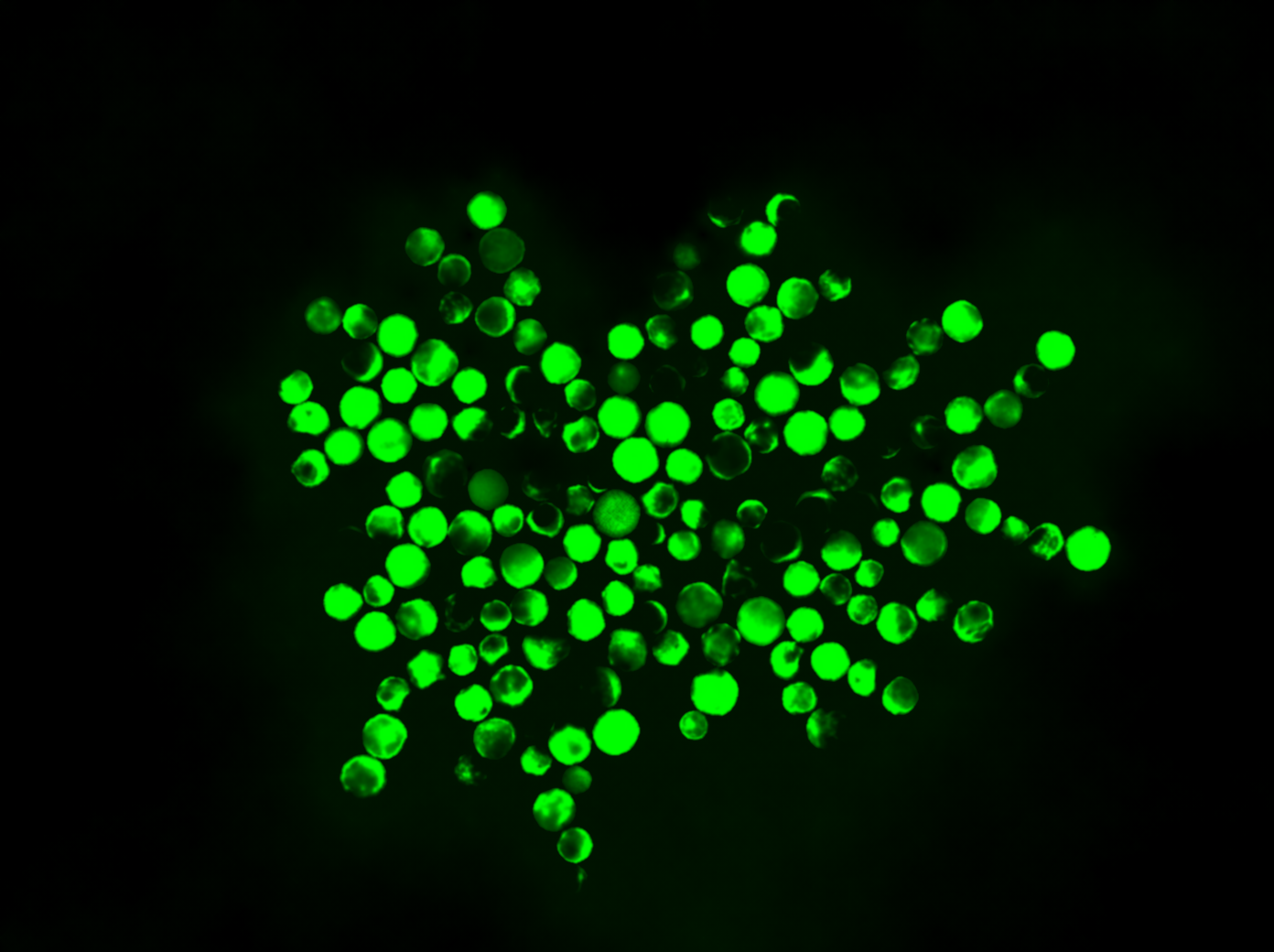

Supplement: Additional file 5 — The zip archive contains simulated images showing protoplasts with corresponding ground truth. (ZIP 72704 kb) [file 12859_2017_1591_MOESM5_ESM.zip › simulated protoplasts/nottouching/nottouching014.png]

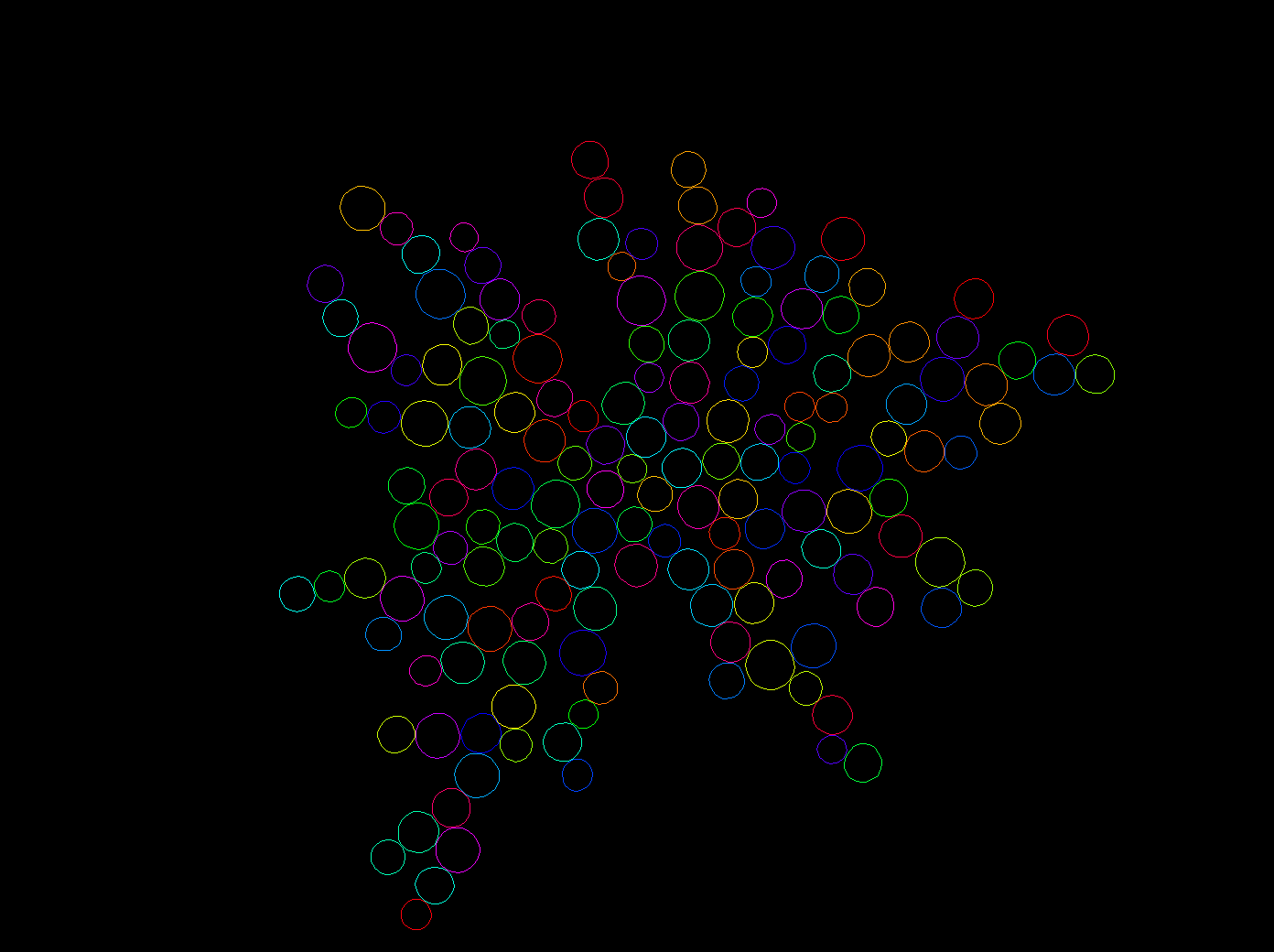

Supplement: Additional file 5 — The zip archive contains simulated images showing protoplasts with corresponding ground truth. (ZIP 72704 kb) [file 12859_2017_1591_MOESM5_ESM.zip › simulated protoplasts/nottouching/nottouching015 gt.png]

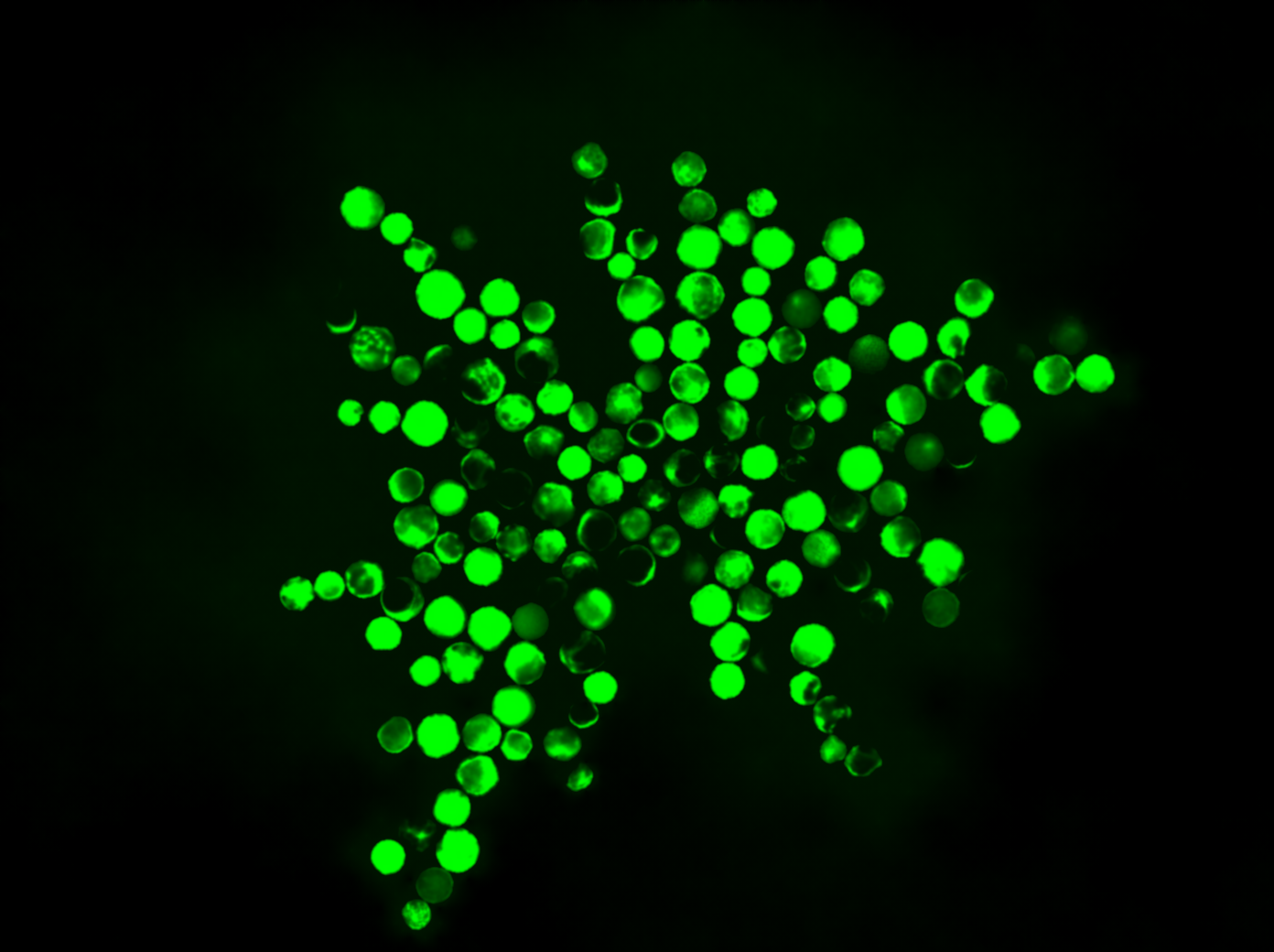

Supplement: Additional file 5 — The zip archive contains simulated images showing protoplasts with corresponding ground truth. (ZIP 72704 kb) [file 12859_2017_1591_MOESM5_ESM.zip › simulated protoplasts/nottouching/nottouching015.png]

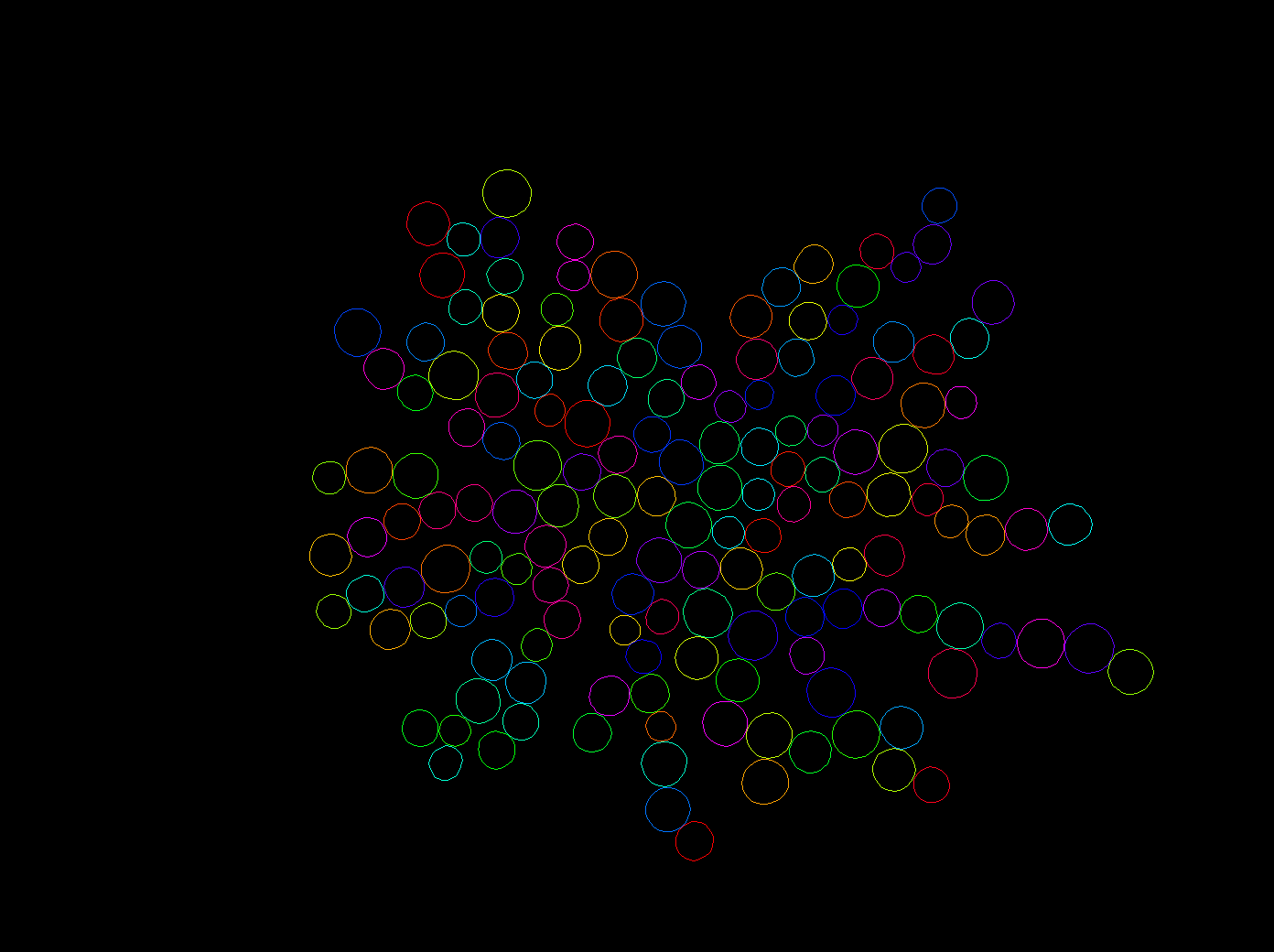

Supplement: Additional file 5 — The zip archive contains simulated images showing protoplasts with corresponding ground truth. (ZIP 72704 kb) [file 12859_2017_1591_MOESM5_ESM.zip › simulated protoplasts/nottouching/nottouching016 gt.png]

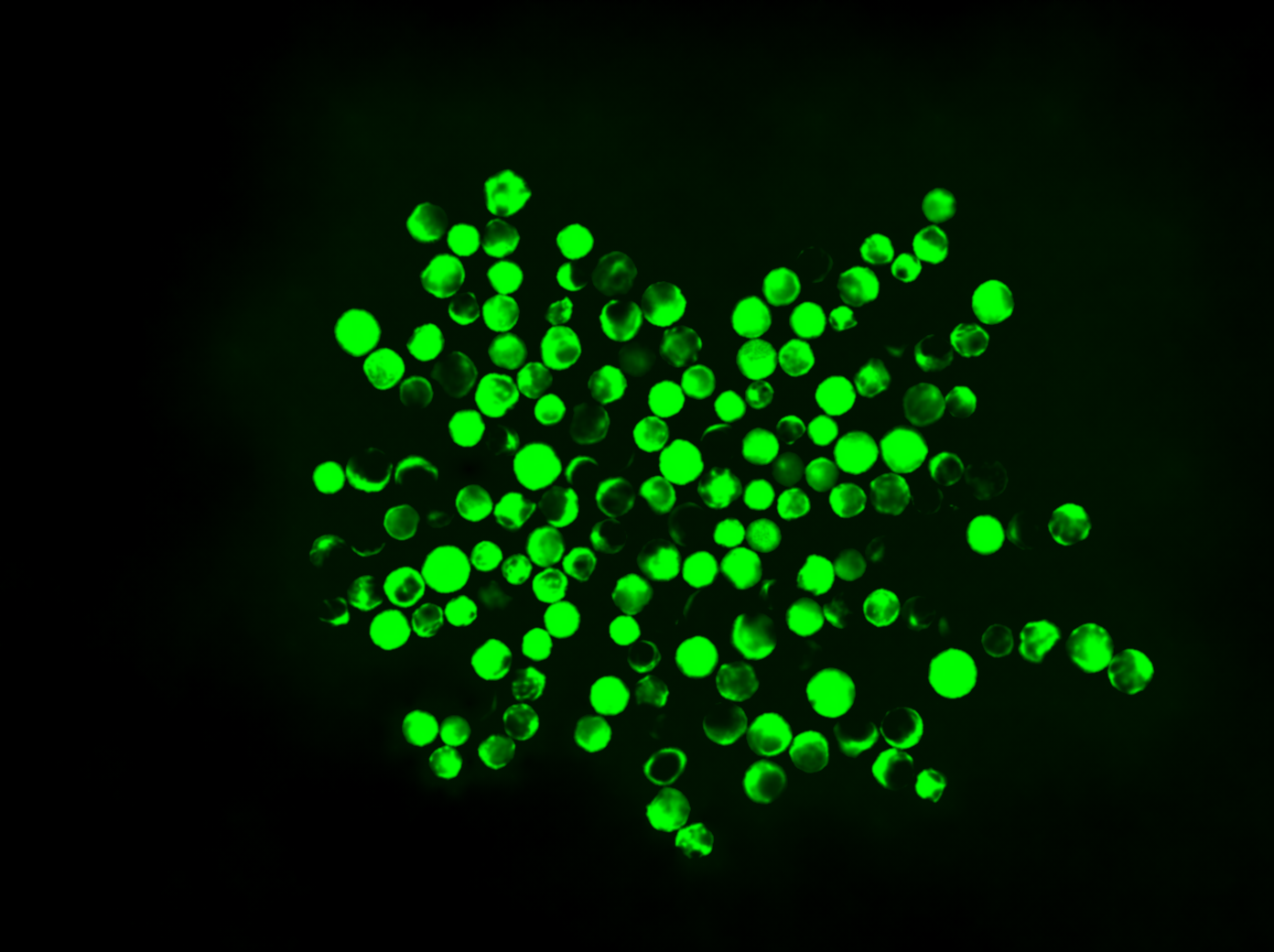

Supplement: Additional file 5 — The zip archive contains simulated images showing protoplasts with corresponding ground truth. (ZIP 72704 kb) [file 12859_2017_1591_MOESM5_ESM.zip › simulated protoplasts/nottouching/nottouching016.png]

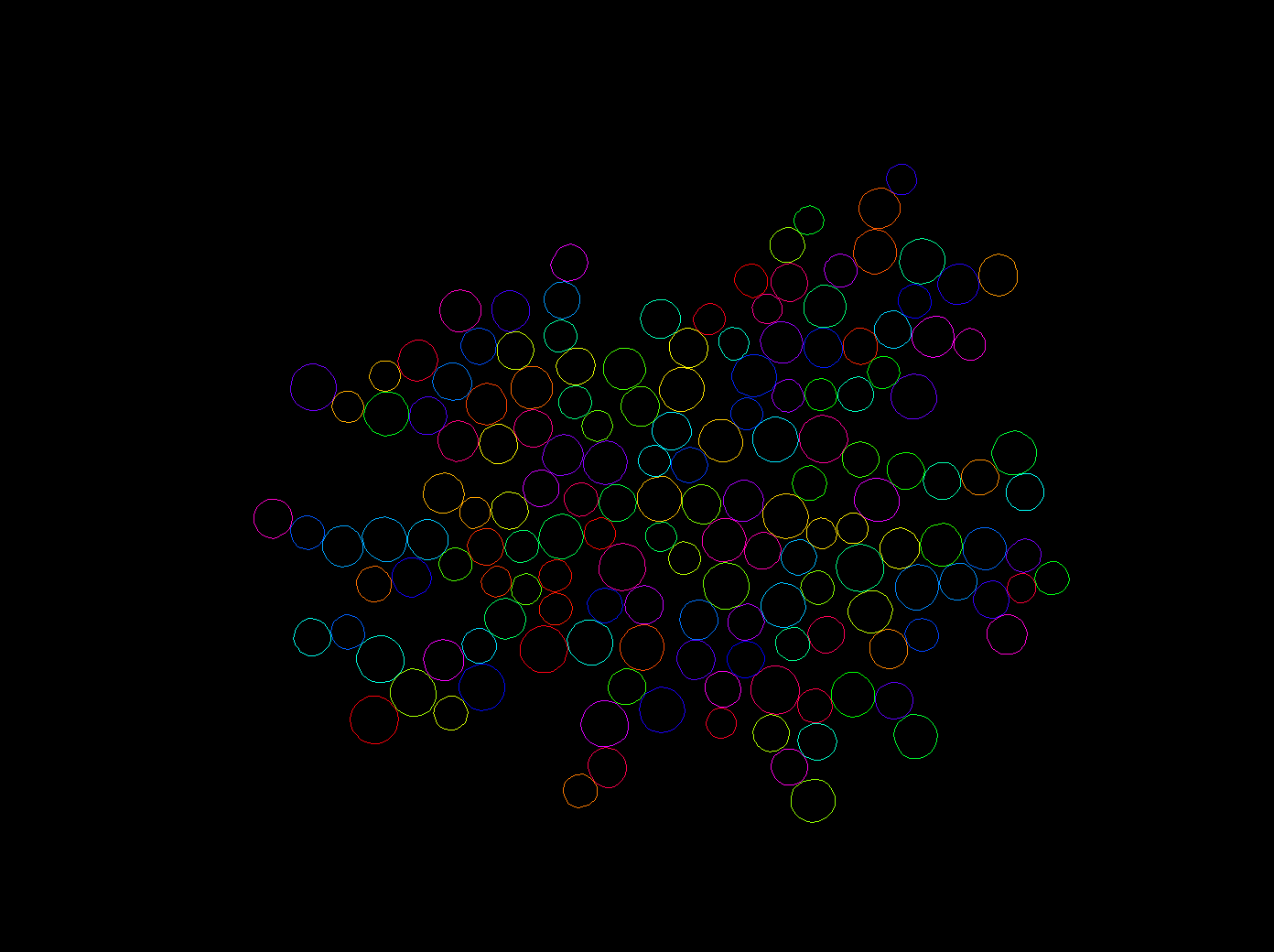

Supplement: Additional file 5 — The zip archive contains simulated images showing protoplasts with corresponding ground truth. (ZIP 72704 kb) [file 12859_2017_1591_MOESM5_ESM.zip › simulated protoplasts/nottouching/nottouching017 gt.png]

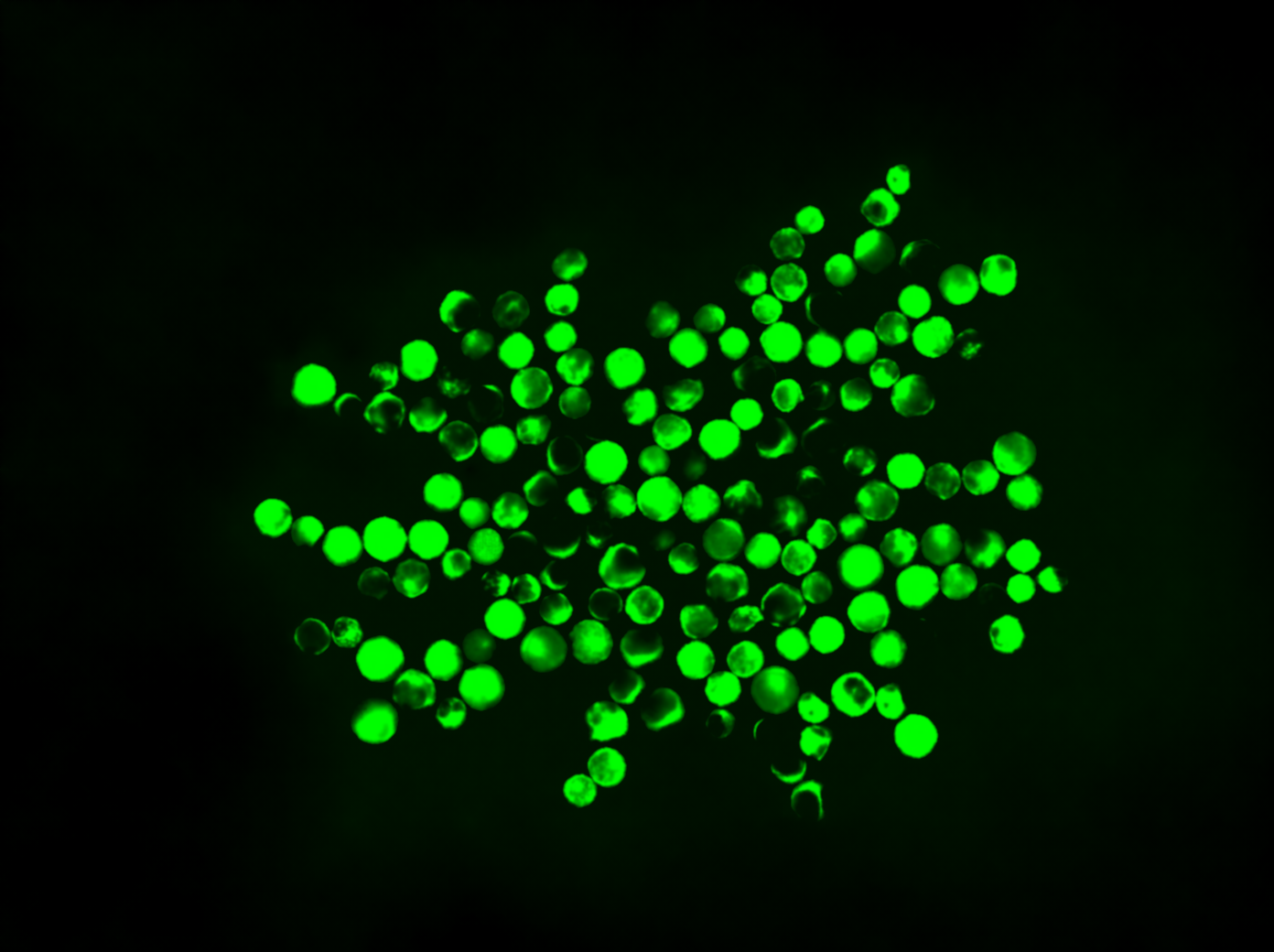

Supplement: Additional file 5 — The zip archive contains simulated images showing protoplasts with corresponding ground truth. (ZIP 72704 kb) [file 12859_2017_1591_MOESM5_ESM.zip › simulated protoplasts/nottouching/nottouching017.png]

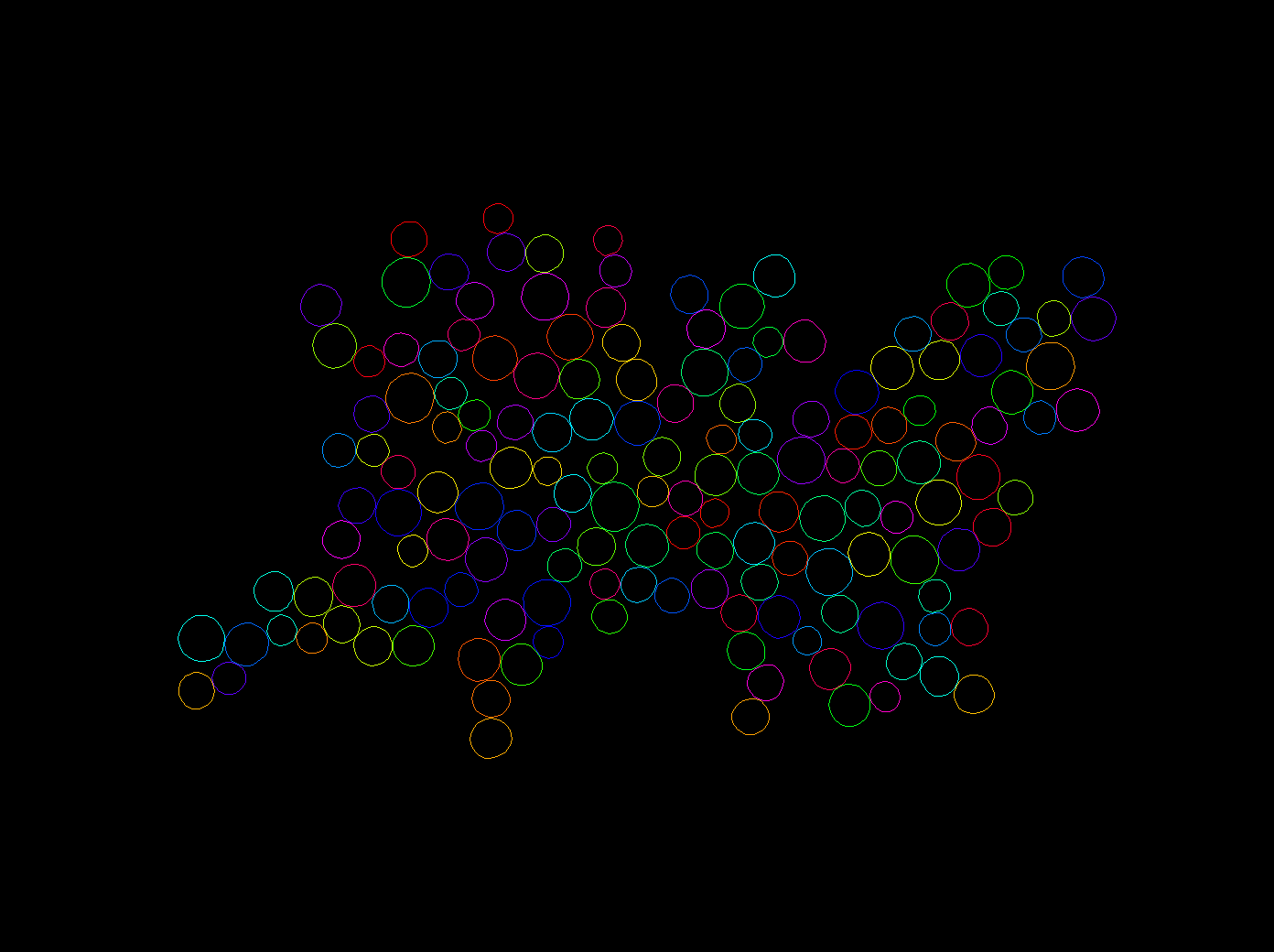

Supplement: Additional file 5 — The zip archive contains simulated images showing protoplasts with corresponding ground truth. (ZIP 72704 kb) [file 12859_2017_1591_MOESM5_ESM.zip › simulated protoplasts/nottouching/nottouching018 gt.png]

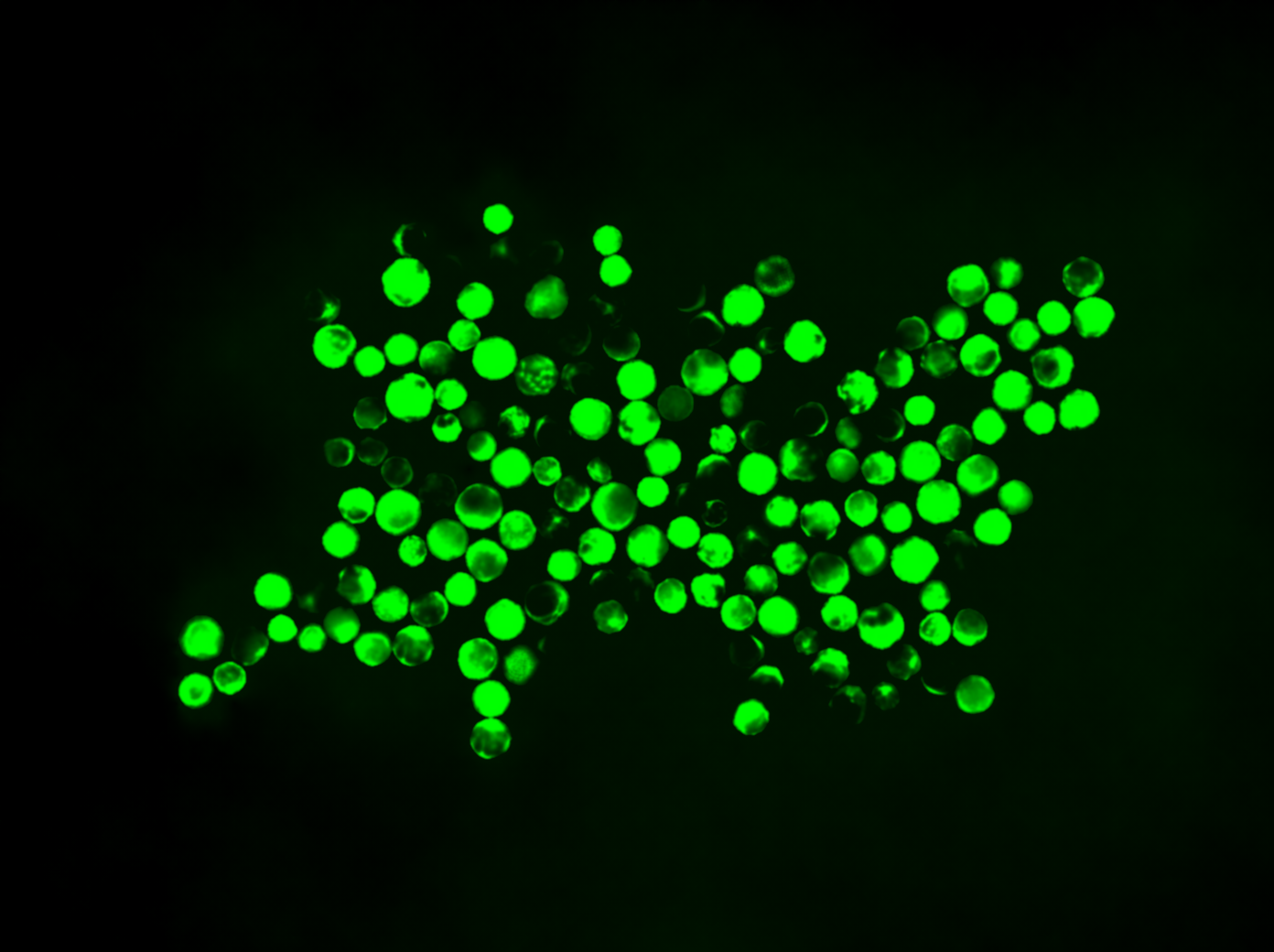

Supplement: Additional file 5 — The zip archive contains simulated images showing protoplasts with corresponding ground truth. (ZIP 72704 kb) [file 12859_2017_1591_MOESM5_ESM.zip › simulated protoplasts/nottouching/nottouching018.png]

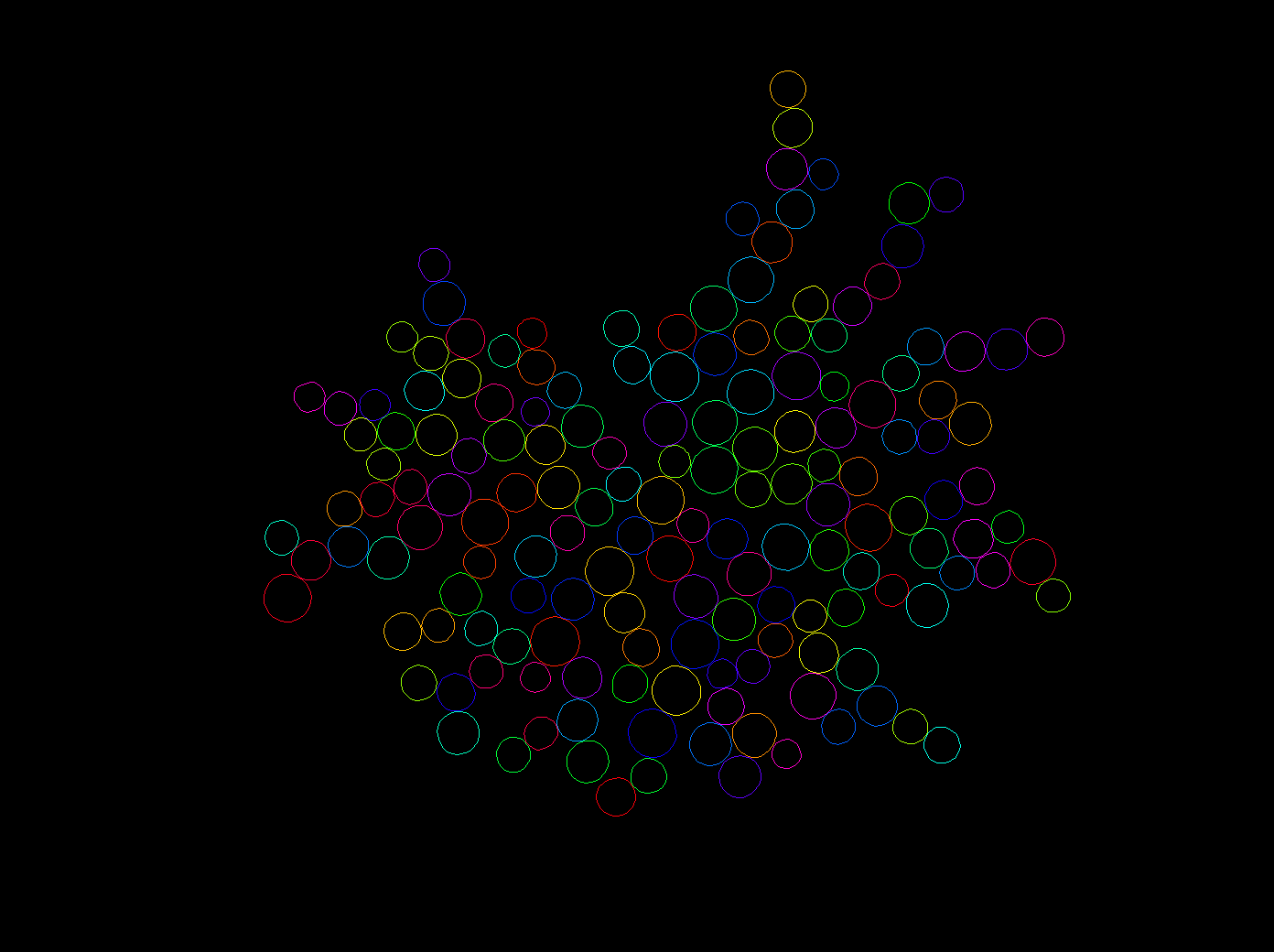

Supplement: Additional file 5 — The zip archive contains simulated images showing protoplasts with corresponding ground truth. (ZIP 72704 kb) [file 12859_2017_1591_MOESM5_ESM.zip › simulated protoplasts/nottouching/nottouching019 gt.png]

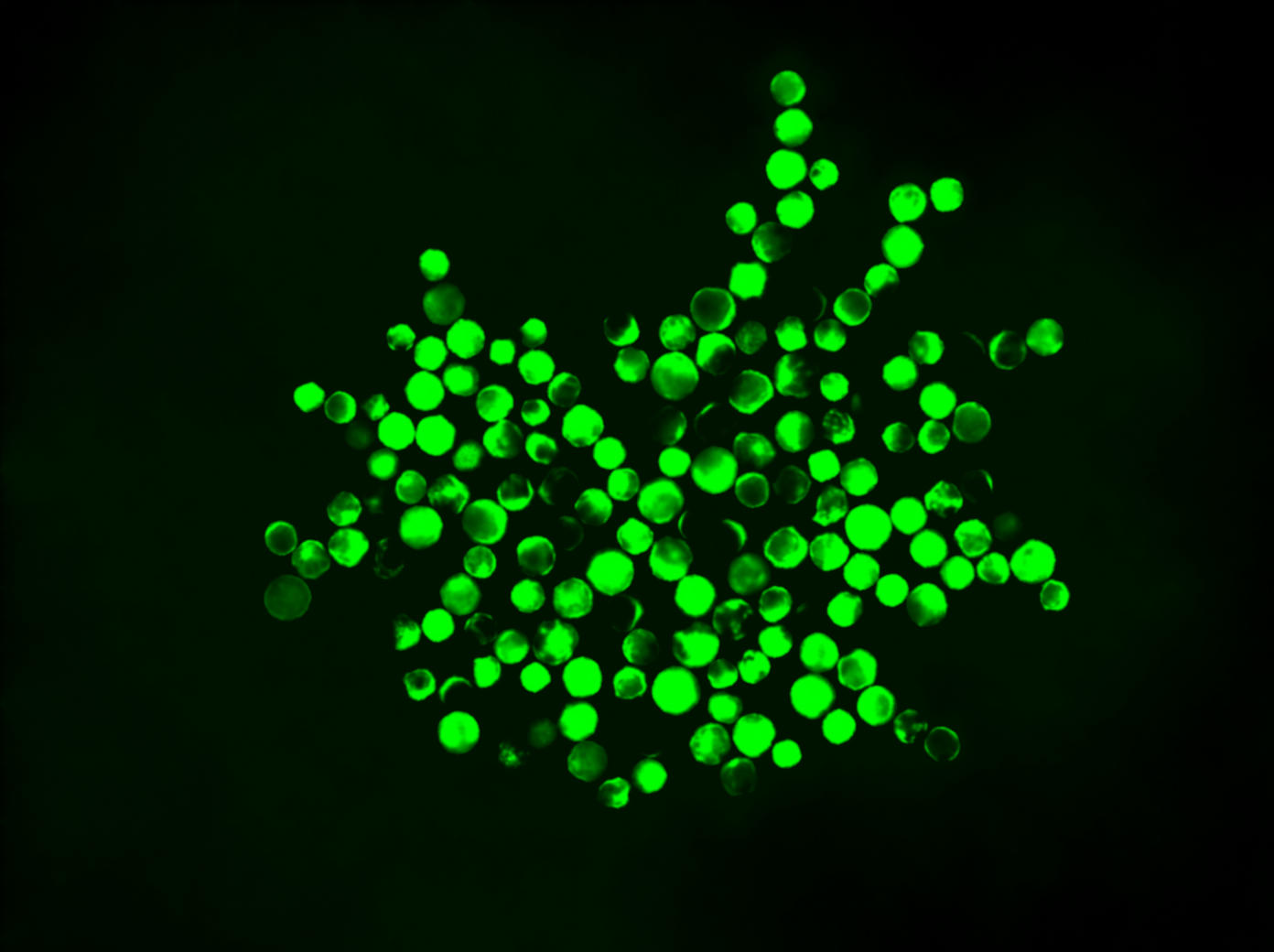

Supplement: Additional file 5 — The zip archive contains simulated images showing protoplasts with corresponding ground truth. (ZIP 72704 kb) [file 12859_2017_1591_MOESM5_ESM.zip › simulated protoplasts/nottouching/nottouching019.png]

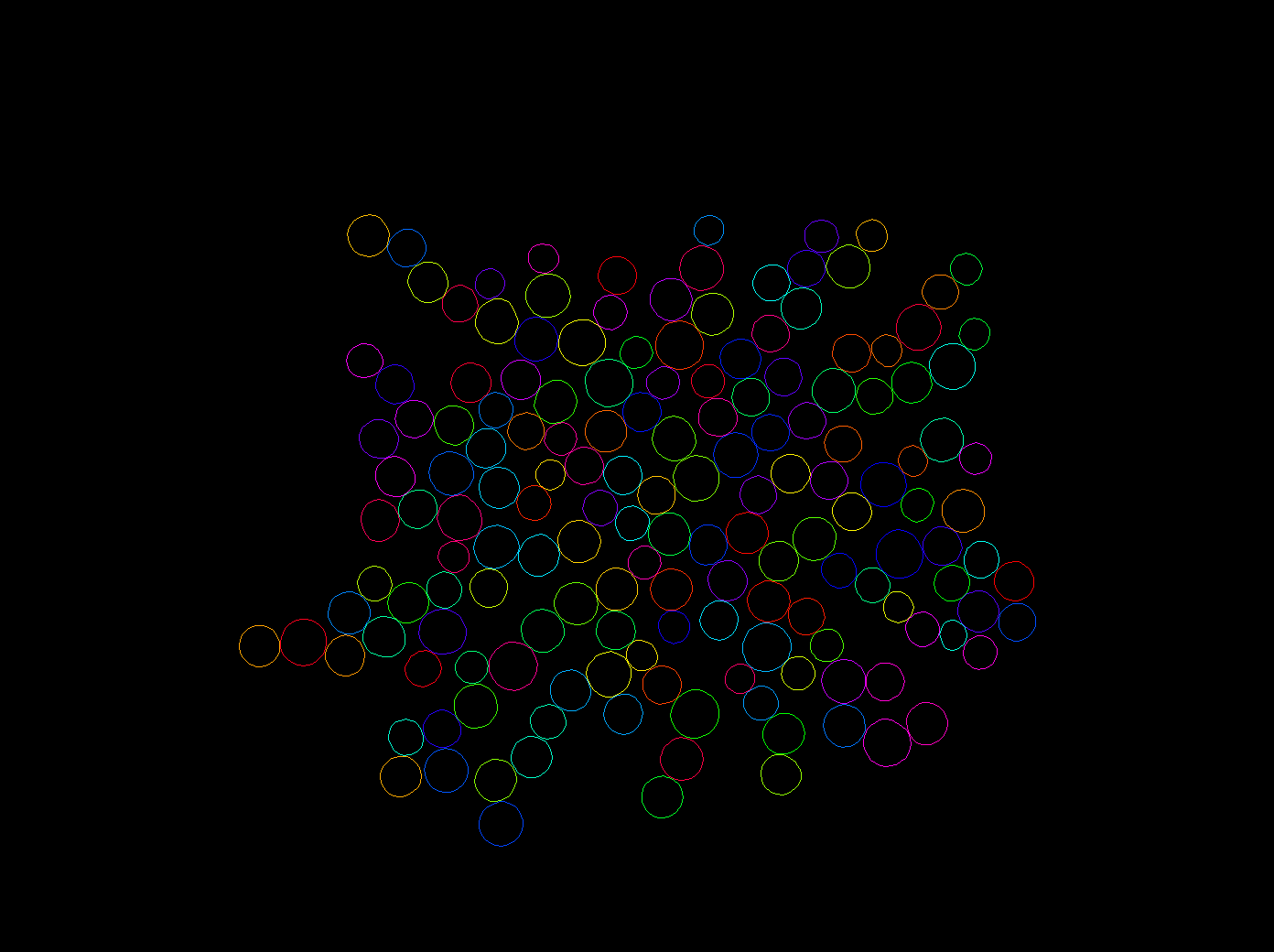

Supplement: Additional file 5 — The zip archive contains simulated images showing protoplasts with corresponding ground truth. (ZIP 72704 kb) [file 12859_2017_1591_MOESM5_ESM.zip › simulated protoplasts/nottouching/nottouching020 gt.png]

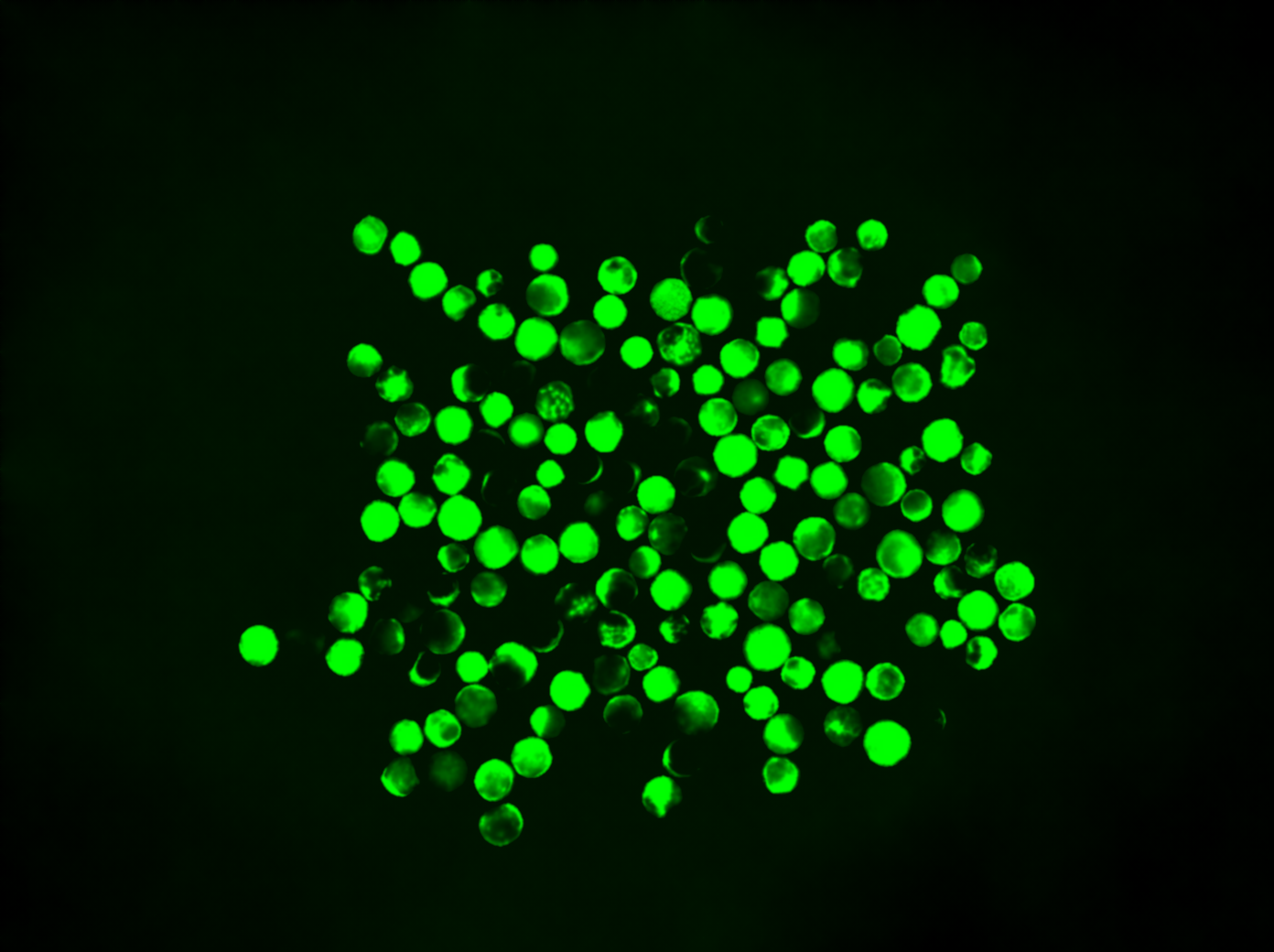

Supplement: Additional file 5 — The zip archive contains simulated images showing protoplasts with corresponding ground truth. (ZIP 72704 kb) [file 12859_2017_1591_MOESM5_ESM.zip › simulated protoplasts/nottouching/nottouching020.png]
