# Supplementary material for: Using simulated fluorescence cell micrographs for the evaluation of cell image segmentation algorithms
Source: BMC Bioinformatics. 2017 Mar 18;18:176. doi: 10.1186/s12859-017-1591-2 (PMC5357336; doi:10.1186/s12859-017-1591-2)
Supplement: Additional file 4 — A second image segmentation pipeline was applied to the images. The results and conclusions for this second pipeline are described in the pdf-file. (PDF 1423 kb) [file 12859_2017_1591_MOESM4_ESM.pdf]

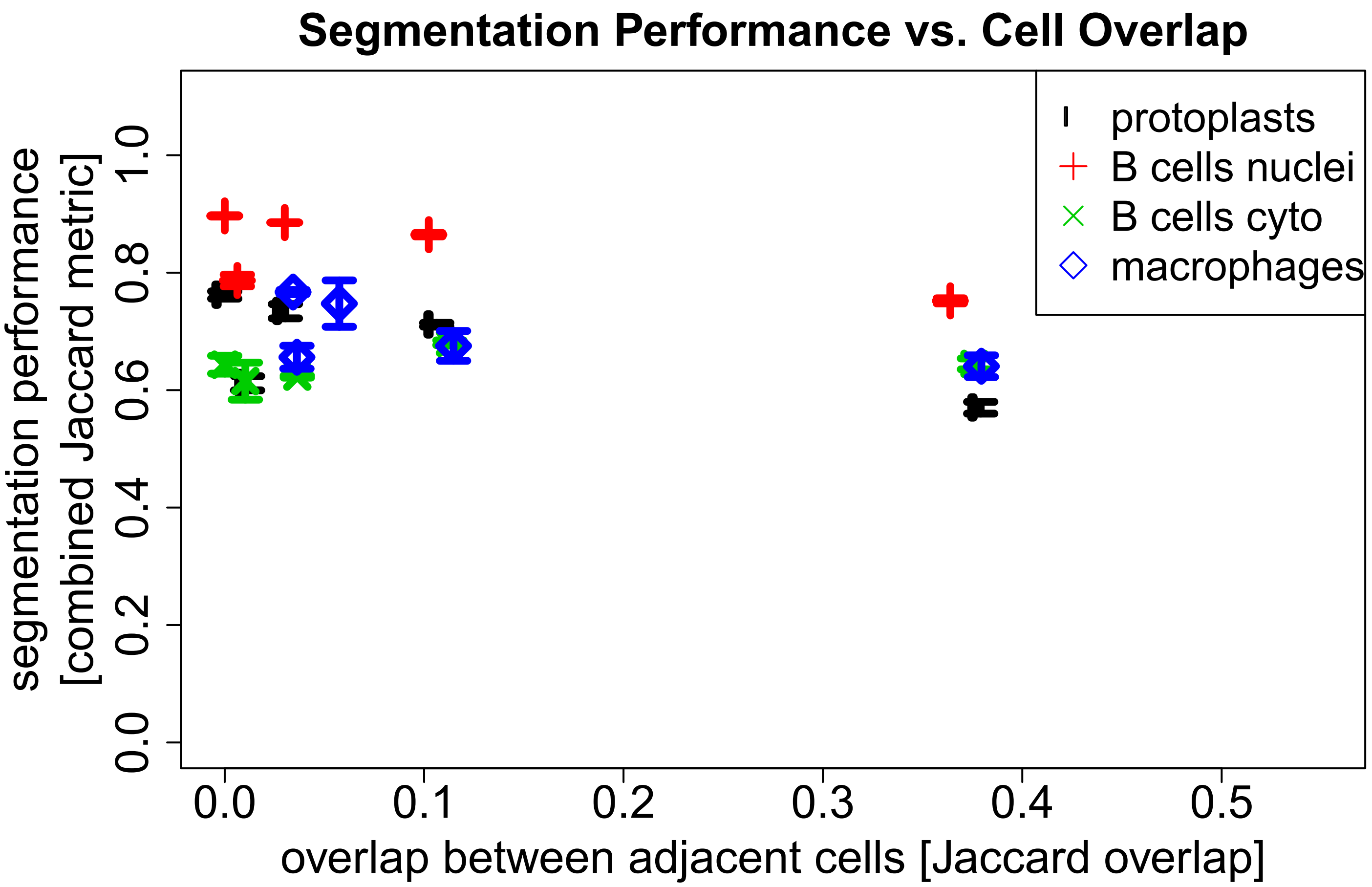

Supplementary figure: We repeated the experiment with a second pipeline. The preprocessing was done with the DoG filter to smooth the foreground and to remove the background. We used a Hough Voting Filter from itk (http://www.insight-journal.org/browse/publication/698) for voting seeds on the images with the protoplasts and the B cell nuclei. For the B cell cytoskeleton and the macrophages we used the nuclei as seeds, again . The figure ground separation was done with a Graph cuts algorithm from [19]. The seeds and the figure ground separation were input to a seeded fast margin algorithm [16]. These segmentation results show also decreasing segmentation performance with increasing overlap for the four data sets of each segmentation task simulated with one cluster and increasing overlap. These findings support the conclusions drawn from the experiments with our previous segmentation pipeline. For data sets simulated with the realisitic overlap settings the performance falls of.
