# Supplementary figures and images for: Using simulated fluorescence cell micrographs for the evaluation of cell image segmentation algorithms (part 4 of 6)
Source: BMC Bioinformatics. 2017 Mar 18;18:176. doi: 10.1186/s12859-017-1591-2 (PMC5357336; doi:10.1186/s12859-017-1591-2)

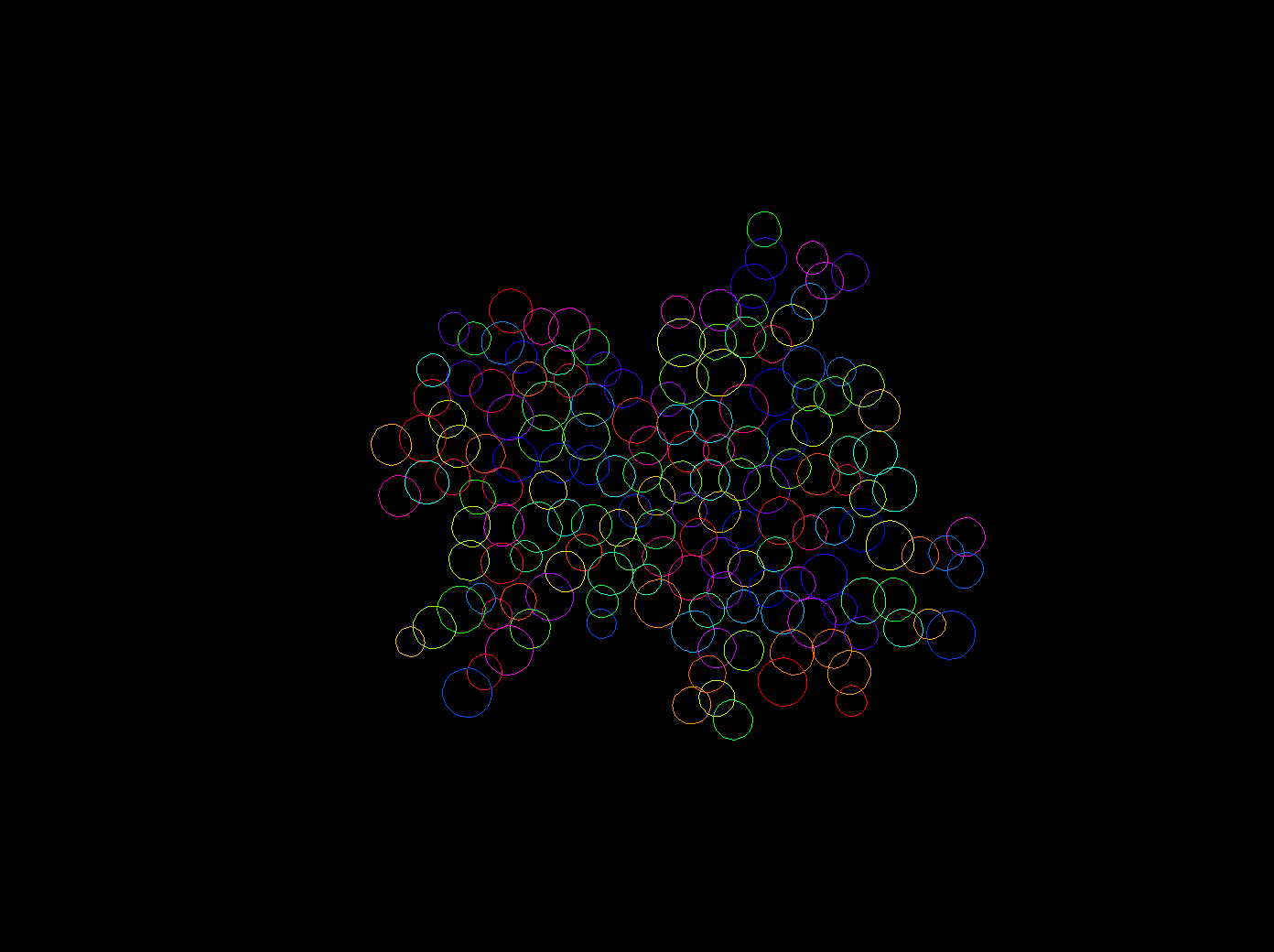

Supplement: Additional file 5 — The zip archive contains simulated images showing protoplasts with corresponding ground truth. (ZIP 72704 kb) [file 12859_2017_1591_MOESM5_ESM.zip › simulated protoplasts/overlaying/overlaying011 gt.png]

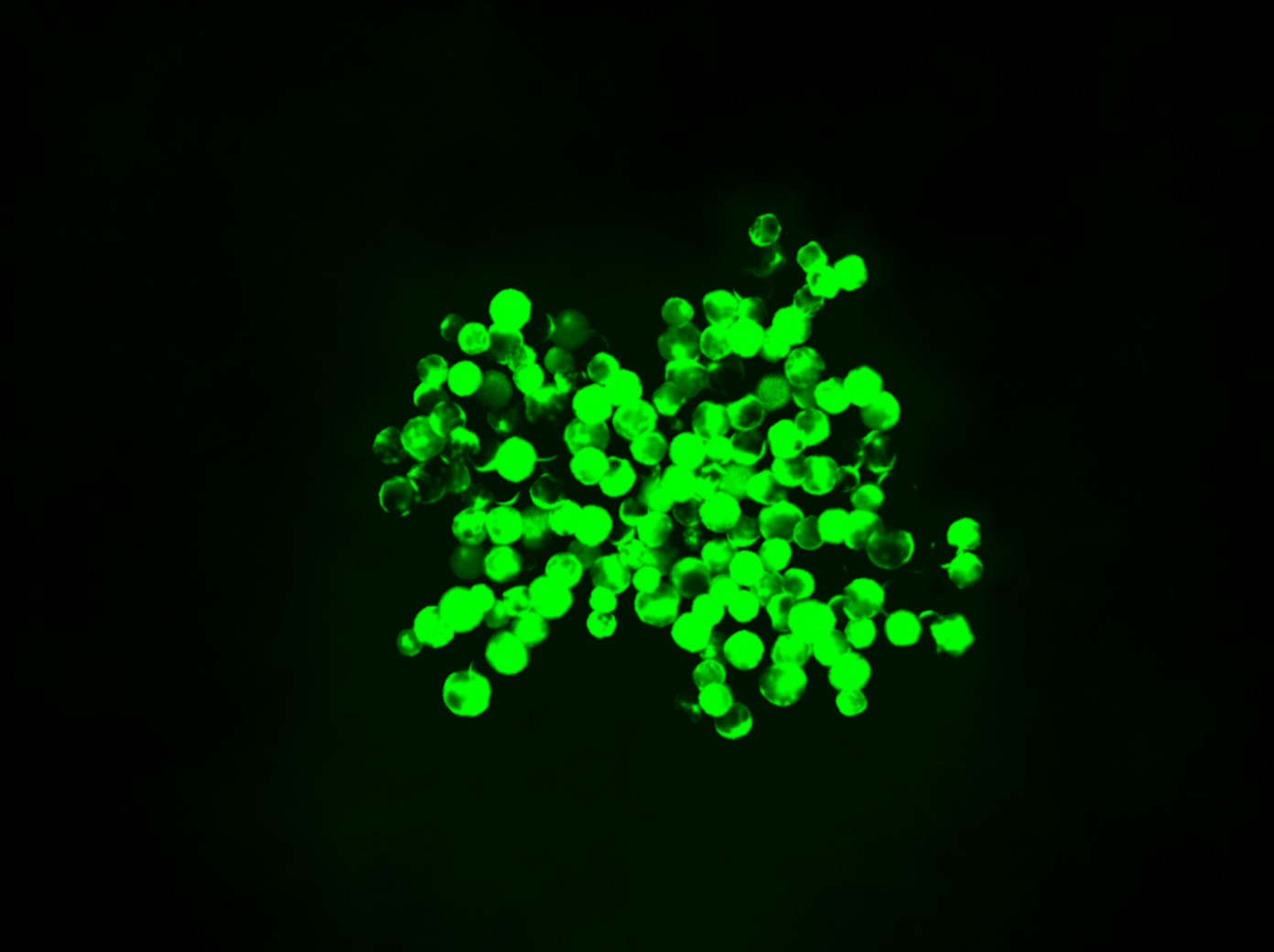

Supplement: Additional file 5 — The zip archive contains simulated images showing protoplasts with corresponding ground truth. (ZIP 72704 kb) [file 12859_2017_1591_MOESM5_ESM.zip › simulated protoplasts/overlaying/overlaying011.png]

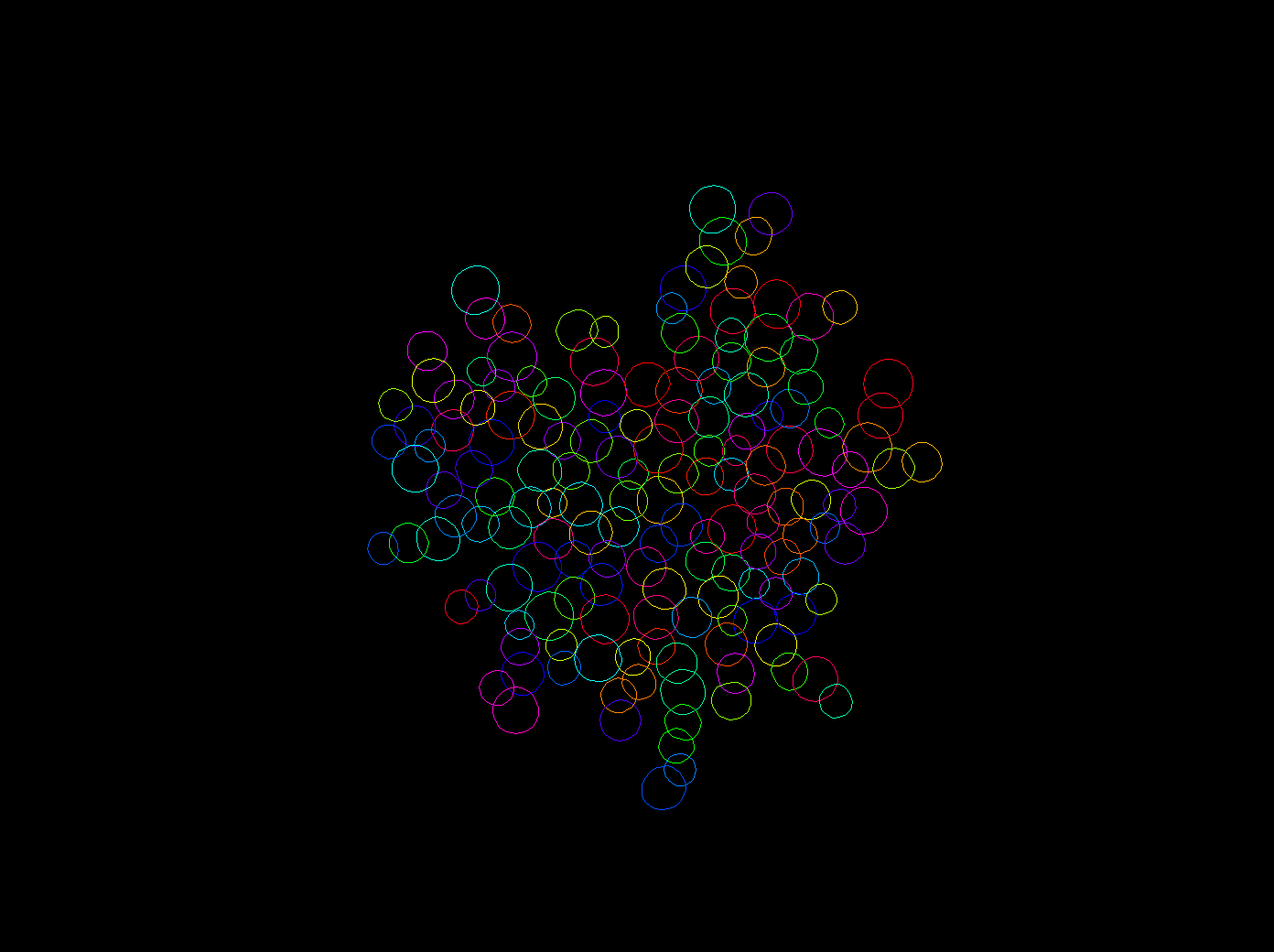

Supplement: Additional file 5 — The zip archive contains simulated images showing protoplasts with corresponding ground truth. (ZIP 72704 kb) [file 12859_2017_1591_MOESM5_ESM.zip › simulated protoplasts/overlaying/overlaying012 gt.png]

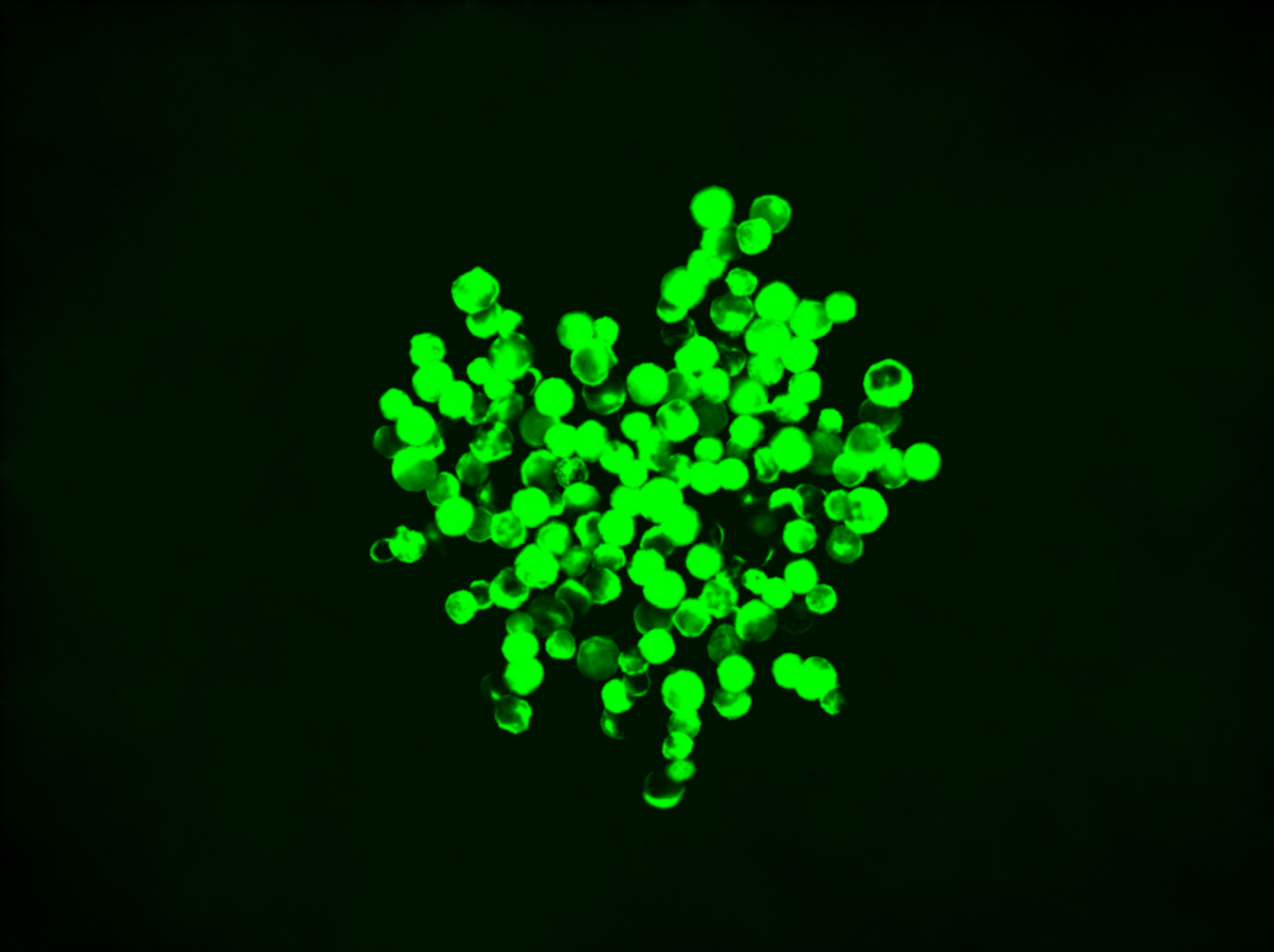

Supplement: Additional file 5 — The zip archive contains simulated images showing protoplasts with corresponding ground truth. (ZIP 72704 kb) [file 12859_2017_1591_MOESM5_ESM.zip › simulated protoplasts/overlaying/overlaying012.png]

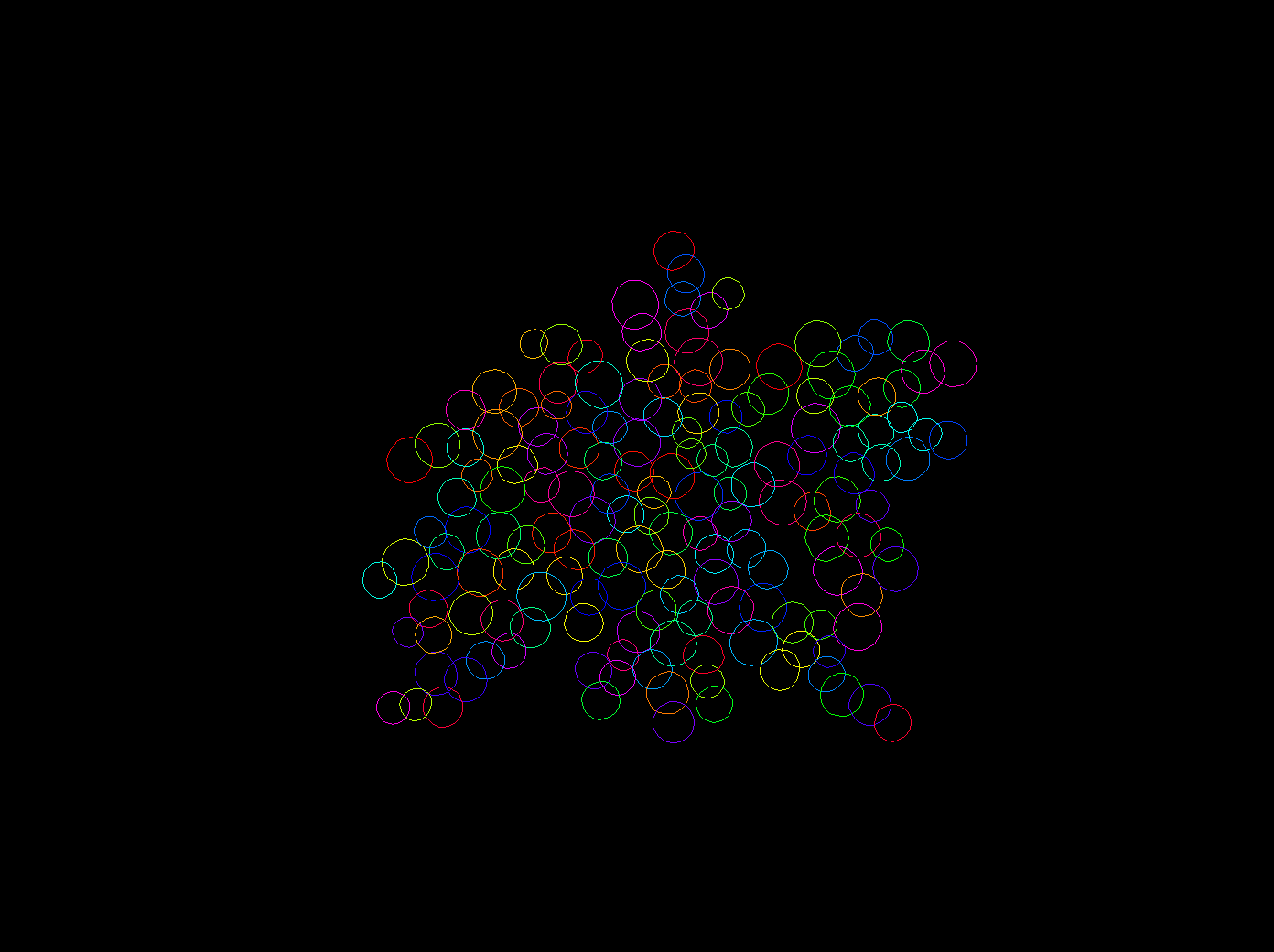

Supplement: Additional file 5 — The zip archive contains simulated images showing protoplasts with corresponding ground truth. (ZIP 72704 kb) [file 12859_2017_1591_MOESM5_ESM.zip › simulated protoplasts/overlaying/overlaying013 gt.png]

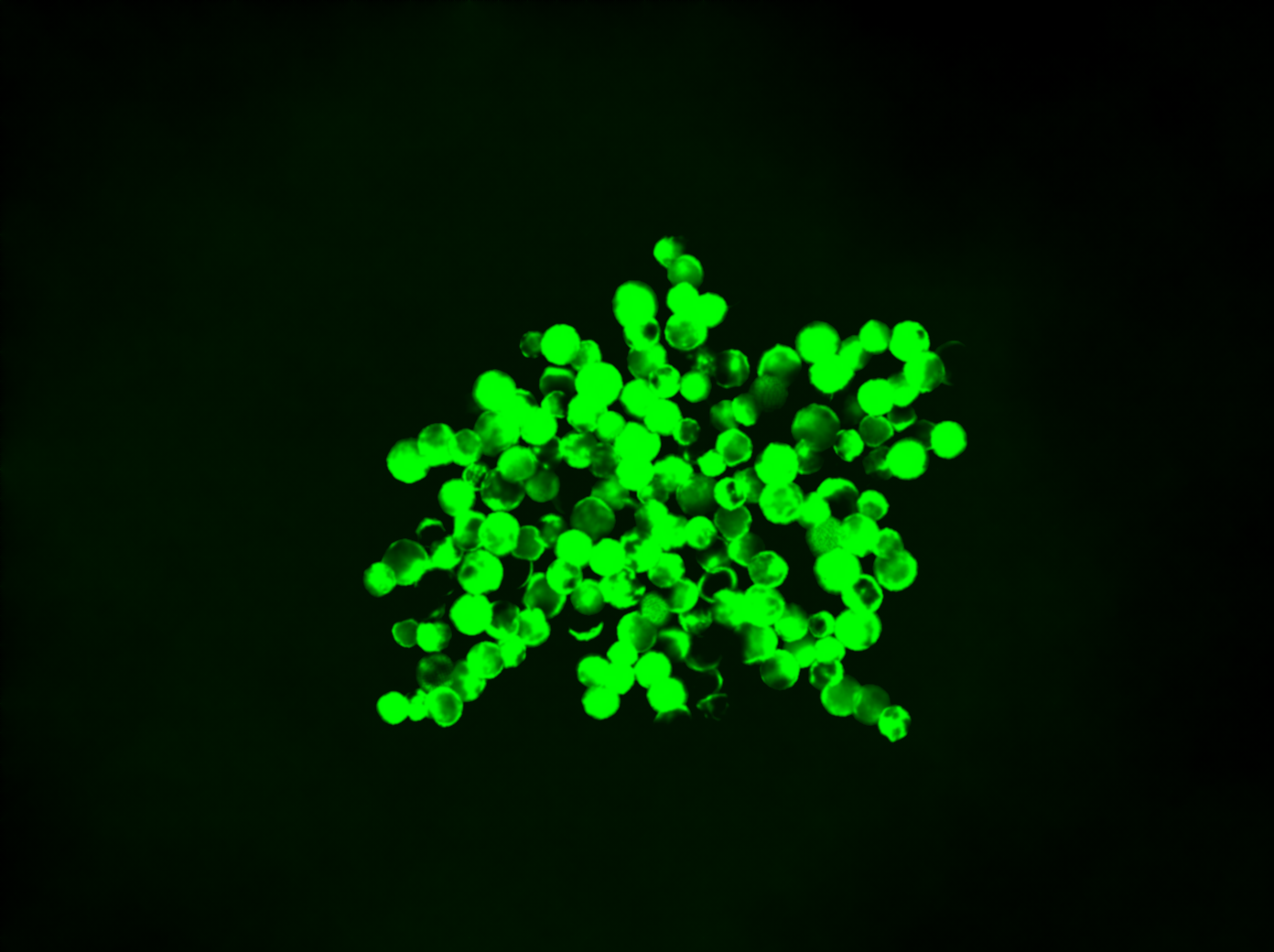

Supplement: Additional file 5 — The zip archive contains simulated images showing protoplasts with corresponding ground truth. (ZIP 72704 kb) [file 12859_2017_1591_MOESM5_ESM.zip › simulated protoplasts/overlaying/overlaying013.png]

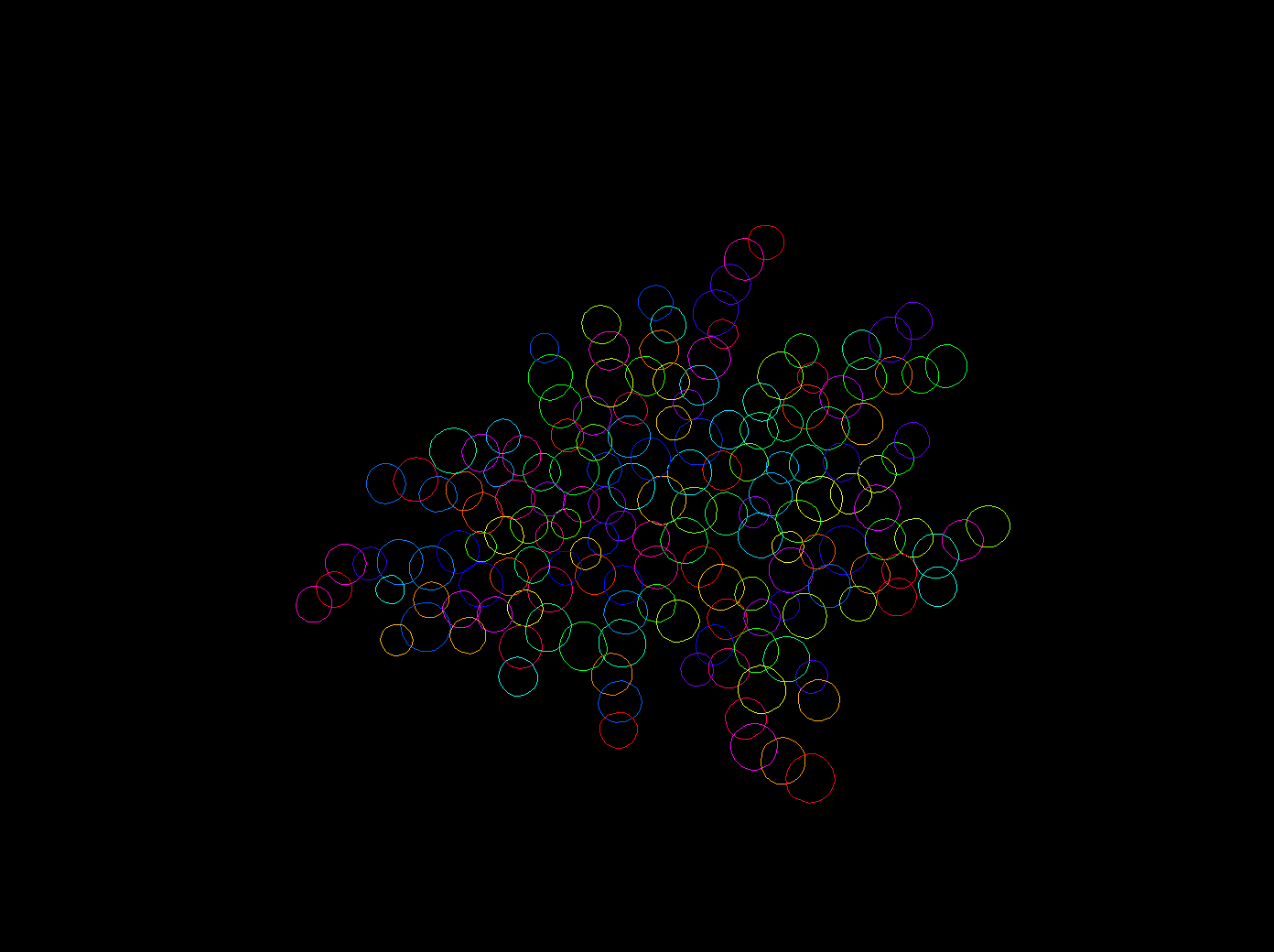

Supplement: Additional file 5 — The zip archive contains simulated images showing protoplasts with corresponding ground truth. (ZIP 72704 kb) [file 12859_2017_1591_MOESM5_ESM.zip › simulated protoplasts/overlaying/overlaying014 gt.png]

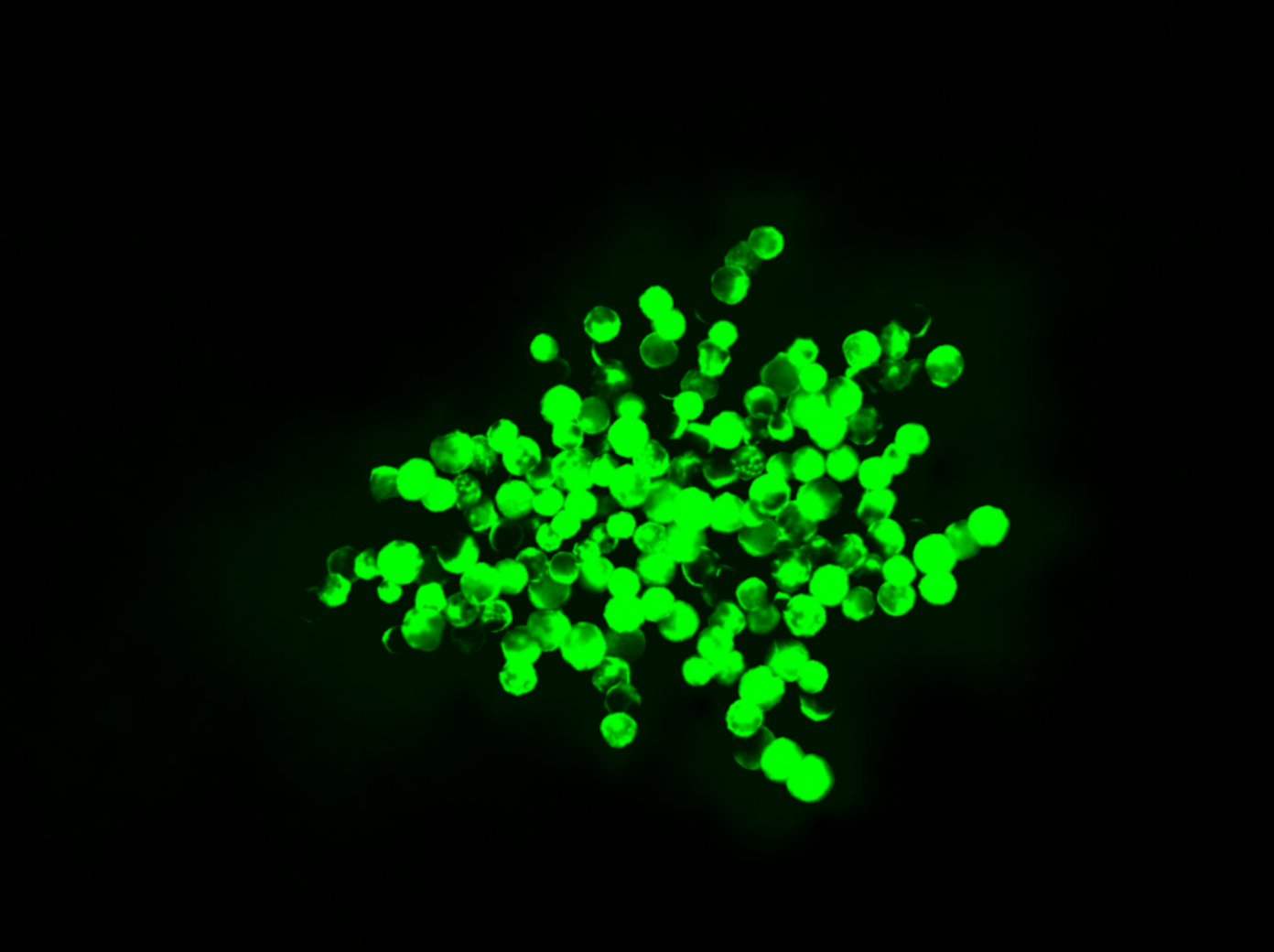

Supplement: Additional file 5 — The zip archive contains simulated images showing protoplasts with corresponding ground truth. (ZIP 72704 kb) [file 12859_2017_1591_MOESM5_ESM.zip › simulated protoplasts/overlaying/overlaying014.png]

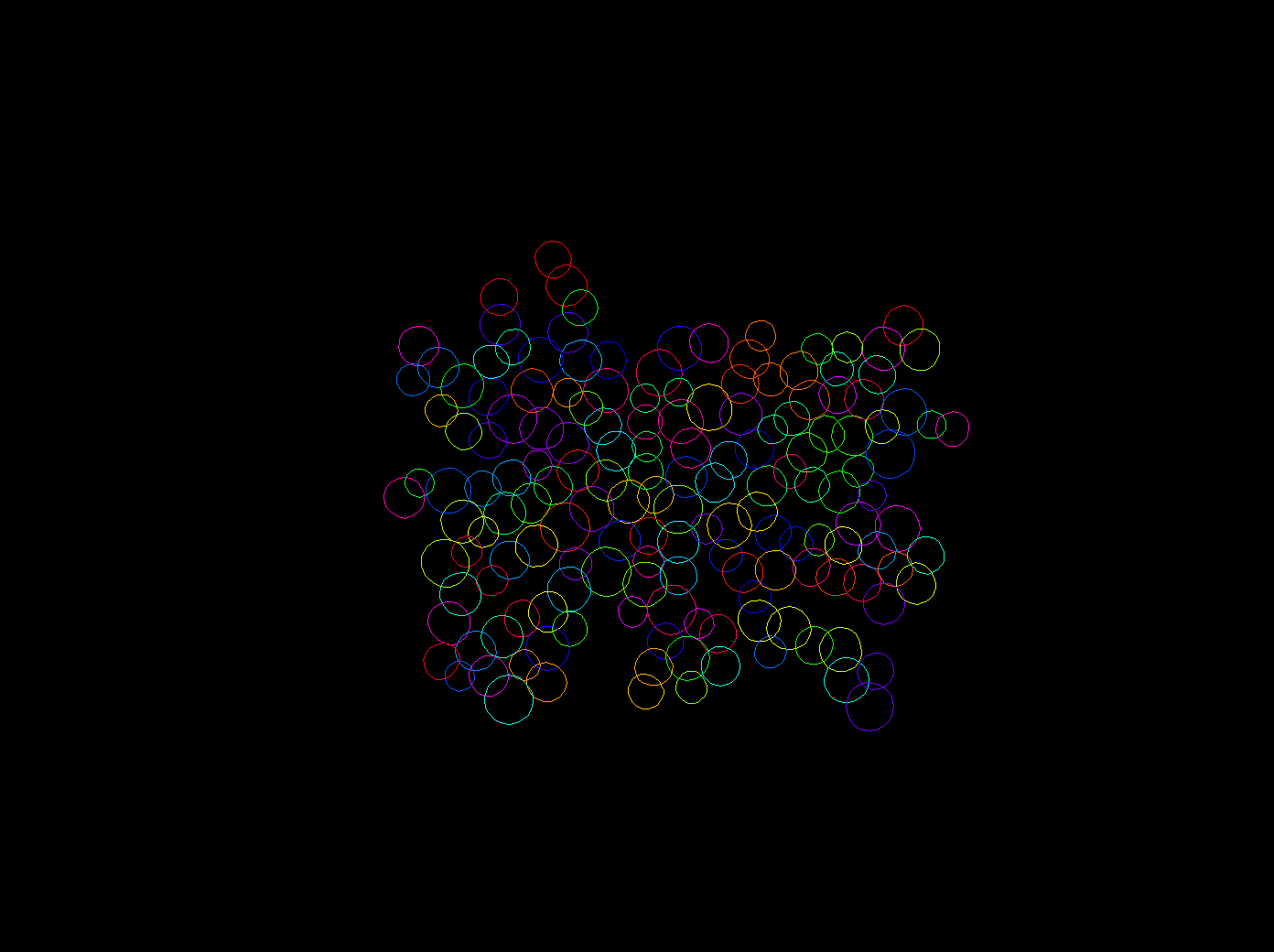

Supplement: Additional file 5 — The zip archive contains simulated images showing protoplasts with corresponding ground truth. (ZIP 72704 kb) [file 12859_2017_1591_MOESM5_ESM.zip › simulated protoplasts/overlaying/overlaying015 gt.png]

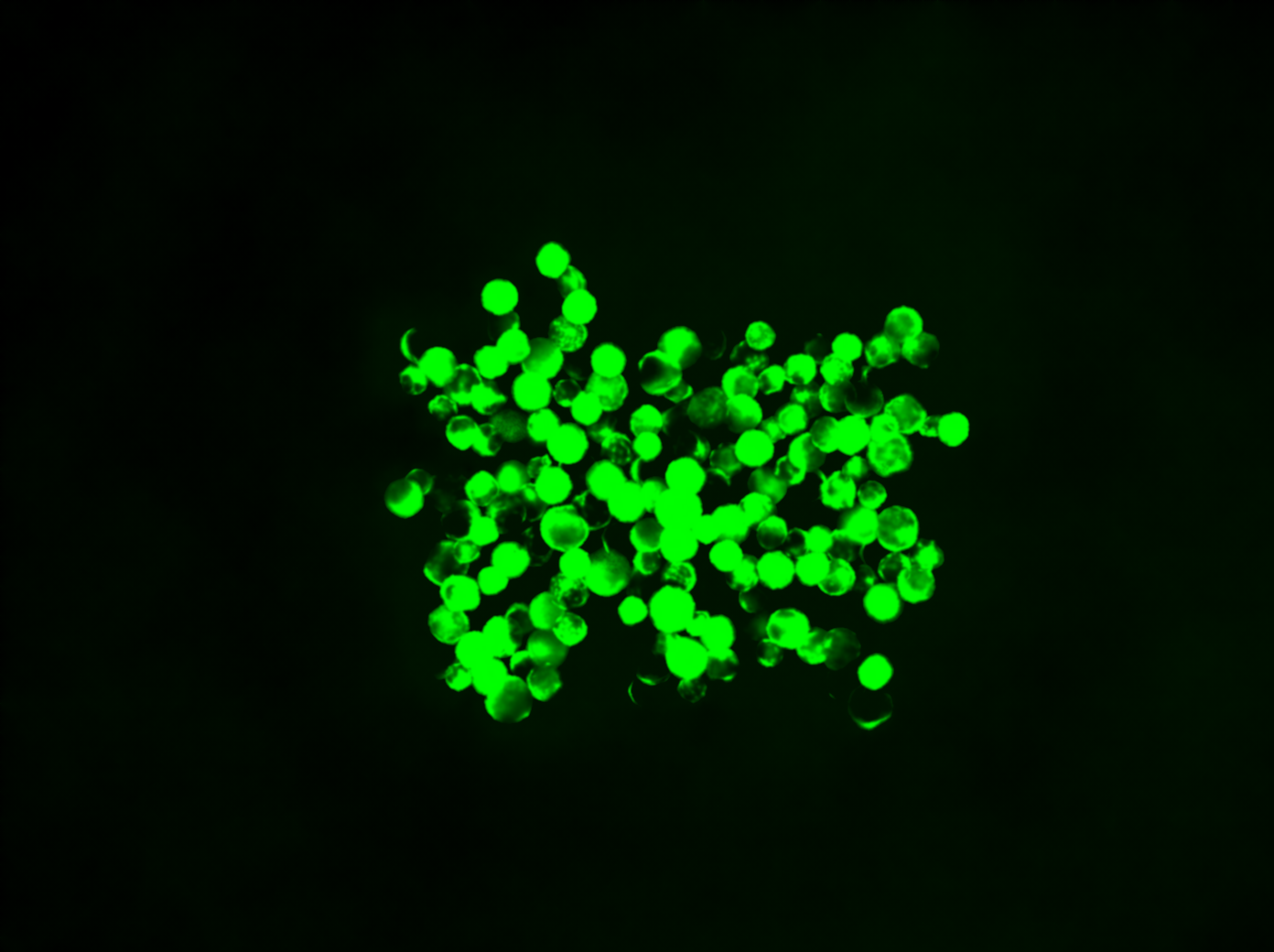

Supplement: Additional file 5 — The zip archive contains simulated images showing protoplasts with corresponding ground truth. (ZIP 72704 kb) [file 12859_2017_1591_MOESM5_ESM.zip › simulated protoplasts/overlaying/overlaying015.png]

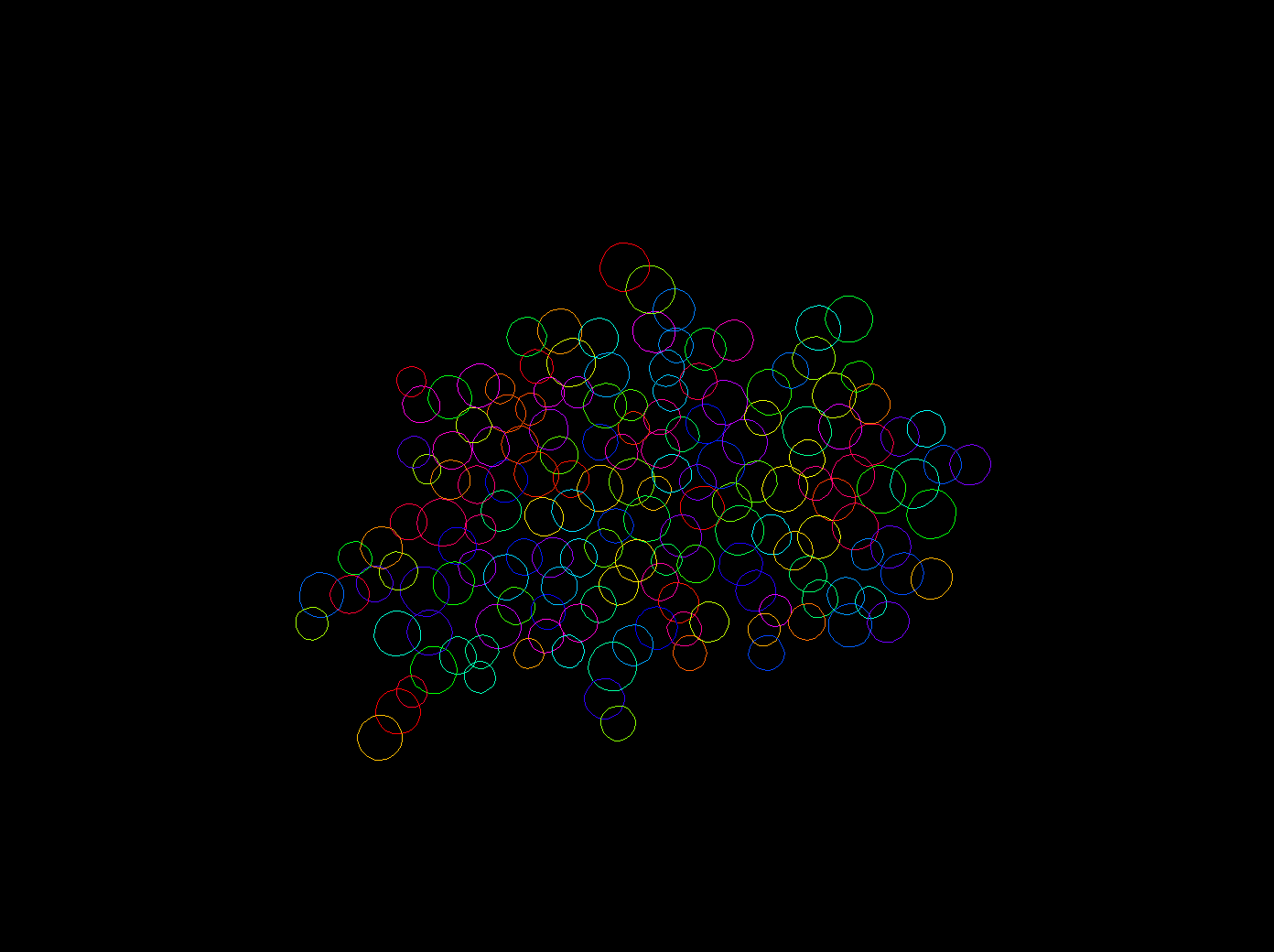

Supplement: Additional file 5 — The zip archive contains simulated images showing protoplasts with corresponding ground truth. (ZIP 72704 kb) [file 12859_2017_1591_MOESM5_ESM.zip › simulated protoplasts/overlaying/overlaying016 gt.png]

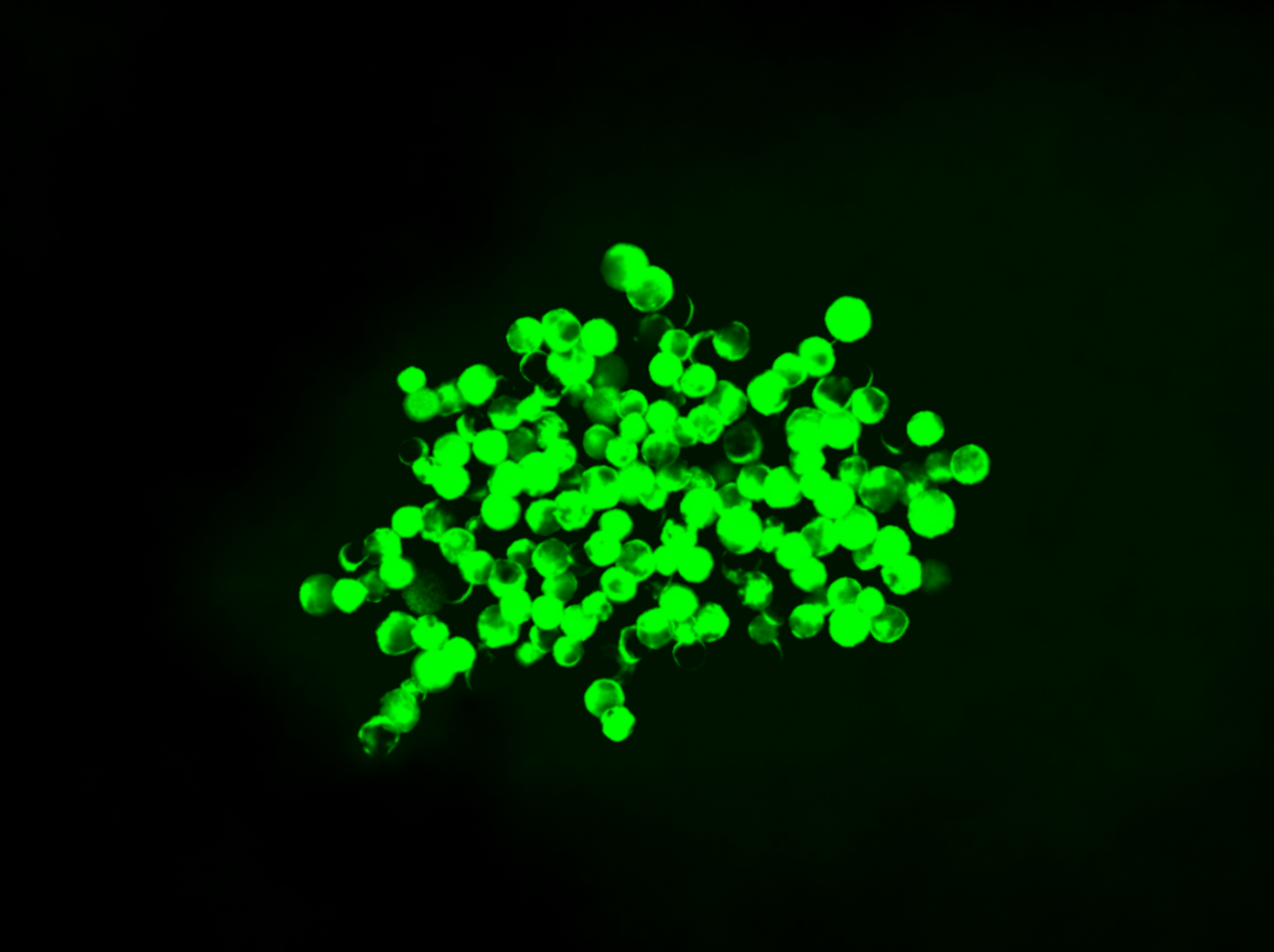

Supplement: Additional file 5 — The zip archive contains simulated images showing protoplasts with corresponding ground truth. (ZIP 72704 kb) [file 12859_2017_1591_MOESM5_ESM.zip › simulated protoplasts/overlaying/overlaying016.png]

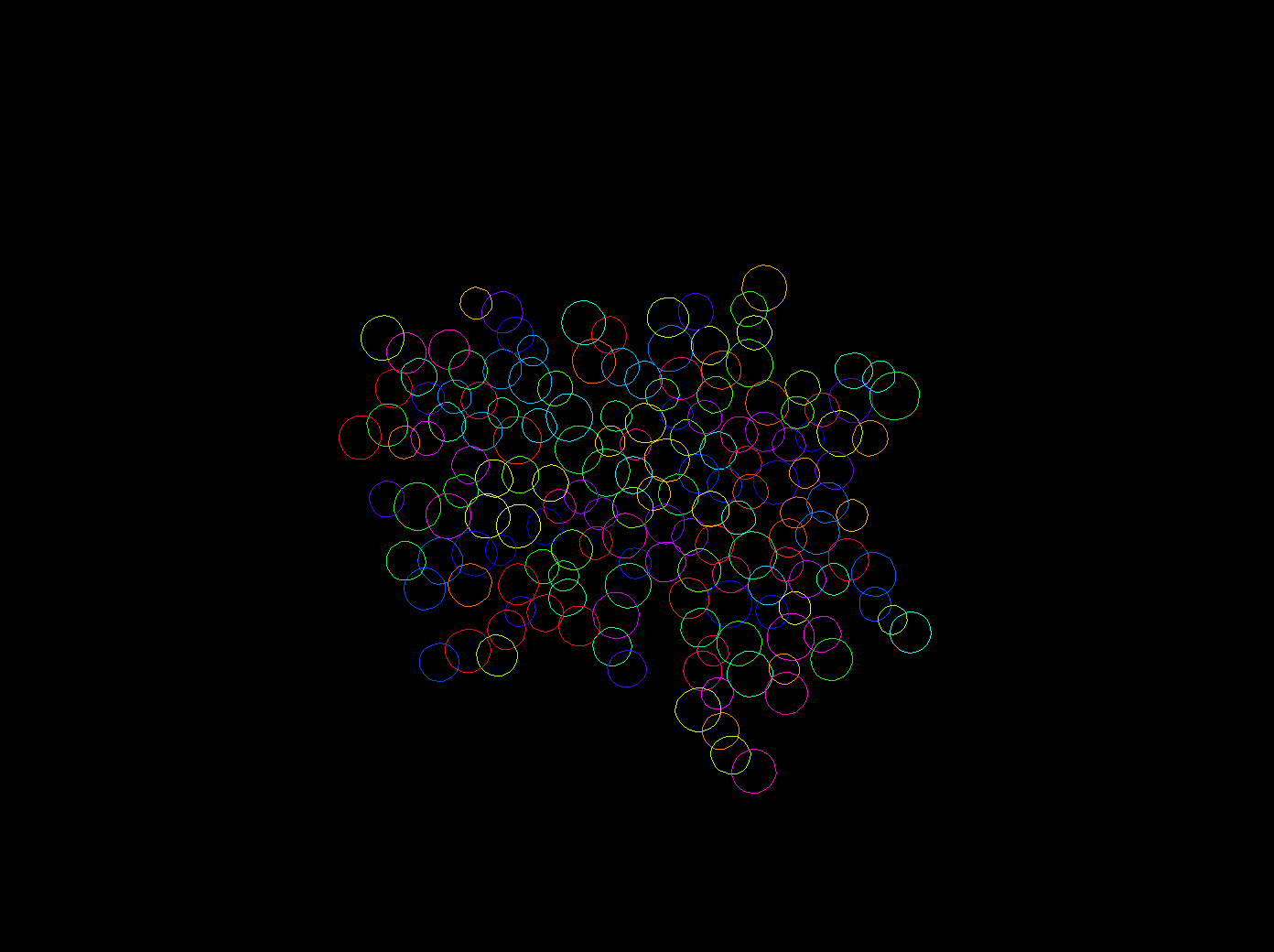

Supplement: Additional file 5 — The zip archive contains simulated images showing protoplasts with corresponding ground truth. (ZIP 72704 kb) [file 12859_2017_1591_MOESM5_ESM.zip › simulated protoplasts/overlaying/overlaying017 gt.png]

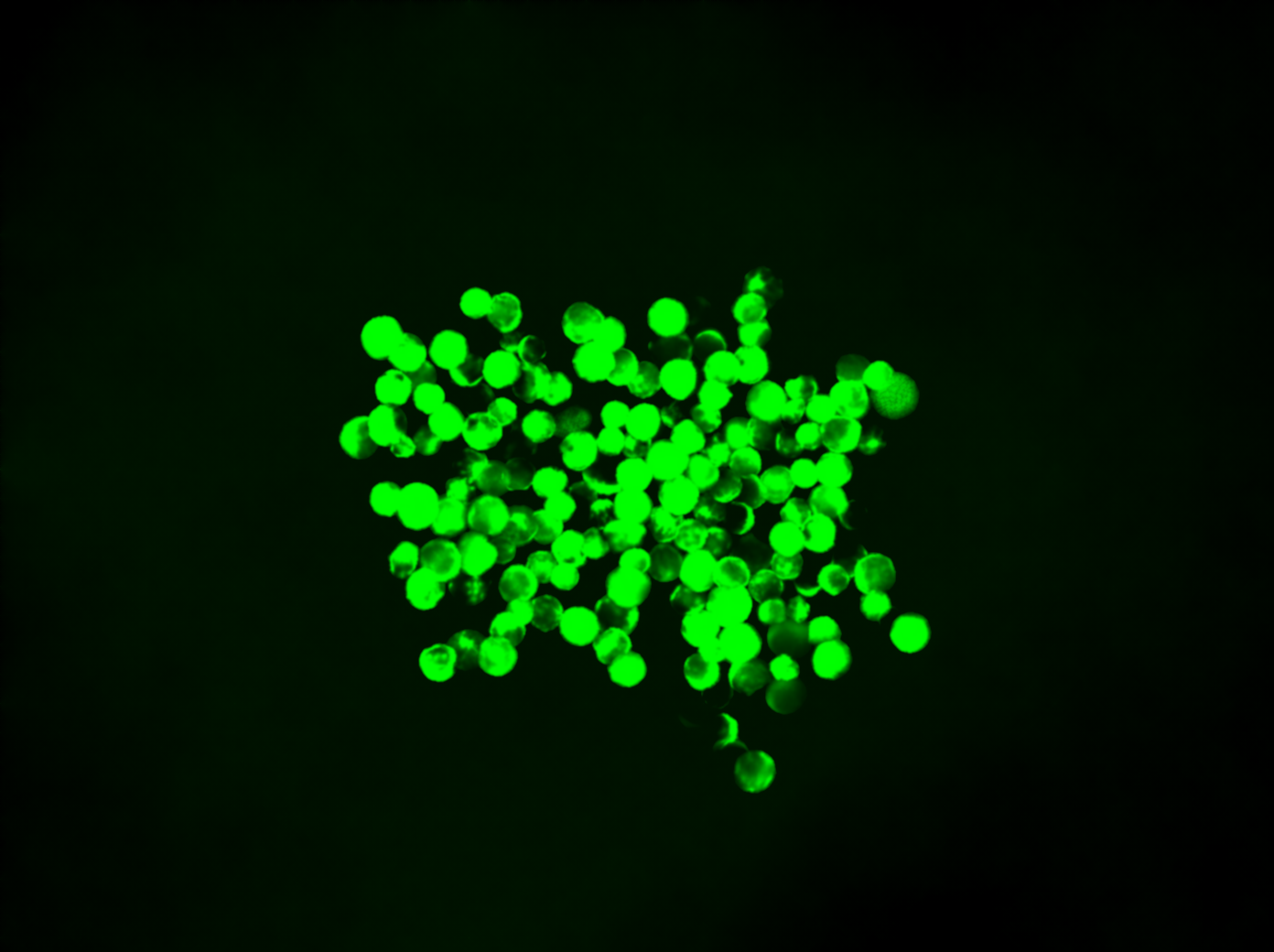

Supplement: Additional file 5 — The zip archive contains simulated images showing protoplasts with corresponding ground truth. (ZIP 72704 kb) [file 12859_2017_1591_MOESM5_ESM.zip › simulated protoplasts/overlaying/overlaying017.png]

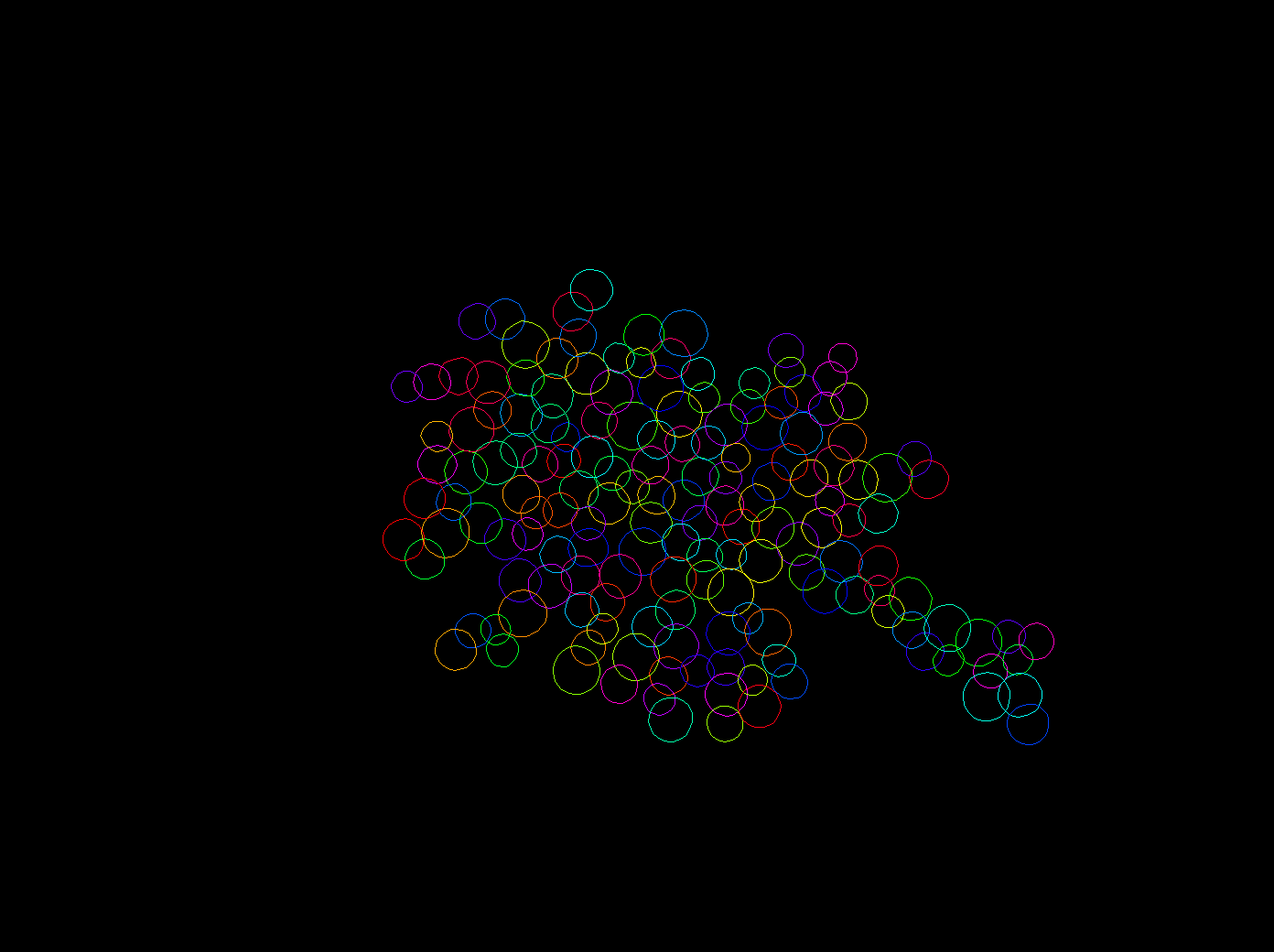

Supplement: Additional file 5 — The zip archive contains simulated images showing protoplasts with corresponding ground truth. (ZIP 72704 kb) [file 12859_2017_1591_MOESM5_ESM.zip › simulated protoplasts/overlaying/overlaying018 gt.png]

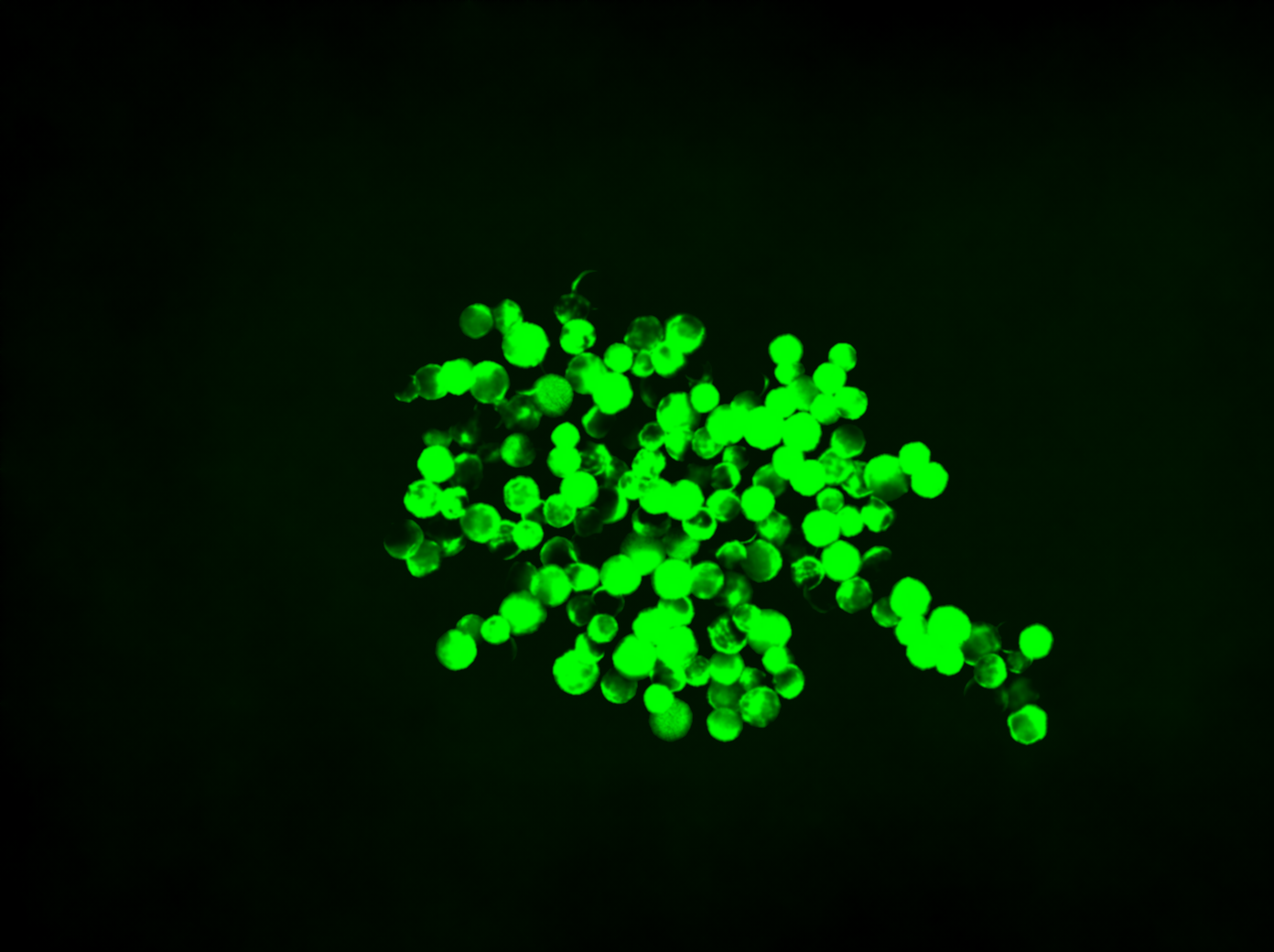

Supplement: Additional file 5 — The zip archive contains simulated images showing protoplasts with corresponding ground truth. (ZIP 72704 kb) [file 12859_2017_1591_MOESM5_ESM.zip › simulated protoplasts/overlaying/overlaying018.png]

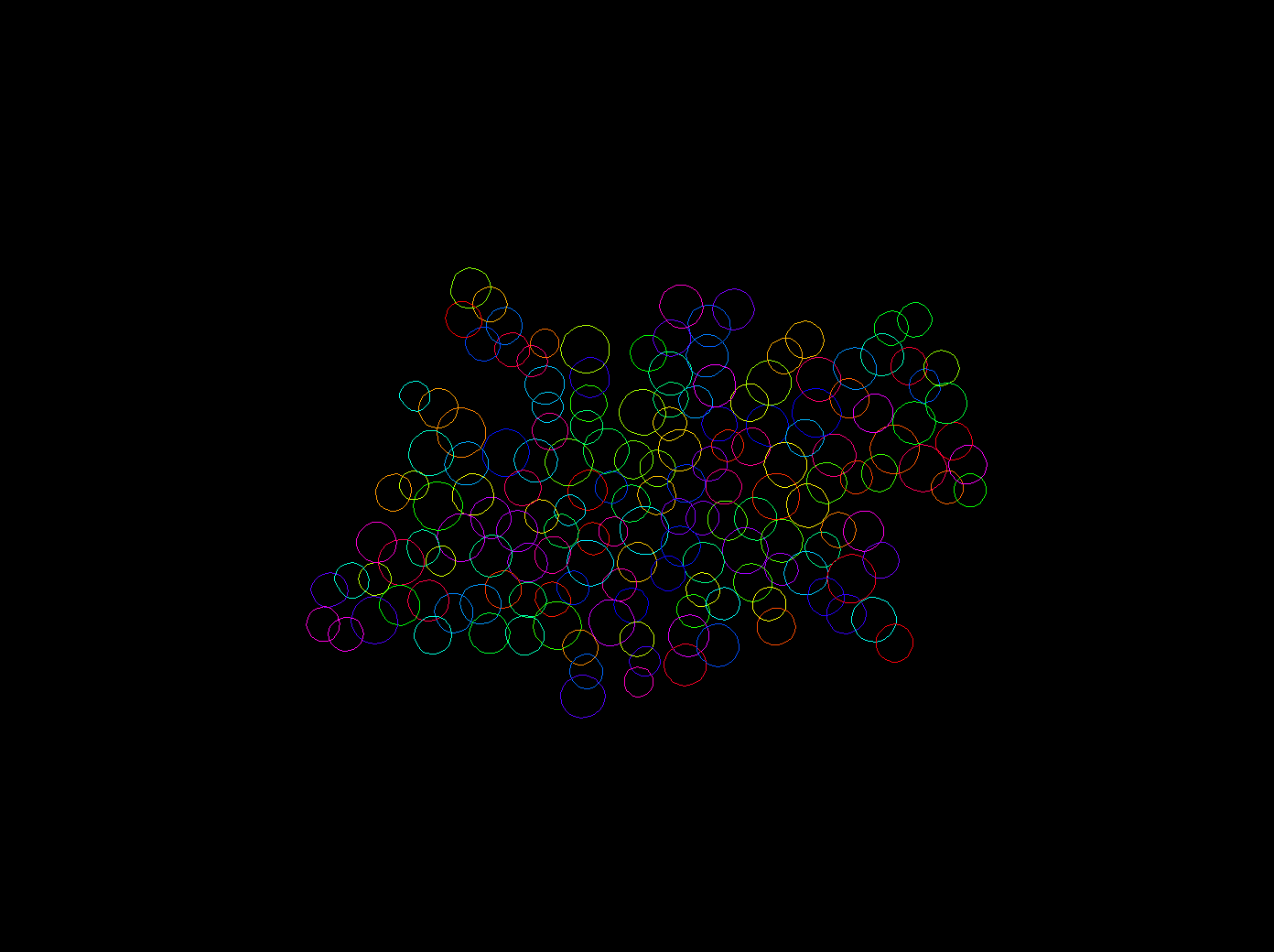

Supplement: Additional file 5 — The zip archive contains simulated images showing protoplasts with corresponding ground truth. (ZIP 72704 kb) [file 12859_2017_1591_MOESM5_ESM.zip › simulated protoplasts/overlaying/overlaying019 gt.png]

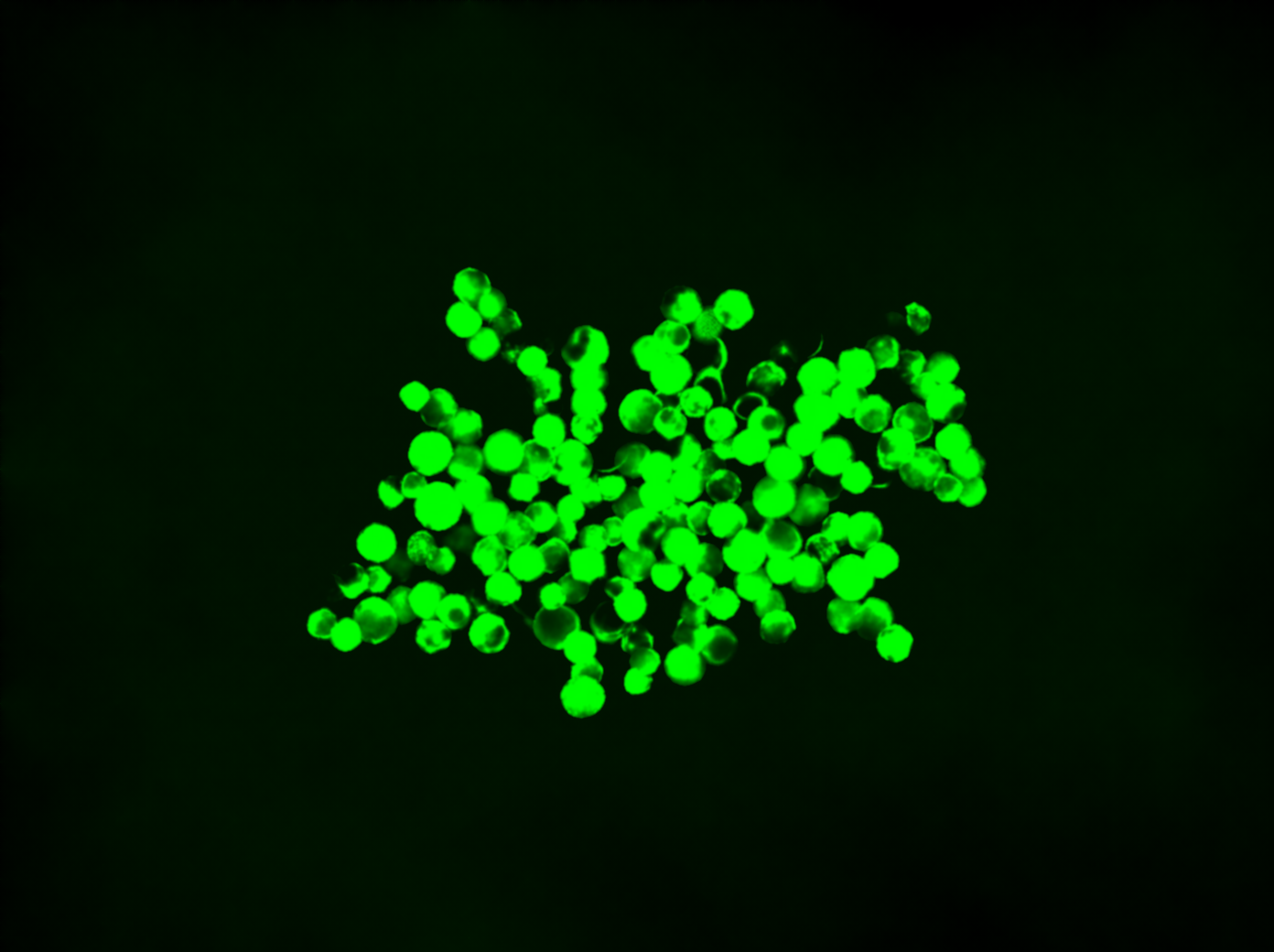

Supplement: Additional file 5 — The zip archive contains simulated images showing protoplasts with corresponding ground truth. (ZIP 72704 kb) [file 12859_2017_1591_MOESM5_ESM.zip › simulated protoplasts/overlaying/overlaying019.png]

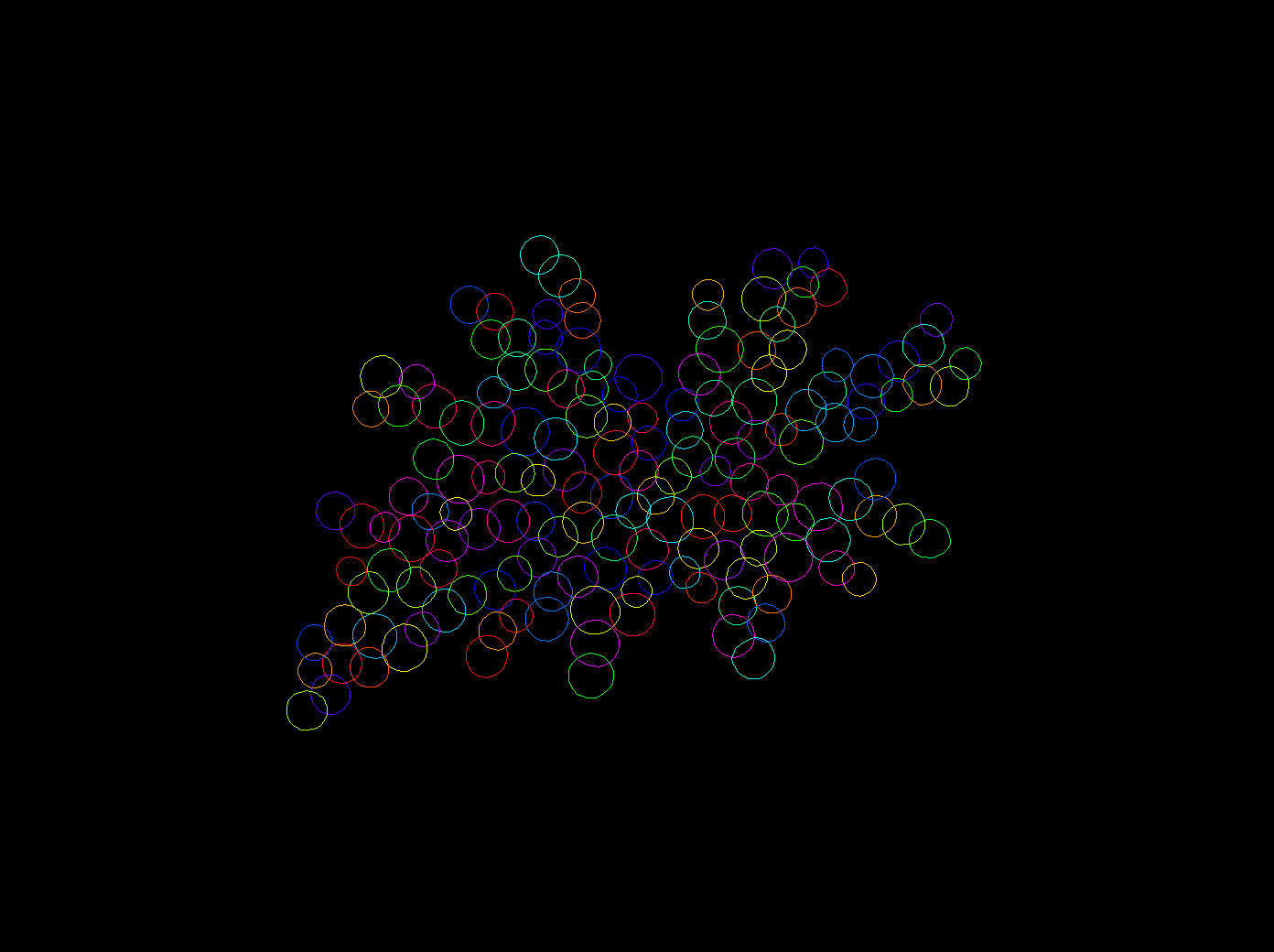

Supplement: Additional file 5 — The zip archive contains simulated images showing protoplasts with corresponding ground truth. (ZIP 72704 kb) [file 12859_2017_1591_MOESM5_ESM.zip › simulated protoplasts/overlaying/overlaying020 gt.png]

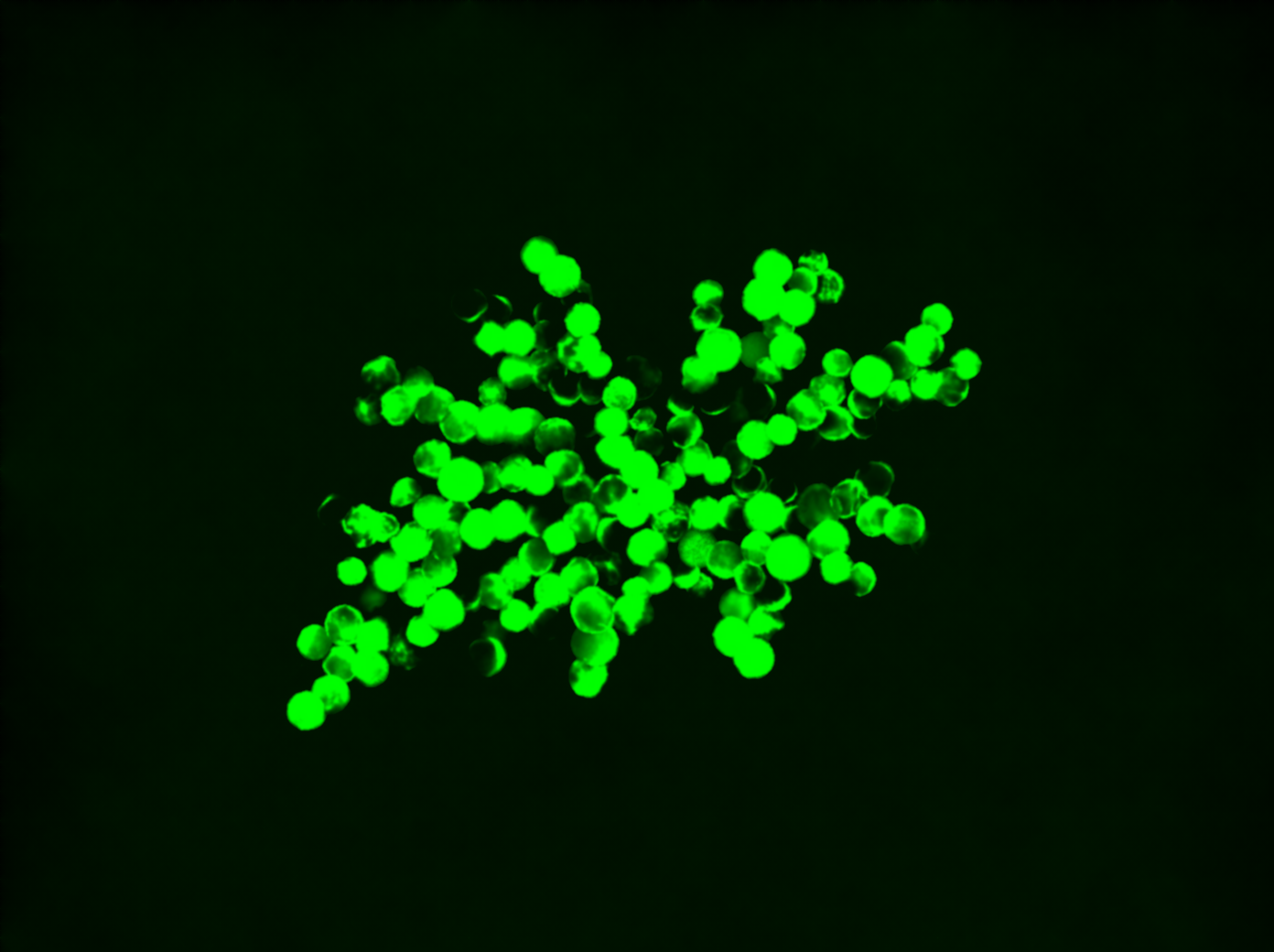

Supplement: Additional file 5 — The zip archive contains simulated images showing protoplasts with corresponding ground truth. (ZIP 72704 kb) [file 12859_2017_1591_MOESM5_ESM.zip › simulated protoplasts/overlaying/overlaying020.png]

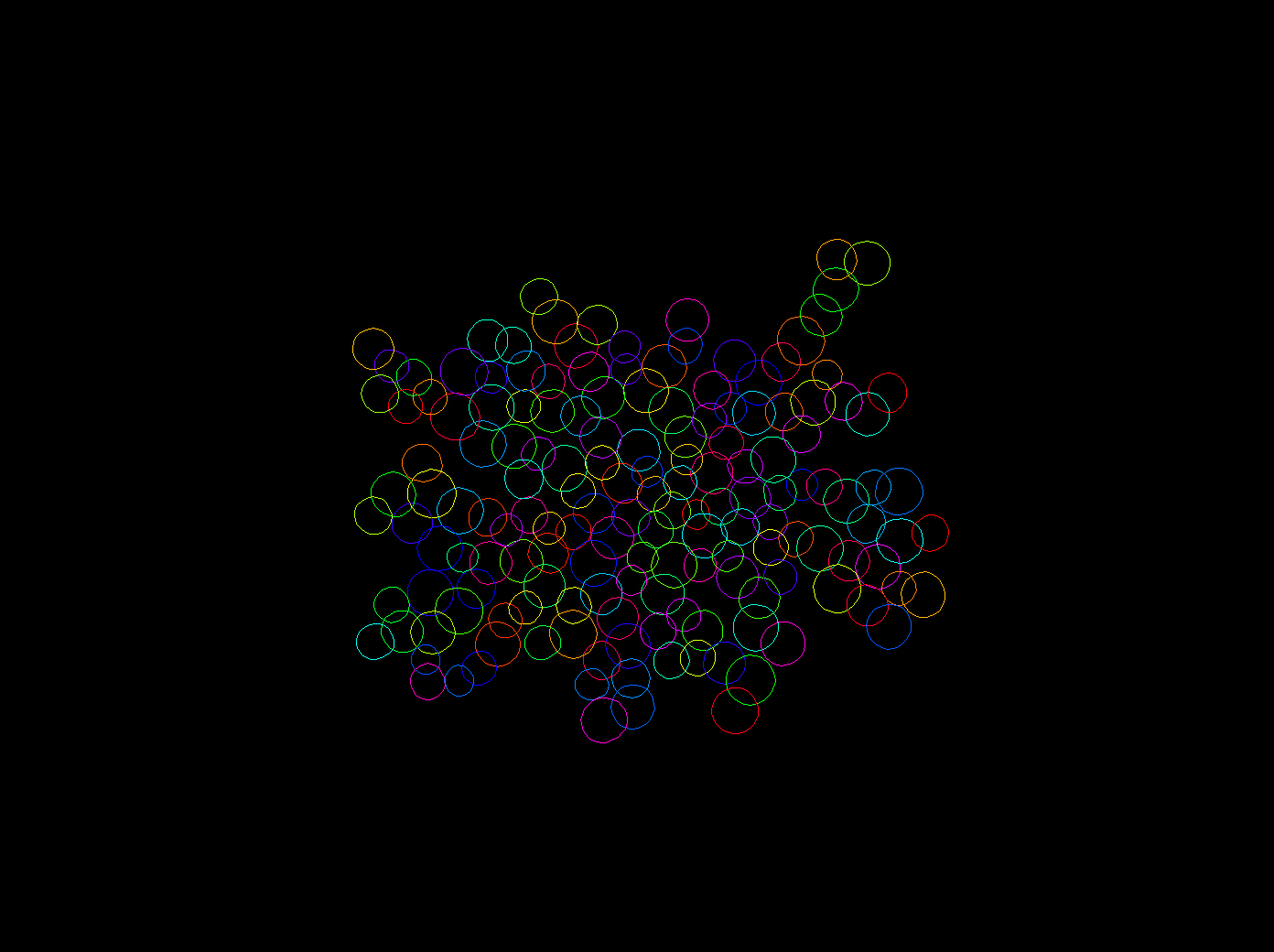

Supplement: Additional file 5 — The zip archive contains simulated images showing protoplasts with corresponding ground truth. (ZIP 72704 kb) [file 12859_2017_1591_MOESM5_ESM.zip › simulated protoplasts/overlaying/overlaying021 gt.png]

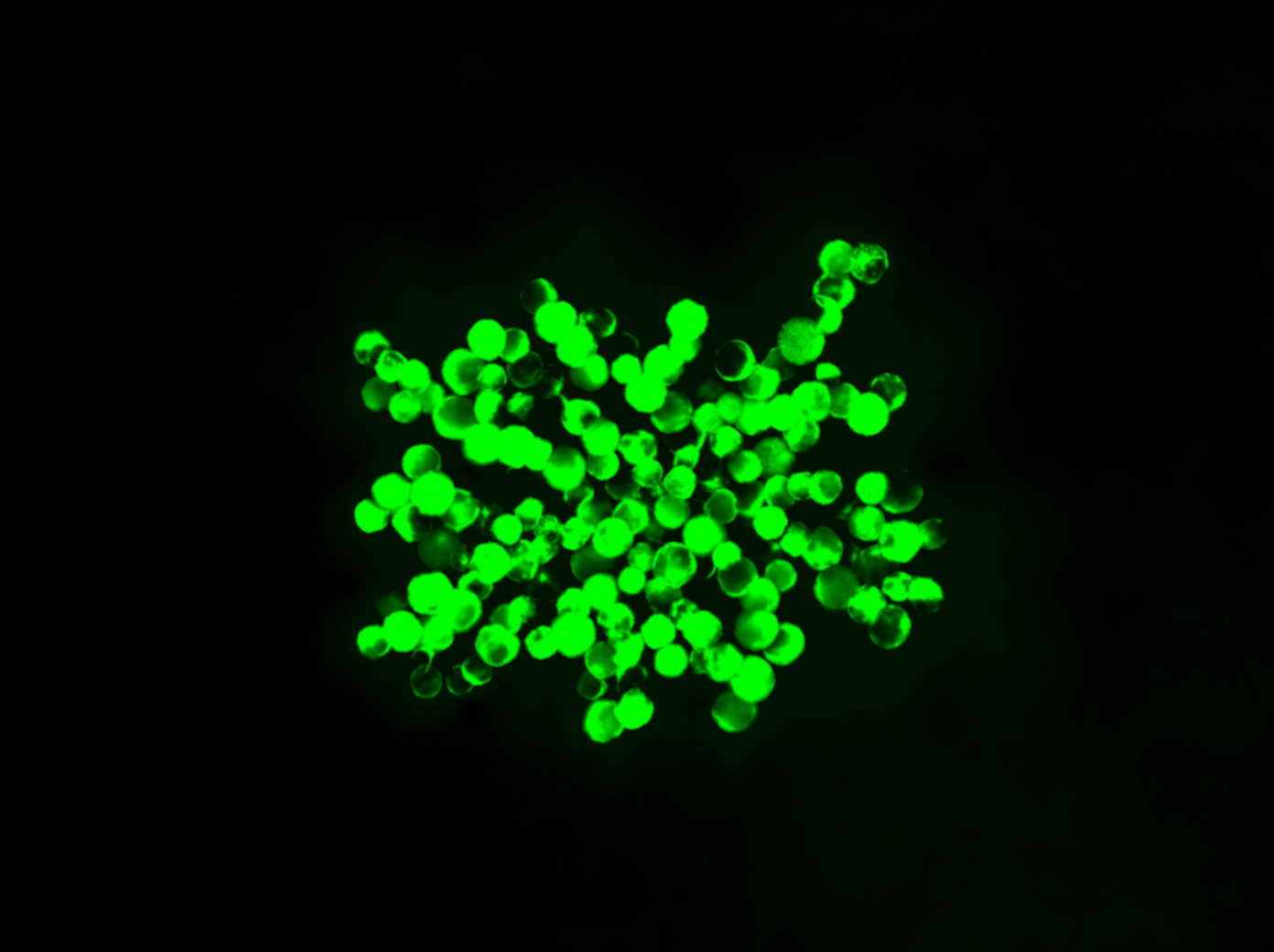

Supplement: Additional file 5 — The zip archive contains simulated images showing protoplasts with corresponding ground truth. (ZIP 72704 kb) [file 12859_2017_1591_MOESM5_ESM.zip › simulated protoplasts/overlaying/overlaying021.png]

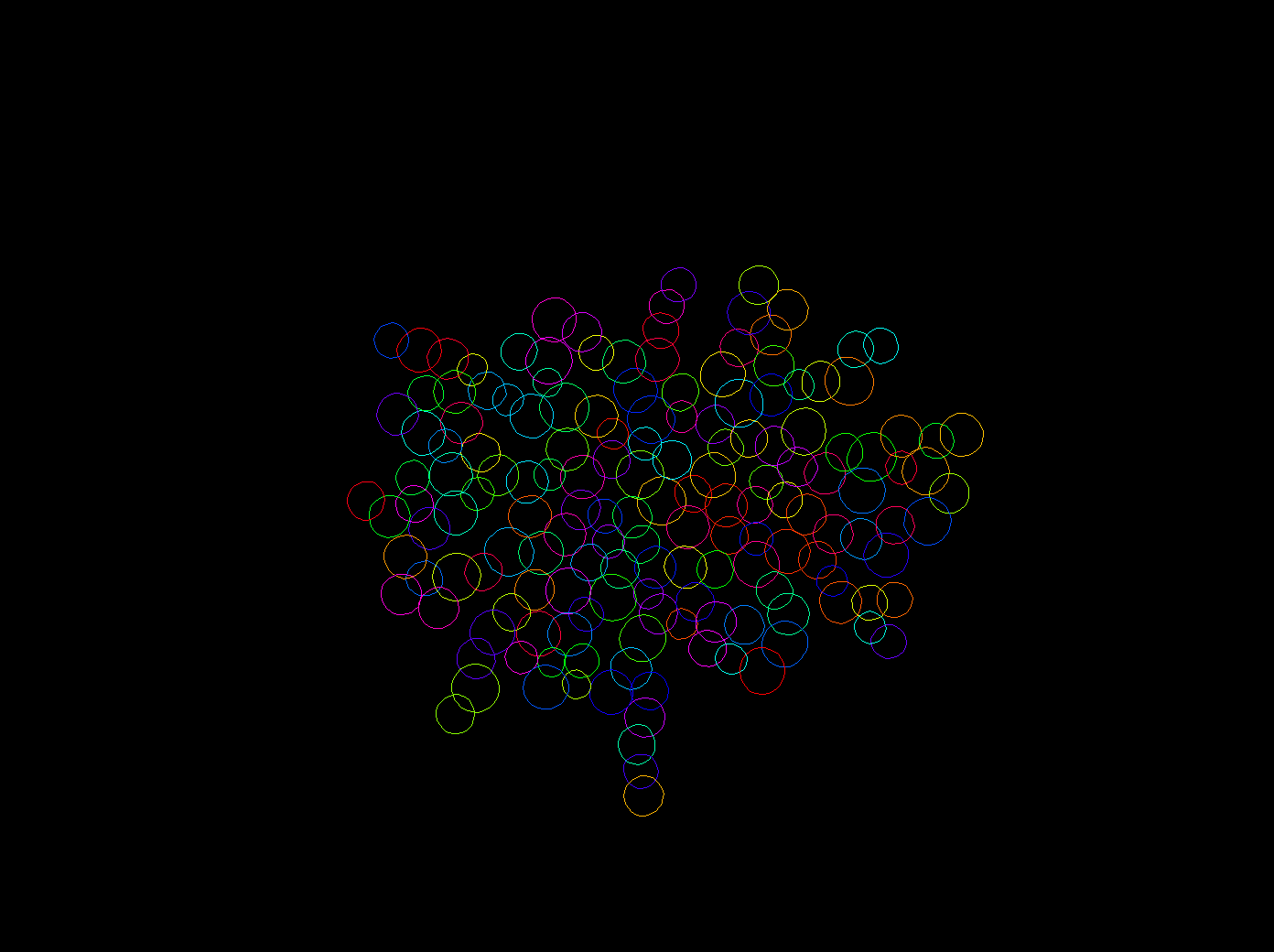

Supplement: Additional file 5 — The zip archive contains simulated images showing protoplasts with corresponding ground truth. (ZIP 72704 kb) [file 12859_2017_1591_MOESM5_ESM.zip › simulated protoplasts/overlaying/overlaying022 gt.png]

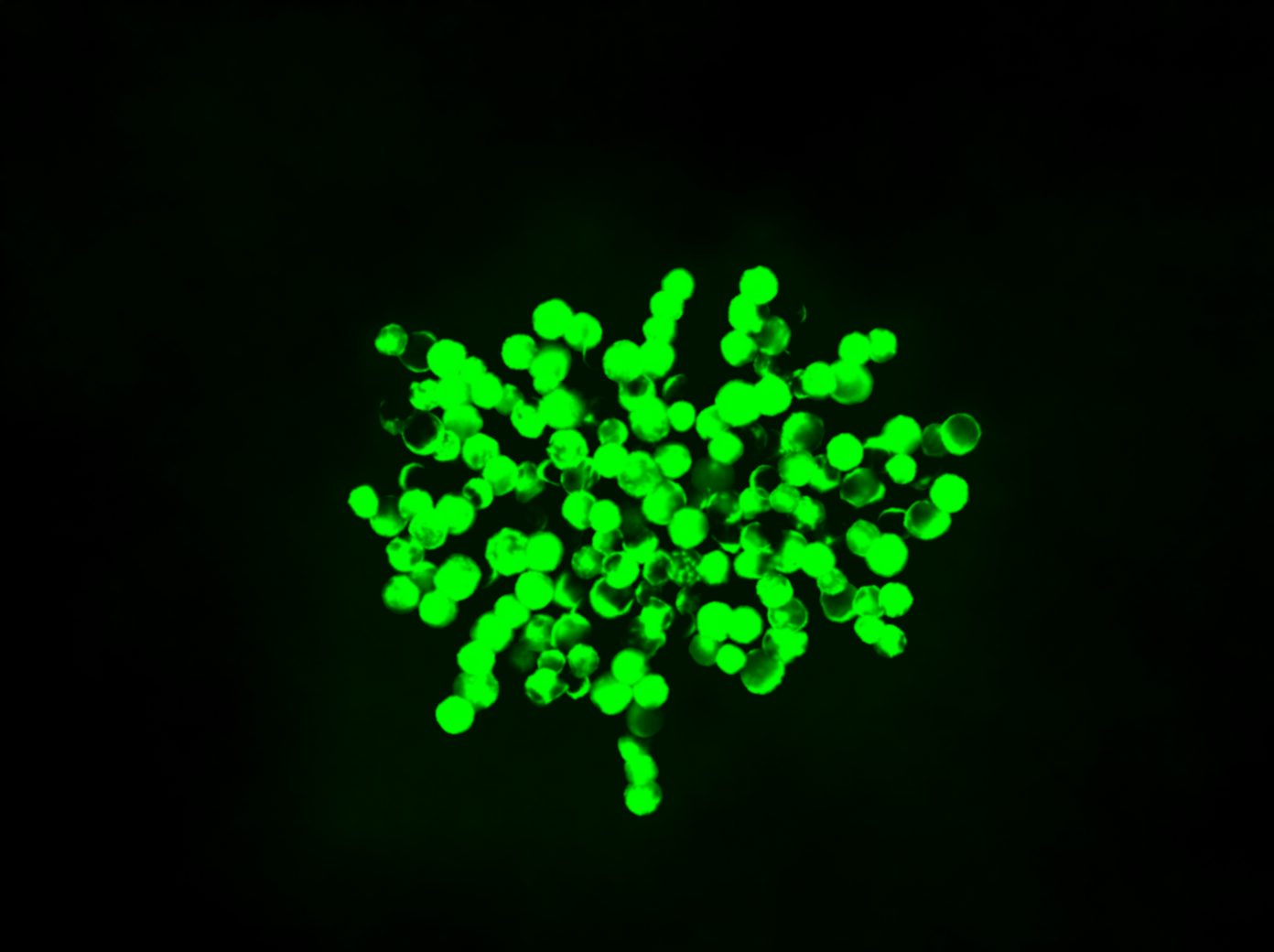

Supplement: Additional file 5 — The zip archive contains simulated images showing protoplasts with corresponding ground truth. (ZIP 72704 kb) [file 12859_2017_1591_MOESM5_ESM.zip › simulated protoplasts/overlaying/overlaying022.png]

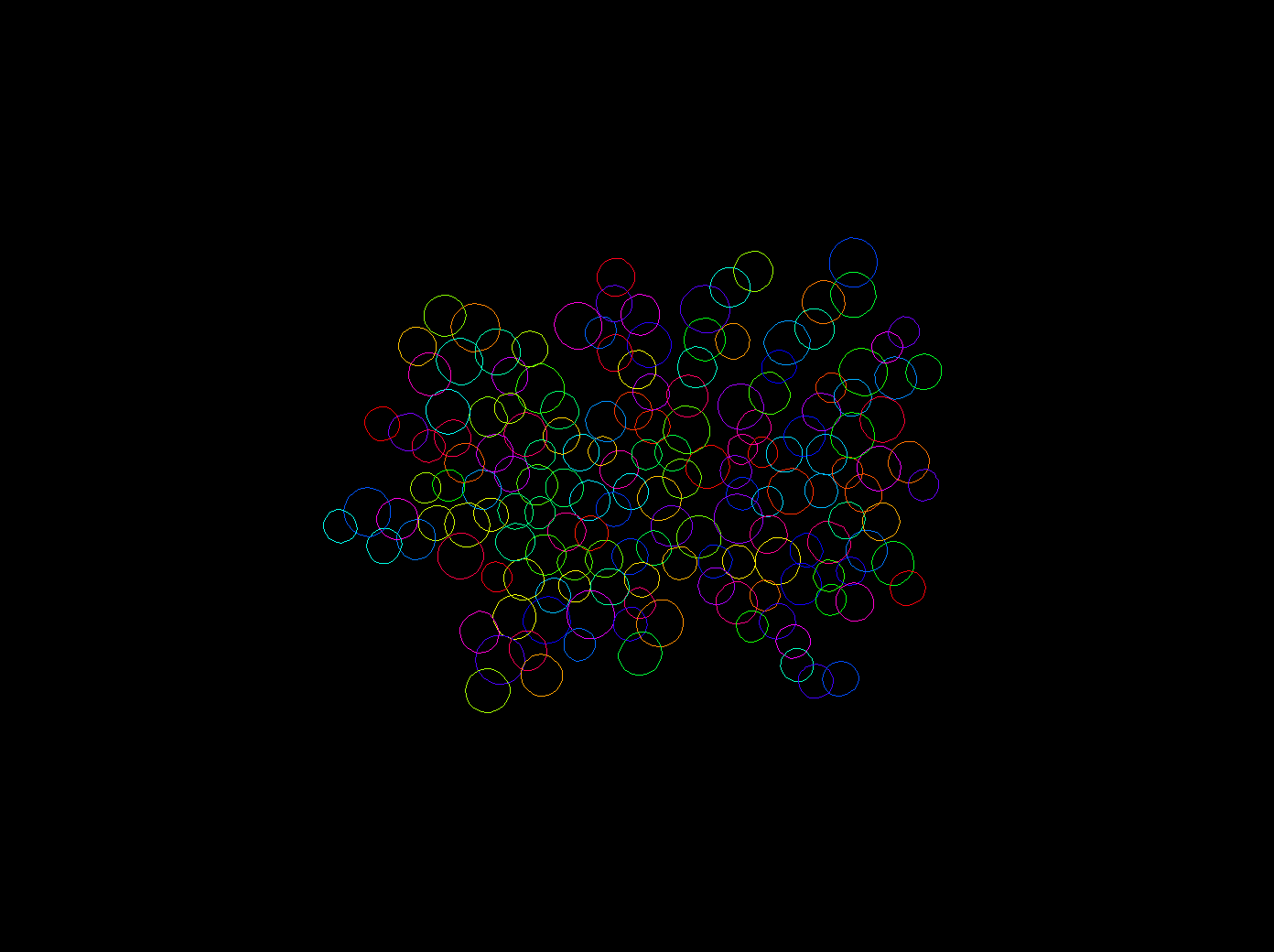

Supplement: Additional file 5 — The zip archive contains simulated images showing protoplasts with corresponding ground truth. (ZIP 72704 kb) [file 12859_2017_1591_MOESM5_ESM.zip › simulated protoplasts/overlaying/overlaying023 gt.png]

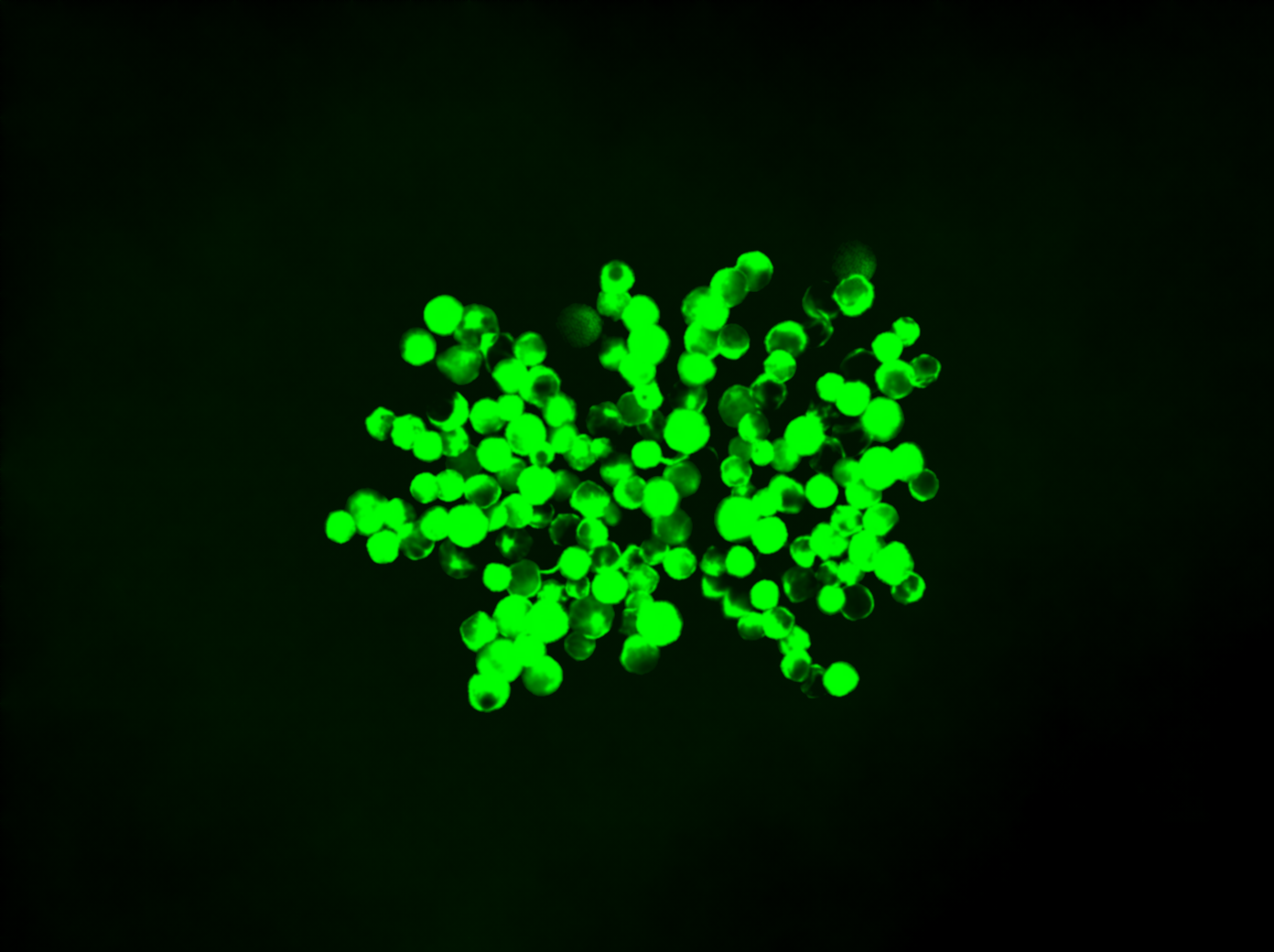

Supplement: Additional file 5 — The zip archive contains simulated images showing protoplasts with corresponding ground truth. (ZIP 72704 kb) [file 12859_2017_1591_MOESM5_ESM.zip › simulated protoplasts/overlaying/overlaying023.png]

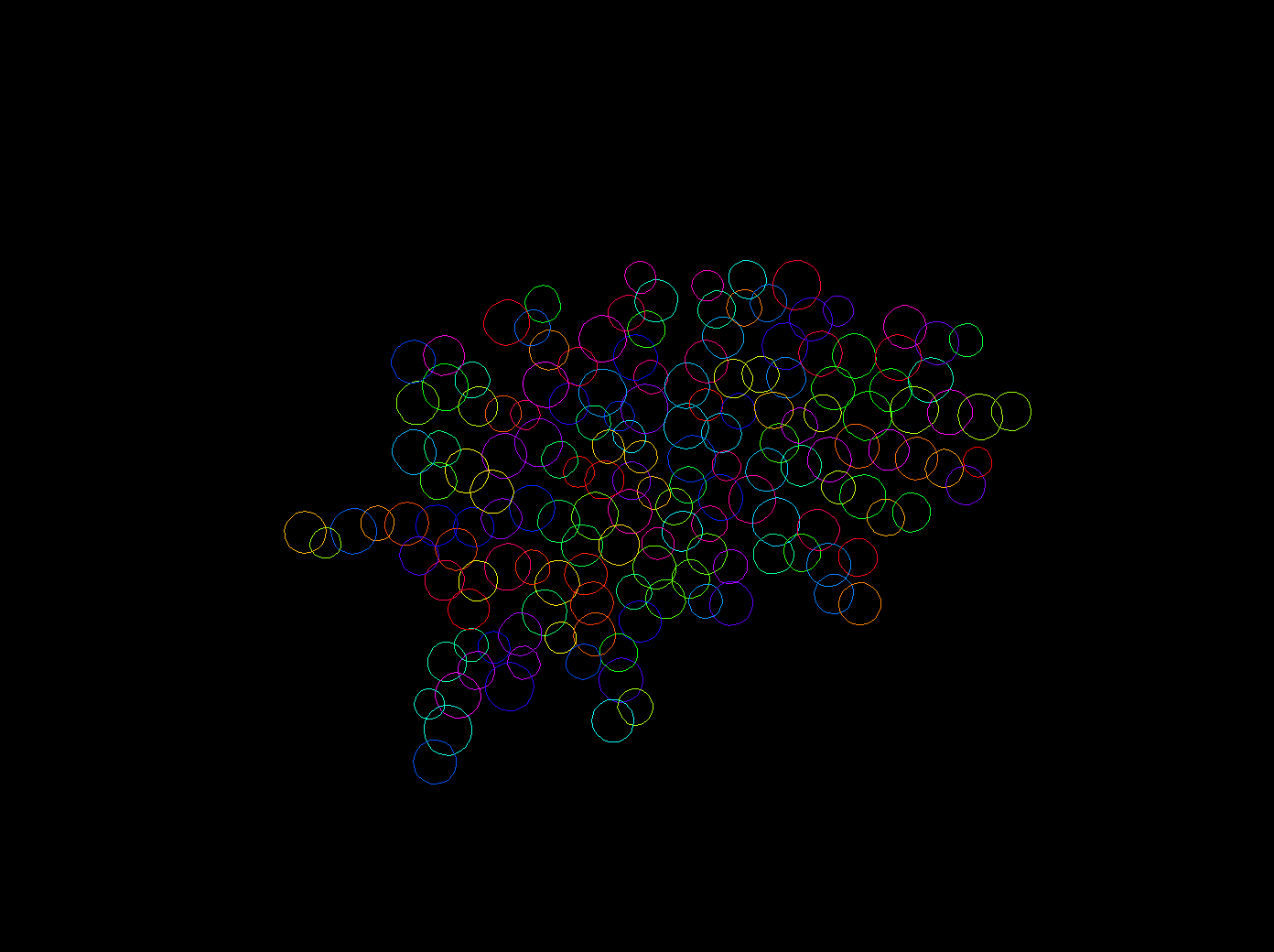

Supplement: Additional file 5 — The zip archive contains simulated images showing protoplasts with corresponding ground truth. (ZIP 72704 kb) [file 12859_2017_1591_MOESM5_ESM.zip › simulated protoplasts/overlaying/overlaying024 gt.png]

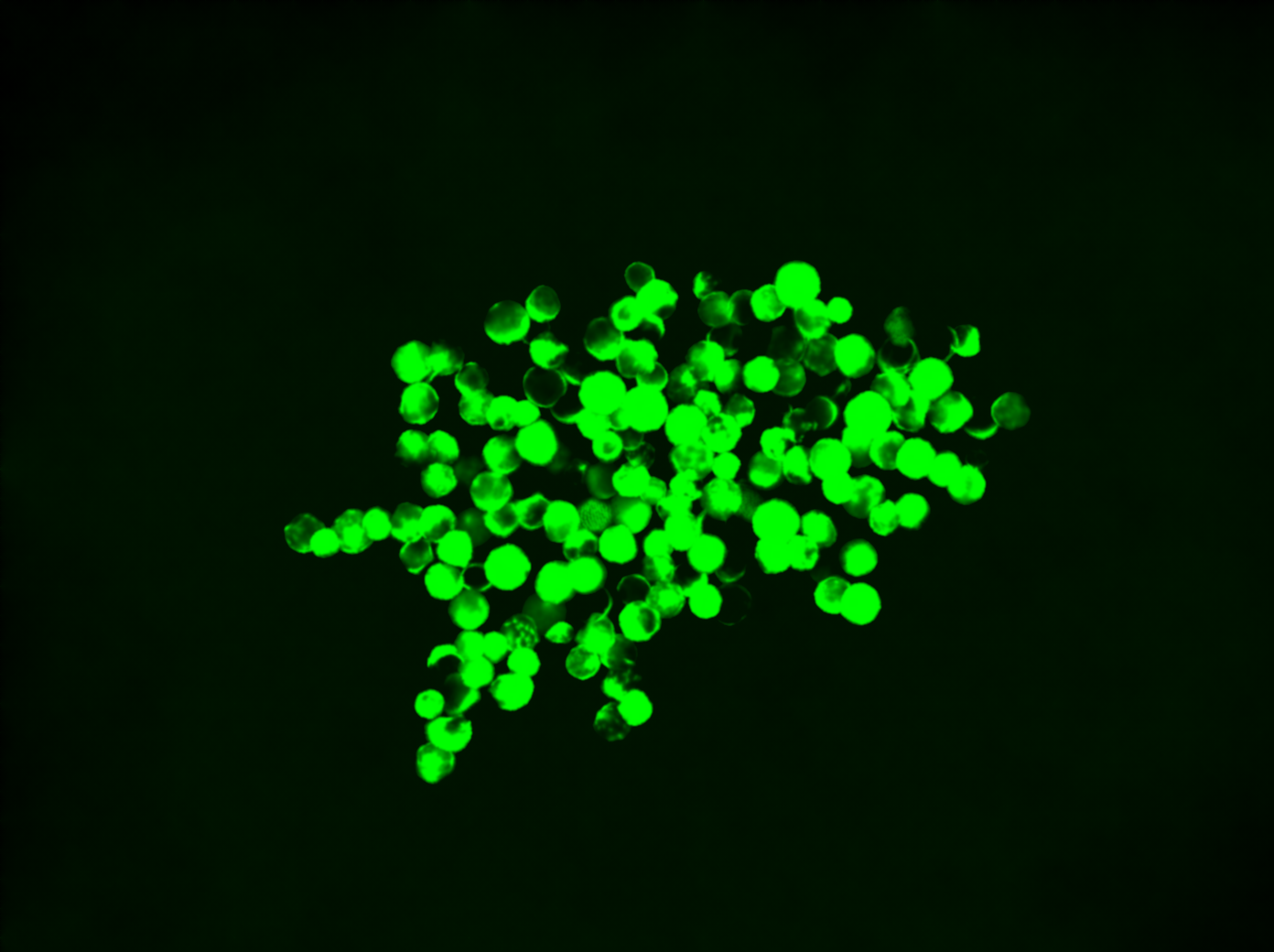

Supplement: Additional file 5 — The zip archive contains simulated images showing protoplasts with corresponding ground truth. (ZIP 72704 kb) [file 12859_2017_1591_MOESM5_ESM.zip › simulated protoplasts/overlaying/overlaying024.png]

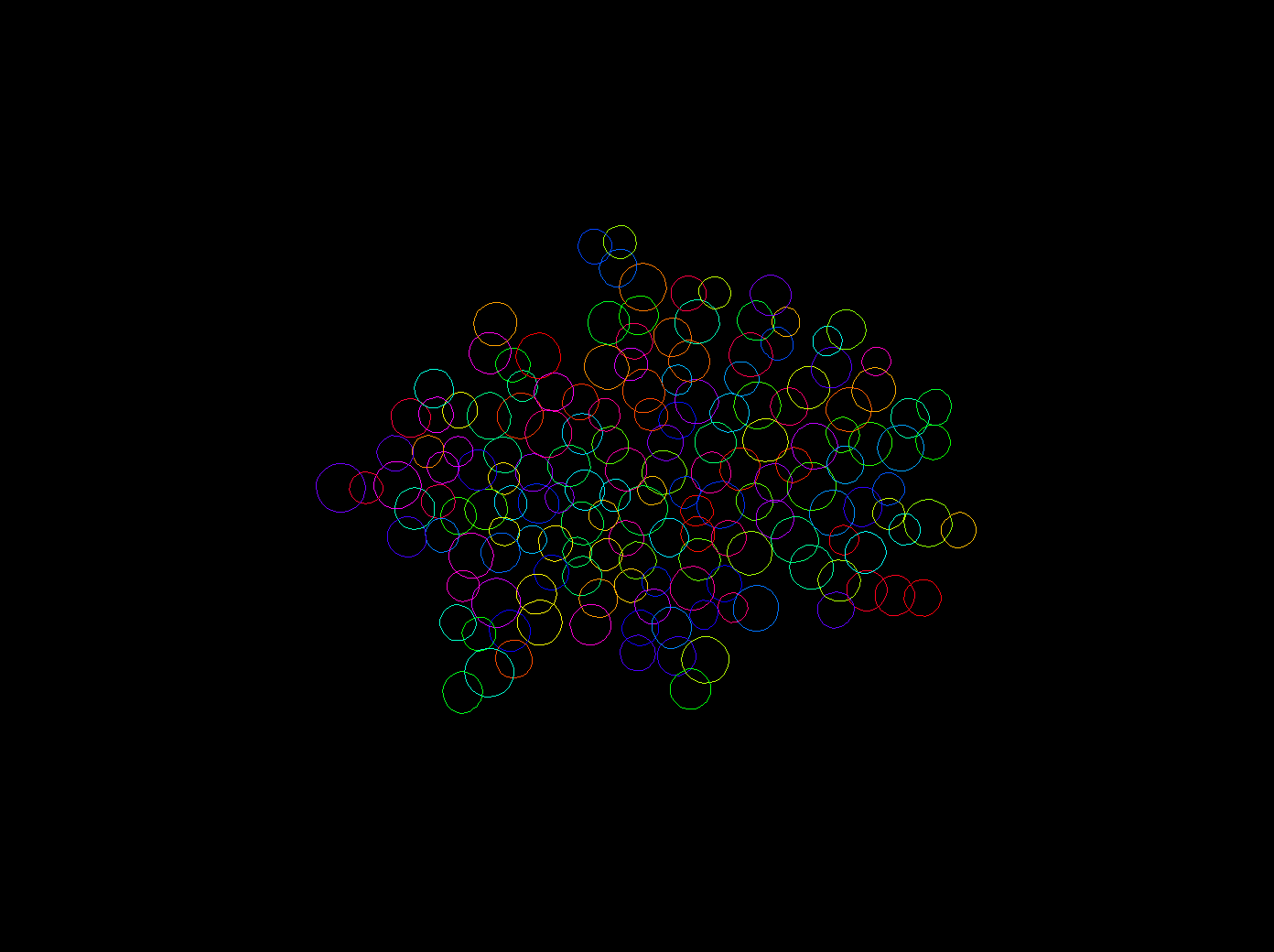

Supplement: Additional file 5 — The zip archive contains simulated images showing protoplasts with corresponding ground truth. (ZIP 72704 kb) [file 12859_2017_1591_MOESM5_ESM.zip › simulated protoplasts/overlaying/overlaying025 gt.png]

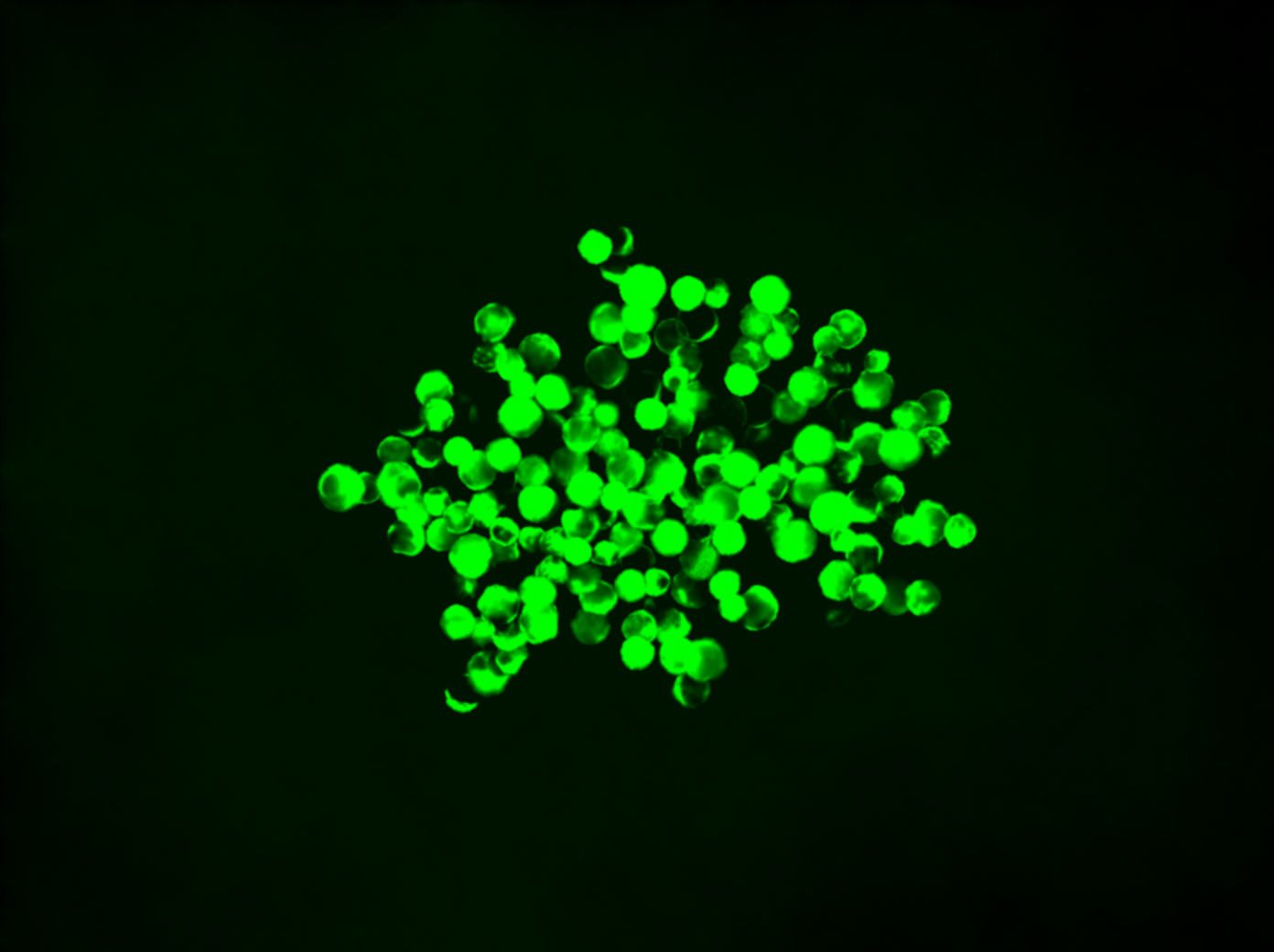

Supplement: Additional file 5 — The zip archive contains simulated images showing protoplasts with corresponding ground truth. (ZIP 72704 kb) [file 12859_2017_1591_MOESM5_ESM.zip › simulated protoplasts/overlaying/overlaying025.png]

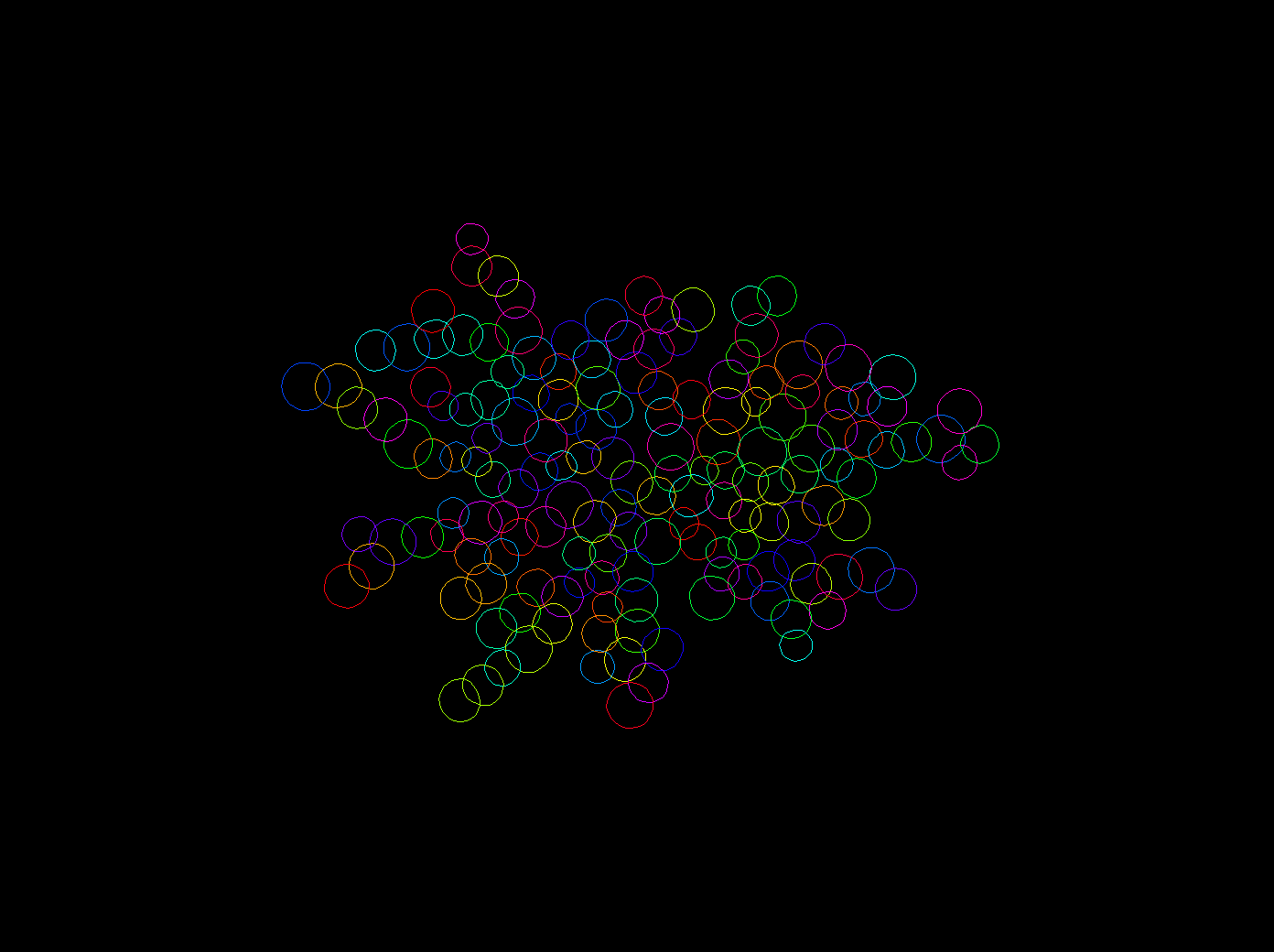

Supplement: Additional file 5 — The zip archive contains simulated images showing protoplasts with corresponding ground truth. (ZIP 72704 kb) [file 12859_2017_1591_MOESM5_ESM.zip › simulated protoplasts/overlaying/overlaying026 gt.png]

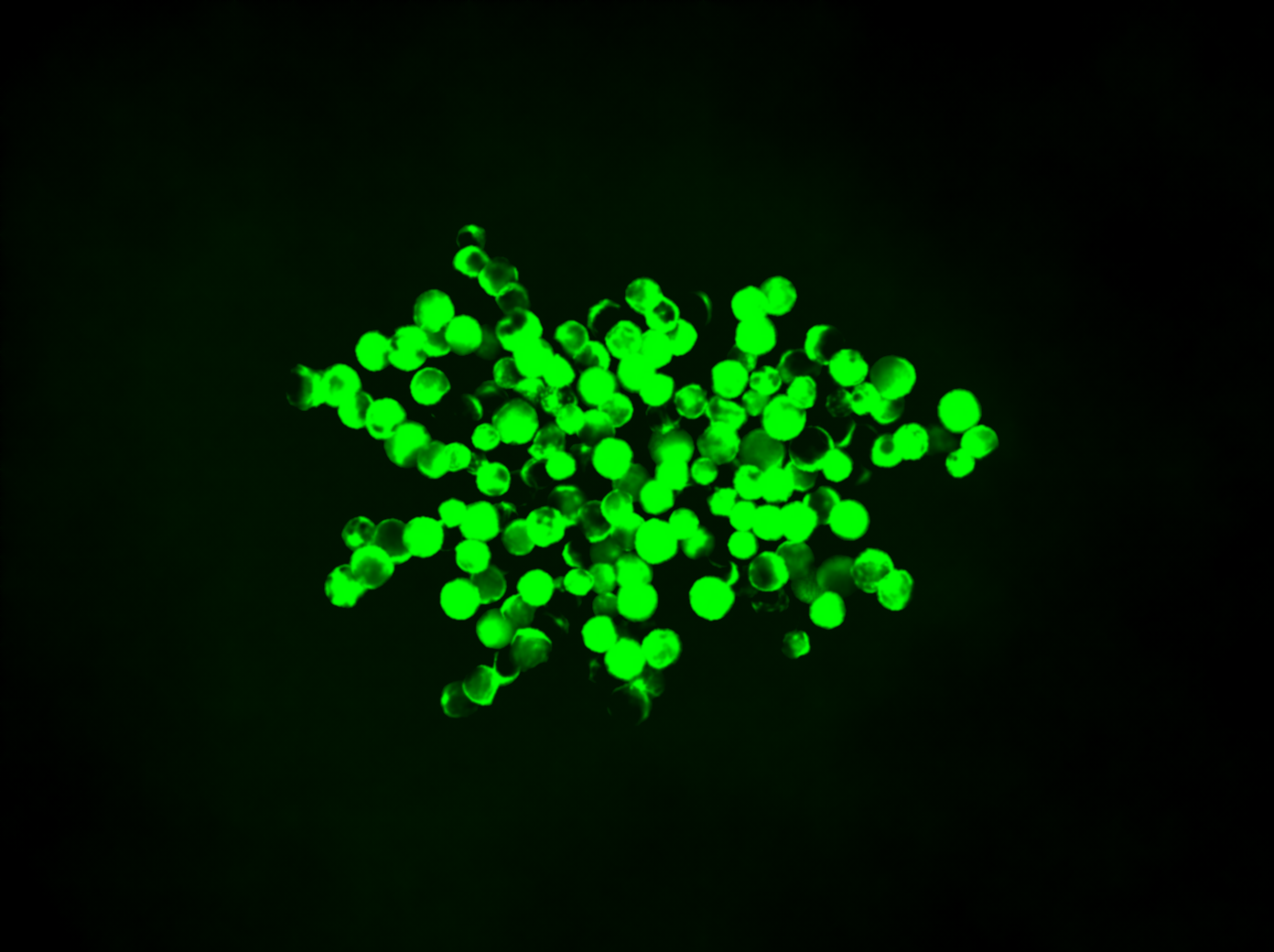

Supplement: Additional file 5 — The zip archive contains simulated images showing protoplasts with corresponding ground truth. (ZIP 72704 kb) [file 12859_2017_1591_MOESM5_ESM.zip › simulated protoplasts/overlaying/overlaying026.png]

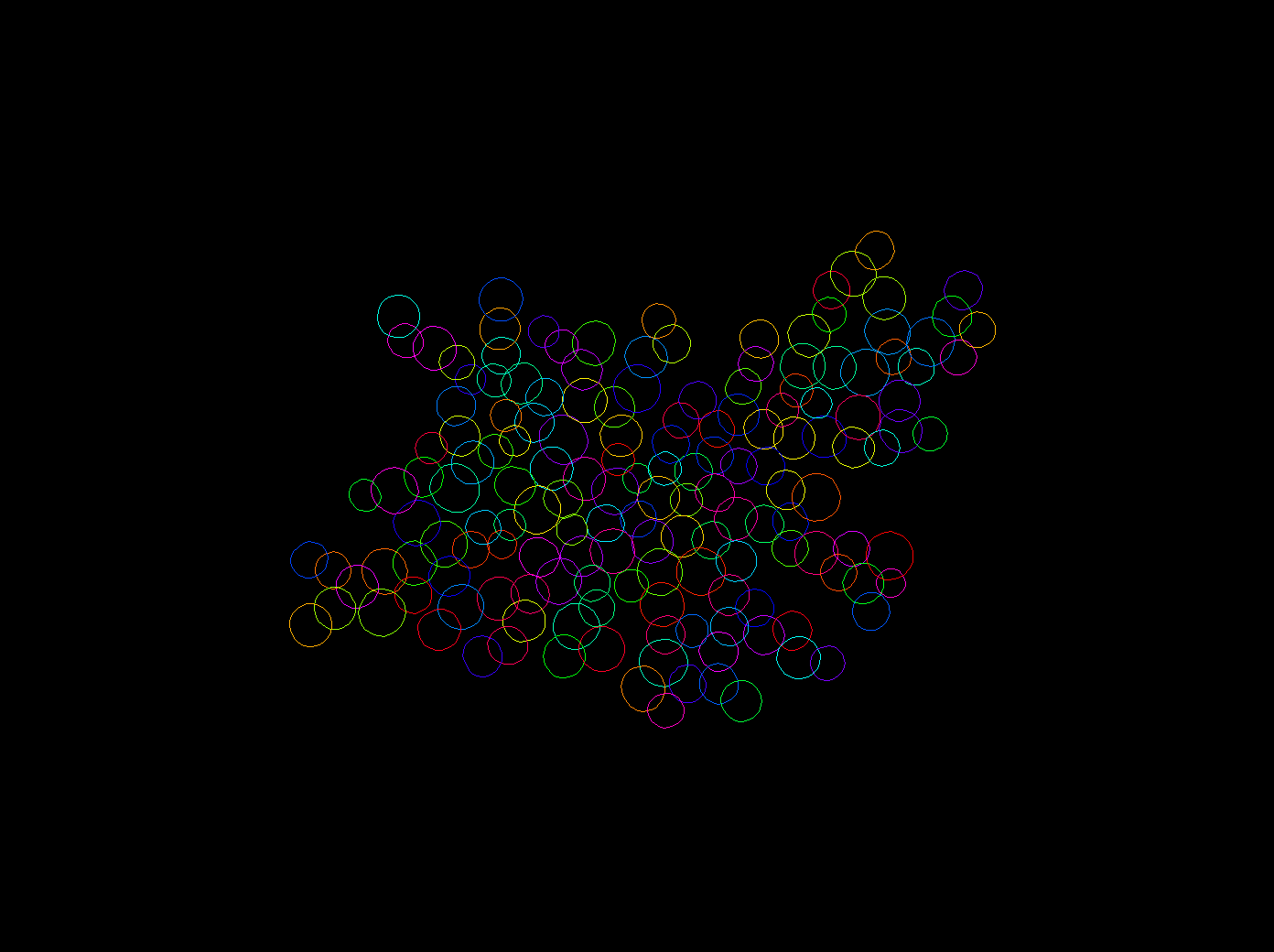

Supplement: Additional file 5 — The zip archive contains simulated images showing protoplasts with corresponding ground truth. (ZIP 72704 kb) [file 12859_2017_1591_MOESM5_ESM.zip › simulated protoplasts/overlaying/overlaying027 gt.png]

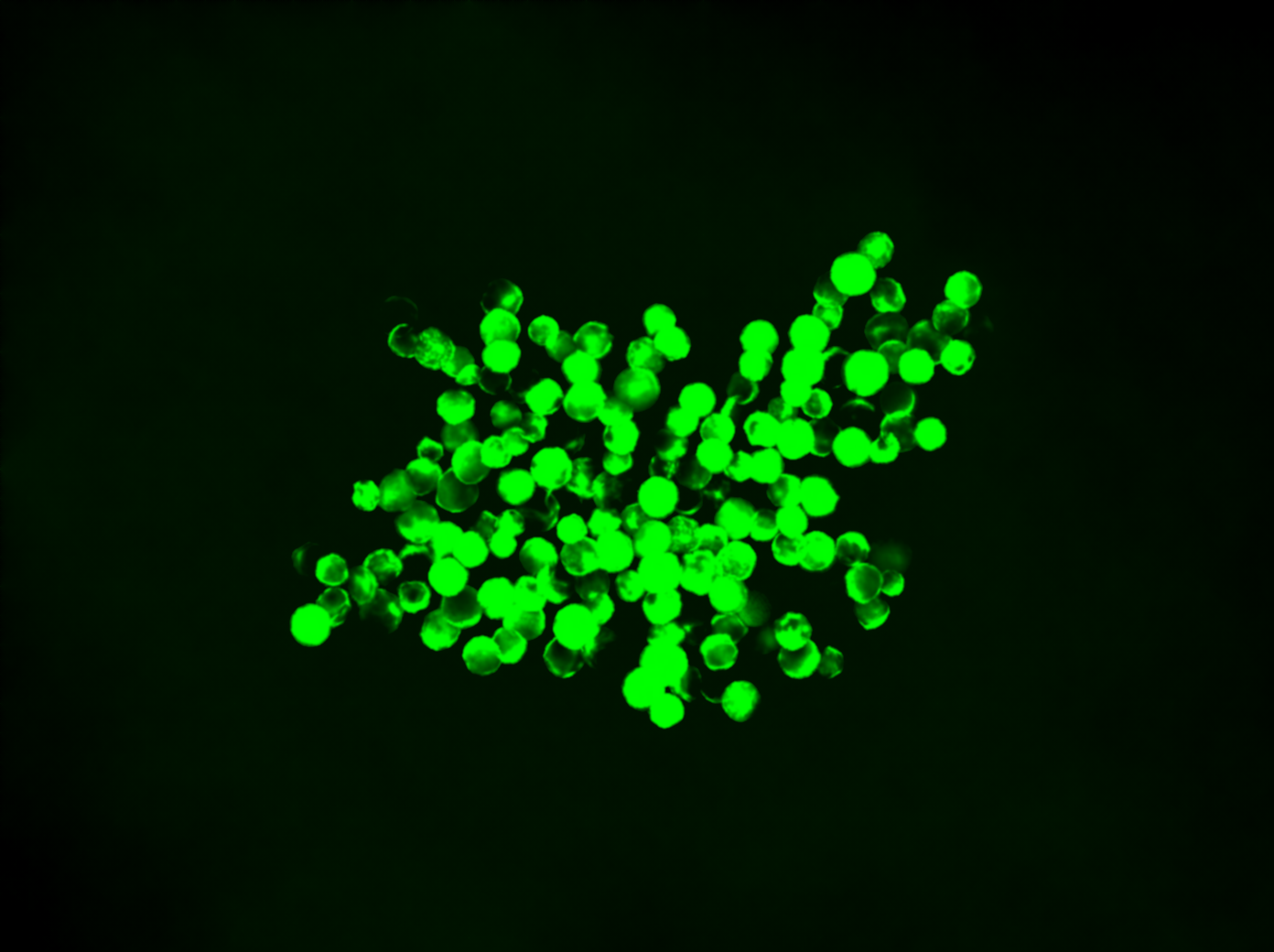

Supplement: Additional file 5 — The zip archive contains simulated images showing protoplasts with corresponding ground truth. (ZIP 72704 kb) [file 12859_2017_1591_MOESM5_ESM.zip › simulated protoplasts/overlaying/overlaying027.png]

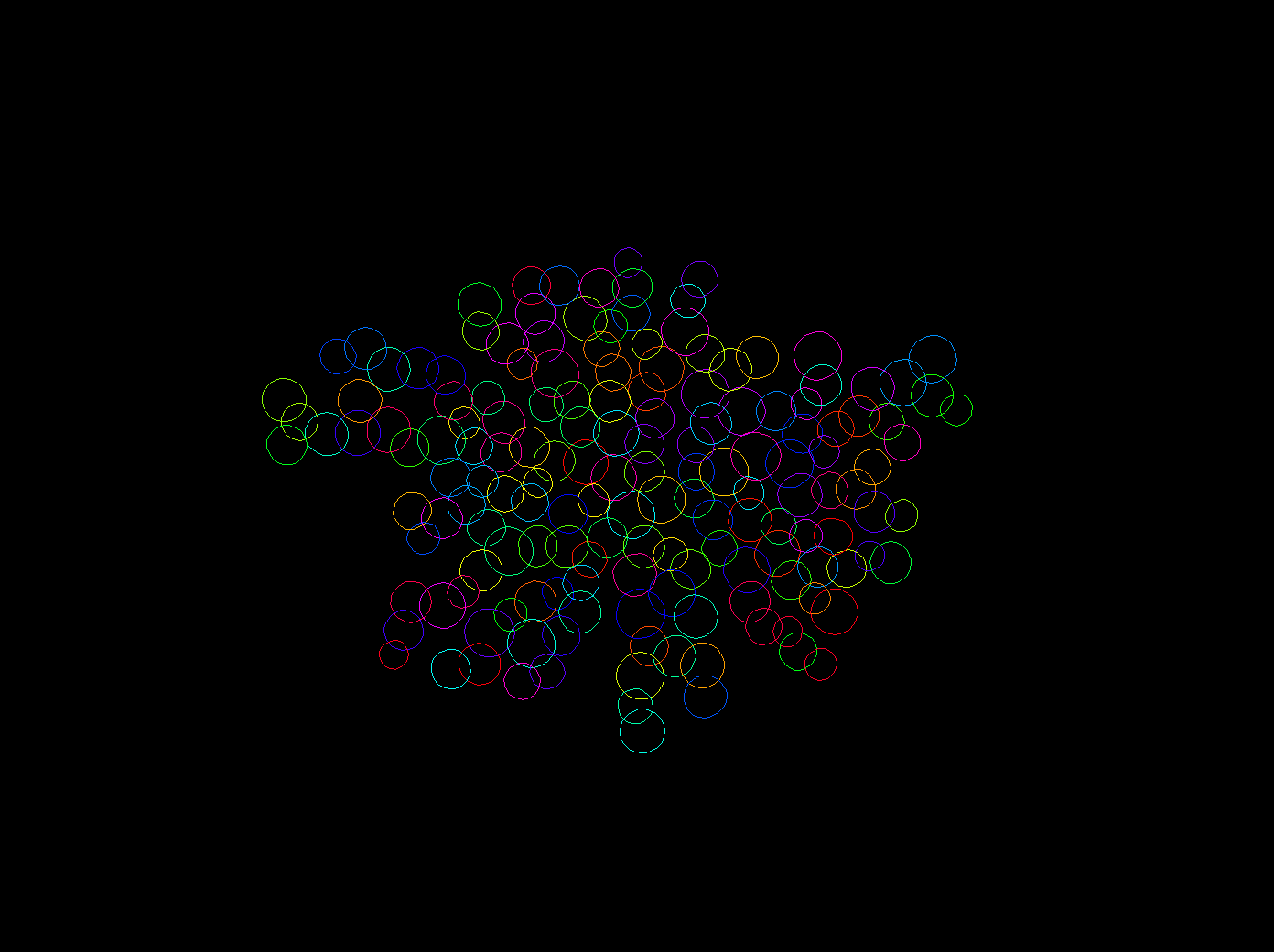

Supplement: Additional file 5 — The zip archive contains simulated images showing protoplasts with corresponding ground truth. (ZIP 72704 kb) [file 12859_2017_1591_MOESM5_ESM.zip › simulated protoplasts/overlaying/overlaying028 gt.png]

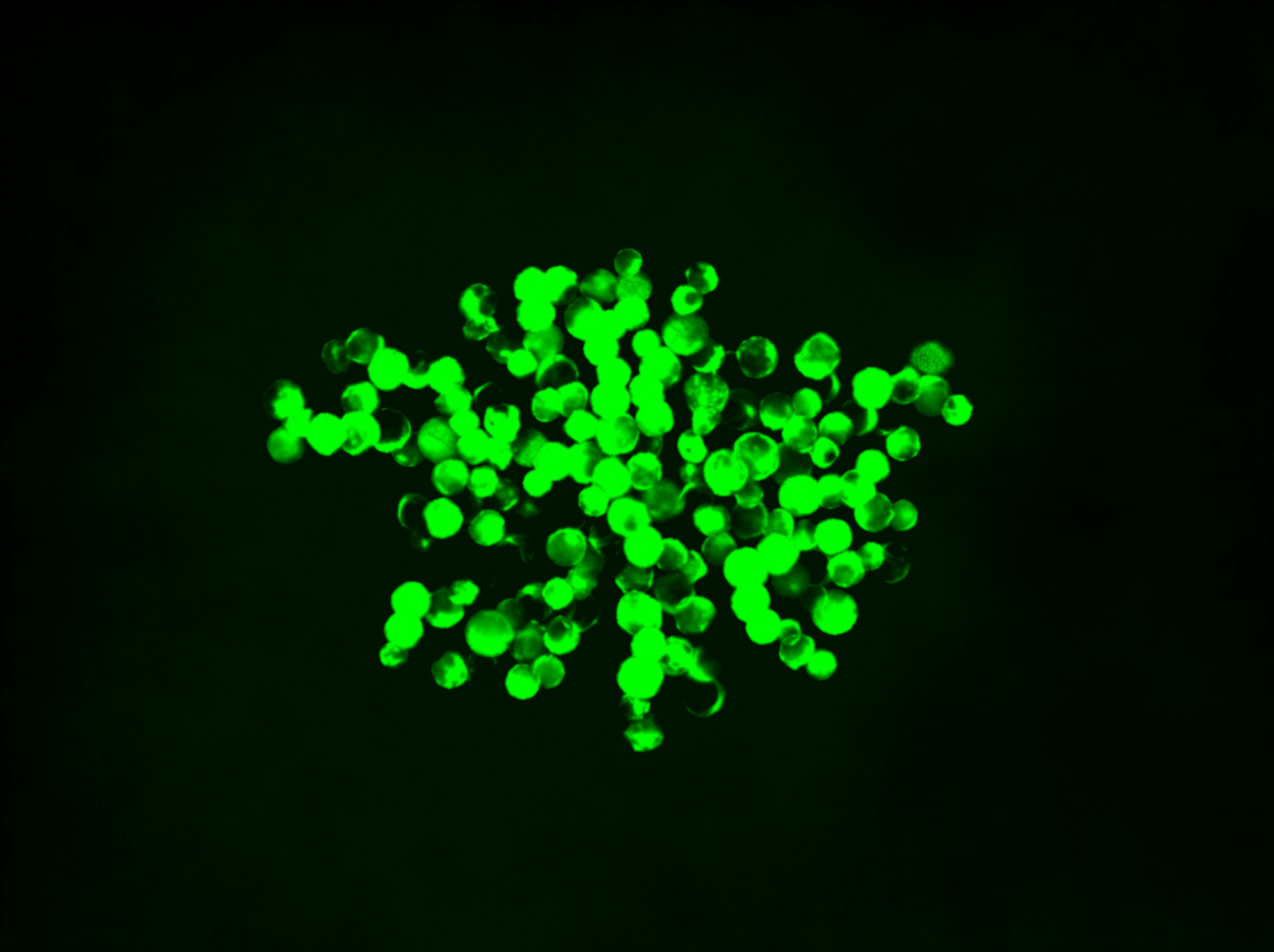

Supplement: Additional file 5 — The zip archive contains simulated images showing protoplasts with corresponding ground truth. (ZIP 72704 kb) [file 12859_2017_1591_MOESM5_ESM.zip › simulated protoplasts/overlaying/overlaying028.png]

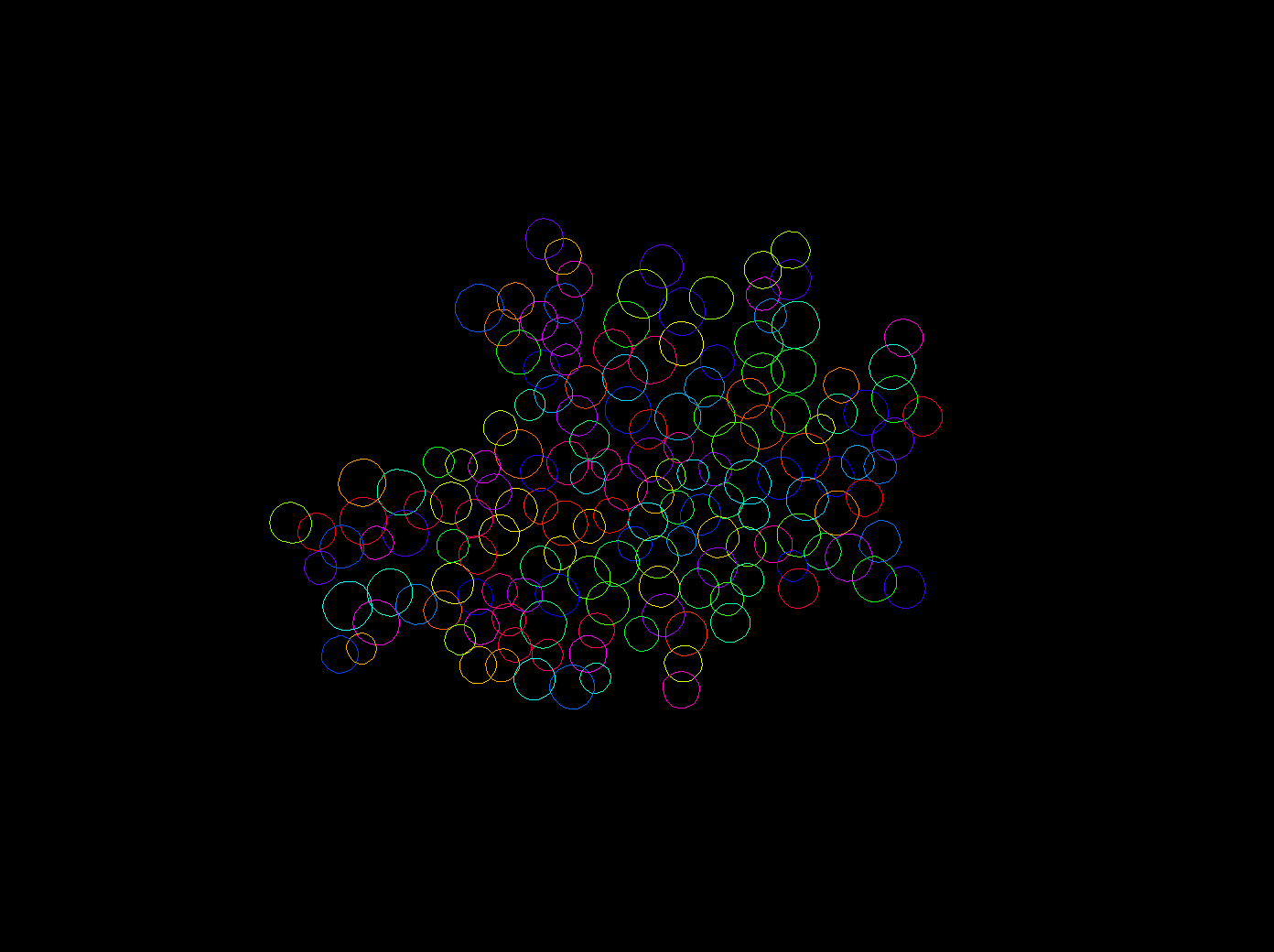

Supplement: Additional file 5 — The zip archive contains simulated images showing protoplasts with corresponding ground truth. (ZIP 72704 kb) [file 12859_2017_1591_MOESM5_ESM.zip › simulated protoplasts/overlaying/overlaying029 gt.png]

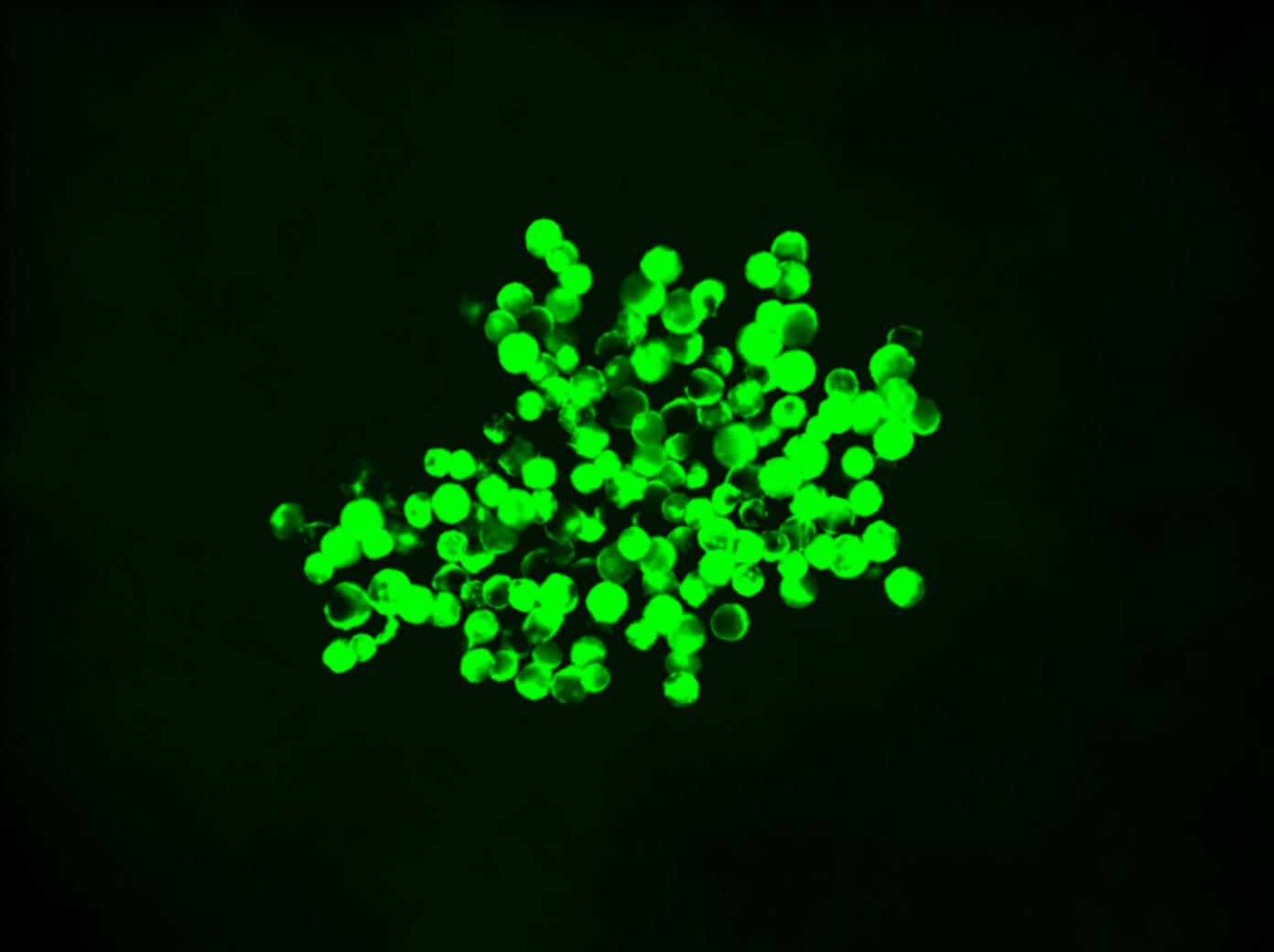

Supplement: Additional file 5 — The zip archive contains simulated images showing protoplasts with corresponding ground truth. (ZIP 72704 kb) [file 12859_2017_1591_MOESM5_ESM.zip › simulated protoplasts/overlaying/overlaying029.png]

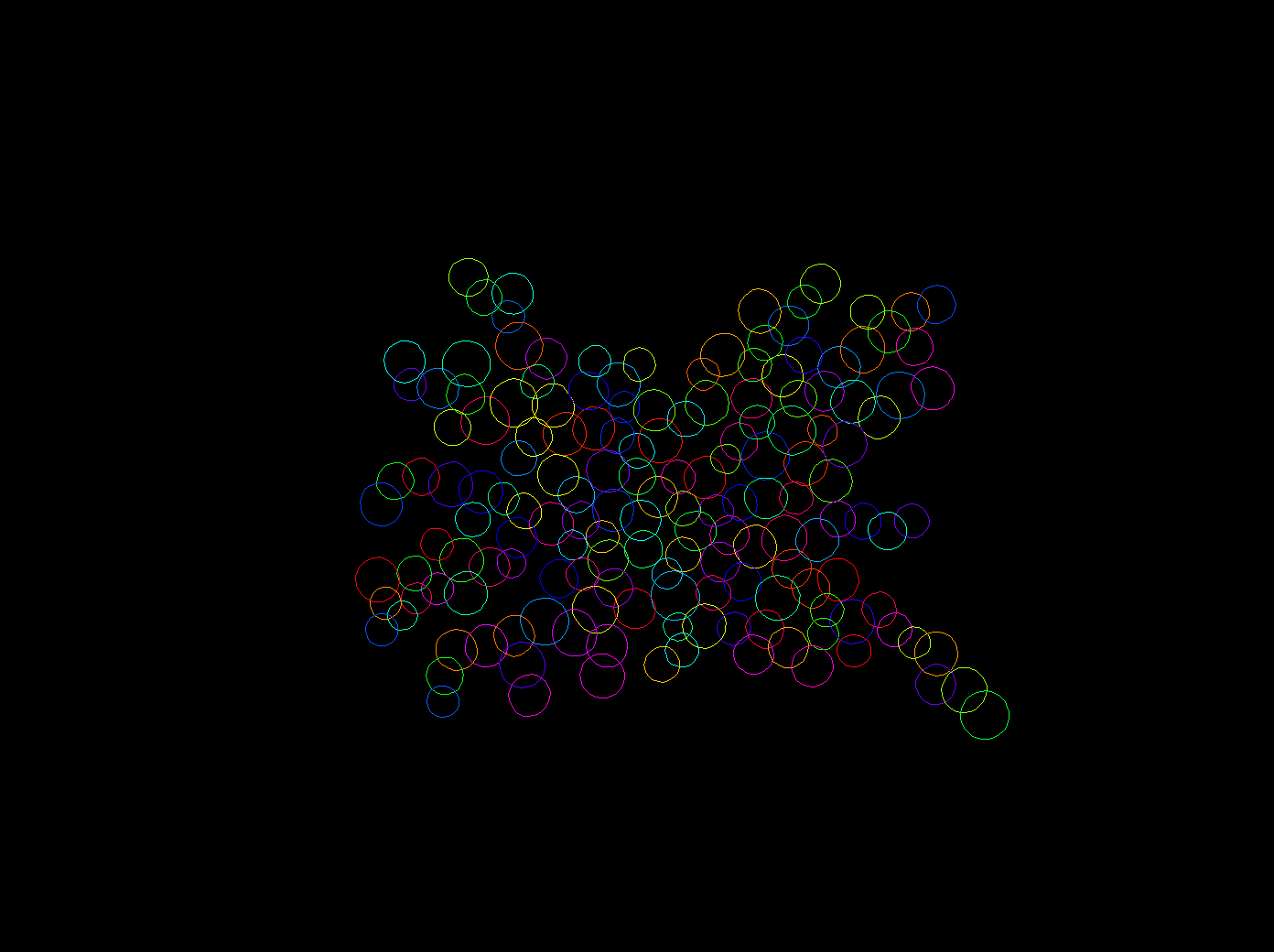

Supplement: Additional file 5 — The zip archive contains simulated images showing protoplasts with corresponding ground truth. (ZIP 72704 kb) [file 12859_2017_1591_MOESM5_ESM.zip › simulated protoplasts/overlaying/overlaying030 gt.png]

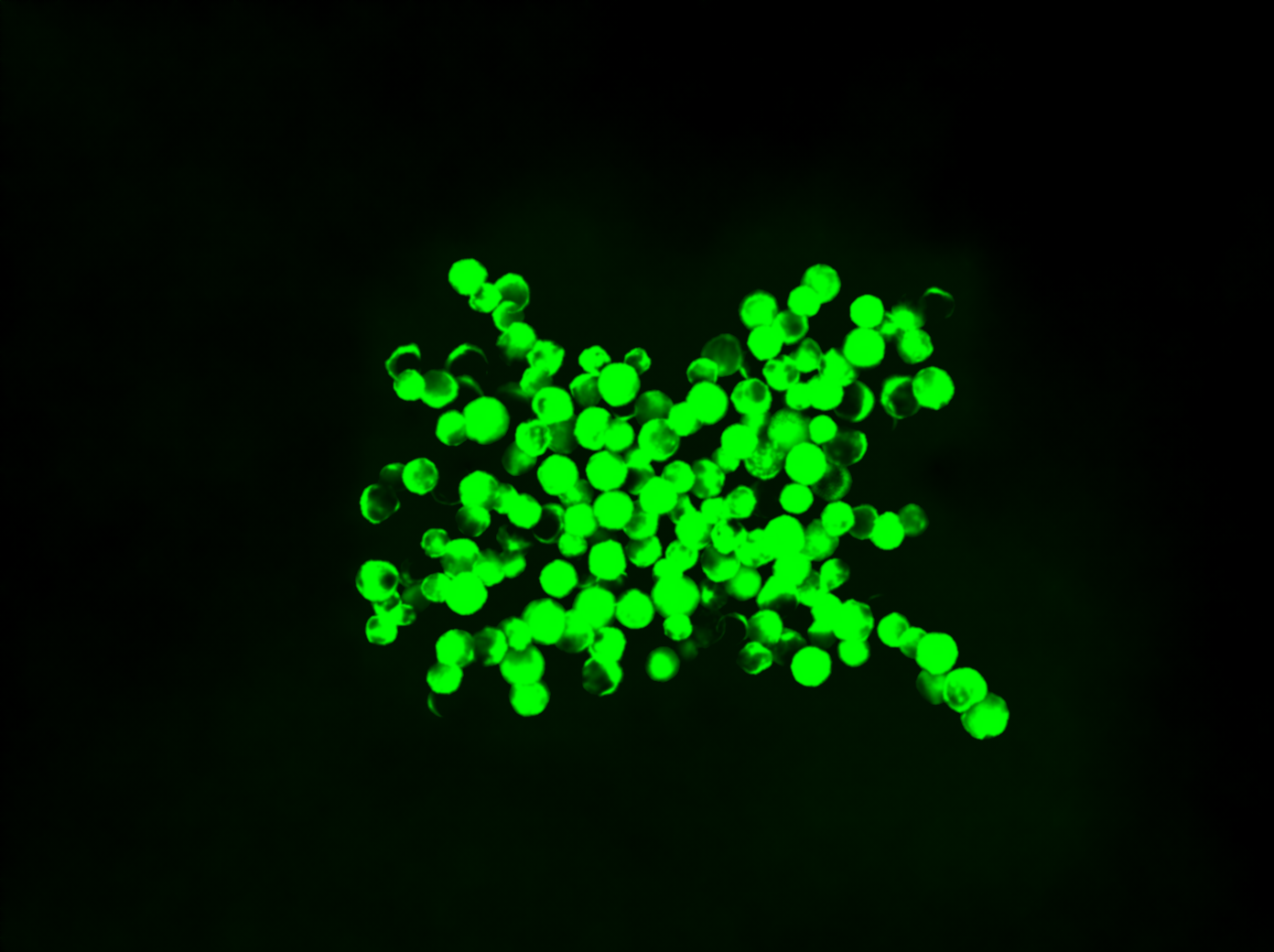

Supplement: Additional file 5 — The zip archive contains simulated images showing protoplasts with corresponding ground truth. (ZIP 72704 kb) [file 12859_2017_1591_MOESM5_ESM.zip › simulated protoplasts/overlaying/overlaying030.png]

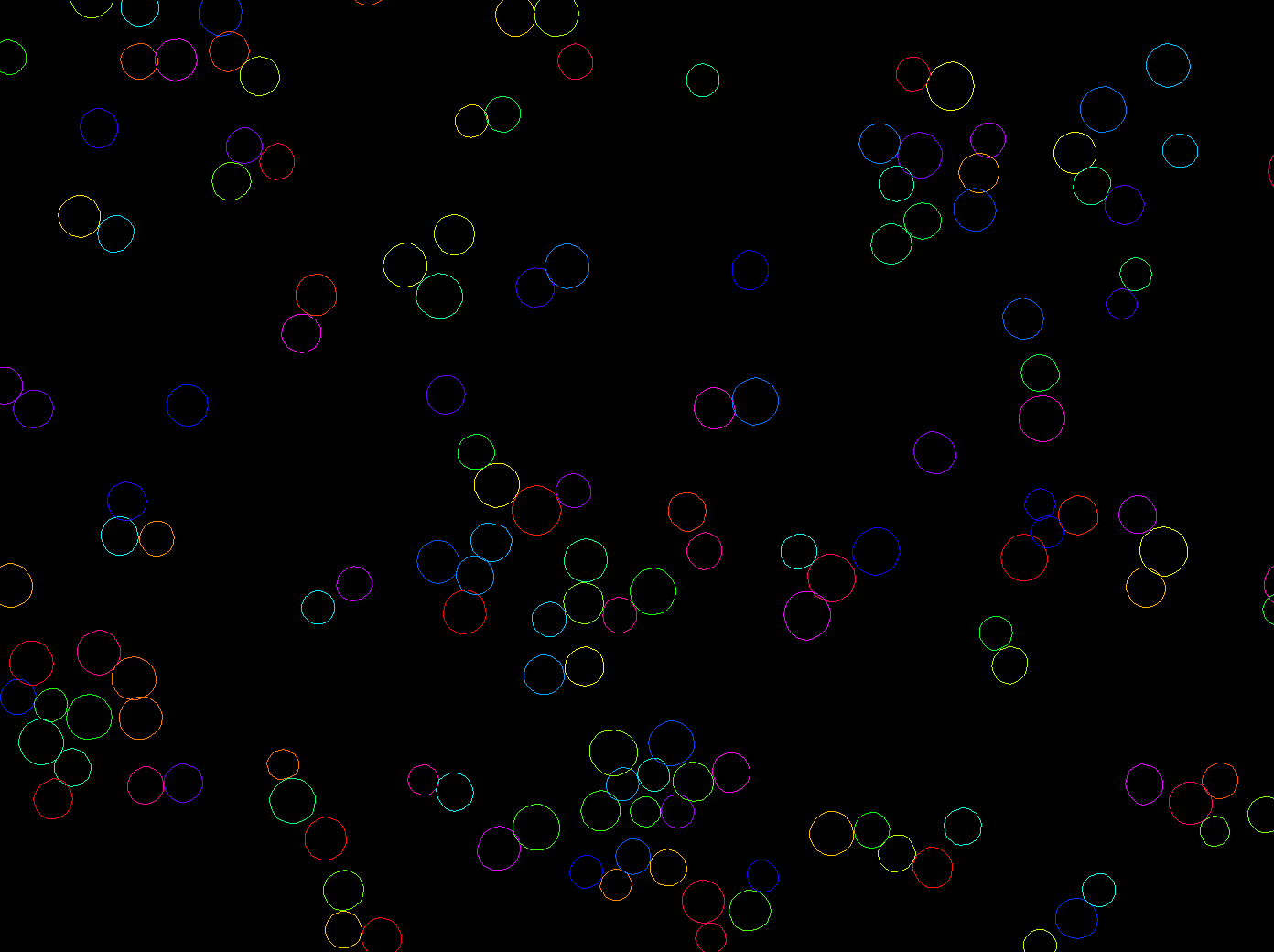

Supplement: Additional file 5 — The zip archive contains simulated images showing protoplasts with corresponding ground truth. (ZIP 72704 kb) [file 12859_2017_1591_MOESM5_ESM.zip › simulated protoplasts/realisticoverlap/realisticoverlap001 gt.png]

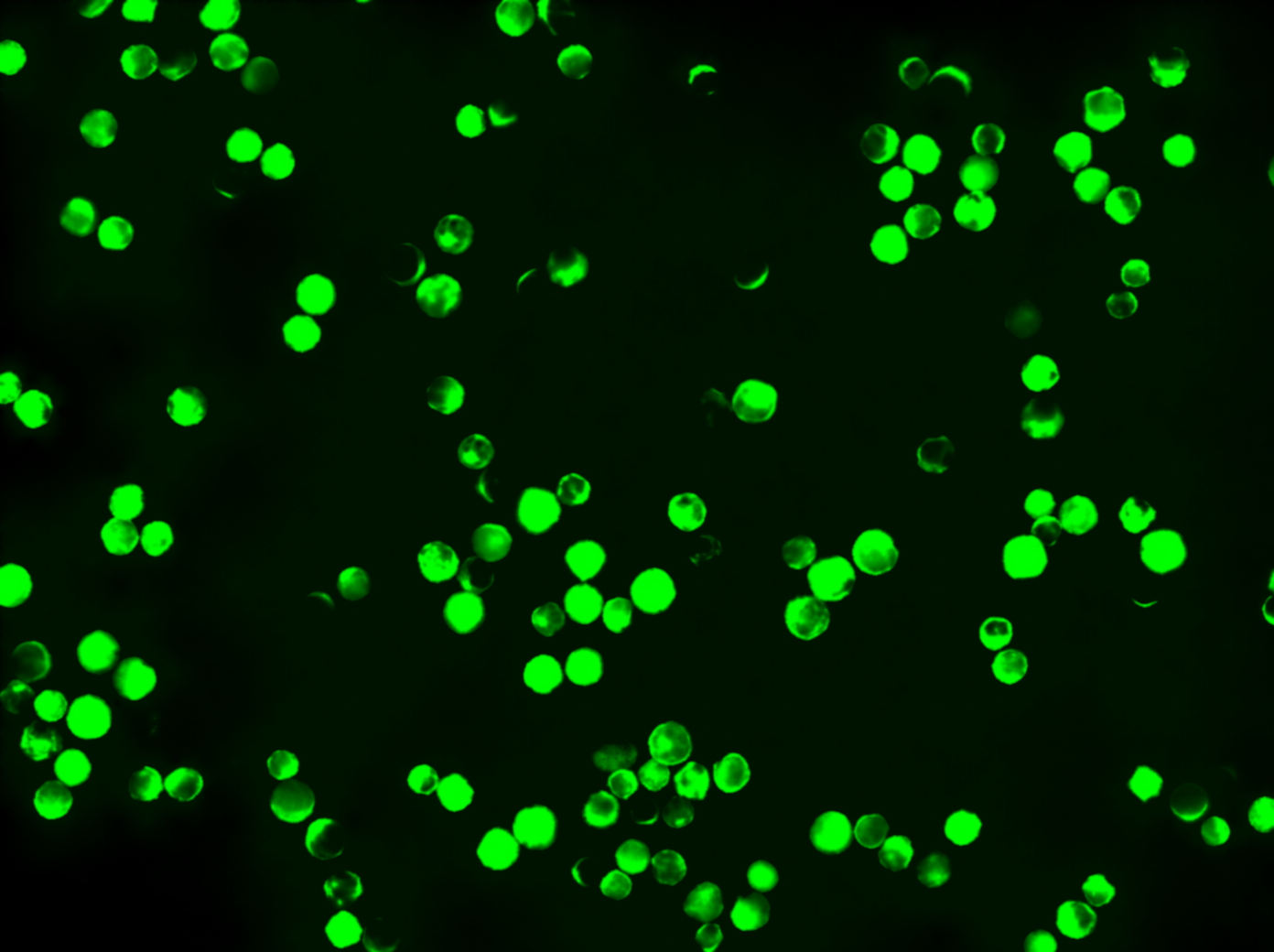

Supplement: Additional file 5 — The zip archive contains simulated images showing protoplasts with corresponding ground truth. (ZIP 72704 kb) [file 12859_2017_1591_MOESM5_ESM.zip › simulated protoplasts/realisticoverlap/realisticoverlap001.png]

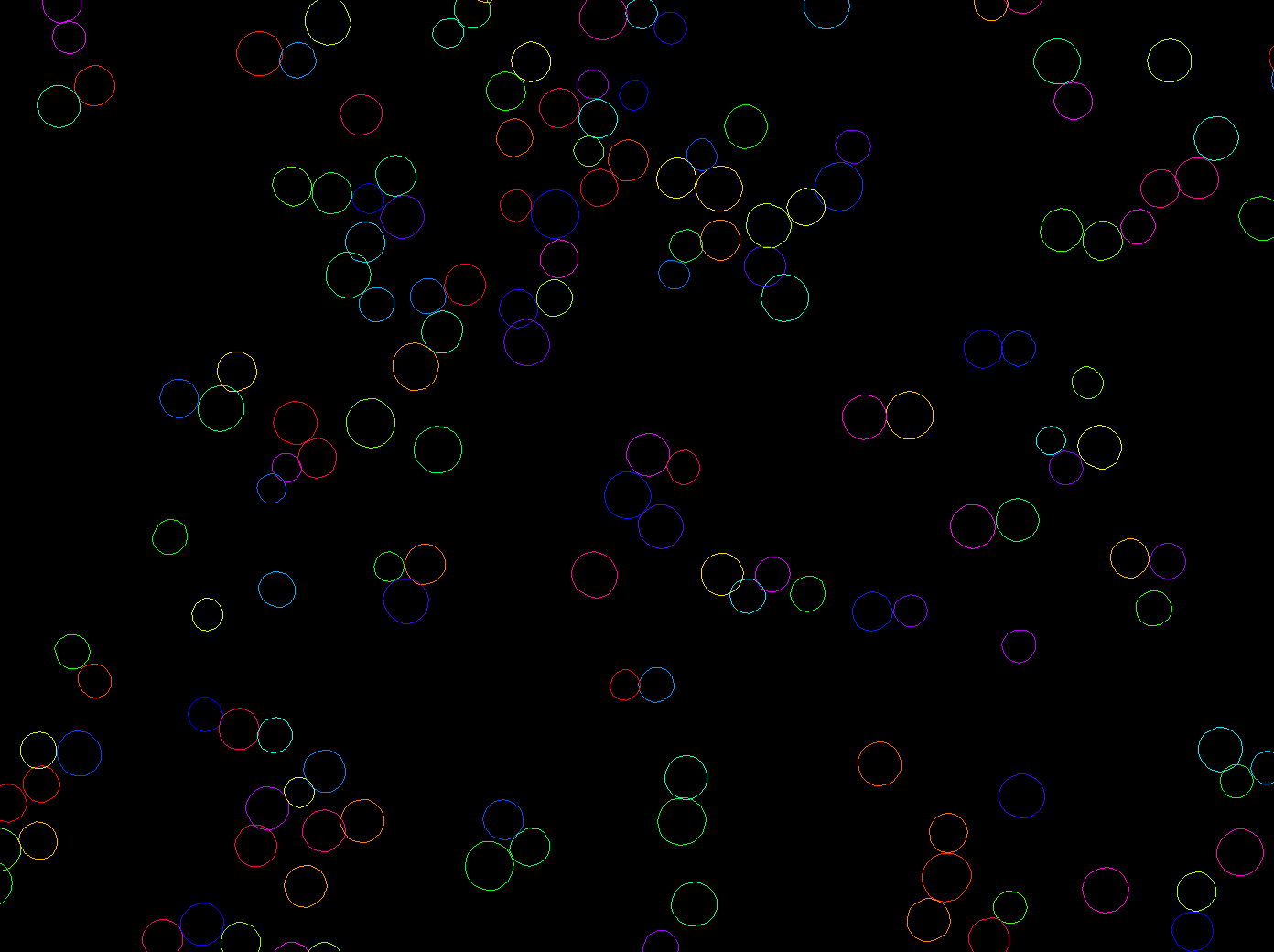

Supplement: Additional file 5 — The zip archive contains simulated images showing protoplasts with corresponding ground truth. (ZIP 72704 kb) [file 12859_2017_1591_MOESM5_ESM.zip › simulated protoplasts/realisticoverlap/realisticoverlap002 gt.png]

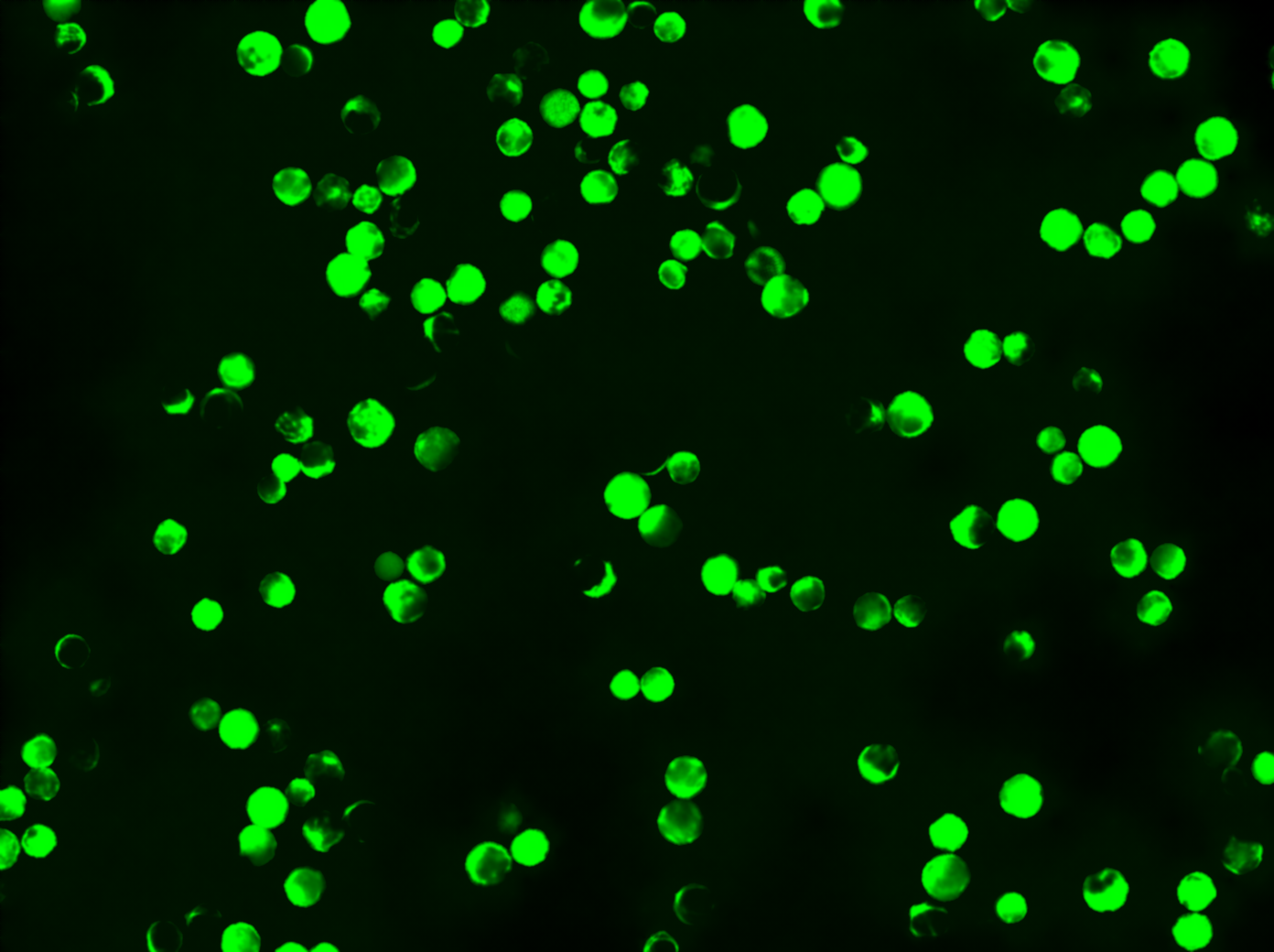

Supplement: Additional file 5 — The zip archive contains simulated images showing protoplasts with corresponding ground truth. (ZIP 72704 kb) [file 12859_2017_1591_MOESM5_ESM.zip › simulated protoplasts/realisticoverlap/realisticoverlap002.png]

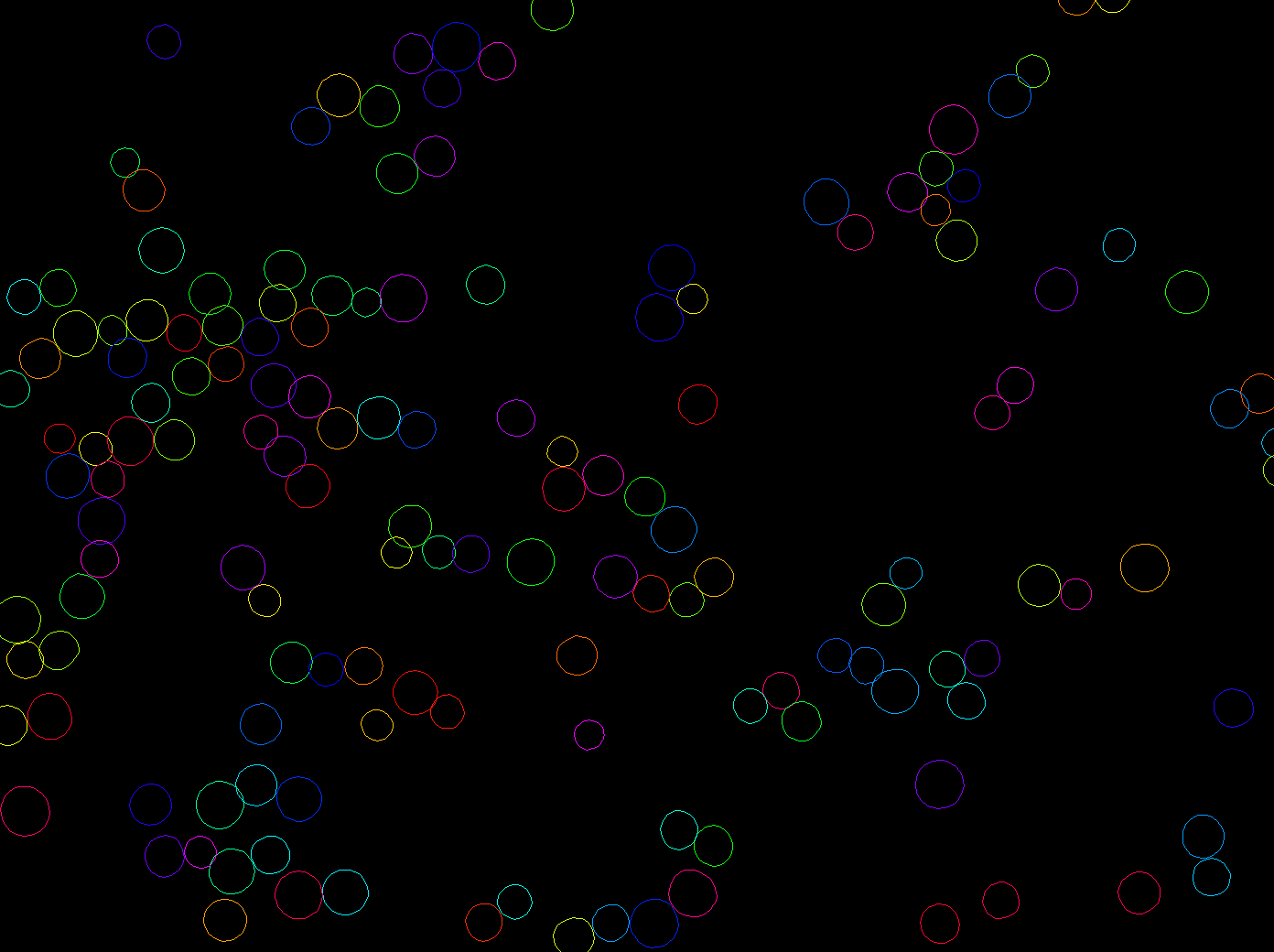

Supplement: Additional file 5 — The zip archive contains simulated images showing protoplasts with corresponding ground truth. (ZIP 72704 kb) [file 12859_2017_1591_MOESM5_ESM.zip › simulated protoplasts/realisticoverlap/realisticoverlap003 gt.png]

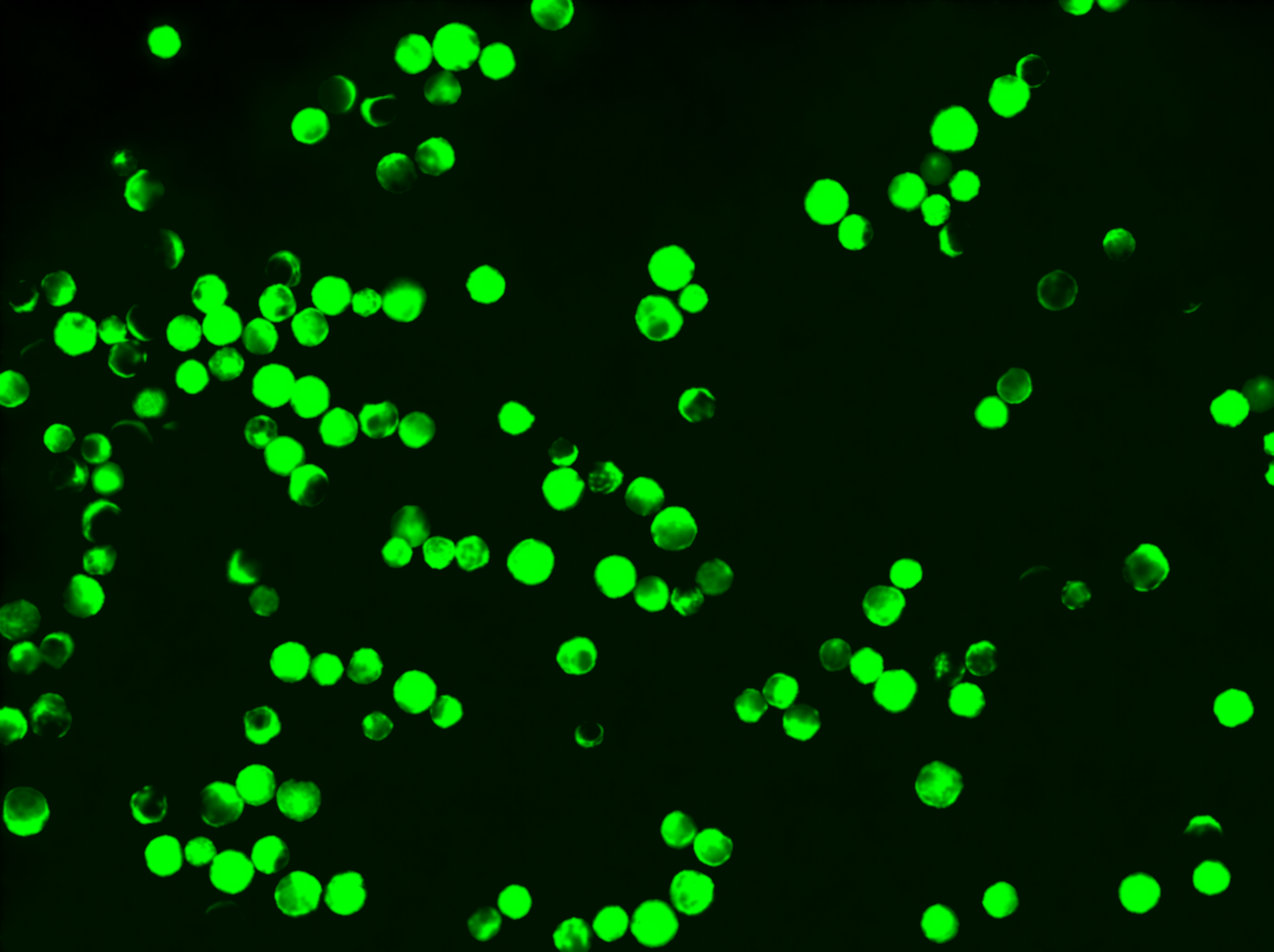

Supplement: Additional file 5 — The zip archive contains simulated images showing protoplasts with corresponding ground truth. (ZIP 72704 kb) [file 12859_2017_1591_MOESM5_ESM.zip › simulated protoplasts/realisticoverlap/realisticoverlap003.png]

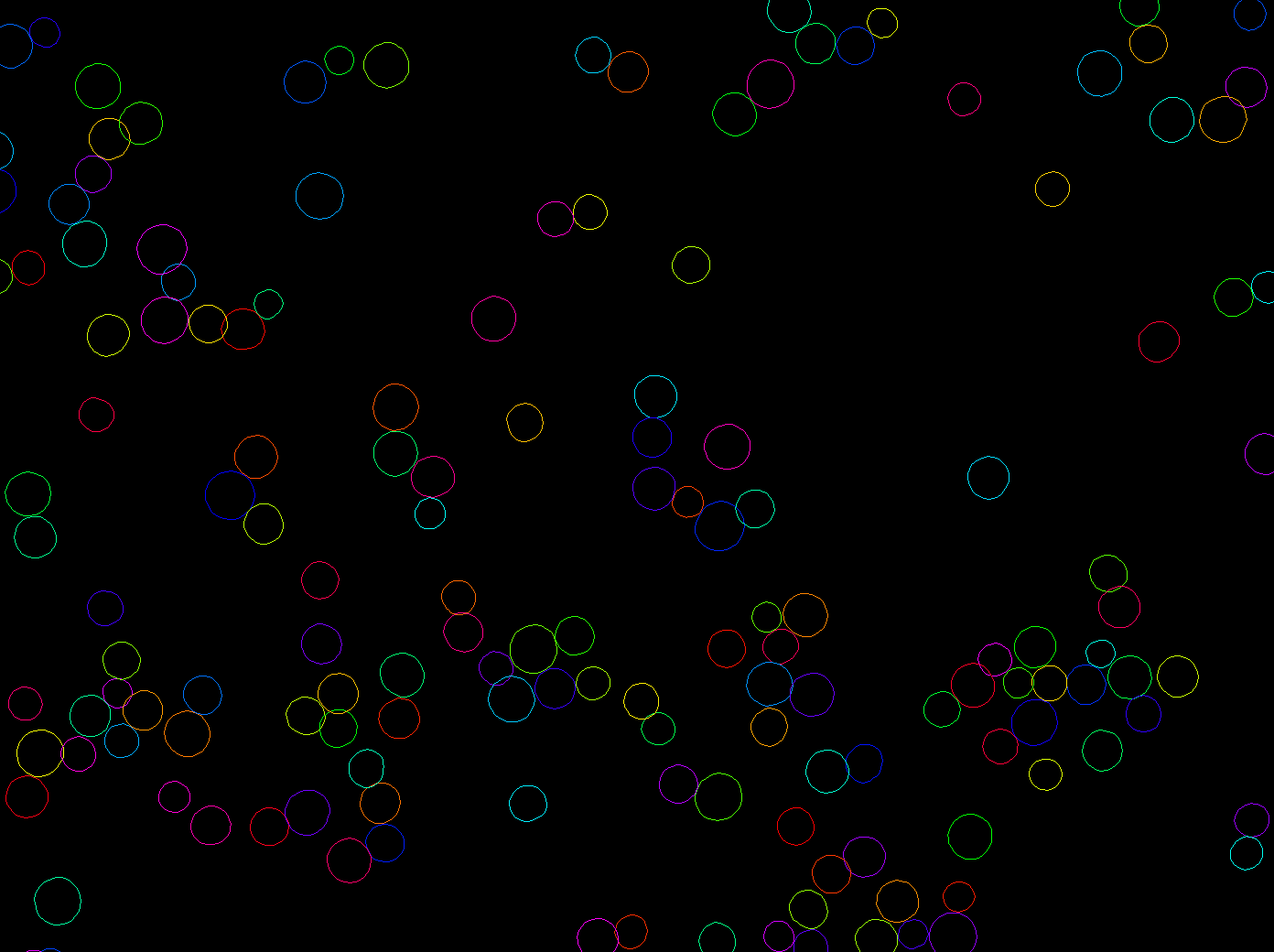

Supplement: Additional file 5 — The zip archive contains simulated images showing protoplasts with corresponding ground truth. (ZIP 72704 kb) [file 12859_2017_1591_MOESM5_ESM.zip › simulated protoplasts/realisticoverlap/realisticoverlap004 gt.png]

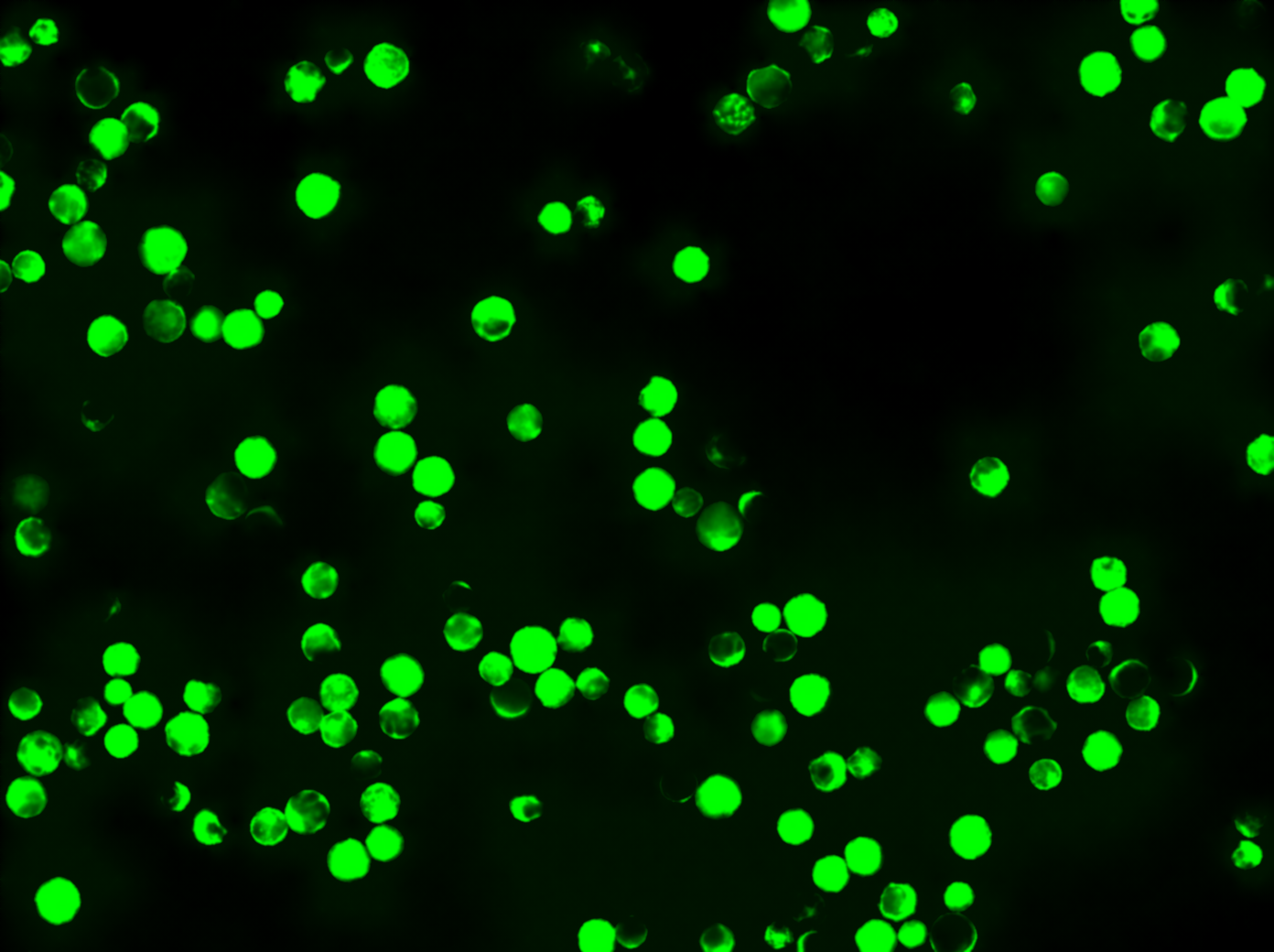

Supplement: Additional file 5 — The zip archive contains simulated images showing protoplasts with corresponding ground truth. (ZIP 72704 kb) [file 12859_2017_1591_MOESM5_ESM.zip › simulated protoplasts/realisticoverlap/realisticoverlap004.png]

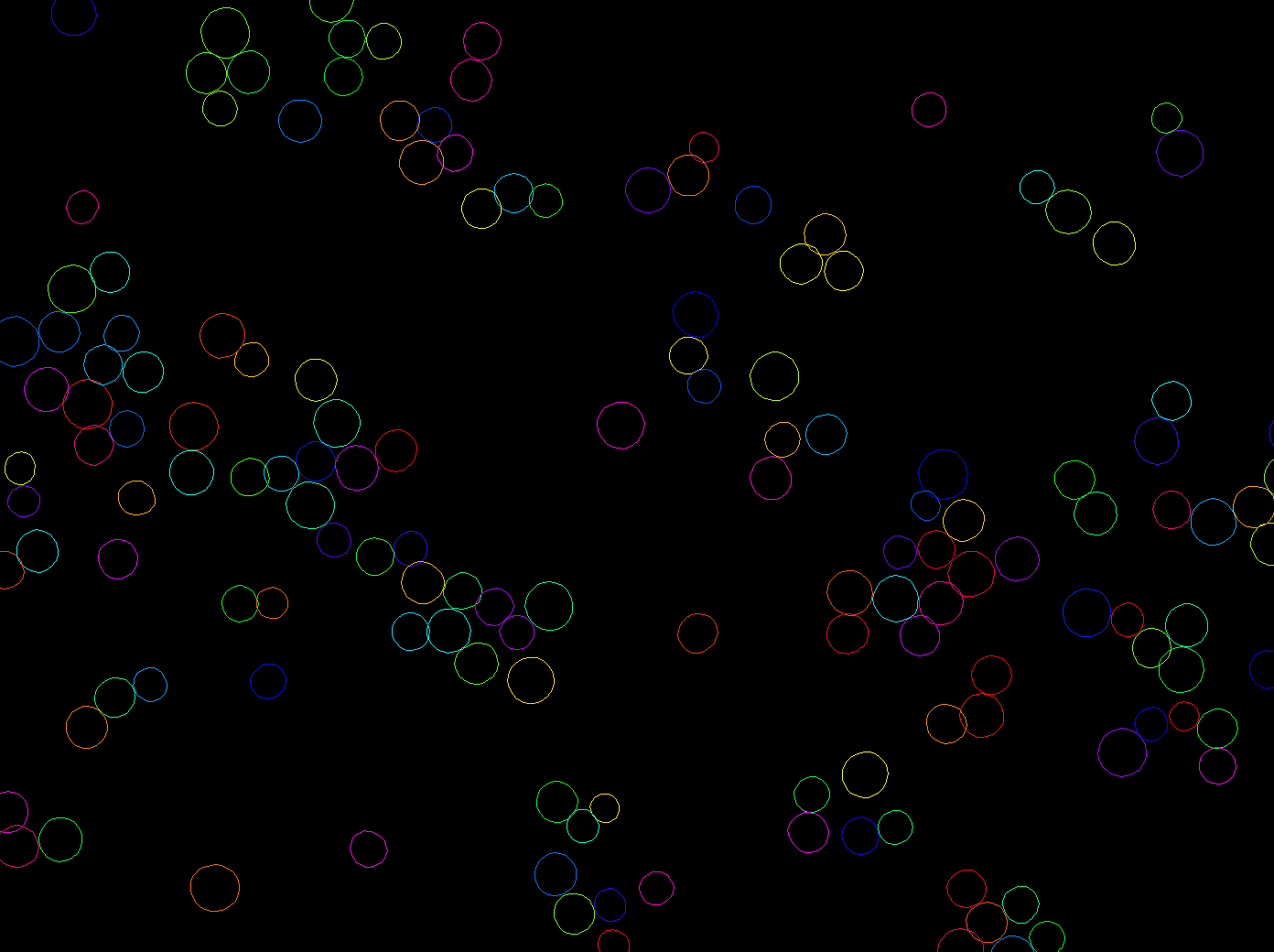

Supplement: Additional file 5 — The zip archive contains simulated images showing protoplasts with corresponding ground truth. (ZIP 72704 kb) [file 12859_2017_1591_MOESM5_ESM.zip › simulated protoplasts/realisticoverlap/realisticoverlap005 gt.png]

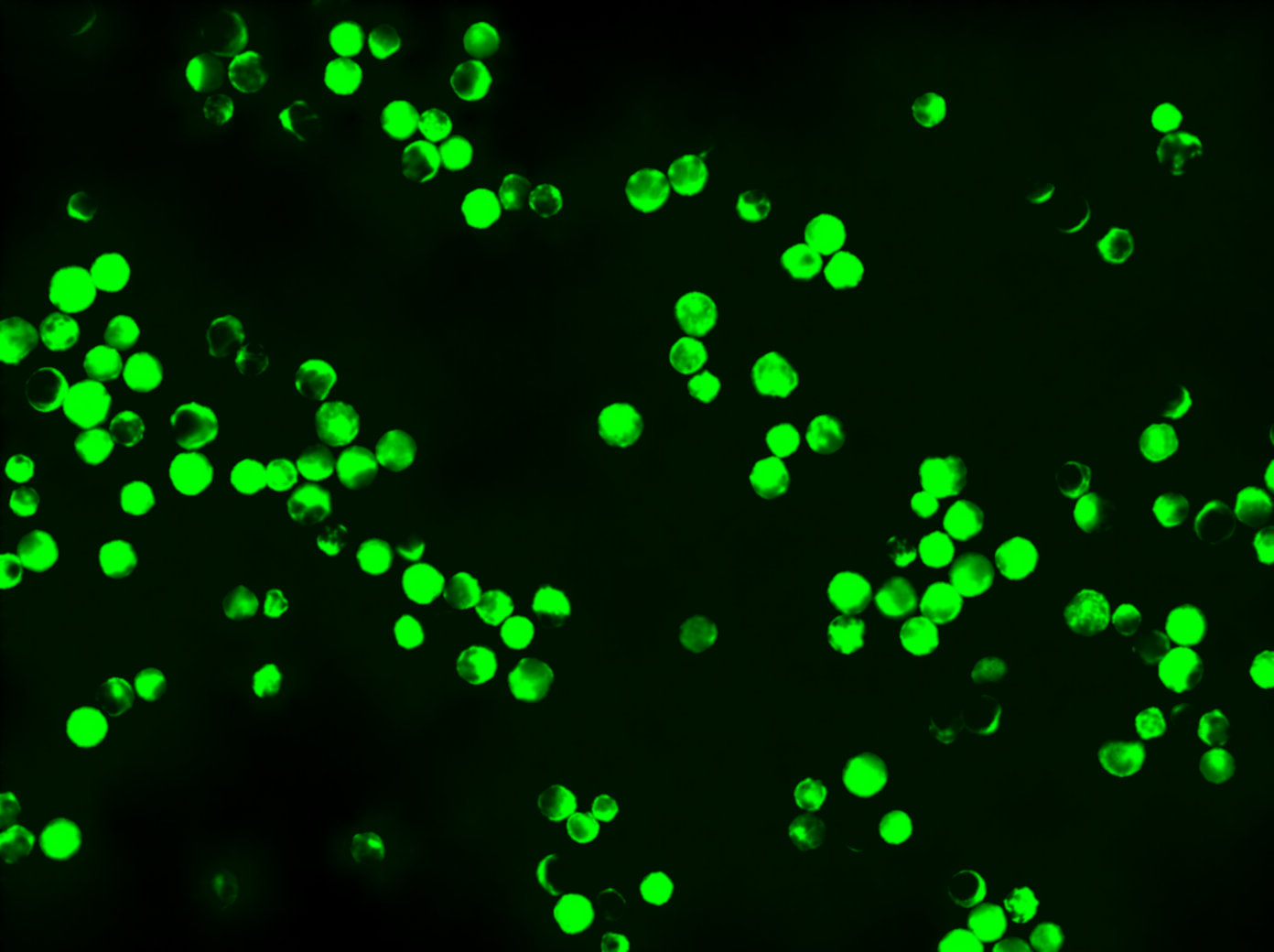

Supplement: Additional file 5 — The zip archive contains simulated images showing protoplasts with corresponding ground truth. (ZIP 72704 kb) [file 12859_2017_1591_MOESM5_ESM.zip › simulated protoplasts/realisticoverlap/realisticoverlap005.png]

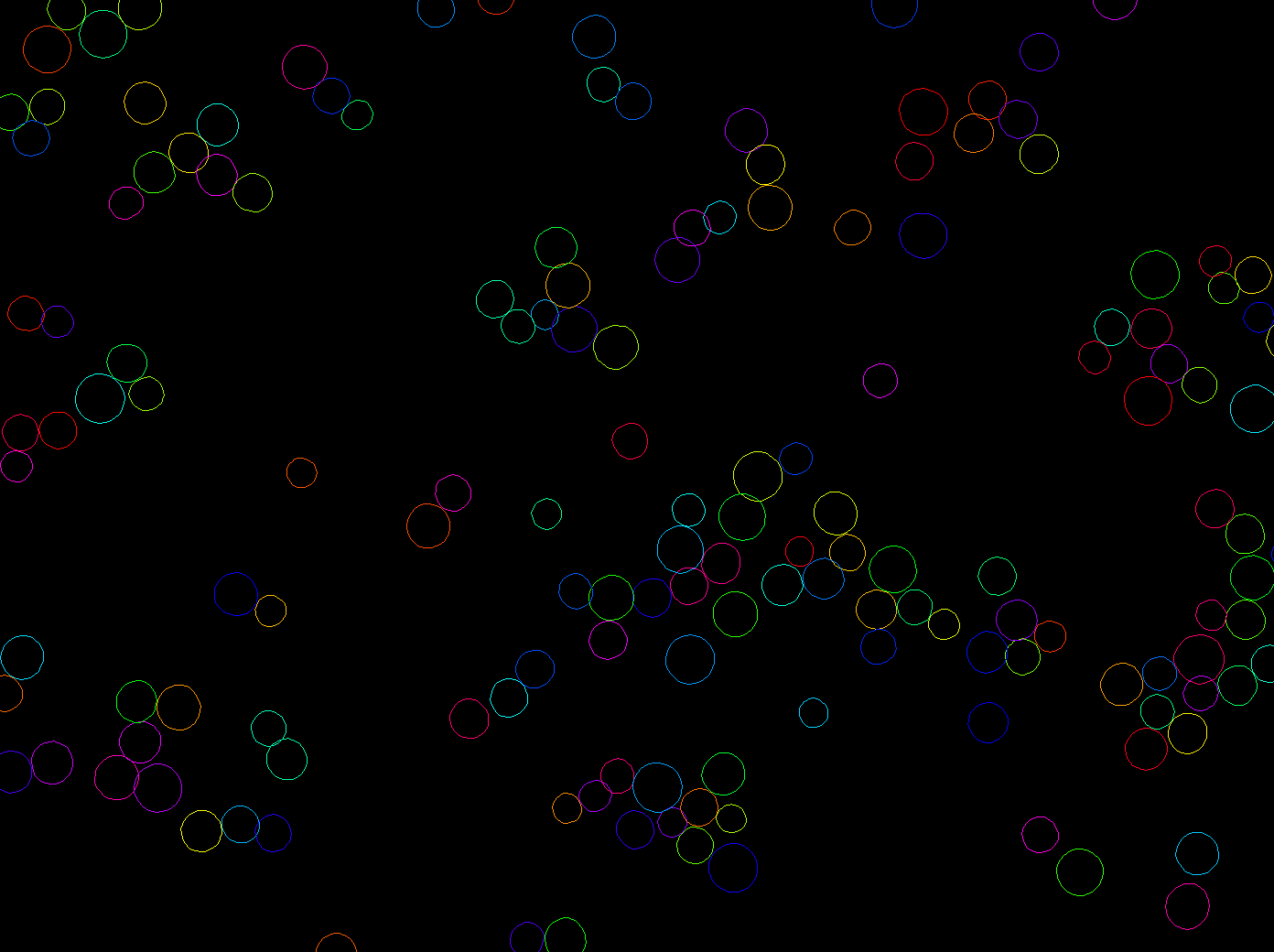

Supplement: Additional file 5 — The zip archive contains simulated images showing protoplasts with corresponding ground truth. (ZIP 72704 kb) [file 12859_2017_1591_MOESM5_ESM.zip › simulated protoplasts/realisticoverlap/realisticoverlap006 gt.png]

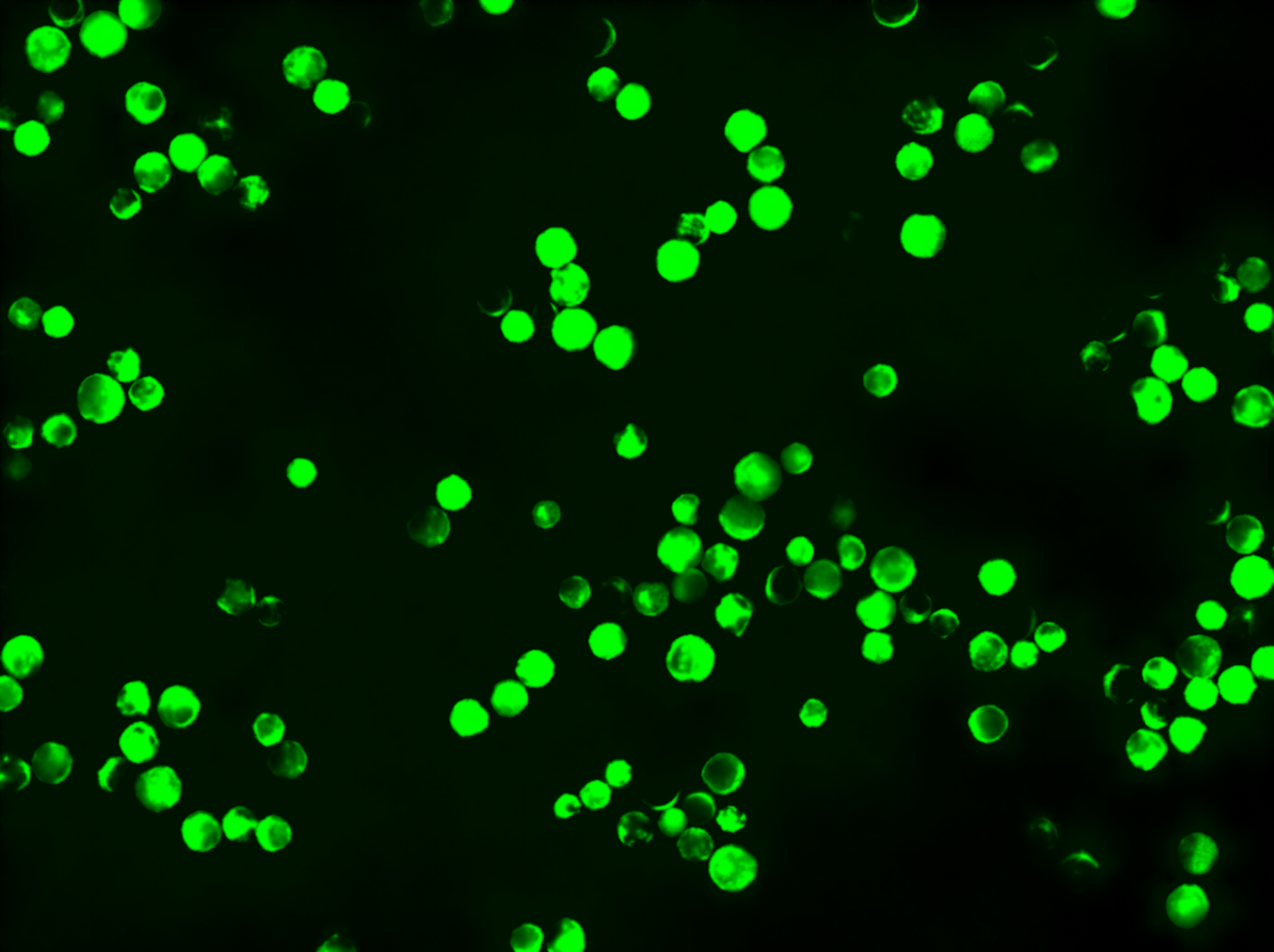

Supplement: Additional file 5 — The zip archive contains simulated images showing protoplasts with corresponding ground truth. (ZIP 72704 kb) [file 12859_2017_1591_MOESM5_ESM.zip › simulated protoplasts/realisticoverlap/realisticoverlap006.png]

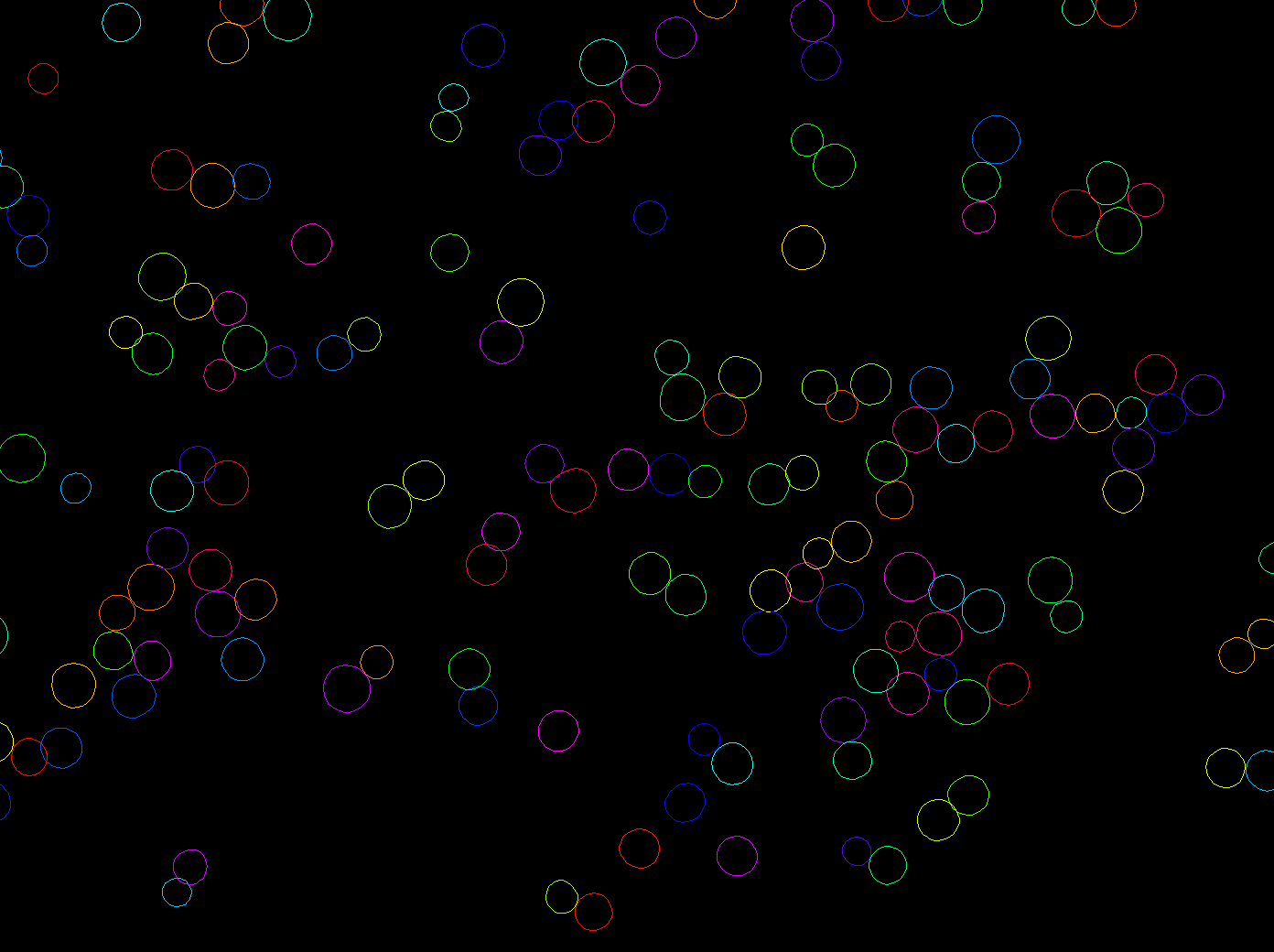

Supplement: Additional file 5 — The zip archive contains simulated images showing protoplasts with corresponding ground truth. (ZIP 72704 kb) [file 12859_2017_1591_MOESM5_ESM.zip › simulated protoplasts/realisticoverlap/realisticoverlap007 gt.png]

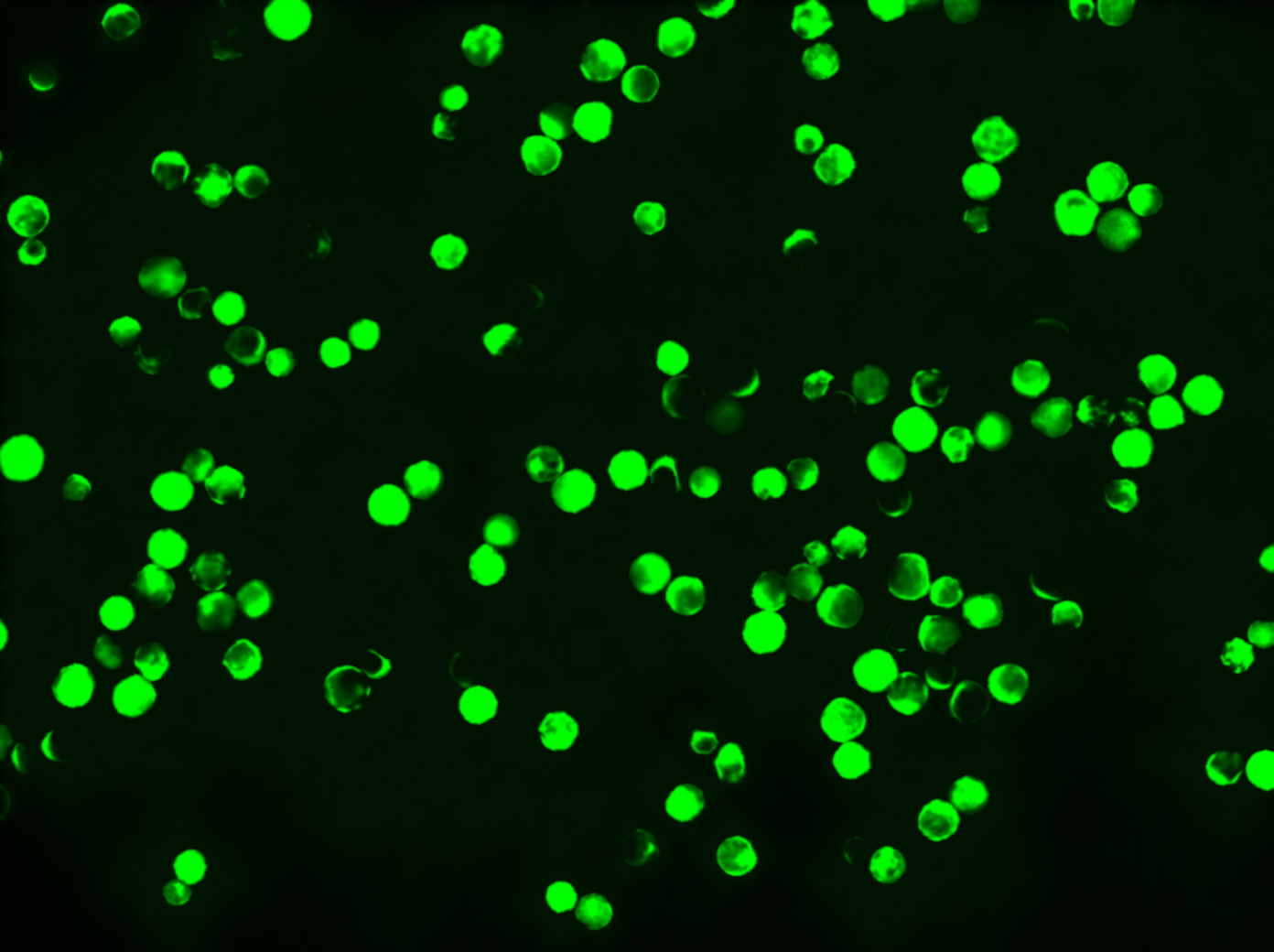

Supplement: Additional file 5 — The zip archive contains simulated images showing protoplasts with corresponding ground truth. (ZIP 72704 kb) [file 12859_2017_1591_MOESM5_ESM.zip › simulated protoplasts/realisticoverlap/realisticoverlap007.png]

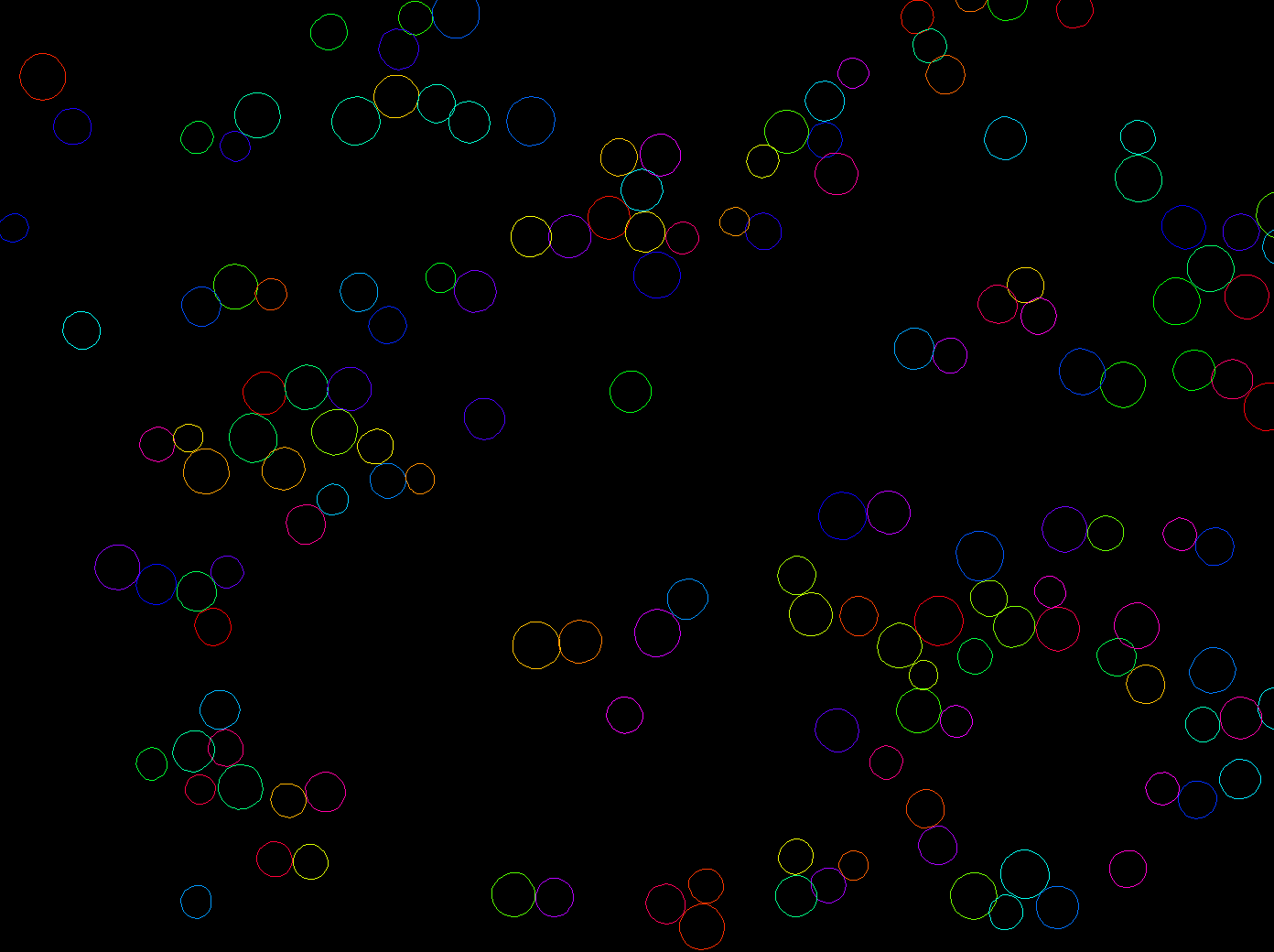

Supplement: Additional file 5 — The zip archive contains simulated images showing protoplasts with corresponding ground truth. (ZIP 72704 kb) [file 12859_2017_1591_MOESM5_ESM.zip › simulated protoplasts/realisticoverlap/realisticoverlap008 gt.png]

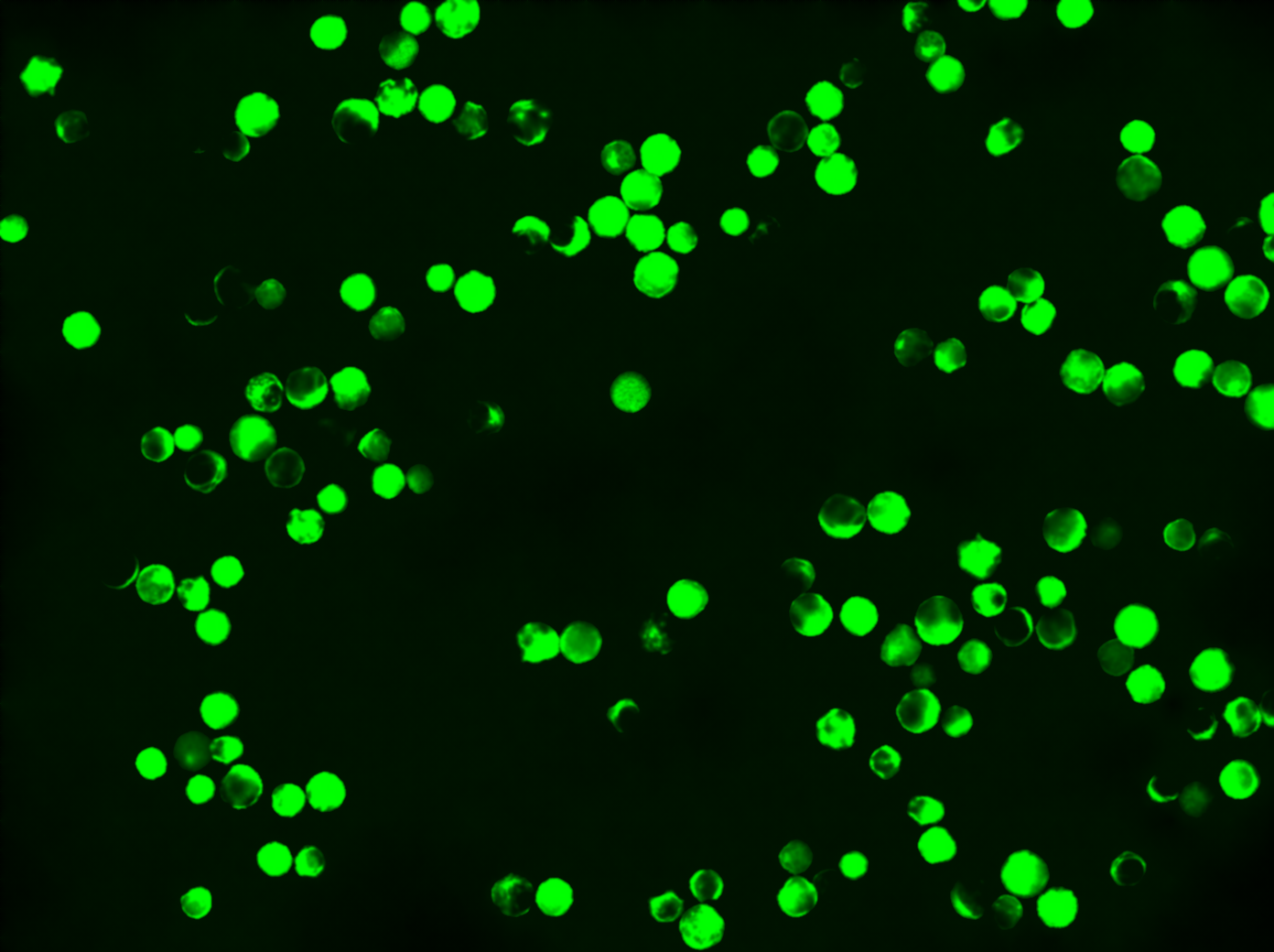

Supplement: Additional file 5 — The zip archive contains simulated images showing protoplasts with corresponding ground truth. (ZIP 72704 kb) [file 12859_2017_1591_MOESM5_ESM.zip › simulated protoplasts/realisticoverlap/realisticoverlap008.png]

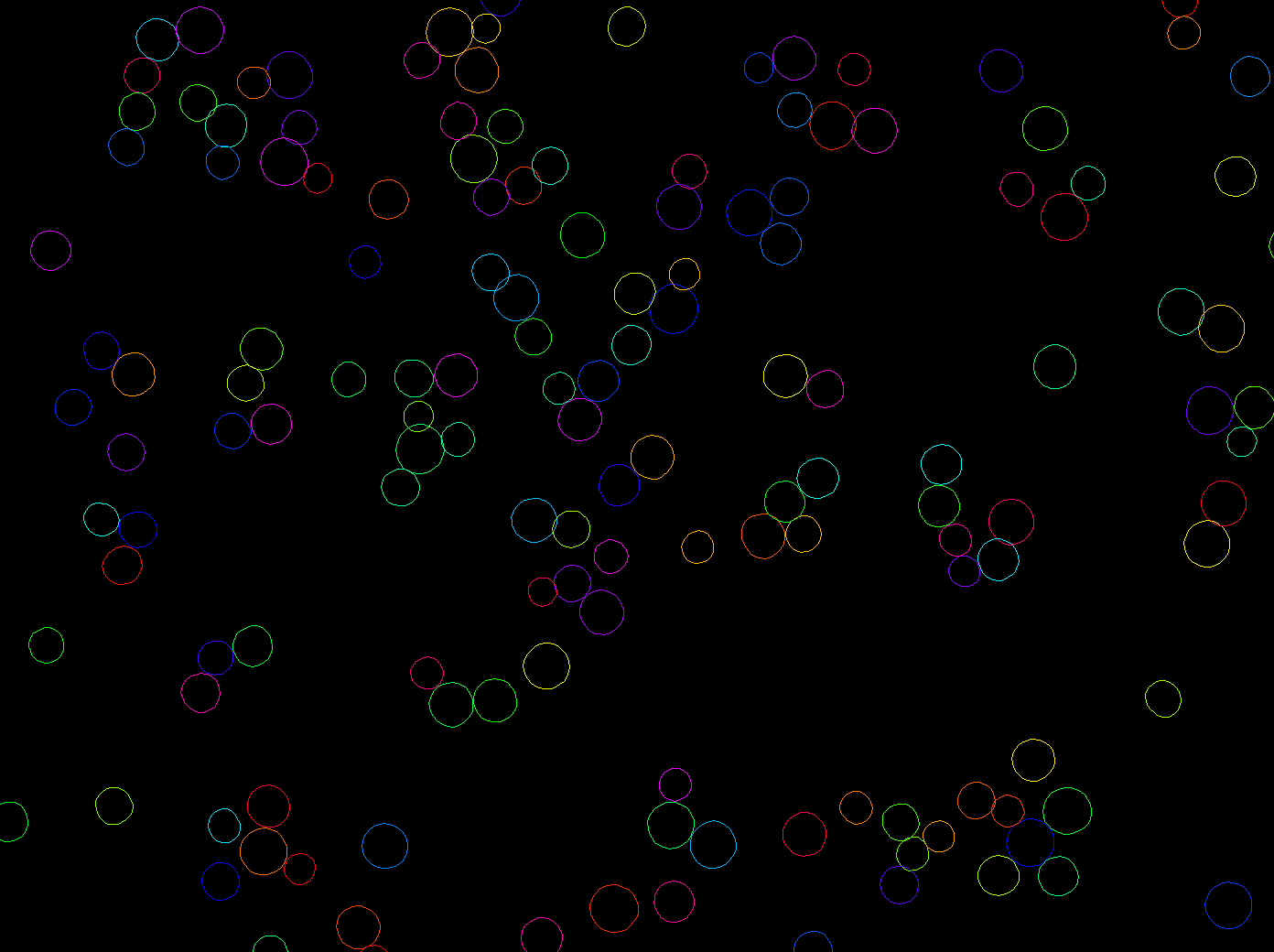

Supplement: Additional file 5 — The zip archive contains simulated images showing protoplasts with corresponding ground truth. (ZIP 72704 kb) [file 12859_2017_1591_MOESM5_ESM.zip › simulated protoplasts/realisticoverlap/realisticoverlap009 gt.png]

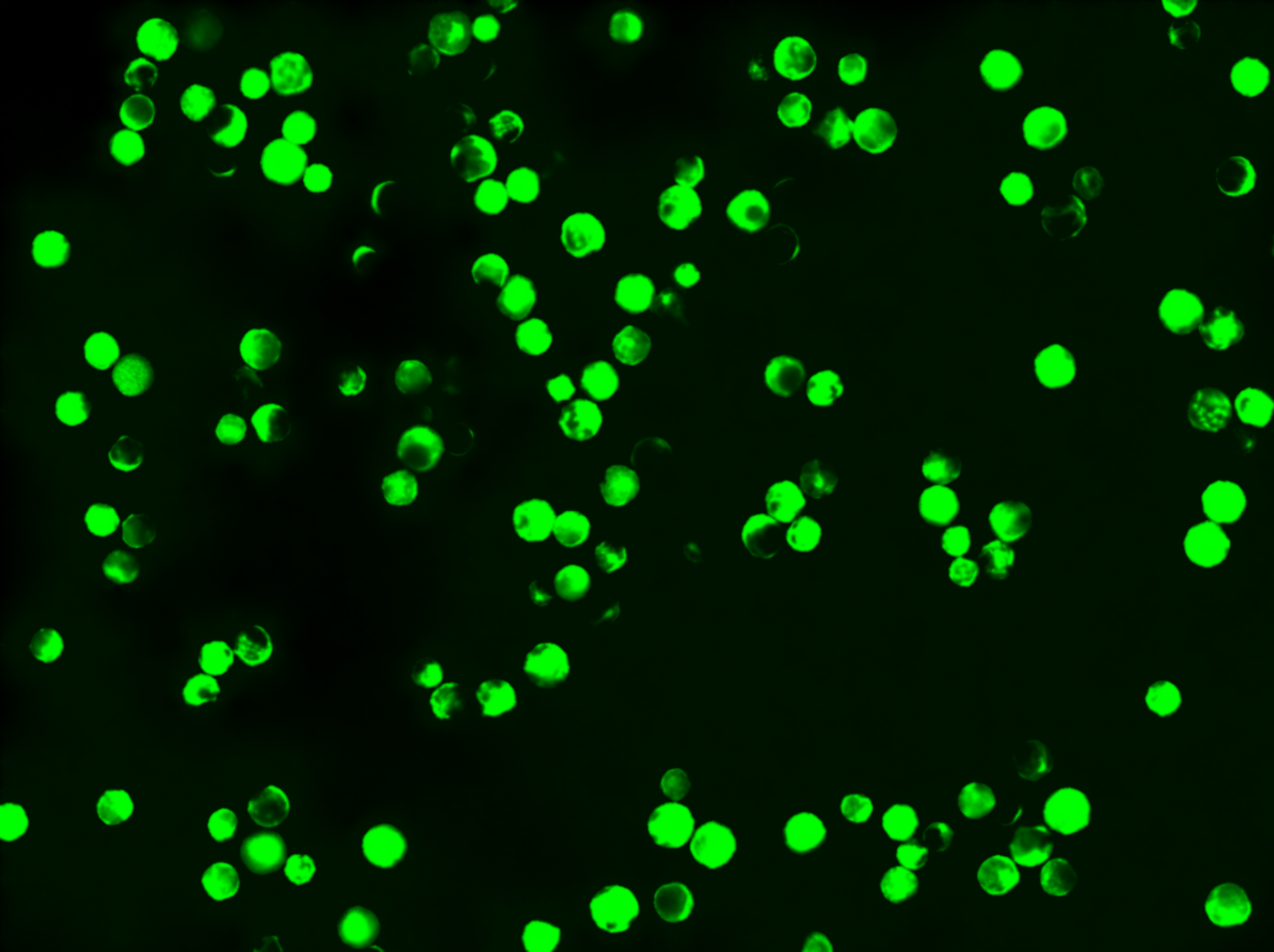

Supplement: Additional file 5 — The zip archive contains simulated images showing protoplasts with corresponding ground truth. (ZIP 72704 kb) [file 12859_2017_1591_MOESM5_ESM.zip › simulated protoplasts/realisticoverlap/realisticoverlap009.png]

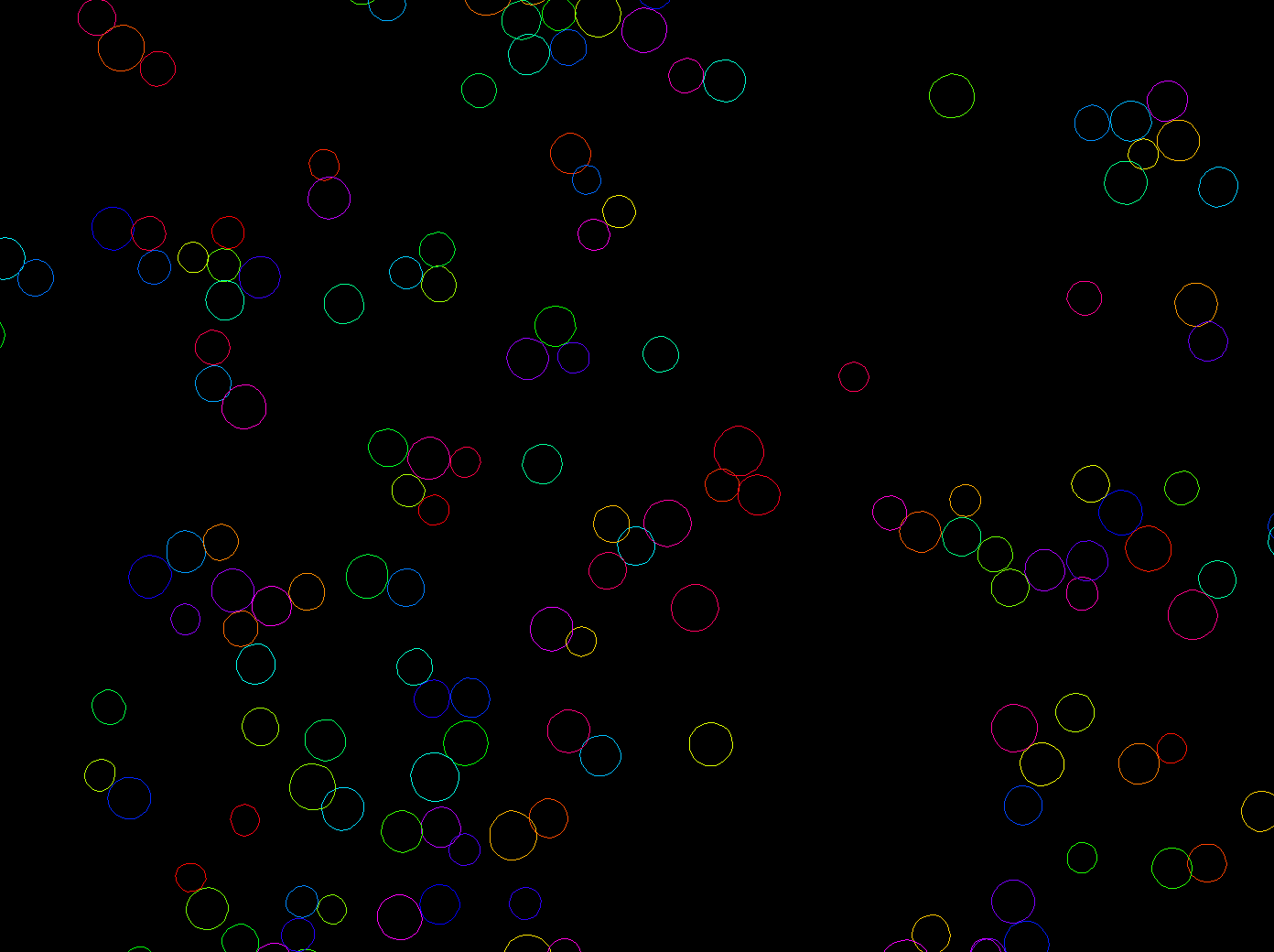

Supplement: Additional file 5 — The zip archive contains simulated images showing protoplasts with corresponding ground truth. (ZIP 72704 kb) [file 12859_2017_1591_MOESM5_ESM.zip › simulated protoplasts/realisticoverlap/realisticoverlap010 gt.png]

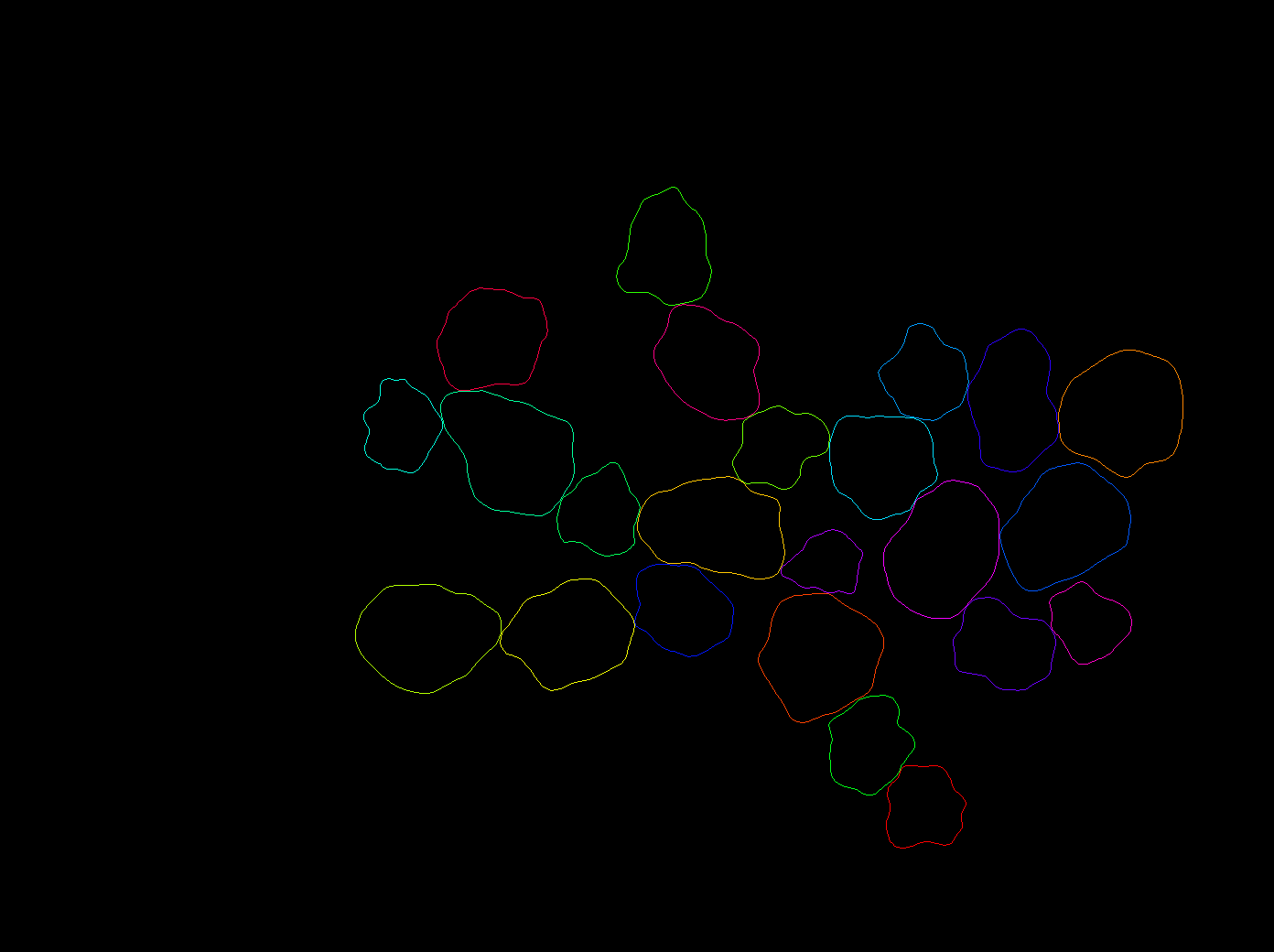

Supplement: Additional file 6 — The zip archive contains simulated images showing B cell nuclei and cytoskeleton with corresponding ground truth. (ZIP 119808 kb) [file 12859_2017_1591_MOESM6_ESM.zip › simulated B cells/cytoskeleton/not touching/cell001 gt.png]

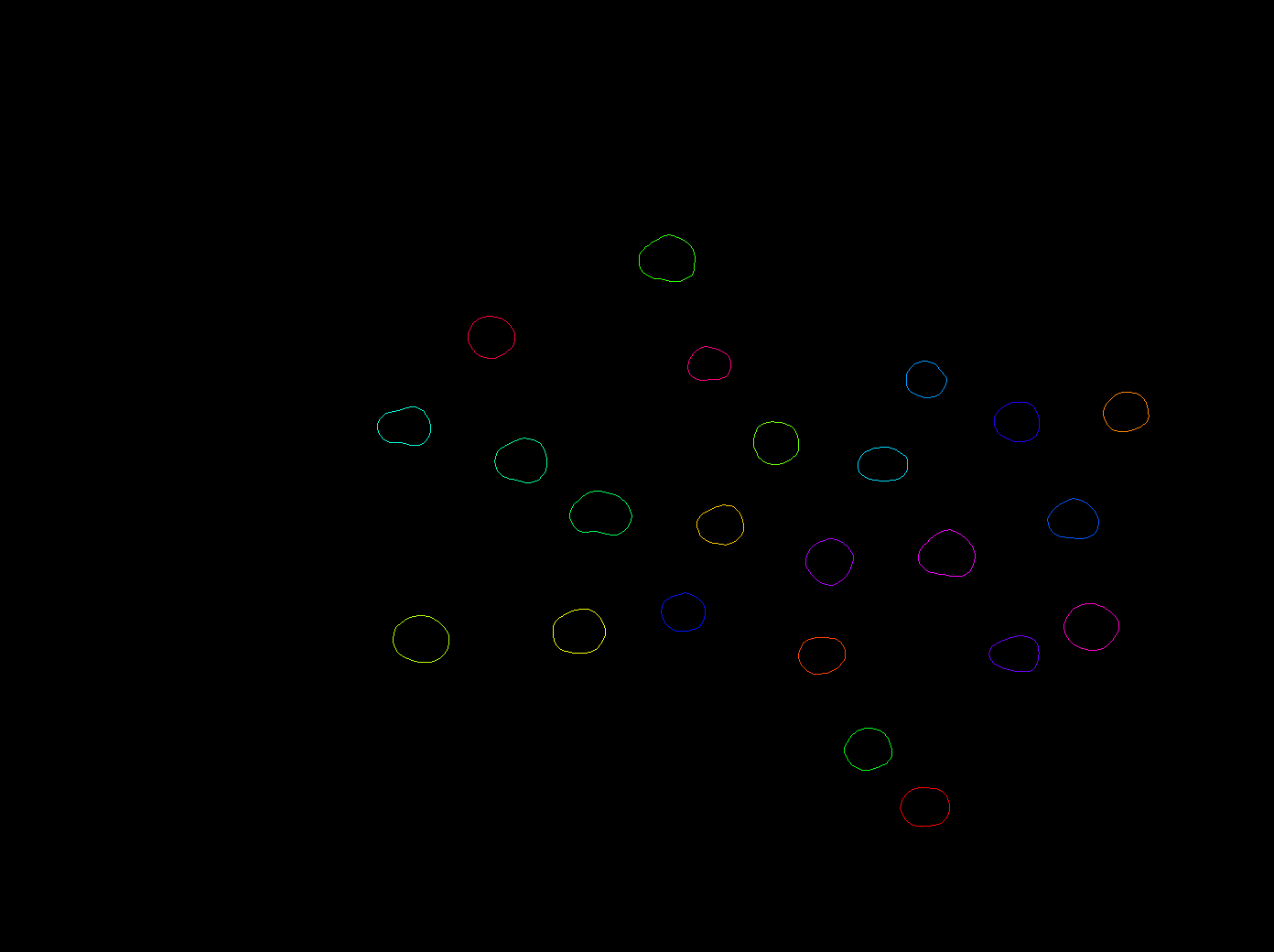

Supplement: Additional file 6 — The zip archive contains simulated images showing B cell nuclei and cytoskeleton with corresponding ground truth. (ZIP 119808 kb) [file 12859_2017_1591_MOESM6_ESM.zip › simulated B cells/cytoskeleton/not touching/cell001 seeds.png]

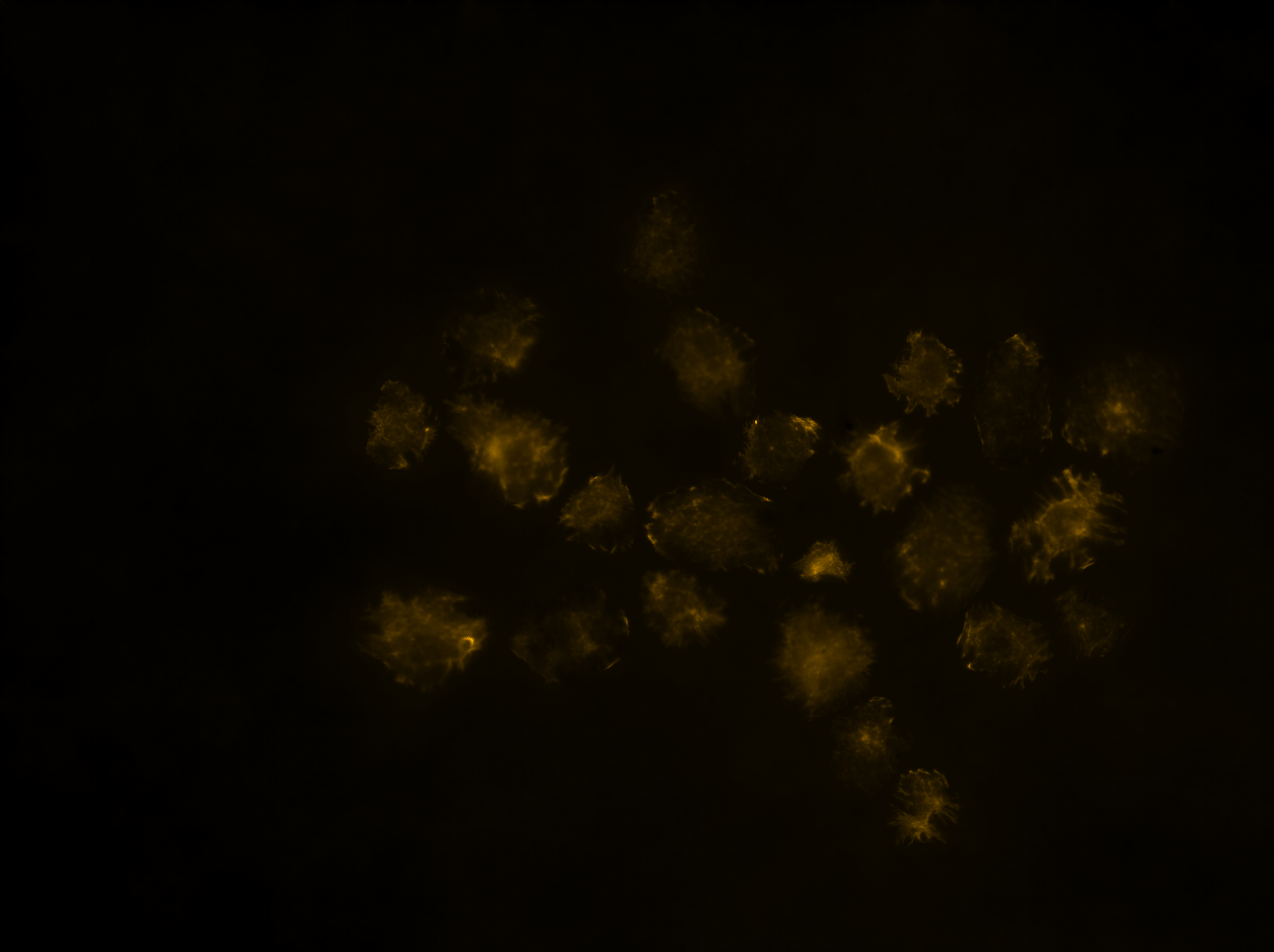

Supplement: Additional file 6 — The zip archive contains simulated images showing B cell nuclei and cytoskeleton with corresponding ground truth. (ZIP 119808 kb) [file 12859_2017_1591_MOESM6_ESM.zip › simulated B cells/cytoskeleton/not touching/cell001.png]

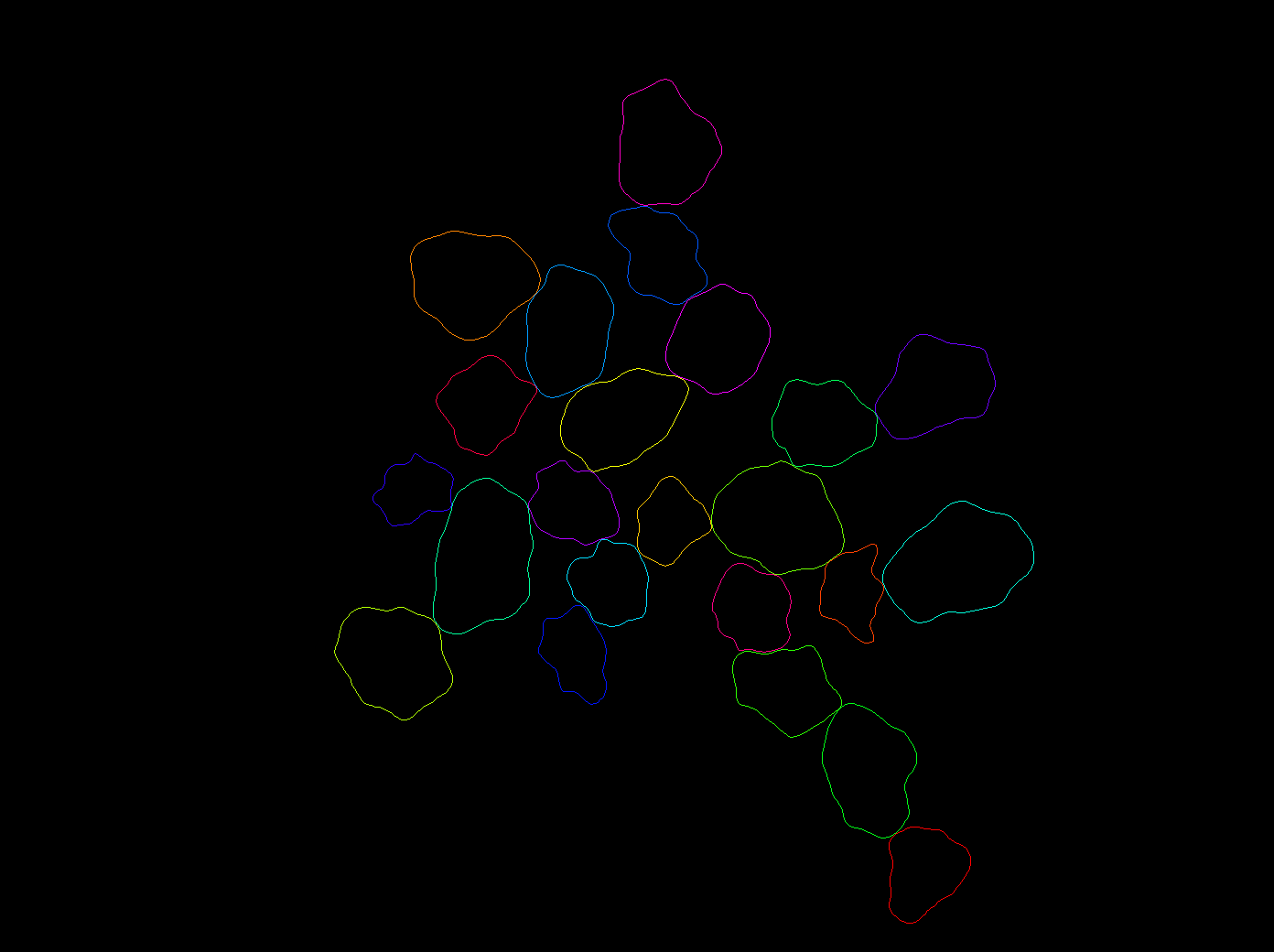

Supplement: Additional file 6 — The zip archive contains simulated images showing B cell nuclei and cytoskeleton with corresponding ground truth. (ZIP 119808 kb) [file 12859_2017_1591_MOESM6_ESM.zip › simulated B cells/cytoskeleton/not touching/cell002 gt.png]

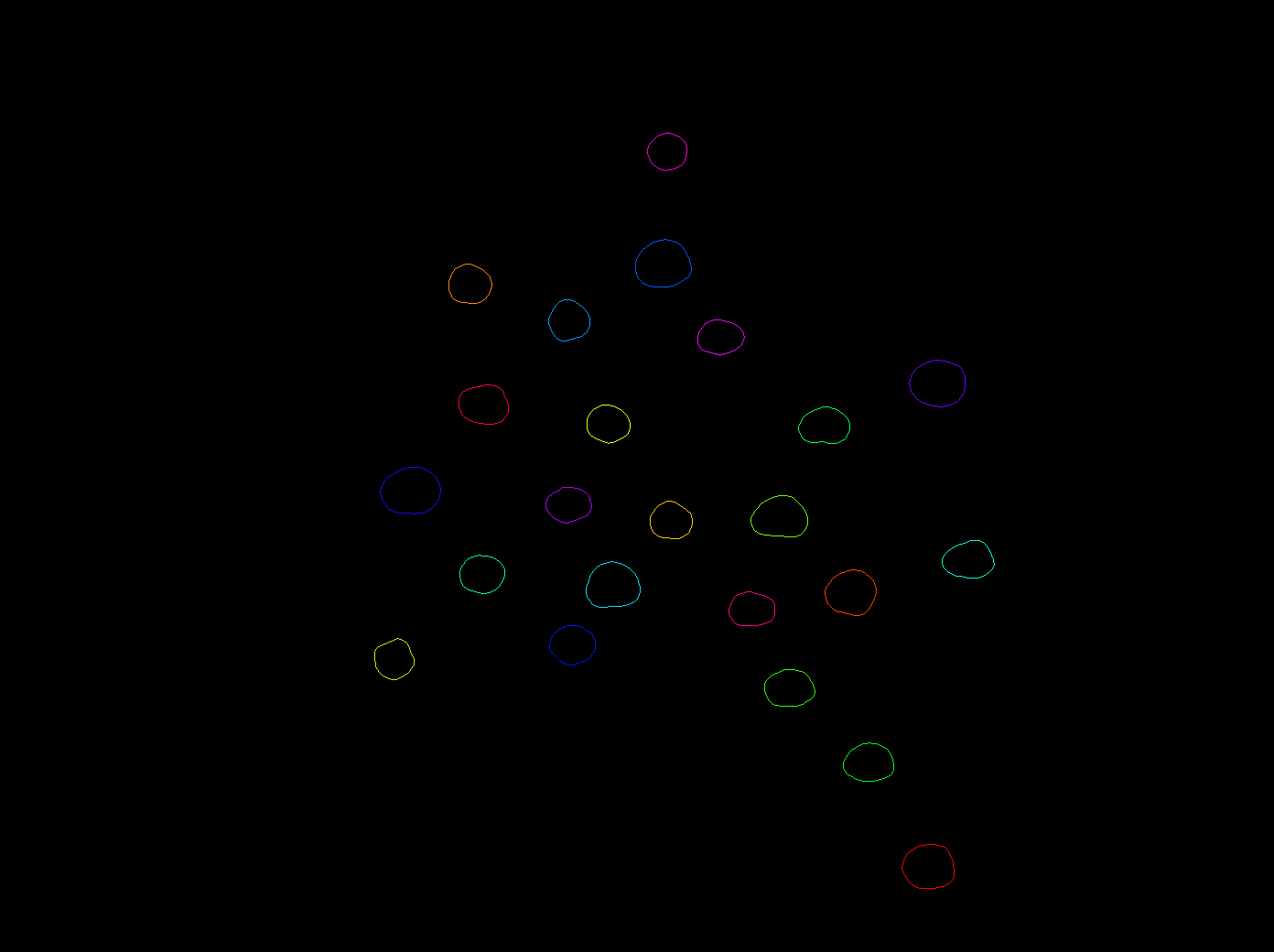

Supplement: Additional file 6 — The zip archive contains simulated images showing B cell nuclei and cytoskeleton with corresponding ground truth. (ZIP 119808 kb) [file 12859_2017_1591_MOESM6_ESM.zip › simulated B cells/cytoskeleton/not touching/cell002 seeds.png]

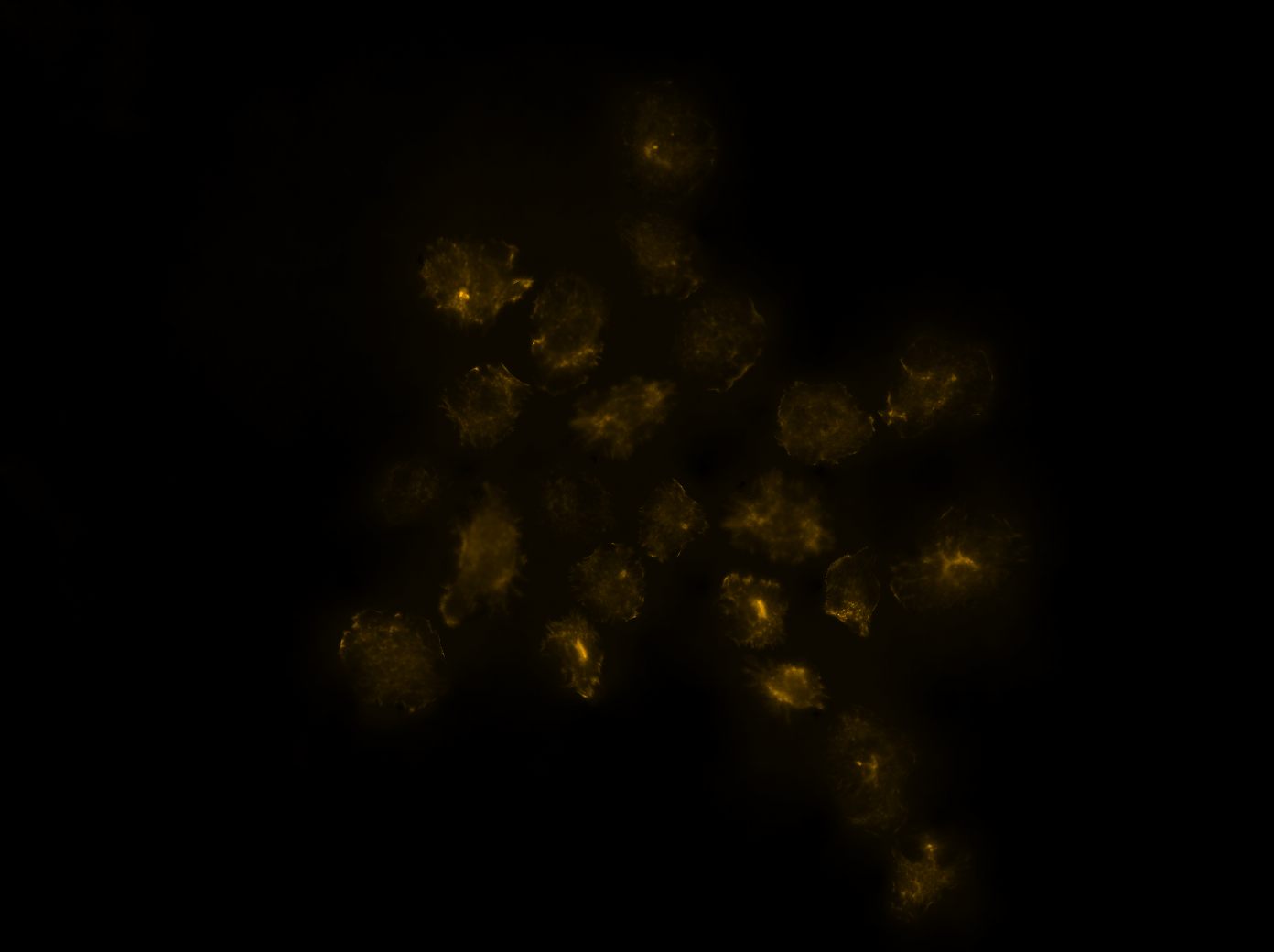

Supplement: Additional file 6 — The zip archive contains simulated images showing B cell nuclei and cytoskeleton with corresponding ground truth. (ZIP 119808 kb) [file 12859_2017_1591_MOESM6_ESM.zip › simulated B cells/cytoskeleton/not touching/cell002.png]

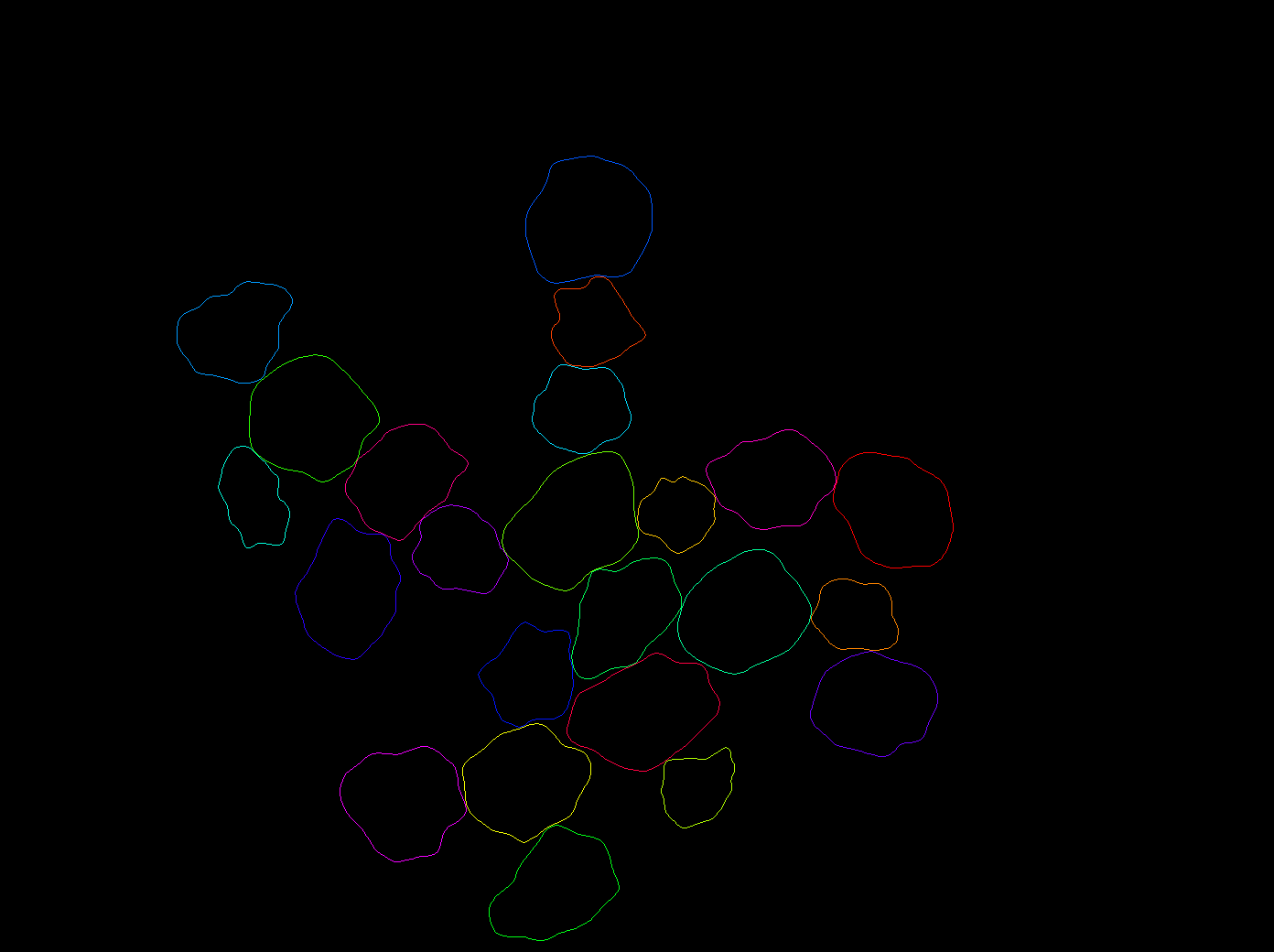

Supplement: Additional file 6 — The zip archive contains simulated images showing B cell nuclei and cytoskeleton with corresponding ground truth. (ZIP 119808 kb) [file 12859_2017_1591_MOESM6_ESM.zip › simulated B cells/cytoskeleton/not touching/cell003 gt.png]

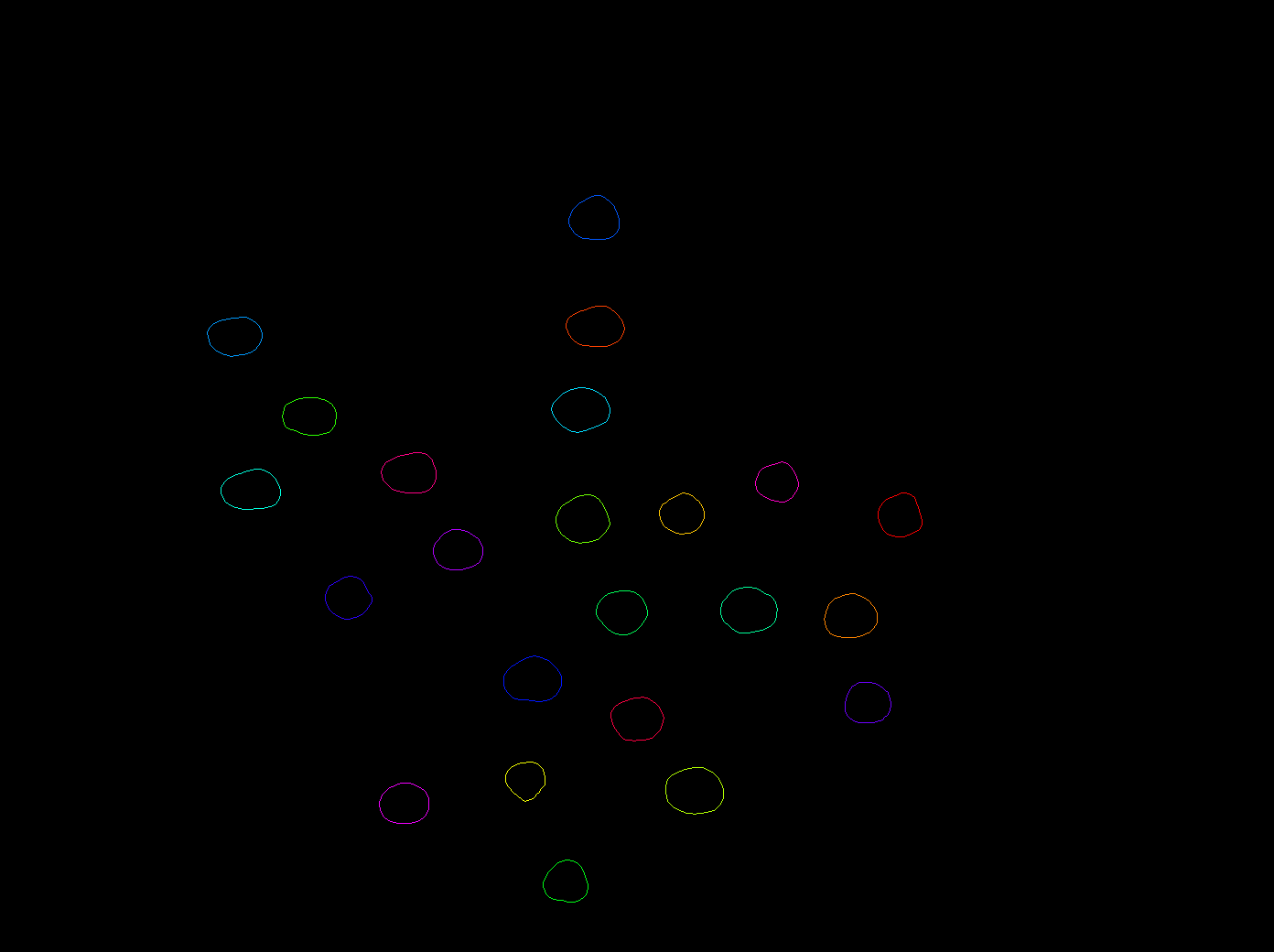

Supplement: Additional file 6 — The zip archive contains simulated images showing B cell nuclei and cytoskeleton with corresponding ground truth. (ZIP 119808 kb) [file 12859_2017_1591_MOESM6_ESM.zip › simulated B cells/cytoskeleton/not touching/cell003 seeds.png]

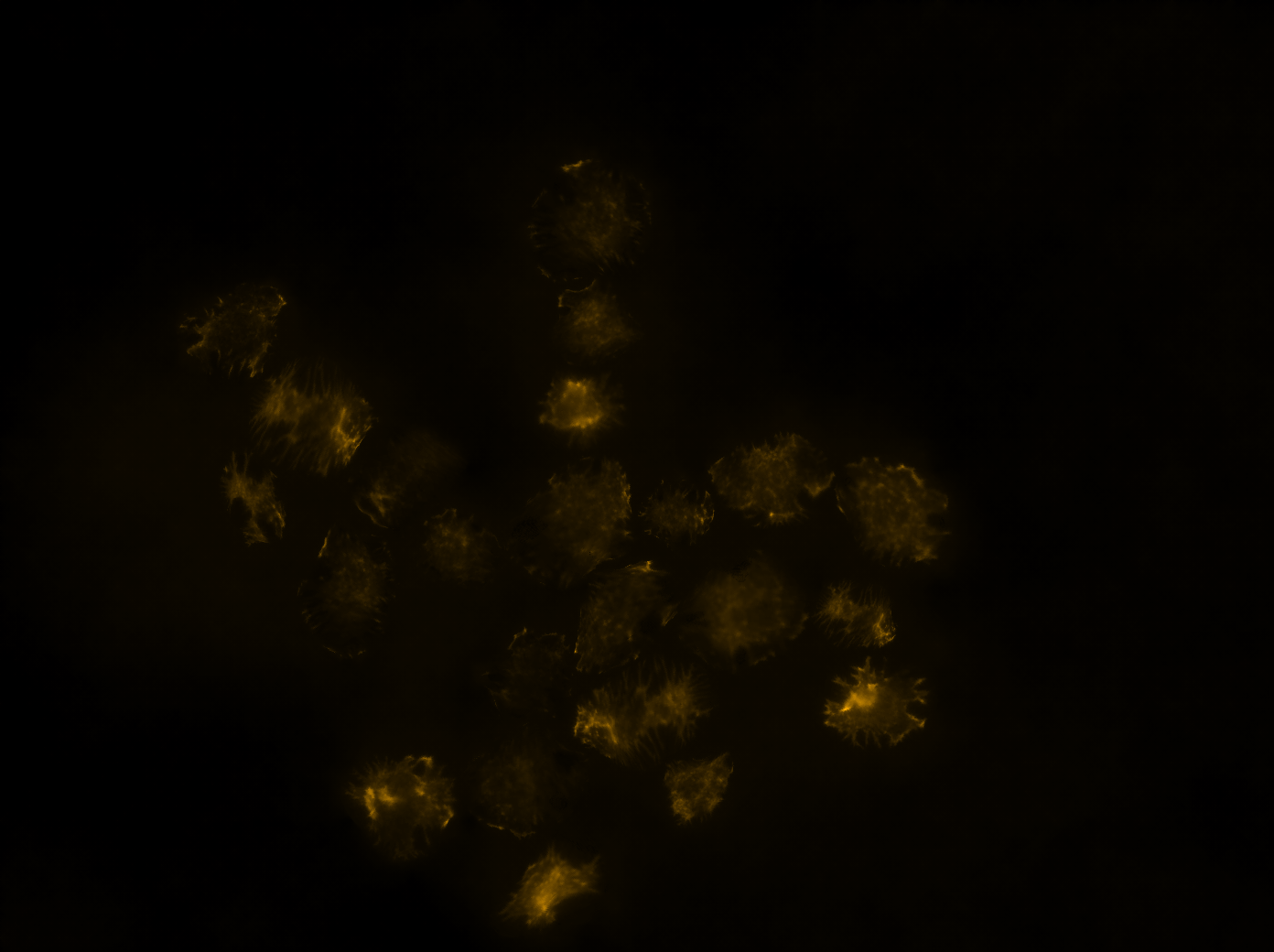

Supplement: Additional file 6 — The zip archive contains simulated images showing B cell nuclei and cytoskeleton with corresponding ground truth. (ZIP 119808 kb) [file 12859_2017_1591_MOESM6_ESM.zip › simulated B cells/cytoskeleton/not touching/cell003.png]

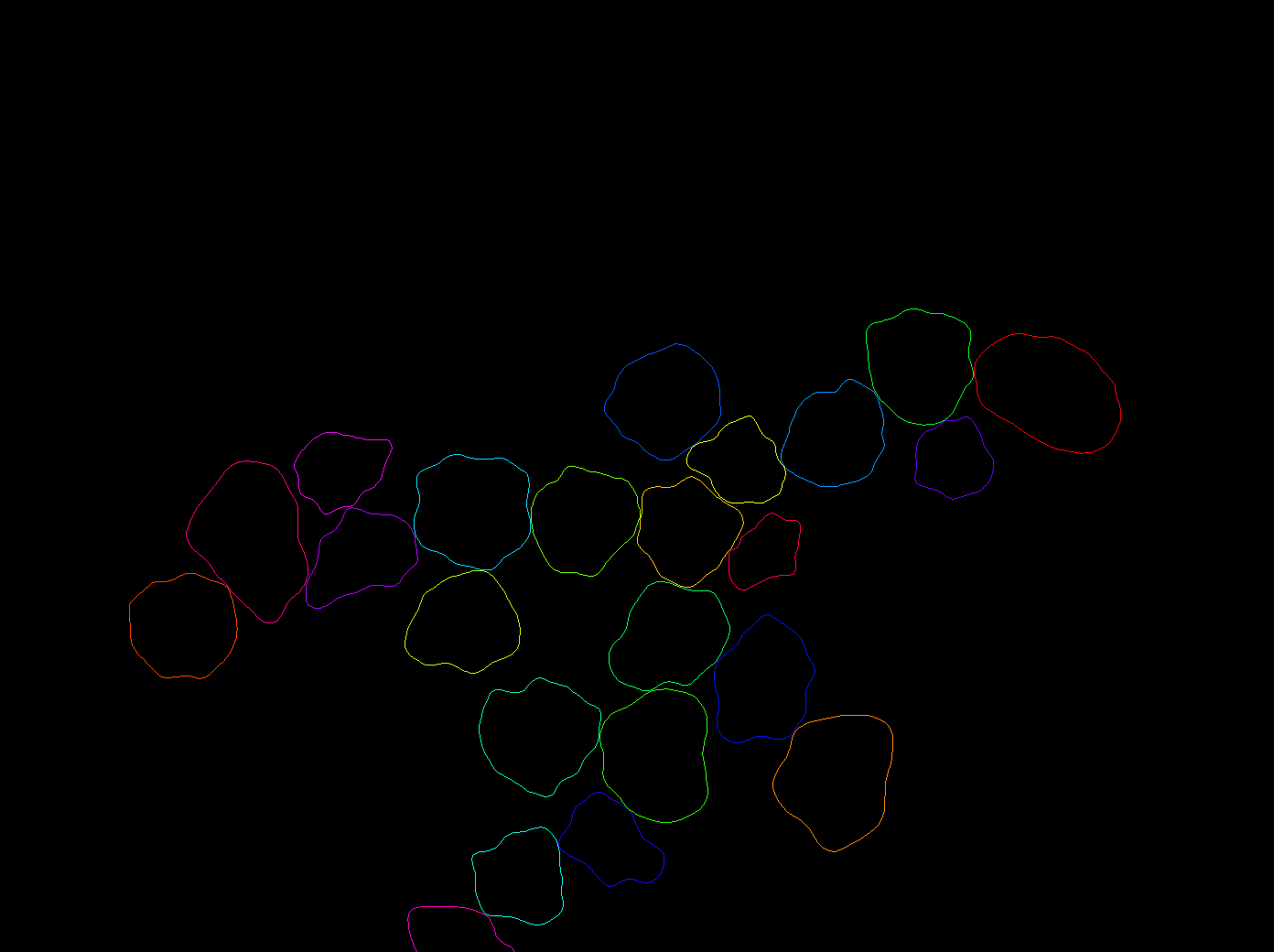

Supplement: Additional file 6 — The zip archive contains simulated images showing B cell nuclei and cytoskeleton with corresponding ground truth. (ZIP 119808 kb) [file 12859_2017_1591_MOESM6_ESM.zip › simulated B cells/cytoskeleton/not touching/cell004 gt.png]

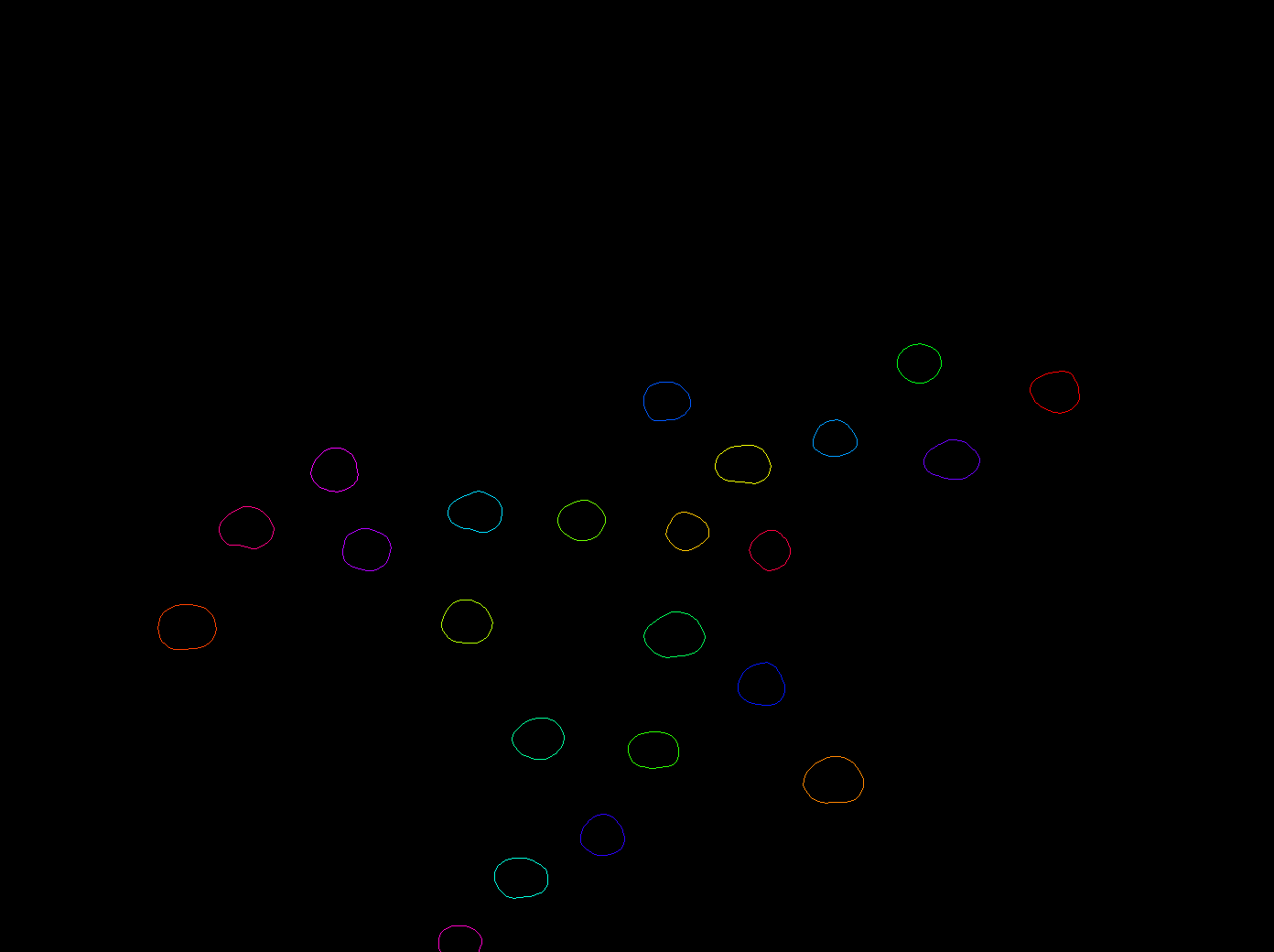

Supplement: Additional file 6 — The zip archive contains simulated images showing B cell nuclei and cytoskeleton with corresponding ground truth. (ZIP 119808 kb) [file 12859_2017_1591_MOESM6_ESM.zip › simulated B cells/cytoskeleton/not touching/cell004 seeds.png]

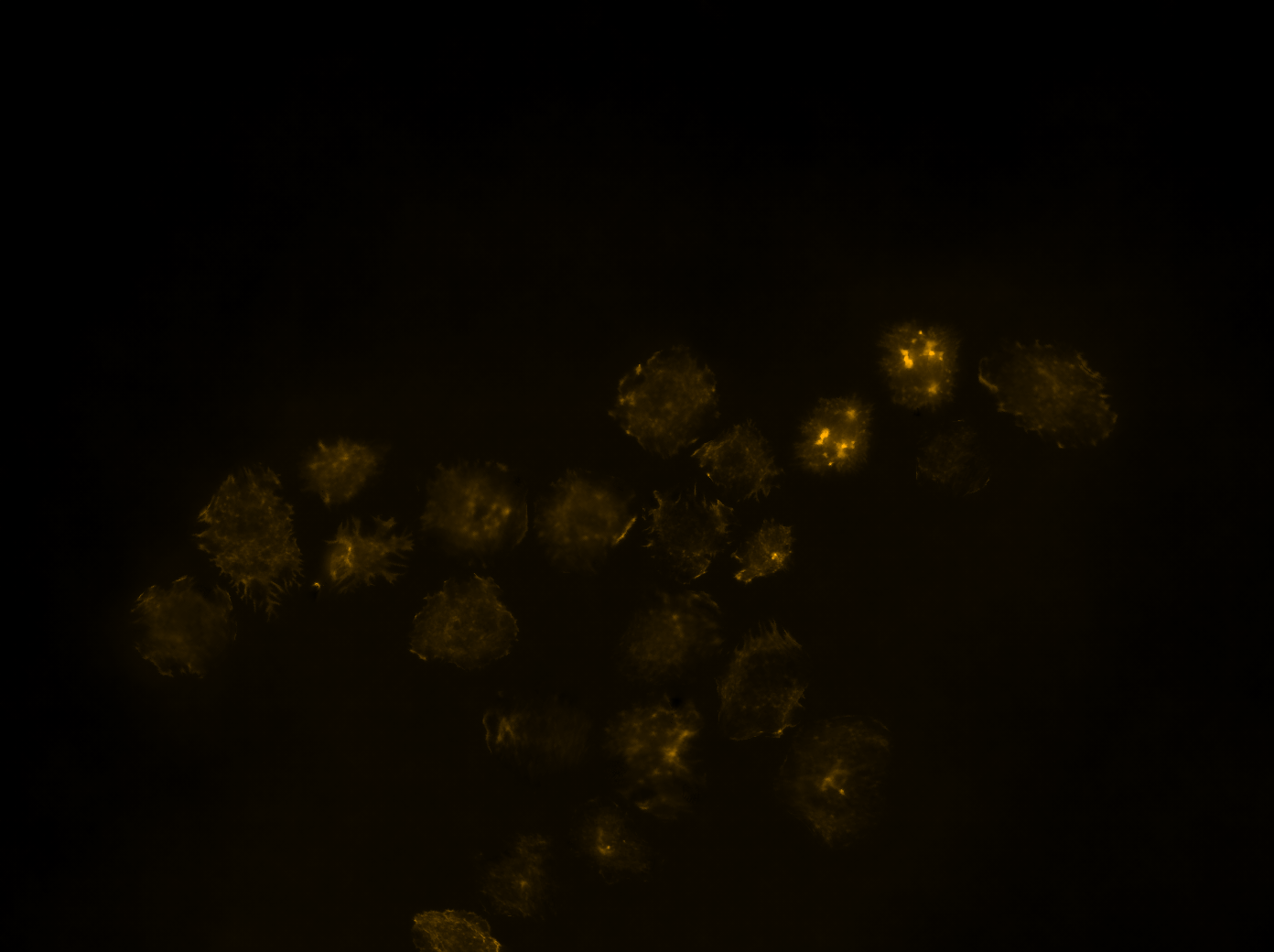

Supplement: Additional file 6 — The zip archive contains simulated images showing B cell nuclei and cytoskeleton with corresponding ground truth. (ZIP 119808 kb) [file 12859_2017_1591_MOESM6_ESM.zip › simulated B cells/cytoskeleton/not touching/cell004.png]

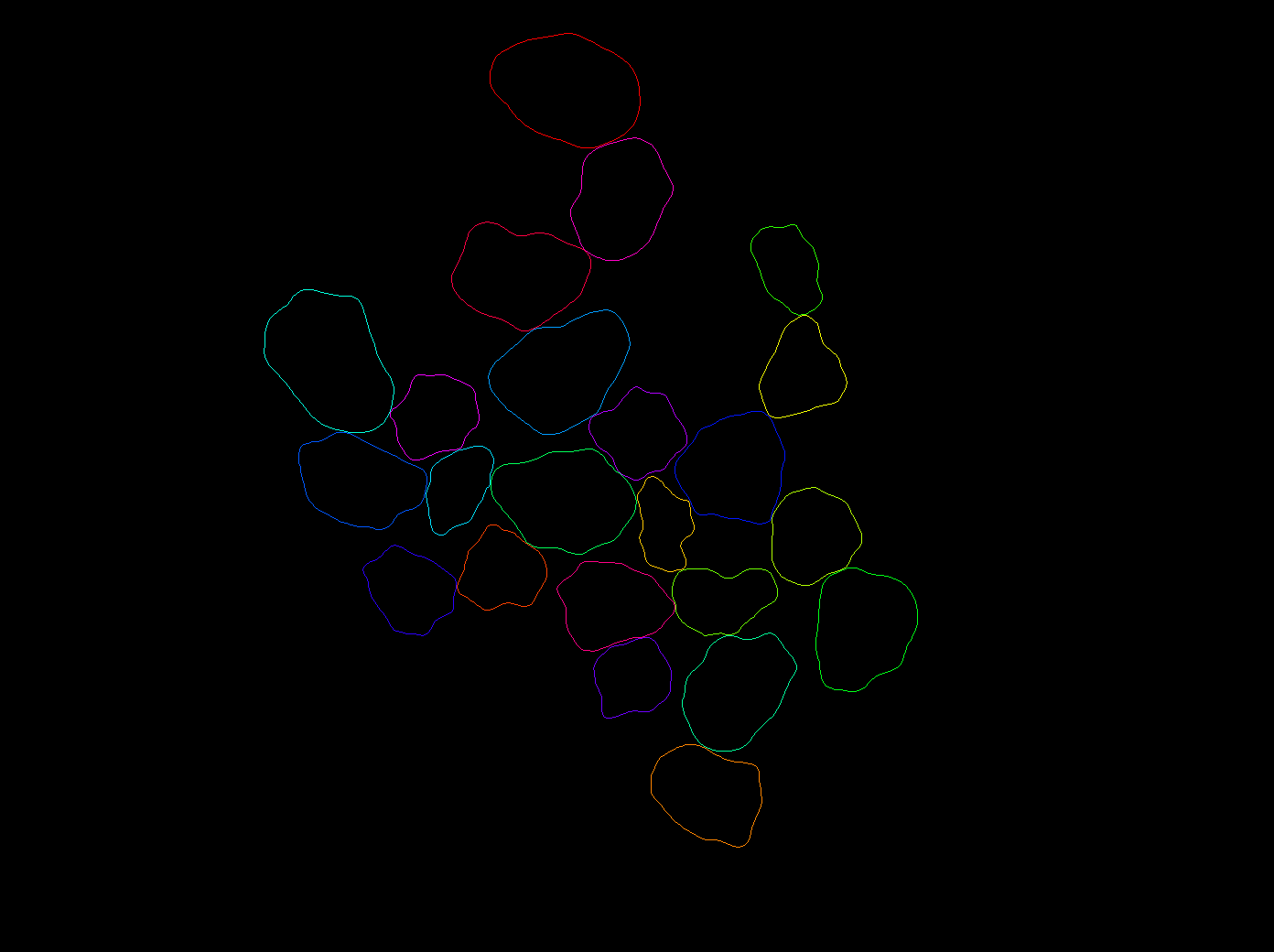

Supplement: Additional file 6 — The zip archive contains simulated images showing B cell nuclei and cytoskeleton with corresponding ground truth. (ZIP 119808 kb) [file 12859_2017_1591_MOESM6_ESM.zip › simulated B cells/cytoskeleton/not touching/cell005 gt.png]

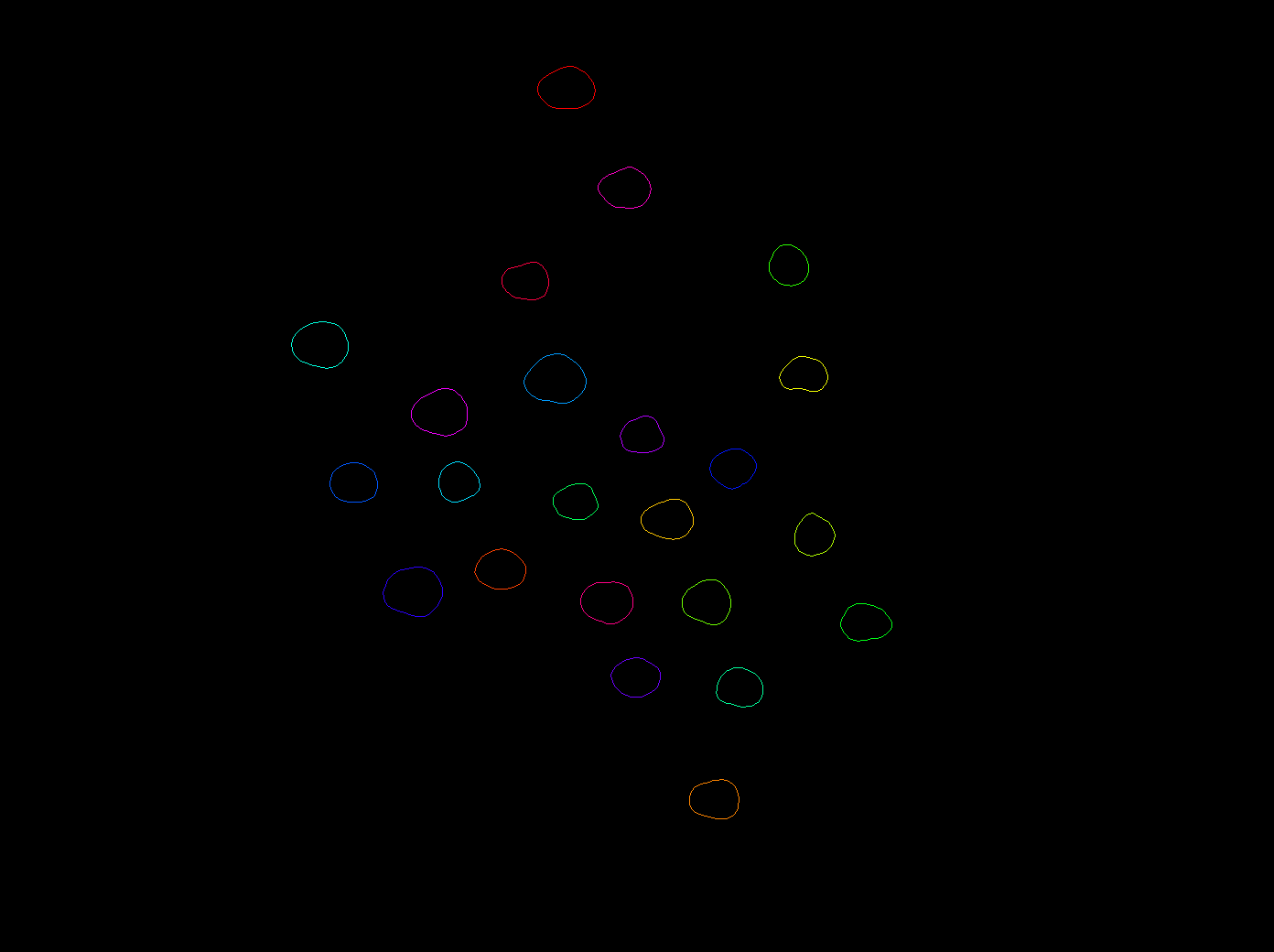

Supplement: Additional file 6 — The zip archive contains simulated images showing B cell nuclei and cytoskeleton with corresponding ground truth. (ZIP 119808 kb) [file 12859_2017_1591_MOESM6_ESM.zip › simulated B cells/cytoskeleton/not touching/cell005 seeds.png]

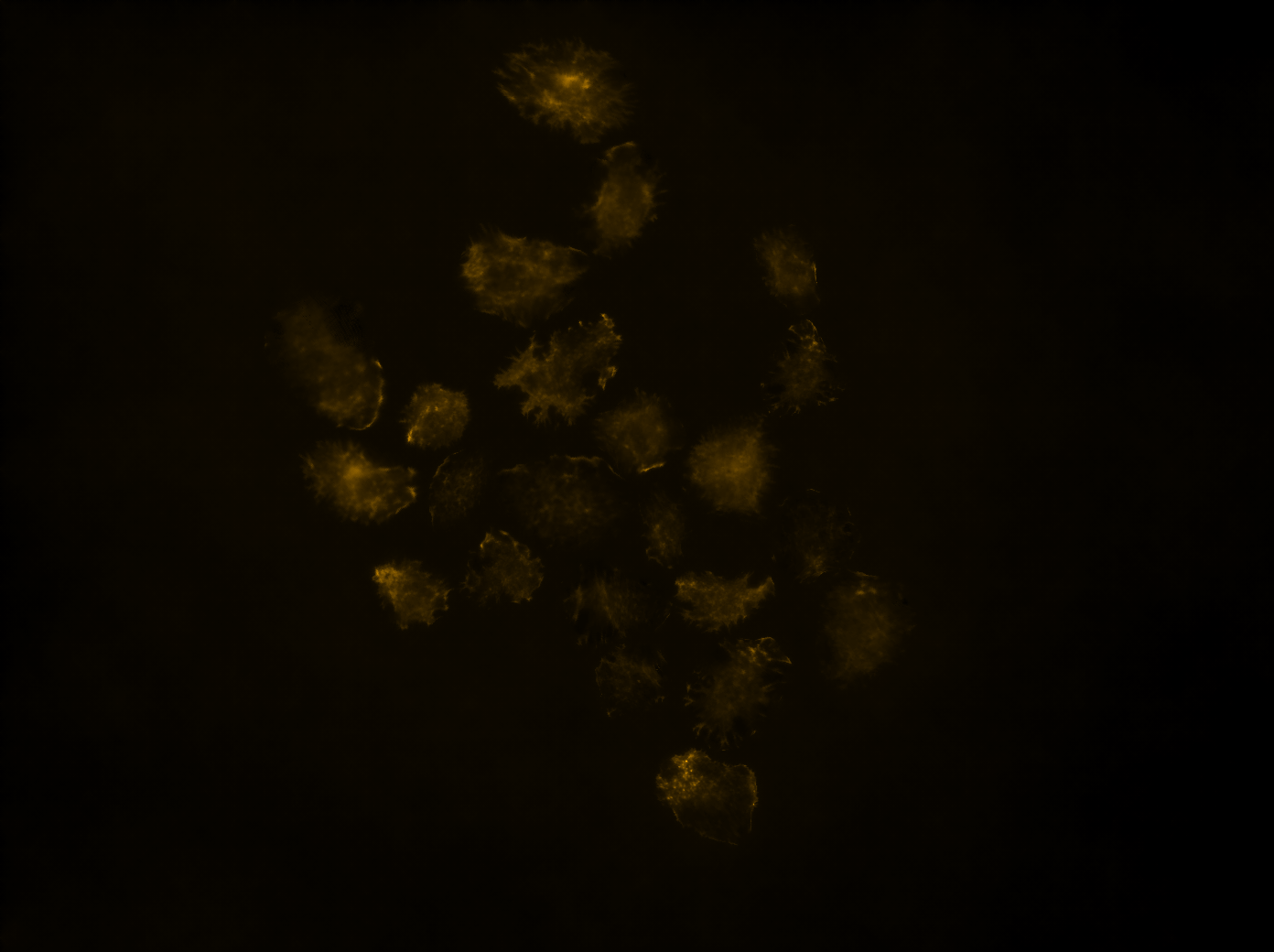

Supplement: Additional file 6 — The zip archive contains simulated images showing B cell nuclei and cytoskeleton with corresponding ground truth. (ZIP 119808 kb) [file 12859_2017_1591_MOESM6_ESM.zip › simulated B cells/cytoskeleton/not touching/cell005.png]

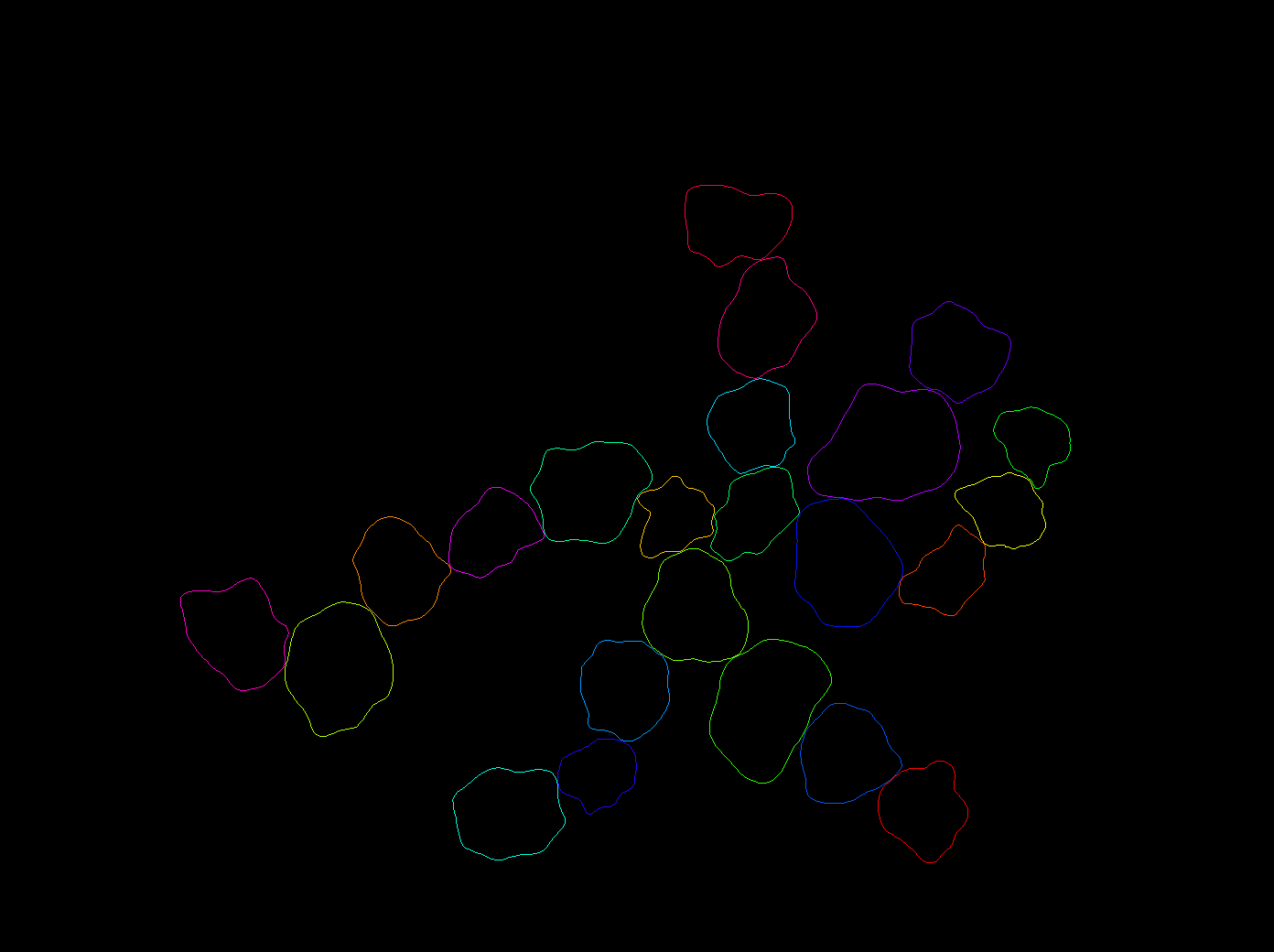

Supplement: Additional file 6 — The zip archive contains simulated images showing B cell nuclei and cytoskeleton with corresponding ground truth. (ZIP 119808 kb) [file 12859_2017_1591_MOESM6_ESM.zip › simulated B cells/cytoskeleton/not touching/cell006 gt.png]

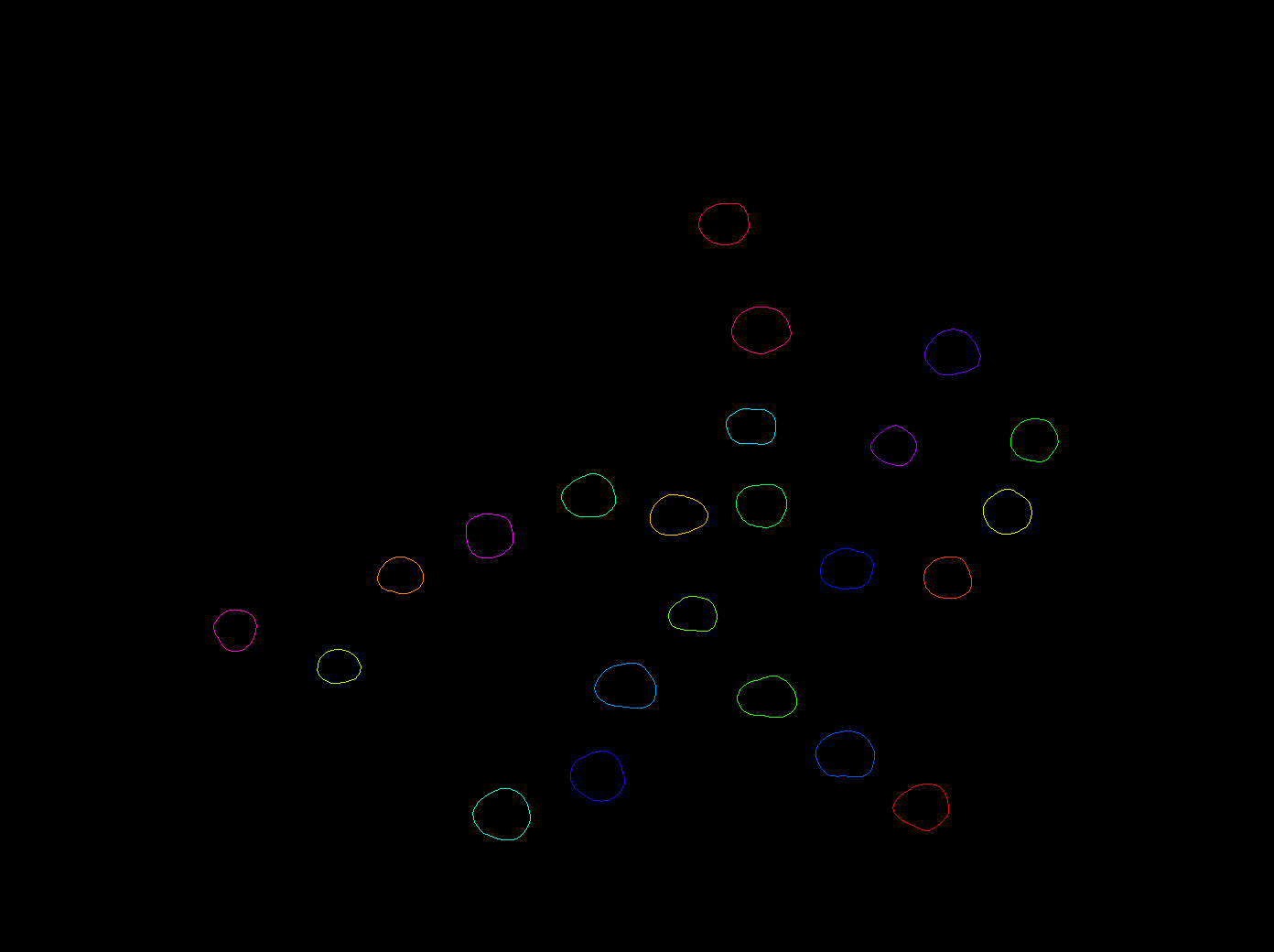

Supplement: Additional file 6 — The zip archive contains simulated images showing B cell nuclei and cytoskeleton with corresponding ground truth. (ZIP 119808 kb) [file 12859_2017_1591_MOESM6_ESM.zip › simulated B cells/cytoskeleton/not touching/cell006 seeds.png]

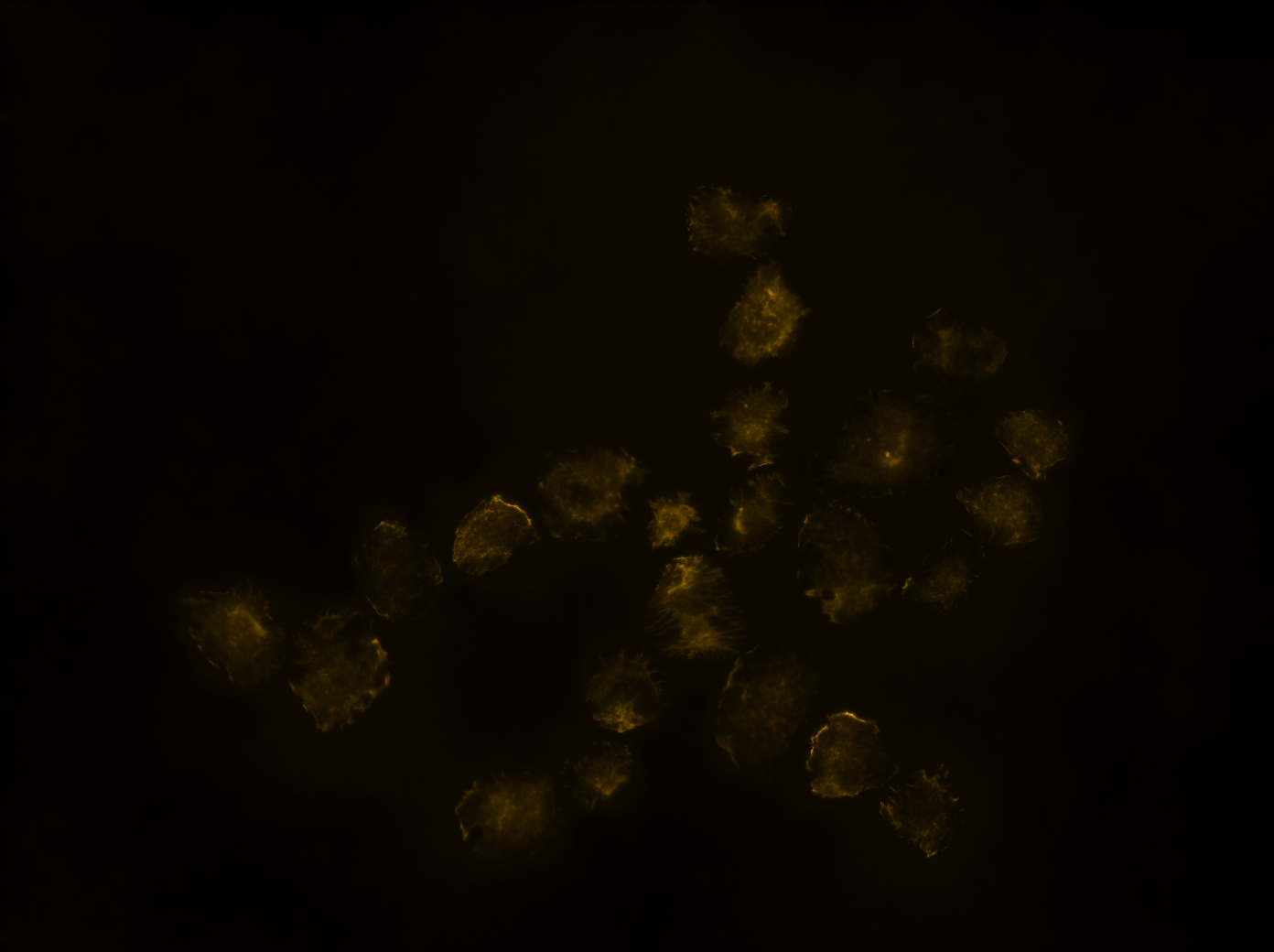

Supplement: Additional file 6 — The zip archive contains simulated images showing B cell nuclei and cytoskeleton with corresponding ground truth. (ZIP 119808 kb) [file 12859_2017_1591_MOESM6_ESM.zip › simulated B cells/cytoskeleton/not touching/cell006.png]

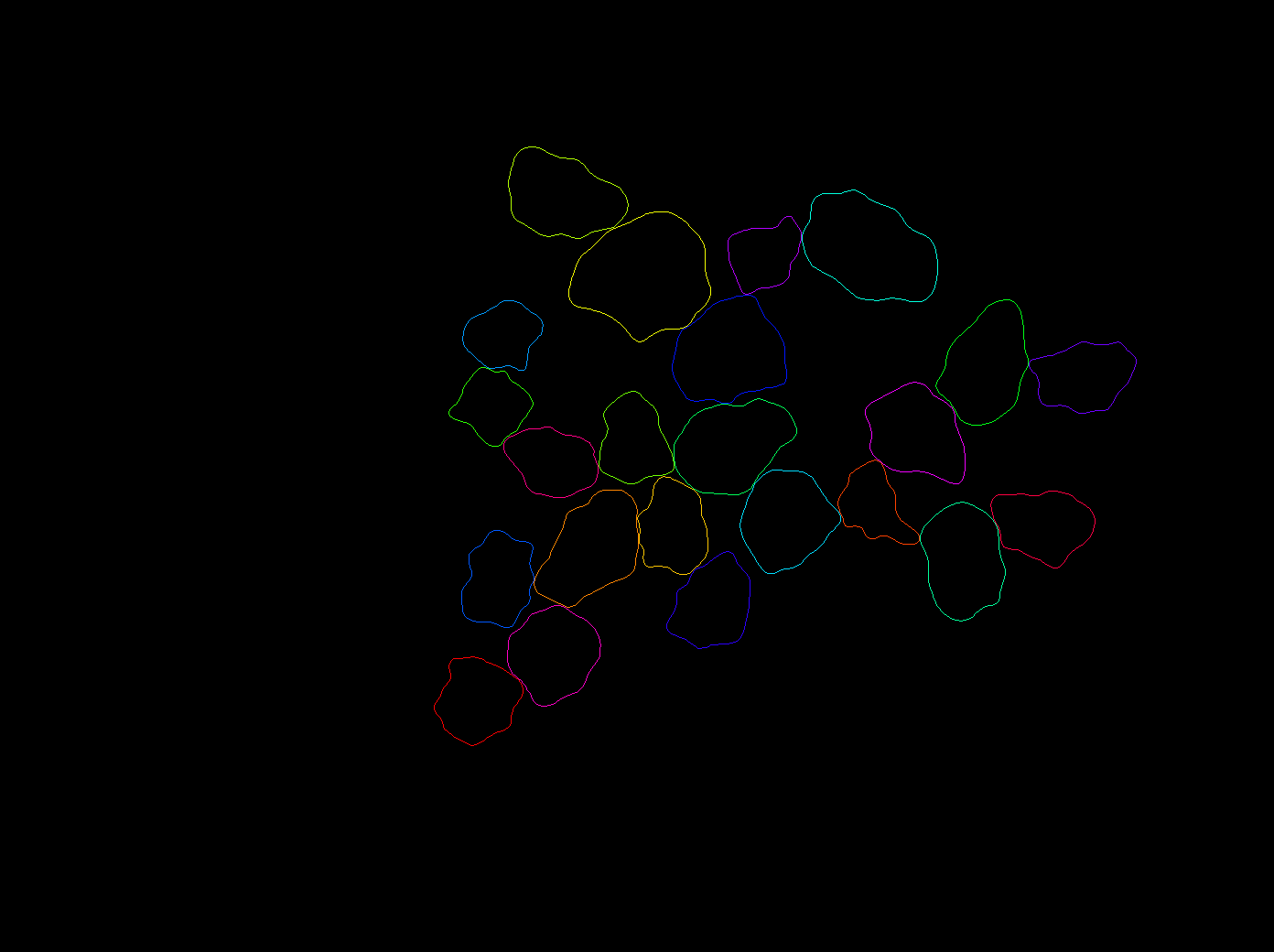

Supplement: Additional file 6 — The zip archive contains simulated images showing B cell nuclei and cytoskeleton with corresponding ground truth. (ZIP 119808 kb) [file 12859_2017_1591_MOESM6_ESM.zip › simulated B cells/cytoskeleton/not touching/cell007 gt.png]

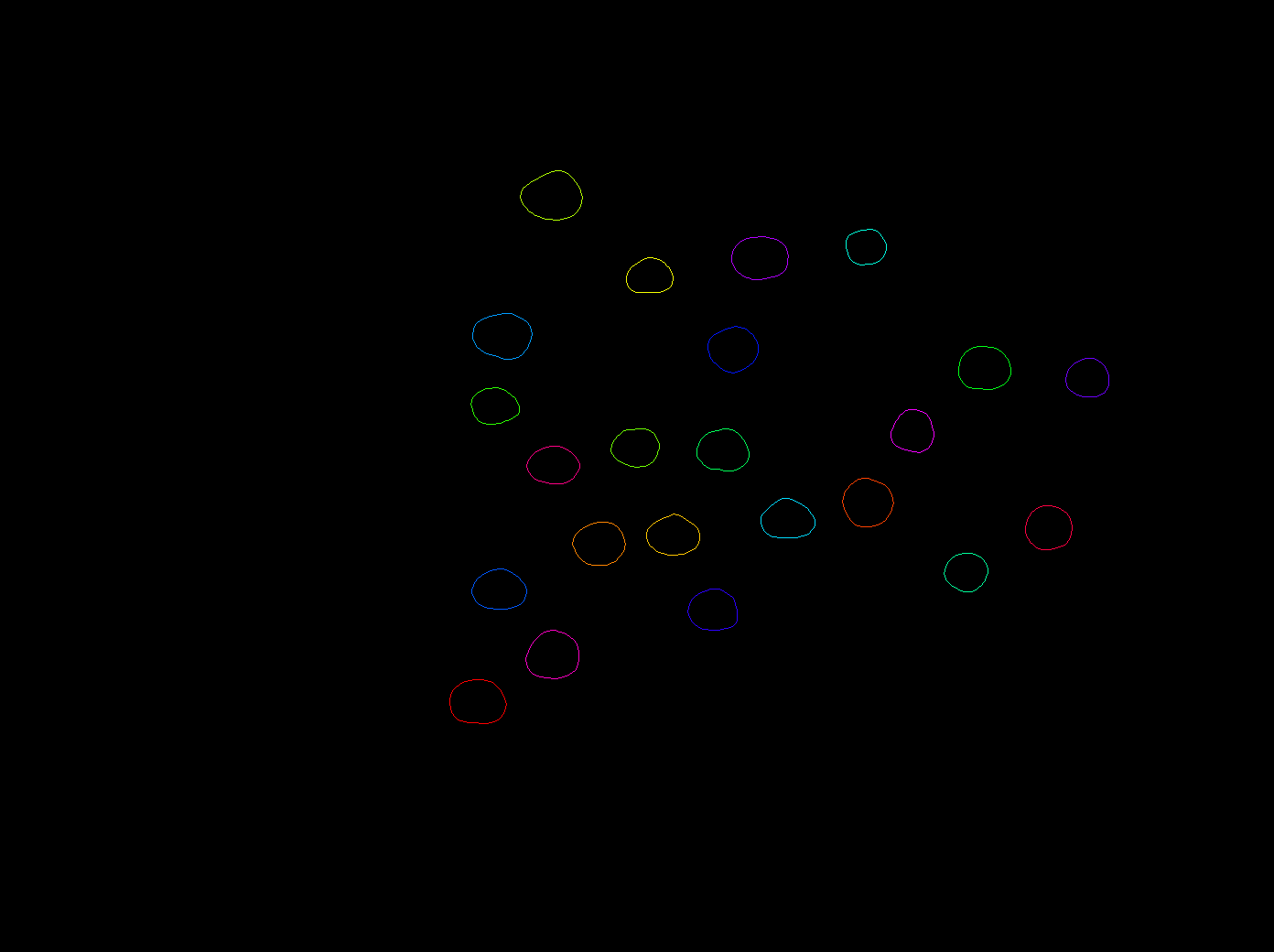

Supplement: Additional file 6 — The zip archive contains simulated images showing B cell nuclei and cytoskeleton with corresponding ground truth. (ZIP 119808 kb) [file 12859_2017_1591_MOESM6_ESM.zip › simulated B cells/cytoskeleton/not touching/cell007 seeds.png]

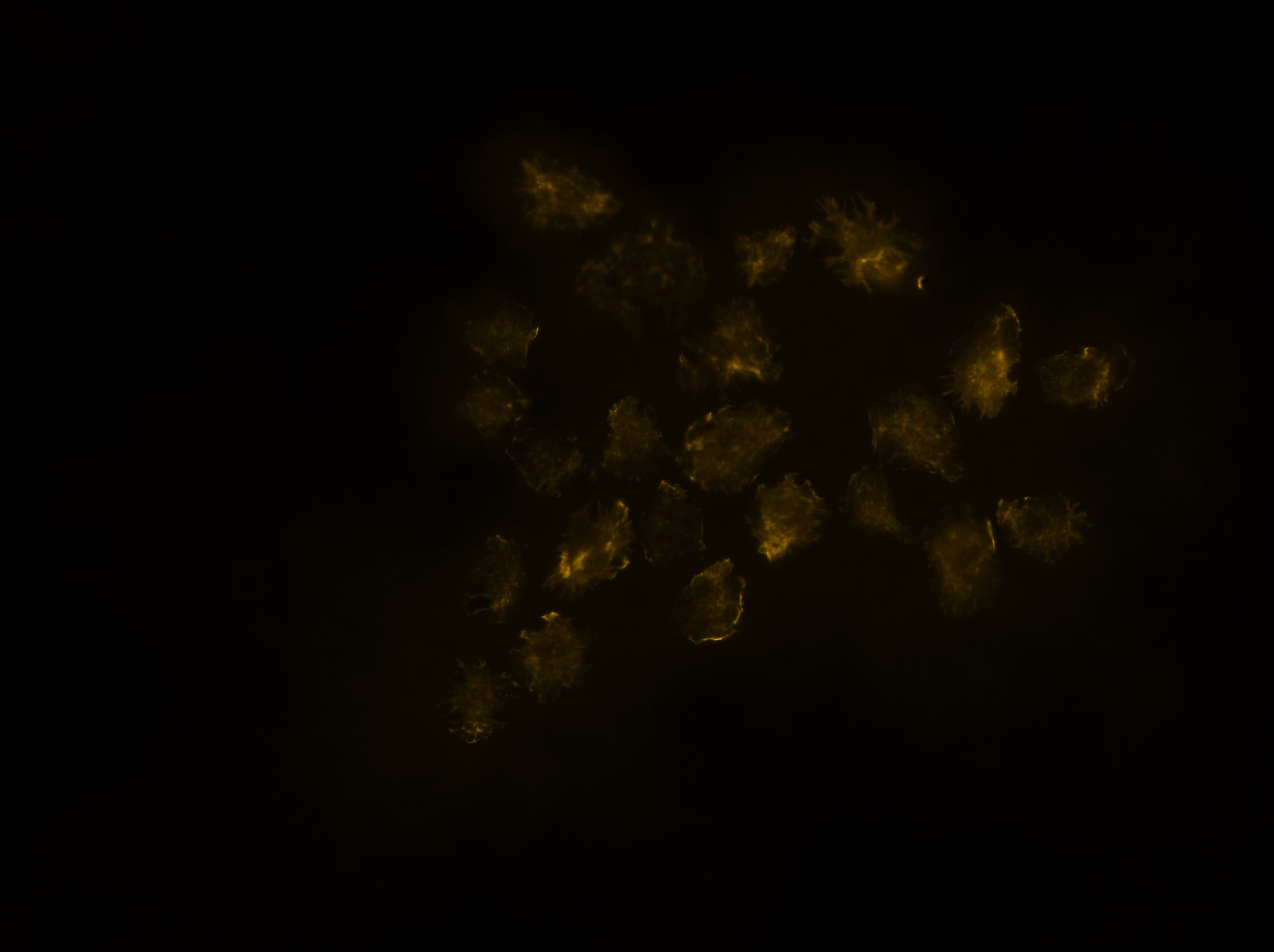

Supplement: Additional file 6 — The zip archive contains simulated images showing B cell nuclei and cytoskeleton with corresponding ground truth. (ZIP 119808 kb) [file 12859_2017_1591_MOESM6_ESM.zip › simulated B cells/cytoskeleton/not touching/cell007.png]

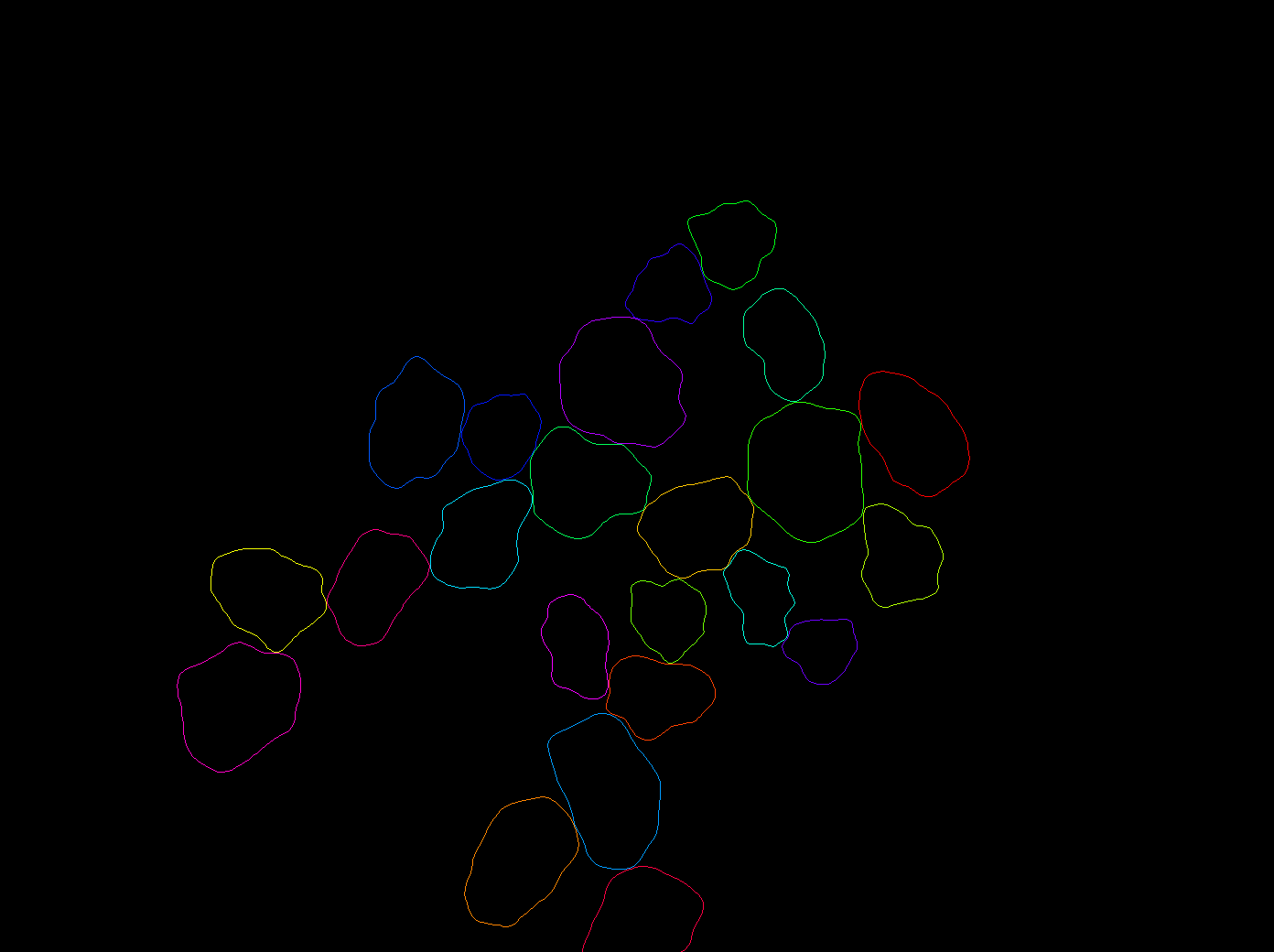

Supplement: Additional file 6 — The zip archive contains simulated images showing B cell nuclei and cytoskeleton with corresponding ground truth. (ZIP 119808 kb) [file 12859_2017_1591_MOESM6_ESM.zip › simulated B cells/cytoskeleton/not touching/cell008 gt.png]

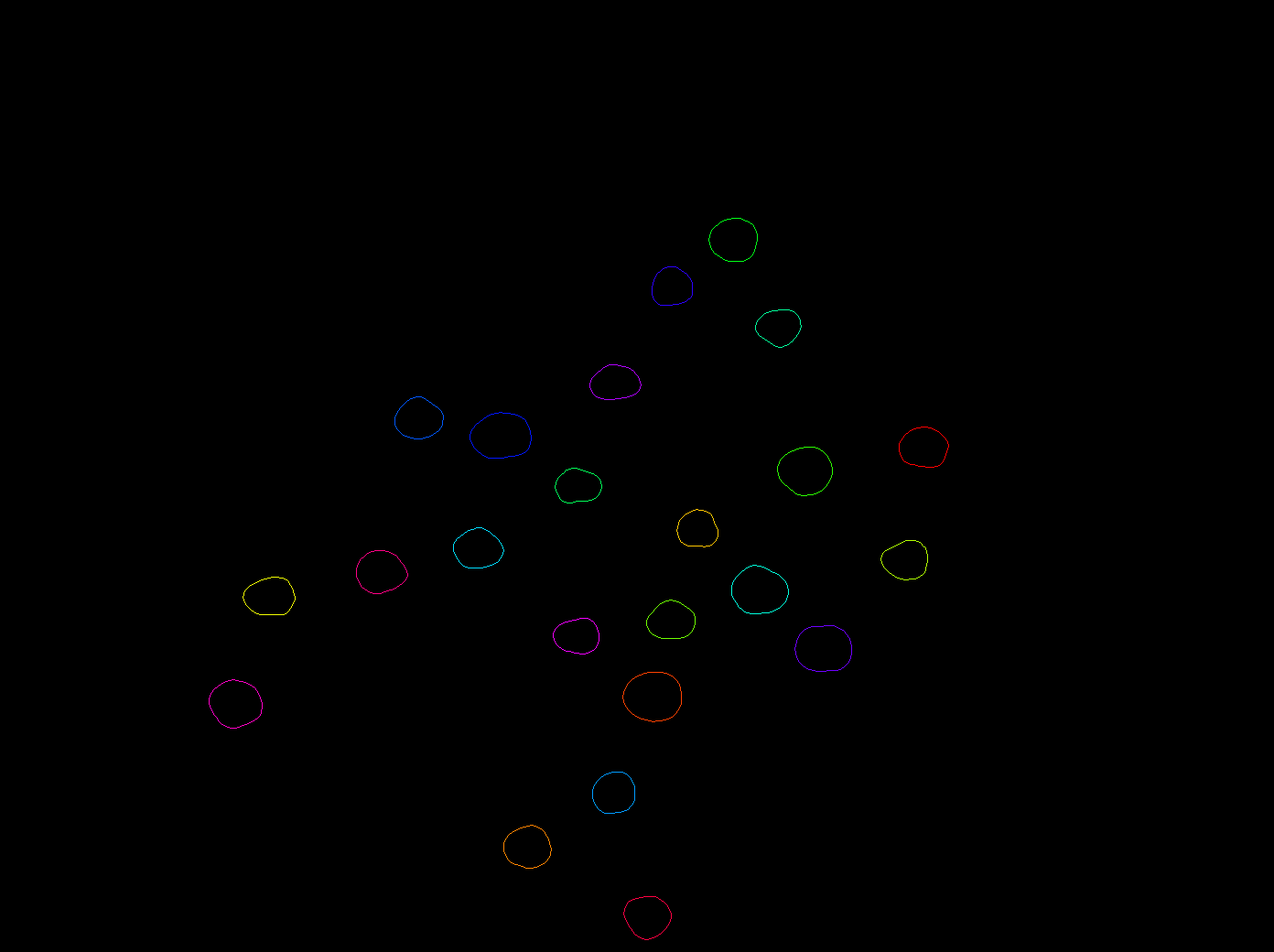

Supplement: Additional file 6 — The zip archive contains simulated images showing B cell nuclei and cytoskeleton with corresponding ground truth. (ZIP 119808 kb) [file 12859_2017_1591_MOESM6_ESM.zip › simulated B cells/cytoskeleton/not touching/cell008 seeds.png]

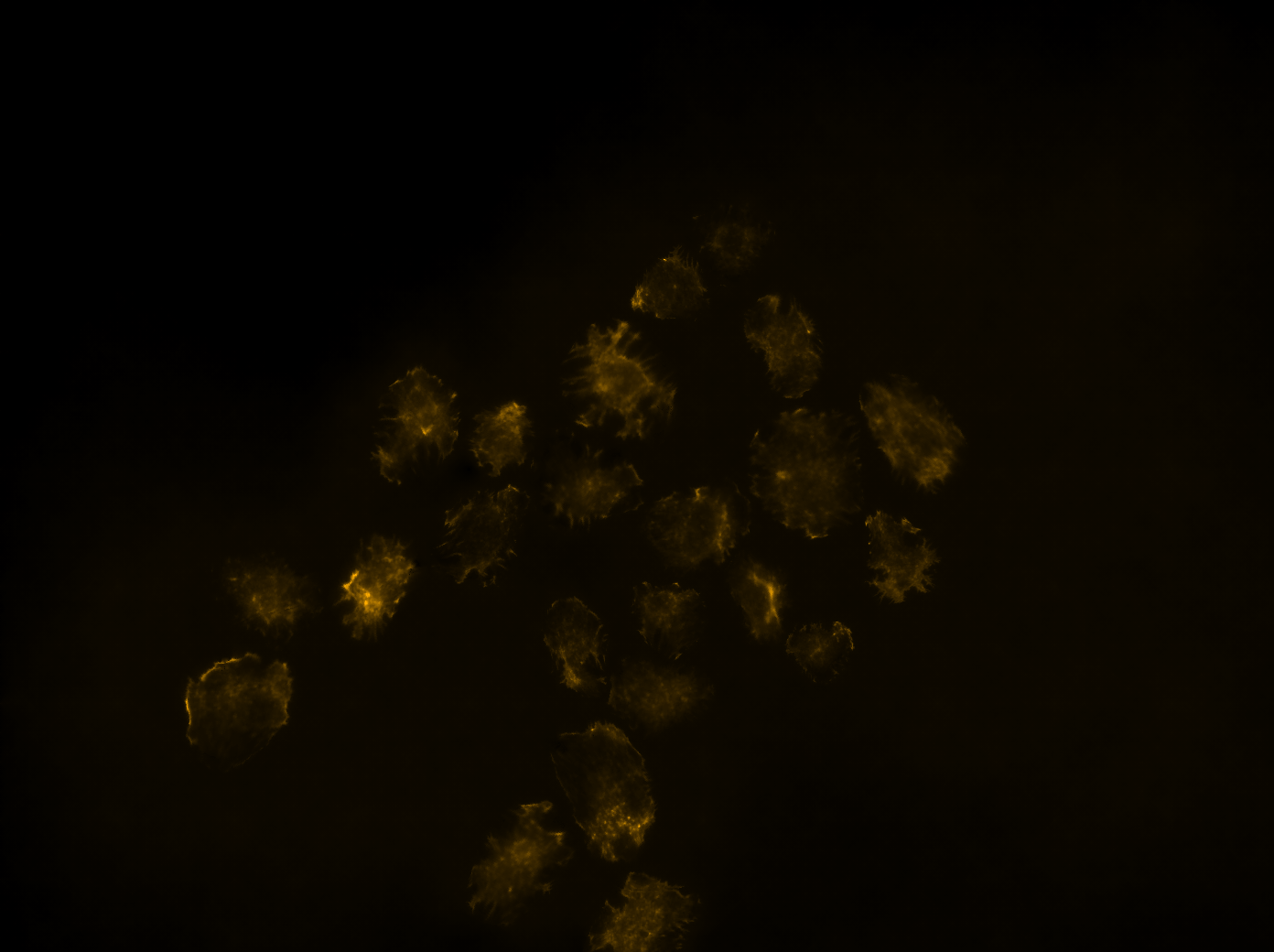

Supplement: Additional file 6 — The zip archive contains simulated images showing B cell nuclei and cytoskeleton with corresponding ground truth. (ZIP 119808 kb) [file 12859_2017_1591_MOESM6_ESM.zip › simulated B cells/cytoskeleton/not touching/cell008.png]

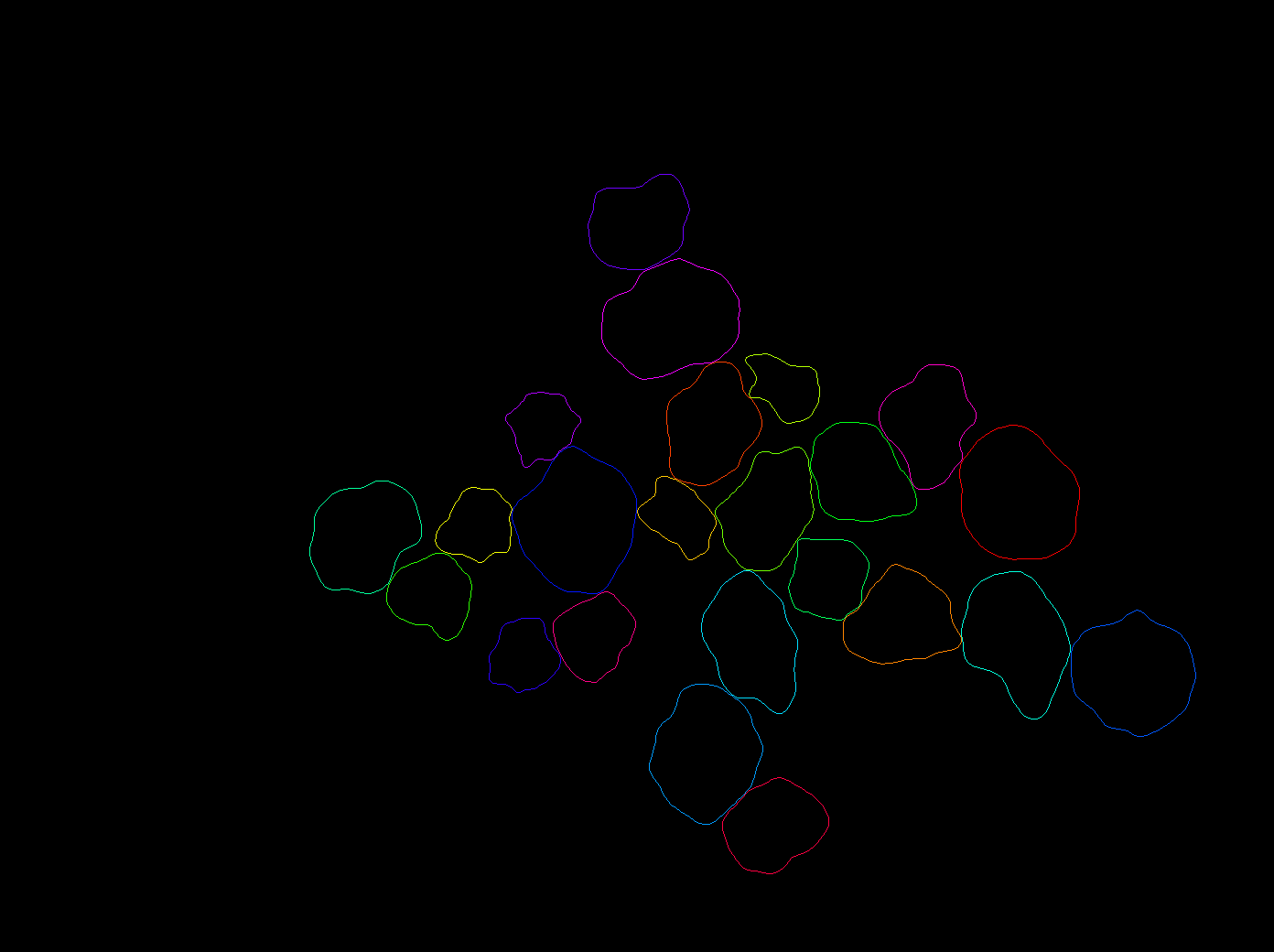

Supplement: Additional file 6 — The zip archive contains simulated images showing B cell nuclei and cytoskeleton with corresponding ground truth. (ZIP 119808 kb) [file 12859_2017_1591_MOESM6_ESM.zip › simulated B cells/cytoskeleton/not touching/cell009 gt.png]

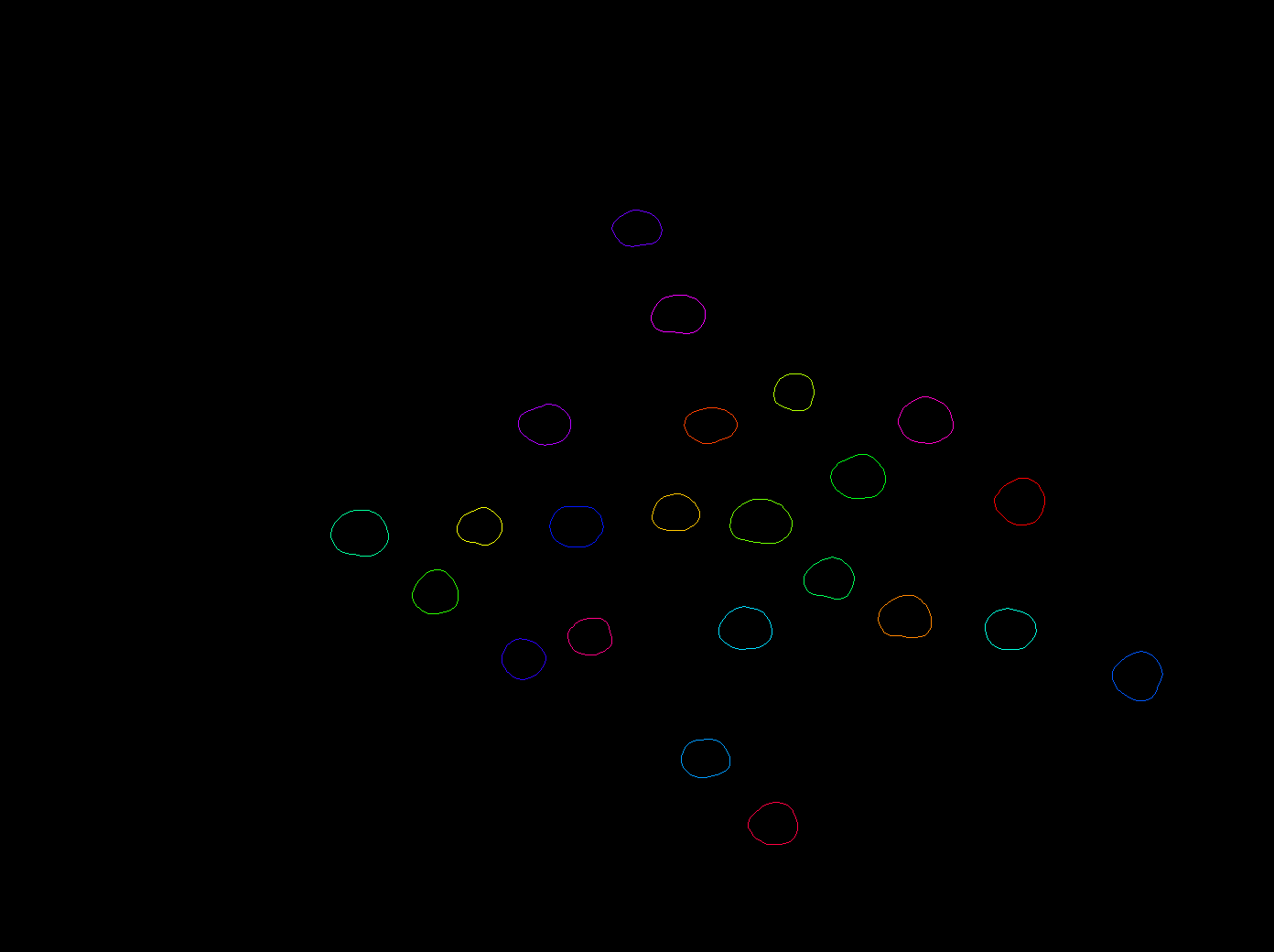

Supplement: Additional file 6 — The zip archive contains simulated images showing B cell nuclei and cytoskeleton with corresponding ground truth. (ZIP 119808 kb) [file 12859_2017_1591_MOESM6_ESM.zip › simulated B cells/cytoskeleton/not touching/cell009 seeds.png]

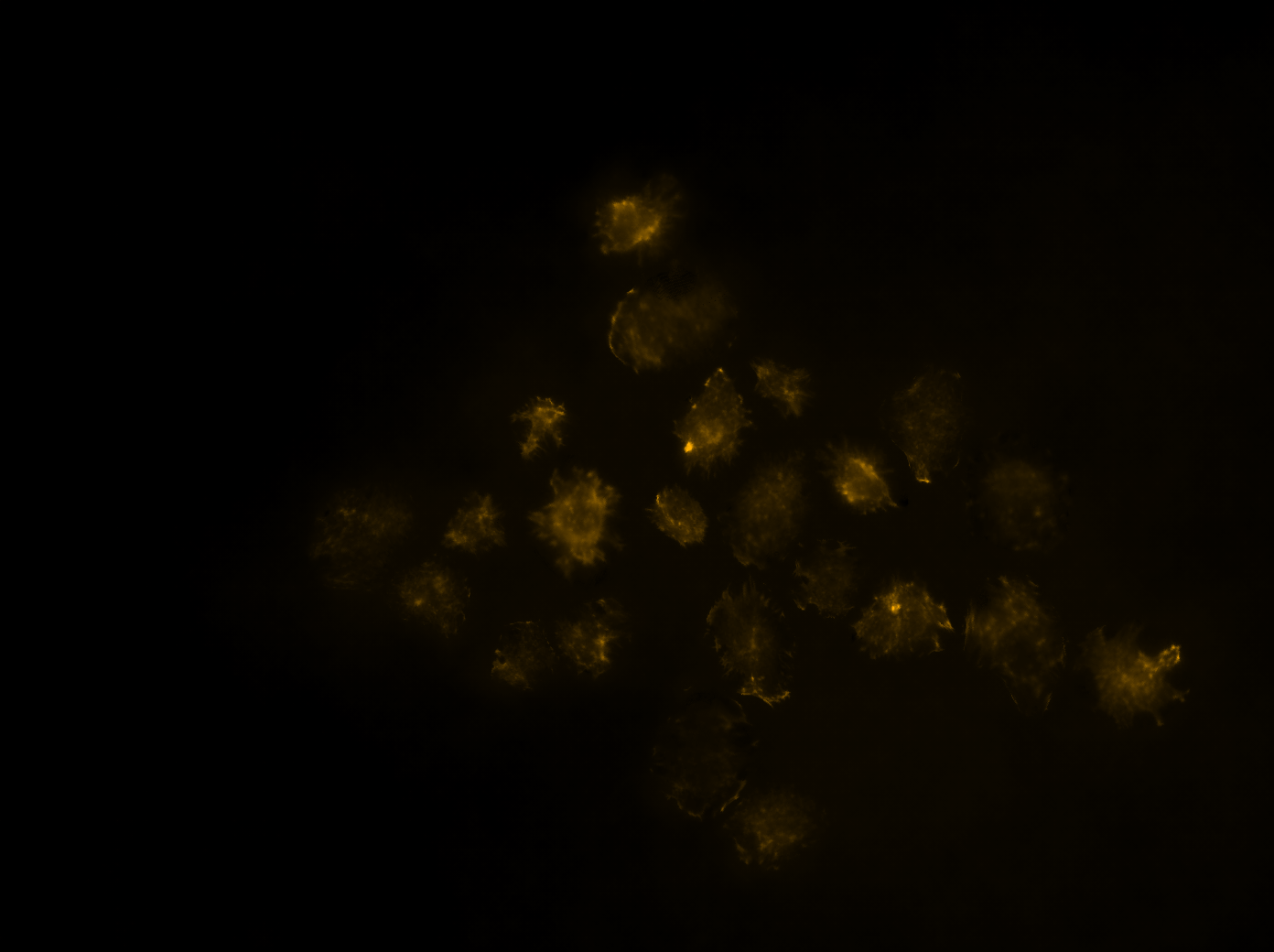

Supplement: Additional file 6 — The zip archive contains simulated images showing B cell nuclei and cytoskeleton with corresponding ground truth. (ZIP 119808 kb) [file 12859_2017_1591_MOESM6_ESM.zip › simulated B cells/cytoskeleton/not touching/cell009.png]

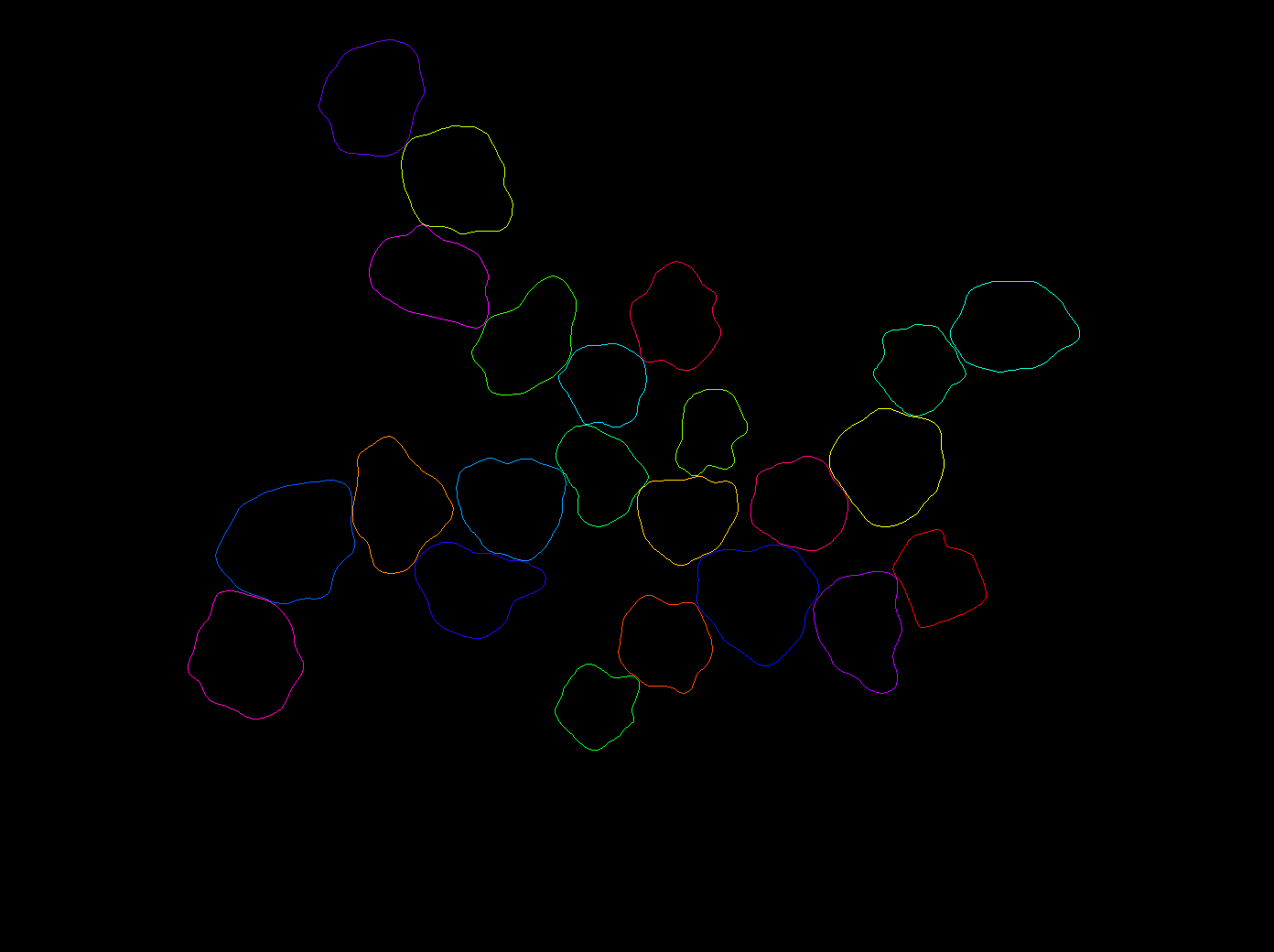

Supplement: Additional file 6 — The zip archive contains simulated images showing B cell nuclei and cytoskeleton with corresponding ground truth. (ZIP 119808 kb) [file 12859_2017_1591_MOESM6_ESM.zip › simulated B cells/cytoskeleton/not touching/cell010 gt.png]

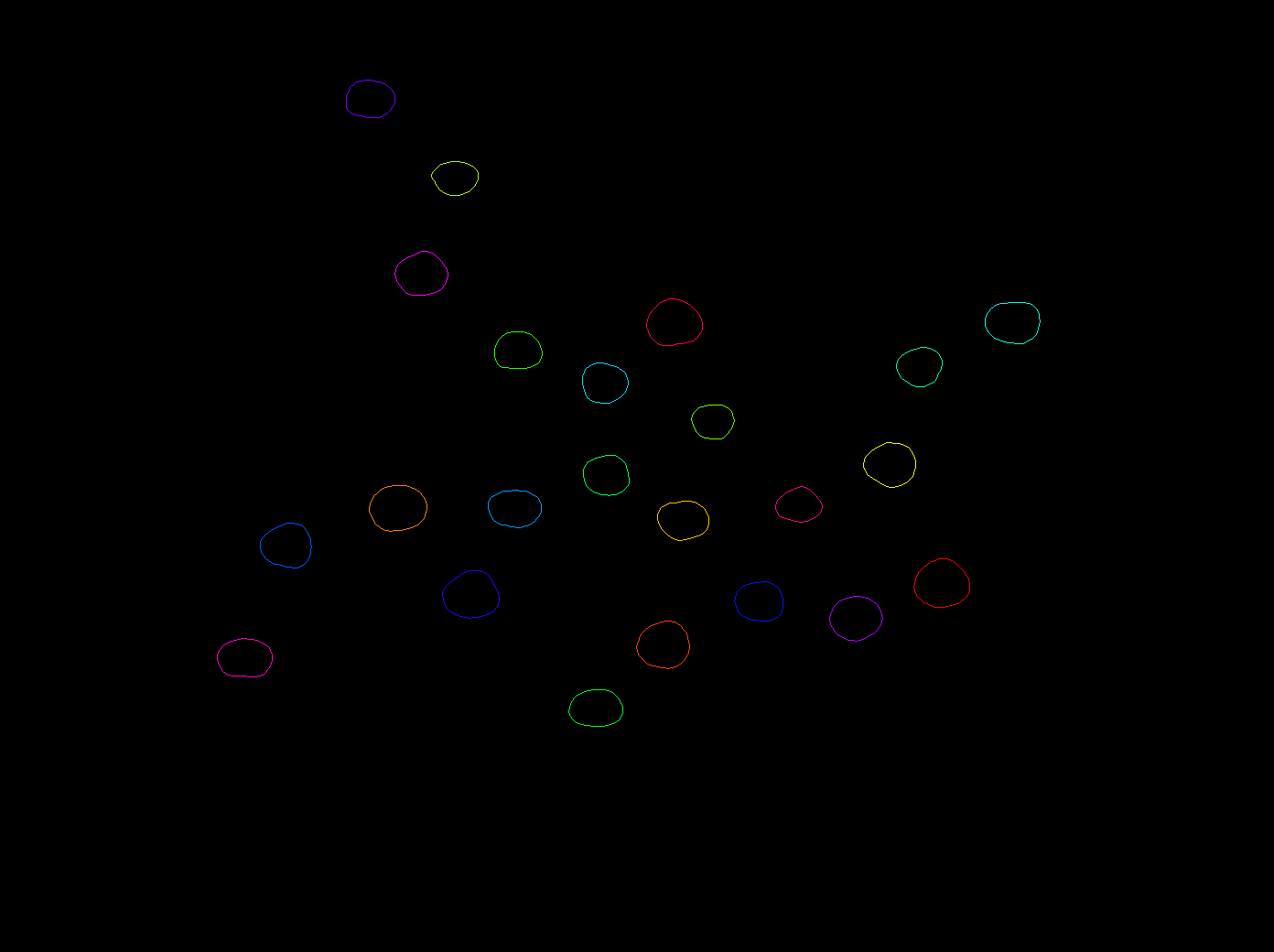

Supplement: Additional file 6 — The zip archive contains simulated images showing B cell nuclei and cytoskeleton with corresponding ground truth. (ZIP 119808 kb) [file 12859_2017_1591_MOESM6_ESM.zip › simulated B cells/cytoskeleton/not touching/cell010 seeds.png]

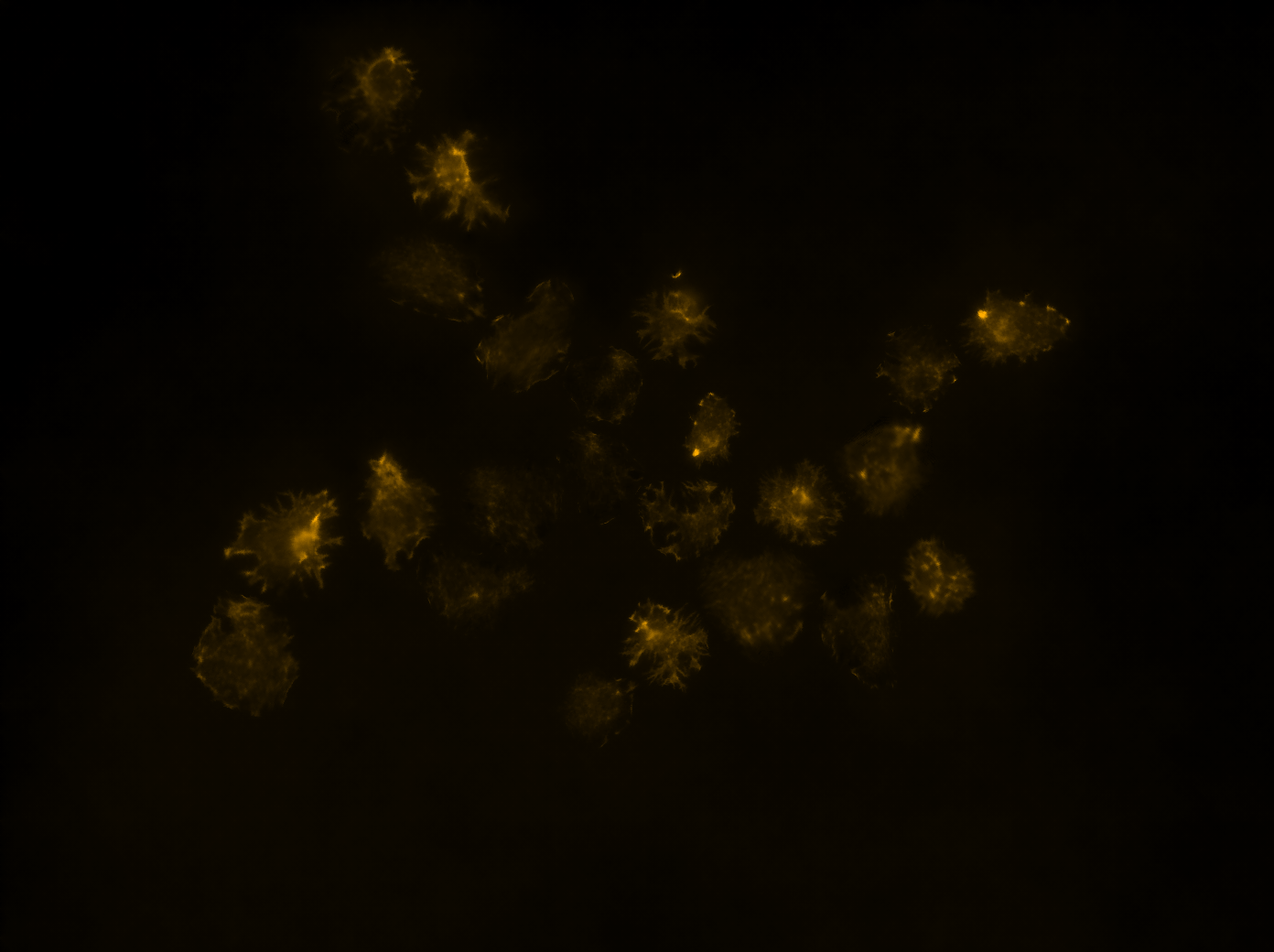

Supplement: Additional file 6 — The zip archive contains simulated images showing B cell nuclei and cytoskeleton with corresponding ground truth. (ZIP 119808 kb) [file 12859_2017_1591_MOESM6_ESM.zip › simulated B cells/cytoskeleton/not touching/cell010.png]

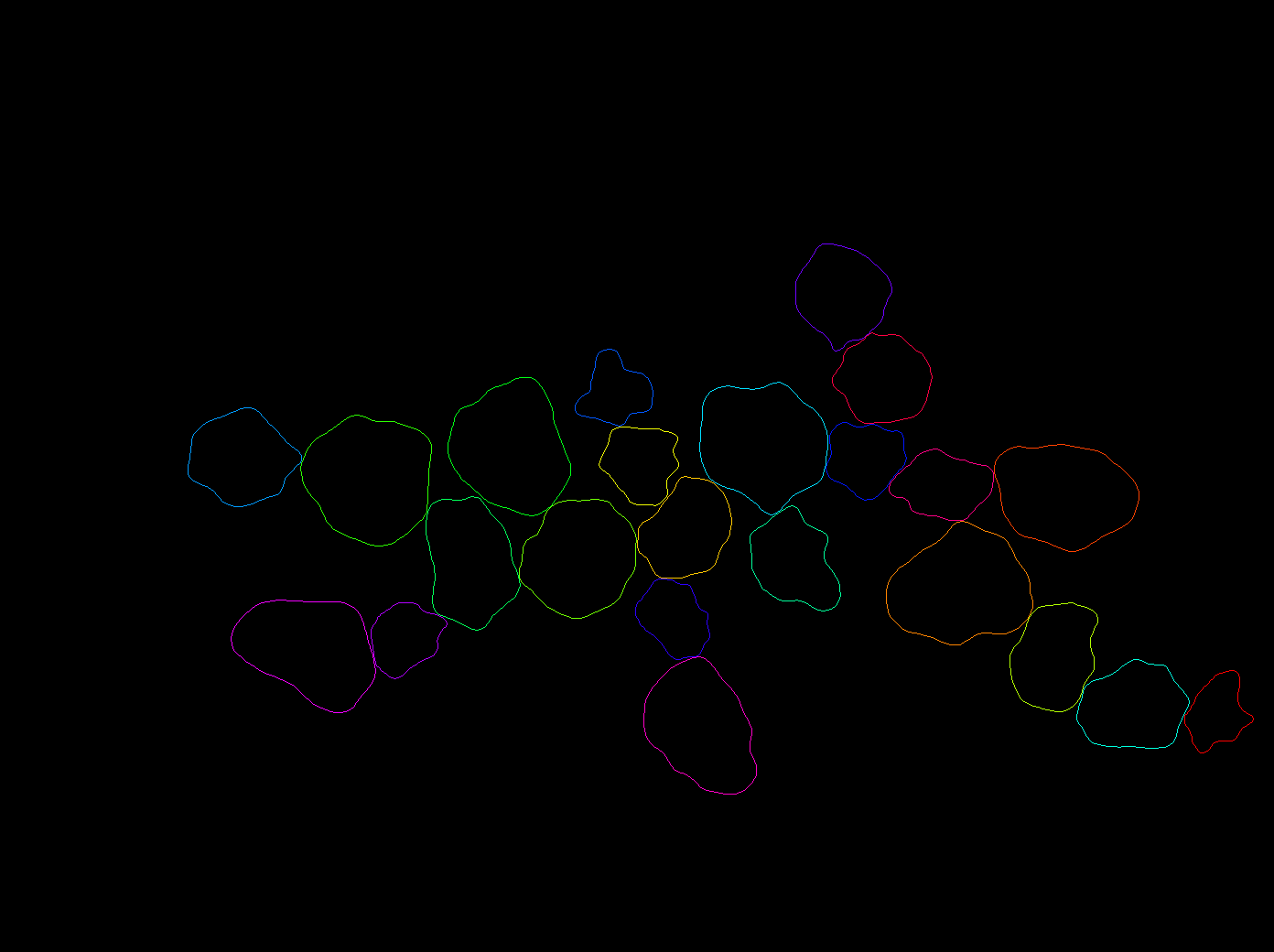

Supplement: Additional file 6 — The zip archive contains simulated images showing B cell nuclei and cytoskeleton with corresponding ground truth. (ZIP 119808 kb) [file 12859_2017_1591_MOESM6_ESM.zip › simulated B cells/cytoskeleton/not touching/cell011 gt.png]

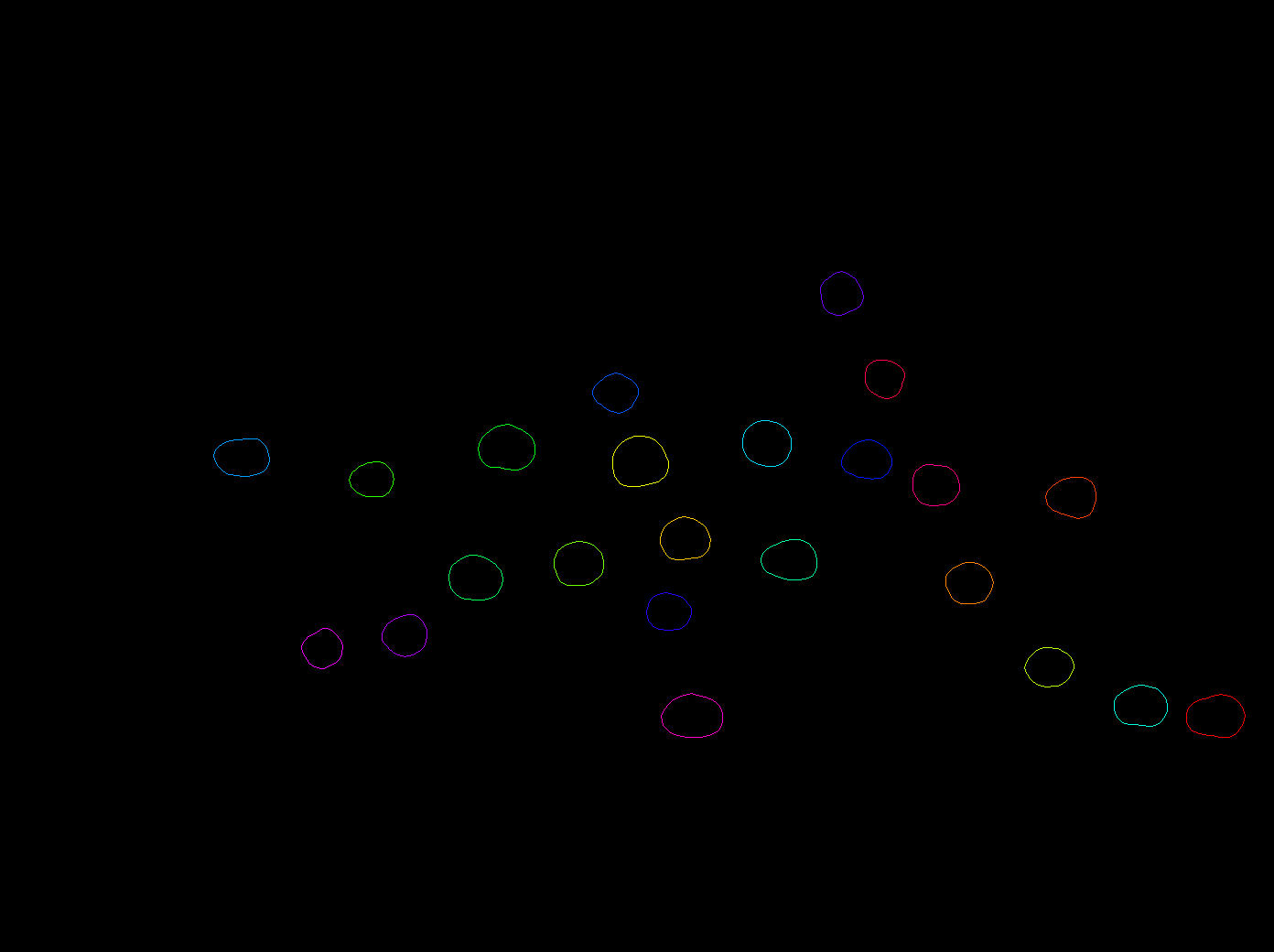

Supplement: Additional file 6 — The zip archive contains simulated images showing B cell nuclei and cytoskeleton with corresponding ground truth. (ZIP 119808 kb) [file 12859_2017_1591_MOESM6_ESM.zip › simulated B cells/cytoskeleton/not touching/cell011 seeds.png]

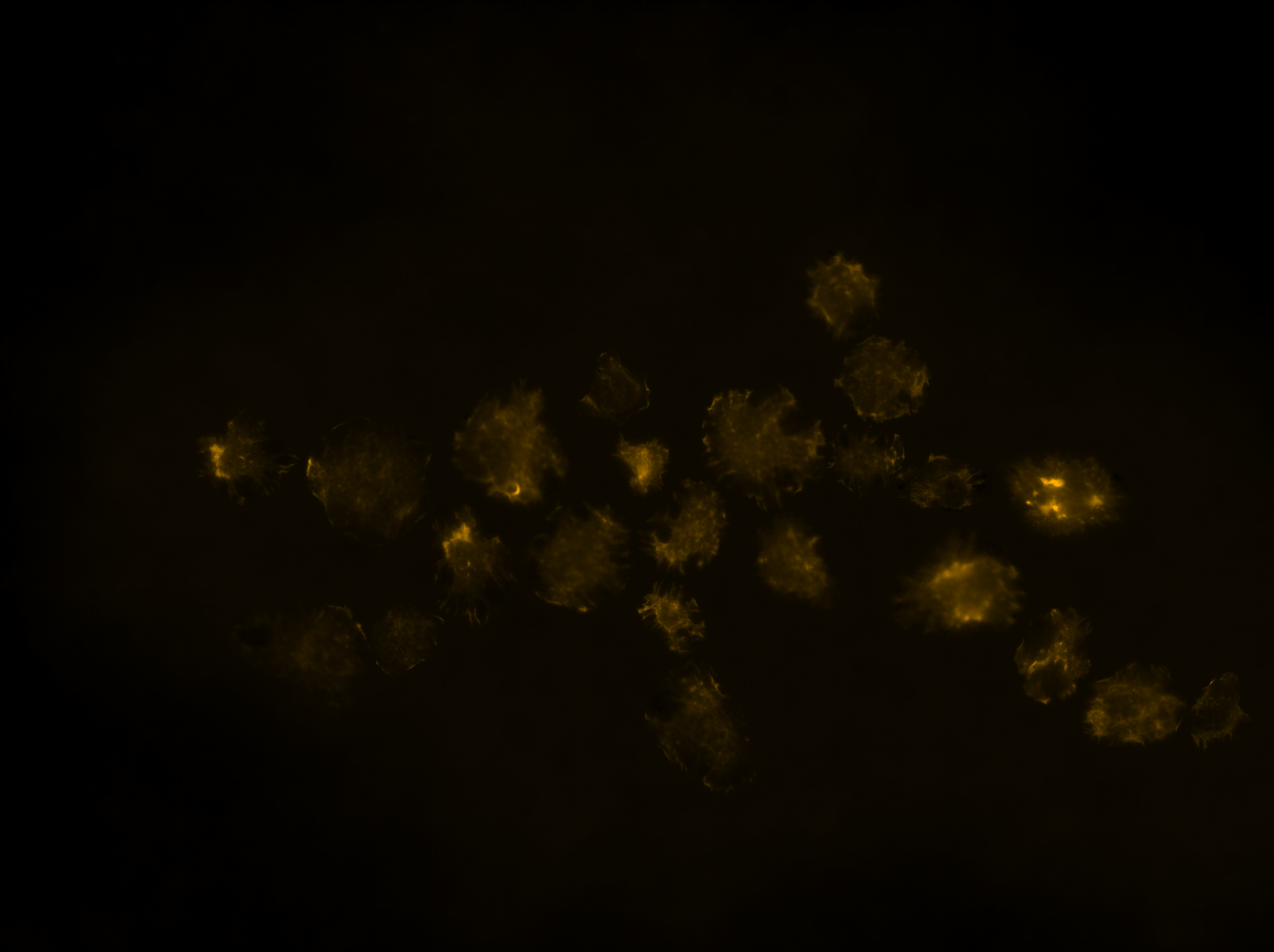

Supplement: Additional file 6 — The zip archive contains simulated images showing B cell nuclei and cytoskeleton with corresponding ground truth. (ZIP 119808 kb) [file 12859_2017_1591_MOESM6_ESM.zip › simulated B cells/cytoskeleton/not touching/cell011.png]

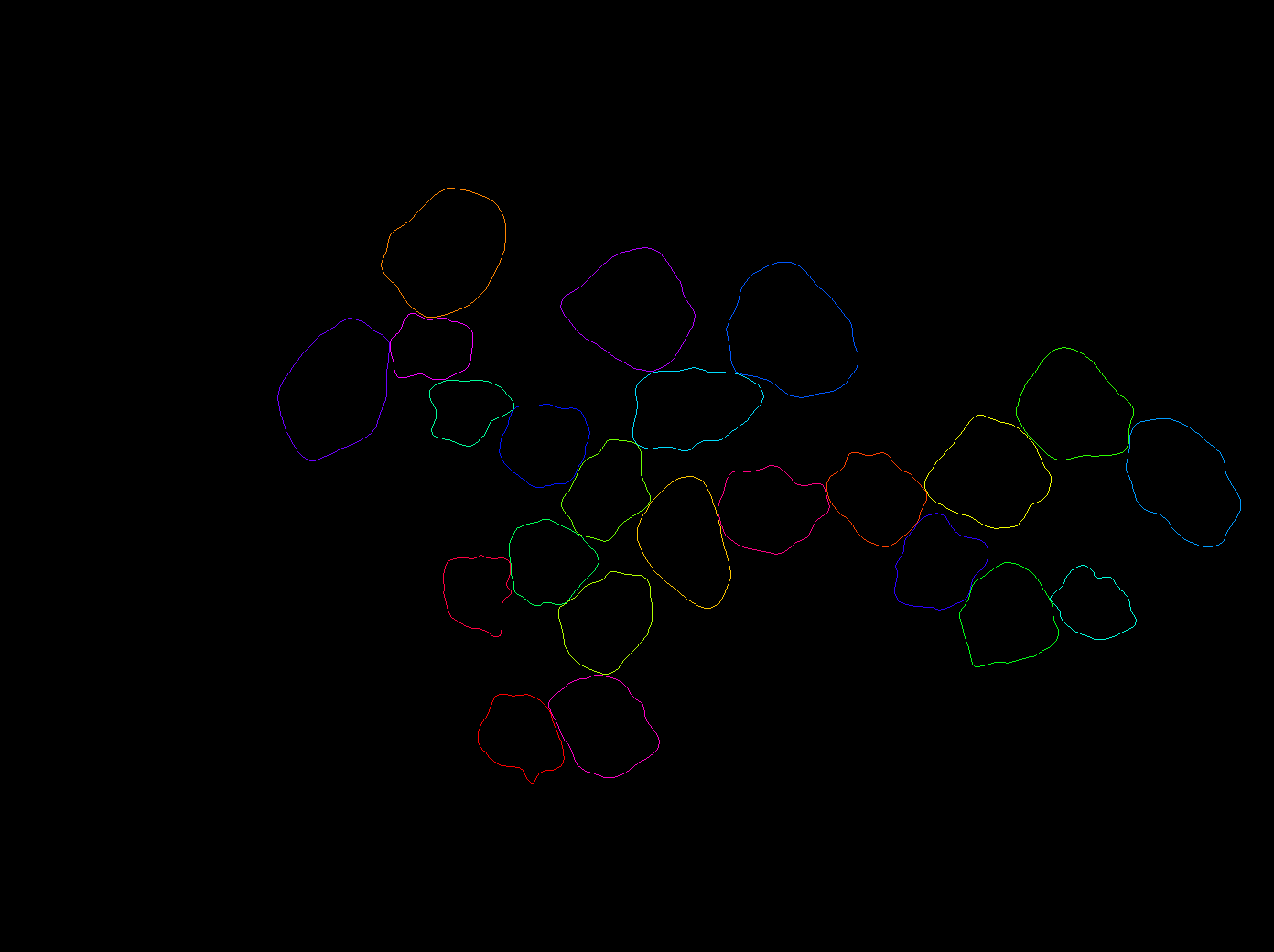

Supplement: Additional file 6 — The zip archive contains simulated images showing B cell nuclei and cytoskeleton with corresponding ground truth. (ZIP 119808 kb) [file 12859_2017_1591_MOESM6_ESM.zip › simulated B cells/cytoskeleton/not touching/cell012 gt.png]

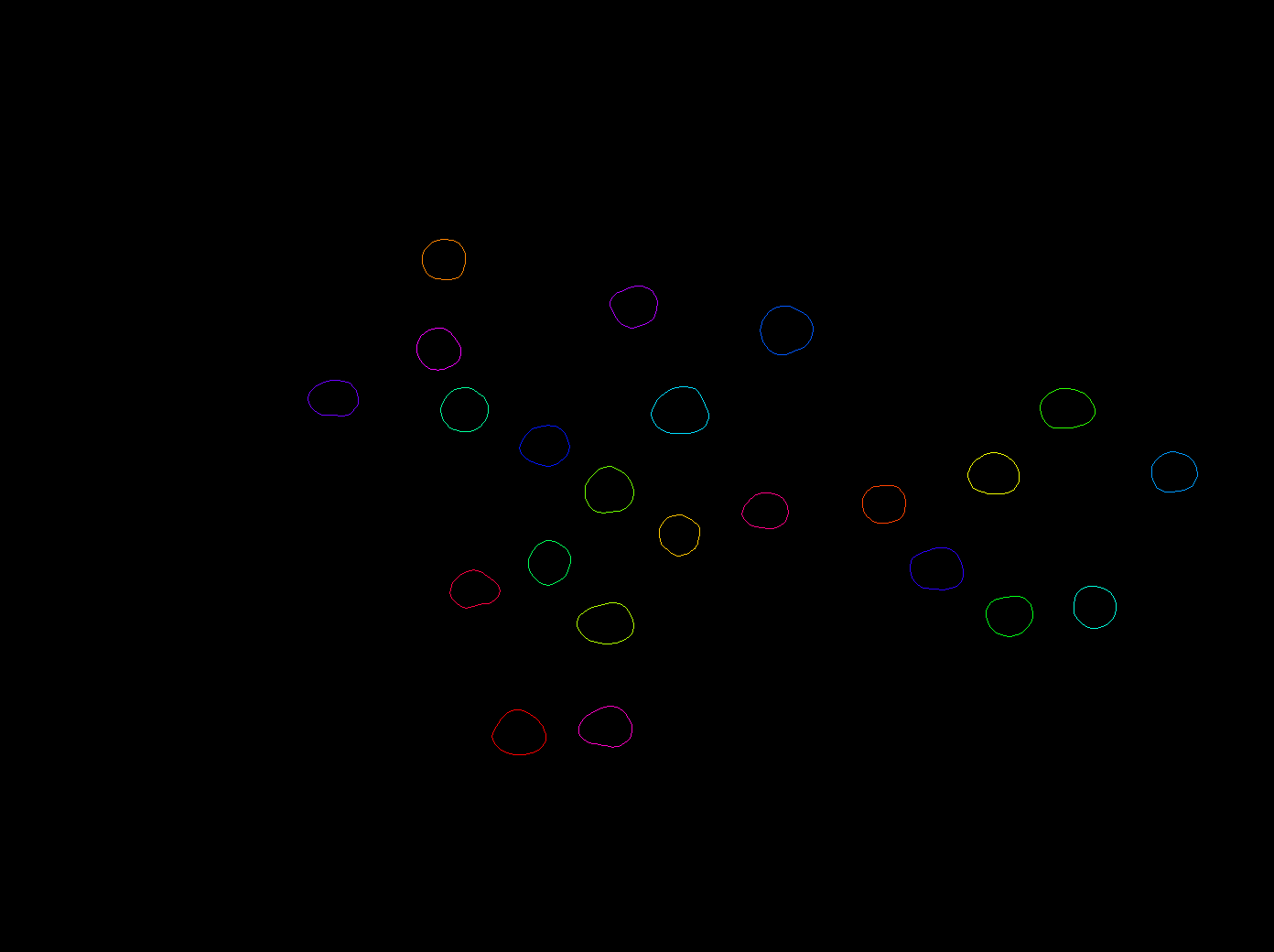

Supplement: Additional file 6 — The zip archive contains simulated images showing B cell nuclei and cytoskeleton with corresponding ground truth. (ZIP 119808 kb) [file 12859_2017_1591_MOESM6_ESM.zip › simulated B cells/cytoskeleton/not touching/cell012 seeds.png]

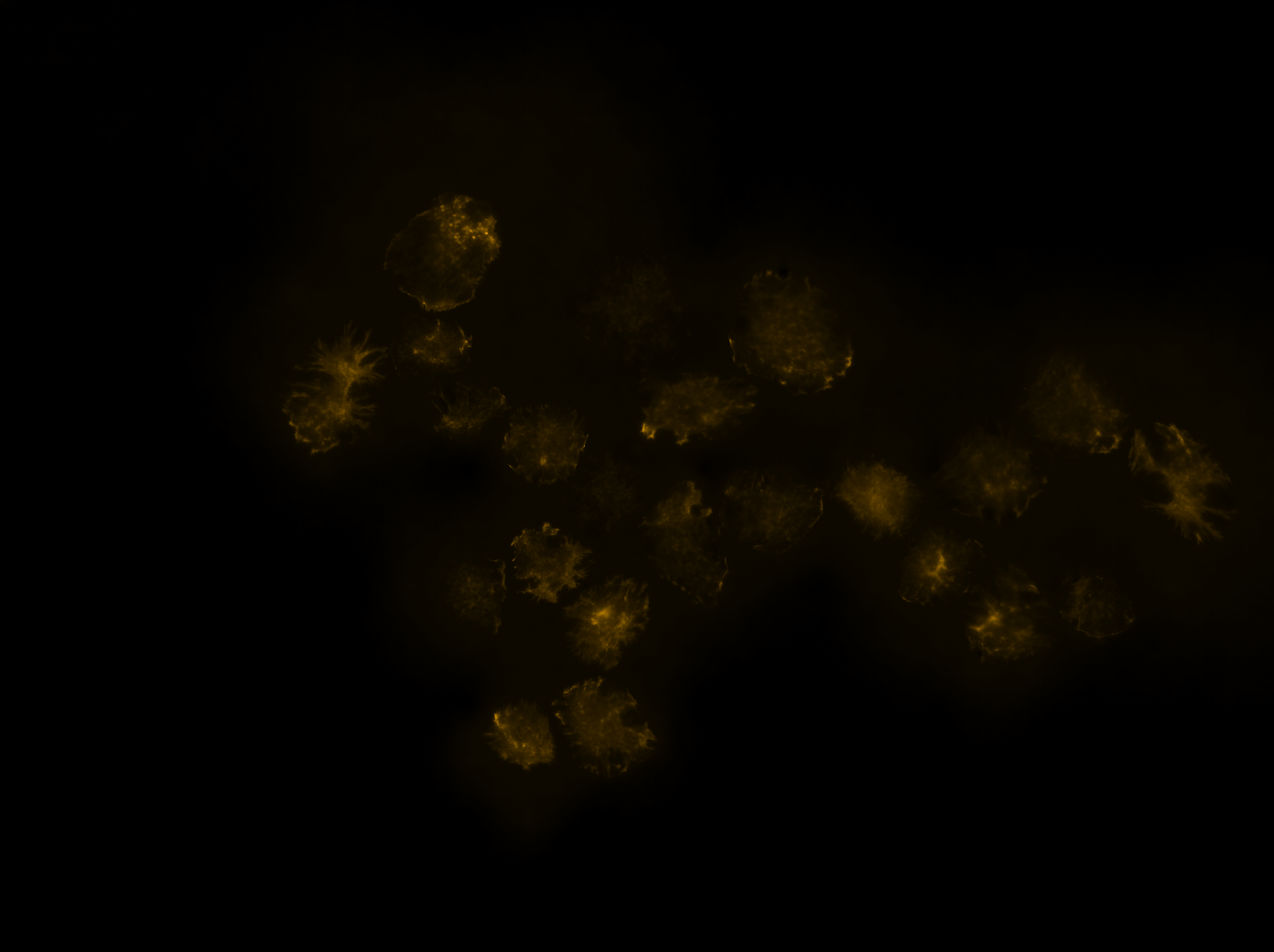

Supplement: Additional file 6 — The zip archive contains simulated images showing B cell nuclei and cytoskeleton with corresponding ground truth. (ZIP 119808 kb) [file 12859_2017_1591_MOESM6_ESM.zip › simulated B cells/cytoskeleton/not touching/cell012.png]

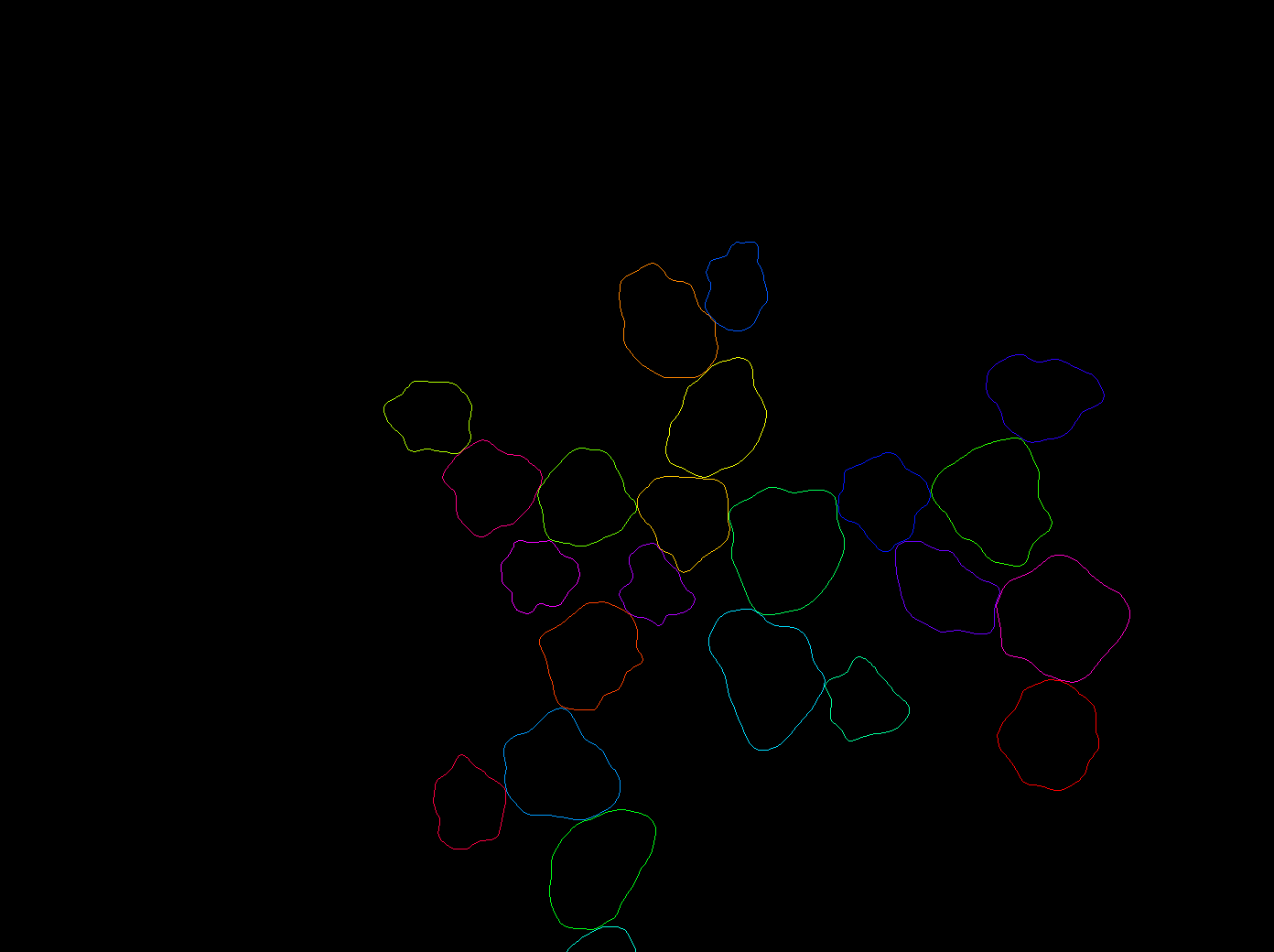

Supplement: Additional file 6 — The zip archive contains simulated images showing B cell nuclei and cytoskeleton with corresponding ground truth. (ZIP 119808 kb) [file 12859_2017_1591_MOESM6_ESM.zip › simulated B cells/cytoskeleton/not touching/cell013 gt.png]

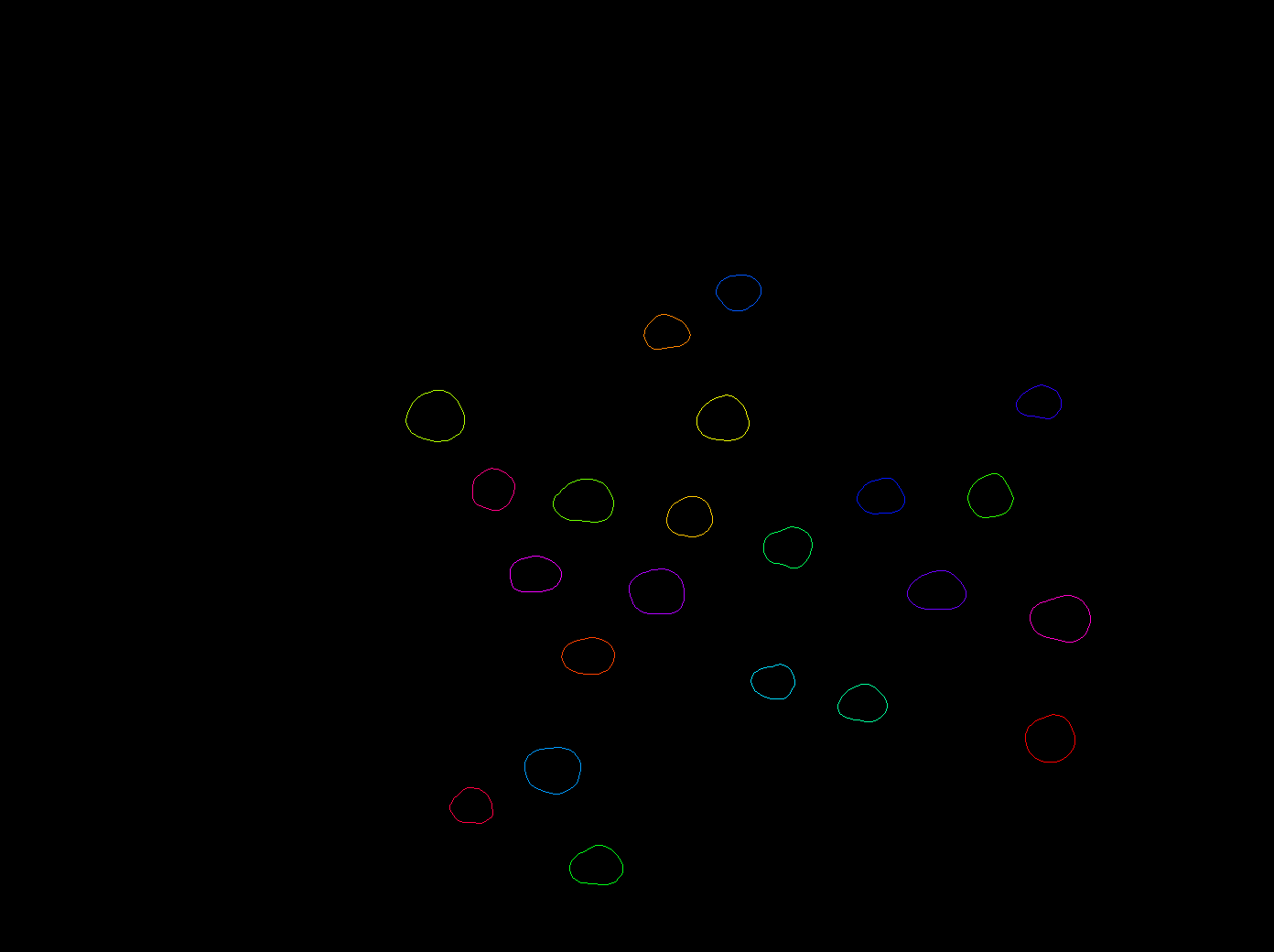

Supplement: Additional file 6 — The zip archive contains simulated images showing B cell nuclei and cytoskeleton with corresponding ground truth. (ZIP 119808 kb) [file 12859_2017_1591_MOESM6_ESM.zip › simulated B cells/cytoskeleton/not touching/cell013 seeds.png]

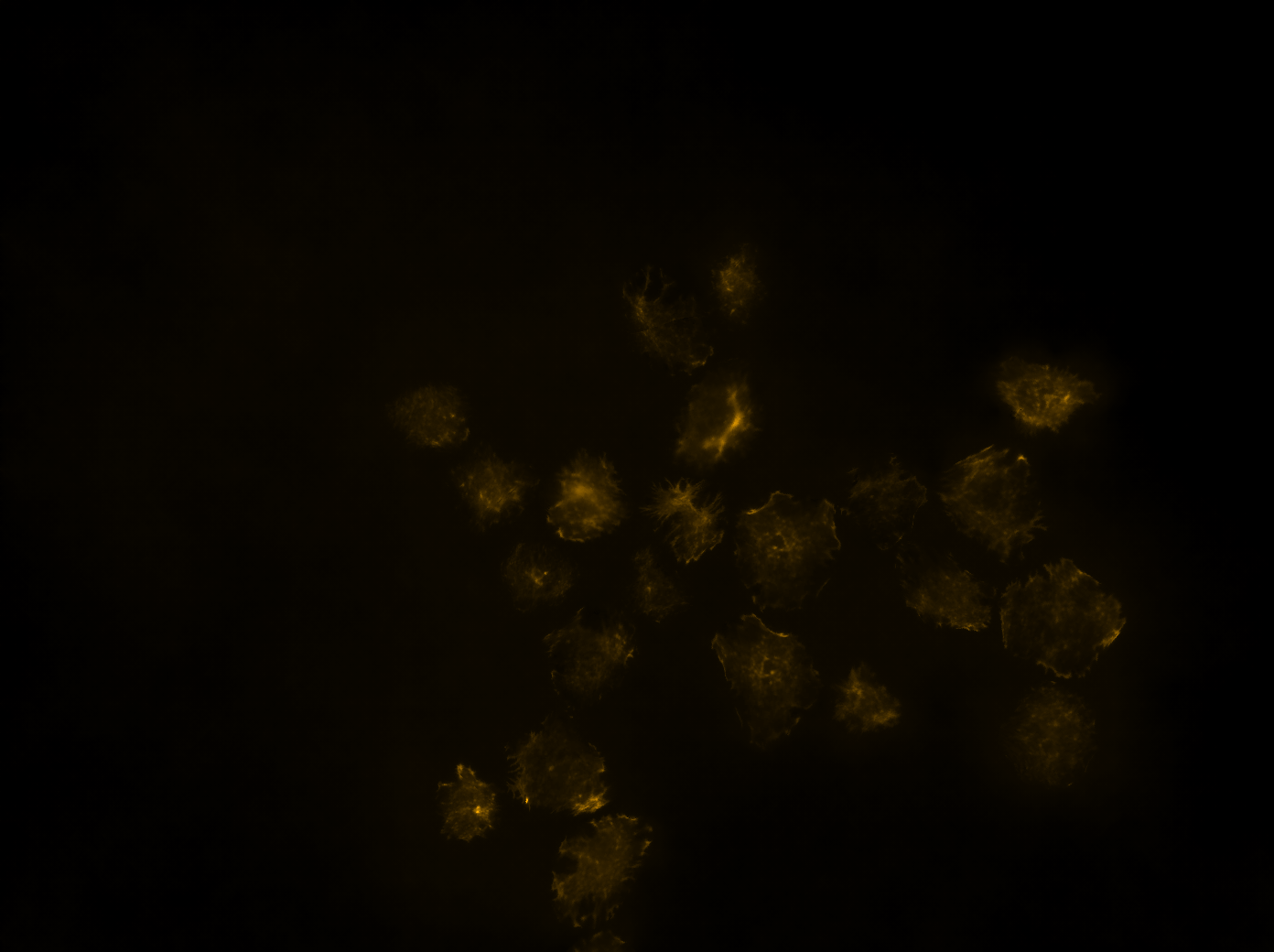

Supplement: Additional file 6 — The zip archive contains simulated images showing B cell nuclei and cytoskeleton with corresponding ground truth. (ZIP 119808 kb) [file 12859_2017_1591_MOESM6_ESM.zip › simulated B cells/cytoskeleton/not touching/cell013.png]

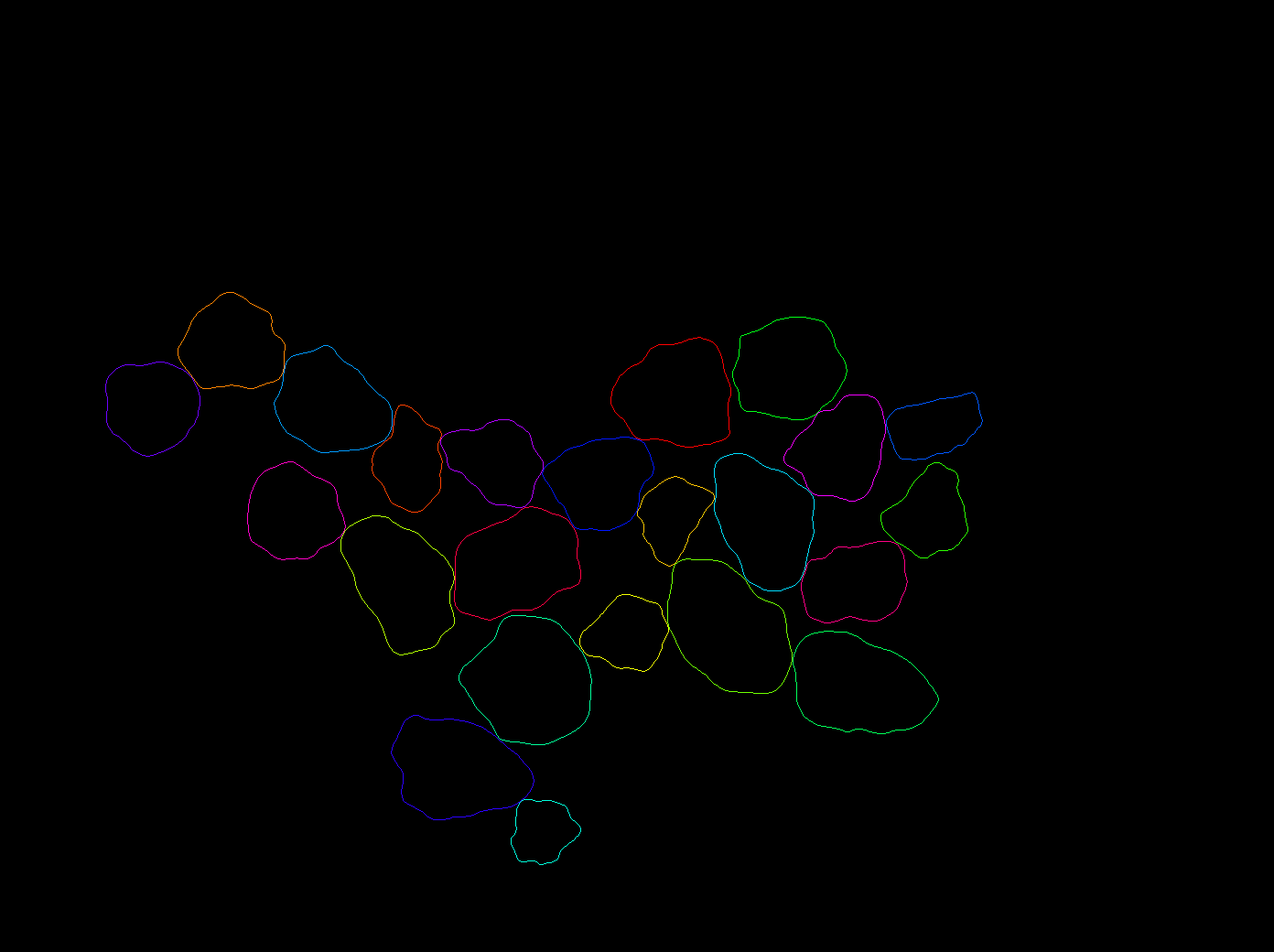

Supplement: Additional file 6 — The zip archive contains simulated images showing B cell nuclei and cytoskeleton with corresponding ground truth. (ZIP 119808 kb) [file 12859_2017_1591_MOESM6_ESM.zip › simulated B cells/cytoskeleton/not touching/cell014 gt.png]

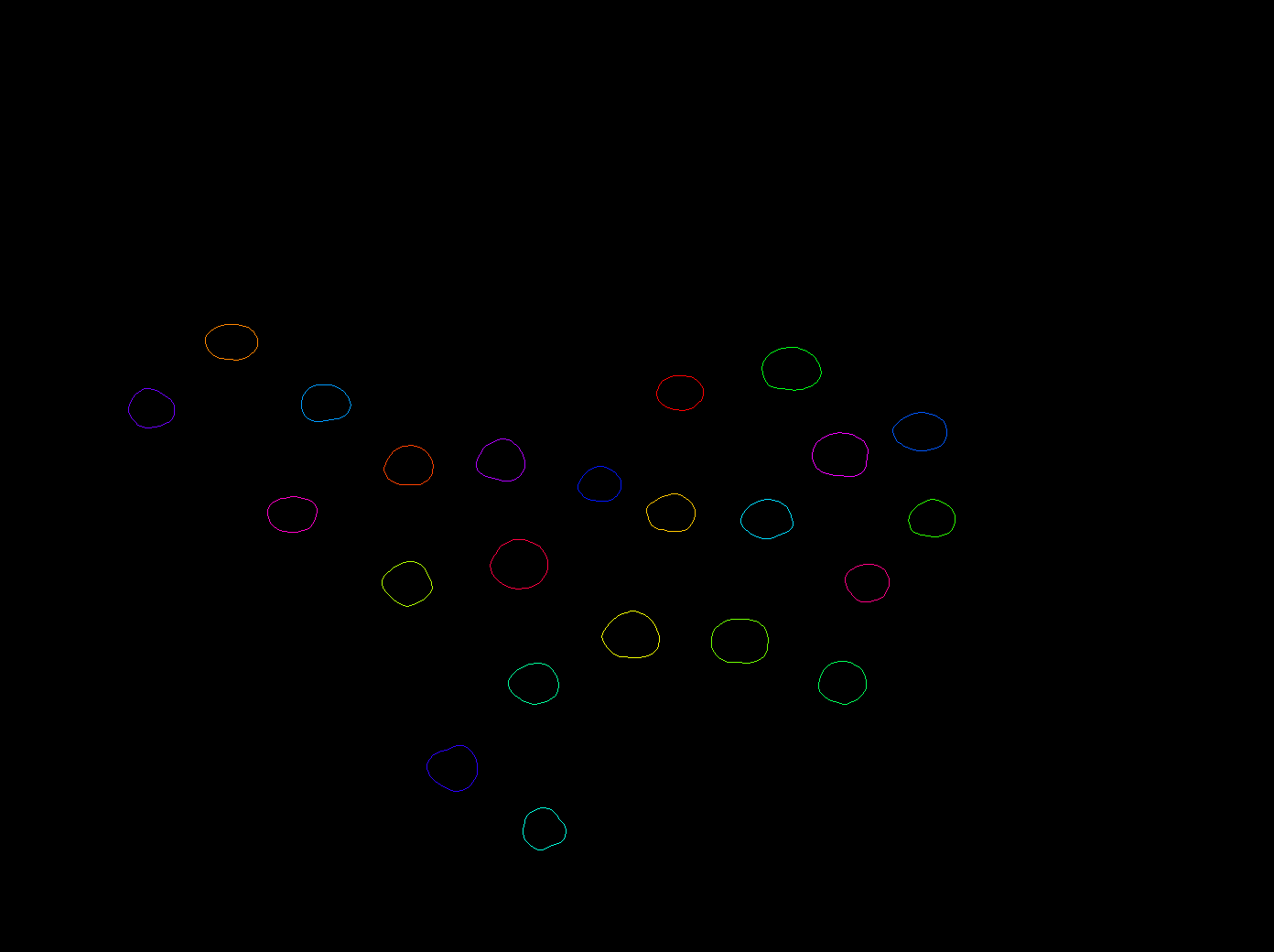

Supplement: Additional file 6 — The zip archive contains simulated images showing B cell nuclei and cytoskeleton with corresponding ground truth. (ZIP 119808 kb) [file 12859_2017_1591_MOESM6_ESM.zip › simulated B cells/cytoskeleton/not touching/cell014 seeds.png]
