# Supplementary figures and images for: Using simulated fluorescence cell micrographs for the evaluation of cell image segmentation algorithms (part 3 of 6)
Source: BMC Bioinformatics. 2017 Mar 18;18:176. doi: 10.1186/s12859-017-1591-2 (PMC5357336; doi:10.1186/s12859-017-1591-2)

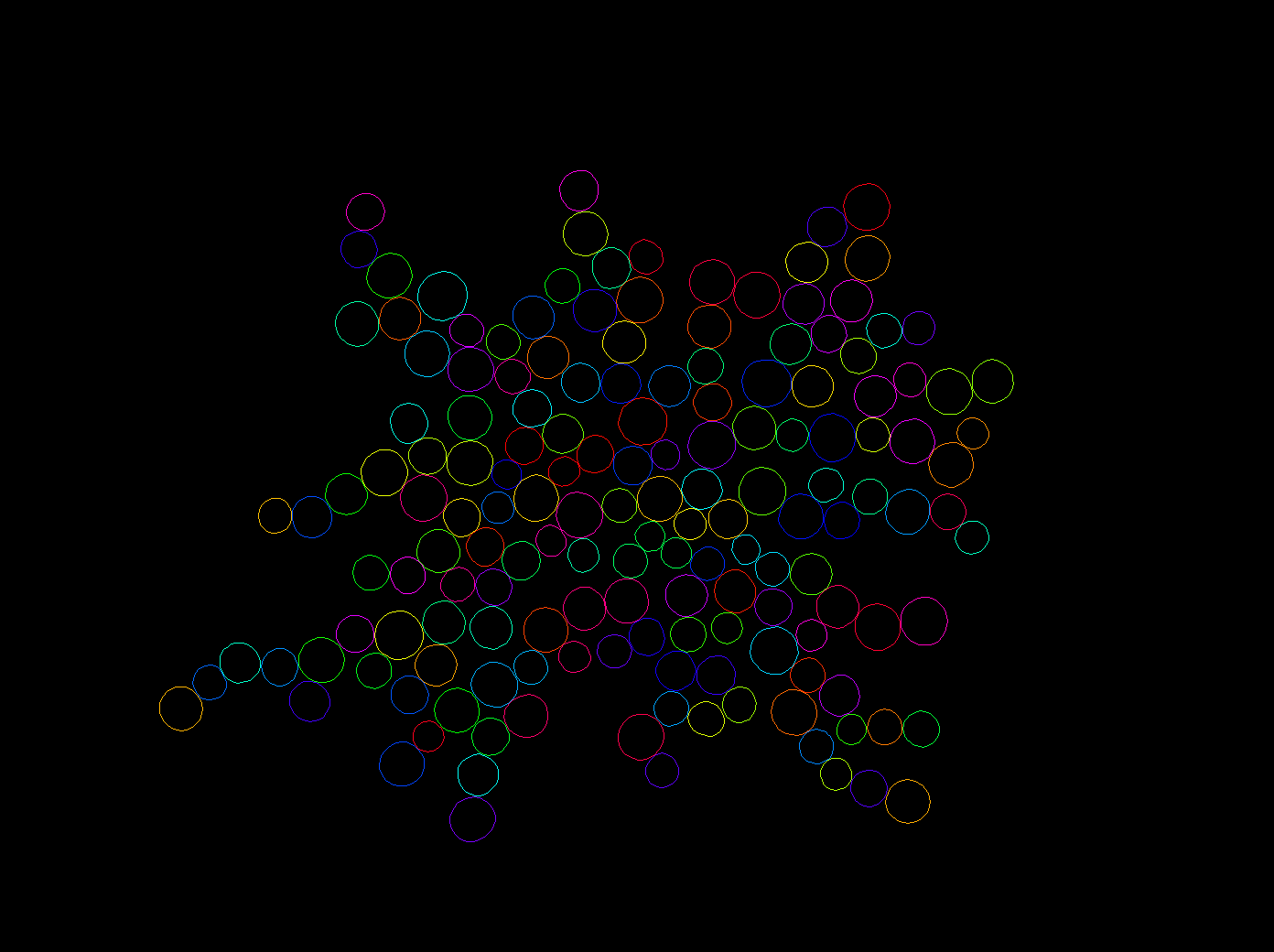

Supplement: Additional file 5 — The zip archive contains simulated images showing protoplasts with corresponding ground truth. (ZIP 72704 kb) [file 12859_2017_1591_MOESM5_ESM.zip › simulated protoplasts/nottouching/nottouching021 gt.png]

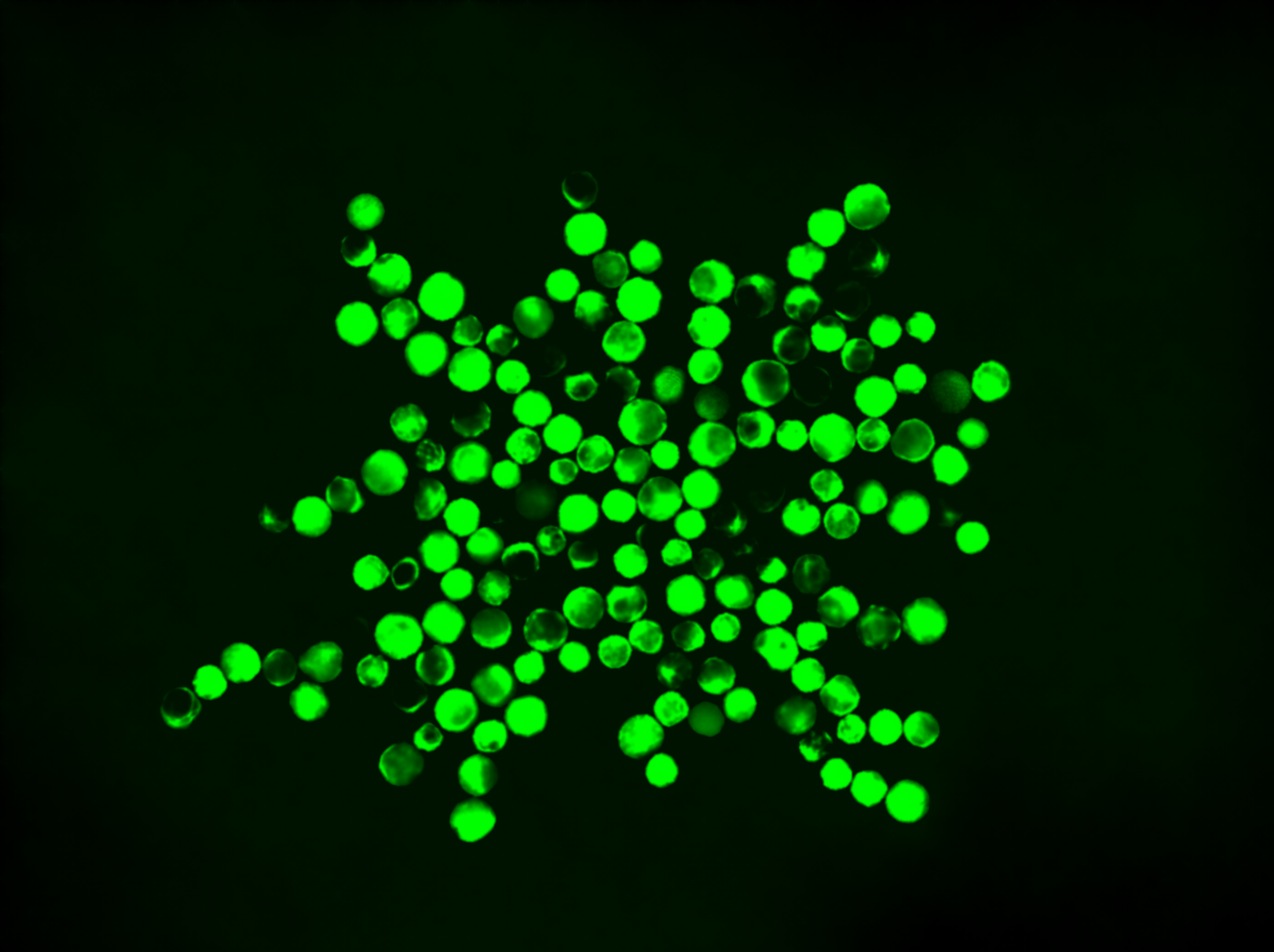

Supplement: Additional file 5 — The zip archive contains simulated images showing protoplasts with corresponding ground truth. (ZIP 72704 kb) [file 12859_2017_1591_MOESM5_ESM.zip › simulated protoplasts/nottouching/nottouching021.png]

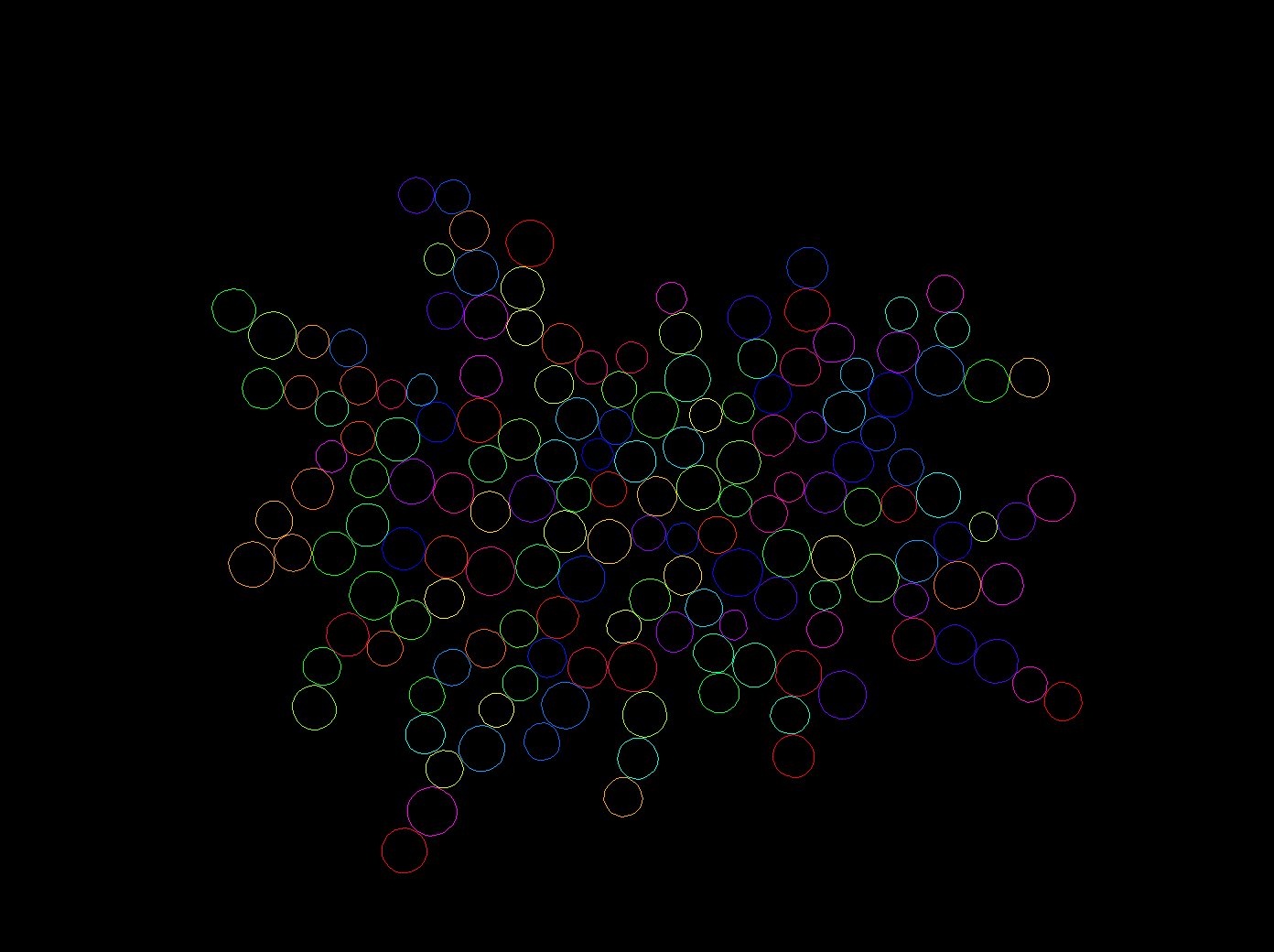

Supplement: Additional file 5 — The zip archive contains simulated images showing protoplasts with corresponding ground truth. (ZIP 72704 kb) [file 12859_2017_1591_MOESM5_ESM.zip › simulated protoplasts/nottouching/nottouching022 gt.png]

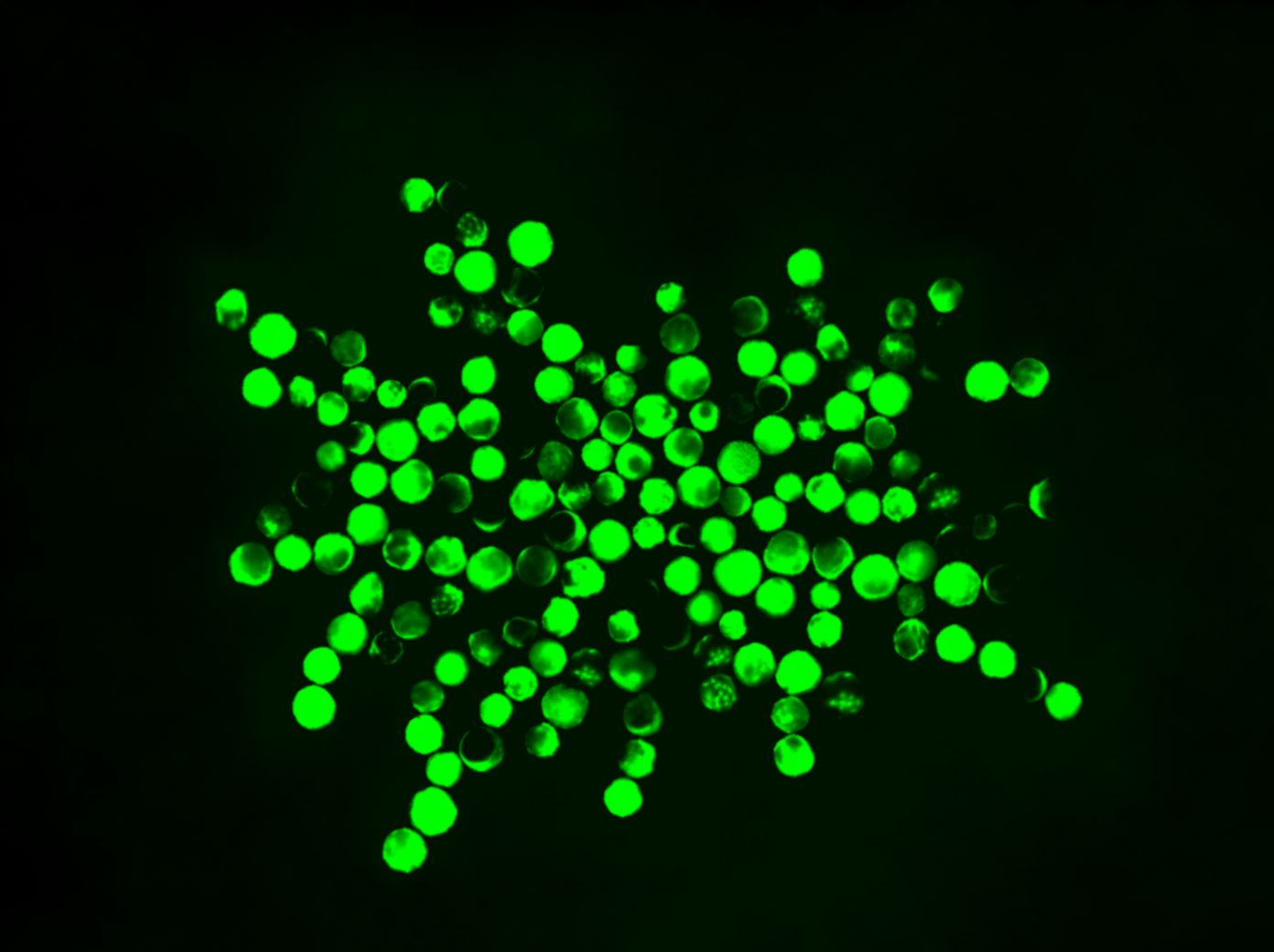

Supplement: Additional file 5 — The zip archive contains simulated images showing protoplasts with corresponding ground truth. (ZIP 72704 kb) [file 12859_2017_1591_MOESM5_ESM.zip › simulated protoplasts/nottouching/nottouching022.png]

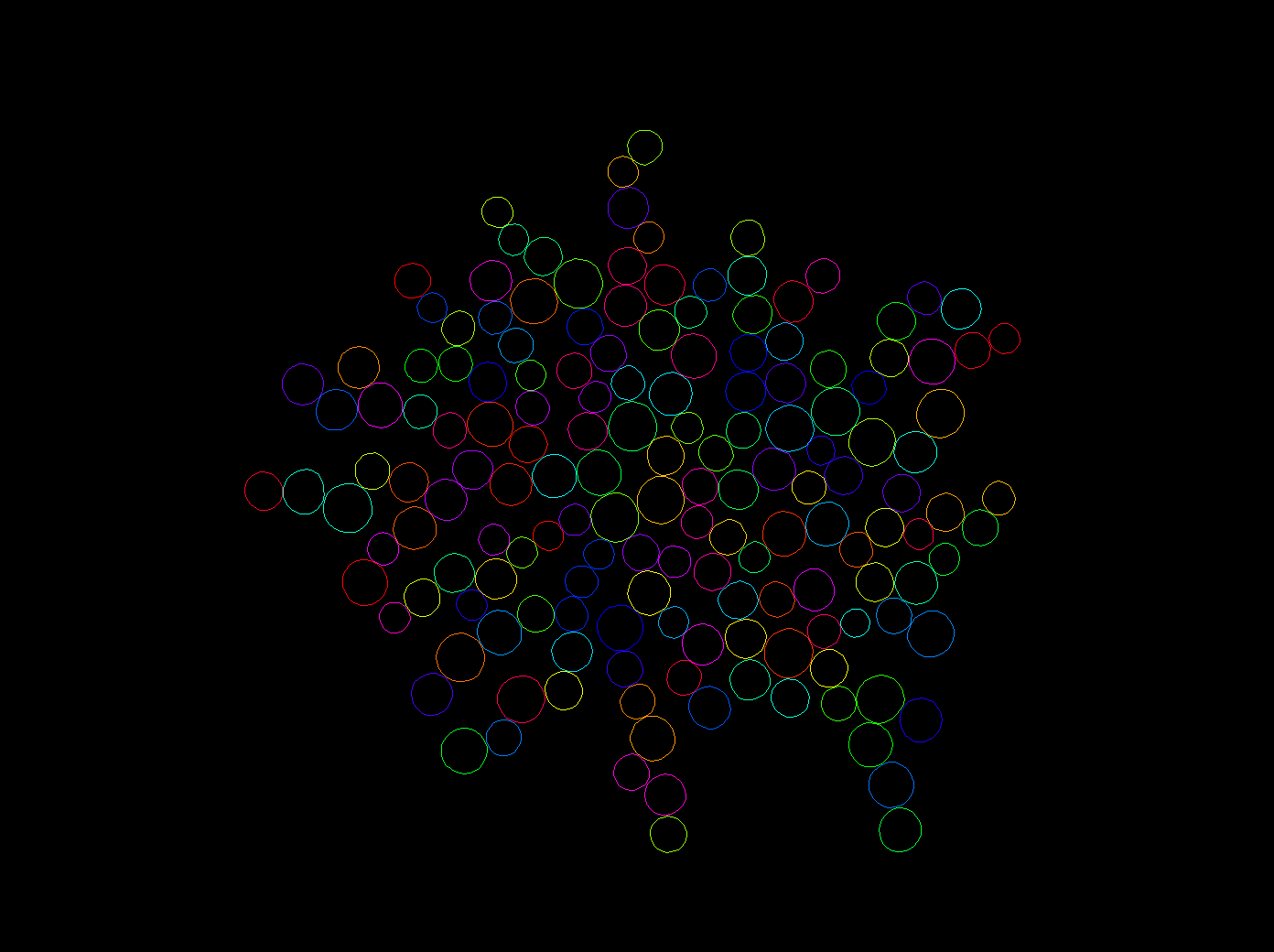

Supplement: Additional file 5 — The zip archive contains simulated images showing protoplasts with corresponding ground truth. (ZIP 72704 kb) [file 12859_2017_1591_MOESM5_ESM.zip › simulated protoplasts/nottouching/nottouching023 gt.png]

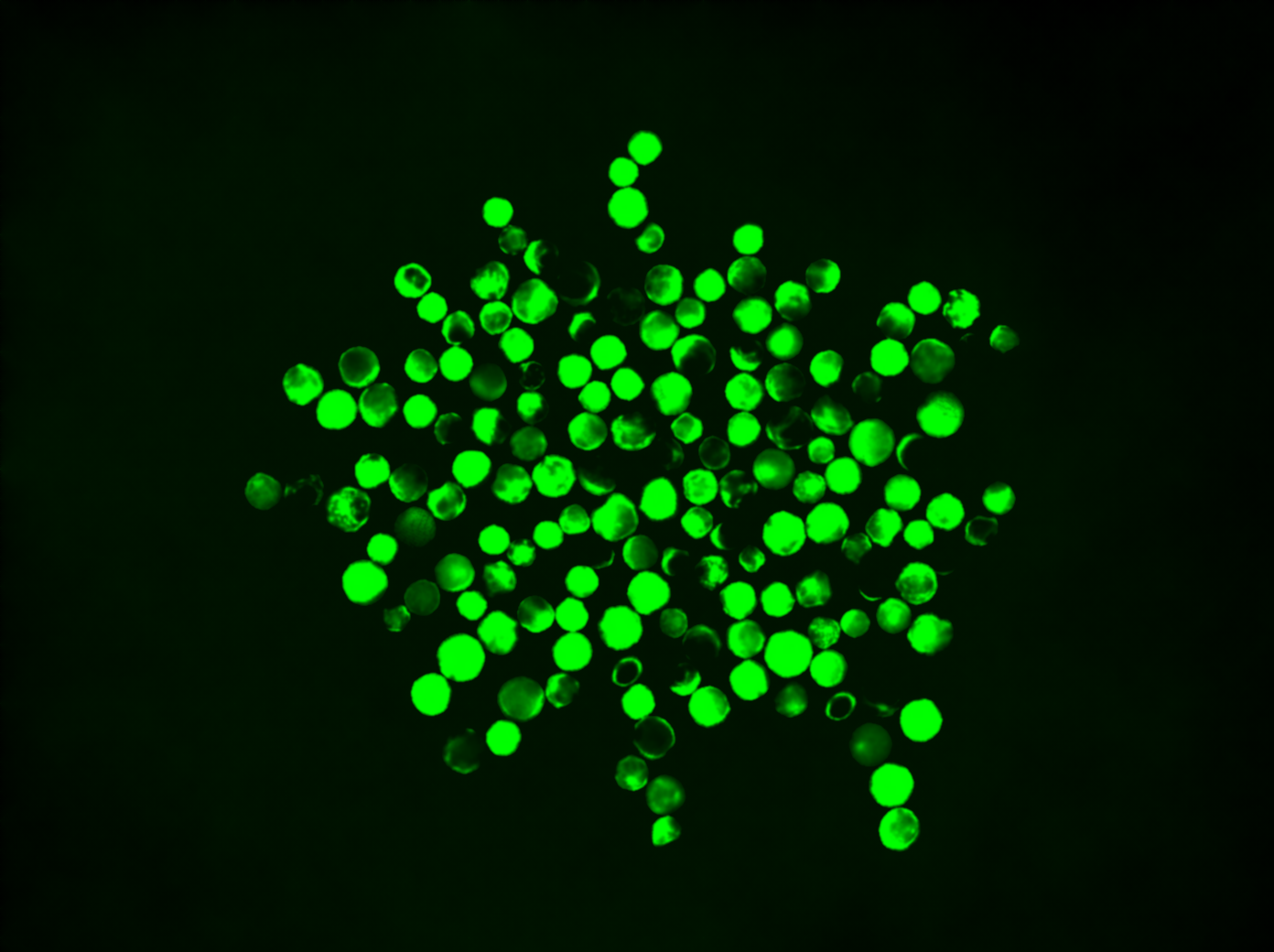

Supplement: Additional file 5 — The zip archive contains simulated images showing protoplasts with corresponding ground truth. (ZIP 72704 kb) [file 12859_2017_1591_MOESM5_ESM.zip › simulated protoplasts/nottouching/nottouching023.png]

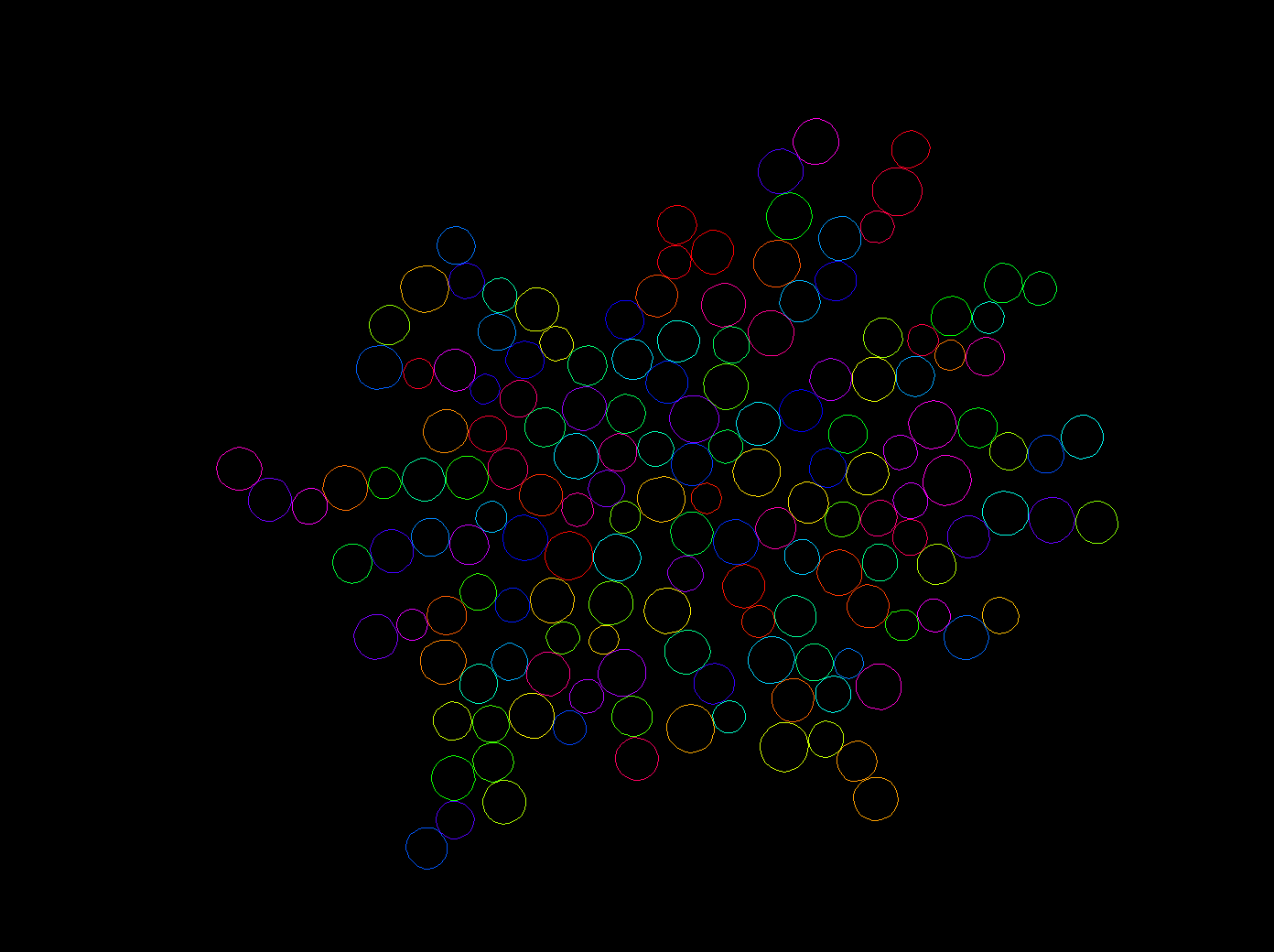

Supplement: Additional file 5 — The zip archive contains simulated images showing protoplasts with corresponding ground truth. (ZIP 72704 kb) [file 12859_2017_1591_MOESM5_ESM.zip › simulated protoplasts/nottouching/nottouching024 gt.png]

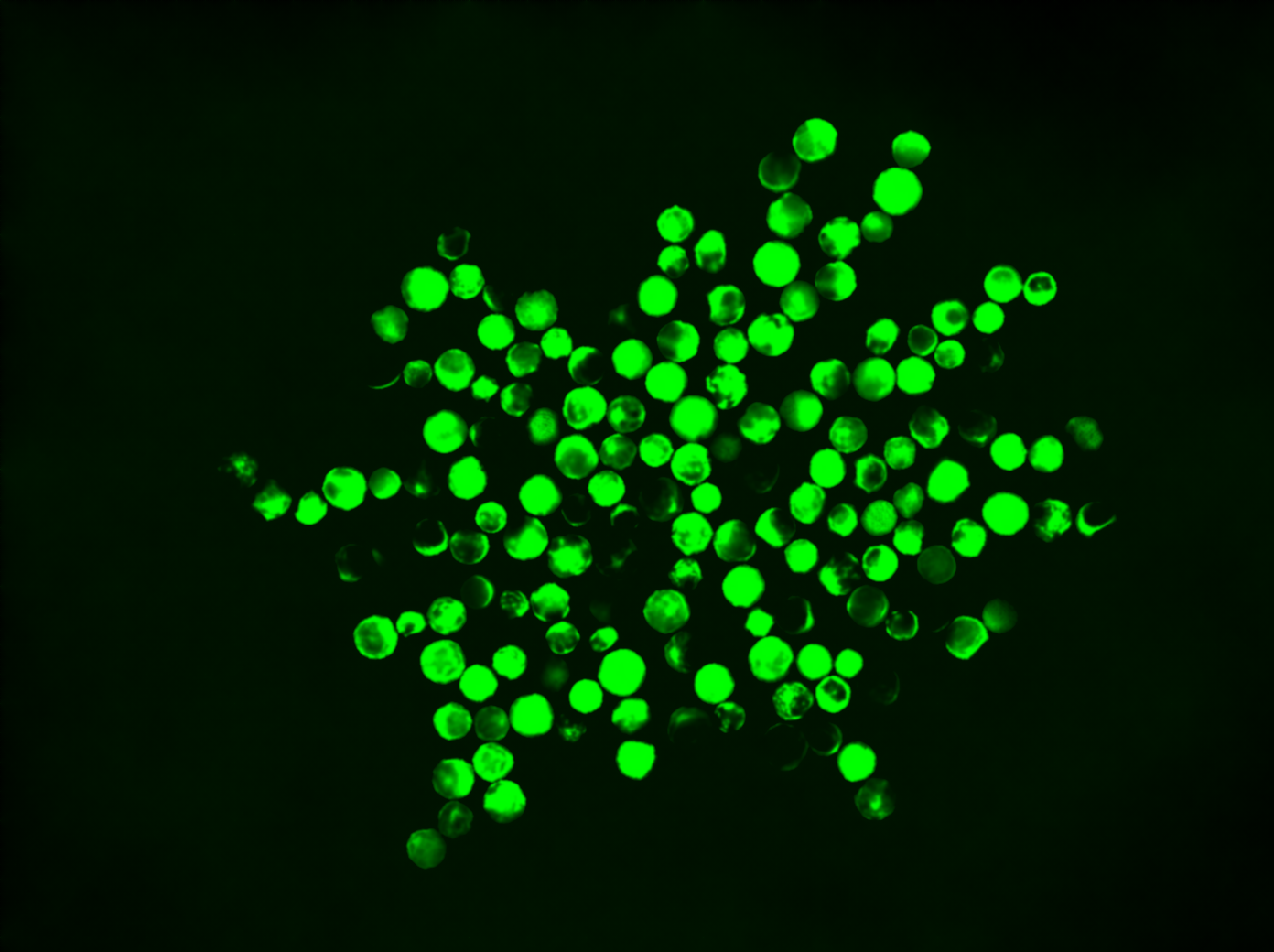

Supplement: Additional file 5 — The zip archive contains simulated images showing protoplasts with corresponding ground truth. (ZIP 72704 kb) [file 12859_2017_1591_MOESM5_ESM.zip › simulated protoplasts/nottouching/nottouching024.png]

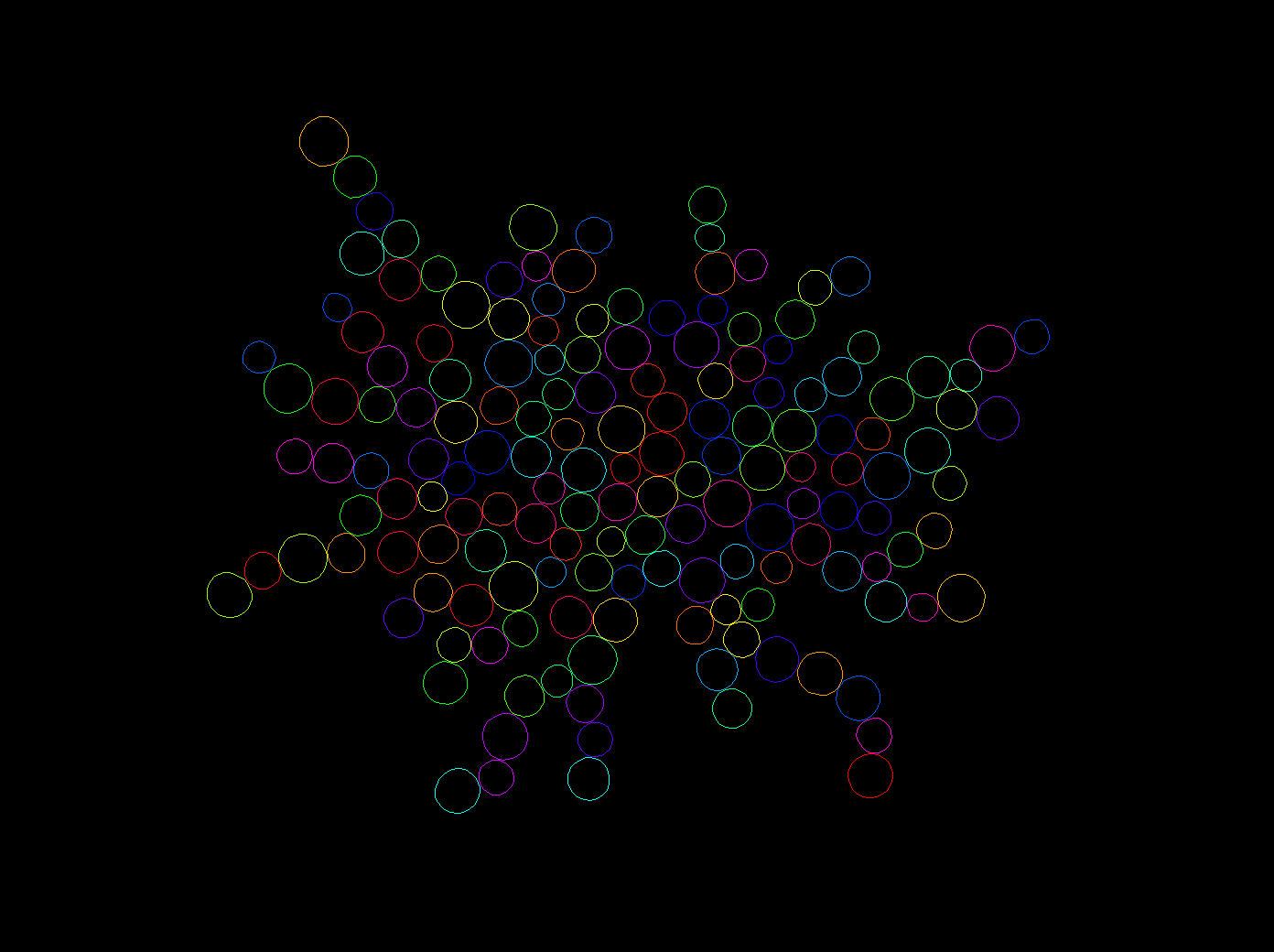

Supplement: Additional file 5 — The zip archive contains simulated images showing protoplasts with corresponding ground truth. (ZIP 72704 kb) [file 12859_2017_1591_MOESM5_ESM.zip › simulated protoplasts/nottouching/nottouching025 gt.png]

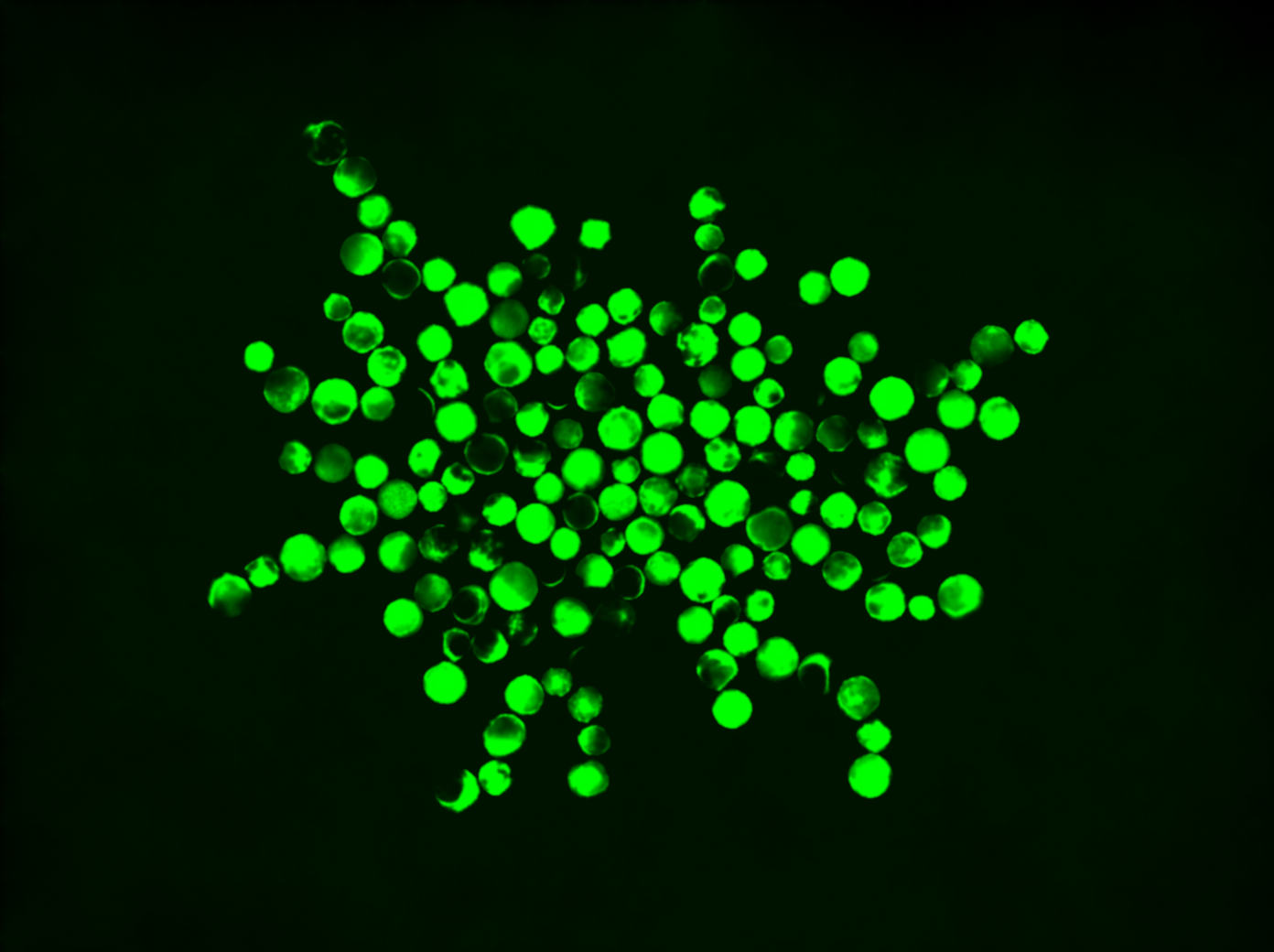

Supplement: Additional file 5 — The zip archive contains simulated images showing protoplasts with corresponding ground truth. (ZIP 72704 kb) [file 12859_2017_1591_MOESM5_ESM.zip › simulated protoplasts/nottouching/nottouching025.png]

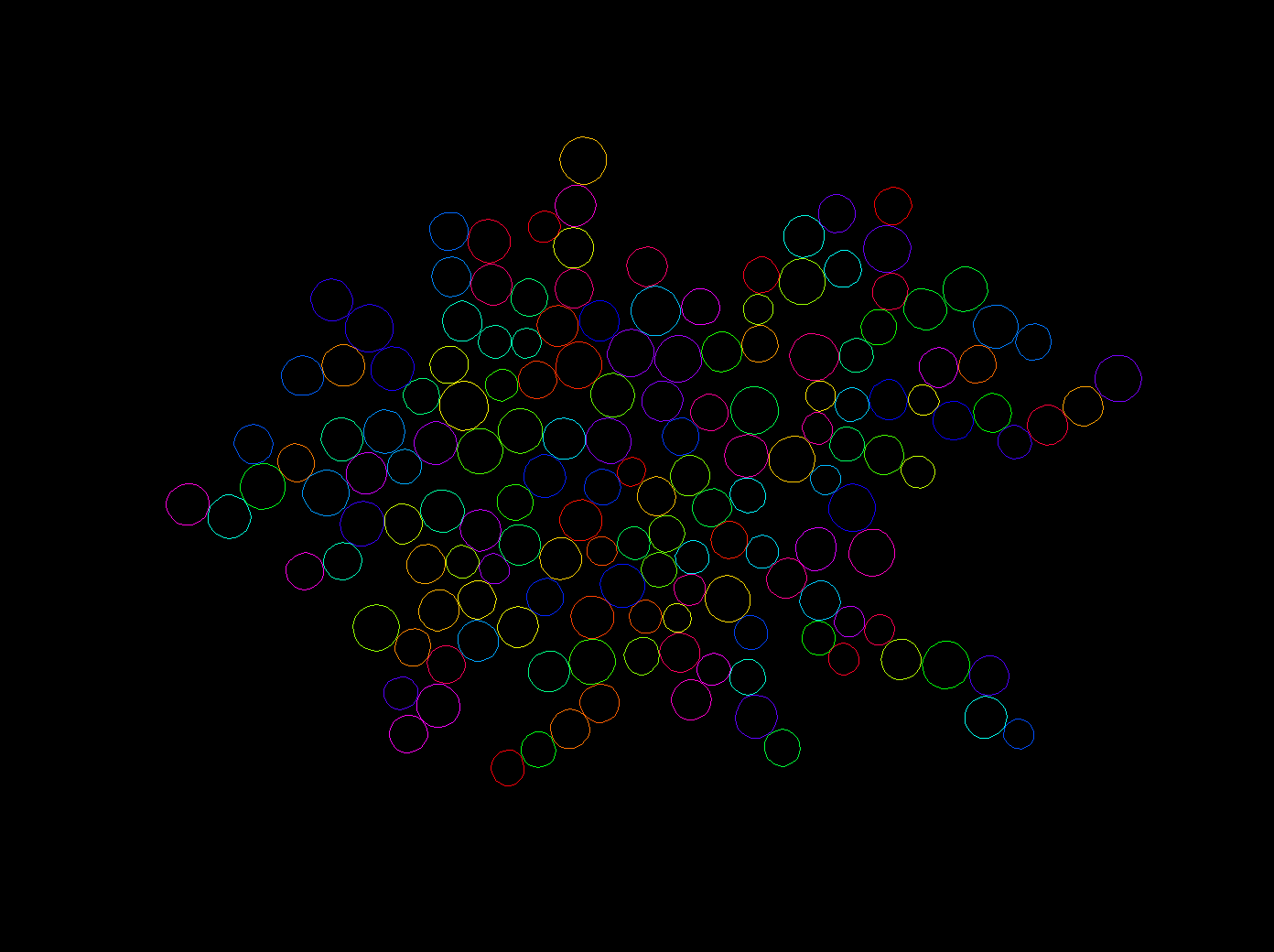

Supplement: Additional file 5 — The zip archive contains simulated images showing protoplasts with corresponding ground truth. (ZIP 72704 kb) [file 12859_2017_1591_MOESM5_ESM.zip › simulated protoplasts/nottouching/nottouching026 gt.png]

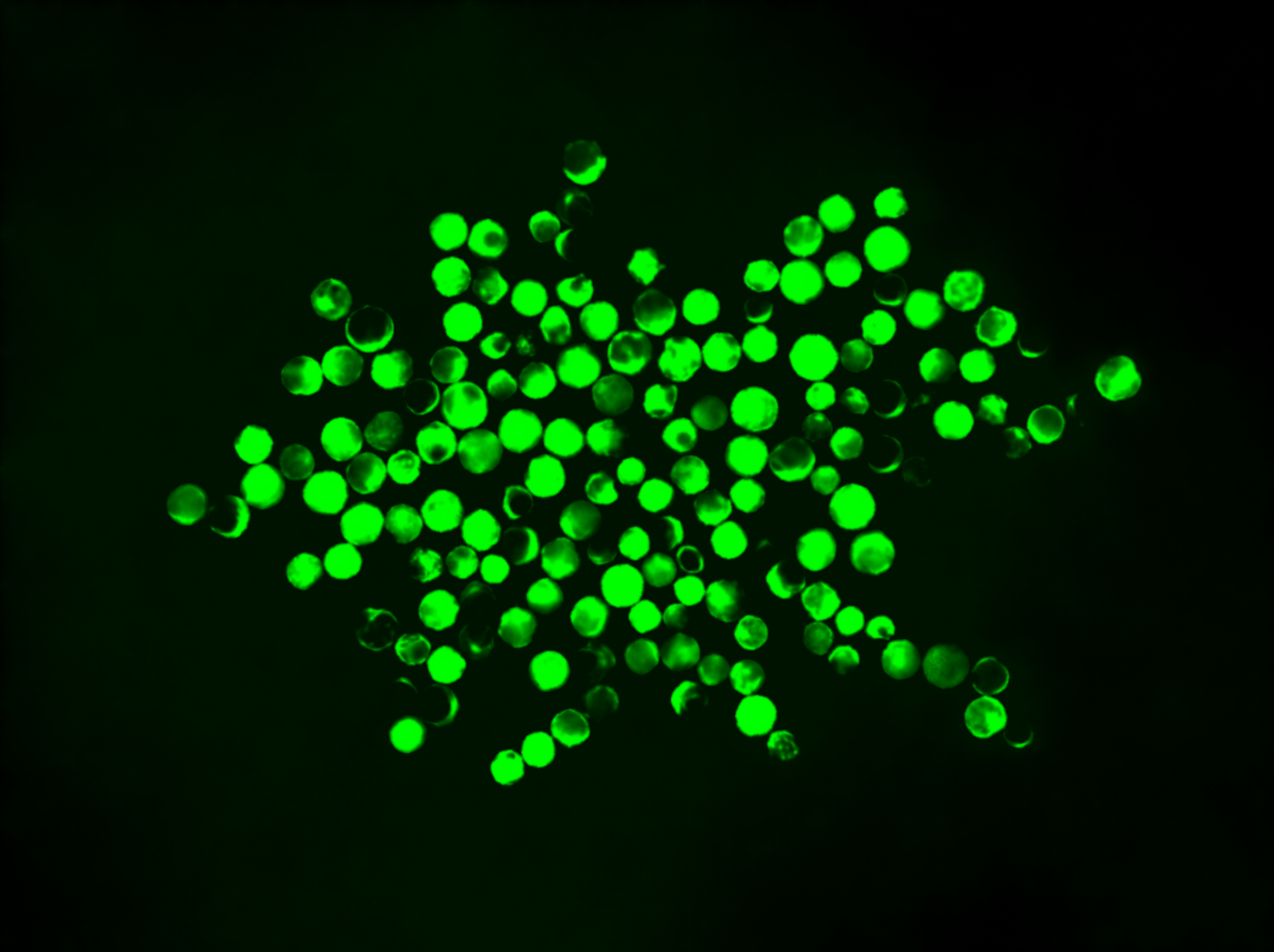

Supplement: Additional file 5 — The zip archive contains simulated images showing protoplasts with corresponding ground truth. (ZIP 72704 kb) [file 12859_2017_1591_MOESM5_ESM.zip › simulated protoplasts/nottouching/nottouching026.png]

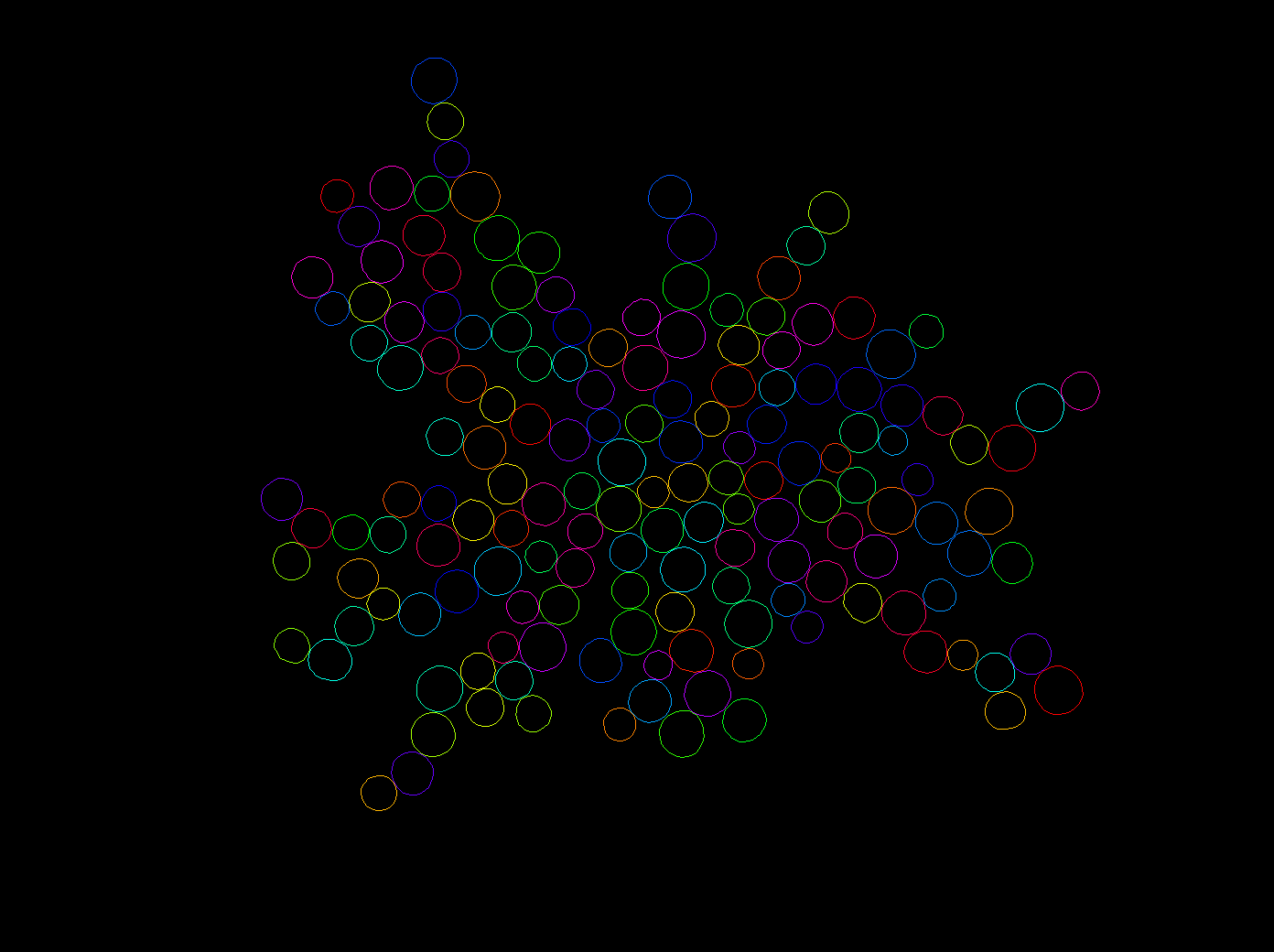

Supplement: Additional file 5 — The zip archive contains simulated images showing protoplasts with corresponding ground truth. (ZIP 72704 kb) [file 12859_2017_1591_MOESM5_ESM.zip › simulated protoplasts/nottouching/nottouching027 gt.png]

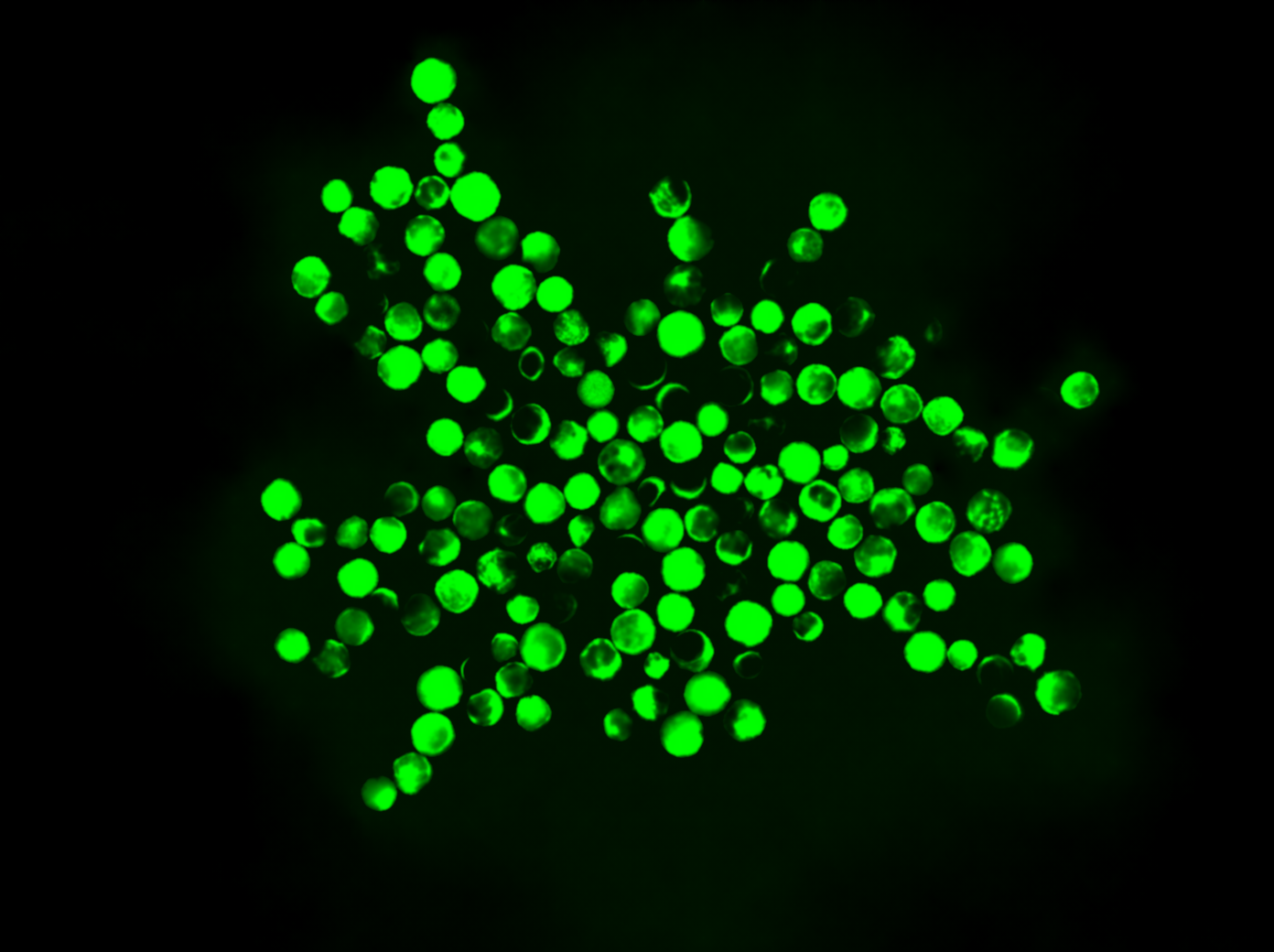

Supplement: Additional file 5 — The zip archive contains simulated images showing protoplasts with corresponding ground truth. (ZIP 72704 kb) [file 12859_2017_1591_MOESM5_ESM.zip › simulated protoplasts/nottouching/nottouching027.png]

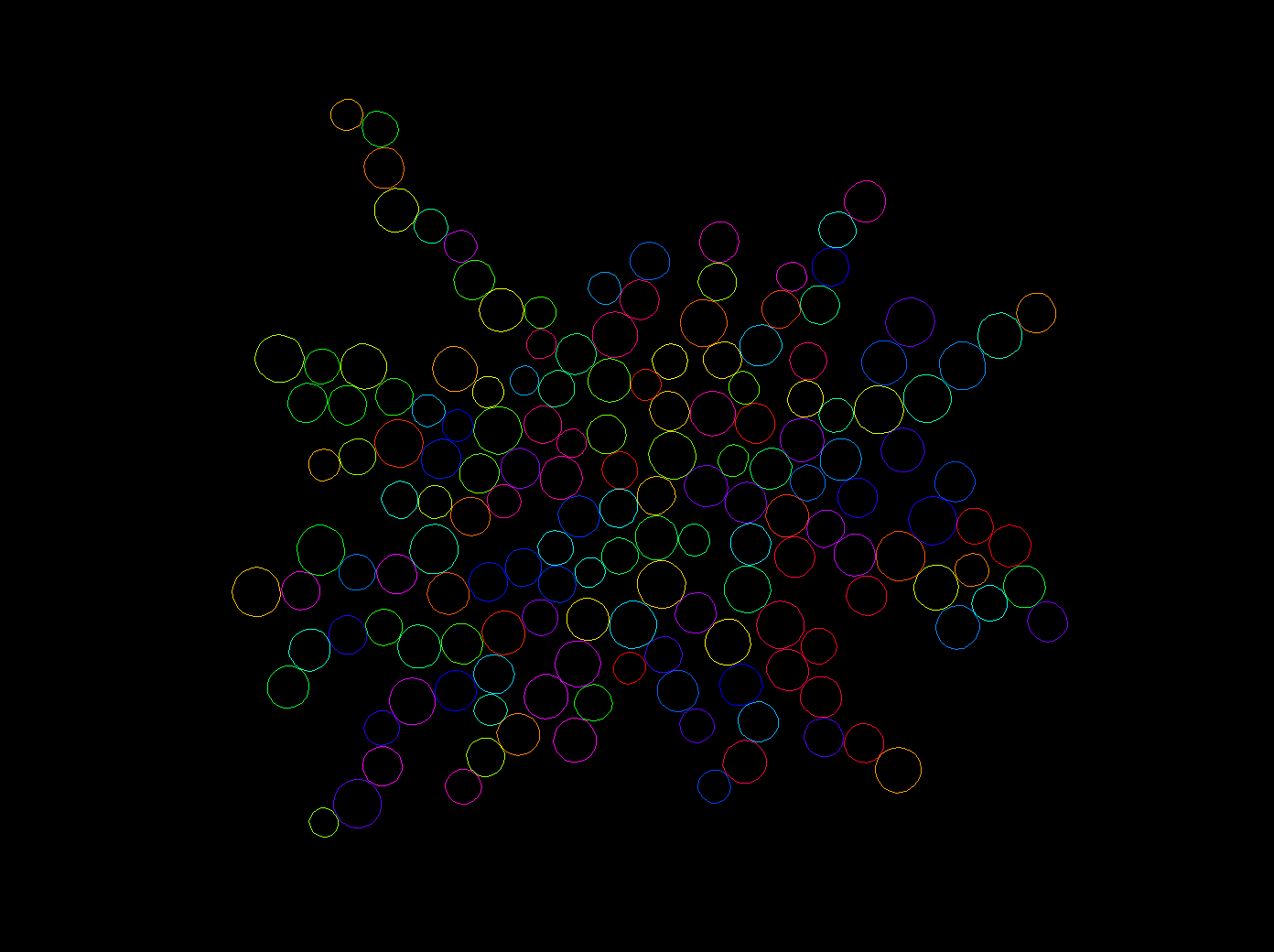

Supplement: Additional file 5 — The zip archive contains simulated images showing protoplasts with corresponding ground truth. (ZIP 72704 kb) [file 12859_2017_1591_MOESM5_ESM.zip › simulated protoplasts/nottouching/nottouching028 gt.png]

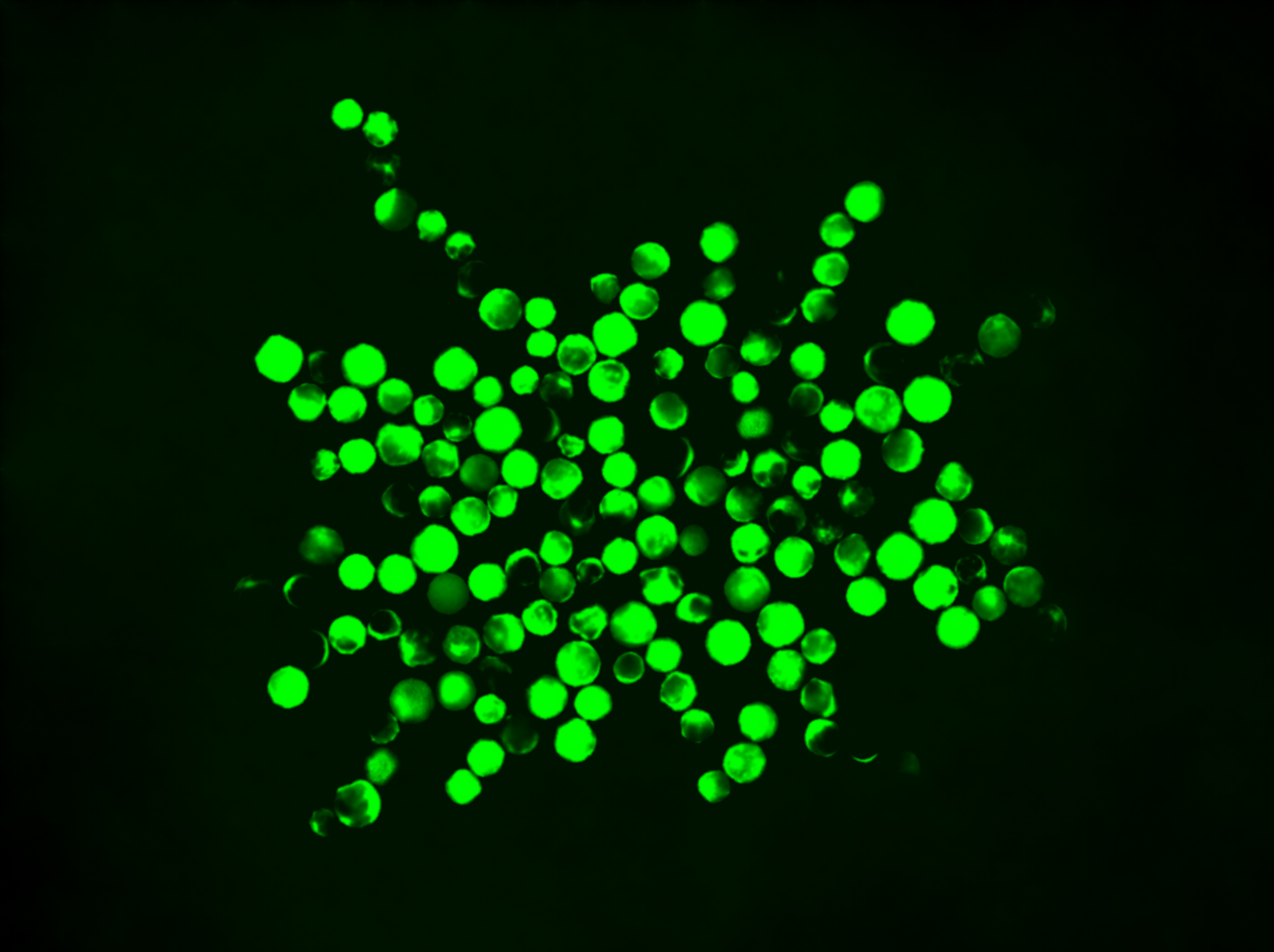

Supplement: Additional file 5 — The zip archive contains simulated images showing protoplasts with corresponding ground truth. (ZIP 72704 kb) [file 12859_2017_1591_MOESM5_ESM.zip › simulated protoplasts/nottouching/nottouching028.png]

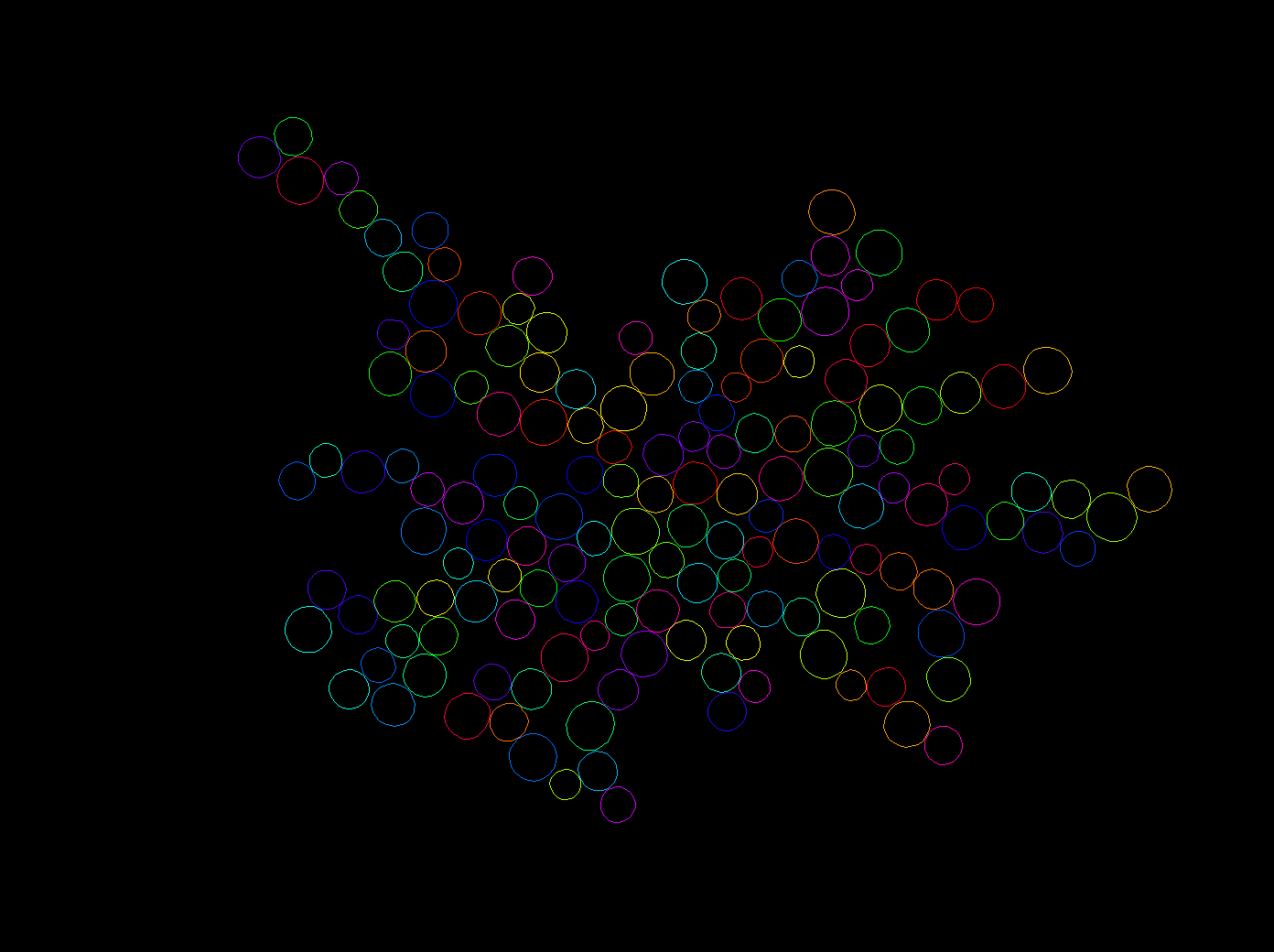

Supplement: Additional file 5 — The zip archive contains simulated images showing protoplasts with corresponding ground truth. (ZIP 72704 kb) [file 12859_2017_1591_MOESM5_ESM.zip › simulated protoplasts/nottouching/nottouching029 gt.png]

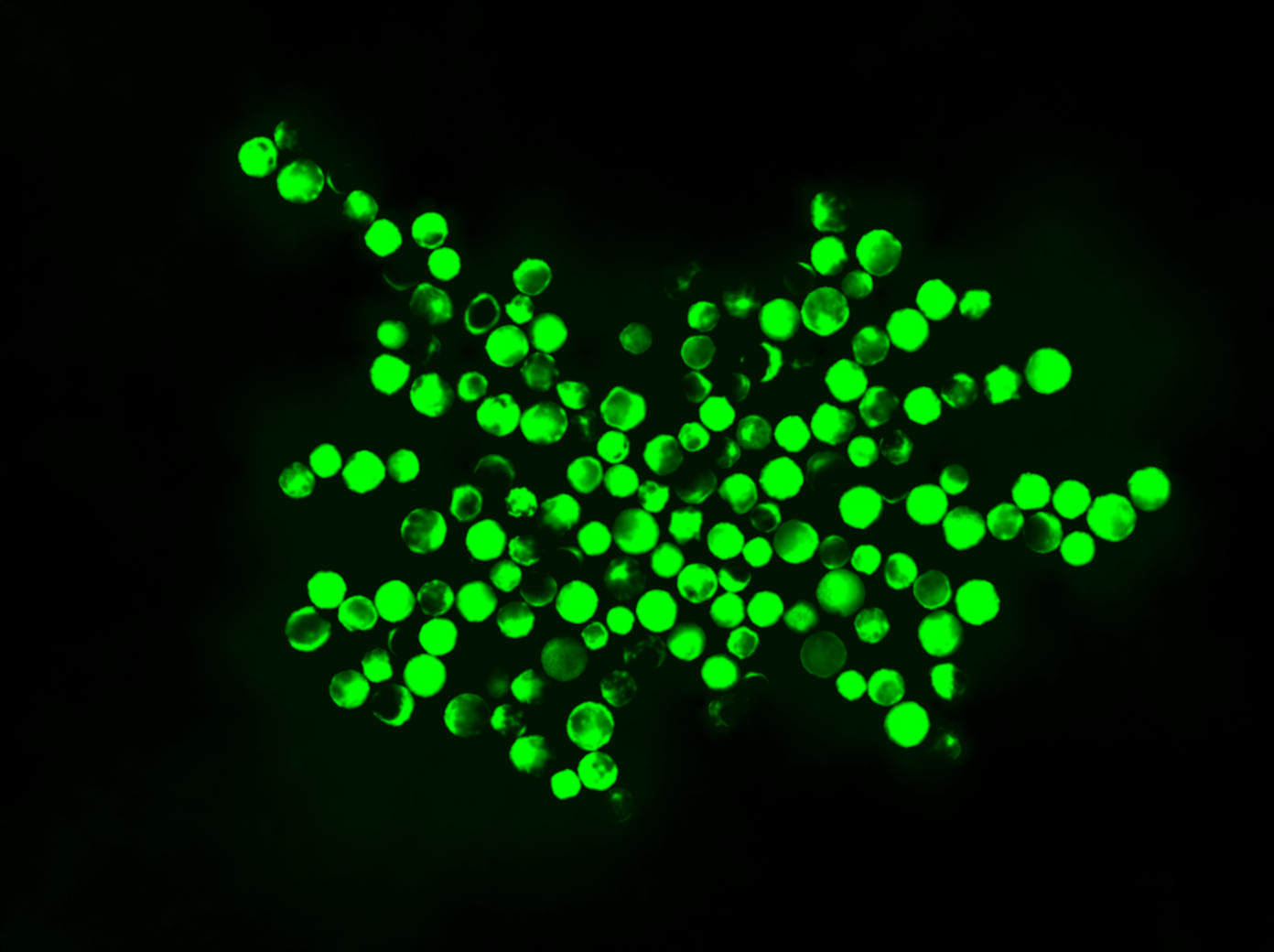

Supplement: Additional file 5 — The zip archive contains simulated images showing protoplasts with corresponding ground truth. (ZIP 72704 kb) [file 12859_2017_1591_MOESM5_ESM.zip › simulated protoplasts/nottouching/nottouching029.png]

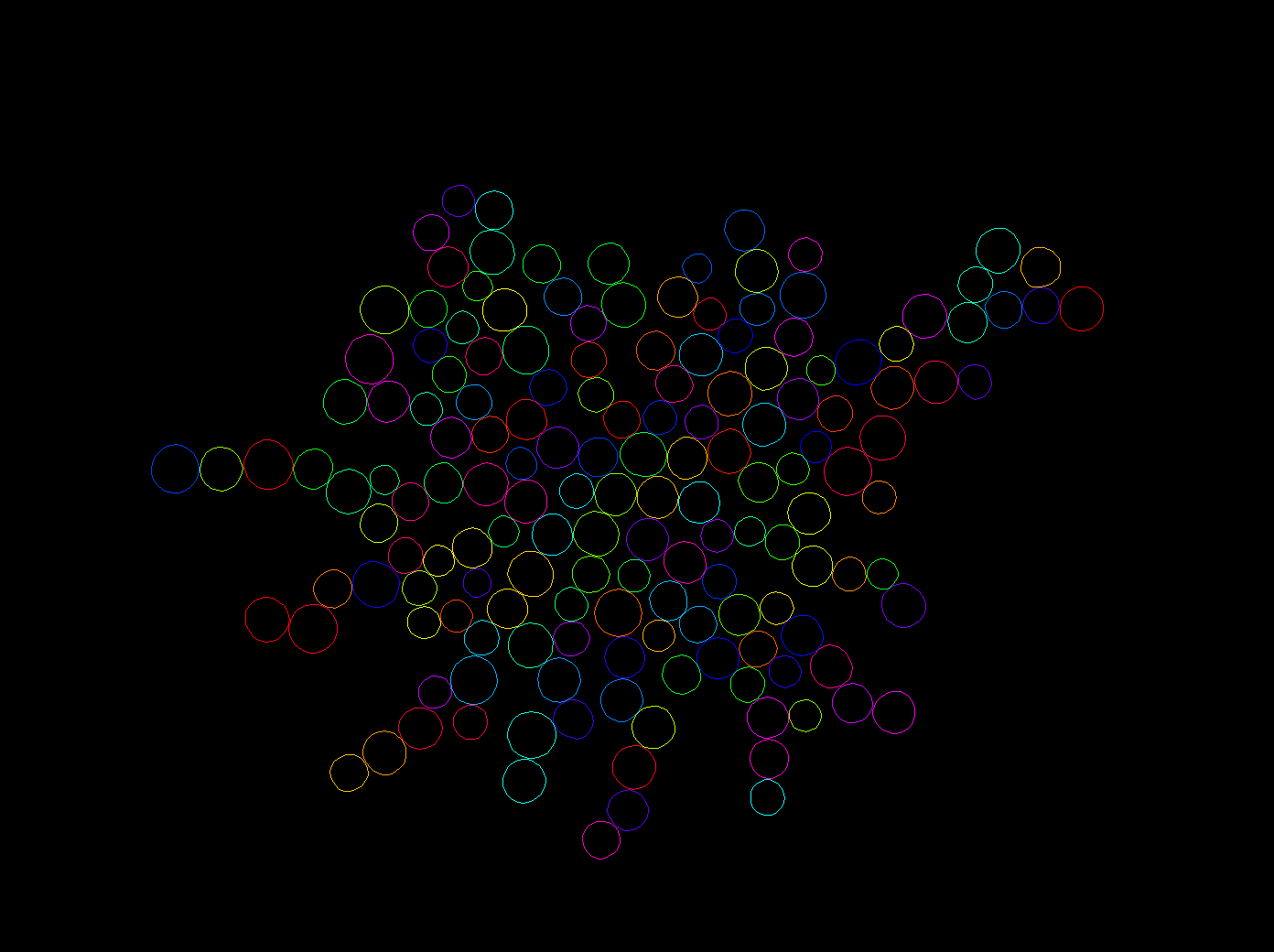

Supplement: Additional file 5 — The zip archive contains simulated images showing protoplasts with corresponding ground truth. (ZIP 72704 kb) [file 12859_2017_1591_MOESM5_ESM.zip › simulated protoplasts/nottouching/nottouching030 gt.png]

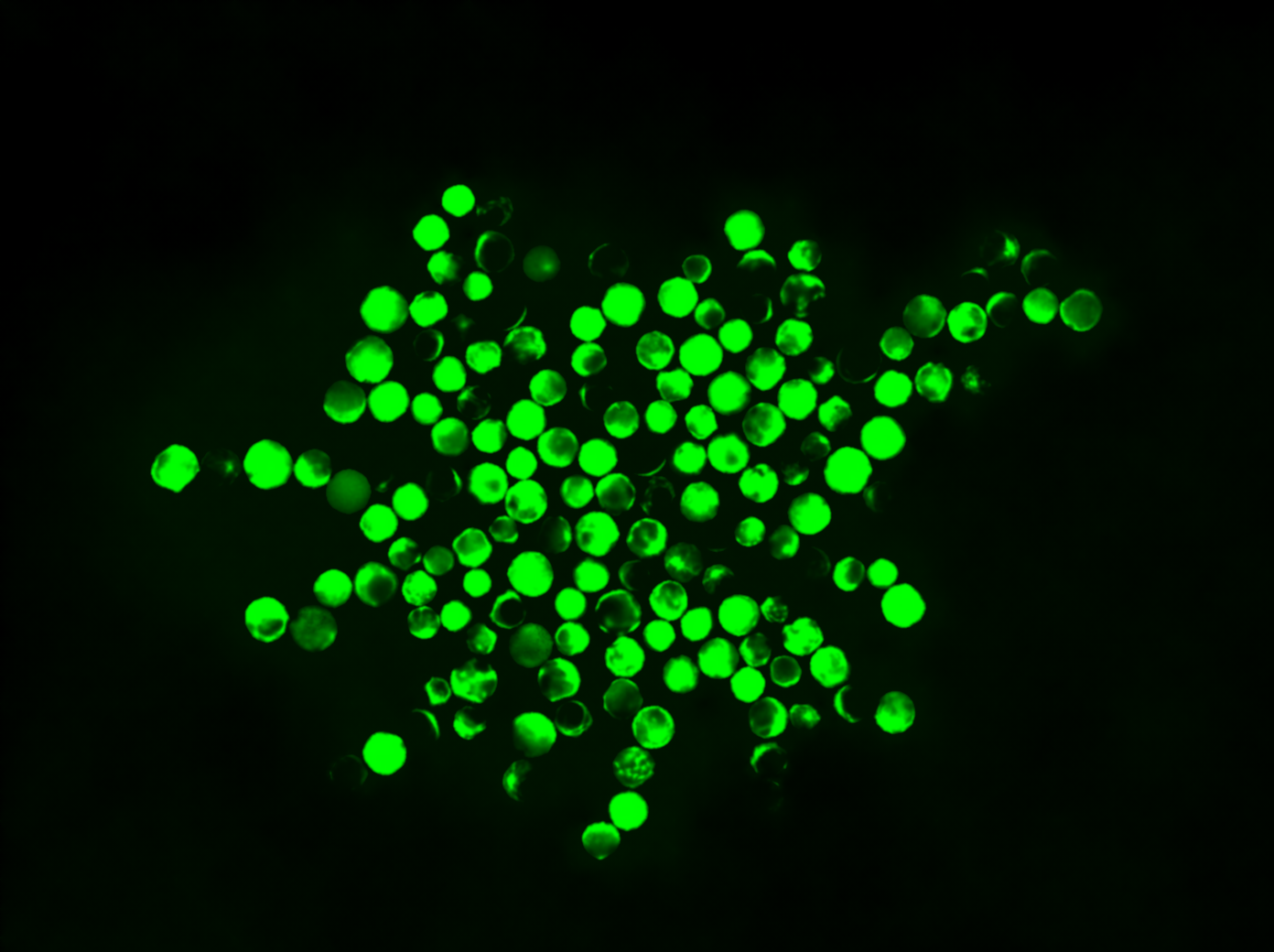

Supplement: Additional file 5 — The zip archive contains simulated images showing protoplasts with corresponding ground truth. (ZIP 72704 kb) [file 12859_2017_1591_MOESM5_ESM.zip › simulated protoplasts/nottouching/nottouching030.png]

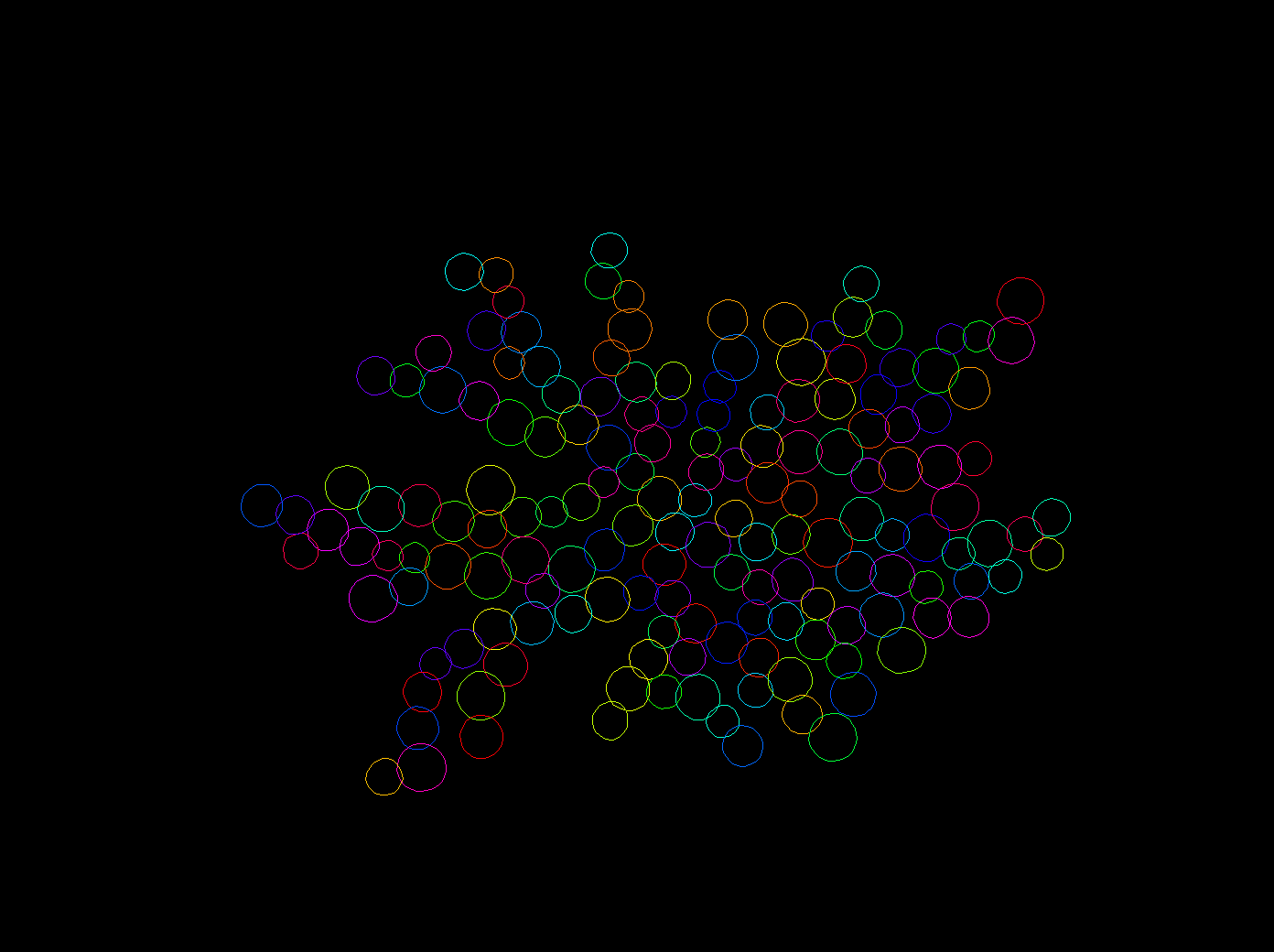

Supplement: Additional file 5 — The zip archive contains simulated images showing protoplasts with corresponding ground truth. (ZIP 72704 kb) [file 12859_2017_1591_MOESM5_ESM.zip › simulated protoplasts/overlapping/overlapping001 gt.png]

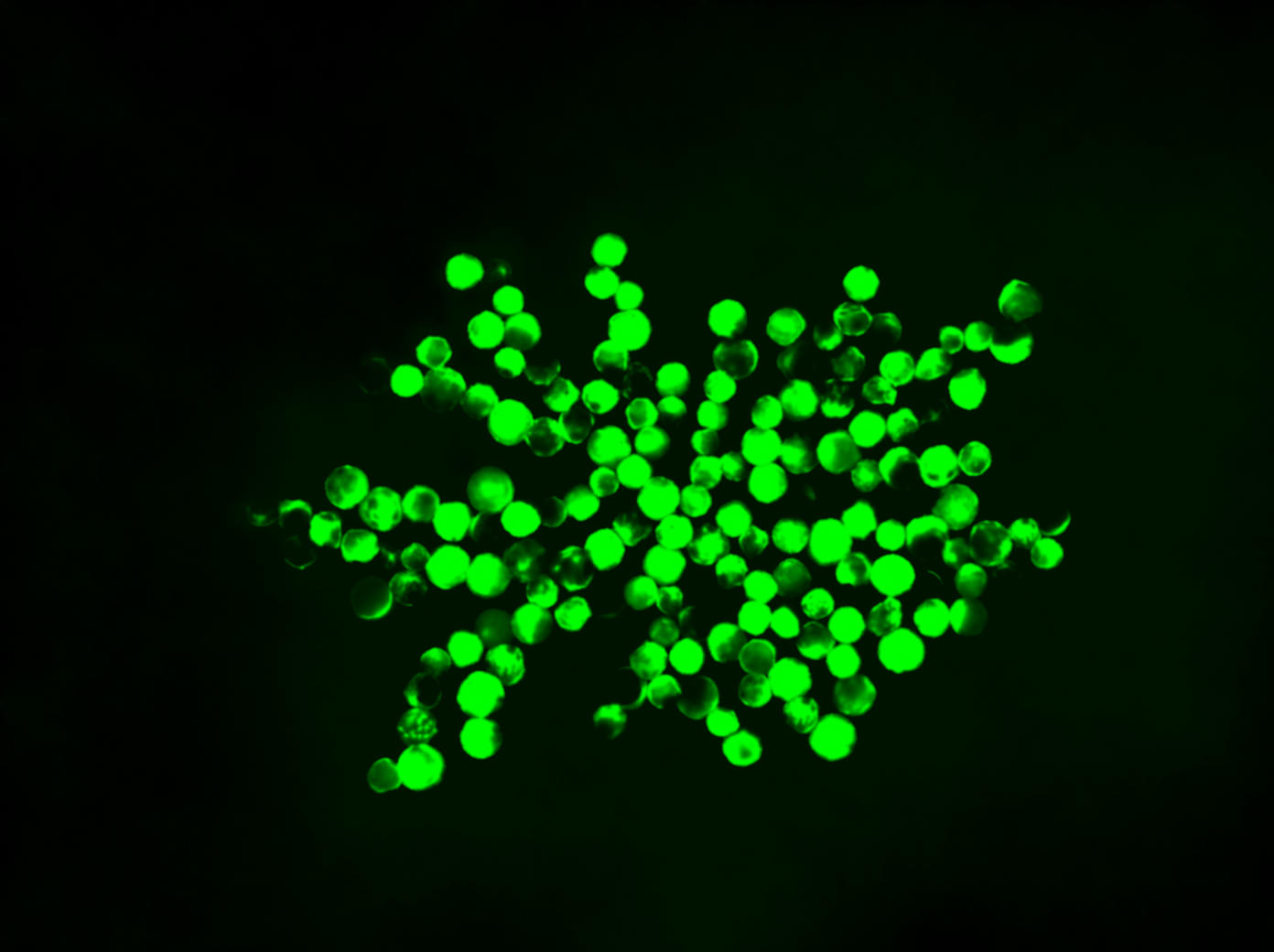

Supplement: Additional file 5 — The zip archive contains simulated images showing protoplasts with corresponding ground truth. (ZIP 72704 kb) [file 12859_2017_1591_MOESM5_ESM.zip › simulated protoplasts/overlapping/overlapping001.png]

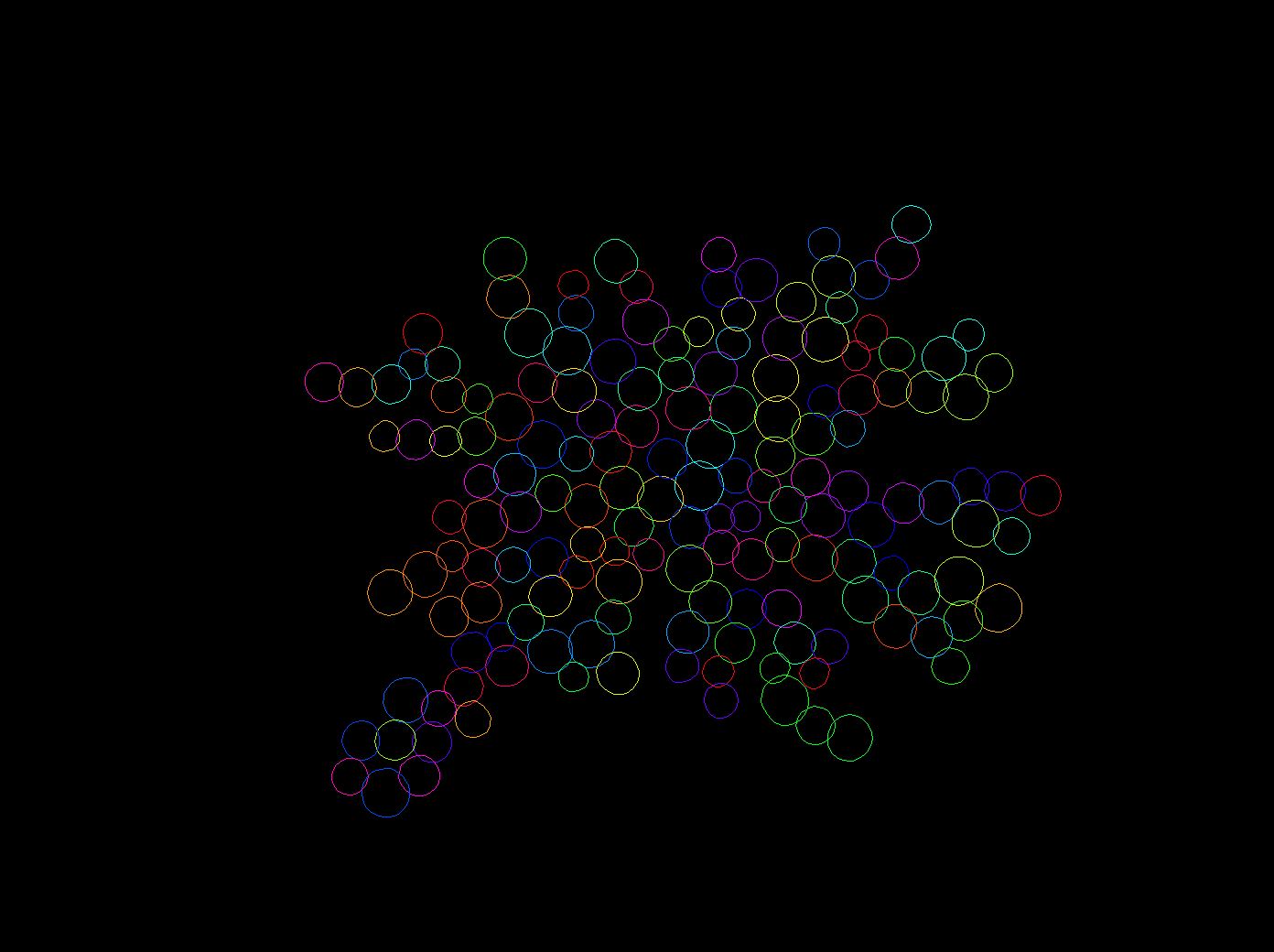

Supplement: Additional file 5 — The zip archive contains simulated images showing protoplasts with corresponding ground truth. (ZIP 72704 kb) [file 12859_2017_1591_MOESM5_ESM.zip › simulated protoplasts/overlapping/overlapping002 gt.png]

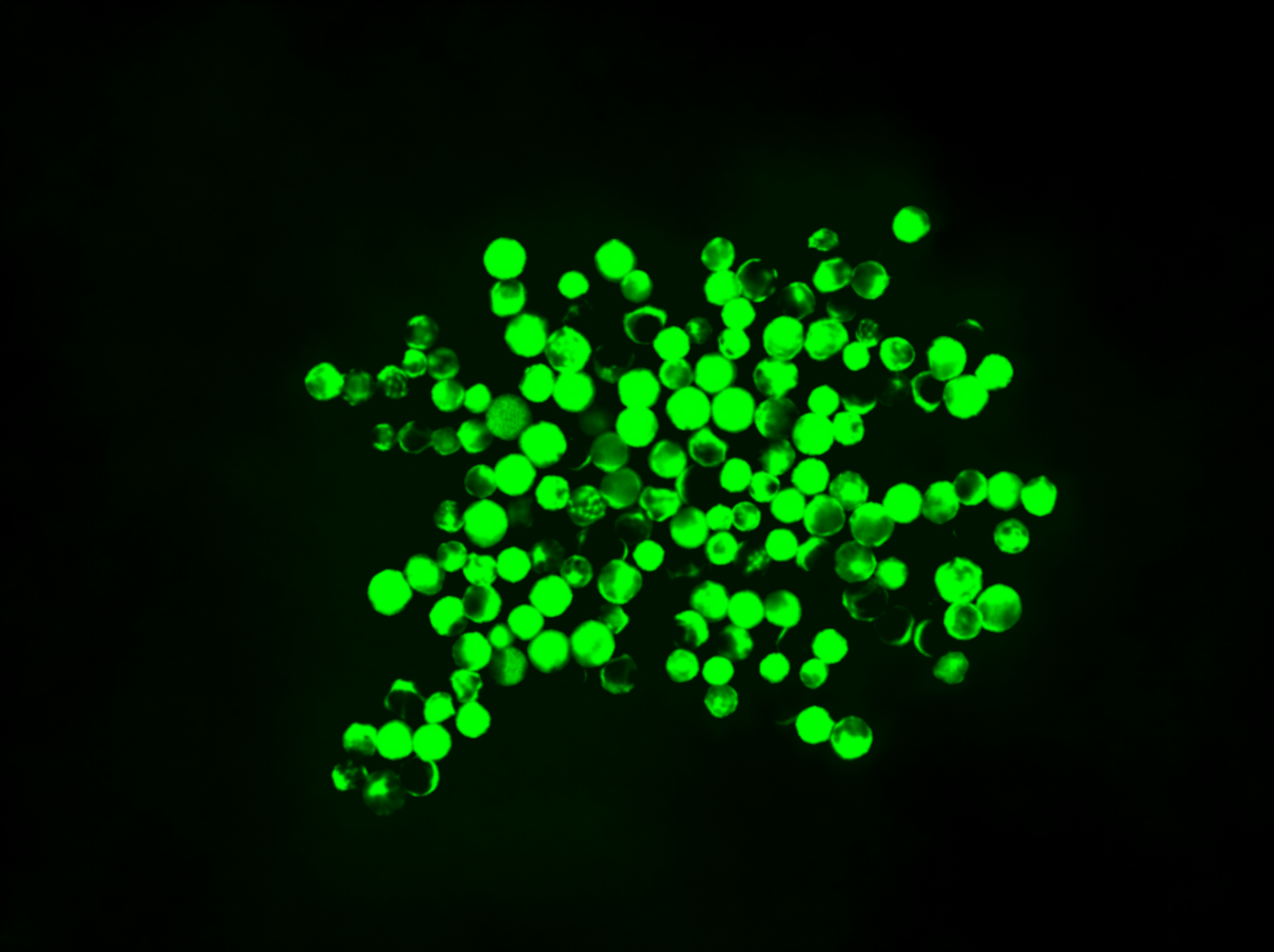

Supplement: Additional file 5 — The zip archive contains simulated images showing protoplasts with corresponding ground truth. (ZIP 72704 kb) [file 12859_2017_1591_MOESM5_ESM.zip › simulated protoplasts/overlapping/overlapping002.png]

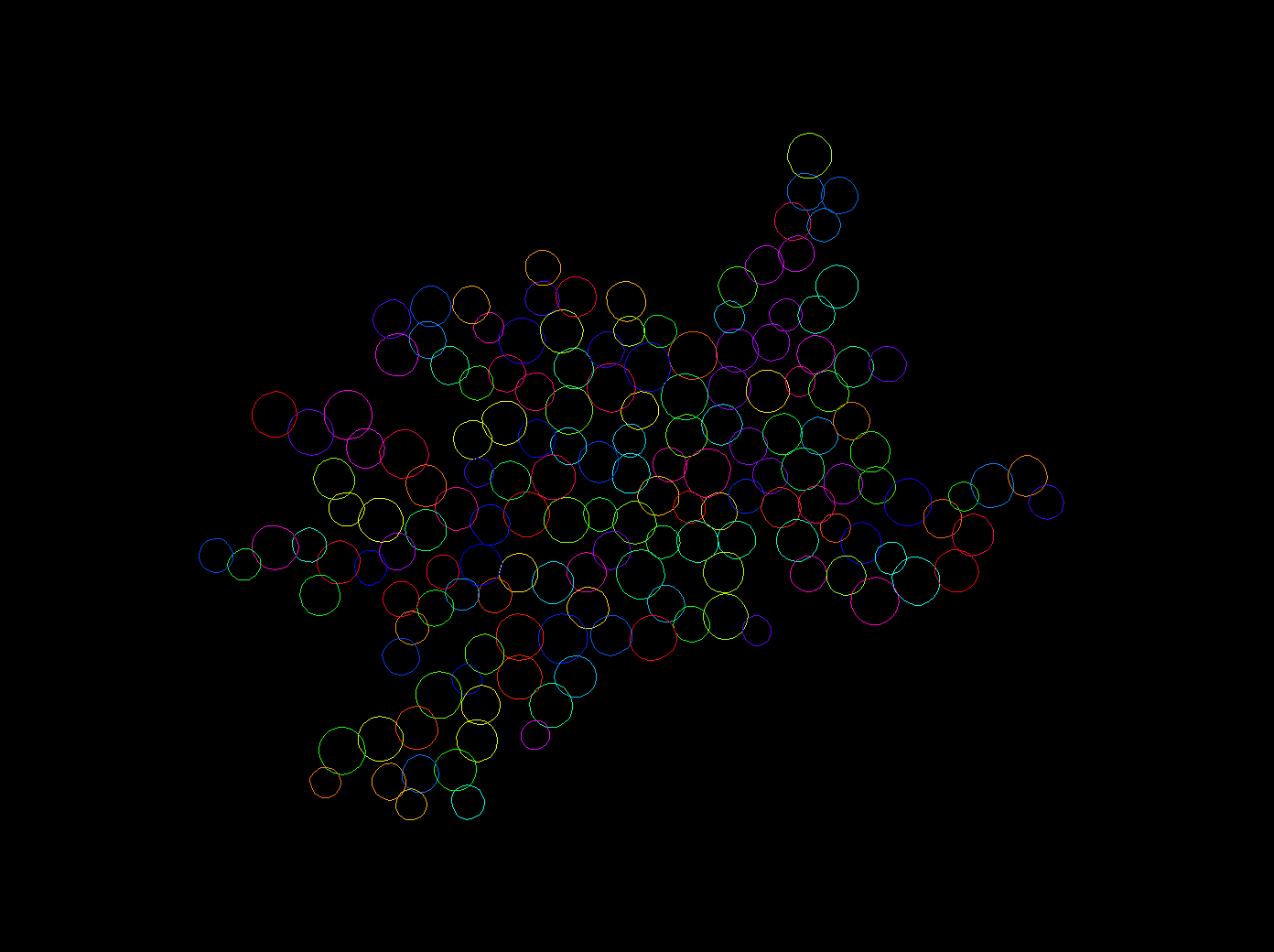

Supplement: Additional file 5 — The zip archive contains simulated images showing protoplasts with corresponding ground truth. (ZIP 72704 kb) [file 12859_2017_1591_MOESM5_ESM.zip › simulated protoplasts/overlapping/overlapping003 gt.png]

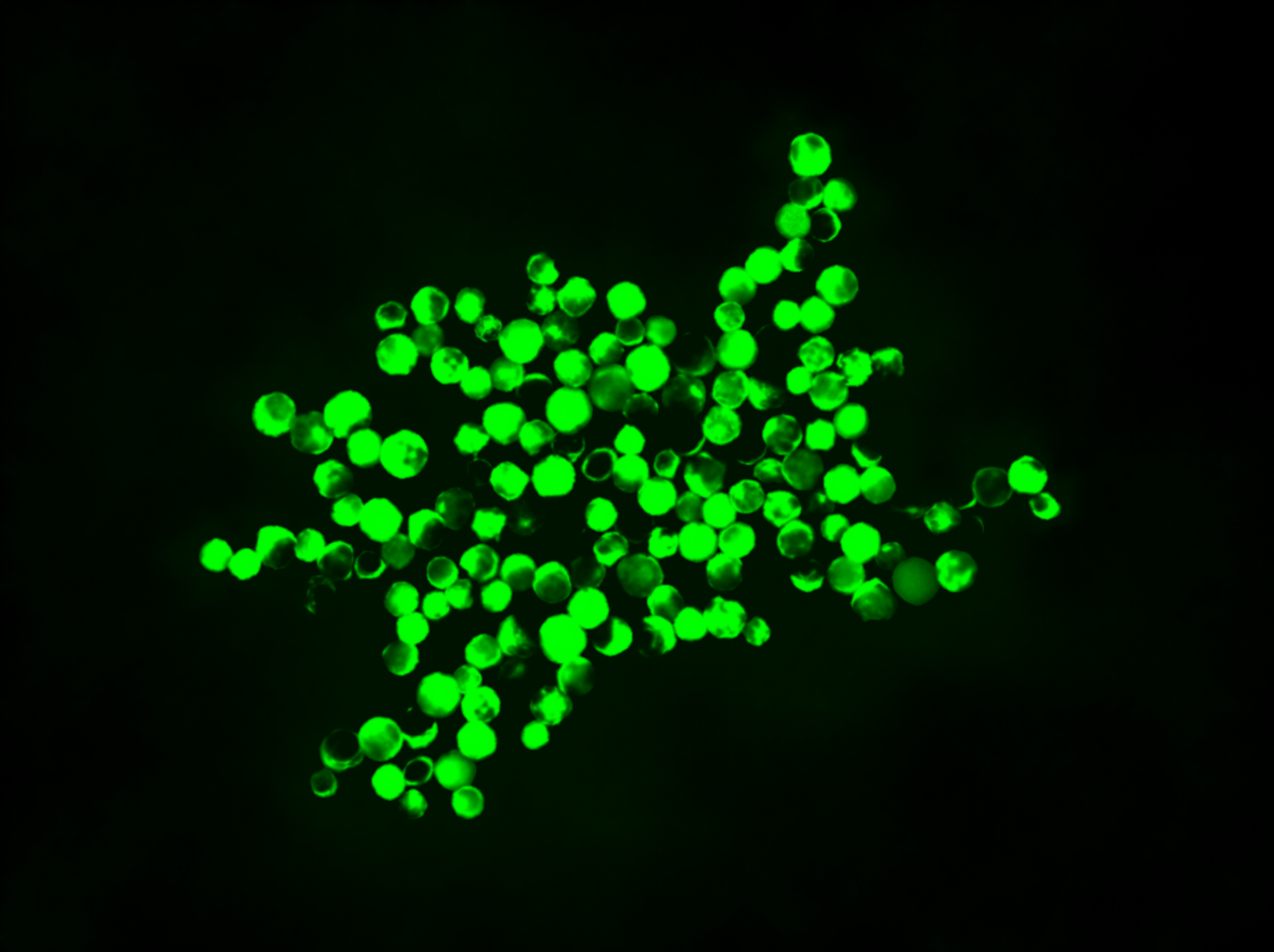

Supplement: Additional file 5 — The zip archive contains simulated images showing protoplasts with corresponding ground truth. (ZIP 72704 kb) [file 12859_2017_1591_MOESM5_ESM.zip › simulated protoplasts/overlapping/overlapping003.png]

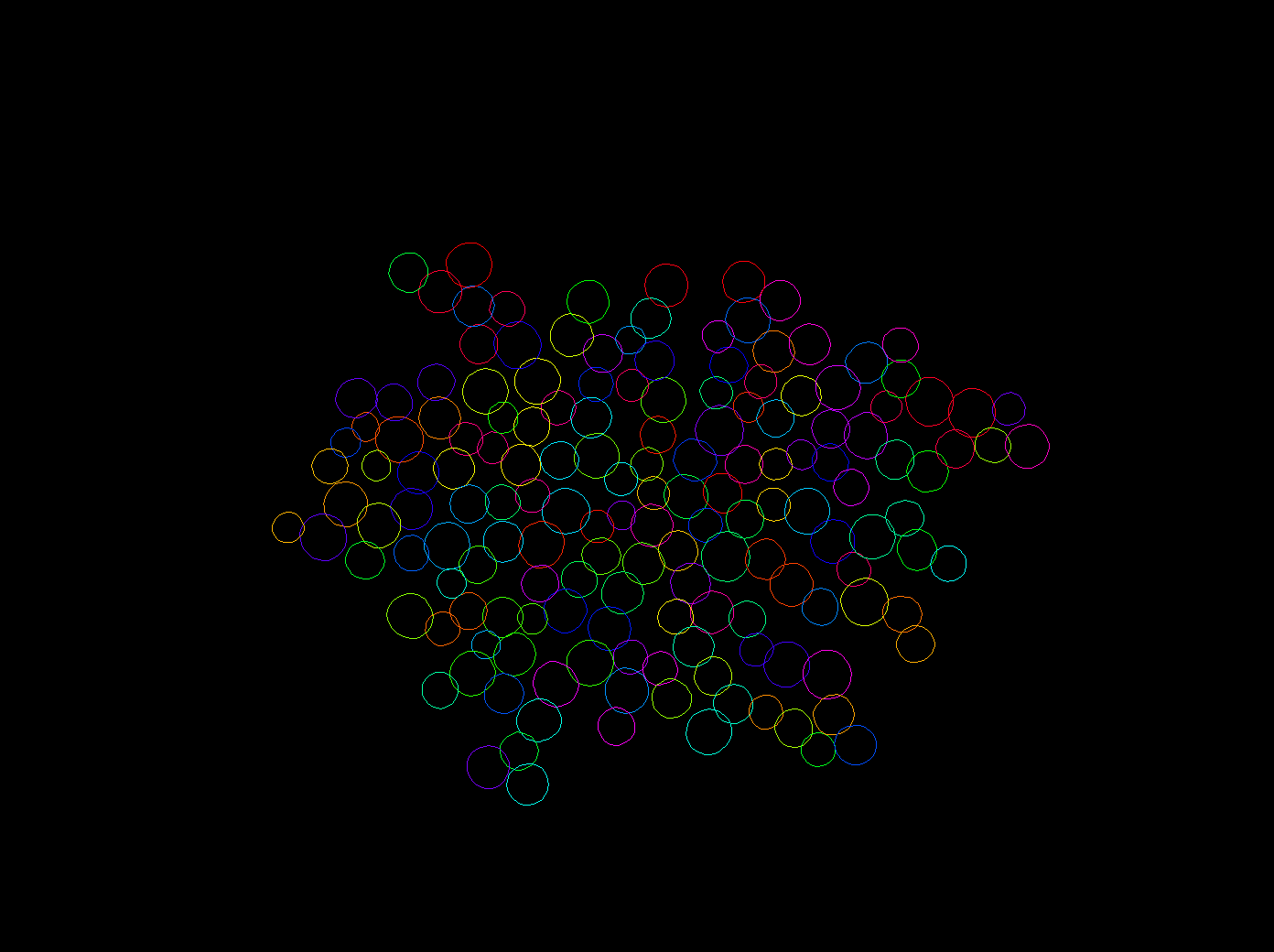

Supplement: Additional file 5 — The zip archive contains simulated images showing protoplasts with corresponding ground truth. (ZIP 72704 kb) [file 12859_2017_1591_MOESM5_ESM.zip › simulated protoplasts/overlapping/overlapping004 gt.png]

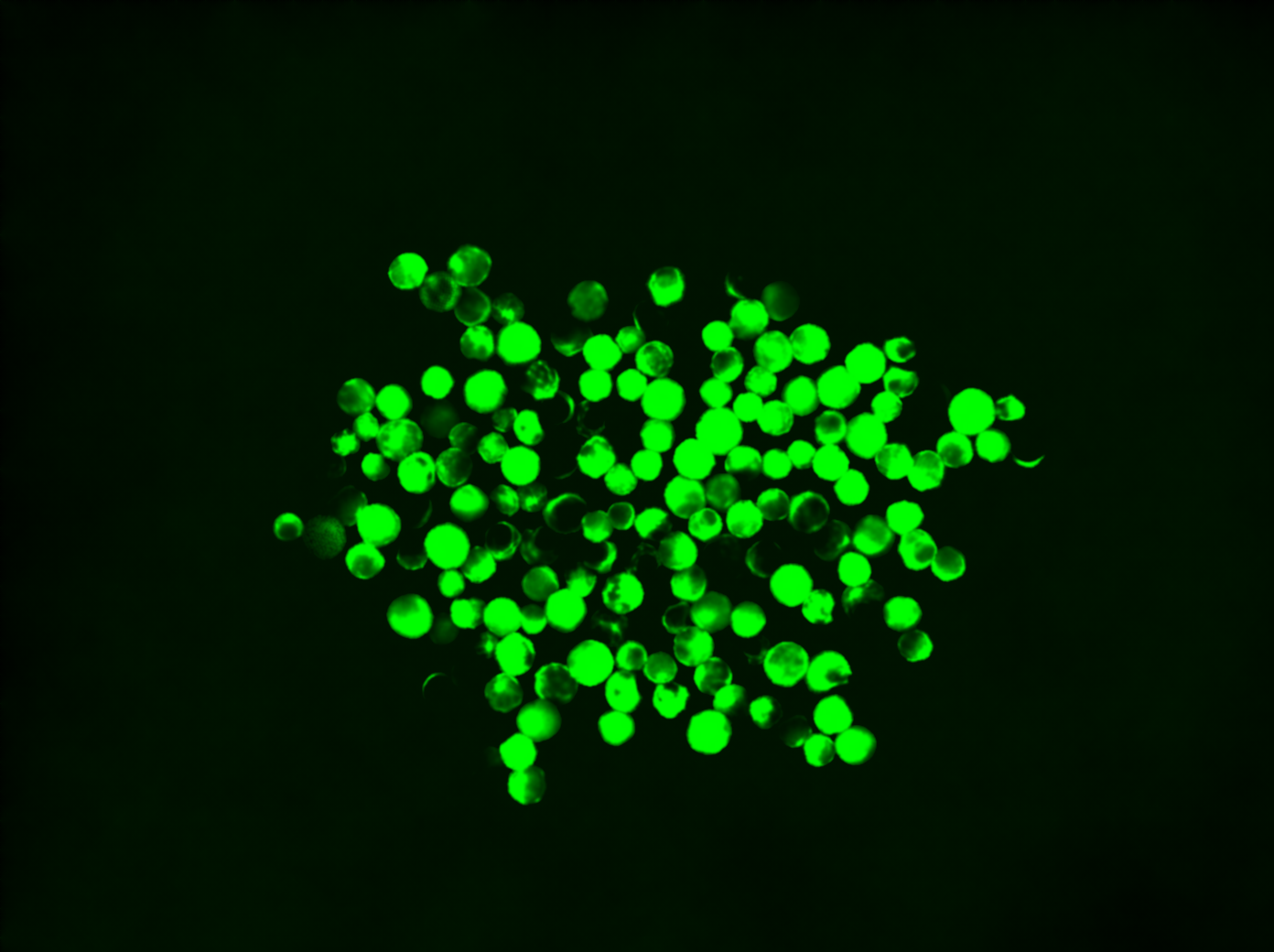

Supplement: Additional file 5 — The zip archive contains simulated images showing protoplasts with corresponding ground truth. (ZIP 72704 kb) [file 12859_2017_1591_MOESM5_ESM.zip › simulated protoplasts/overlapping/overlapping004.png]

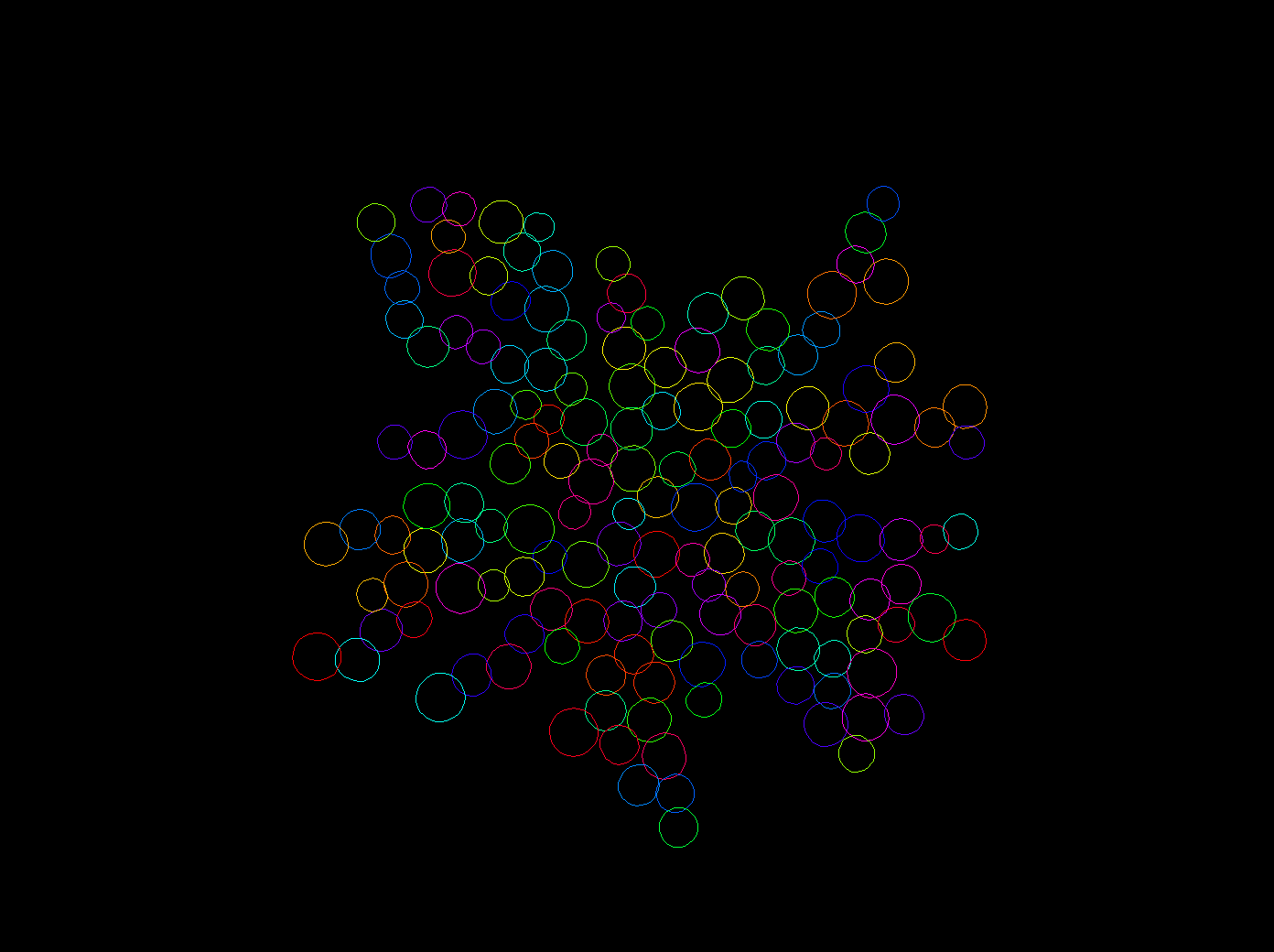

Supplement: Additional file 5 — The zip archive contains simulated images showing protoplasts with corresponding ground truth. (ZIP 72704 kb) [file 12859_2017_1591_MOESM5_ESM.zip › simulated protoplasts/overlapping/overlapping005 gt.png]

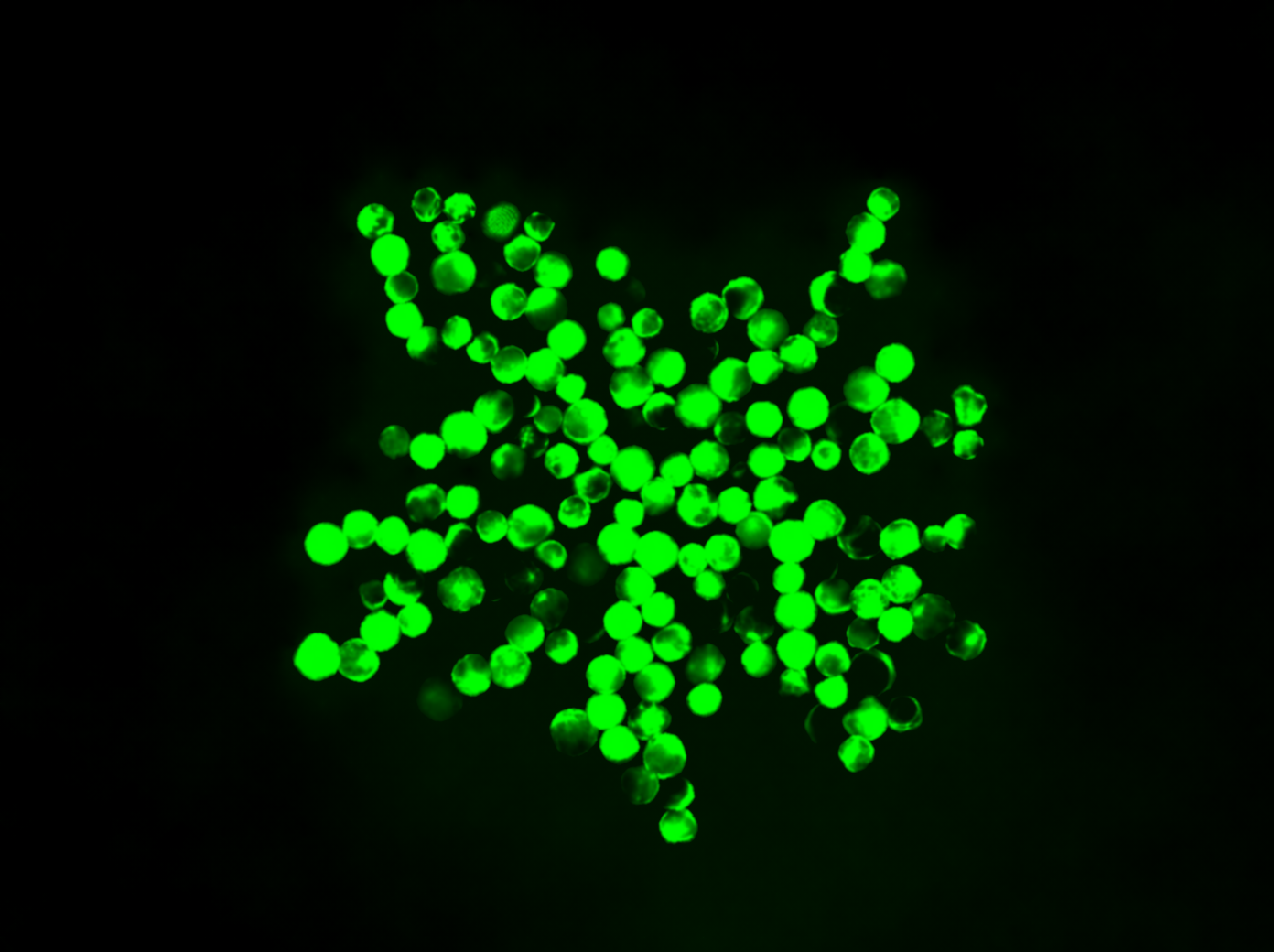

Supplement: Additional file 5 — The zip archive contains simulated images showing protoplasts with corresponding ground truth. (ZIP 72704 kb) [file 12859_2017_1591_MOESM5_ESM.zip › simulated protoplasts/overlapping/overlapping005.png]

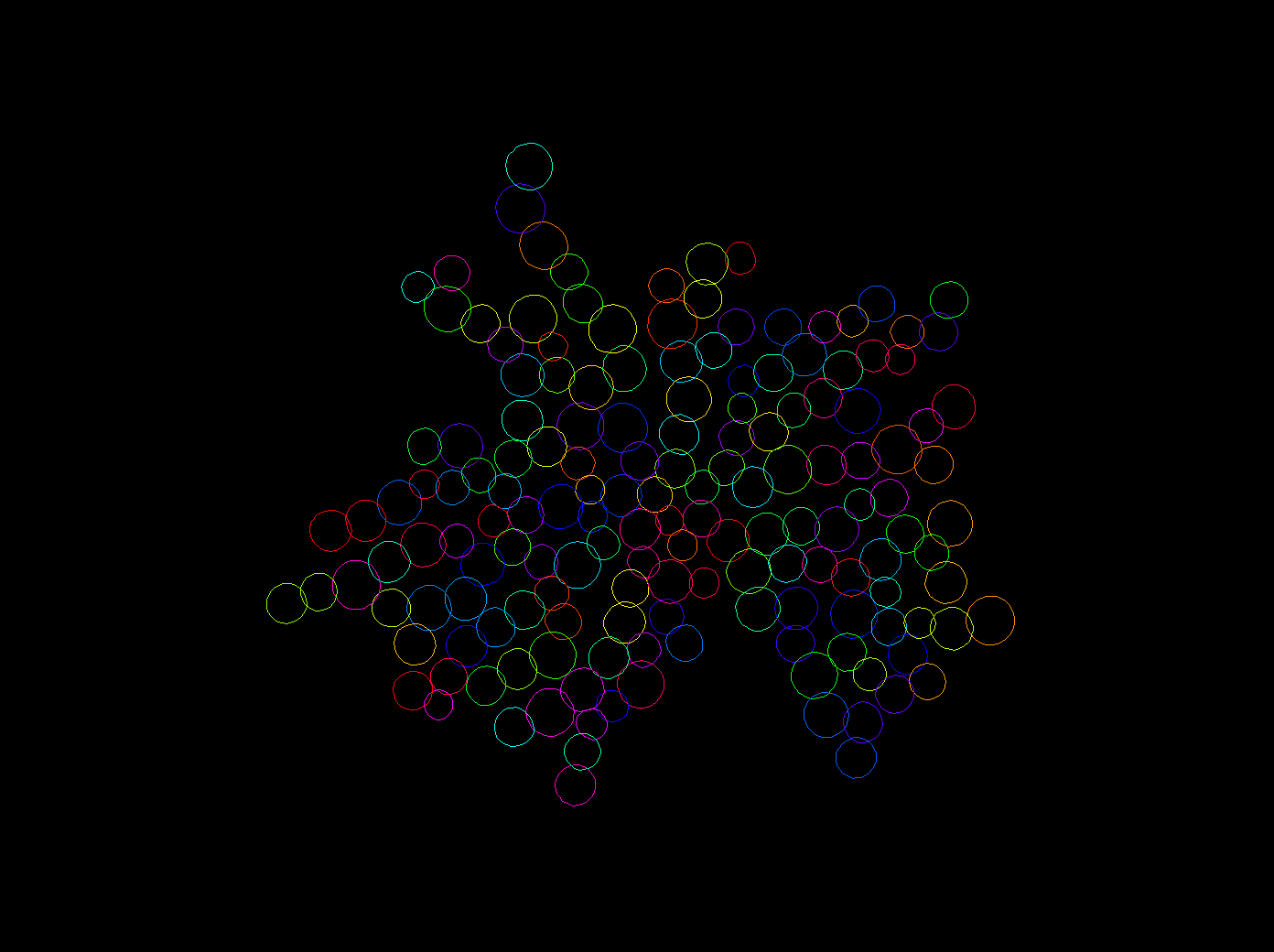

Supplement: Additional file 5 — The zip archive contains simulated images showing protoplasts with corresponding ground truth. (ZIP 72704 kb) [file 12859_2017_1591_MOESM5_ESM.zip › simulated protoplasts/overlapping/overlapping006 gt.png]

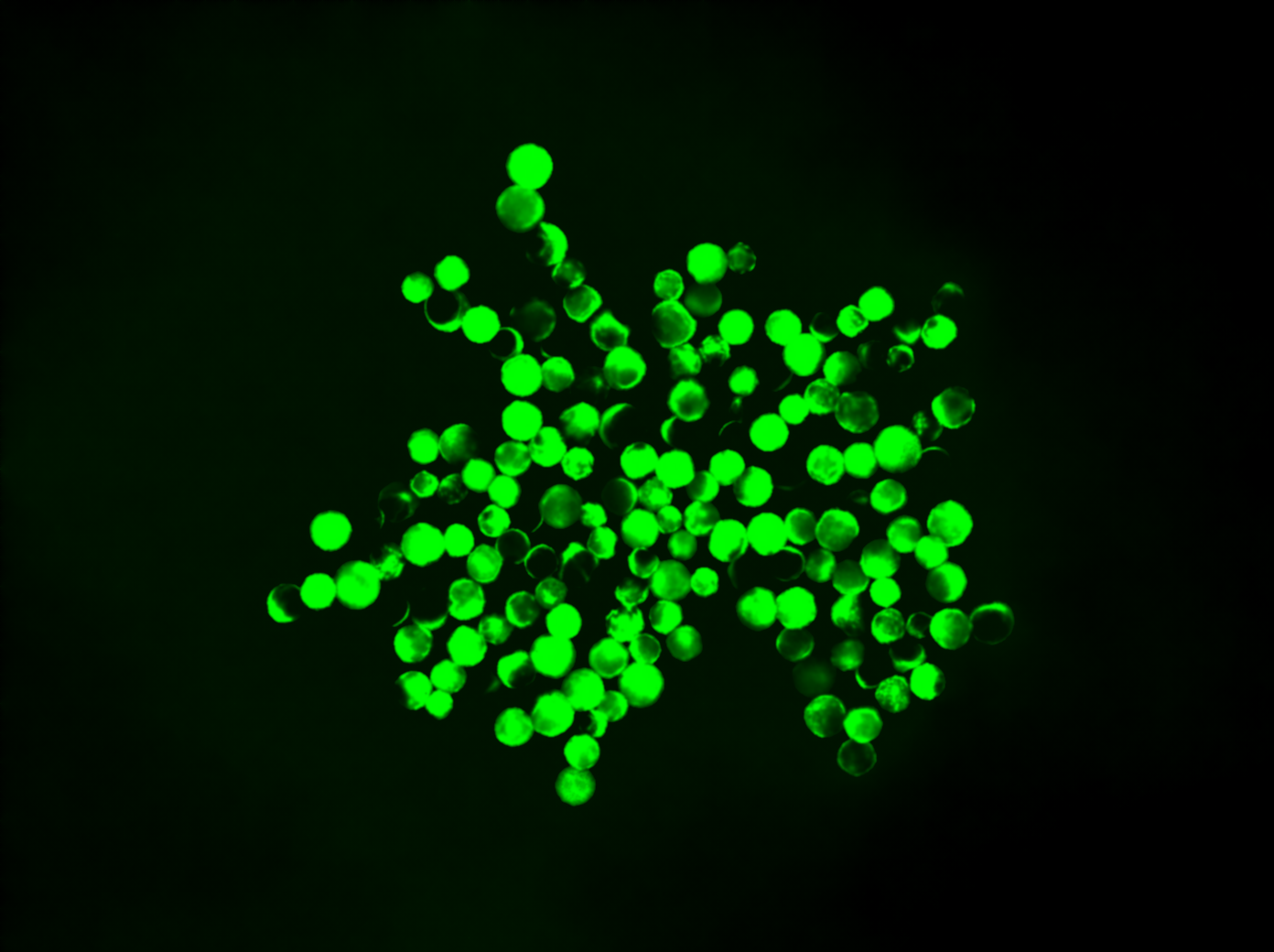

Supplement: Additional file 5 — The zip archive contains simulated images showing protoplasts with corresponding ground truth. (ZIP 72704 kb) [file 12859_2017_1591_MOESM5_ESM.zip › simulated protoplasts/overlapping/overlapping006.png]

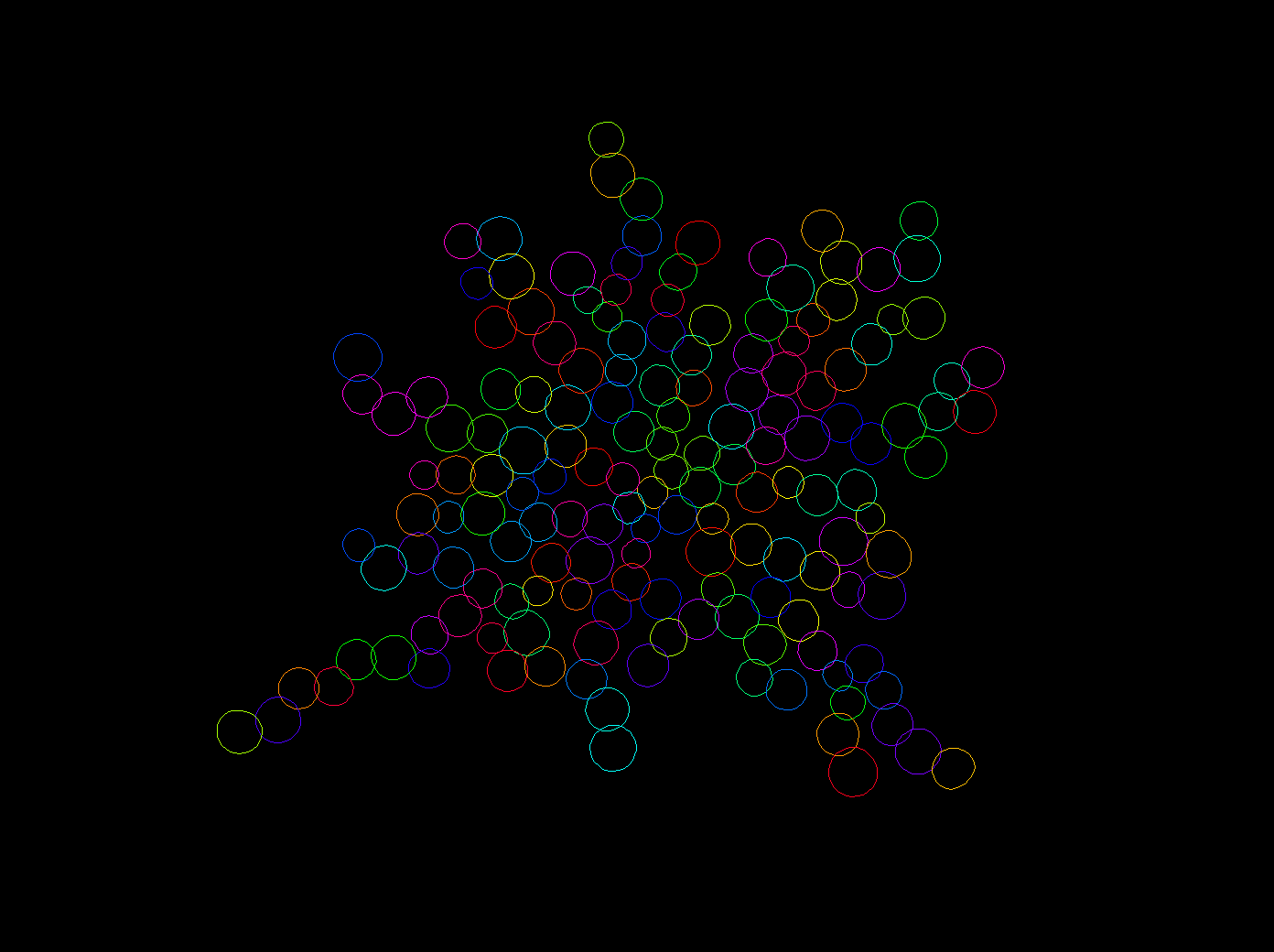

Supplement: Additional file 5 — The zip archive contains simulated images showing protoplasts with corresponding ground truth. (ZIP 72704 kb) [file 12859_2017_1591_MOESM5_ESM.zip › simulated protoplasts/overlapping/overlapping007 gt.png]

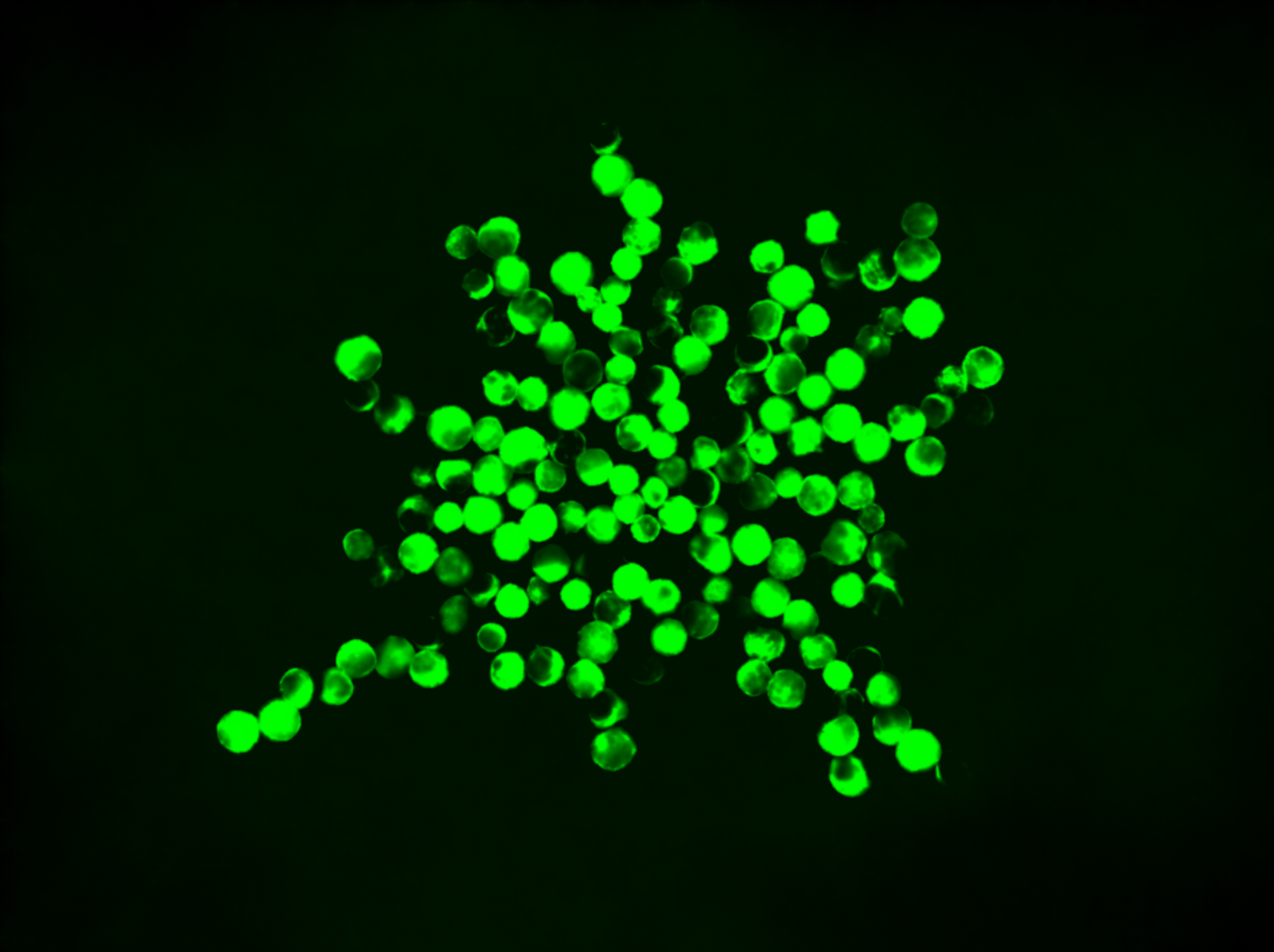

Supplement: Additional file 5 — The zip archive contains simulated images showing protoplasts with corresponding ground truth. (ZIP 72704 kb) [file 12859_2017_1591_MOESM5_ESM.zip › simulated protoplasts/overlapping/overlapping007.png]

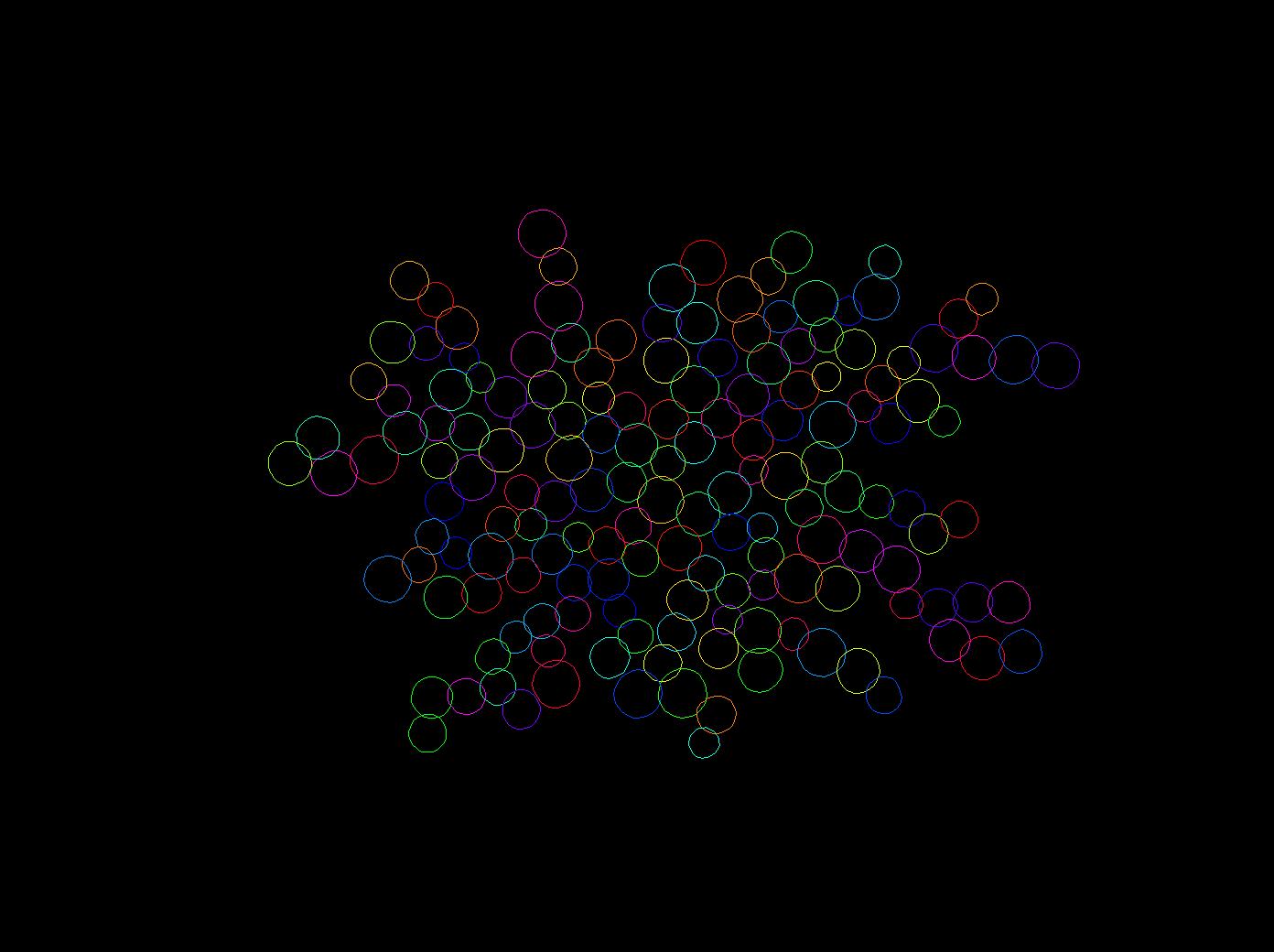

Supplement: Additional file 5 — The zip archive contains simulated images showing protoplasts with corresponding ground truth. (ZIP 72704 kb) [file 12859_2017_1591_MOESM5_ESM.zip › simulated protoplasts/overlapping/overlapping008 gt.png]

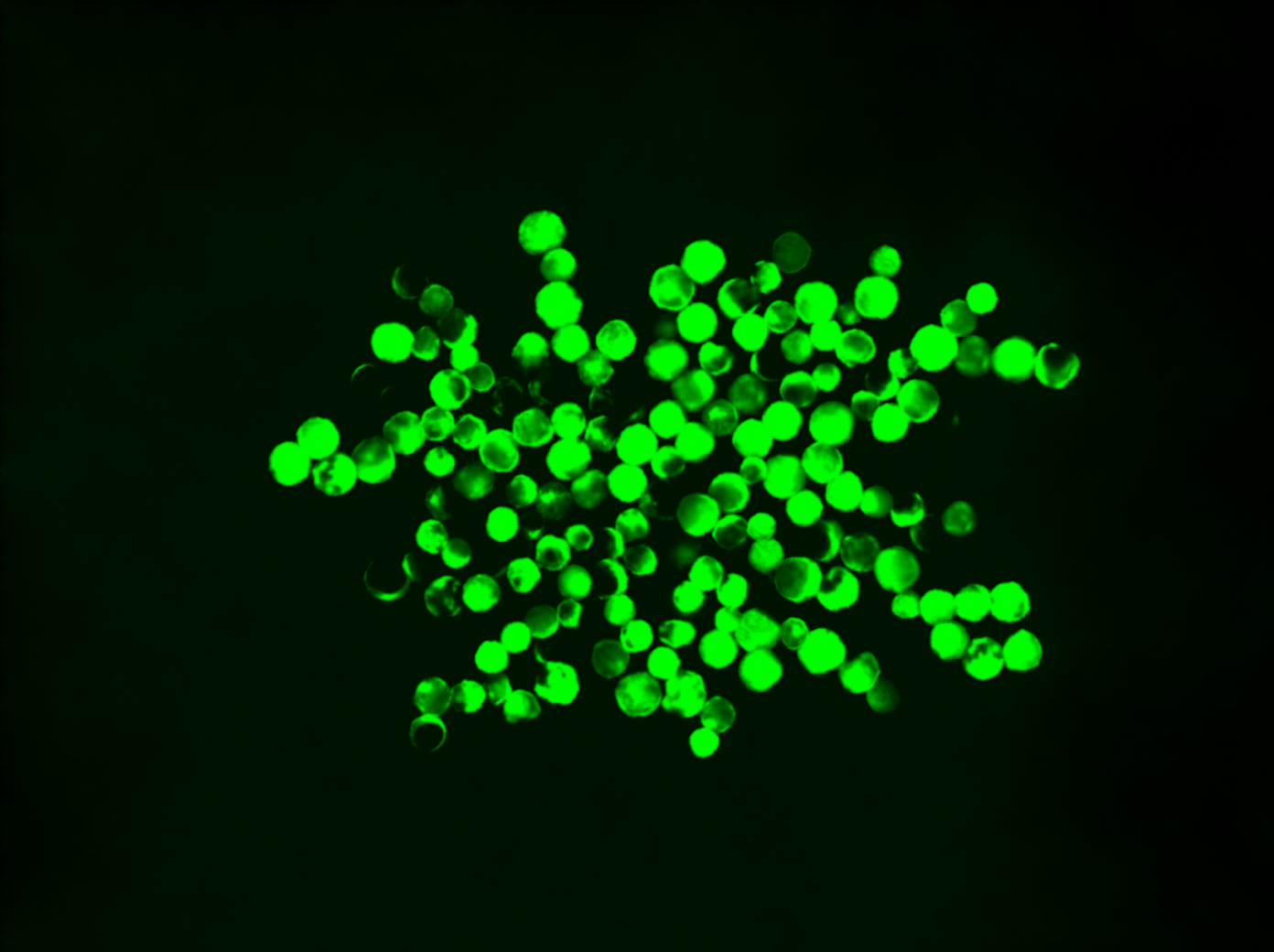

Supplement: Additional file 5 — The zip archive contains simulated images showing protoplasts with corresponding ground truth. (ZIP 72704 kb) [file 12859_2017_1591_MOESM5_ESM.zip › simulated protoplasts/overlapping/overlapping008.png]

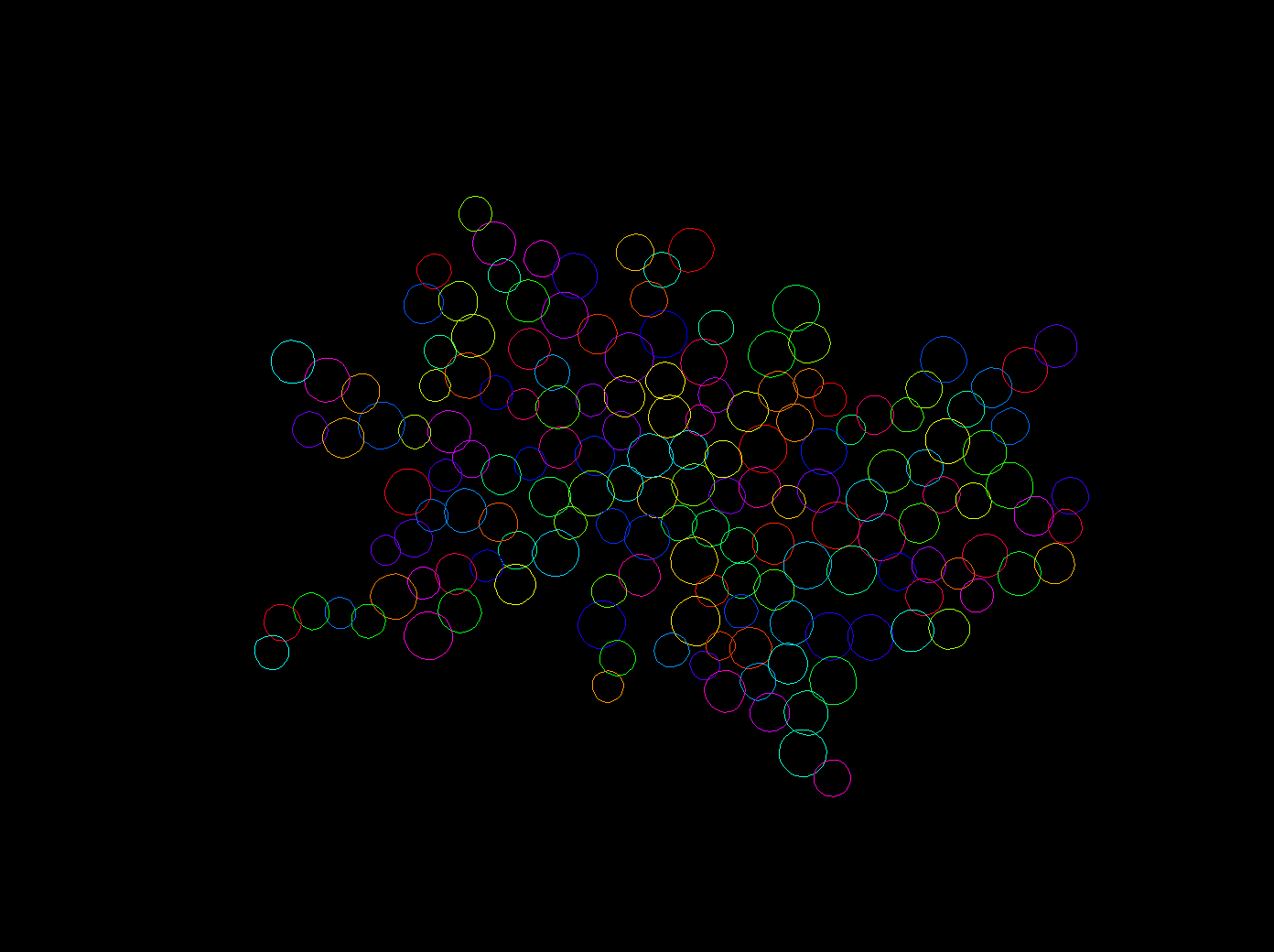

Supplement: Additional file 5 — The zip archive contains simulated images showing protoplasts with corresponding ground truth. (ZIP 72704 kb) [file 12859_2017_1591_MOESM5_ESM.zip › simulated protoplasts/overlapping/overlapping009 gt.png]

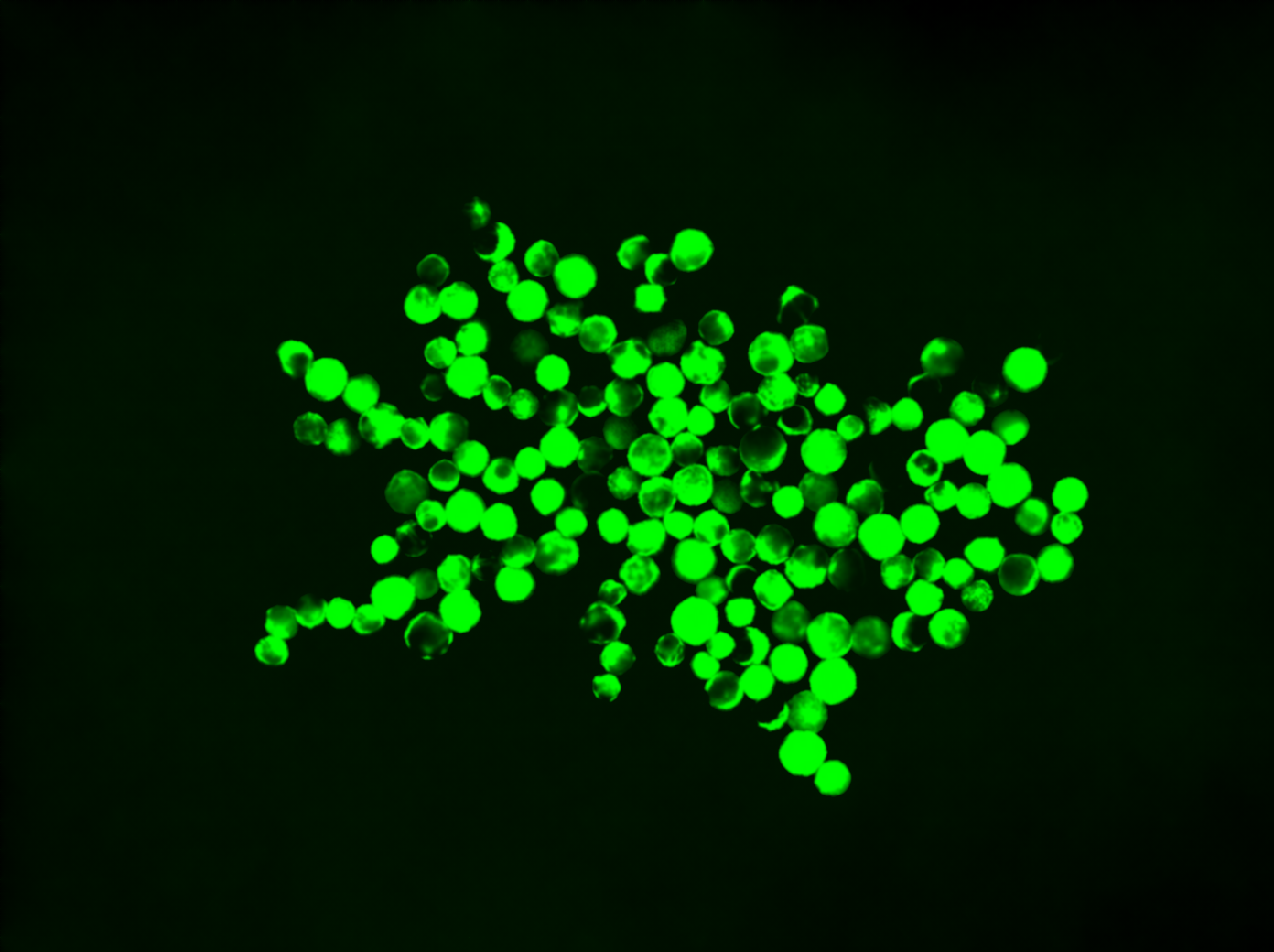

Supplement: Additional file 5 — The zip archive contains simulated images showing protoplasts with corresponding ground truth. (ZIP 72704 kb) [file 12859_2017_1591_MOESM5_ESM.zip › simulated protoplasts/overlapping/overlapping009.png]

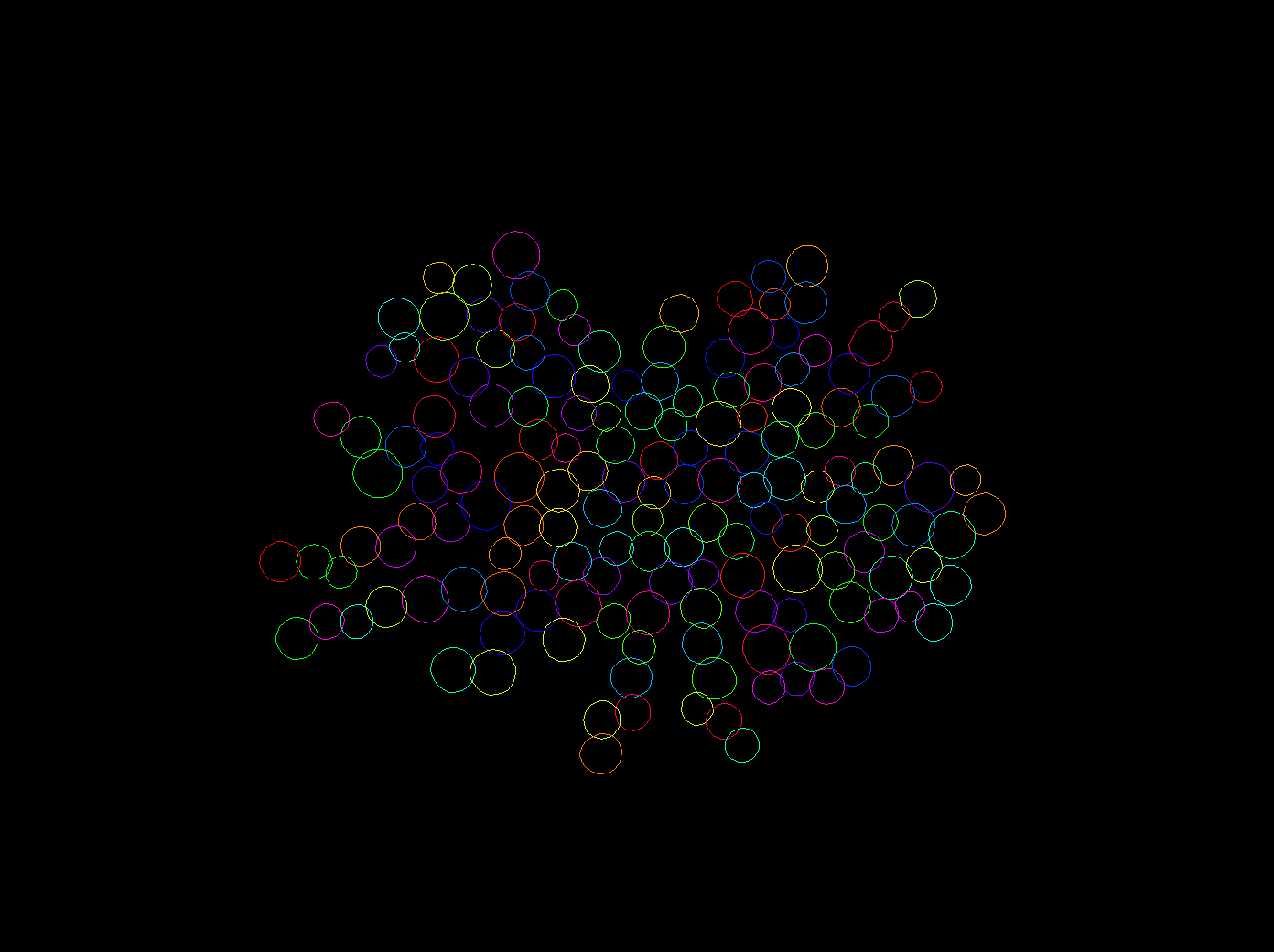

Supplement: Additional file 5 — The zip archive contains simulated images showing protoplasts with corresponding ground truth. (ZIP 72704 kb) [file 12859_2017_1591_MOESM5_ESM.zip › simulated protoplasts/overlapping/overlapping010 gt.png]

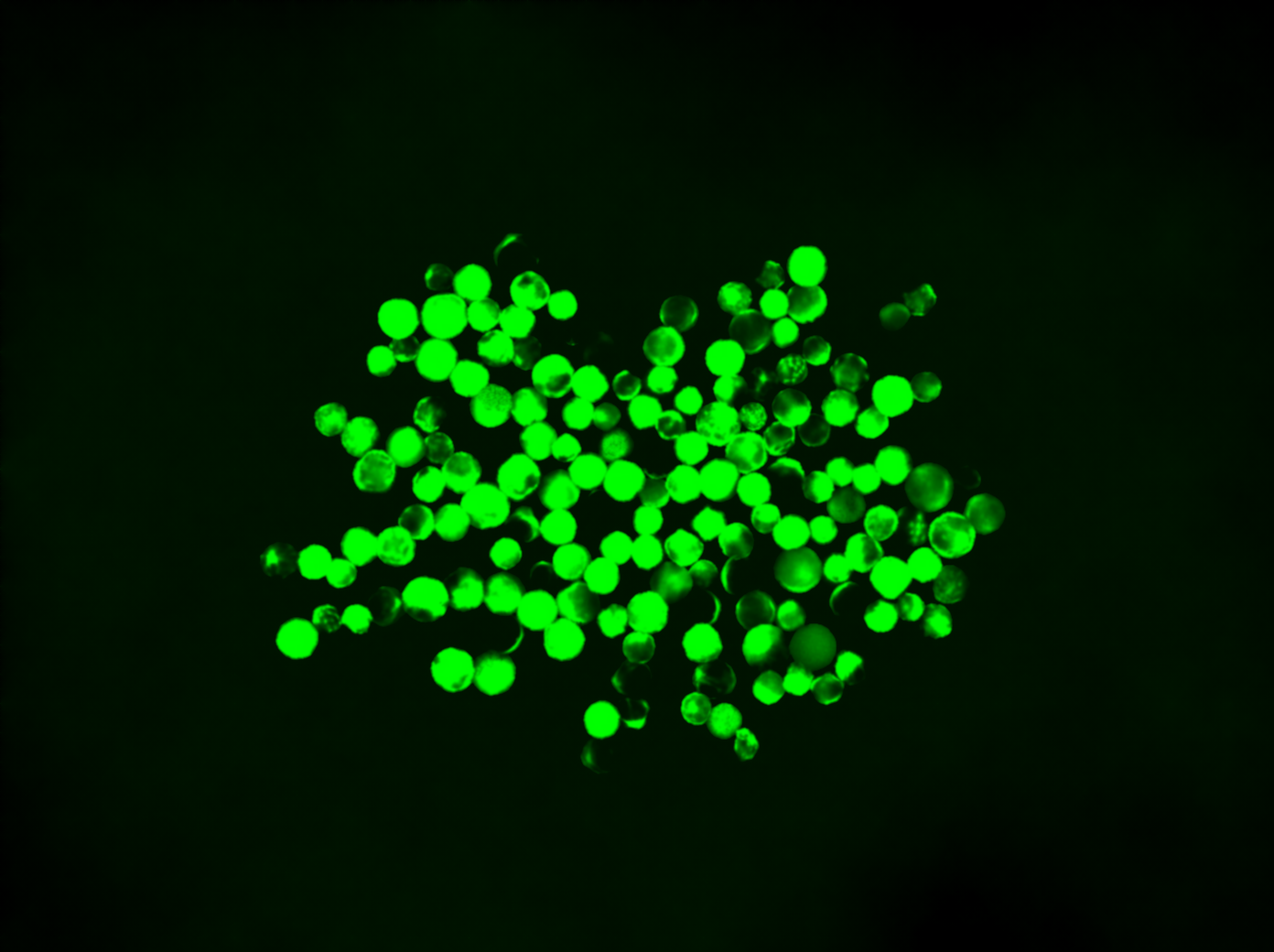

Supplement: Additional file 5 — The zip archive contains simulated images showing protoplasts with corresponding ground truth. (ZIP 72704 kb) [file 12859_2017_1591_MOESM5_ESM.zip › simulated protoplasts/overlapping/overlapping010.png]

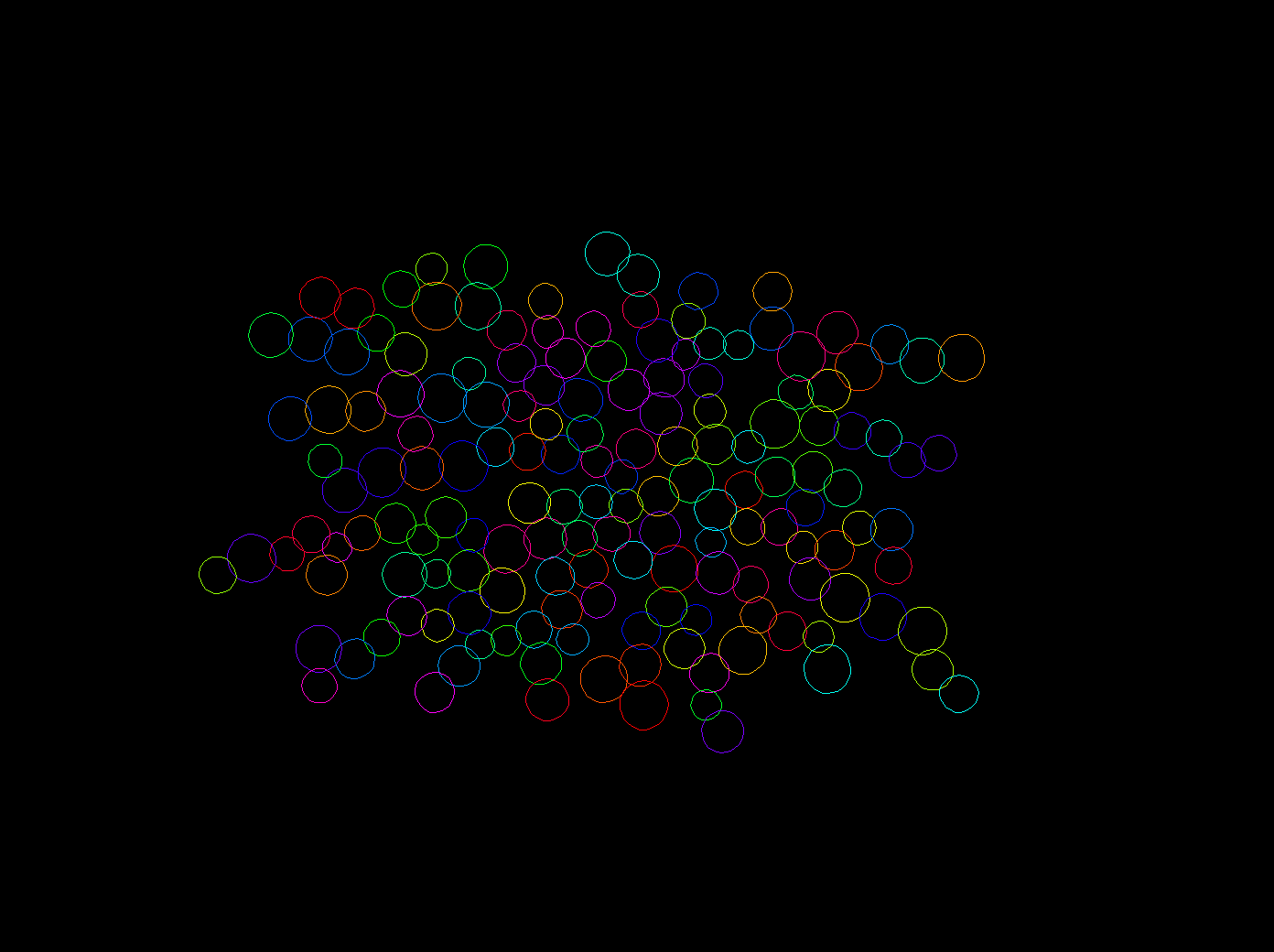

Supplement: Additional file 5 — The zip archive contains simulated images showing protoplasts with corresponding ground truth. (ZIP 72704 kb) [file 12859_2017_1591_MOESM5_ESM.zip › simulated protoplasts/overlapping/overlapping011 gt.png]

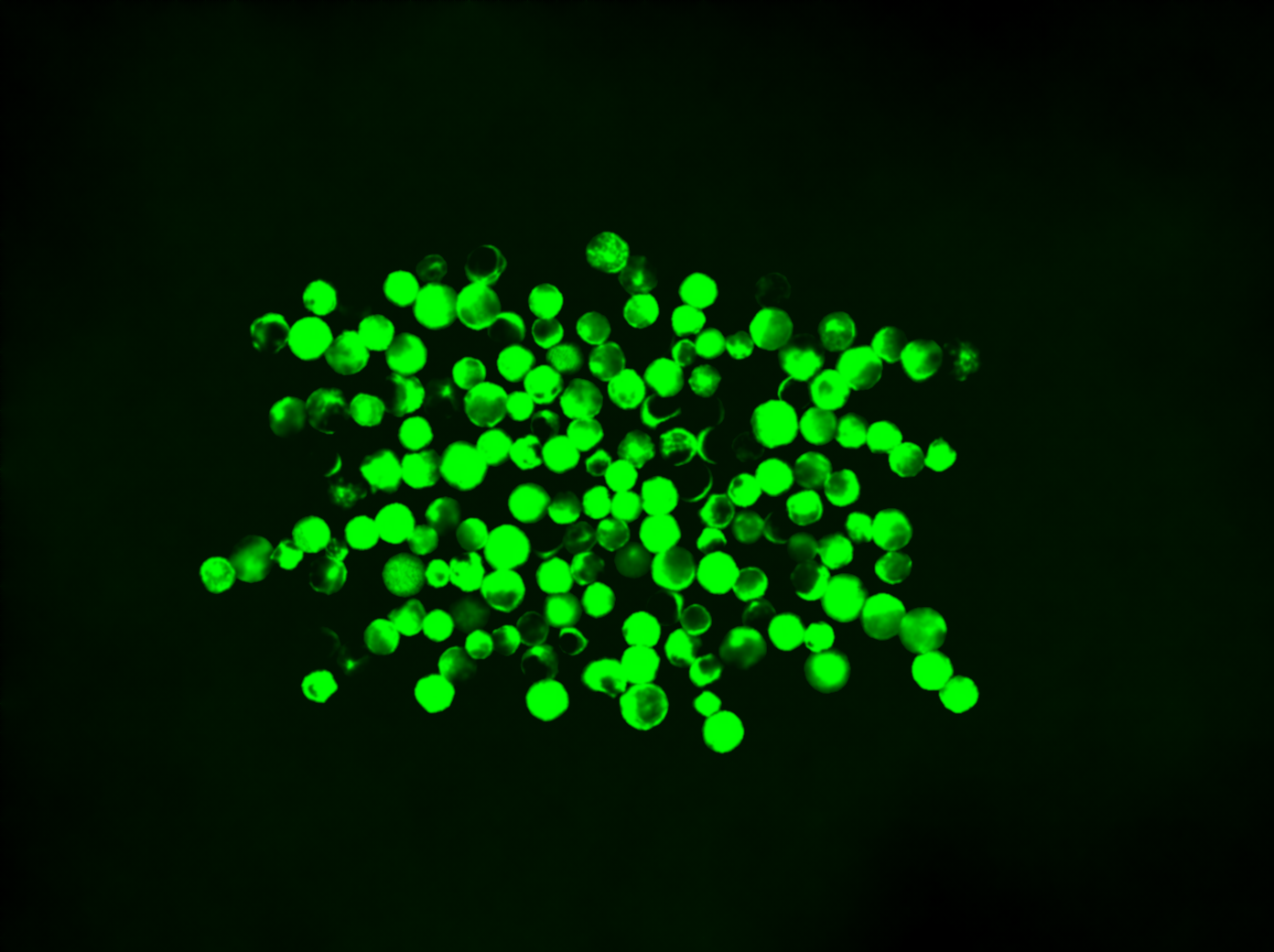

Supplement: Additional file 5 — The zip archive contains simulated images showing protoplasts with corresponding ground truth. (ZIP 72704 kb) [file 12859_2017_1591_MOESM5_ESM.zip › simulated protoplasts/overlapping/overlapping011.png]

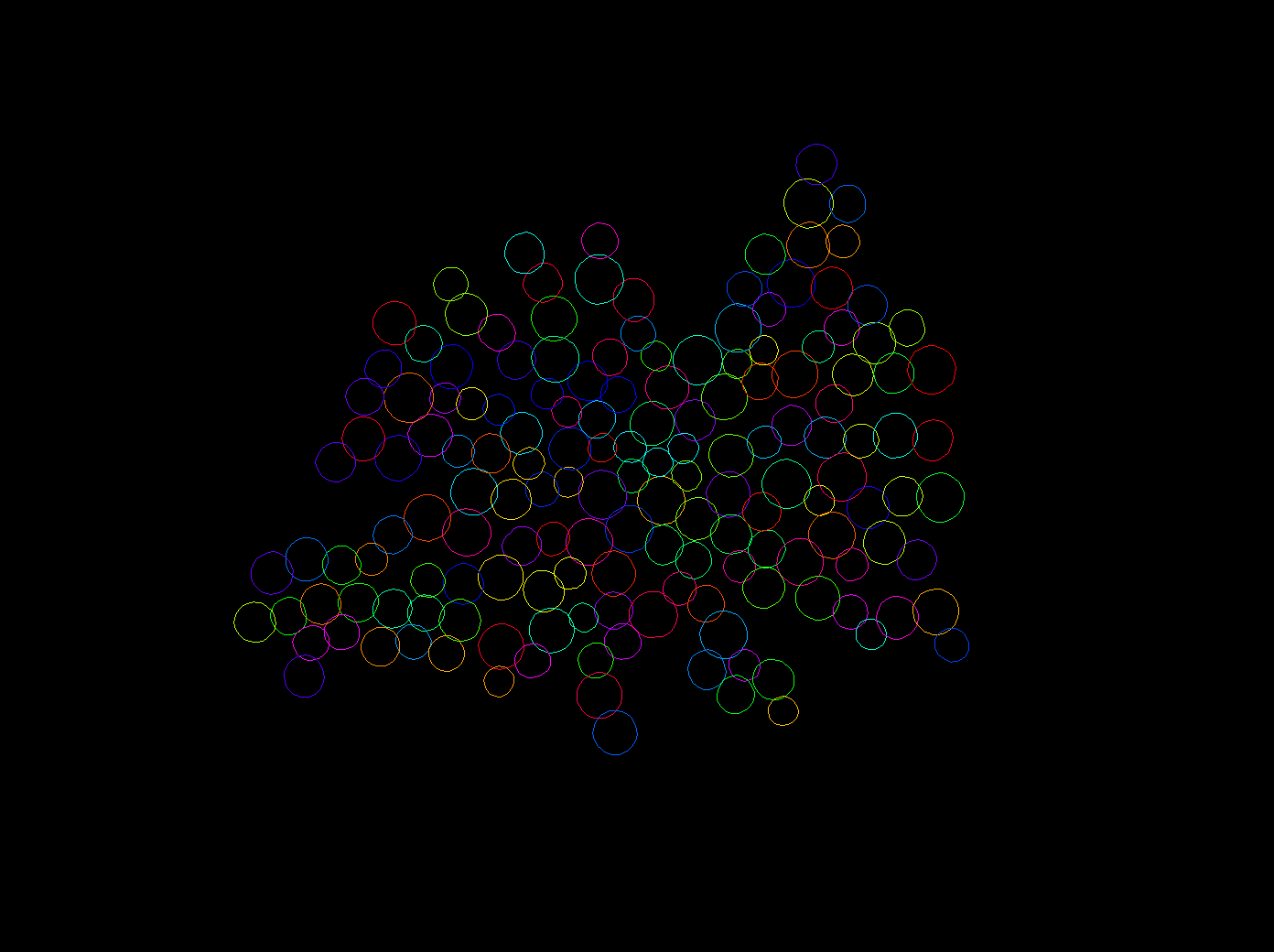

Supplement: Additional file 5 — The zip archive contains simulated images showing protoplasts with corresponding ground truth. (ZIP 72704 kb) [file 12859_2017_1591_MOESM5_ESM.zip › simulated protoplasts/overlapping/overlapping012 gt.png]

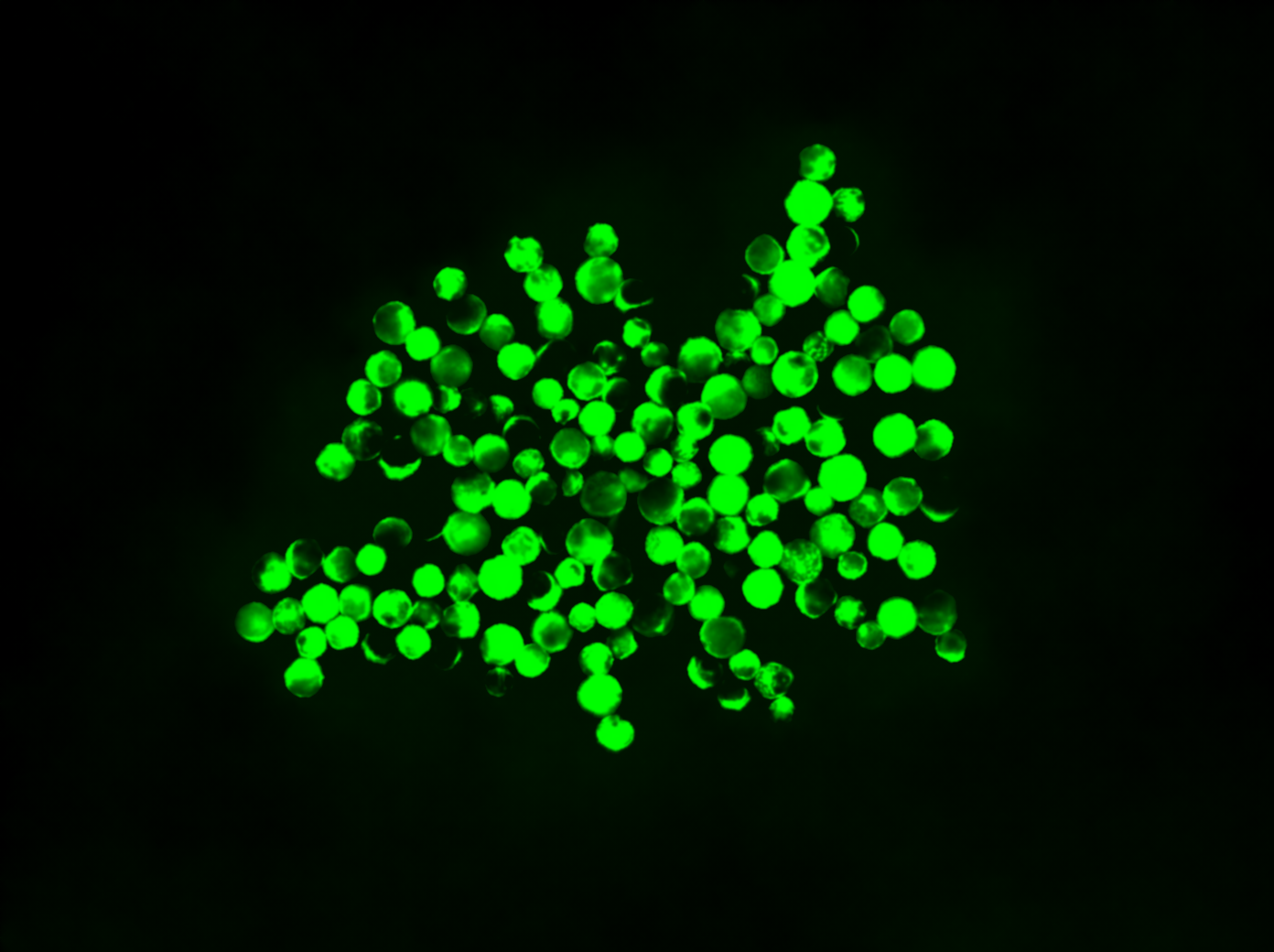

Supplement: Additional file 5 — The zip archive contains simulated images showing protoplasts with corresponding ground truth. (ZIP 72704 kb) [file 12859_2017_1591_MOESM5_ESM.zip › simulated protoplasts/overlapping/overlapping012.png]

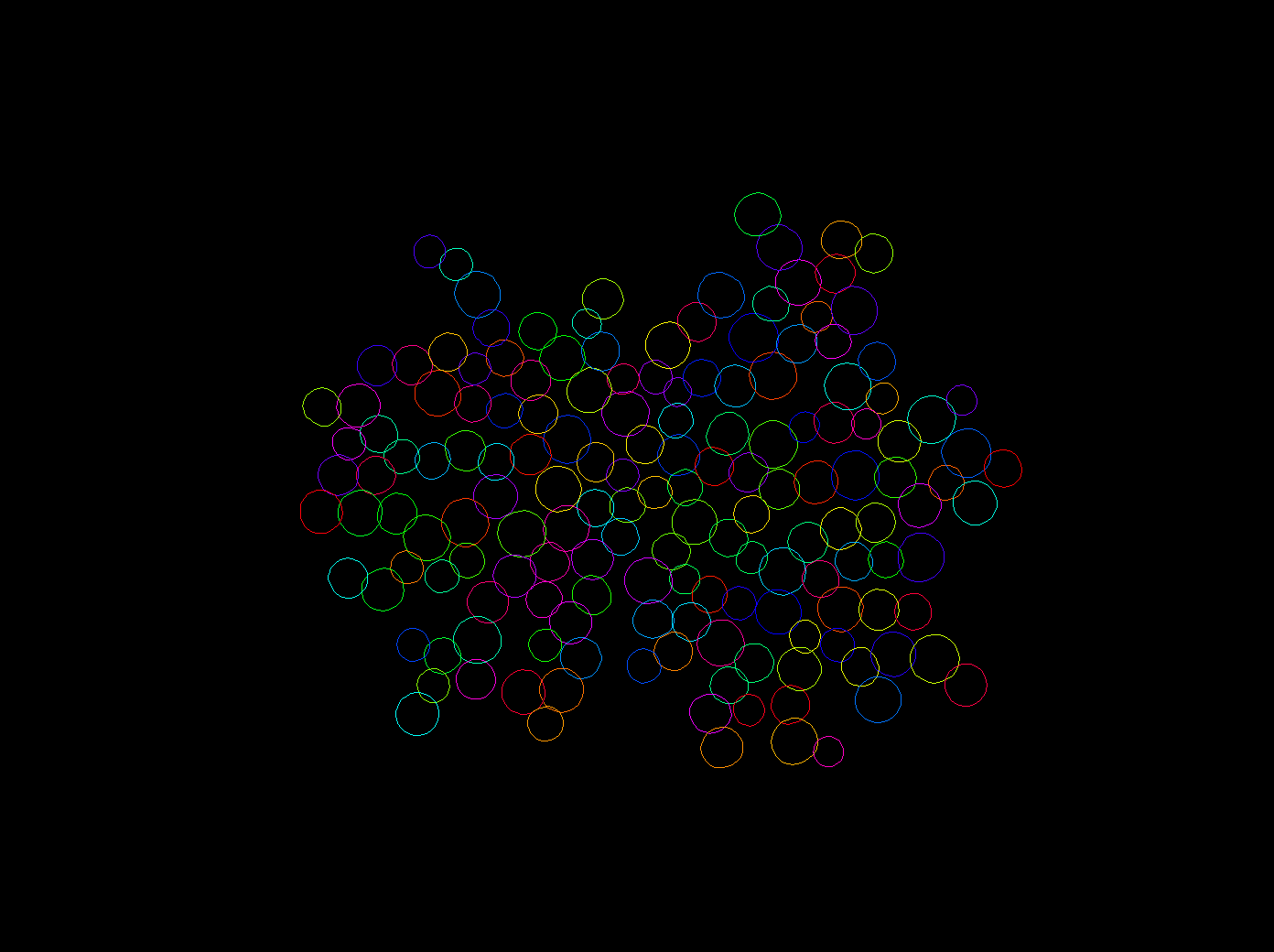

Supplement: Additional file 5 — The zip archive contains simulated images showing protoplasts with corresponding ground truth. (ZIP 72704 kb) [file 12859_2017_1591_MOESM5_ESM.zip › simulated protoplasts/overlapping/overlapping013 gt.png]

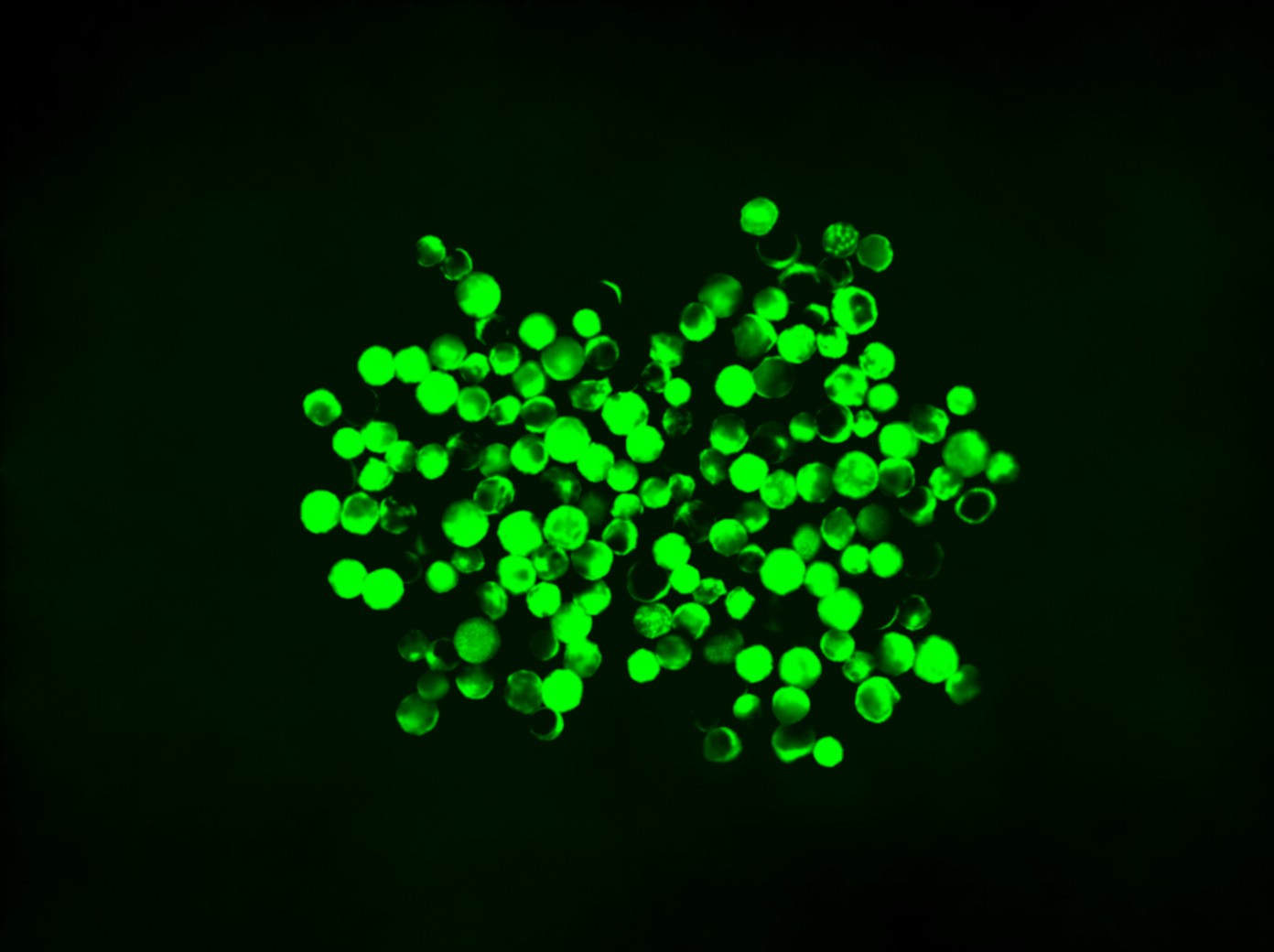

Supplement: Additional file 5 — The zip archive contains simulated images showing protoplasts with corresponding ground truth. (ZIP 72704 kb) [file 12859_2017_1591_MOESM5_ESM.zip › simulated protoplasts/overlapping/overlapping013.png]

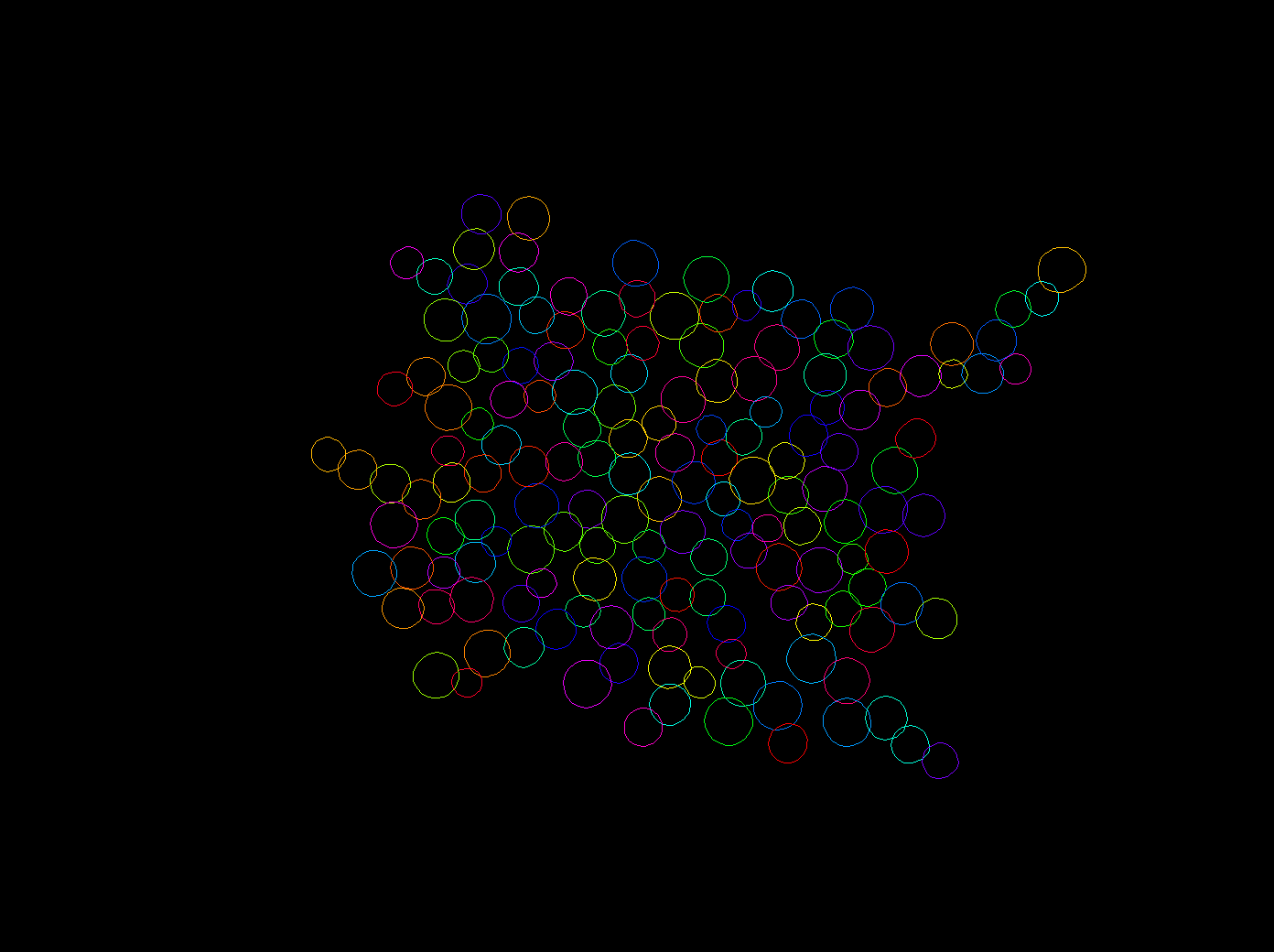

Supplement: Additional file 5 — The zip archive contains simulated images showing protoplasts with corresponding ground truth. (ZIP 72704 kb) [file 12859_2017_1591_MOESM5_ESM.zip › simulated protoplasts/overlapping/overlapping014 gt.png]

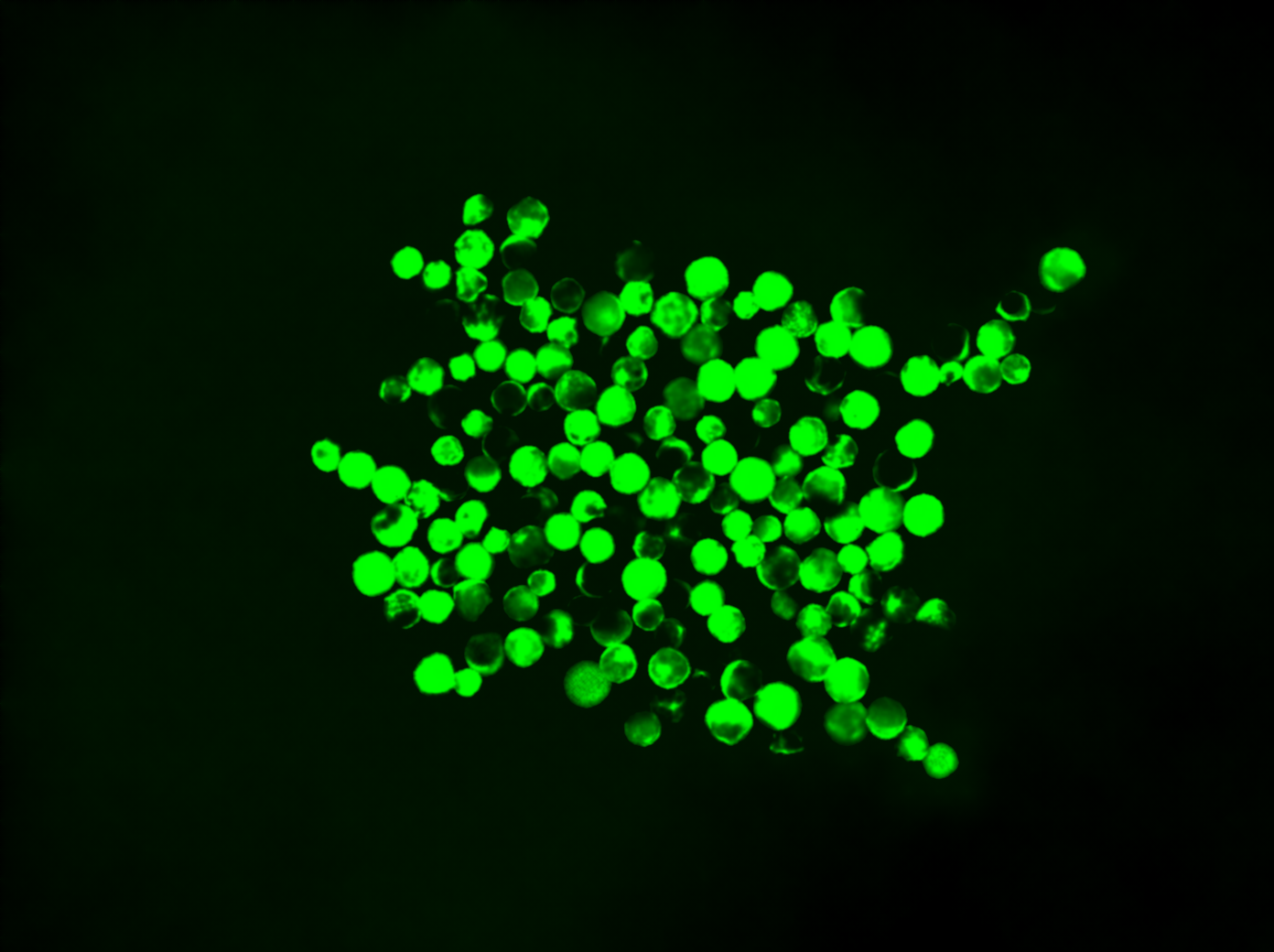

Supplement: Additional file 5 — The zip archive contains simulated images showing protoplasts with corresponding ground truth. (ZIP 72704 kb) [file 12859_2017_1591_MOESM5_ESM.zip › simulated protoplasts/overlapping/overlapping014.png]

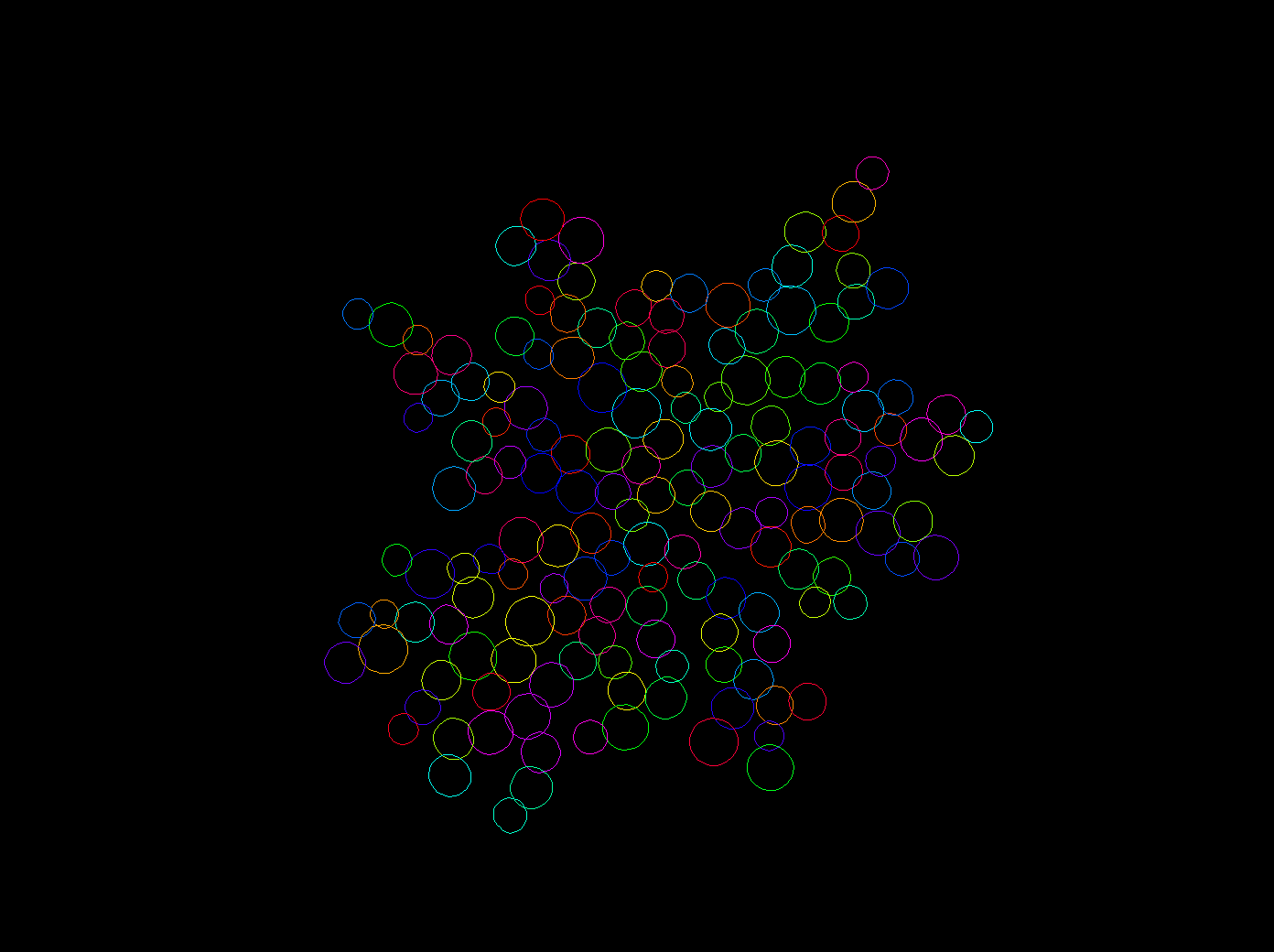

Supplement: Additional file 5 — The zip archive contains simulated images showing protoplasts with corresponding ground truth. (ZIP 72704 kb) [file 12859_2017_1591_MOESM5_ESM.zip › simulated protoplasts/overlapping/overlapping015 gt.png]

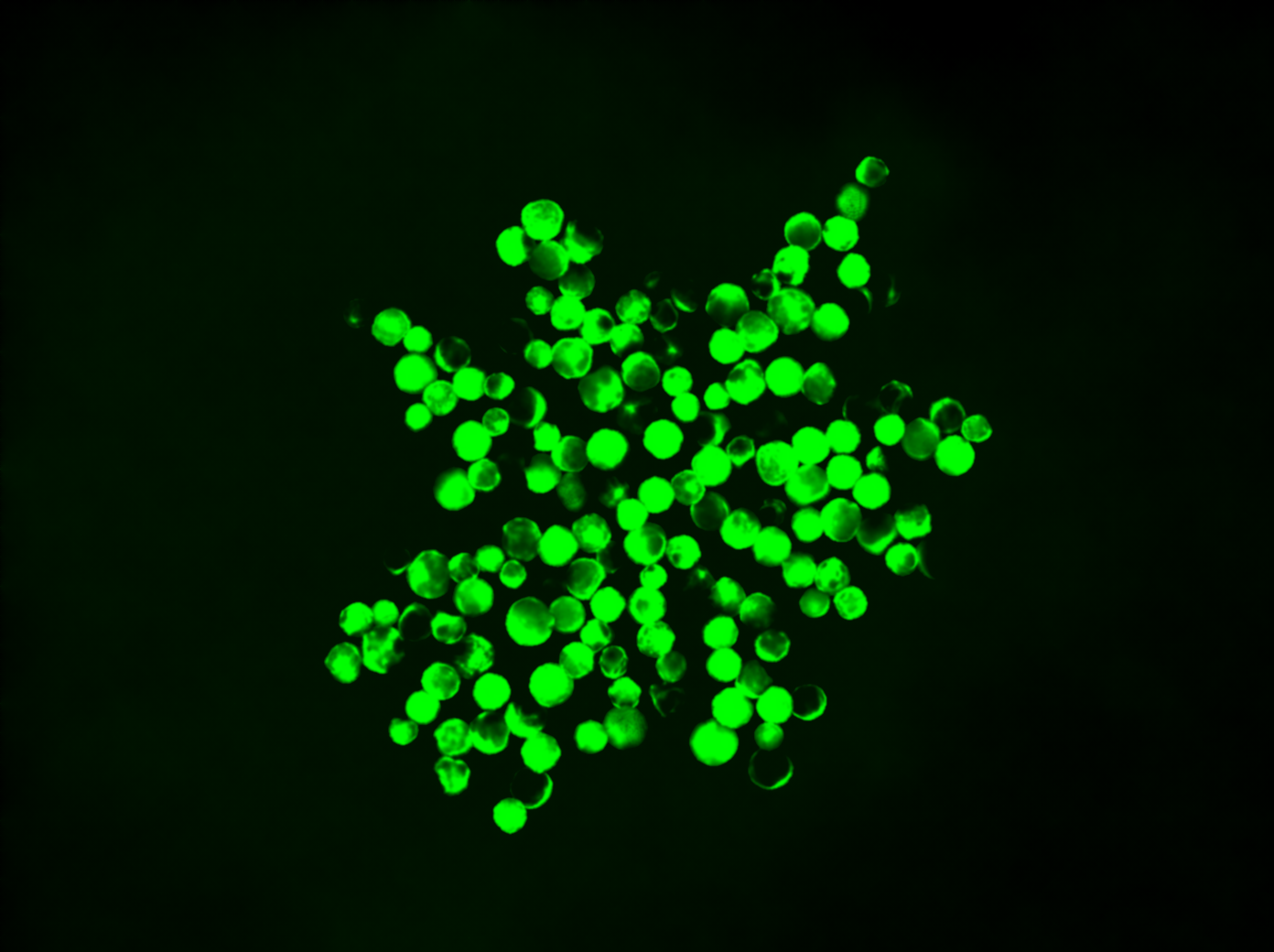

Supplement: Additional file 5 — The zip archive contains simulated images showing protoplasts with corresponding ground truth. (ZIP 72704 kb) [file 12859_2017_1591_MOESM5_ESM.zip › simulated protoplasts/overlapping/overlapping015.png]

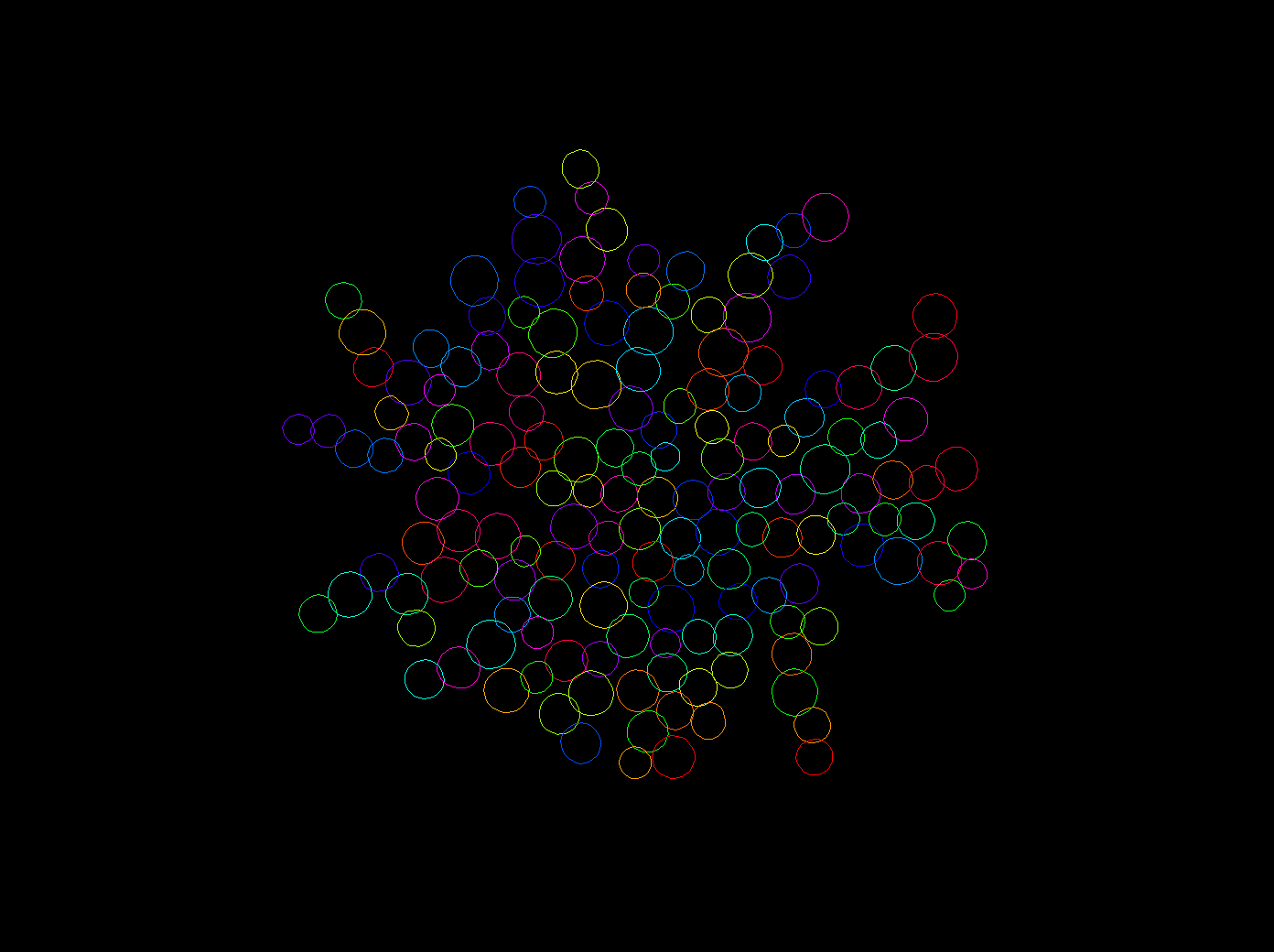

Supplement: Additional file 5 — The zip archive contains simulated images showing protoplasts with corresponding ground truth. (ZIP 72704 kb) [file 12859_2017_1591_MOESM5_ESM.zip › simulated protoplasts/overlapping/overlapping016 gt.png]

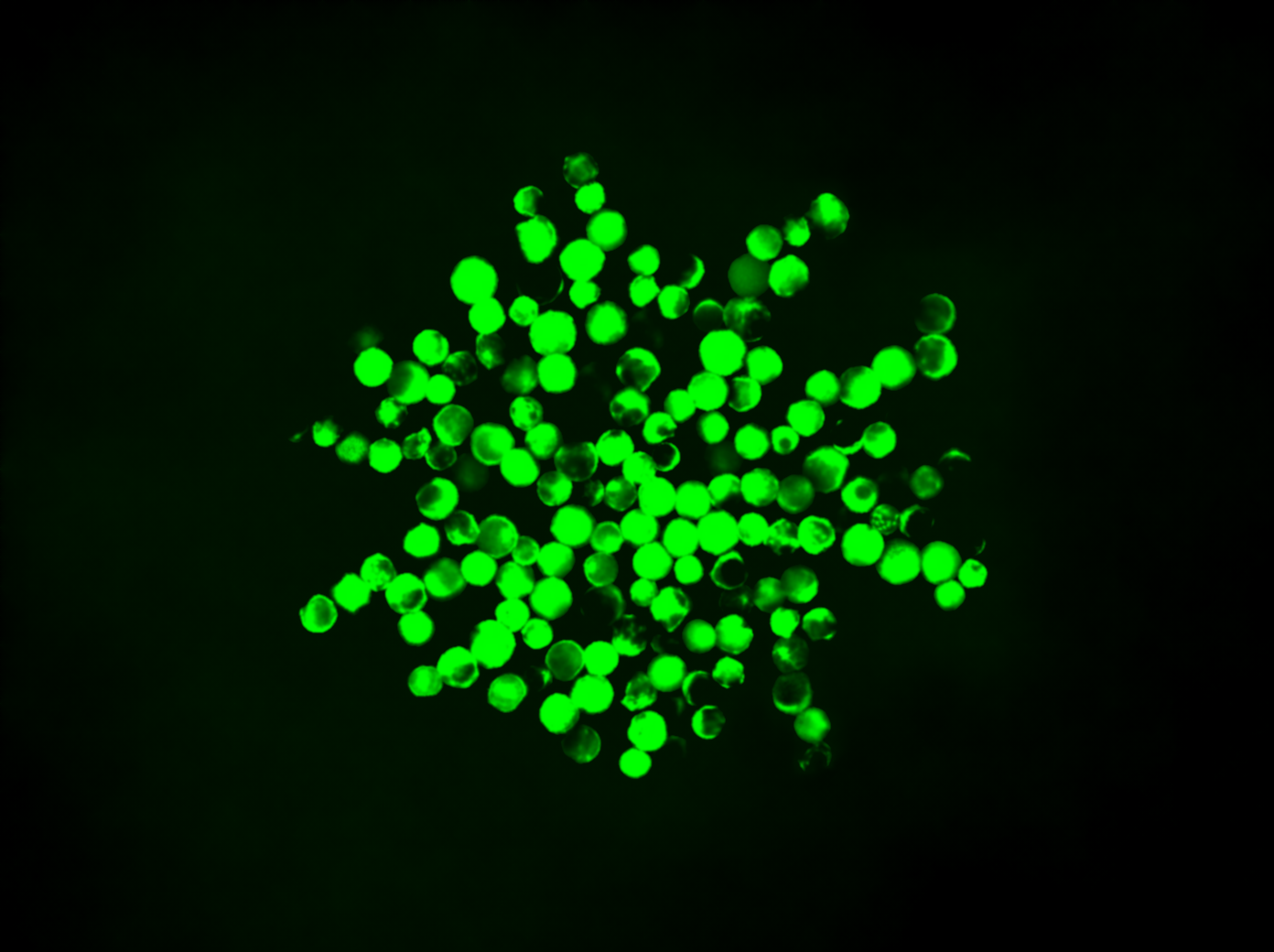

Supplement: Additional file 5 — The zip archive contains simulated images showing protoplasts with corresponding ground truth. (ZIP 72704 kb) [file 12859_2017_1591_MOESM5_ESM.zip › simulated protoplasts/overlapping/overlapping016.png]

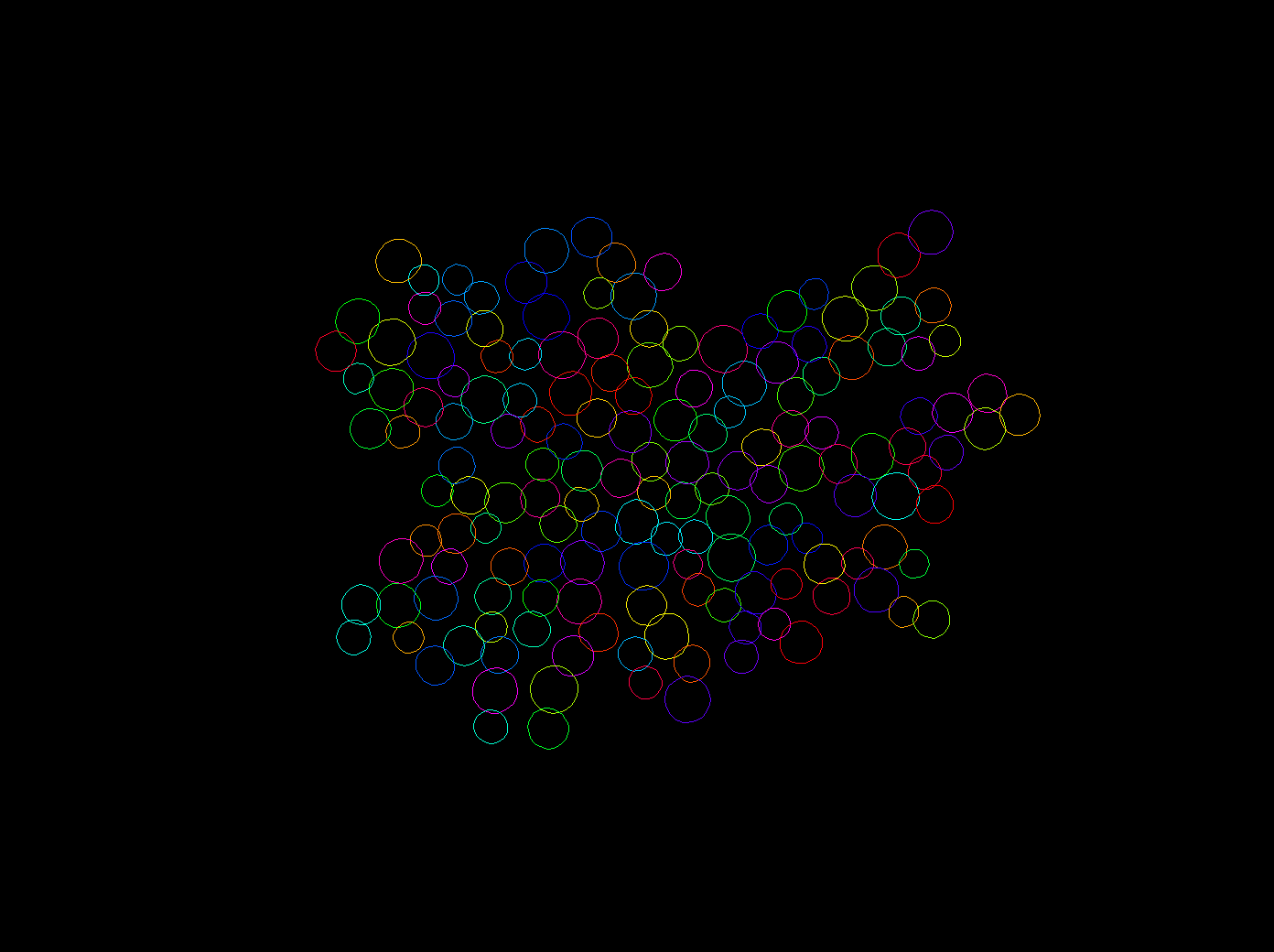

Supplement: Additional file 5 — The zip archive contains simulated images showing protoplasts with corresponding ground truth. (ZIP 72704 kb) [file 12859_2017_1591_MOESM5_ESM.zip › simulated protoplasts/overlapping/overlapping017 gt.png]

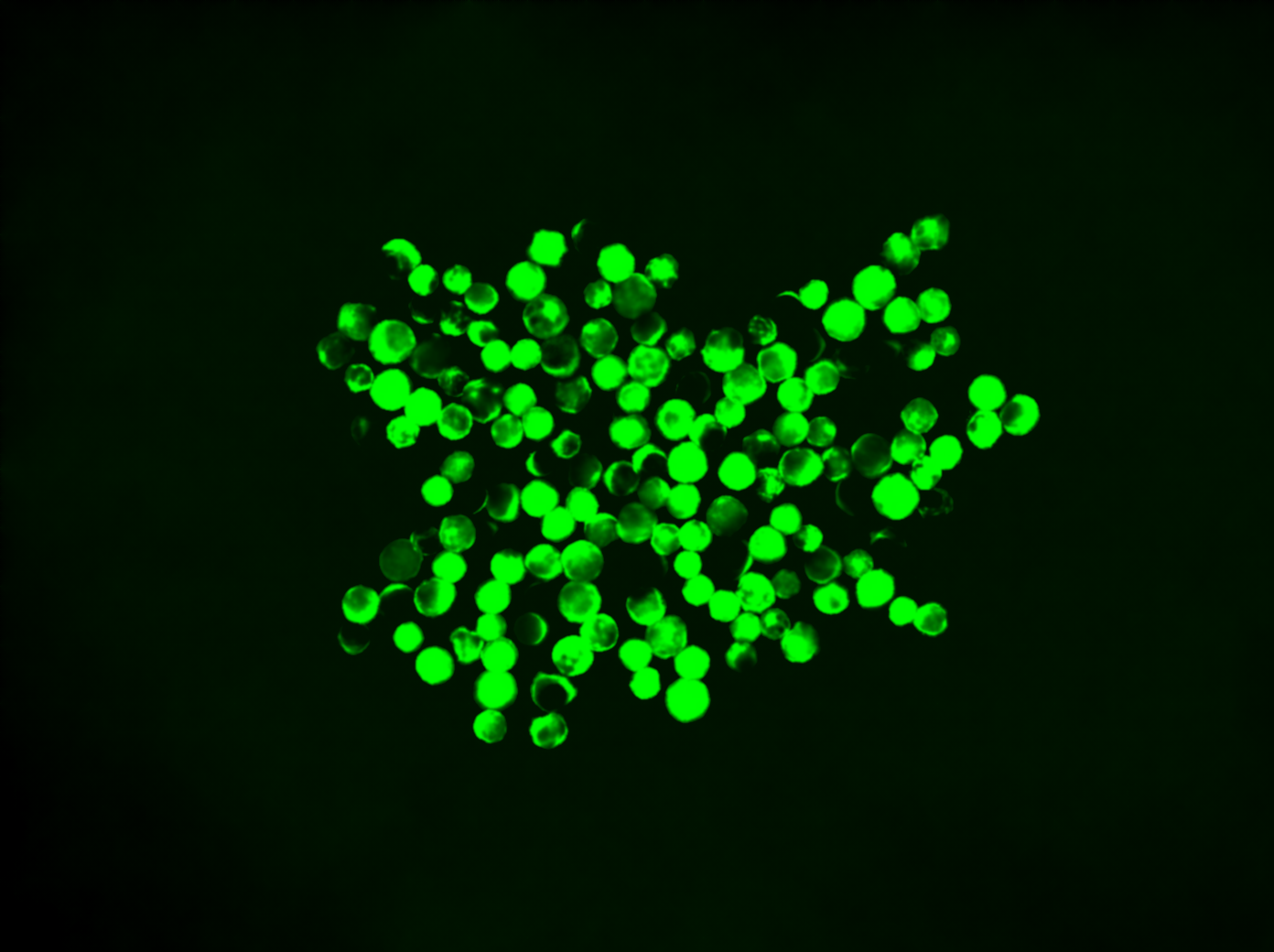

Supplement: Additional file 5 — The zip archive contains simulated images showing protoplasts with corresponding ground truth. (ZIP 72704 kb) [file 12859_2017_1591_MOESM5_ESM.zip › simulated protoplasts/overlapping/overlapping017.png]

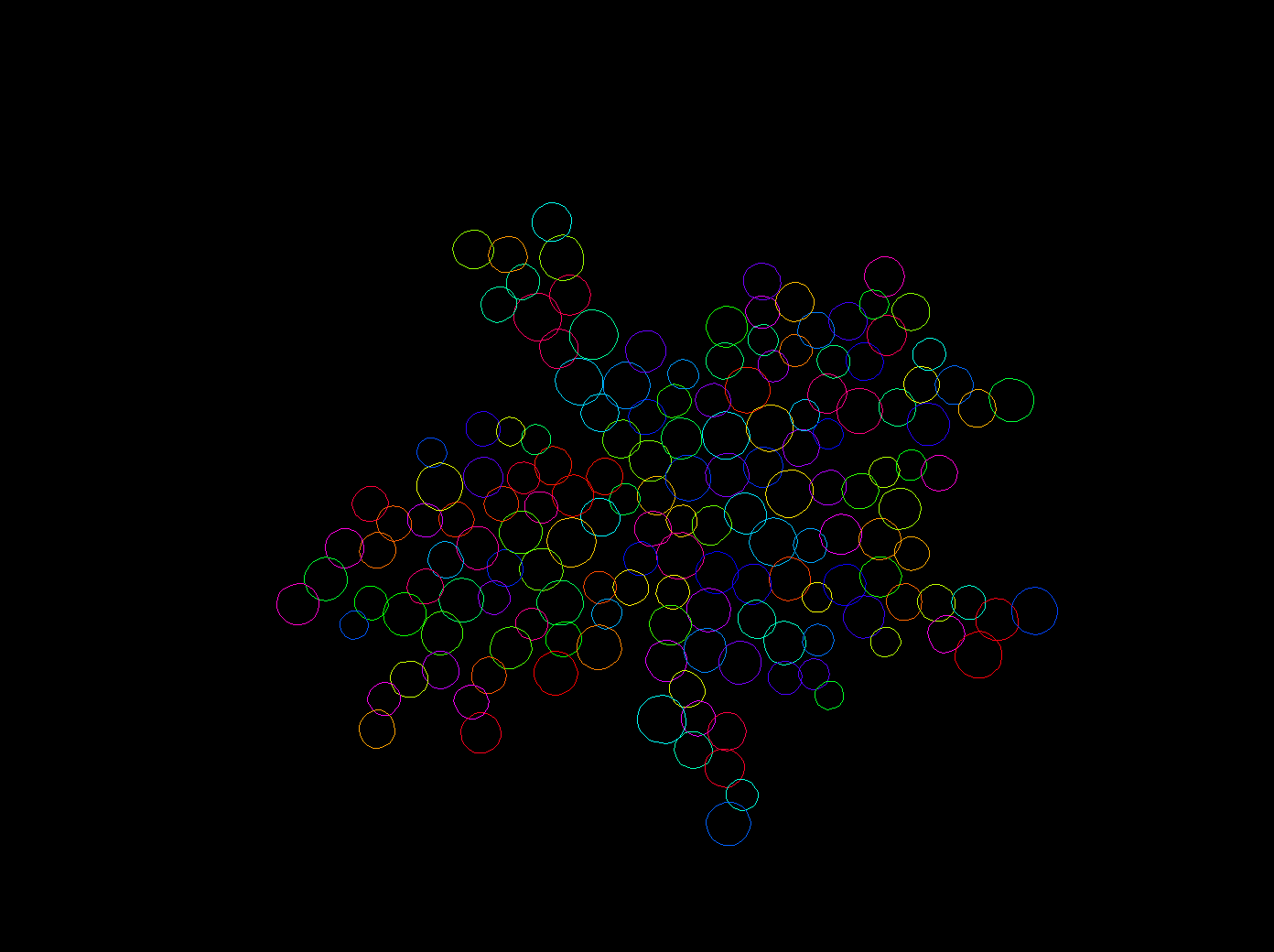

Supplement: Additional file 5 — The zip archive contains simulated images showing protoplasts with corresponding ground truth. (ZIP 72704 kb) [file 12859_2017_1591_MOESM5_ESM.zip › simulated protoplasts/overlapping/overlapping018 gt.png]

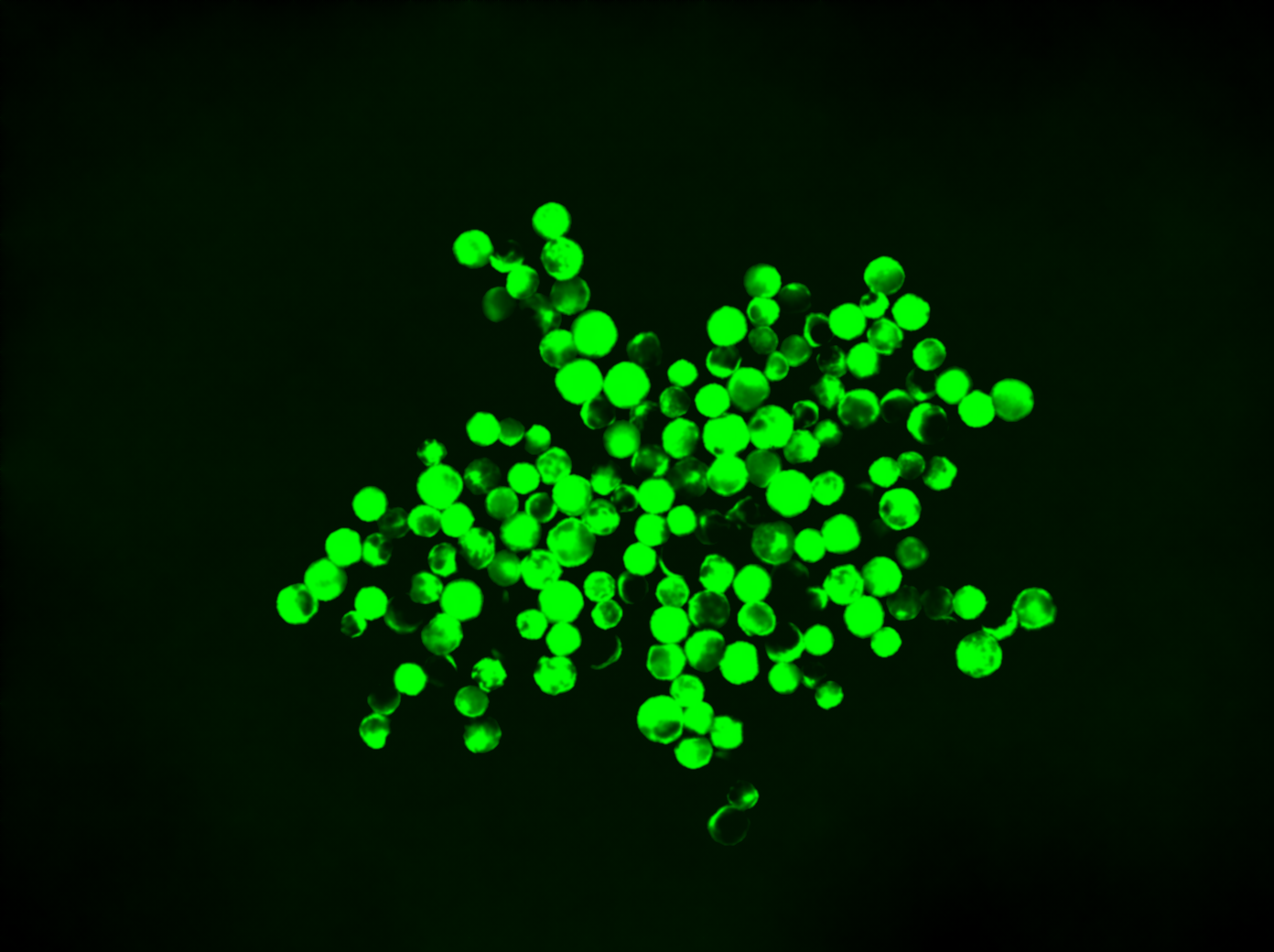

Supplement: Additional file 5 — The zip archive contains simulated images showing protoplasts with corresponding ground truth. (ZIP 72704 kb) [file 12859_2017_1591_MOESM5_ESM.zip › simulated protoplasts/overlapping/overlapping018.png]

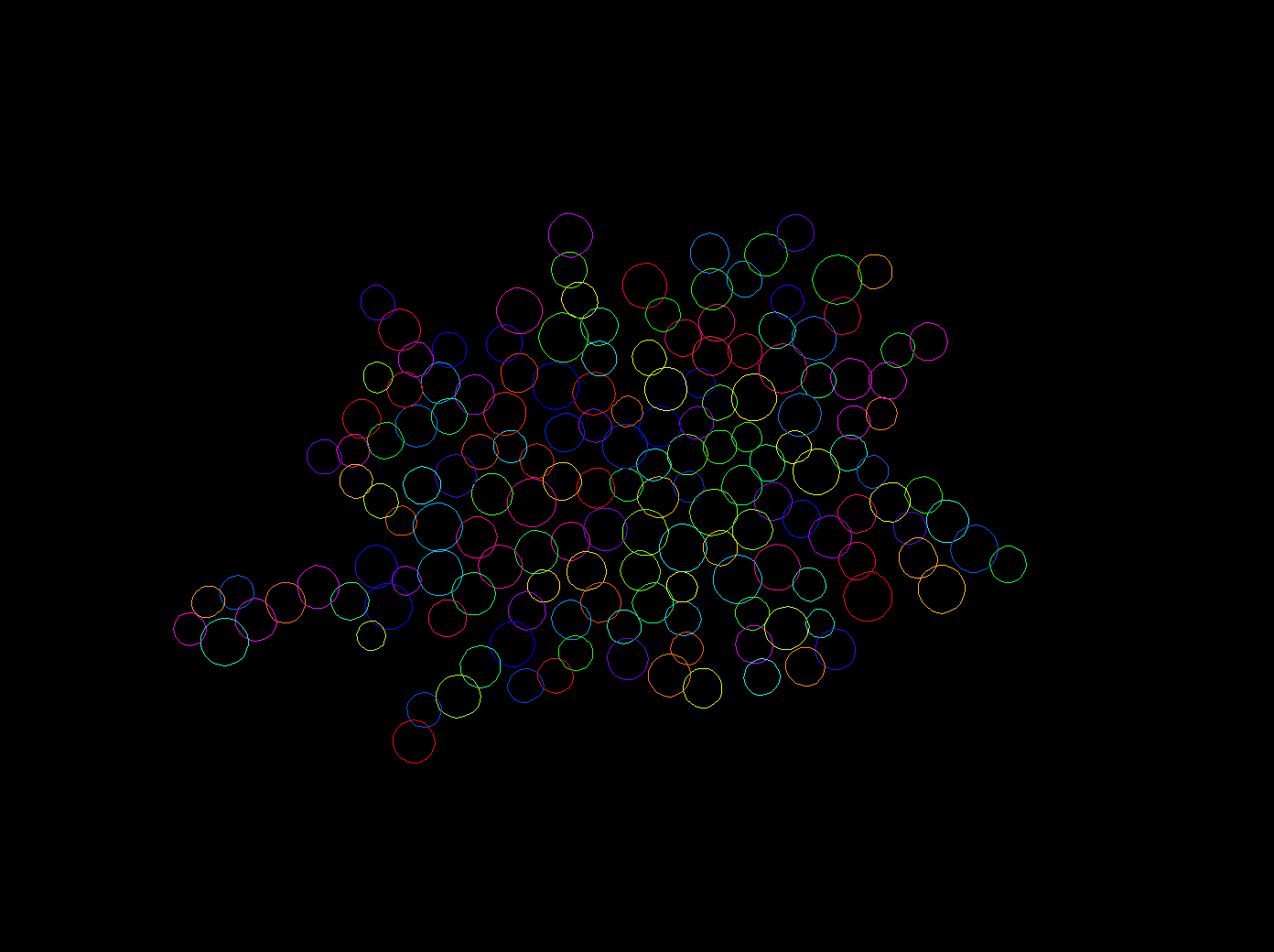

Supplement: Additional file 5 — The zip archive contains simulated images showing protoplasts with corresponding ground truth. (ZIP 72704 kb) [file 12859_2017_1591_MOESM5_ESM.zip › simulated protoplasts/overlapping/overlapping019 gt.png]

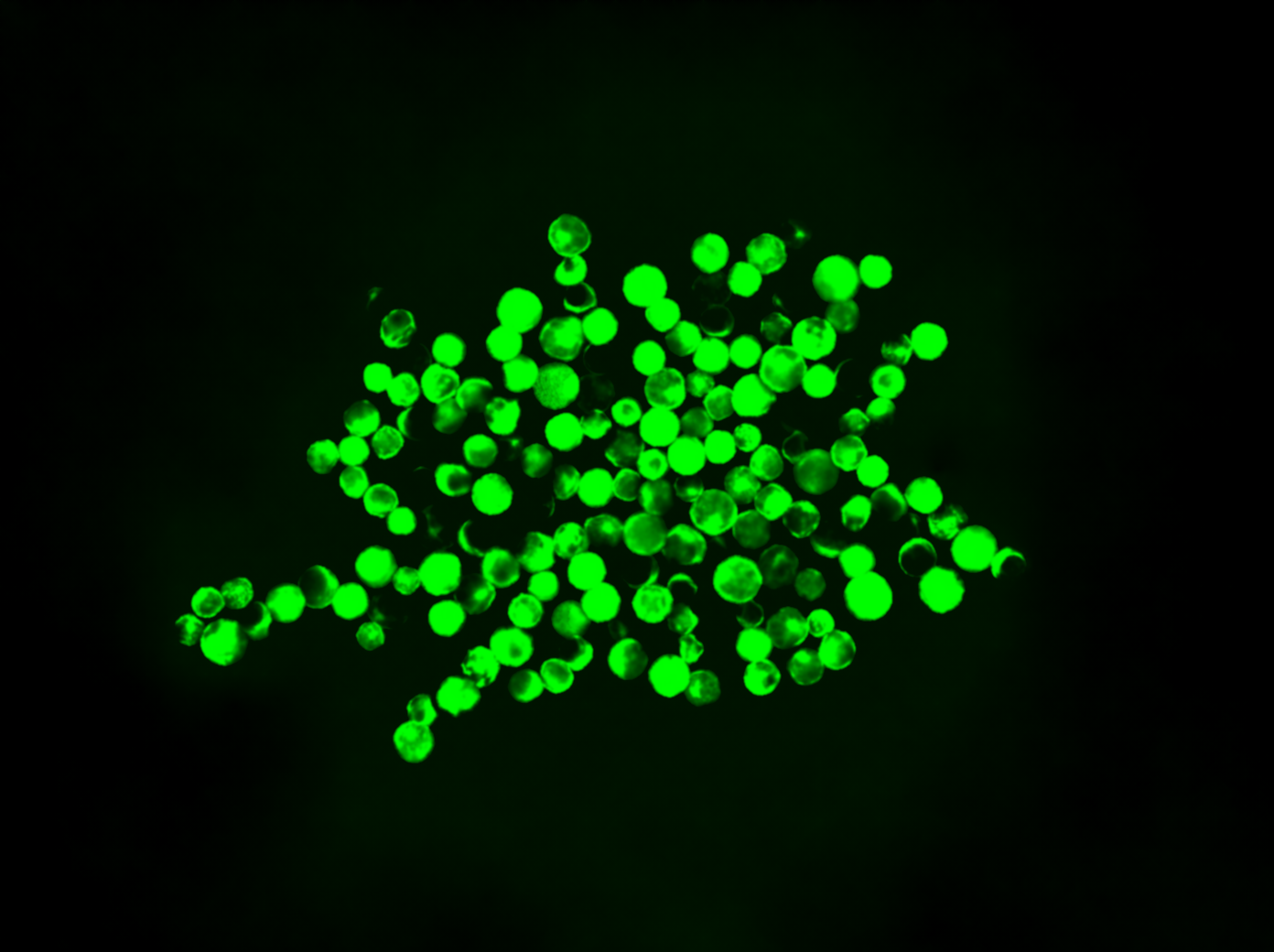

Supplement: Additional file 5 — The zip archive contains simulated images showing protoplasts with corresponding ground truth. (ZIP 72704 kb) [file 12859_2017_1591_MOESM5_ESM.zip › simulated protoplasts/overlapping/overlapping019.png]

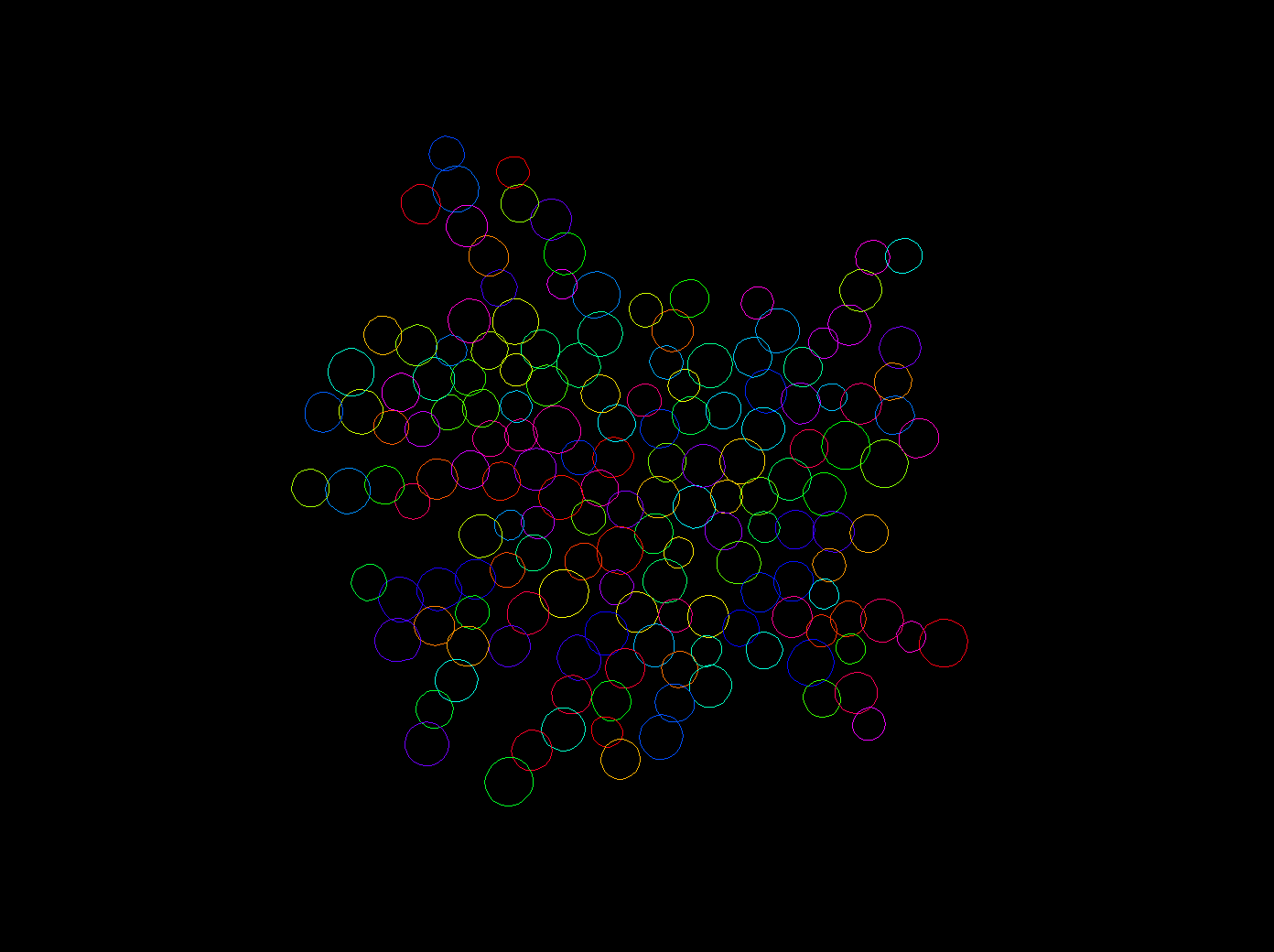

Supplement: Additional file 5 — The zip archive contains simulated images showing protoplasts with corresponding ground truth. (ZIP 72704 kb) [file 12859_2017_1591_MOESM5_ESM.zip › simulated protoplasts/overlapping/overlapping020 gt.png]

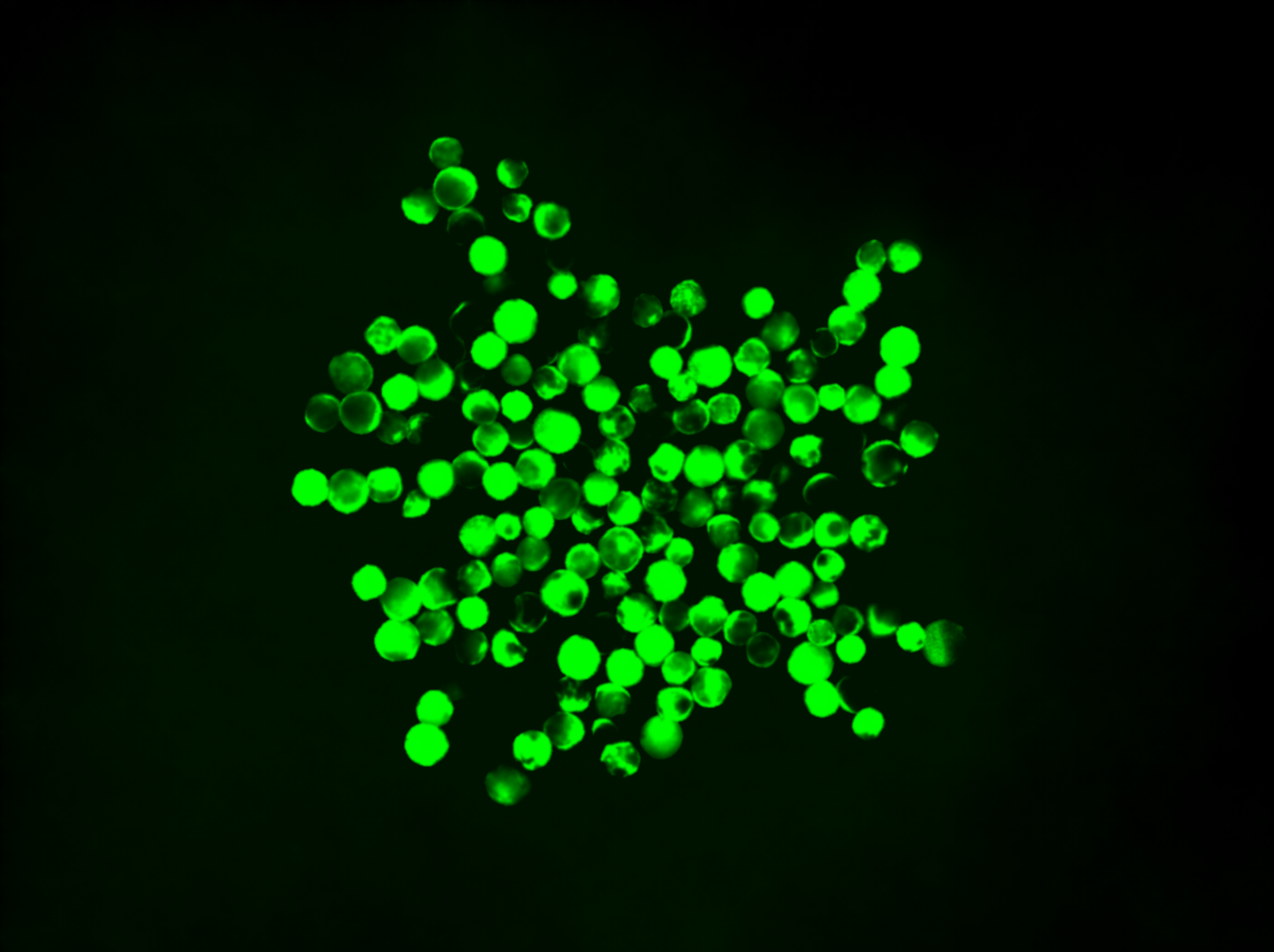

Supplement: Additional file 5 — The zip archive contains simulated images showing protoplasts with corresponding ground truth. (ZIP 72704 kb) [file 12859_2017_1591_MOESM5_ESM.zip › simulated protoplasts/overlapping/overlapping020.png]

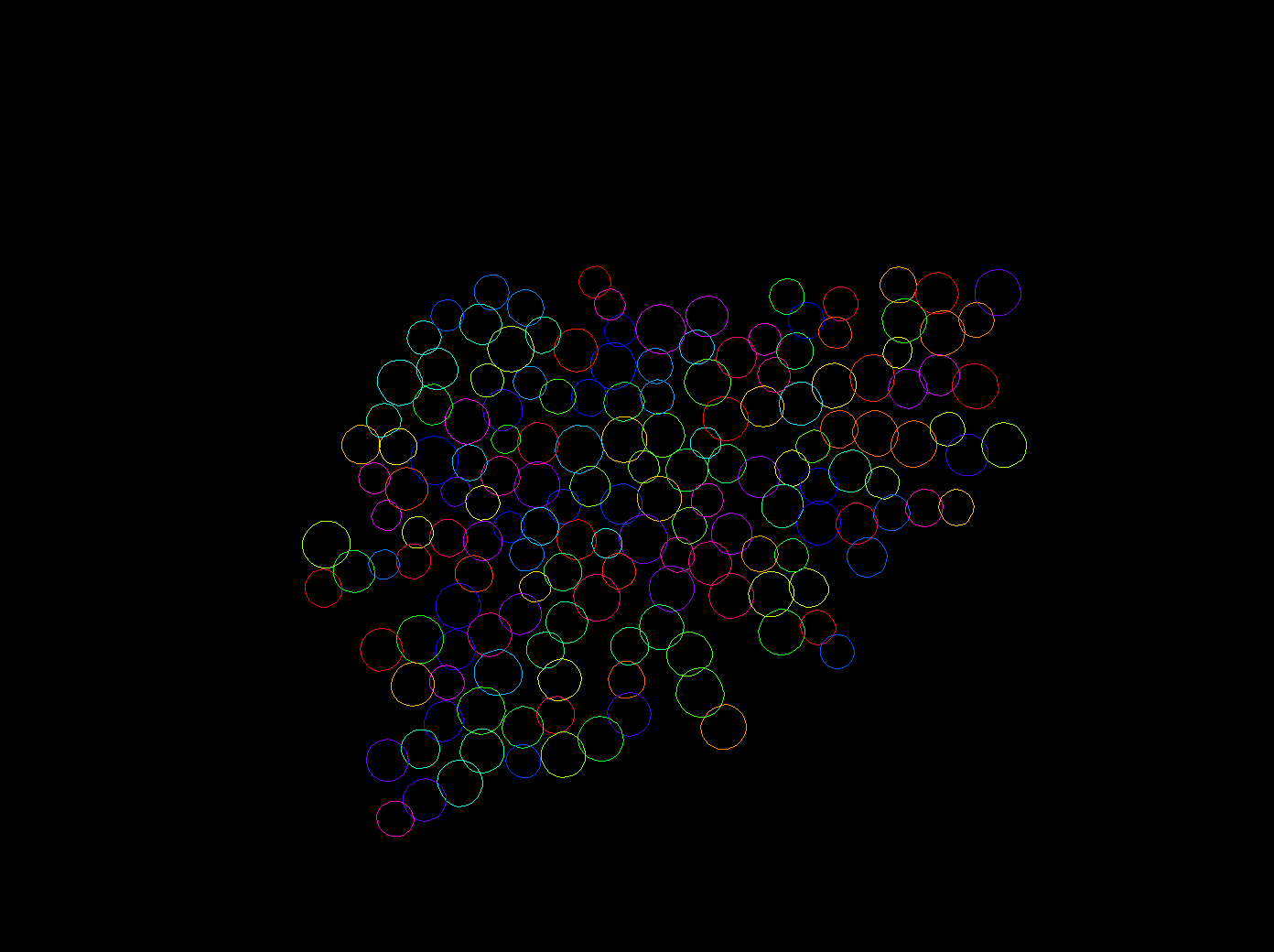

Supplement: Additional file 5 — The zip archive contains simulated images showing protoplasts with corresponding ground truth. (ZIP 72704 kb) [file 12859_2017_1591_MOESM5_ESM.zip › simulated protoplasts/overlapping/overlapping021 gt.png]

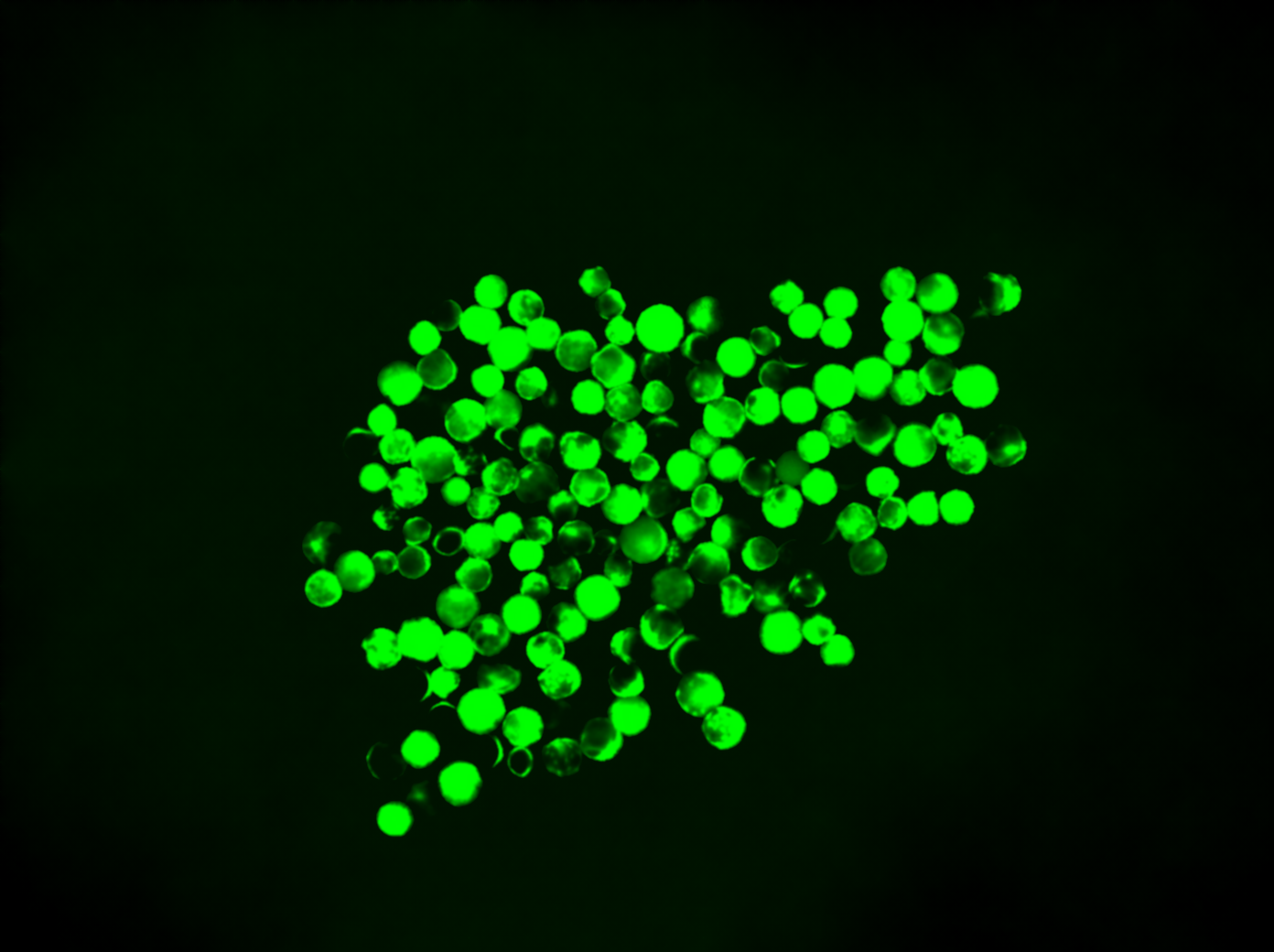

Supplement: Additional file 5 — The zip archive contains simulated images showing protoplasts with corresponding ground truth. (ZIP 72704 kb) [file 12859_2017_1591_MOESM5_ESM.zip › simulated protoplasts/overlapping/overlapping021.png]

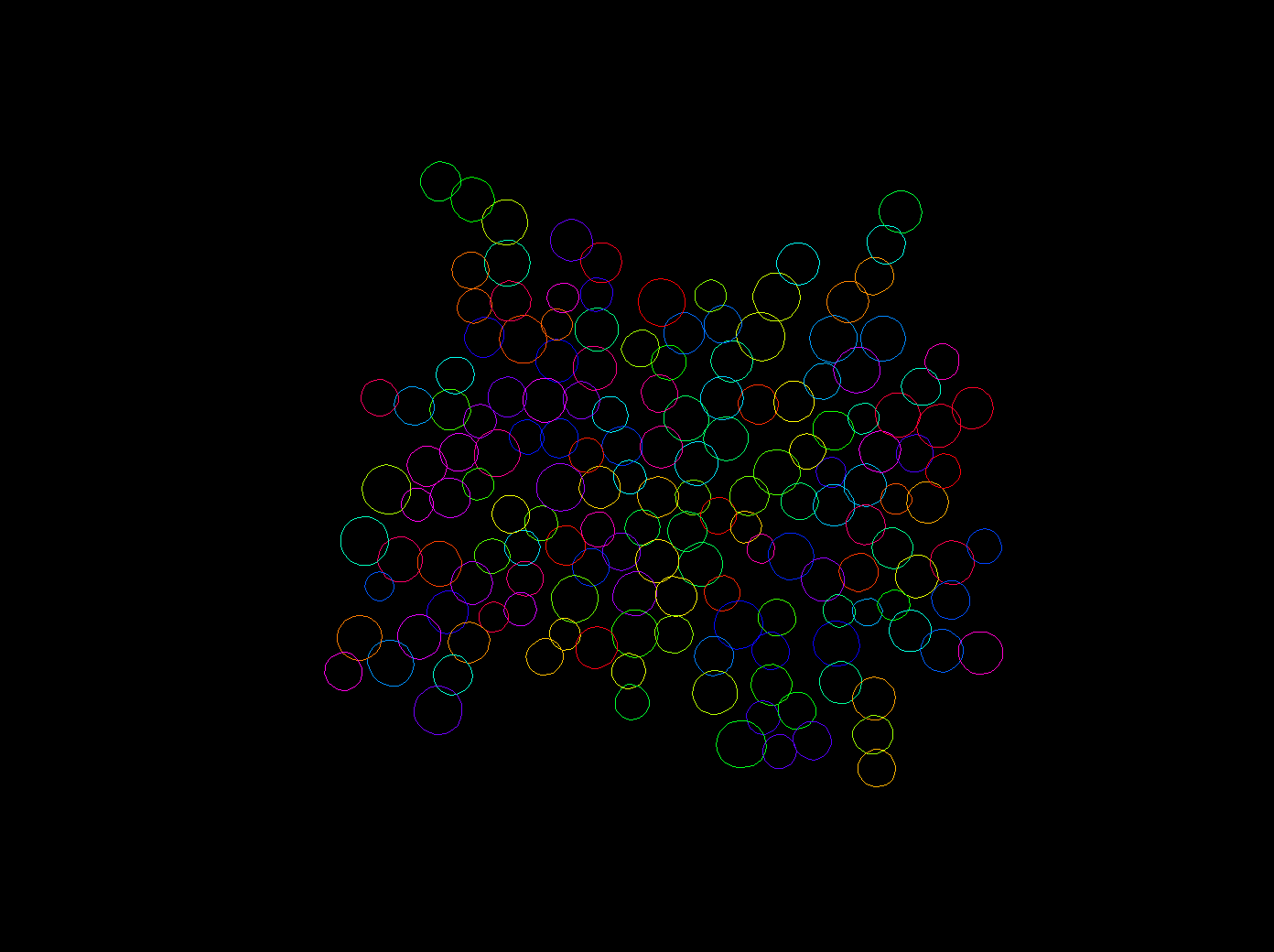

Supplement: Additional file 5 — The zip archive contains simulated images showing protoplasts with corresponding ground truth. (ZIP 72704 kb) [file 12859_2017_1591_MOESM5_ESM.zip › simulated protoplasts/overlapping/overlapping022 gt.png]

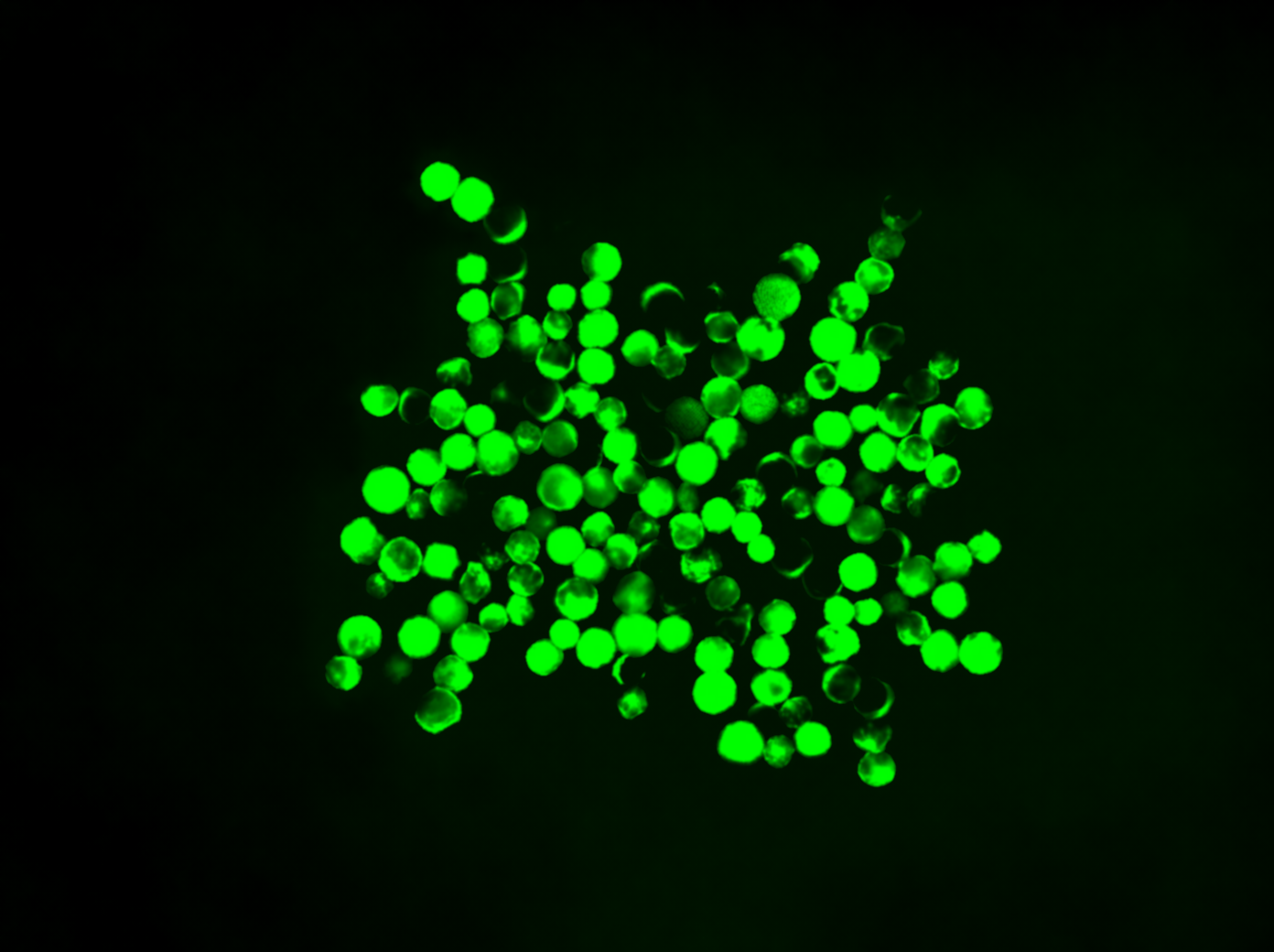

Supplement: Additional file 5 — The zip archive contains simulated images showing protoplasts with corresponding ground truth. (ZIP 72704 kb) [file 12859_2017_1591_MOESM5_ESM.zip › simulated protoplasts/overlapping/overlapping022.png]

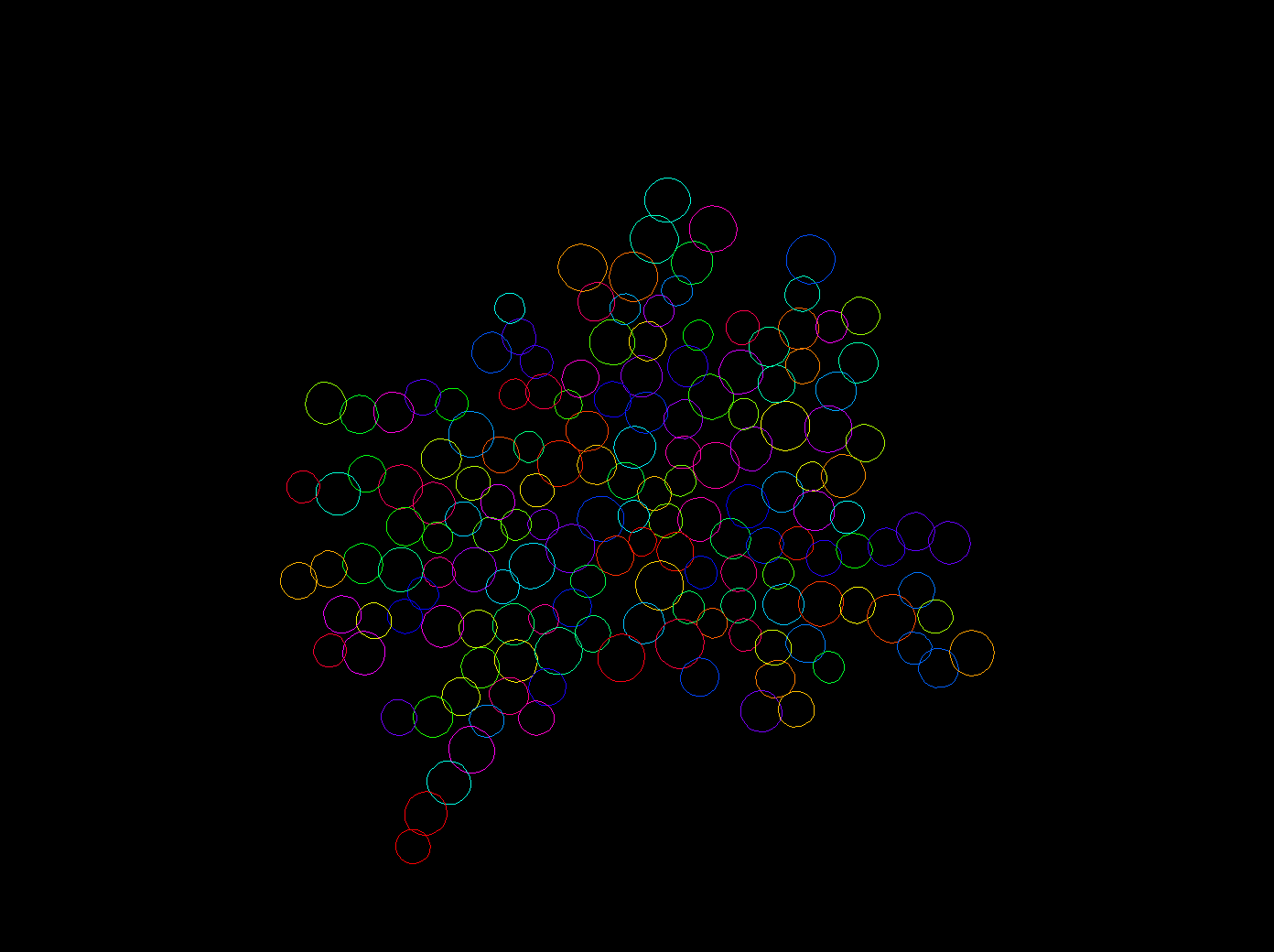

Supplement: Additional file 5 — The zip archive contains simulated images showing protoplasts with corresponding ground truth. (ZIP 72704 kb) [file 12859_2017_1591_MOESM5_ESM.zip › simulated protoplasts/overlapping/overlapping023 gt.png]

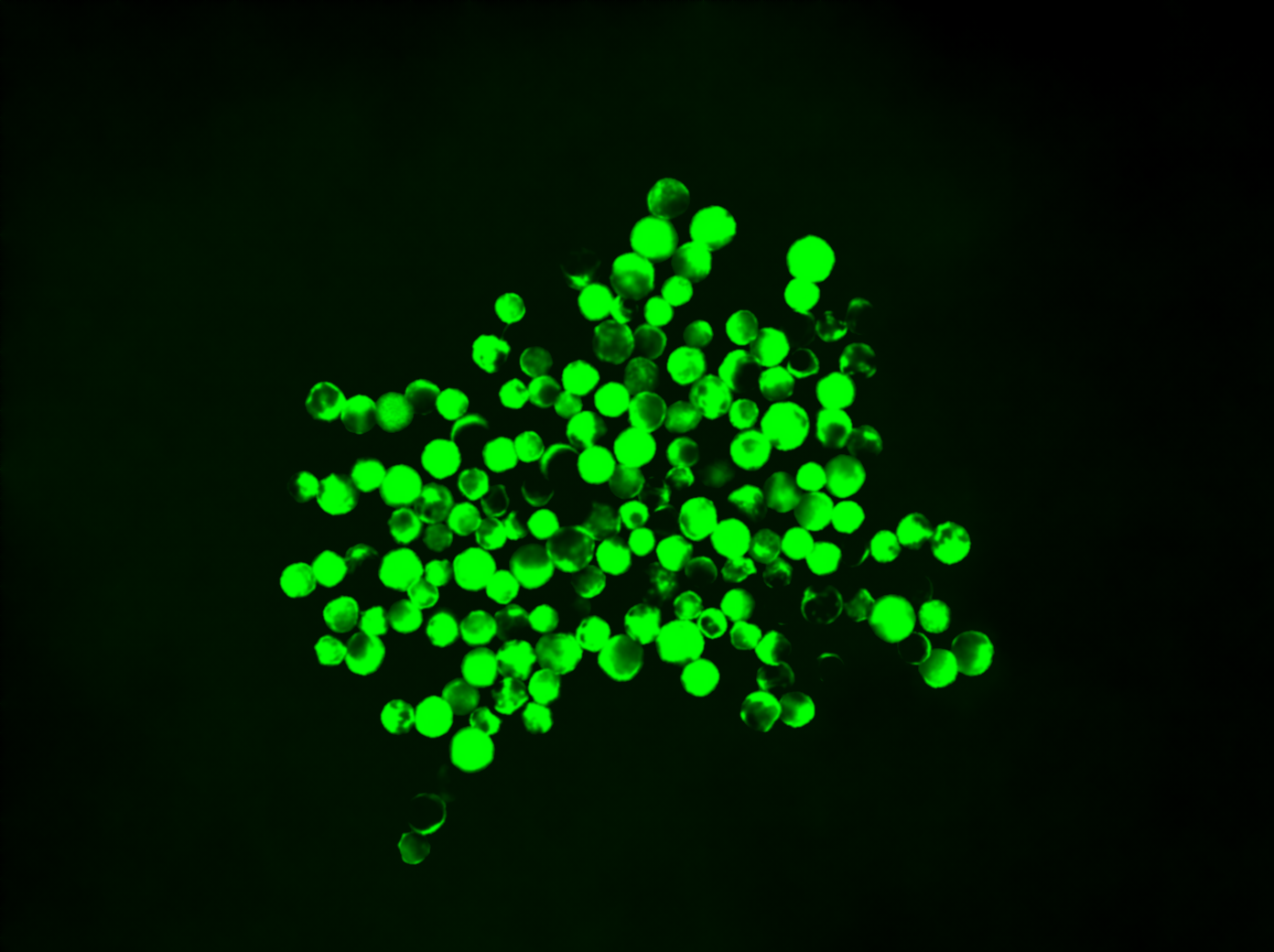

Supplement: Additional file 5 — The zip archive contains simulated images showing protoplasts with corresponding ground truth. (ZIP 72704 kb) [file 12859_2017_1591_MOESM5_ESM.zip › simulated protoplasts/overlapping/overlapping023.png]

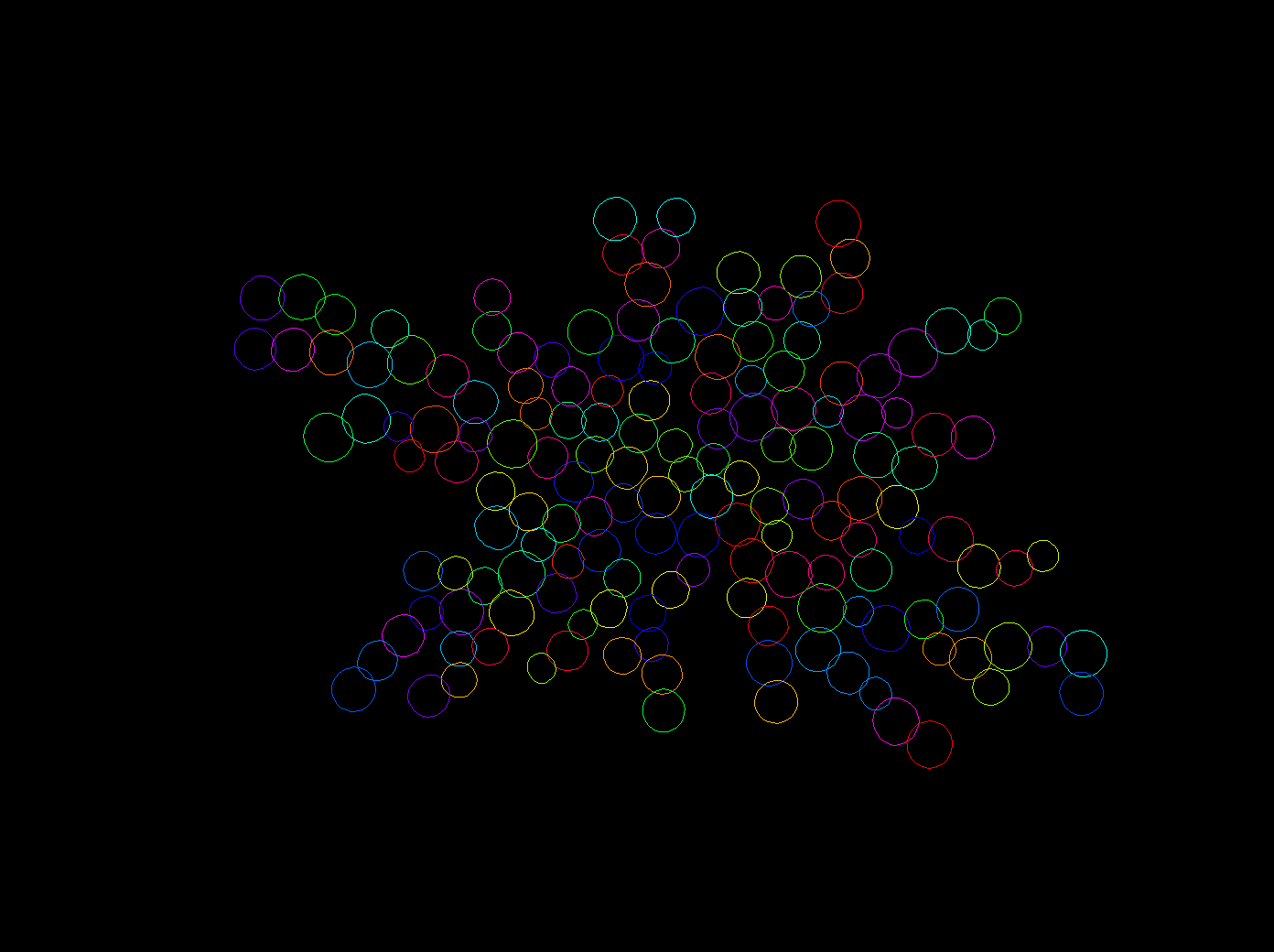

Supplement: Additional file 5 — The zip archive contains simulated images showing protoplasts with corresponding ground truth. (ZIP 72704 kb) [file 12859_2017_1591_MOESM5_ESM.zip › simulated protoplasts/overlapping/overlapping024 gt.png]

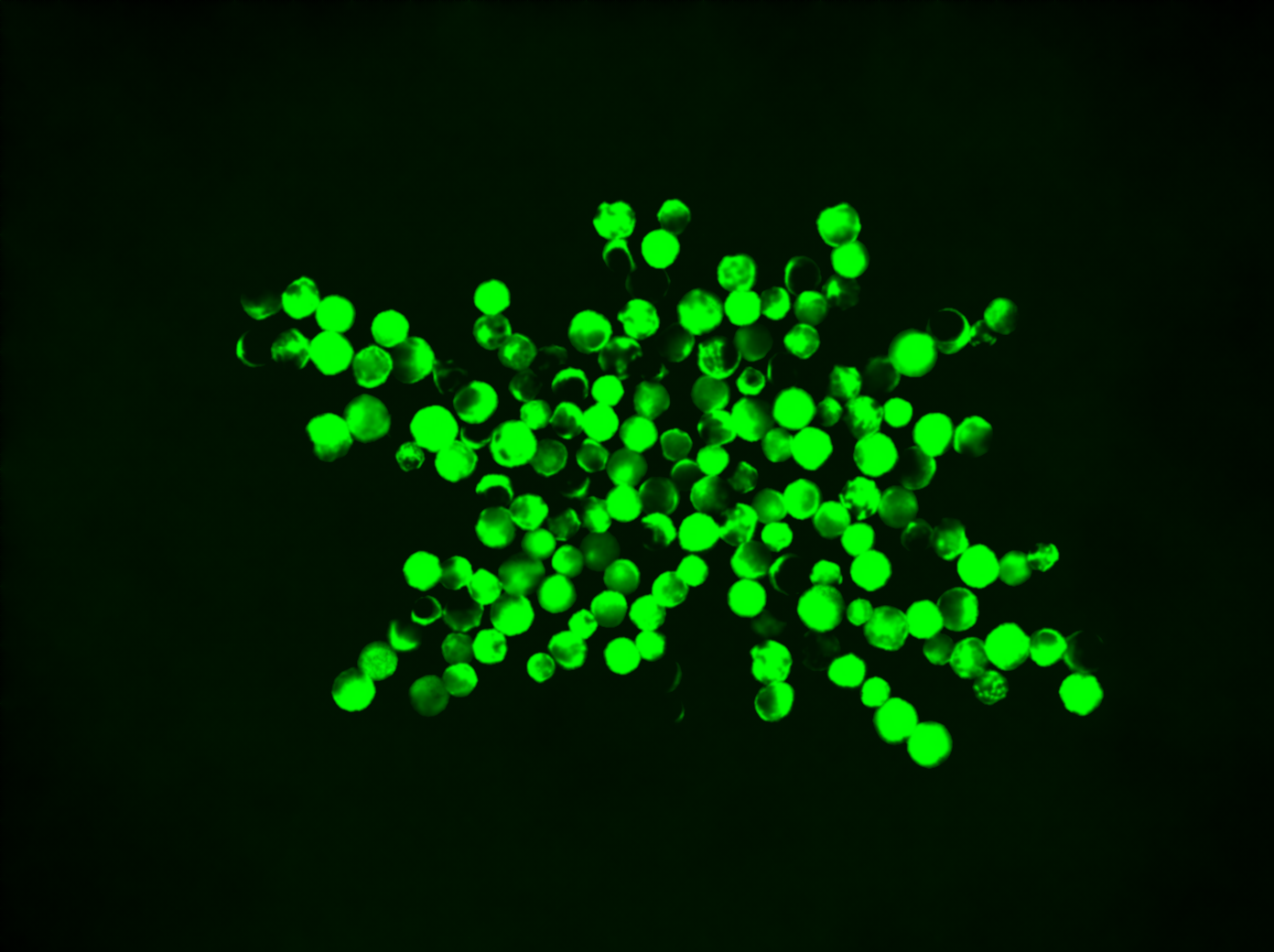

Supplement: Additional file 5 — The zip archive contains simulated images showing protoplasts with corresponding ground truth. (ZIP 72704 kb) [file 12859_2017_1591_MOESM5_ESM.zip › simulated protoplasts/overlapping/overlapping024.png]

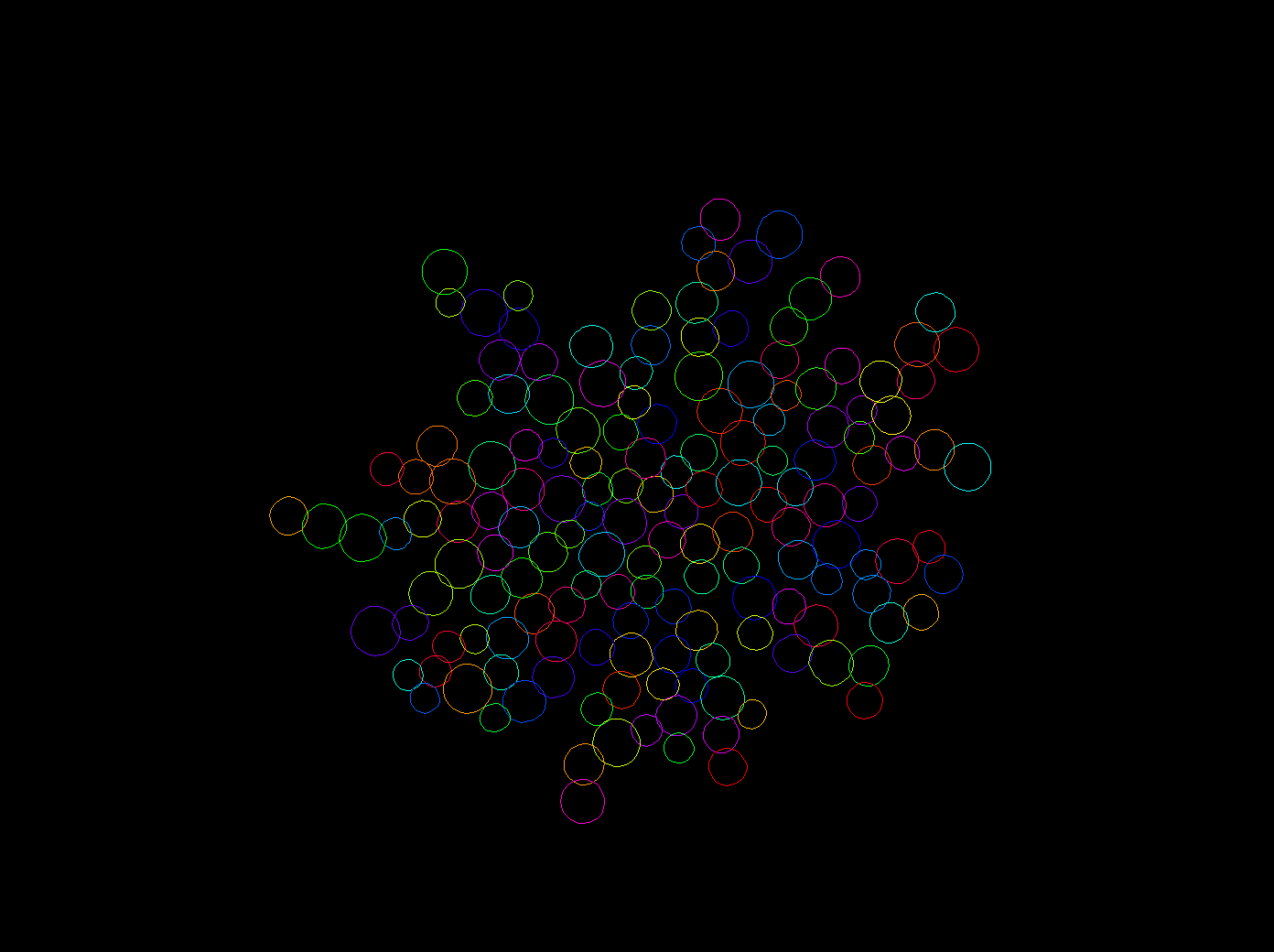

Supplement: Additional file 5 — The zip archive contains simulated images showing protoplasts with corresponding ground truth. (ZIP 72704 kb) [file 12859_2017_1591_MOESM5_ESM.zip › simulated protoplasts/overlapping/overlapping025 gt.png]

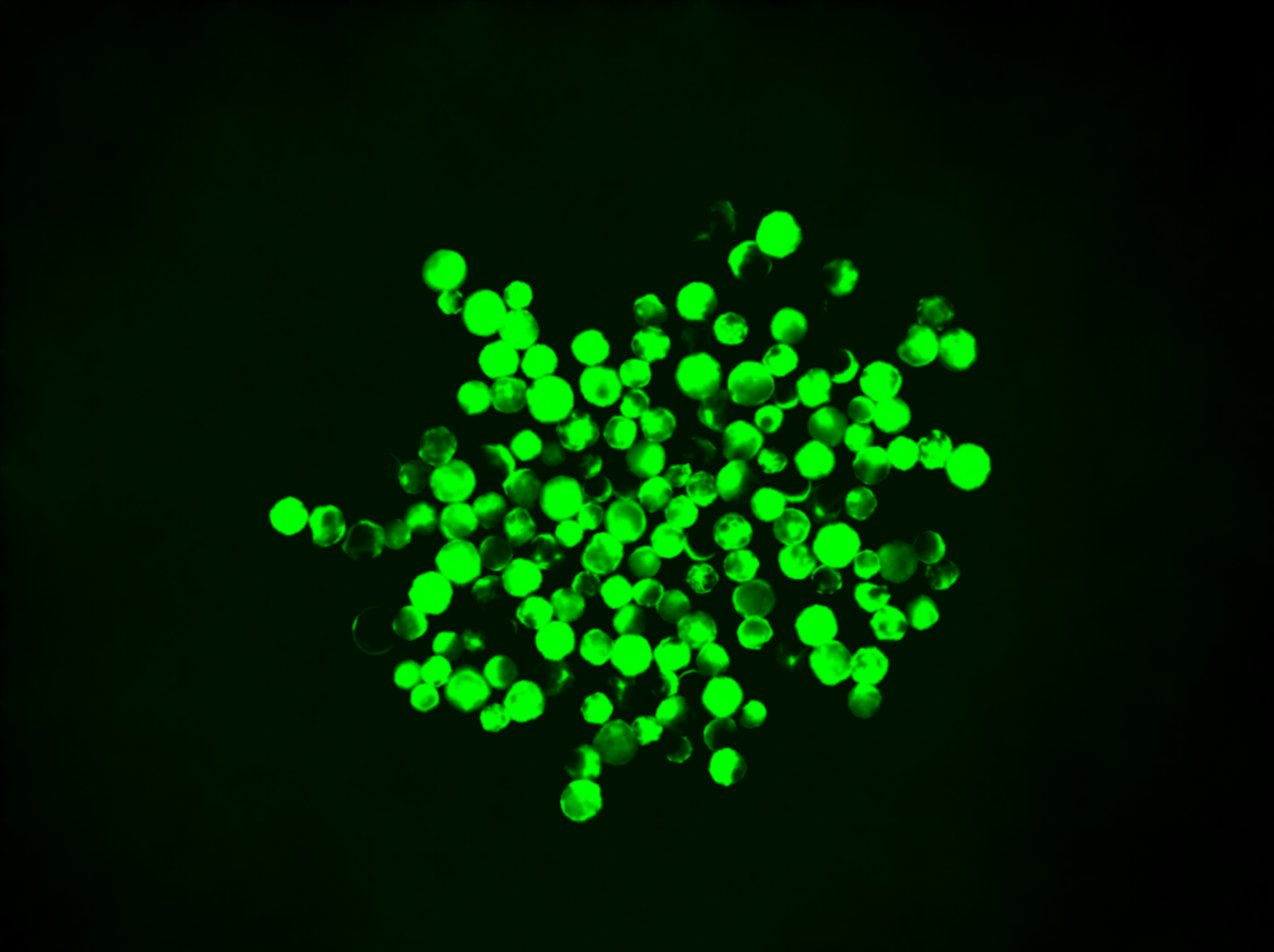

Supplement: Additional file 5 — The zip archive contains simulated images showing protoplasts with corresponding ground truth. (ZIP 72704 kb) [file 12859_2017_1591_MOESM5_ESM.zip › simulated protoplasts/overlapping/overlapping025.png]

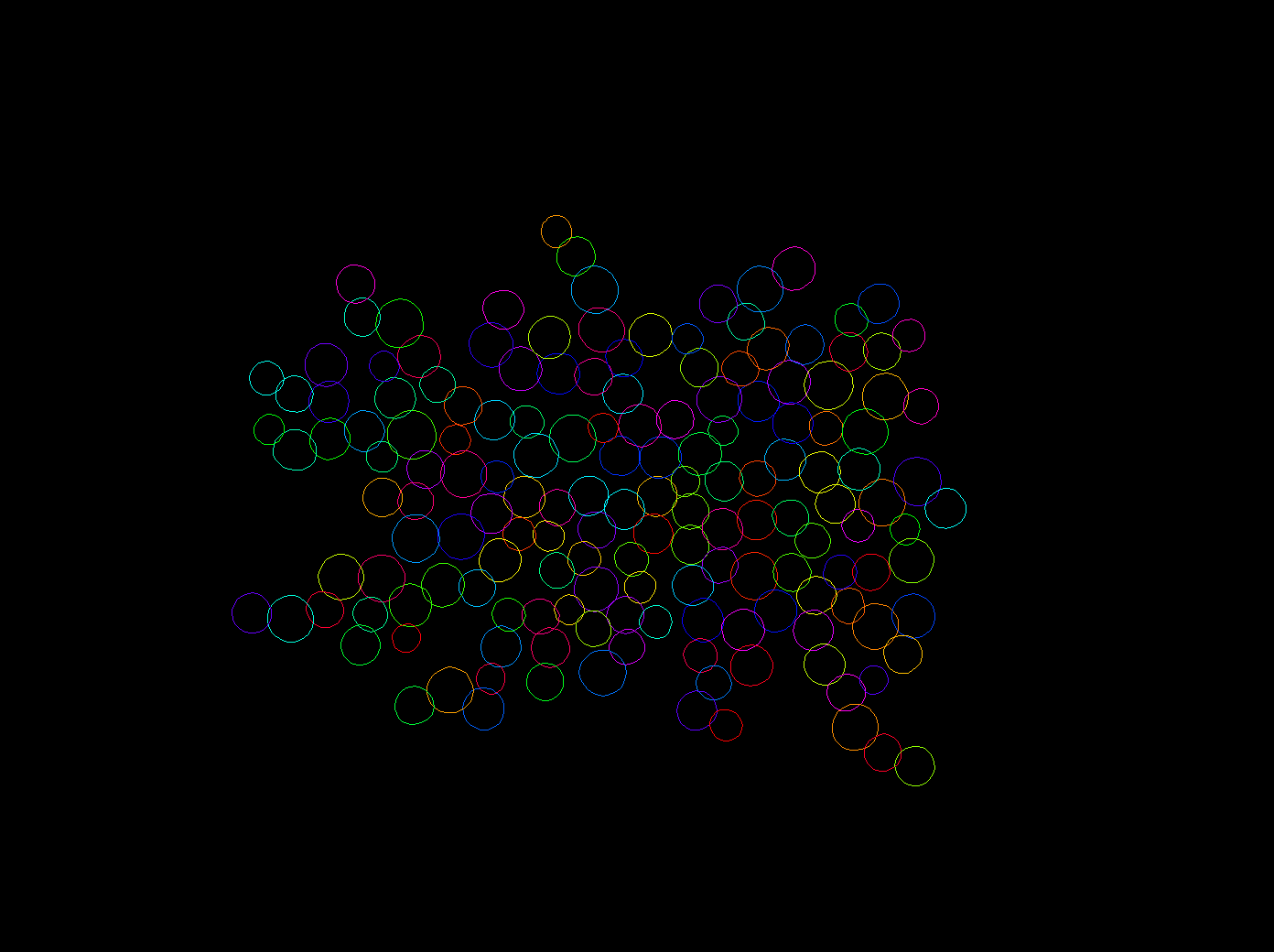

Supplement: Additional file 5 — The zip archive contains simulated images showing protoplasts with corresponding ground truth. (ZIP 72704 kb) [file 12859_2017_1591_MOESM5_ESM.zip › simulated protoplasts/overlapping/overlapping026 gt.png]

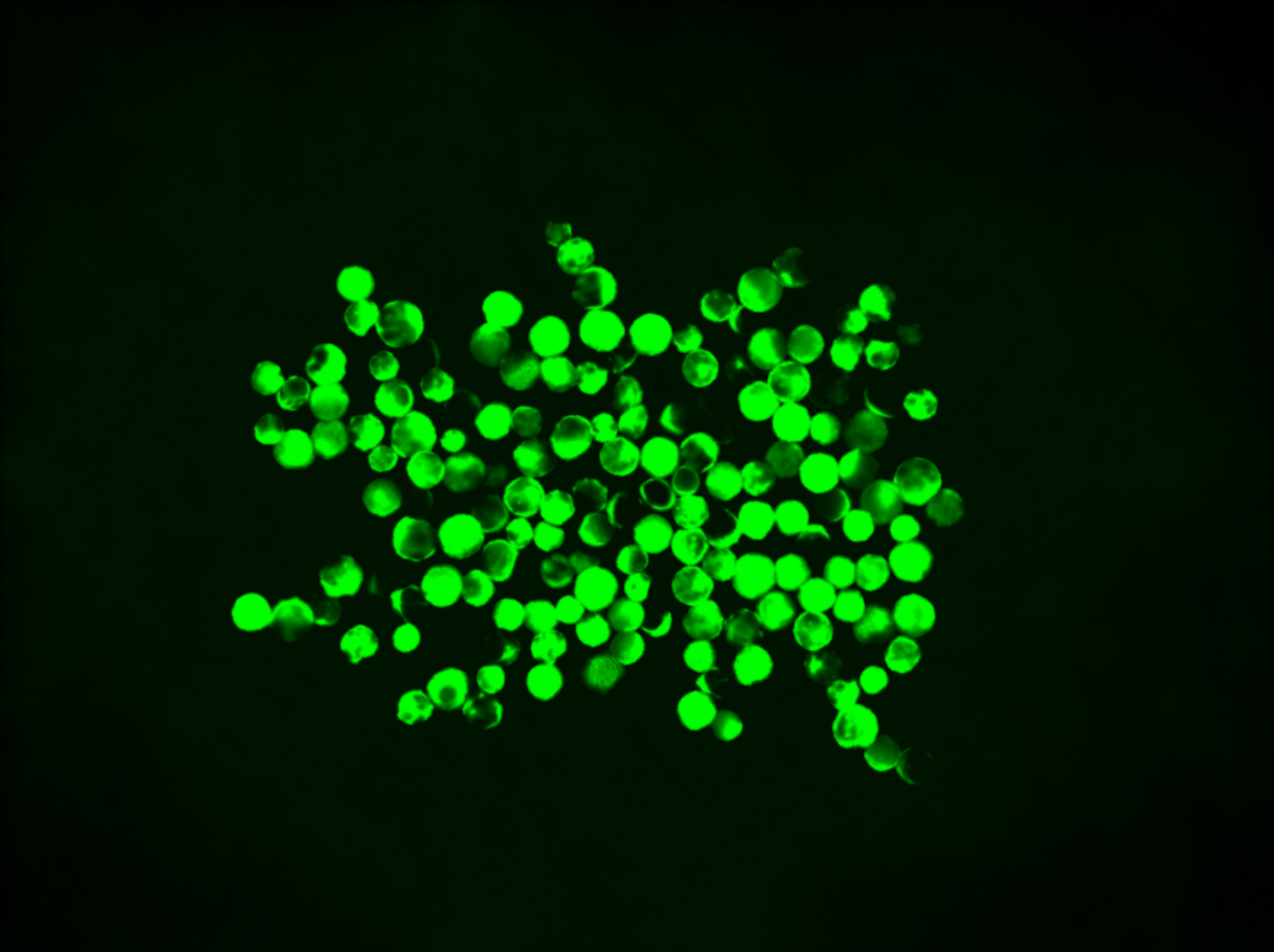

Supplement: Additional file 5 — The zip archive contains simulated images showing protoplasts with corresponding ground truth. (ZIP 72704 kb) [file 12859_2017_1591_MOESM5_ESM.zip › simulated protoplasts/overlapping/overlapping026.png]

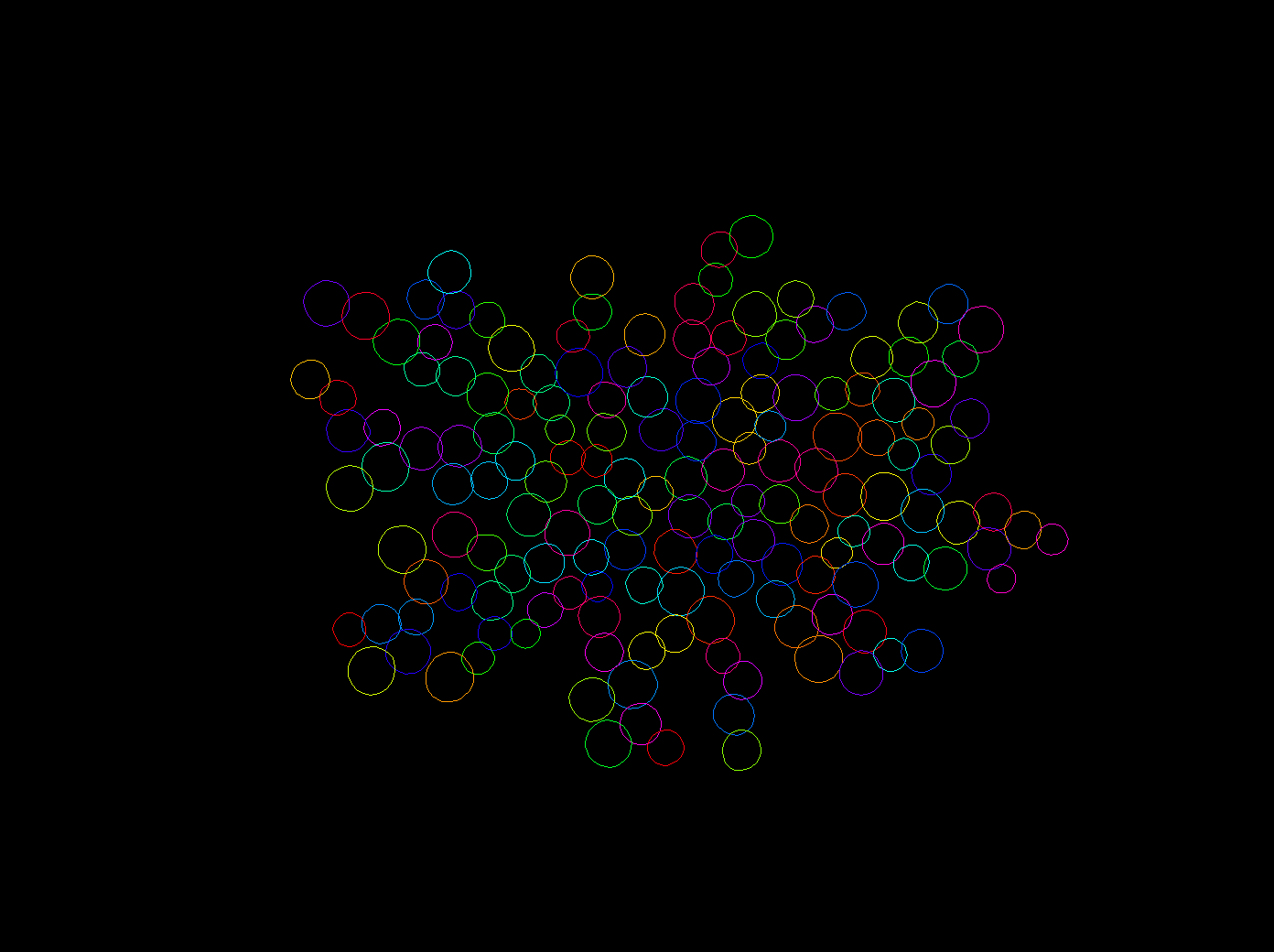

Supplement: Additional file 5 — The zip archive contains simulated images showing protoplasts with corresponding ground truth. (ZIP 72704 kb) [file 12859_2017_1591_MOESM5_ESM.zip › simulated protoplasts/overlapping/overlapping027 gt.png]

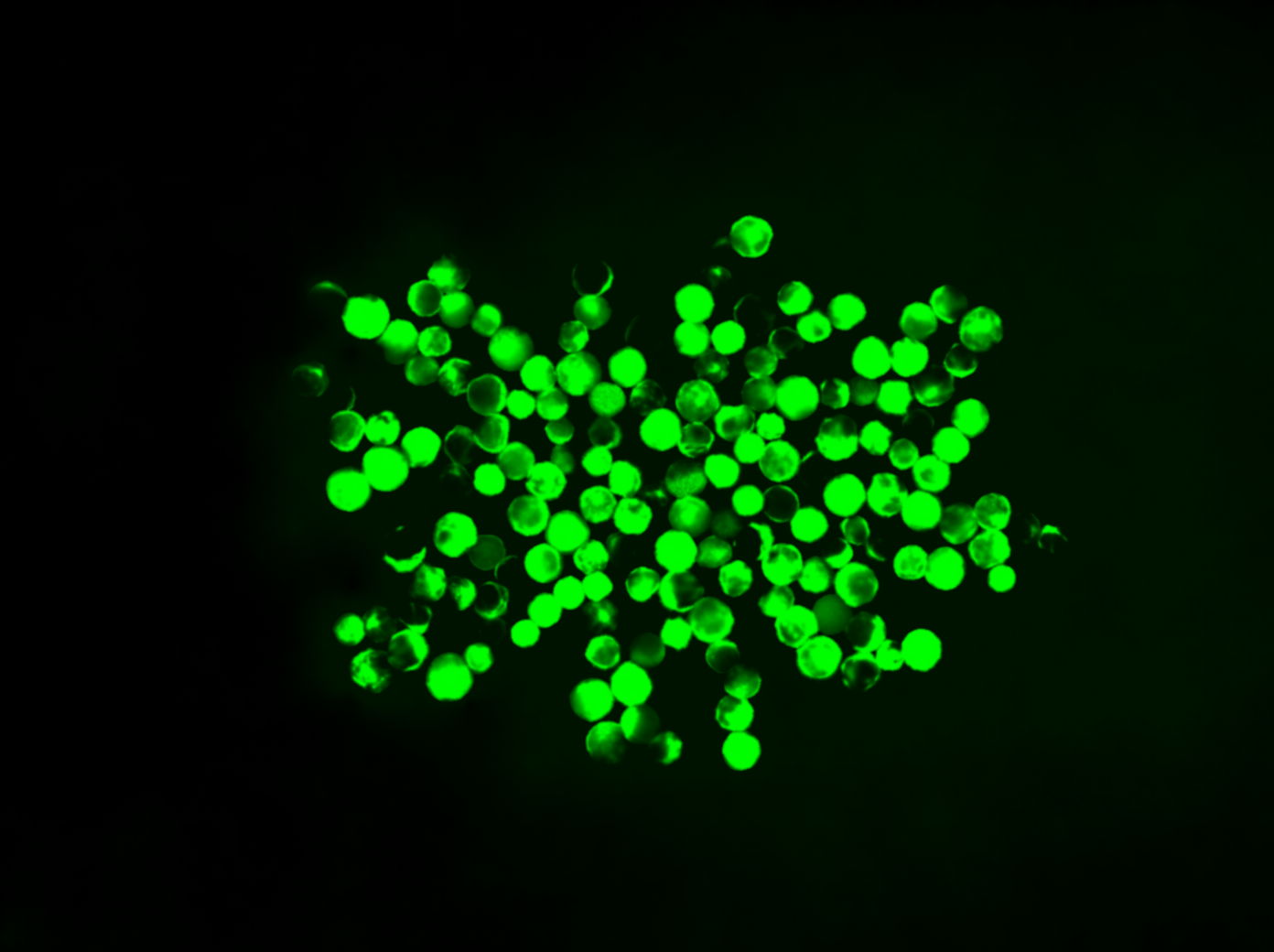

Supplement: Additional file 5 — The zip archive contains simulated images showing protoplasts with corresponding ground truth. (ZIP 72704 kb) [file 12859_2017_1591_MOESM5_ESM.zip › simulated protoplasts/overlapping/overlapping027.png]

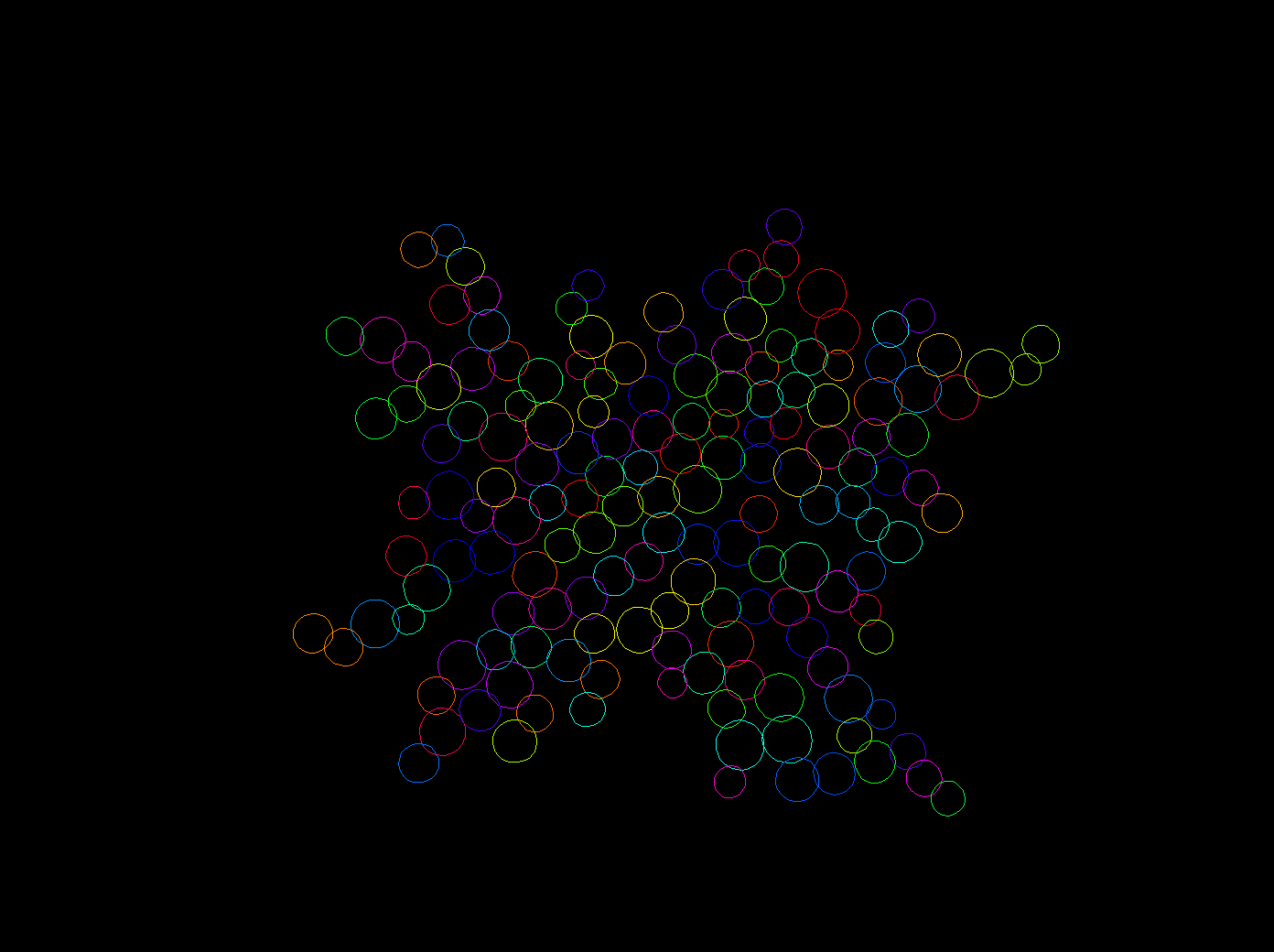

Supplement: Additional file 5 — The zip archive contains simulated images showing protoplasts with corresponding ground truth. (ZIP 72704 kb) [file 12859_2017_1591_MOESM5_ESM.zip › simulated protoplasts/overlapping/overlapping028 gt.png]

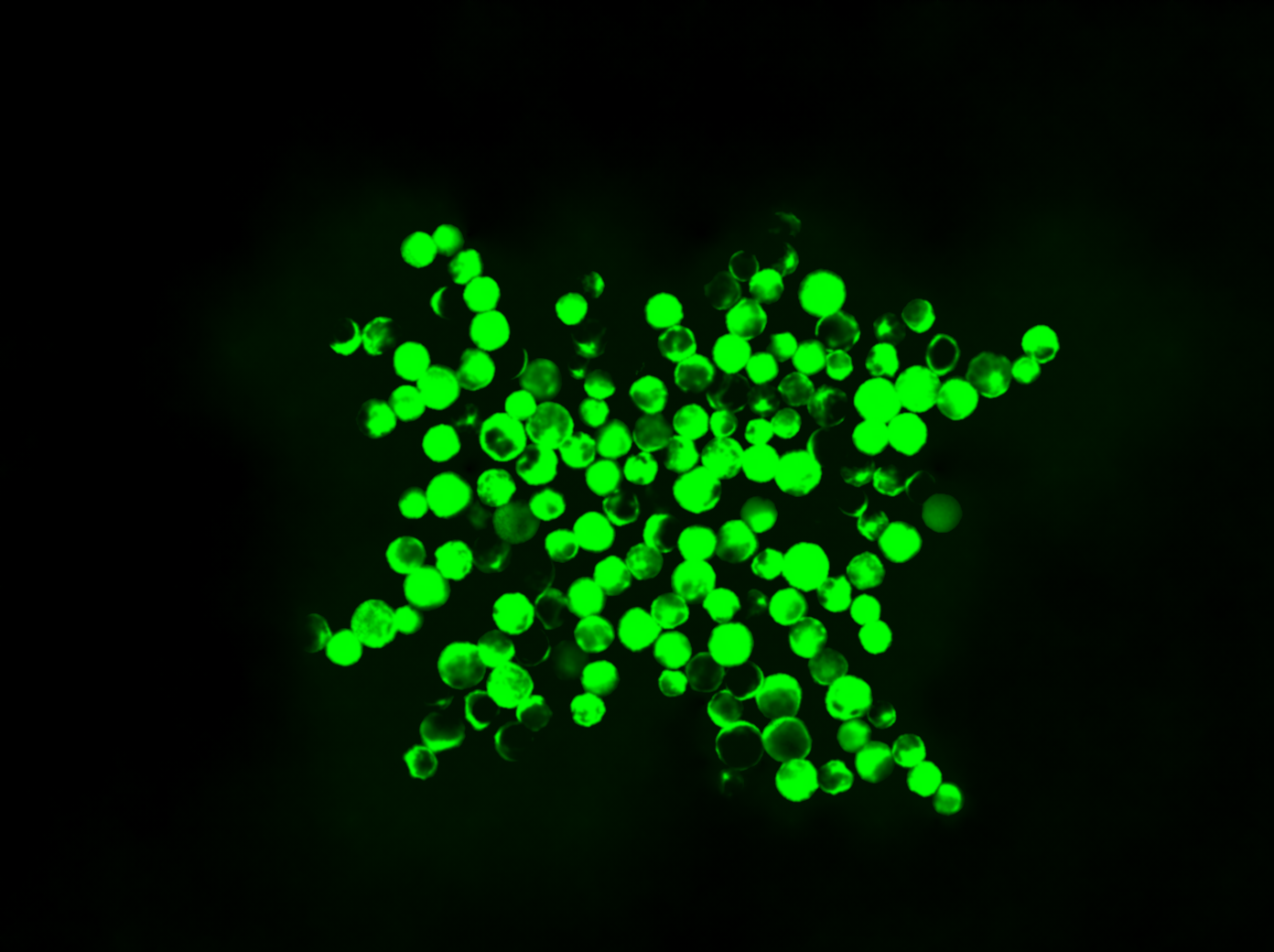

Supplement: Additional file 5 — The zip archive contains simulated images showing protoplasts with corresponding ground truth. (ZIP 72704 kb) [file 12859_2017_1591_MOESM5_ESM.zip › simulated protoplasts/overlapping/overlapping028.png]

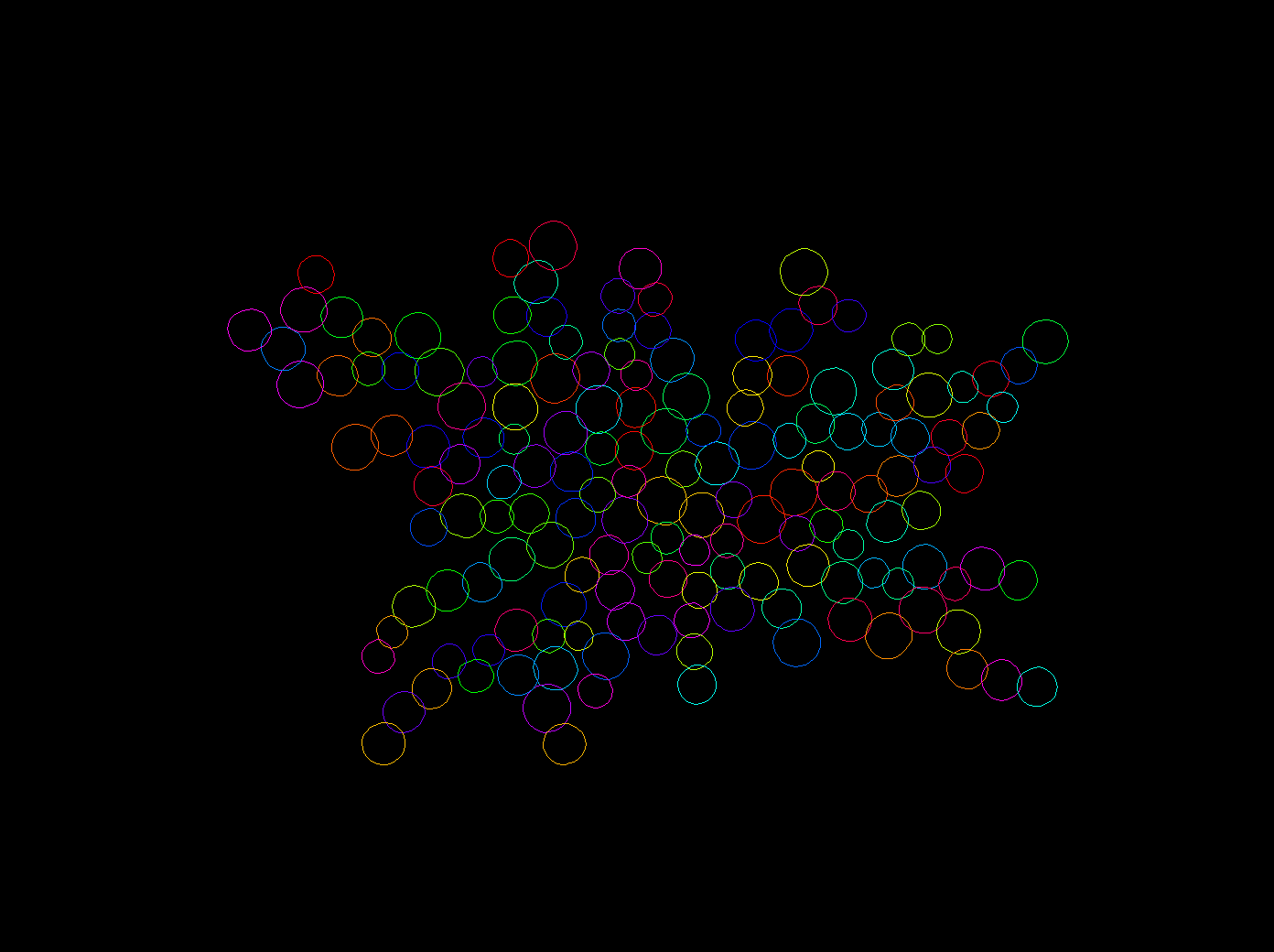

Supplement: Additional file 5 — The zip archive contains simulated images showing protoplasts with corresponding ground truth. (ZIP 72704 kb) [file 12859_2017_1591_MOESM5_ESM.zip › simulated protoplasts/overlapping/overlapping029 gt.png]

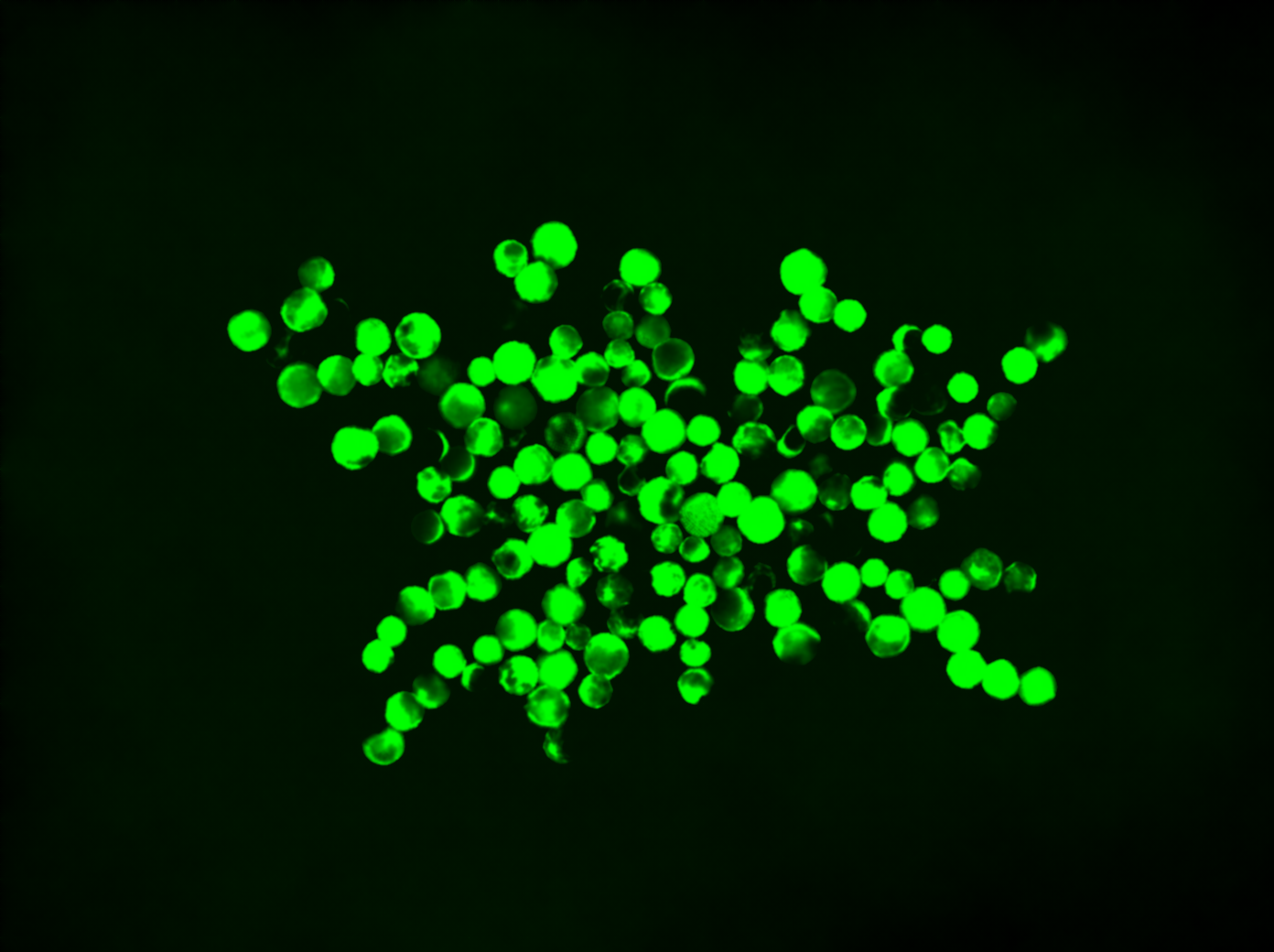

Supplement: Additional file 5 — The zip archive contains simulated images showing protoplasts with corresponding ground truth. (ZIP 72704 kb) [file 12859_2017_1591_MOESM5_ESM.zip › simulated protoplasts/overlapping/overlapping029.png]

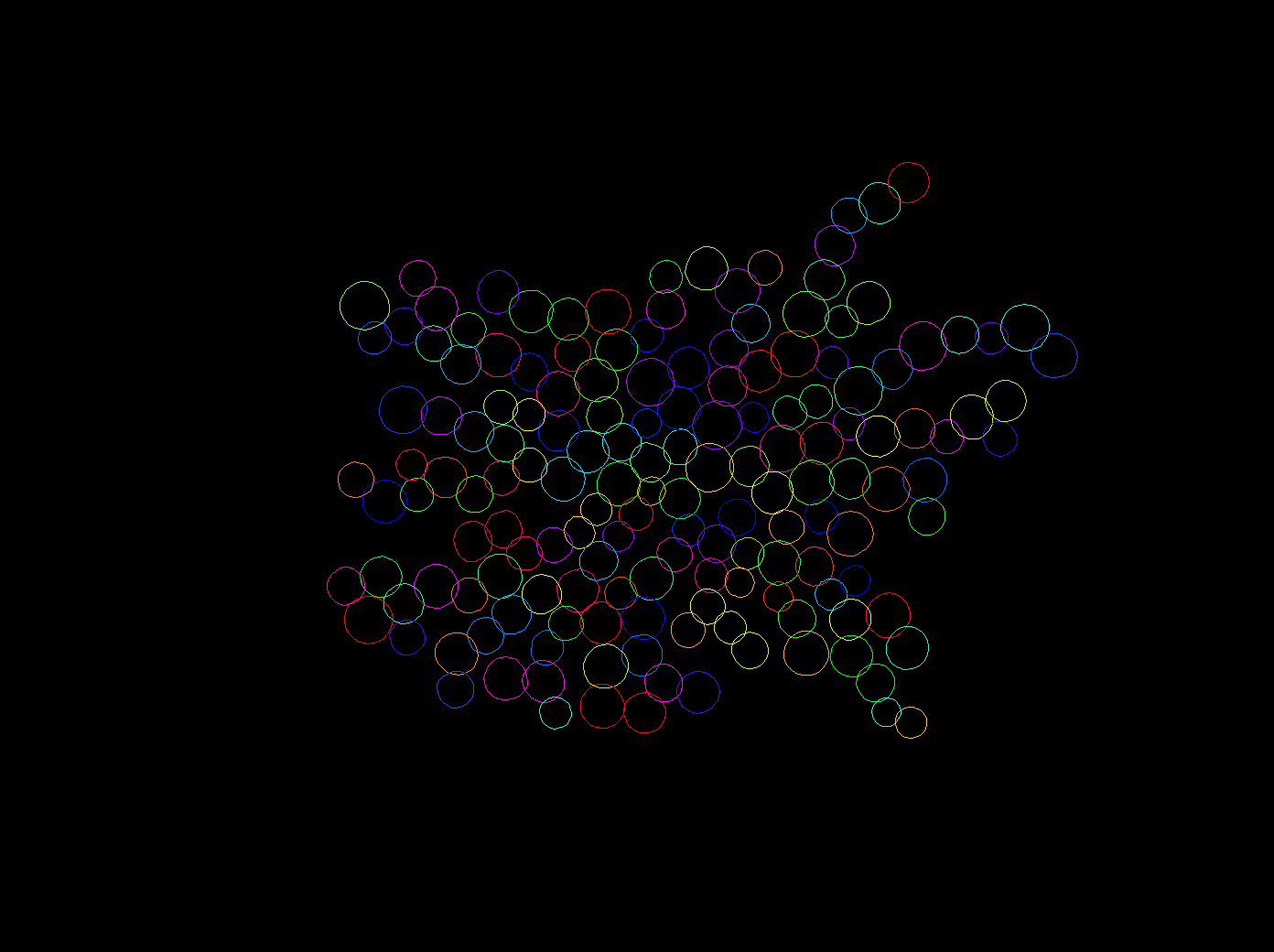

Supplement: Additional file 5 — The zip archive contains simulated images showing protoplasts with corresponding ground truth. (ZIP 72704 kb) [file 12859_2017_1591_MOESM5_ESM.zip › simulated protoplasts/overlapping/overlapping030 gt.png]

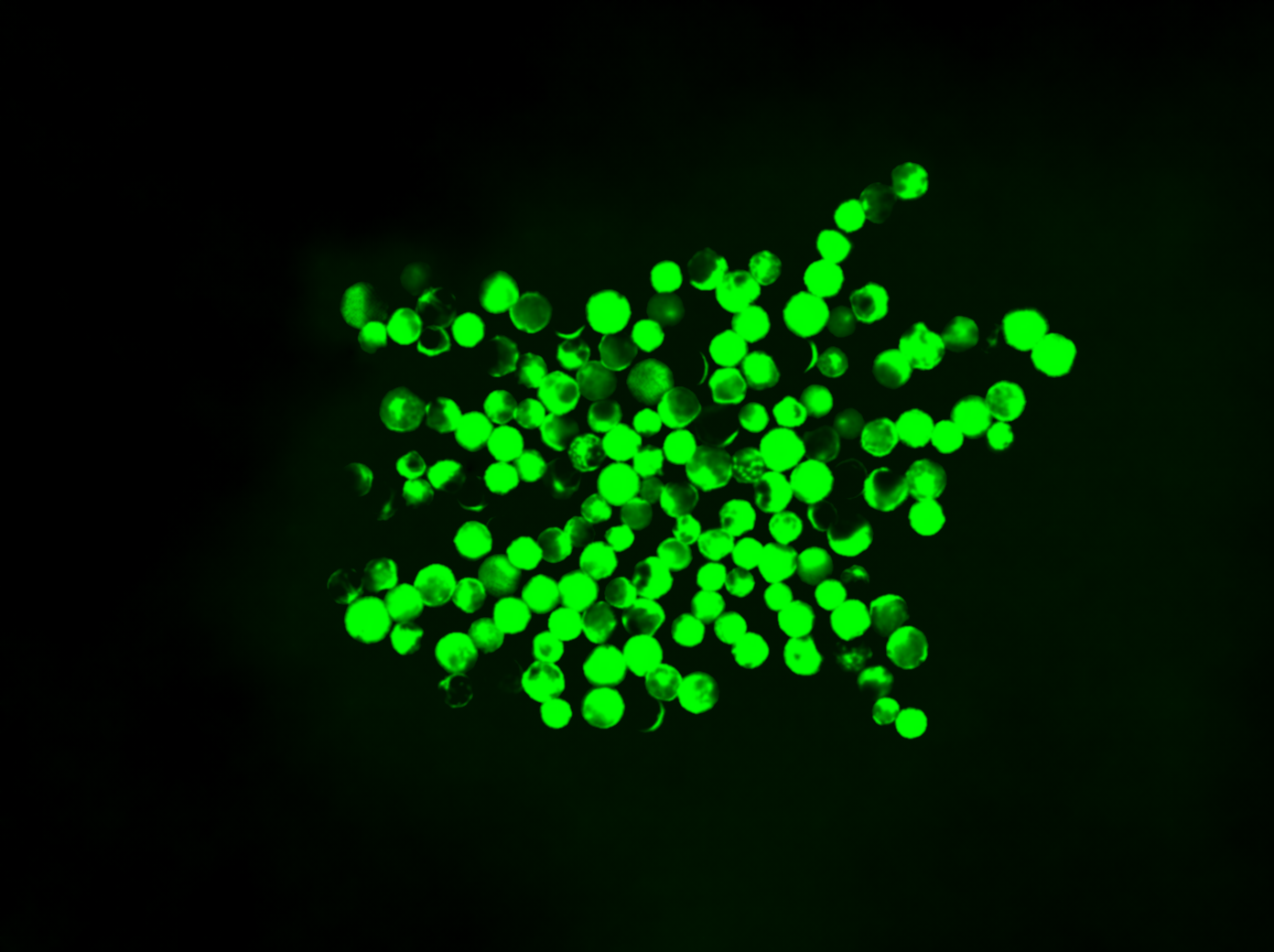

Supplement: Additional file 5 — The zip archive contains simulated images showing protoplasts with corresponding ground truth. (ZIP 72704 kb) [file 12859_2017_1591_MOESM5_ESM.zip › simulated protoplasts/overlapping/overlapping030.png]

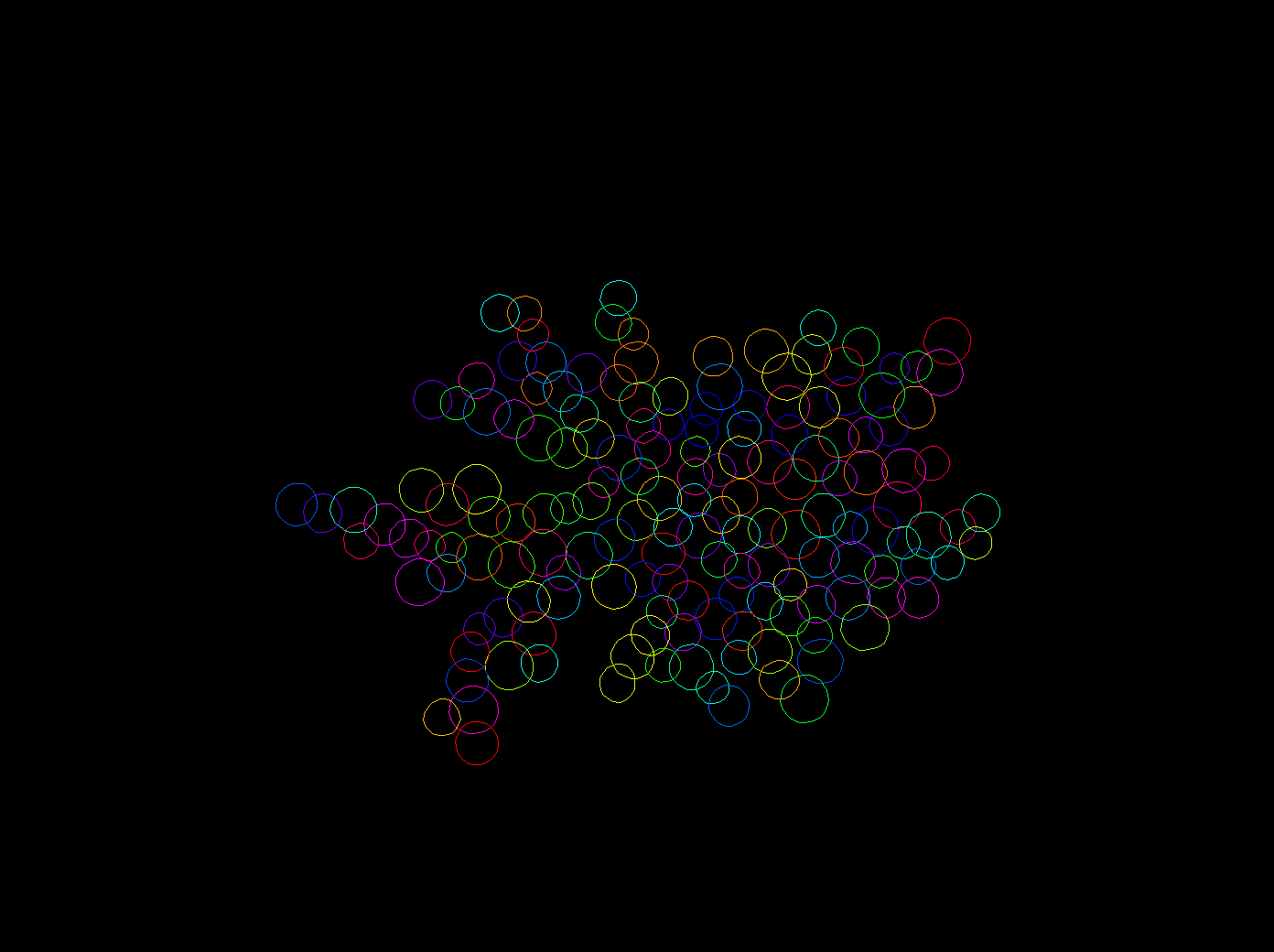

Supplement: Additional file 5 — The zip archive contains simulated images showing protoplasts with corresponding ground truth. (ZIP 72704 kb) [file 12859_2017_1591_MOESM5_ESM.zip › simulated protoplasts/overlaying/overlaying001 gt.png]

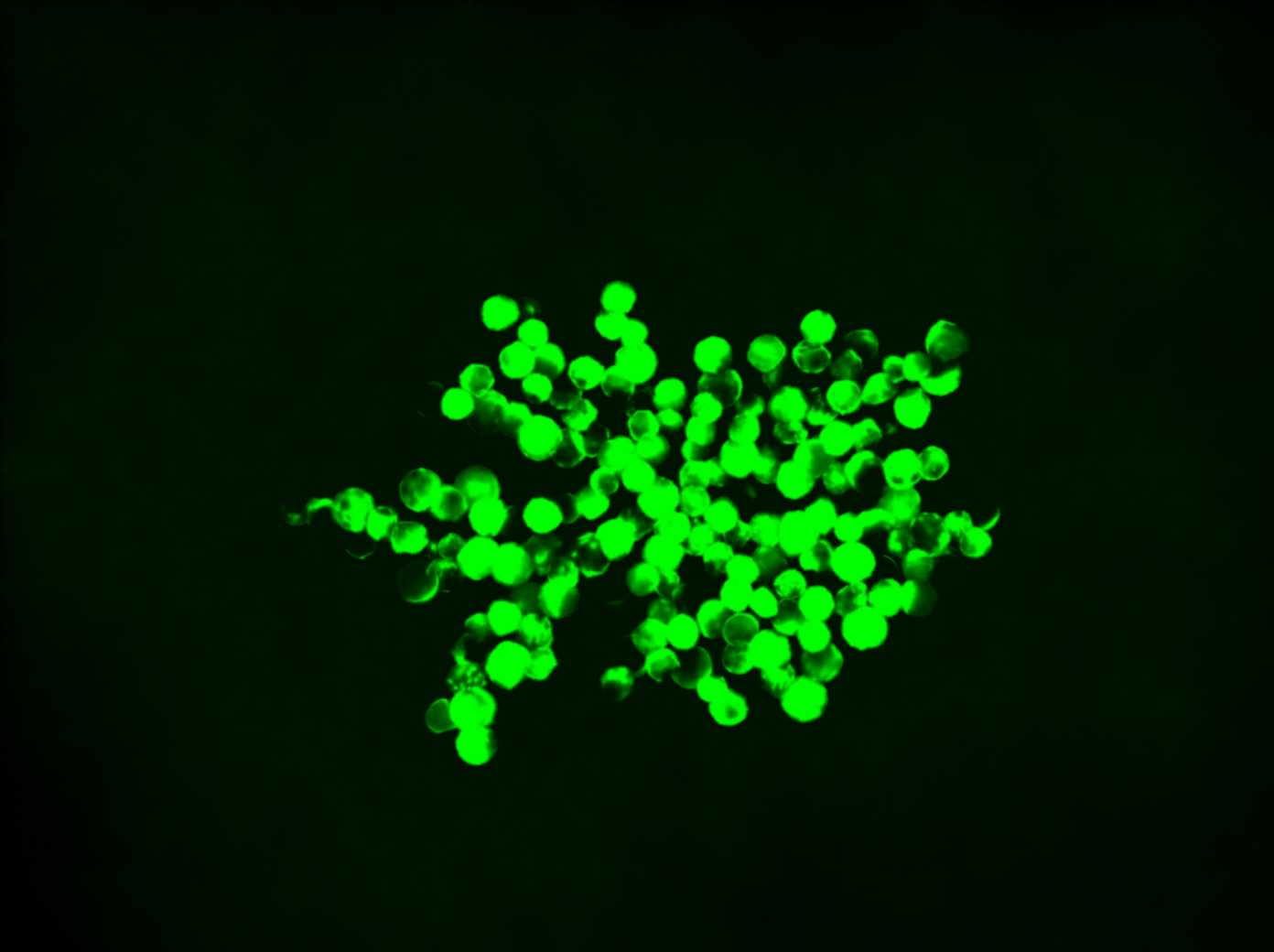

Supplement: Additional file 5 — The zip archive contains simulated images showing protoplasts with corresponding ground truth. (ZIP 72704 kb) [file 12859_2017_1591_MOESM5_ESM.zip › simulated protoplasts/overlaying/overlaying001.png]

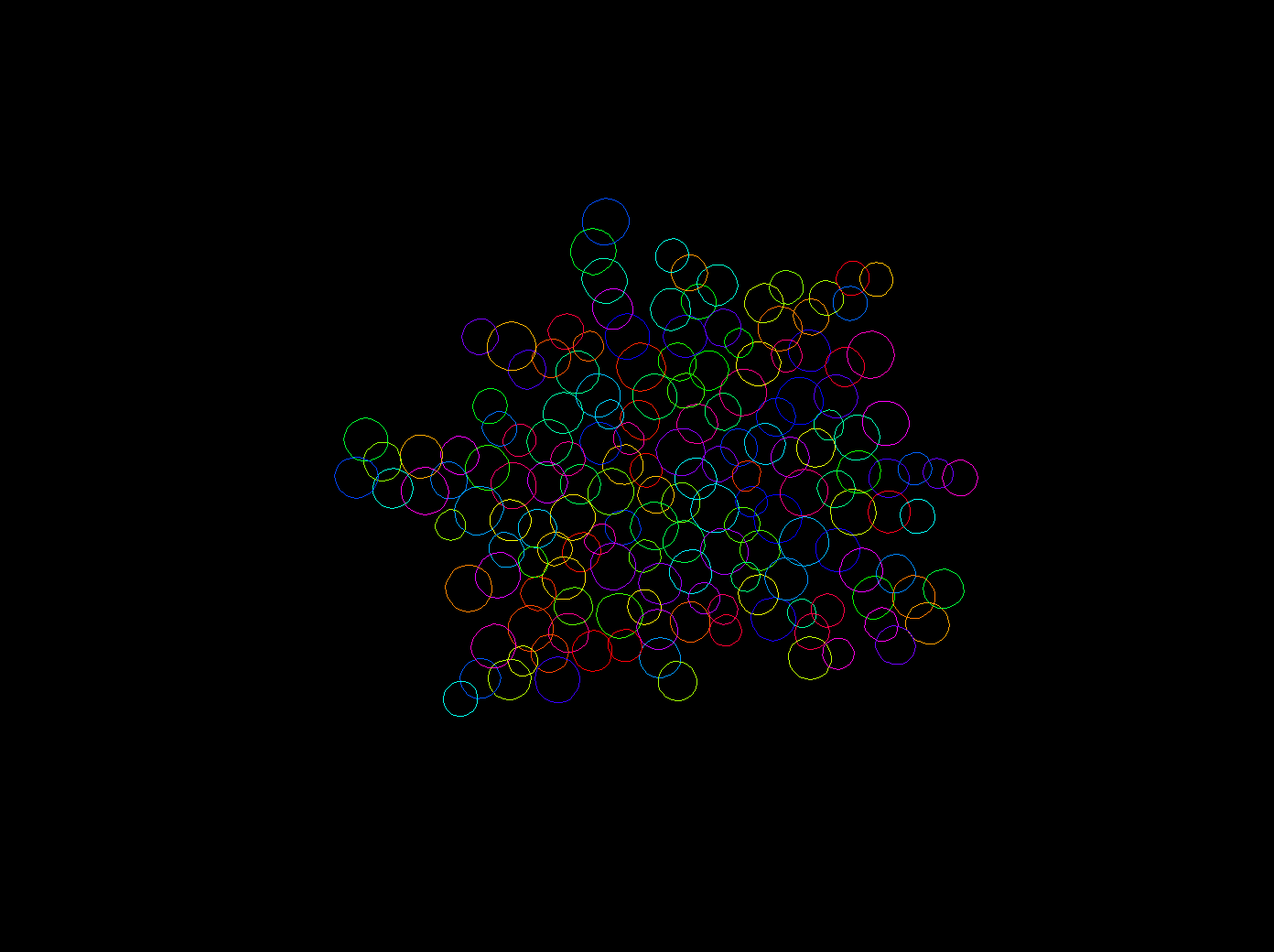

Supplement: Additional file 5 — The zip archive contains simulated images showing protoplasts with corresponding ground truth. (ZIP 72704 kb) [file 12859_2017_1591_MOESM5_ESM.zip › simulated protoplasts/overlaying/overlaying002 gt.png]

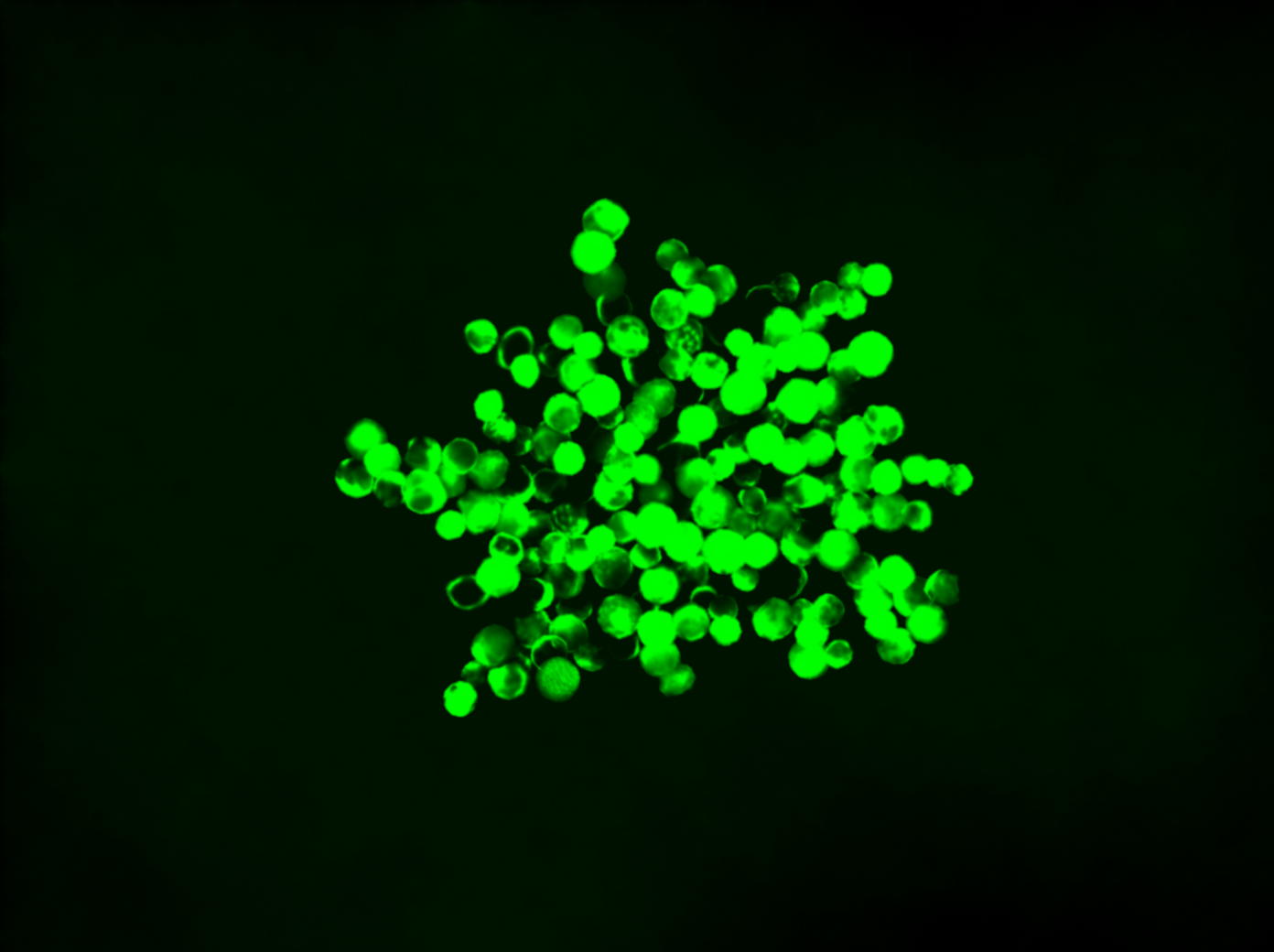

Supplement: Additional file 5 — The zip archive contains simulated images showing protoplasts with corresponding ground truth. (ZIP 72704 kb) [file 12859_2017_1591_MOESM5_ESM.zip › simulated protoplasts/overlaying/overlaying002.png]

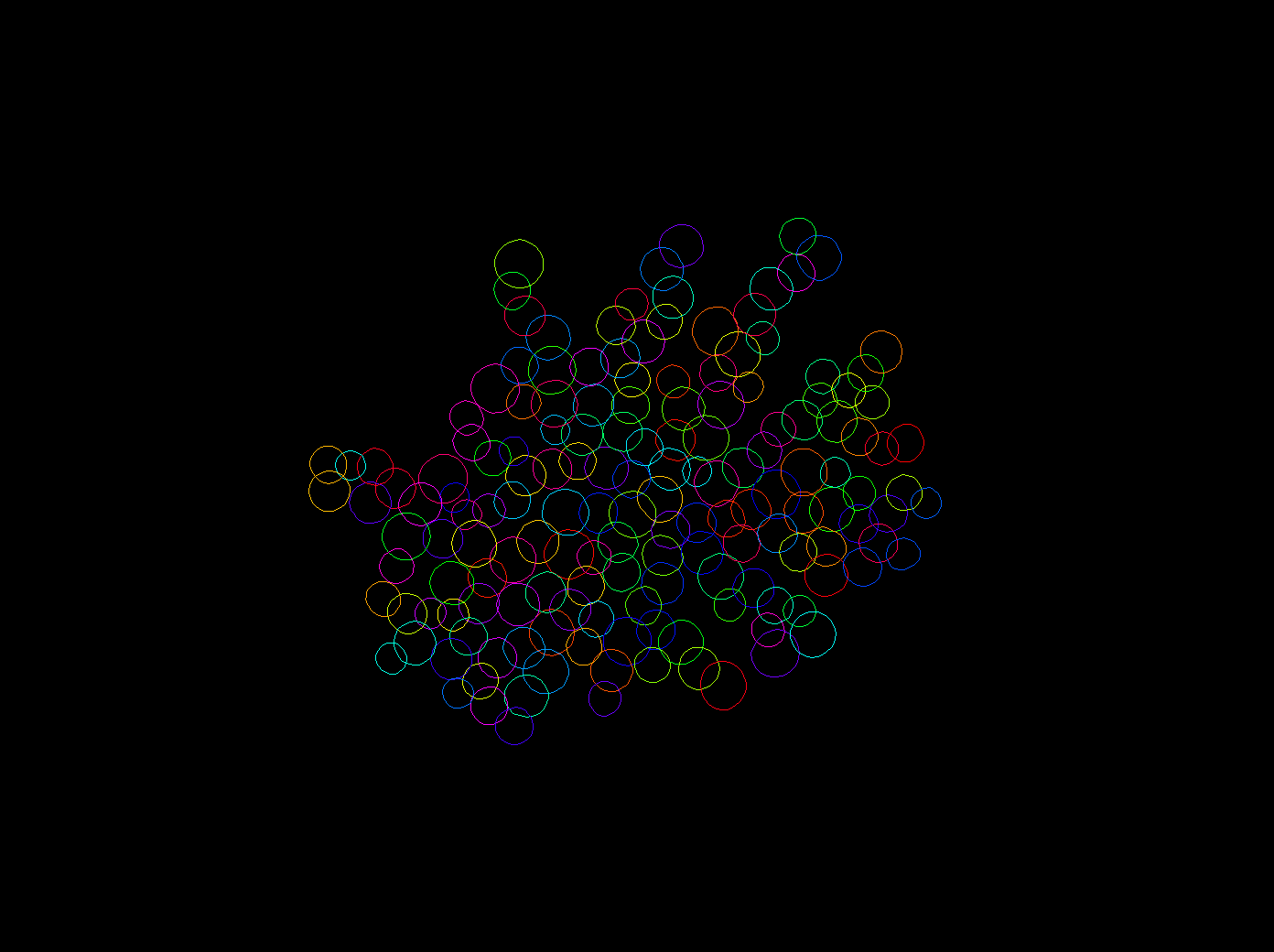

Supplement: Additional file 5 — The zip archive contains simulated images showing protoplasts with corresponding ground truth. (ZIP 72704 kb) [file 12859_2017_1591_MOESM5_ESM.zip › simulated protoplasts/overlaying/overlaying003 gt.png]

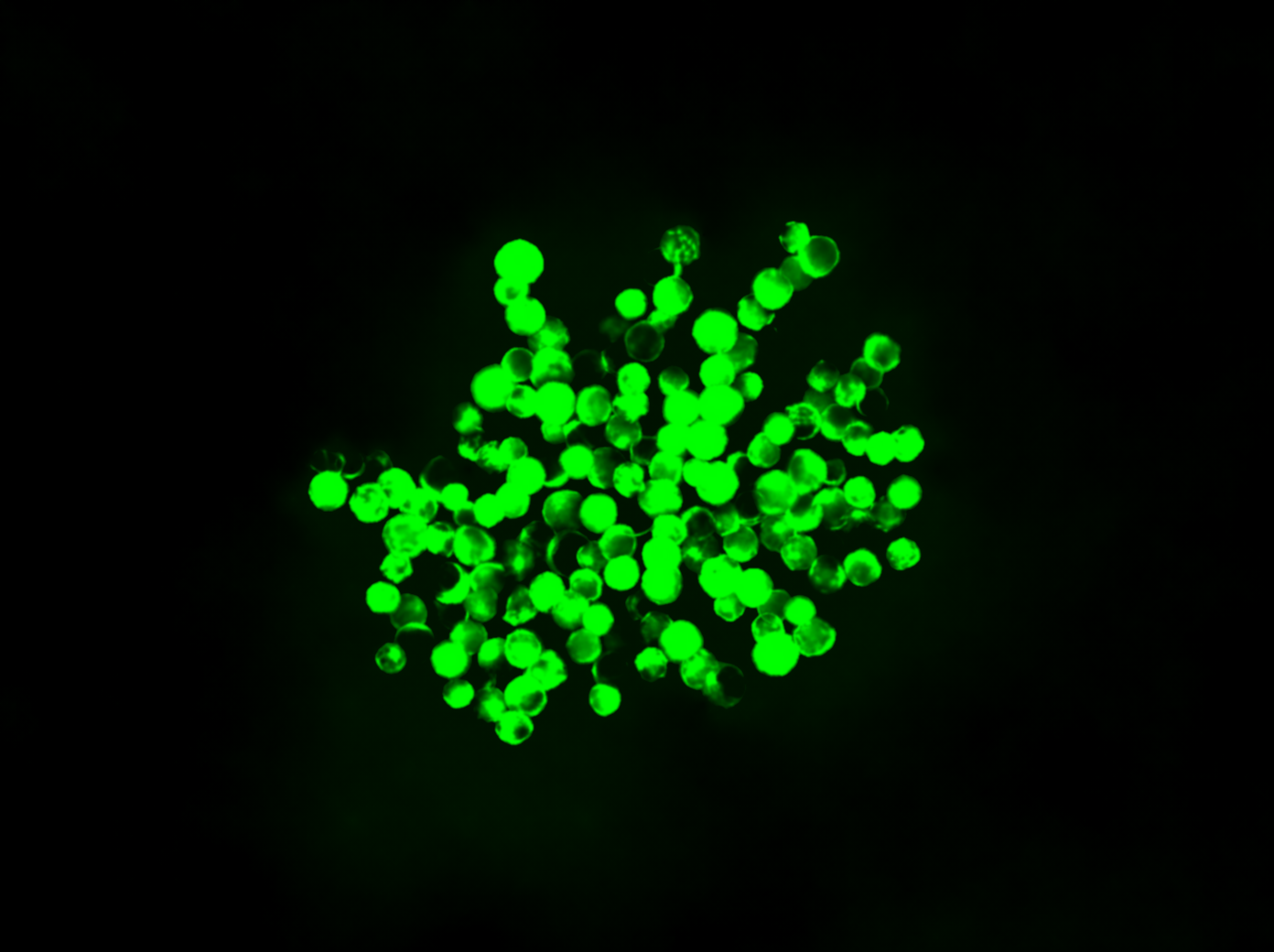

Supplement: Additional file 5 — The zip archive contains simulated images showing protoplasts with corresponding ground truth. (ZIP 72704 kb) [file 12859_2017_1591_MOESM5_ESM.zip › simulated protoplasts/overlaying/overlaying003.png]

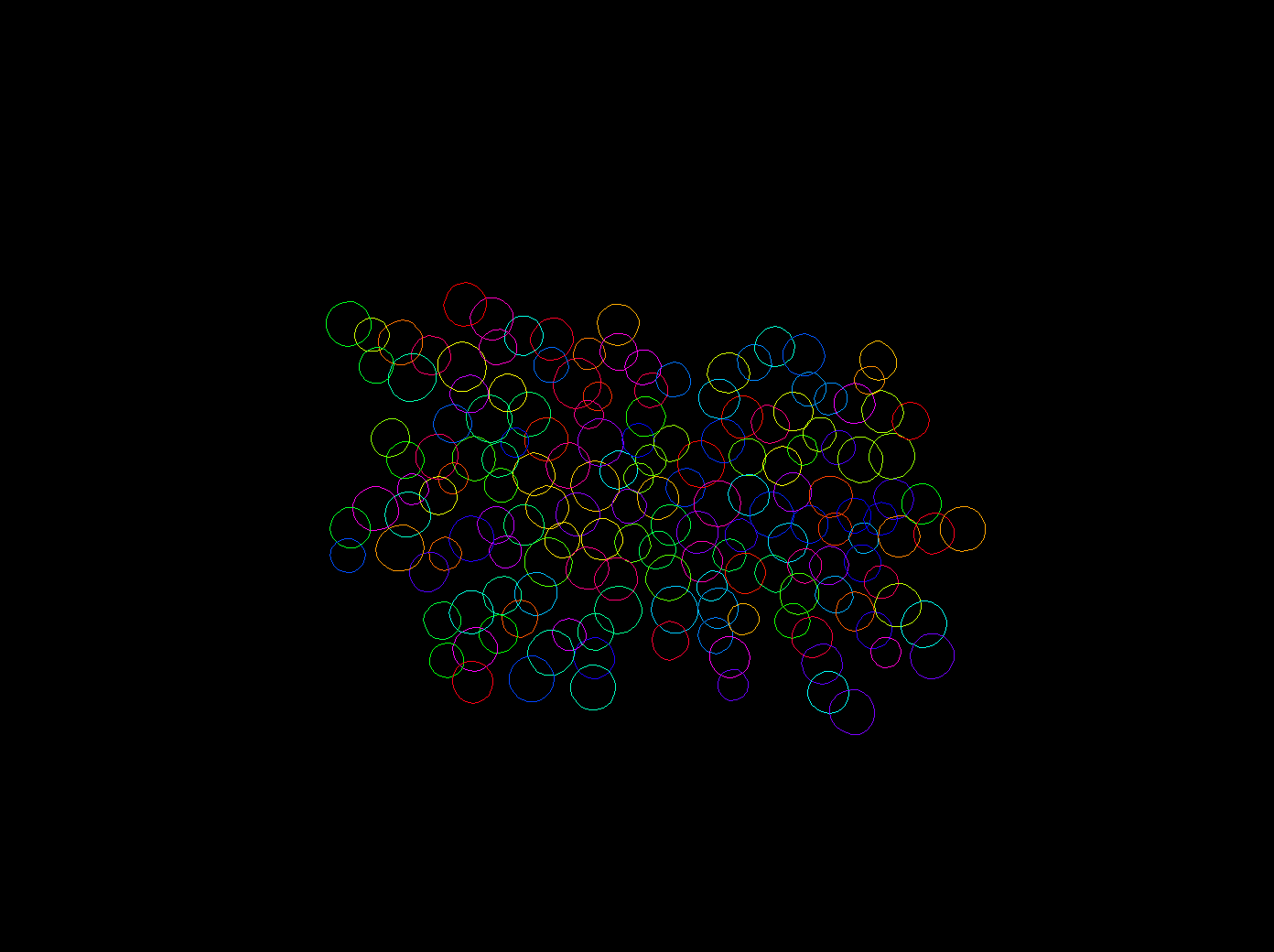

Supplement: Additional file 5 — The zip archive contains simulated images showing protoplasts with corresponding ground truth. (ZIP 72704 kb) [file 12859_2017_1591_MOESM5_ESM.zip › simulated protoplasts/overlaying/overlaying004 gt.png]

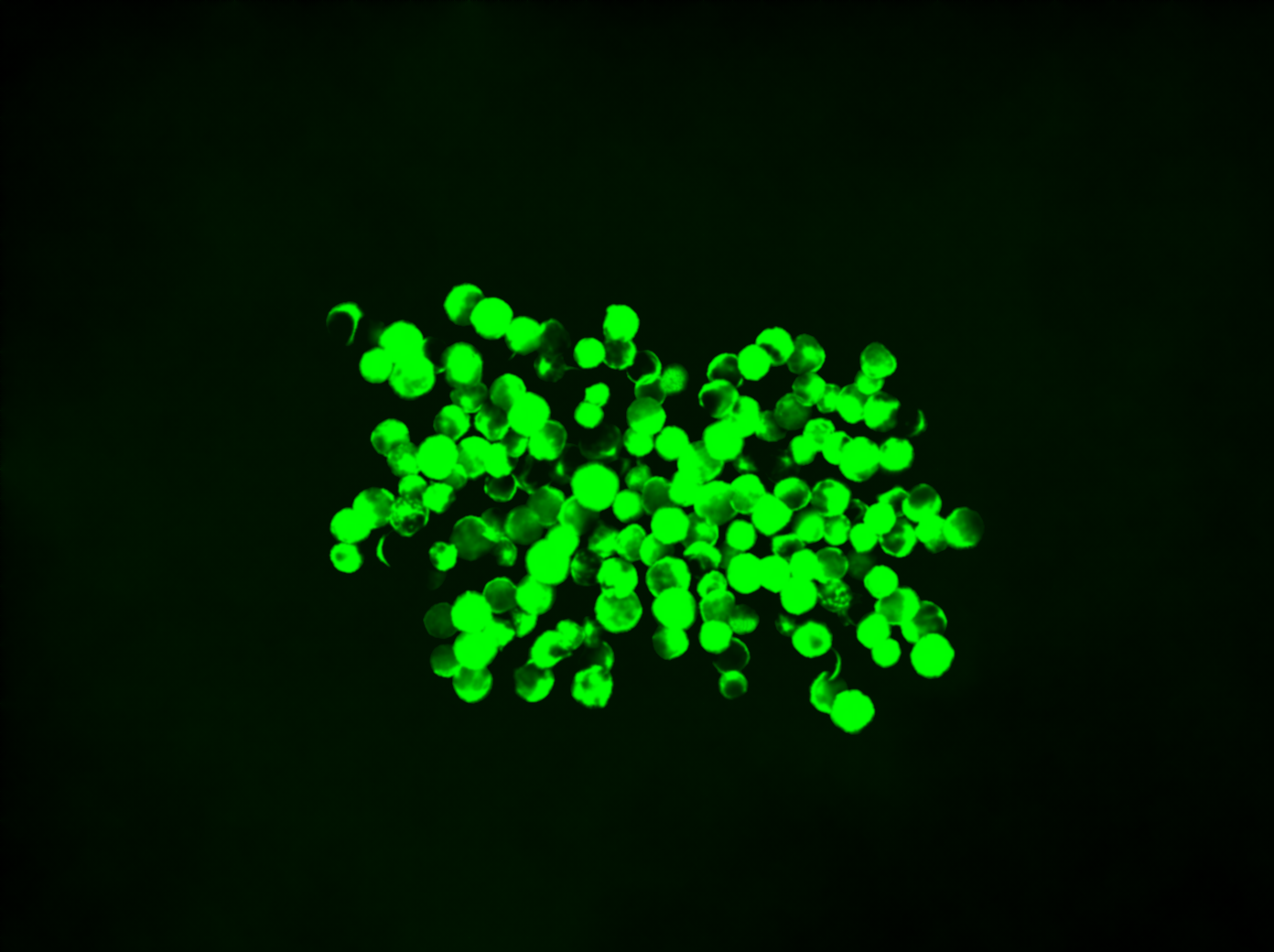

Supplement: Additional file 5 — The zip archive contains simulated images showing protoplasts with corresponding ground truth. (ZIP 72704 kb) [file 12859_2017_1591_MOESM5_ESM.zip › simulated protoplasts/overlaying/overlaying004.png]

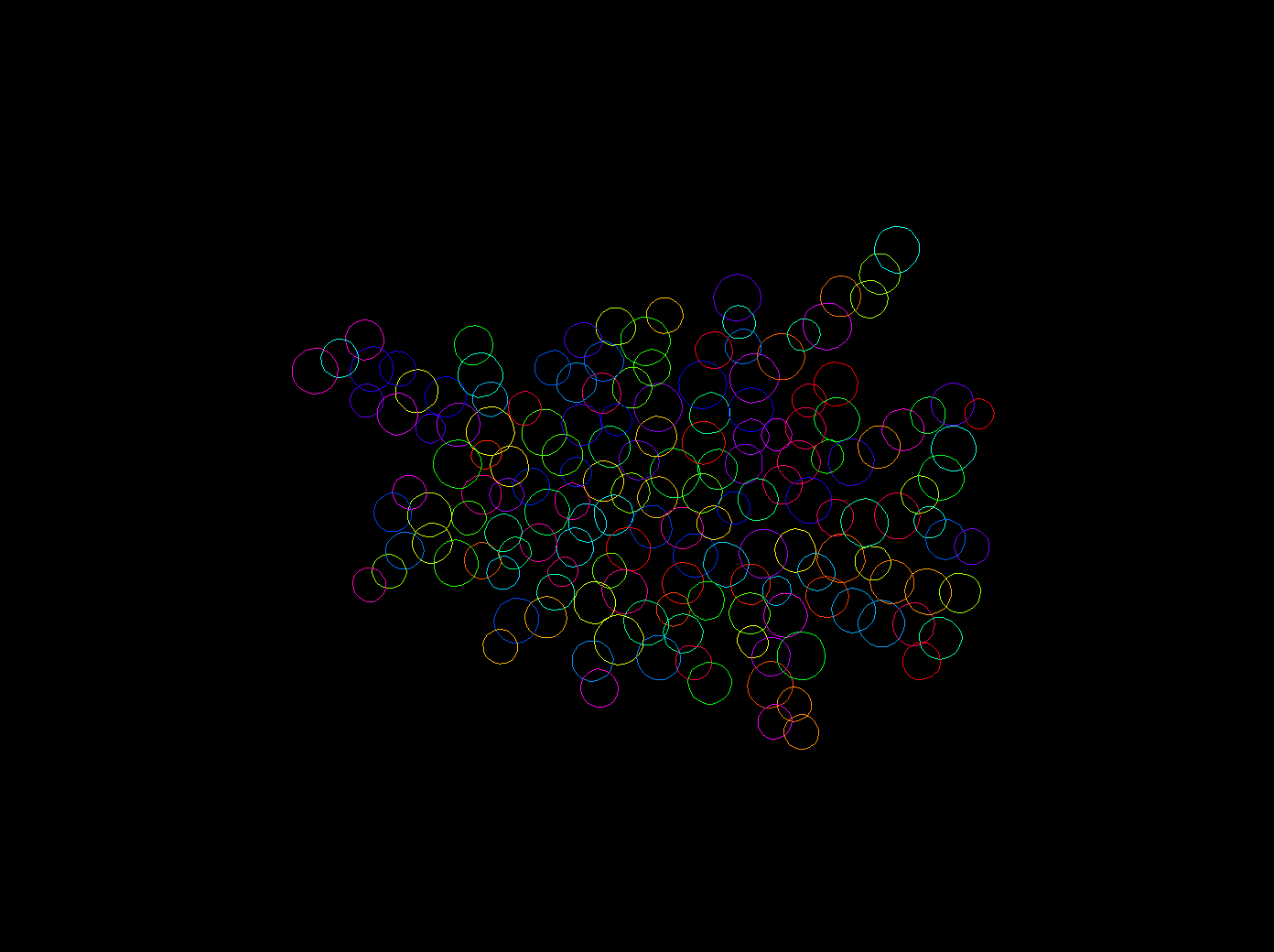

Supplement: Additional file 5 — The zip archive contains simulated images showing protoplasts with corresponding ground truth. (ZIP 72704 kb) [file 12859_2017_1591_MOESM5_ESM.zip › simulated protoplasts/overlaying/overlaying005 gt.png]

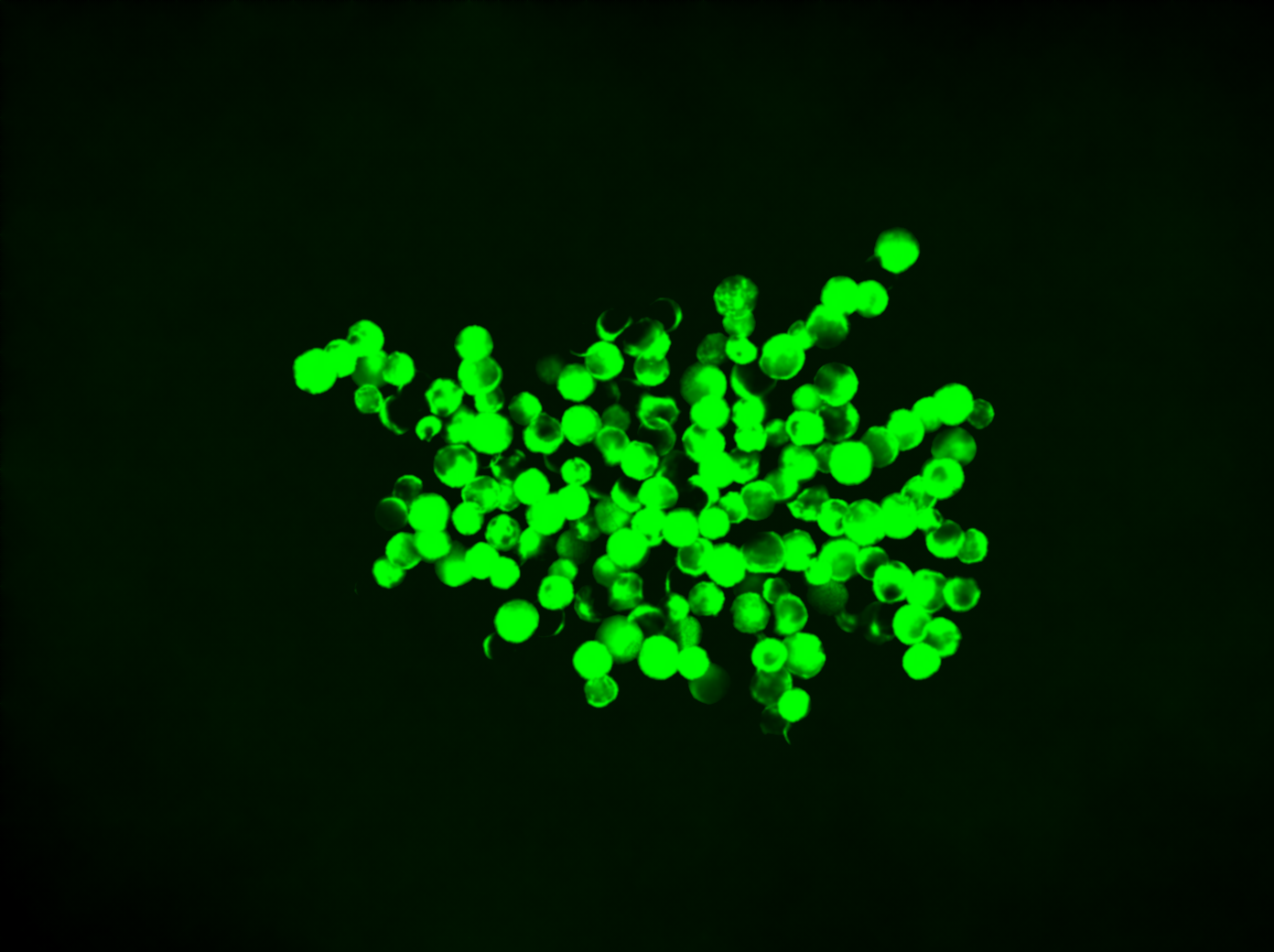

Supplement: Additional file 5 — The zip archive contains simulated images showing protoplasts with corresponding ground truth. (ZIP 72704 kb) [file 12859_2017_1591_MOESM5_ESM.zip › simulated protoplasts/overlaying/overlaying005.png]

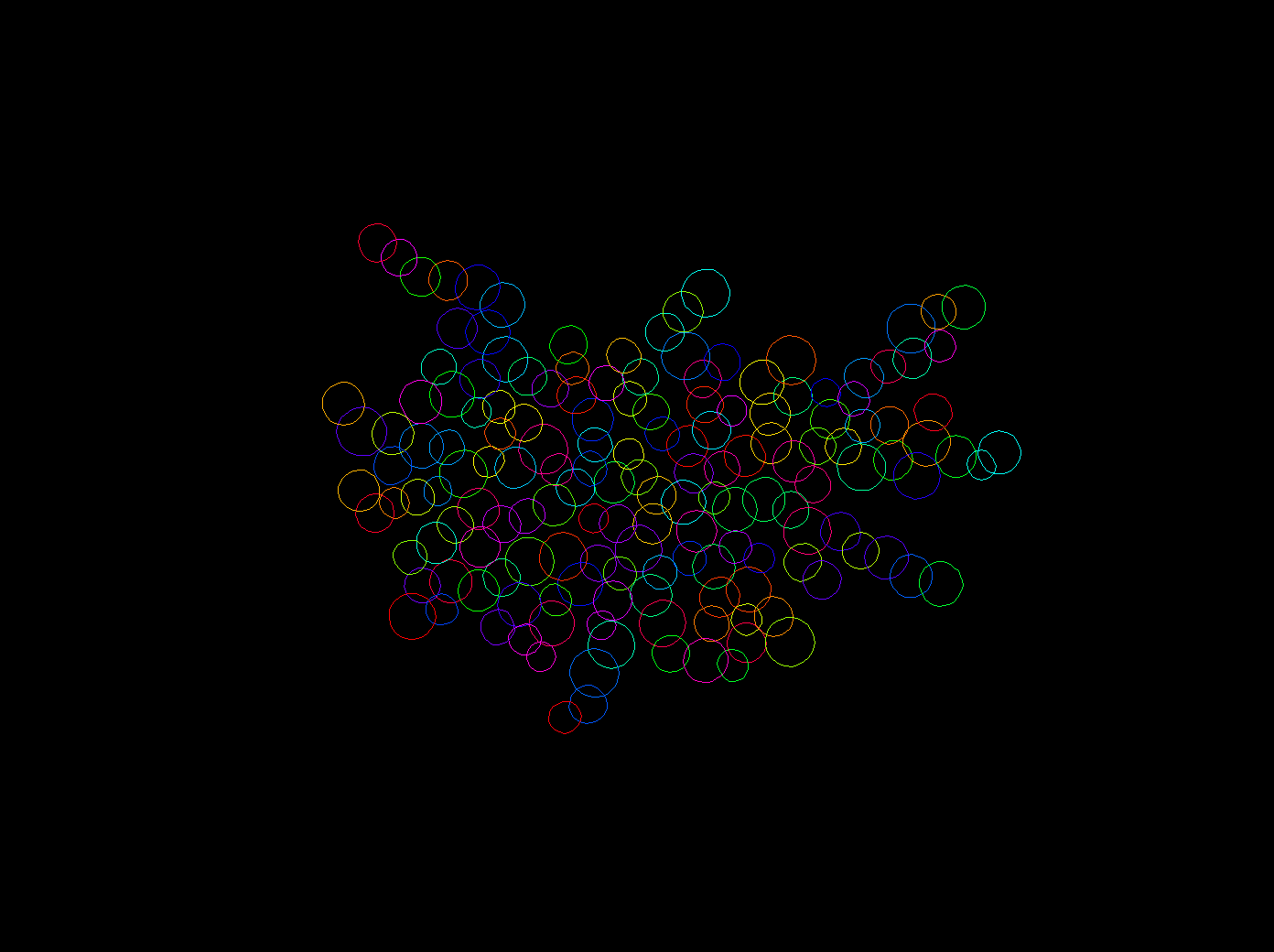

Supplement: Additional file 5 — The zip archive contains simulated images showing protoplasts with corresponding ground truth. (ZIP 72704 kb) [file 12859_2017_1591_MOESM5_ESM.zip › simulated protoplasts/overlaying/overlaying006 gt.png]

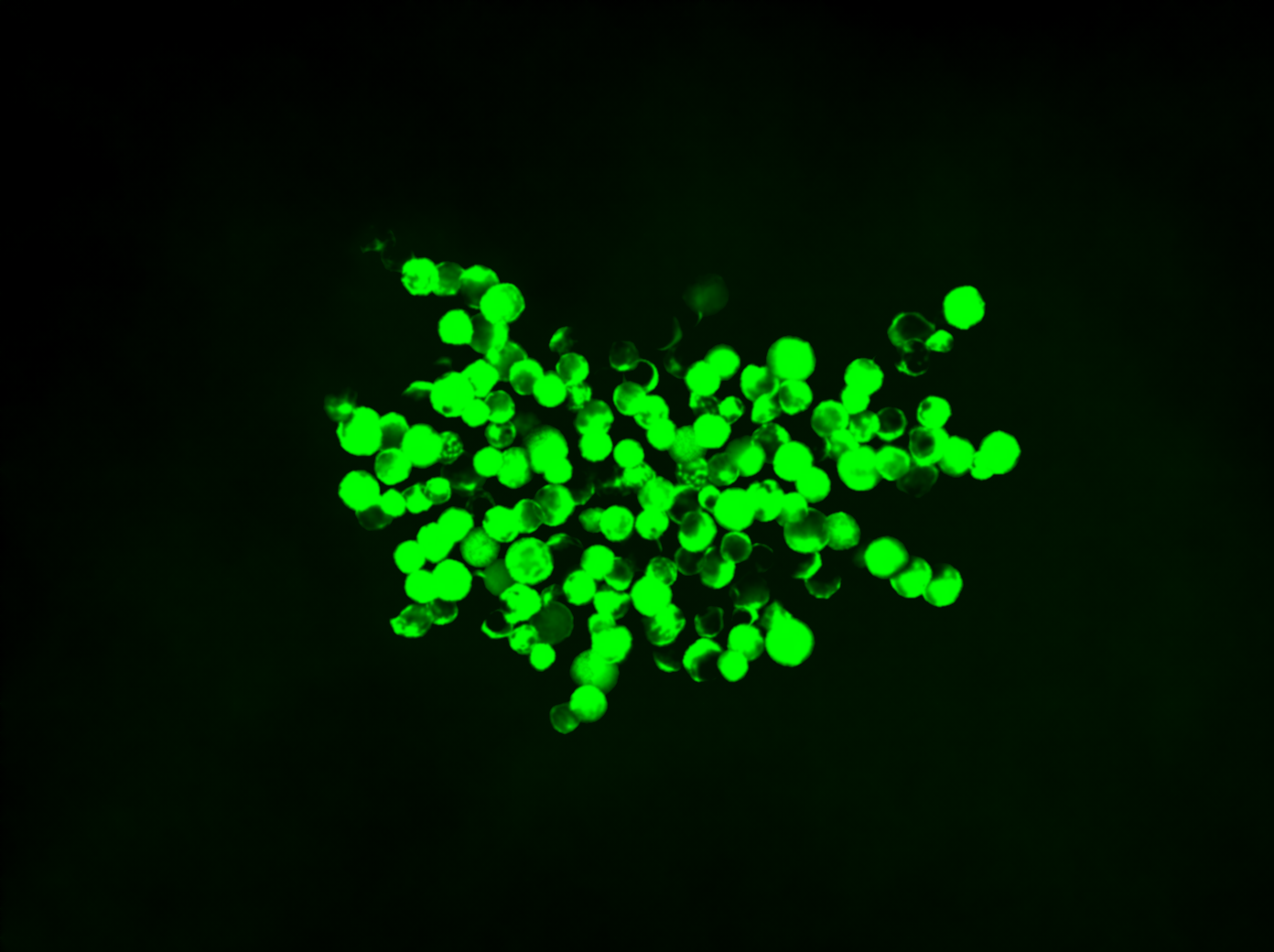

Supplement: Additional file 5 — The zip archive contains simulated images showing protoplasts with corresponding ground truth. (ZIP 72704 kb) [file 12859_2017_1591_MOESM5_ESM.zip › simulated protoplasts/overlaying/overlaying006.png]

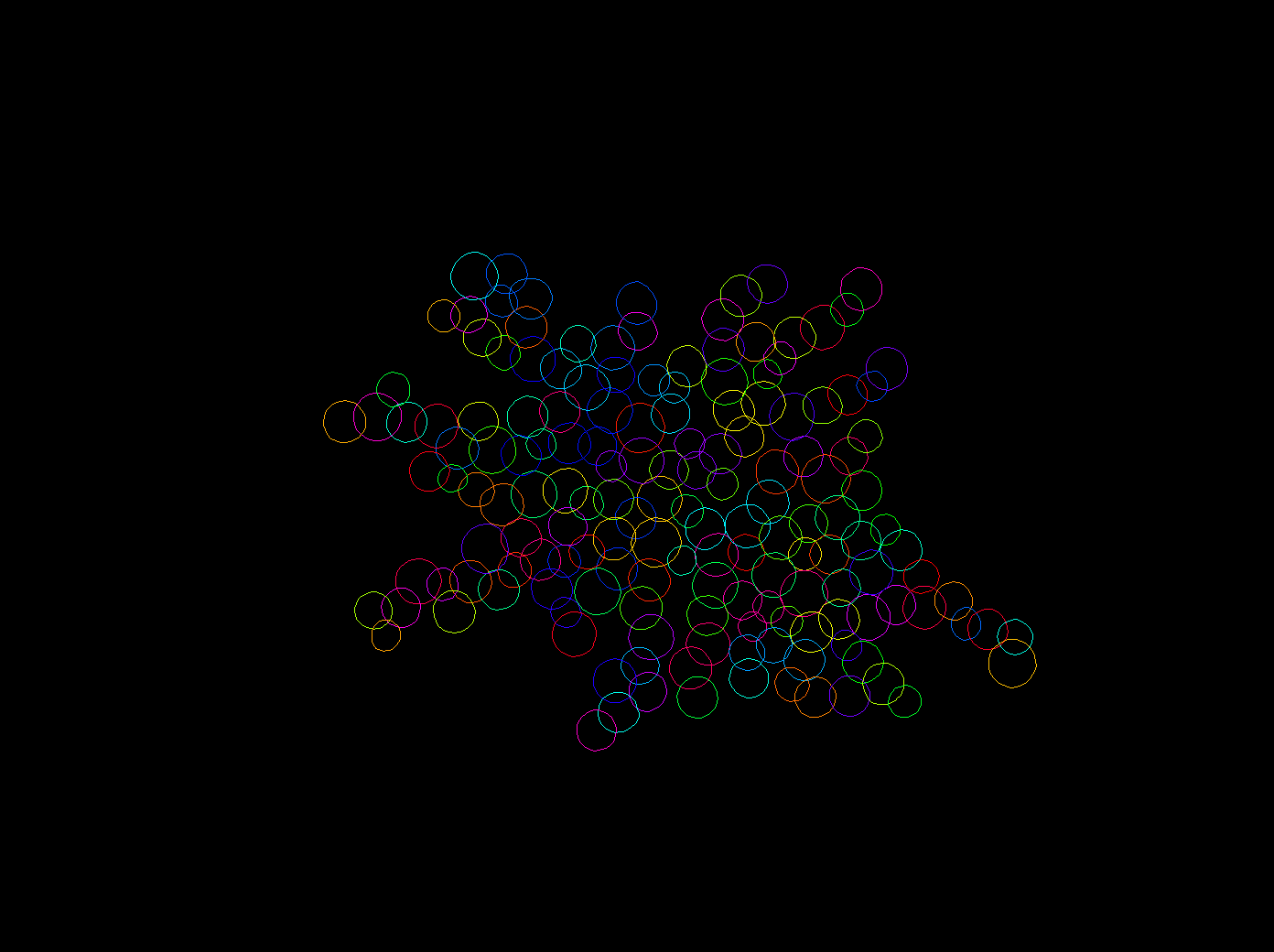

Supplement: Additional file 5 — The zip archive contains simulated images showing protoplasts with corresponding ground truth. (ZIP 72704 kb) [file 12859_2017_1591_MOESM5_ESM.zip › simulated protoplasts/overlaying/overlaying007 gt.png]

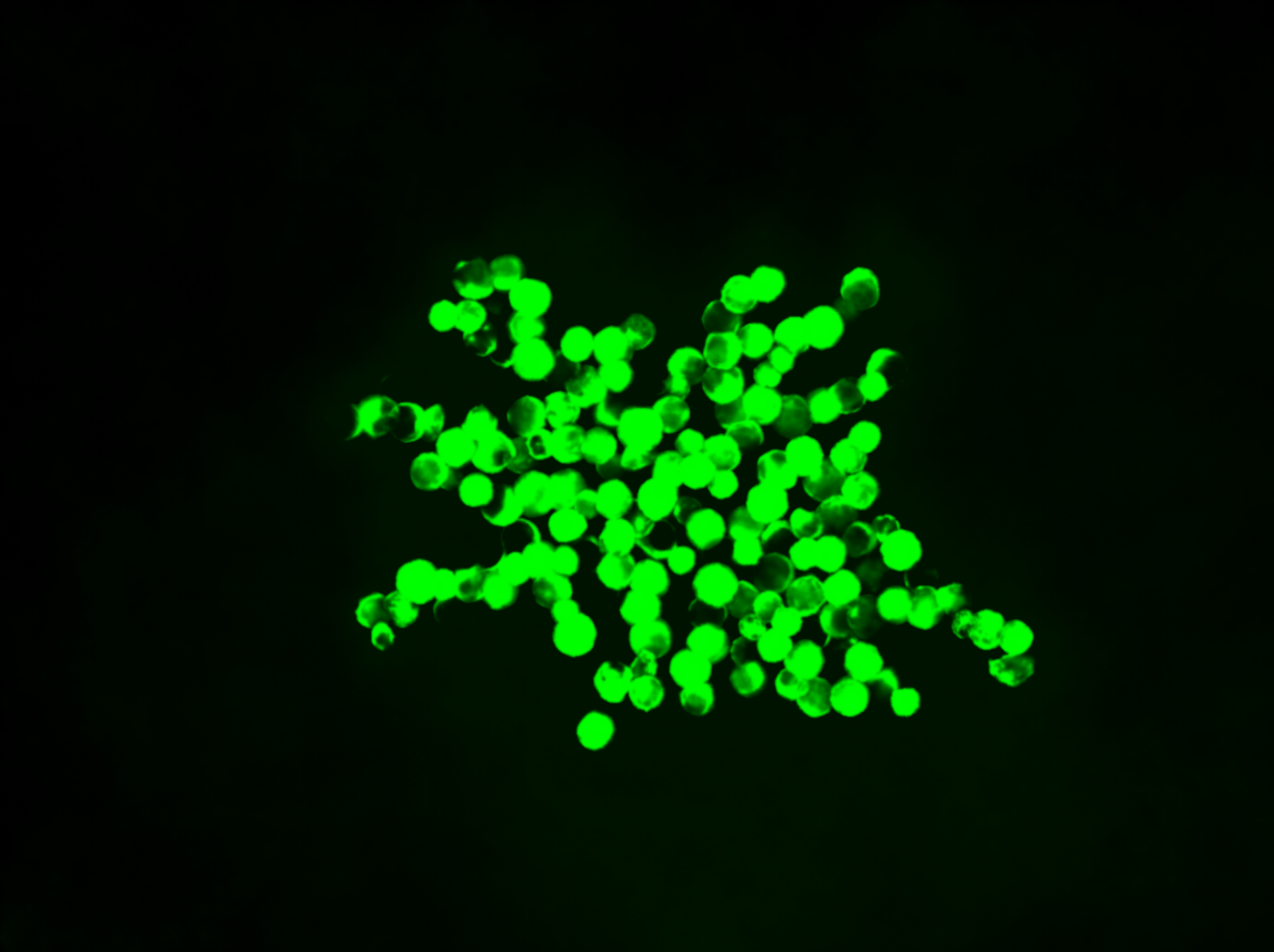

Supplement: Additional file 5 — The zip archive contains simulated images showing protoplasts with corresponding ground truth. (ZIP 72704 kb) [file 12859_2017_1591_MOESM5_ESM.zip › simulated protoplasts/overlaying/overlaying007.png]

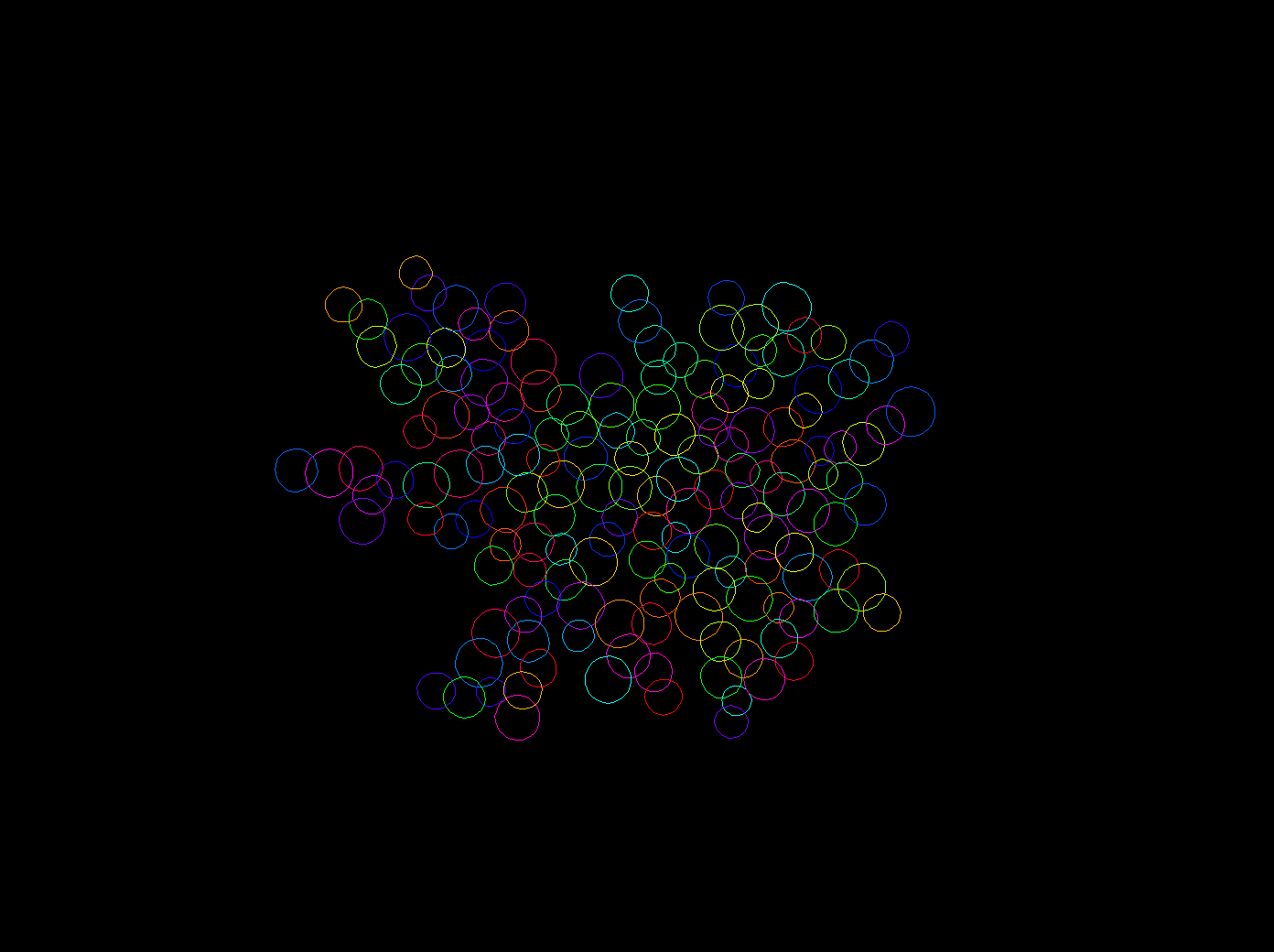

Supplement: Additional file 5 — The zip archive contains simulated images showing protoplasts with corresponding ground truth. (ZIP 72704 kb) [file 12859_2017_1591_MOESM5_ESM.zip › simulated protoplasts/overlaying/overlaying008 gt.png]

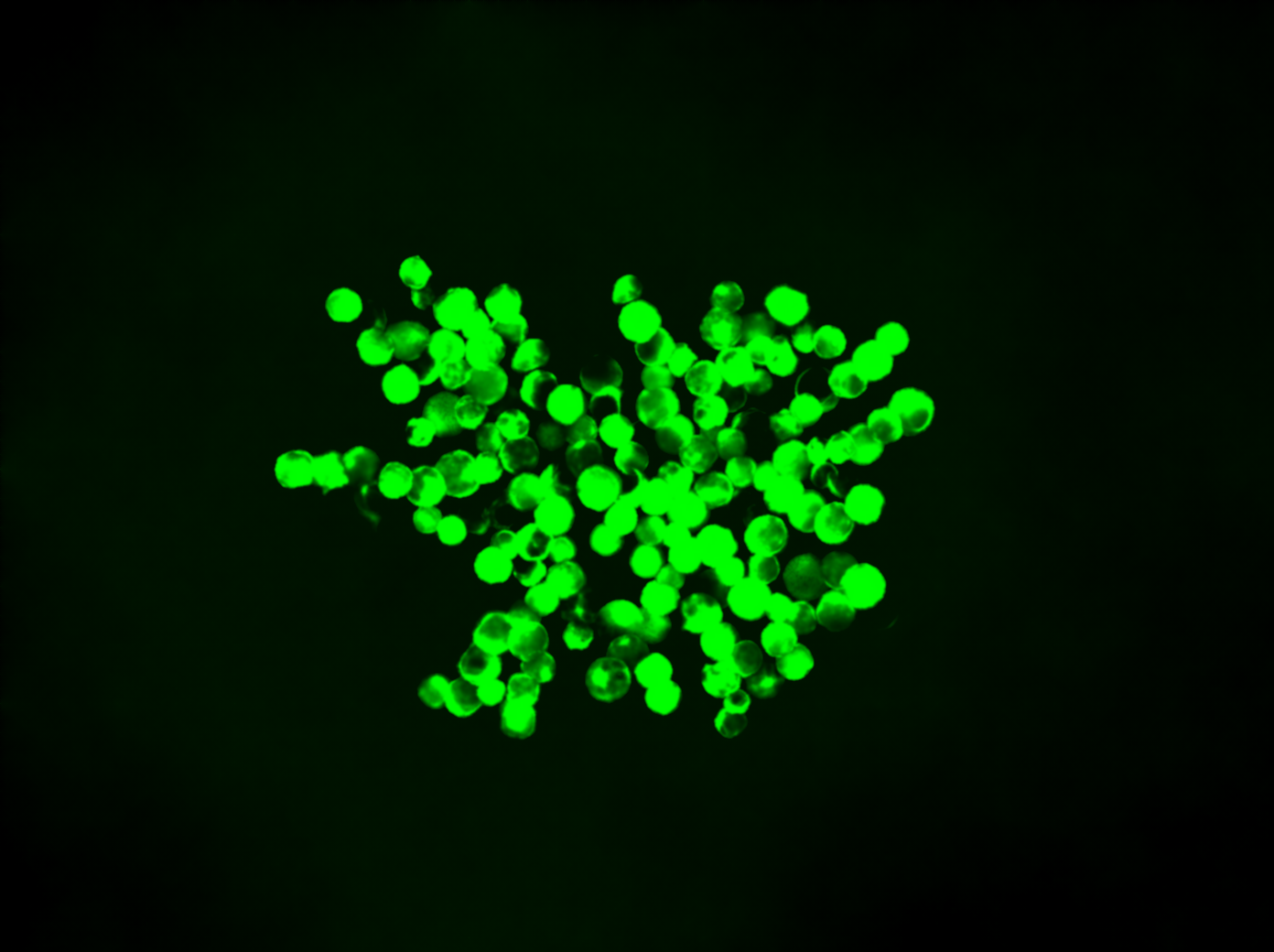

Supplement: Additional file 5 — The zip archive contains simulated images showing protoplasts with corresponding ground truth. (ZIP 72704 kb) [file 12859_2017_1591_MOESM5_ESM.zip › simulated protoplasts/overlaying/overlaying008.png]

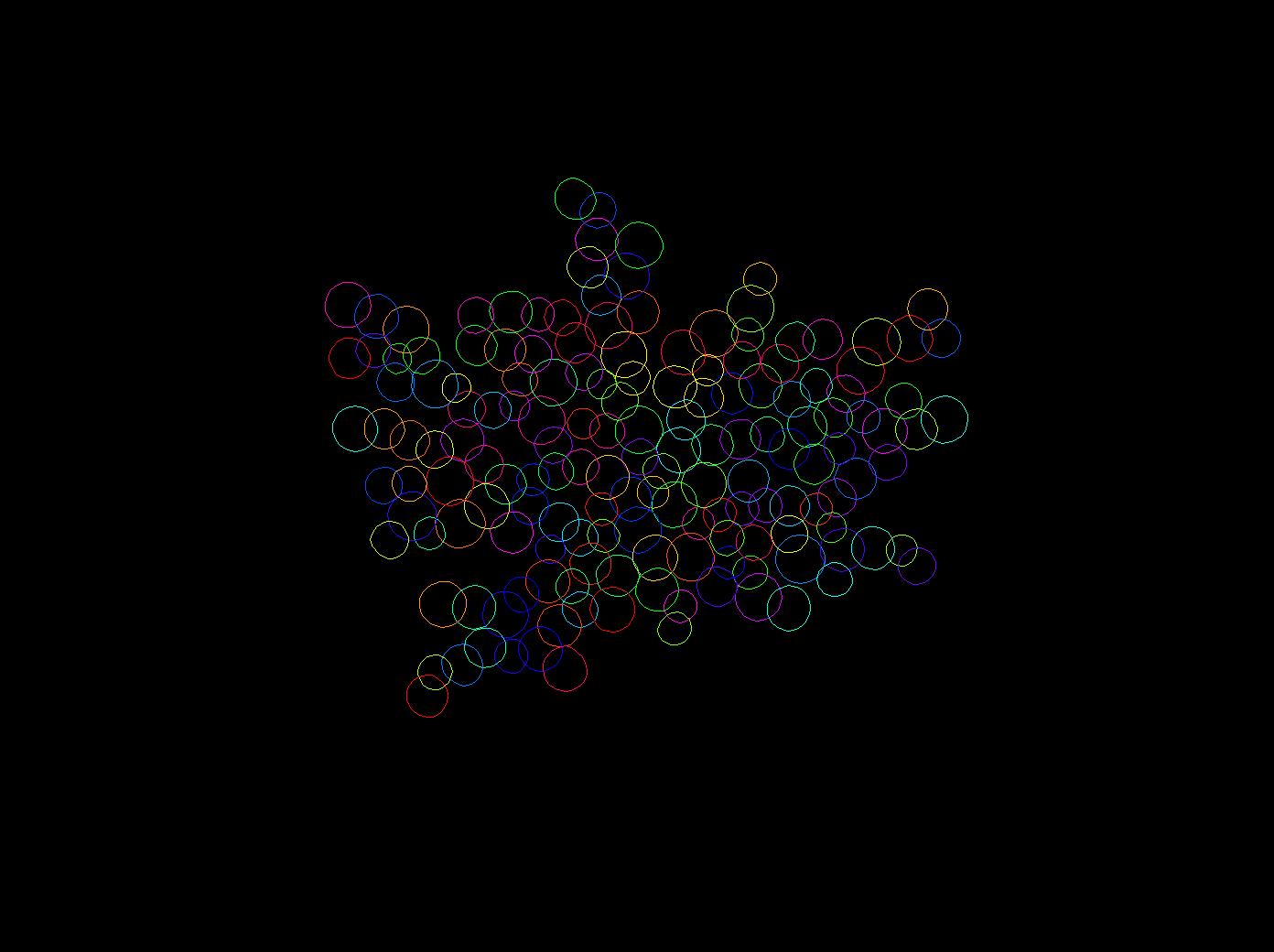

Supplement: Additional file 5 — The zip archive contains simulated images showing protoplasts with corresponding ground truth. (ZIP 72704 kb) [file 12859_2017_1591_MOESM5_ESM.zip › simulated protoplasts/overlaying/overlaying009 gt.png]

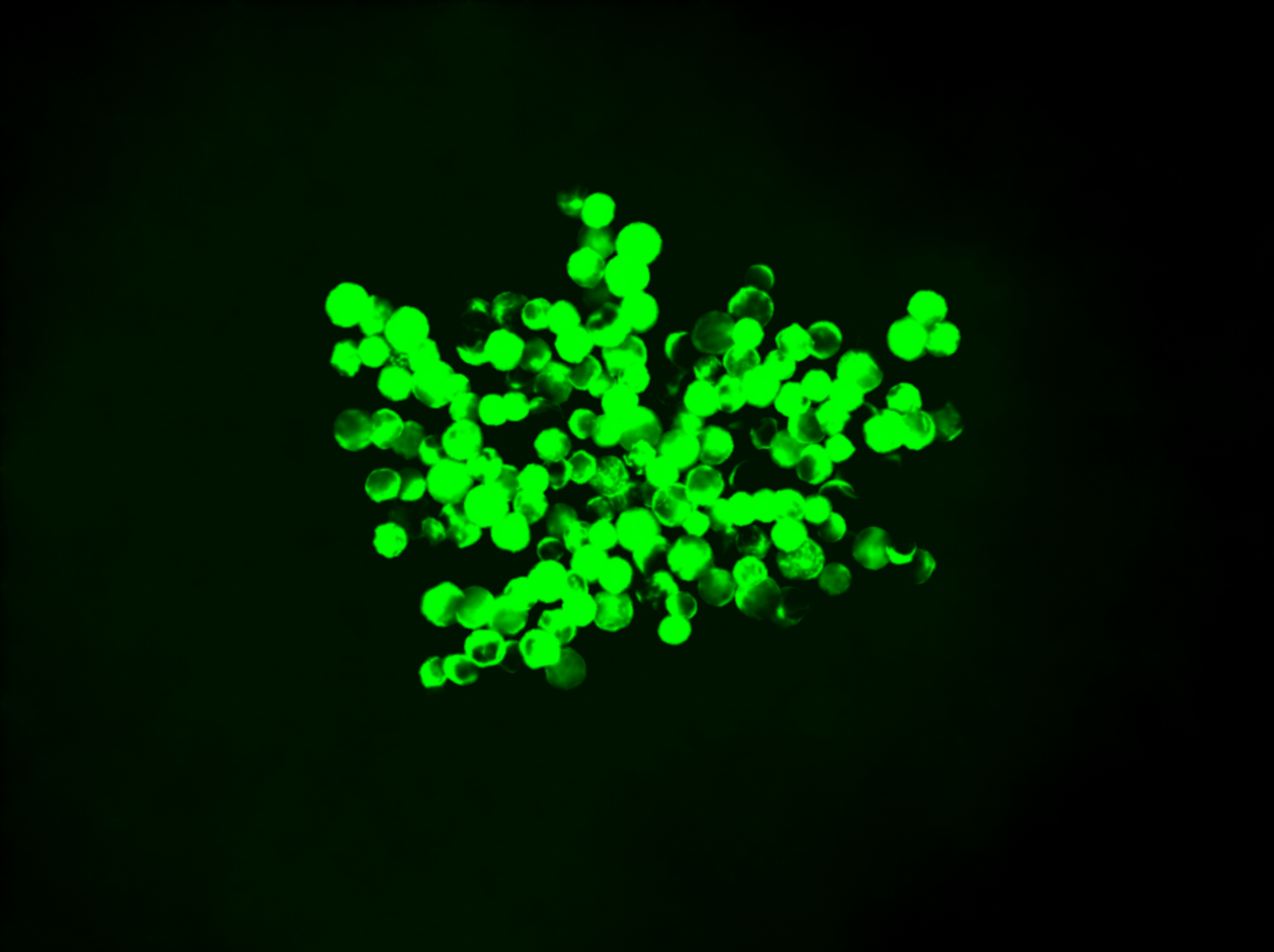

Supplement: Additional file 5 — The zip archive contains simulated images showing protoplasts with corresponding ground truth. (ZIP 72704 kb) [file 12859_2017_1591_MOESM5_ESM.zip › simulated protoplasts/overlaying/overlaying009.png]

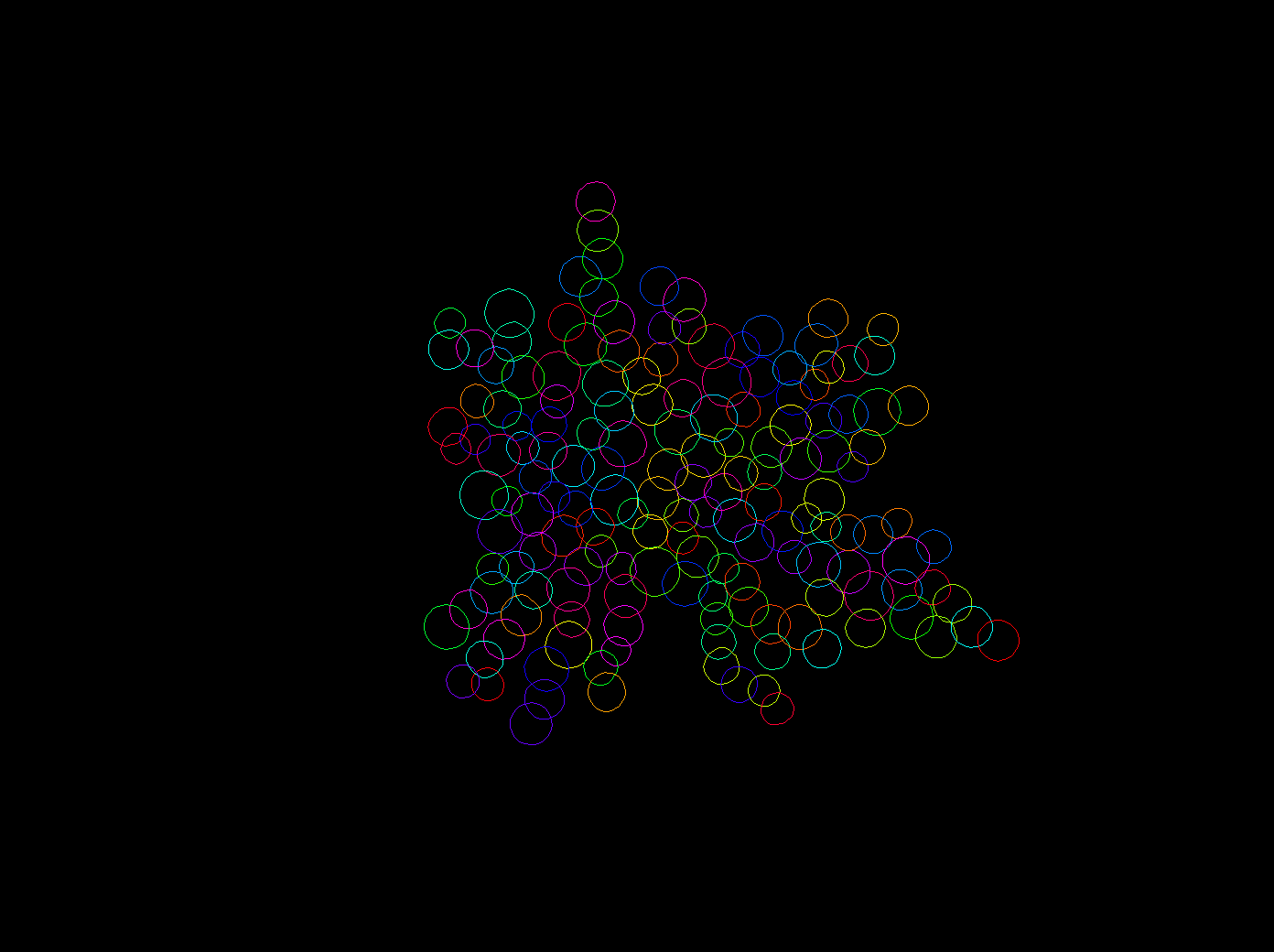

Supplement: Additional file 5 — The zip archive contains simulated images showing protoplasts with corresponding ground truth. (ZIP 72704 kb) [file 12859_2017_1591_MOESM5_ESM.zip › simulated protoplasts/overlaying/overlaying010 gt.png]

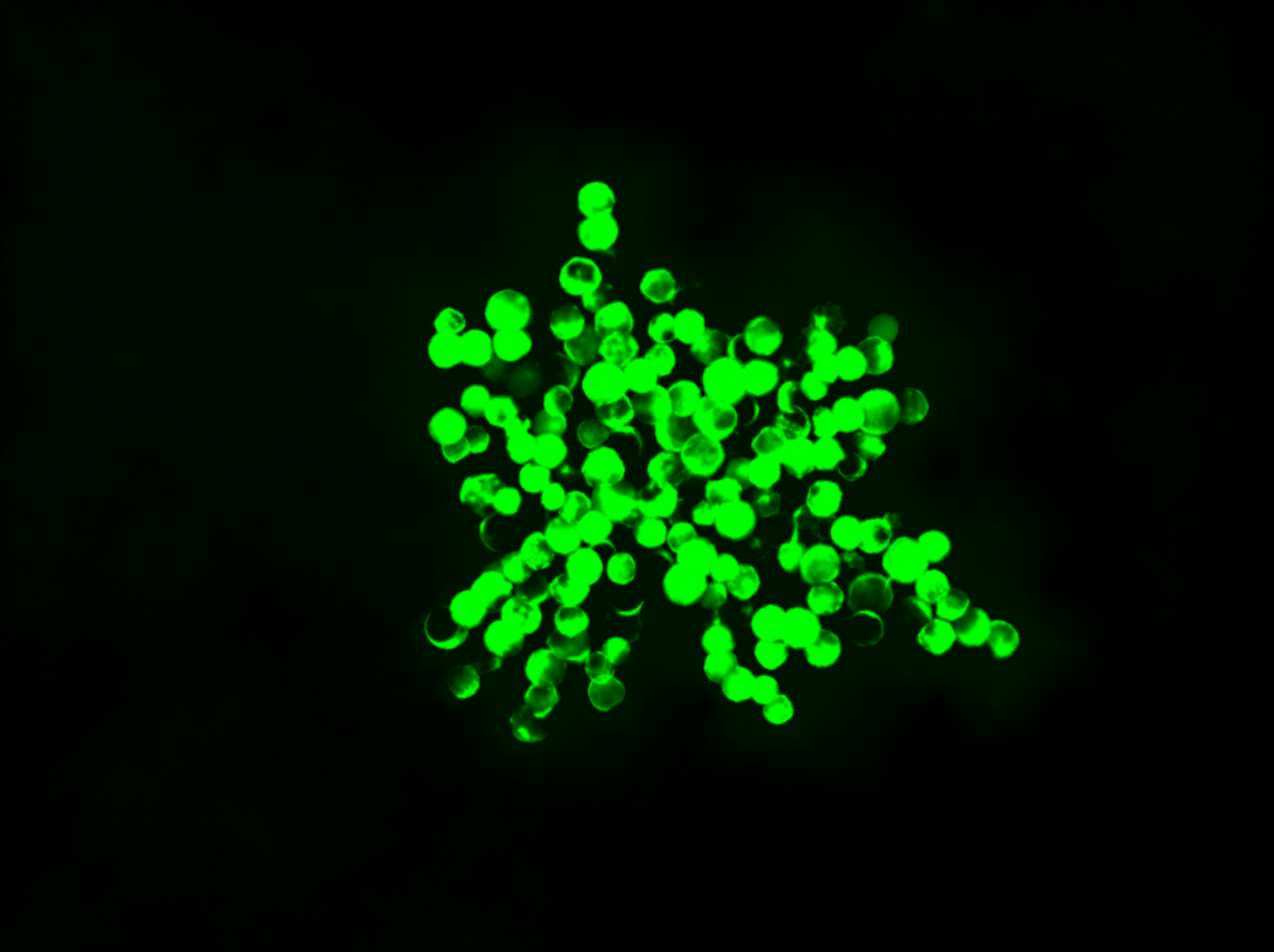

Supplement: Additional file 5 — The zip archive contains simulated images showing protoplasts with corresponding ground truth. (ZIP 72704 kb) [file 12859_2017_1591_MOESM5_ESM.zip › simulated protoplasts/overlaying/overlaying010.png]
