# Supplementary figures and images for: Using simulated fluorescence cell micrographs for the evaluation of cell image segmentation algorithms (part 6 of 6)
Source: BMC Bioinformatics. 2017 Mar 18;18:176. doi: 10.1186/s12859-017-1591-2 (PMC5357336; doi:10.1186/s12859-017-1591-2)

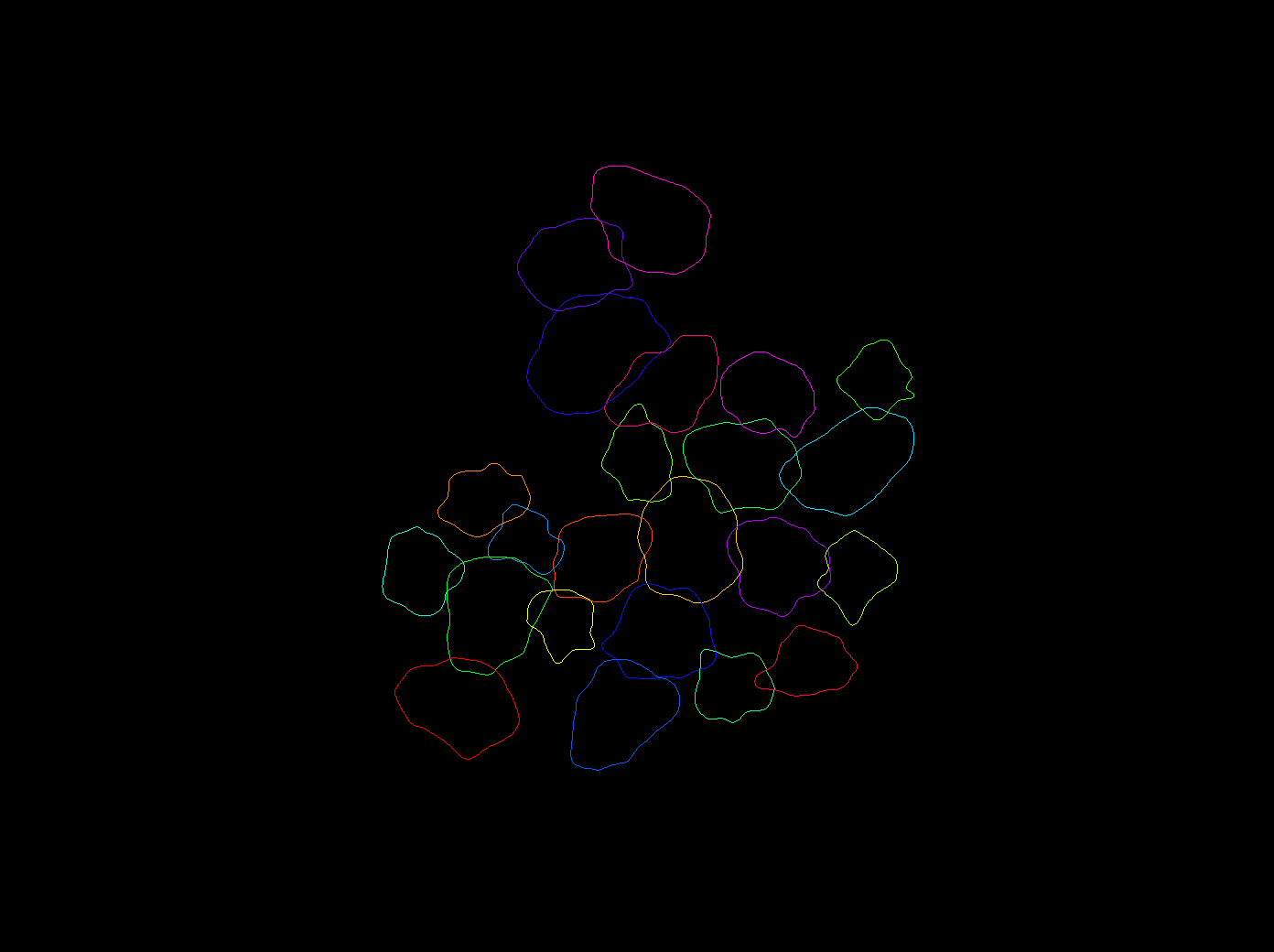

Supplement: Additional file 6 — The zip archive contains simulated images showing B cell nuclei and cytoskeleton with corresponding ground truth. (ZIP 119808 kb) [file 12859_2017_1591_MOESM6_ESM.zip › simulated B cells/cytoskeleton/overlapping/cell018 gt.png]

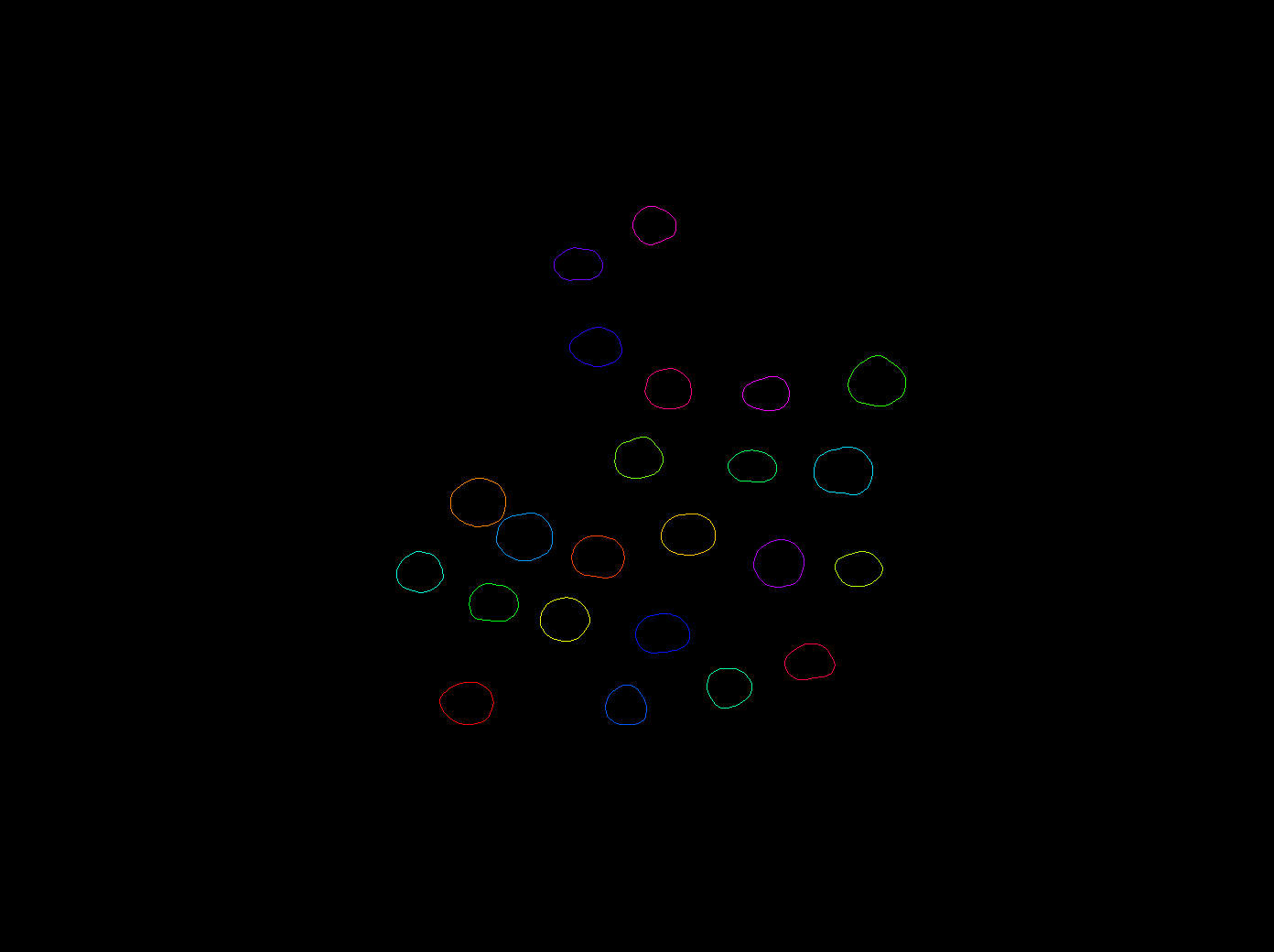

Supplement: Additional file 6 — The zip archive contains simulated images showing B cell nuclei and cytoskeleton with corresponding ground truth. (ZIP 119808 kb) [file 12859_2017_1591_MOESM6_ESM.zip › simulated B cells/cytoskeleton/overlapping/cell018 seeds.png]

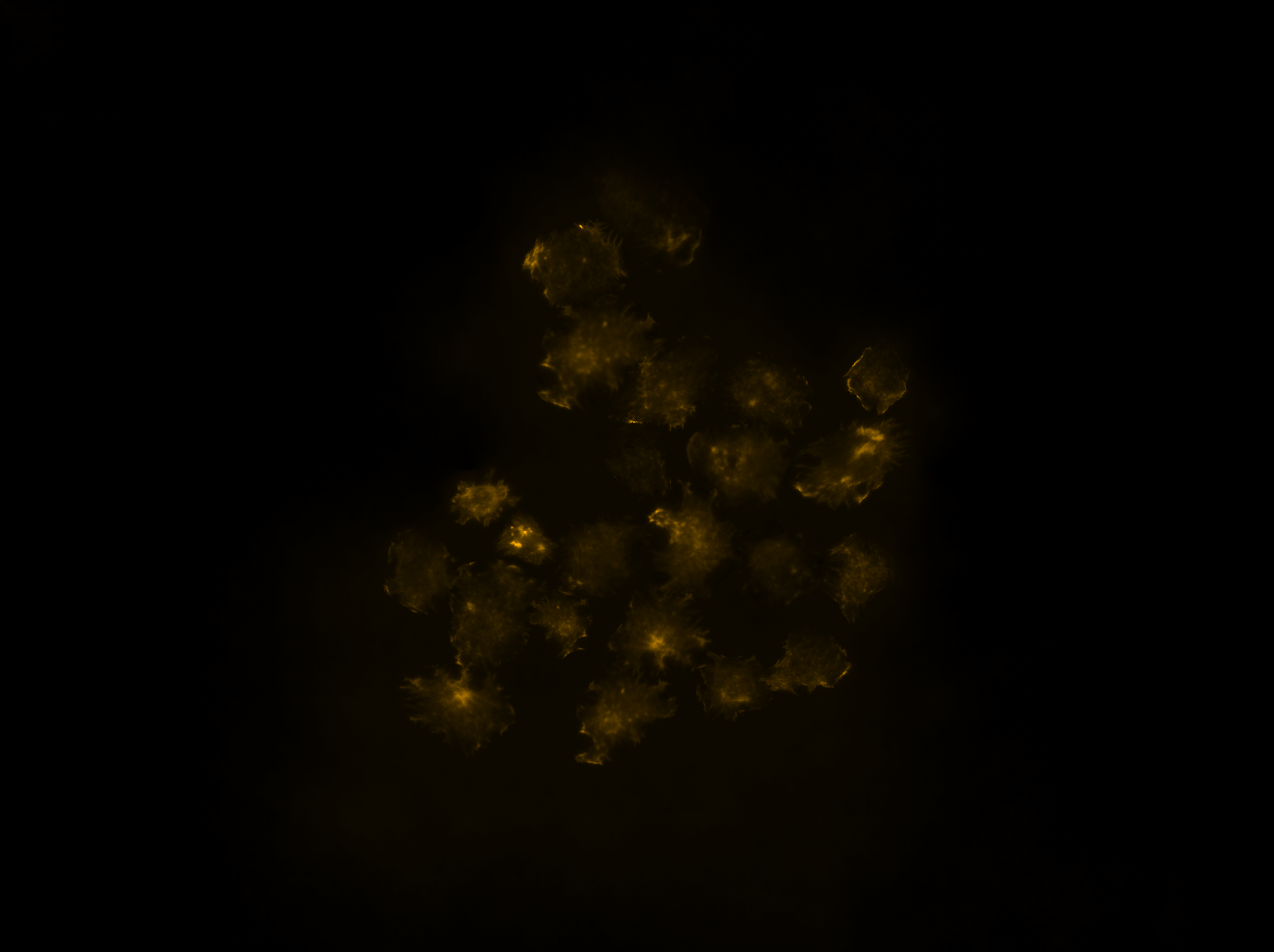

Supplement: Additional file 6 — The zip archive contains simulated images showing B cell nuclei and cytoskeleton with corresponding ground truth. (ZIP 119808 kb) [file 12859_2017_1591_MOESM6_ESM.zip › simulated B cells/cytoskeleton/overlapping/cell018.png]

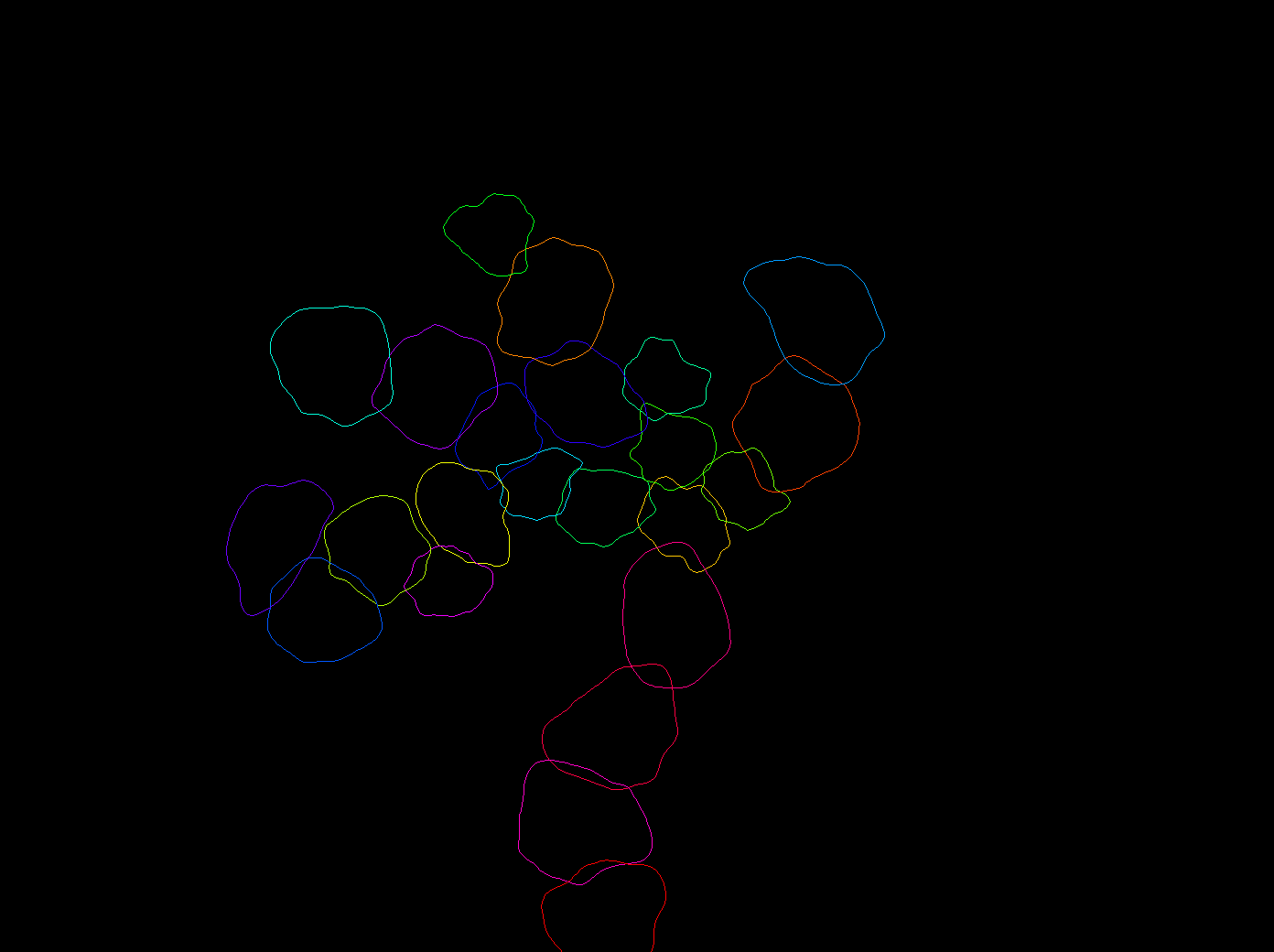

Supplement: Additional file 6 — The zip archive contains simulated images showing B cell nuclei and cytoskeleton with corresponding ground truth. (ZIP 119808 kb) [file 12859_2017_1591_MOESM6_ESM.zip › simulated B cells/cytoskeleton/overlapping/cell019 gt.png]

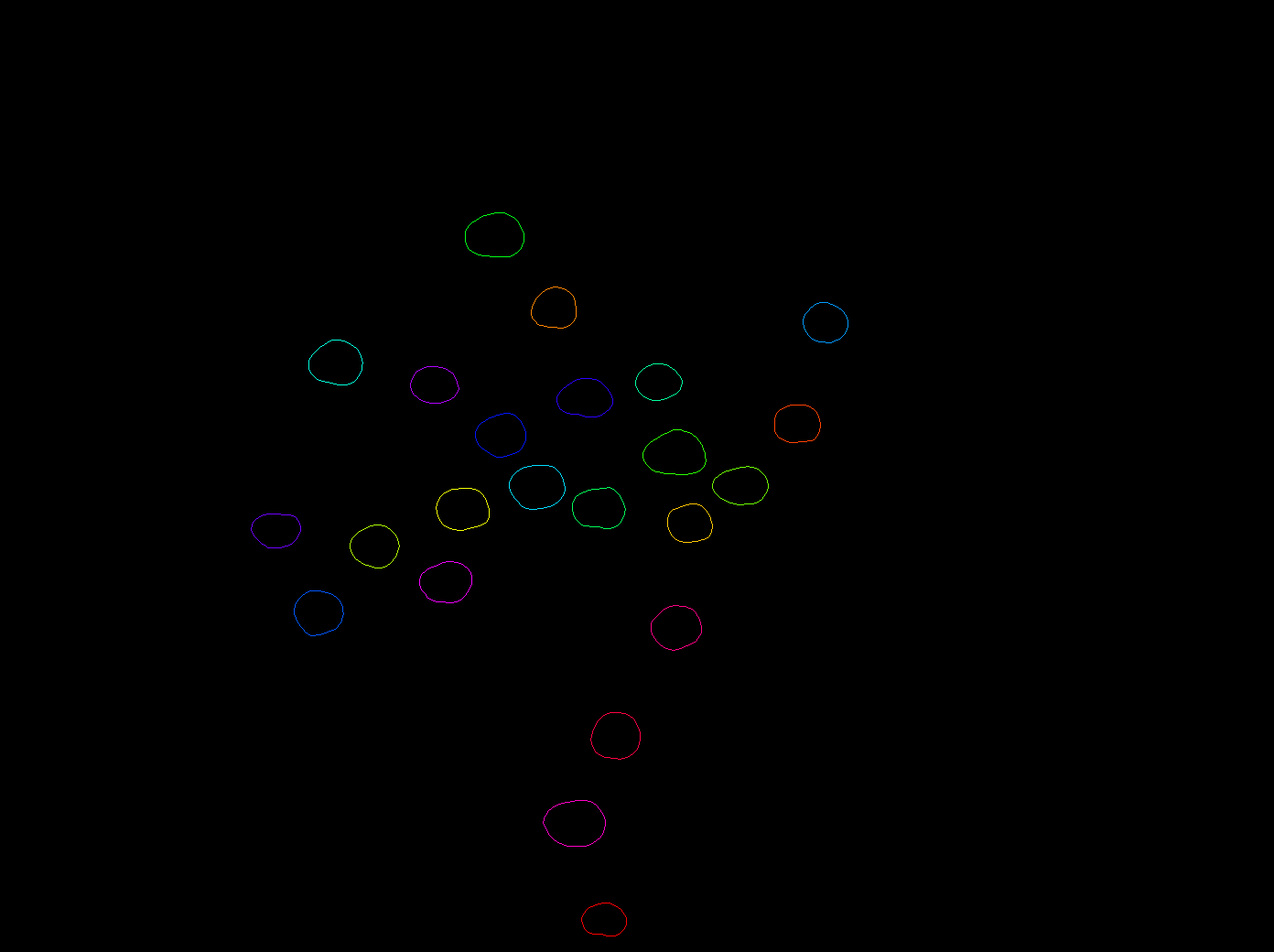

Supplement: Additional file 6 — The zip archive contains simulated images showing B cell nuclei and cytoskeleton with corresponding ground truth. (ZIP 119808 kb) [file 12859_2017_1591_MOESM6_ESM.zip › simulated B cells/cytoskeleton/overlapping/cell019 seeds.png]

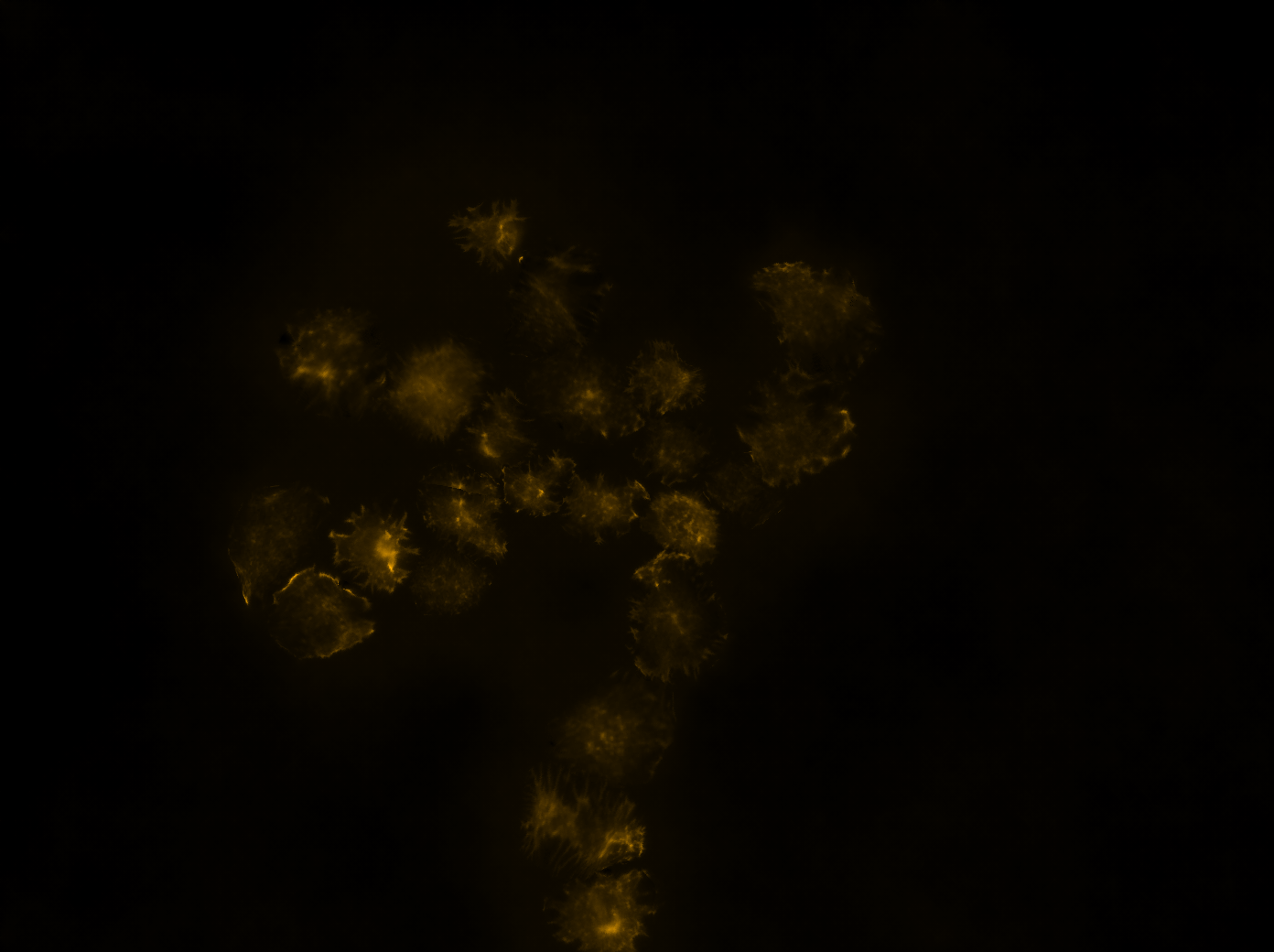

Supplement: Additional file 6 — The zip archive contains simulated images showing B cell nuclei and cytoskeleton with corresponding ground truth. (ZIP 119808 kb) [file 12859_2017_1591_MOESM6_ESM.zip › simulated B cells/cytoskeleton/overlapping/cell019.png]

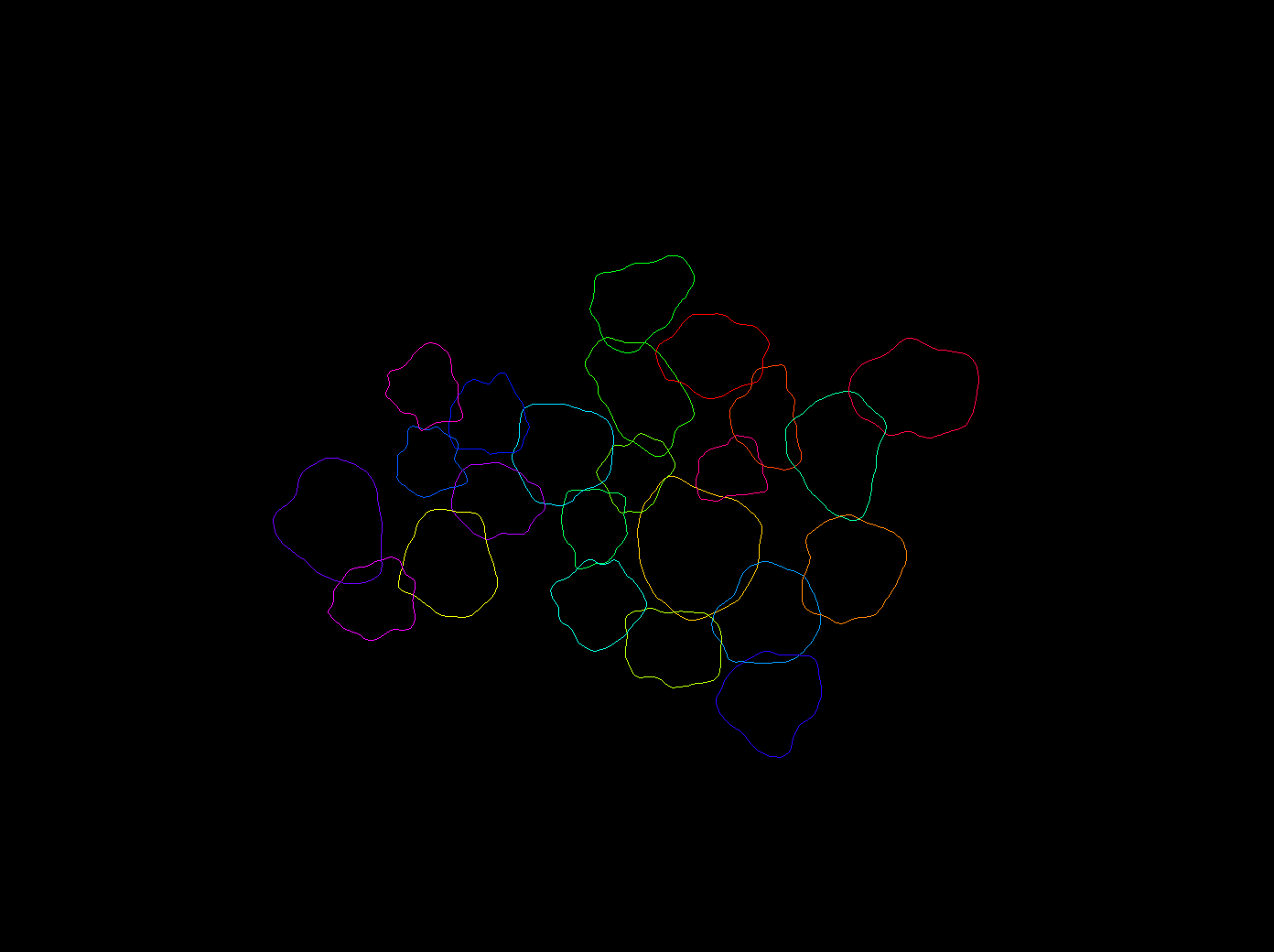

Supplement: Additional file 6 — The zip archive contains simulated images showing B cell nuclei and cytoskeleton with corresponding ground truth. (ZIP 119808 kb) [file 12859_2017_1591_MOESM6_ESM.zip › simulated B cells/cytoskeleton/overlapping/cell020 gt.png]

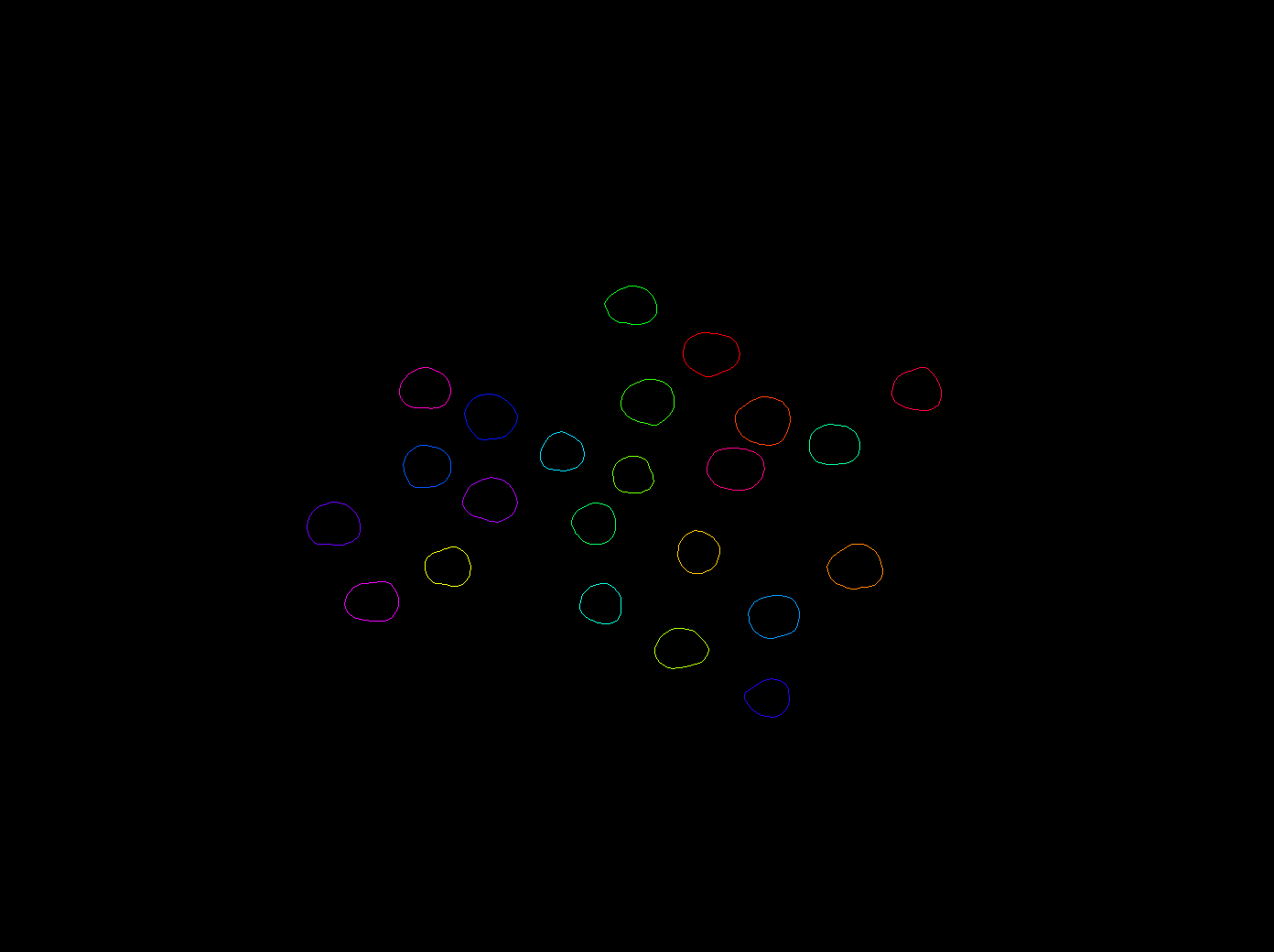

Supplement: Additional file 6 — The zip archive contains simulated images showing B cell nuclei and cytoskeleton with corresponding ground truth. (ZIP 119808 kb) [file 12859_2017_1591_MOESM6_ESM.zip › simulated B cells/cytoskeleton/overlapping/cell020 seeds.png]

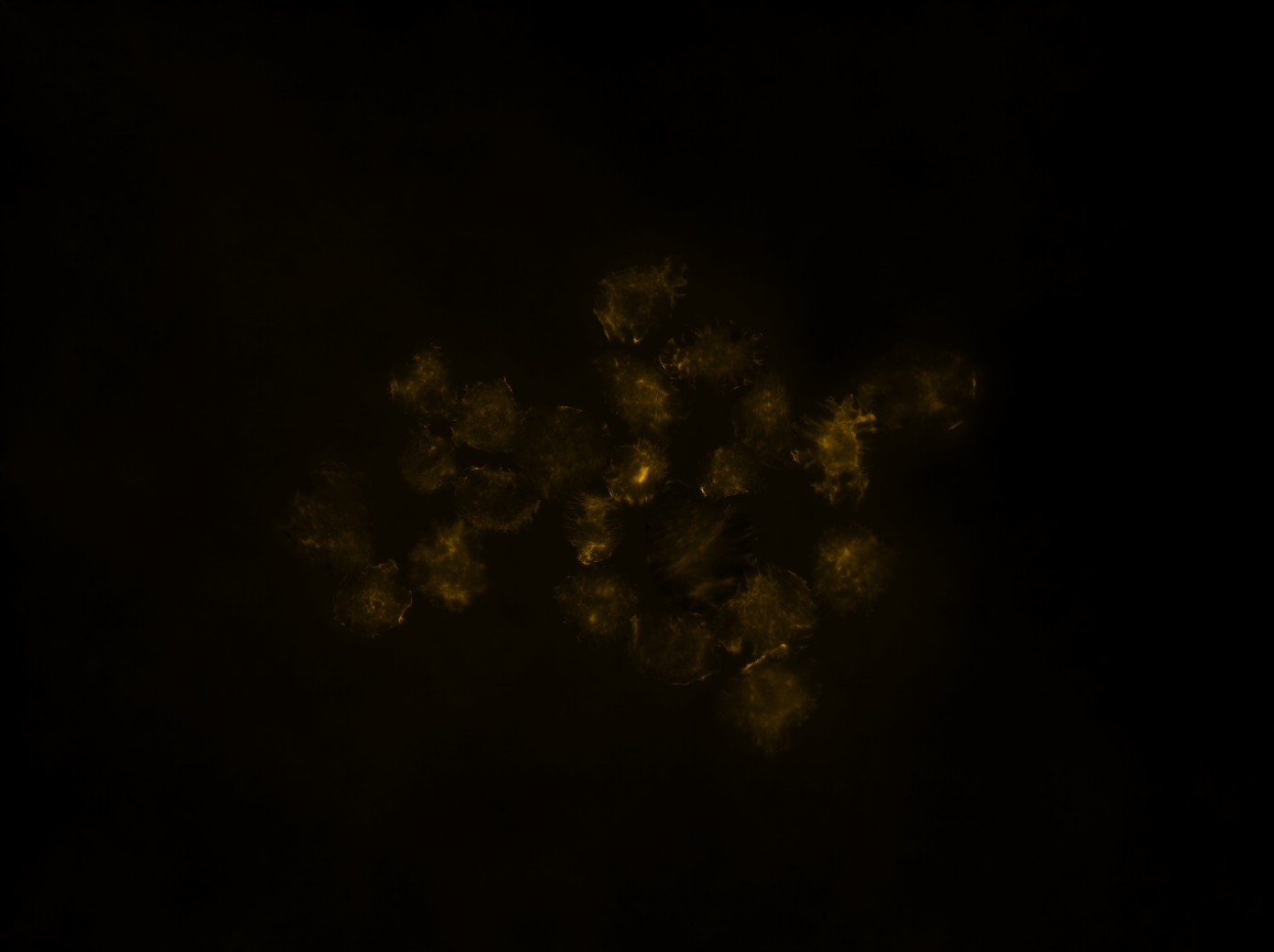

Supplement: Additional file 6 — The zip archive contains simulated images showing B cell nuclei and cytoskeleton with corresponding ground truth. (ZIP 119808 kb) [file 12859_2017_1591_MOESM6_ESM.zip › simulated B cells/cytoskeleton/overlapping/cell020.png]

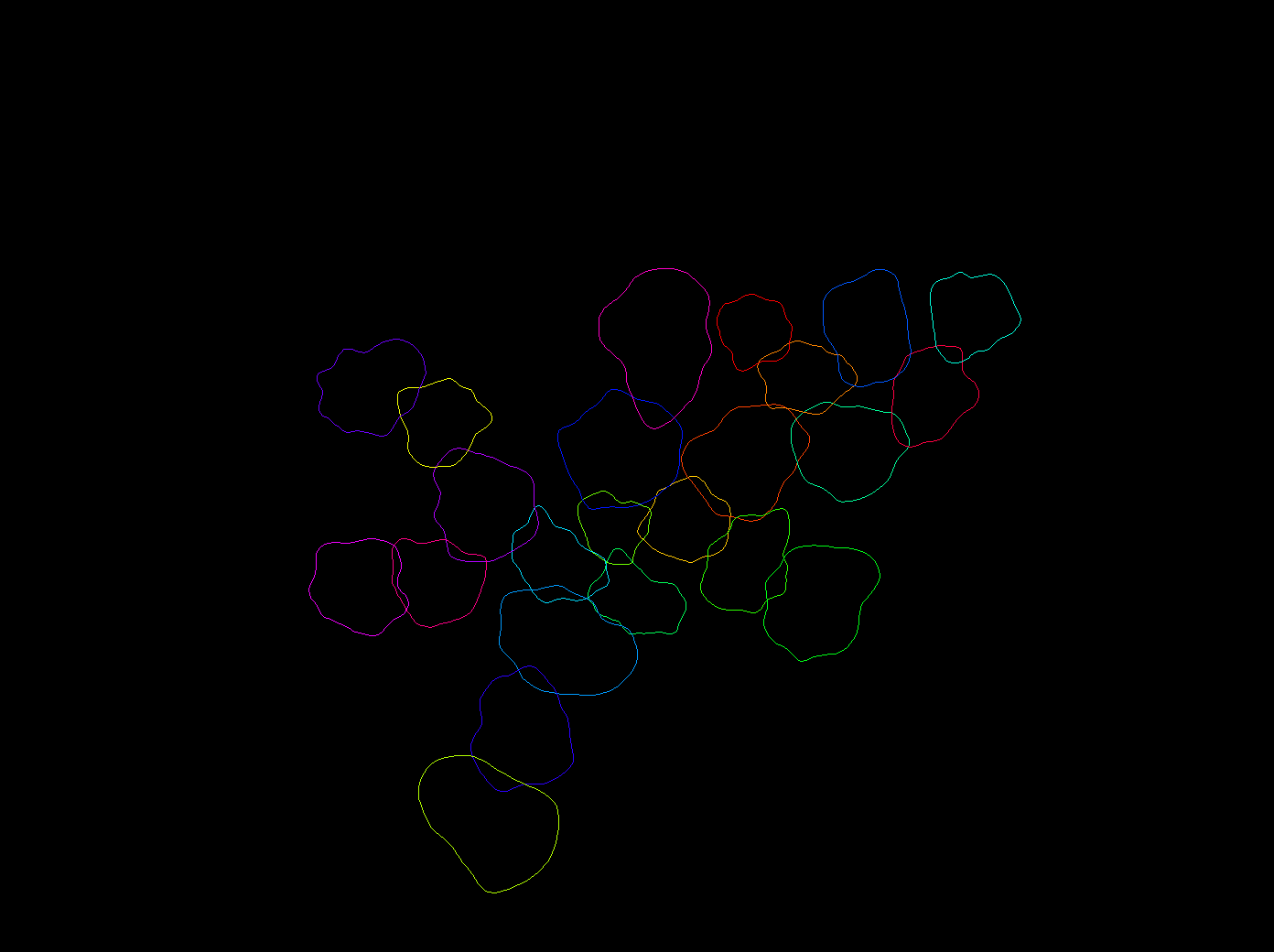

Supplement: Additional file 6 — The zip archive contains simulated images showing B cell nuclei and cytoskeleton with corresponding ground truth. (ZIP 119808 kb) [file 12859_2017_1591_MOESM6_ESM.zip › simulated B cells/cytoskeleton/overlapping/cell021 gt.png]

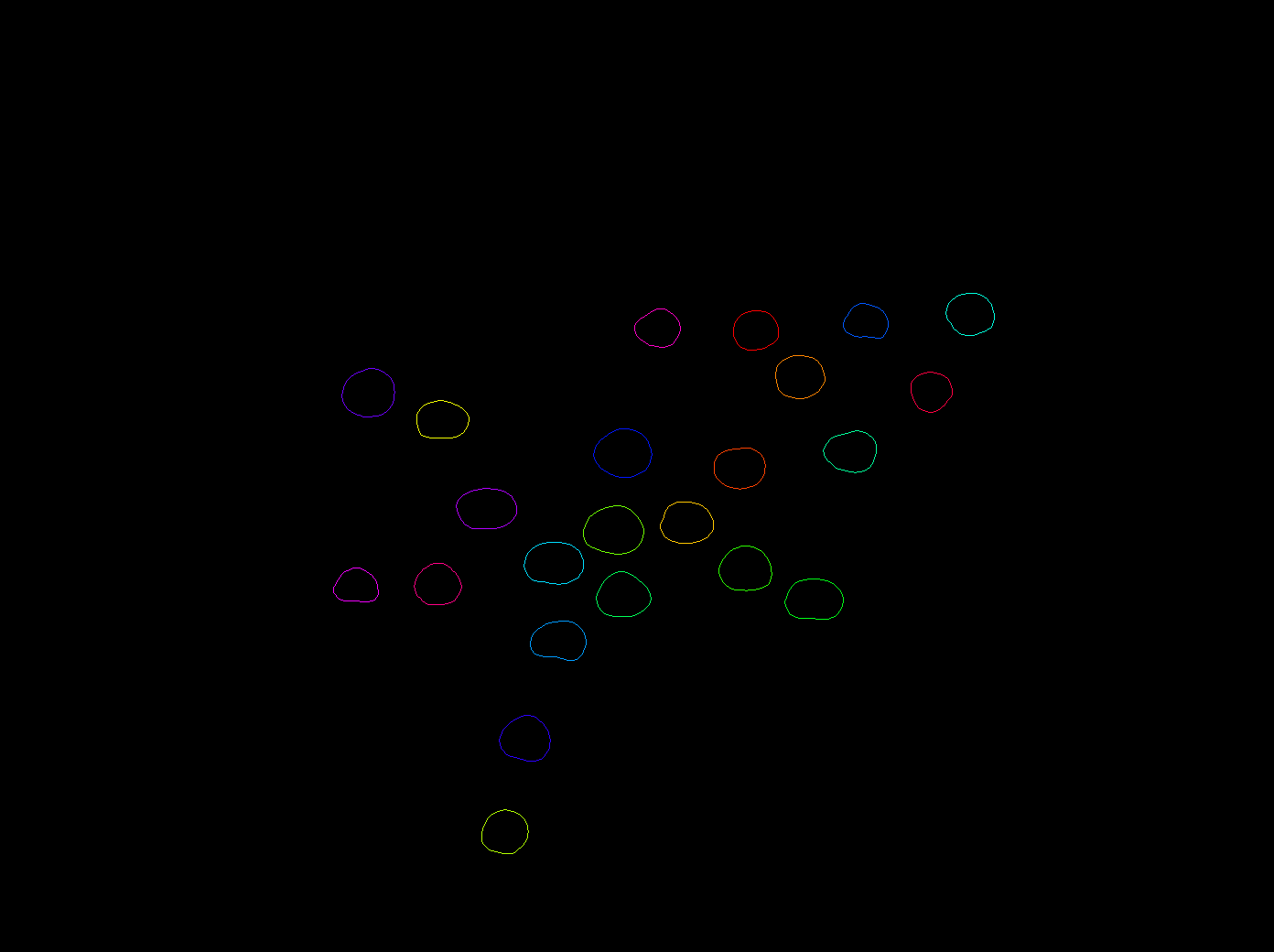

Supplement: Additional file 6 — The zip archive contains simulated images showing B cell nuclei and cytoskeleton with corresponding ground truth. (ZIP 119808 kb) [file 12859_2017_1591_MOESM6_ESM.zip › simulated B cells/cytoskeleton/overlapping/cell021 seeds.png]

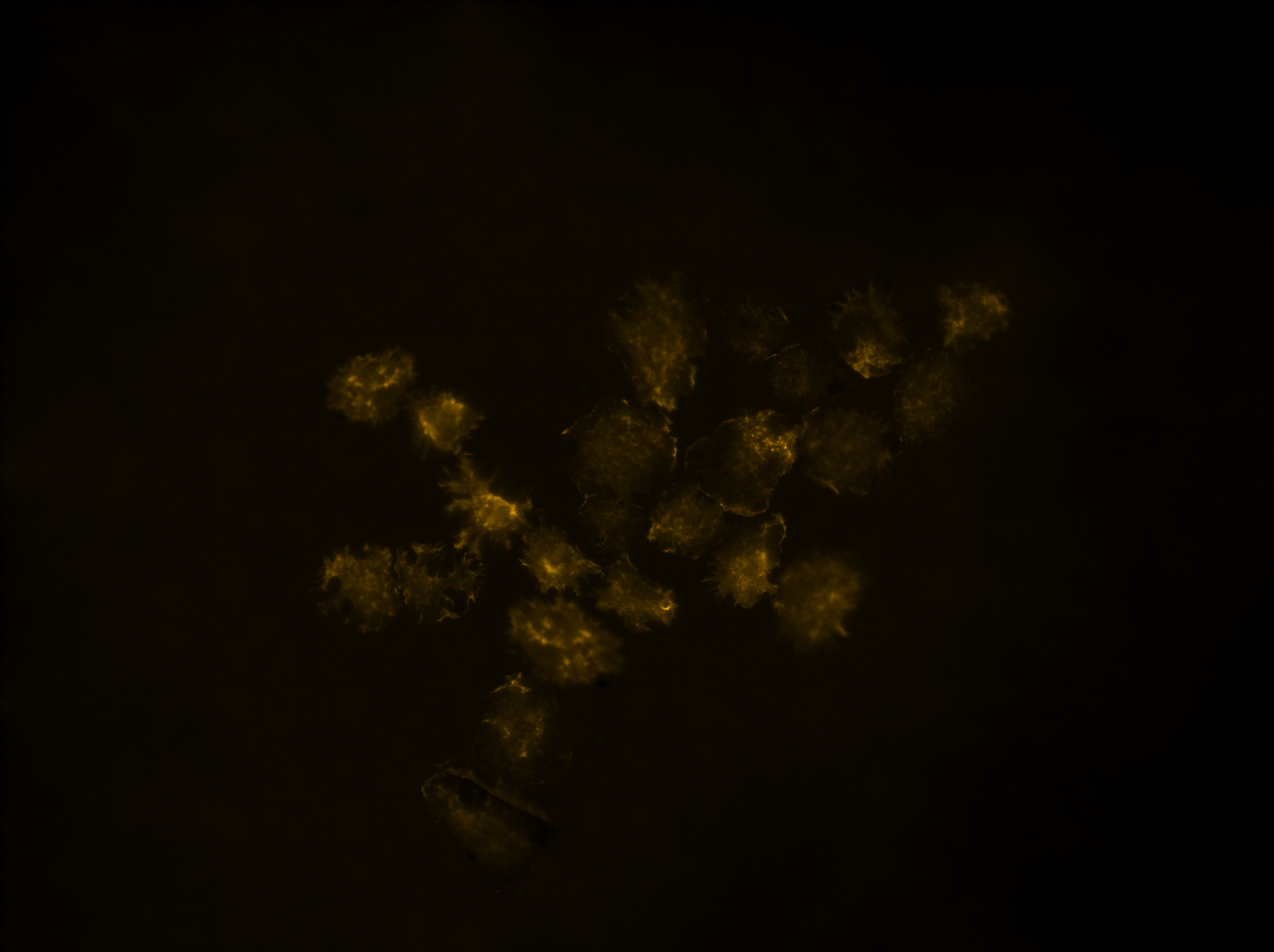

Supplement: Additional file 6 — The zip archive contains simulated images showing B cell nuclei and cytoskeleton with corresponding ground truth. (ZIP 119808 kb) [file 12859_2017_1591_MOESM6_ESM.zip › simulated B cells/cytoskeleton/overlapping/cell021.png]

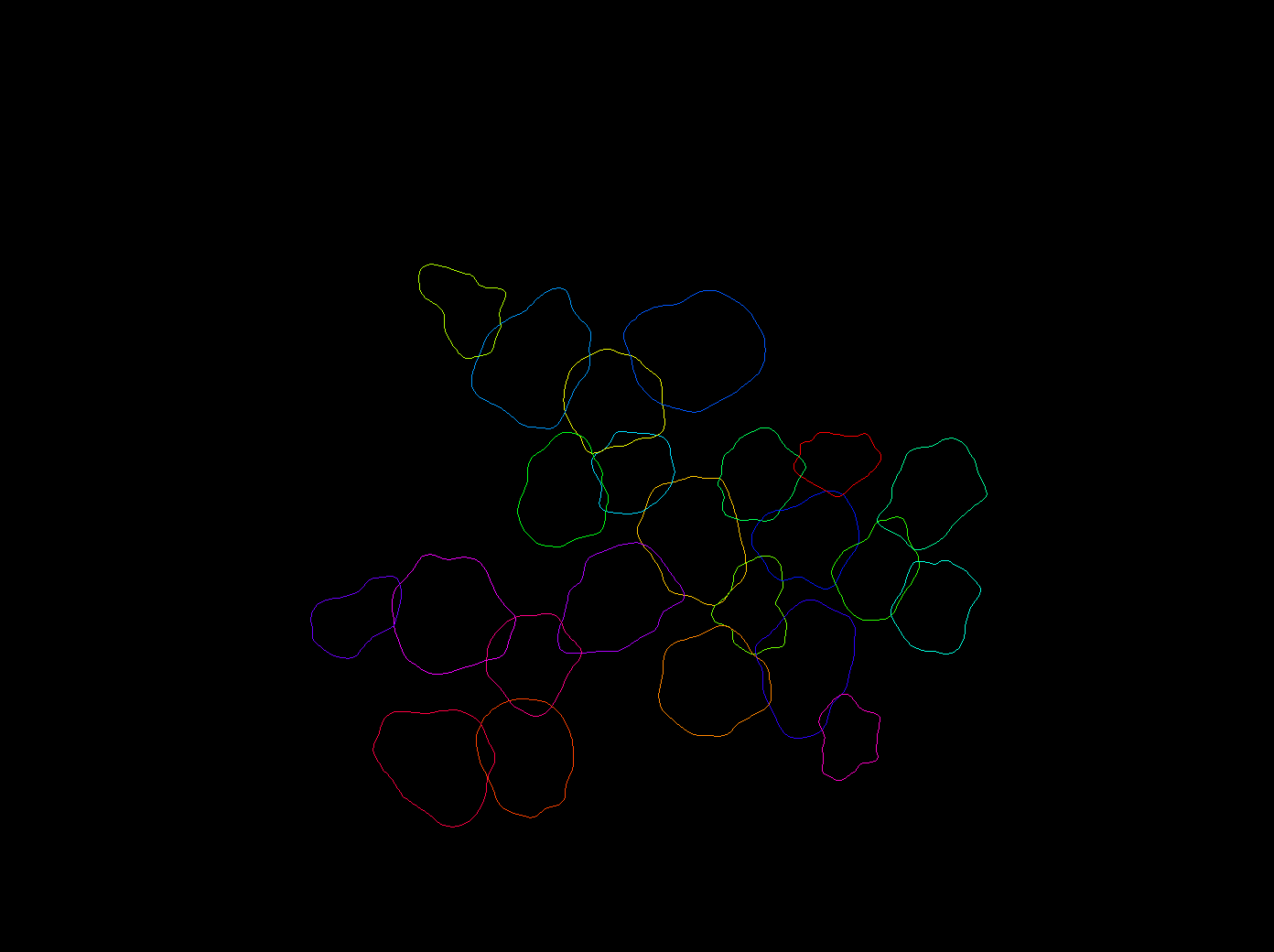

Supplement: Additional file 6 — The zip archive contains simulated images showing B cell nuclei and cytoskeleton with corresponding ground truth. (ZIP 119808 kb) [file 12859_2017_1591_MOESM6_ESM.zip › simulated B cells/cytoskeleton/overlapping/cell022 gt.png]

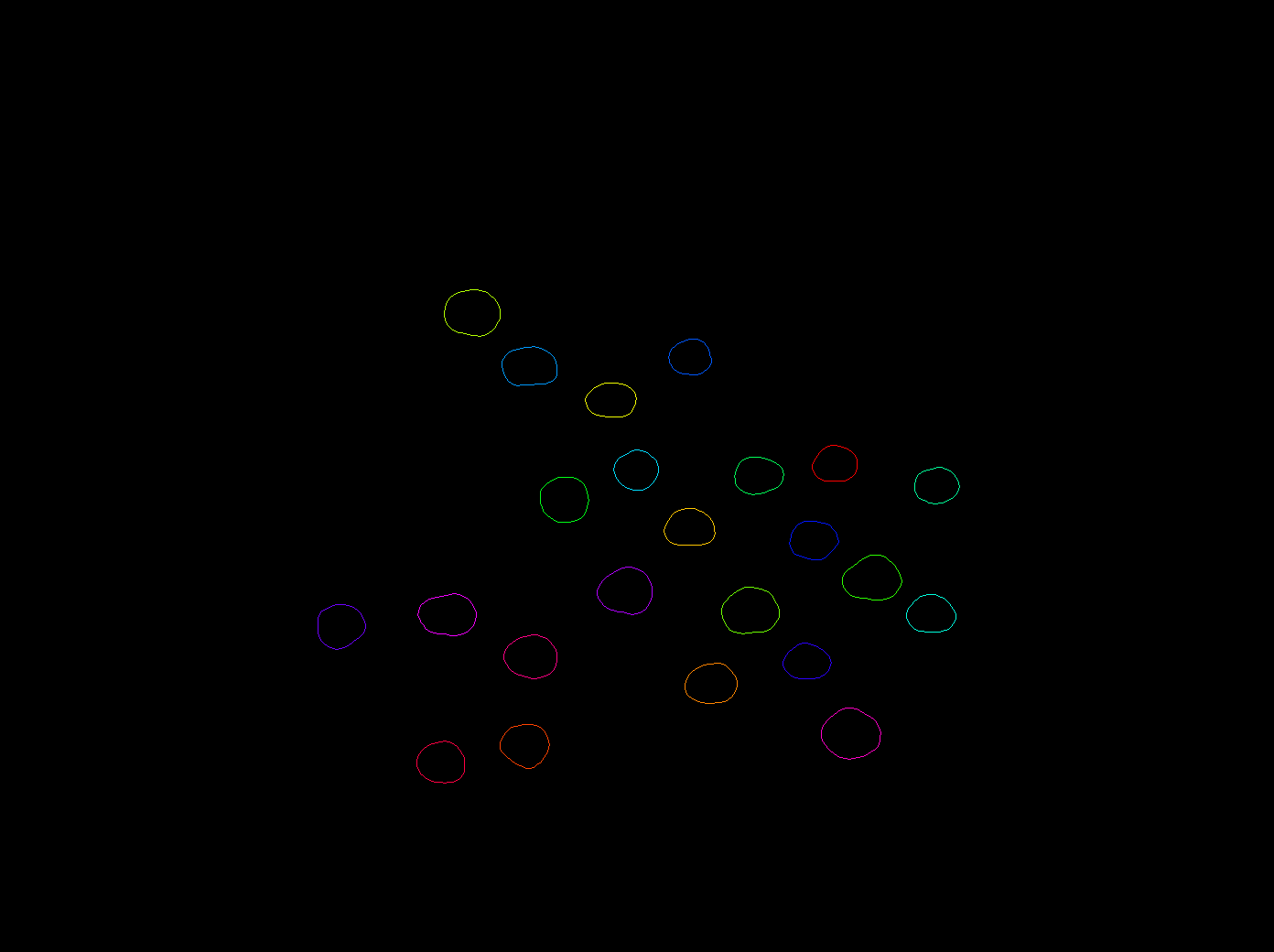

Supplement: Additional file 6 — The zip archive contains simulated images showing B cell nuclei and cytoskeleton with corresponding ground truth. (ZIP 119808 kb) [file 12859_2017_1591_MOESM6_ESM.zip › simulated B cells/cytoskeleton/overlapping/cell022 seeds.png]

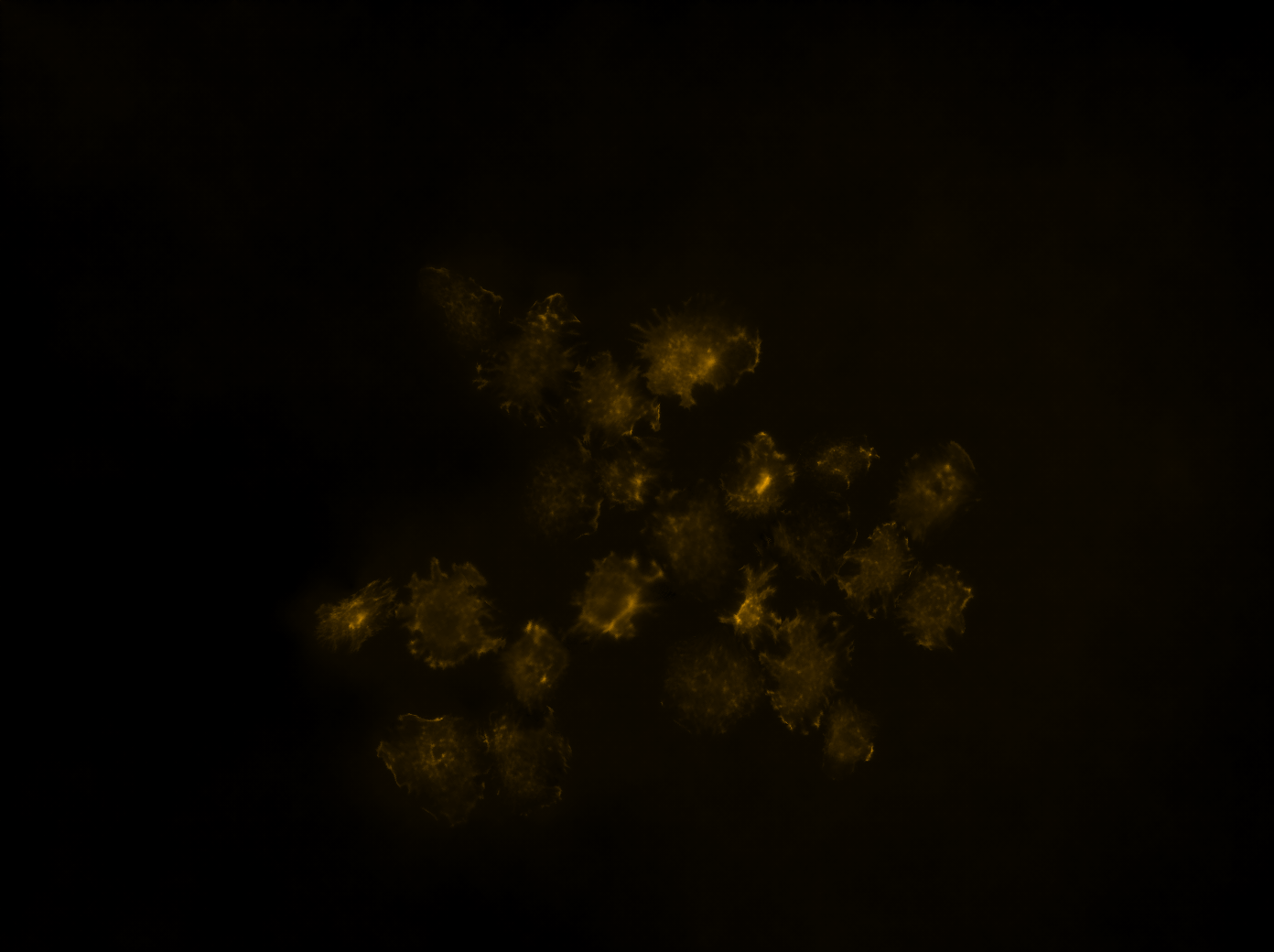

Supplement: Additional file 6 — The zip archive contains simulated images showing B cell nuclei and cytoskeleton with corresponding ground truth. (ZIP 119808 kb) [file 12859_2017_1591_MOESM6_ESM.zip › simulated B cells/cytoskeleton/overlapping/cell022.png]

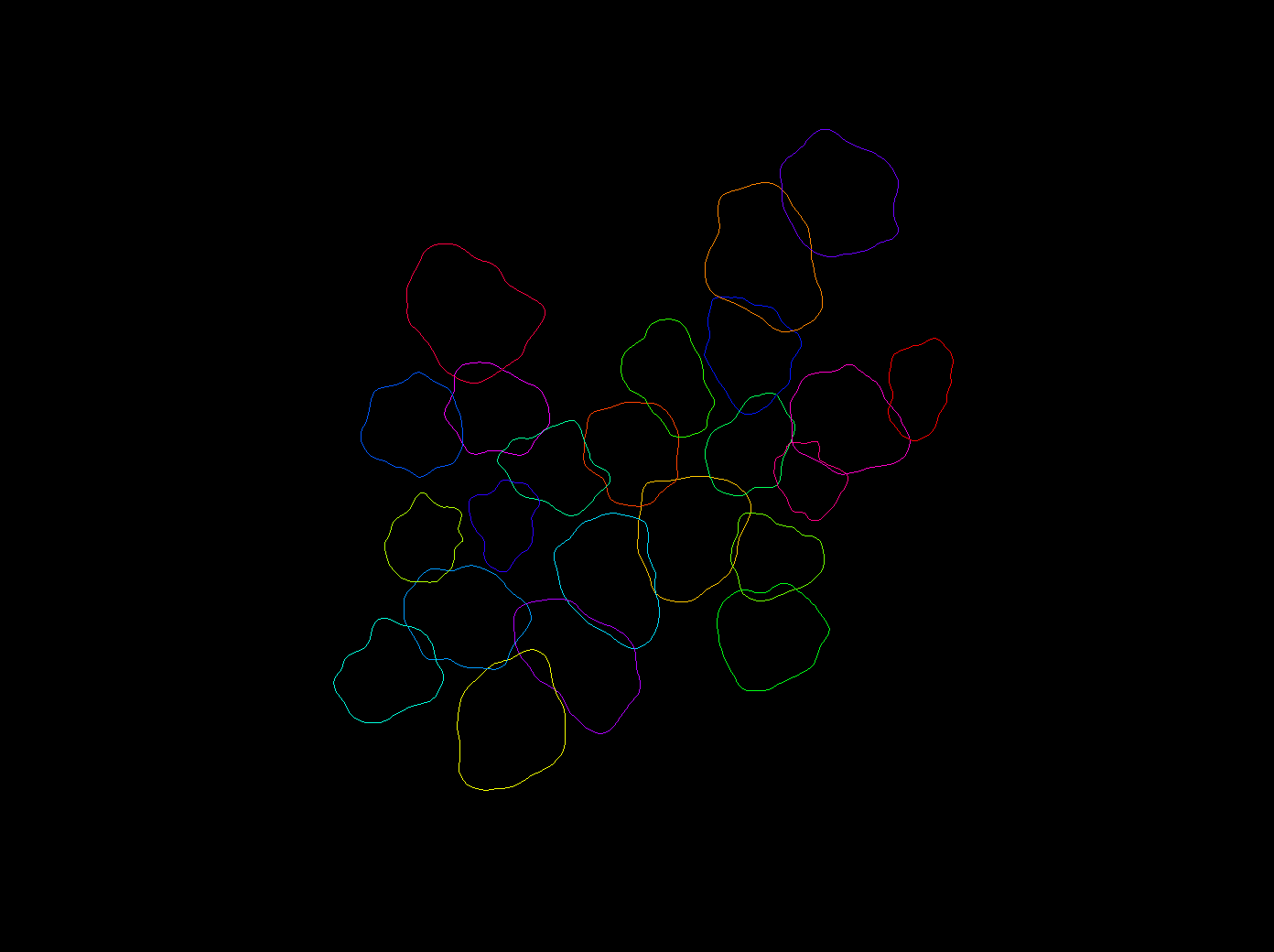

Supplement: Additional file 6 — The zip archive contains simulated images showing B cell nuclei and cytoskeleton with corresponding ground truth. (ZIP 119808 kb) [file 12859_2017_1591_MOESM6_ESM.zip › simulated B cells/cytoskeleton/overlapping/cell023 gt.png]

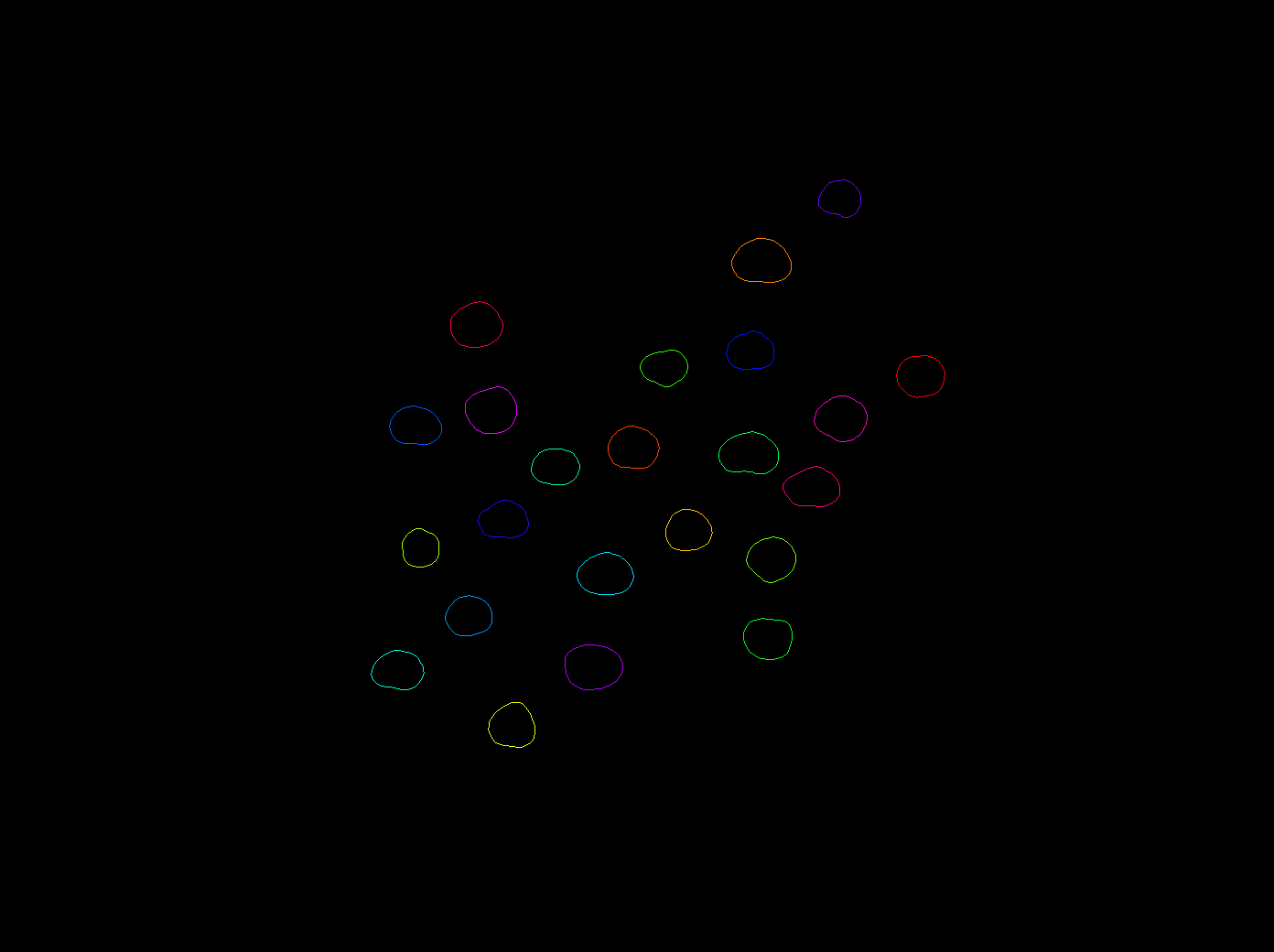

Supplement: Additional file 6 — The zip archive contains simulated images showing B cell nuclei and cytoskeleton with corresponding ground truth. (ZIP 119808 kb) [file 12859_2017_1591_MOESM6_ESM.zip › simulated B cells/cytoskeleton/overlapping/cell023 seeds.png]

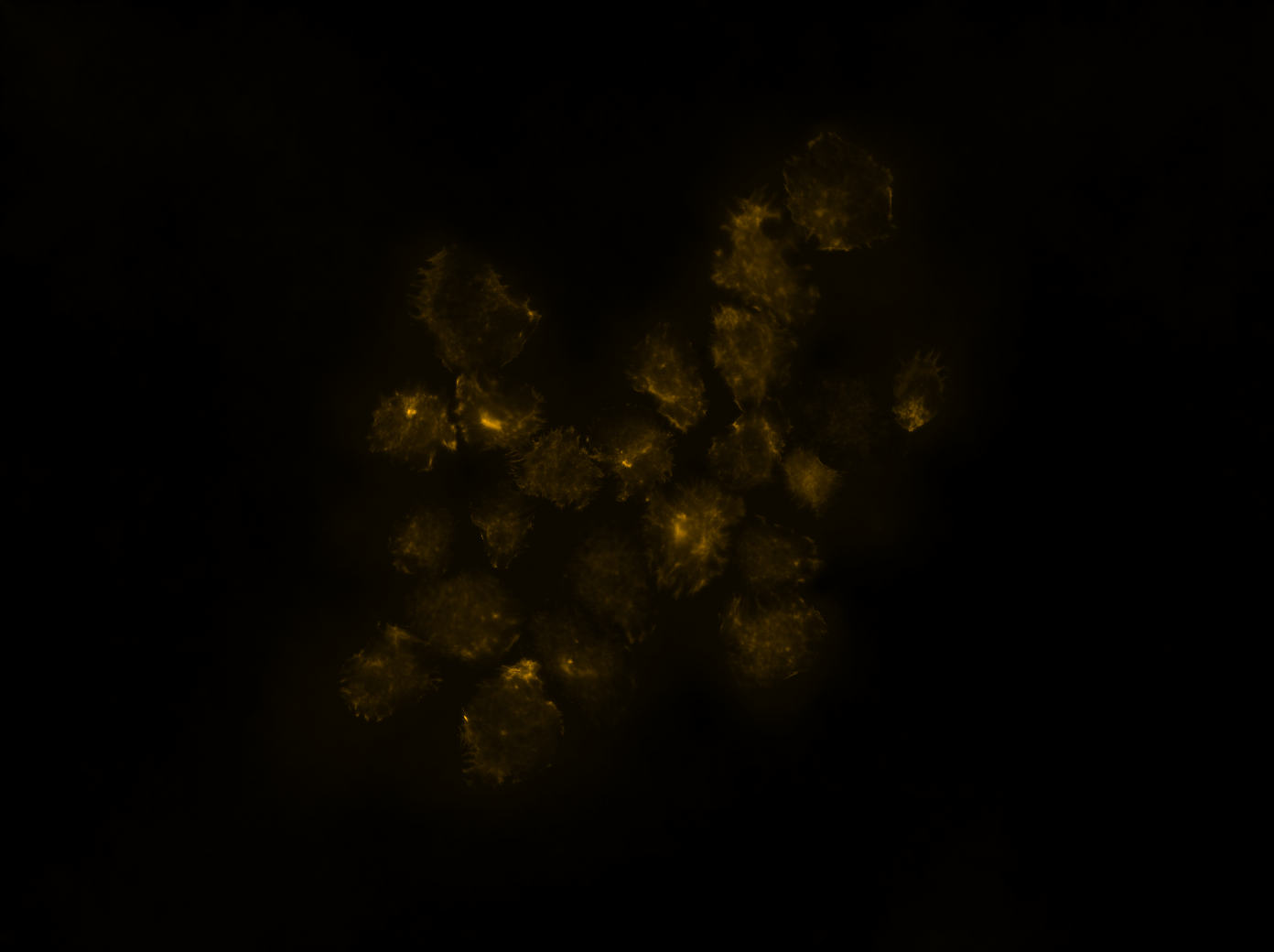

Supplement: Additional file 6 — The zip archive contains simulated images showing B cell nuclei and cytoskeleton with corresponding ground truth. (ZIP 119808 kb) [file 12859_2017_1591_MOESM6_ESM.zip › simulated B cells/cytoskeleton/overlapping/cell023.png]

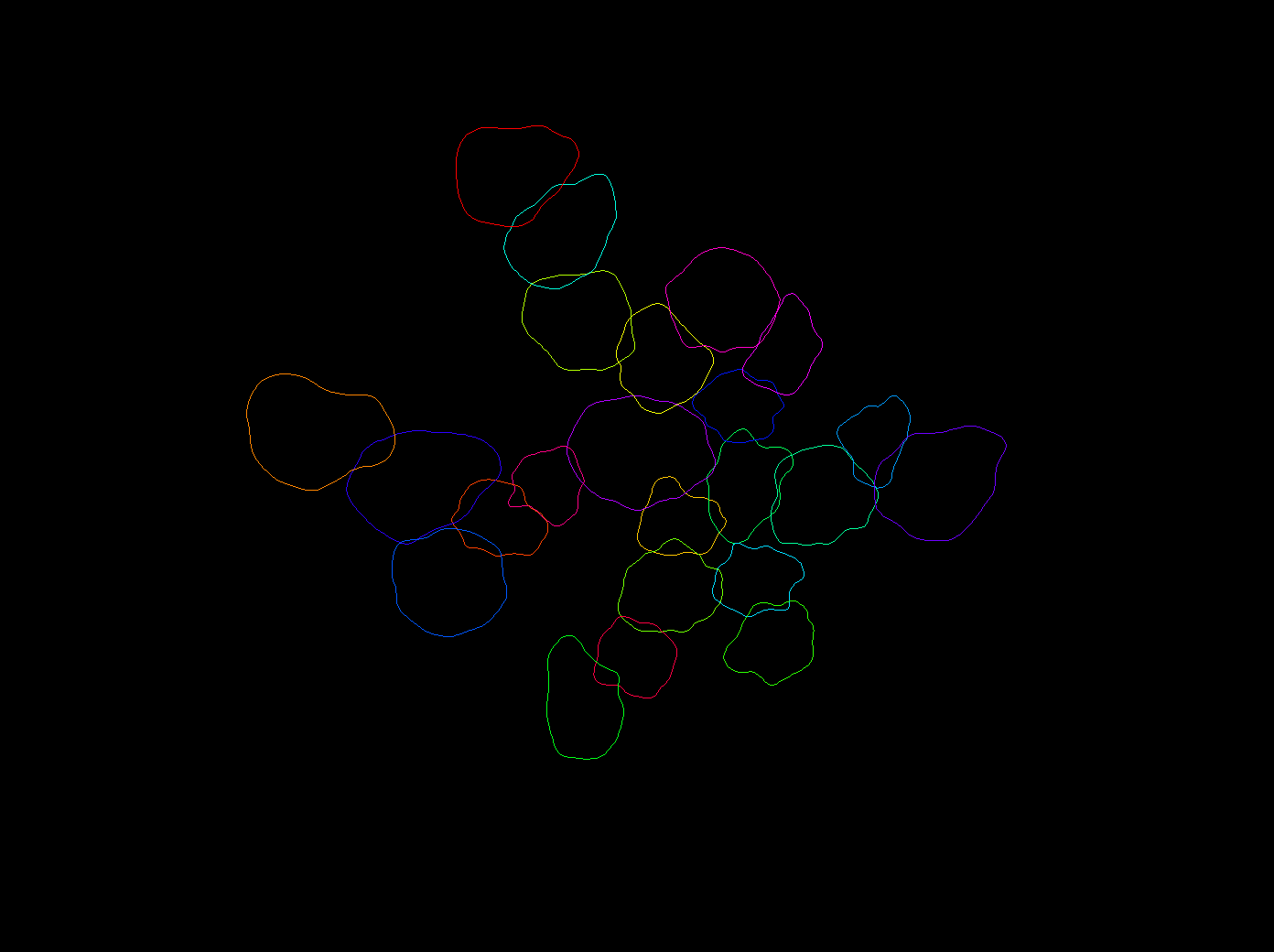

Supplement: Additional file 6 — The zip archive contains simulated images showing B cell nuclei and cytoskeleton with corresponding ground truth. (ZIP 119808 kb) [file 12859_2017_1591_MOESM6_ESM.zip › simulated B cells/cytoskeleton/overlapping/cell024 gt.png]

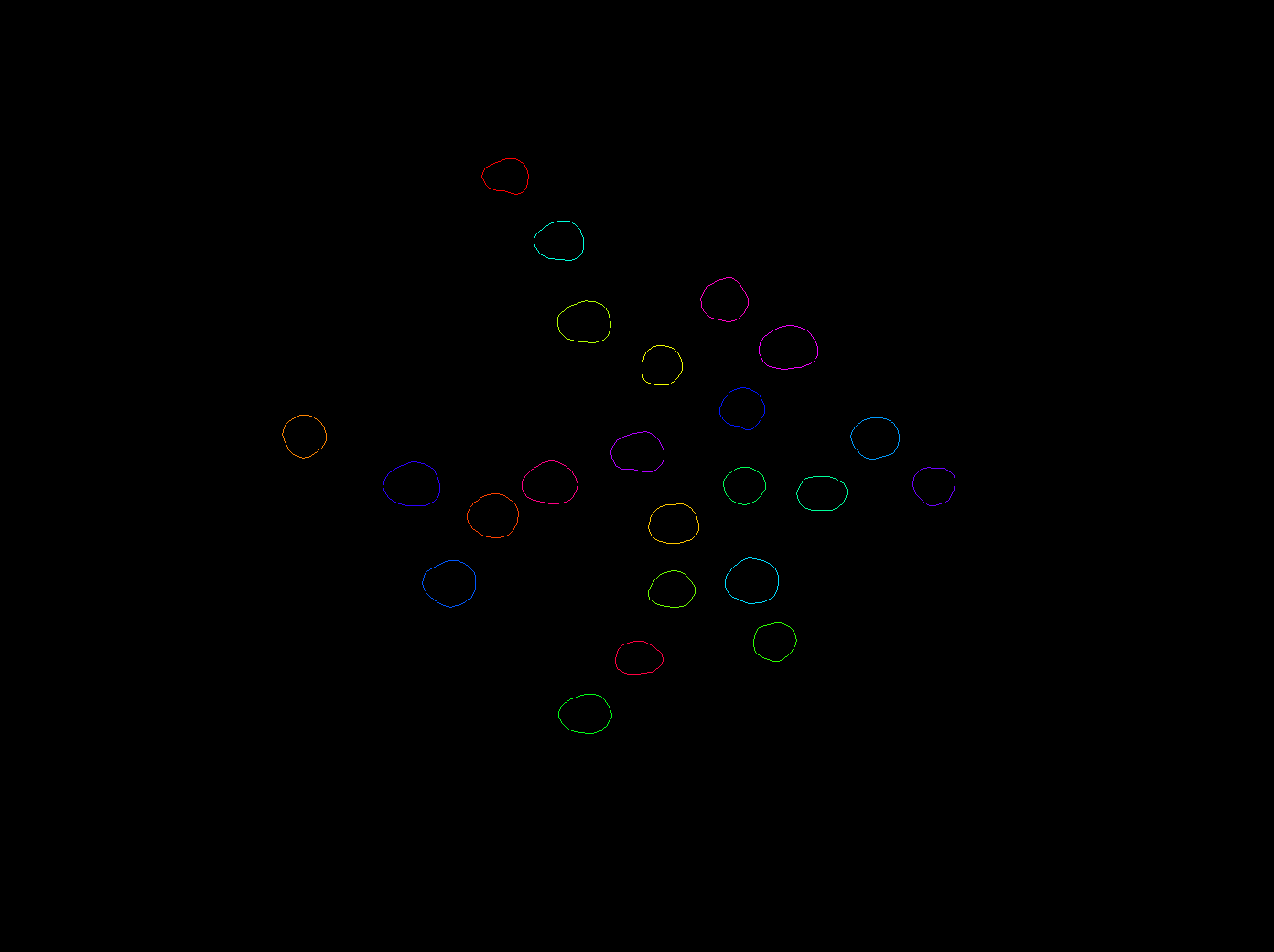

Supplement: Additional file 6 — The zip archive contains simulated images showing B cell nuclei and cytoskeleton with corresponding ground truth. (ZIP 119808 kb) [file 12859_2017_1591_MOESM6_ESM.zip › simulated B cells/cytoskeleton/overlapping/cell024 seeds.png]

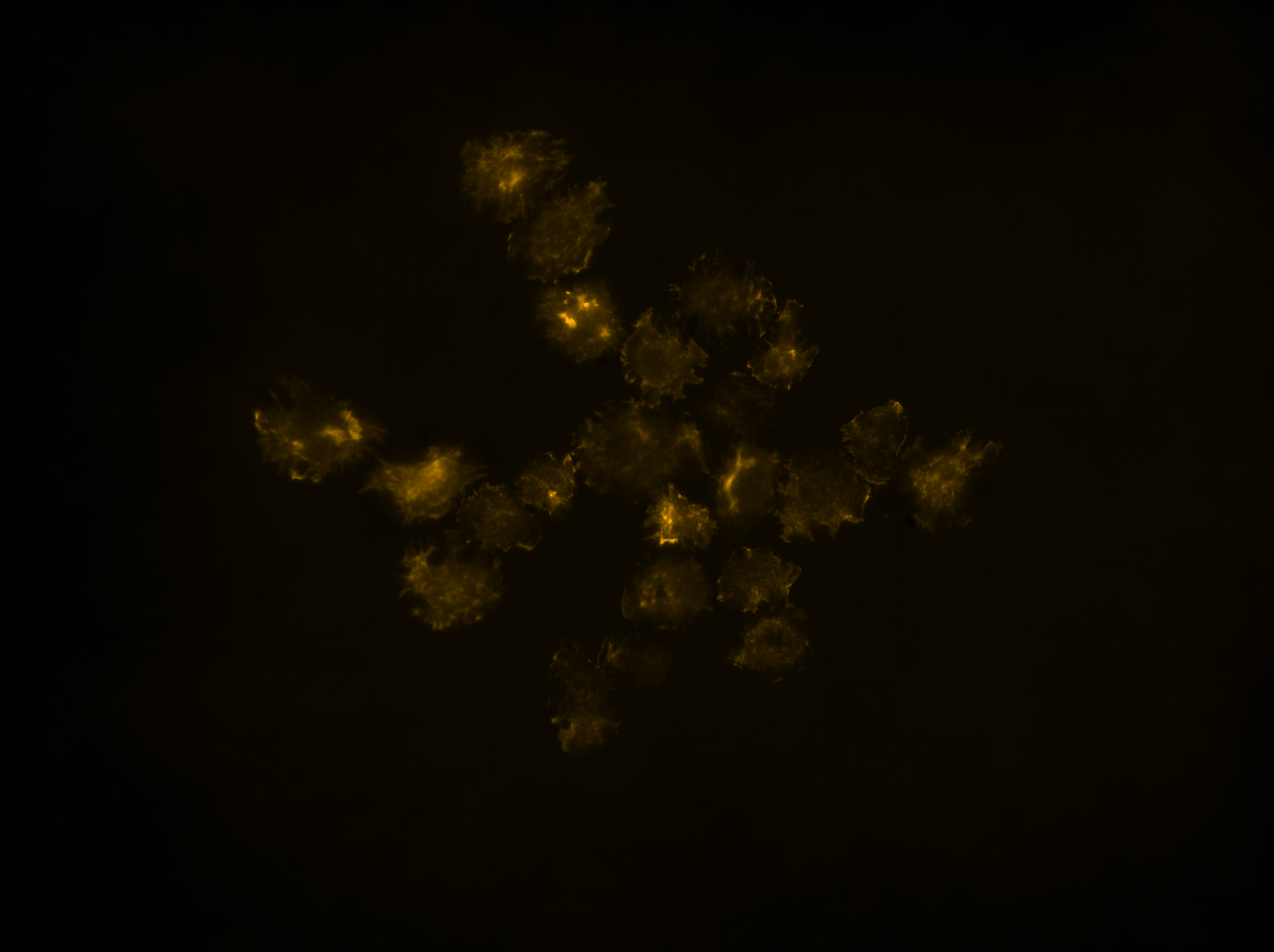

Supplement: Additional file 6 — The zip archive contains simulated images showing B cell nuclei and cytoskeleton with corresponding ground truth. (ZIP 119808 kb) [file 12859_2017_1591_MOESM6_ESM.zip › simulated B cells/cytoskeleton/overlapping/cell024.png]

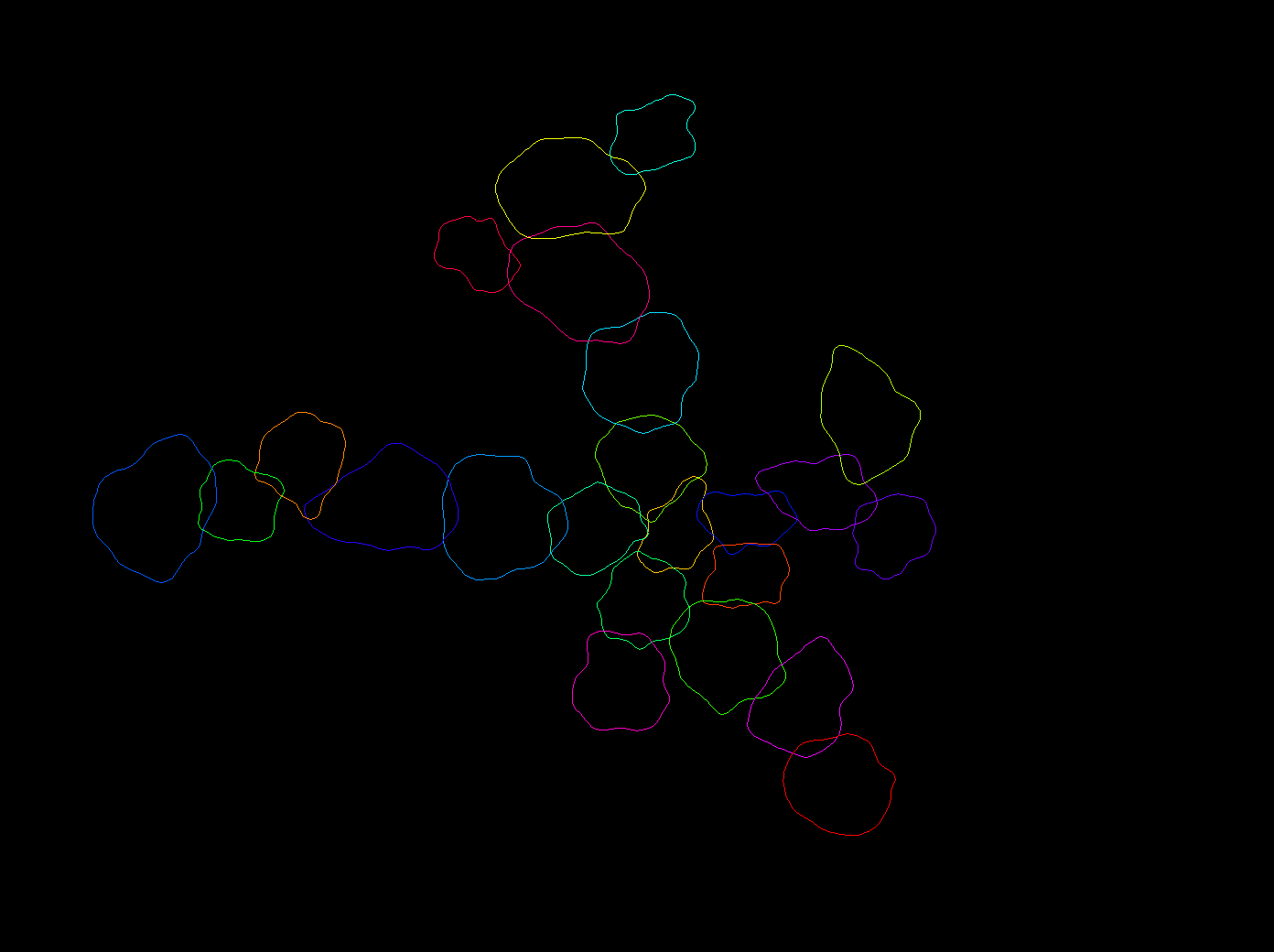

Supplement: Additional file 6 — The zip archive contains simulated images showing B cell nuclei and cytoskeleton with corresponding ground truth. (ZIP 119808 kb) [file 12859_2017_1591_MOESM6_ESM.zip › simulated B cells/cytoskeleton/overlapping/cell025 gt.png]

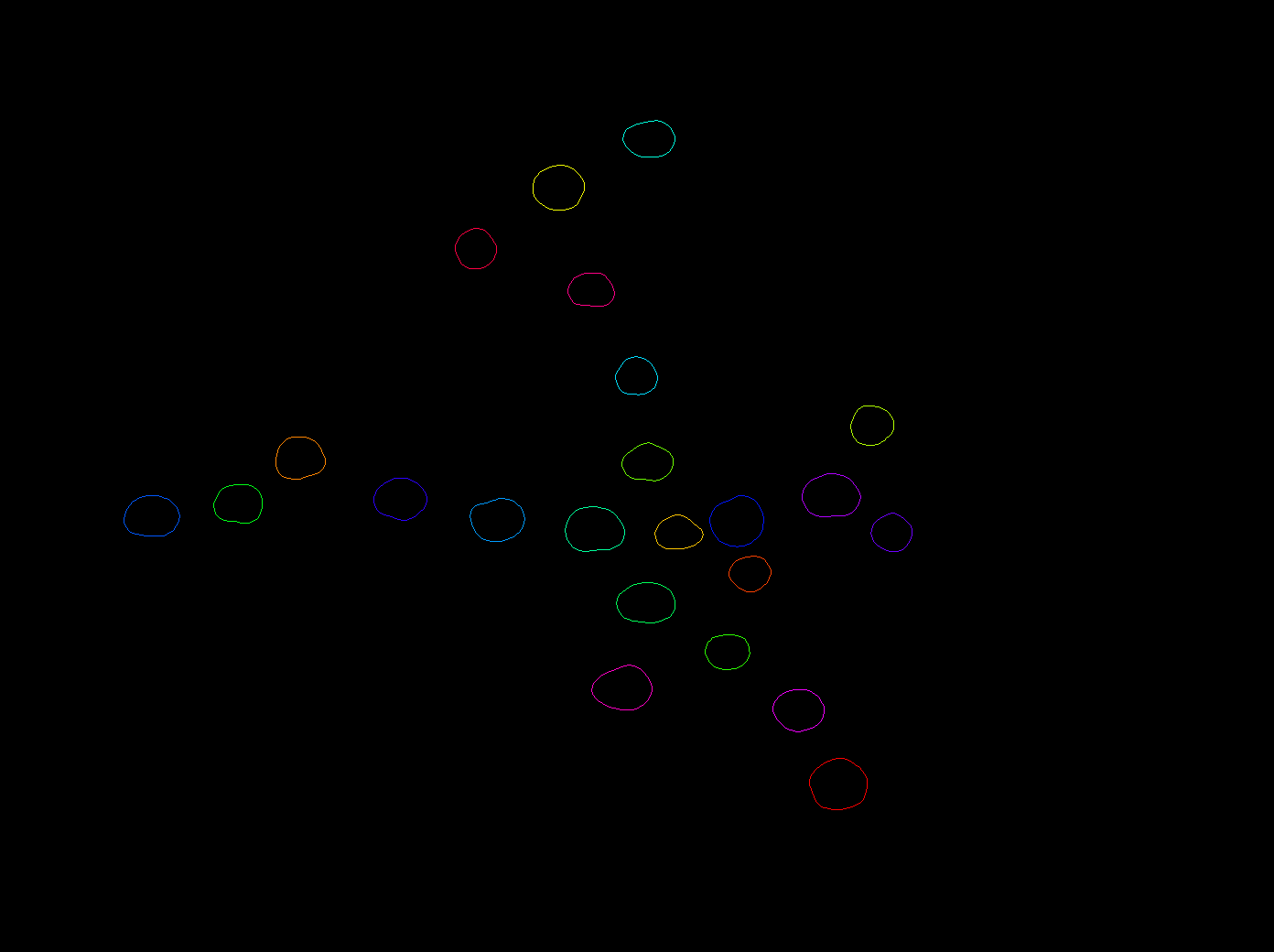

Supplement: Additional file 6 — The zip archive contains simulated images showing B cell nuclei and cytoskeleton with corresponding ground truth. (ZIP 119808 kb) [file 12859_2017_1591_MOESM6_ESM.zip › simulated B cells/cytoskeleton/overlapping/cell025 seeds.png]

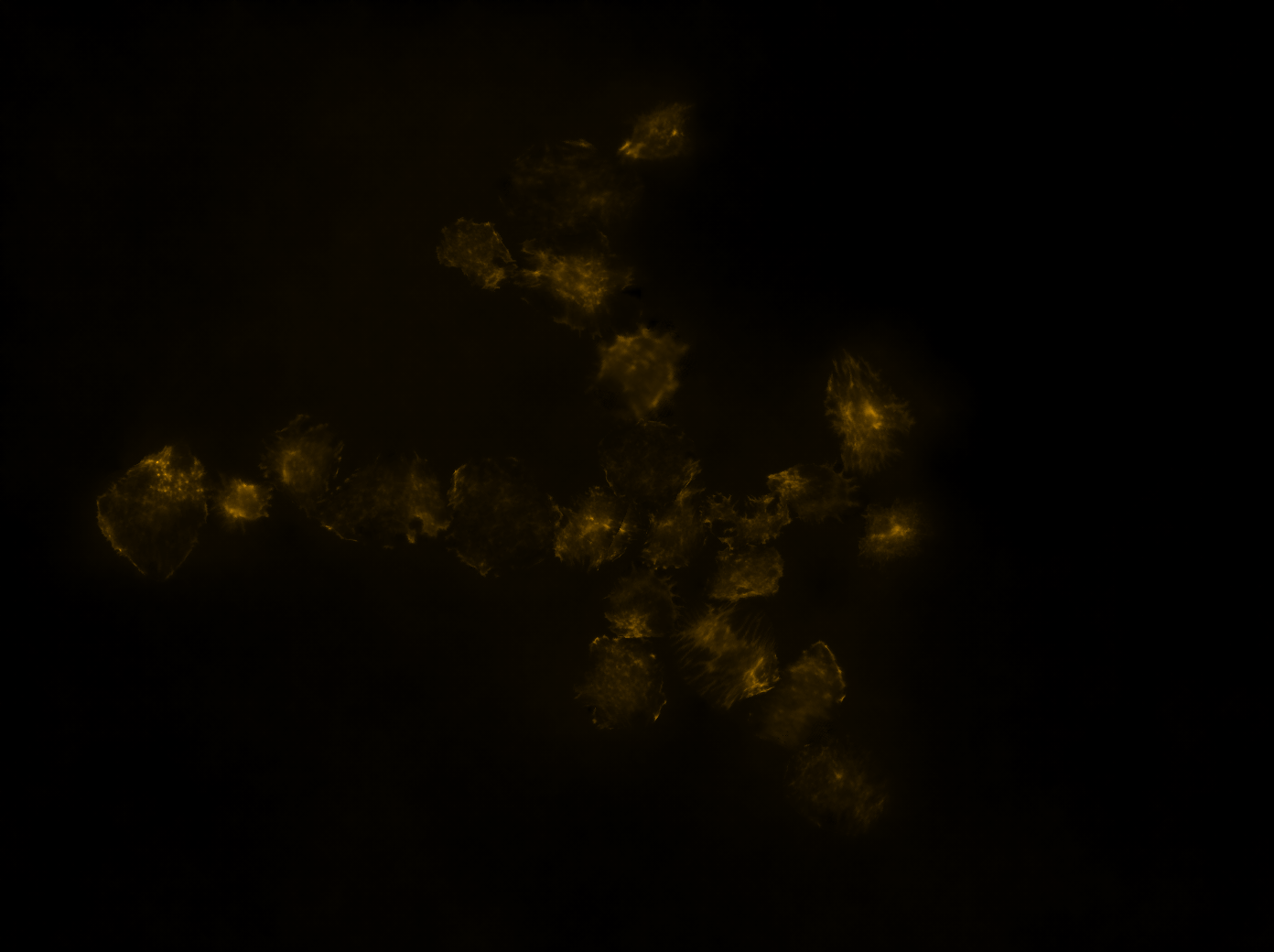

Supplement: Additional file 6 — The zip archive contains simulated images showing B cell nuclei and cytoskeleton with corresponding ground truth. (ZIP 119808 kb) [file 12859_2017_1591_MOESM6_ESM.zip › simulated B cells/cytoskeleton/overlapping/cell025.png]

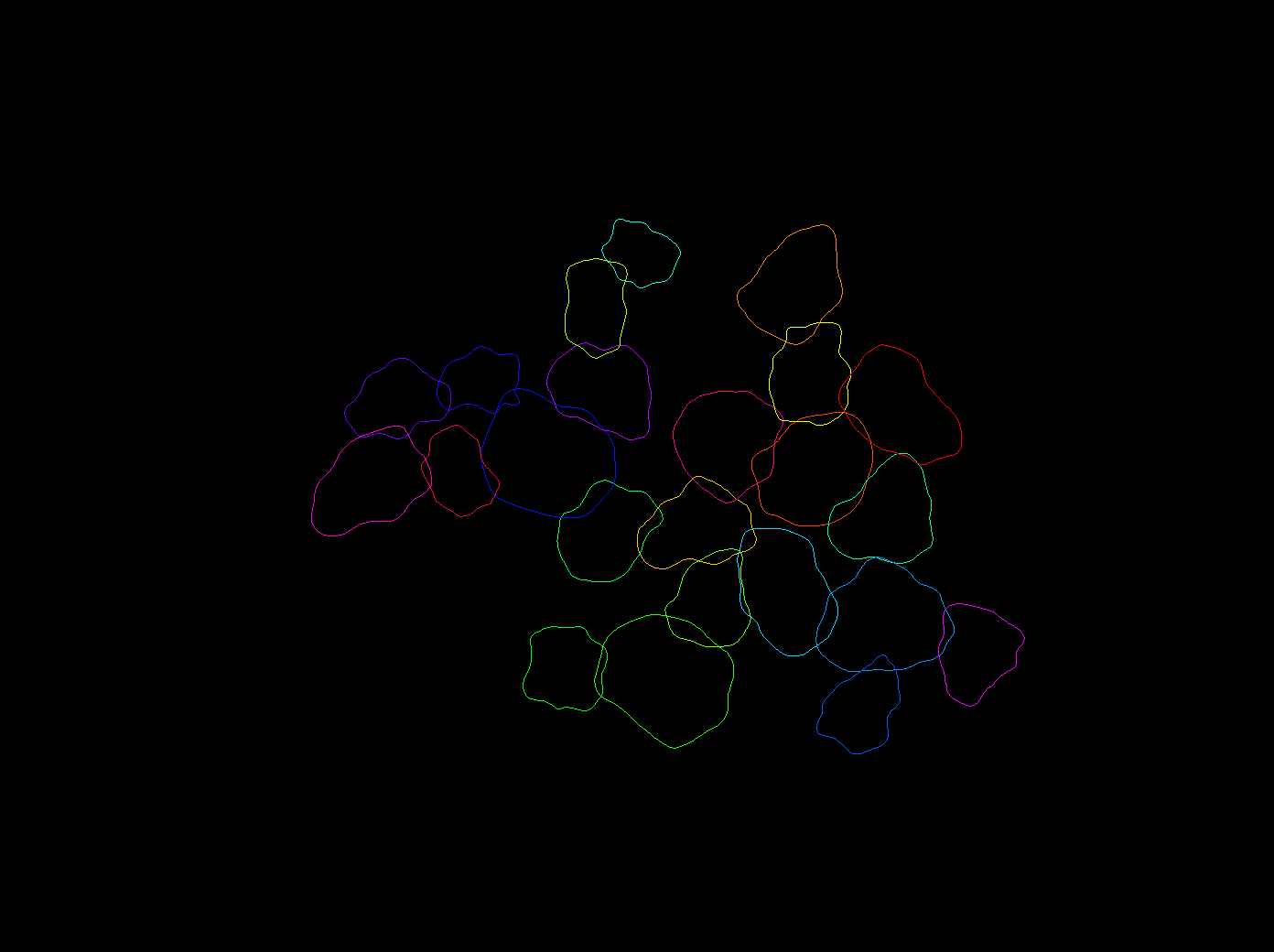

Supplement: Additional file 6 — The zip archive contains simulated images showing B cell nuclei and cytoskeleton with corresponding ground truth. (ZIP 119808 kb) [file 12859_2017_1591_MOESM6_ESM.zip › simulated B cells/cytoskeleton/overlapping/cell026 gt.png]

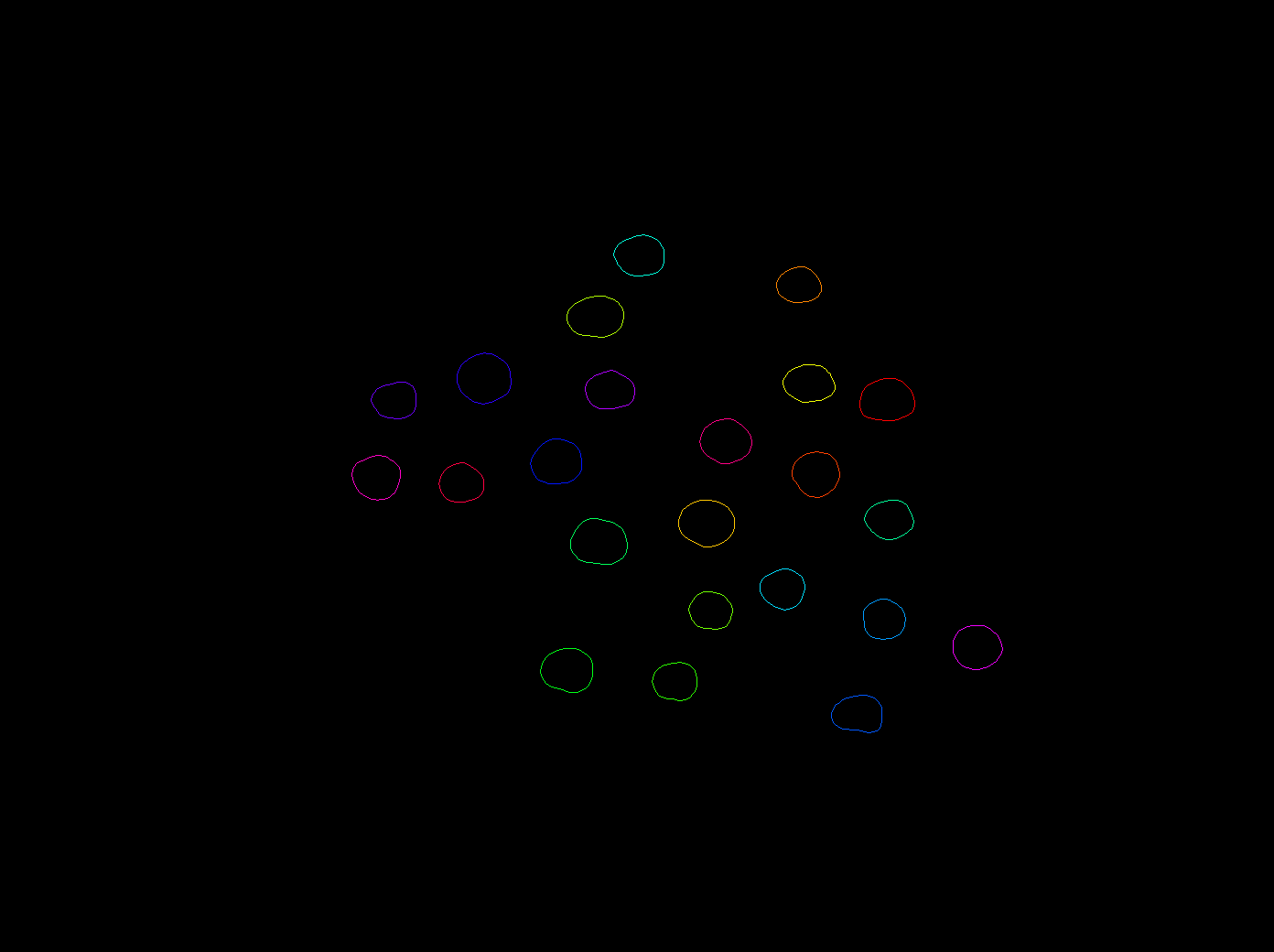

Supplement: Additional file 6 — The zip archive contains simulated images showing B cell nuclei and cytoskeleton with corresponding ground truth. (ZIP 119808 kb) [file 12859_2017_1591_MOESM6_ESM.zip › simulated B cells/cytoskeleton/overlapping/cell026 seeds.png]

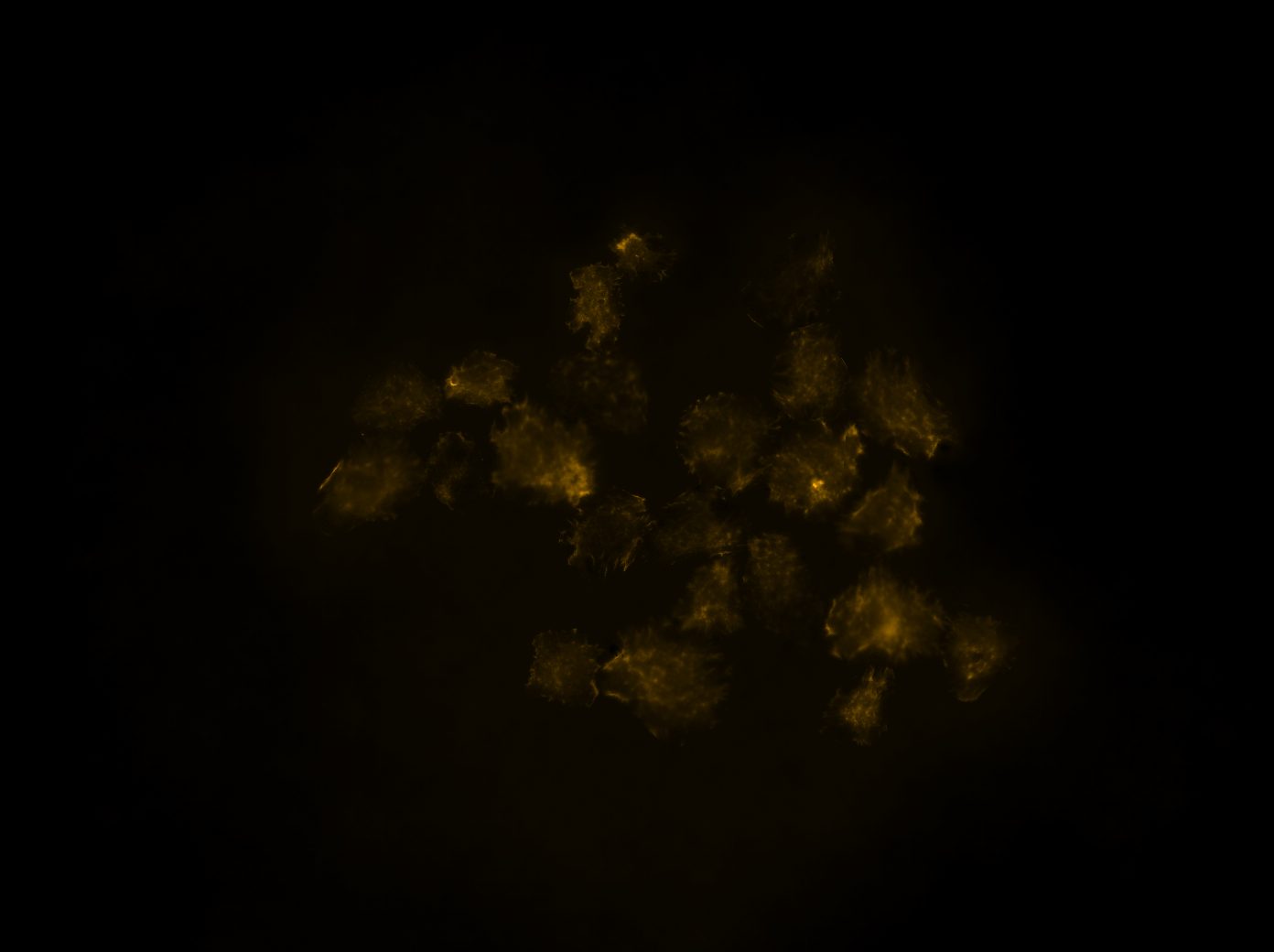

Supplement: Additional file 6 — The zip archive contains simulated images showing B cell nuclei and cytoskeleton with corresponding ground truth. (ZIP 119808 kb) [file 12859_2017_1591_MOESM6_ESM.zip › simulated B cells/cytoskeleton/overlapping/cell026.png]

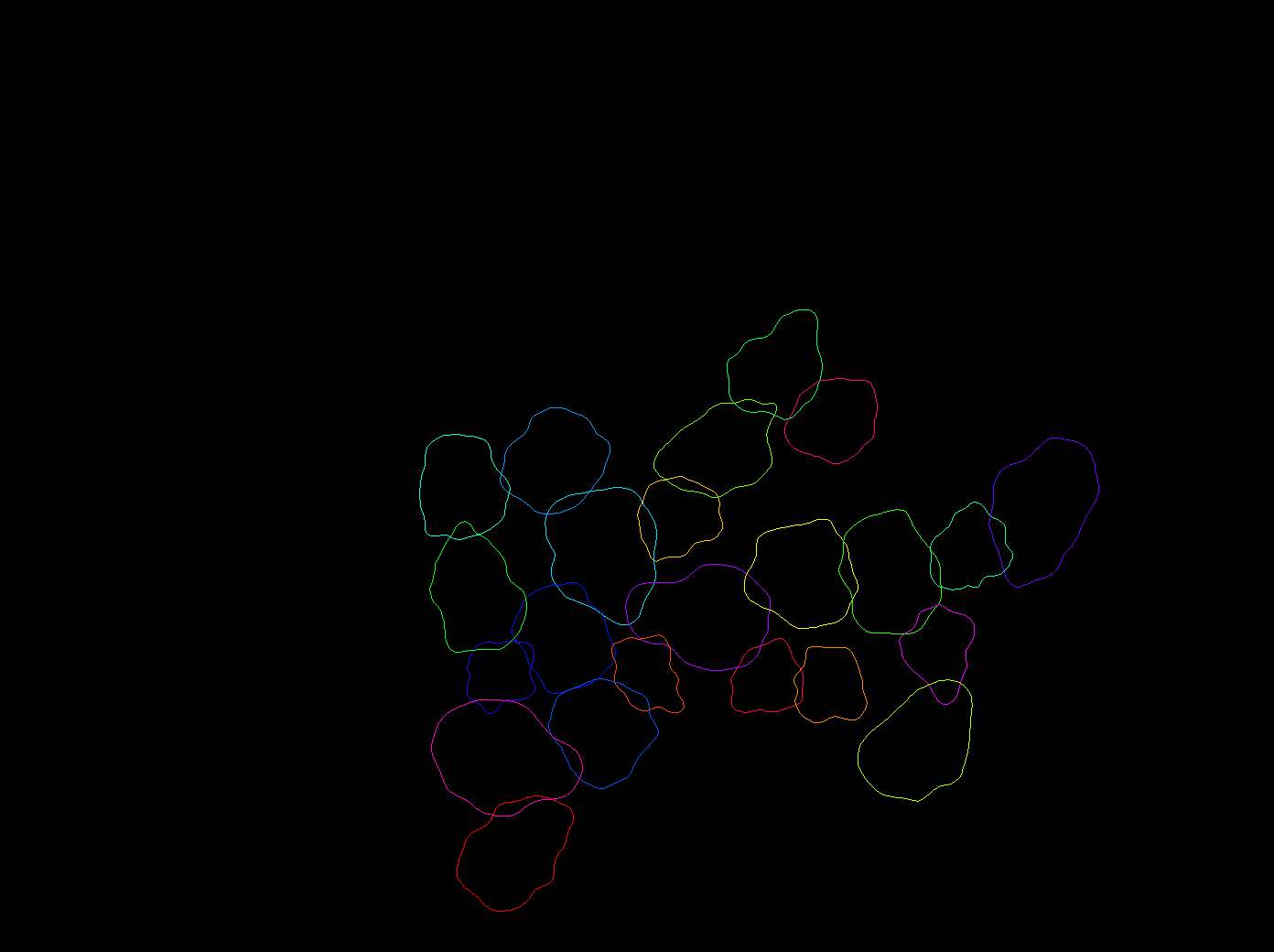

Supplement: Additional file 6 — The zip archive contains simulated images showing B cell nuclei and cytoskeleton with corresponding ground truth. (ZIP 119808 kb) [file 12859_2017_1591_MOESM6_ESM.zip › simulated B cells/cytoskeleton/overlapping/cell027 gt.png]

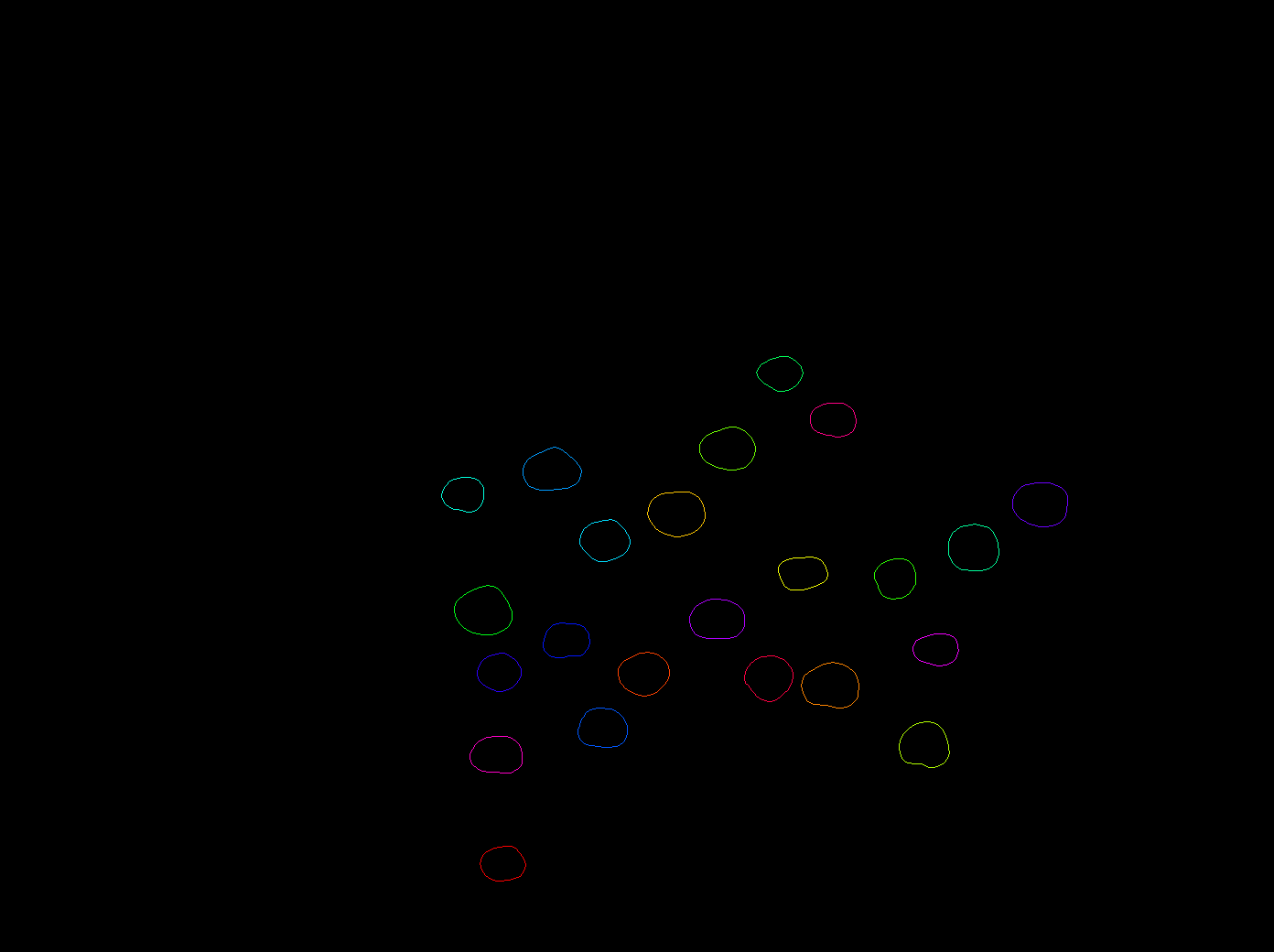

Supplement: Additional file 6 — The zip archive contains simulated images showing B cell nuclei and cytoskeleton with corresponding ground truth. (ZIP 119808 kb) [file 12859_2017_1591_MOESM6_ESM.zip › simulated B cells/cytoskeleton/overlapping/cell027 seeds.png]

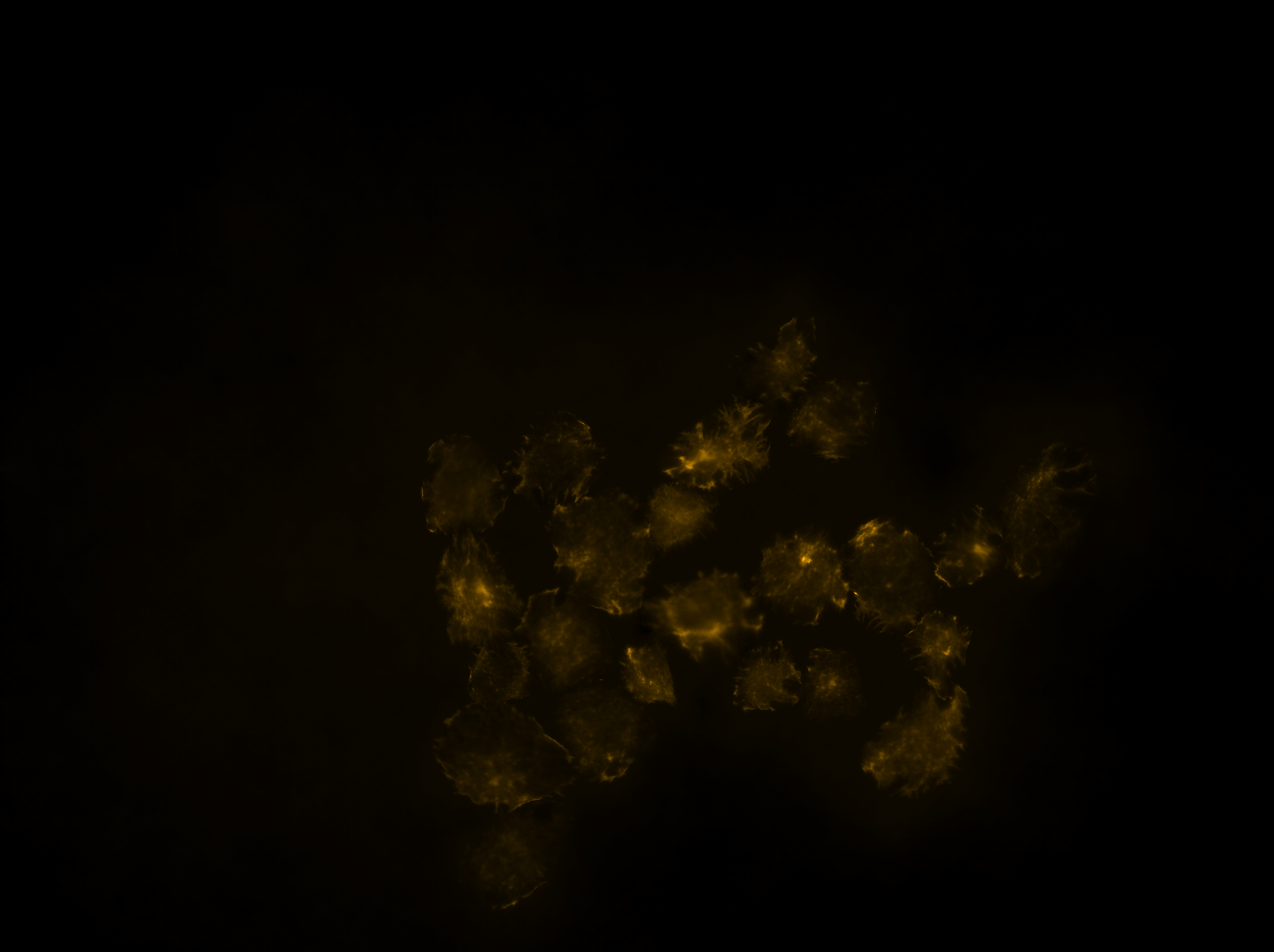

Supplement: Additional file 6 — The zip archive contains simulated images showing B cell nuclei and cytoskeleton with corresponding ground truth. (ZIP 119808 kb) [file 12859_2017_1591_MOESM6_ESM.zip › simulated B cells/cytoskeleton/overlapping/cell027.png]

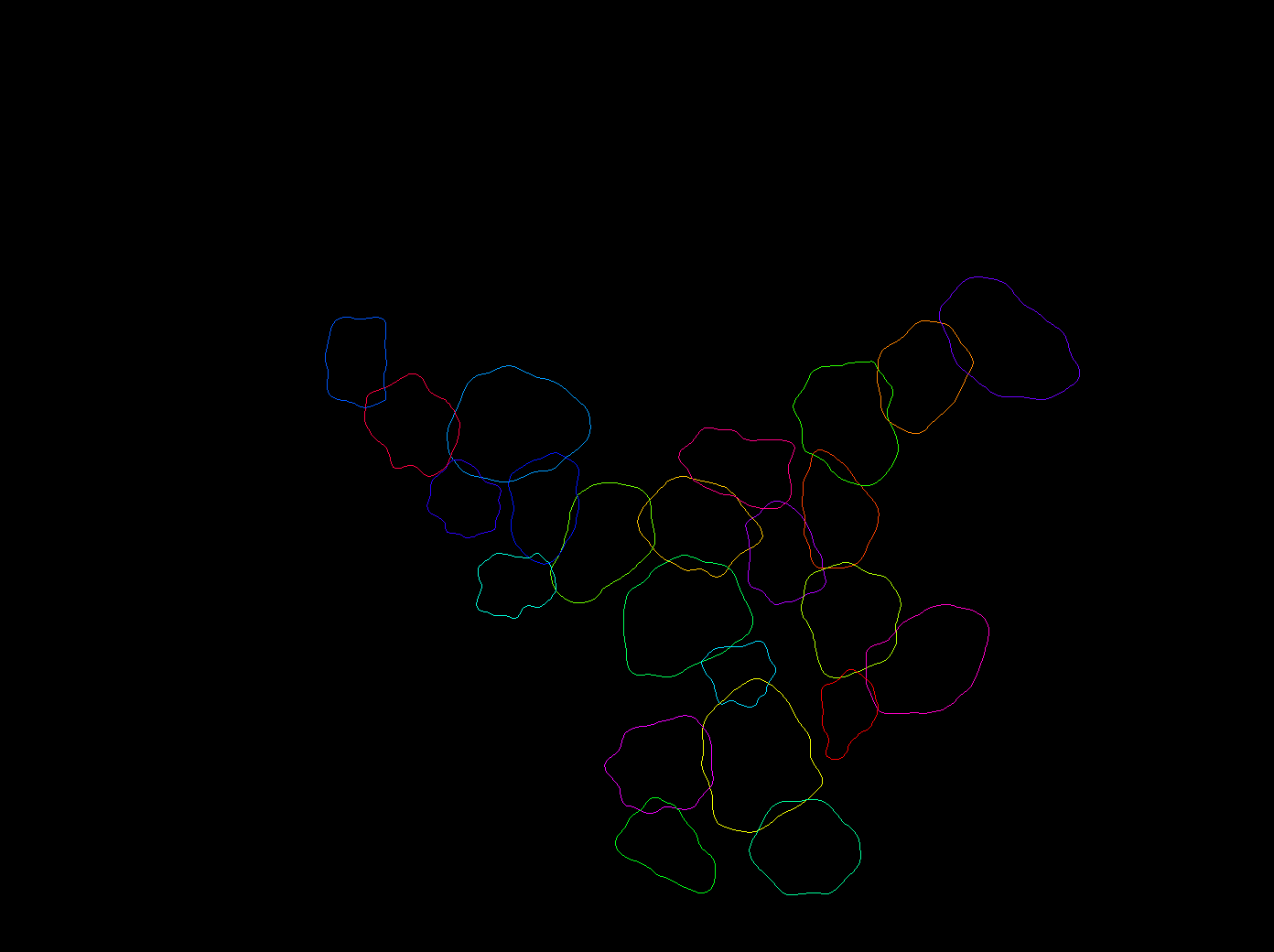

Supplement: Additional file 6 — The zip archive contains simulated images showing B cell nuclei and cytoskeleton with corresponding ground truth. (ZIP 119808 kb) [file 12859_2017_1591_MOESM6_ESM.zip › simulated B cells/cytoskeleton/overlapping/cell028 gt.png]

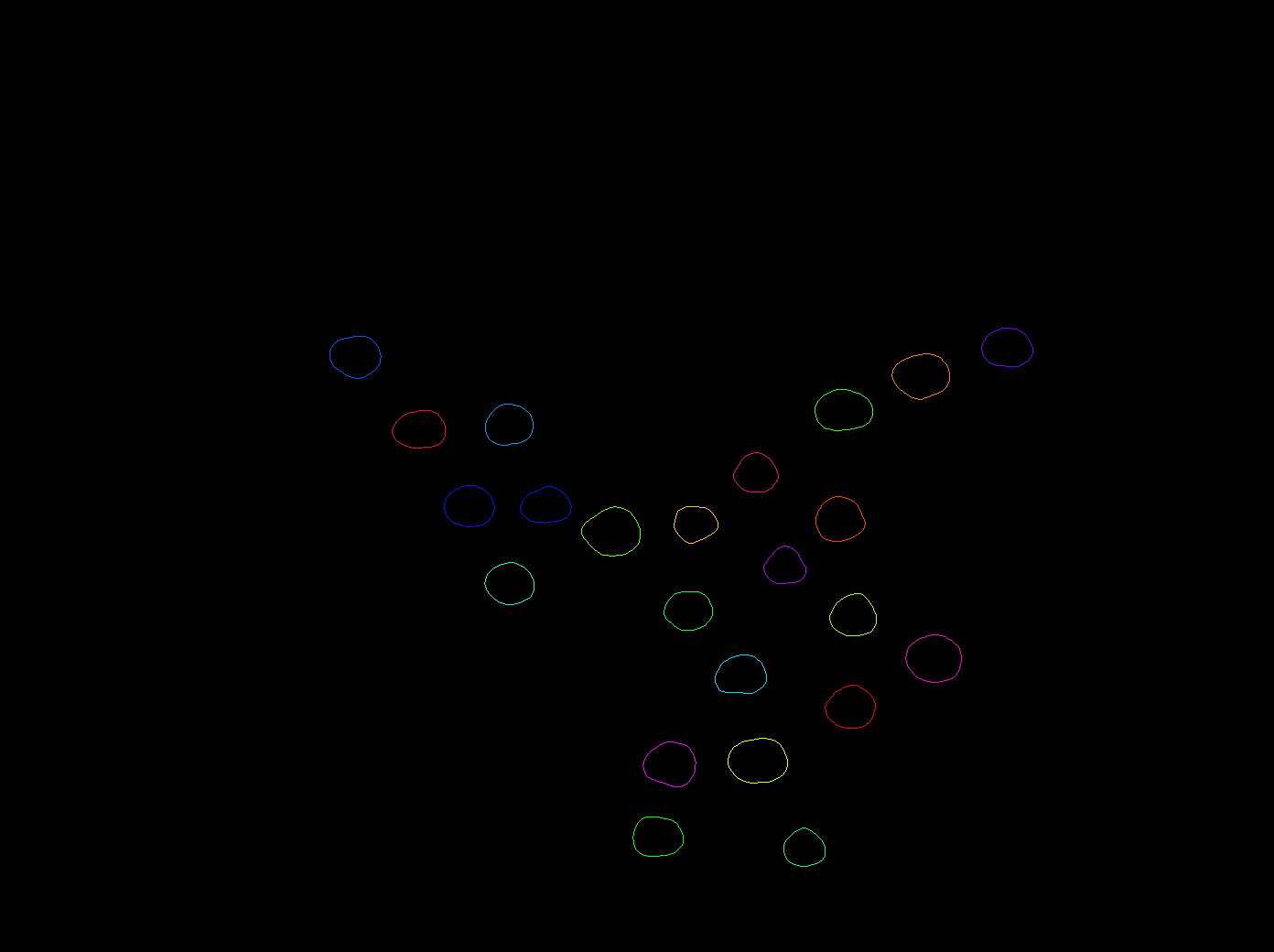

Supplement: Additional file 6 — The zip archive contains simulated images showing B cell nuclei and cytoskeleton with corresponding ground truth. (ZIP 119808 kb) [file 12859_2017_1591_MOESM6_ESM.zip › simulated B cells/cytoskeleton/overlapping/cell028 seeds.png]

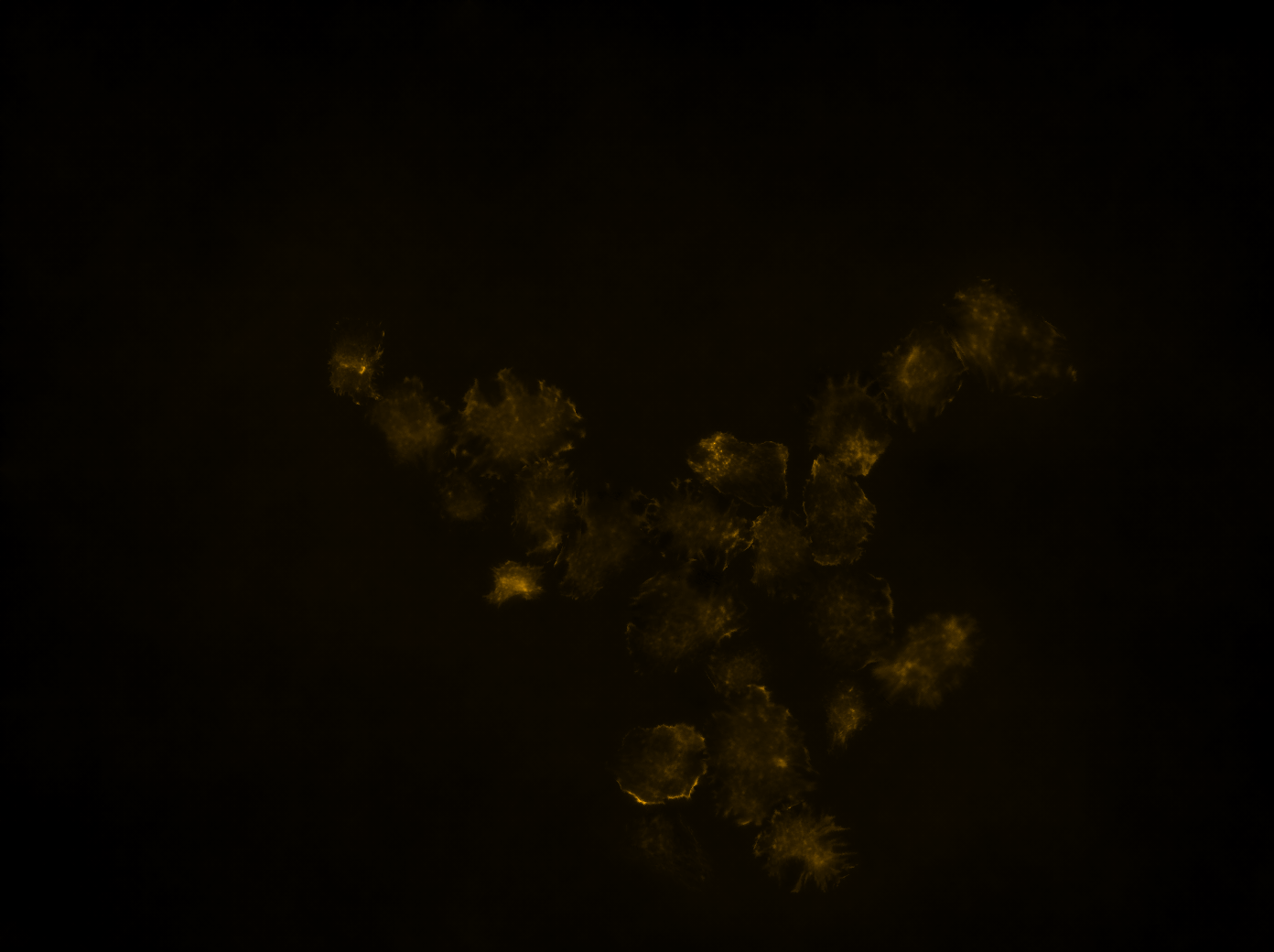

Supplement: Additional file 6 — The zip archive contains simulated images showing B cell nuclei and cytoskeleton with corresponding ground truth. (ZIP 119808 kb) [file 12859_2017_1591_MOESM6_ESM.zip › simulated B cells/cytoskeleton/overlapping/cell028.png]

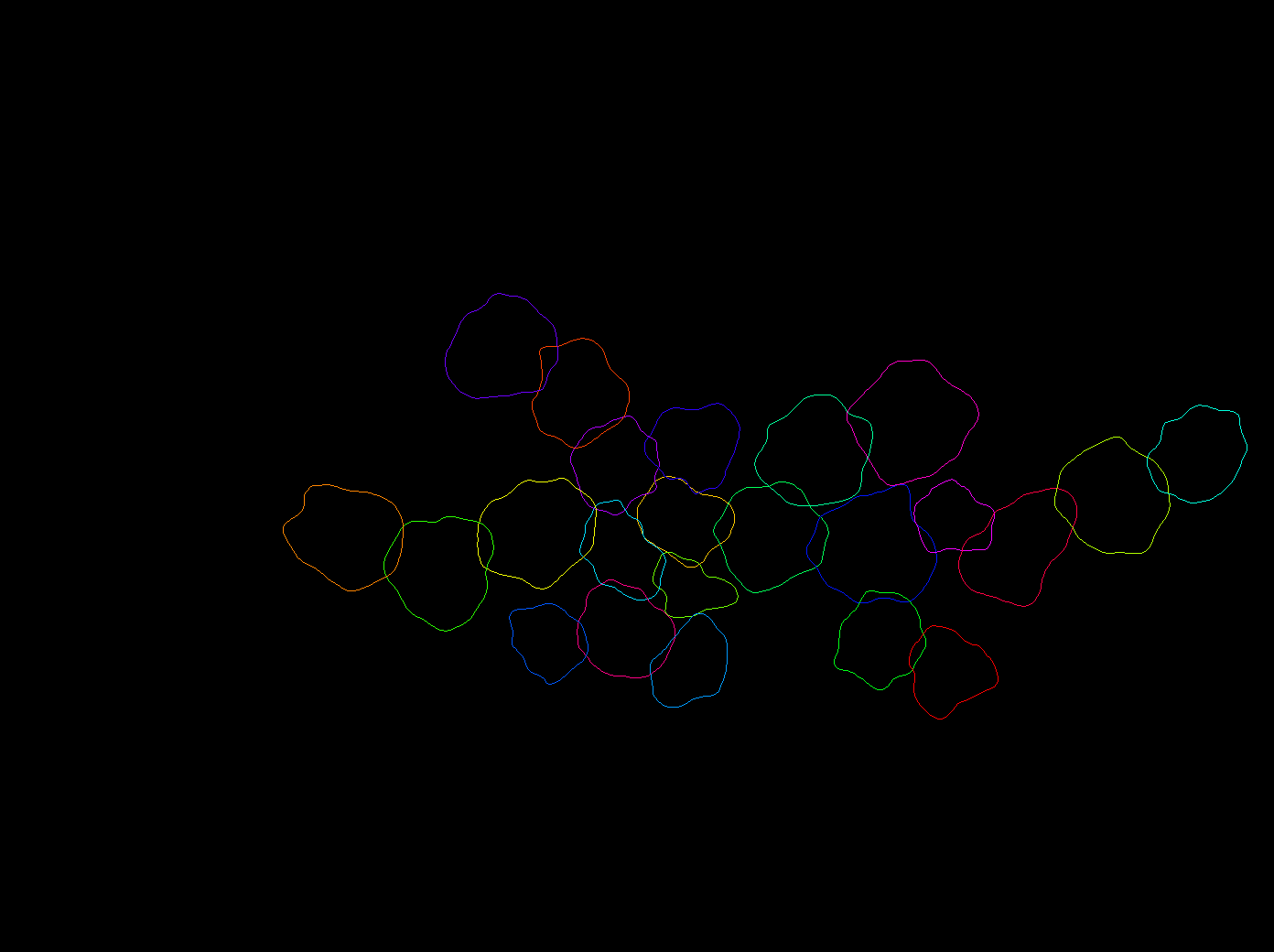

Supplement: Additional file 6 — The zip archive contains simulated images showing B cell nuclei and cytoskeleton with corresponding ground truth. (ZIP 119808 kb) [file 12859_2017_1591_MOESM6_ESM.zip › simulated B cells/cytoskeleton/overlapping/cell029 gt.png]

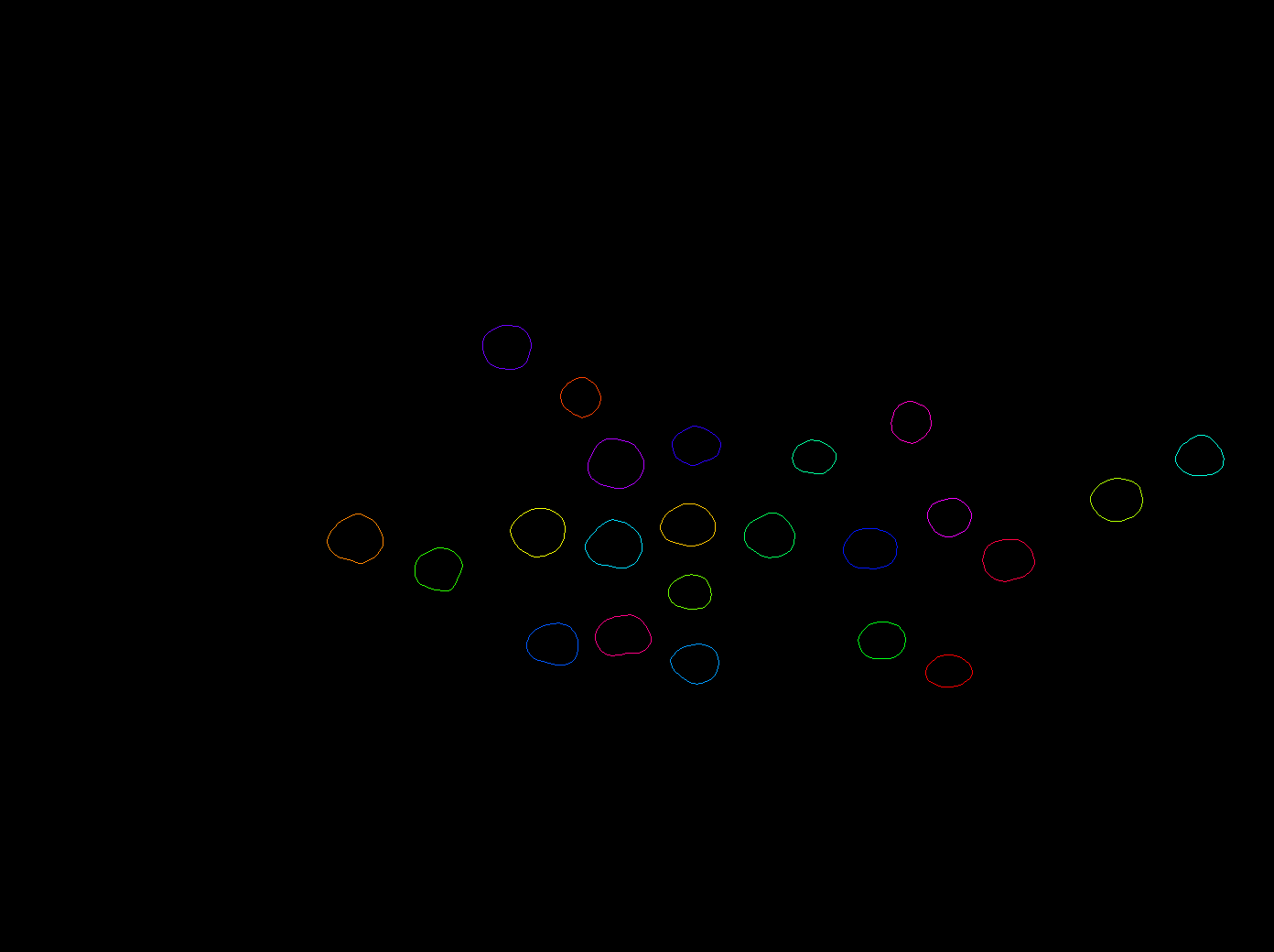

Supplement: Additional file 6 — The zip archive contains simulated images showing B cell nuclei and cytoskeleton with corresponding ground truth. (ZIP 119808 kb) [file 12859_2017_1591_MOESM6_ESM.zip › simulated B cells/cytoskeleton/overlapping/cell029 seeds.png]

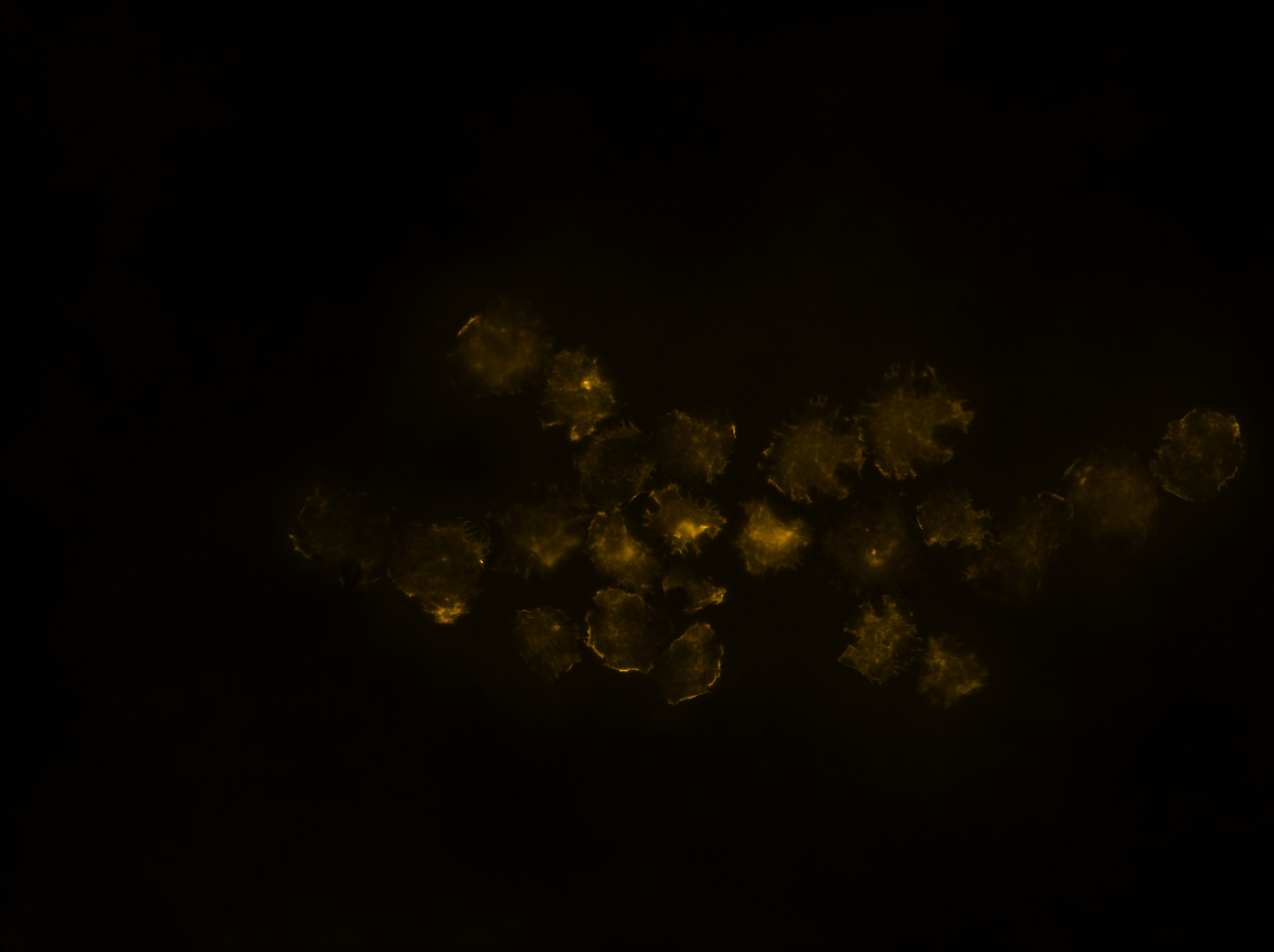

Supplement: Additional file 6 — The zip archive contains simulated images showing B cell nuclei and cytoskeleton with corresponding ground truth. (ZIP 119808 kb) [file 12859_2017_1591_MOESM6_ESM.zip › simulated B cells/cytoskeleton/overlapping/cell029.png]

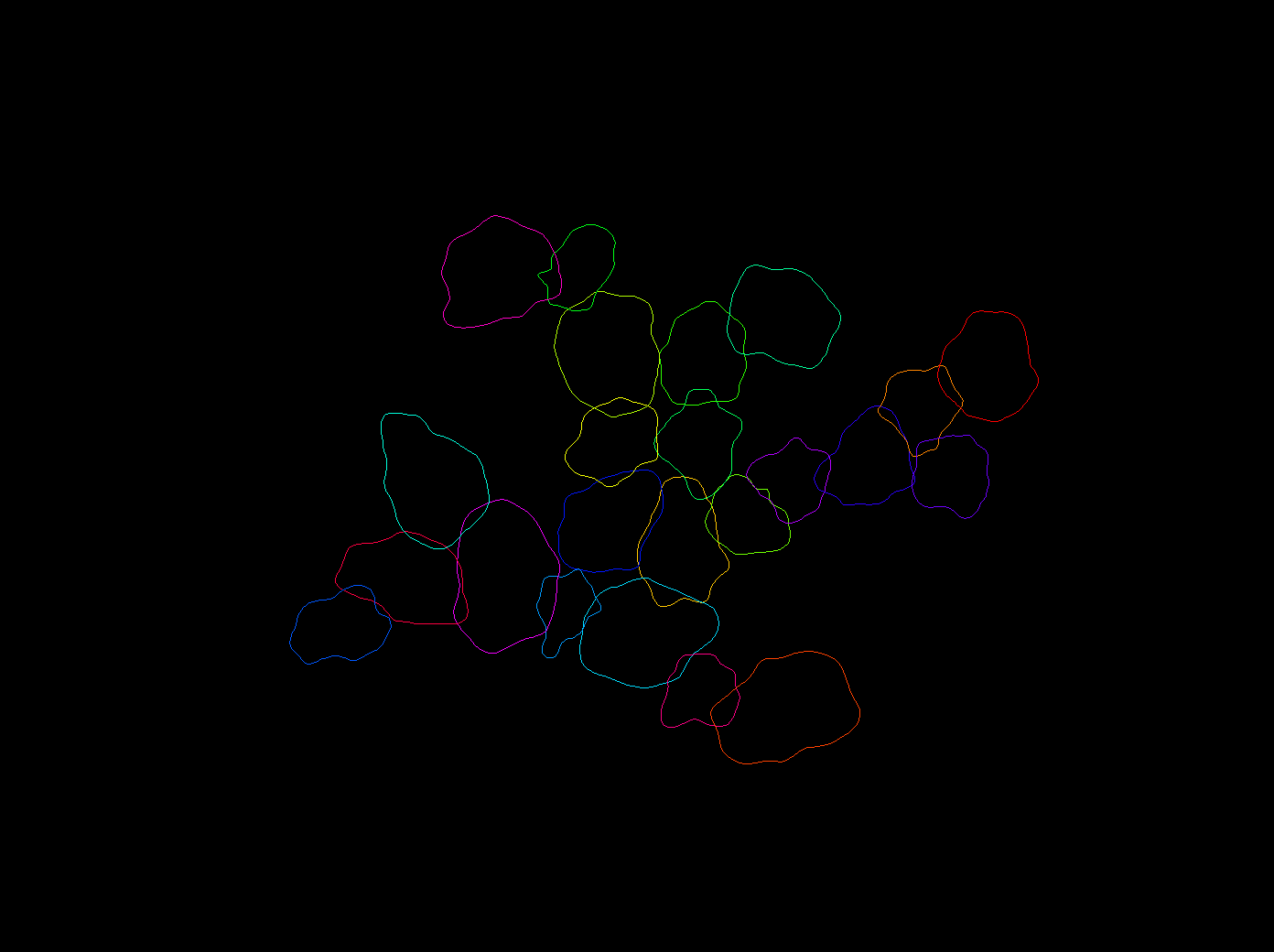

Supplement: Additional file 6 — The zip archive contains simulated images showing B cell nuclei and cytoskeleton with corresponding ground truth. (ZIP 119808 kb) [file 12859_2017_1591_MOESM6_ESM.zip › simulated B cells/cytoskeleton/overlapping/cell030 gt.png]

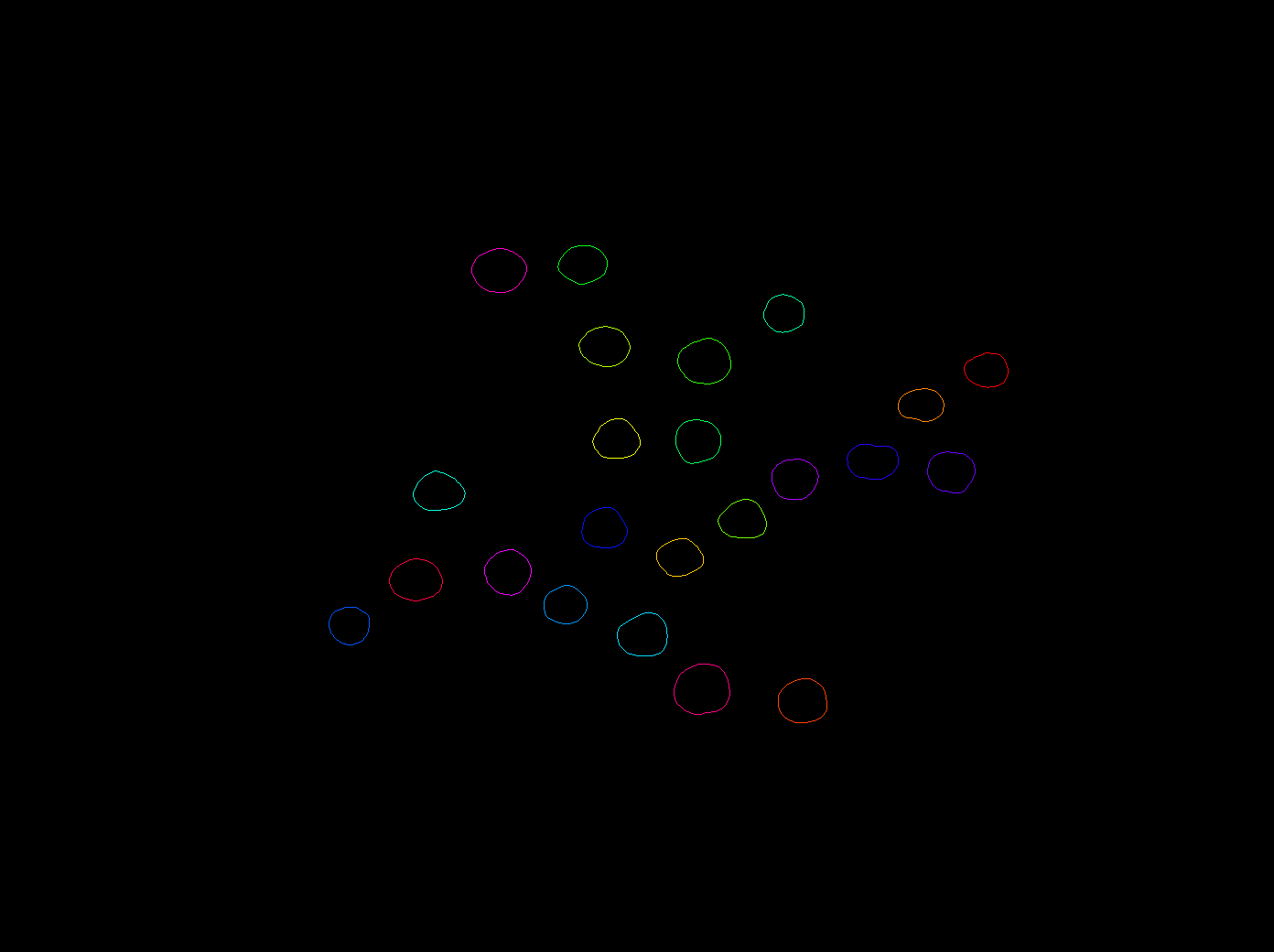

Supplement: Additional file 6 — The zip archive contains simulated images showing B cell nuclei and cytoskeleton with corresponding ground truth. (ZIP 119808 kb) [file 12859_2017_1591_MOESM6_ESM.zip › simulated B cells/cytoskeleton/overlapping/cell030 seeds.png]

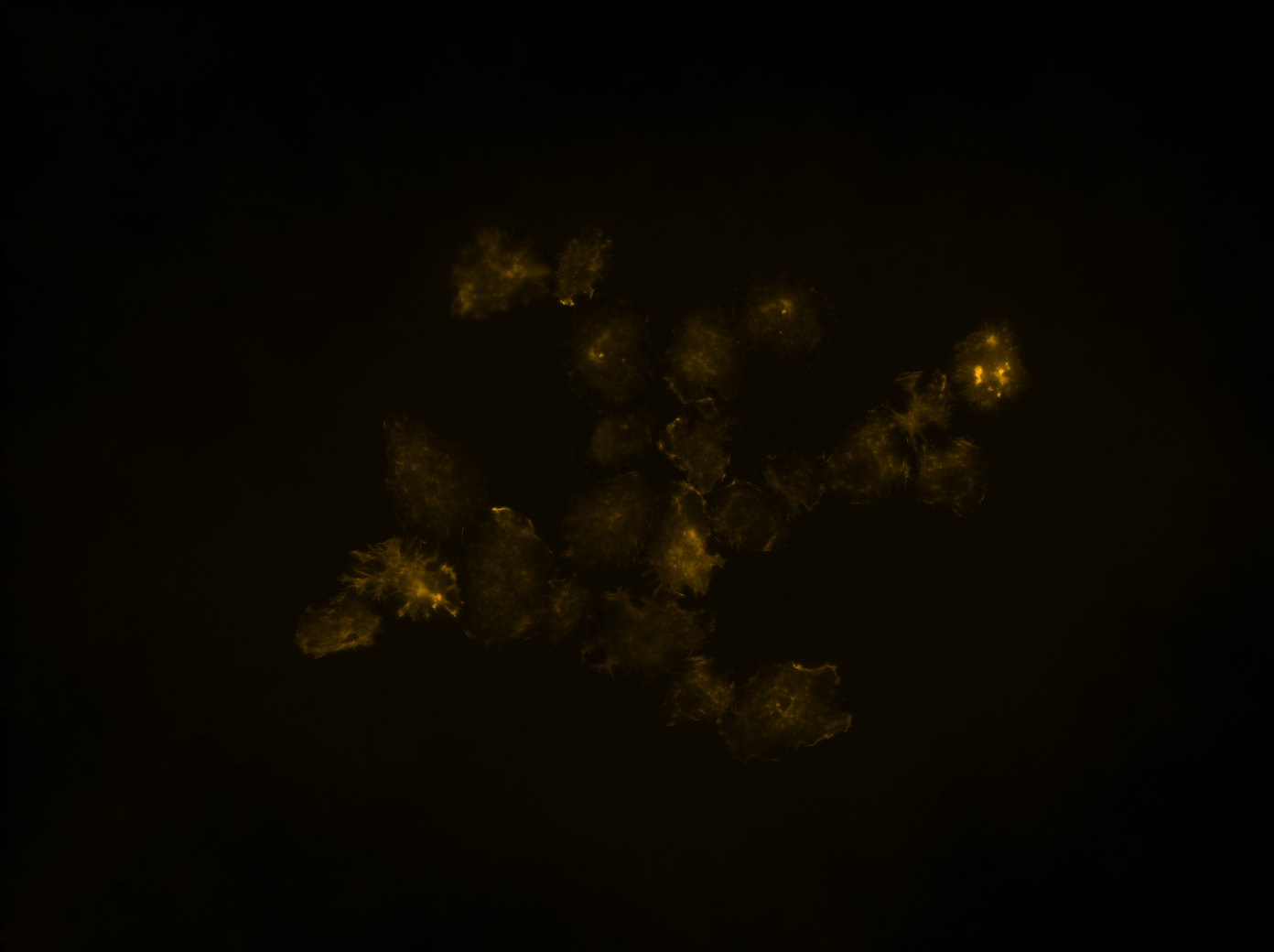

Supplement: Additional file 6 — The zip archive contains simulated images showing B cell nuclei and cytoskeleton with corresponding ground truth. (ZIP 119808 kb) [file 12859_2017_1591_MOESM6_ESM.zip › simulated B cells/cytoskeleton/overlapping/cell030.png]

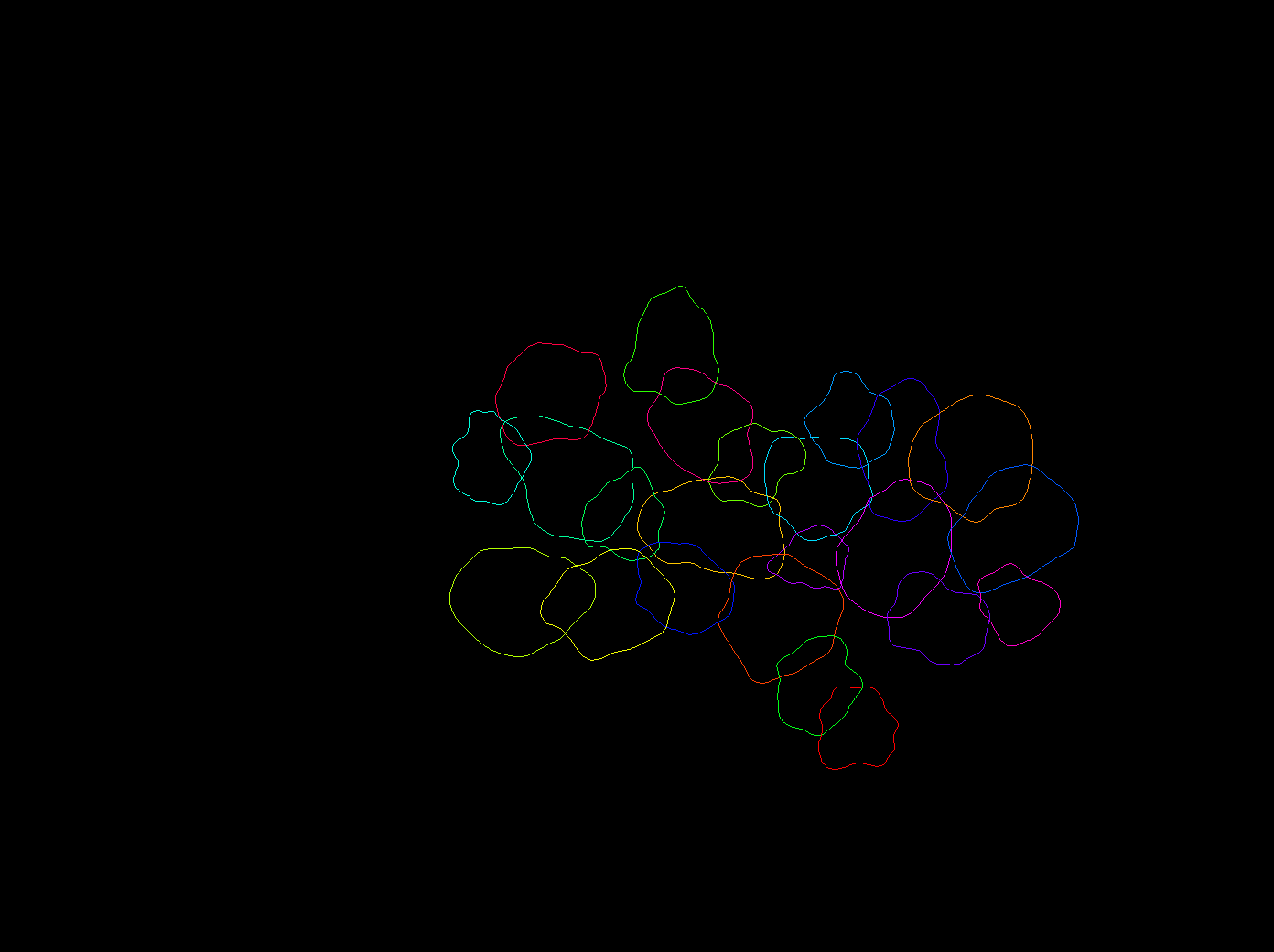

Supplement: Additional file 6 — The zip archive contains simulated images showing B cell nuclei and cytoskeleton with corresponding ground truth. (ZIP 119808 kb) [file 12859_2017_1591_MOESM6_ESM.zip › simulated B cells/cytoskeleton/overlaying/cell001 gt.png]

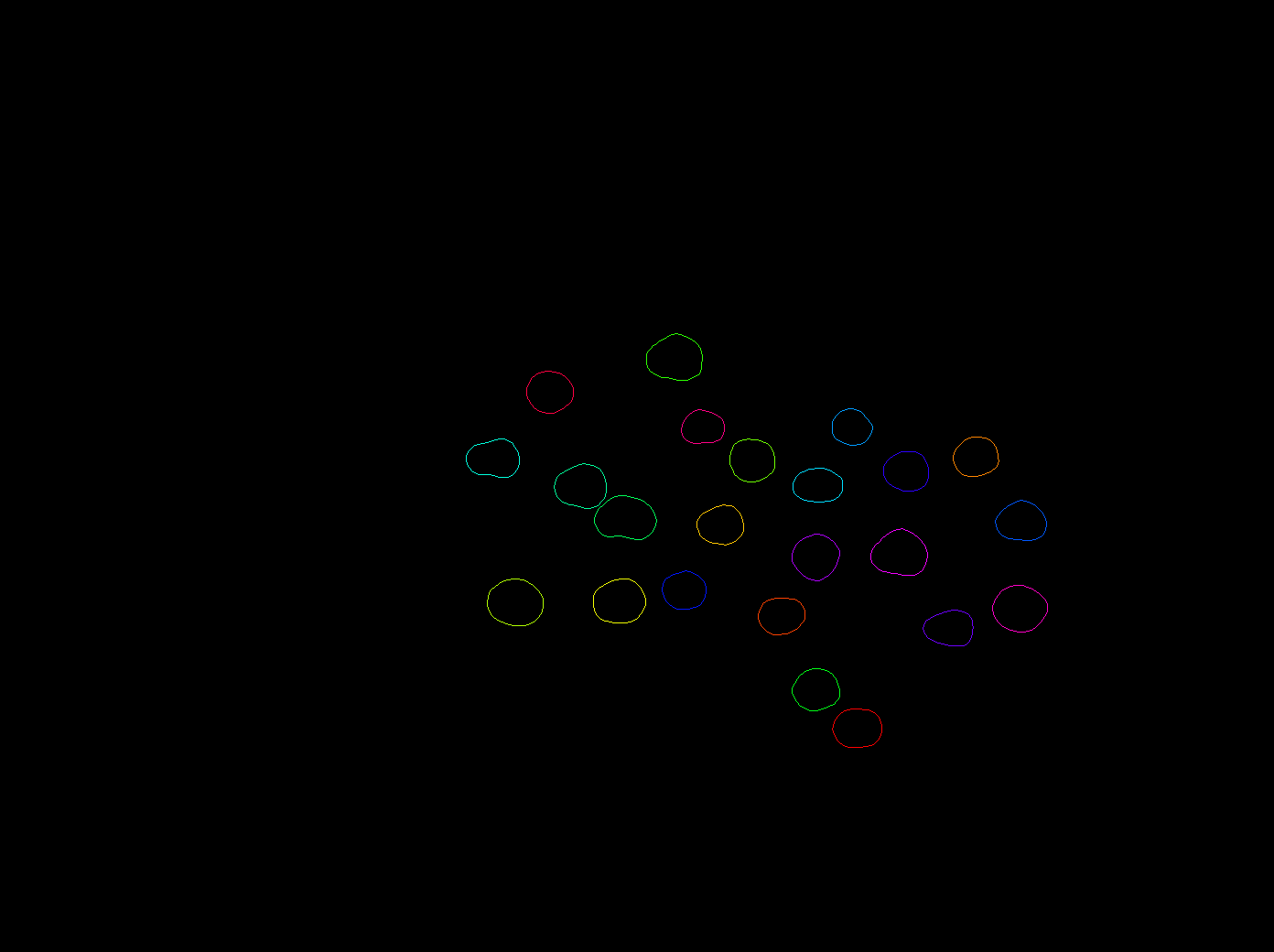

Supplement: Additional file 6 — The zip archive contains simulated images showing B cell nuclei and cytoskeleton with corresponding ground truth. (ZIP 119808 kb) [file 12859_2017_1591_MOESM6_ESM.zip › simulated B cells/cytoskeleton/overlaying/cell001 seeds.png]

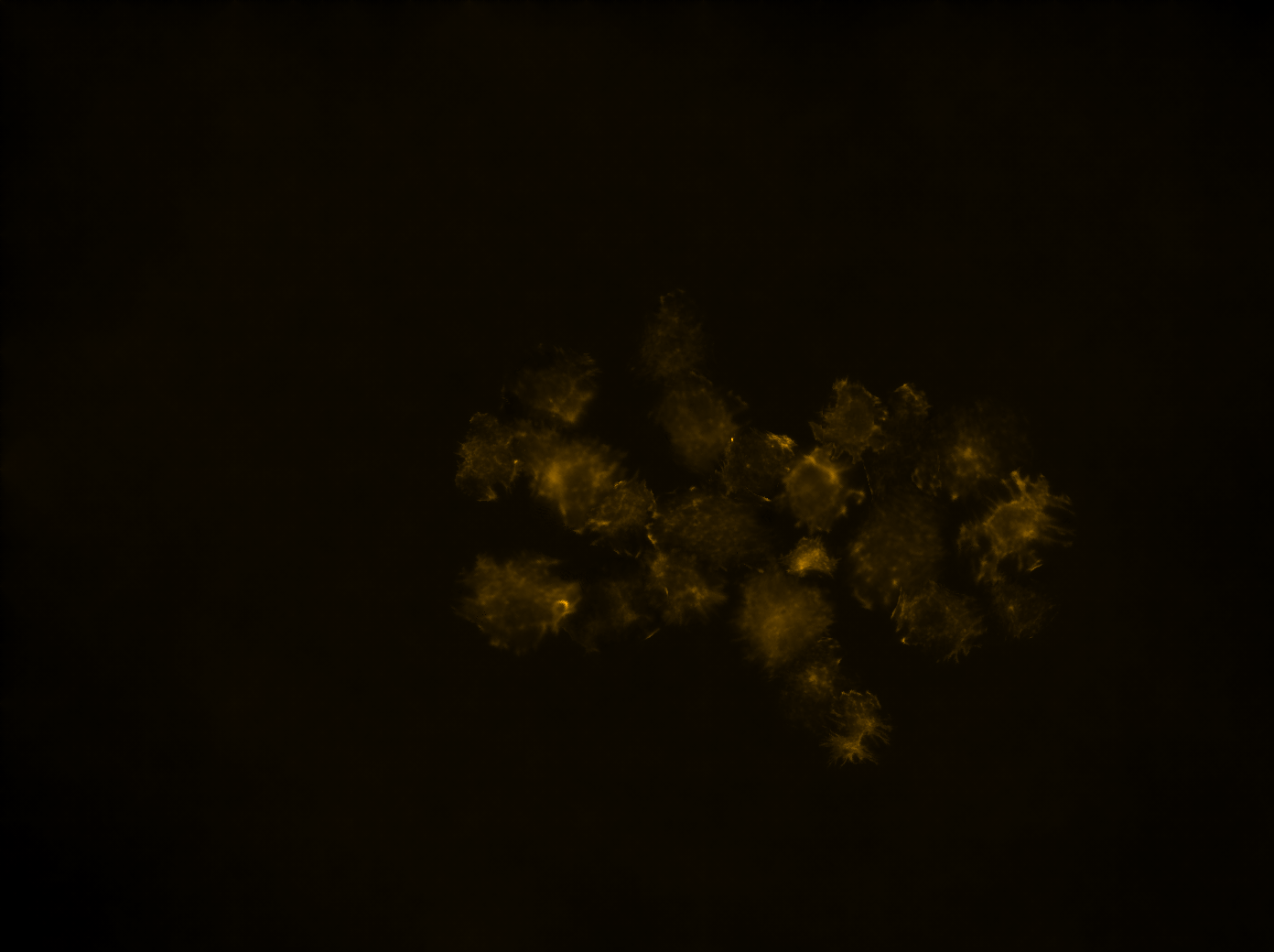

Supplement: Additional file 6 — The zip archive contains simulated images showing B cell nuclei and cytoskeleton with corresponding ground truth. (ZIP 119808 kb) [file 12859_2017_1591_MOESM6_ESM.zip › simulated B cells/cytoskeleton/overlaying/cell001.png]

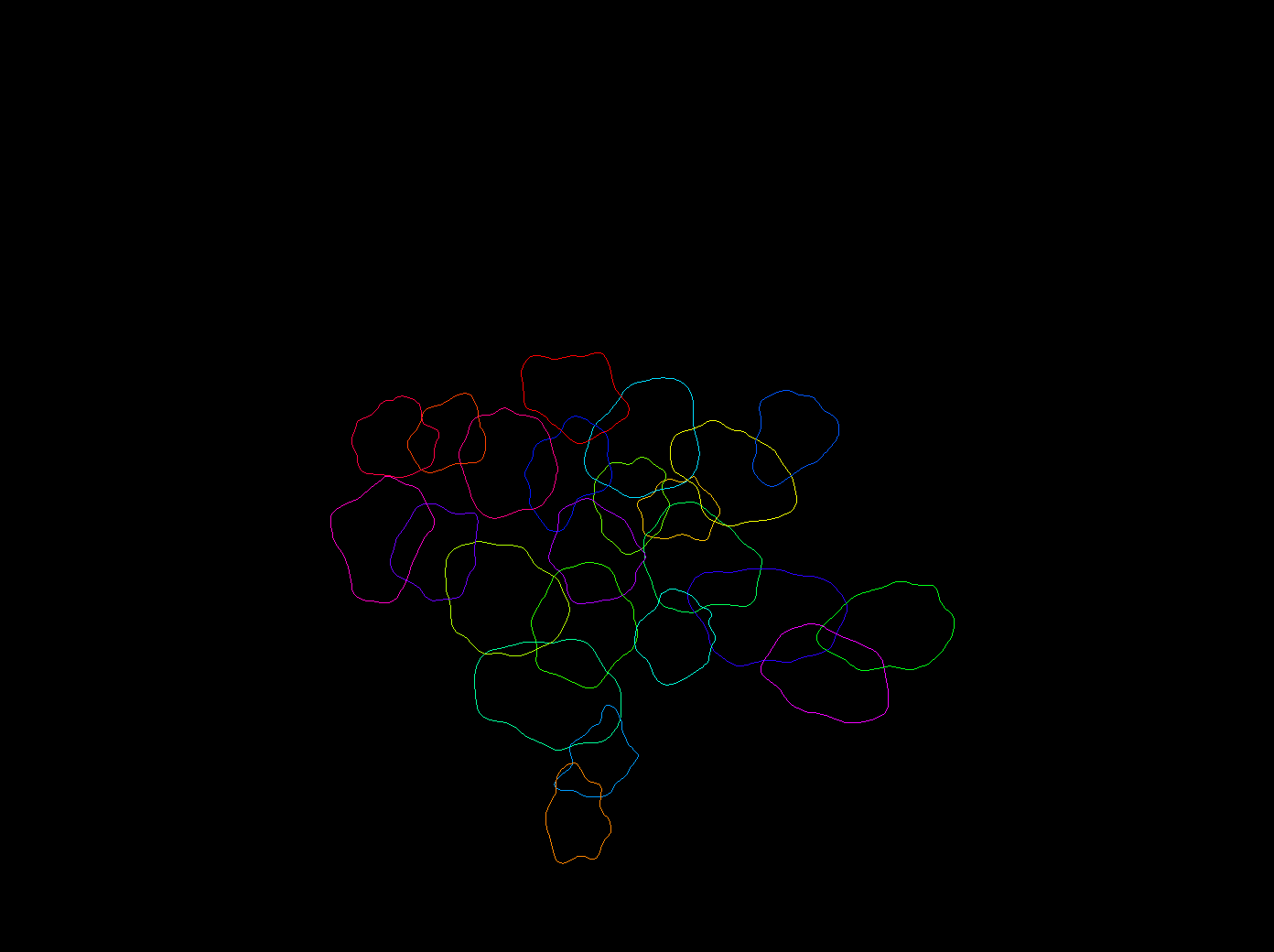

Supplement: Additional file 6 — The zip archive contains simulated images showing B cell nuclei and cytoskeleton with corresponding ground truth. (ZIP 119808 kb) [file 12859_2017_1591_MOESM6_ESM.zip › simulated B cells/cytoskeleton/overlaying/cell002 gt.png]

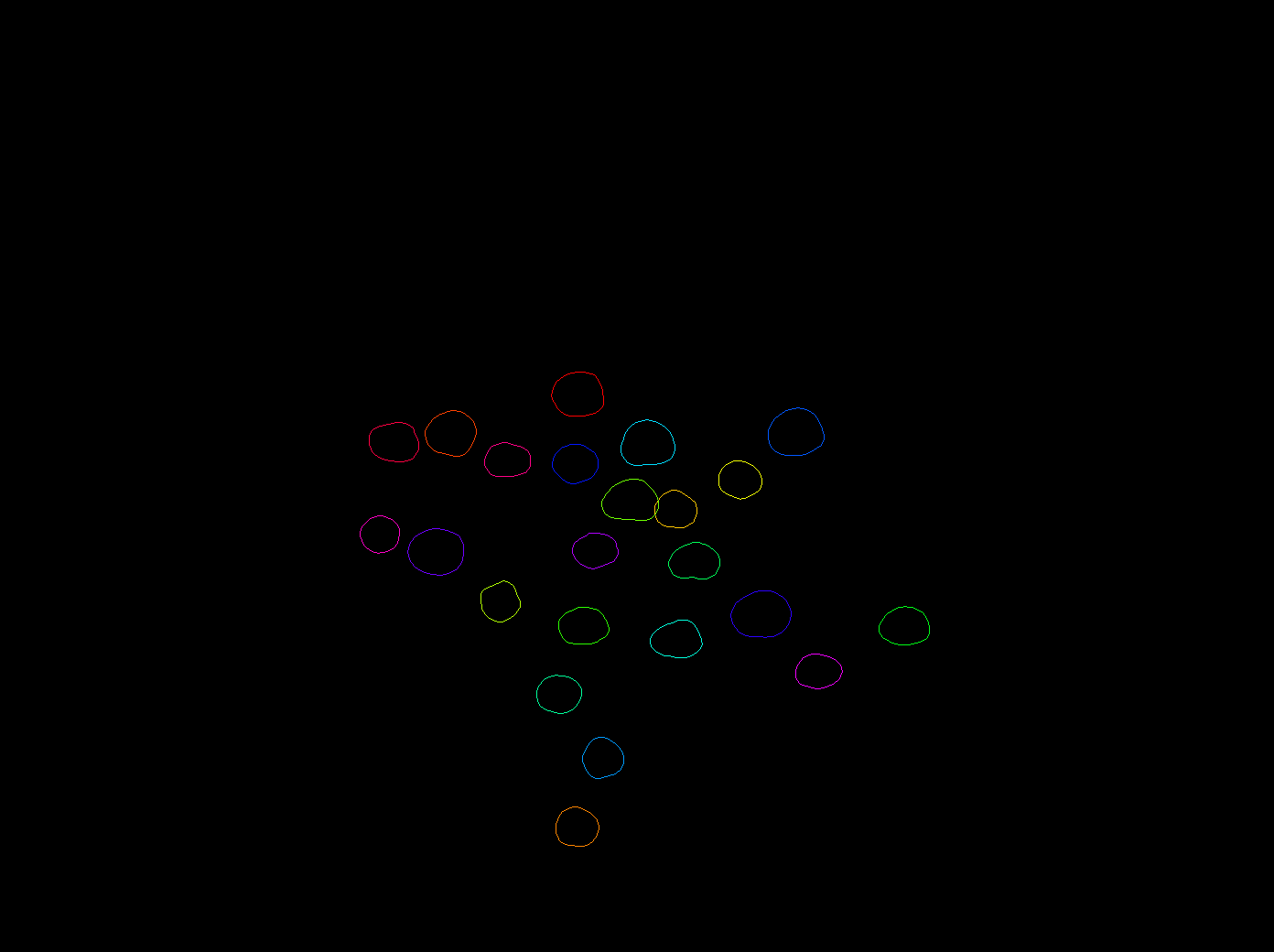

Supplement: Additional file 6 — The zip archive contains simulated images showing B cell nuclei and cytoskeleton with corresponding ground truth. (ZIP 119808 kb) [file 12859_2017_1591_MOESM6_ESM.zip › simulated B cells/cytoskeleton/overlaying/cell002 seeds.png]

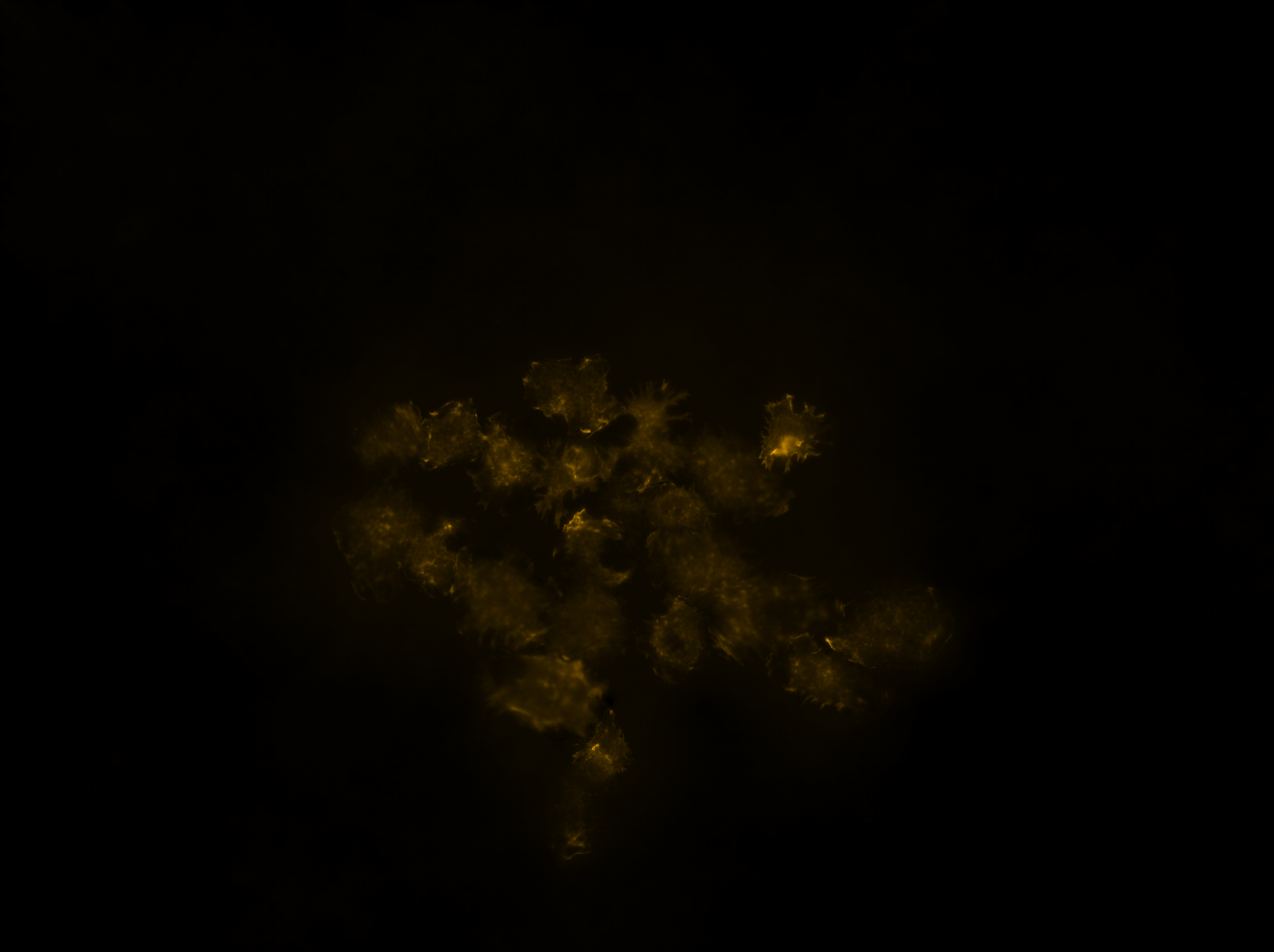

Supplement: Additional file 6 — The zip archive contains simulated images showing B cell nuclei and cytoskeleton with corresponding ground truth. (ZIP 119808 kb) [file 12859_2017_1591_MOESM6_ESM.zip › simulated B cells/cytoskeleton/overlaying/cell002.png]

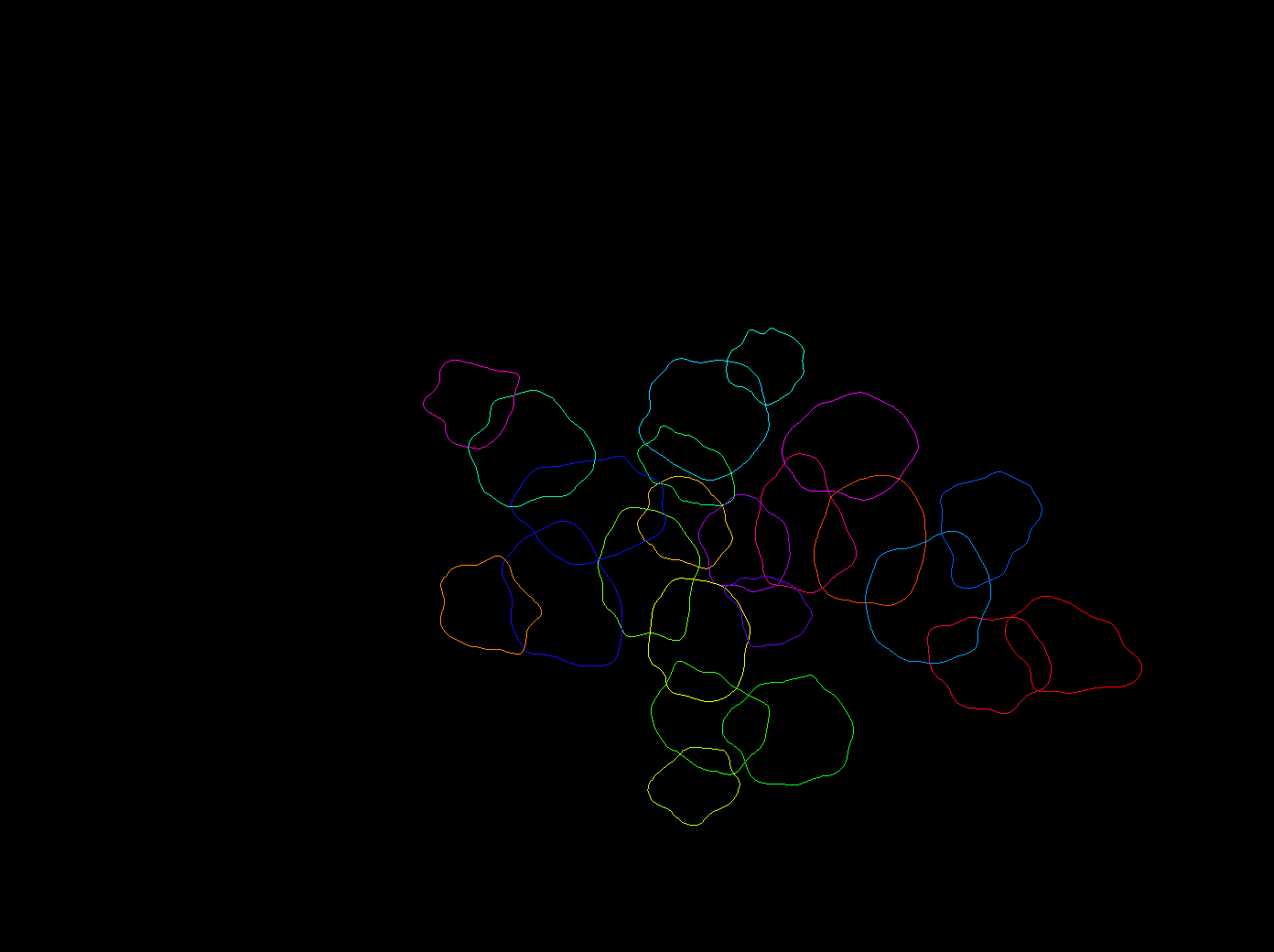

Supplement: Additional file 6 — The zip archive contains simulated images showing B cell nuclei and cytoskeleton with corresponding ground truth. (ZIP 119808 kb) [file 12859_2017_1591_MOESM6_ESM.zip › simulated B cells/cytoskeleton/overlaying/cell003 gt.png]

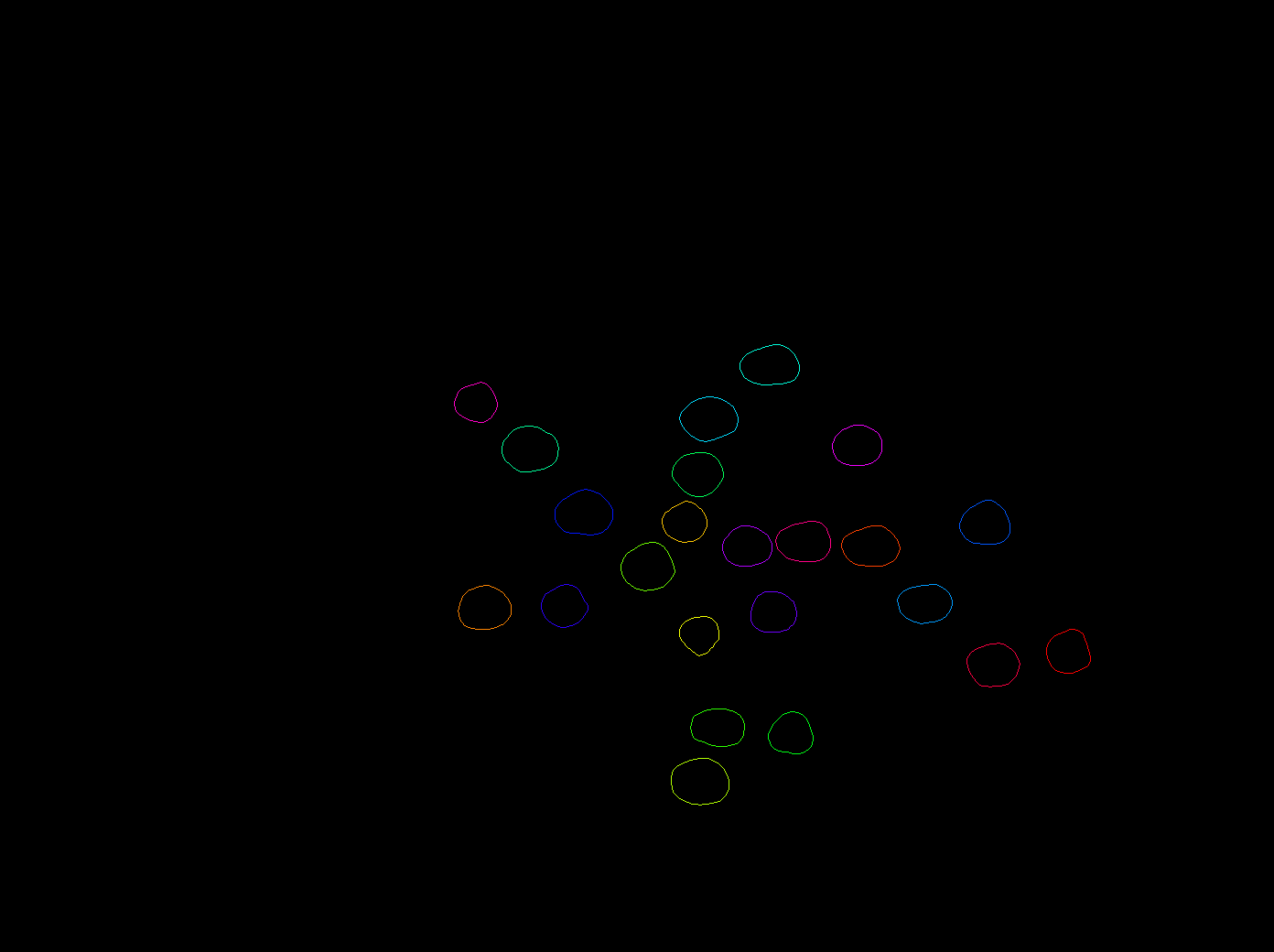

Supplement: Additional file 6 — The zip archive contains simulated images showing B cell nuclei and cytoskeleton with corresponding ground truth. (ZIP 119808 kb) [file 12859_2017_1591_MOESM6_ESM.zip › simulated B cells/cytoskeleton/overlaying/cell003 seeds.png]

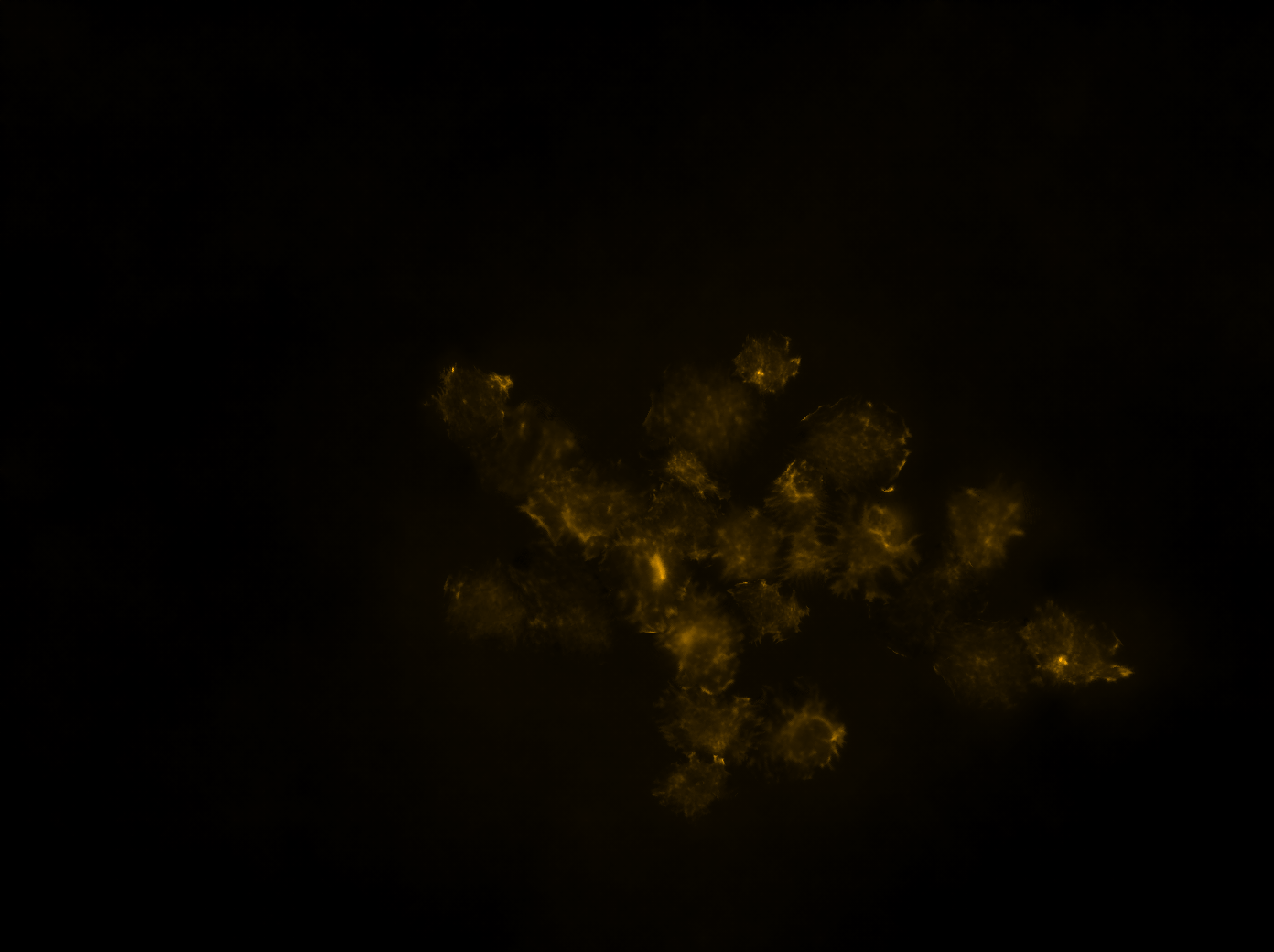

Supplement: Additional file 6 — The zip archive contains simulated images showing B cell nuclei and cytoskeleton with corresponding ground truth. (ZIP 119808 kb) [file 12859_2017_1591_MOESM6_ESM.zip › simulated B cells/cytoskeleton/overlaying/cell003.png]

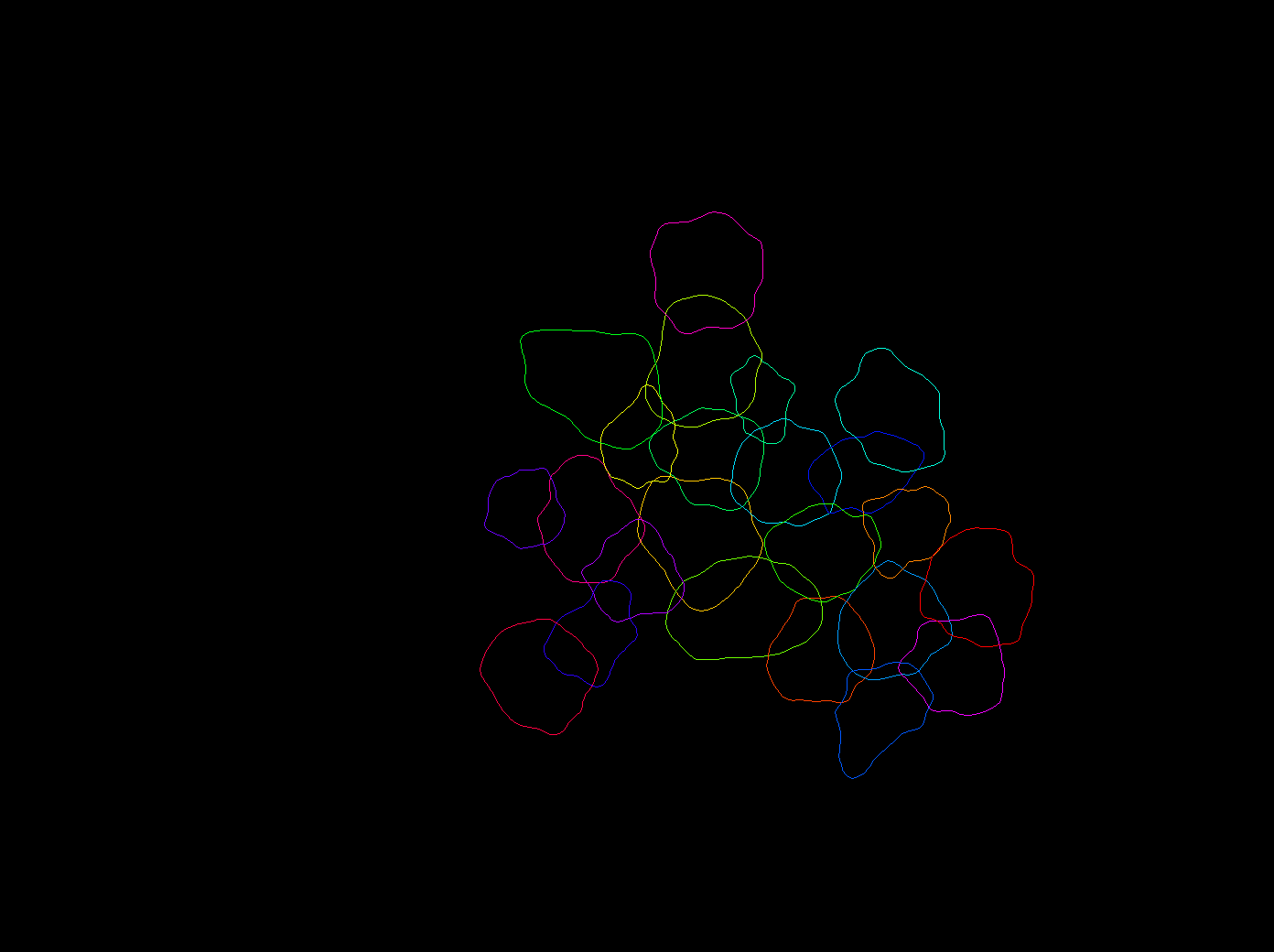

Supplement: Additional file 6 — The zip archive contains simulated images showing B cell nuclei and cytoskeleton with corresponding ground truth. (ZIP 119808 kb) [file 12859_2017_1591_MOESM6_ESM.zip › simulated B cells/cytoskeleton/overlaying/cell004 gt.png]

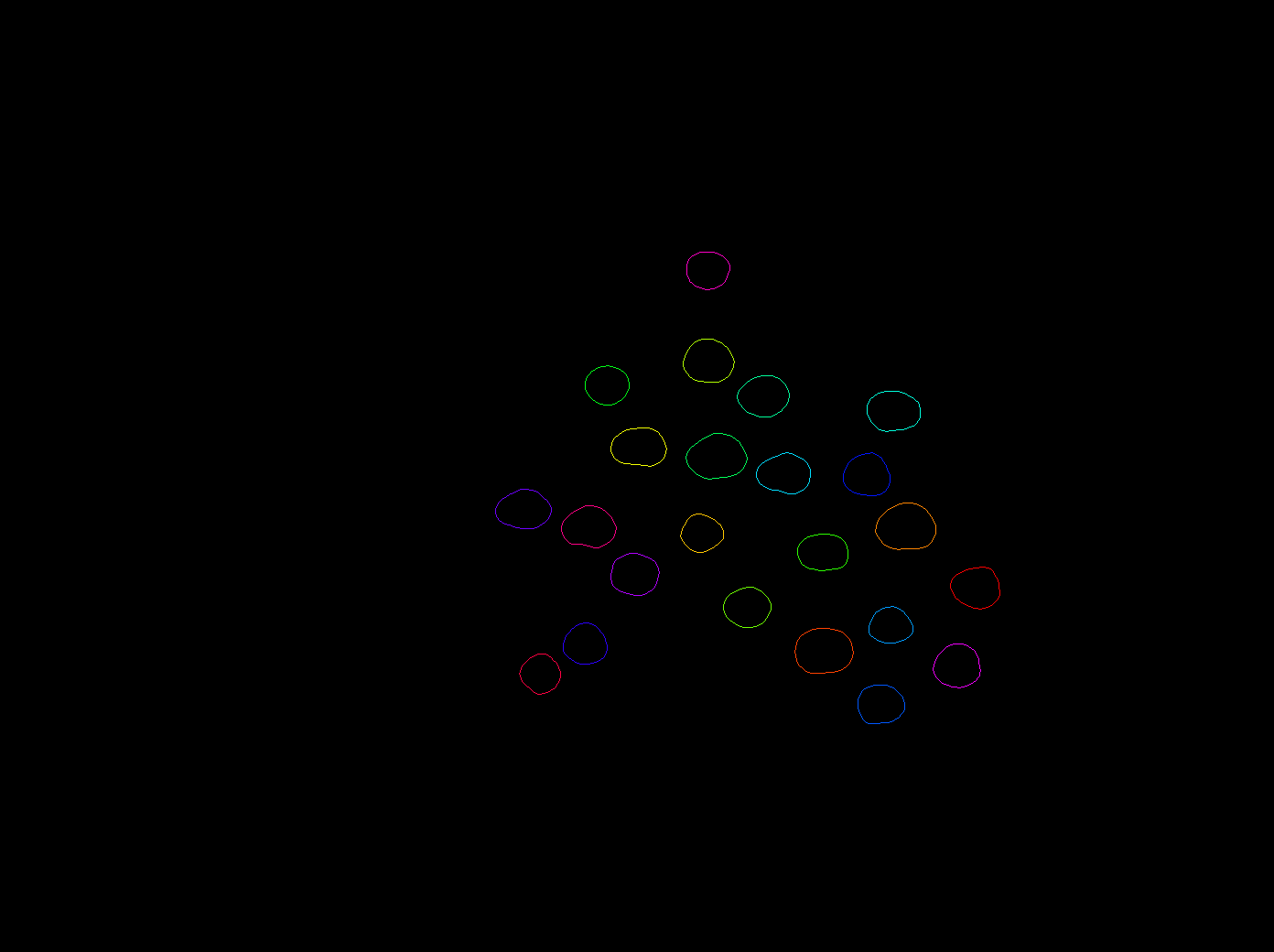

Supplement: Additional file 6 — The zip archive contains simulated images showing B cell nuclei and cytoskeleton with corresponding ground truth. (ZIP 119808 kb) [file 12859_2017_1591_MOESM6_ESM.zip › simulated B cells/cytoskeleton/overlaying/cell004 seeds.png]

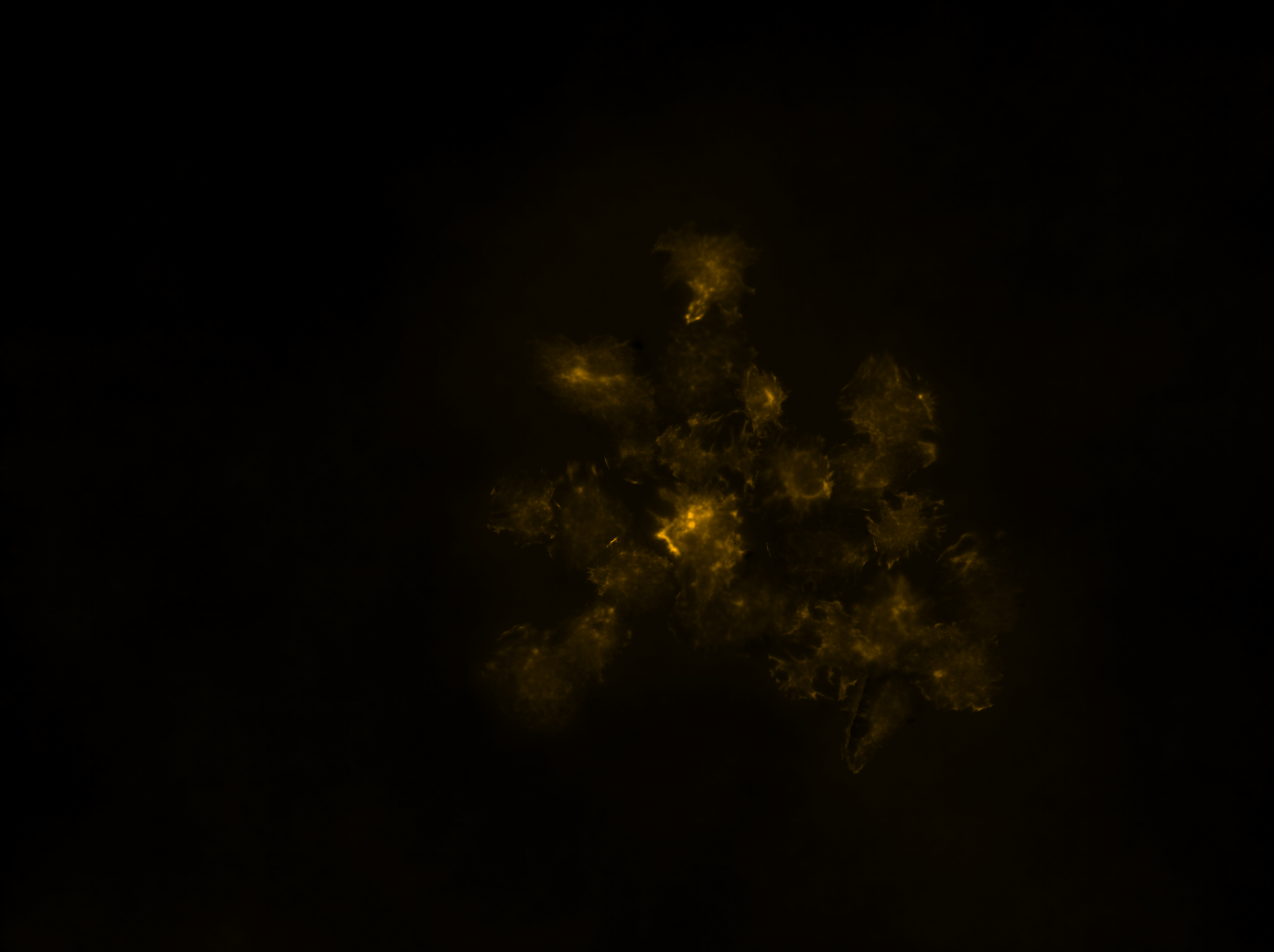

Supplement: Additional file 6 — The zip archive contains simulated images showing B cell nuclei and cytoskeleton with corresponding ground truth. (ZIP 119808 kb) [file 12859_2017_1591_MOESM6_ESM.zip › simulated B cells/cytoskeleton/overlaying/cell004.png]

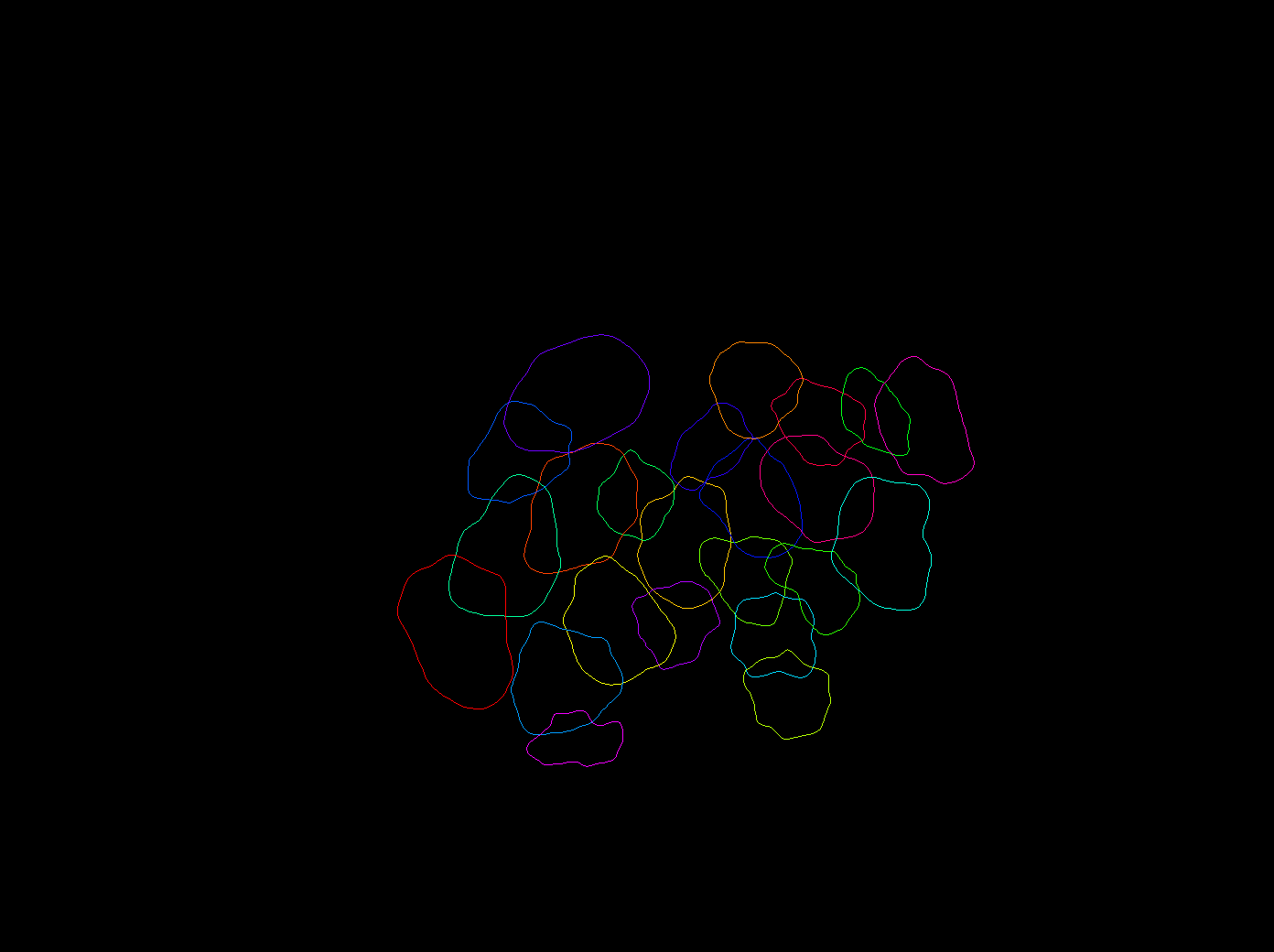

Supplement: Additional file 6 — The zip archive contains simulated images showing B cell nuclei and cytoskeleton with corresponding ground truth. (ZIP 119808 kb) [file 12859_2017_1591_MOESM6_ESM.zip › simulated B cells/cytoskeleton/overlaying/cell005 gt.png]

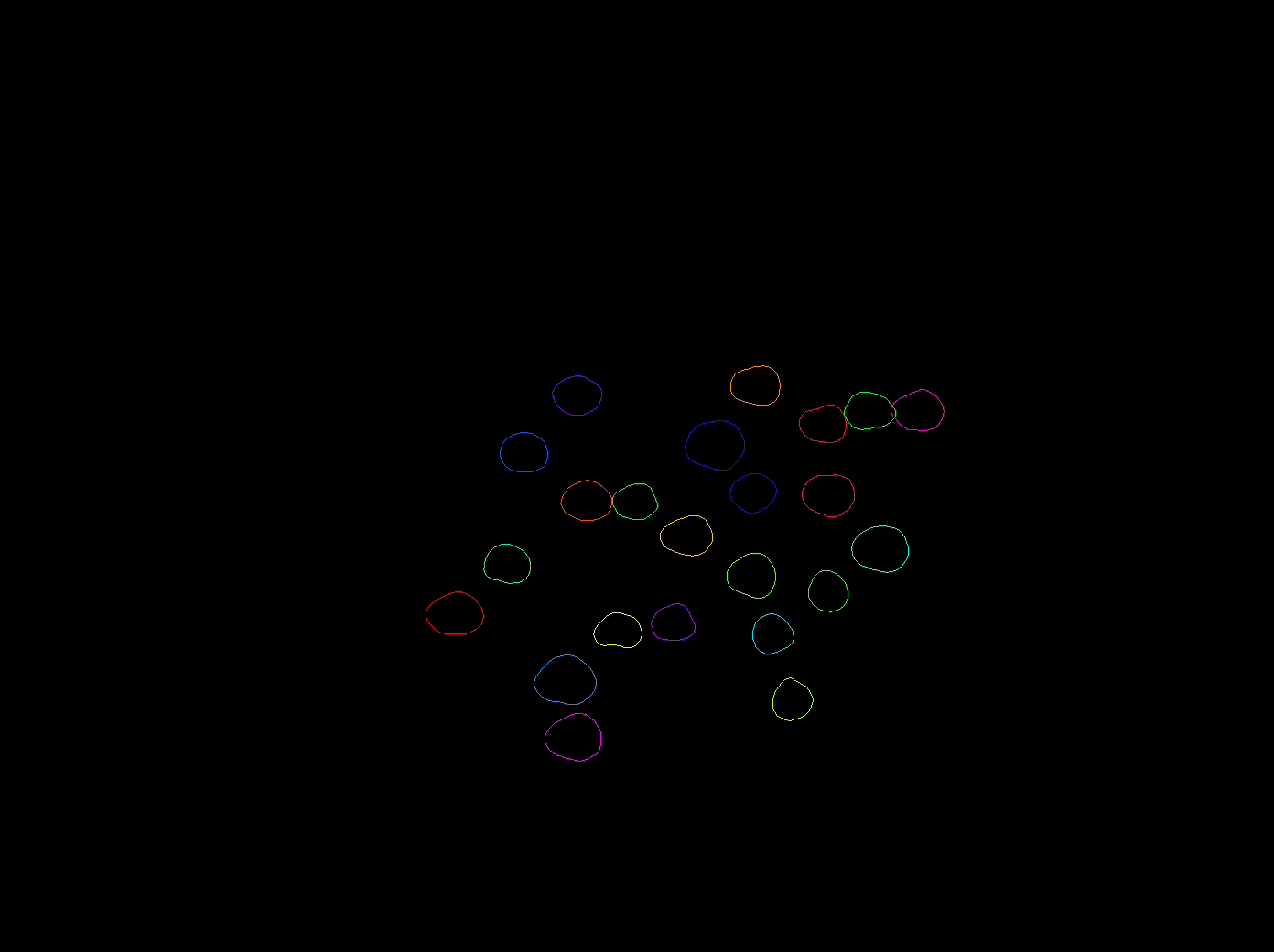

Supplement: Additional file 6 — The zip archive contains simulated images showing B cell nuclei and cytoskeleton with corresponding ground truth. (ZIP 119808 kb) [file 12859_2017_1591_MOESM6_ESM.zip › simulated B cells/cytoskeleton/overlaying/cell005 seeds.png]

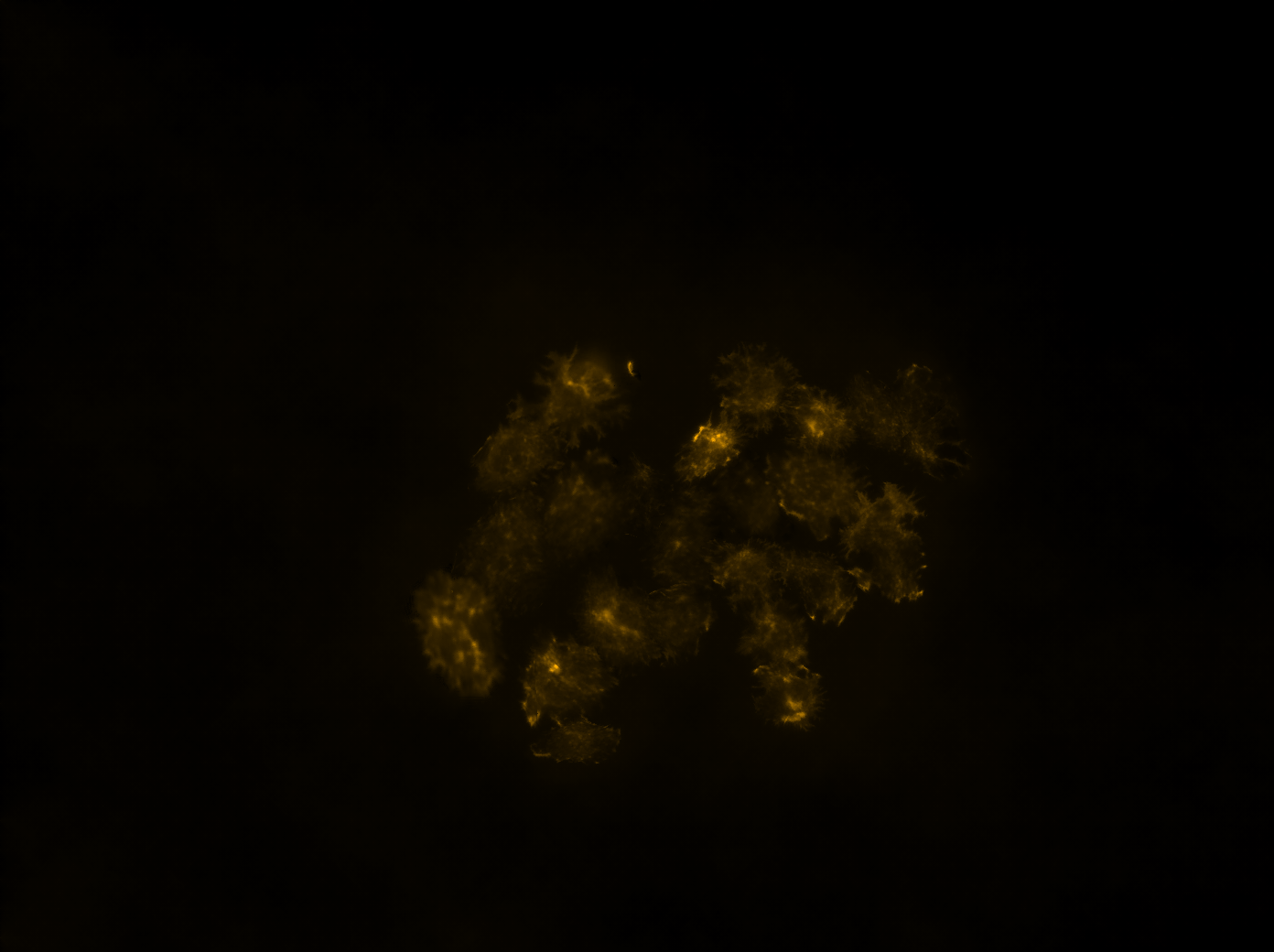

Supplement: Additional file 6 — The zip archive contains simulated images showing B cell nuclei and cytoskeleton with corresponding ground truth. (ZIP 119808 kb) [file 12859_2017_1591_MOESM6_ESM.zip › simulated B cells/cytoskeleton/overlaying/cell005.png]

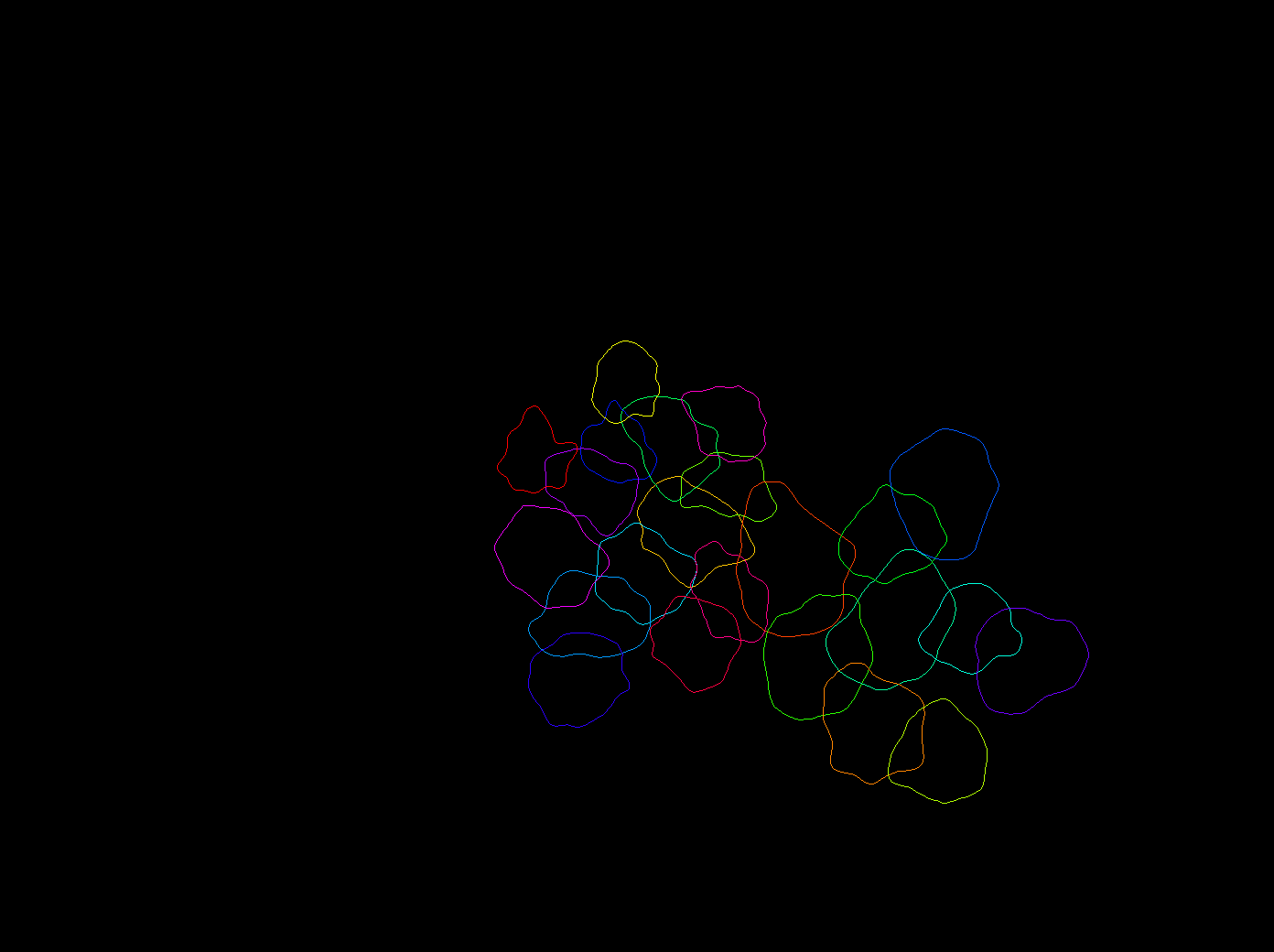

Supplement: Additional file 6 — The zip archive contains simulated images showing B cell nuclei and cytoskeleton with corresponding ground truth. (ZIP 119808 kb) [file 12859_2017_1591_MOESM6_ESM.zip › simulated B cells/cytoskeleton/overlaying/cell006 gt.png]

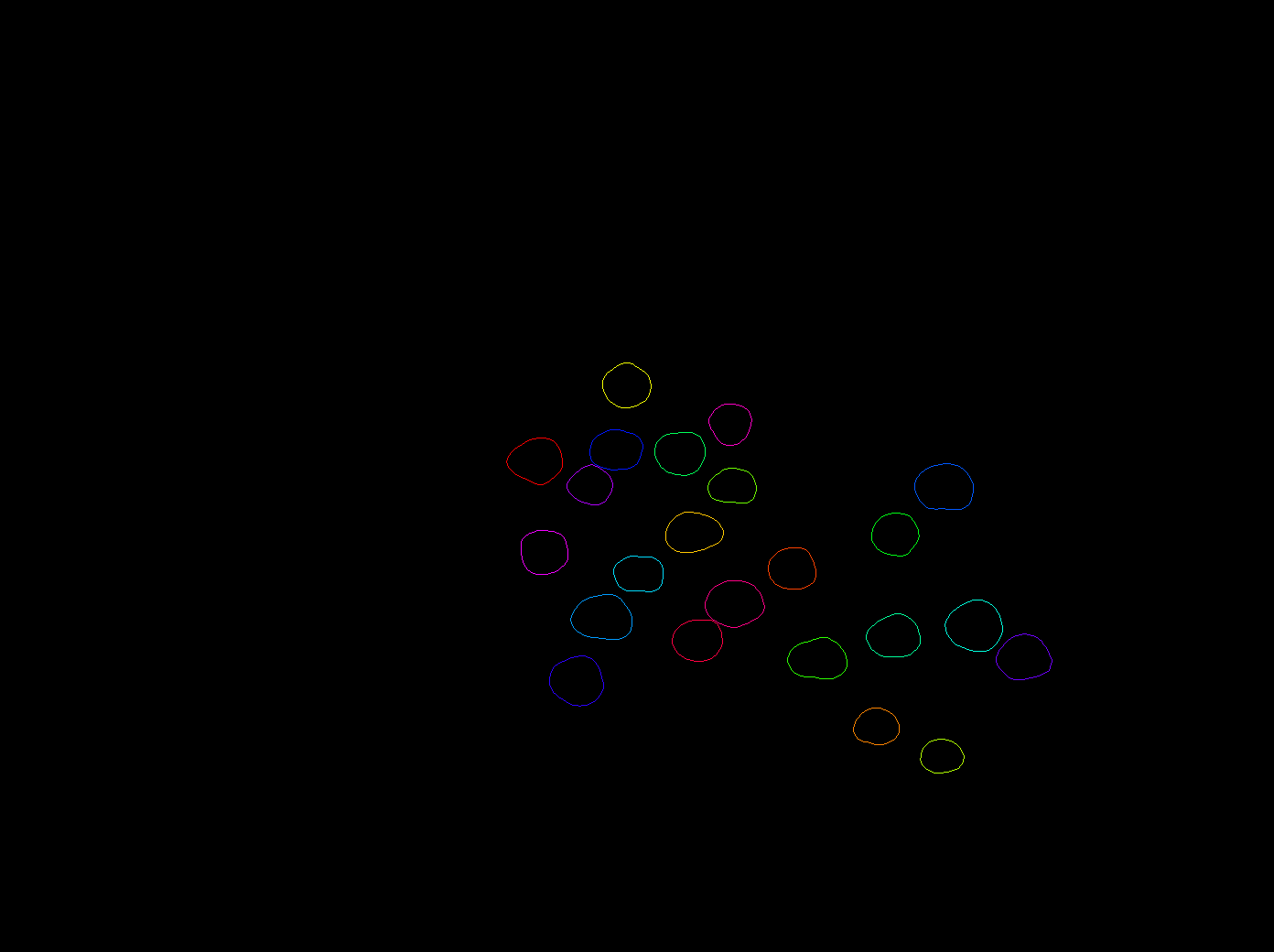

Supplement: Additional file 6 — The zip archive contains simulated images showing B cell nuclei and cytoskeleton with corresponding ground truth. (ZIP 119808 kb) [file 12859_2017_1591_MOESM6_ESM.zip › simulated B cells/cytoskeleton/overlaying/cell006 seeds.png]

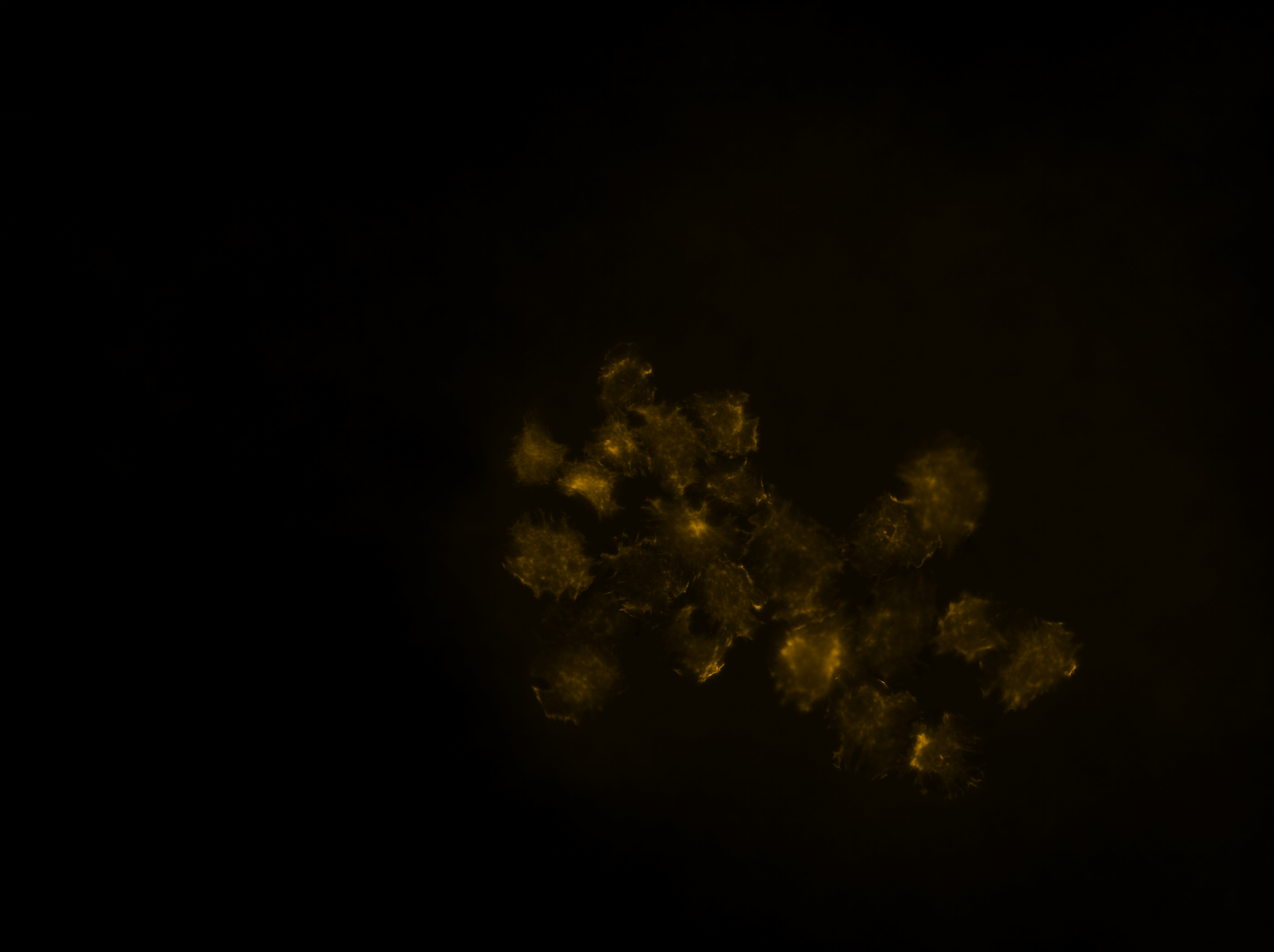

Supplement: Additional file 6 — The zip archive contains simulated images showing B cell nuclei and cytoskeleton with corresponding ground truth. (ZIP 119808 kb) [file 12859_2017_1591_MOESM6_ESM.zip › simulated B cells/cytoskeleton/overlaying/cell006.png]

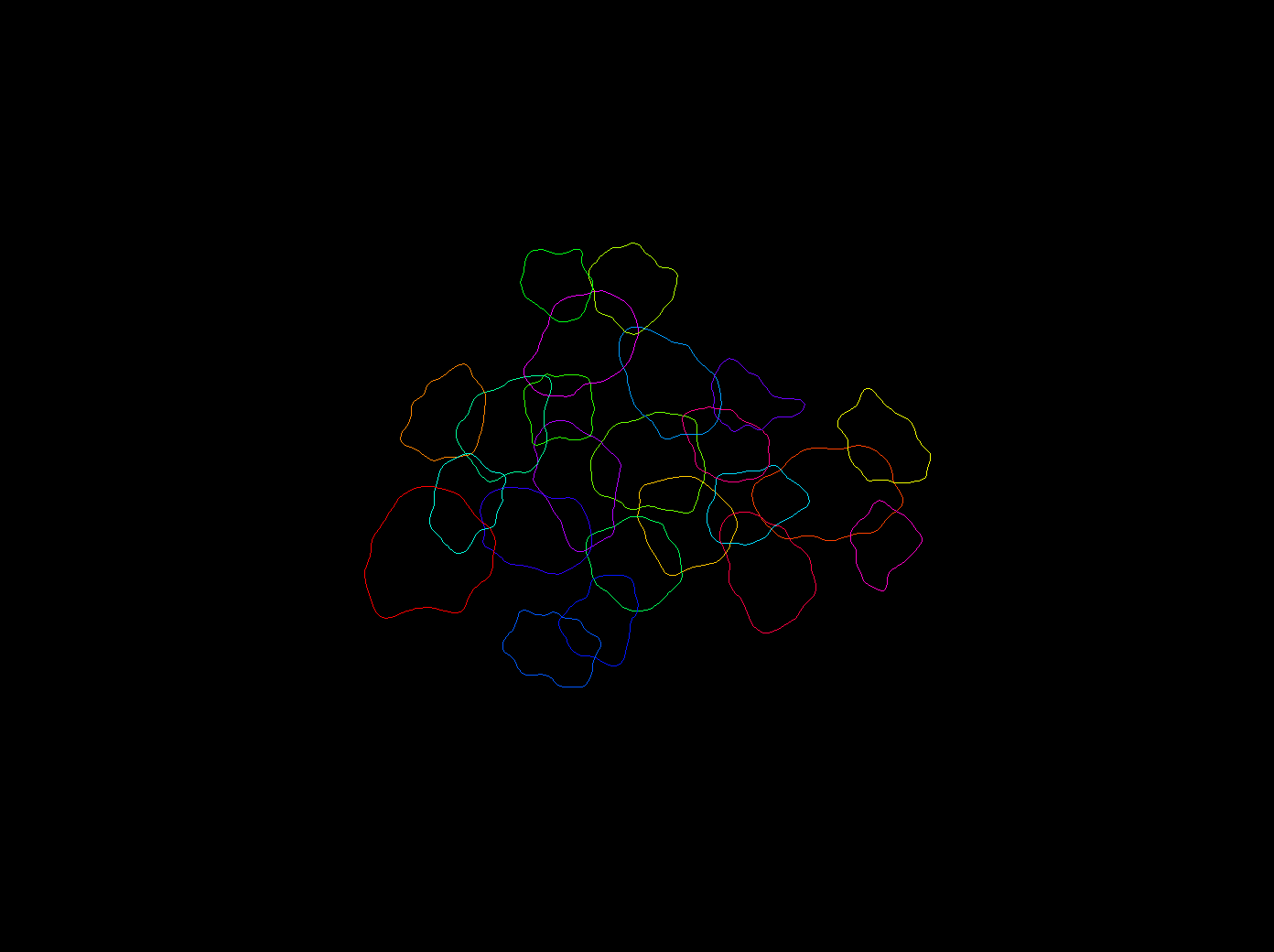

Supplement: Additional file 6 — The zip archive contains simulated images showing B cell nuclei and cytoskeleton with corresponding ground truth. (ZIP 119808 kb) [file 12859_2017_1591_MOESM6_ESM.zip › simulated B cells/cytoskeleton/overlaying/cell007 gt.png]

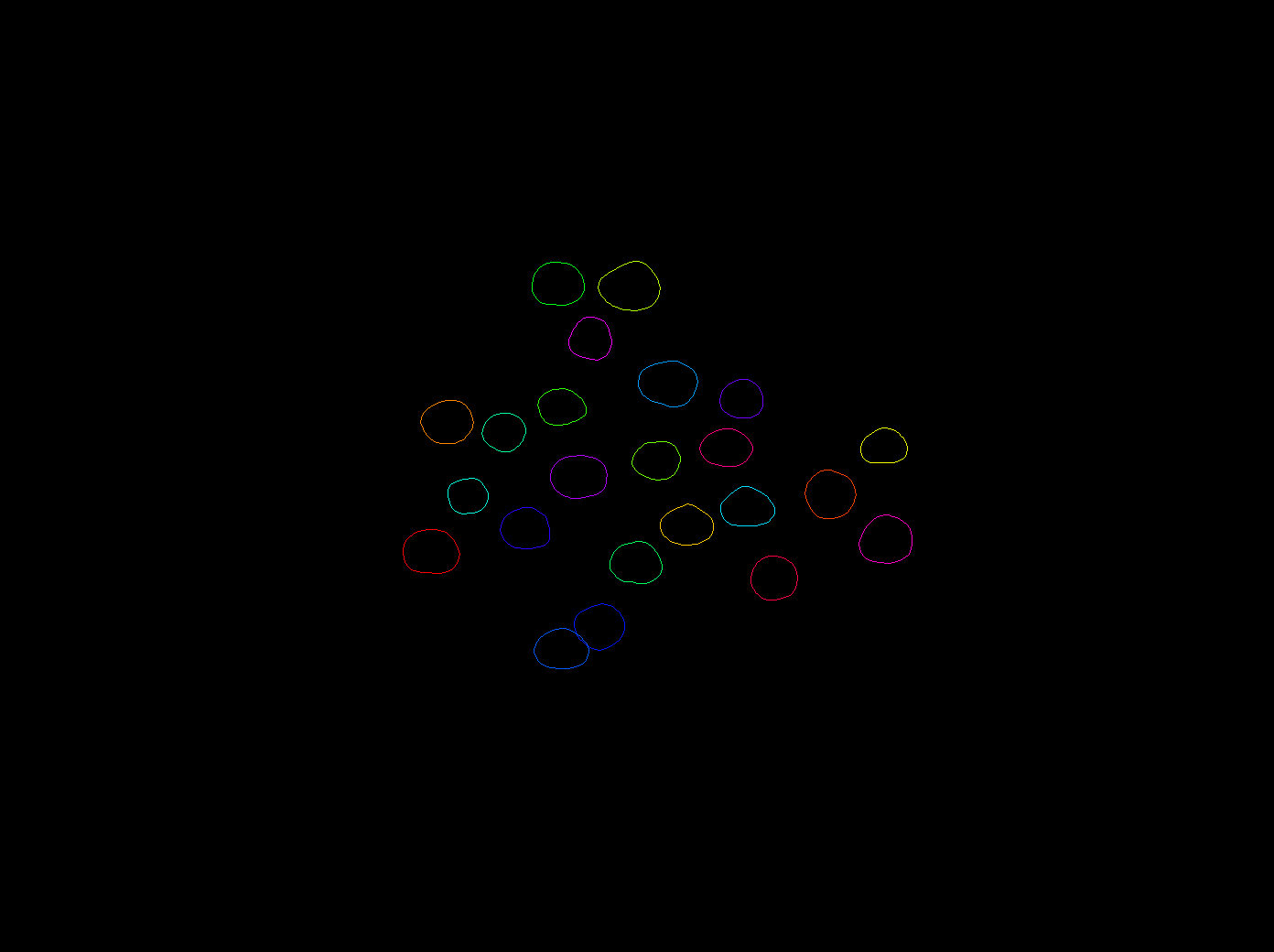

Supplement: Additional file 6 — The zip archive contains simulated images showing B cell nuclei and cytoskeleton with corresponding ground truth. (ZIP 119808 kb) [file 12859_2017_1591_MOESM6_ESM.zip › simulated B cells/cytoskeleton/overlaying/cell007 seeds.png]

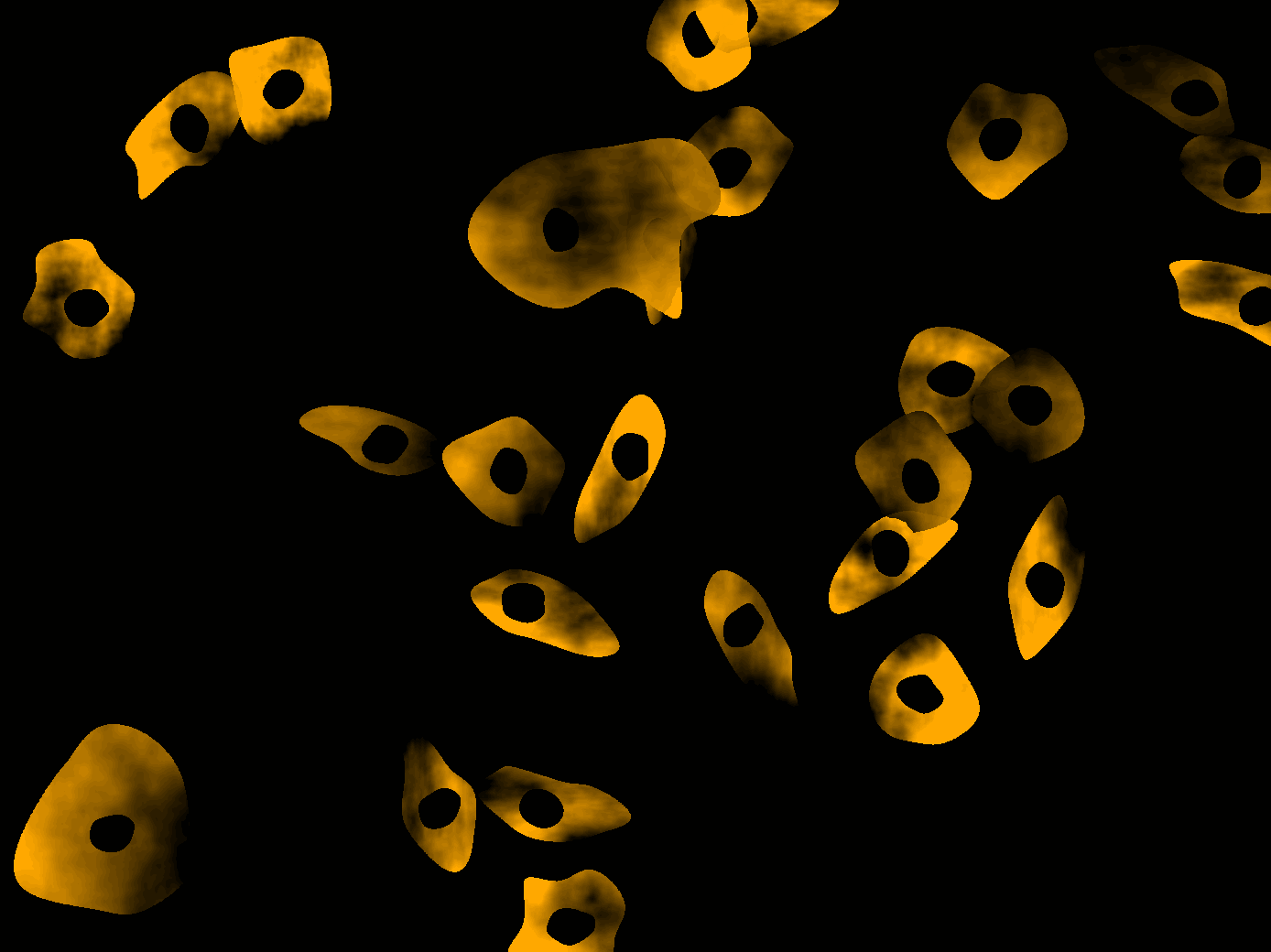

Supplement: Additional file 8 — The zip archive contains all files simulated with SimuCell. These images have been used during the expert observer study. (ZIP 7618.56 kb) [file 12859_2017_1591_MOESM8_ESM.zip › SimuCell - Simulated Images/B cell cytoskeleton/1.png]

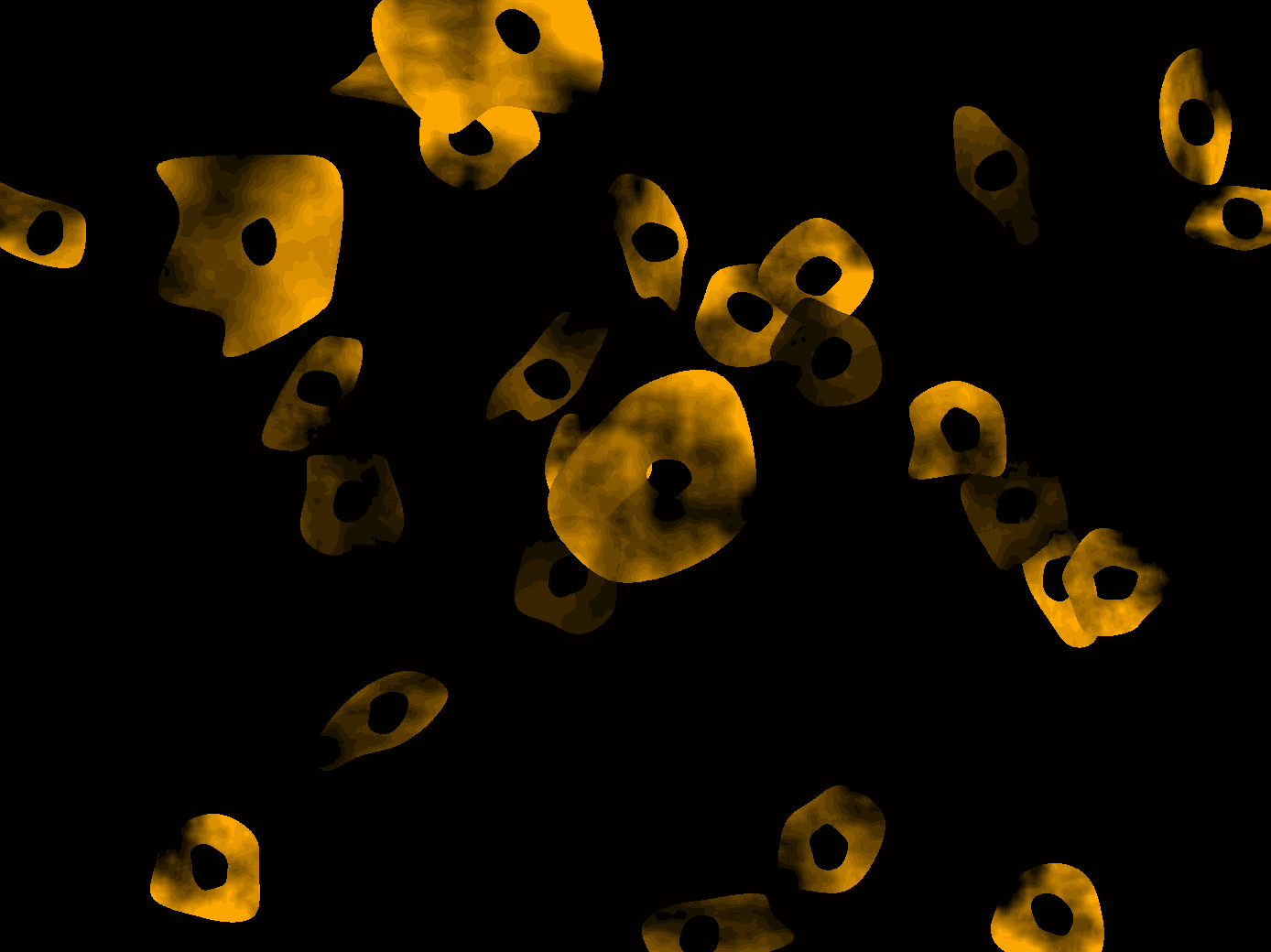

Supplement: Additional file 8 — The zip archive contains all files simulated with SimuCell. These images have been used during the expert observer study. (ZIP 7618.56 kb) [file 12859_2017_1591_MOESM8_ESM.zip › SimuCell - Simulated Images/B cell cytoskeleton/10.png]

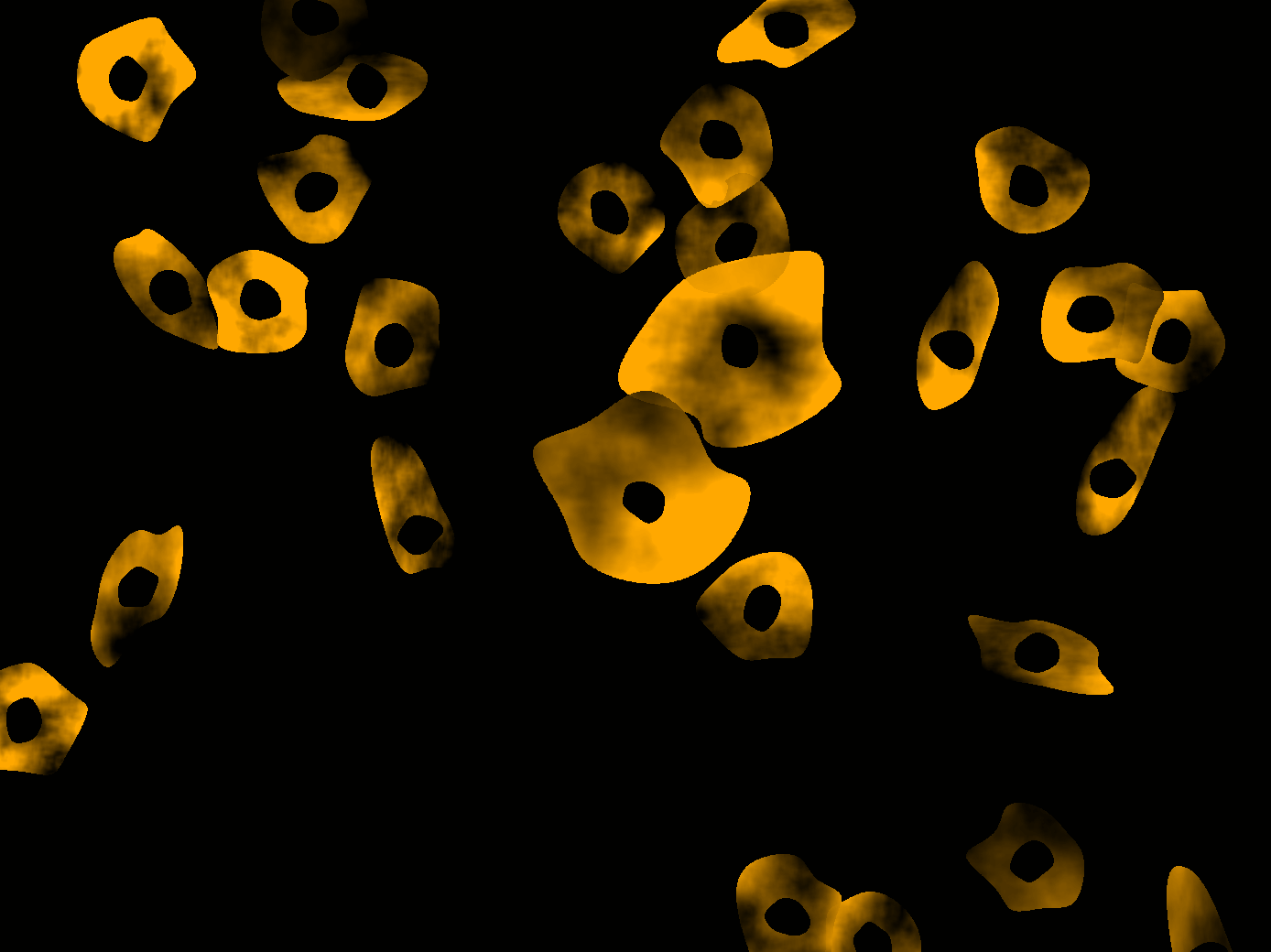

Supplement: Additional file 8 — The zip archive contains all files simulated with SimuCell. These images have been used during the expert observer study. (ZIP 7618.56 kb) [file 12859_2017_1591_MOESM8_ESM.zip › SimuCell - Simulated Images/B cell cytoskeleton/2.png]

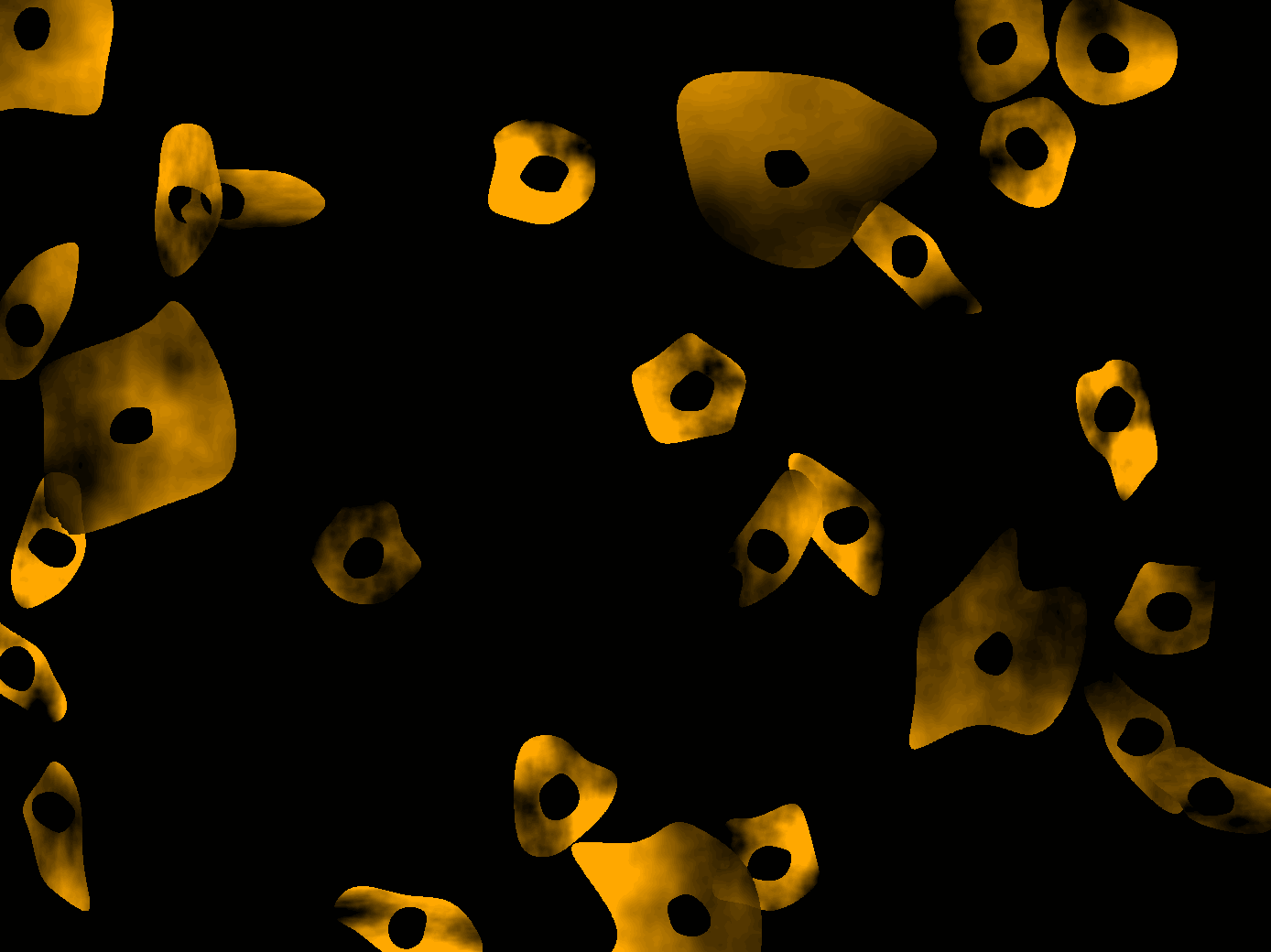

Supplement: Additional file 8 — The zip archive contains all files simulated with SimuCell. These images have been used during the expert observer study. (ZIP 7618.56 kb) [file 12859_2017_1591_MOESM8_ESM.zip › SimuCell - Simulated Images/B cell cytoskeleton/3.png]

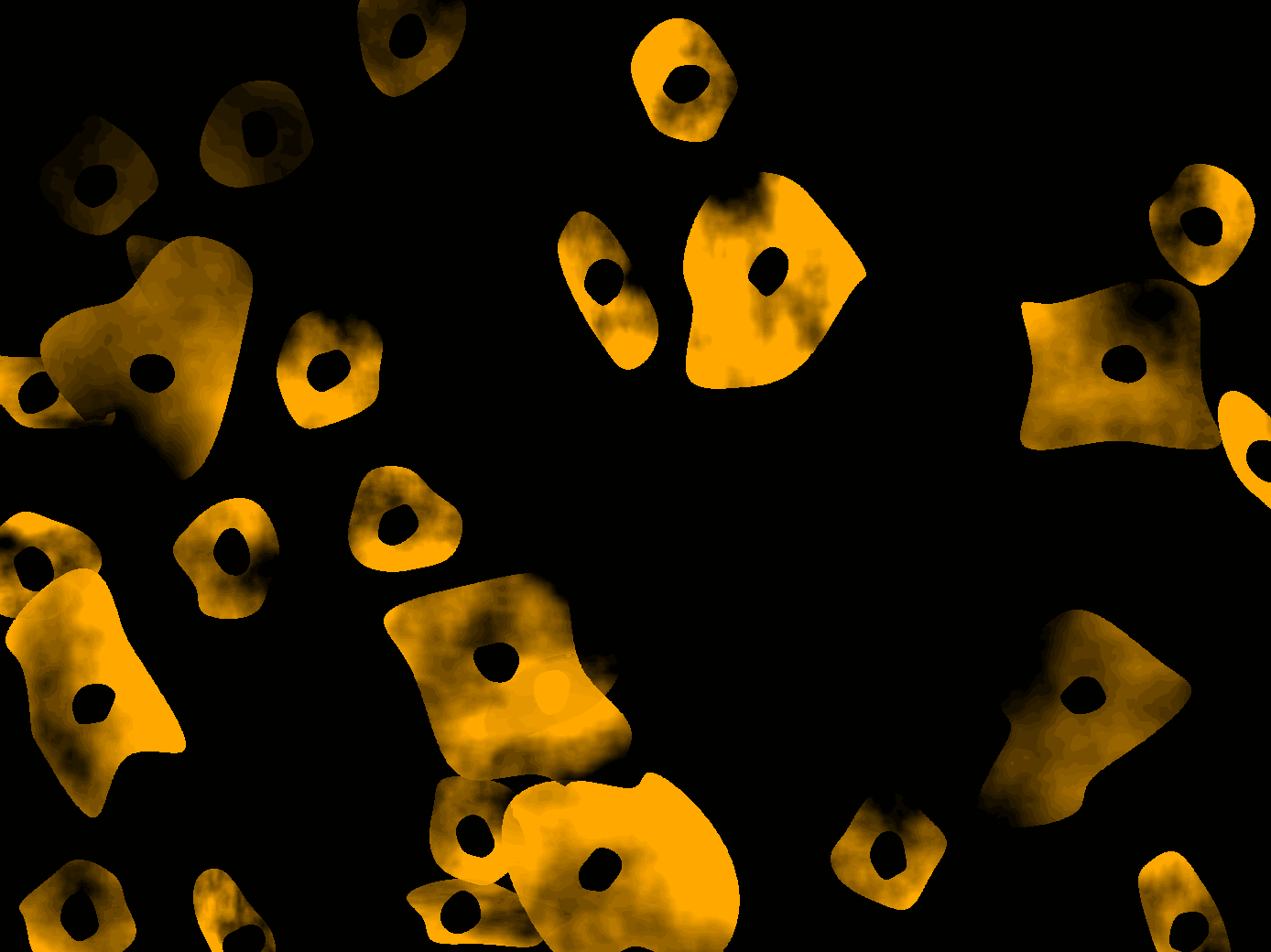

Supplement: Additional file 8 — The zip archive contains all files simulated with SimuCell. These images have been used during the expert observer study. (ZIP 7618.56 kb) [file 12859_2017_1591_MOESM8_ESM.zip › SimuCell - Simulated Images/B cell cytoskeleton/4.png]

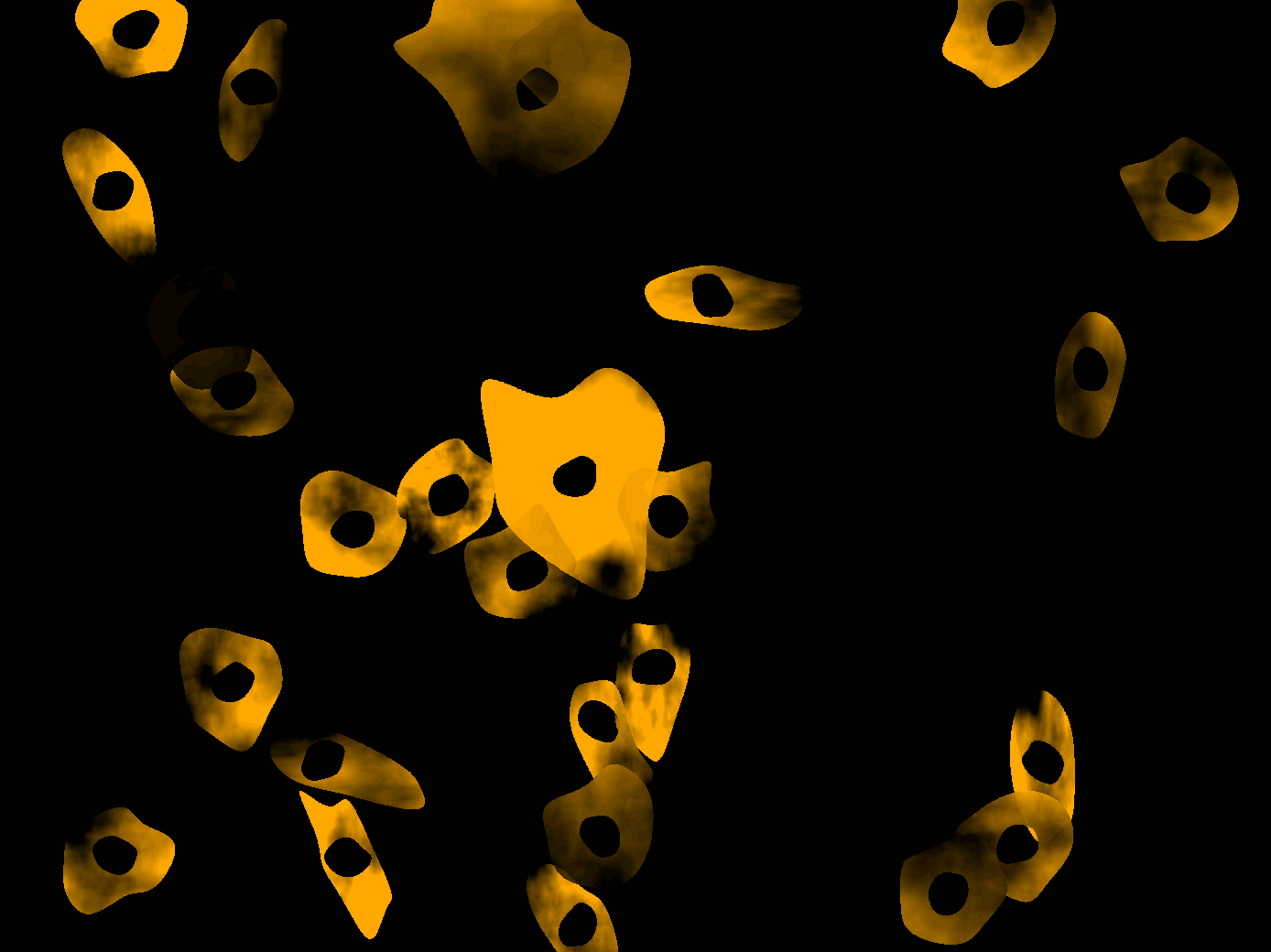

Supplement: Additional file 8 — The zip archive contains all files simulated with SimuCell. These images have been used during the expert observer study. (ZIP 7618.56 kb) [file 12859_2017_1591_MOESM8_ESM.zip › SimuCell - Simulated Images/B cell cytoskeleton/5.png]

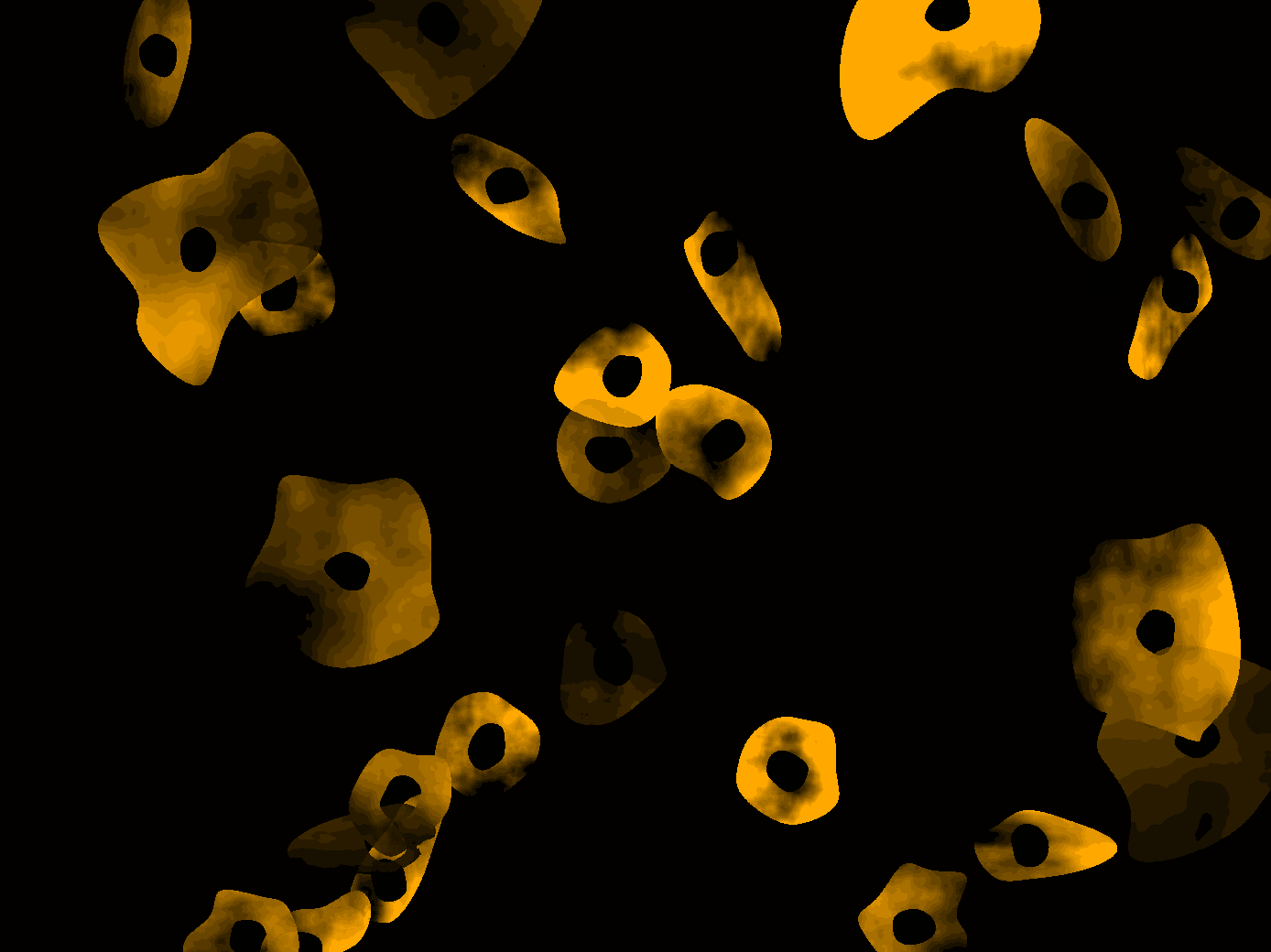

Supplement: Additional file 8 — The zip archive contains all files simulated with SimuCell. These images have been used during the expert observer study. (ZIP 7618.56 kb) [file 12859_2017_1591_MOESM8_ESM.zip › SimuCell - Simulated Images/B cell cytoskeleton/6.png]

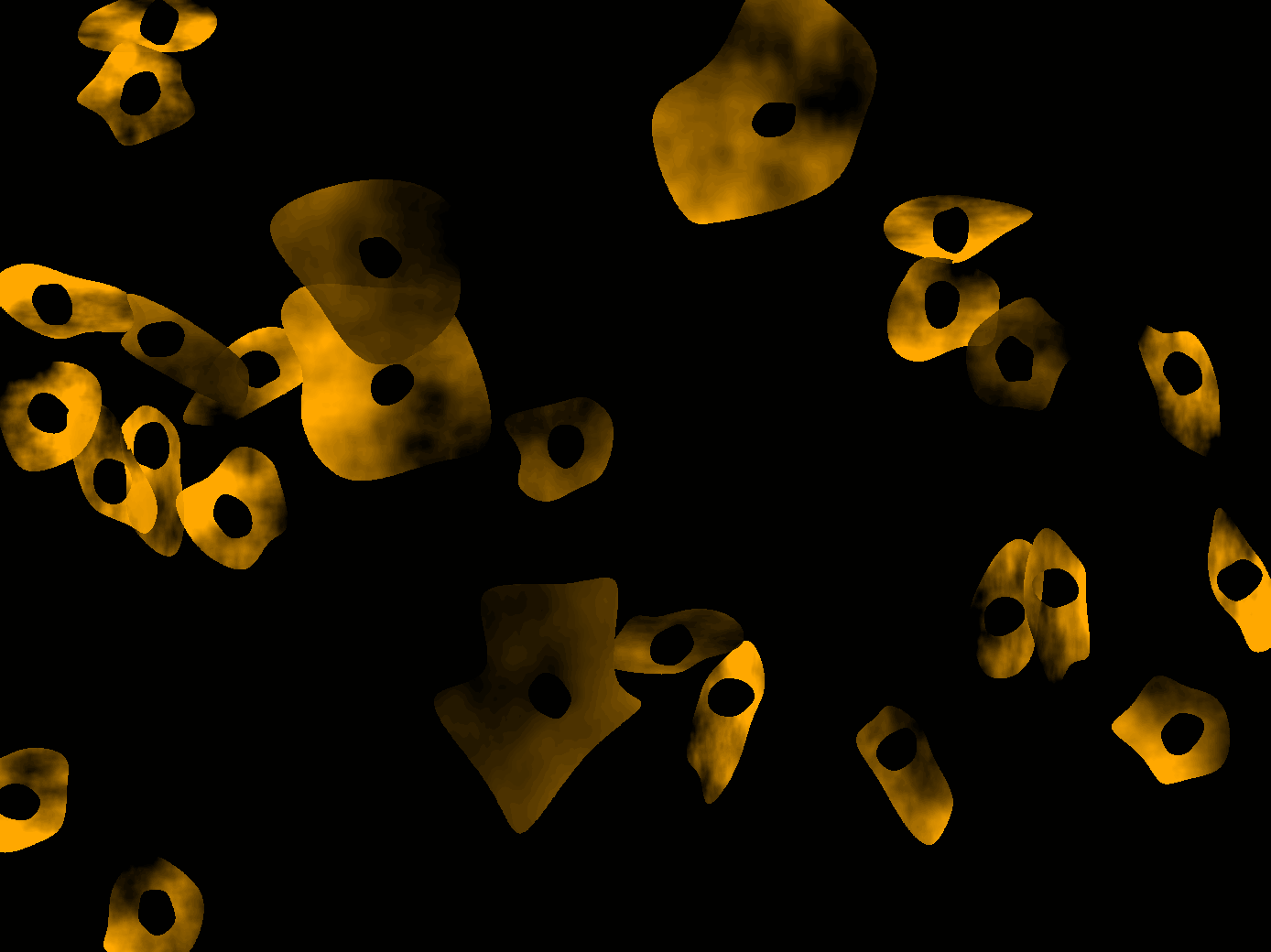

Supplement: Additional file 8 — The zip archive contains all files simulated with SimuCell. These images have been used during the expert observer study. (ZIP 7618.56 kb) [file 12859_2017_1591_MOESM8_ESM.zip › SimuCell - Simulated Images/B cell cytoskeleton/7.png]

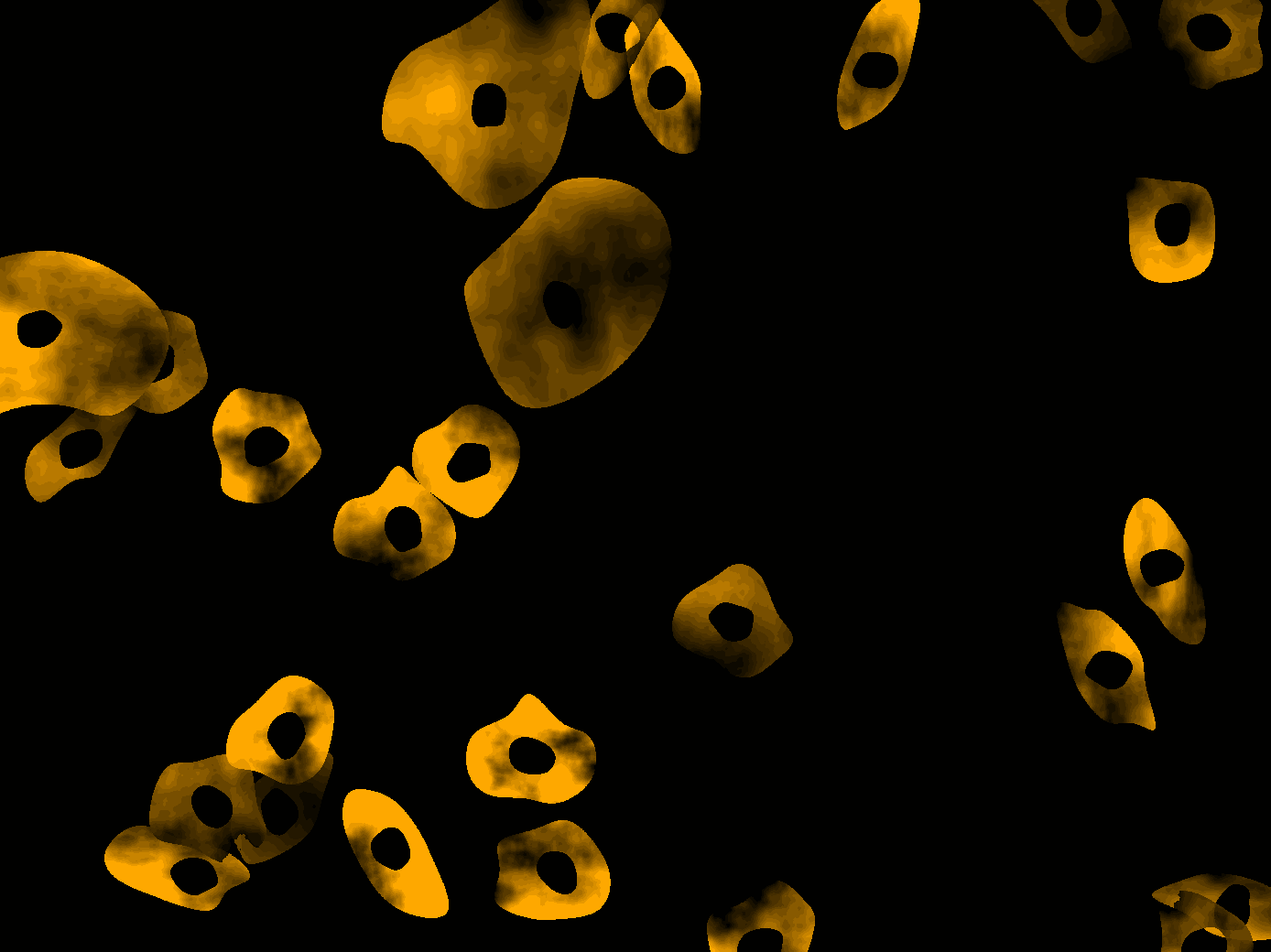

Supplement: Additional file 8 — The zip archive contains all files simulated with SimuCell. These images have been used during the expert observer study. (ZIP 7618.56 kb) [file 12859_2017_1591_MOESM8_ESM.zip › SimuCell - Simulated Images/B cell cytoskeleton/8.png]

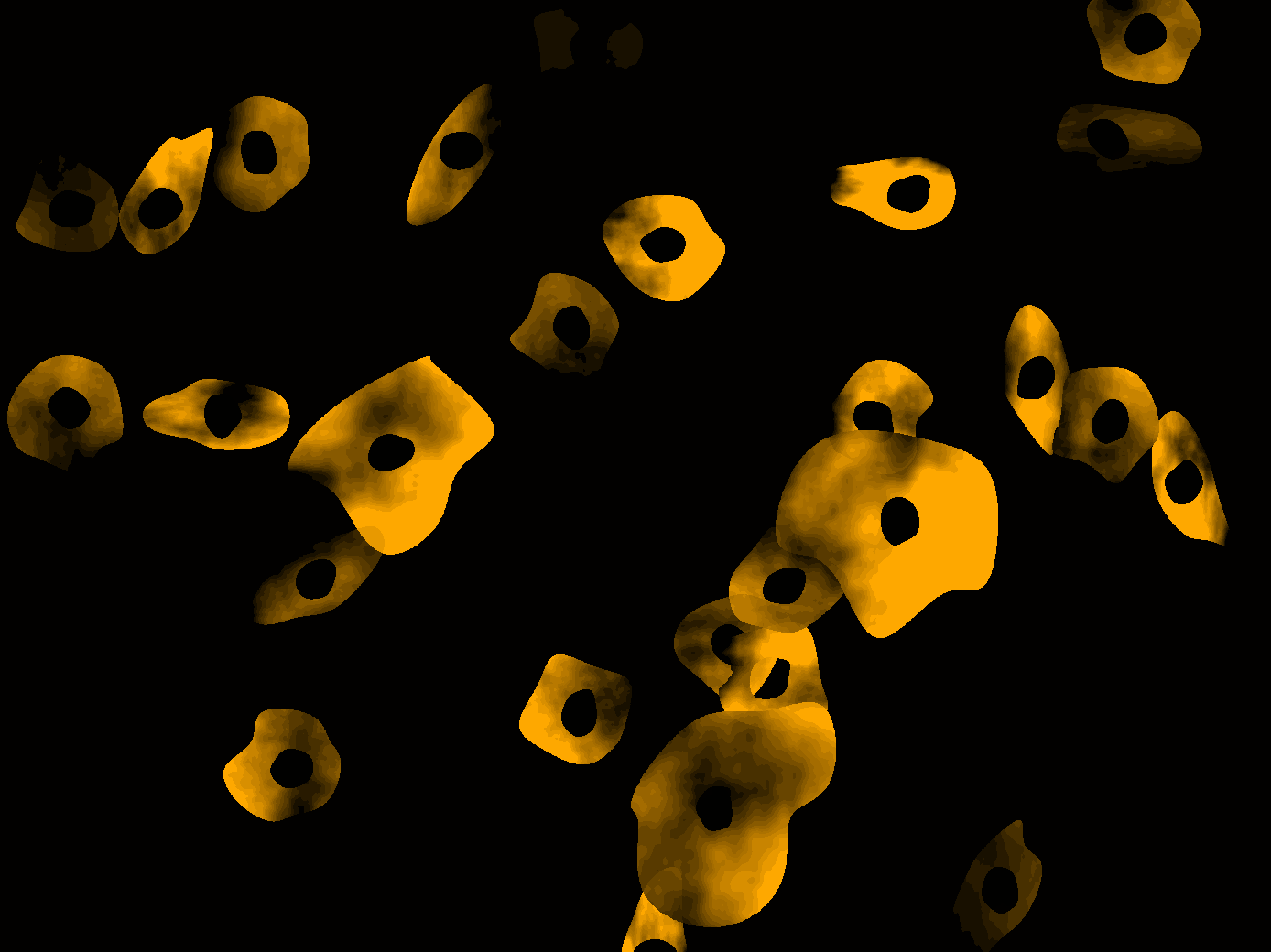

Supplement: Additional file 8 — The zip archive contains all files simulated with SimuCell. These images have been used during the expert observer study. (ZIP 7618.56 kb) [file 12859_2017_1591_MOESM8_ESM.zip › SimuCell - Simulated Images/B cell cytoskeleton/9.png]

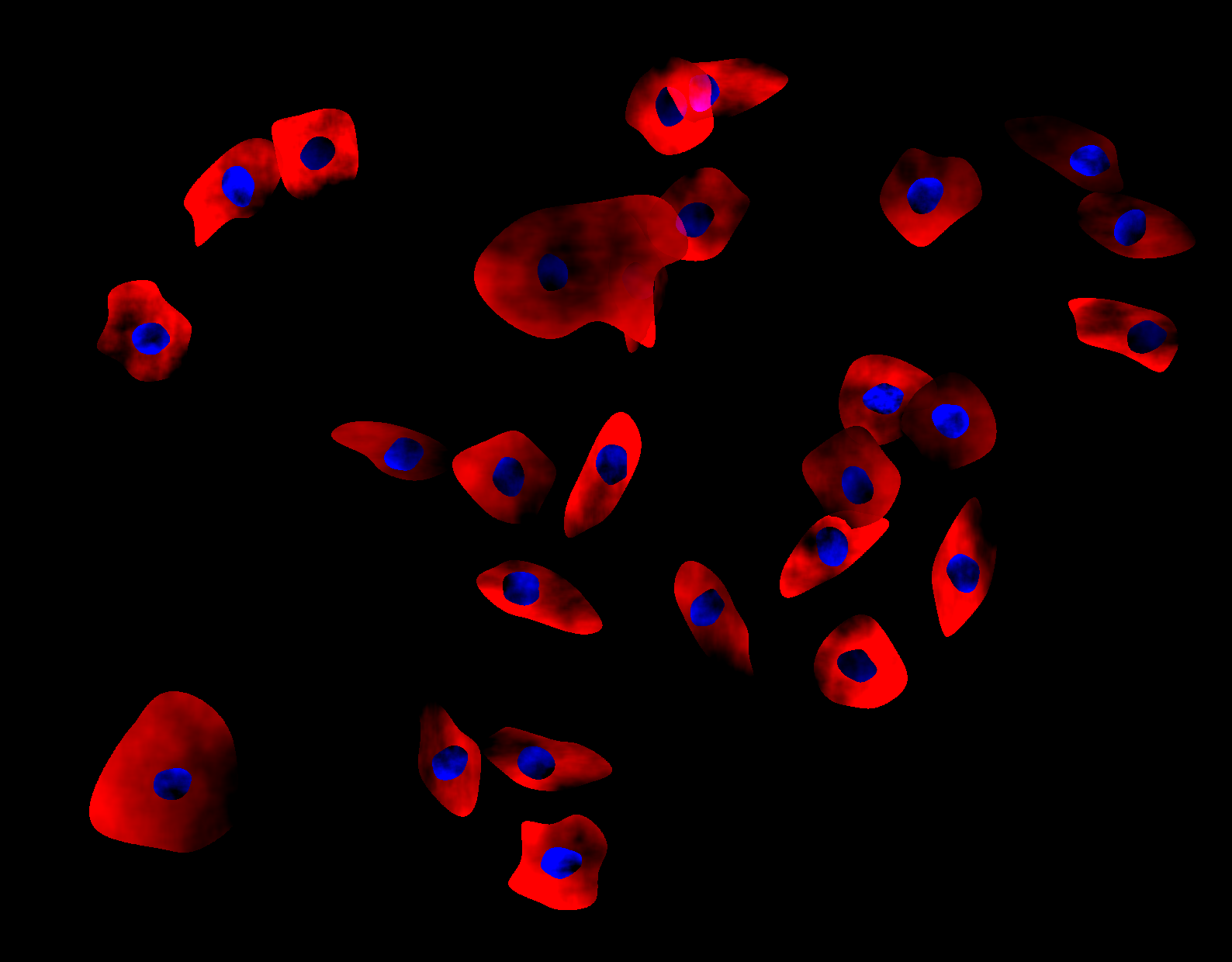

Supplement: Additional file 8 — The zip archive contains all files simulated with SimuCell. These images have been used during the expert observer study. (ZIP 7618.56 kb) [file 12859_2017_1591_MOESM8_ESM.zip › SimuCell - Simulated Images/B cell nuclei/image_01.png]

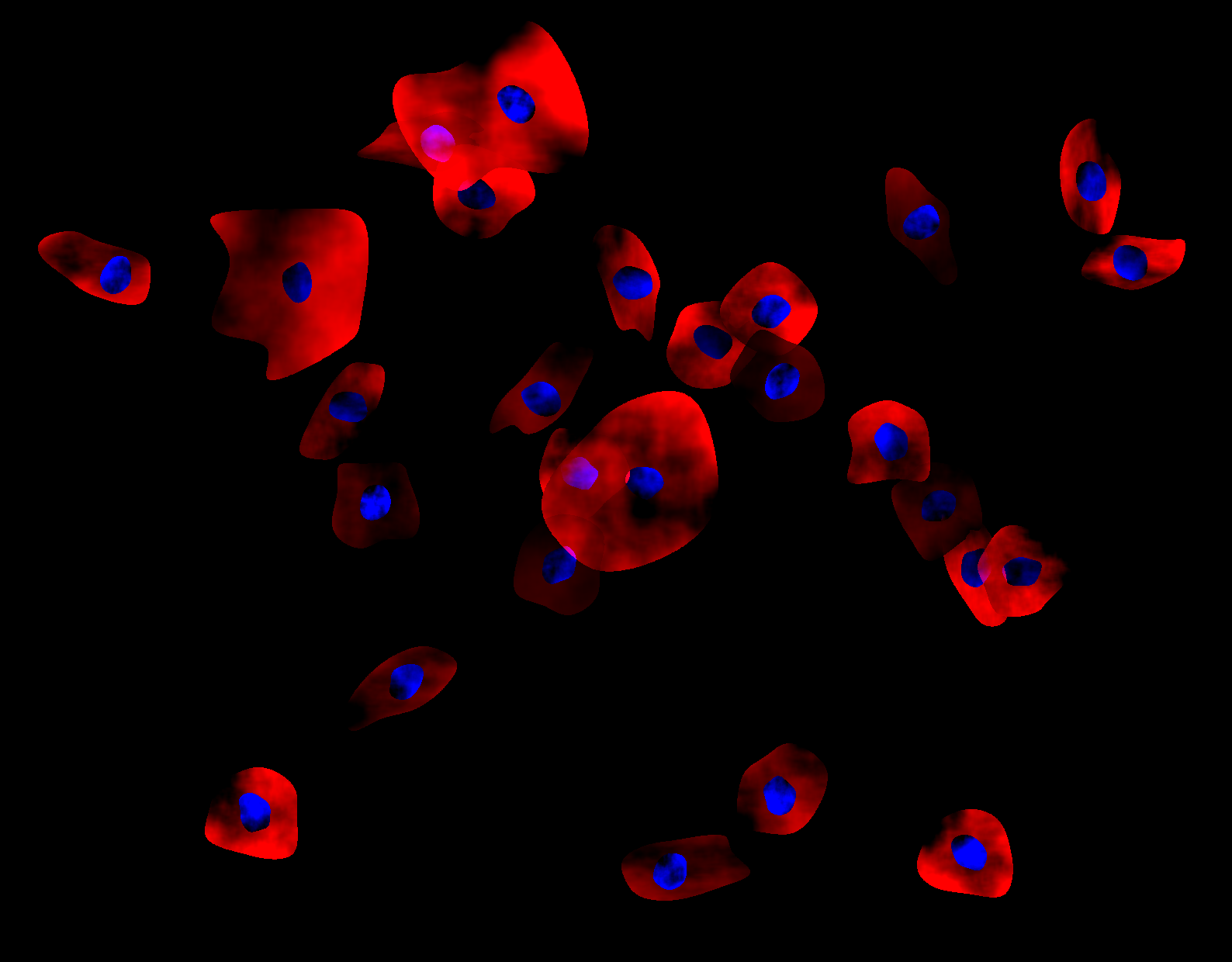

Supplement: Additional file 8 — The zip archive contains all files simulated with SimuCell. These images have been used during the expert observer study. (ZIP 7618.56 kb) [file 12859_2017_1591_MOESM8_ESM.zip › SimuCell - Simulated Images/B cell nuclei/image_101.png]

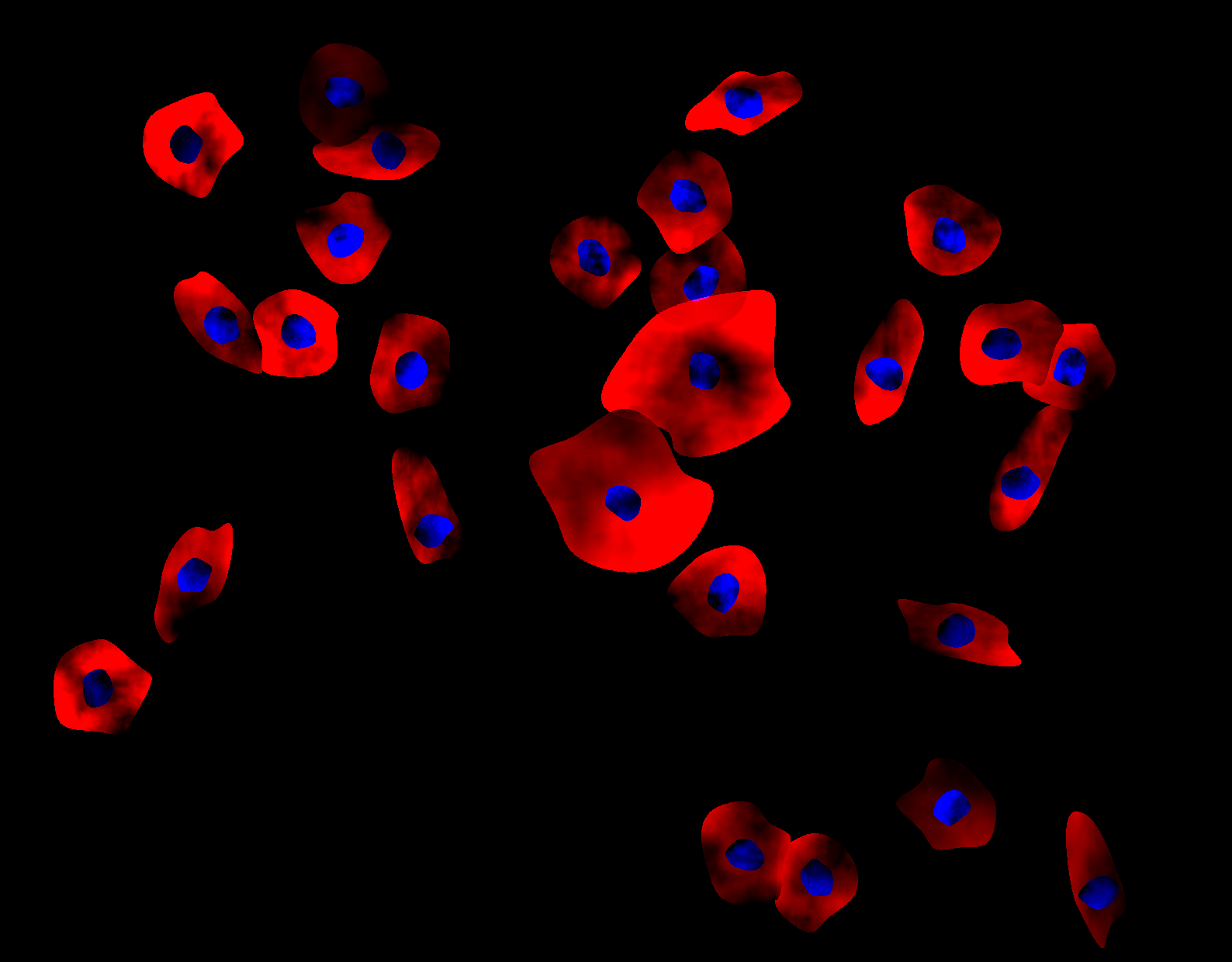

Supplement: Additional file 8 — The zip archive contains all files simulated with SimuCell. These images have been used during the expert observer study. (ZIP 7618.56 kb) [file 12859_2017_1591_MOESM8_ESM.zip › SimuCell - Simulated Images/B cell nuclei/image_11.png]

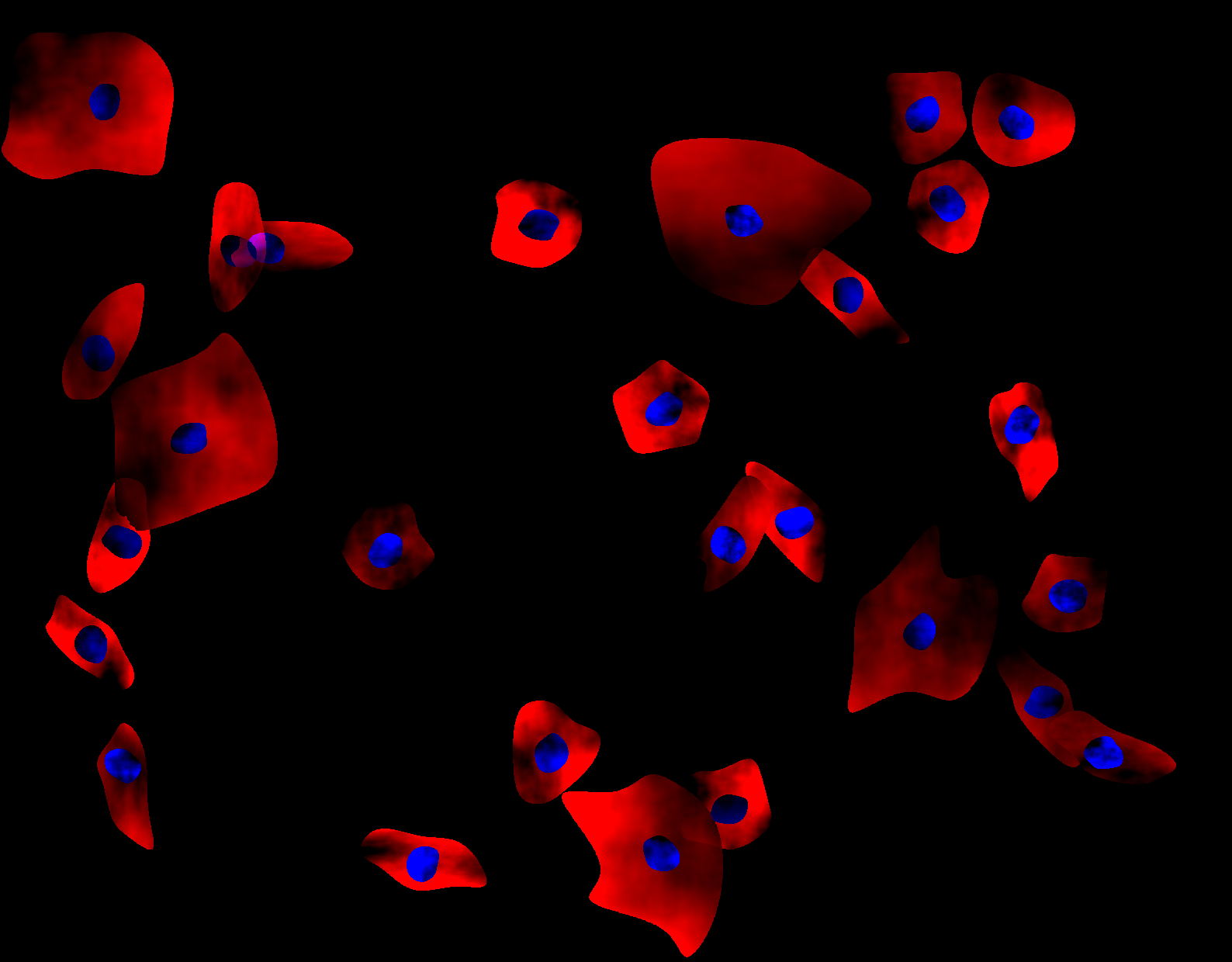

Supplement: Additional file 8 — The zip archive contains all files simulated with SimuCell. These images have been used during the expert observer study. (ZIP 7618.56 kb) [file 12859_2017_1591_MOESM8_ESM.zip › SimuCell - Simulated Images/B cell nuclei/image_21.png]

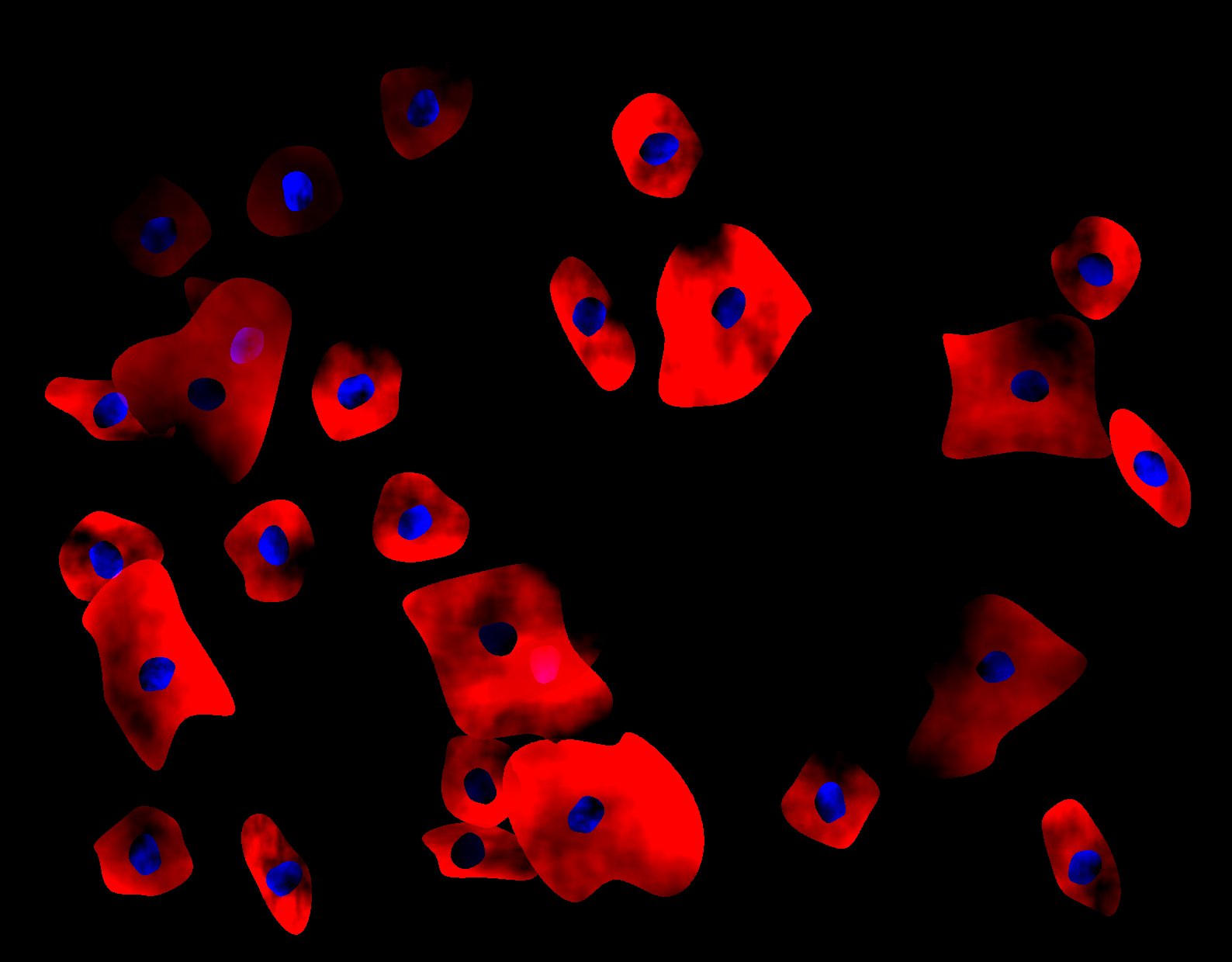

Supplement: Additional file 8 — The zip archive contains all files simulated with SimuCell. These images have been used during the expert observer study. (ZIP 7618.56 kb) [file 12859_2017_1591_MOESM8_ESM.zip › SimuCell - Simulated Images/B cell nuclei/image_31.png]

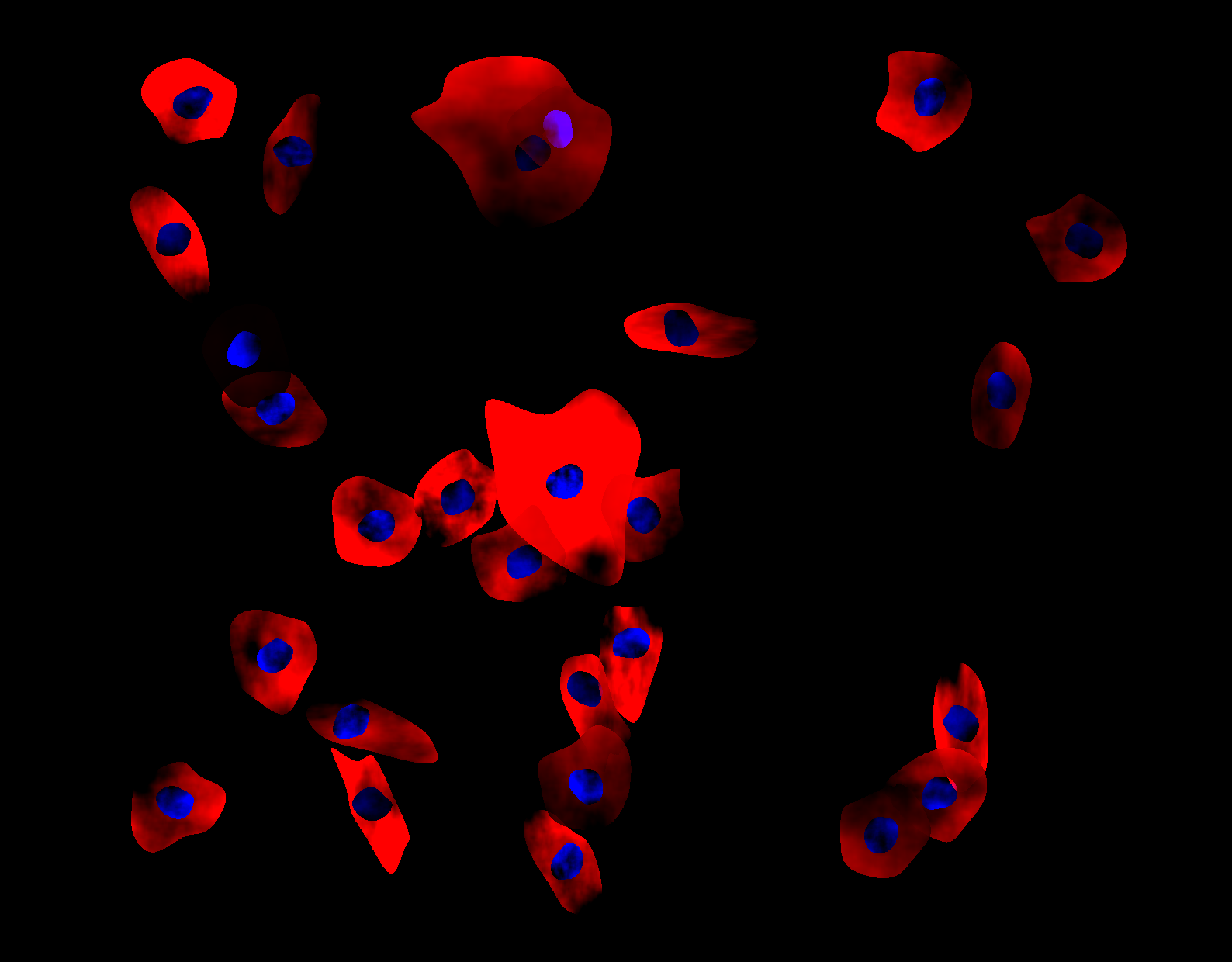

Supplement: Additional file 8 — The zip archive contains all files simulated with SimuCell. These images have been used during the expert observer study. (ZIP 7618.56 kb) [file 12859_2017_1591_MOESM8_ESM.zip › SimuCell - Simulated Images/B cell nuclei/image_41.png]

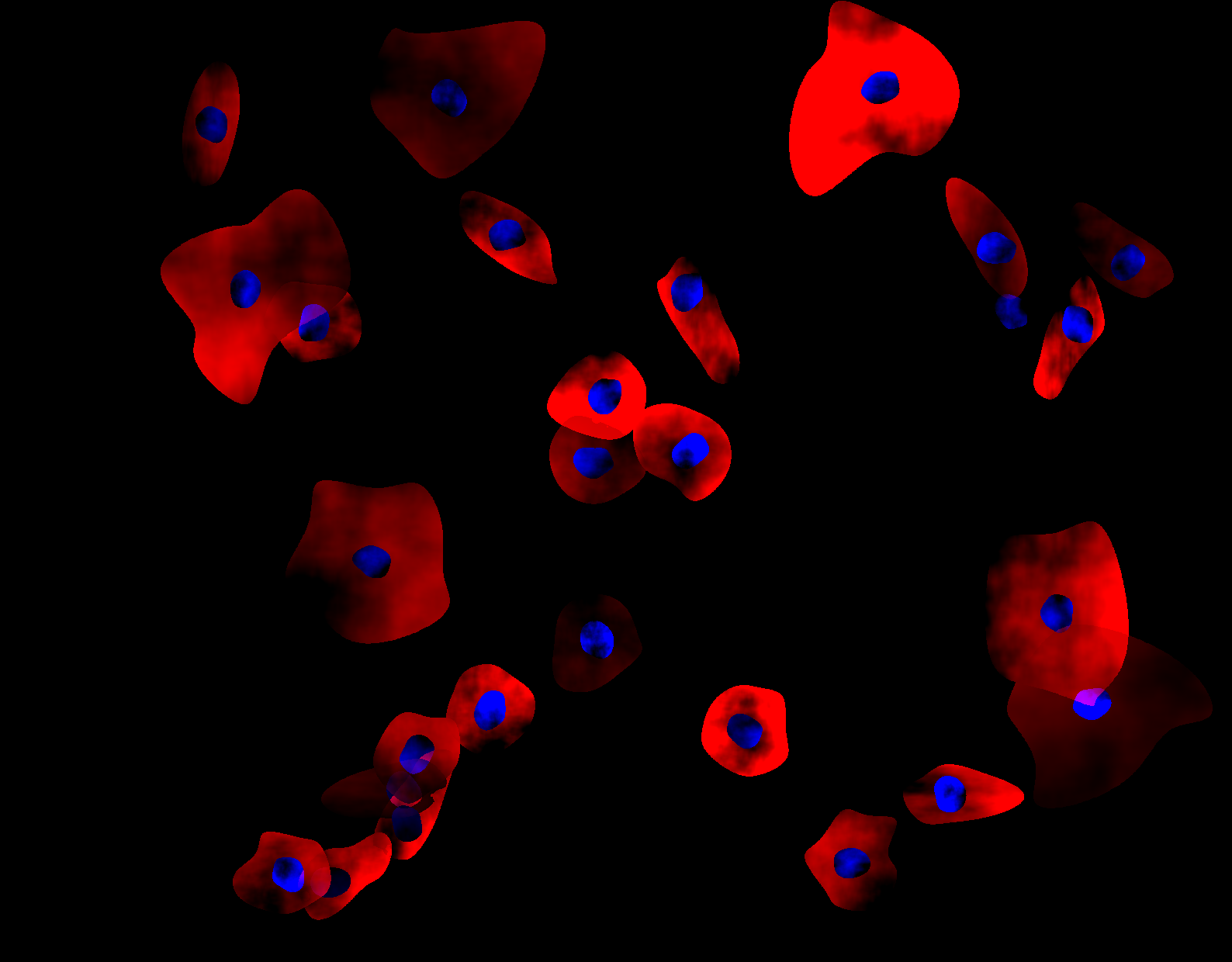

Supplement: Additional file 8 — The zip archive contains all files simulated with SimuCell. These images have been used during the expert observer study. (ZIP 7618.56 kb) [file 12859_2017_1591_MOESM8_ESM.zip › SimuCell - Simulated Images/B cell nuclei/image_51.png]

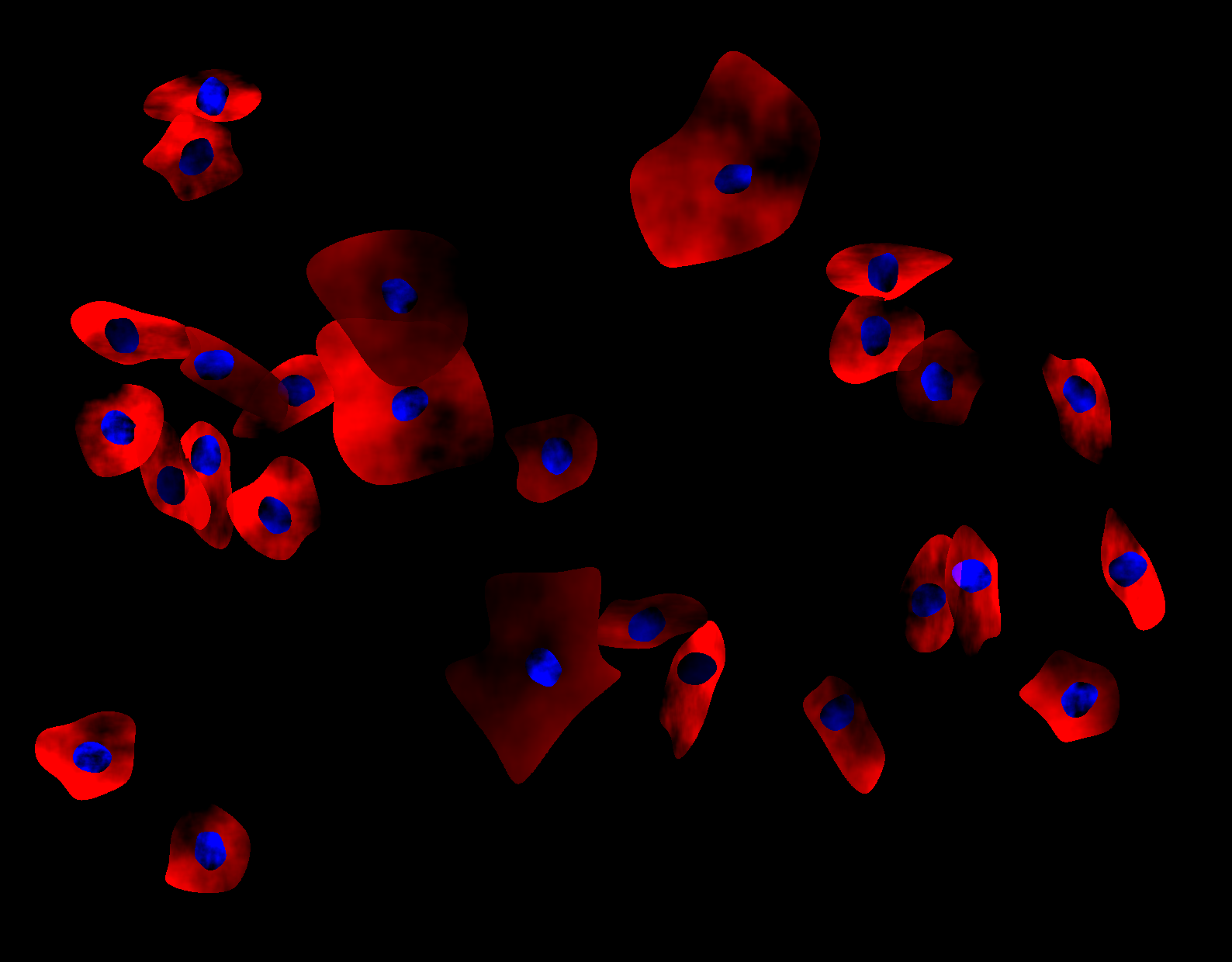

Supplement: Additional file 8 — The zip archive contains all files simulated with SimuCell. These images have been used during the expert observer study. (ZIP 7618.56 kb) [file 12859_2017_1591_MOESM8_ESM.zip › SimuCell - Simulated Images/B cell nuclei/image_71.png]

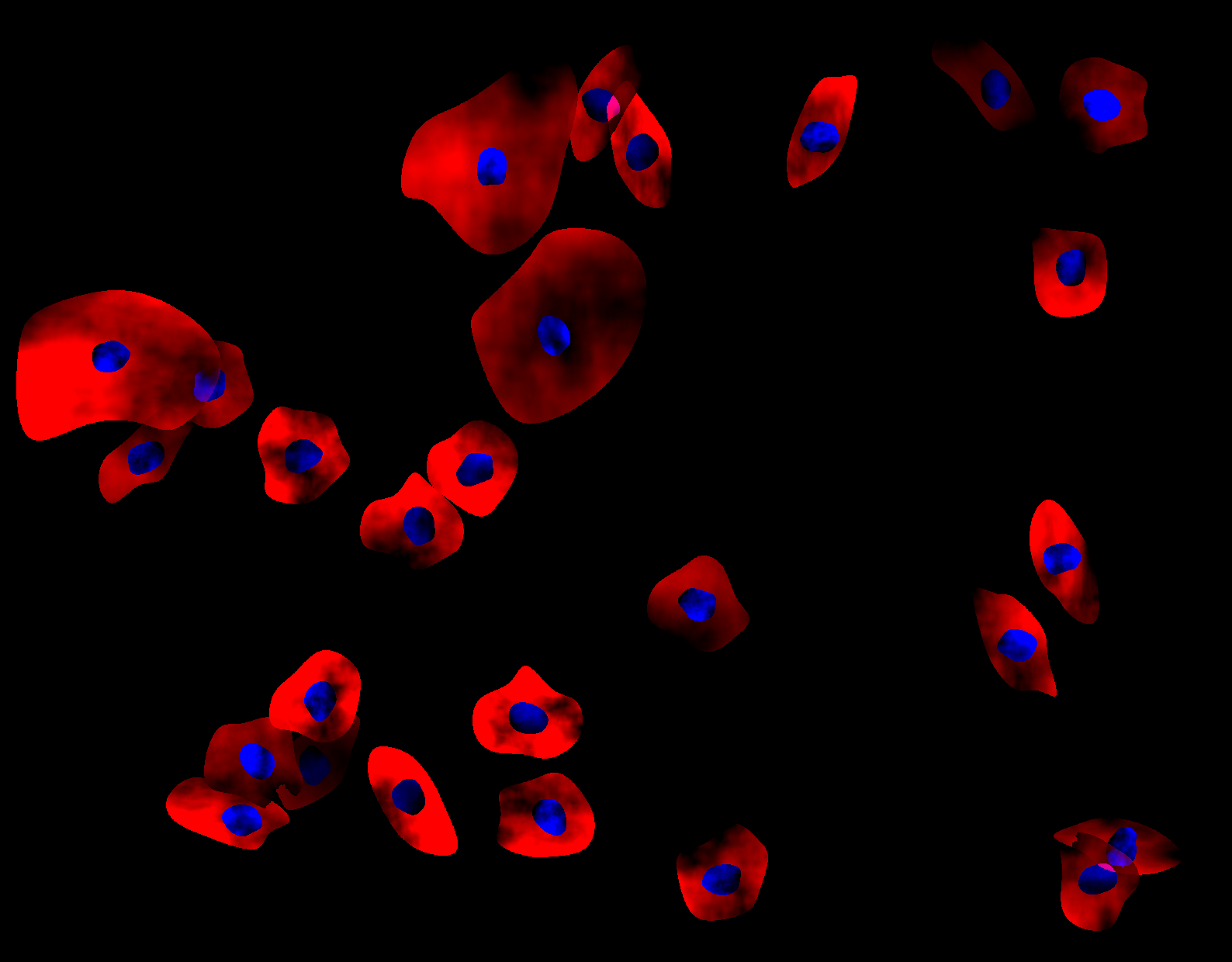

Supplement: Additional file 8 — The zip archive contains all files simulated with SimuCell. These images have been used during the expert observer study. (ZIP 7618.56 kb) [file 12859_2017_1591_MOESM8_ESM.zip › SimuCell - Simulated Images/B cell nuclei/image_81.png]

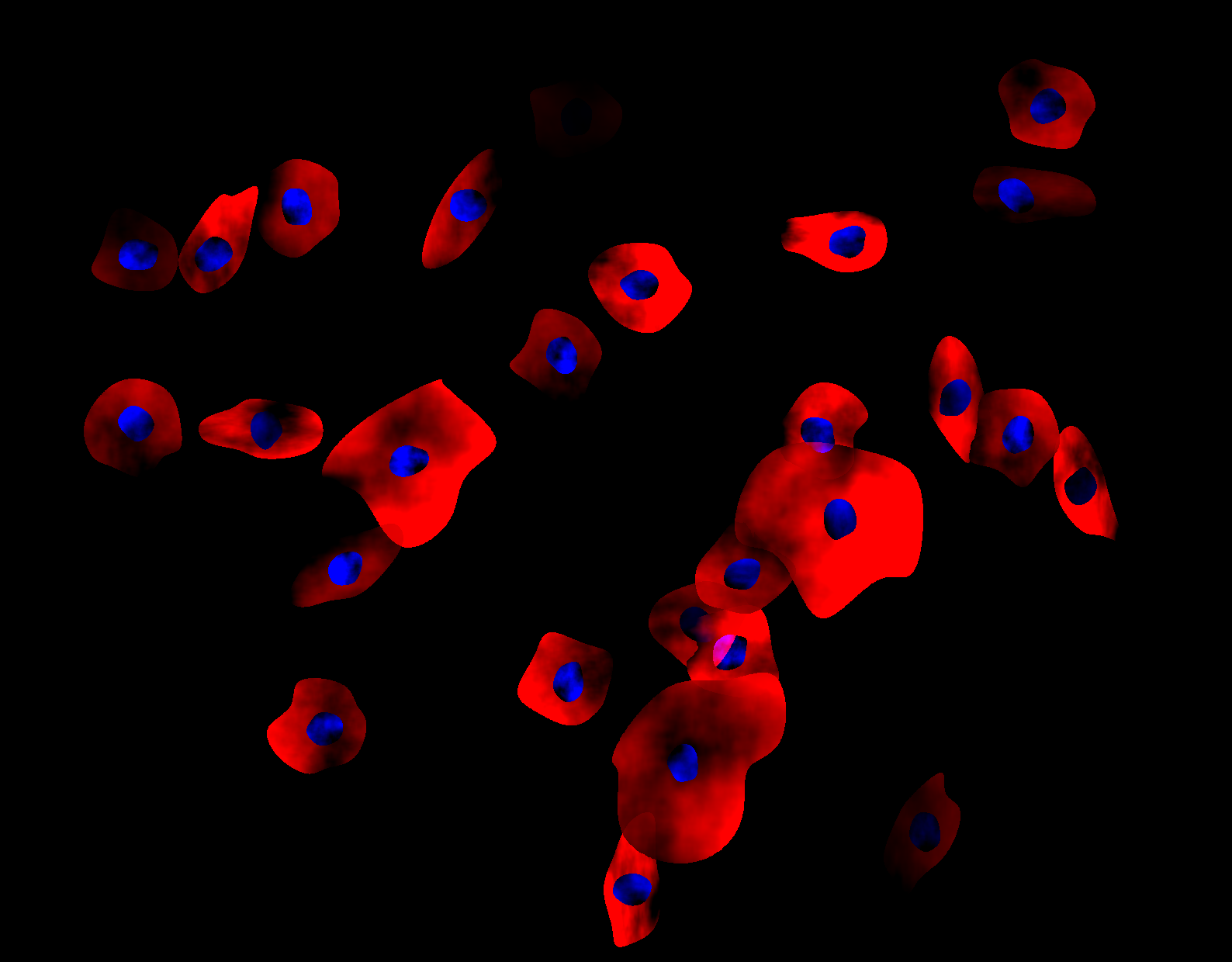

Supplement: Additional file 8 — The zip archive contains all files simulated with SimuCell. These images have been used during the expert observer study. (ZIP 7618.56 kb) [file 12859_2017_1591_MOESM8_ESM.zip › SimuCell - Simulated Images/B cell nuclei/image_91.png]

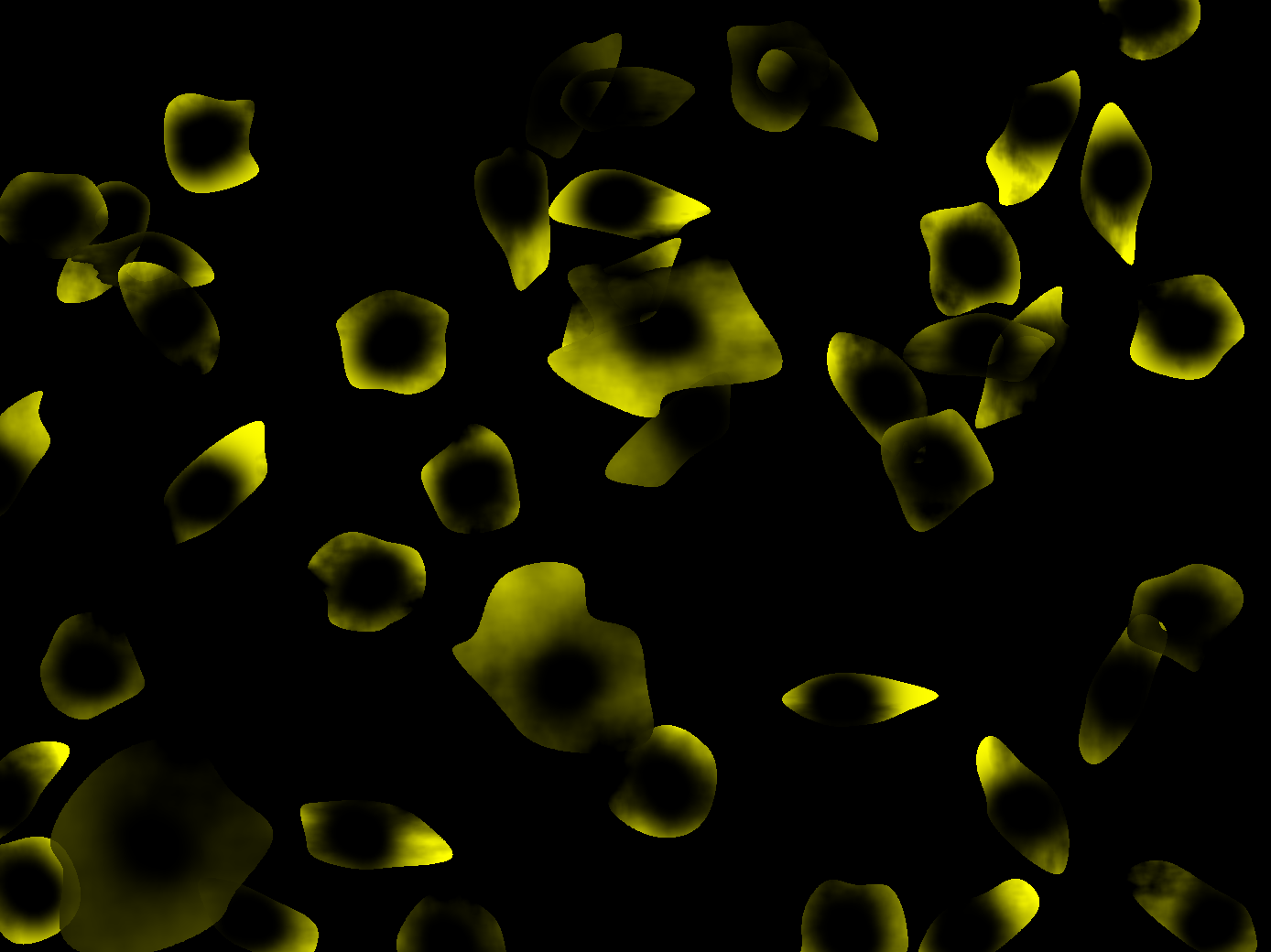

Supplement: Additional file 8 — The zip archive contains all files simulated with SimuCell. These images have been used during the expert observer study. (ZIP 7618.56 kb) [file 12859_2017_1591_MOESM8_ESM.zip › SimuCell - Simulated Images/macrophages/10image_1.png]

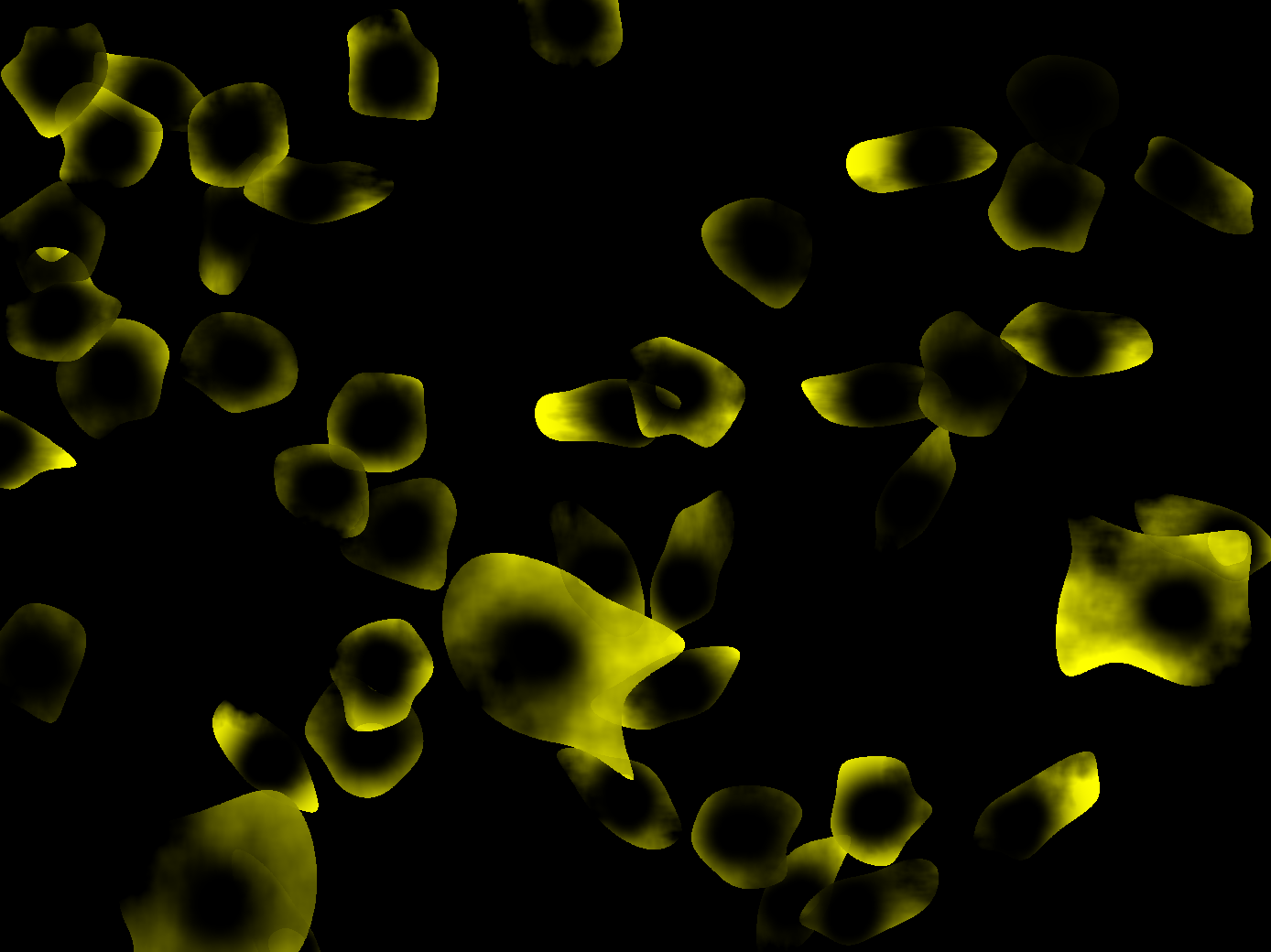

Supplement: Additional file 8 — The zip archive contains all files simulated with SimuCell. These images have been used during the expert observer study. (ZIP 7618.56 kb) [file 12859_2017_1591_MOESM8_ESM.zip › SimuCell - Simulated Images/macrophages/1image_1.png]

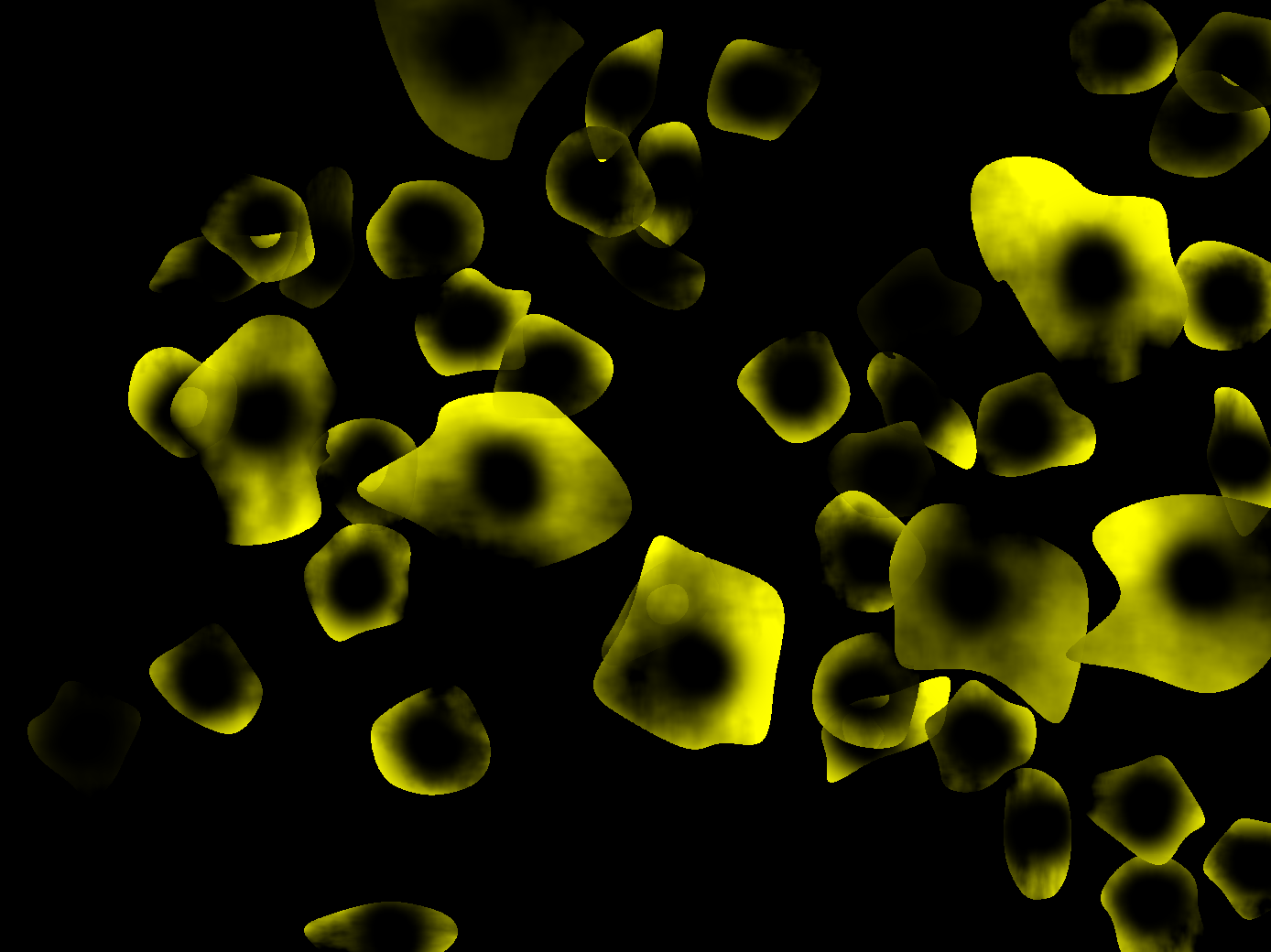

Supplement: Additional file 8 — The zip archive contains all files simulated with SimuCell. These images have been used during the expert observer study. (ZIP 7618.56 kb) [file 12859_2017_1591_MOESM8_ESM.zip › SimuCell - Simulated Images/macrophages/2image_1.png]

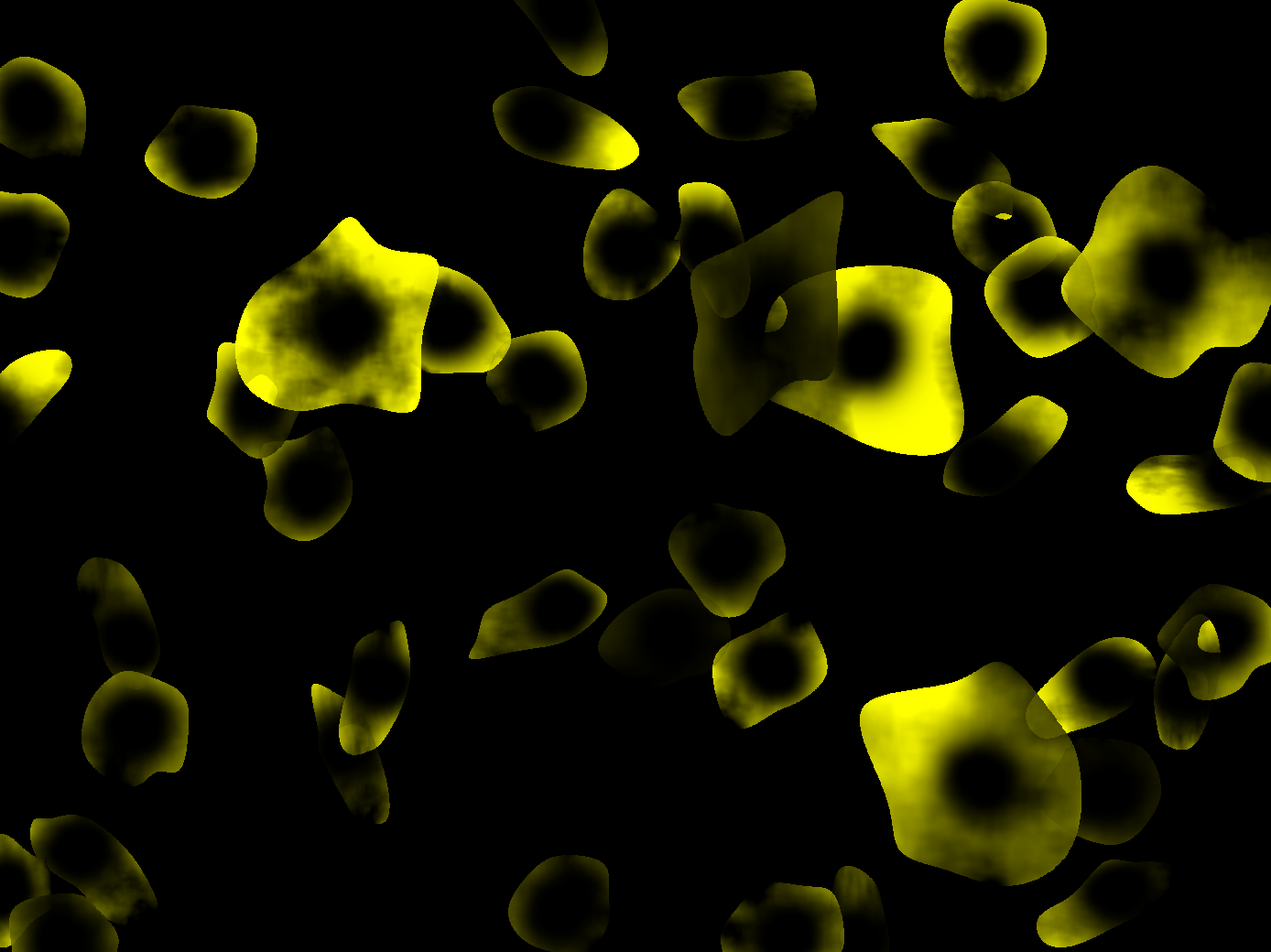

Supplement: Additional file 8 — The zip archive contains all files simulated with SimuCell. These images have been used during the expert observer study. (ZIP 7618.56 kb) [file 12859_2017_1591_MOESM8_ESM.zip › SimuCell - Simulated Images/macrophages/3image_1.png]

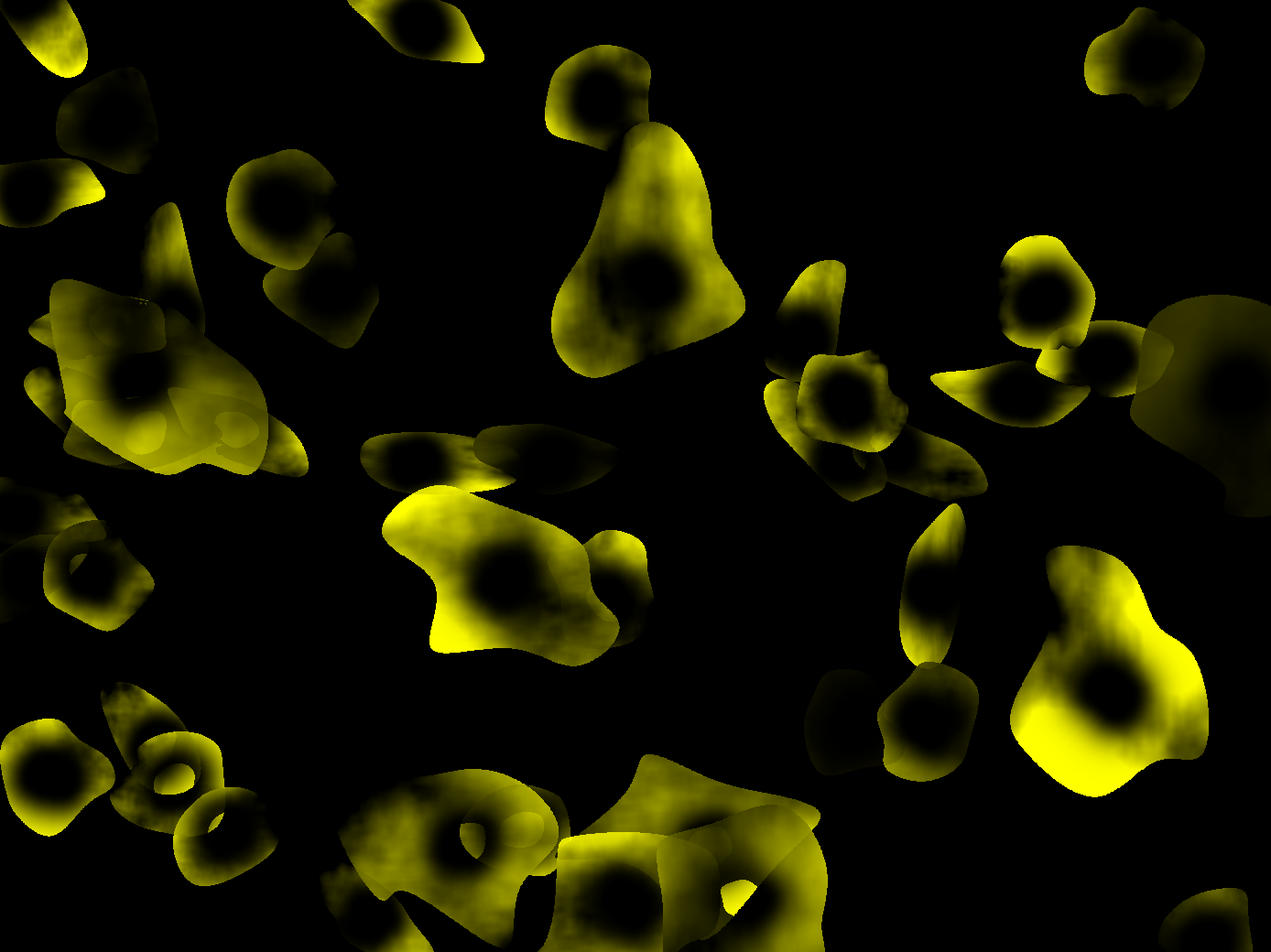

Supplement: Additional file 8 — The zip archive contains all files simulated with SimuCell. These images have been used during the expert observer study. (ZIP 7618.56 kb) [file 12859_2017_1591_MOESM8_ESM.zip › SimuCell - Simulated Images/macrophages/4image_1.png]

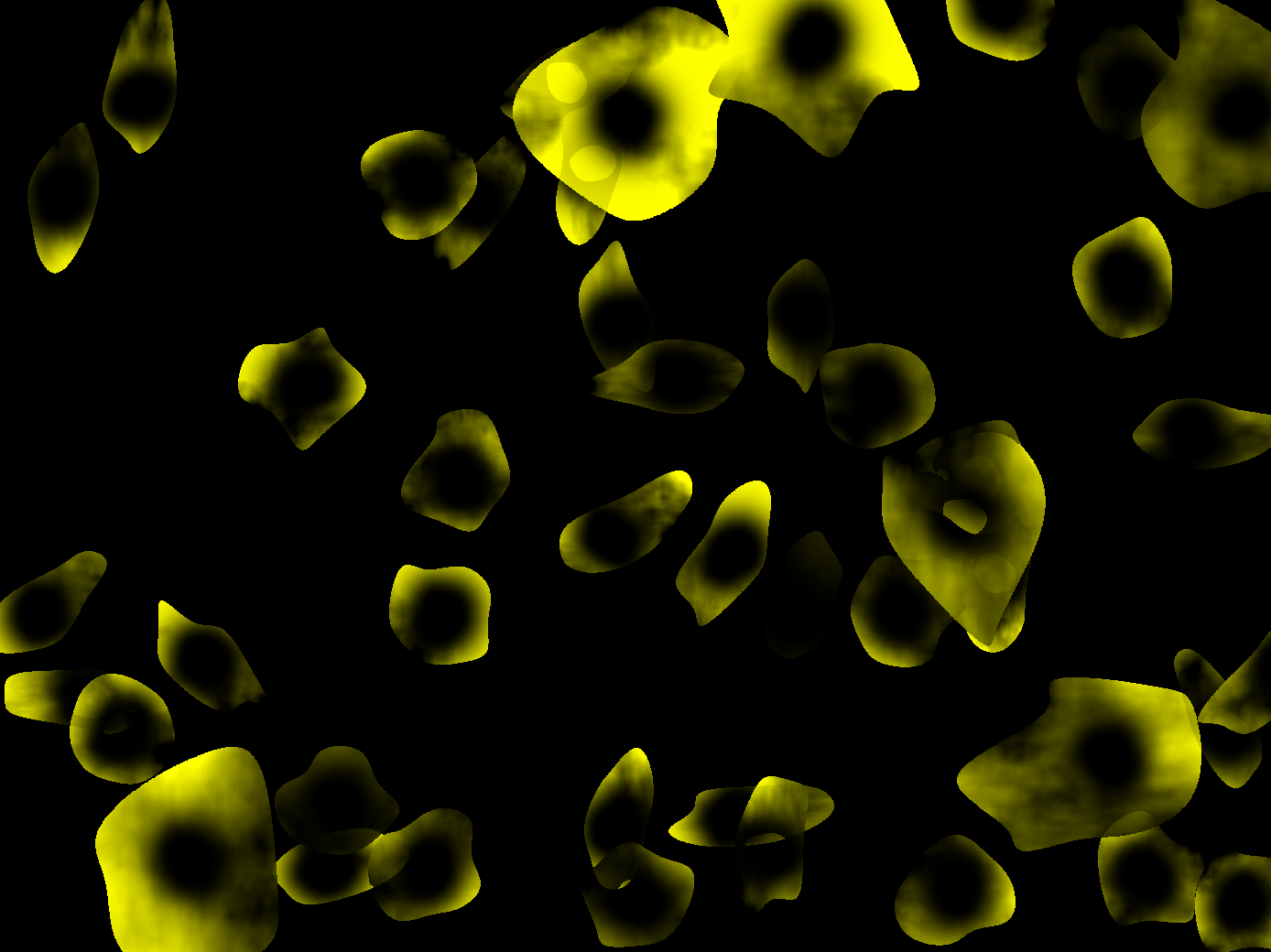

Supplement: Additional file 8 — The zip archive contains all files simulated with SimuCell. These images have been used during the expert observer study. (ZIP 7618.56 kb) [file 12859_2017_1591_MOESM8_ESM.zip › SimuCell - Simulated Images/macrophages/5image_1.png]

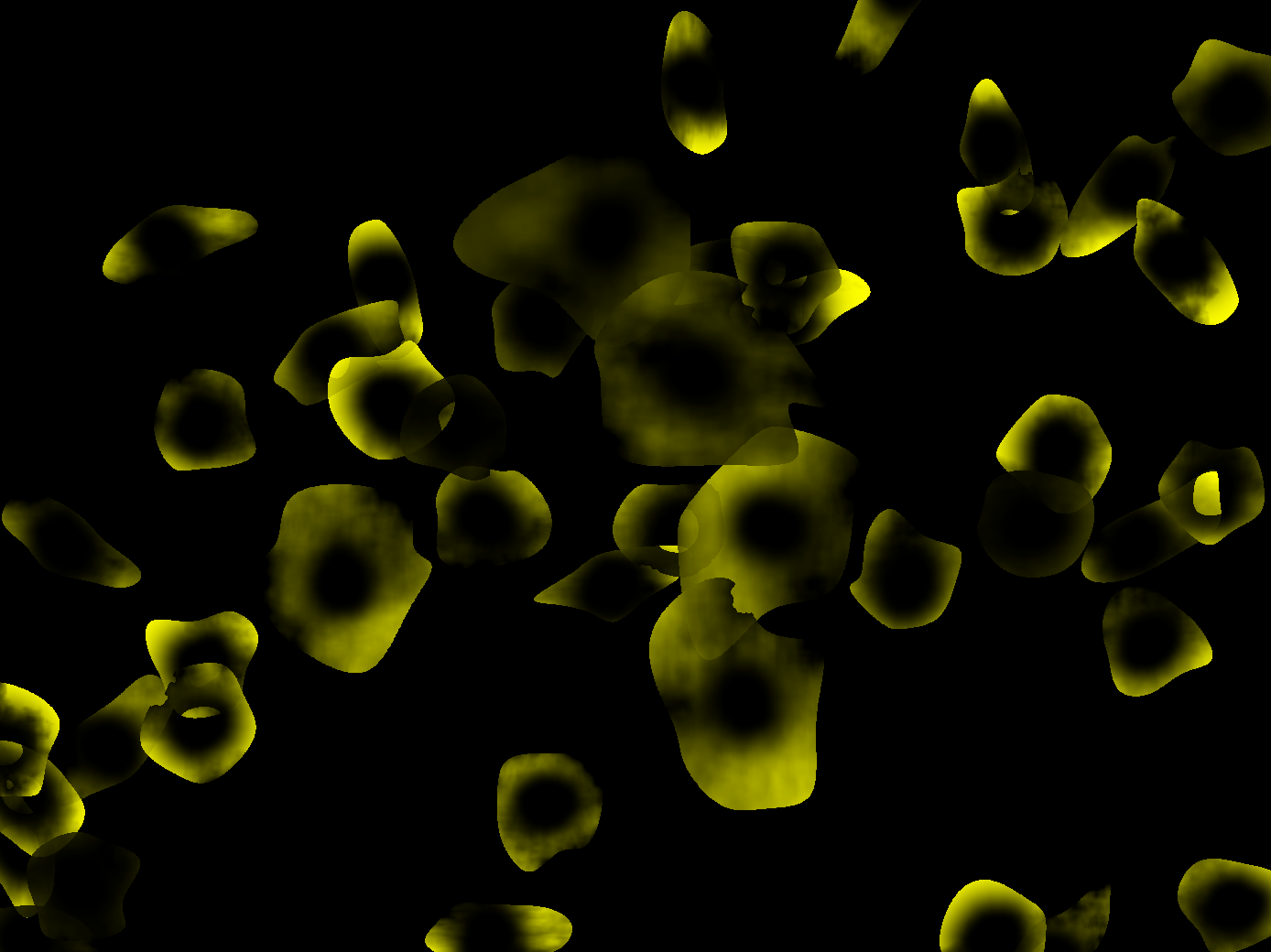

Supplement: Additional file 8 — The zip archive contains all files simulated with SimuCell. These images have been used during the expert observer study. (ZIP 7618.56 kb) [file 12859_2017_1591_MOESM8_ESM.zip › SimuCell - Simulated Images/macrophages/6image_1.png]

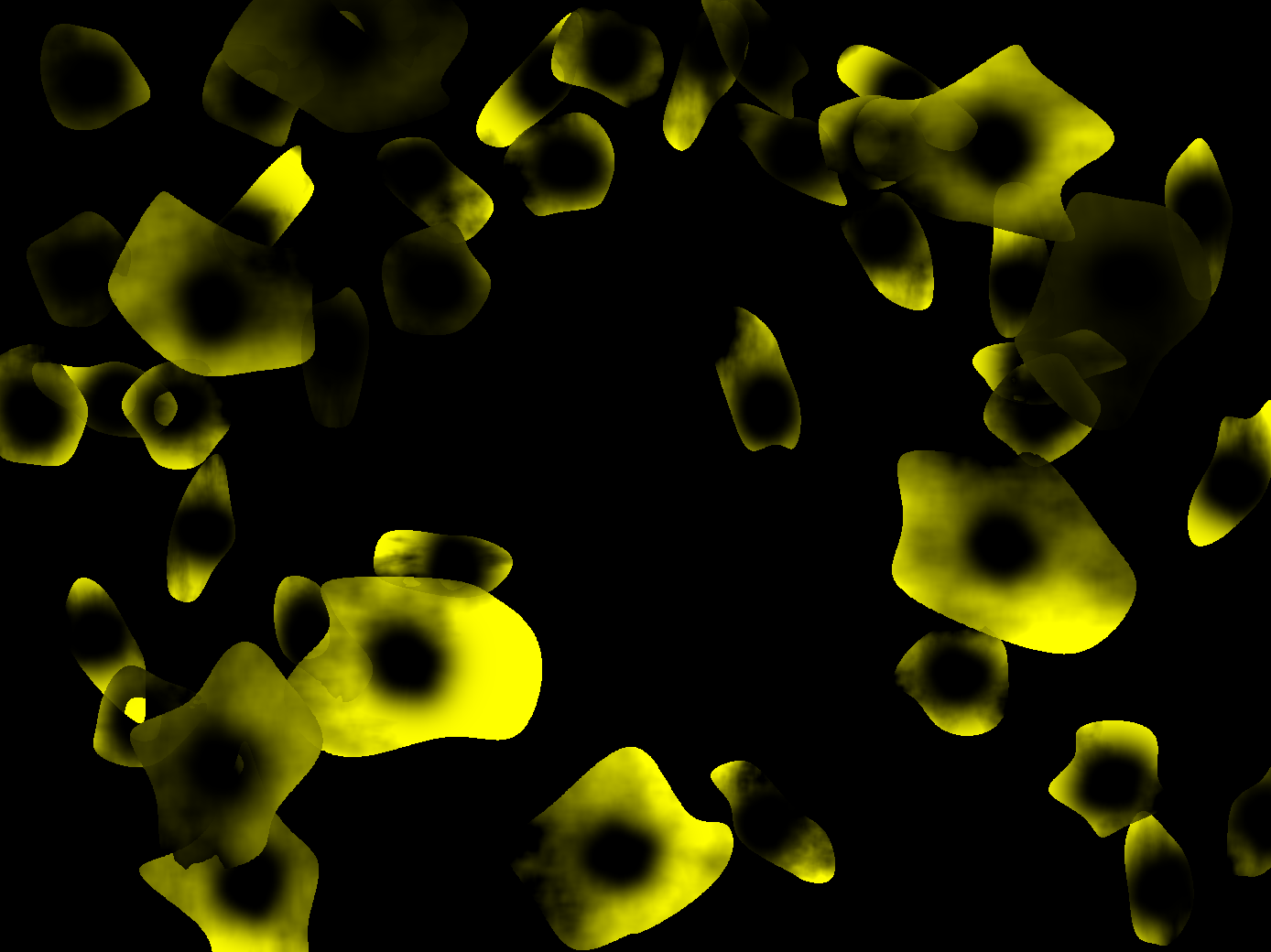

Supplement: Additional file 8 — The zip archive contains all files simulated with SimuCell. These images have been used during the expert observer study. (ZIP 7618.56 kb) [file 12859_2017_1591_MOESM8_ESM.zip › SimuCell - Simulated Images/macrophages/7image_1.png]

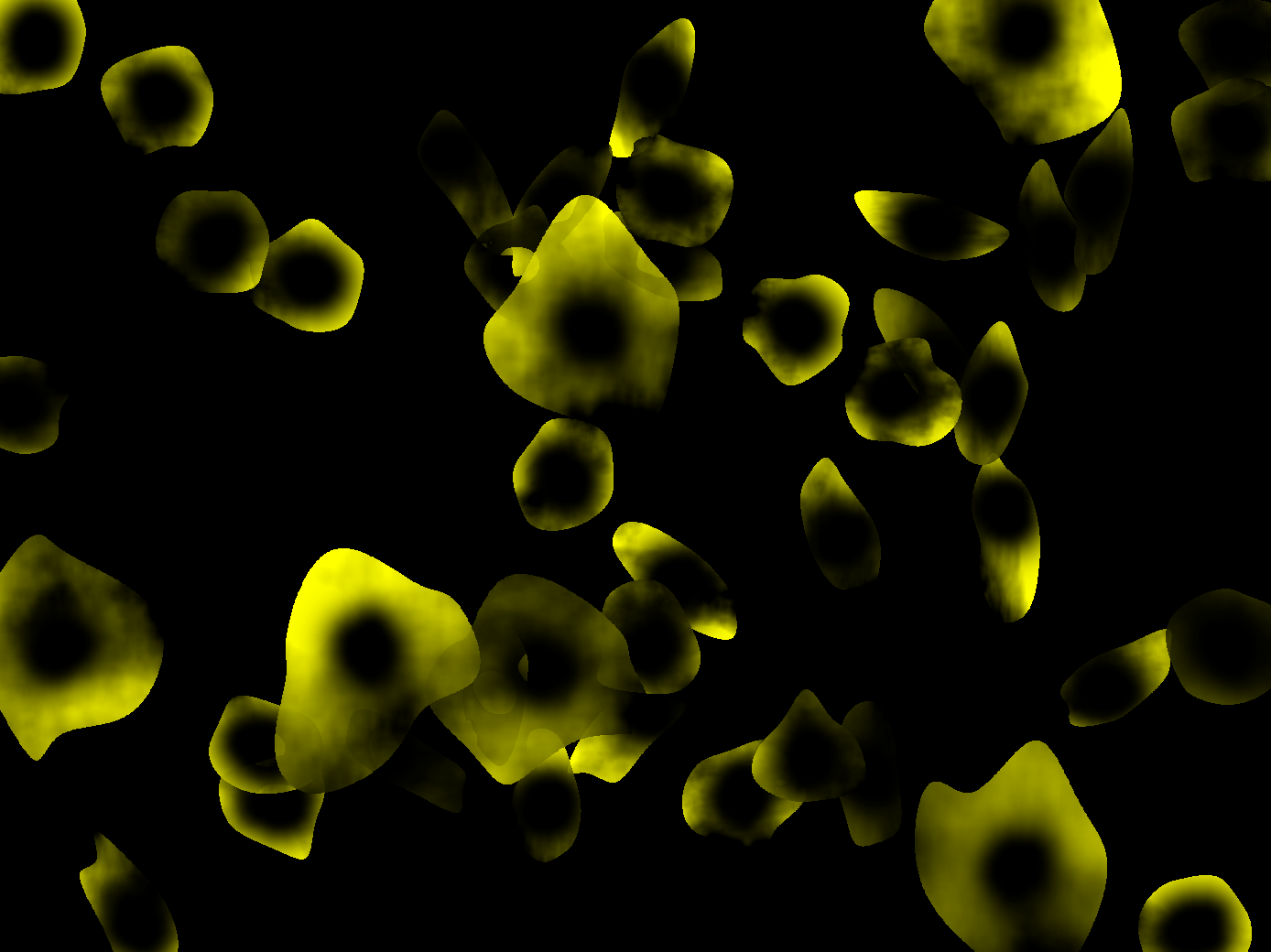

Supplement: Additional file 8 — The zip archive contains all files simulated with SimuCell. These images have been used during the expert observer study. (ZIP 7618.56 kb) [file 12859_2017_1591_MOESM8_ESM.zip › SimuCell - Simulated Images/macrophages/8image_1.png]

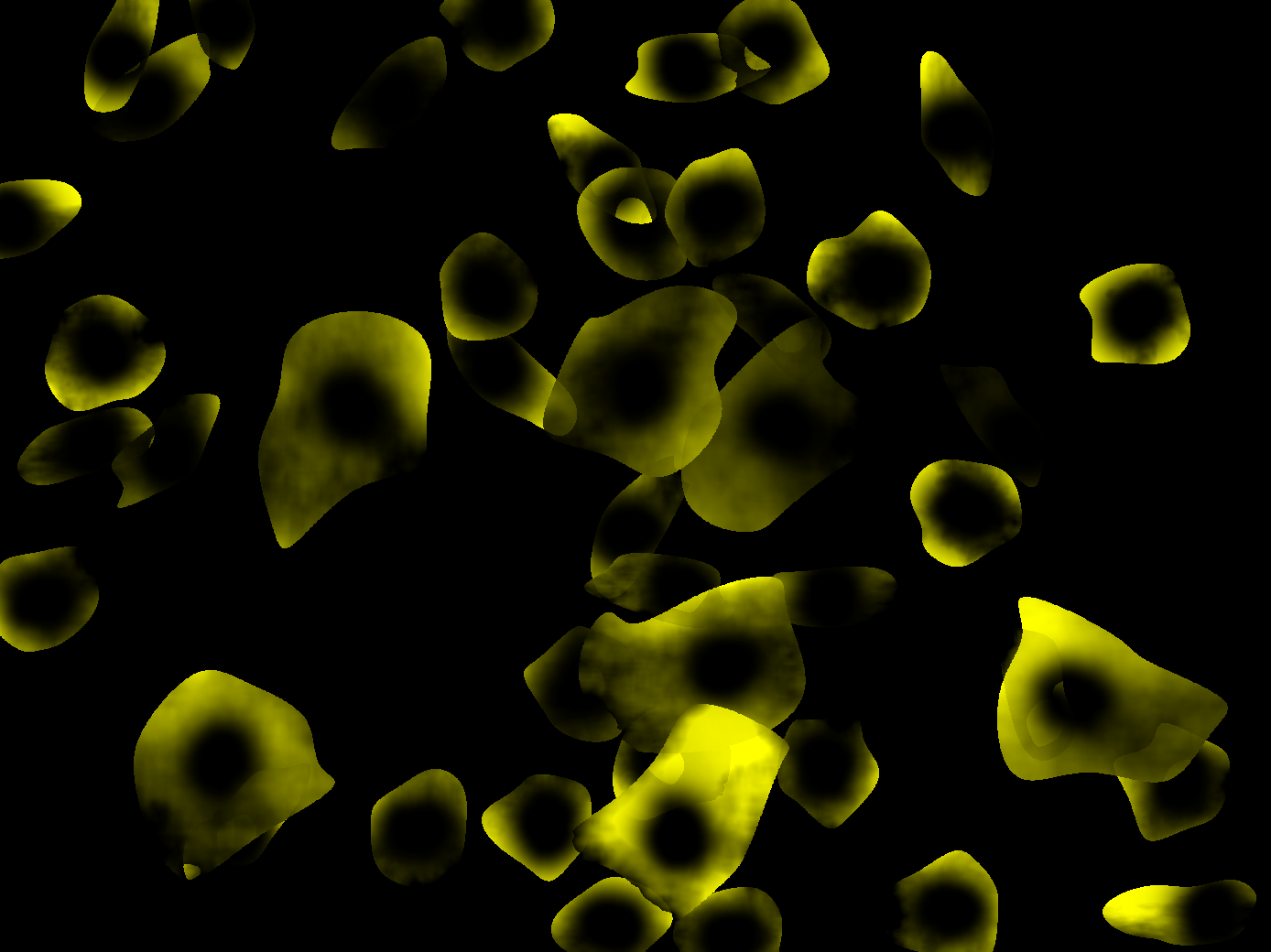

Supplement: Additional file 8 — The zip archive contains all files simulated with SimuCell. These images have been used during the expert observer study. (ZIP 7618.56 kb) [file 12859_2017_1591_MOESM8_ESM.zip › SimuCell - Simulated Images/macrophages/9image_1.png]

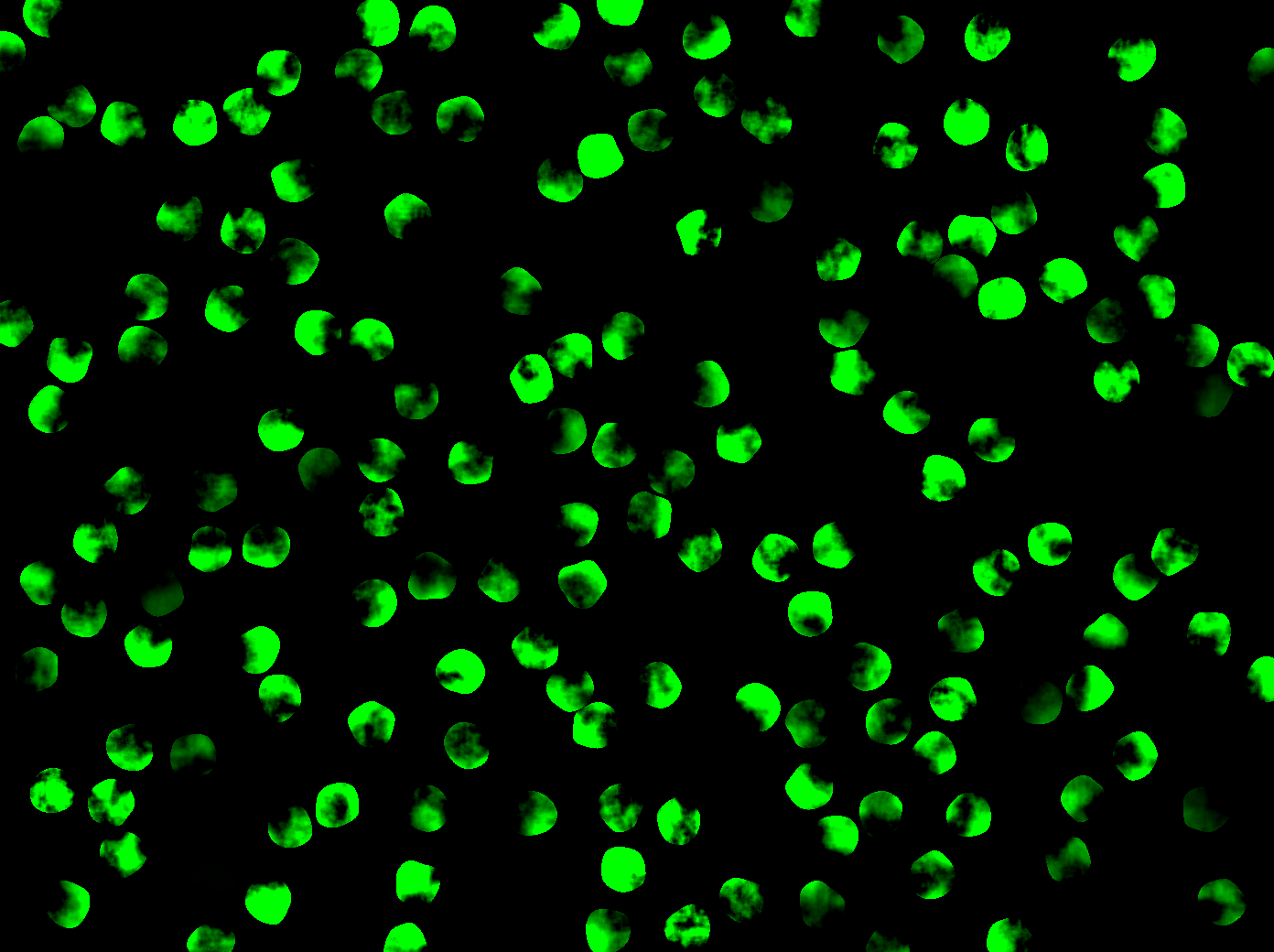

Supplement: Additional file 8 — The zip archive contains all files simulated with SimuCell. These images have been used during the expert observer study. (ZIP 7618.56 kb) [file 12859_2017_1591_MOESM8_ESM.zip › SimuCell - Simulated Images/protoplasts/1image_1.png]

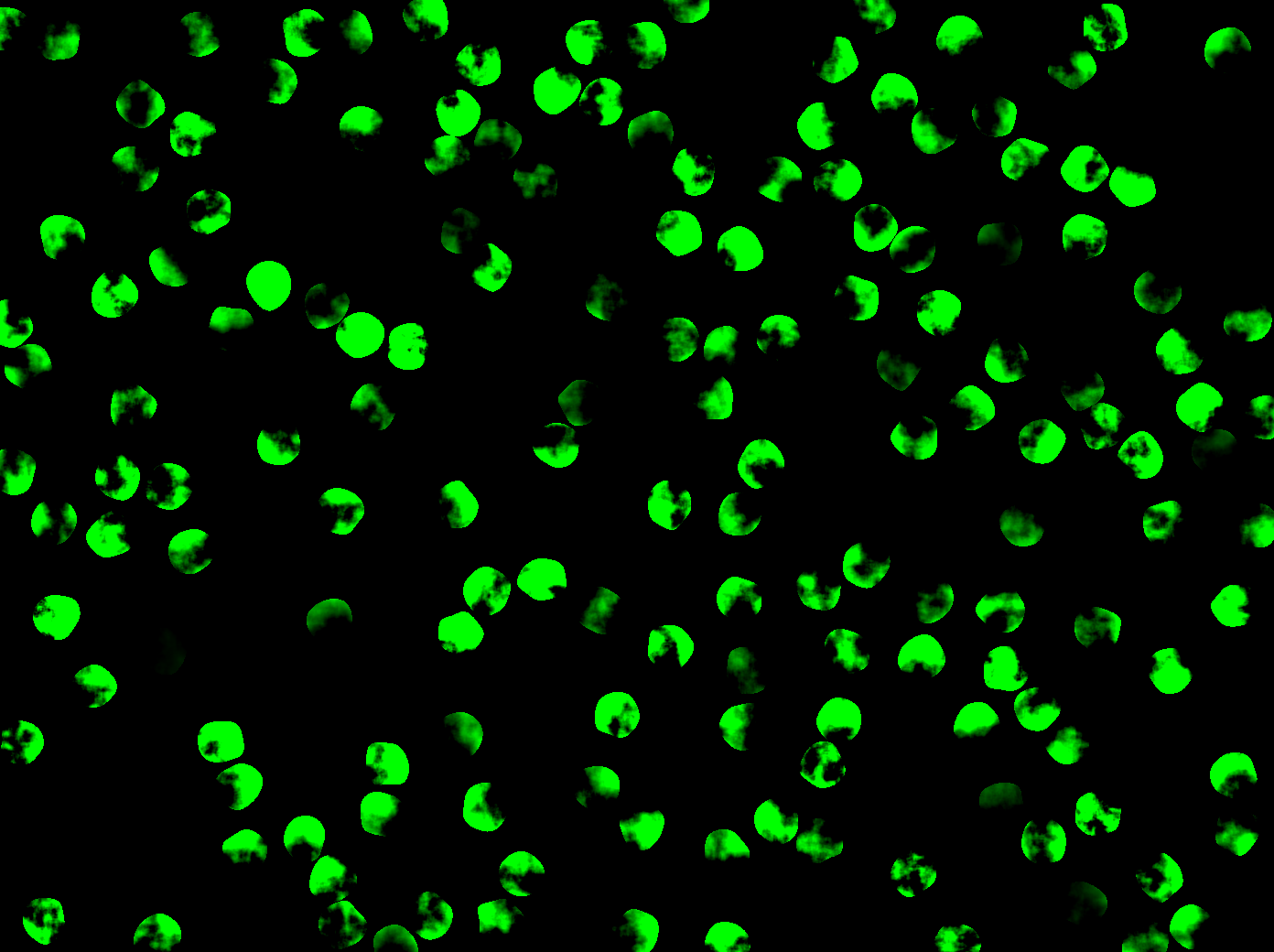

Supplement: Additional file 8 — The zip archive contains all files simulated with SimuCell. These images have been used during the expert observer study. (ZIP 7618.56 kb) [file 12859_2017_1591_MOESM8_ESM.zip › SimuCell - Simulated Images/protoplasts/2image_1.png]

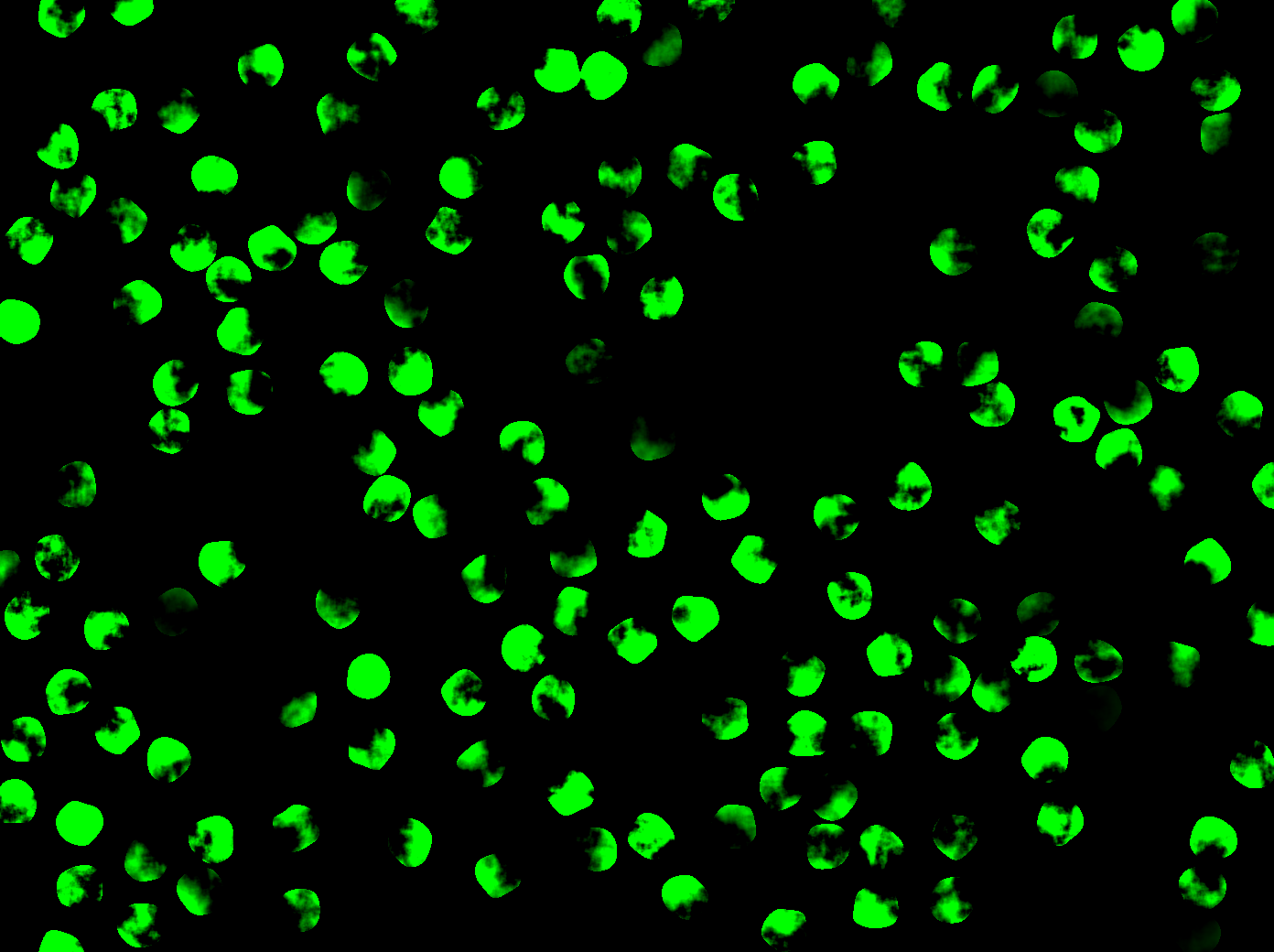

Supplement: Additional file 8 — The zip archive contains all files simulated with SimuCell. These images have been used during the expert observer study. (ZIP 7618.56 kb) [file 12859_2017_1591_MOESM8_ESM.zip › SimuCell - Simulated Images/protoplasts/3image_1.png]

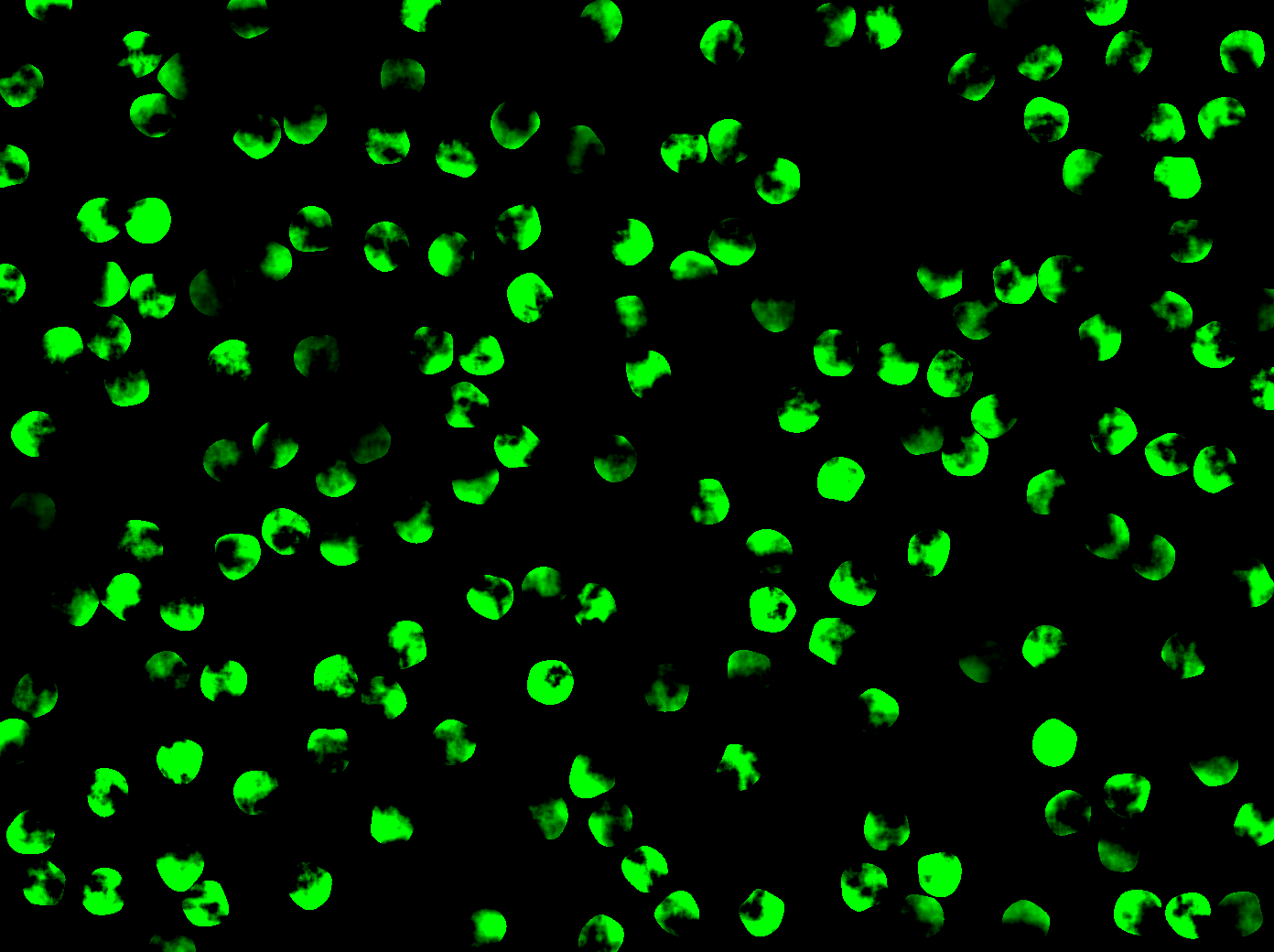

Supplement: Additional file 8 — The zip archive contains all files simulated with SimuCell. These images have been used during the expert observer study. (ZIP 7618.56 kb) [file 12859_2017_1591_MOESM8_ESM.zip › SimuCell - Simulated Images/protoplasts/4image_1.png]

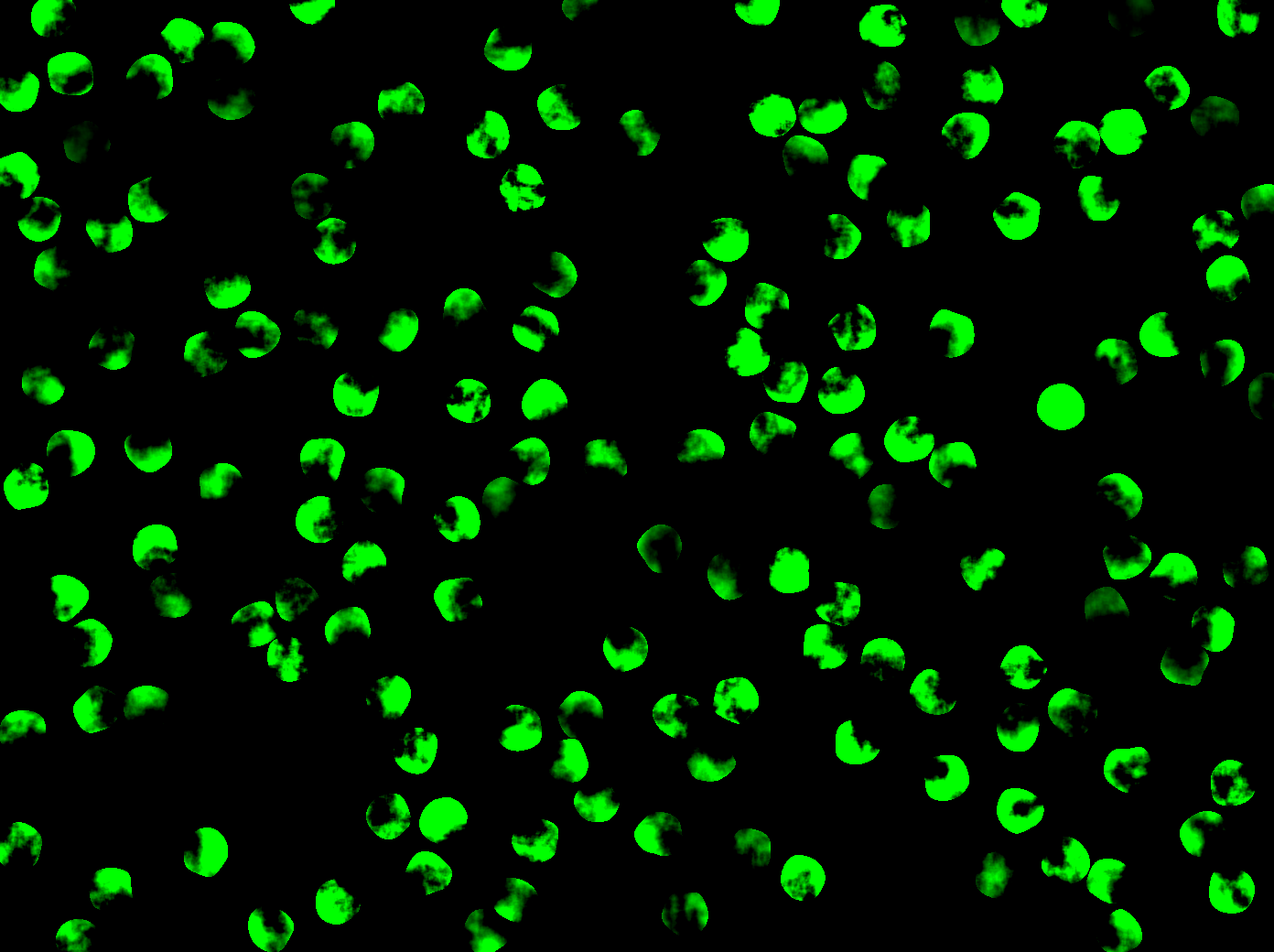

Supplement: Additional file 8 — The zip archive contains all files simulated with SimuCell. These images have been used during the expert observer study. (ZIP 7618.56 kb) [file 12859_2017_1591_MOESM8_ESM.zip › SimuCell - Simulated Images/protoplasts/5image_1.png]

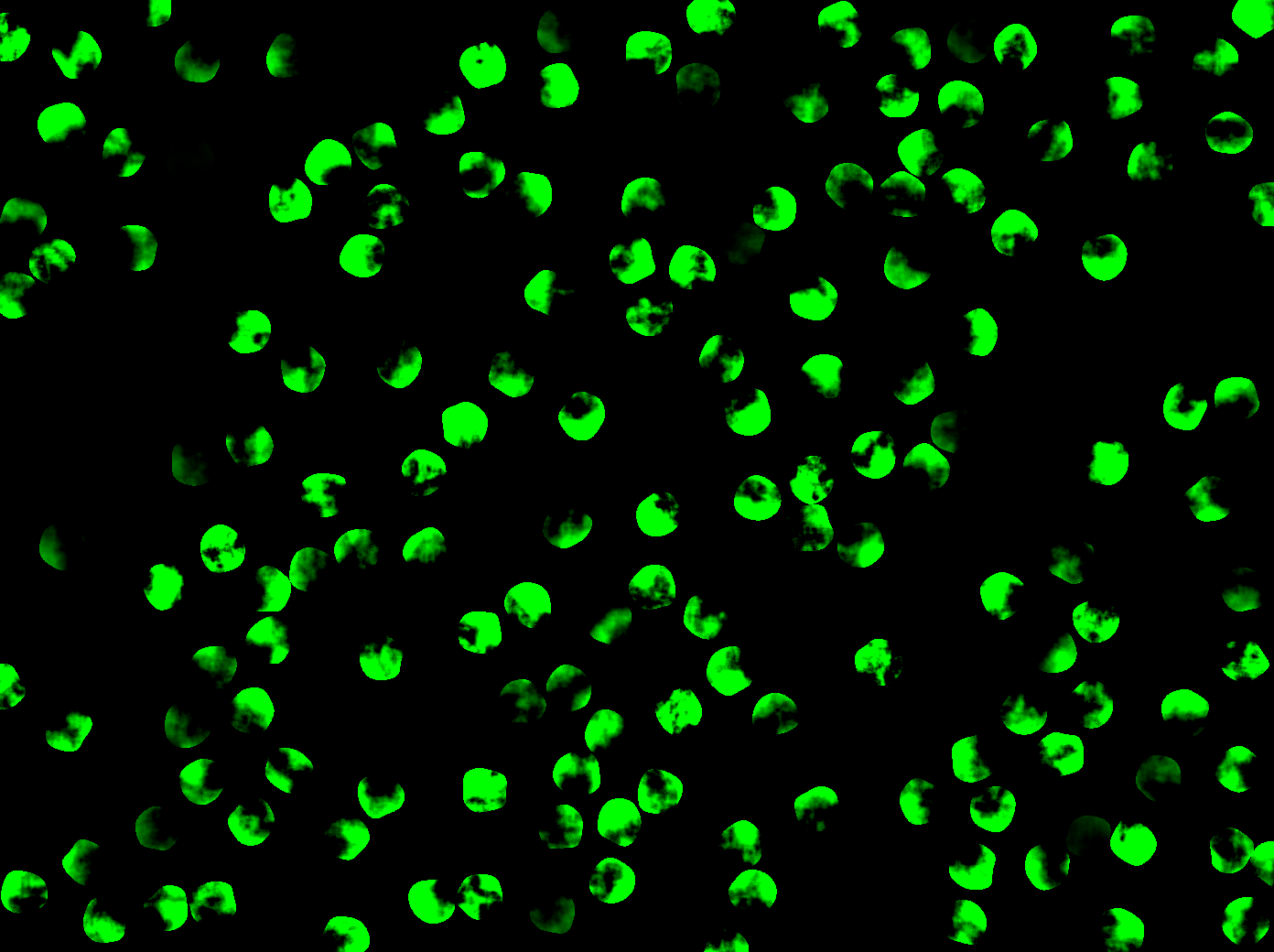

Supplement: Additional file 8 — The zip archive contains all files simulated with SimuCell. These images have been used during the expert observer study. (ZIP 7618.56 kb) [file 12859_2017_1591_MOESM8_ESM.zip › SimuCell - Simulated Images/protoplasts/6image_1.png]

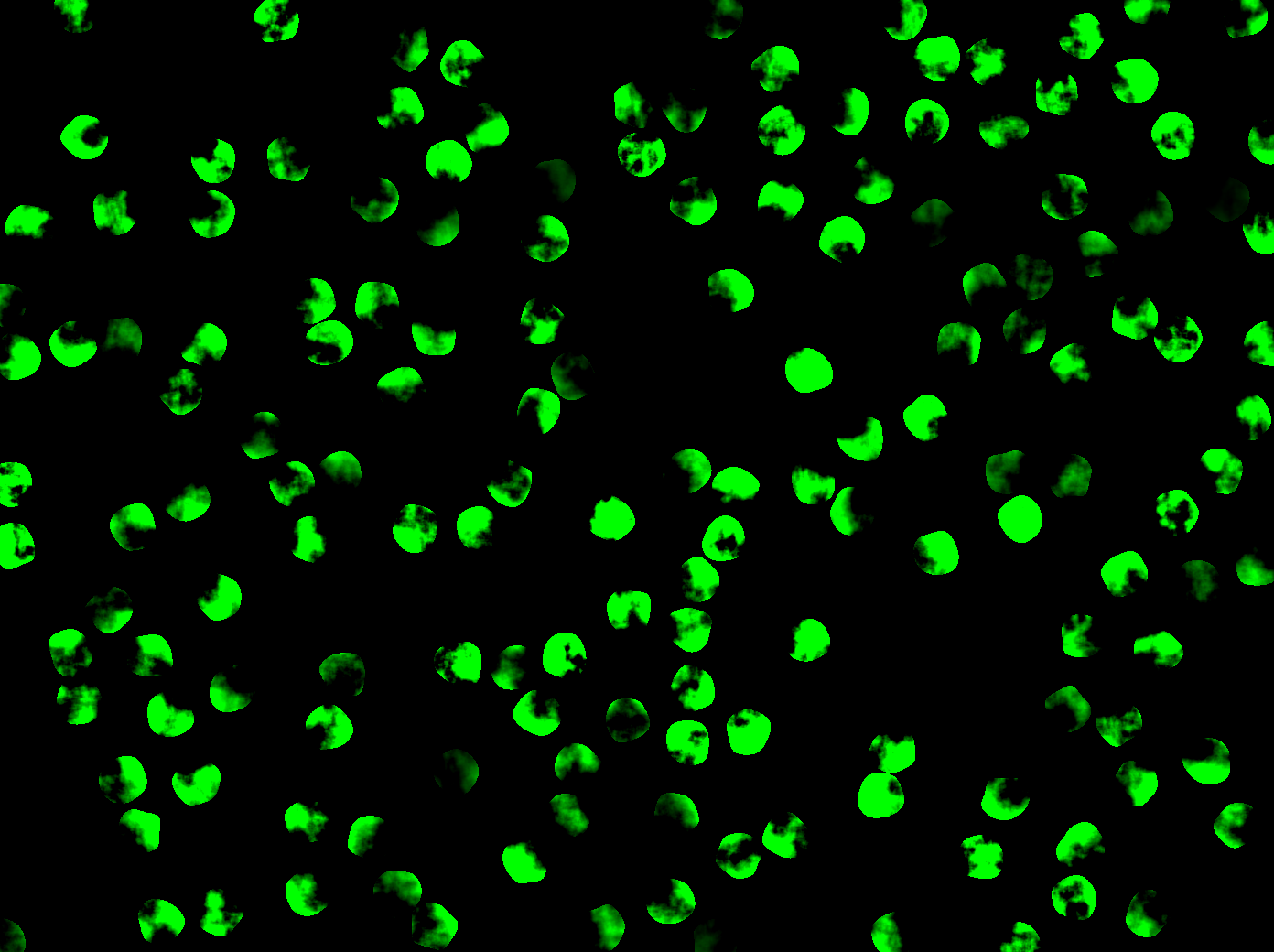

Supplement: Additional file 8 — The zip archive contains all files simulated with SimuCell. These images have been used during the expert observer study. (ZIP 7618.56 kb) [file 12859_2017_1591_MOESM8_ESM.zip › SimuCell - Simulated Images/protoplasts/7image_1.png]

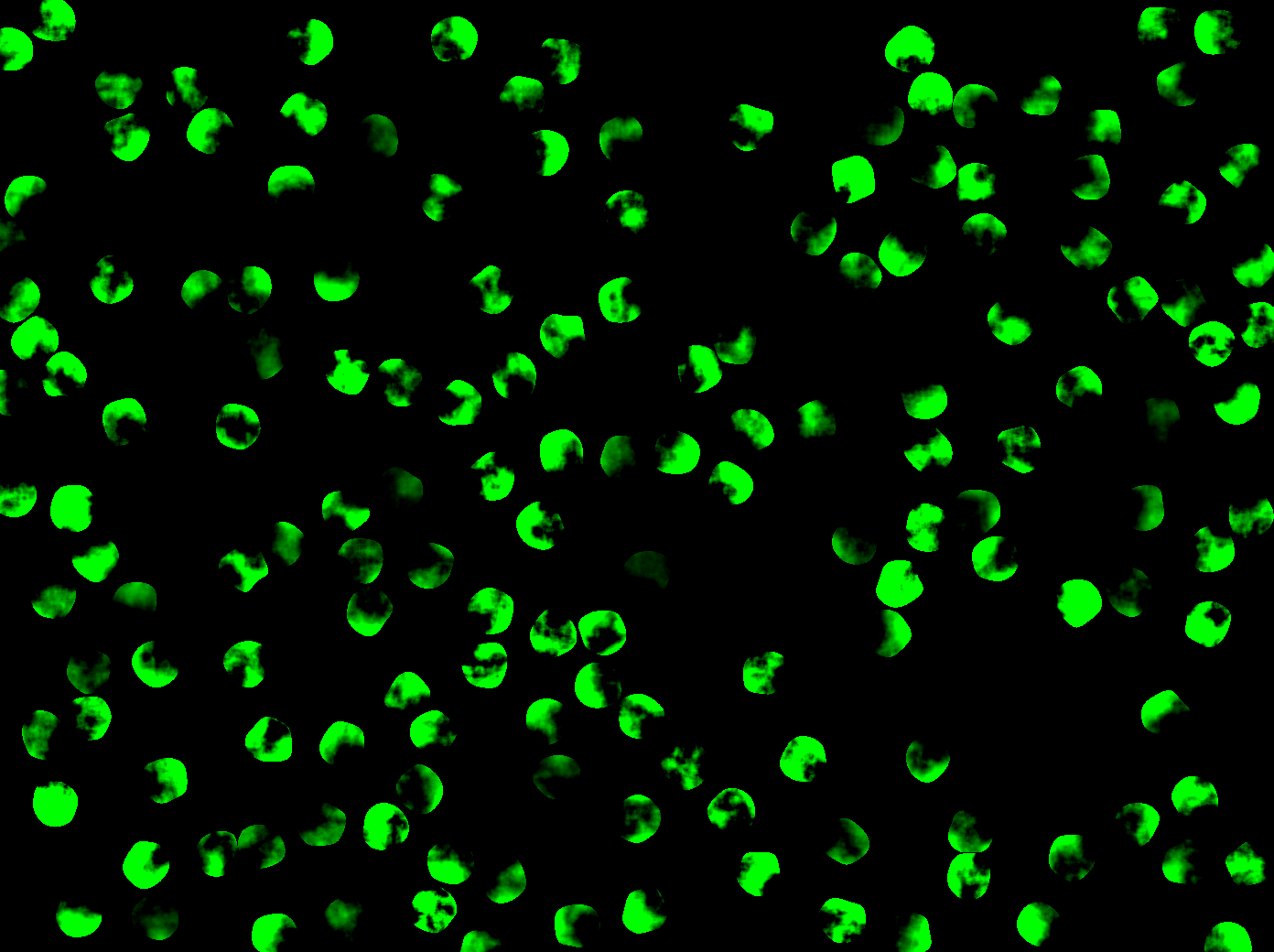

Supplement: Additional file 8 — The zip archive contains all files simulated with SimuCell. These images have been used during the expert observer study. (ZIP 7618.56 kb) [file 12859_2017_1591_MOESM8_ESM.zip › SimuCell - Simulated Images/protoplasts/8image_1.png]

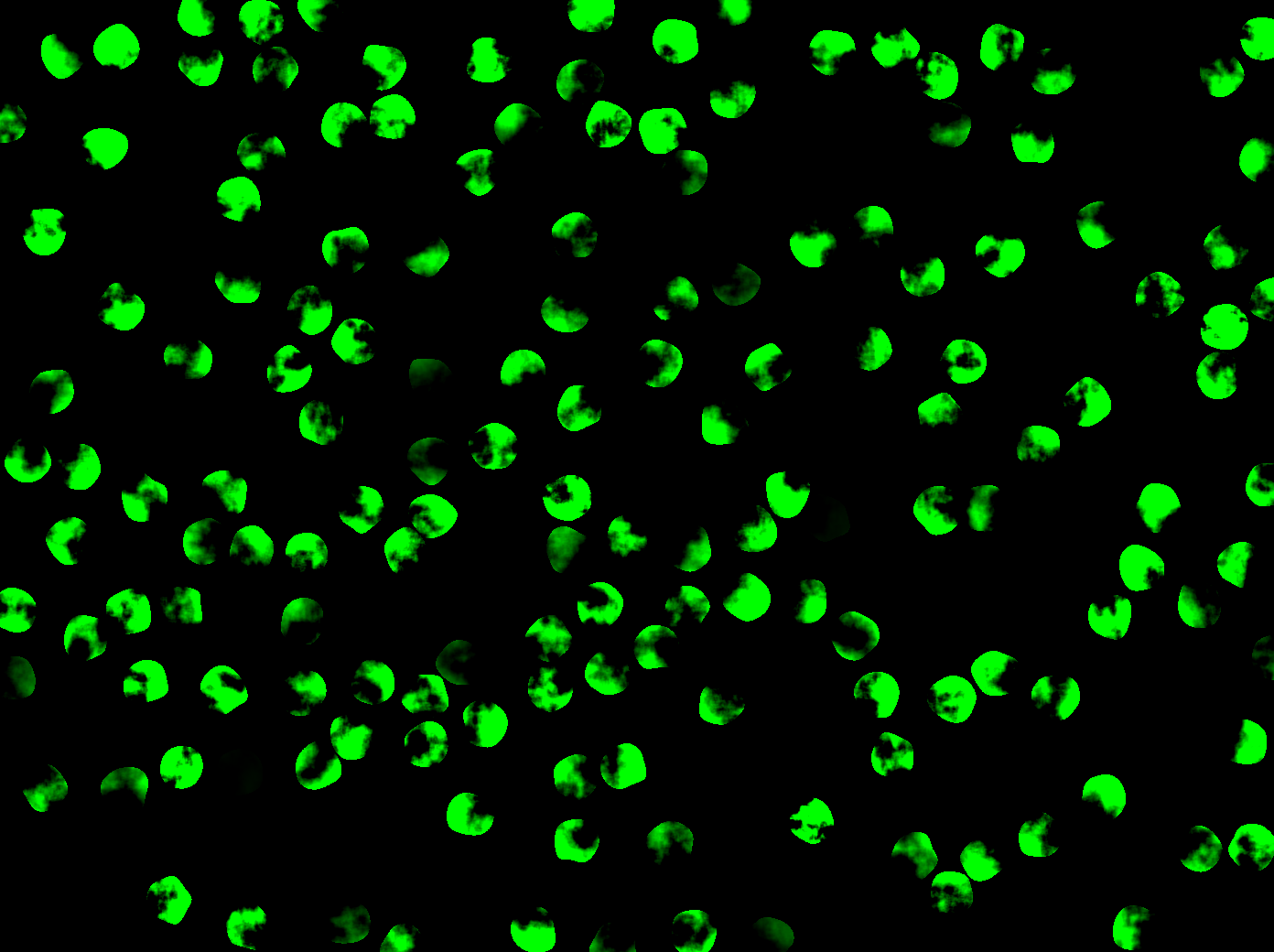

Supplement: Additional file 8 — The zip archive contains all files simulated with SimuCell. These images have been used during the expert observer study. (ZIP 7618.56 kb) [file 12859_2017_1591_MOESM8_ESM.zip › SimuCell - Simulated Images/protoplasts/9image_1.png]

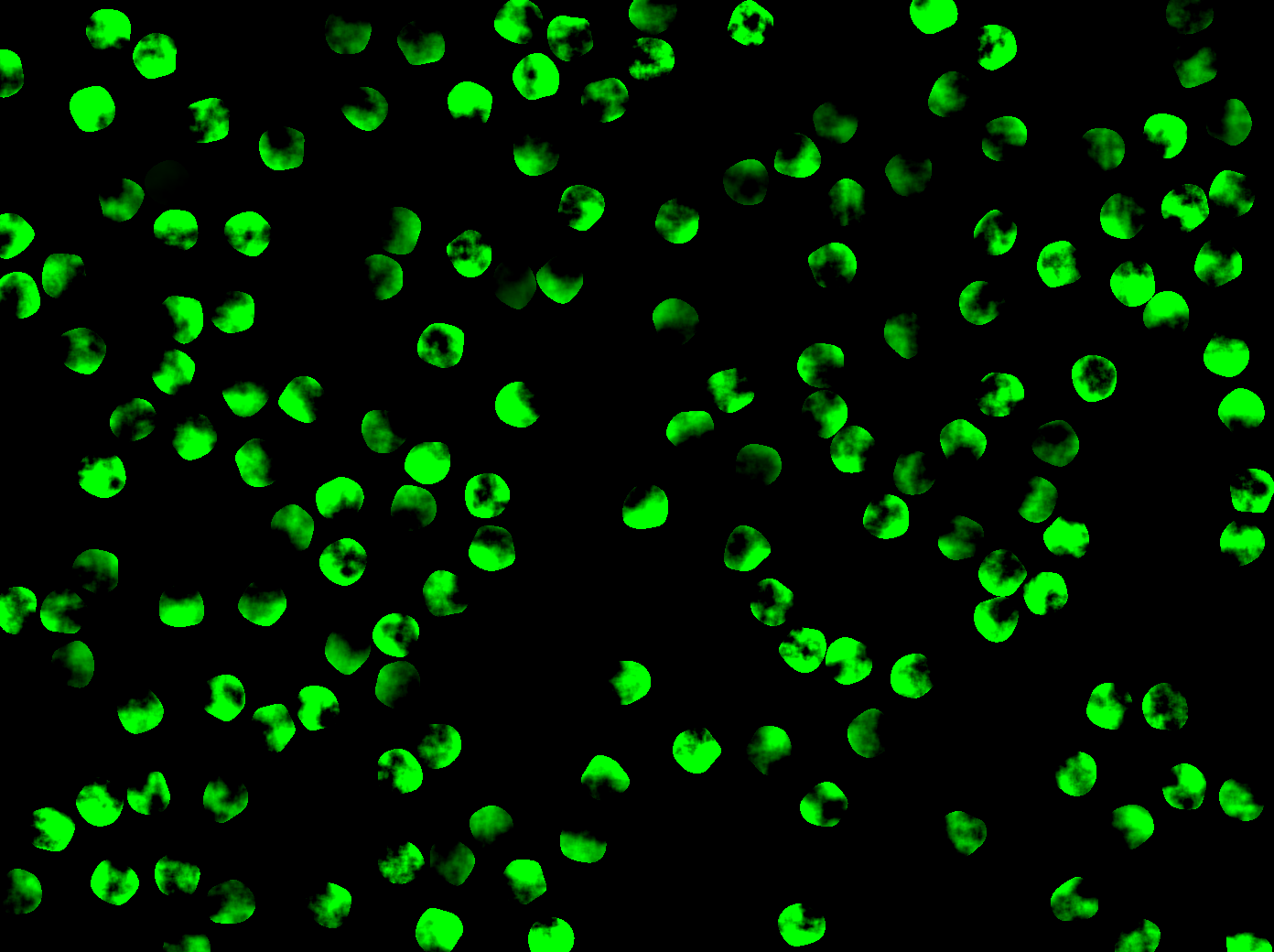

Supplement: Additional file 8 — The zip archive contains all files simulated with SimuCell. These images have been used during the expert observer study. (ZIP 7618.56 kb) [file 12859_2017_1591_MOESM8_ESM.zip › SimuCell - Simulated Images/protoplasts/image_1.png]
