# Supplementary figures and images for: Using simulated fluorescence cell micrographs for the evaluation of cell image segmentation algorithms (part 5 of 6)
Source: BMC Bioinformatics. 2017 Mar 18;18:176. doi: 10.1186/s12859-017-1591-2 (PMC5357336; doi:10.1186/s12859-017-1591-2)

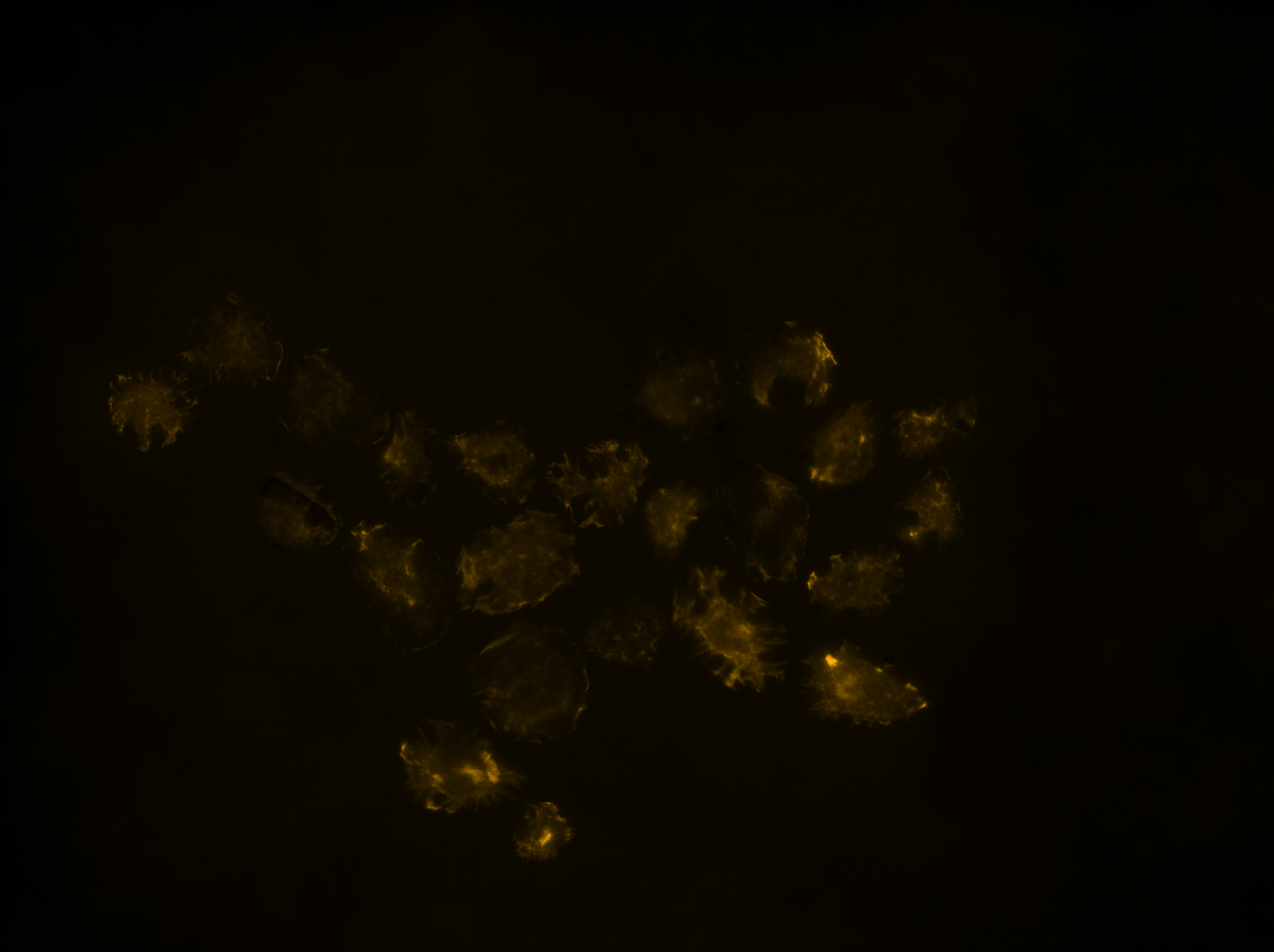

Supplement: Additional file 6 — The zip archive contains simulated images showing B cell nuclei and cytoskeleton with corresponding ground truth. (ZIP 119808 kb) [file 12859_2017_1591_MOESM6_ESM.zip › simulated B cells/cytoskeleton/not touching/cell014.png]

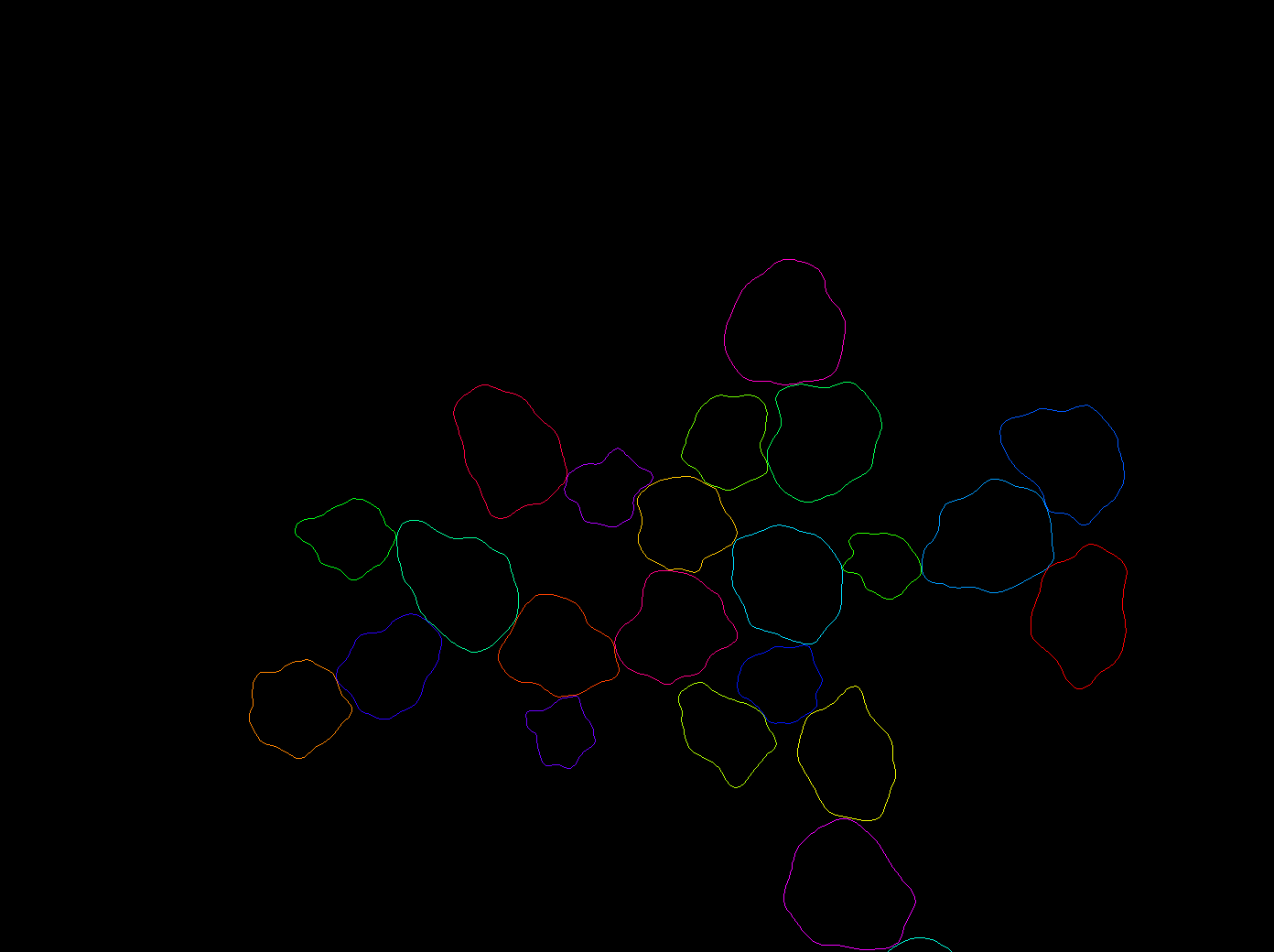

Supplement: Additional file 6 — The zip archive contains simulated images showing B cell nuclei and cytoskeleton with corresponding ground truth. (ZIP 119808 kb) [file 12859_2017_1591_MOESM6_ESM.zip › simulated B cells/cytoskeleton/not touching/cell015 gt.png]

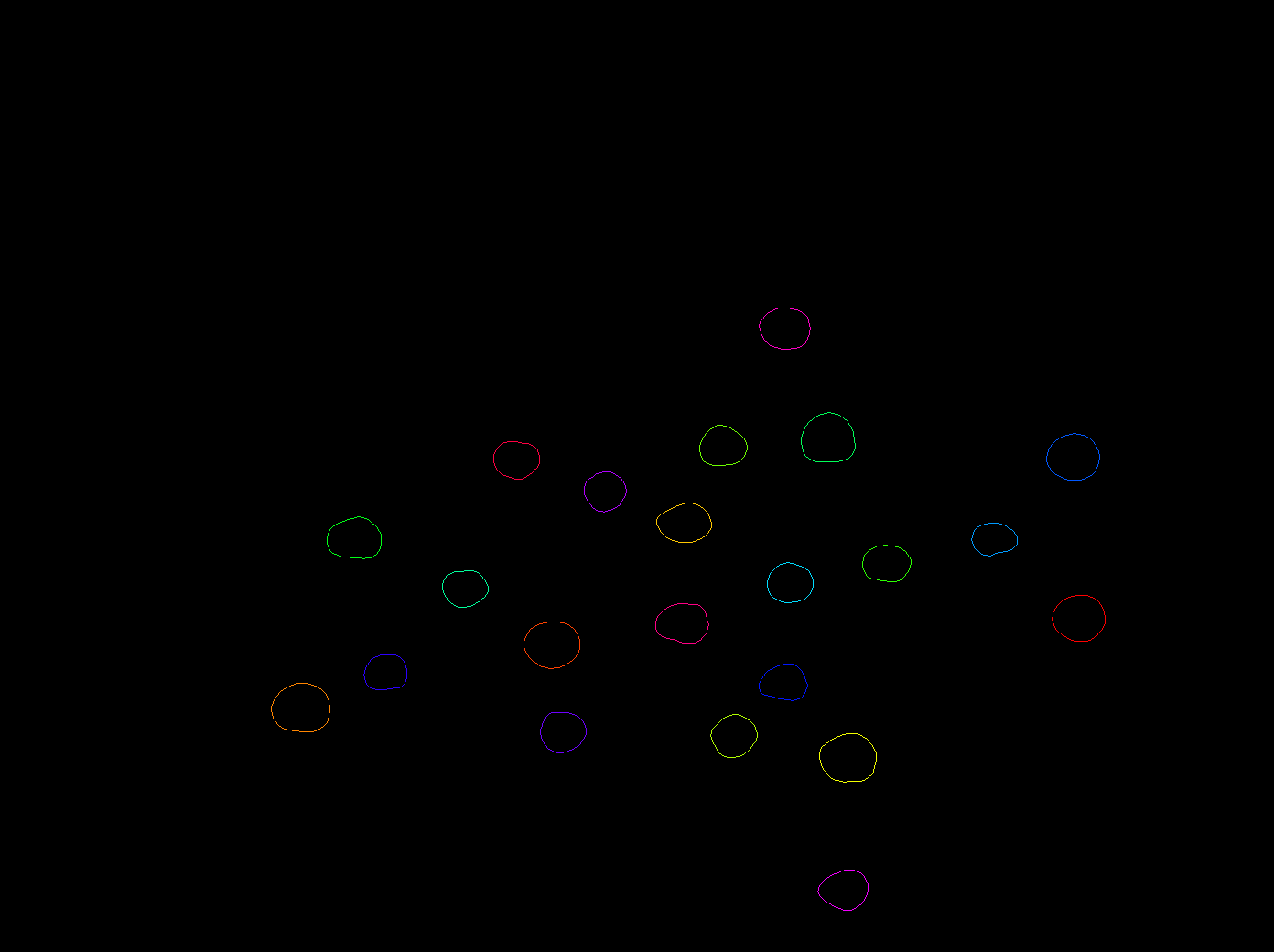

Supplement: Additional file 6 — The zip archive contains simulated images showing B cell nuclei and cytoskeleton with corresponding ground truth. (ZIP 119808 kb) [file 12859_2017_1591_MOESM6_ESM.zip › simulated B cells/cytoskeleton/not touching/cell015 seeds.png]

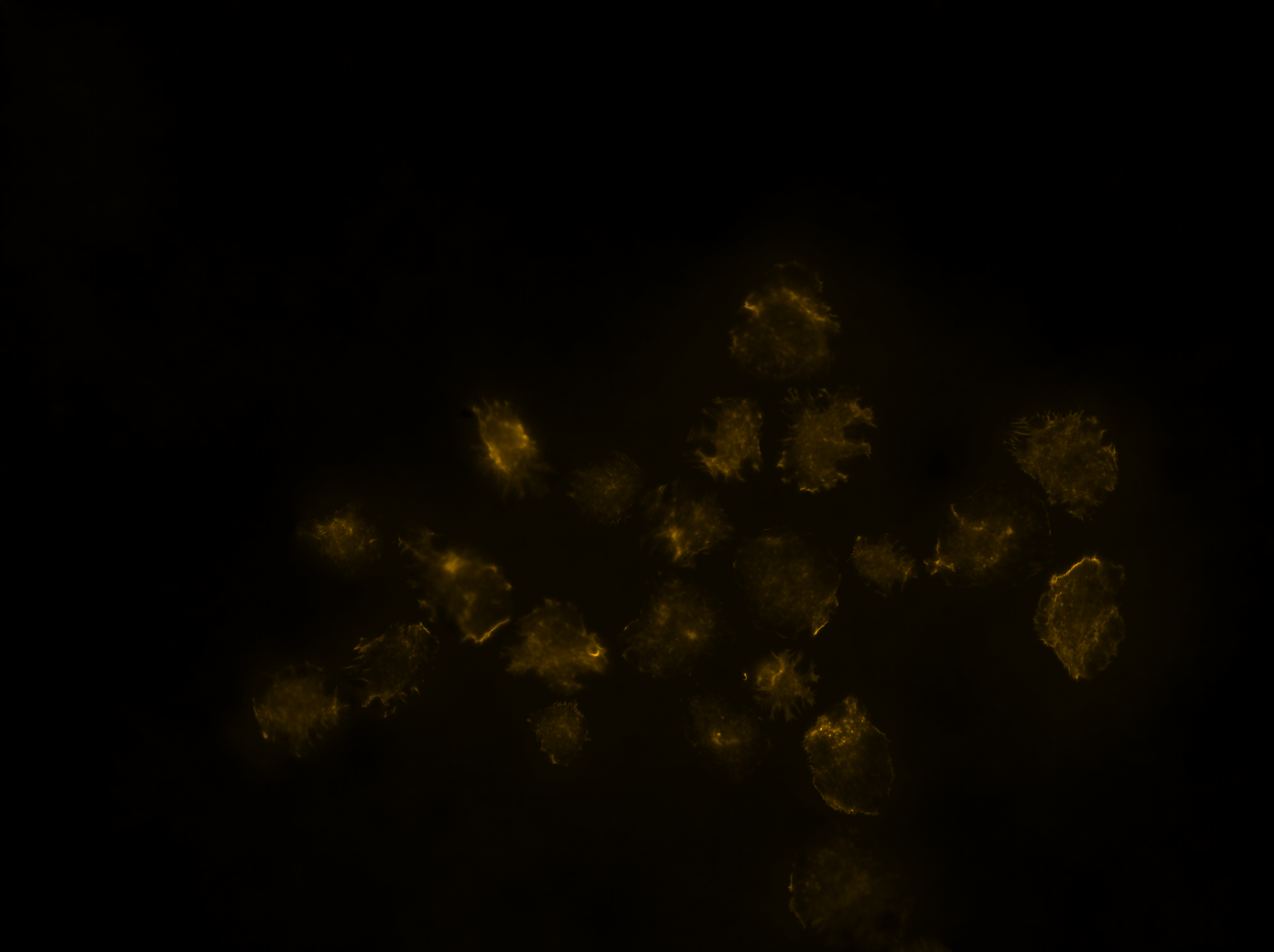

Supplement: Additional file 6 — The zip archive contains simulated images showing B cell nuclei and cytoskeleton with corresponding ground truth. (ZIP 119808 kb) [file 12859_2017_1591_MOESM6_ESM.zip › simulated B cells/cytoskeleton/not touching/cell015.png]

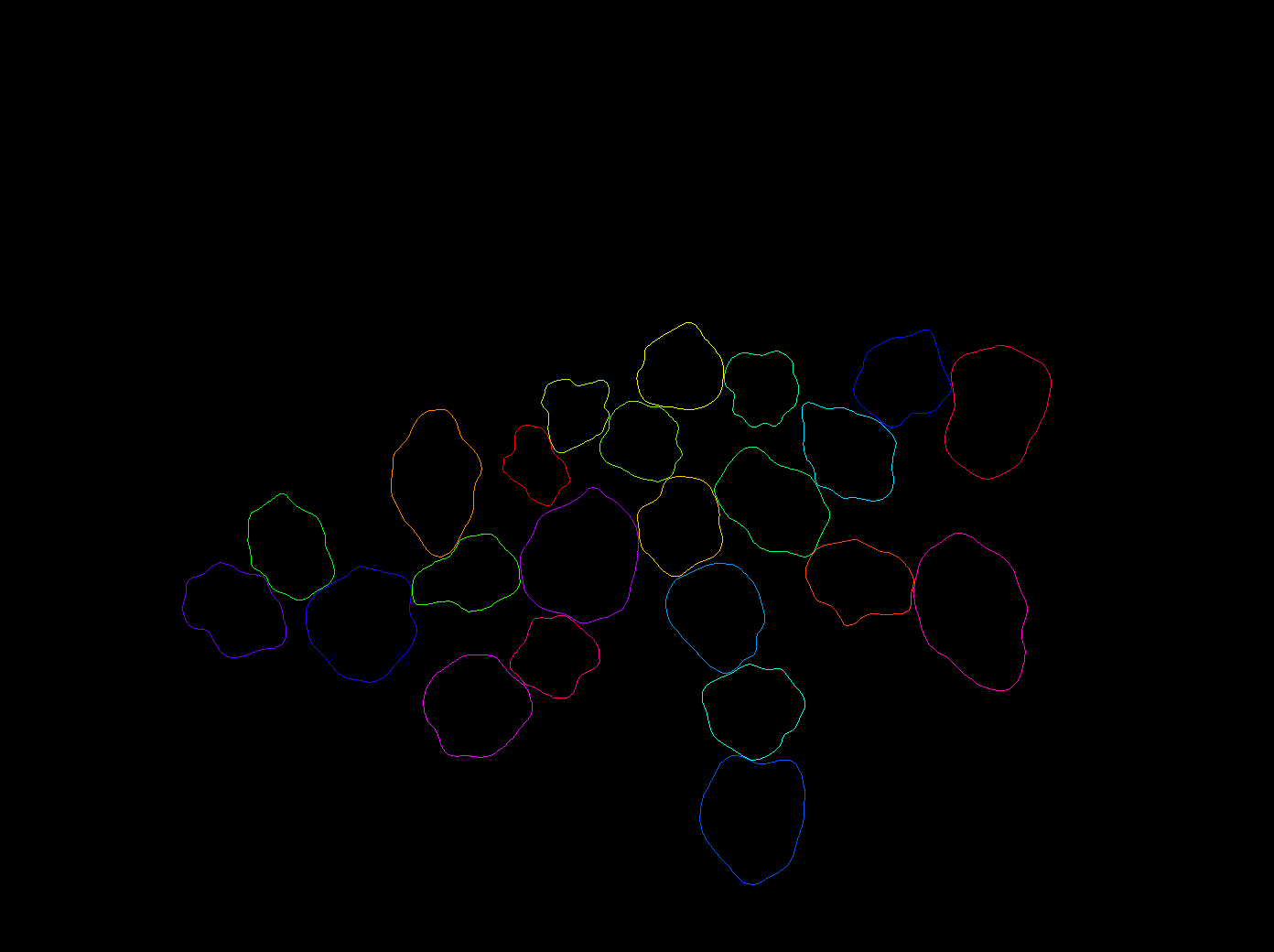

Supplement: Additional file 6 — The zip archive contains simulated images showing B cell nuclei and cytoskeleton with corresponding ground truth. (ZIP 119808 kb) [file 12859_2017_1591_MOESM6_ESM.zip › simulated B cells/cytoskeleton/not touching/cell016 gt.png]

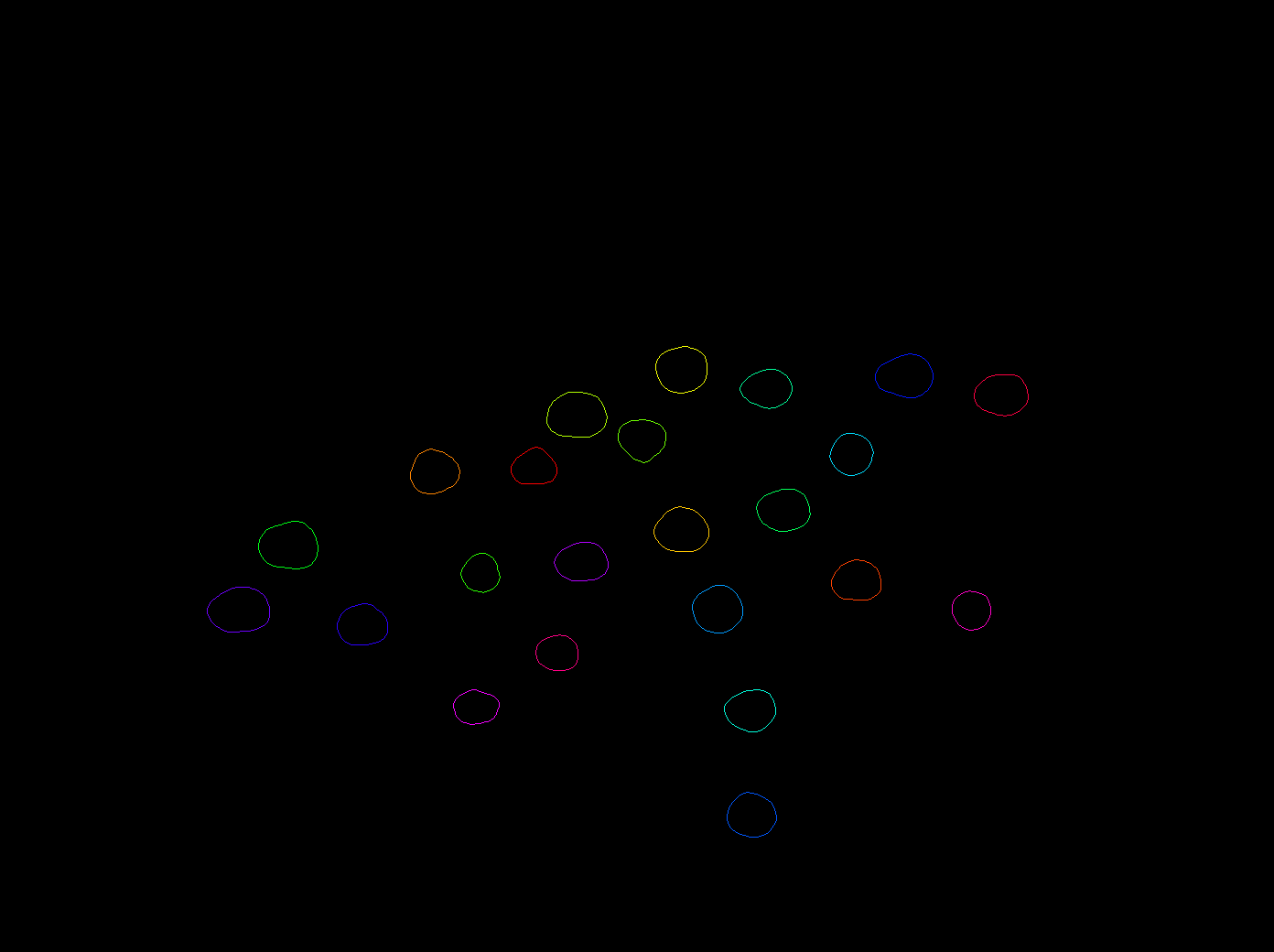

Supplement: Additional file 6 — The zip archive contains simulated images showing B cell nuclei and cytoskeleton with corresponding ground truth. (ZIP 119808 kb) [file 12859_2017_1591_MOESM6_ESM.zip › simulated B cells/cytoskeleton/not touching/cell016 seeds.png]

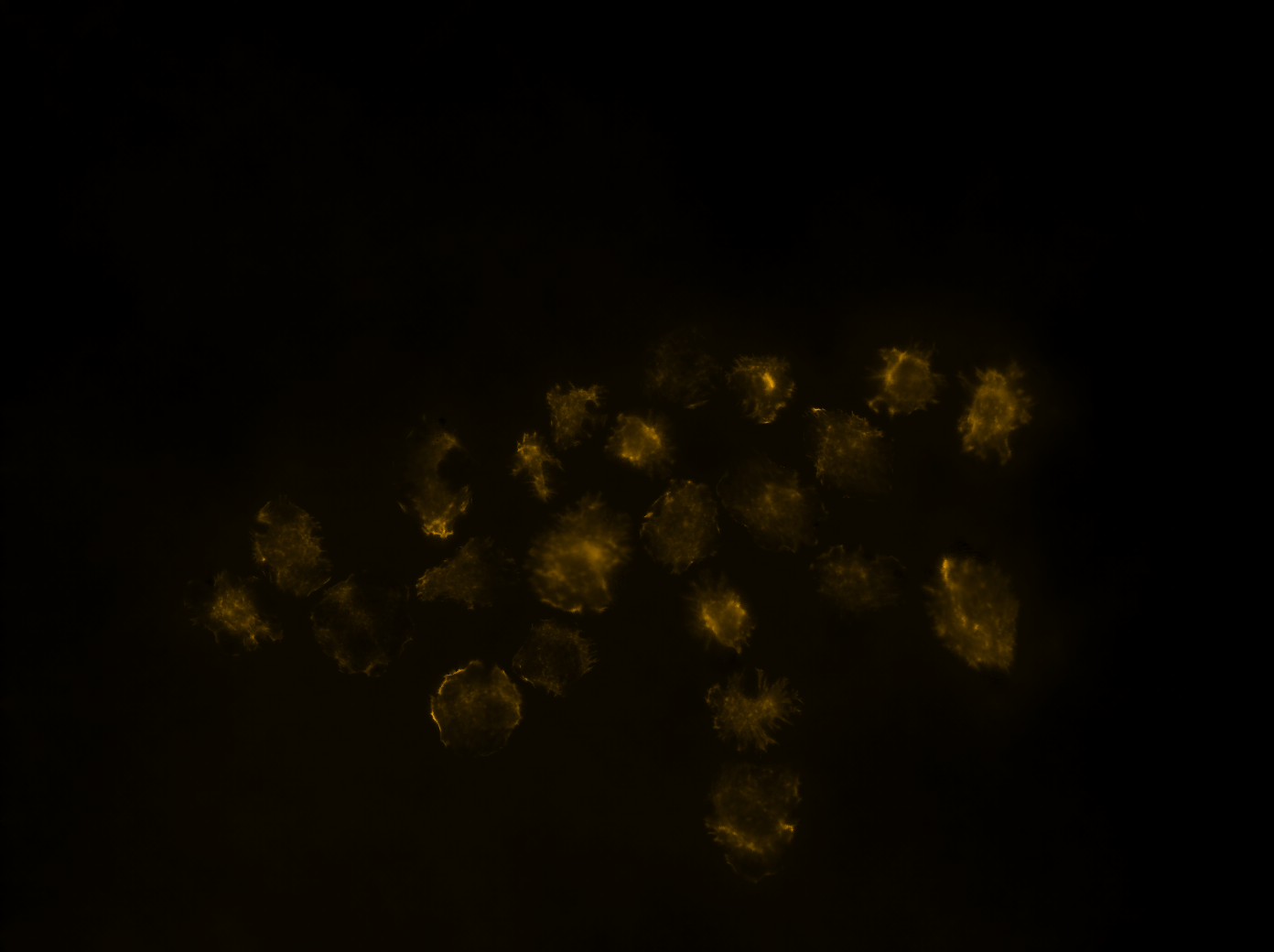

Supplement: Additional file 6 — The zip archive contains simulated images showing B cell nuclei and cytoskeleton with corresponding ground truth. (ZIP 119808 kb) [file 12859_2017_1591_MOESM6_ESM.zip › simulated B cells/cytoskeleton/not touching/cell016.png]

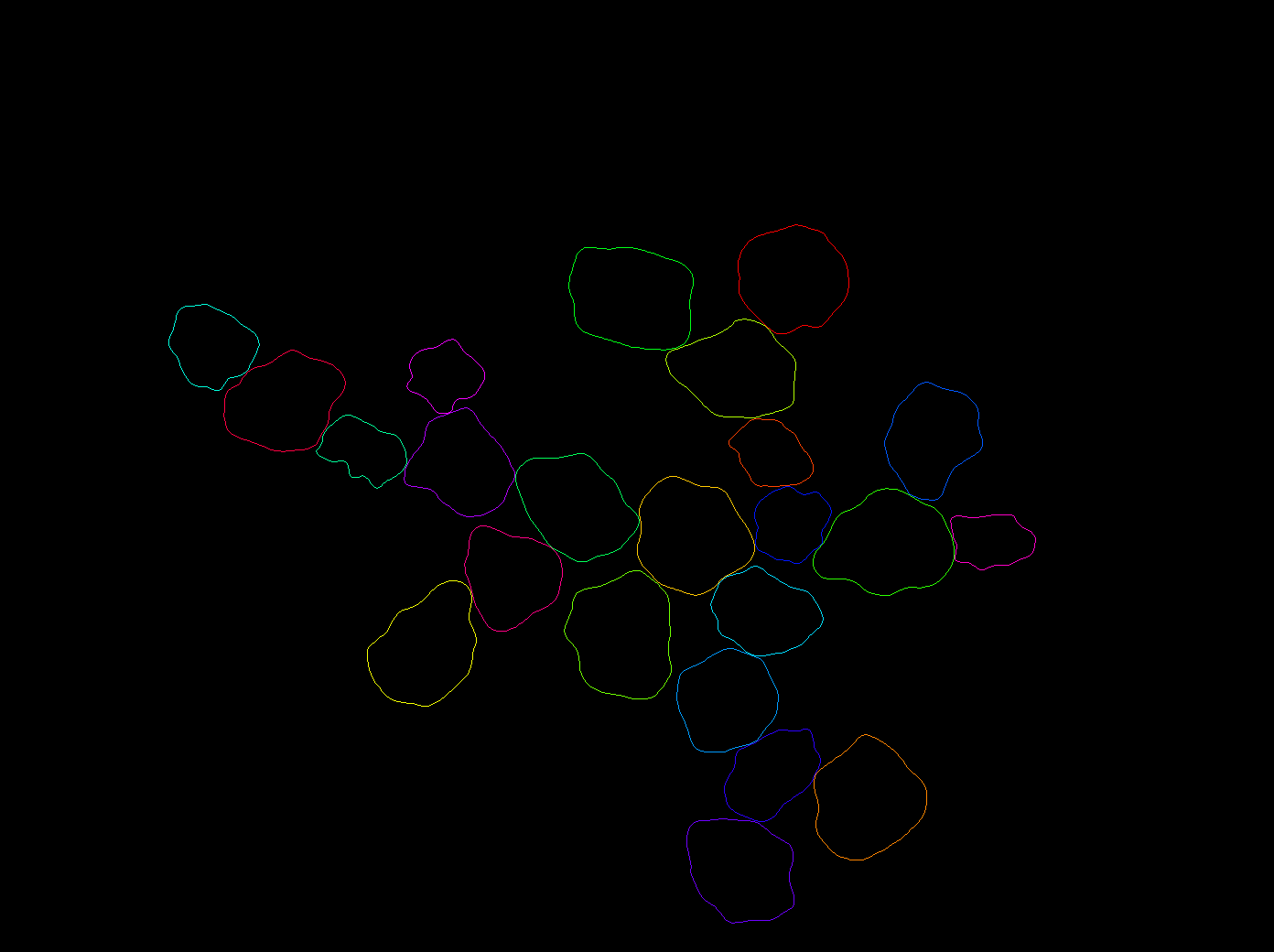

Supplement: Additional file 6 — The zip archive contains simulated images showing B cell nuclei and cytoskeleton with corresponding ground truth. (ZIP 119808 kb) [file 12859_2017_1591_MOESM6_ESM.zip › simulated B cells/cytoskeleton/not touching/cell017 gt.png]

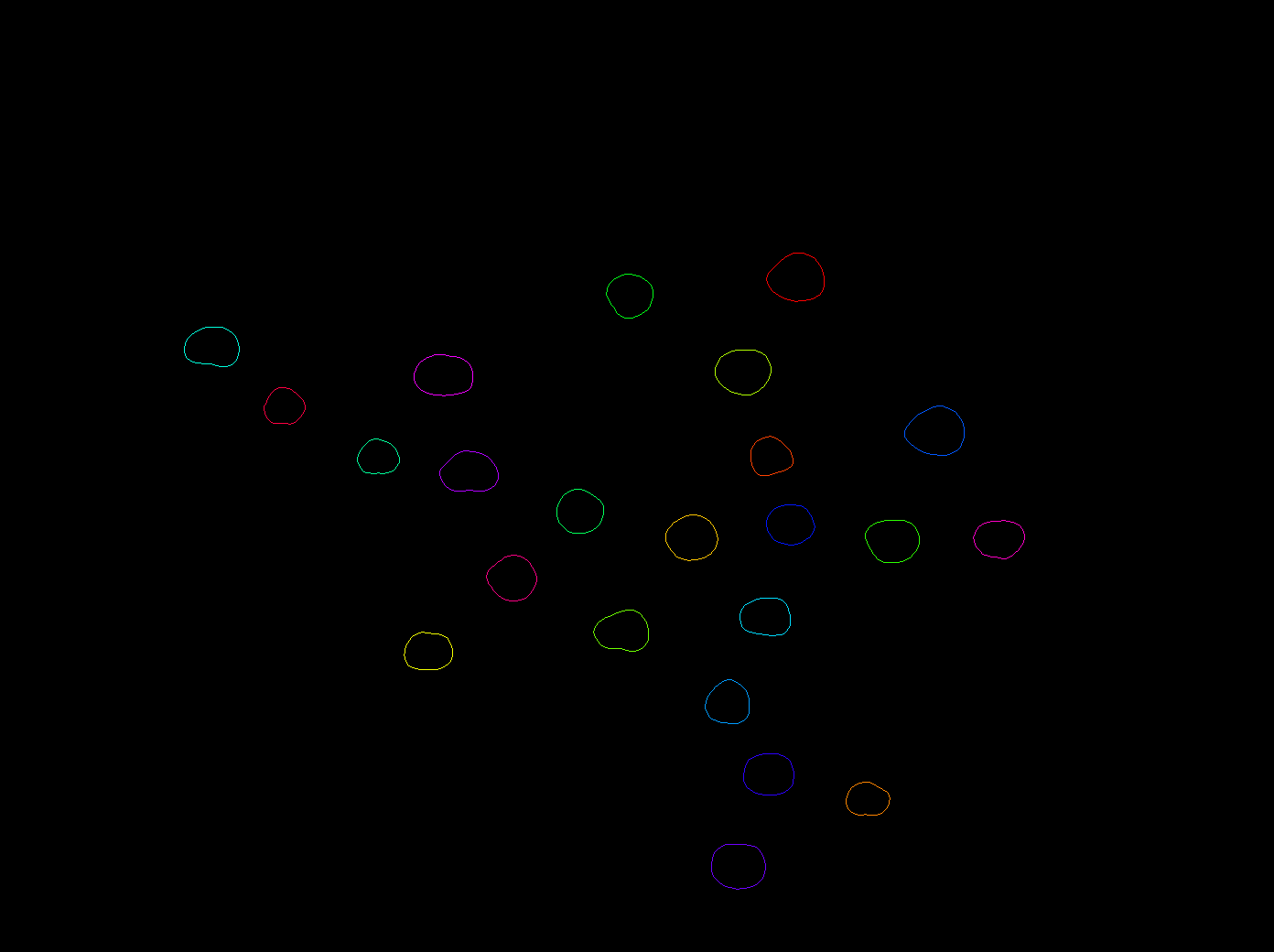

Supplement: Additional file 6 — The zip archive contains simulated images showing B cell nuclei and cytoskeleton with corresponding ground truth. (ZIP 119808 kb) [file 12859_2017_1591_MOESM6_ESM.zip › simulated B cells/cytoskeleton/not touching/cell017 seeds.png]

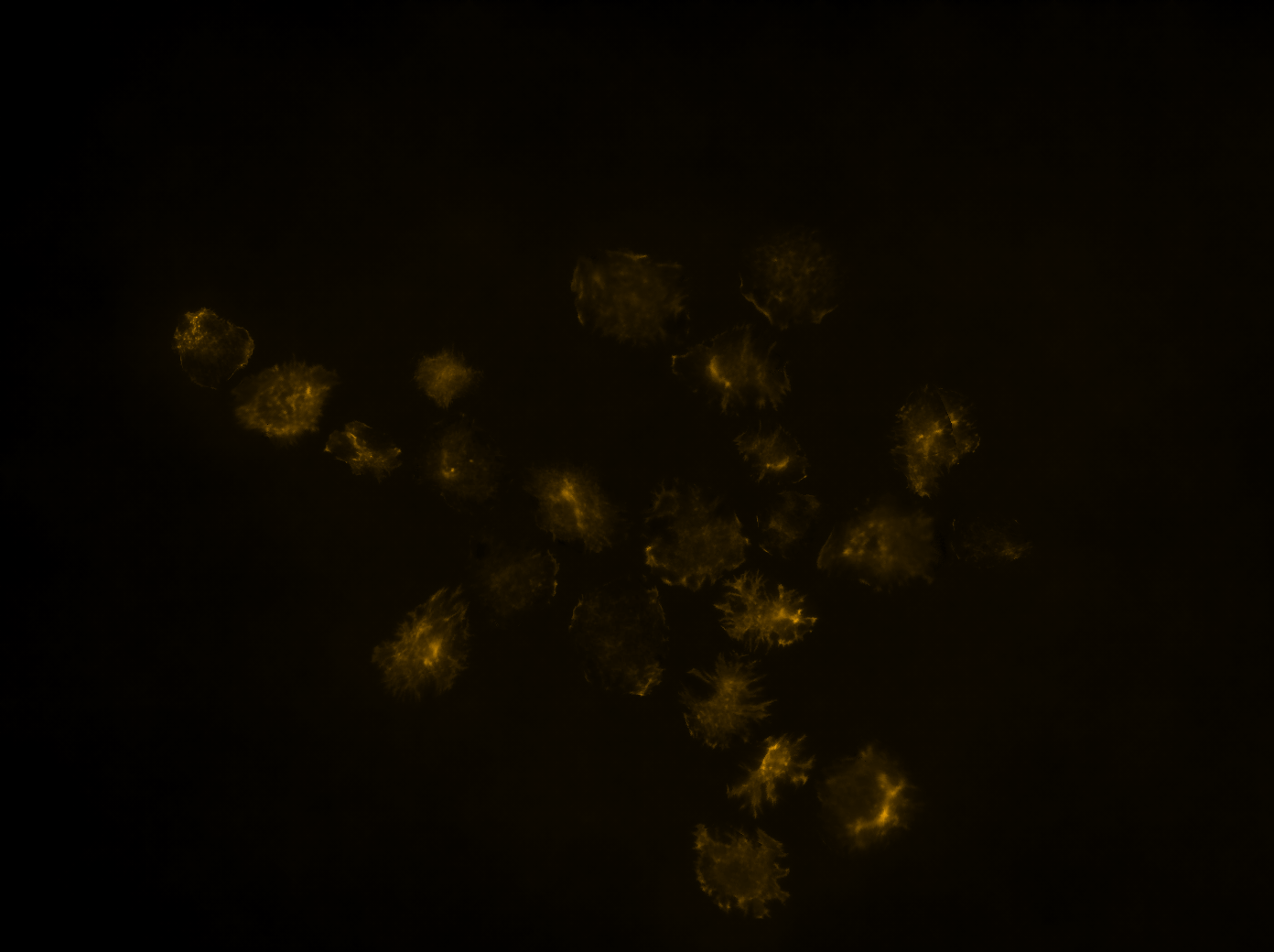

Supplement: Additional file 6 — The zip archive contains simulated images showing B cell nuclei and cytoskeleton with corresponding ground truth. (ZIP 119808 kb) [file 12859_2017_1591_MOESM6_ESM.zip › simulated B cells/cytoskeleton/not touching/cell017.png]

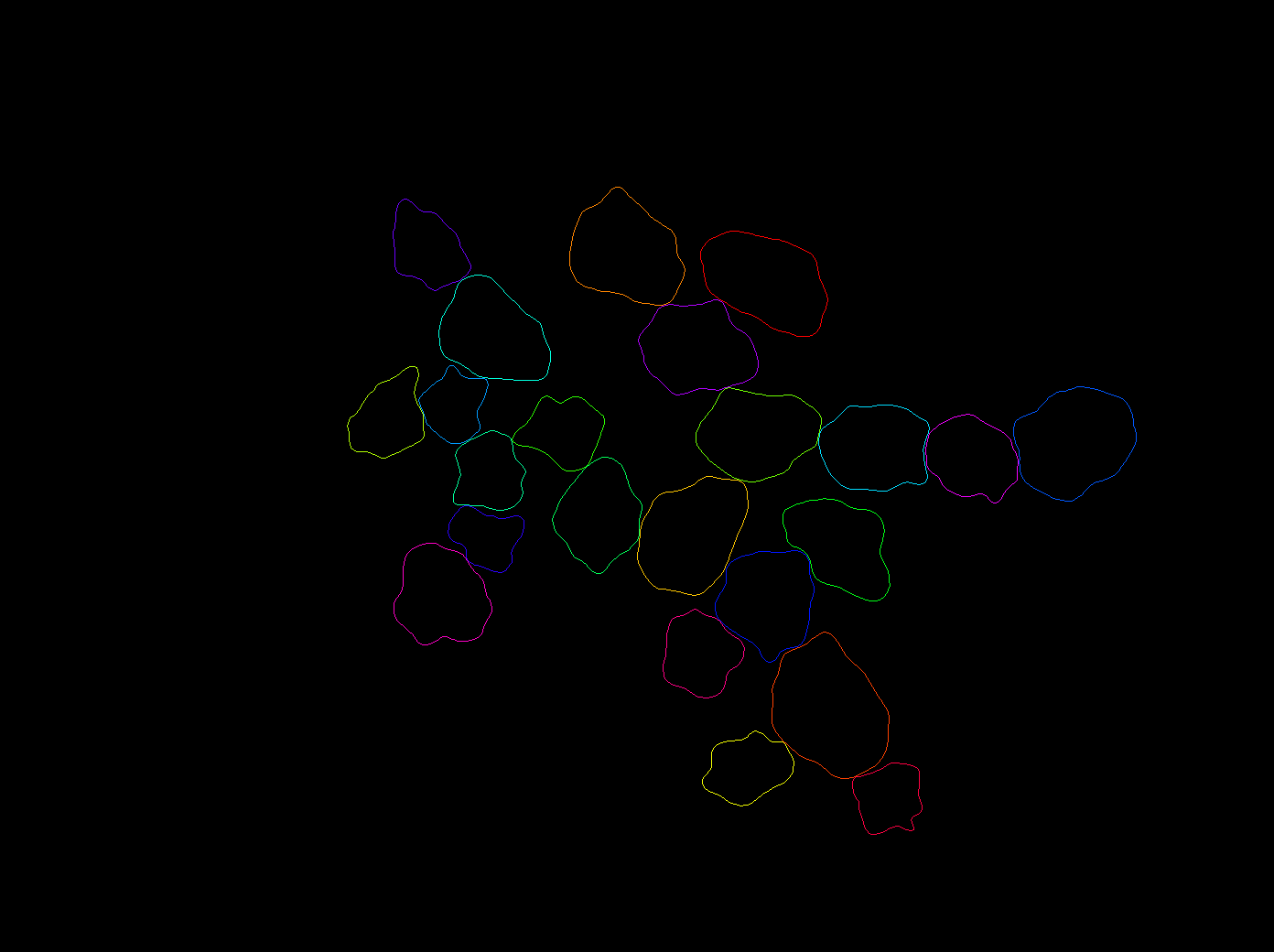

Supplement: Additional file 6 — The zip archive contains simulated images showing B cell nuclei and cytoskeleton with corresponding ground truth. (ZIP 119808 kb) [file 12859_2017_1591_MOESM6_ESM.zip › simulated B cells/cytoskeleton/not touching/cell018 gt.png]

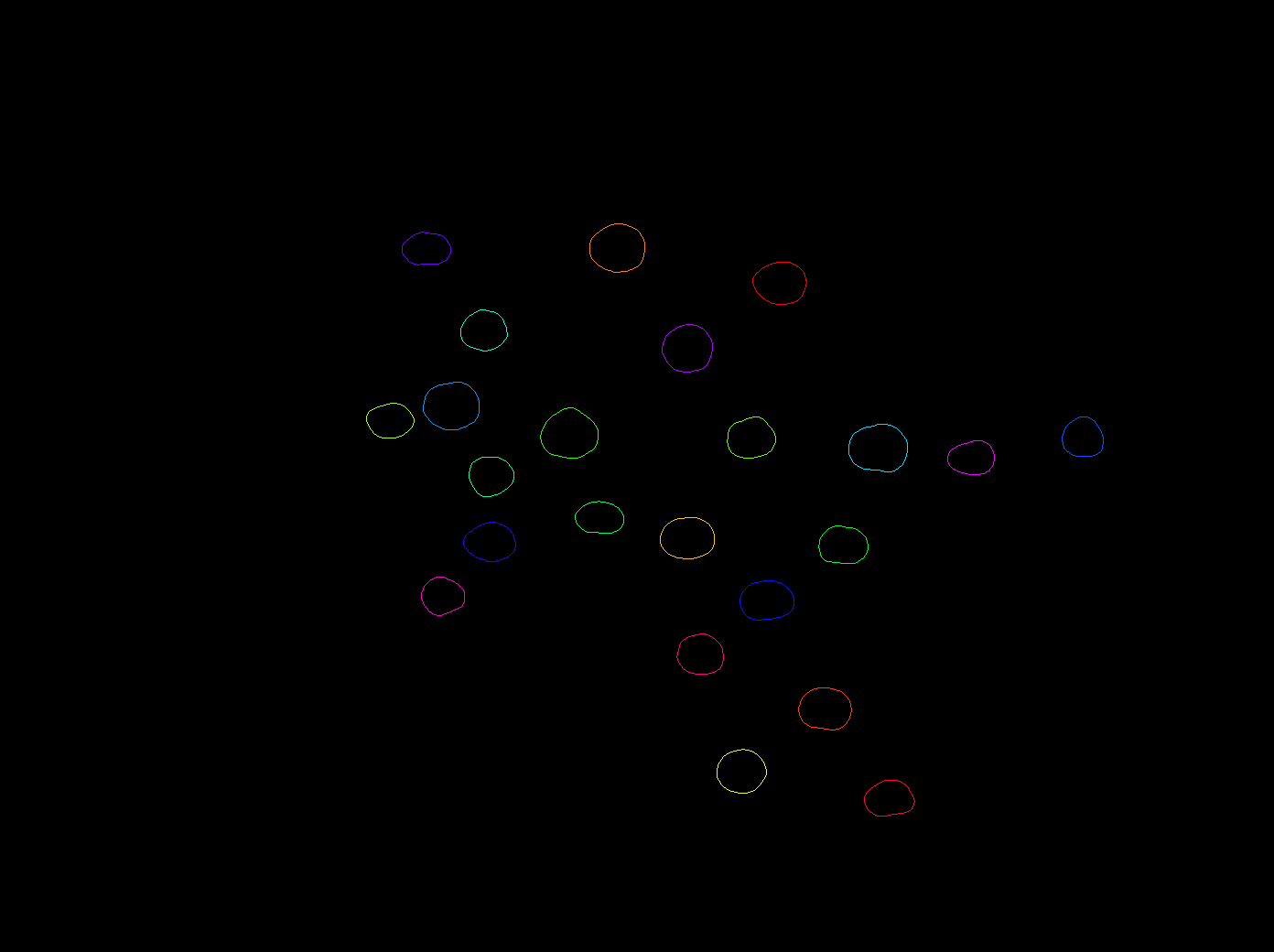

Supplement: Additional file 6 — The zip archive contains simulated images showing B cell nuclei and cytoskeleton with corresponding ground truth. (ZIP 119808 kb) [file 12859_2017_1591_MOESM6_ESM.zip › simulated B cells/cytoskeleton/not touching/cell018 seeds.png]

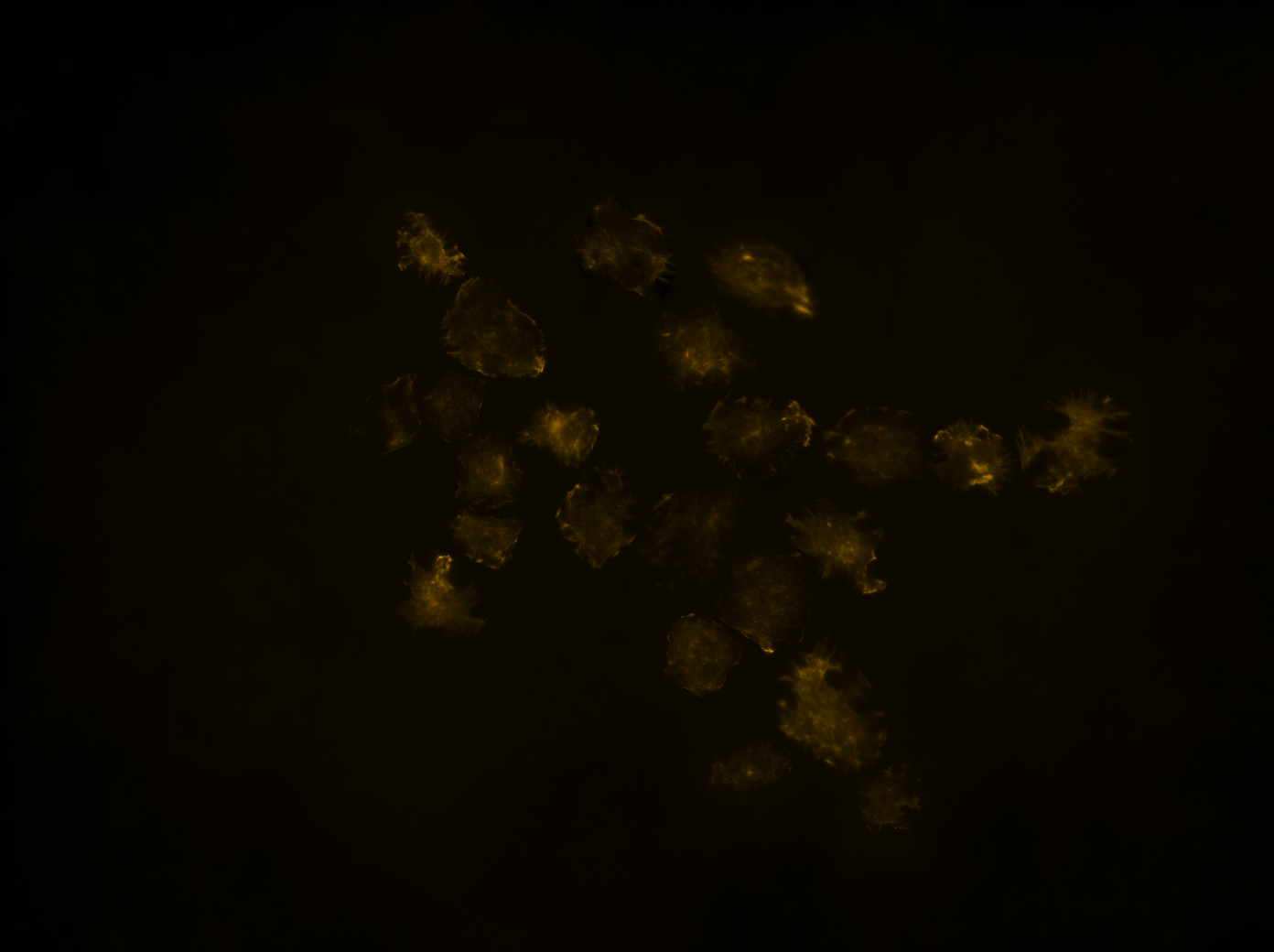

Supplement: Additional file 6 — The zip archive contains simulated images showing B cell nuclei and cytoskeleton with corresponding ground truth. (ZIP 119808 kb) [file 12859_2017_1591_MOESM6_ESM.zip › simulated B cells/cytoskeleton/not touching/cell018.png]

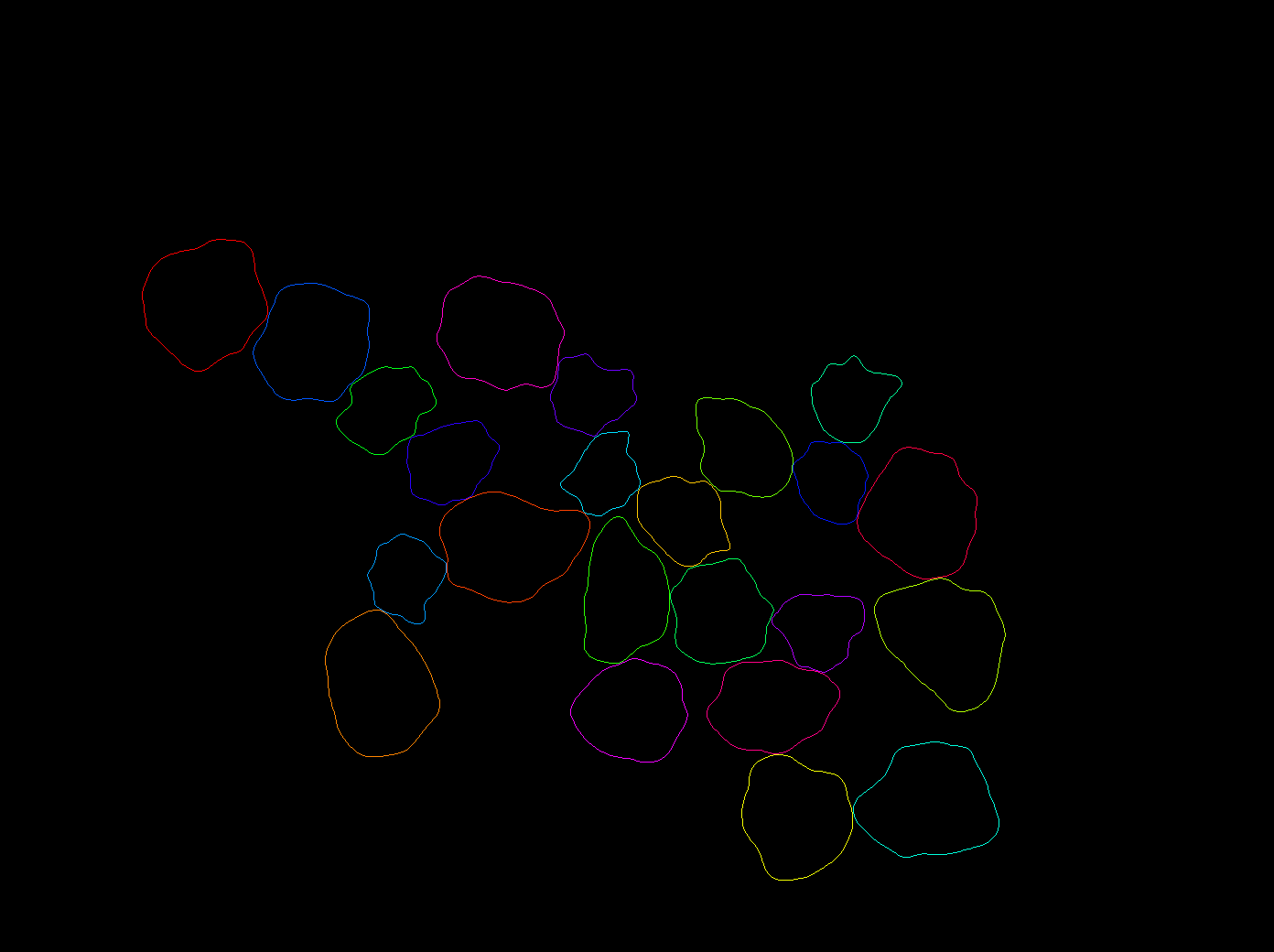

Supplement: Additional file 6 — The zip archive contains simulated images showing B cell nuclei and cytoskeleton with corresponding ground truth. (ZIP 119808 kb) [file 12859_2017_1591_MOESM6_ESM.zip › simulated B cells/cytoskeleton/not touching/cell019 gt.png]

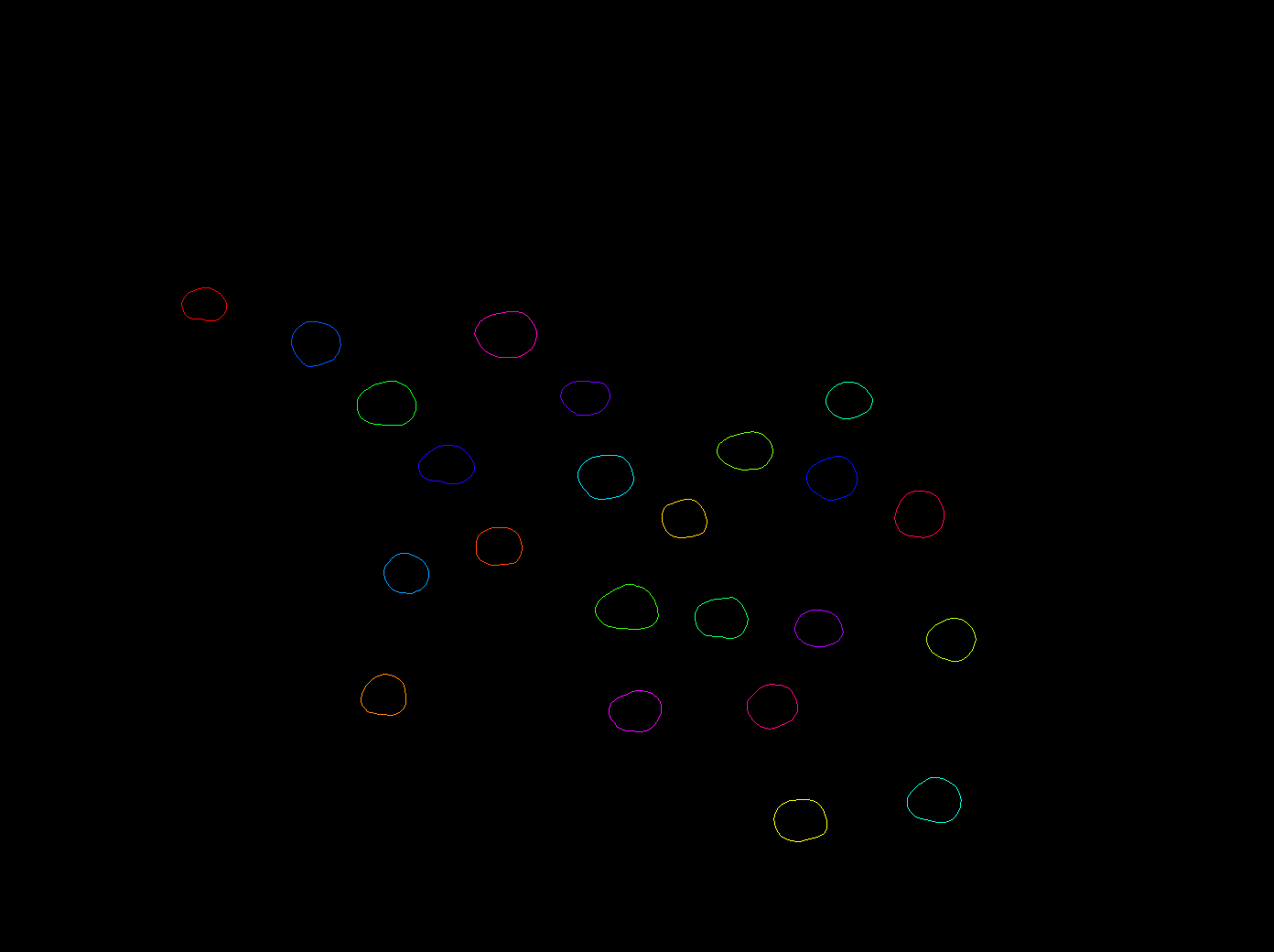

Supplement: Additional file 6 — The zip archive contains simulated images showing B cell nuclei and cytoskeleton with corresponding ground truth. (ZIP 119808 kb) [file 12859_2017_1591_MOESM6_ESM.zip › simulated B cells/cytoskeleton/not touching/cell019 seeds.png]

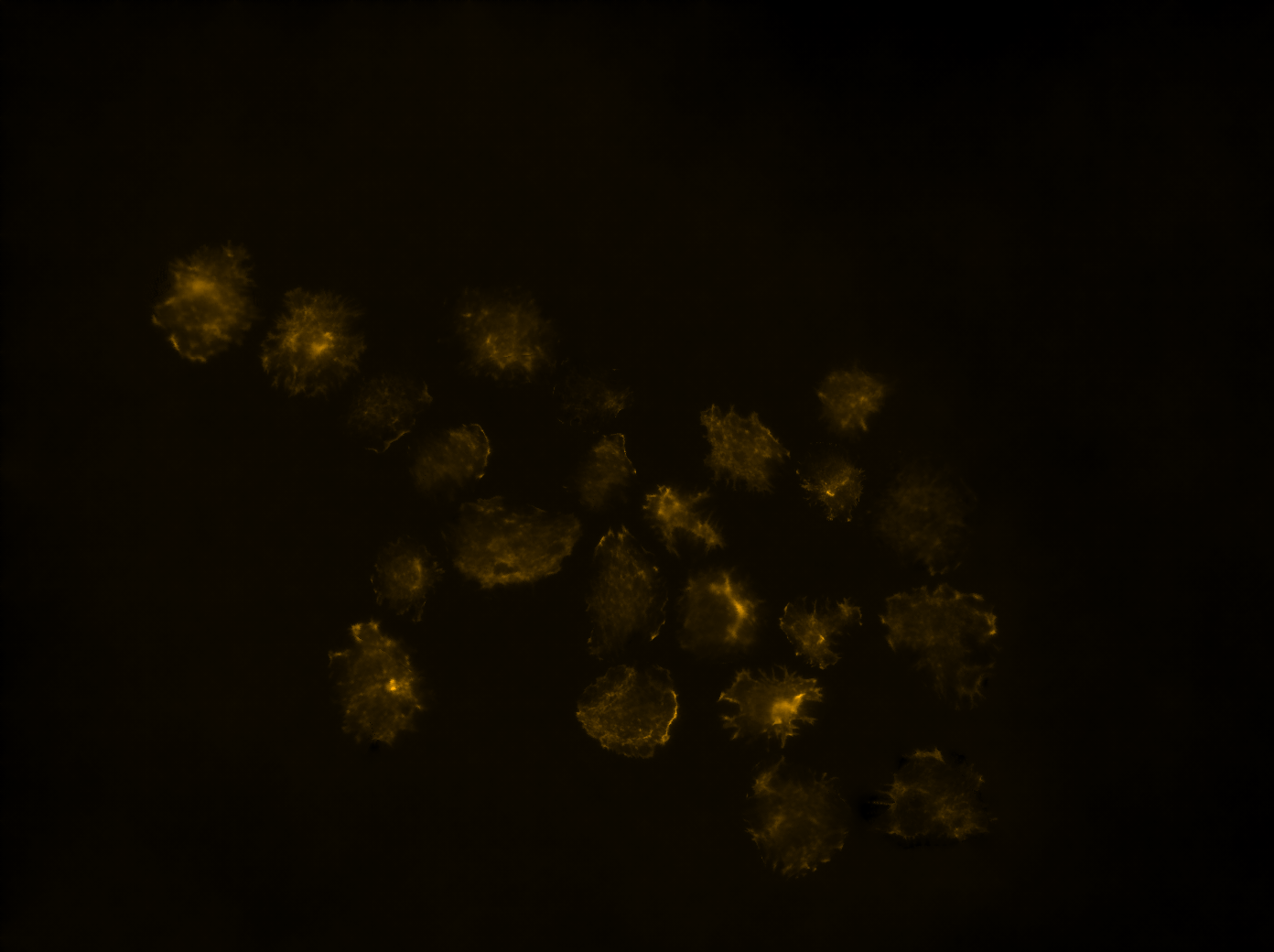

Supplement: Additional file 6 — The zip archive contains simulated images showing B cell nuclei and cytoskeleton with corresponding ground truth. (ZIP 119808 kb) [file 12859_2017_1591_MOESM6_ESM.zip › simulated B cells/cytoskeleton/not touching/cell019.png]

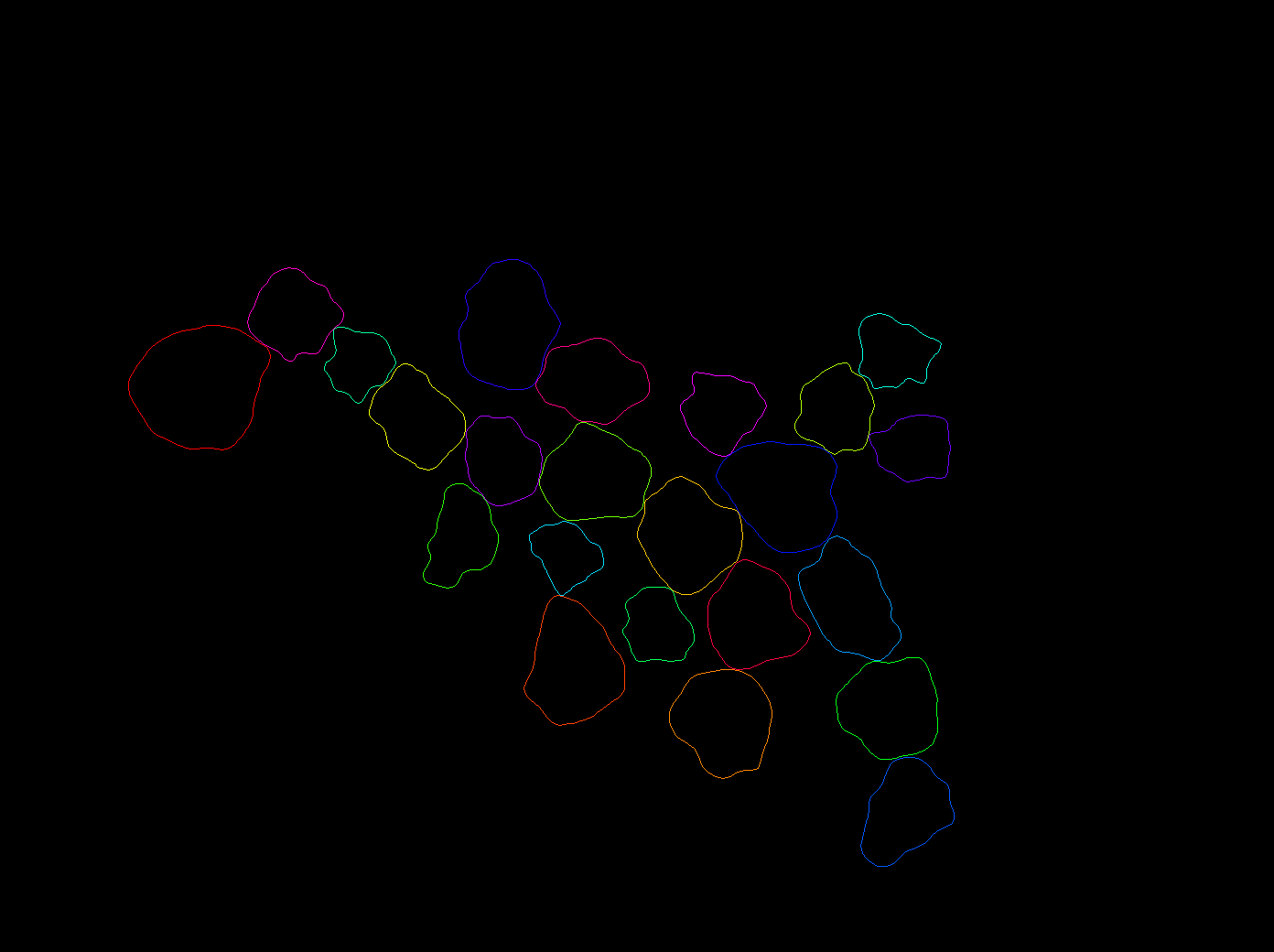

Supplement: Additional file 6 — The zip archive contains simulated images showing B cell nuclei and cytoskeleton with corresponding ground truth. (ZIP 119808 kb) [file 12859_2017_1591_MOESM6_ESM.zip › simulated B cells/cytoskeleton/not touching/cell020 gt.png]

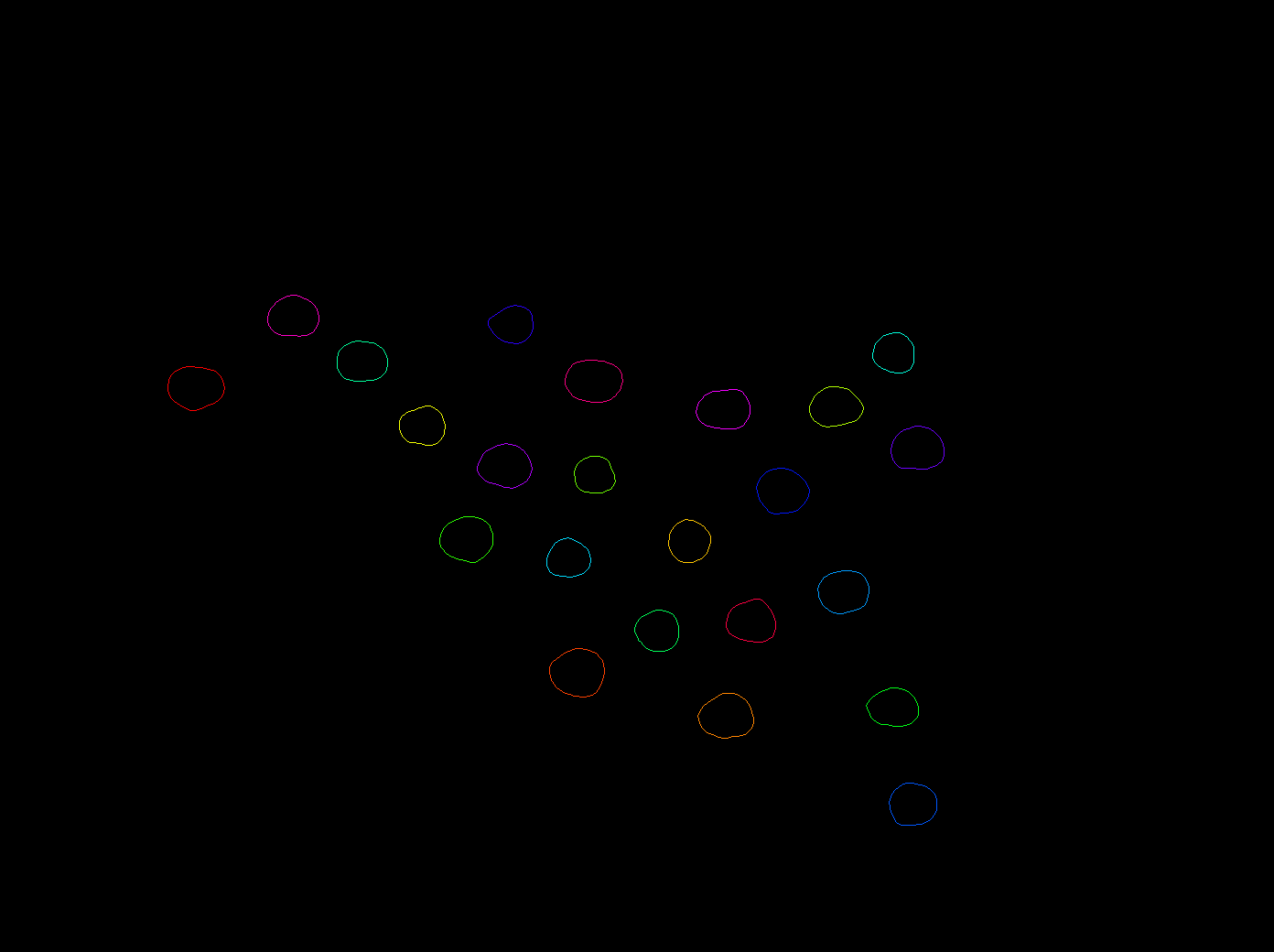

Supplement: Additional file 6 — The zip archive contains simulated images showing B cell nuclei and cytoskeleton with corresponding ground truth. (ZIP 119808 kb) [file 12859_2017_1591_MOESM6_ESM.zip › simulated B cells/cytoskeleton/not touching/cell020 seeds.png]

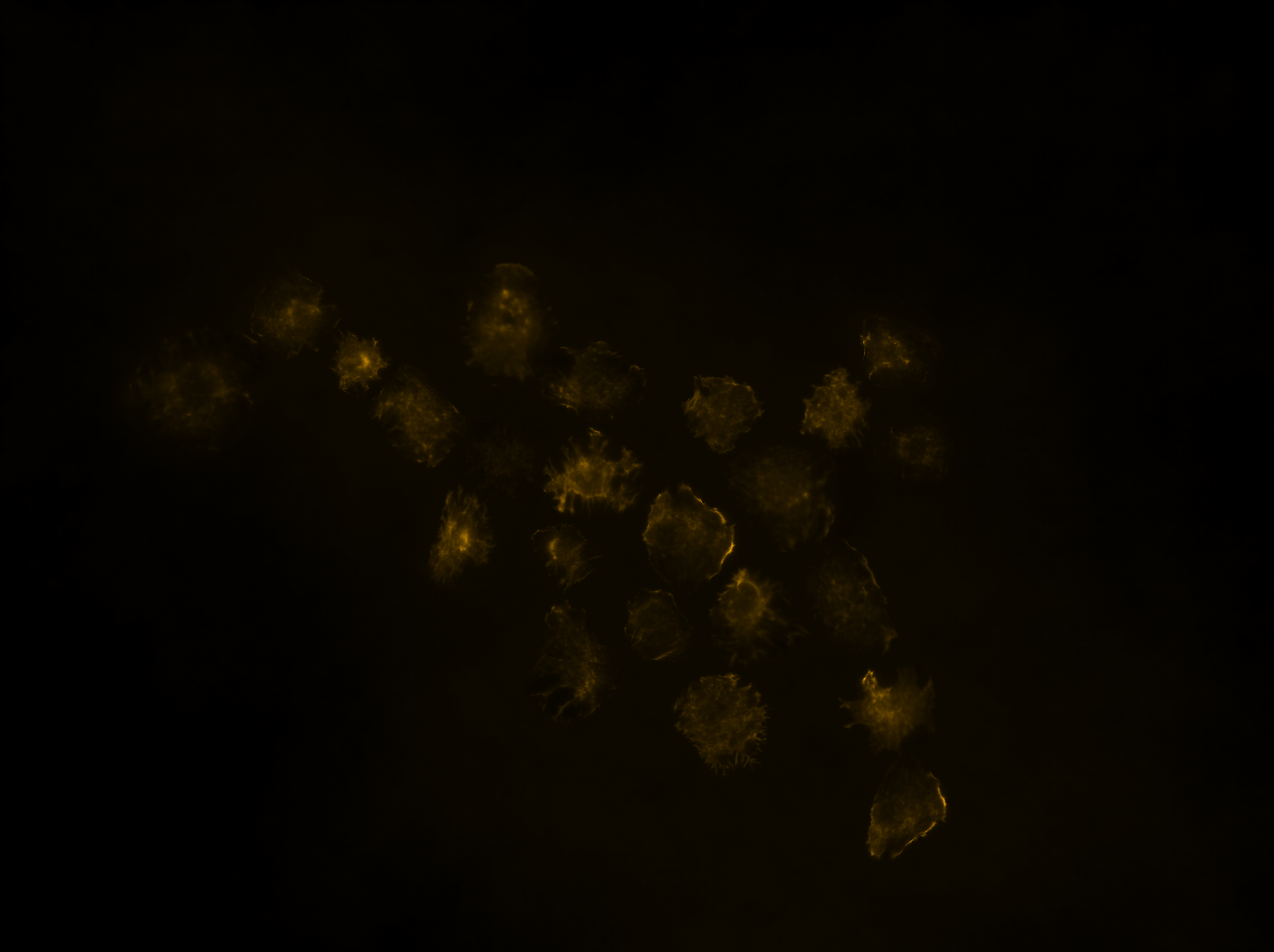

Supplement: Additional file 6 — The zip archive contains simulated images showing B cell nuclei and cytoskeleton with corresponding ground truth. (ZIP 119808 kb) [file 12859_2017_1591_MOESM6_ESM.zip › simulated B cells/cytoskeleton/not touching/cell020.png]

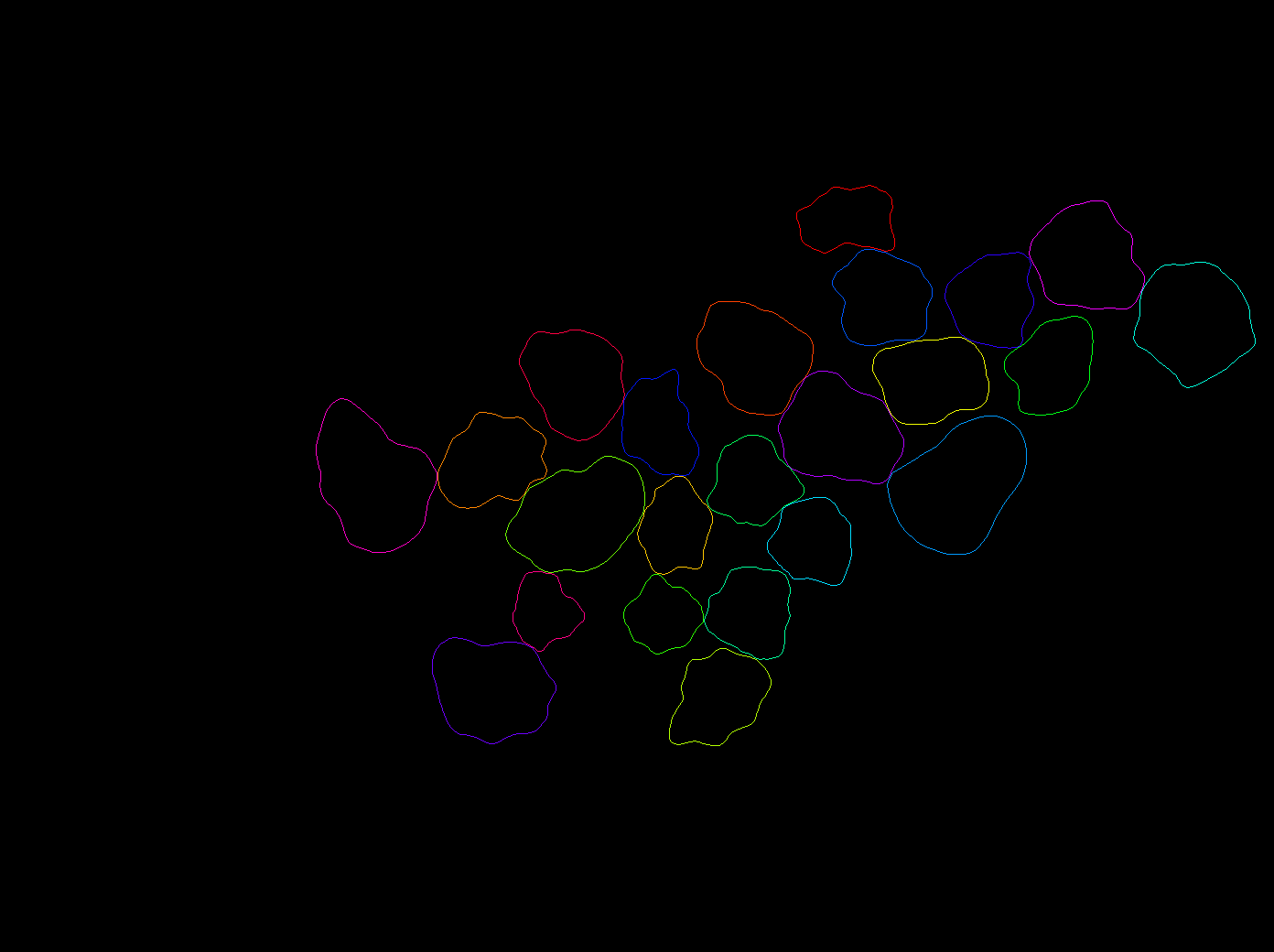

Supplement: Additional file 6 — The zip archive contains simulated images showing B cell nuclei and cytoskeleton with corresponding ground truth. (ZIP 119808 kb) [file 12859_2017_1591_MOESM6_ESM.zip › simulated B cells/cytoskeleton/not touching/cell021 gt.png]

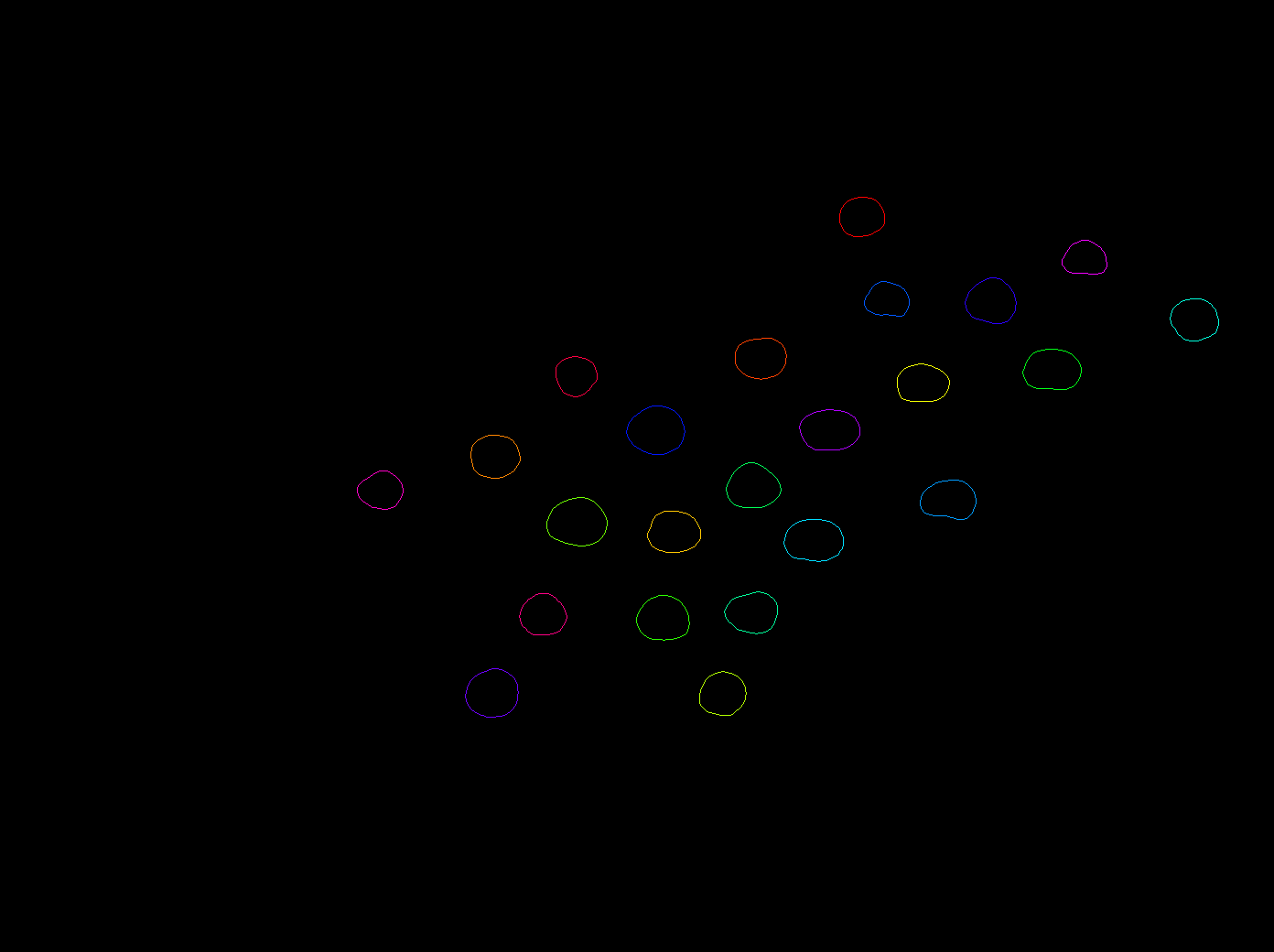

Supplement: Additional file 6 — The zip archive contains simulated images showing B cell nuclei and cytoskeleton with corresponding ground truth. (ZIP 119808 kb) [file 12859_2017_1591_MOESM6_ESM.zip › simulated B cells/cytoskeleton/not touching/cell021 seeds.png]

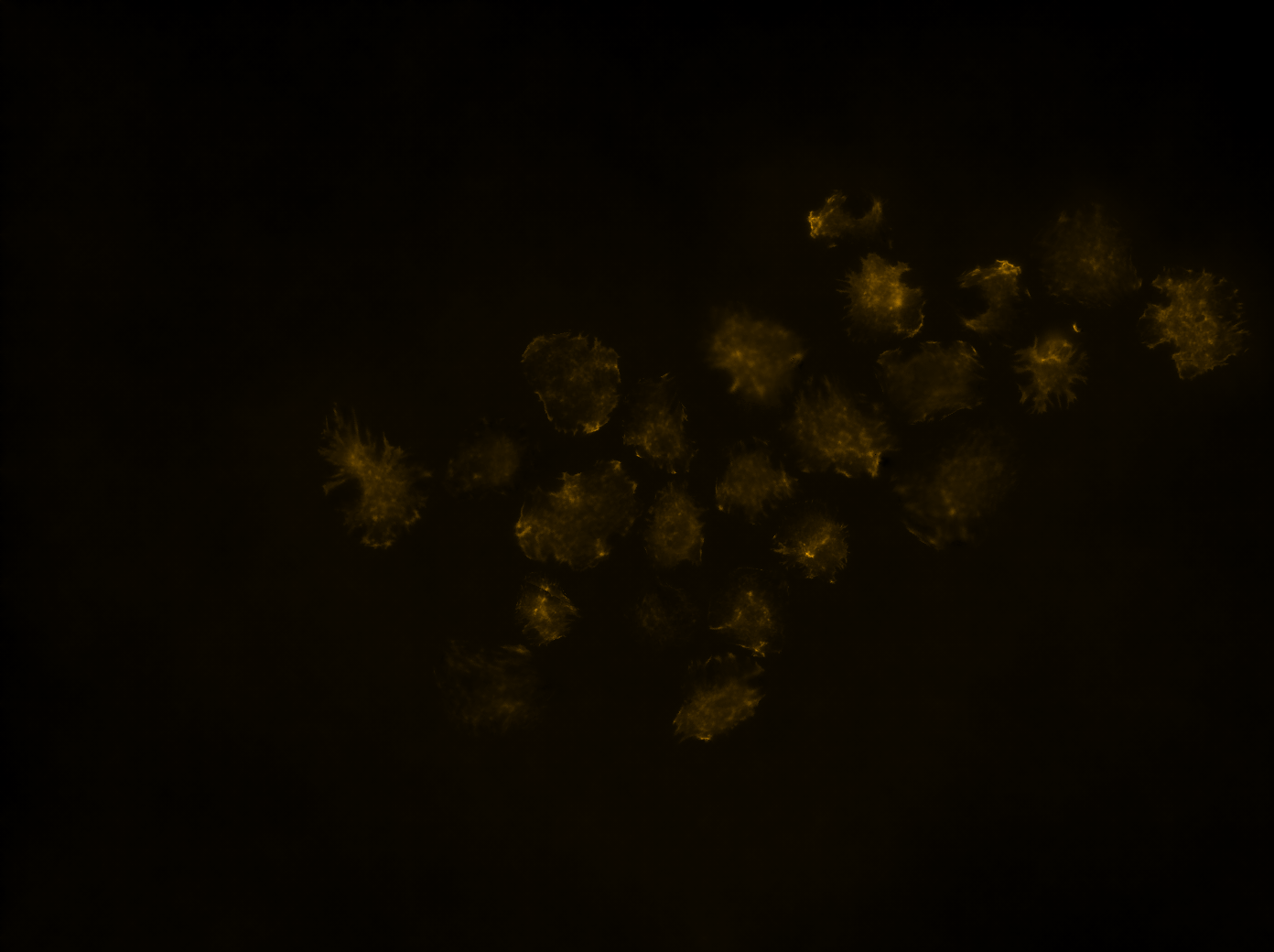

Supplement: Additional file 6 — The zip archive contains simulated images showing B cell nuclei and cytoskeleton with corresponding ground truth. (ZIP 119808 kb) [file 12859_2017_1591_MOESM6_ESM.zip › simulated B cells/cytoskeleton/not touching/cell021.png]

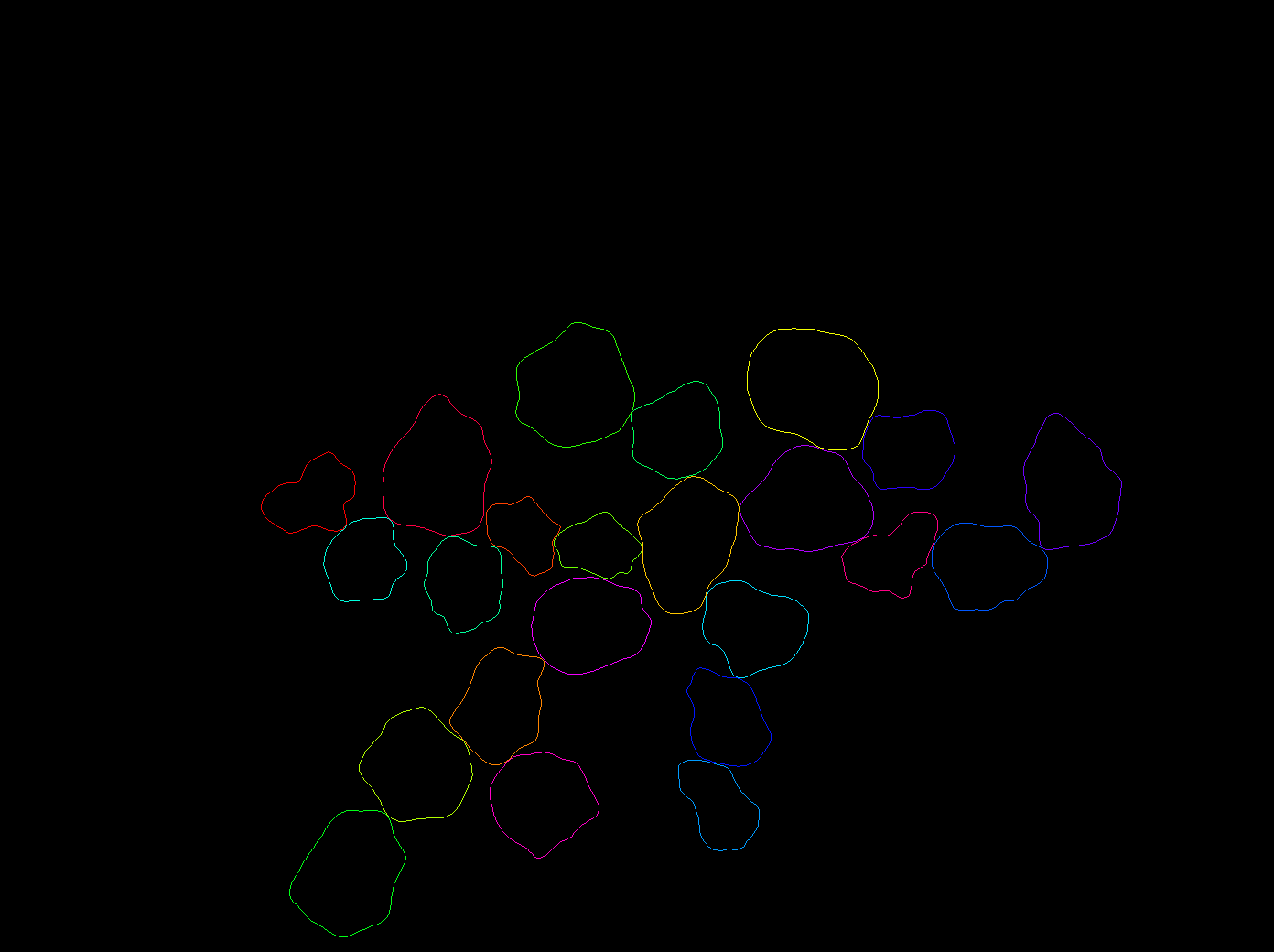

Supplement: Additional file 6 — The zip archive contains simulated images showing B cell nuclei and cytoskeleton with corresponding ground truth. (ZIP 119808 kb) [file 12859_2017_1591_MOESM6_ESM.zip › simulated B cells/cytoskeleton/not touching/cell022 gt.png]

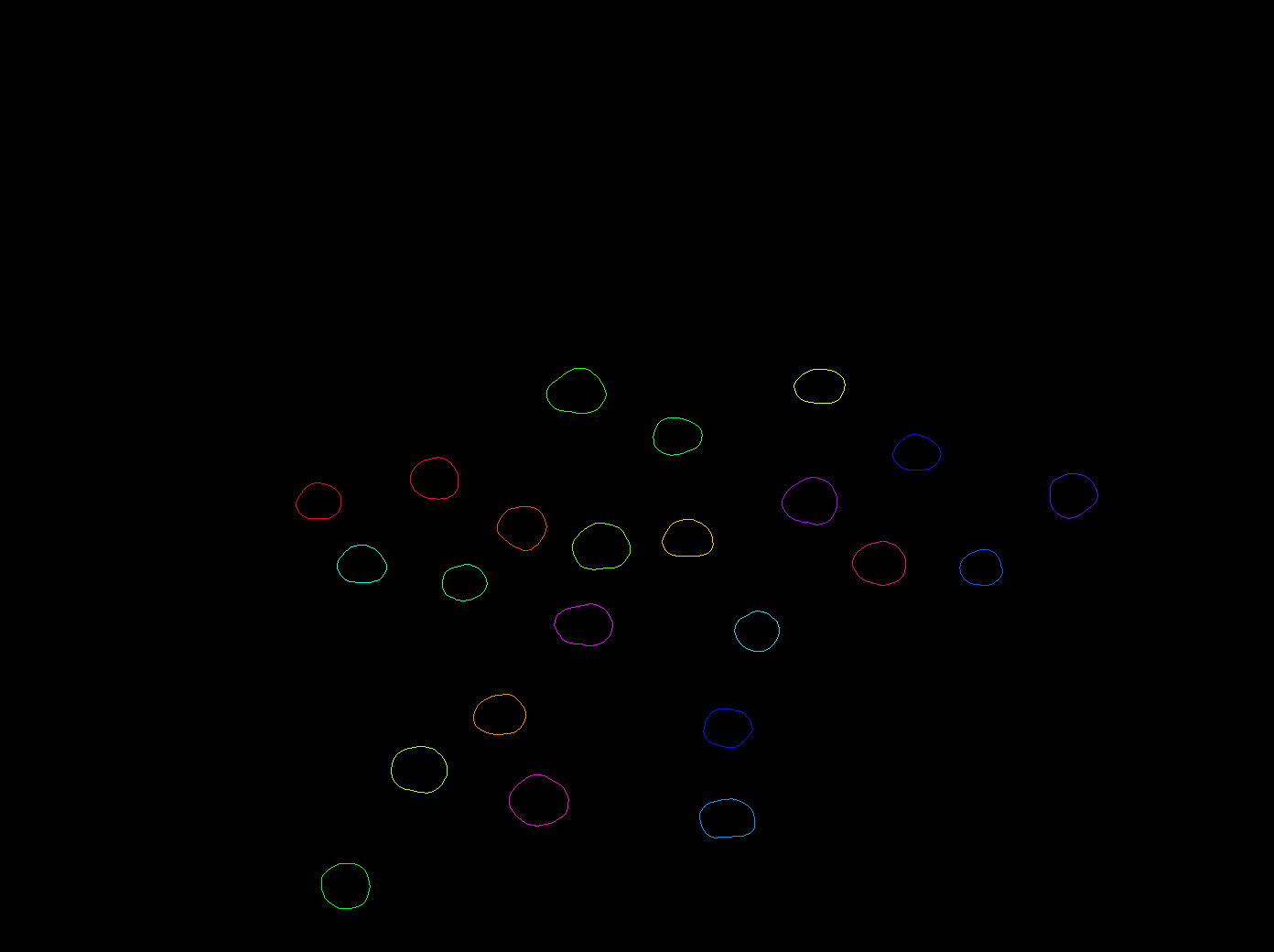

Supplement: Additional file 6 — The zip archive contains simulated images showing B cell nuclei and cytoskeleton with corresponding ground truth. (ZIP 119808 kb) [file 12859_2017_1591_MOESM6_ESM.zip › simulated B cells/cytoskeleton/not touching/cell022 seeds.png]

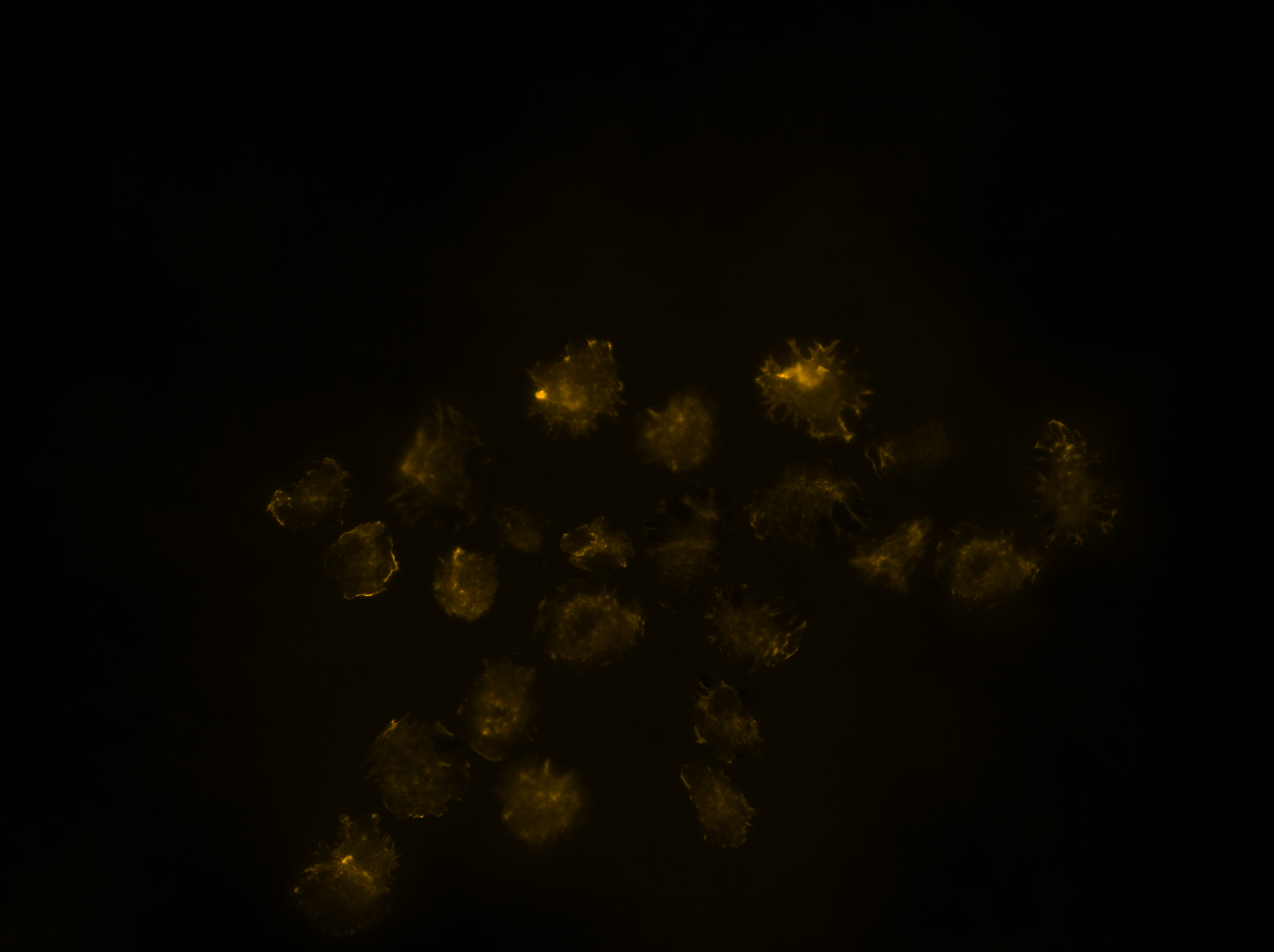

Supplement: Additional file 6 — The zip archive contains simulated images showing B cell nuclei and cytoskeleton with corresponding ground truth. (ZIP 119808 kb) [file 12859_2017_1591_MOESM6_ESM.zip › simulated B cells/cytoskeleton/not touching/cell022.png]

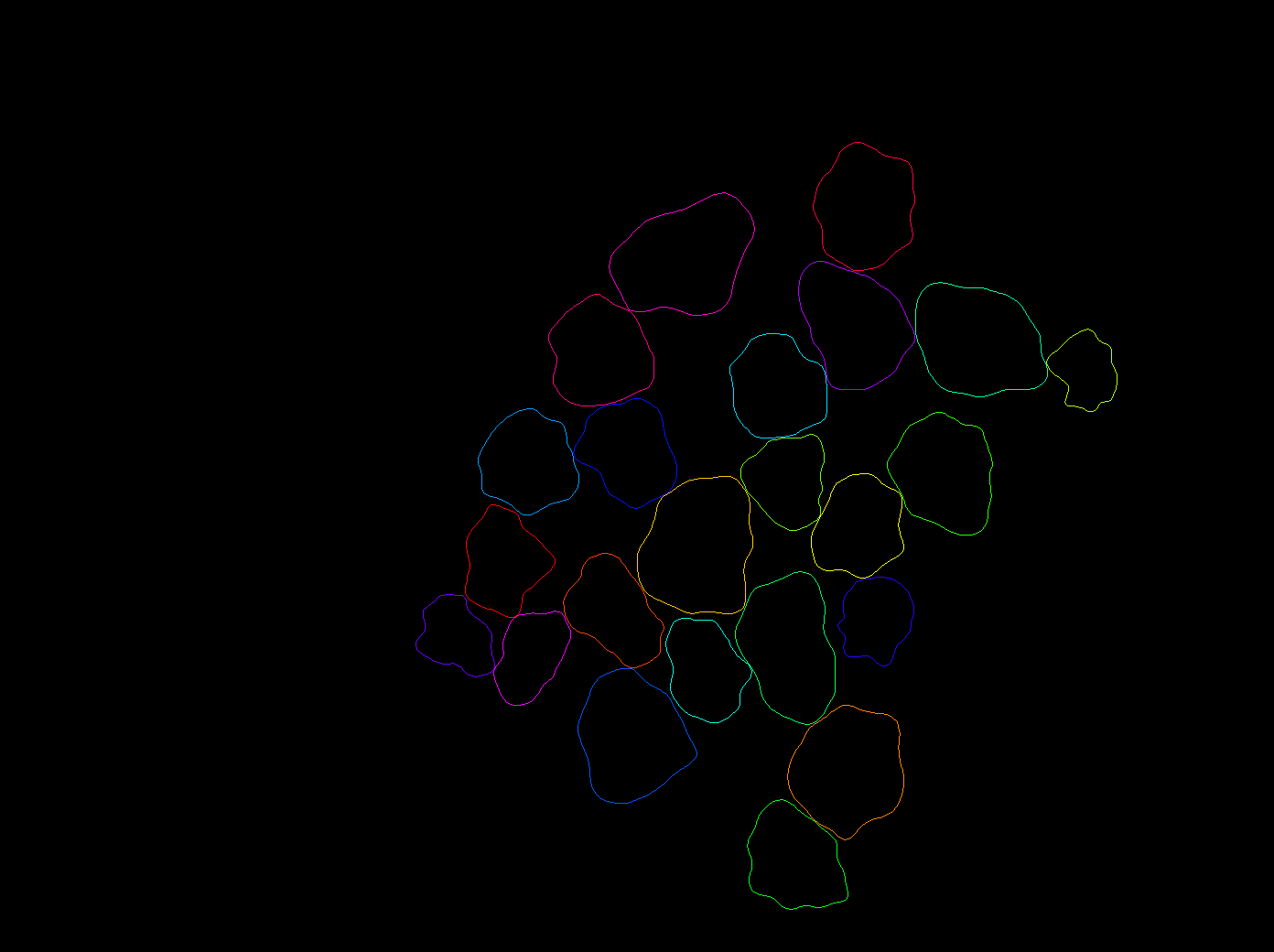

Supplement: Additional file 6 — The zip archive contains simulated images showing B cell nuclei and cytoskeleton with corresponding ground truth. (ZIP 119808 kb) [file 12859_2017_1591_MOESM6_ESM.zip › simulated B cells/cytoskeleton/not touching/cell023 gt.png]

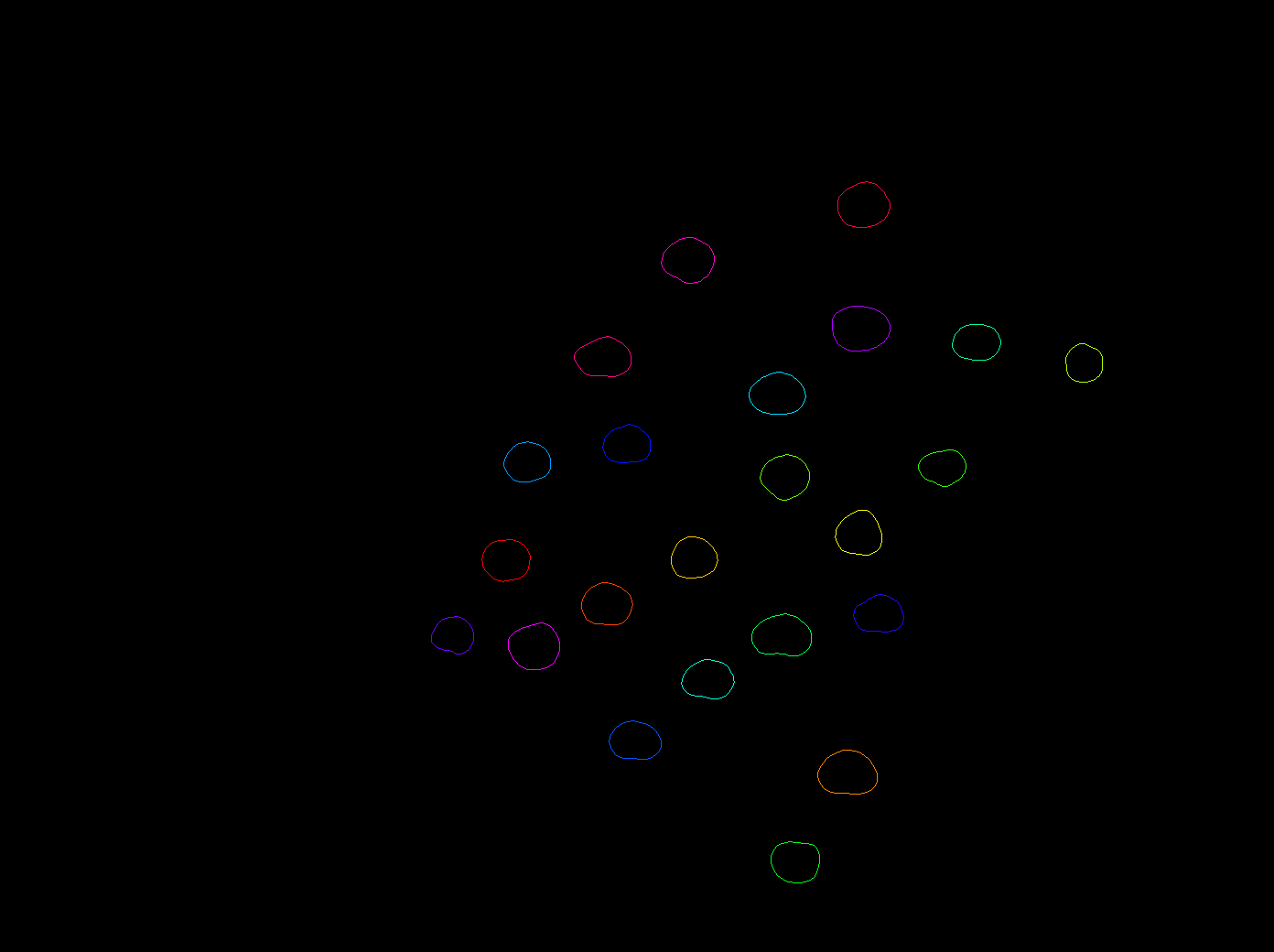

Supplement: Additional file 6 — The zip archive contains simulated images showing B cell nuclei and cytoskeleton with corresponding ground truth. (ZIP 119808 kb) [file 12859_2017_1591_MOESM6_ESM.zip › simulated B cells/cytoskeleton/not touching/cell023 seeds.png]

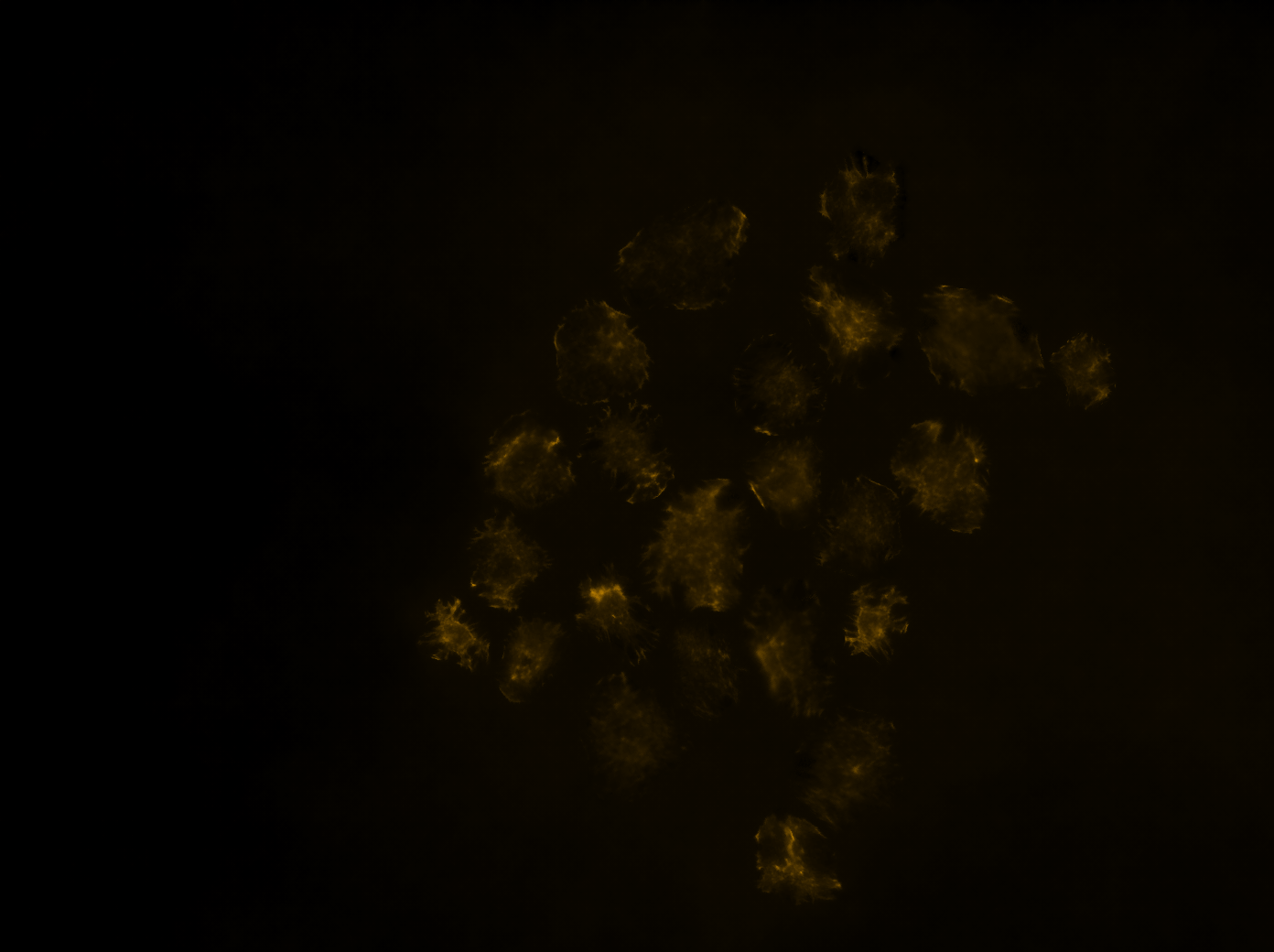

Supplement: Additional file 6 — The zip archive contains simulated images showing B cell nuclei and cytoskeleton with corresponding ground truth. (ZIP 119808 kb) [file 12859_2017_1591_MOESM6_ESM.zip › simulated B cells/cytoskeleton/not touching/cell023.png]

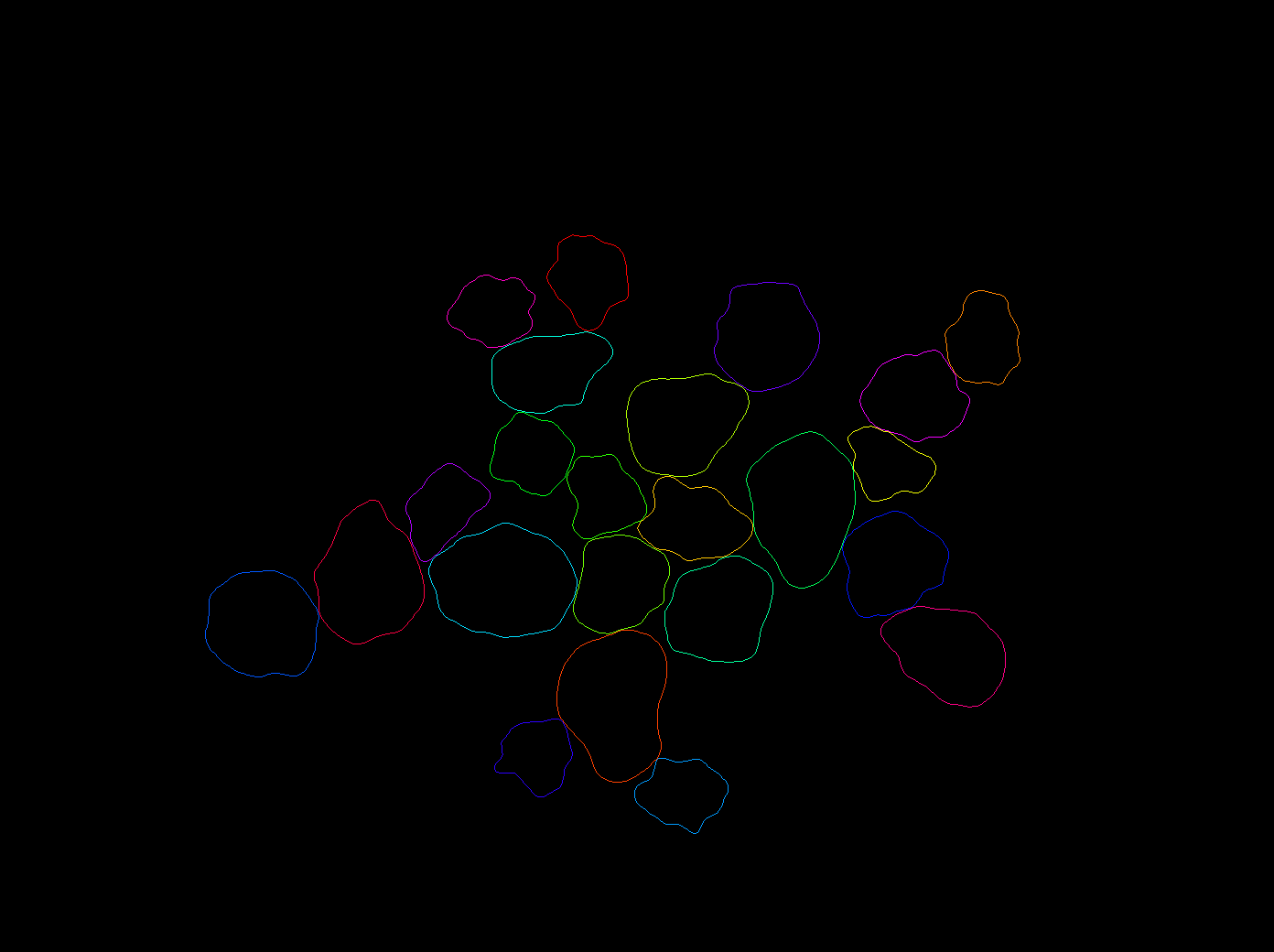

Supplement: Additional file 6 — The zip archive contains simulated images showing B cell nuclei and cytoskeleton with corresponding ground truth. (ZIP 119808 kb) [file 12859_2017_1591_MOESM6_ESM.zip › simulated B cells/cytoskeleton/not touching/cell024 gt.png]

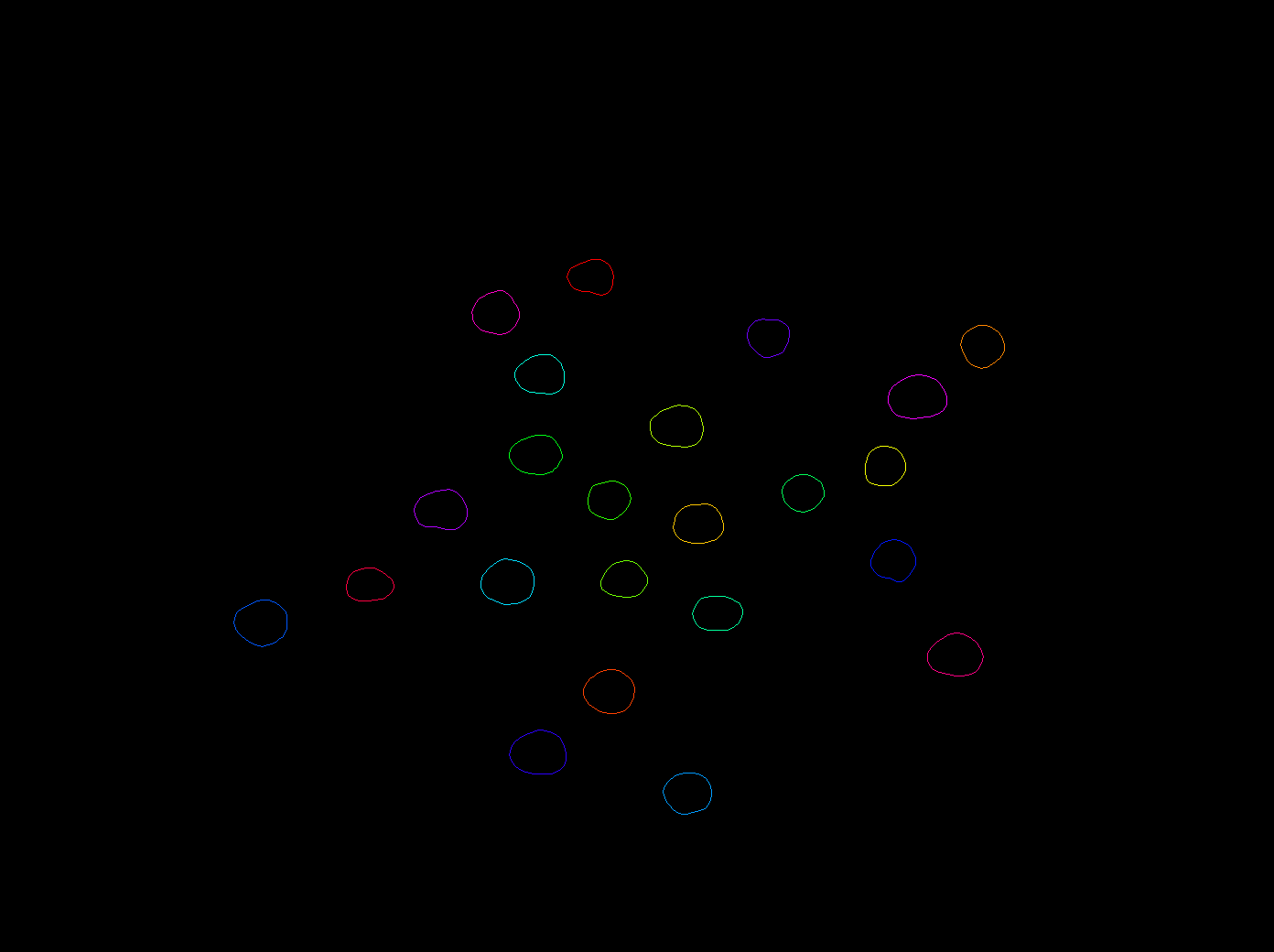

Supplement: Additional file 6 — The zip archive contains simulated images showing B cell nuclei and cytoskeleton with corresponding ground truth. (ZIP 119808 kb) [file 12859_2017_1591_MOESM6_ESM.zip › simulated B cells/cytoskeleton/not touching/cell024 seeds.png]

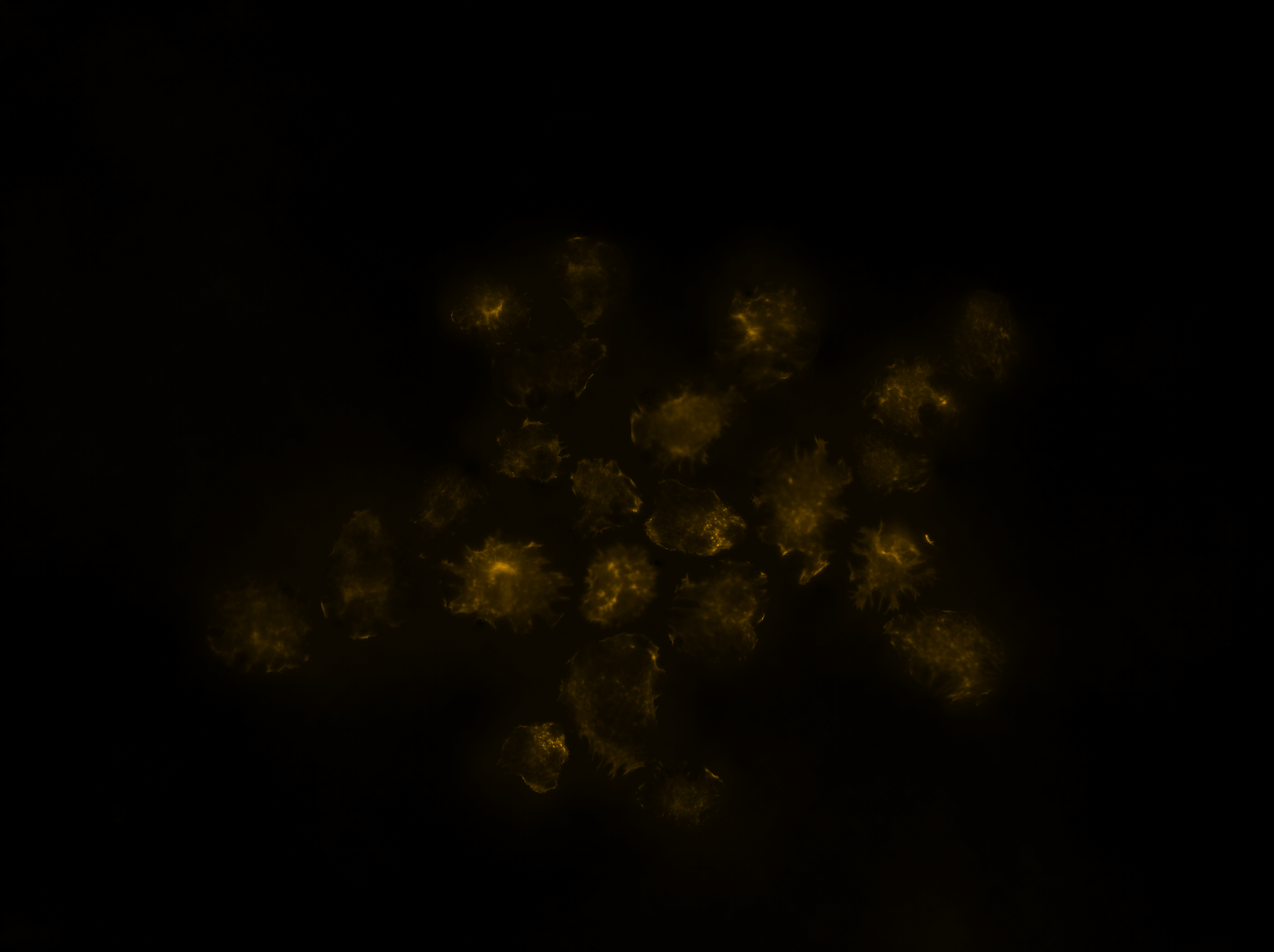

Supplement: Additional file 6 — The zip archive contains simulated images showing B cell nuclei and cytoskeleton with corresponding ground truth. (ZIP 119808 kb) [file 12859_2017_1591_MOESM6_ESM.zip › simulated B cells/cytoskeleton/not touching/cell024.png]

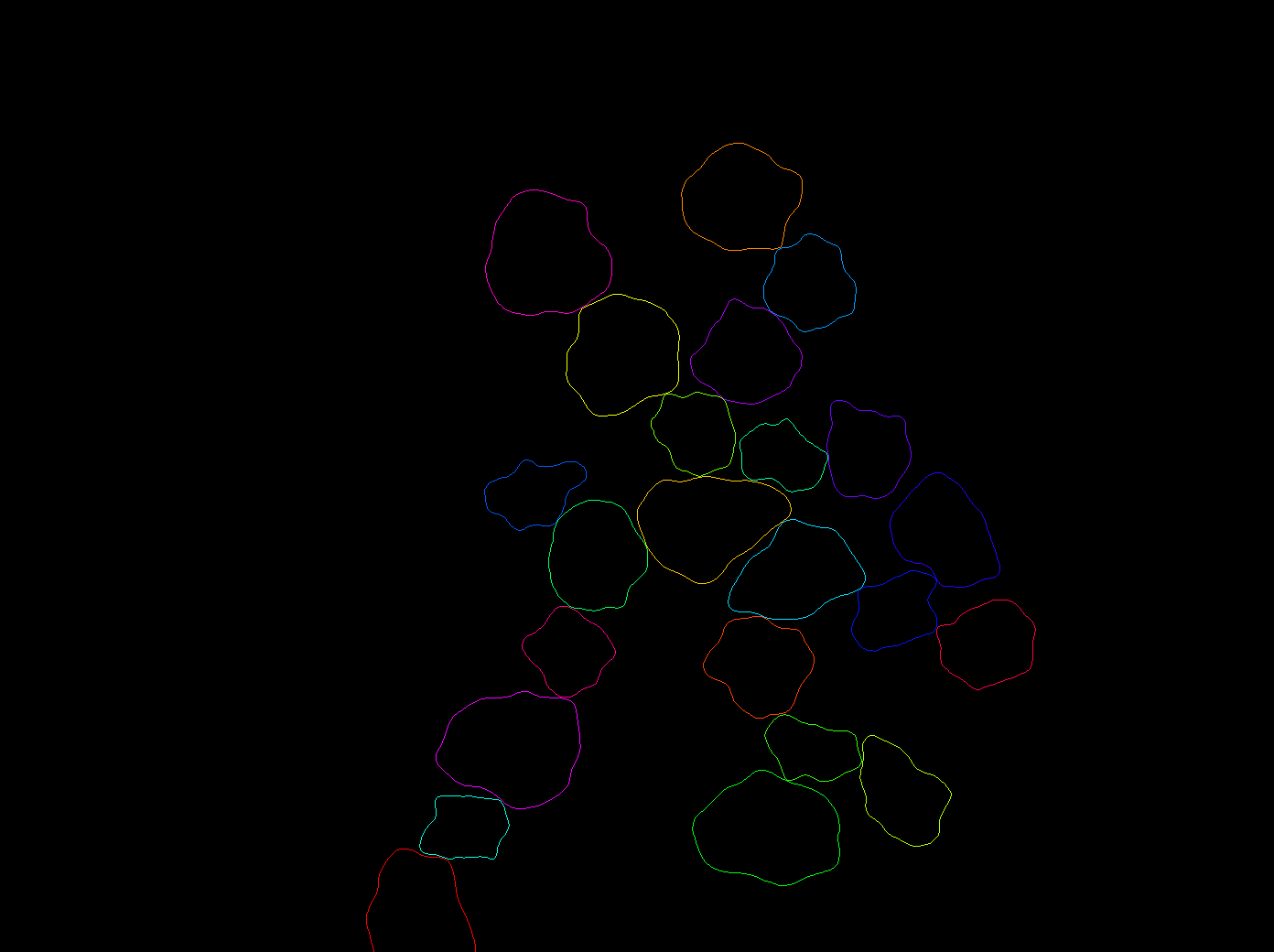

Supplement: Additional file 6 — The zip archive contains simulated images showing B cell nuclei and cytoskeleton with corresponding ground truth. (ZIP 119808 kb) [file 12859_2017_1591_MOESM6_ESM.zip › simulated B cells/cytoskeleton/not touching/cell025 gt.png]

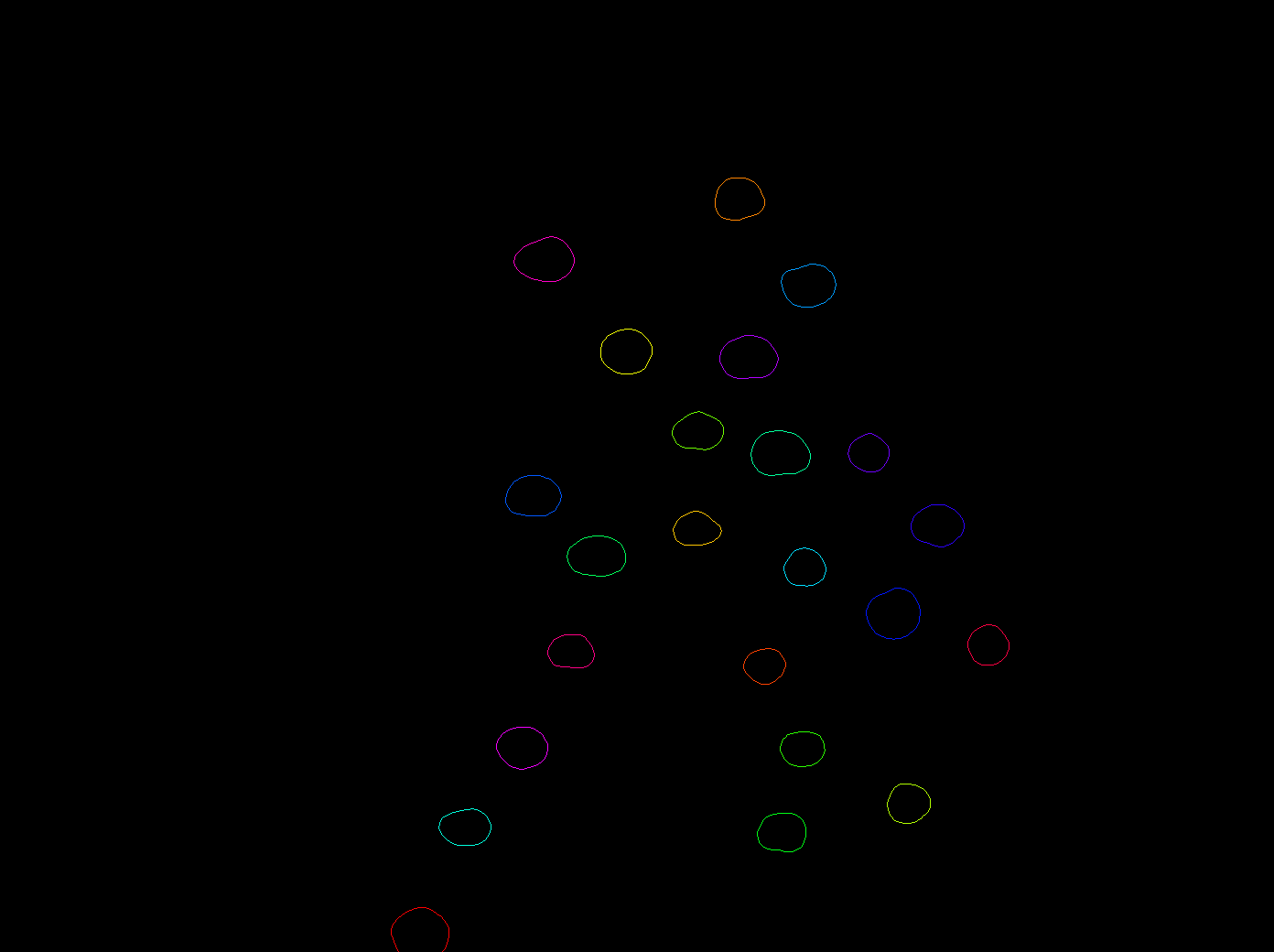

Supplement: Additional file 6 — The zip archive contains simulated images showing B cell nuclei and cytoskeleton with corresponding ground truth. (ZIP 119808 kb) [file 12859_2017_1591_MOESM6_ESM.zip › simulated B cells/cytoskeleton/not touching/cell025 seeds.png]

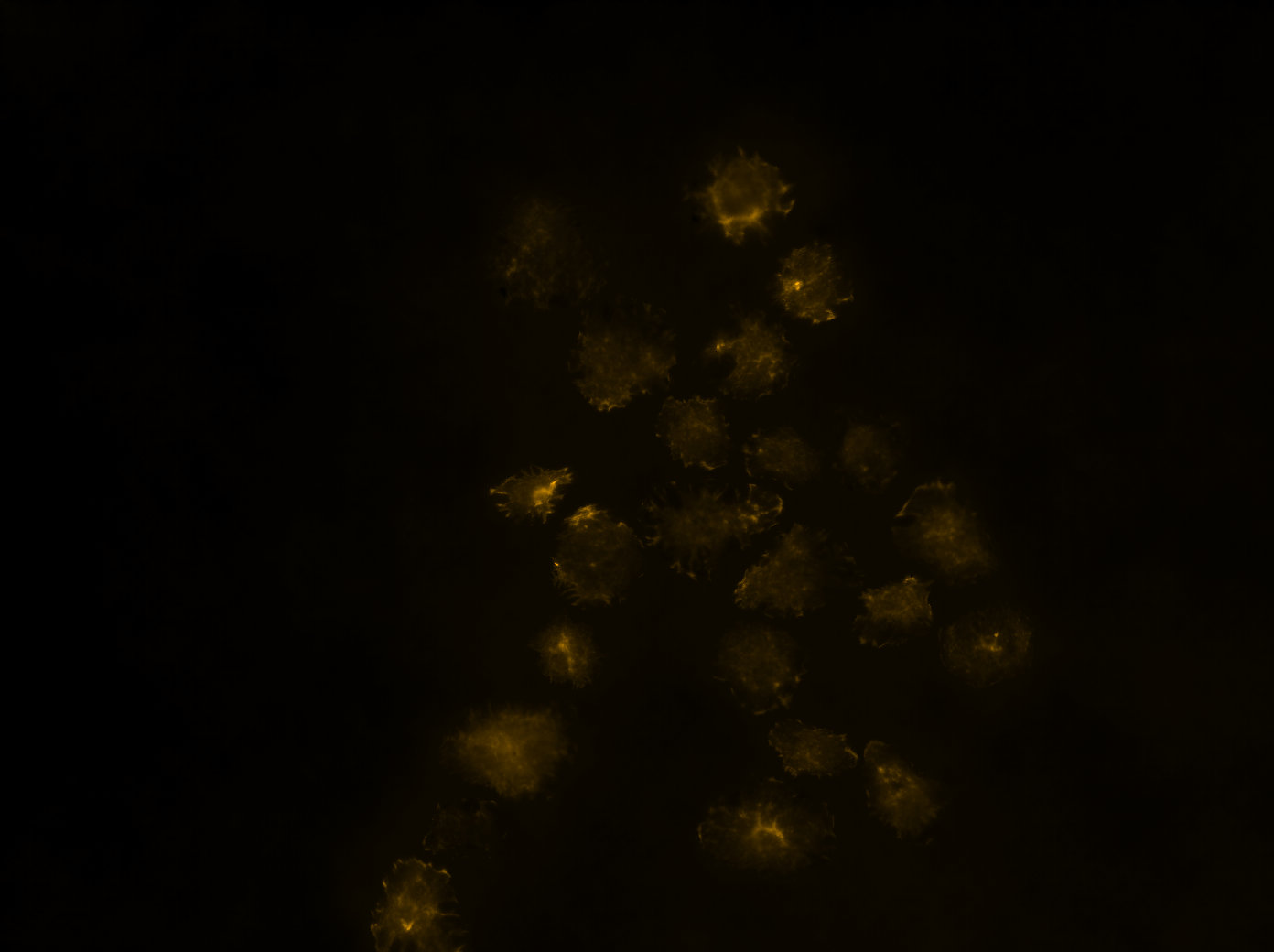

Supplement: Additional file 6 — The zip archive contains simulated images showing B cell nuclei and cytoskeleton with corresponding ground truth. (ZIP 119808 kb) [file 12859_2017_1591_MOESM6_ESM.zip › simulated B cells/cytoskeleton/not touching/cell025.png]

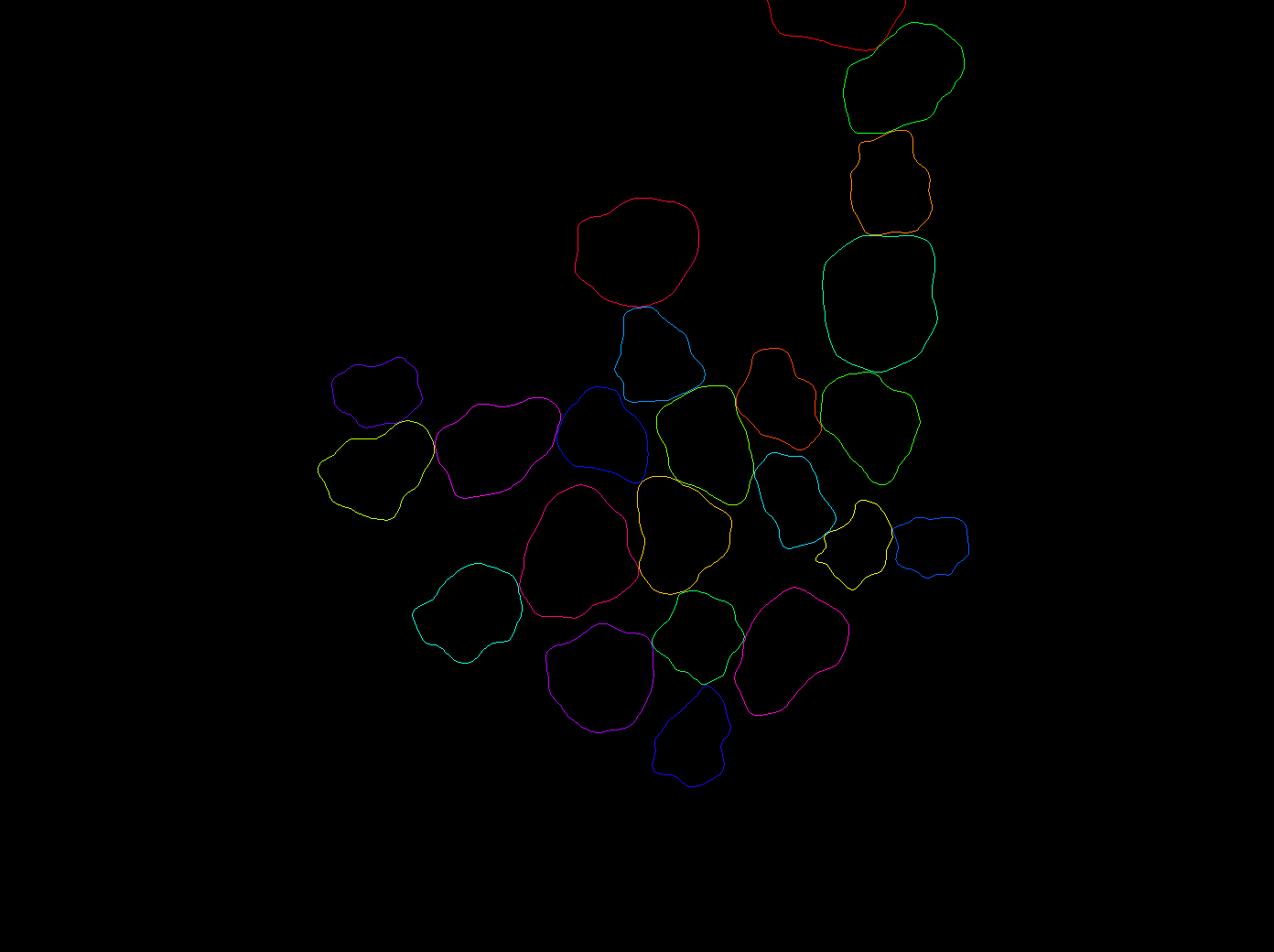

Supplement: Additional file 6 — The zip archive contains simulated images showing B cell nuclei and cytoskeleton with corresponding ground truth. (ZIP 119808 kb) [file 12859_2017_1591_MOESM6_ESM.zip › simulated B cells/cytoskeleton/not touching/cell026 gt.png]

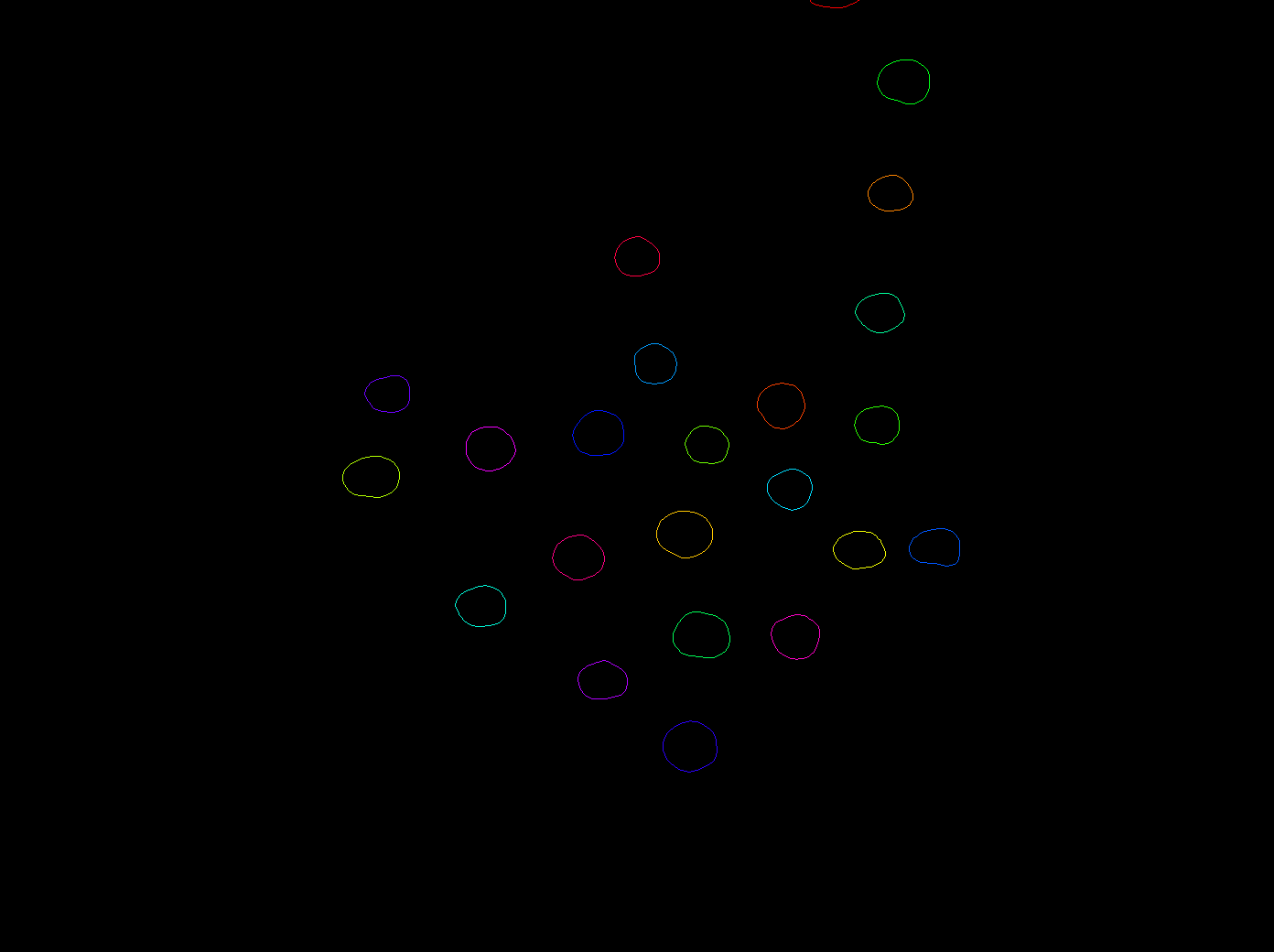

Supplement: Additional file 6 — The zip archive contains simulated images showing B cell nuclei and cytoskeleton with corresponding ground truth. (ZIP 119808 kb) [file 12859_2017_1591_MOESM6_ESM.zip › simulated B cells/cytoskeleton/not touching/cell026 seeds.png]

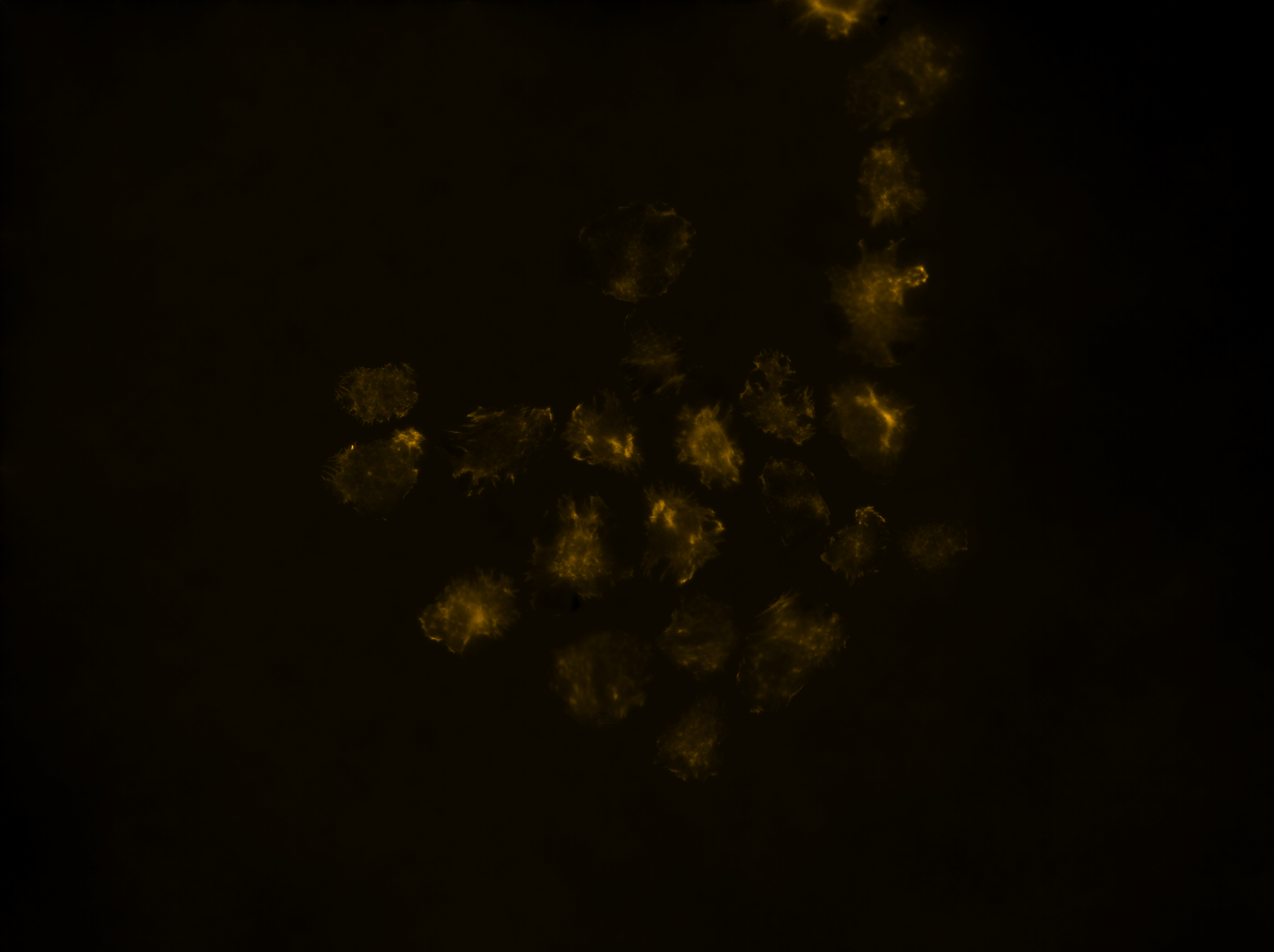

Supplement: Additional file 6 — The zip archive contains simulated images showing B cell nuclei and cytoskeleton with corresponding ground truth. (ZIP 119808 kb) [file 12859_2017_1591_MOESM6_ESM.zip › simulated B cells/cytoskeleton/not touching/cell026.png]

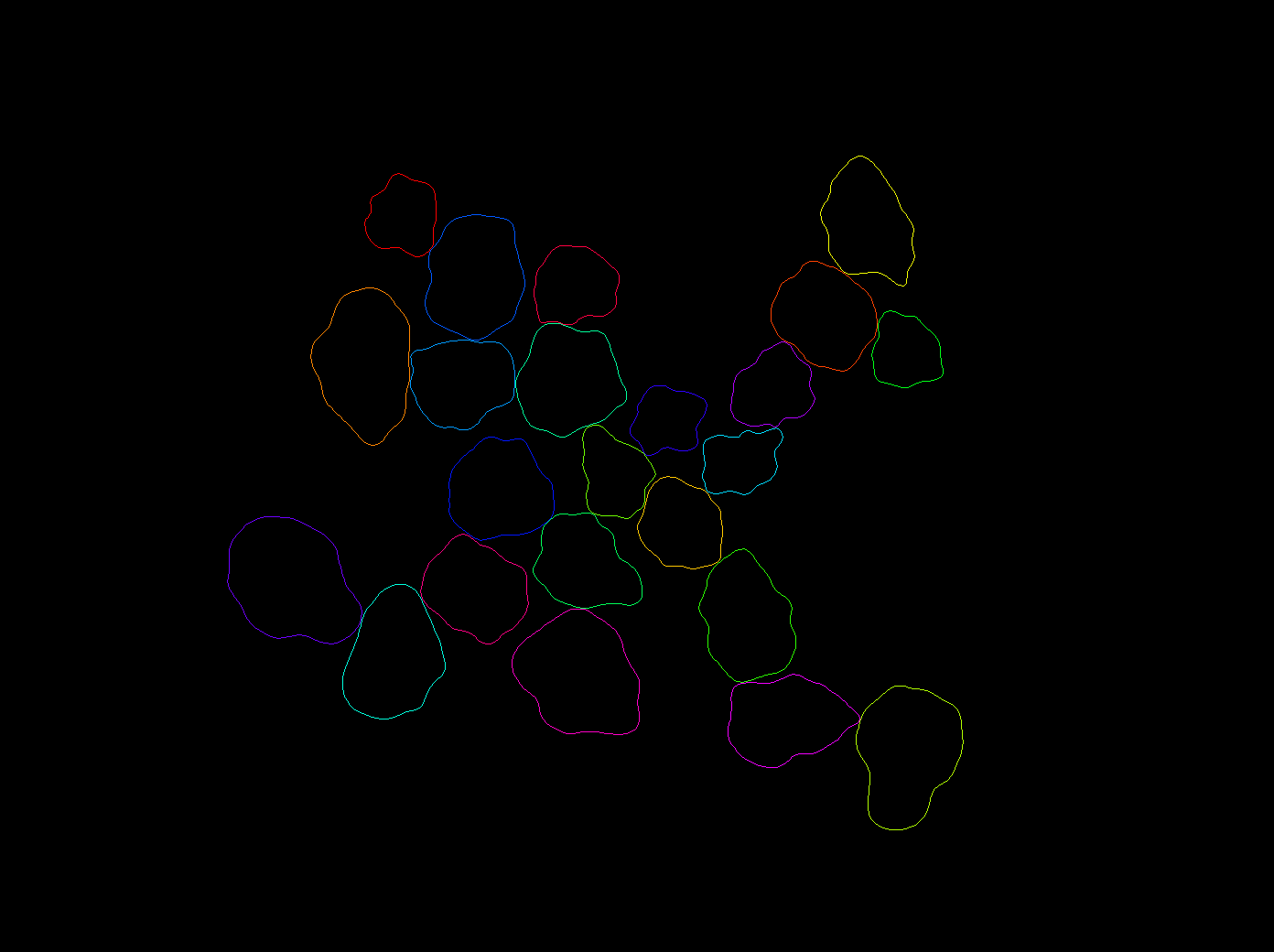

Supplement: Additional file 6 — The zip archive contains simulated images showing B cell nuclei and cytoskeleton with corresponding ground truth. (ZIP 119808 kb) [file 12859_2017_1591_MOESM6_ESM.zip › simulated B cells/cytoskeleton/not touching/cell027 gt.png]

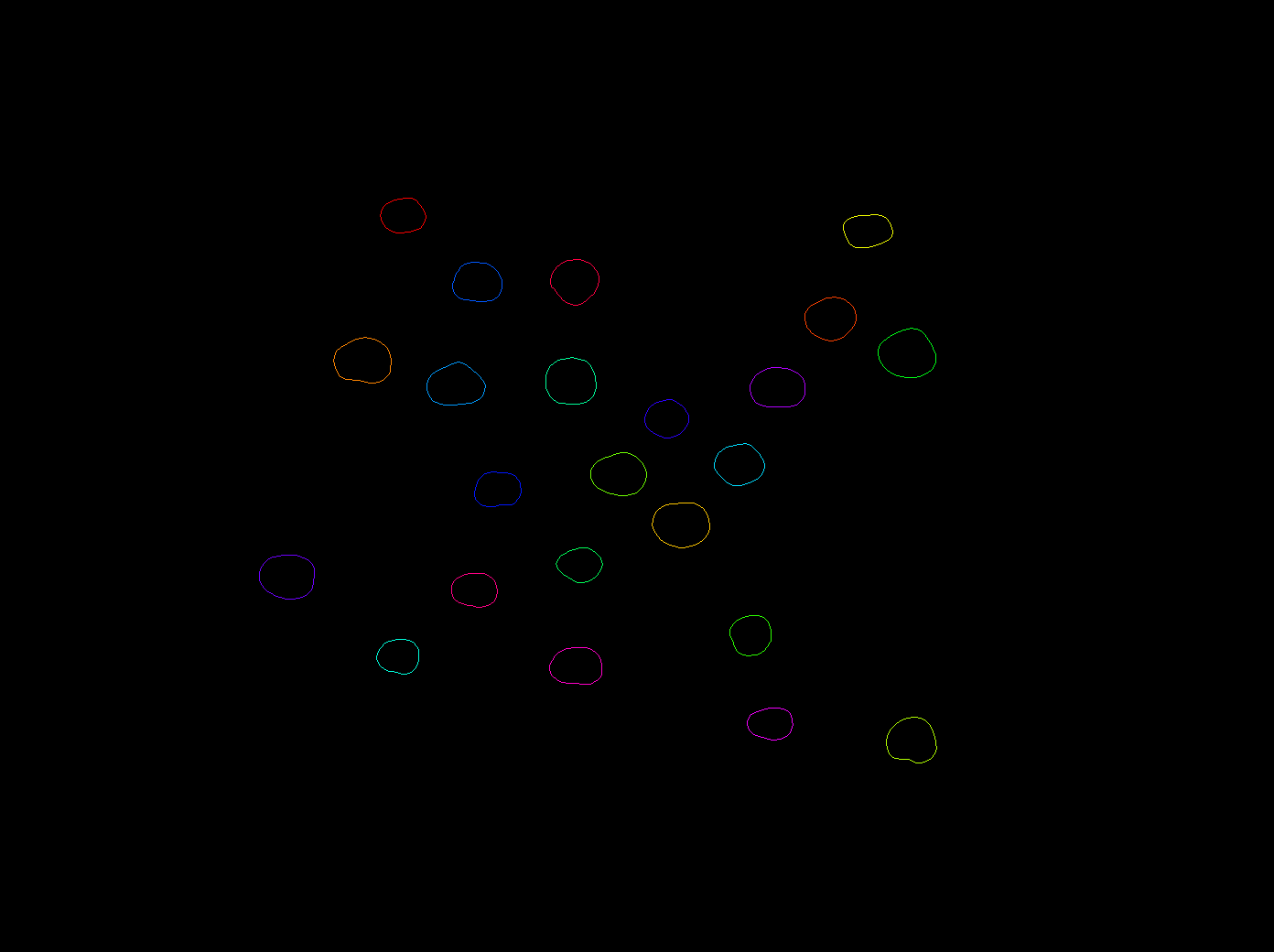

Supplement: Additional file 6 — The zip archive contains simulated images showing B cell nuclei and cytoskeleton with corresponding ground truth. (ZIP 119808 kb) [file 12859_2017_1591_MOESM6_ESM.zip › simulated B cells/cytoskeleton/not touching/cell027 seeds.png]

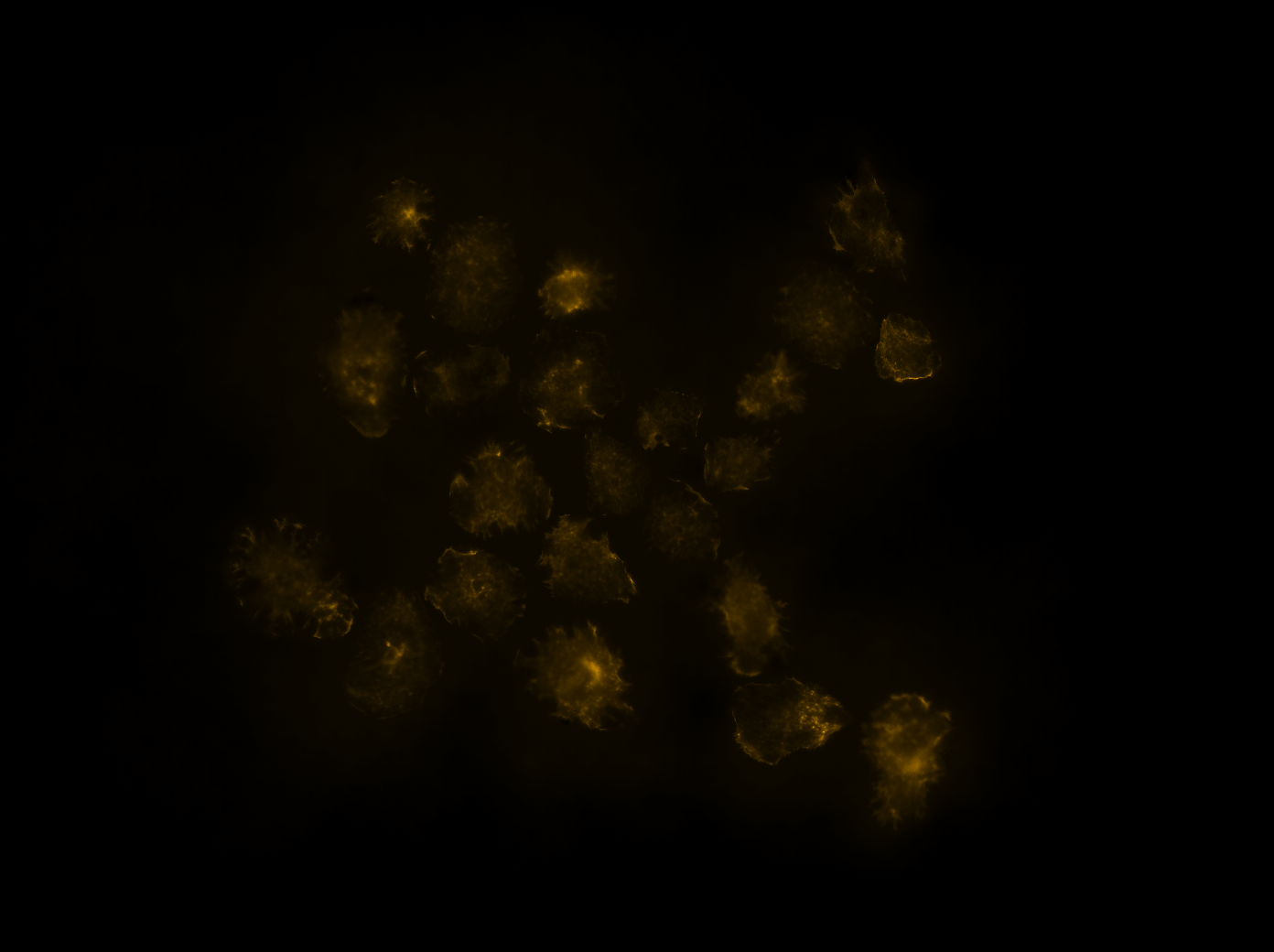

Supplement: Additional file 6 — The zip archive contains simulated images showing B cell nuclei and cytoskeleton with corresponding ground truth. (ZIP 119808 kb) [file 12859_2017_1591_MOESM6_ESM.zip › simulated B cells/cytoskeleton/not touching/cell027.png]

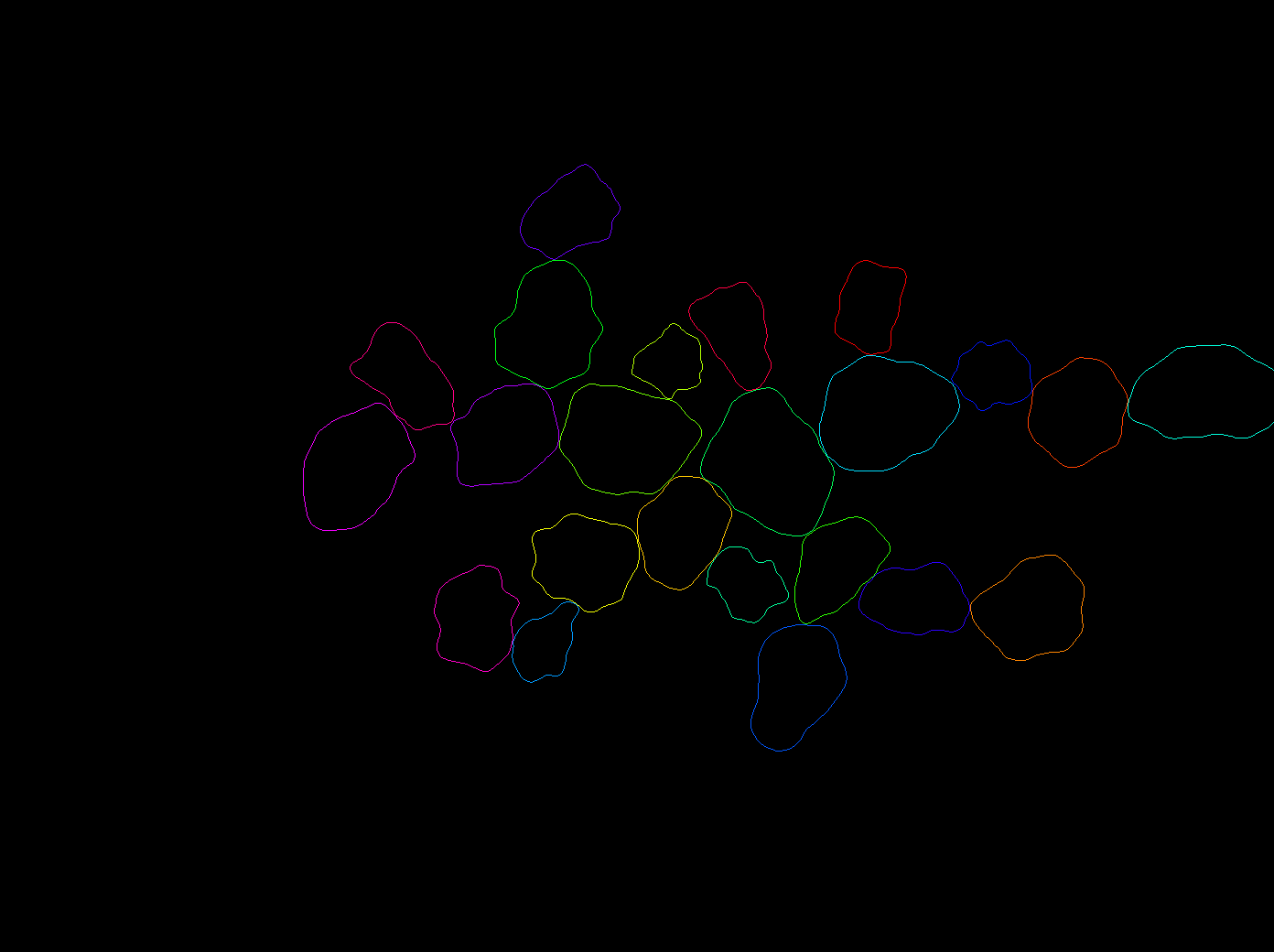

Supplement: Additional file 6 — The zip archive contains simulated images showing B cell nuclei and cytoskeleton with corresponding ground truth. (ZIP 119808 kb) [file 12859_2017_1591_MOESM6_ESM.zip › simulated B cells/cytoskeleton/not touching/cell028 gt.png]

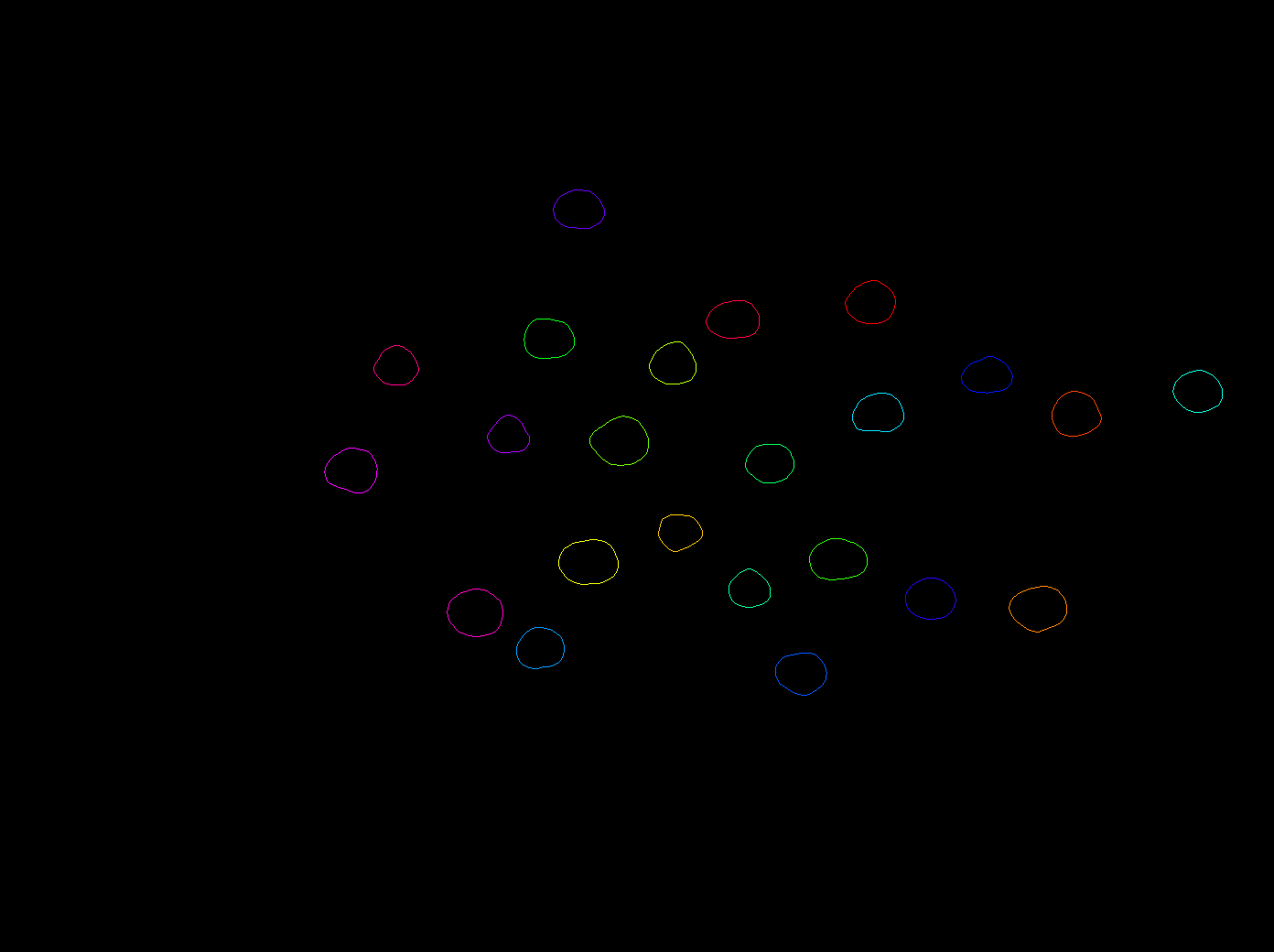

Supplement: Additional file 6 — The zip archive contains simulated images showing B cell nuclei and cytoskeleton with corresponding ground truth. (ZIP 119808 kb) [file 12859_2017_1591_MOESM6_ESM.zip › simulated B cells/cytoskeleton/not touching/cell028 seeds.png]

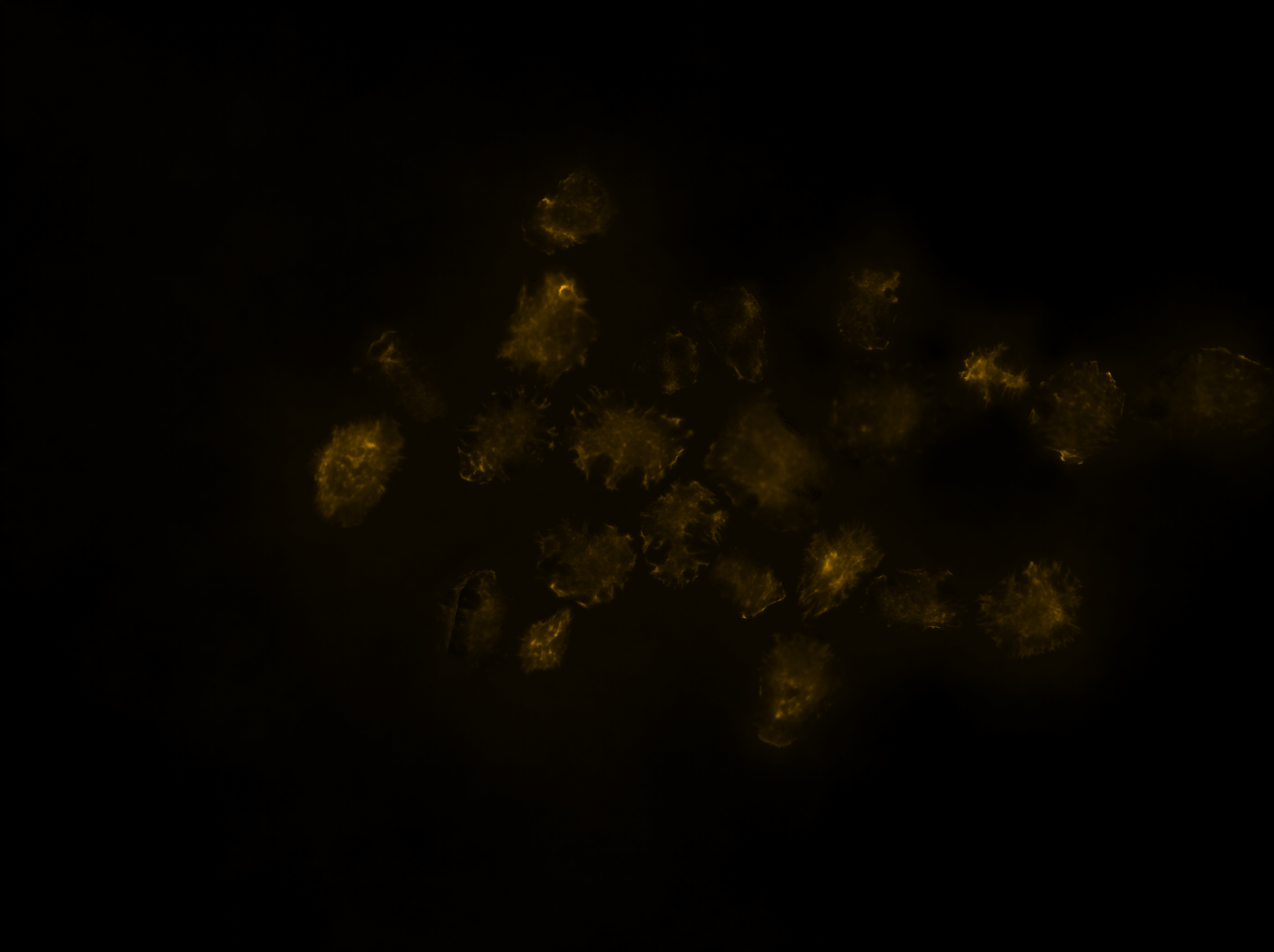

Supplement: Additional file 6 — The zip archive contains simulated images showing B cell nuclei and cytoskeleton with corresponding ground truth. (ZIP 119808 kb) [file 12859_2017_1591_MOESM6_ESM.zip › simulated B cells/cytoskeleton/not touching/cell028.png]

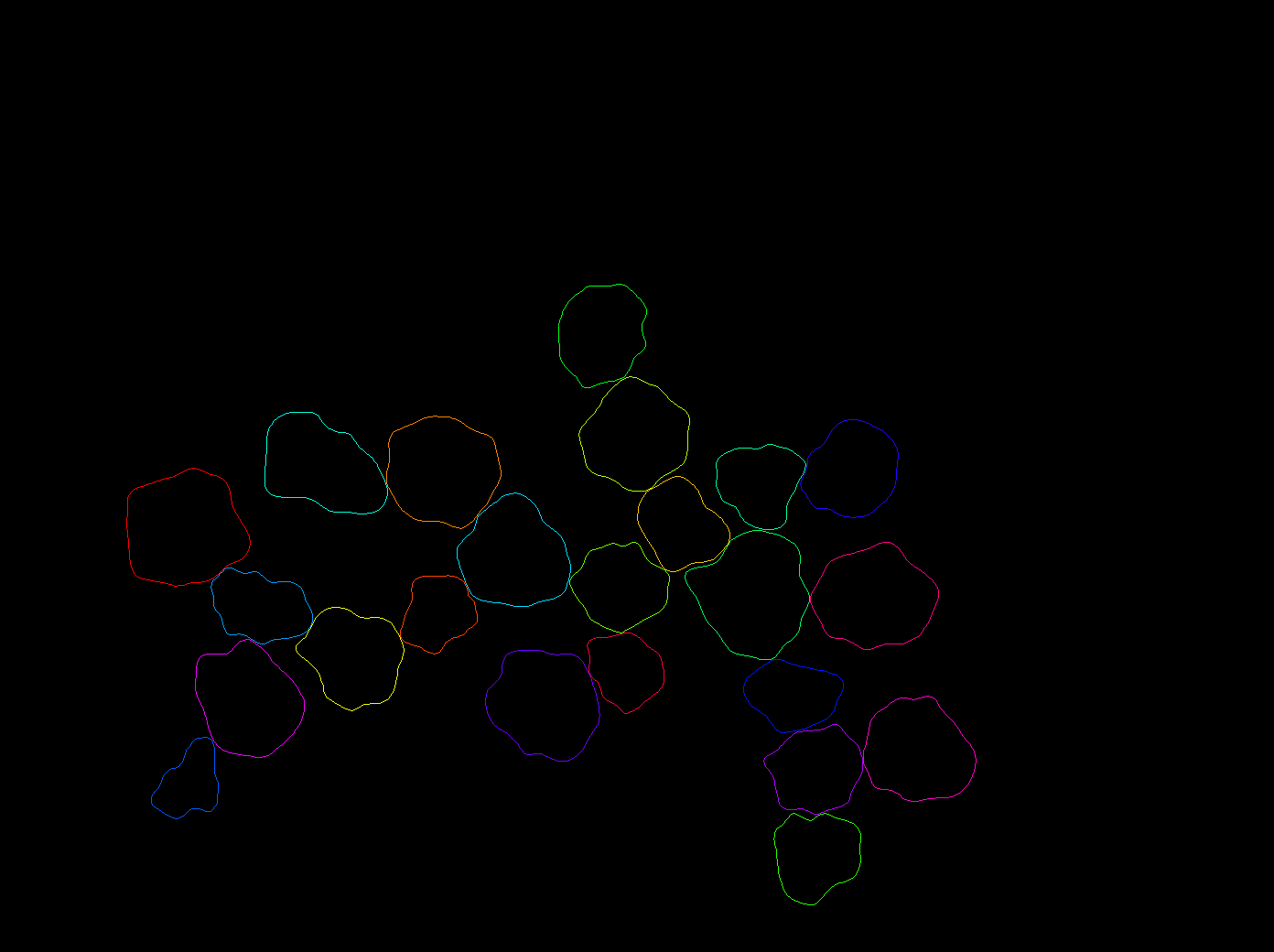

Supplement: Additional file 6 — The zip archive contains simulated images showing B cell nuclei and cytoskeleton with corresponding ground truth. (ZIP 119808 kb) [file 12859_2017_1591_MOESM6_ESM.zip › simulated B cells/cytoskeleton/not touching/cell029 gt.png]

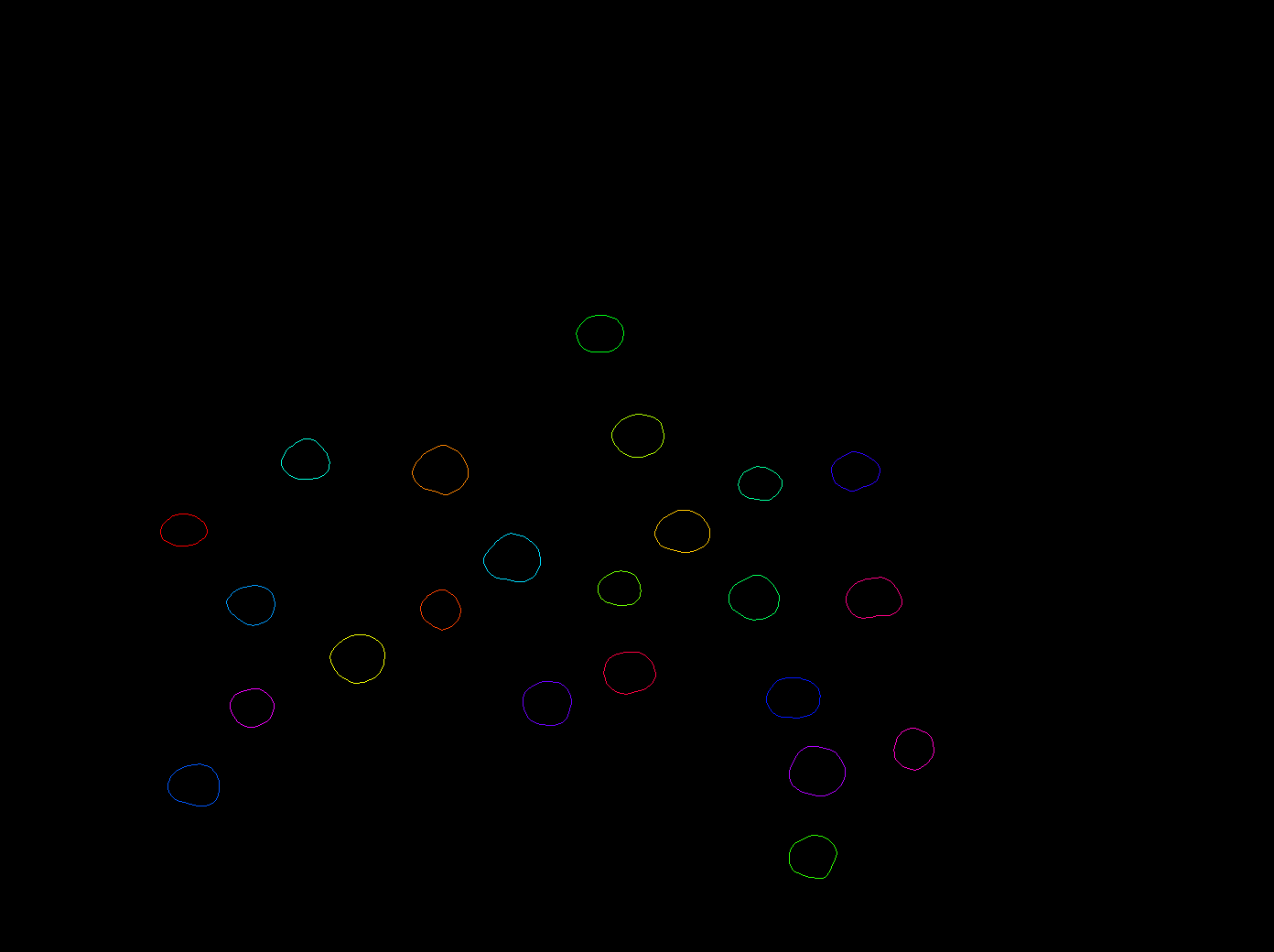

Supplement: Additional file 6 — The zip archive contains simulated images showing B cell nuclei and cytoskeleton with corresponding ground truth. (ZIP 119808 kb) [file 12859_2017_1591_MOESM6_ESM.zip › simulated B cells/cytoskeleton/not touching/cell029 seeds.png]

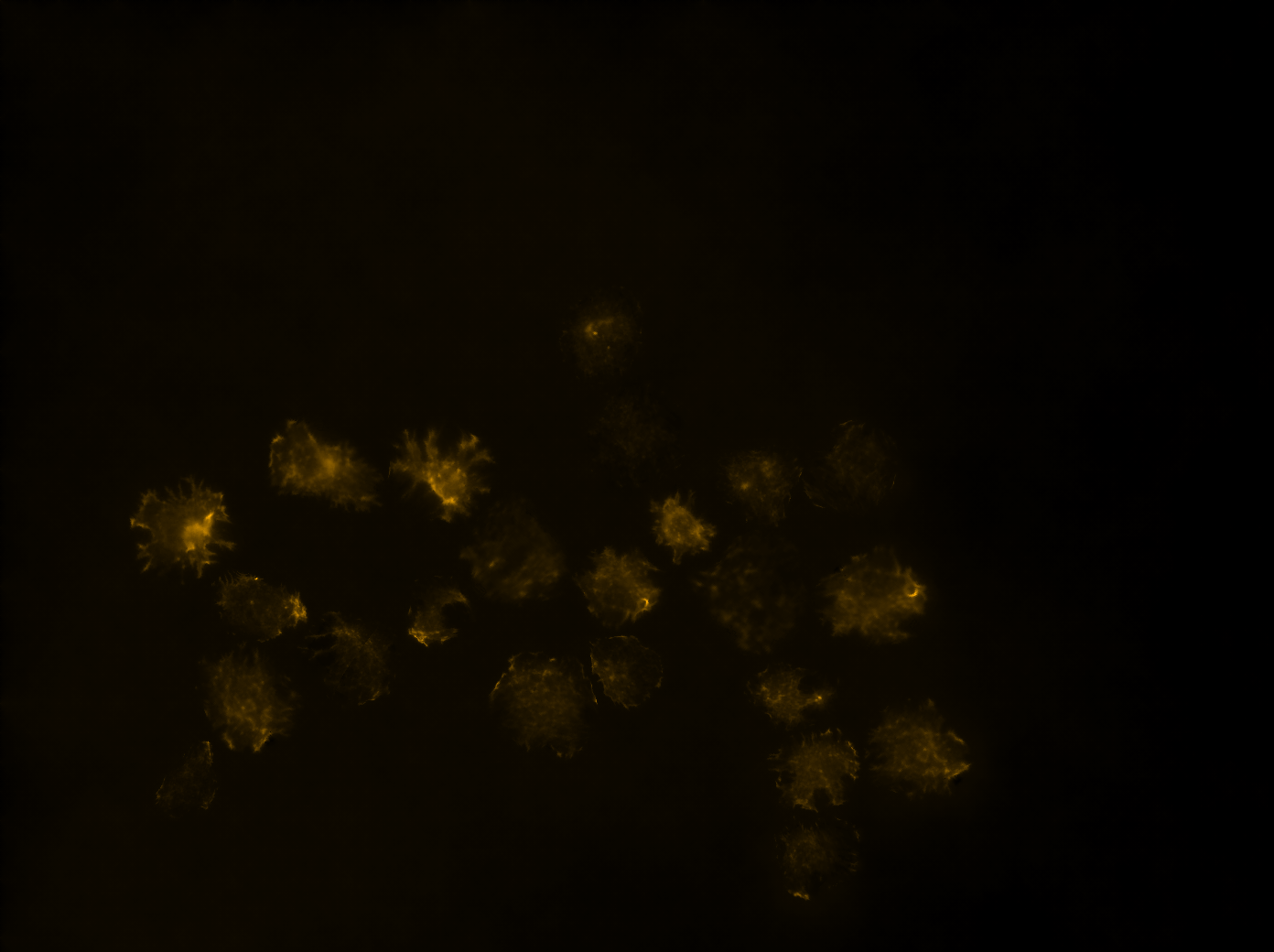

Supplement: Additional file 6 — The zip archive contains simulated images showing B cell nuclei and cytoskeleton with corresponding ground truth. (ZIP 119808 kb) [file 12859_2017_1591_MOESM6_ESM.zip › simulated B cells/cytoskeleton/not touching/cell029.png]

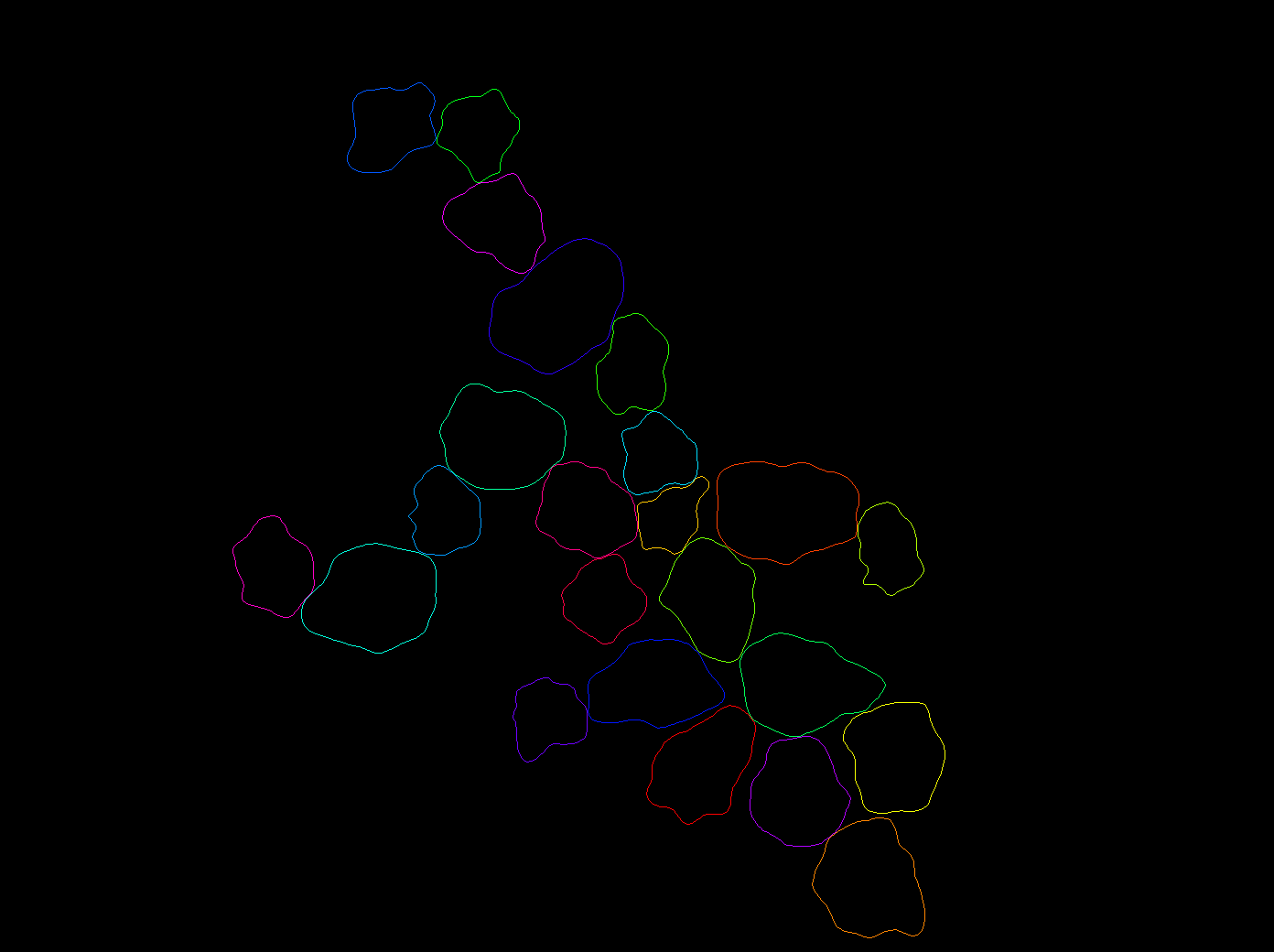

Supplement: Additional file 6 — The zip archive contains simulated images showing B cell nuclei and cytoskeleton with corresponding ground truth. (ZIP 119808 kb) [file 12859_2017_1591_MOESM6_ESM.zip › simulated B cells/cytoskeleton/not touching/cell030 gt.png]

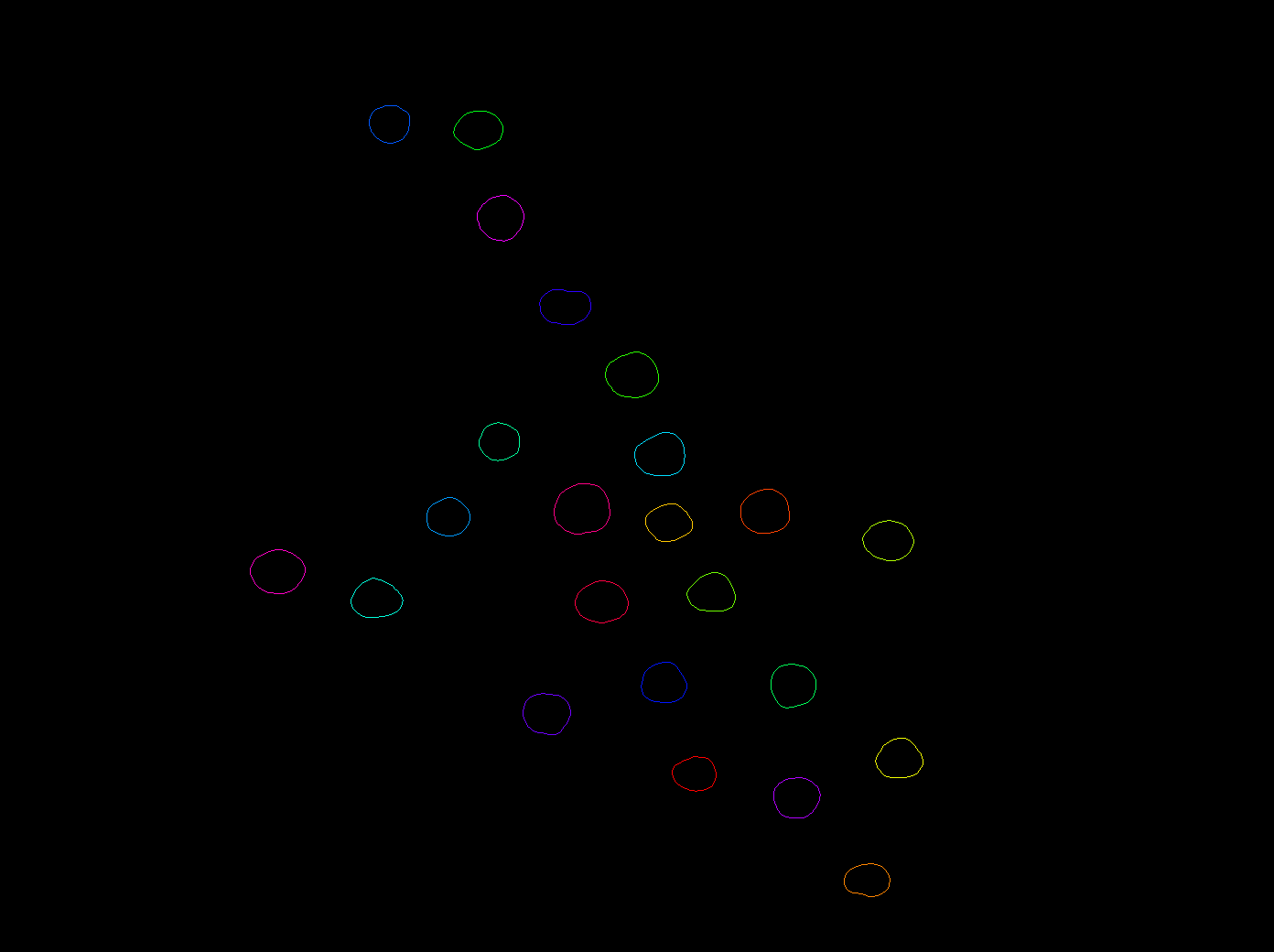

Supplement: Additional file 6 — The zip archive contains simulated images showing B cell nuclei and cytoskeleton with corresponding ground truth. (ZIP 119808 kb) [file 12859_2017_1591_MOESM6_ESM.zip › simulated B cells/cytoskeleton/not touching/cell030 seeds.png]

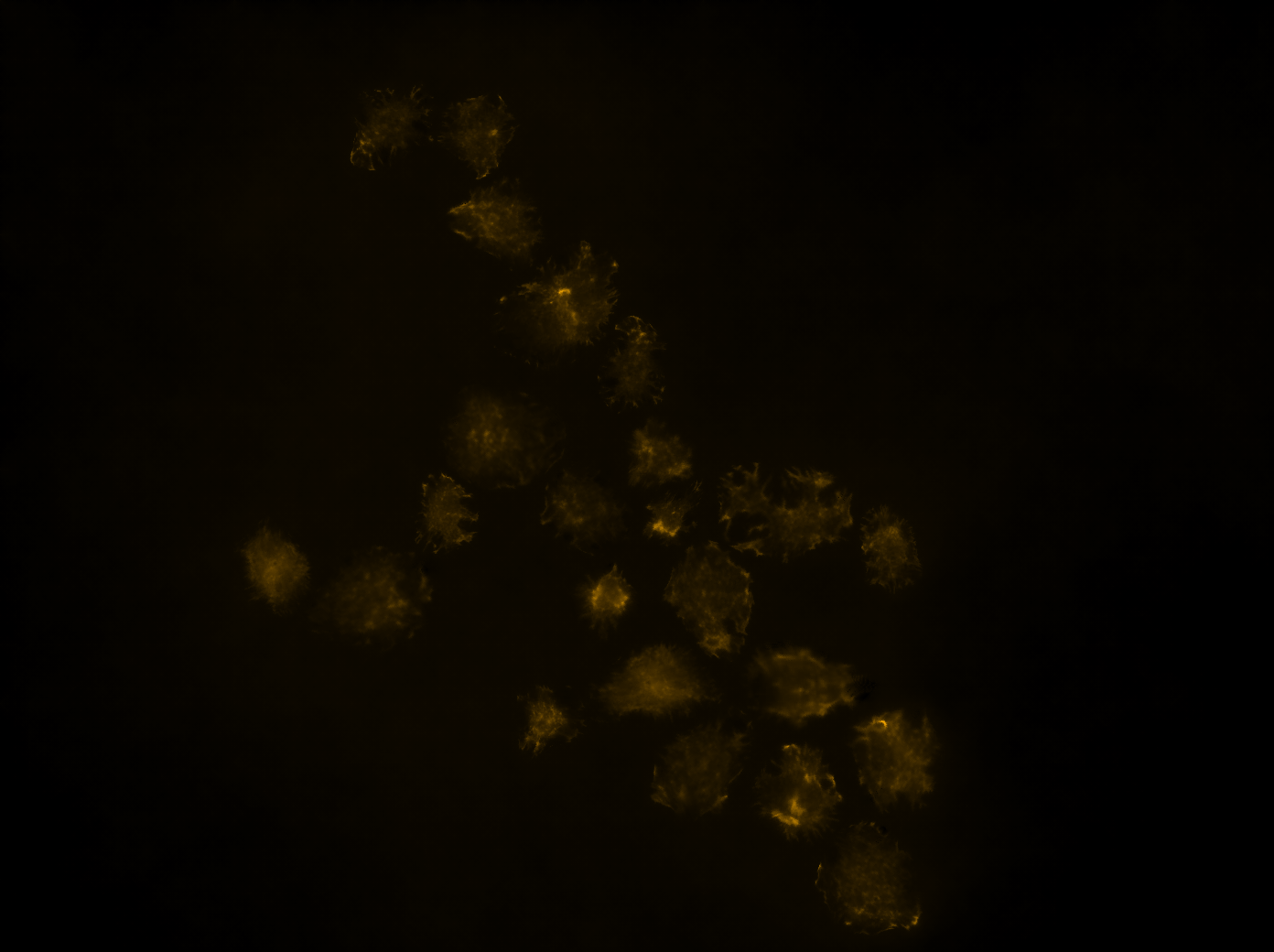

Supplement: Additional file 6 — The zip archive contains simulated images showing B cell nuclei and cytoskeleton with corresponding ground truth. (ZIP 119808 kb) [file 12859_2017_1591_MOESM6_ESM.zip › simulated B cells/cytoskeleton/not touching/cell030.png]

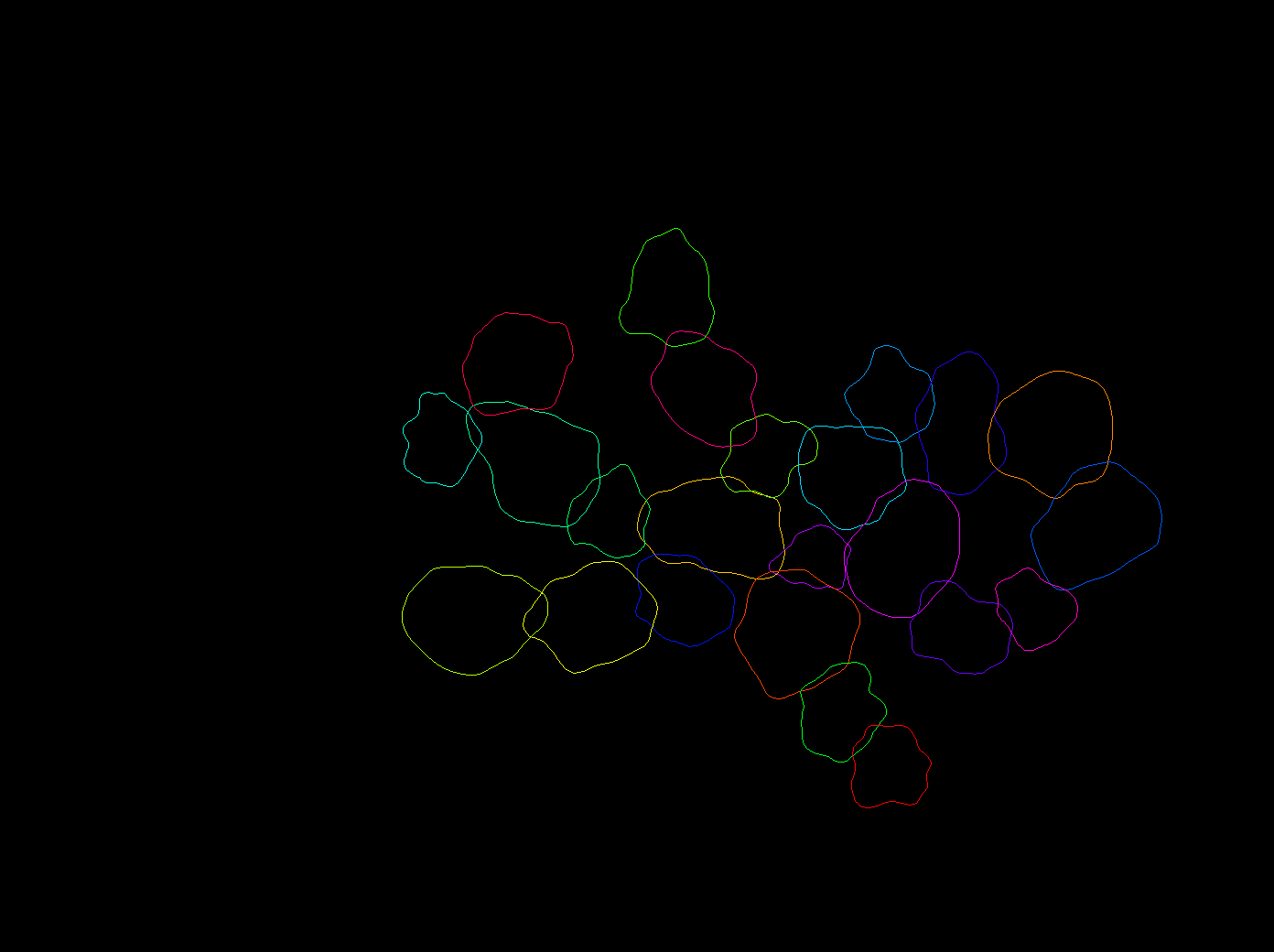

Supplement: Additional file 6 — The zip archive contains simulated images showing B cell nuclei and cytoskeleton with corresponding ground truth. (ZIP 119808 kb) [file 12859_2017_1591_MOESM6_ESM.zip › simulated B cells/cytoskeleton/overlapping/cell001 gt.png]

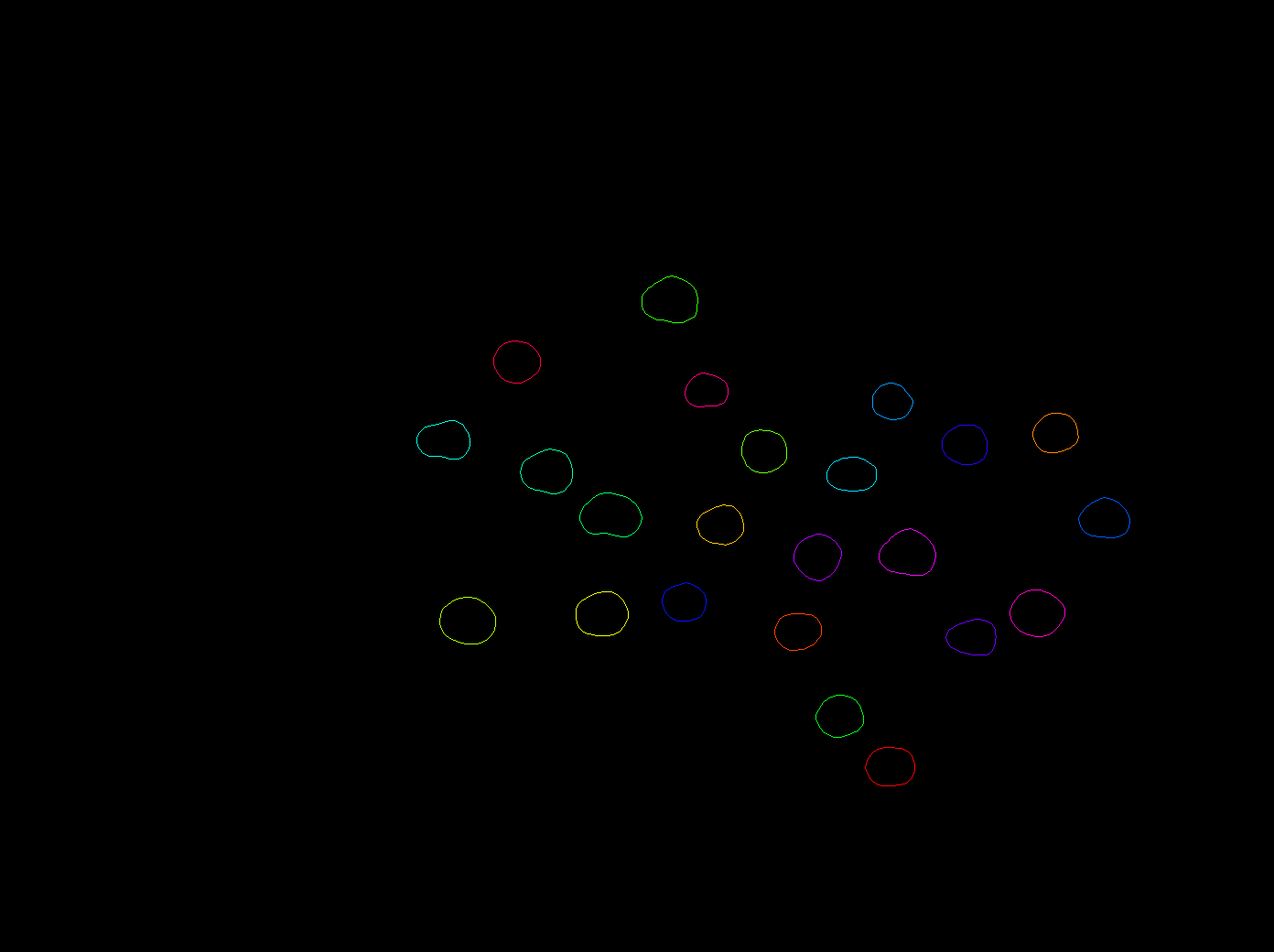

Supplement: Additional file 6 — The zip archive contains simulated images showing B cell nuclei and cytoskeleton with corresponding ground truth. (ZIP 119808 kb) [file 12859_2017_1591_MOESM6_ESM.zip › simulated B cells/cytoskeleton/overlapping/cell001 seeds.png]

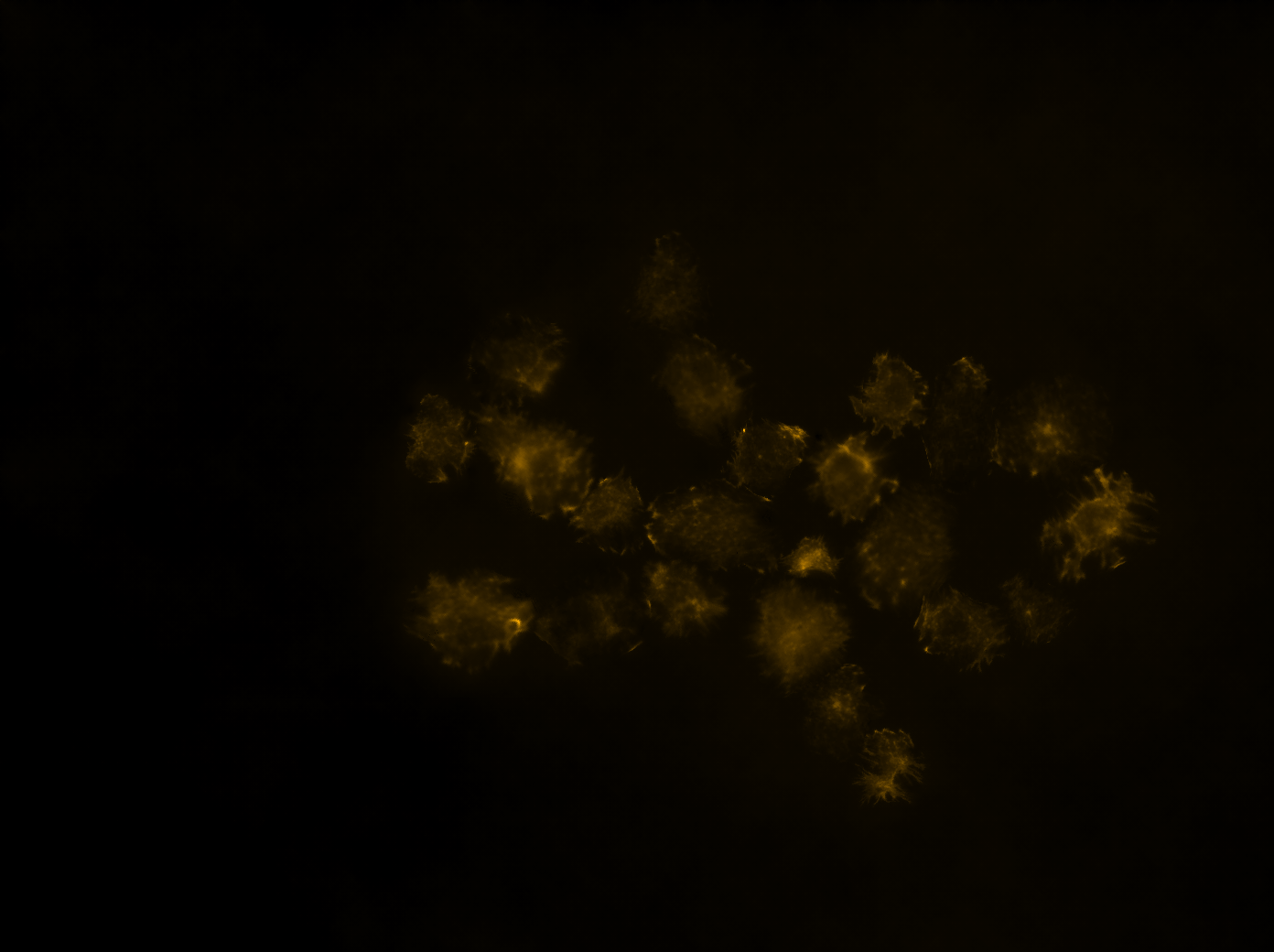

Supplement: Additional file 6 — The zip archive contains simulated images showing B cell nuclei and cytoskeleton with corresponding ground truth. (ZIP 119808 kb) [file 12859_2017_1591_MOESM6_ESM.zip › simulated B cells/cytoskeleton/overlapping/cell001.png]

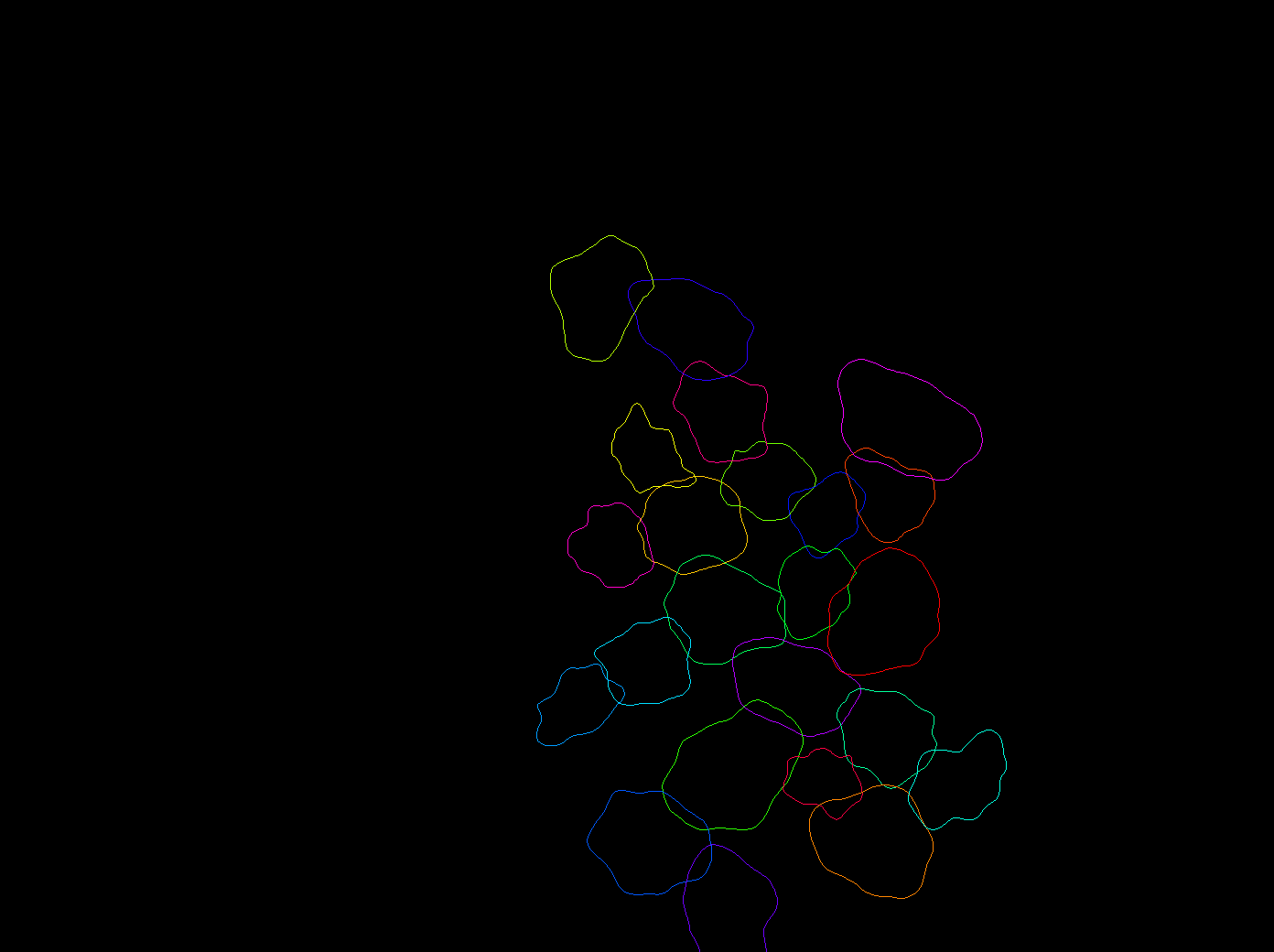

Supplement: Additional file 6 — The zip archive contains simulated images showing B cell nuclei and cytoskeleton with corresponding ground truth. (ZIP 119808 kb) [file 12859_2017_1591_MOESM6_ESM.zip › simulated B cells/cytoskeleton/overlapping/cell002 gt.png]

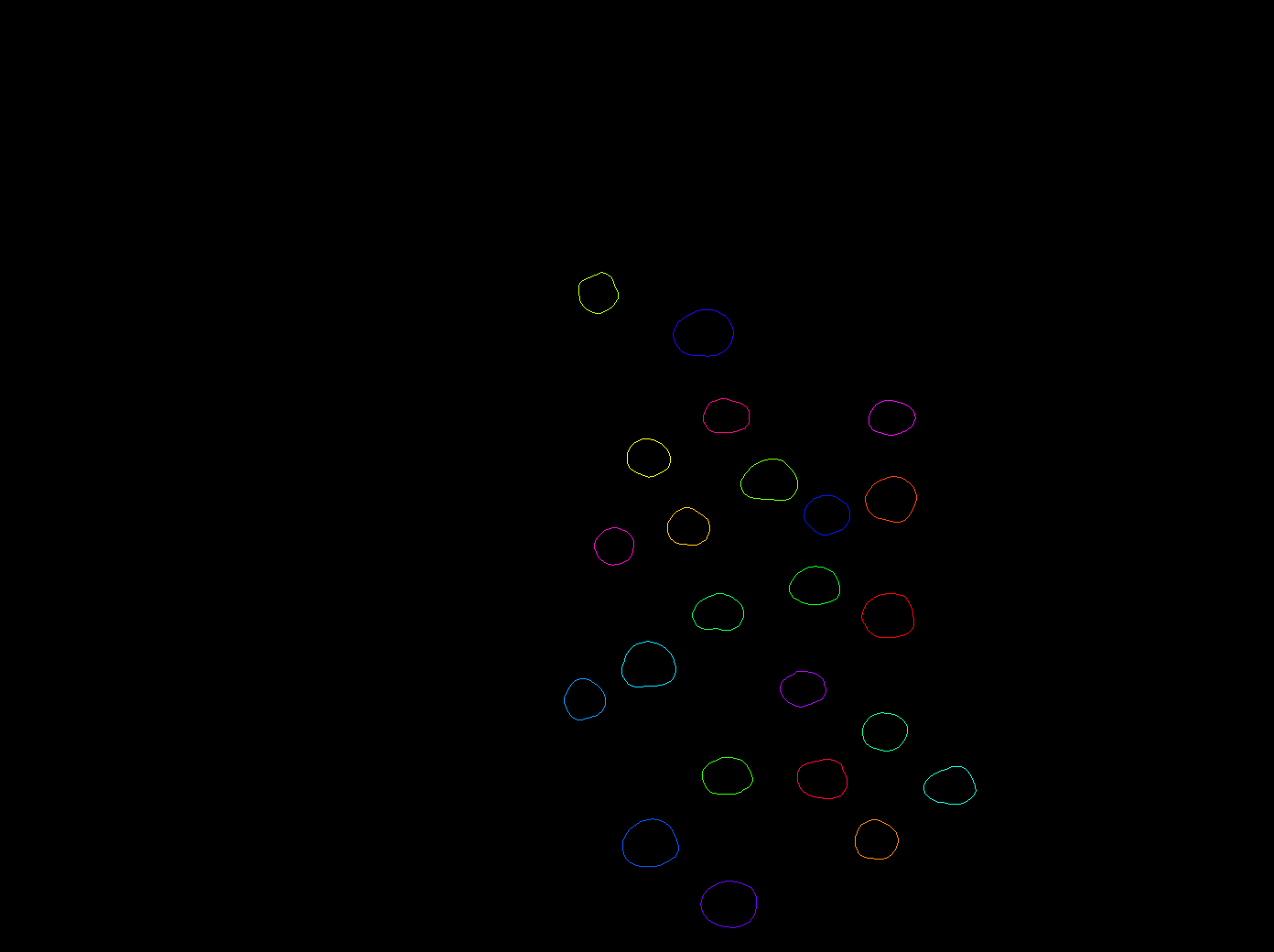

Supplement: Additional file 6 — The zip archive contains simulated images showing B cell nuclei and cytoskeleton with corresponding ground truth. (ZIP 119808 kb) [file 12859_2017_1591_MOESM6_ESM.zip › simulated B cells/cytoskeleton/overlapping/cell002 seeds.png]

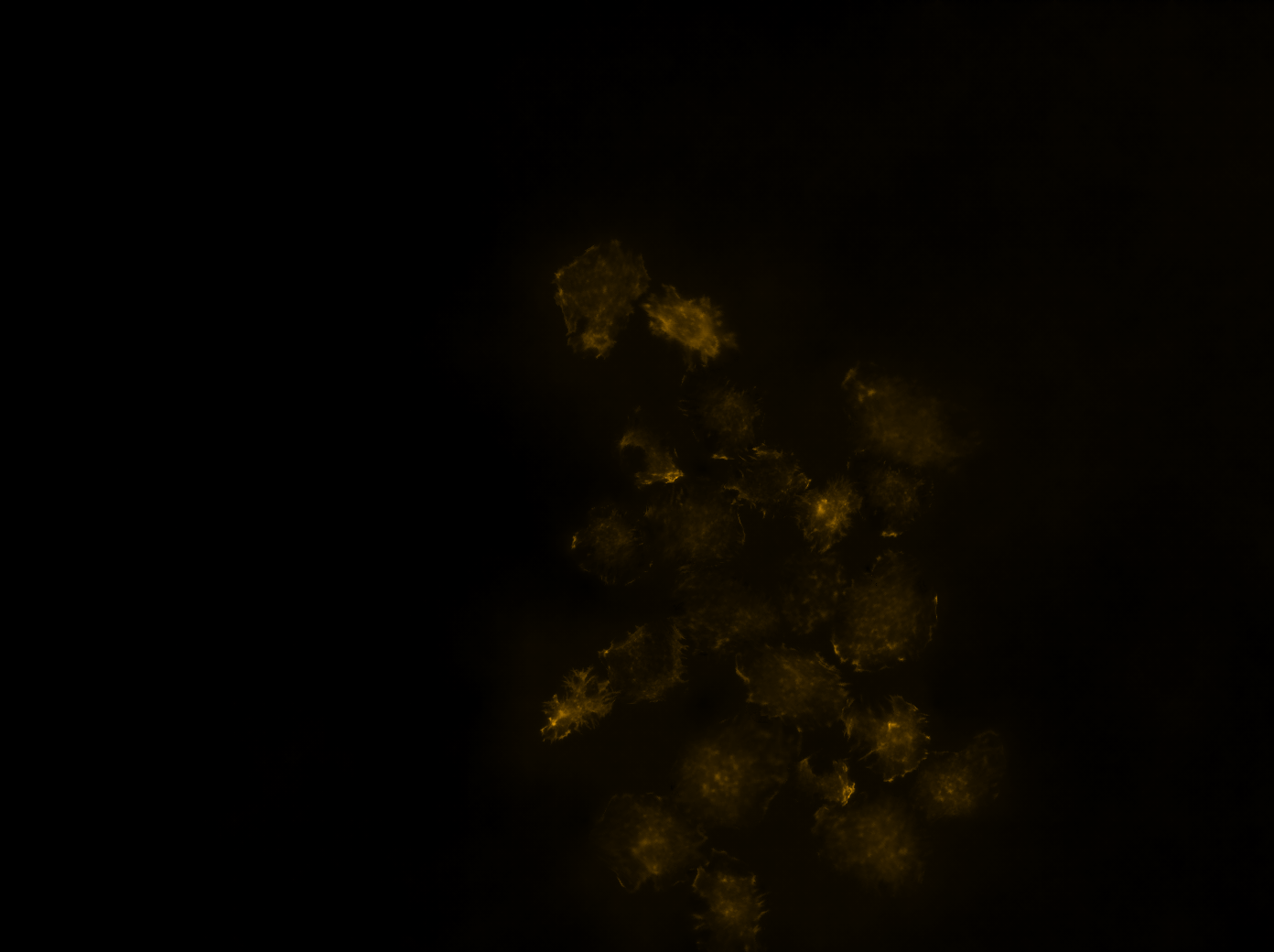

Supplement: Additional file 6 — The zip archive contains simulated images showing B cell nuclei and cytoskeleton with corresponding ground truth. (ZIP 119808 kb) [file 12859_2017_1591_MOESM6_ESM.zip › simulated B cells/cytoskeleton/overlapping/cell002.png]

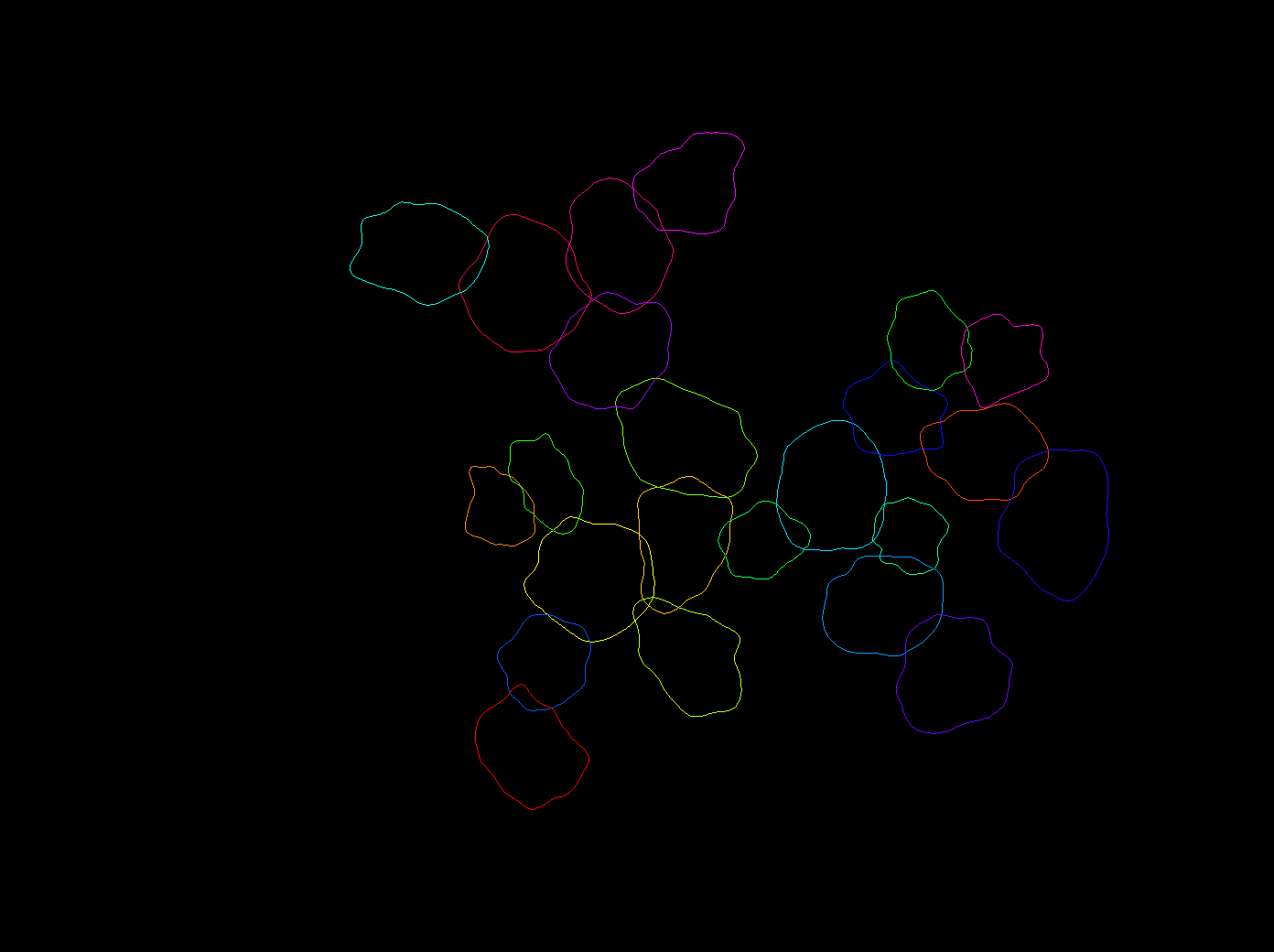

Supplement: Additional file 6 — The zip archive contains simulated images showing B cell nuclei and cytoskeleton with corresponding ground truth. (ZIP 119808 kb) [file 12859_2017_1591_MOESM6_ESM.zip › simulated B cells/cytoskeleton/overlapping/cell003 gt.png]

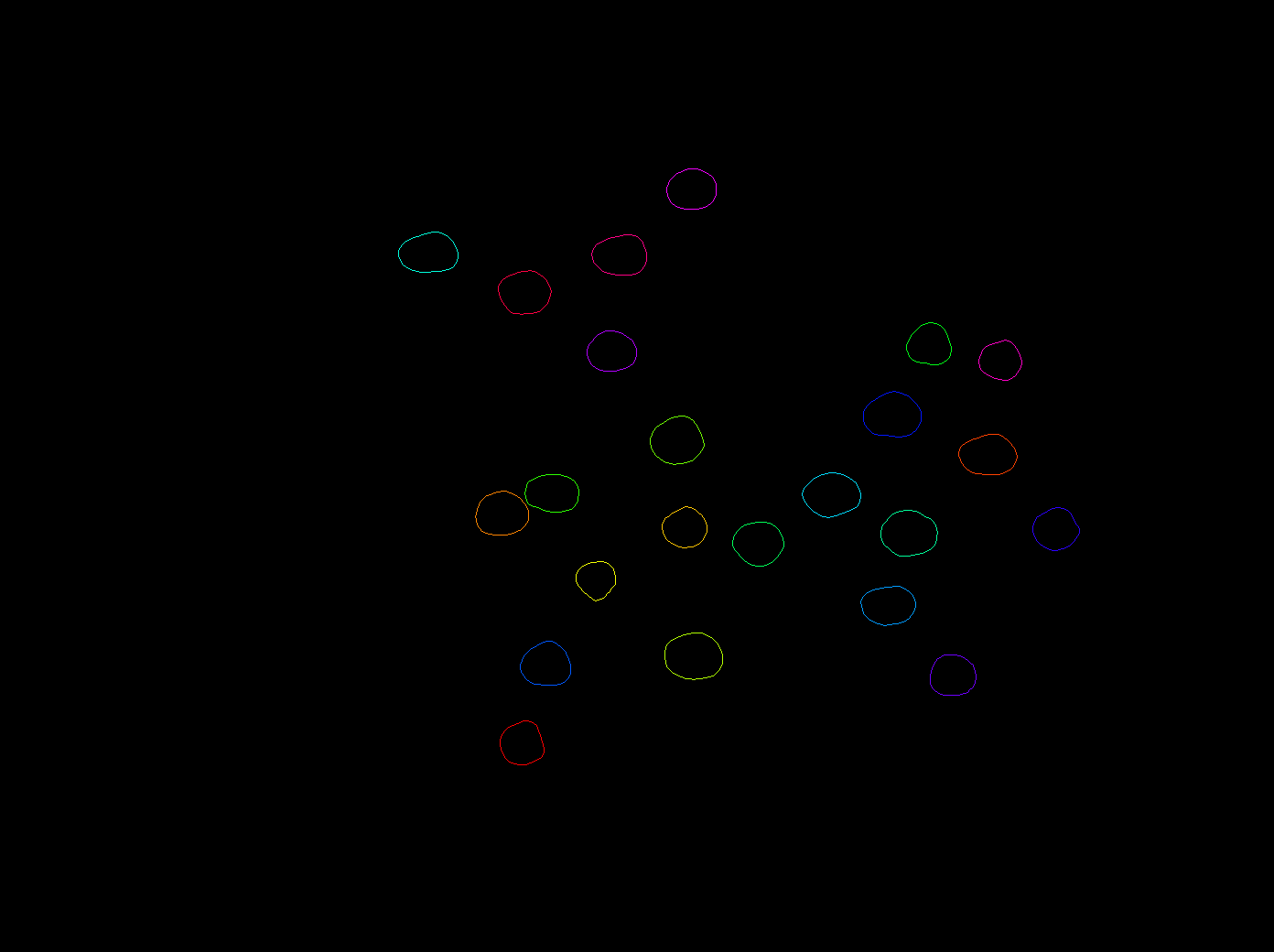

Supplement: Additional file 6 — The zip archive contains simulated images showing B cell nuclei and cytoskeleton with corresponding ground truth. (ZIP 119808 kb) [file 12859_2017_1591_MOESM6_ESM.zip › simulated B cells/cytoskeleton/overlapping/cell003 seeds.png]

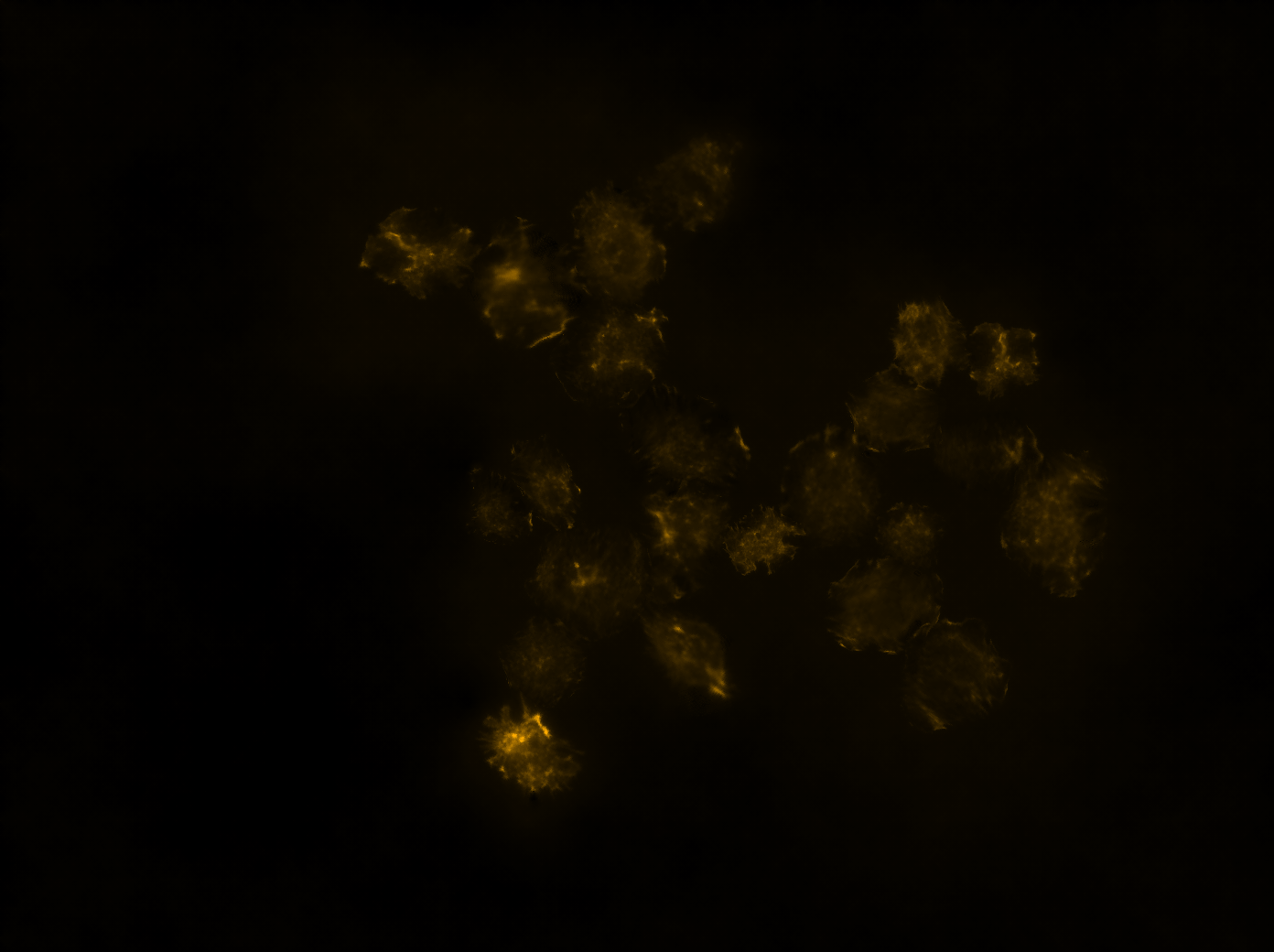

Supplement: Additional file 6 — The zip archive contains simulated images showing B cell nuclei and cytoskeleton with corresponding ground truth. (ZIP 119808 kb) [file 12859_2017_1591_MOESM6_ESM.zip › simulated B cells/cytoskeleton/overlapping/cell003.png]

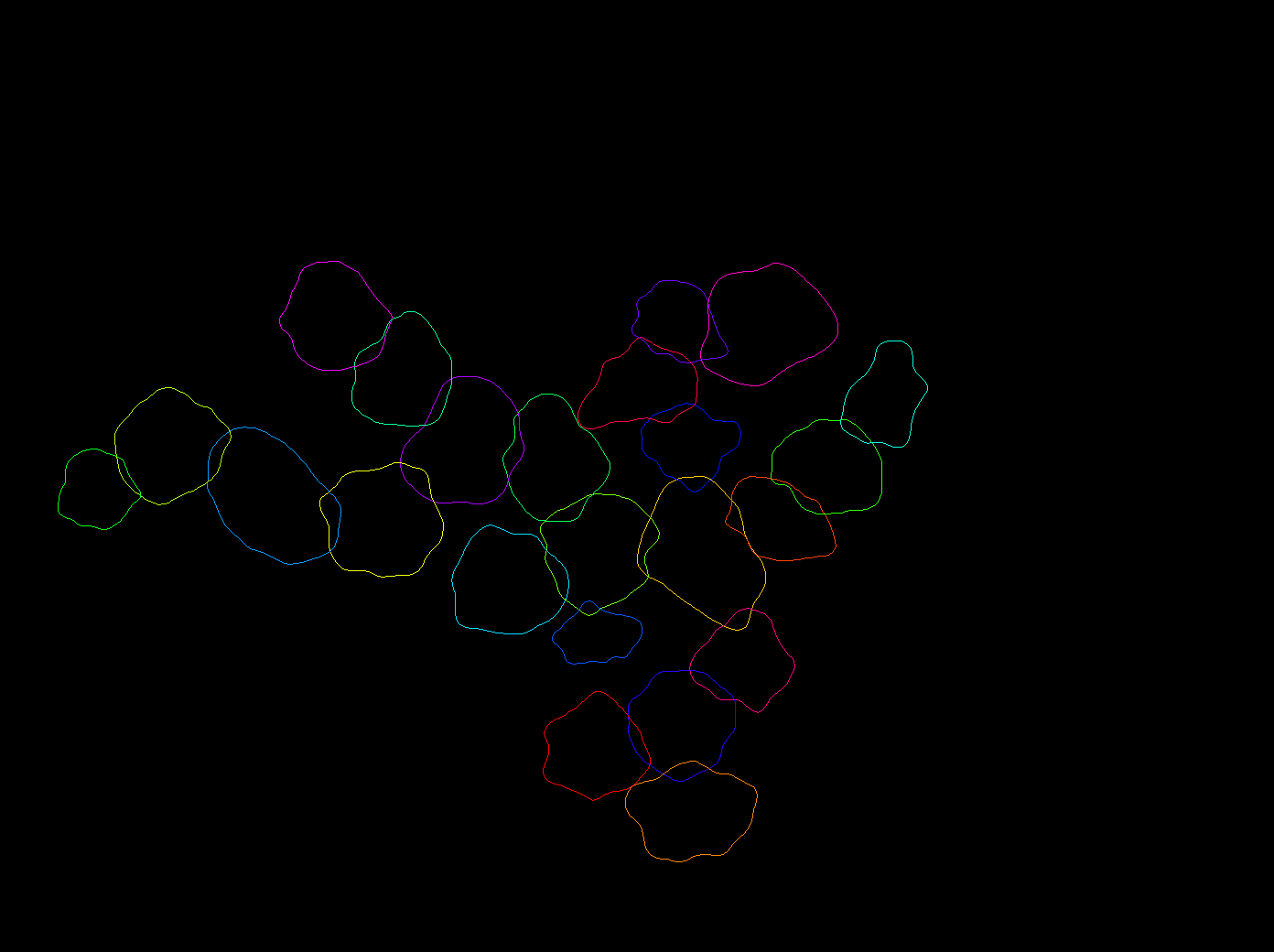

Supplement: Additional file 6 — The zip archive contains simulated images showing B cell nuclei and cytoskeleton with corresponding ground truth. (ZIP 119808 kb) [file 12859_2017_1591_MOESM6_ESM.zip › simulated B cells/cytoskeleton/overlapping/cell004 gt.png]

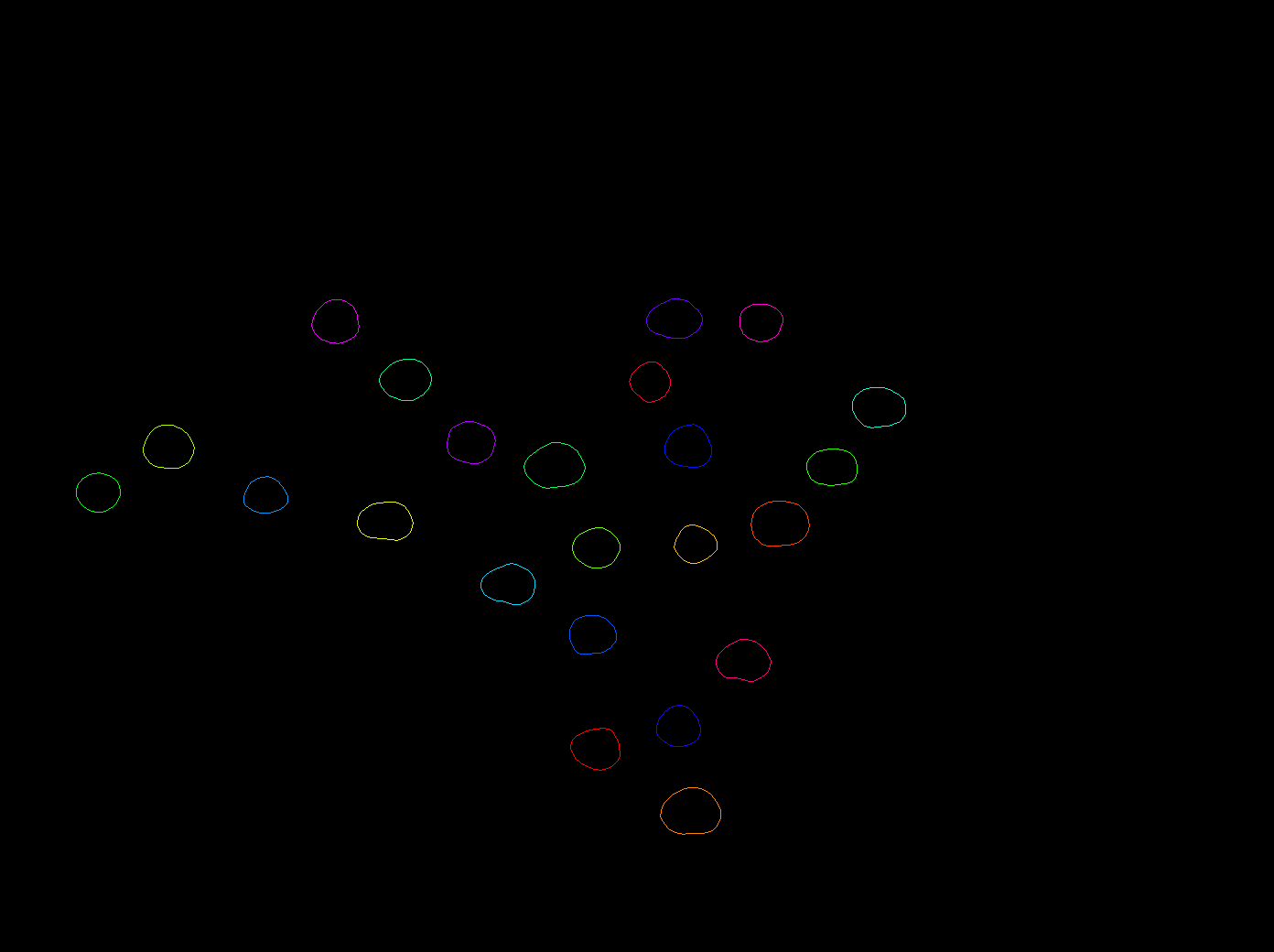

Supplement: Additional file 6 — The zip archive contains simulated images showing B cell nuclei and cytoskeleton with corresponding ground truth. (ZIP 119808 kb) [file 12859_2017_1591_MOESM6_ESM.zip › simulated B cells/cytoskeleton/overlapping/cell004 seeds.png]

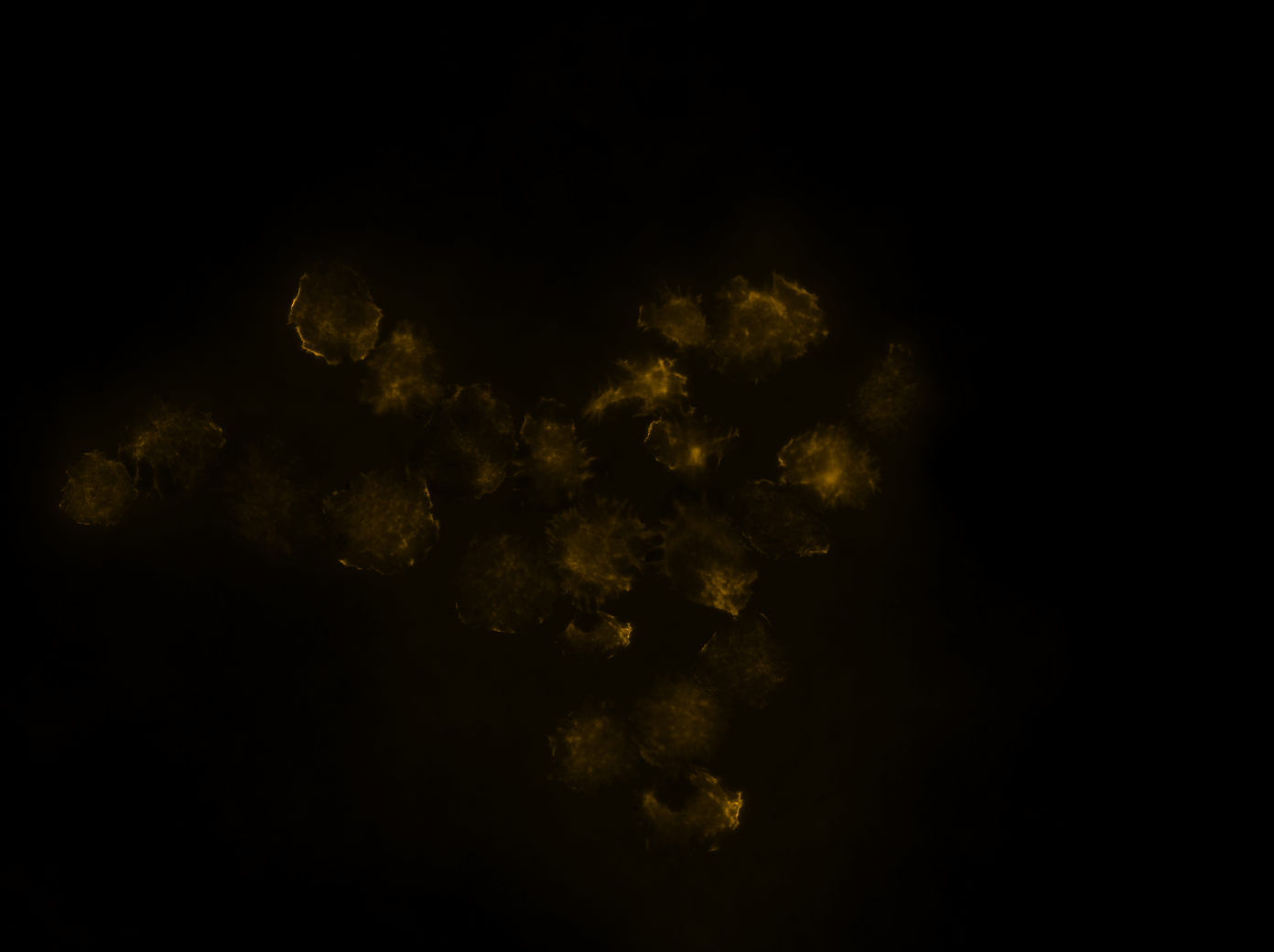

Supplement: Additional file 6 — The zip archive contains simulated images showing B cell nuclei and cytoskeleton with corresponding ground truth. (ZIP 119808 kb) [file 12859_2017_1591_MOESM6_ESM.zip › simulated B cells/cytoskeleton/overlapping/cell004.png]

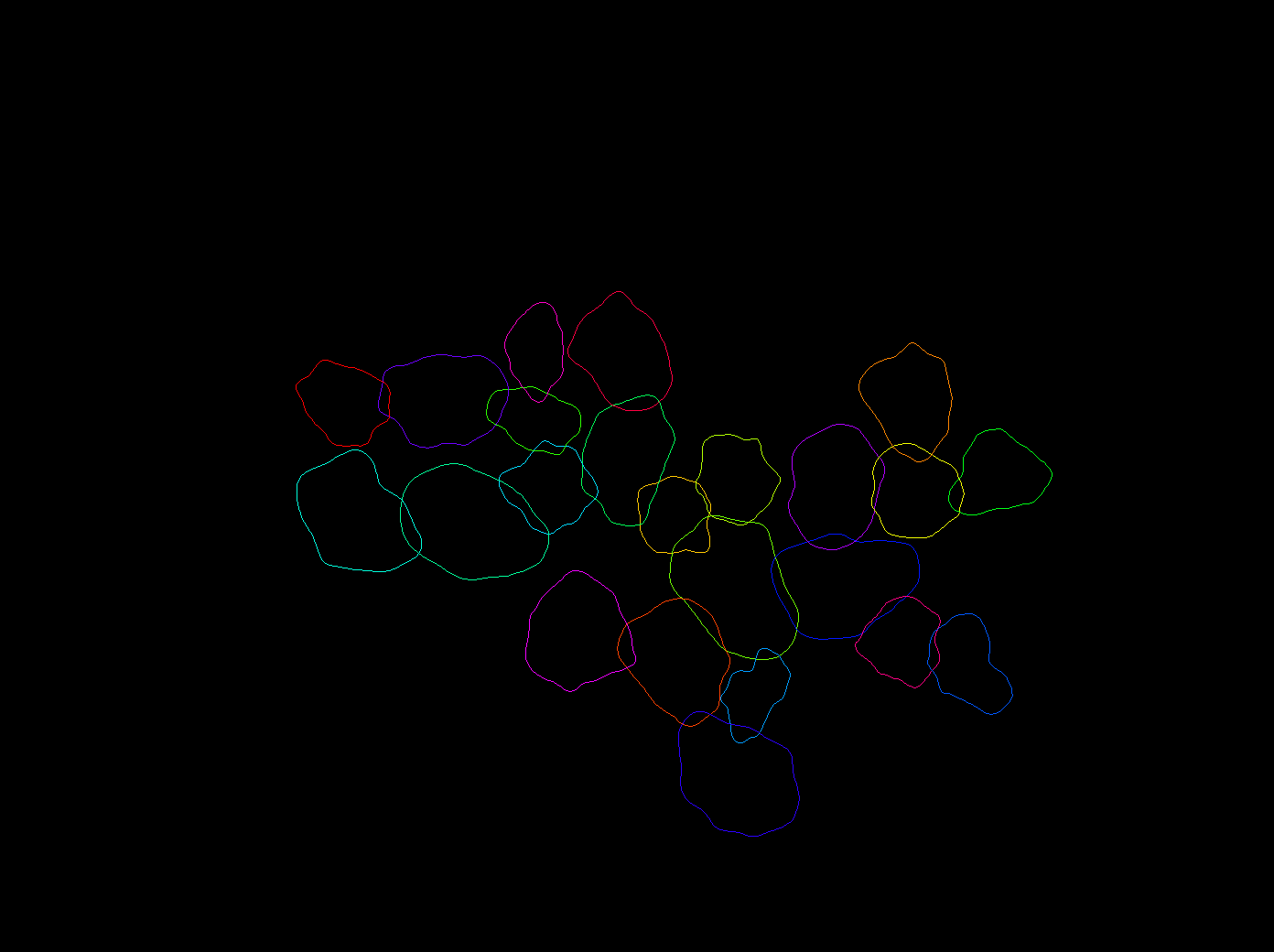

Supplement: Additional file 6 — The zip archive contains simulated images showing B cell nuclei and cytoskeleton with corresponding ground truth. (ZIP 119808 kb) [file 12859_2017_1591_MOESM6_ESM.zip › simulated B cells/cytoskeleton/overlapping/cell005 gt.png]

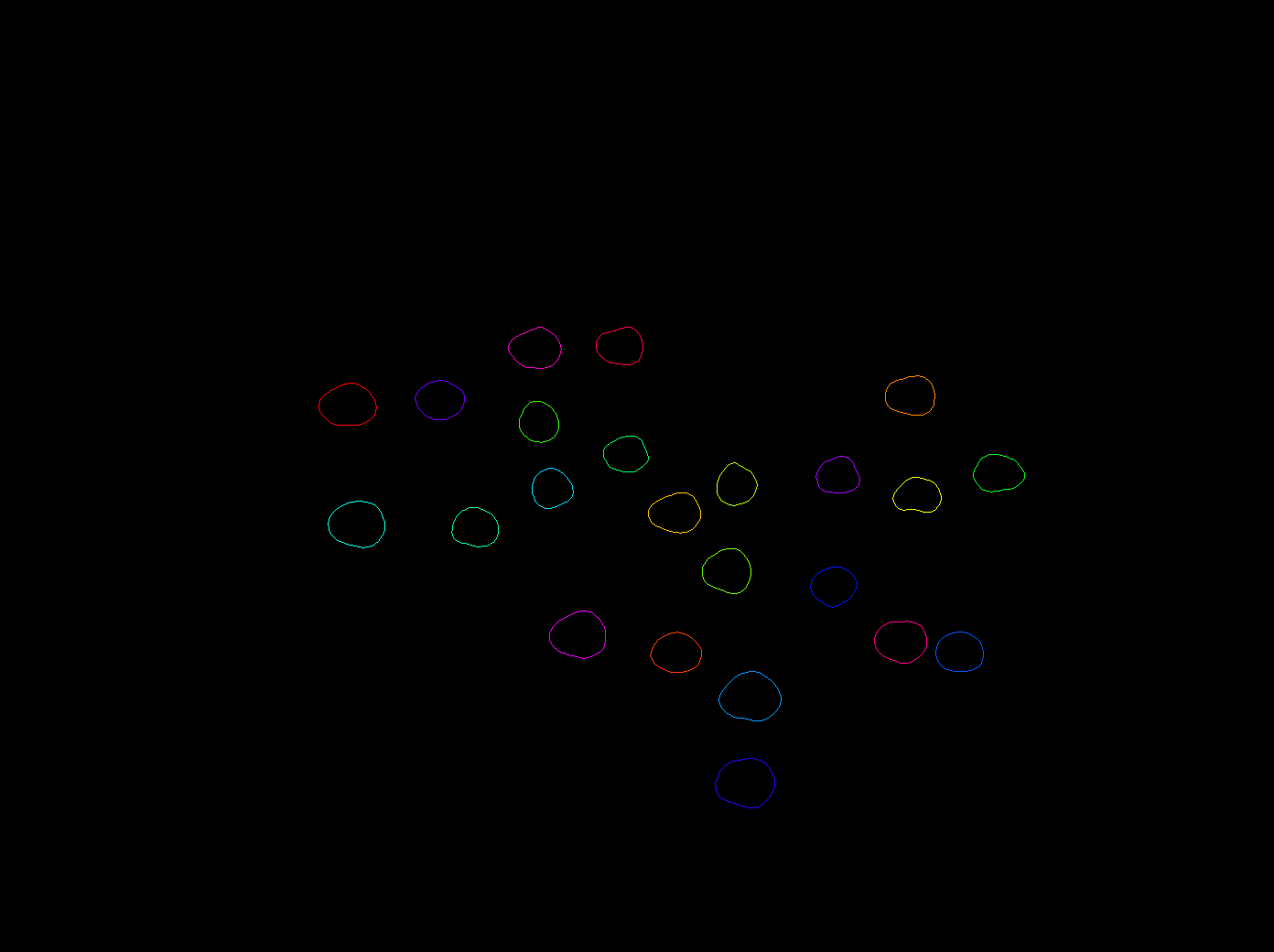

Supplement: Additional file 6 — The zip archive contains simulated images showing B cell nuclei and cytoskeleton with corresponding ground truth. (ZIP 119808 kb) [file 12859_2017_1591_MOESM6_ESM.zip › simulated B cells/cytoskeleton/overlapping/cell005 seeds.png]

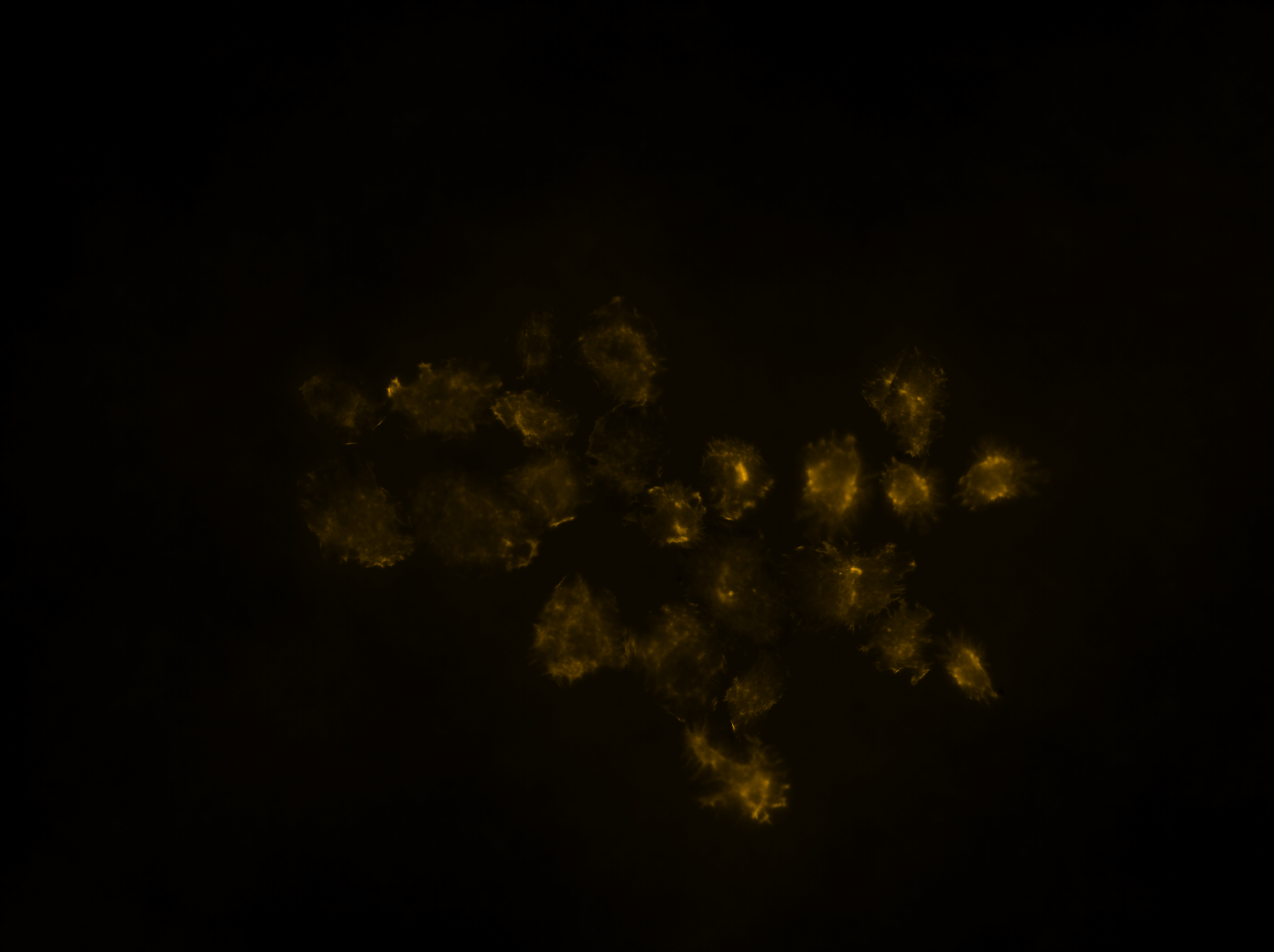

Supplement: Additional file 6 — The zip archive contains simulated images showing B cell nuclei and cytoskeleton with corresponding ground truth. (ZIP 119808 kb) [file 12859_2017_1591_MOESM6_ESM.zip › simulated B cells/cytoskeleton/overlapping/cell005.png]

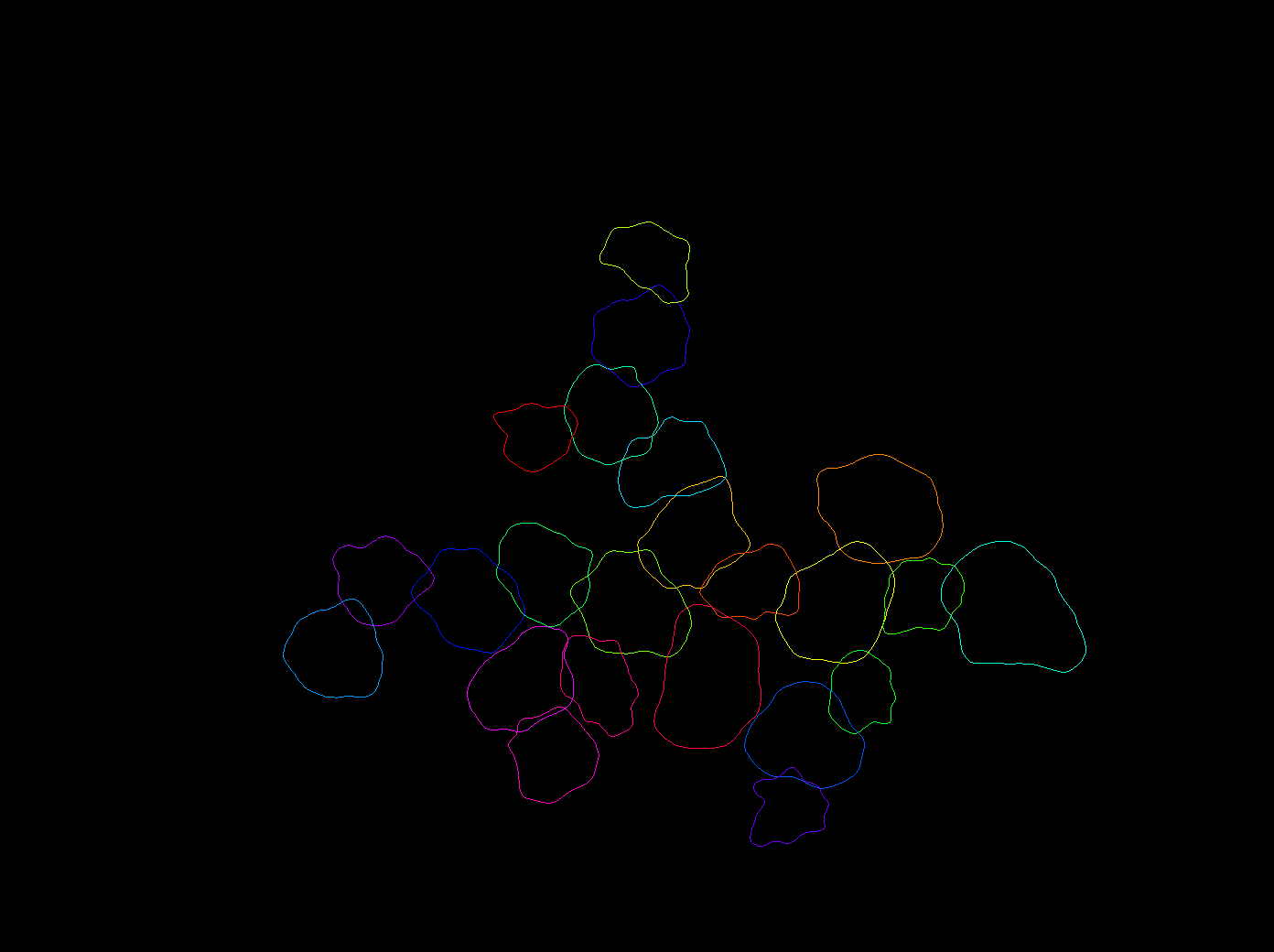

Supplement: Additional file 6 — The zip archive contains simulated images showing B cell nuclei and cytoskeleton with corresponding ground truth. (ZIP 119808 kb) [file 12859_2017_1591_MOESM6_ESM.zip › simulated B cells/cytoskeleton/overlapping/cell006 gt.png]

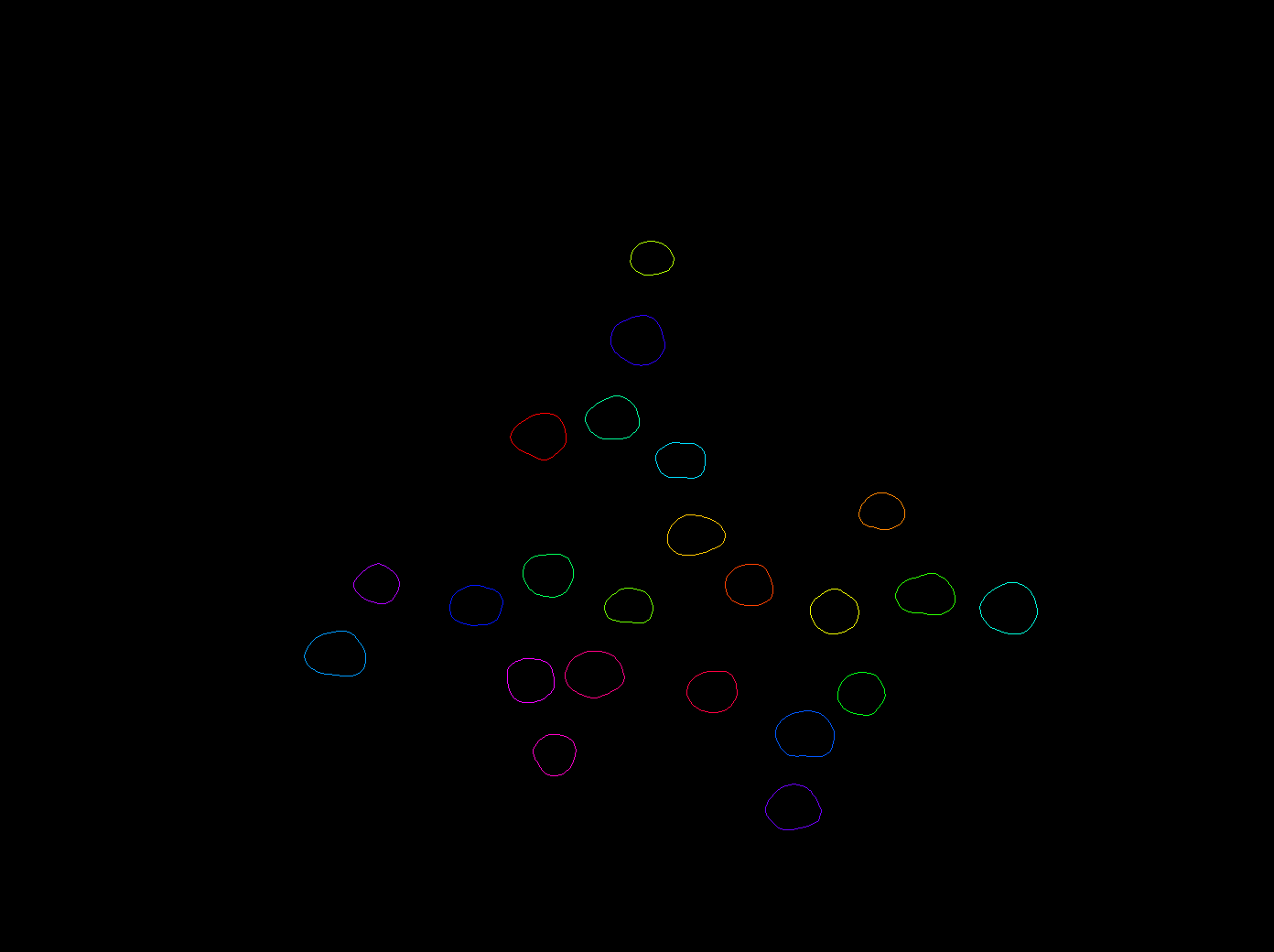

Supplement: Additional file 6 — The zip archive contains simulated images showing B cell nuclei and cytoskeleton with corresponding ground truth. (ZIP 119808 kb) [file 12859_2017_1591_MOESM6_ESM.zip › simulated B cells/cytoskeleton/overlapping/cell006 seeds.png]

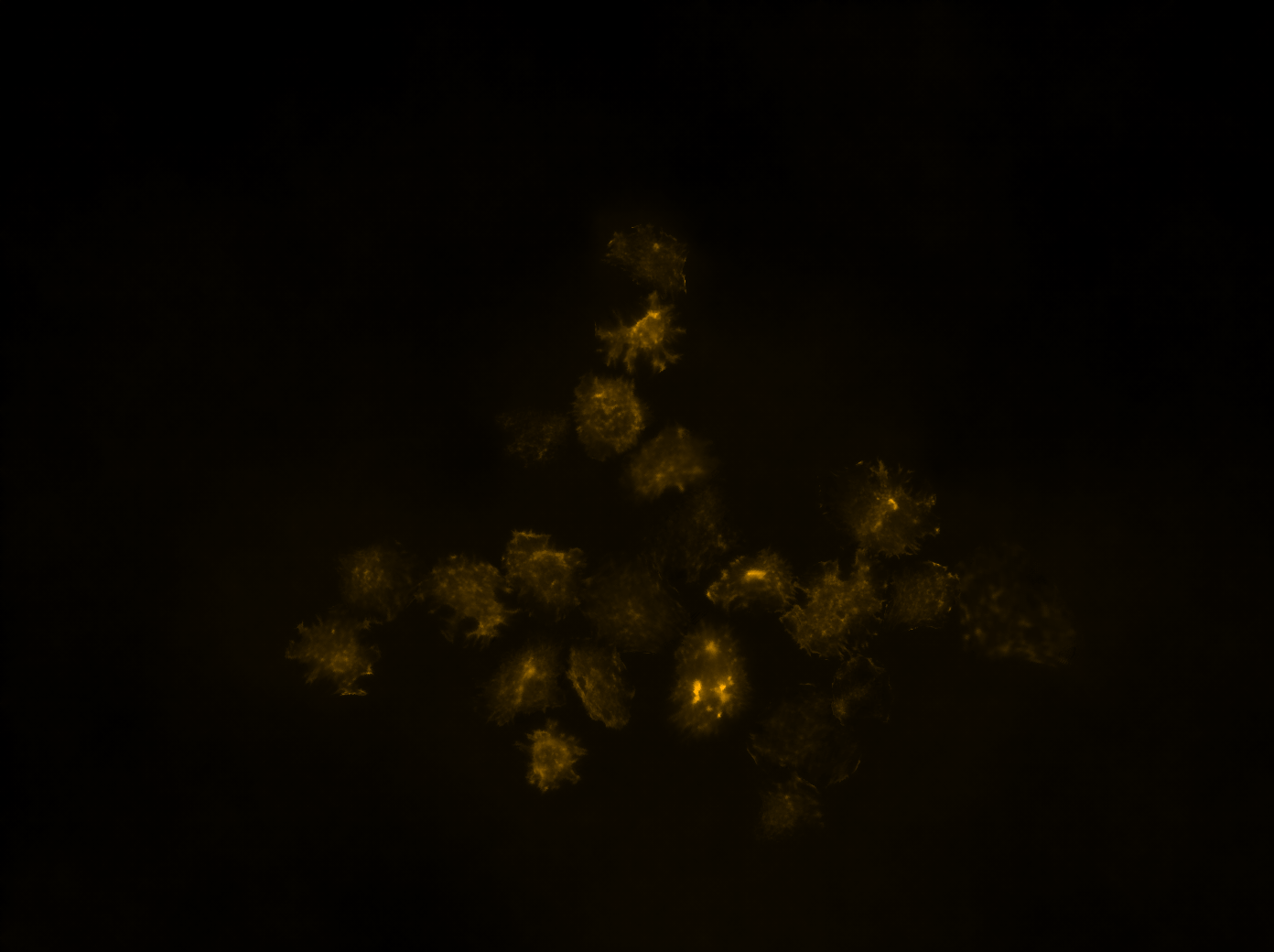

Supplement: Additional file 6 — The zip archive contains simulated images showing B cell nuclei and cytoskeleton with corresponding ground truth. (ZIP 119808 kb) [file 12859_2017_1591_MOESM6_ESM.zip › simulated B cells/cytoskeleton/overlapping/cell006.png]

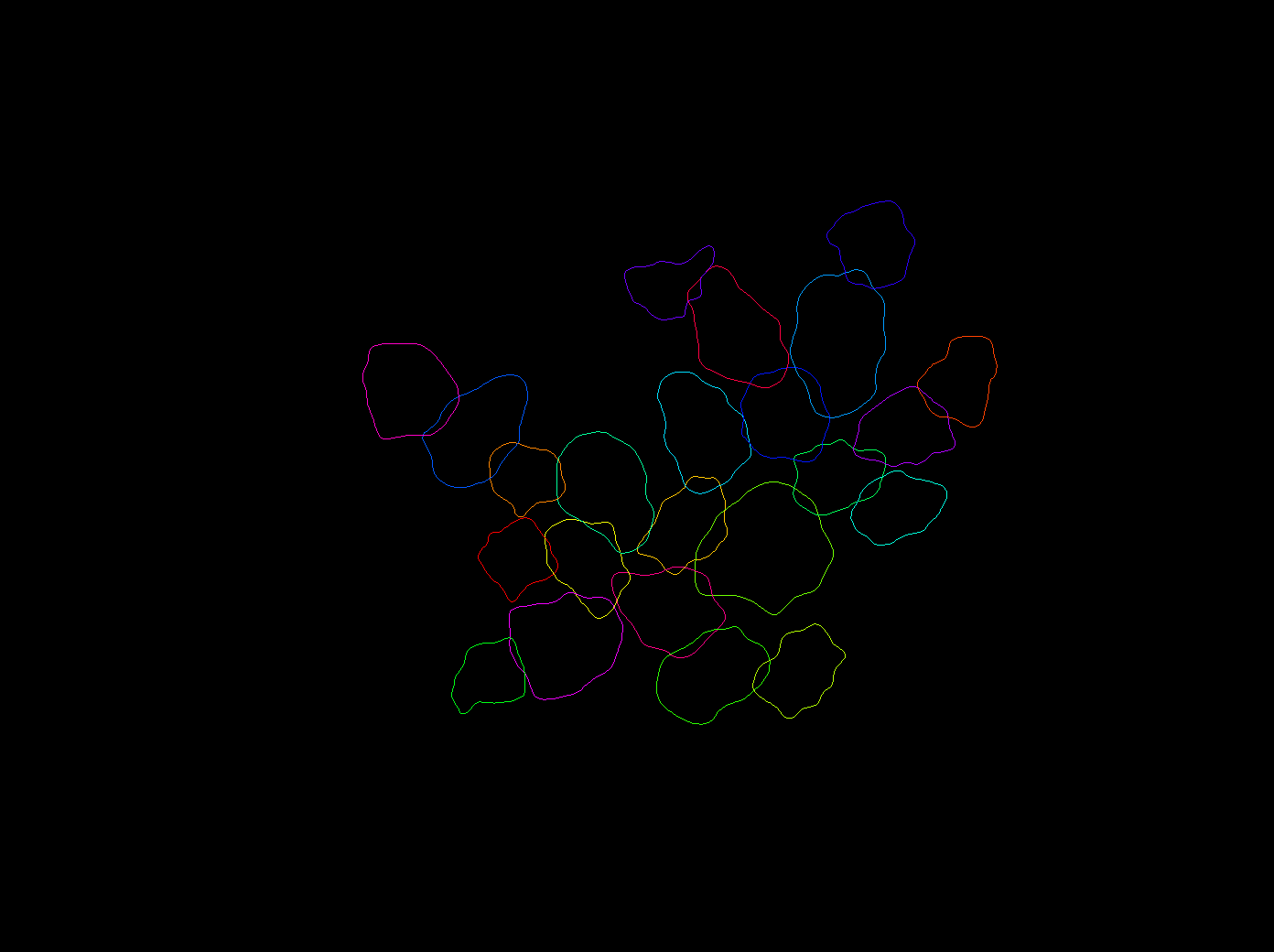

Supplement: Additional file 6 — The zip archive contains simulated images showing B cell nuclei and cytoskeleton with corresponding ground truth. (ZIP 119808 kb) [file 12859_2017_1591_MOESM6_ESM.zip › simulated B cells/cytoskeleton/overlapping/cell007 gt.png]

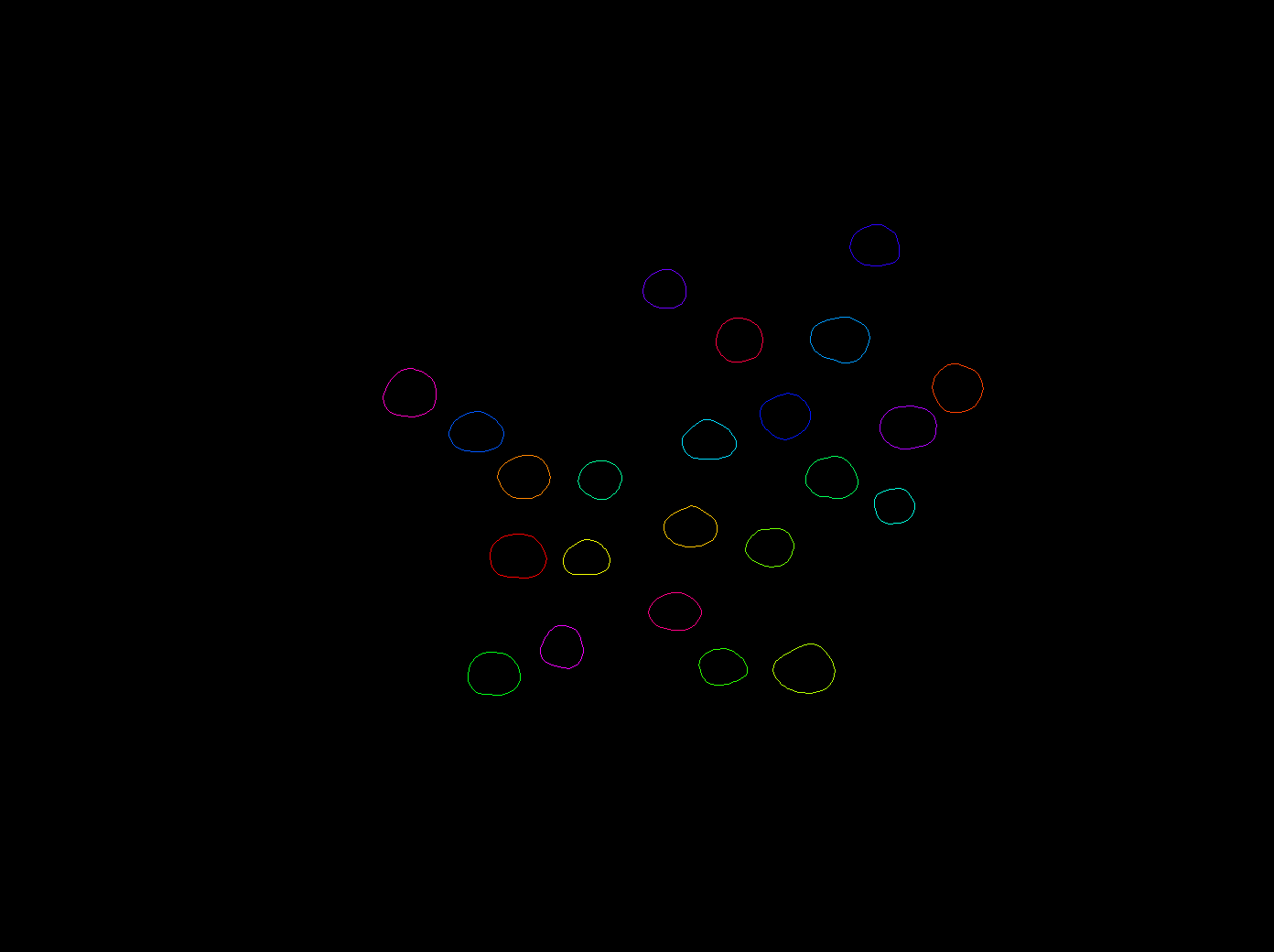

Supplement: Additional file 6 — The zip archive contains simulated images showing B cell nuclei and cytoskeleton with corresponding ground truth. (ZIP 119808 kb) [file 12859_2017_1591_MOESM6_ESM.zip › simulated B cells/cytoskeleton/overlapping/cell007 seeds.png]

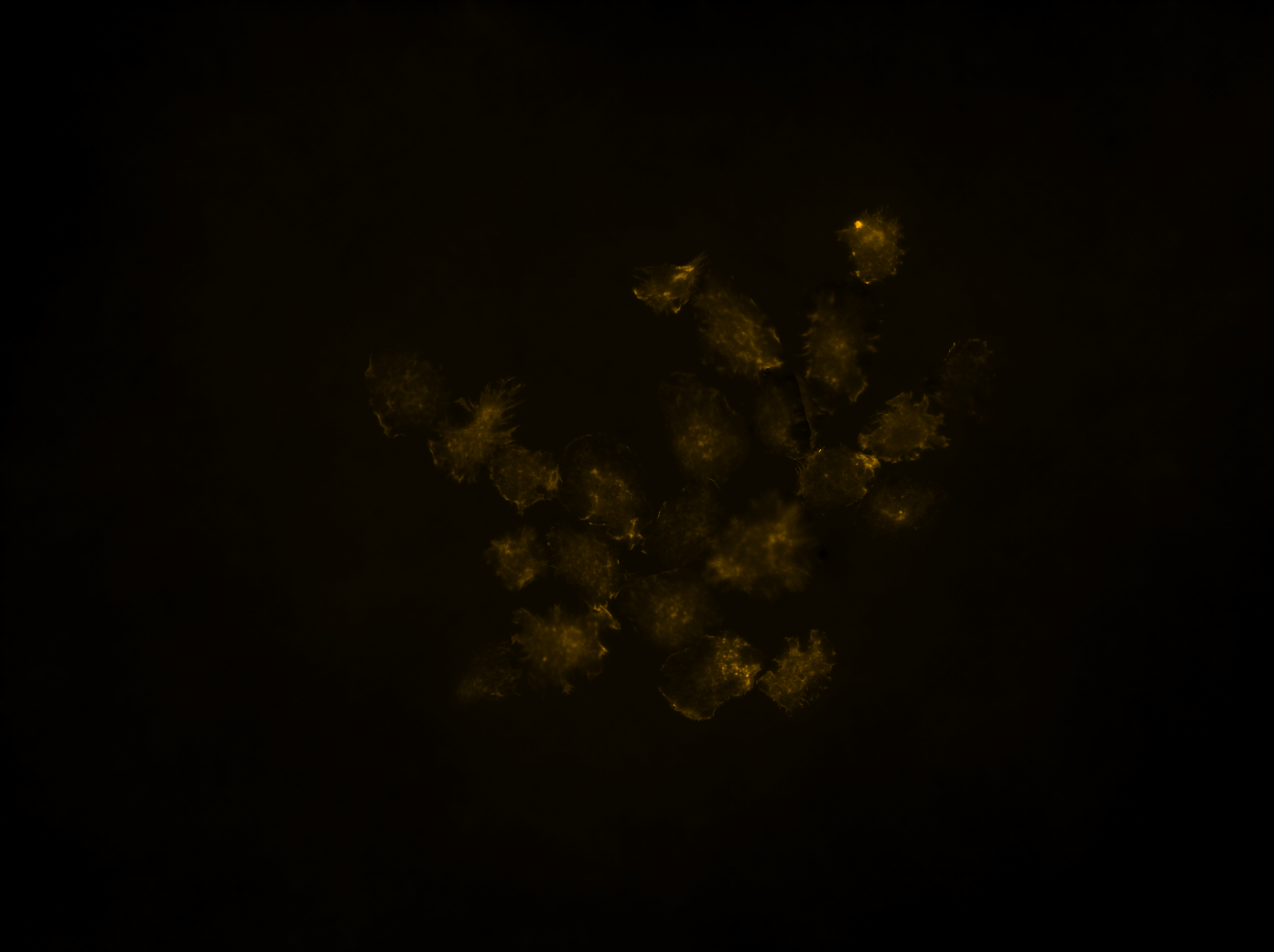

Supplement: Additional file 6 — The zip archive contains simulated images showing B cell nuclei and cytoskeleton with corresponding ground truth. (ZIP 119808 kb) [file 12859_2017_1591_MOESM6_ESM.zip › simulated B cells/cytoskeleton/overlapping/cell007.png]

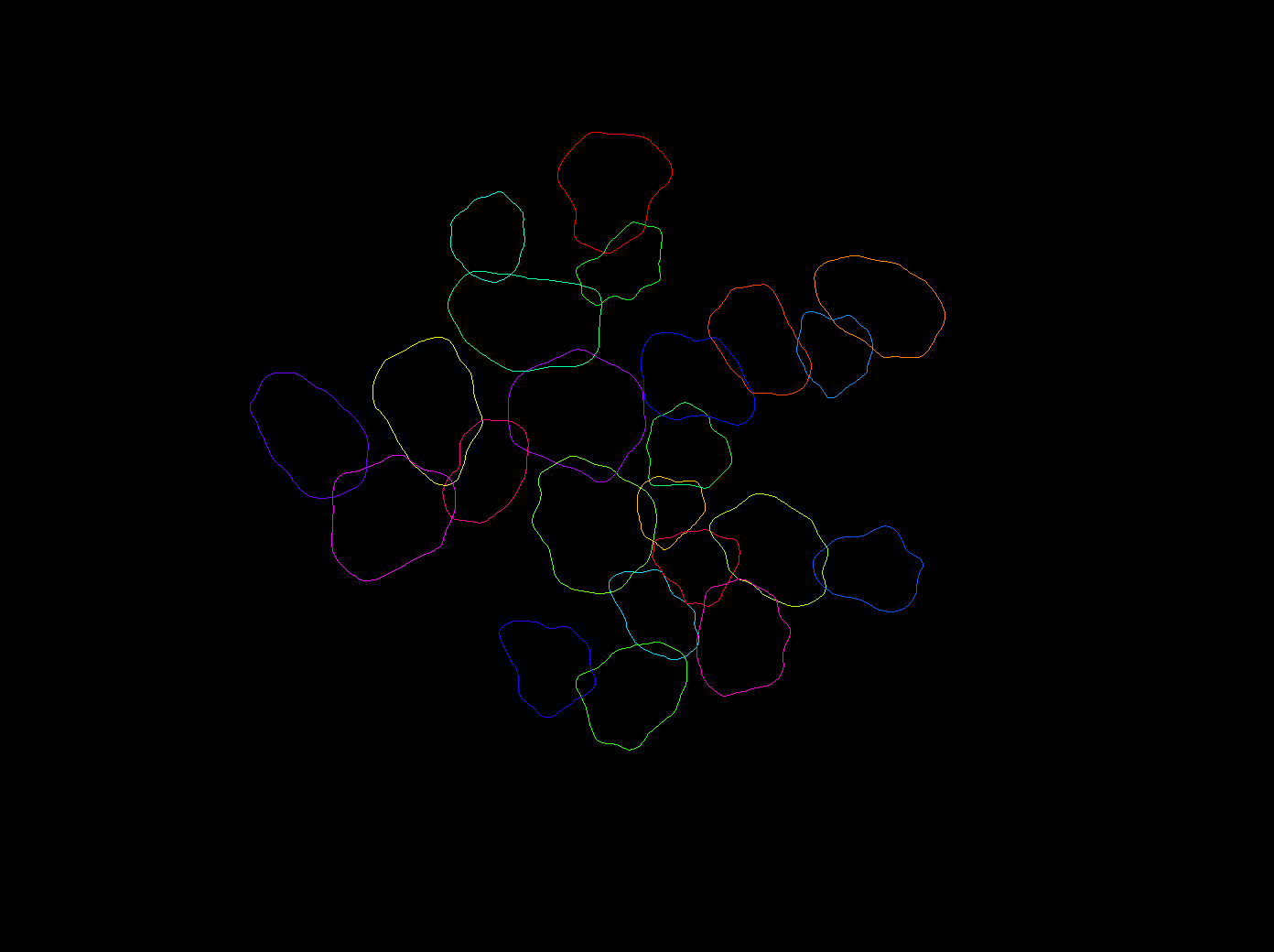

Supplement: Additional file 6 — The zip archive contains simulated images showing B cell nuclei and cytoskeleton with corresponding ground truth. (ZIP 119808 kb) [file 12859_2017_1591_MOESM6_ESM.zip › simulated B cells/cytoskeleton/overlapping/cell008 gt.png]

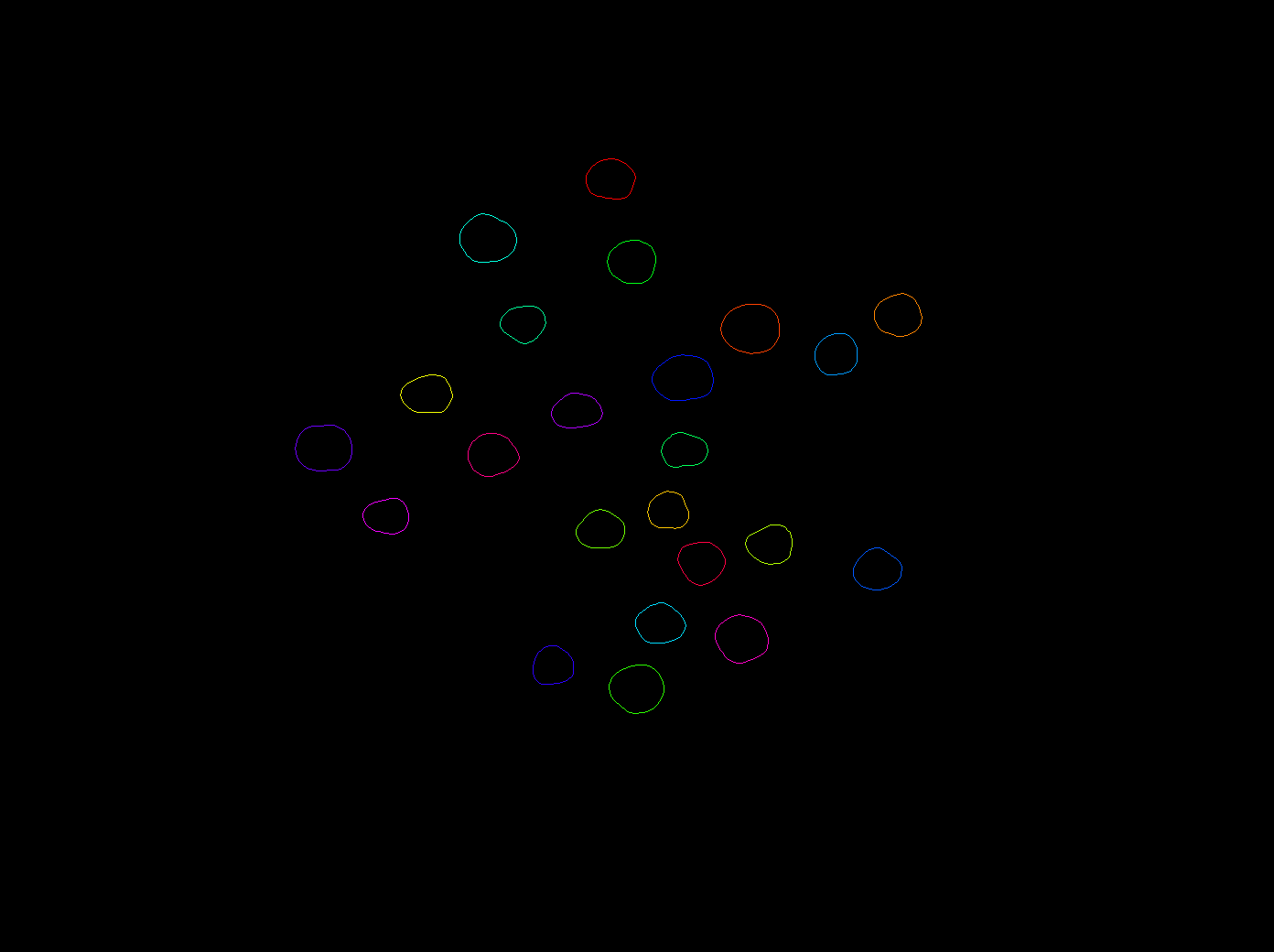

Supplement: Additional file 6 — The zip archive contains simulated images showing B cell nuclei and cytoskeleton with corresponding ground truth. (ZIP 119808 kb) [file 12859_2017_1591_MOESM6_ESM.zip › simulated B cells/cytoskeleton/overlapping/cell008 seeds.png]

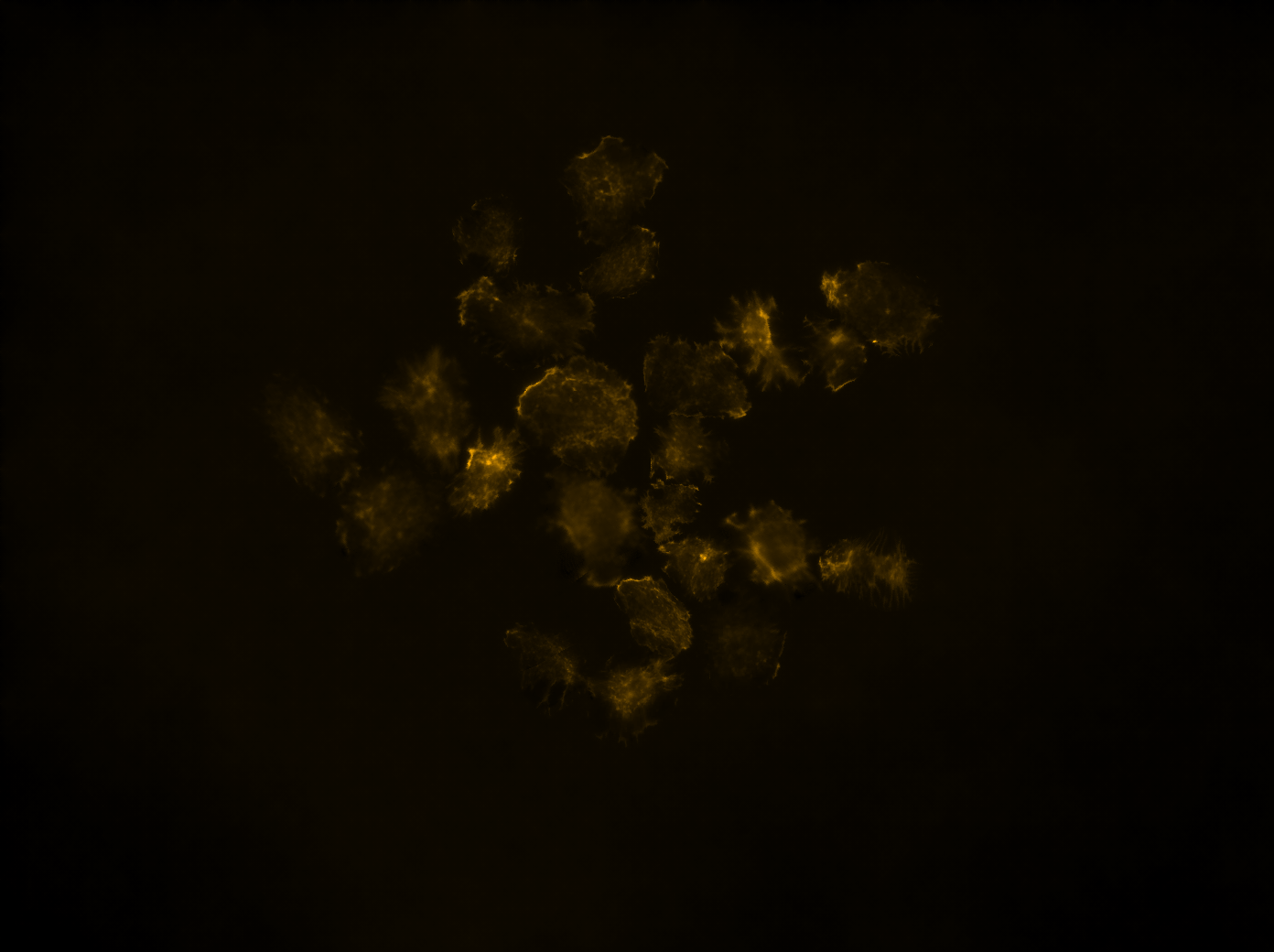

Supplement: Additional file 6 — The zip archive contains simulated images showing B cell nuclei and cytoskeleton with corresponding ground truth. (ZIP 119808 kb) [file 12859_2017_1591_MOESM6_ESM.zip › simulated B cells/cytoskeleton/overlapping/cell008.png]

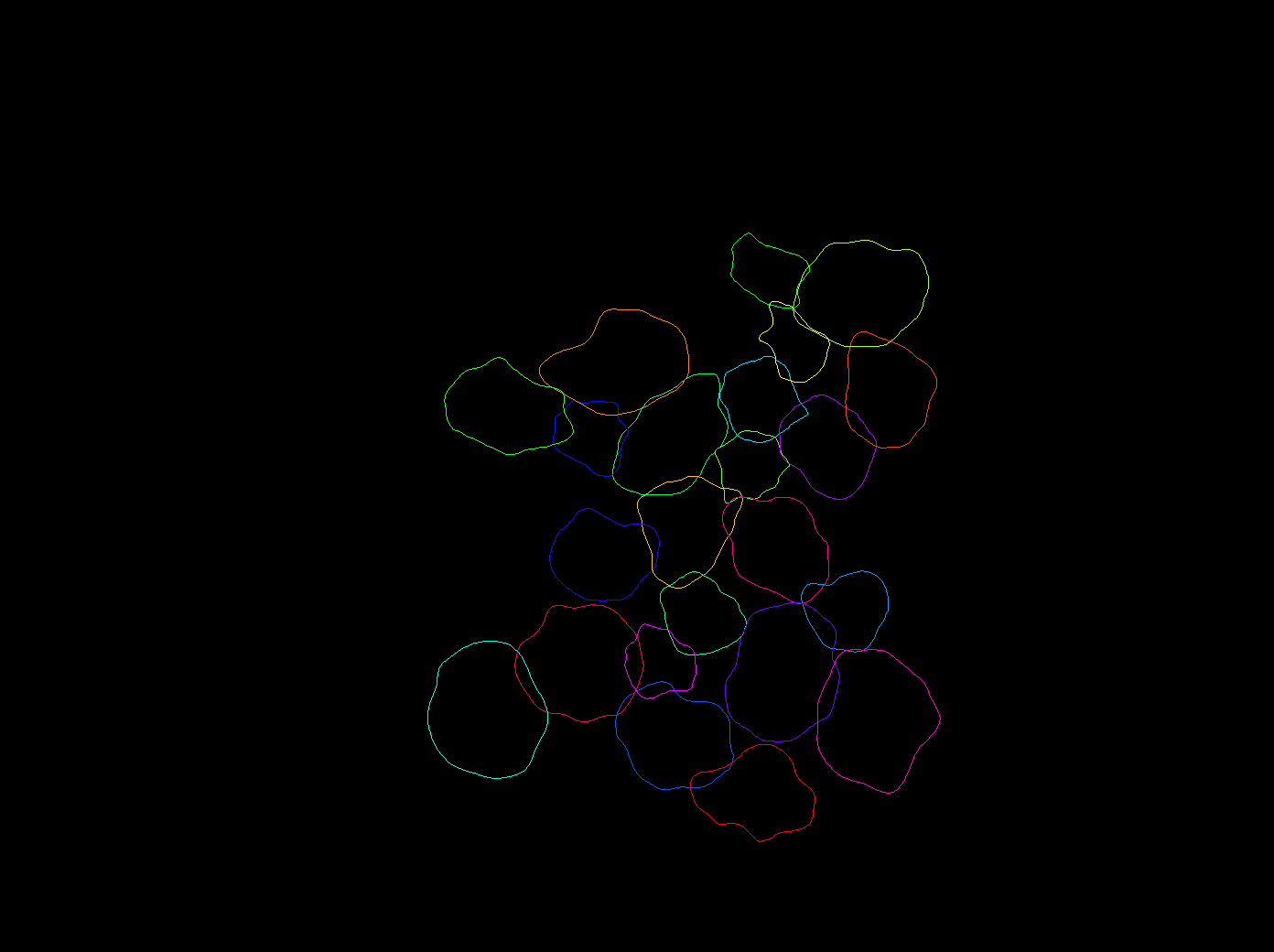

Supplement: Additional file 6 — The zip archive contains simulated images showing B cell nuclei and cytoskeleton with corresponding ground truth. (ZIP 119808 kb) [file 12859_2017_1591_MOESM6_ESM.zip › simulated B cells/cytoskeleton/overlapping/cell009 gt.png]

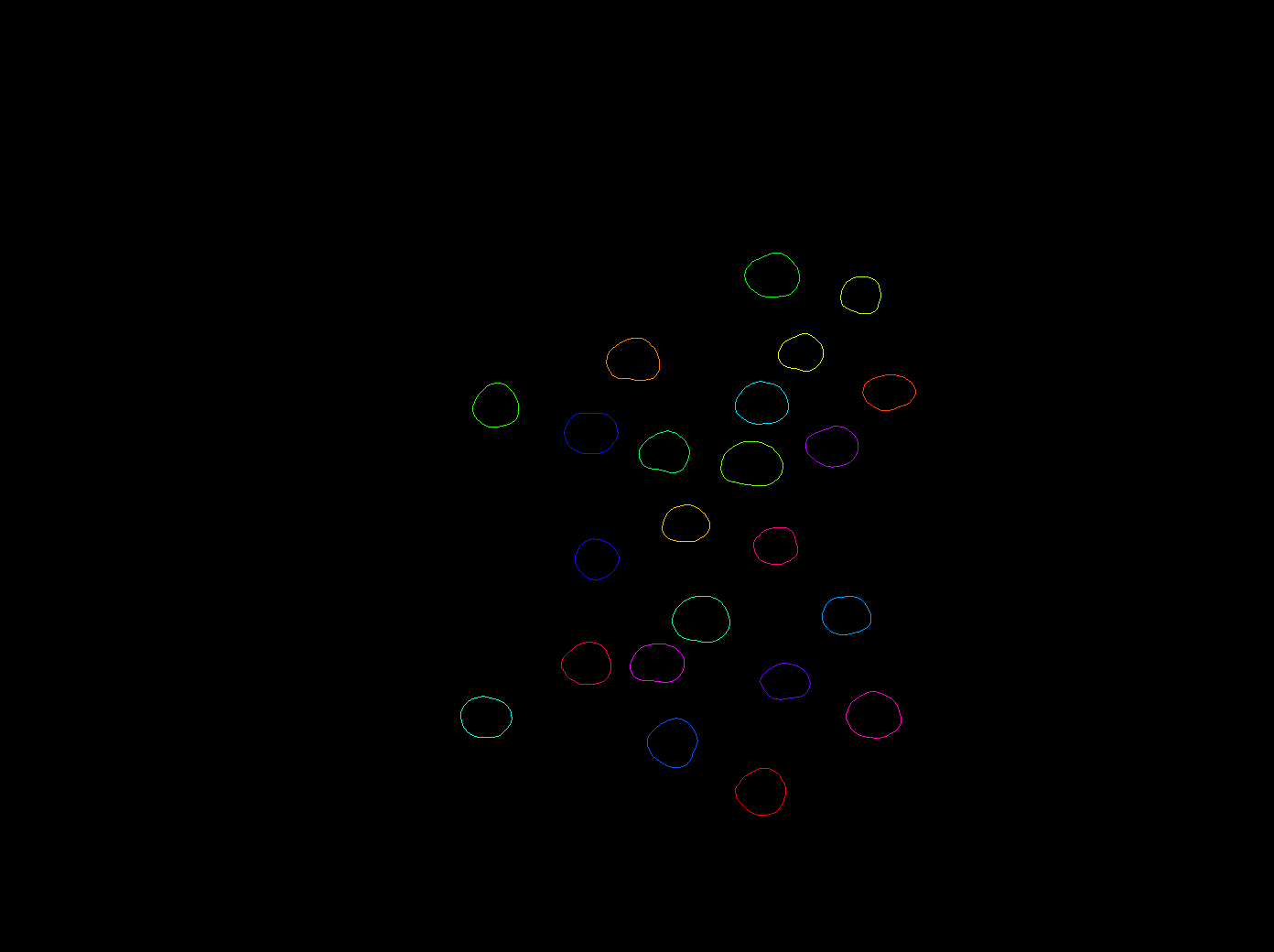

Supplement: Additional file 6 — The zip archive contains simulated images showing B cell nuclei and cytoskeleton with corresponding ground truth. (ZIP 119808 kb) [file 12859_2017_1591_MOESM6_ESM.zip › simulated B cells/cytoskeleton/overlapping/cell009 seeds.png]

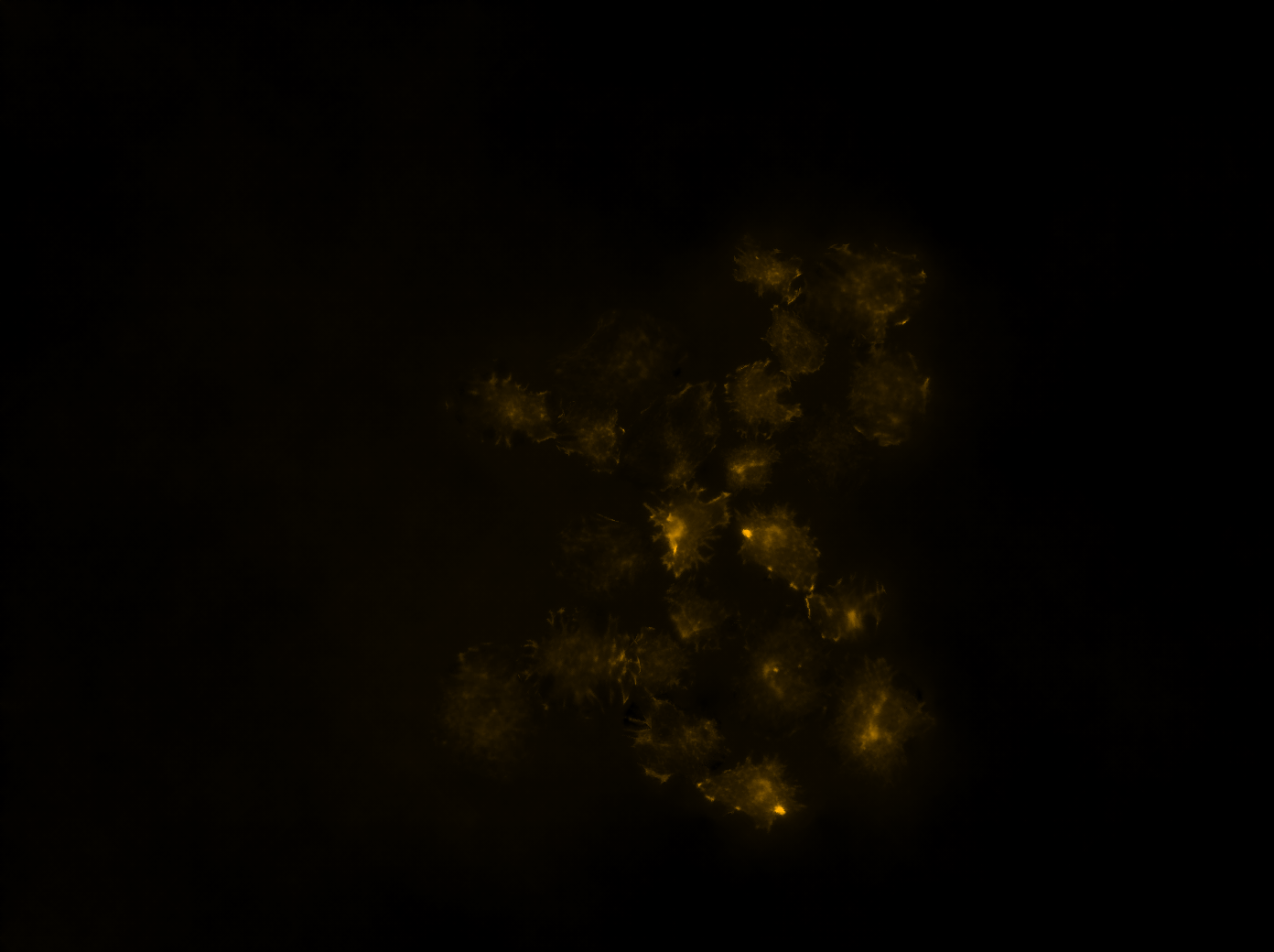

Supplement: Additional file 6 — The zip archive contains simulated images showing B cell nuclei and cytoskeleton with corresponding ground truth. (ZIP 119808 kb) [file 12859_2017_1591_MOESM6_ESM.zip › simulated B cells/cytoskeleton/overlapping/cell009.png]

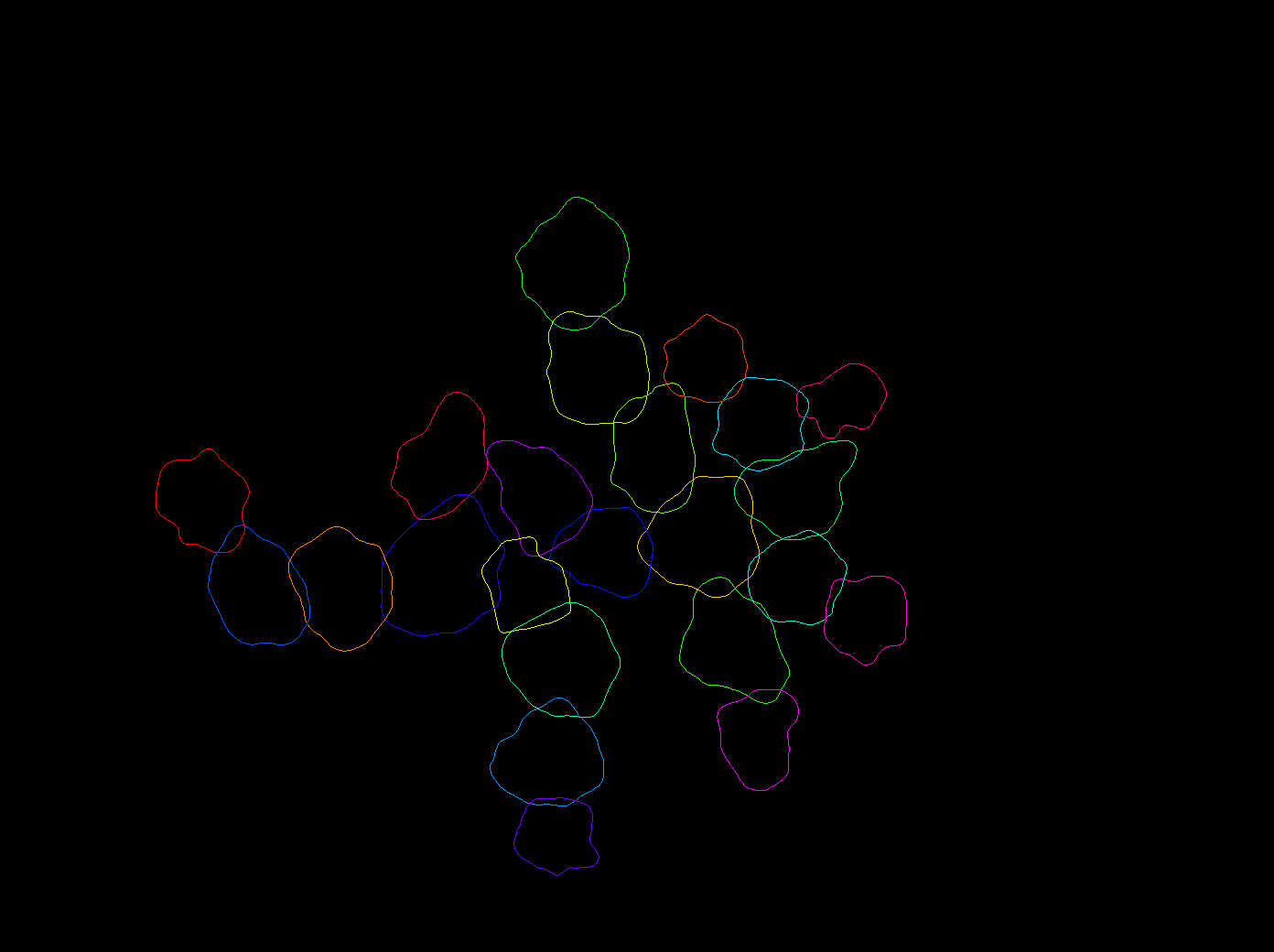

Supplement: Additional file 6 — The zip archive contains simulated images showing B cell nuclei and cytoskeleton with corresponding ground truth. (ZIP 119808 kb) [file 12859_2017_1591_MOESM6_ESM.zip › simulated B cells/cytoskeleton/overlapping/cell010 gt.png]

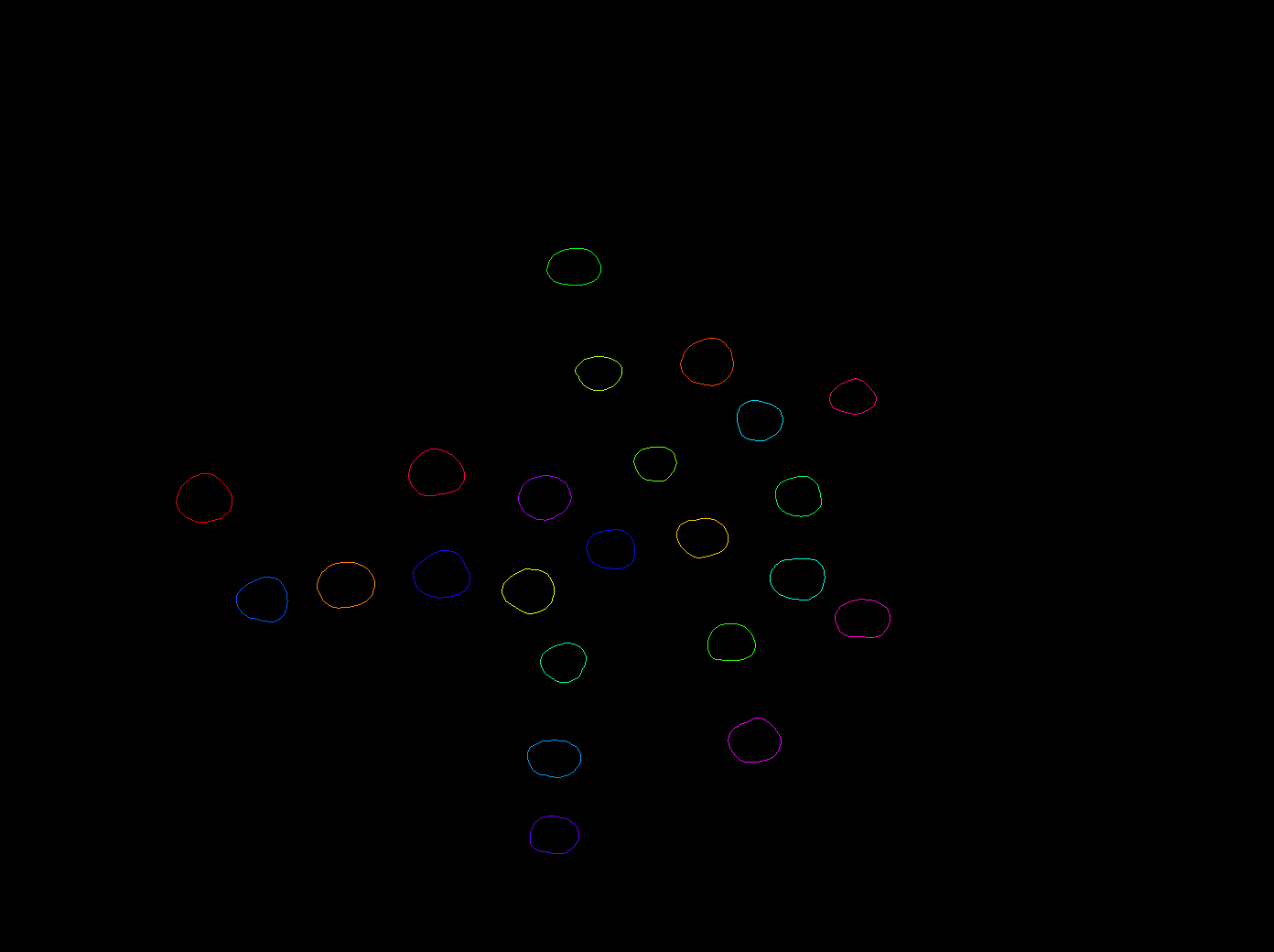

Supplement: Additional file 6 — The zip archive contains simulated images showing B cell nuclei and cytoskeleton with corresponding ground truth. (ZIP 119808 kb) [file 12859_2017_1591_MOESM6_ESM.zip › simulated B cells/cytoskeleton/overlapping/cell010 seeds.png]

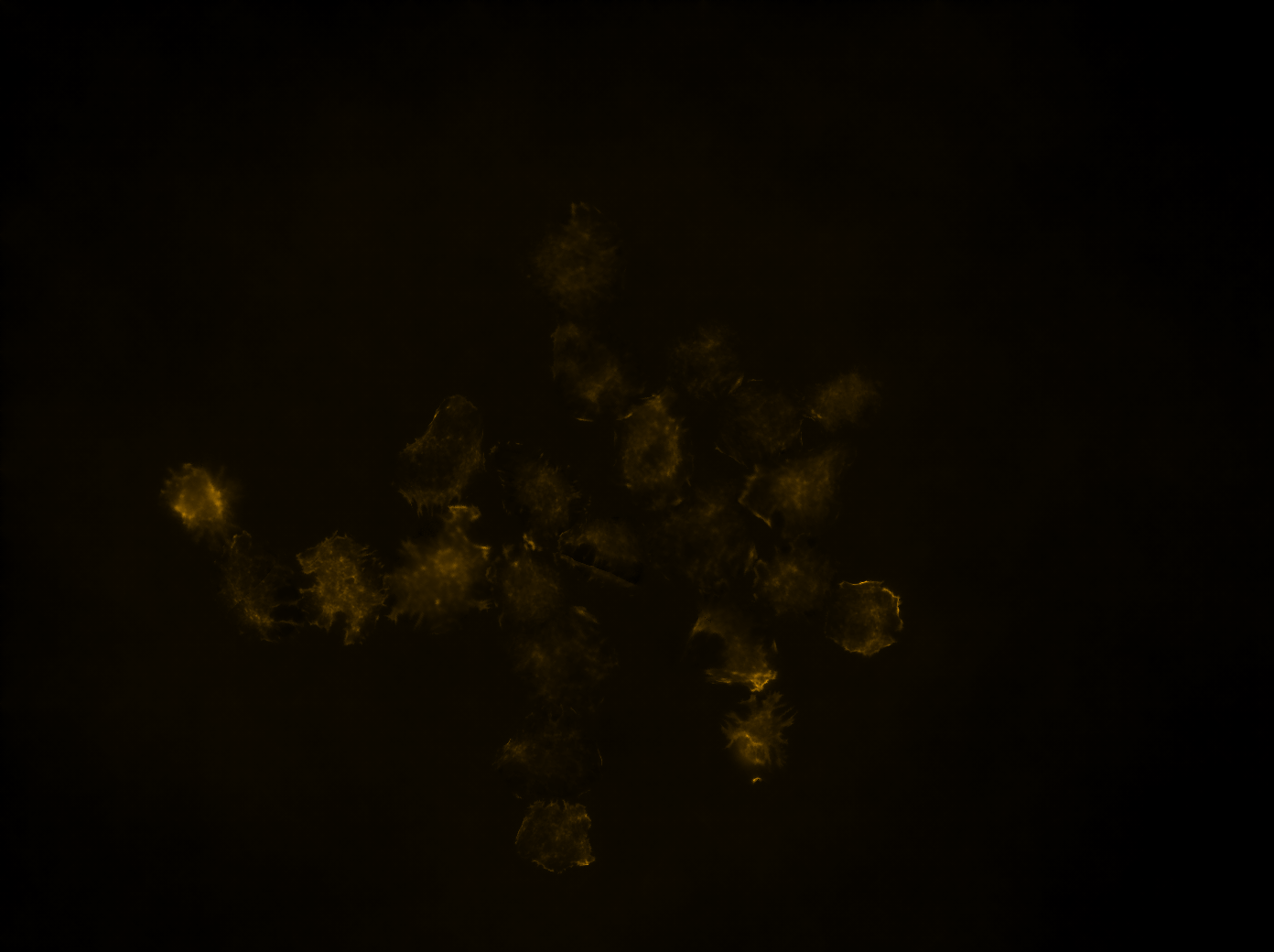

Supplement: Additional file 6 — The zip archive contains simulated images showing B cell nuclei and cytoskeleton with corresponding ground truth. (ZIP 119808 kb) [file 12859_2017_1591_MOESM6_ESM.zip › simulated B cells/cytoskeleton/overlapping/cell010.png]

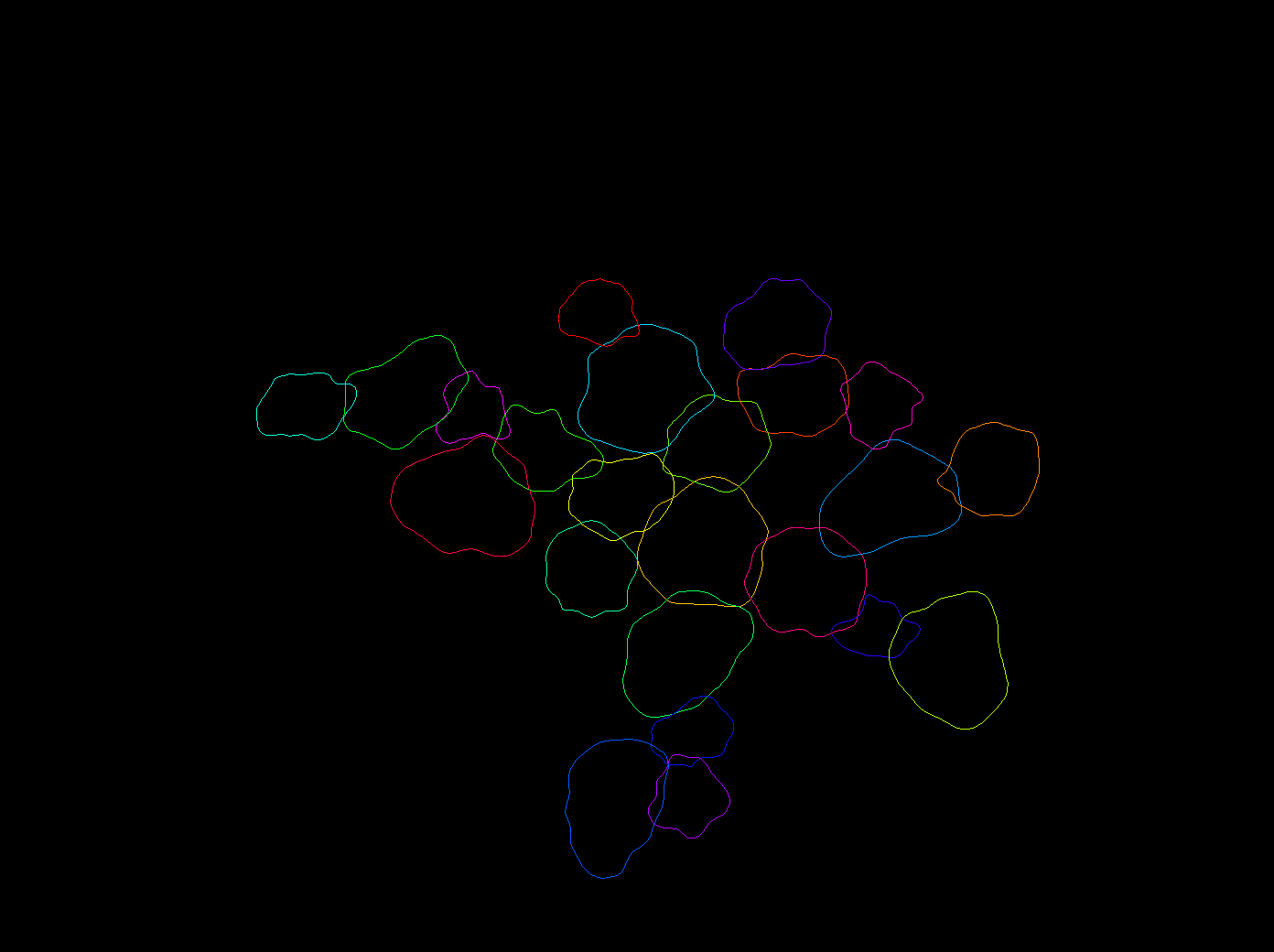

Supplement: Additional file 6 — The zip archive contains simulated images showing B cell nuclei and cytoskeleton with corresponding ground truth. (ZIP 119808 kb) [file 12859_2017_1591_MOESM6_ESM.zip › simulated B cells/cytoskeleton/overlapping/cell011 gt.png]

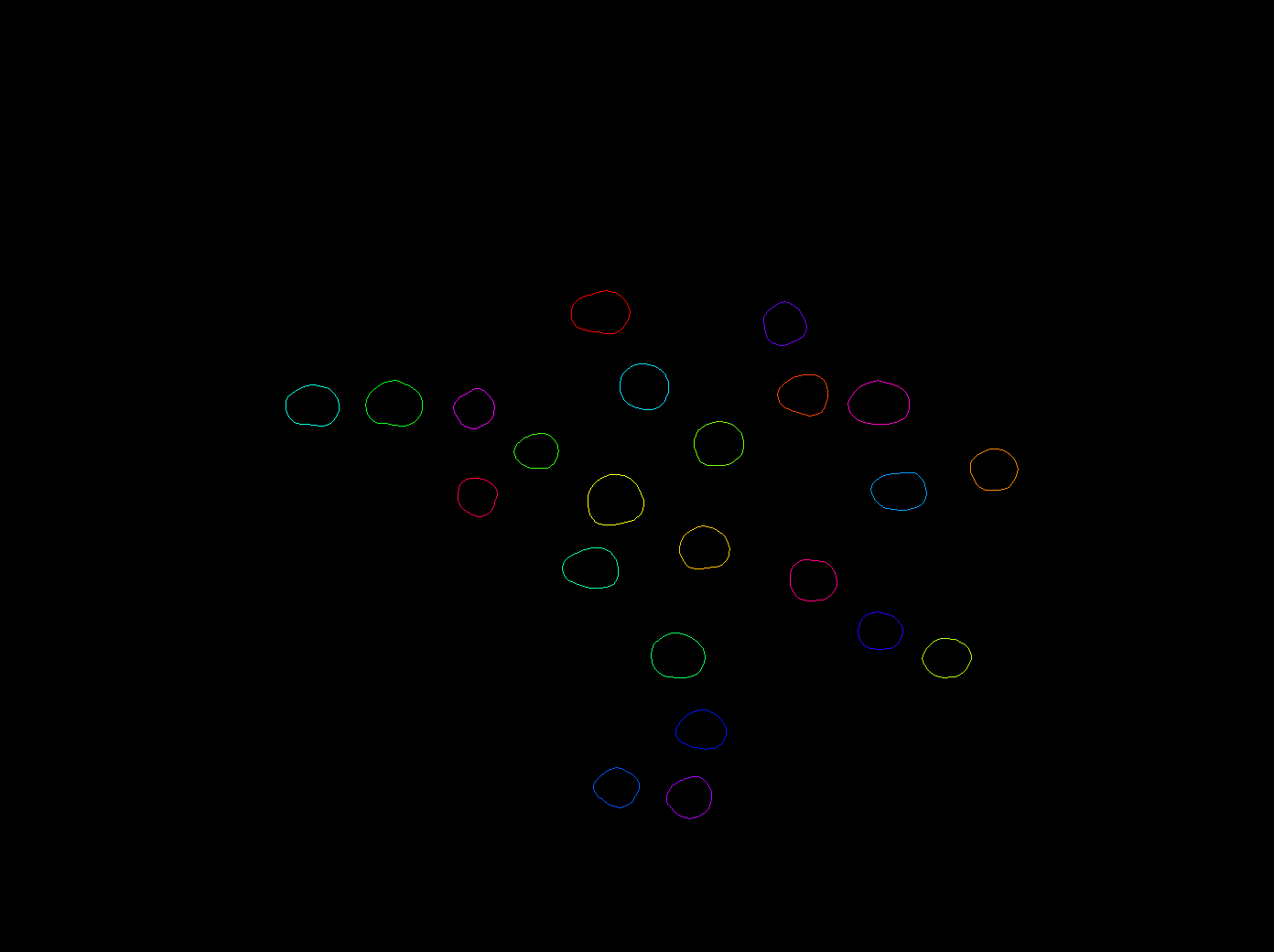

Supplement: Additional file 6 — The zip archive contains simulated images showing B cell nuclei and cytoskeleton with corresponding ground truth. (ZIP 119808 kb) [file 12859_2017_1591_MOESM6_ESM.zip › simulated B cells/cytoskeleton/overlapping/cell011 seeds.png]

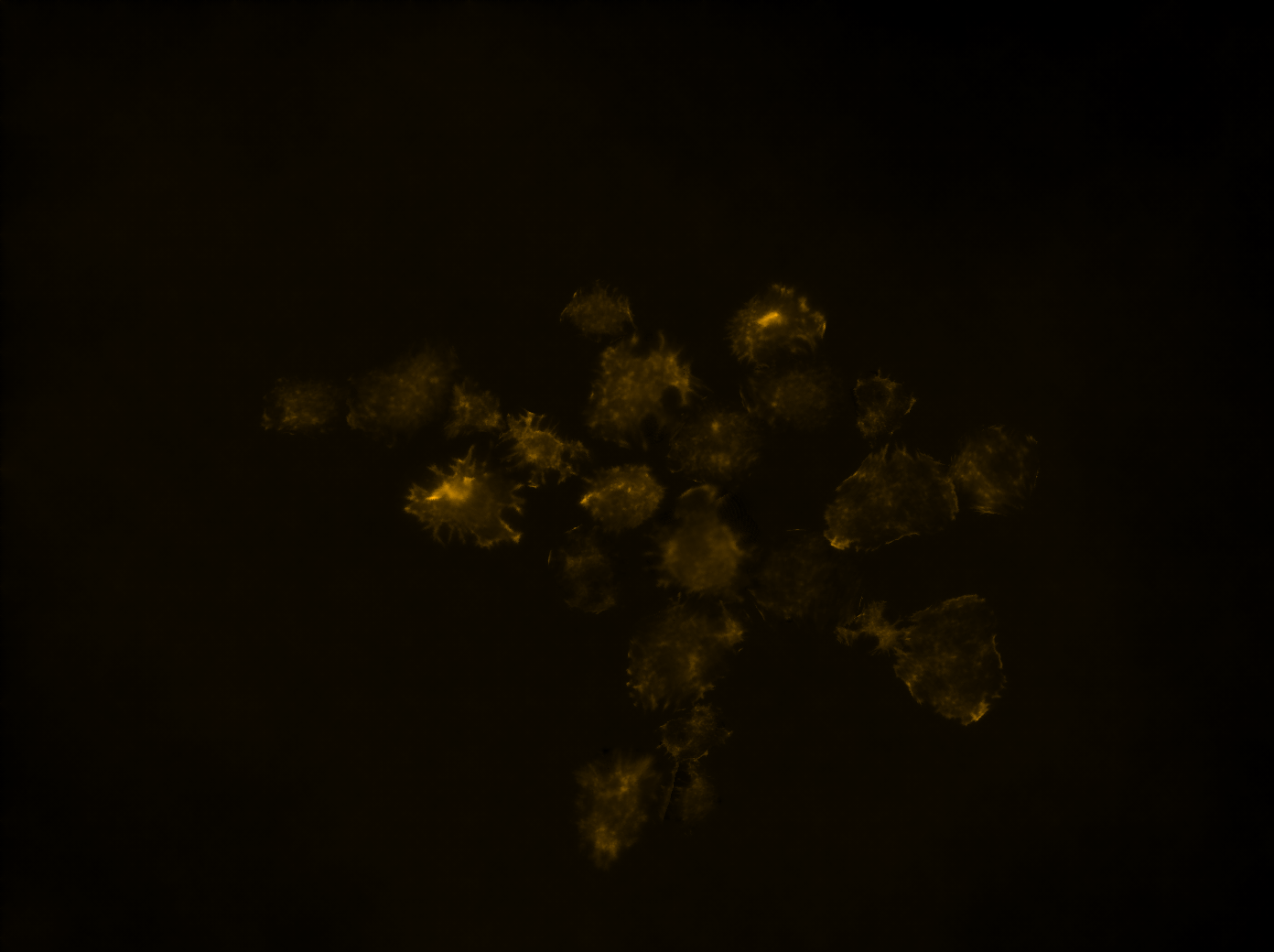

Supplement: Additional file 6 — The zip archive contains simulated images showing B cell nuclei and cytoskeleton with corresponding ground truth. (ZIP 119808 kb) [file 12859_2017_1591_MOESM6_ESM.zip › simulated B cells/cytoskeleton/overlapping/cell011.png]

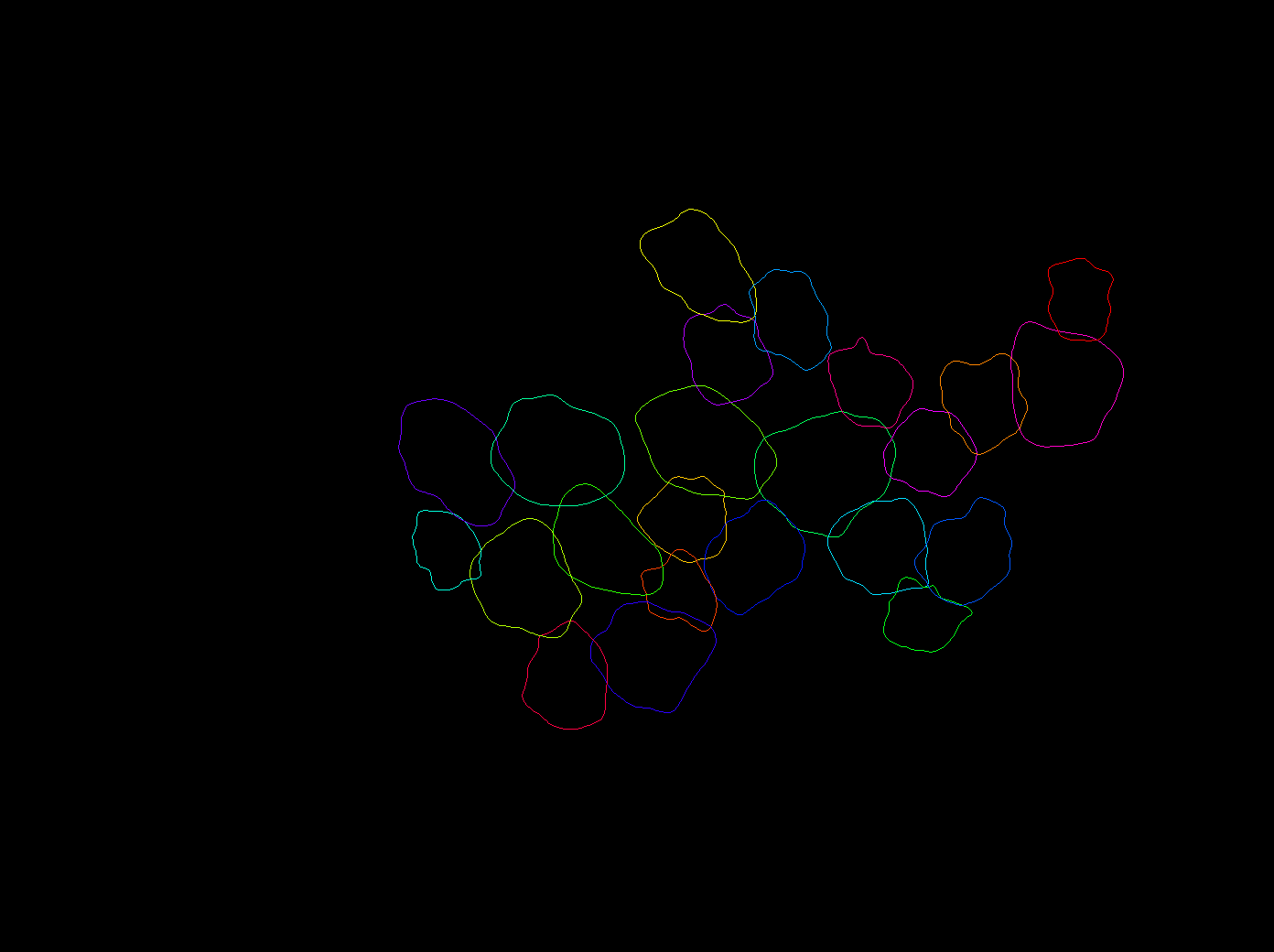

Supplement: Additional file 6 — The zip archive contains simulated images showing B cell nuclei and cytoskeleton with corresponding ground truth. (ZIP 119808 kb) [file 12859_2017_1591_MOESM6_ESM.zip › simulated B cells/cytoskeleton/overlapping/cell012 gt.png]

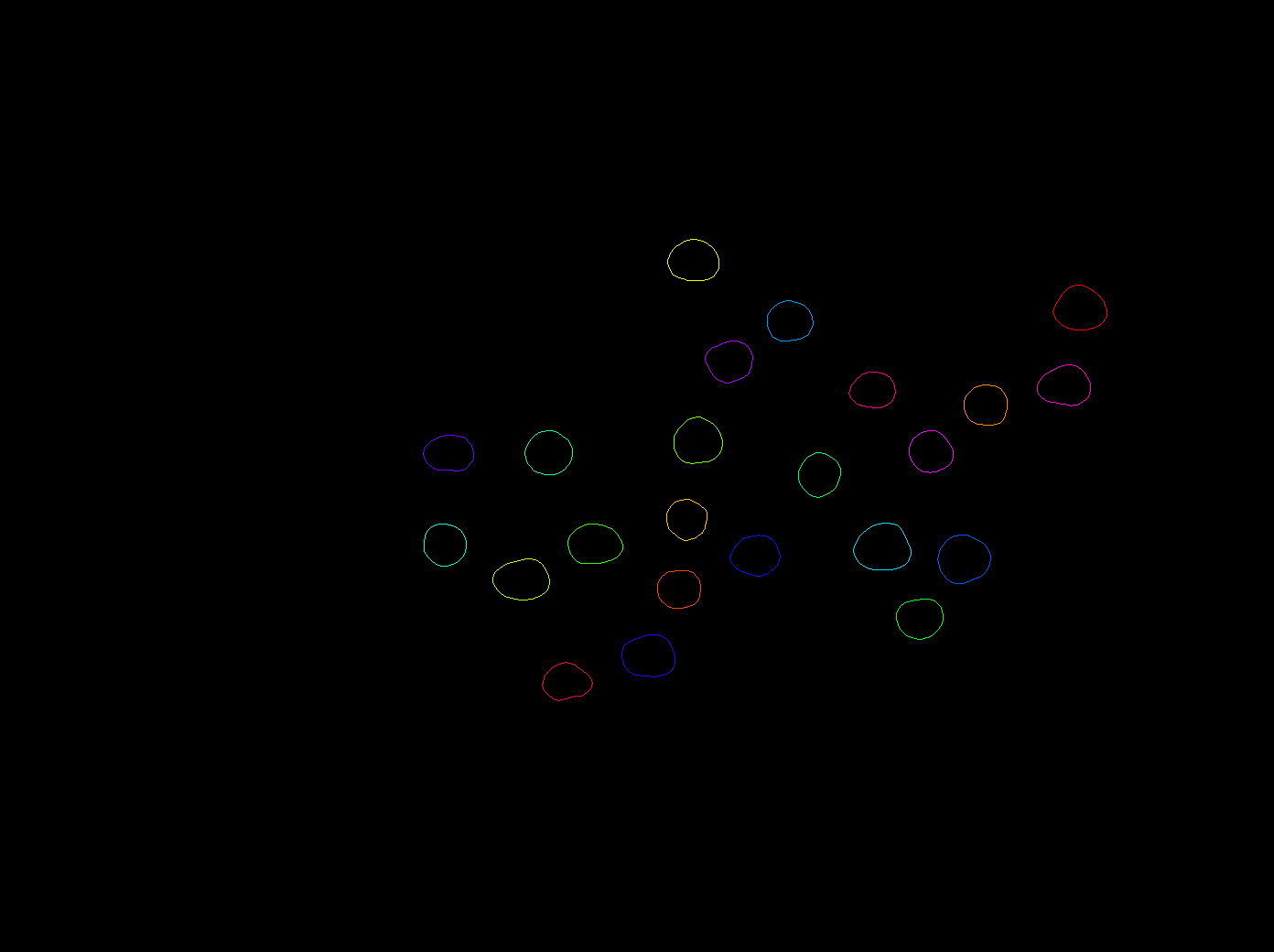

Supplement: Additional file 6 — The zip archive contains simulated images showing B cell nuclei and cytoskeleton with corresponding ground truth. (ZIP 119808 kb) [file 12859_2017_1591_MOESM6_ESM.zip › simulated B cells/cytoskeleton/overlapping/cell012 seeds.png]

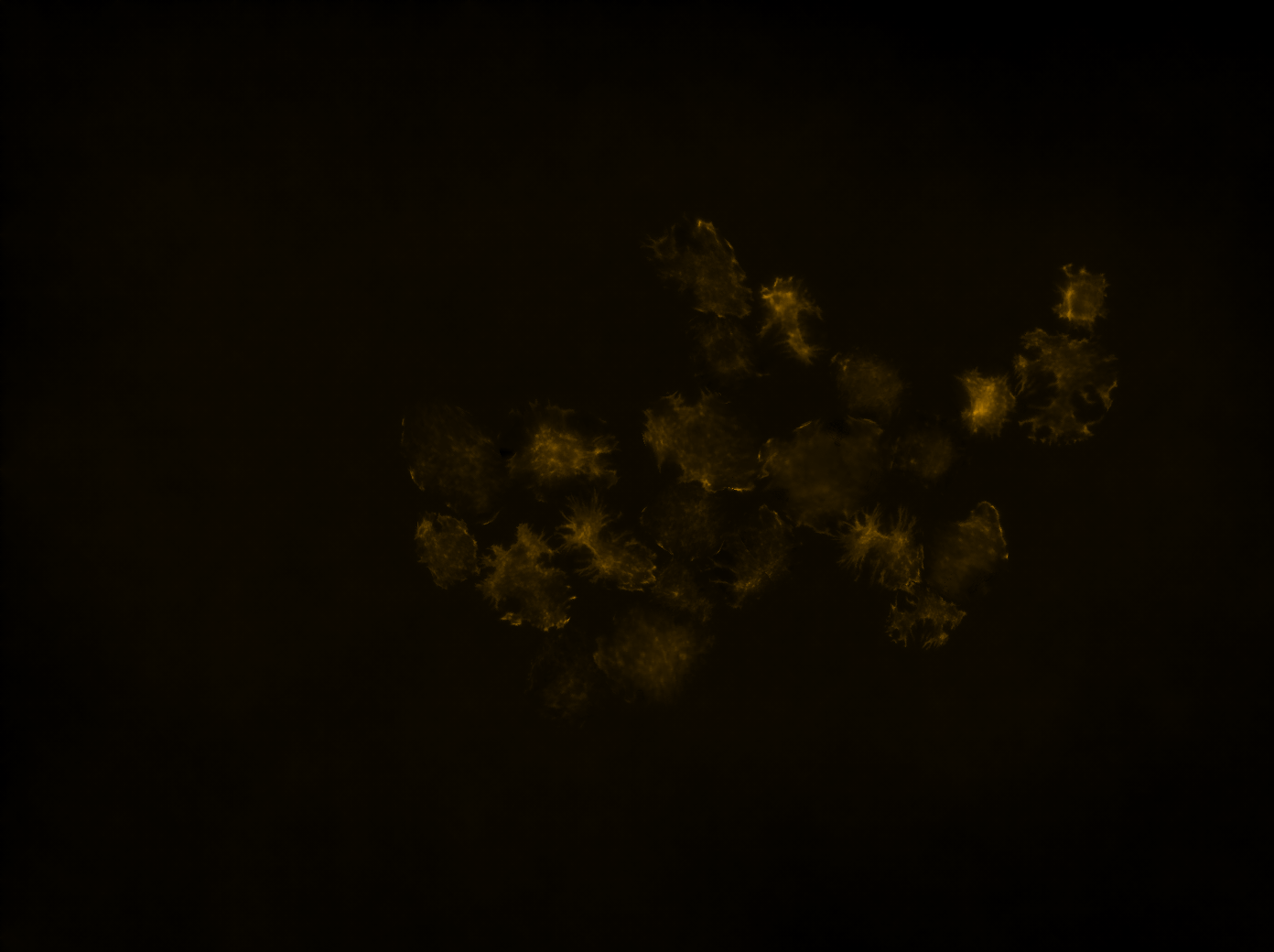

Supplement: Additional file 6 — The zip archive contains simulated images showing B cell nuclei and cytoskeleton with corresponding ground truth. (ZIP 119808 kb) [file 12859_2017_1591_MOESM6_ESM.zip › simulated B cells/cytoskeleton/overlapping/cell012.png]

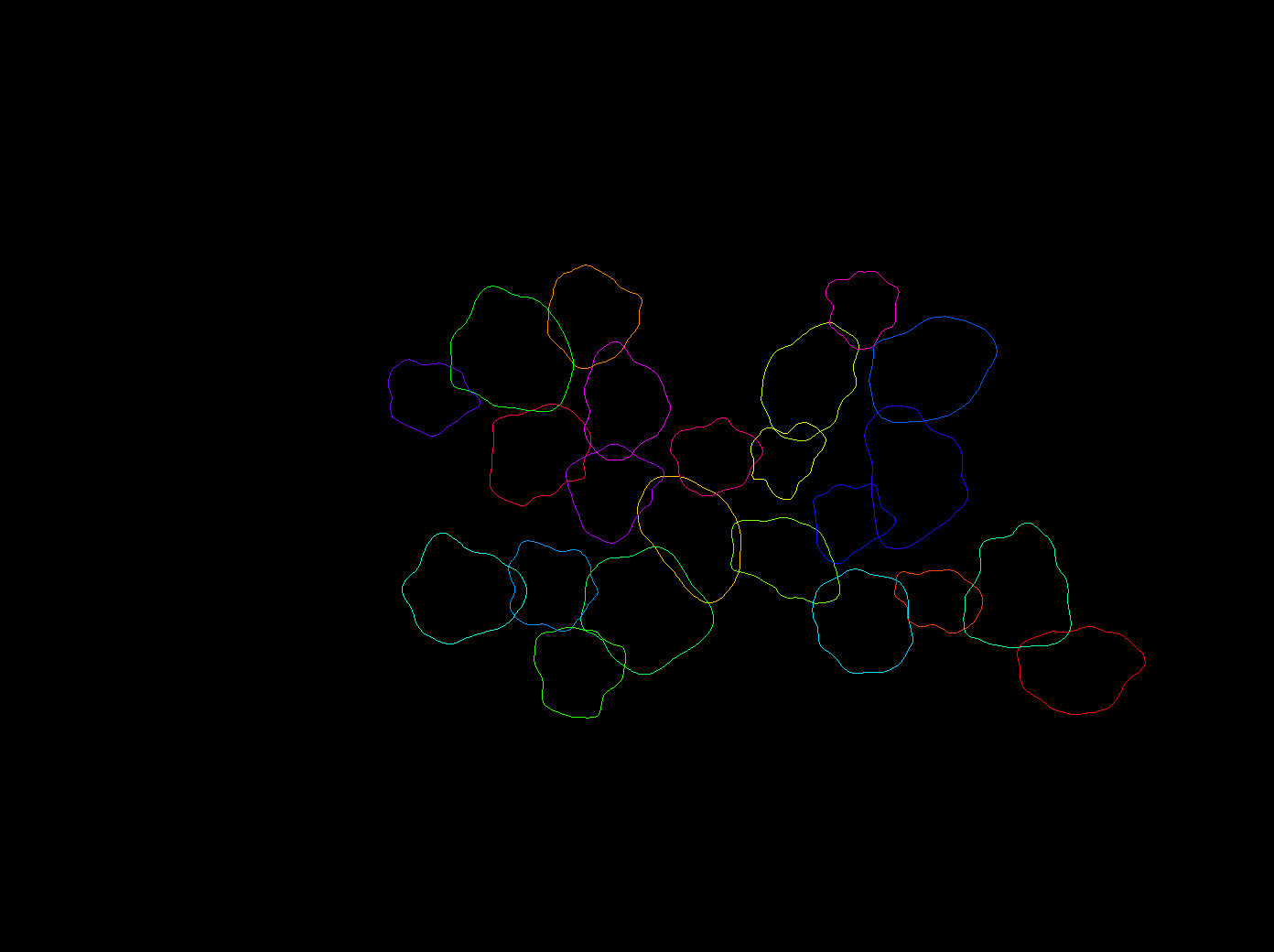

Supplement: Additional file 6 — The zip archive contains simulated images showing B cell nuclei and cytoskeleton with corresponding ground truth. (ZIP 119808 kb) [file 12859_2017_1591_MOESM6_ESM.zip › simulated B cells/cytoskeleton/overlapping/cell013 gt.png]

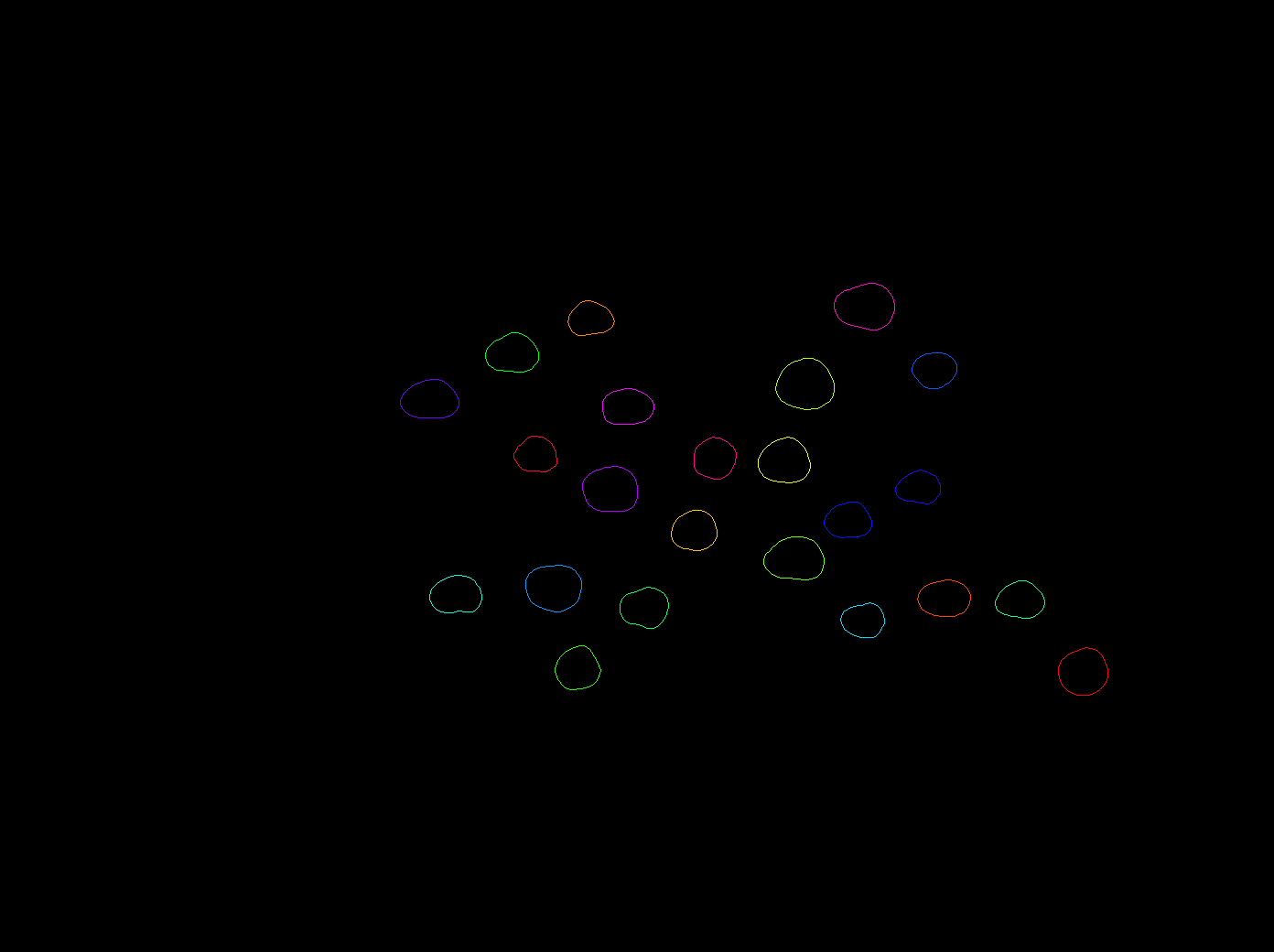

Supplement: Additional file 6 — The zip archive contains simulated images showing B cell nuclei and cytoskeleton with corresponding ground truth. (ZIP 119808 kb) [file 12859_2017_1591_MOESM6_ESM.zip › simulated B cells/cytoskeleton/overlapping/cell013 seeds.png]

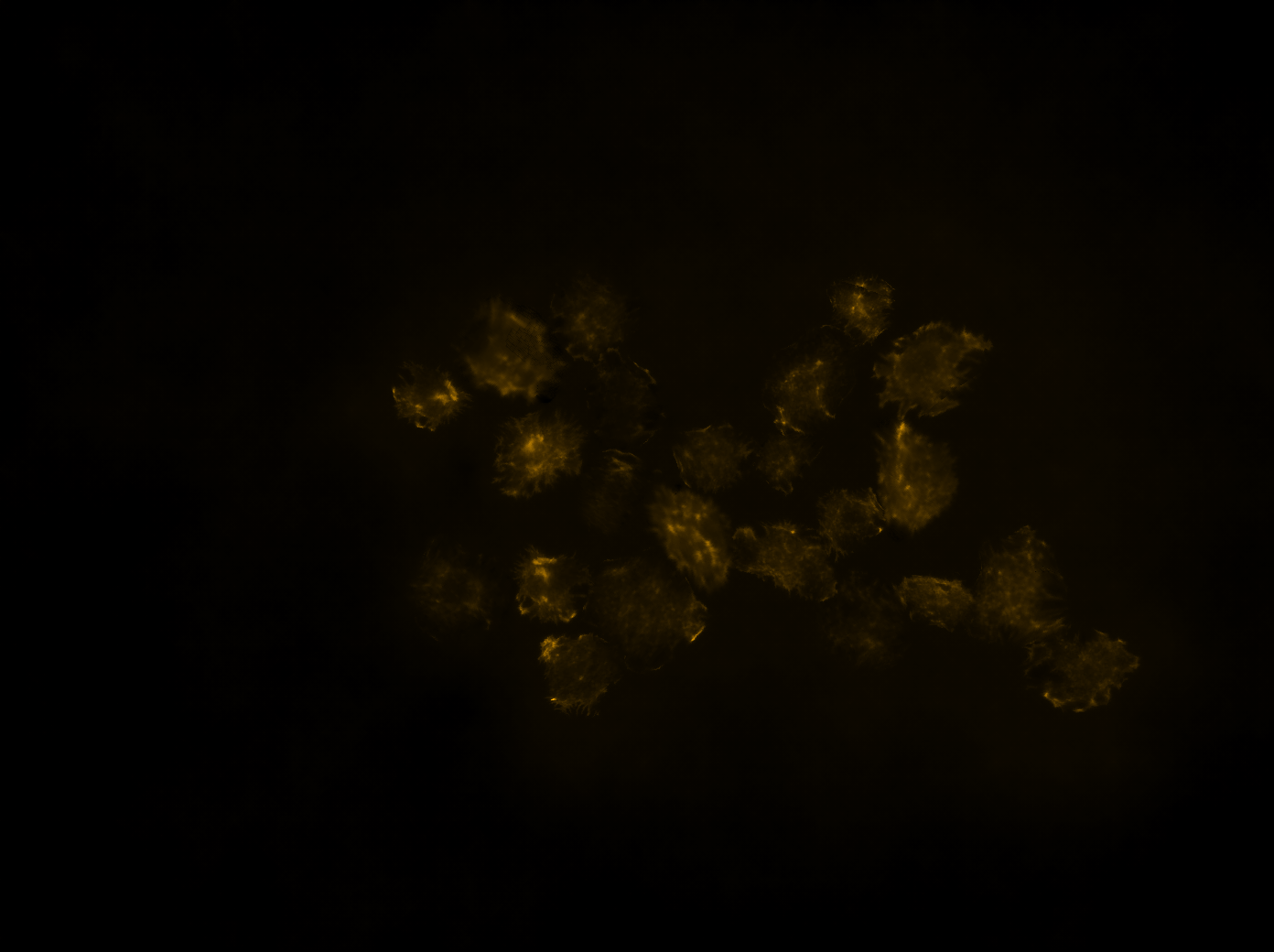

Supplement: Additional file 6 — The zip archive contains simulated images showing B cell nuclei and cytoskeleton with corresponding ground truth. (ZIP 119808 kb) [file 12859_2017_1591_MOESM6_ESM.zip › simulated B cells/cytoskeleton/overlapping/cell013.png]

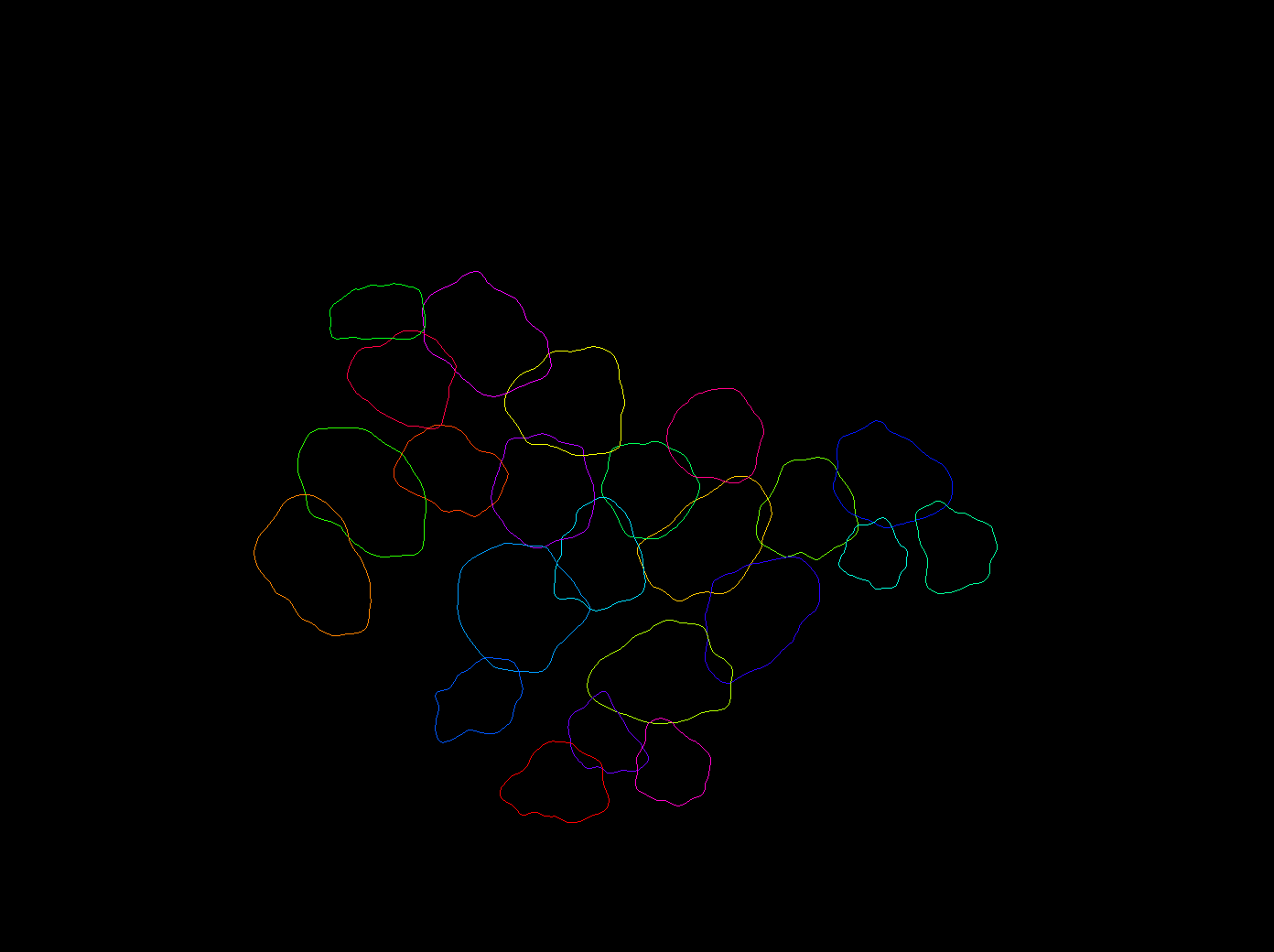

Supplement: Additional file 6 — The zip archive contains simulated images showing B cell nuclei and cytoskeleton with corresponding ground truth. (ZIP 119808 kb) [file 12859_2017_1591_MOESM6_ESM.zip › simulated B cells/cytoskeleton/overlapping/cell014 gt.png]

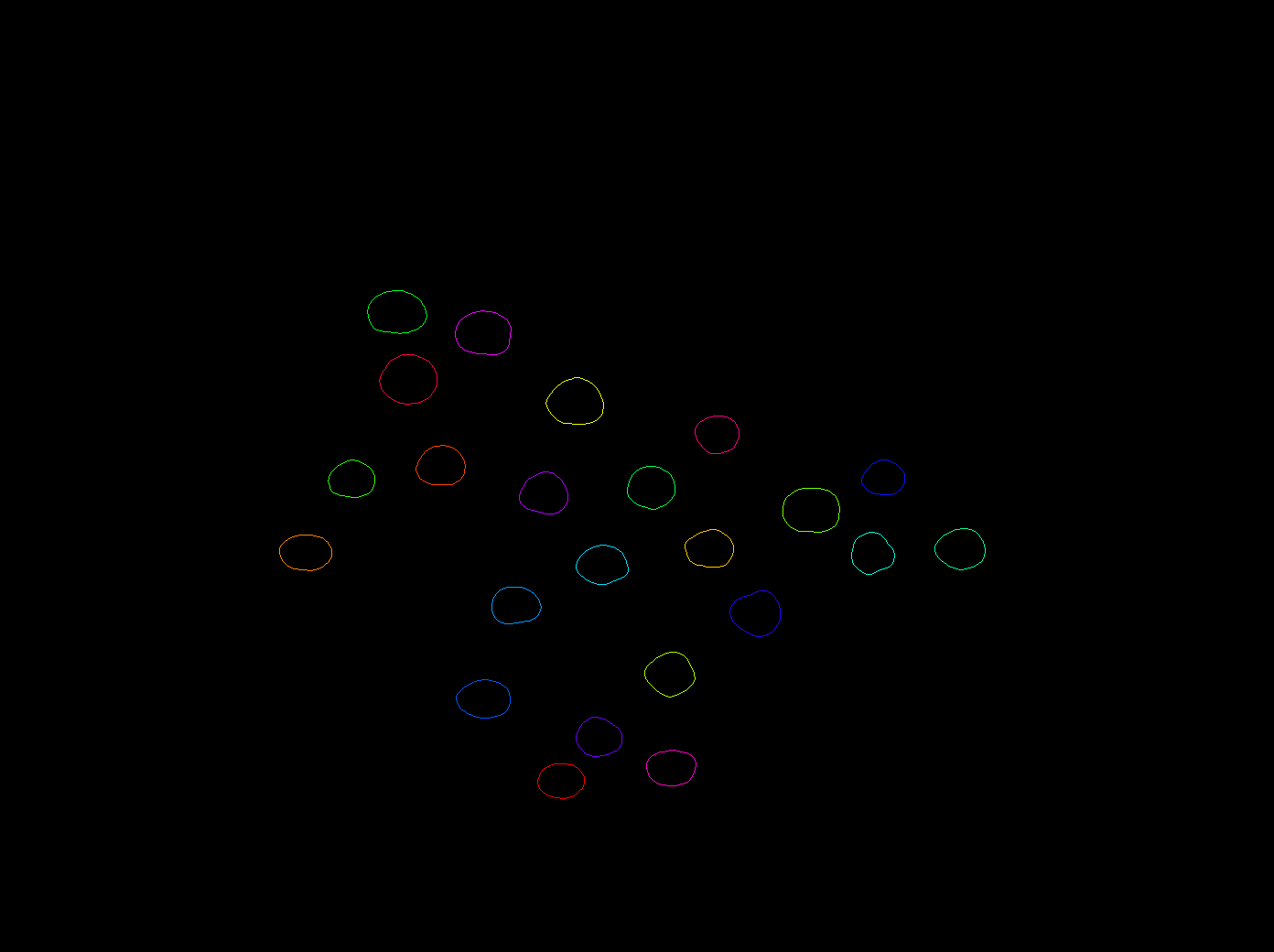

Supplement: Additional file 6 — The zip archive contains simulated images showing B cell nuclei and cytoskeleton with corresponding ground truth. (ZIP 119808 kb) [file 12859_2017_1591_MOESM6_ESM.zip › simulated B cells/cytoskeleton/overlapping/cell014 seeds.png]

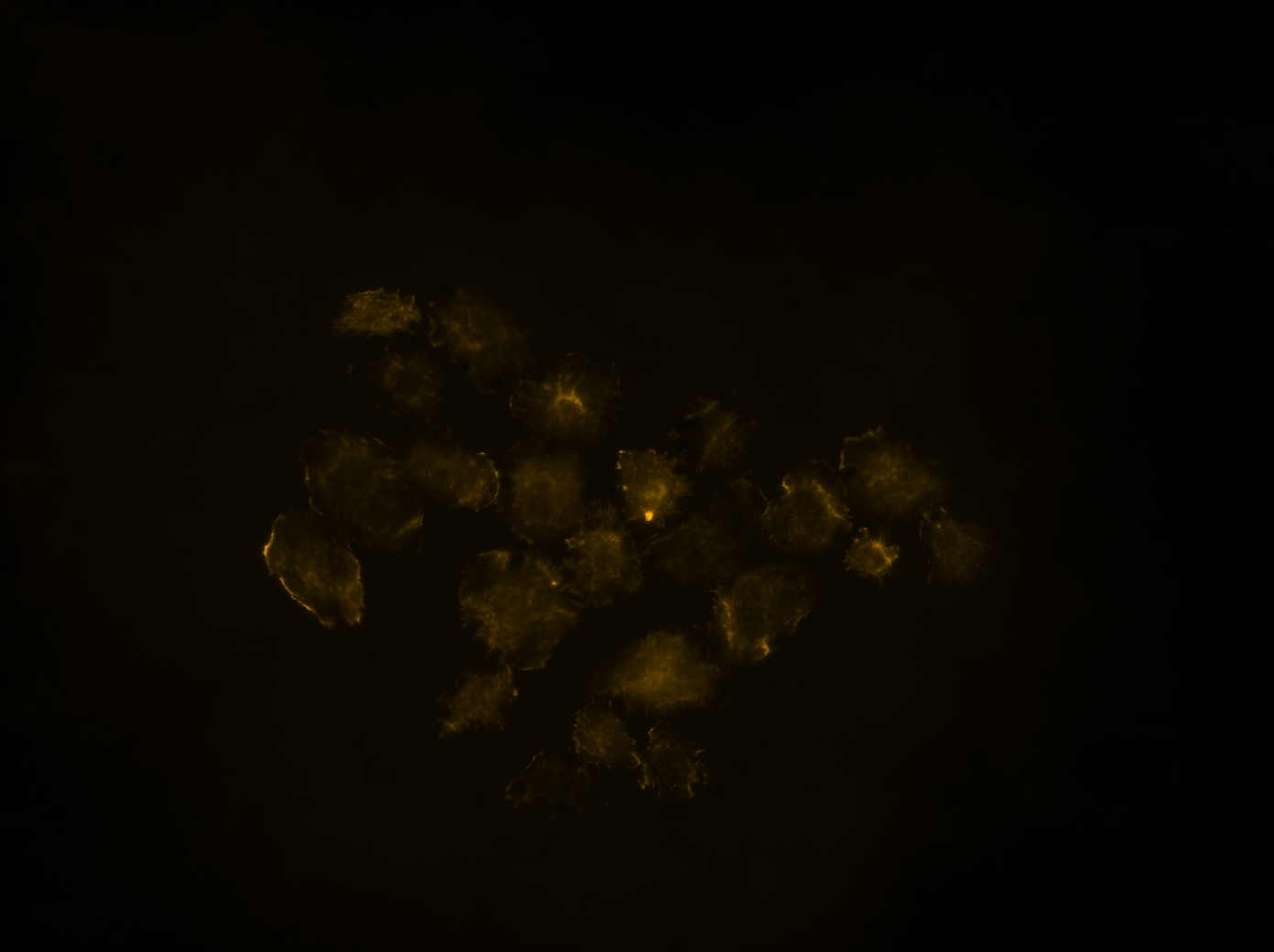

Supplement: Additional file 6 — The zip archive contains simulated images showing B cell nuclei and cytoskeleton with corresponding ground truth. (ZIP 119808 kb) [file 12859_2017_1591_MOESM6_ESM.zip › simulated B cells/cytoskeleton/overlapping/cell014.png]

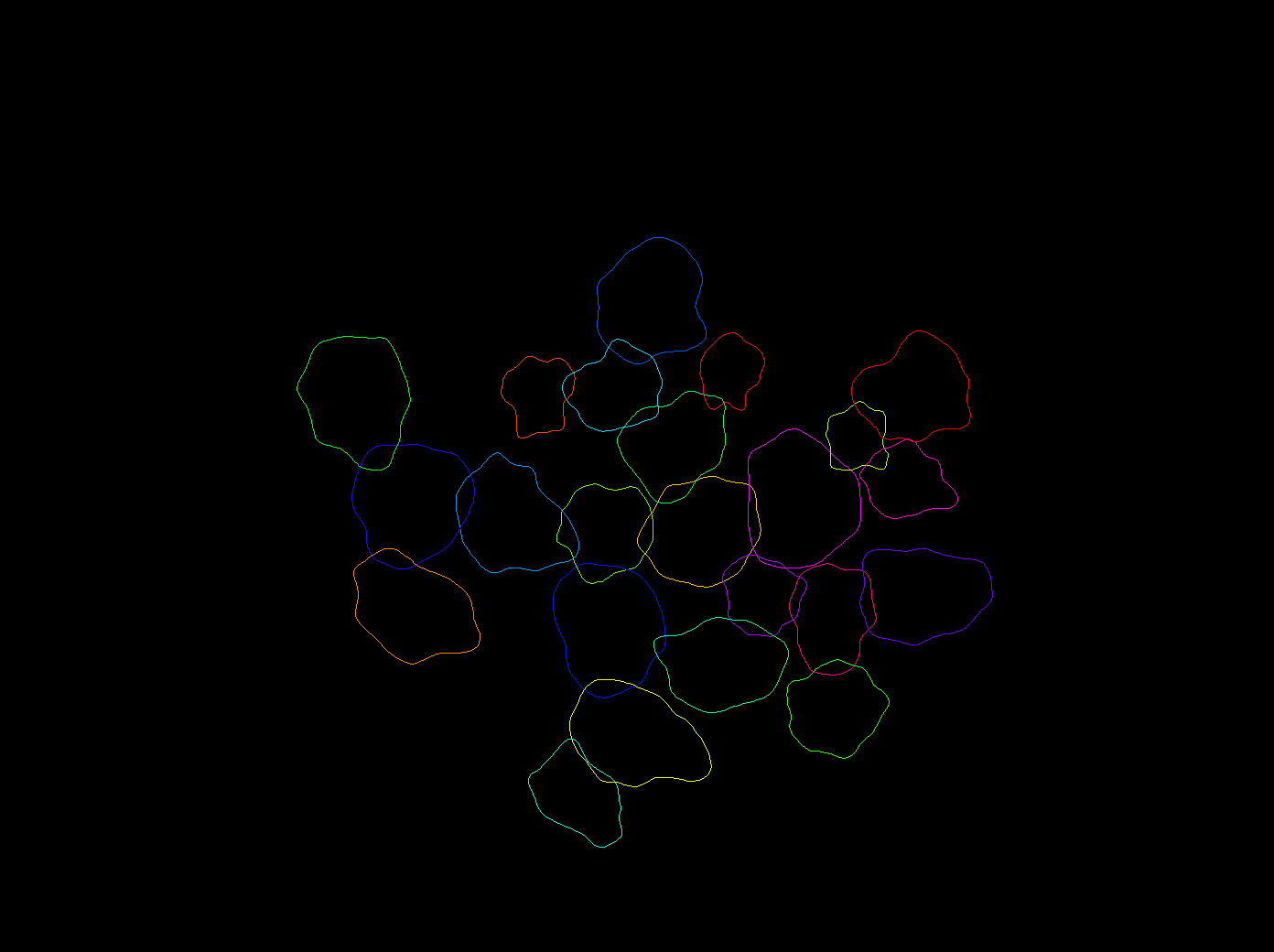

Supplement: Additional file 6 — The zip archive contains simulated images showing B cell nuclei and cytoskeleton with corresponding ground truth. (ZIP 119808 kb) [file 12859_2017_1591_MOESM6_ESM.zip › simulated B cells/cytoskeleton/overlapping/cell015 gt.png]

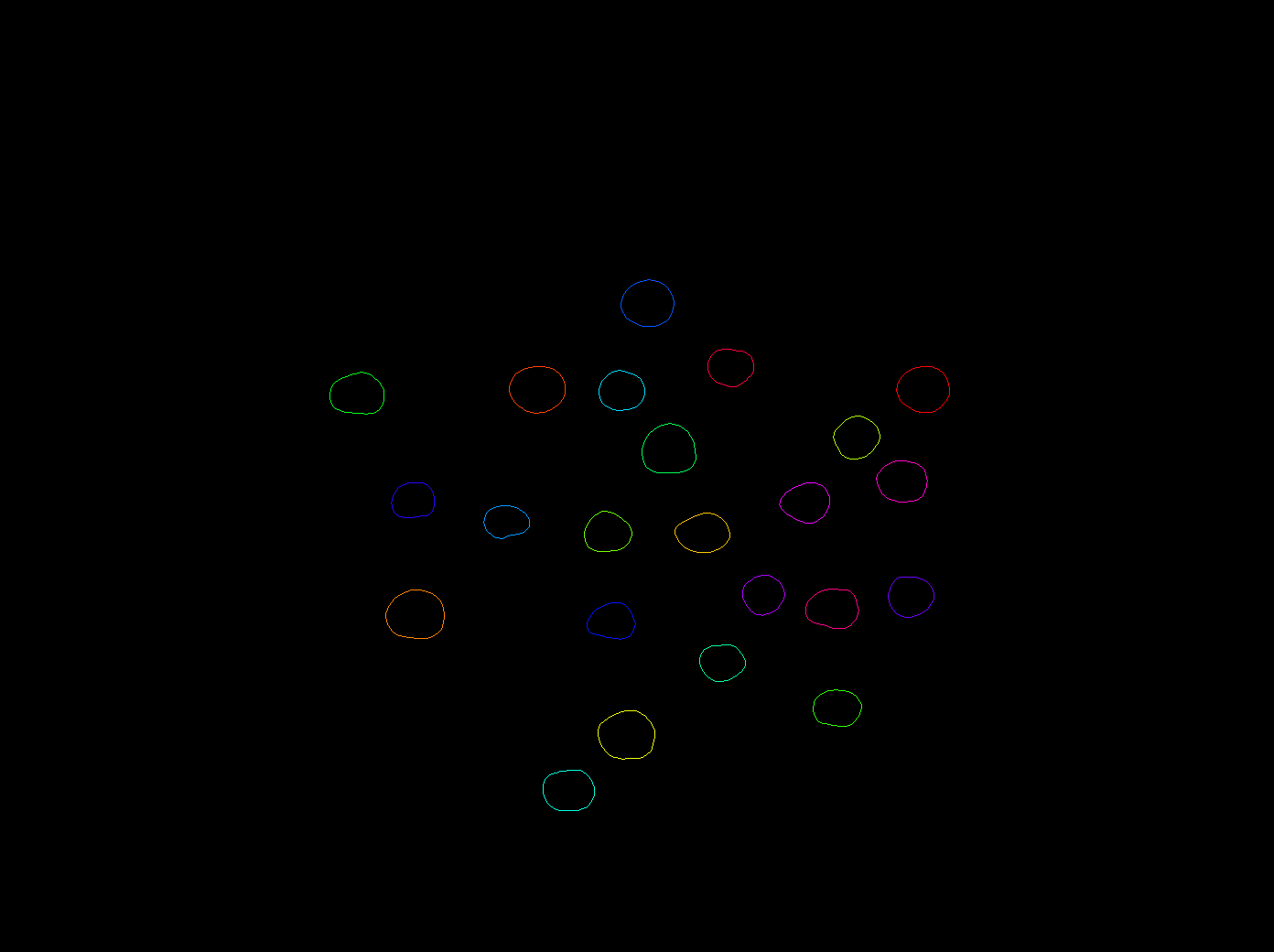

Supplement: Additional file 6 — The zip archive contains simulated images showing B cell nuclei and cytoskeleton with corresponding ground truth. (ZIP 119808 kb) [file 12859_2017_1591_MOESM6_ESM.zip › simulated B cells/cytoskeleton/overlapping/cell015 seeds.png]

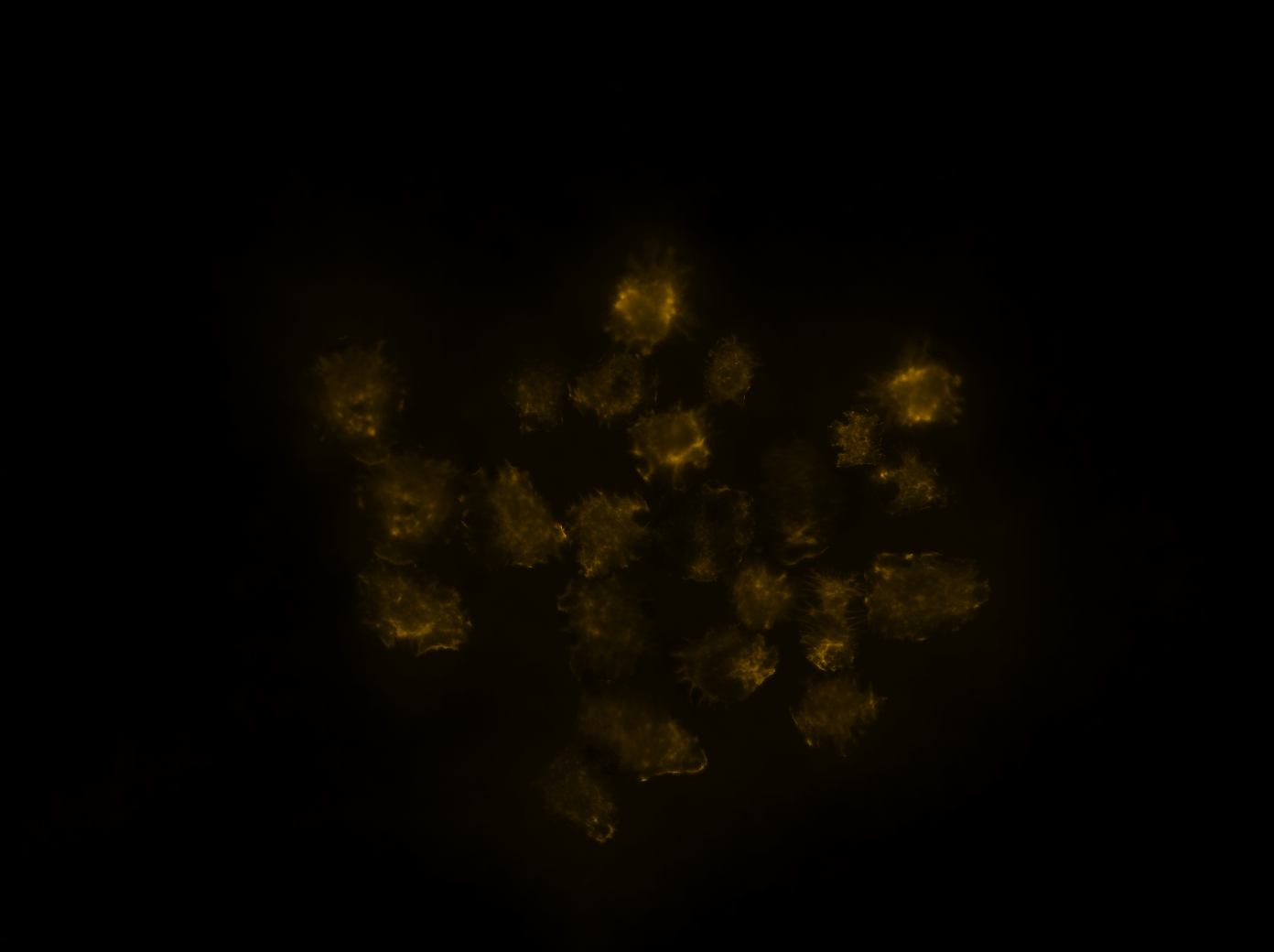

Supplement: Additional file 6 — The zip archive contains simulated images showing B cell nuclei and cytoskeleton with corresponding ground truth. (ZIP 119808 kb) [file 12859_2017_1591_MOESM6_ESM.zip › simulated B cells/cytoskeleton/overlapping/cell015.png]

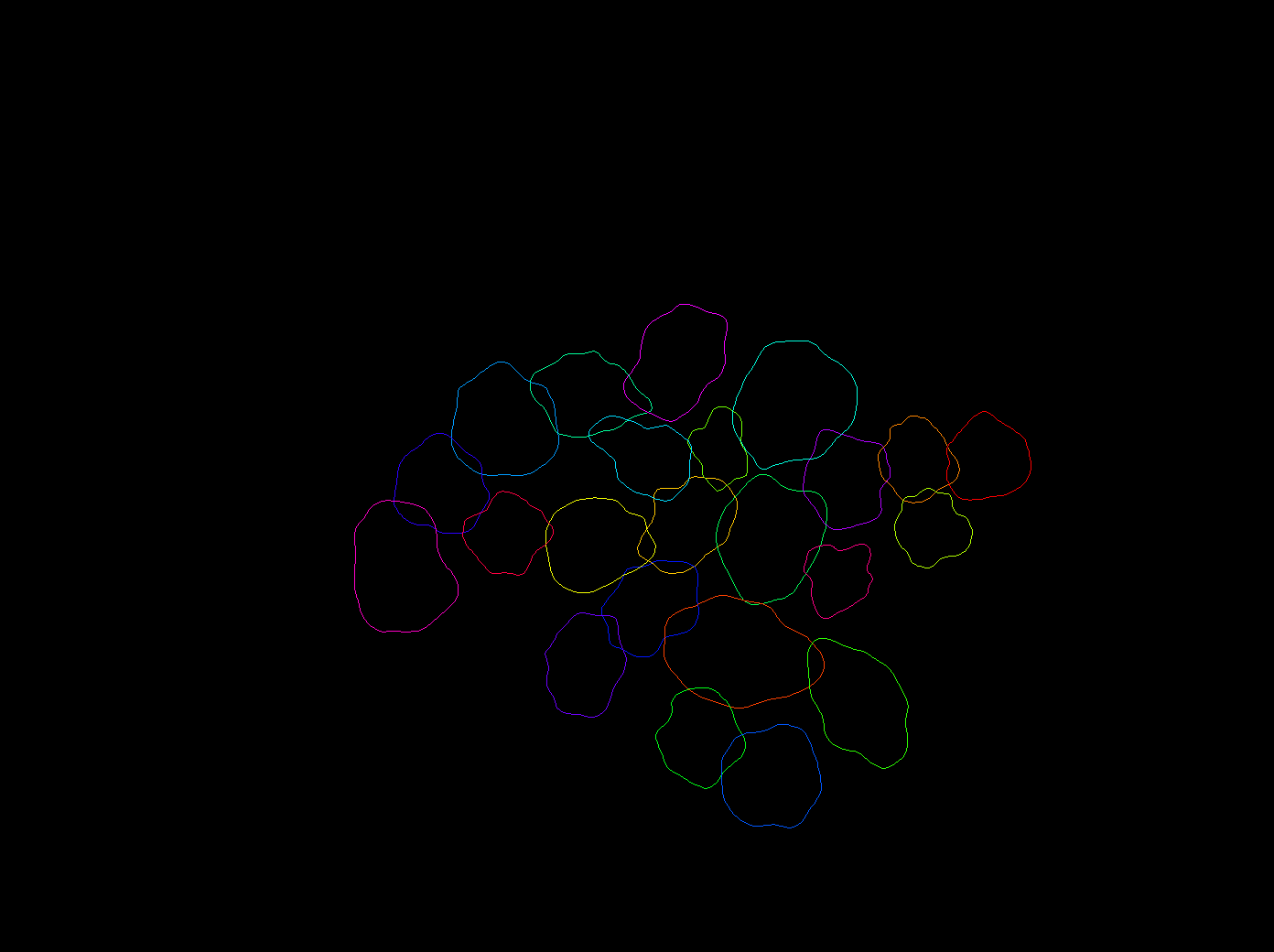

Supplement: Additional file 6 — The zip archive contains simulated images showing B cell nuclei and cytoskeleton with corresponding ground truth. (ZIP 119808 kb) [file 12859_2017_1591_MOESM6_ESM.zip › simulated B cells/cytoskeleton/overlapping/cell016 gt.png]

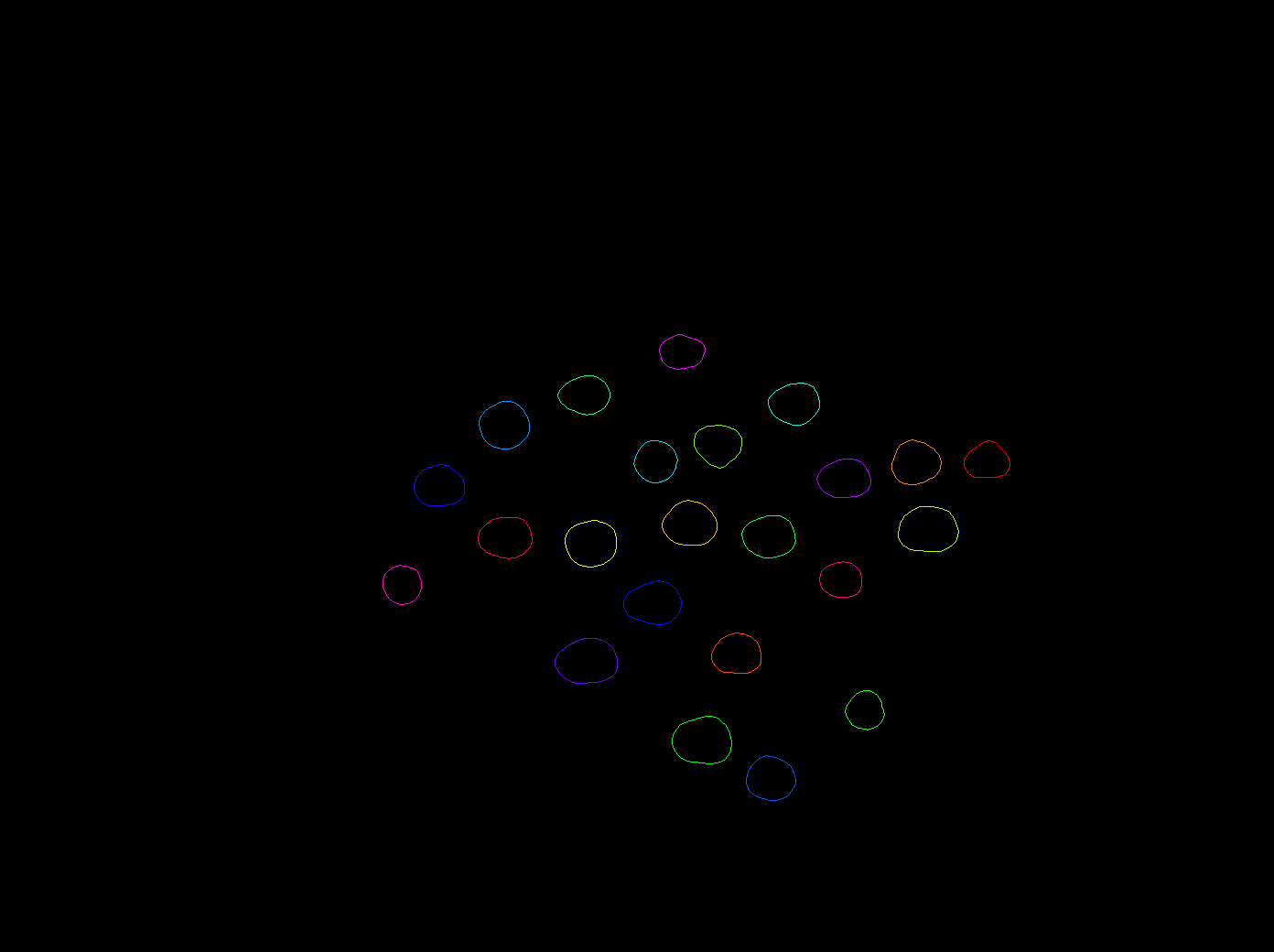

Supplement: Additional file 6 — The zip archive contains simulated images showing B cell nuclei and cytoskeleton with corresponding ground truth. (ZIP 119808 kb) [file 12859_2017_1591_MOESM6_ESM.zip › simulated B cells/cytoskeleton/overlapping/cell016 seeds.png]

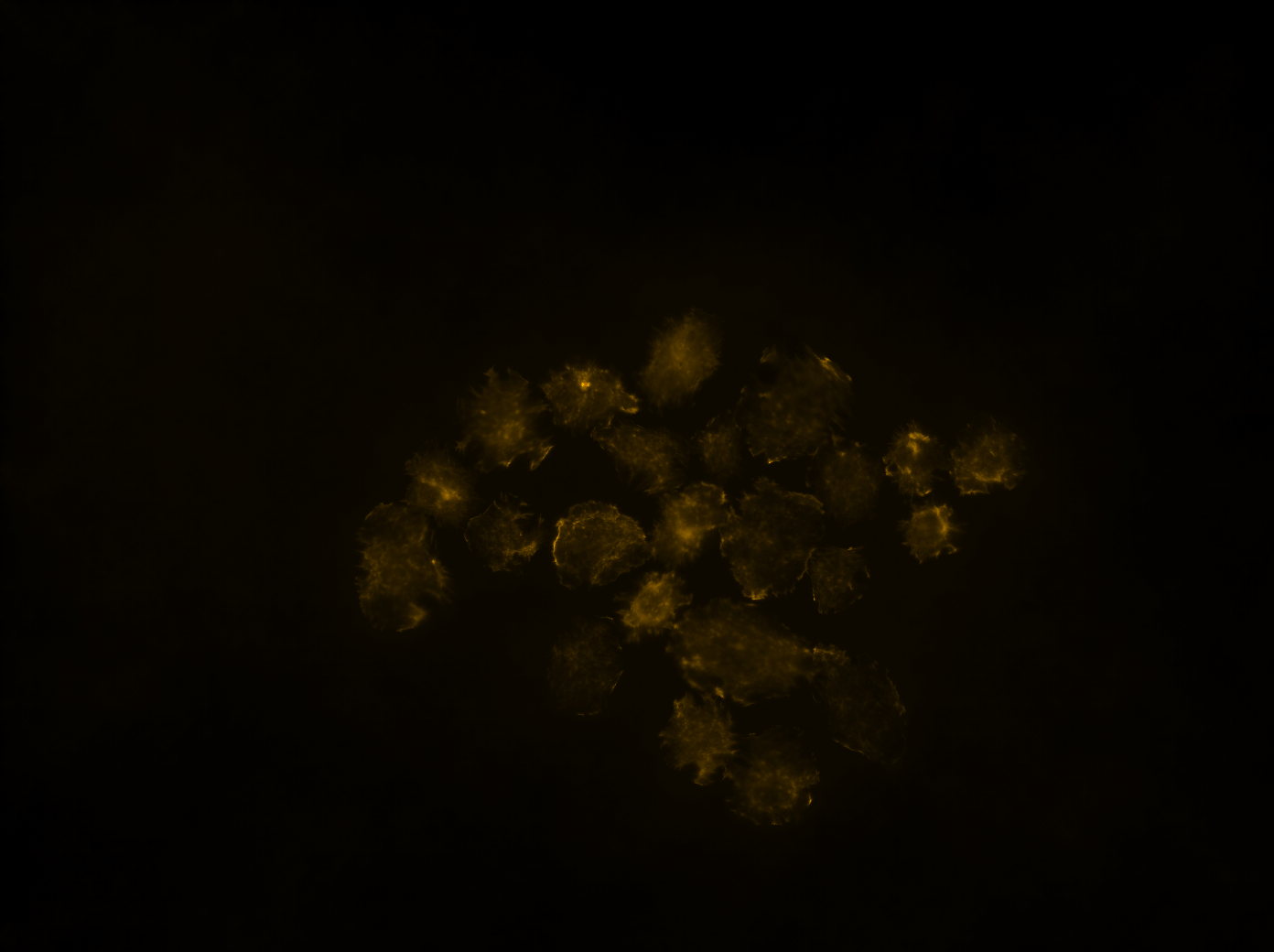

Supplement: Additional file 6 — The zip archive contains simulated images showing B cell nuclei and cytoskeleton with corresponding ground truth. (ZIP 119808 kb) [file 12859_2017_1591_MOESM6_ESM.zip › simulated B cells/cytoskeleton/overlapping/cell016.png]

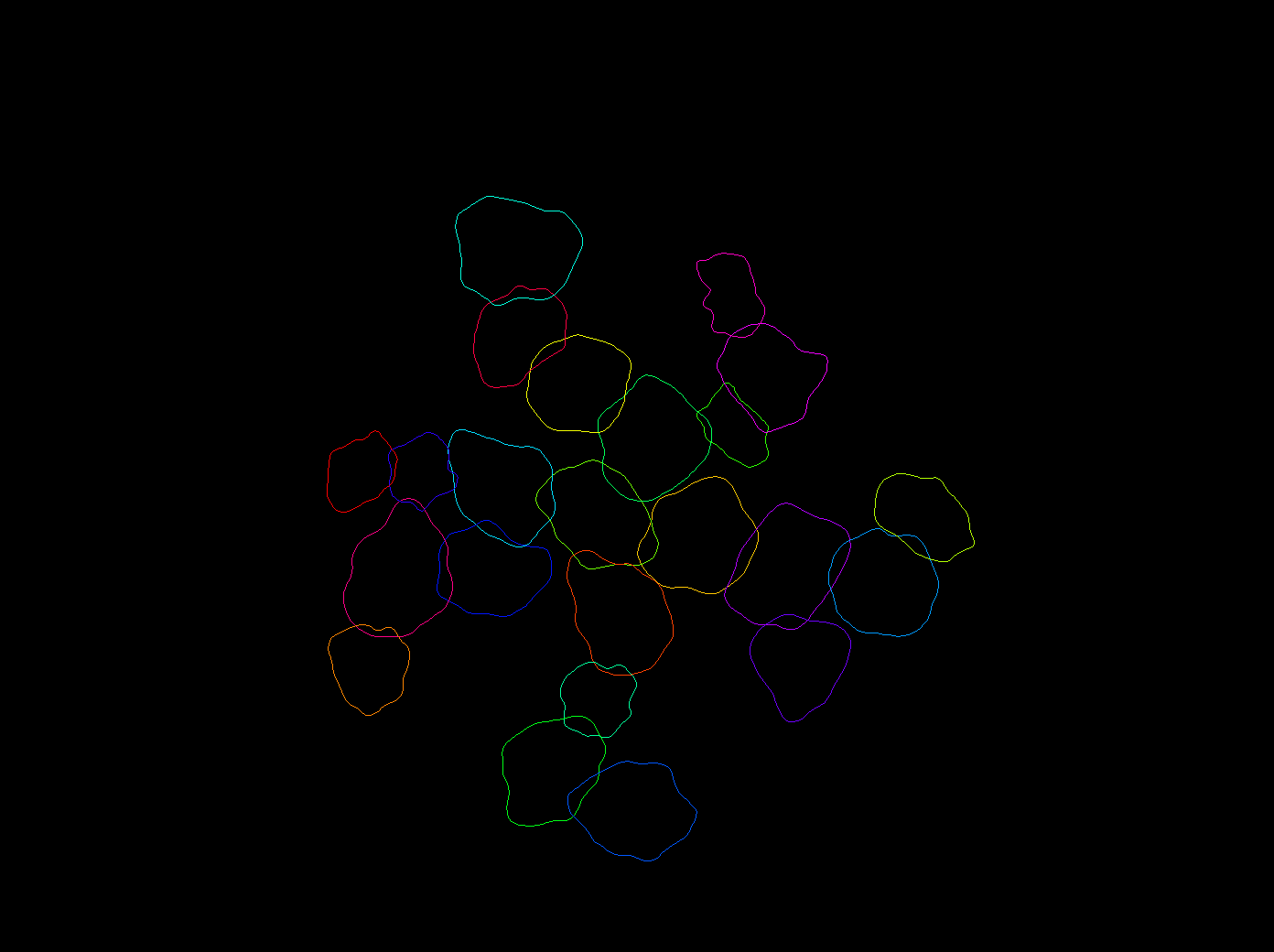

Supplement: Additional file 6 — The zip archive contains simulated images showing B cell nuclei and cytoskeleton with corresponding ground truth. (ZIP 119808 kb) [file 12859_2017_1591_MOESM6_ESM.zip › simulated B cells/cytoskeleton/overlapping/cell017 gt.png]

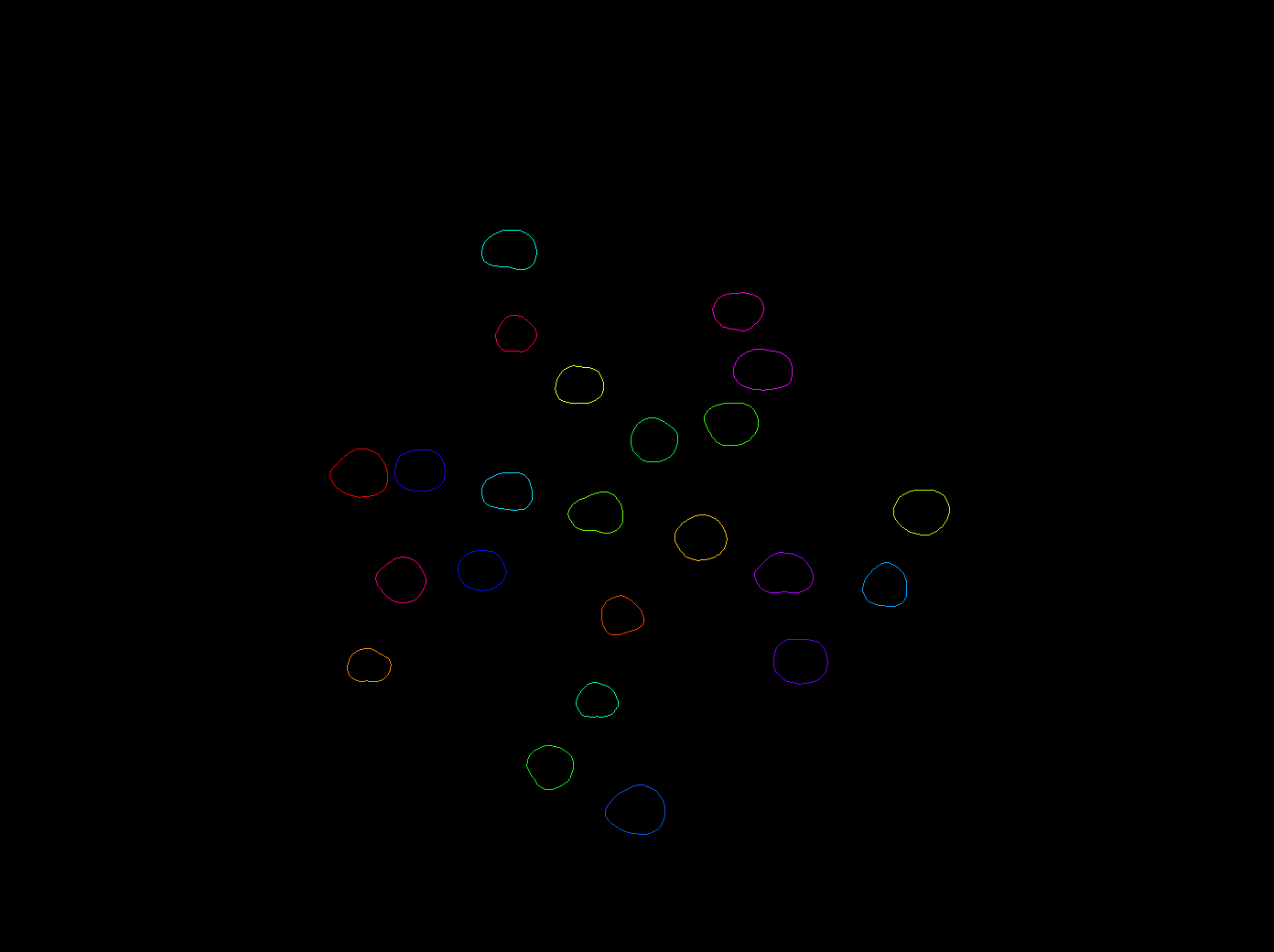

Supplement: Additional file 6 — The zip archive contains simulated images showing B cell nuclei and cytoskeleton with corresponding ground truth. (ZIP 119808 kb) [file 12859_2017_1591_MOESM6_ESM.zip › simulated B cells/cytoskeleton/overlapping/cell017 seeds.png]

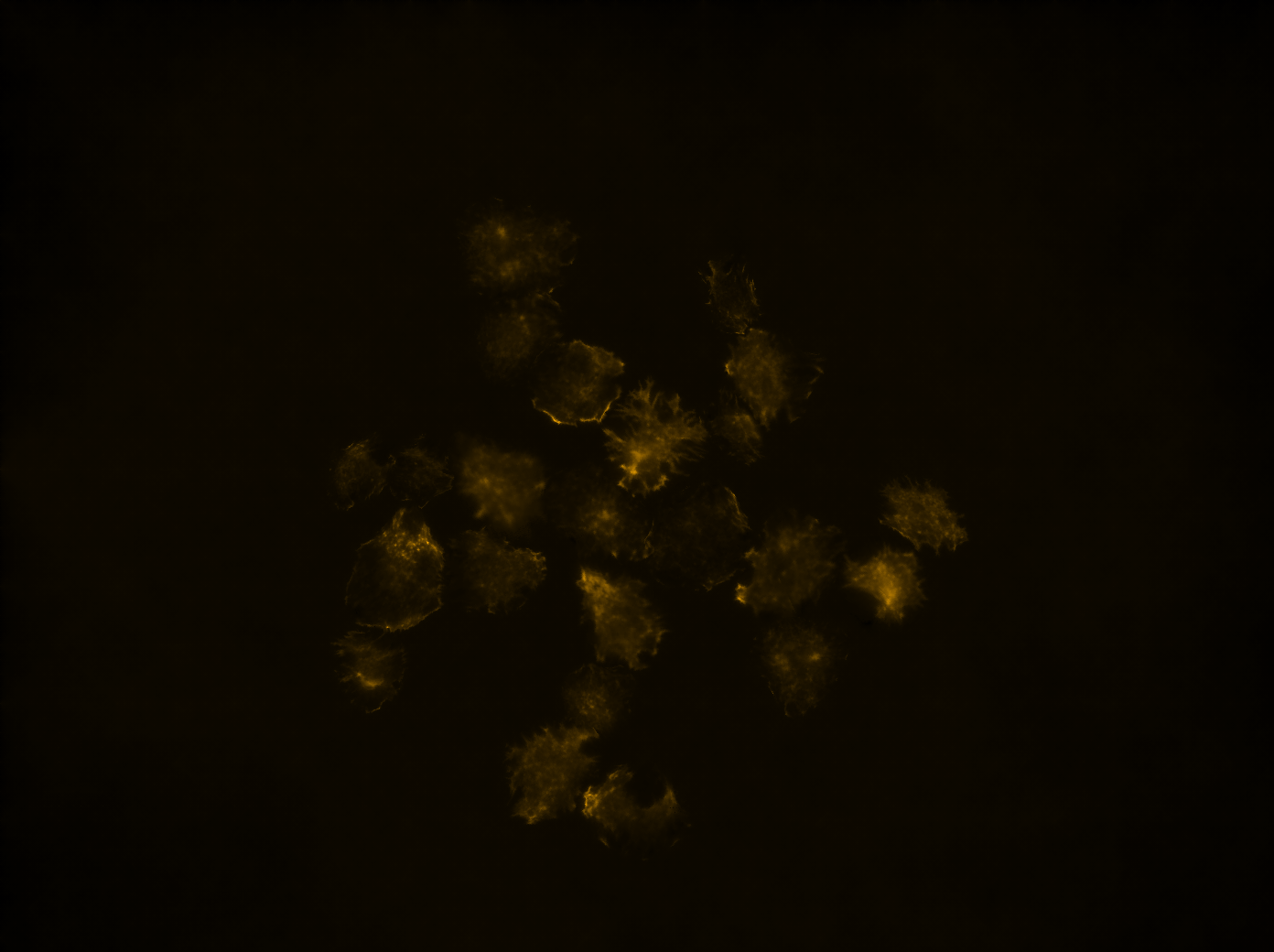

Supplement: Additional file 6 — The zip archive contains simulated images showing B cell nuclei and cytoskeleton with corresponding ground truth. (ZIP 119808 kb) [file 12859_2017_1591_MOESM6_ESM.zip › simulated B cells/cytoskeleton/overlapping/cell017.png]
